# Are we restoring functional fens? – The outcomes of restoration projects in fens re-analysed with plant functional traits

**DOI:** 10.1371/journal.pone.0215645

**Published:** 2019-04-24

**Authors:** Agata Klimkowska, Klara Goldstein, Tomasz Wyszomirski, Łukasz Kozub, Mateusz Wilk, Camiel Aggenbach, Jan P. Bakker, Heinrich Belting, Boudewijn Beltman, Volker Blüml, Yzaak De Vries, Beate Geiger-Udod, Ab P. Grootjans, Petter Hedberg, Henk J. Jager, Dick Kerkhof, Johannes Kollmann, Paweł Pawlikowski, Elisabeth Pleyl, Warner Reinink, Hakan Rydin, Joachim Schrautzer, Jan Sliva, Robert Stańko, Sebastian Sundberg, Tiemo Timmermann, Lesław Wołejko, Rob F. van der Burg, Dick van der Hoek, Jose M. H. van Diggelen, Adrie van Heerden, Loekie van Tweel, Kees Vegelin, Wiktor Kotowski

**Affiliations:** 1 Department of Plant Ecology & Environmental Conservation, Institute of Botany, Biological and Chemical Research Centre, University of Warsaw, Warsaw, Poland; 2 Ecosystem Management Research Group, Department of Biology, University of Antwerp, Antwerp, Belgium; 3 The Community and Conservation Ecology Group, University of Groningen, Groningen, the Netherlands; 4 Staatliche Vogelschutzwarte—Naturschutzstation Dümmer–Niedersächsischer Landesbetrieb für Wasserwirtschaft, Küsten- und Naturschutz Am Ochsenmoor, Ochsenmoor, Germany; 5 Environmental Biology, Utrecht University, Utrecht, the Netherlands; 6 BMS-Umweltplanung, Osnabrück, Germany in association with University of Bremen, Germany; 7 Natura 2000 Team, Regierung von Oberbayern, München, Germany; 8 Department of Plant Ecology and Evolution, Evolutionary Biology Centre, Uppsala University, Uppsala, Sweden; 9 It Fryske Gea (Frysian Landscape), Olterterp, the Nederlands; 10 Zuid-Hollands Landschap, Delft, the Netherlands; 11 Department of Ecology and Ecosystem management, Technische Universität München, Freising-Weihenstephan, Germany; 12 Zentrum für Umwelt und Kultur Benediktbeuern (ZUK), Gebietsbetreuerin Isar-Loisach-Moore, Benediktbeuern, Germany; 13 Institute for Ecosystem Research, University of Kiel, Kiel, Germany; 14 Klub Przyrodników, Świebodzin, Poland; 15 Landscape Ecology and Ecosystem Dynamics, Institute of Botany and Landscape Ecology, Ernst-Moritz-Arndt-University Greifswald, Greifswald, Germany; 16 Department of Botany, Institute of Botany and Nature Conservation, West Pomeranian University of Technology, Szczecin, Poland; 17 Coöperatie Bosgroep Zuid Nederland, Heeze & Floristische Werkgroep KNNV, Eindhoven, the Netherlands; 18 Nature Conservation and Plant Ecology Group, Wageningen University, Wageningen, the Netherlands; 19 B-WARE B.V. Research Center, Nijmegen, the Netherlands; 20 Province Zuid-Holland, The Hague, the Netherlands; 21 Landschap Overijssel, Dalfsen, the Netherlands; 22 BNL-Vegelin, Murchin, Germany; Qingdao Agricultural University, CHINA

## Abstract

In peatland restoration we often lack an information whether re-established ecosystems are functionally similar to non-degraded ones. We re-analysed the long-term outcomes of restoration on vegetation and plant functional traits in 38 European fens restored by rewetting (18 sites) and topsoil removal (20 sites). We used traits related to nutrient acquisition strategies, competitiveness, seed traits, and used single- and multi-trait metrics. A separate set of vegetation records from near-natural fens with diverse plant communities was used to generate reference values to aid the comparisons. We found that both restoration methods enhanced the similarity of species composition to non-degraded systems but trait analysis revealed differences between the two approaches. Traits linked to nutrient acquisition strategies indicated that topsoil removal was more effective than rewetting. After topsoil removal competitive species in plant communities had decreased, while stress-tolerant species had increased. A substantial reduction in nutrient availability ruled out the effect of initial disturbance. An ability to survive and grow in anoxic conditions was enhanced after restoration, but the reference values were not achieved. Rewetting was more effective than topsoil removal in restricting variation in traits values permitted in re-developing vegetation. We found no indication of a shift towards reference in seed traits, which suggested that dispersal constraint and colonization deficit can be a widespread phenomena. Two functional diversity indices: functional richness and functional dispersion showed response to restoration and shifted values towards reference mires and away from the degraded systems.

We concluded that targeting only one type of environmental stressor does not lead to a recovery of fens, as it provides insufficient level of stress to restore a functional ecosystem. In general, restoration efforts do not ensure the re-establishment and long-term persistence of fens. Restoration efforts result in recovery of fen ecosystems, confirmed with our functional trait analysis, although more rigid actions are needed for restoring fully functional mires, by achieving high and constant levels of anoxia and nutrient stresses.

## Introduction

Fens (i.e. groundwater-fed mires) have declined strongly during the past century, mainly due to drainage and nutrient enrichment, which caused extinctions of specialised species and large release of carbon dioxide and nutrients from decomposing peat soils [1]. The understanding of these losses is boosting restoration projects [2, 3], exposing a need to better understand the relationships between composition of plant communities and mire ecosystem processes.

Fen plant assemblages have developed under two strong stresses, interplaying with each other: (1) a lack of oxygen in the root zone (i.e. anoxia stress), mainly due to permanently high groundwater levels and (2) a low nutrient availability, mainly phosphorus (P) and nitrogen (N), due to supply of nutrient-poor and mineral-rich groundwater. These two stressors result in a low productivity, low growth rates [4–6], a slow decomposition and slow nutrient turn-over [7], and thus allow for a sequestration of organic matter, contributing to storage of carbon. They also control vegetation dynamics, holding down competitive plant species [8] including those that may trigger a change in this ecosystem [9, 10], e.g. shrubs and trees (hereafter ‘phanerophytes’) or *Sphagnum* mosses.

Degradation of fens implies alleviation of these environmental stressors, causing a replacement of stress-tolerant species by stronger competitors and cessation of peat formation due to enhancement of aerobic decomposition. Restoration aims to re-establish these stressors, however, whether this is achieved remains unclear. Restoration outcomes are usually judged based on presence of target species or vegetation similarity to reference [11], but this says little about ecosystem functioning or sustainability of the outcome. Hereby, we bring together results of long-term, standardised and quantifiable data from a range of sites to assess the recovery of fen ecosystem functions using framework of functional plant ecology.

Two commonly applied fen restoration measures are rewetting (RE) and topsoil removal (TSR) [11]. RE is by far the most common measure, typically achieved by blocking drainage ditches or flooding with surface water [1]. By inducing low redox potential in decomposed peats RE often results in phosphorus (P) mobilization and subsequent eutrophication [12, 13]. RE often leads to surface inundation, due to changes in peat structure and its hydrological conductivity [14, 15]. In contrast, TSR is used to reduce nutrient availability (mainly P), but also allows to remove ruderal and competitive plants and their seedbank, expose a bare soil for newly establishing species and increase wetness [16, 17]. TSR is increasingly applied in small-scale restoration projects on severely degraded sites in Western Europe [17, 18]. After TSR, re-establishment from seeds follows the initial disturbance. The same may happen after radical RE and die-off of the vegetation but often a more gradual change in community follows. Previous studies showed that the vegetation in restored systems usually differs from near-natural ones and that different sets of species benefit from RE and TSR [11, 19].

While the dissimilarity of restored fens from the undrained ones might be partly explained by dispersal constraints of target species [20, 21], the differences between fens restored by RE and TSR may result from their different effectiveness in re-instalment of anoxia and nutrient stresses. One can expect that, whereas RE is effective in restoring of soil anoxia, it is less likely to re-establish nutrient stress due to P remobilization. On the other hand, as TSR is directly targeting the removal of degraded peat, it should efficiently impoverish nutrients but may fail to re-install anoxia [22].

As dispersal constraints and stress factors act as filters during community assembly, they become reflected in, and can therefore be inferred from, plant functional traits (PFT) of the established community and its functional diversity (FD) [20, 23–26]. Fen degradation results in PFT shifts in the vegetation [27] and the reverse shift could be expected in restoration. Yet, how much the restored fen communities resemble functionally undrained fens remains largely unknown. In the present study, we try to fill this gap in knowledge by analysing outcomes of both methods in terms of PFT characteristics of restored communities, as well as their FD indices and compar to degraded sites and near-natural reference fens.

The operational framework linking PFTs to fen ecosystem functions is still not well-established. Relative contribution of certain ecological groups (e.g. bryophytes (bry), phanerophytes (pha), brown mosses (BM), Sphagnum (SPH), sedges (CY), grasses (PO), forbs (FO), ferns, spore-plants (PT)) is a simple method to assess the similarity of restored fens to reference ecosystems [28]. However, disentangling between filtering effects of anoxia and nutrients stress is not a straight-forward task. Thought plants develop various adaptations to anoxia at physiological levels, growth form or tissue adaptations [6] and references within], they are hard to quantify and generally absent in trait databases. As a proxy, summarising different adaptations, Ellenberg indicator value for moisture [29] proved useful [26].

Both anoxia and nutrient stress may promote PFTs related to conservative leaf economics, such as high leaf dry matter content (LDMC) and small specific leaf area (SLA) [24, 26, 30, 31]. CRS life-strategies [32] can also be used to learn about general levels of resource stress do not allow to distinguish effects of anoxia and nutrient limitation. Leaf nutrient (N, P) content might be more a reliable indicator of site nutrient-richness [5, 33]. Both, SLA and LDMC reflect plant acquisition strategies, relate to leaf economic spectrum [33] and indicate decomposability [34].

Another way to estimate nutrient stress, independently of anoxia, is to infer it indirectly from PFTs related to competition for light. High nutrient levels (low stress) promote tall, fast-growing species–in fens mainly large sedges and grasses [8], in contrast to small-sized plants, with late flowering and late seed-set onset that dominate in low-productive sites.

The relative importance of nutrient and anoxia stresses can also be indicated by species associations with mycorrhizal fungi and N-fixing bacteria [35, 36]. Restoring P-limited conditions should restrict species that are not able to gain additional P from mycorrhiza. On the other hand, as mycorrhizal fungi require oxygen, dominance of non-mycorrhizal species may indicate a permanent and widespread anoxia in the topsoil. Therefore, we expected an increase of non-mycorrhizal species after RE [37]. After TSR the presence of mycorrhizal fungi in the soil is likely low [38, 39] thus species relaying on mycorrhiza might be in disadvantage, but flexible species, growing with or without mycorrhizal fungi, should be favoured. The share of N-fixing species indicates N deficiency, so we expect it to increase especially after TSR, which removes most of plant-available N from the system.

The importance of disturbance during community assembly can be detected from the relative share of species with vegetative vs generative reproduction strategies.

In fens the clonal reproduction prevails over reproduction by seeds, which is more frequent in disturbed environments [27, 40]. Effective clonal spread occurs in mire dominants but also in competitive species [40, 41]. We expected high abundance of such species after RE, whereas after TSR we expected higher abundance of species re-establishing from seeds, due to exposure of bare substrate and soil seedbanks.

Dispersal limitation can be detected from seed and seed investment traits [20, 21]. Species with small seeds produce smaller seedlings [42], with less advantage in establishment, but such seeds survive longer in the soil [43] and disperse further [44]. Species producing more seeds per ramet may have more chances to colonize new sites, due to stochastic processes [45], however, due to a trade-off between seed mass and seed production, may be less capable to germinate in sward [42]. Species associated with soil disturbance have high germination rates, whereas many fen species produce dormant seeds or have low germination rates [17]. Seed buoyancy was found to be associated with inundation [46] and indicates adaptation to frequent flooding. Probability of dispersal is also related to adaptations to (multiple) dispersal vectors [47]. Both restoration methods can be affected by dispersal limitation, but they result in different conditions for establishment and seedling survival. After RE we expect species with larger seeds and higher buoyancy. After TSR, we expect a large proportion of fecund species with smaller seeds and large seed number, and larger effect of the dispersal limitation.

Finally, multi-trait indices of FD can be used to compare the relative strength of habitat filtering versus filtering by competition [48–52] and may indicate ecosystem’s resilience and stability [53, 54]. High level of habitat filtering restricts the spectrum of possible life strategies in fens as compared to drained sites [26], therefore a decrease of functional richness (FRich) and functional dispersion (FDis) is expected after restoration. While FRich is related to species numbers, other metrics, such as FDis or functional divergence (FDiv) are independent of it [52, 55]. While re-establishing the environmental stresses should be a priority for a sustainable fen restoration, maximizing species richness may not contribute to it. We expect that the multi-trait metrics, representing FD of restored fens gain similarity to non-degraded mires, irrespective to their species richness.

To summarise, we formulated following hypotheses in this study:

1. Restoration measures enhance the similarity to non-degraded systems in terms of PFTs.
2. In terms of the effects of restoration measures on PFTs, we expect that TSR mainly affects PFTs linked to competitive ability and leaf nutrient economics, whereas RE chiefly alters PFTs related to tolerance of anoxia, but also general stress indicators.
3. The outcomes after both restoration methods result in dispersal-related PFT dissimilar from non-degraded mires, due to dispersal limitation. Further we expect that TSR will stronger benefit fast colonizers and species that are relatively easily dispersing.
4. In restored systems, a decrease of functional diversity (FD) is expected, as they become more similar to non-degraded systems, and this process is independent of species richness.

## Methods

### Site selection and vegetation data

This study aimed at detecting the main patterns in response of PFT after restoration in fen ecosystems by re-analysing monitoring data from multiple projects. This allows us to generalize the outcomes for a wide range of conditions. Monitoring records were gathered from restored groundwater-fed lowland fens across Western and Central Europe, representing various landscape types and levels of degradation severity. We selected only those data sets, where full vegetation records from before and several years after the restoration were present, to only include relatively mature and stabilized communities.

**Fig 1:**
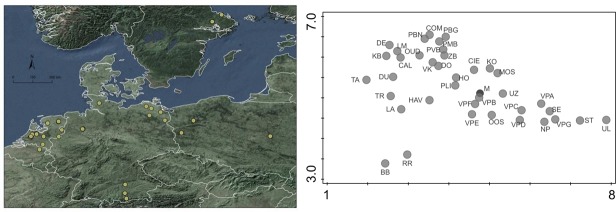
**Geographic distribution of the sites (left) and DCA graphs of sites (centre of centroids) (right).** Ordination with 5476 plots, 837 species. Eigenvalues for first and second ordination axis: 0.88, 0.65 (gradient length 9.0, 7.2 SD units, respectively). Explained variation (cumulative): 2% and 3.4%. A relatively low explained variation was possibly due to large number of species. For site symbols see S1 Table. ‘M’ indicates ‘MIRE’- the set of reference data and partly overlaps with other community data. When site identity was included as supplementary variable it accounted for 22.2% variation in data (adjusted).

We gathered monitoring vegetation records from 38 sites, 18 with RE and 21 with TSR, distributed across Western and Central Europe (Fig 1 and S1 Table). Only several of the data sets included appropriate control plots. In total, records from 5476 plots with 837 species (vascular plants and mosses) were gathered, of which 1264 records were used (no temporal analysis was possible). Data sets covered 4 to 37 years, with a median value of 10 years. Extensive description of data selection and site’s characteristics are provided in S1 Supplementary Materials.

Restoration outcomes were compared with the characteristics of reference fen mires (MIRE). For this we used comprehensive data set from well-preserved groundwater-fed fen mires from North-Eastern and Central Poland representing a diversity of conditions and vegetation types [56]. Their vegetation was characterised by high coverage of mosses and the presence of plants associated with the classes of *Caricetalia davallianae* and *Scheuchzerio-Caricetea nigrae*, commonly with a dominance of *Carex rostrata*, *C*. *diandra*, or presence of tall sedge species. These fens were concluded to be peat-accumulating and hosted typical specialist species of fens. We explicitly choose this independent data set for reference, because it represented a non-degraded state, while many of the local references were to some degree impaired. Fen mires communities in Europe are similar, independent of landscape type [57], therefore, this data set can function as a proxy for reference PFTs profile. We considered it useful to set a course for restoration and to project it together with the results of restoration, as an indication. In our approach, the potential problem with difference in species pools between regions are overcome by the use of PFT’s instead of species identities.

### Aggregation of the trait data to the community level

Different scales used to quantify species abundance were standardized for all records and translated to percentage-abundance, and all species names were standardised according to Flora Europaea species list. Vegetation records were translated into PFT values and expressed as community means (CMs), community weighted means (CWMs), and functional ranges (frange), using trait estimates from TRY database [58]. Hence, these analyses address shifts in composition and abundance of species and do not account for intra-specific variation in PFTs. In CMs, all species are contributing to the means equally, while in CWMs with weighing by abundance, dominant species determine the values. According to the mass-ratio hypothesis, traits of abundant species affect strongest the processes in ecosystem [59, 60]. Consequently, the two metrics denote different information. CM indicates species filtering and signals selection for some trait values, whereas CWM indicates possible effects on ecosystem processes. CWMs and CMs were calculated without bryophytes, because of low data coverage and a different evolutionary meaning of their traits than in vascular plants. Also, phanerogams were excluded from those calculations, because in most cases they occurred as juveniles and their representation with traits characterising adult plants would be misleading.

A single–trait diversity index of functional range (frange) was calculated for each quantitative (numeric) trait, as the range of trait values occurring in a vegetation record, divided by the range of the trait within the species pool of all the data set [55]. Frange indicates the strength of the filtering process, as filtering reduces the range of trait values available in the regional species pool [48]. For describing FD, multiple-trait indices were used, namely functional evenness (FEve), FRich, FDiv, and FDis, which is similar to Rao index [49, 52]. Full details of calculations of trait characteristics and indices based on PFT were described in S1 Supplementary Materials.

### Selection and processing of PFTs

We selected PFTs that are related to the main stressors or are likely to respond to the changes in abiotic and biotic conditions due to RE and TSR. As elaborated above, we used W Ellenberg number as an indicator for plant tolerance to anoxia. Further we used PFTs, that are related to nutrient acquisitions strategies i.e. SLA, LDMC, Leaf N content and Leaf P content. Regarding competitiveness, we selected canopy height (ch), onset of flowering (fl) lateral clonal spread (cs) and hummockness (h)(ability to form hummocks and tussocks). In addition, we used CSR strategies [32, 61], as an indicator of general patterns in trait sets. Concerning mycorrhizal traits we divided species in non-mycorrhizal (MStatusN), obligatory–mycorrhizal (MStatusO) and flexible species (MFlexi), which grow with or without connection with mycorrhizal fungi. We also consider several PFTs that are related to reproduction, probability of re-establishment and dispersal. We used seed mass (SM), seed number per ramet (SNB), seed buoyancy, dispersal syndromes and number of adaptations to dispersal vectors (diversity dispersal syndromes). Also, a contribution of relevant ecological groups and life-strategies in the vegetation were used. For a detailed list of PFTs, along with data type, scales, pre-processing, data sources and data coverage see S2 Table. Also, the traits that had to be skipped from analyses due to low data coverage were indicated in S2 Table.

### Analysis approach

Depending on the context, a successful outcome in one project can be similar to a degraded state in another. CM and CWM data were aggregated per site in order to avoid pseudo-replication and over-representation of sites with many records (each site becomes a replicate), and then combined to estimate the effect of RE and TSR, in comparison to reference mires (MIRE). For the subset of data from permanent plots (paired samples, 322 plots) an estimation of an absolute change was calculated as a simple difference between the value before and after RE or TSR for a more accurate assessment of an effect on quantitative traits. Due to large differences in the number of records and monitoring set-ups, an application of a rigid statistical tools was not possible with this heterogeneous, unbalanced data set, where we could not account for external or site-specific factors. We inclined to the recent criticism of the overreliance on null hypothesis testing in scientific reasoning in the ecological studies [62, 63]. To focus on quantitative rather than qualitative methods of comparisons, we used confidence intervals (CI) approach to explore this data. This provides information by how much the parameter after restoration may differ from degraded or reference states, ranges of the observed effects, and what are the limits of our knowledge about this difference. Although 95% CI is the common standard, under more flexible approach and due to various considerations quite different confidence levels are being used [64]. Analyses in this study have an explorative character and we expected high level of variability (noise), preventing precise estimation of effects with very high confidence. Therefore, we choose the 80% confidence level.

We also applied multivariate analyses with ordination techniques to explore the patterns of species composition and of PFTs combinations (for details see S1 Supplementary Materials). This approach was justified as both species and PFTs were not independent of each other, but co-varied, due to positive relations and trade-offs. We performed a classical ordination analysis with Detrended Correspondent Analysis (DCA) on species composition and abundance data with records from restored sites and reference sites, to explore the general patterns of similarity in the vegetation and change due to restoration measures. Next, we used Principal Component Analysis (PCA) using the CMs of PFT for exploring the response in functional spectra. PCA analysis is sensitive to the similarity in trait composition. By using CMs it also emphasizes changes in the assembly processes, as changes in environmental filters benefit or imped species with particular sets of traits [23] (and references within). In PCA, we used all available PFT information except data that were omitted (S2 Table). No weighing of the input data was applied. We used the ordination scores, averaged per site and treatment, and the data from before restoration and from the last year of observations, for a graphic representation of shifts in vegetation or composition of PFTs. Multivariate analysis was used to confirm if data covered a variety of species combinations as data sites were distributed across a broad climatic and geo-botanical gradient. Also, we could confirm the overall effects of the restoration measures, explore a variation in the response to these measures and infer which environmental gradients or sets of PFTs were the most important.

We used multiple lines of evidence, inspecting individual traits, FD, as well as multivariate analysis, to check the consistency of restoration effects on plant community responses and coherence with other studies.

All computations of PFTs were made using R open source software [65]. For further analysis and presentation of the results we used STATISTICA 12 (StatSoft) and Canoco 5 for Windows (Microcomputer Power).

## Results

### Shifts in species composition

As revealed by a species-based DCA, our data set was characterized by a long compositional gradient of 9.0 SD units. Data were not clustered due to geographical location or applied restoration methods (Fig 1). The two main gradients of DCA based on plant species ecology can be interpreted as nutrient availability and differences in moisture or inundation (S1 Fig). Species-based DCA ordination indicated a shift towards the reference, but sites with RE and TSR differed (S2 Fig). We concluded that a shift in species composition indicated inundation and eutrophic condition after RE, while the shift towards reference was more evident after TSR, with no signs of eutrophication or inundation, indicating however drier conditions in several cases. Details of the results of multivariate analysis are provided in S2 Supplementary Materials.

### Shifts in functional traits

**Fig 2:**
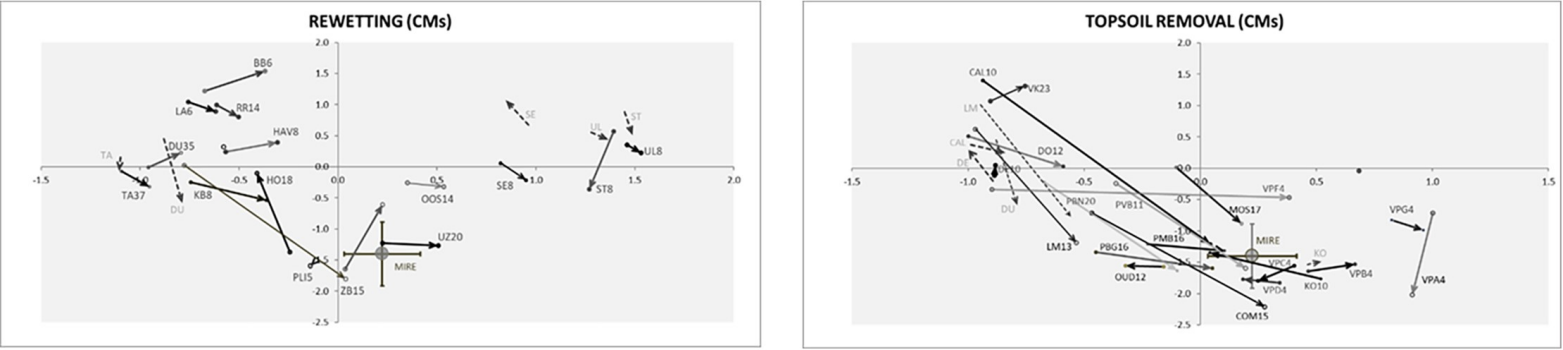
Graphs illustrating the shift in the trait space after restoration with Rewetting and Topsoil removal. Only the situation before restoration and the last year of observation were plotted. The community means (CMs) for PFTs, aggregated per site, were used as ordination variables in this PCA analysis. Arrows connect the two points and indicate the direction of change. Graphs are constructed using scores of samples for first two axes from PCA analysis, which explained cumulatively 39.5% and 47.3% of variation in data on first and second ordination axis (eigenvalues of 0.395, 0.08 respectively, short gradient of 1.3 SD unit). All available PFT data were used for PCA analysis, except these that were omitted due to insufficient data coverage (see S2 Table). For quantitative traits, the standardised values were used. Scores were aggregated (average) per site x treatment x time combination. For MIRES (reference records), the score average and the standard deviation were plotted (error bars), to indicate a range of values. For site symbols see S1 Table. The number behind the site letter code indicates a number of years since the restoration measure was applied. Stripped arrows and grey letter codes indicate the controls when these were available. For the controls the age was not indicated as it is the same as for corresponding restored sites.

We used PCA for exploring shifts in the functional composition after restoration (justified by short gradient of 1.3 SD units). PCA analysis explained 47% of variation in functional data on the first two axes (Fig 2, see also S3 Fig). A graph representing TSR showed a more clear shift towards the reference values, indicating a response for both anoxia- and nutrient limitation-related traits, as compared to RE plot.

**Fig 3:**
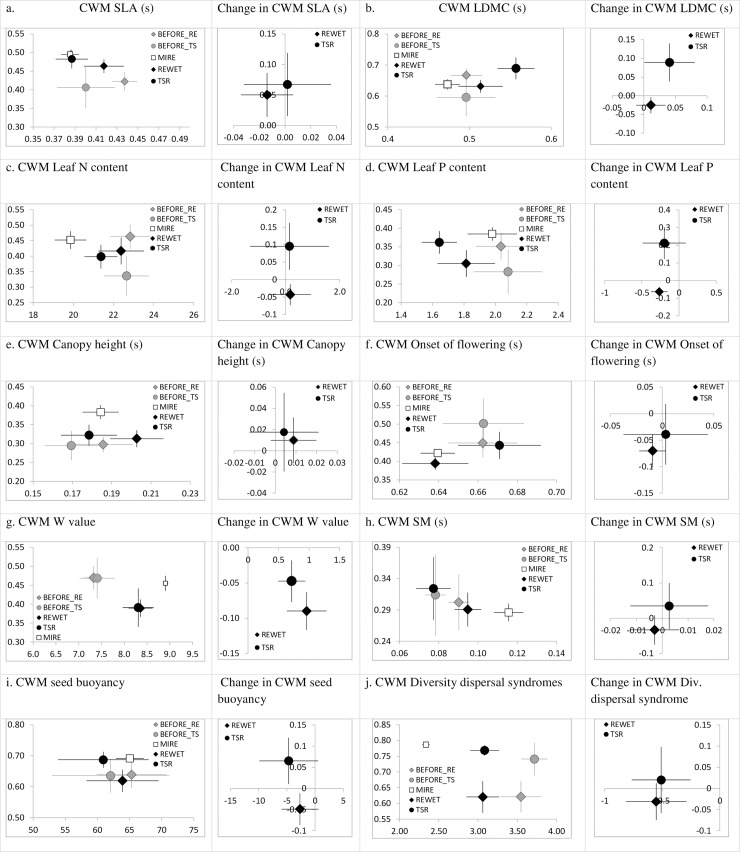
Results of the quantitative compressions in selected PFTs. On the X axis the mean value of the trait was plotted, while on the Y axis the mean value of the functional range (frange) of the trait were plotted. Error bars indicate 80% confidence interval. Change in PFT values was calculated based on a set of 322 paired samples, then aggregated per site and later aggregated per treatment (14 sites with RE, 11 sites with TSR). (s) indicates standardized values (between 0 and 1) in this data set. Symbols: BEFORE_RE–characteristics of PFT in degraded state from the sites where rewetting was applied; BEFORE_TS—characteristics of PFT in degraded state from the sites where topsoil removal was applied; REWET—characteristics of PFT after rewetting (last year of observations); TSR—characteristics of PFT after topsoil removal (last year of observations); MIRE—characteristics of PFT in reference sites (peat forming plant communities, typical for fens, with occurrence of fen specialists). For rewetting and before rewetting N = 18, for topsoil removal and before topsoil removal N = 21, for Mire N = 37.

The results for CWMs and CMs in most quantitative PFTs were similar. As we considered CWMs more relevant for the ecosystem-level functions assessment, these characteristics for individual PFTs, together with their frange and results for absolute change, were presented on panel in Fig 3. All abbreviations of traits and data characteristics are provided in S2 Table. All CM results are presented for a comparison on a panel in S4 Fig. The frange values were independent of the method of averaging. All results for qualitative traits (seed number, mycorrhizal status, clonal spread (ordinal data), contribution of CRS strategies, ecological groups and dispersal syndromes in the vegetation) are presented on panel in S5 Fig. The most important results are highlighted in the following sections. Not all results are described in detail, due to space limitation. For qualitative traits, CWM data were used as only relevant parameters and frange values were irrelevant. Exceptions were the seed number, mycorrhizal status and the dispersal syndromes. For mycorrhizal status and seed number we considered information on both CM and CWM as relevant, hence this data was presented together (CWM plotted vs. CM). For dispersal syndromes only CM values are used, as these are indicative for dispersal limitation linked with various dispersal vectors. Information on several pairs of traits was considered complementary and therefore the results are presented together. This is the case for life strategy C and S, vegetative growth (cs and h), contribution of bry and pha, contribution of SPH and BM, contribution of CY and PO (as both groups can be and play similar role in vegetation structure), contribution of FO and PT.

### Change in PFTs related to stress and competition

An ability to survive and grow in anoxic conditions, which is related to Ellenberg moisture values, was clearly enhanced after restoration, but the reference values were not achieved (using 80% confidence criterion) (Fig 3G). We also observed a clear lowering of frange of this trait after both methods. Considering the absolute change in CWM of this trait, there was no difference between TSR and RE, whereas the absolute change in CMs indicated a stronger effect of RE than of TSR.

The two PFTs related to nutrient acquisition, namely SLA and LDMC, shifted towards values typical for nutrient-poor conditions and more conservative nutrient strategy after TSR, whereas no indication for such a shift was observed after RE (Fig 3A, Fig 3B). The reference values for CWM SLA were achieved after TSR (based on 80% CI) but not after RE. For SLA the functional range increased after both methods, becoming more similar to the reference situation. For CWM LDMC the reference values were not well-distinguished from situation before restoration, but it increased after TSR, even above values of reference mires. The functional range of LDMC increased after TSR, and slightly decreased after RE. We did not observe a response in CWM values of leaf N and P contents (Fig 3C, Fig 3D). Interestingly, there was an indication for a slight shift towards reference situation in CM values of these two traits. For P content, the CM values decreased after TSR, while frange values for this trait increased falling in the range of reference values (80% CI). This was not observed after RE. For N content, the CM values after TSR decreased and were lowest and closest to reference values (though they were not achieved). We did not find any response of the canopy height after restoration (Fig 3E). The only response of the onset of flowering in the community was the decrease of frange values after RE (Fig 3F), which indicated a shift towards reference values (80% CI). This could indicate a stronger filtering of late-flowering species after RE.

The share of non-mycorrhizal species in the community (CWM) was highest in non-degraded mires and increased after RE (although did not reach reference values based on 80% CI), whereas no increase and a larger spread in values was observed after TSR. Yet these differences were less clear if CM values were considered, indicating that the number of non-mycorrhizal species increased after both measures (S5C Fig). The contribution of competitive species (C-strategy) in plant communities decreased after restoration, whereas stress-tolerant species (S-strategy) increased, but only TSR resulted in values overlapping with reference fens (S5D Fig).

CWM of clonal spread rate was higher after RE than in reference fens (with 80% confidence), but there was no clear difference between communities before and after restoration, neither between sites restored with TSR and reference fens (S5E Fig).

We observed no changes in contribution of Bryophytes and Phanerophytes after restoration (S5F Fig). Contribution of Bryophytes was high in reference mires, intermediate in areas with TSR and low in rewetted areas (both before and after restoration). Ecological groups of brown mosses, sedges and grasses clearly showed differences between non-degraded and degraded fens (S5G and S5H Fig). Whereas the contribution of sedges and rushes in vegetation was enhanced after restoration (after TSR reached values of reference mires), proportion of the most important builders of rich fens—brown mosses, wasn’t. The contribution of grasses decreased after restoration, but the observed values were highly variable and did not overlap with low values typical for mires (with 80% CI). The restored sites had predominantly a meadow vegetation structure with 20–40% of grasses. We also observed a low contribution of ferns after restoration, whereas they were more frequent and abundant in reference fens (S5I Fig). We found a very low contribution of N-fixing plants in reference mires, while such species were more represented in degraded and restored sites (S5J Fig).

### Change in PFTs related to dispersal and recruitment potential

We found no indication of a shift towards reference values in SM, neither in TSR, nor in RE (Fig 3H). Mire plants have generally larger seeds than those growing on degraded or restored fens [27]. The seed number CWM, generally low in mires, was higher in degraded fens and did not change after restoration. For degraded and restored systems this parameter was also more variable than for mires (S5A Fig). After TSR more species with low seed number were found (effect observed in CM), while no effect was observed after RE. The number of adaptations to dispersal vectors was lowest in reference mires, highest in degraded fens and intermediate after restoration (Fig 3J). A shift towards reference values was found after restoration, but the values were still larger than in reference fens and the estimated intervals did not overlap. Judging from a large overlap of 80% CI, there was no meaningful difference in absolute change after TSR and RE regarding the number of dispersal adaptations. There was no clear pattern of response in trait value of seed buoyancy (Fig 3I). We found an increased contribution of autochory after TSR (but not after RE), a tendency for a decrease in species dispersed by men and by wind after restoration (while for mires low values were observed), and an overall high number of species dispersed by animals in both degraded and restored fens (while values for mires were low) (S5K and S5L Fig).

### Functional diversity

**Fig 4:**
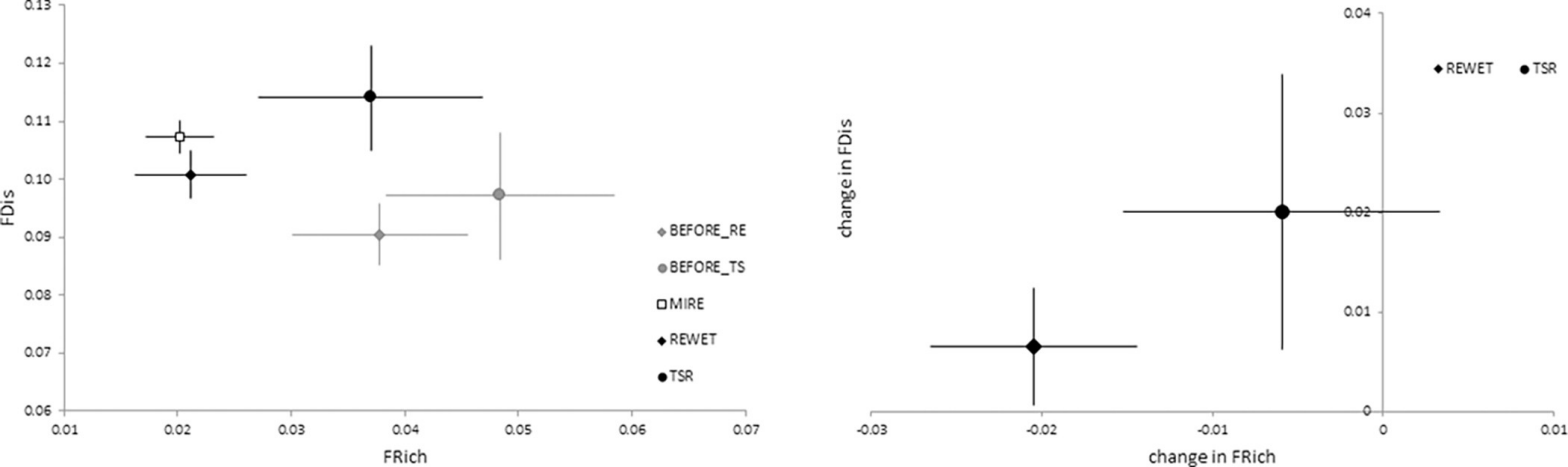
The values of selected functional diversity metrics and the (absolute) change after RE and TSR. Left: FRich (X) vs FDis(Y). Right: Change in Frich (X) and FDis (Y); Error bars indicate 80% CI. Meaning of symbols explained on Fig 3.

We only found a differentiation between non-degraded, degraded and restored fens in FRich and FDis. FRich and FDis explained also the most variation in data along the first two PCA axes with all functional diversity indices (not shown). FRich was lower in reference systems than in degraded fens, and it was reduced after RE, while TSR did not have such effect (Fig 4). The variability of values (range of 80% CI), both in FRich and FDis was larger in the degraded fens and after TSR, than in the reference fens. FDis increased after restoration. When an absolute change in FDis was assessed, an increase was stronger after TSR, while after RE the change was only marginal, and, in general, the CI (evaluated with 80% CI criterion) in RE and TSR overlapped (Fig 4).

## Discussion

Restoration outcomes are difficult to generalize, as they depend on many factors [11]. By using PFTs, entire functional spectra and multivariate analysis, we were exploring the general response to restoration. In multivariate analysis we found a larger and more consistent shift towards reference situations after TSR than after RE, determined by a shift towards nutrient-poor and permanently wet conditions. This confirmed our hypothesis 1 and partly hypothesis 2. We found that the shift in traits related to reproduction after restoration was opposite than during fen degradation [27]. As expected, we found indications of dispersal limitations after restoration, which partly confirmed hypothesis 3, but we did not detect any differences between RE and TSR in this respect. This suggests that TSR is effective in enforcing a shift in traits related to nutrient acquisition strategies, towards values similar to non-degraded fens (particularly when species are used as indicators, with CMs). The effect of TSR could be enhanced due to higher establishment of low-competitive mire specialists from soil seed bank or higher microsite availability. After RE, non-adopted species disappeared but, as the new colonization was limited by a closed canopy, this resulted in lower species richness, restricted values for some PFT’s permitted in vegetation (lower range), and species pool representing a limited array of life strategies. The two restoration measures—TSR and RE primarily affect two different aspects of functional characteristics, respectively *value* and *range*.

As already identified in the introduction, PFTs cannot be easily separated into those responding (only) to anoxia stress, nutrient availability stress, or competitiveness, as PFT are interrelated due to inherent trade-offs and evolved not independently in plants’ adaptations. Below we discuss results in individual traits and FD, in reference to the effectiveness of the two restoration methods on with regard to re-instalment of anoxia and nutrient stress and to the limiting effects of seed dispersal constraints.

### Efficiency of restoring anoxia

The results for traits linked with anoxic stress did not point unwaveringly at a success of restoration. RE resulted in stronger lowering of the functional range of Ellenberg moisture values [6,66], p ossibly due to elimination of species not adopted to constantly high water levels or frequent inundation. Indirectly, an increase in general stress indicators such as SLA (see also next section) might also be due to increased anoxia. Yet, characteristics, such as proportions of browns mosses, sedges and grasses pointed out that the level of anoxic stress after restoration did not match the reference situation.

An increase of non-mycorrhizal species and a decrease of obligatory mycorrhizal species observed after RE probably reflected a higher or more frequent inundation [67], but the proportion of non-mycorrhizal species typical for mires was not reached in restored sites. Concluding, the intensity of anoxia stress matching reference mires was not reached in restored systems. Contrary to our hypothesis 2, the results for RE and TSR were similar. This is in line with studies showing that hydrological regimes and the vegetation after restoration are different than in non-degraded mires [68], while other studies demonstrated that the mire specialists associated with anoxic conditions, were found after TSR [69, 70].

### Efficiency of restoring nutrient stress

As expected, TSR triggered a response in traits linked with nutrient acquisition strategies, indicating a decrease of nutrient availability, but RE did not. This can be related to a direct effect of the measures: while TSR removes nutrients locked in the top layer of mineralizing peat physically, RE may cause mobilization of phosphates bound with iron-complexes [2, 12]. Additionally, biomass decomposition after die-off of inundated vegetation provides plant-available nutrients [71]. When rewetting with polluted river water nutrients could also have come from outside of the restored area, whereas P could additionally be remobilised due to sulphate input [2].

A successful reduction of nutrient availability by TSR could be detected from the PFTs response, mainly SLA, LDMC and leaf N content.

After restoration, particularly after TSR, there was a tendency for lower values and a larger functional range in SLA, which could indicate less competition pressure after TSR. This is in agreement with Emsens et al. [18], who reported a higher light availability at the soil surface, associated with a higher species diversity after TSR. Inclusion of low SLA values and a large variation in values in degraded situations is probably related to potassium limitation observed in desiccated peatlands [72]. LDMC proved useful for revealing nutrient strategy, due to relatively low plasticity [73].

A higher LDMC, lower leaf P content and more species with a low leaf N content were found after TSR, placing it closer to reference mires. Lack of effect in P or N leaf content after RE was possibly due to enhanced P availability [2, 13]. We concluded that TSR was effective in enhancing conservative nutrient strategy and a low litter decomposability, both typical for nutrient-poor habitats. This explained also a stronger decline of C- strategy and an increase in S-strategy after TSR than after RE. Various PFTs, i.e. SLA, LDMC, and, to some extent, seed investment have been also linked to N:P ratio and suggested as indicative for P-limitation [74]. A response in those traits would suggest a possibility of P-limitation after TSR, but not after RE.

### Efficiency of restoring low competition intensity

A response in the individual PFT related to competitiveness was limited. We did not find any response in the canopy height, which was probably related to a high plasticity and dependence on the site productivity. The degraded and restored systems had a uniform vegetation structure, whereas the reference mires had a diverse structure in terms of plant height (larger functional ranges), indicating a low selection pressure for that trait. Canopy height is often used as an indicator of competitive pressure and is negatively related to species diversity in fens [8]. We found no differences in reference fens, degraded and restored systems, regarding clonal spread or in species ability to form hummocks. Several authors indicated that the habitat heterogeneity is limited in natural fen mires, which reduces the probability of establishment of invasive or expansive species [75, 76]. A large variation and the lack of clear response in those traits could also have resulted from management.

Sedges, typical fen dominants, are wind-pollinated and characterized by early onset of flowering. An advantage of early flowering in high-productive fens is related to higher chances of subordinate species to produce seeds under moderate shading from the standing biomass that increases later during the growing season [8]. In our study, the frange for onset of flowering after RE had decreased and became even lower than in reference fens. This suggested a strong filtration for an earlier flowering, possibly due to a strong competition during the growing season under uniform, dense canopy. This corresponded with a lower contribution of bryophytes after RE, compared to their high abundance in reference mires and after TSR.

Concluding, restoration affected the PFTs linked to nutrient acquisition strategies and enhanced the similarity to non-degraded systems, but TSR was more effective than RE (in accordance with hypotheses 1 & 2).

### Is dispersal an obstacle in restoring fens?

Seed traits, although not directly related to environmental stressors in mires, have been indicated as crucial in vegetation recovery after restoration [21, 47, 77]. The constrains of dispersal or an absence of seeds may be equally important as abiotic constrains [78, 79]. Specialist mire species usually have a low number of large seeds and are poorly adapted to long-distance dispersal [74, 80]. We found high values for SM, low values for SNB and a low number of dispersal syndromes in reference mires. We observed no change in CWMs for SM and SNB after restoration, indicating that restored systems are hardly changing regarding reproductive traits (supporting hypothesis 3). We expected to find differences in seed traits between RE and TSR, as after TSR the vegetation development depends strongly on the soil seed banks or rapid colonization of bare soil. High seed production, numerous dispersal adaptations, smaller seeds (and high seed longevity) are generally related to the soil disturbance [43, 81]. We expected to find these characteristics in degraded systems, as well as after TSR. Instead, we found more species with low seed number and less diverse dispersal adaptation after TSR, which pointed out that a substantial reduction in nutrient availability controlled species assembly and the effect of disturbance disappeared over time (opposite to our hypothesis 3). In terms of the number of adaptations to dispersal vectors, restored systems shifted towards the reference, but did not reach values characteristic for mires and the same was true for the spectrum of dispersal syndromes (supporting hypothesis 3). We found a decrease in the functional range of seed buoyancy after RE, which could result from inundation favouring floating seeds. Seed buoyancy enhances hydrochory [46, 82], but possibly it is relevant in riparian wetlands, but not in groundwater-fed fens with limited lateral water flow.

Fujita et al. [74] proposed that a low plant investment in reproduction through seed in mires is a by-product of P shortage conditions, but it could also be an adaptation to a naturally isolated occurrence in the landscape, for which a long-distance dispersal might be a disadvantageous strategy [83]. In any case, this impeded dispersal and re-colonization in restored sites and therefore many mire plant specialists may contribute to the ‘dark’ diversity in the contemporary landscapes [83, 84]. As we used observations of matured vegetation, with low chances of establishment from seeds [85], it can be assumed that species with typical mire seed traits are absent from restored fens even after a long time, suggesting that the ‘colonization deficit’ [21] can be a widespread phenomenon in fen restoration.

### Effects of restoration on functional diversity

In our study only FRich and FDis responded to drainage and restoration, but these two indices were identified by Mason et al [52] as the best to explore assembly processes along stress gradients. Both FRich and FDis shifted towards non-degraded mires after restoration, when compared with the degraded systems (confirming our hypothesis 4), however the direction of this shift was opposite. FRich was lowest in reference mires, which is in accordance with Hedberg et al. [26] and consistent with the theoretical framework proposed by Garnier et al. [86], see also [48, 51]. We interpreted this as an outcome of strong environmental filtering in well-functioning fens, resulting in trait convergence of co-occurring species. The more intensive environmental stress ‘experienced’ by plants after restoration, the more probable is that the fens will become functionally similar to reference sites and long-term stable. On the contrary, FDis was higher in reference mires than in the degraded ones, indicating that different strategies are more equally represented in the earlier. An increase in FDis can be interpreted as an increase in differences between individual species and the community mean. This indicated more heterogeneous functional characteristics within the limits of what the environmental filters allow. An increase in FDis may suggest more different mire-specialists establishing. After restoration there was a tendency for a decline in FRich, but mainly after RE, and an increase in FDis, although the later was less pronounced.

A decrease in FRich pointed out to less diverse trait values permitted in wetter sites, which was also observed in some individual traits (after RE). Partly, this can be related to a lower species richness after RE [49]. After TSR such an effect was small, or some traits showed an increase of functional ranges, reflecting a less intensive filtering. The restoration outcomes were characterised by a large variability in responses, possibly related to an insufficient improvement of abiotic or priority or legacy effects affecting community assembly [87]. High species diversity is often believed to be a pre-condition for a good ecosystem functioning [53]. Trait divergence and high functional diversity were suggested as necessary for coexistence of many species, although this was mostly demonstrated in grasslands [55, 88]. In our study, we did not find a clear indication for a trade-off between species diversity and functional diversity though a more in-depth exploration is needed.

### Reliability of PFT’s information and limitations of this study

Despite a large trait database, data for some fen species was not available or insufficient. In general, more accurate and habitat-specific measurements and traits better suitable for examined processes are needed to further improve the analysis. A lack of response or its large variation could be related to the accuracy of estimations, especially for plastic traits in extreme habitats [73]. Functional ranges and FD indices need to be interpreted with caution and in relation to individual traits. The differences in the FD or CWMs within one ecosystem type are expected to be small [24, 89]. Different than in experimental study, we included the entire variation of responses under field conditions and site-dependent contexts. Understandably, results varied strongly between sites, but despite these shortcomings we could detect general patterns of response to restoration.

### Way forward for fen restoration

Restoring functional fens seems more difficult than once thought. Restoration resulted in PFT composition more similar to the reference mires than in degraded peatlands, but changes over time were often not directional and the restored systems still did not function in the same way as the original ones. TSR induced a response in traits related to nutrient acquisition strategies, indicating nutrient impoverishment and prompted establishment of fen specialists. RE resulted in a lower functional richness and indicated a stronger filtering in some traits. Response in individual PFTs also suggested eutrophication effects and a stronger competition after RE. Our results suggested that targeting and maximizing only one type of an environmental stressor, or broader-seen, removing only one type of constrain, does not lead to a quick recovery of functional fens. Restoring the hydrological regimes up to the level of thresholds that allow ecosystem recovery is necessary. Regarding an applied measure, RE or TSR, or both should be considered for successful restoration in different sites. If the site has been moderately or only recently drained, and the stress related to nutrient availability was not strongly relaxed, applying rewetting as a sole measure could be sufficient. If both hydrological conditions and nutrient limitation have substantially been modified, also in the surrounding landscape topsoil removal and seed addition can be more effective [11, 22, 90]. Different restoration strategies are also justified by different socio-ecological contexts [3]. TSR is more costly in application, but rewetting requires more land under nature management and has a bigger risk of compensation claims. Due to an extent of transformation in hydrological systems and socio-economic constraints, the re-establishment of hydrological regimes relevant for fen mires is hardly feasible [91] or the applied measures are moderately effective [92].

Usually, the intensity of environmental stresses matching non-degraded mires was not achieved. Partly guilty may be our tendency to optimize restoration for high species diversity (e.g. slightly drained and managed fen meadows), which provides insufficient level of environmental stress to restore stable and functional fen ecosystems.

Very often it is argued that a long time is needed for restoration. Long-term studies (>10yr) with regular observations are extremely rare but without such data an assessment of the functional characteristics of vegetation is hardly possible. In this study we used medium to long-term observations, which, to our knowledge are the longest monitored projects, relevant for the contemporary circumstances, such as declining abiotic quality (e.g. nitrogen deposition) and declining biodiversity. We reckon that, in general, the restoration efforts do not fully ensure the enhancement of functioning and long-term persistence of fens. More rigid actions, when setting the expected levels of the environmental stress, are needed. Still, however, one should also acknowledge the positive message coming from our results. Finding restored fens half-way between the degraded and the near-natural ones shows that we are going into the right direction and restoration efforts are rewarded with some success. What we need now is to upscale and sharpen our approach, e.g. by cessing to compromise restoration of fen ecosystem functioning with maximization of species richness in semi-natural systems.

## Supporting information

S1 Supplementary Materials Details of the methods. (DOCX)

S2 Supplementary Materials Results. (DOCX)

S1 Table Site characteristics of the fen restoration projects. Peatland characteristics (system type, presence of gyttja, size, depth of peat, peat type and catchment characteristics) were provided by authors. Climatic characteristics were based on the open access European Environmental Agency data on Precipitation and Evapotranspiration. Column descriptions: max. depth—maximum depth of peat in meters; T–mean annual air temperature in degree Celsius, P—mean annual precipitation in millimetres, T winter—mean winter air temperature in degree Celsius, T summer—mean summer air temperature in degree Celsius.
(DOCX)

S2 Table Characteristics of PFT data used in the analysis. Log-transformation was applied to adjust the right-skewed frequency distribution. % data stands for data coverage and indicates for how many species the trait estimation was available (in total 828 species).
(DOCX)

S1 Fig Results of DCA ordination analysis for species (75 best fitted species plotted). (TIF)

S2 Fig Graphs of the shift in species composition after restoration with RE and TSR. (TIF)

S3 Fig Graph illustrating the main gradients with the related PFTs and the (expected) shift in the trait space after restoration with RE and TSR. (TIF)

S4 Fig Results of the comparisons in selected quantitative PFTs. (TIF)

S5 Fig Selected results of comparisons in qualitative traits between non-degraded (MIRE), degraded (BEFORE_RE, BEFORE_TSR) and restored fens (after rewetting—RE or topsoil removal—TSR). (TIF)

S1 File Basic data for CWM, CM and frange values of the PFTs. (XLSX)

S2 File Basic data for CWM values of the PFTs. (PDF)

S3 File Basic data for CM values of the PFTs. (PDF)

S4 File Basic data for frange values of the PFTs. (PDF)
