# Supplementary material for: Are we restoring functional fens? – The outcomes of restoration projects in fens re-analysed with plant functional traits
Source: PLoS One. 2019 Apr 24;14(4):e0215645. doi: 10.1371/journal.pone.0215645 (PMC6481837; doi:10.1371/journal.pone.0215645)
Supplement: S1 Supplementary Materials — (DOCX) [file pone.0215645.s001.docx]

**S1 Supplementary materials - Details of the methods**

Data selection and processing

This study aimed at detecting the main patterns in response of PFT after restoration in fen ecosystem, by re-analysing the monitoring data from multiple projects. This allows for synthesizing results from different sites and to generalize for wide range of conditions. In some analysis the results are relative and had to be computed for entire data set. We selected only those data sets, where (i) full vegetation relevés from before the restoration were present, (ii) data were collected over time in fixed places (permanent quadrats method) or at least the vegetation representative for the situation before restoration was sampled, (iii) observations after/over longer time were available – minimum 4-5 yr and optimum 10 yr or longer, to only include relatively mature and stabilized communities (see also Klimkowska et al. 2007). For full list of sites and their characteristics see S1 Table .

As many data series from the same locations as possible were gathered to account for the variation in the vegetation and a patchy character of the vegetation. In this way we hoped also to minimalize a bias related to a strong dependence of restoration outcomes on the situation before the restoration (Klimkowska et al 2007) and dependence of outcomes of some analysis on species presence and abundance. In trait characteristics calculations these data were later aggregated (by calculating a mean value), in order to avoid pseudo replication, for site x treatment for situation before and after restoration.

Data of reference fen mires were selected randomly from a database (Pawlikowski et al. 2013) of 170 relevés characterizing mires in North-Eastern and Central Poland. In total 60 relevés from 37 sites were selected (no more than 3 from one fen site), so the number of records was similar to other data sets and a full variation in vegetation, species richness and sites, with no subjective pre-selection, was included. At reference sites the communities typical for rich fens and peat accumulation process were occurring, indicating stable, high water levels, with no signs of degradation.

**S1 Table. Site characteristics of the fen restoration projects.** Peatland characteristics (system type, presence of gyttja, size, depth of peat, peat type and catchment characteristics) were provided by authors. Climatic characteristics were based on the open access European Environmental Agency data on Precipitation and Evapotranspiration. Column descriptions: max. depth - maximum depth of peat in meters; T – mean annual air temperature in degree Celsius, P - mean annual precipitation in millimetres, T winter - mean winter air temperature in degree Celsius, T summer - mean summer air temperature in degree Celsius.

| No. | code | Site | Coordinates | RE/TSR | System type | Gyttja 0 /1 | max. depth [m] | T | P | T winter | T  Summer | Soil/ peat type | Catchment | References |
| --- | --- | --- | --- | --- | --- | --- | --- | --- | --- | --- | --- | --- | --- | --- |
| 1 | BB | Biesenbrow | 53°07'56"N,  13°59'37"E | REWET | River valley system | 1 | 2 | 7.67 | 551.4 | 0.98 | 15.56 | Week groundwater seepage, not continues gyttja , peat 1-2 m deep, sedge type | long-term deforested, predominantly intensive agriculture use | Timmermann 1999 |
| 2 | CIE | Cieszynka | 53°05'42"N, 16°00'18"E | REWET | Lake basin | 1 | 1 | 7.37 | 585.6 | 0.89 | 15.65 | gyttja 6-8m deep, moss- sedge peat | Forest | Wołejko et al. 2001; Stańko, R.  unpublished data |
| 3 | DU | Dümmer Lake | 52°29'07"N, 8°19'26"E | REWET | Lake basin | 1 | 1.25 | 8.31 | 800.9 | 2.81 | 15.37 | sedge -reed peat | intensive agriculture use | Blüml 2011; Ganzert & Pfadenhauer 1988 |
| 4 | HAV | Havel | 53°27'8.5"N,  12°56'59"E | REWET | Lake basin | 1 | 4 | 7.25 | 571.2 | 1.21 | 15.62 | Heavily degraded peat, moss- sedge peat | predominantly intensive agriculture use | Vegelin, K. 2014, unpublished data |
| 5 | HO | Hohner See | 54°17'54"N,  9°28'03"E | REWET | Lake basin | 0 | 2 | 7.67 | 829.5 | 1.94 | 15.52 | sedge -reed peat | intensive agriculture use and build-up areas | Schrautzer et al. 2013 |
| 6 | KB | Klosterland Benediktbeuern | 47°43'28"N,  11°22'28"E | REWET | River valley system | 1 | 6 | 6.62 | 1002.9 | -1.01 | 14.11 | On peatland - intensive agriculture, gyttja 3 m deep, sedge - 7reed peat | lower Alps, mixed land use: low intensity agriculture and forests | Geiger-Udod 2001;  Quinger 2009 |
| 7 | LA | Landgrobental | 53°40'38"N, 13°43'22"E | REWET | River valley system & Spring area | 0 | 6 | 7.58 | 555.8 | 0.98 | 15.56 | moss- sedge peat, sedge peat | predominantly intensive agriculture use | Vegelin, K. 2014, unpublished data |
| 8 | OOS | Oosterschar | 52°54'57"N,  5°53'45"E | REWET | River valley system | 0 | 1.2 | 7.60 | 785.6 | 5.41 | 12.30 | clay-loam layer under peat, sedge peat | predominantly intensive agriculture use | Jager, H.J. unpublished data |
| 9 | PLI | Kosobudki | 52°14'48"N, 15°10'09"E | REWET | River valley system | 1 | 5 | 7.96 | 562.1 | 0.01 | 15.99 | sedge and woody peat, gyttja present locally | Forest, not transformed | Stańko, R., unpublished data |
| 10 | RR | Randow - Rustow | 53°56'28"N,  N13°04'50"E | REWET | River valley system | 1 | 7 | 7.72 | 557.2 | 1.08 | 15.59 | moss-sedge peat, sedge peat | predominantly intensive agriculture use | Vegelin, K. 2014, unpublished data |
| 11 | SE | Sevenmosses | 60°26'13"N,  17°57'35"E | REWET | River valley system | 0 | 1 | 4.87 | 577.0 | -5.88 | 12.75 | moss-sedge peat | Forest (coniferous) | Hedberg et al. 2012 |
| 12 | ST | Styggkärret | 59°57'35"N,  17°18'20"E | REWET | River valley system | 0 | 2 | 4.84 | 574.9 | -3.82 | 13.11 | moss-sedge peat | Forest (coniferous) | Hedberg et al. 2012 |
| 13 | TA | Taarlo | 53°02'20"N,  6°38'19"E | REWET | River valley system | 0 | 1.2 | 7.68 | 788.0 |  |  | sedge and woody peat | predominantly intensive agriculture use | Bakker, J.P. & De Vries, Y. 2014, unpublished data; Bakker 1989 |
| 14 | TR | Trebeltal | 54°6'50"N,  12°41'28"E | REWET | River valley system | 1 | 9 | 7.64 | 558.1 | 1.28 | 15.64 | moss-sedge peat, sedge peat | Forest and agricultural use | Vegelin, K. 2014, unpublished data |
| 15 | UL | Ultunaviken | 59°57'13"N,  17°18'21"E | REWET | River valley system | 0 | 3 | 4.91 | 573.4 |  |  | moss-sedge peat | Forest (coniferous) | Hedberg et al. 2012 |
| 16 | UZ | Vloweitje Urkhoven | 51°26'15"N,  5°32'17"E | REWET | River valley system | 0 | 1.5 | 8.39 | 775.5 | 4.42 | 15.90 | sedge peat, reed peat and woody peat | predominantly intensive agriculture use and build-up areas | van der Burg, R.F. 2015, unpublished data |
| 17 | ZB | Zwarte Beek | 51°05'43"N, 5°17'16"E | REWET | River valley system | 0 | 3 | 8.27 | 810.9 | 5.01 | 15.87 | moss-sedge peat, woody peat | Forest (coniferous) and agriculture use | Aggenbach, C. unpublished data |
| 18 | CAL | Całowanie | 52°00'40"N, 21°21'00"E | TSR | River valley system & spring area | 0 | 4 | 6.78 | 511.3 | -0.98 | 18.40 | moss-sedge peat | Forest (coniferous) | Klimkowska et al. 2010  & unpublished data |
| 19 | COM | Commissarissenlanden | 51°57'03"N,  4°44'09"E | TSR | River valley system | 0 | 10 | 8.41 | 793.3 | 5.01 | 15.87 | peatland is under low intensity use, sedge - woody peat | predominantly intensive agriculture use | Kerkhof, D., Reinink, W., van Heerden, A. 2013, unpublished data |
| 20 | DE | Delling | 48°03'14"N, 11°14'52"E | TSR | Spring area | 0 | 1.5 | 7.21 | 954.3 | -0.99 | 14.00 | sedge peat | predominantly intensive agriculture use | Sliva 1996; Sliva, J. unpublished data |
| 21 | DO | Donaumoos Dachsholz | 48°42'00"N,  11°11'00"E | TSR | River valley system | 0 | 1.5 | 7.61 | 769.4 | -0.99 | 14.00 | flow-through mire type, sedge peat, reed peat and woody peat | predominantly intensive agriculture use, also on peatland | Wild 1997; Patzelt 1998; Schachtele 2004 |
| 22 | KO | Korenburgerveen | 51°59'13"N,  6°39'53"E | TSR | River valley system | 0 | 1 | 8.29 | 777.9 | 3.84 | 15.92 | edge of bog, former leg zone, transition towards a local brook valley, groundwater 23influence, sedge -sphagnum peat | long-term deforested, predominantly intensive agriculture use | Van der Hoek & Heijmans 2007 |
| 23 | LM | Lage Maden | 53°0'50"N,  6°37'43"E | TSR | River valley system | 0 | 1.2 | 7.63 | 787.5 | 5.41 | 12.30 | sedge peat, and woody peat | predominantly intensive agriculture use | Grootjans, A.P. unpublished data; Klimkowska et al. 2015 |
| 24 | MOS | Mosbeek | 52°26'47"N,  6°52'13"E | TSR | Spring area | 0 | 0.5 | 8.47 | 776.8 | 3.84 | 15.92 | Moss - sedge peat | predominantly intensive agriculture use, forest | van Tweel 2014, unpublished data |
| 25 | NP | Nieuwkoopse Plassen | 52°08'46"N, 04°49'02" E | TSR | Lake basin | 0 | 1 | 8.52 | 799.8 | 5.01 | 15.87 | Intensive drainage (deep polders), sphagnum peat - reed peat | predominantly intensive agriculture use | van Diggelen et al. 2015 |
| 26 | OUD | Oudeland | 51°56'16"N 4°40'43"E | TSR | River valley system | 0 | 4 | 8.77 | 787.6 | 5.01 | 15.87 | peatland is under low intensity use, sedge peat, and woody peat | predominantly intensive agriculture use and build-up areas | Kerkhof, D., Reinink, W., van Heerden, A. 2013, unpublished data |
| 27 | PBG | Polder Berkenwoude Graafkade | 51°57'24"N,  4°41'49"E | TSR | River valley system | 0 | 10 | 8.35 | 785.9 | 5.01 | 15.87 | peatland is under low intensity use, sedge peat, and woody peat | predominantly intensive agriculture use and build-up areas | Kerkhof, D., Reinink, W., van Heerden, A. 2013, unpublished data |
| 28 | PBN | Polder Berkenwoude Nooitgedacht | 51°57'47"N,  4°42'30"E | TSR | River valley system | 0 | 10 | 8.67 | 790.0 | - | - | peatland is under low intensity use, sedge peat, and woody peat | predominantly intensive agriculture use and build-up areas | Kerkhof, D., Reinink, W., van Heerden, A. 2013, unpublished data |
| 29 | PMB | Polder Middelblok | 51°59'21"N, 4°41'28"E | TSR | River valley system | 0 | 10 | 8.05 | 786.9 | - | - | peatland is under low intensity use, sedge peat, and woody peat | predominantly intensive agriculture use and build-up areas | Kerkhof, D., Reinink, W., van Heerden, A. 2013, unpublished data |
| 30 | PVB | Polder Veerstalblok | 51°59'53"N,  4°43'01"E | TSR | River valley system | 0 | 3 | 7.72 | 791.0 | - | - | peatland is under low intensity use, sedge peat, and woody peat | predominantly intensive agriculture use and build-up areas | Kerkhof, D., Reinink, W., van Heerden, A. 2013, unpublished data |
| 31 | VK | Veenkampen | 52°00'26"N, 5°35'43.5"E | TSR / REWET | River valley system | 0 | 1 | 7.91 | 784.5 | 4.13 | 15.91 | peaty soils, with clay intrusion | long-term deforested, predominantly intensive agriculture use | Van der Hoek & Sykora 2006 |
| 32 | VPA | Suikerpot A | 52°13'11.5"N, 5°06'31"E | TSR | River valley system | 0 | 2 | 8.57 | 803.2 | 5.01 | 15.87 | sedge peat, and reed peat | predominantly intensive agriculture use and build-up areas | Beltman et al. 1999; Faasen et al. 2000; Beltman 2002 unpublished data |
| 33 | VPB | Suikerpot B | 52°13'12"N, 5°06'28"E | TSR | River valley system | 0 | 2 | 8.57 | 803.2 | 5.01 | 15.87 | sedge peat, and reed peat | predominantly intensive agriculture use and build-up areas | Beltman et al. 1999; Faasen et al. 2000; Beltman 2002 unpublished data |
| 34 | VPC | het Hol | 52°13'18"N, 5°05'01"E | TSR | River valley system | 0 | 2 | 8.57 | 803.2 | 5.01 | 15.87 | sedge peat, and reed peat | predominantly intensive agriculture use and build-up areas | Beltman et al. 1999; Faasen et al. 2000; Beltman 2002 unpublished data |
| 35 | VPD | Ster | 52°11'29"N,  5°06'28"E | TSR | River valley system | 0 | 2 | 8.59 | 802.5 | 5.01 | 15.87 | sedge peat, and reed peat | predominantly intensive agriculture use and build-up areas | Beltman et al. 1999; Faasen et al. 2000; Beltman 2002 unpublished data |
| 36 | VPE | Weersloot | 52°11'22"N, 5°07'14"E | TSR | River valley system | 0 | 2 | 8.59 | 802.5 | 5.01 | 15.87 | sedge peat, and reed peat | predominantly intensive agriculture use and build-up areas | Beltman et al. 1999; Faasen et al. 2000; Beltman 2002 unpublished data |
| 37 | VPF | Tienhoven | 52°10'09"N, 5°05'37"E | TSR | River valley system | 0 | 2 | 8.62 | 802.6 | 5.01 | 15.87 | sedge peat, and reed peat | predominantly intensive agriculture use and build-up areas | Beltman et al. 1999; Faasen et al. 2000; Beltman 2002 unpublished data |
| 38 | VPG | Molenpolder | 52°08'49"N, 5°05'28"E | TSR | River valley system | 0 | 2 | 8.62 | 802.6 | 5.01 | 15.87 | sedge peat, and reed peat | predominantly intensive agriculture use and build-up areas | Beltman et al. 1999; Faasen et al. 2000; Beltman 2002 unpublished data |

Bakker JP. Nature Management by Grazing and Cutting. Geobotany 1989;14:121-184.

Beltman B, Barendregt T, Broek T, van den Bootsma MC. Effectgerichte maatregelen tegen verzuring, OBN-proefprojekten ILperveld en Vechtstreek. Tussenrapportage 1998. Milieukunde & Hydro-ecologie, Faculteit Biologie, Universiteit Utrecht; 1999.

Blüml V. Langfristige Veränderungen von Flora und Vegetation des Grünlandes in der Dümmerniederung (Niedersachsen) unter dem Einfluss von Naturschutzmaßnahmen. [Dissertation] Unversity of Bremen; 2011.

Faasen T, van den Broek T, Beltman B. Effecten van bekalken, al of niet in combinati met plaggen, in verzuurde trilveenvegetaties in het vechtplassengebied. Leerstoelgroep Landschapoekologie, Universiteit Utrecht; 2000.

Ganzert CJ, Pfadenhauer J. Vegetation und Nutzung des Grunlandes am Dummer. - Naturschutz Landschaftspfl. Niedersachs 1988;16:78s.

Geiger-Udod B. Effizienzkontrolle der Renaturierungsmaßnahmen auf den Flächen des Klosters Benediktbeuern [dissertation]. Lehrstuhl für Vegetationsökologie der TUM Freising-Weihenstephan, Freising; 2001.

Hedberg P, Kotowski W, Saetre P, Malson K, Rydin H, Sundberg S. Vegetation recovery after multiple-site experimental fen restorations. Biolog Conserv 2012;147(1):60–67.

Klimkowska A, van der Elst DJD, Grootjans AP. Understanding long-term effects of topsoil removal in peatlands: overcoming thresholds for fen meadows restoration Appl Veg Sci 2015;18:110–120.

Patzelt A. Vegetationsökologische und populationsbiologische Grundlagen für die Etablierung von Magerwiesen in Niedermooren [dissertation].Dissertationes Botanicae Band 297, Gebrüder Borntraeger, D-14129 Berlin, D-70176 Stuttgart; ISBN 3-443-64209-8. Printed in Germany by Strauss offsetdruck gmbh, D-69509 Mörlenbach; 1998.

Quinger B. Untersuchungen zur Vegetationsentwicklung auf den Grünlandflächen des Klosters Benediktbeuern in den nordöstlichen Loisach-Kochelseemooren mit Empfehlungen zum weiteren Management Zentrum für Umwelt und Kultur Benediktbeuern (ZUK), Heidehoh Stiftung; 2009.

Schachtele M. Einfluss von Bodenabschub und Mahgutubertragung af die langfristige Vegetationsentwicklung neu angelegter Magerwiesen in Kalkflachmooren [dissertation]. Diplomarbeit 2004, Lehrstuhl fur Vegetationsokologie der Technischen Universitat Munchen – Weinhenstephan; 2004.

Schrautzer J, Sival F, Breuera M, Runhaar H, Fichtner A, 2013. Characterizing and evaluating successional pathways of fen degradation and restoration.Ecol Indic 2013;25:108–120.

Sliva J. Untersuchungen zur Renaturierung ehemals landwirtschaftlich genutzter Niedermoorflachen und teilabgetorfter Hochmoore. Techische Universitat Munchen; 1996.

Timmermann T. Anbau von Schilf (Phragmites australis) als ein Weg zur Sanierung von Niedermooren - Eine Fallstudie zu Etablierungsmethoden, Vegetationsentwicklung und Konsequenzen für die Praxis. Archiv für Naturschutz und Landschaftsforschung 1999;38(2-4):111-143.

Van der Hoek D, Heijmans MMPD. Effectiveness of Turf Stripping as a Measure for Restoring Species-Rich Fen Meadows in Suboptimal Hydrological Conditions. Restor Ecol 2007;15(4):627–637.

Van der Hoek D, Sykora KV. Fen-meadow succession in relation to spatial and temporal differences in hydrological and soil conditions. Appl Veg Sci 2006;9:185-194.

van Diggelen J, Bense IHM, Brouwer E, Limpens J, van Schie JMM, Smolders AJP, Lamers LPM. Restoration of acidified and eutrophied rich fens: Long-term effects of traditional management and experimental liming. Ecol Eng 2015;75:208–216.

Wild U. Renaturierung entwässerter Niedermoore am Beispiel des Donaumooses bei Ingolstadt : Vegetationsentwicklung und Stoffhaushalt [dissertation].TU Munchen,Freising-Weihenstephan, München Utz, Wiss. Germany; 1997.

Wołejko L,Grootjans AP, Veeman I, Verschoor A, Stańko R. Development and Degradation of groundwater-fed wetlands in the Drawa National Park, Poland. Water-Environment-Rural Areas 2001;1(1):105-122

Processing the trait data

For each species occurring in our data we used a median of TRY database (Kattge et al. 2011) records (multiple records per species) or a value itself when only a single record existed. As many of species in our data set are rather rare, we could not set a minimum number of records for estimation. Plasticity of species response was omitted, and no phylogenetic correction was applied. We selected PFT that are related to the main stressors or respond to the changes in abiotic and biotic conditions due to RE and TSR. Because of inconsistencies between data sources, for some PFTs we could only use a sub-set of the database, which resulted in a lower data coverage. Some data was of insufficient quality and was omitted (e.g. Leaf C:N rario, rooting depth) or replaced with supplementary information from literature (e.g. start of flowering in months, canopy height), in order to increase data coverage.

**S2 Table. Characteristics of PFT data used in the analysis.** Log-transformation was applied to adjust the right-skewed frequency distribution. % data stands for data coverage and indicates for how many species the trait estimation was available (in total 828 species).

| Short name | Trait name and [unit] | Scale | Standardisation algorithm (.s) | % data | Related to | Source |
| --- | --- | --- | --- | --- | --- | --- |
| **Traits related to stress and competition** | | | | | | |
| ch/ch.s | canopy height [m] | Quantitative | log-transformed,  scaled into 0-1 | 84.5%  78.5%^ | Competitiveness for light | TRY (Kattge et al. 2011) |
| rh/ rh.s | releasing height [m] | Quantitative | log-transformed,  scaled into 0-1 | 65% | *Strongly correlated with ch, omitted* | TRY (Kattge et al. 2011; Rutkowski 2011) |
| sla /sla.s | specific leaf area [mm2/mg] | Quantitative | log-transformed,  scaled into 0-1 | 70% | Nutrient acquisition strategy, competitiveness, Tolerance to anoxic conditions | TRY (Kattge et al. 2011) |
| ldmc / ldmc.s | leaf dry matter content [g/g] | Quantitative | scaled into 0-1 | 64.5% | Nutrient acquisition strategy, decomposition of litter | LEDA (Kleyer et al. 2008) |
| cs | lateral spread | Ordinal scale: 0-annual plant, 0.3-lateral spread <0.1m/yr; 0.6-lateral spread 0.1-0.25m/yr; 1-lateral spread >0.25m/yr |  | 72% | Reproduction & competitiveness (space occupation) | CLO-PLA (Klimešová & De Bello 2009) |
| fl/ fl.s | start of flowering in months | Ordinal | scaled into 0-1 | 59% | Reproduction, adaptation to competition (for light) | Rutkowski 2011; Moraczewski et al. 2000 |
| w/w.s | Ellenberg moisture value | Ordinal | scaled into 0-1 | 81% | Tolerance to and ability to grow in anoxic conditions, not ‘functional trait’ *sensu stricto,* used commonly as indicator of adaptation to survive and grow under anoxia | Ellenberg et al. 1992 |
| cn | Leaf carbon/nitrogen (C/N) ratio [g/g] | Quantitative |  | 33% | Nutrient acquisition strategy, decomposition of litter. *Omitted due to insufficient data coverage.* | TRY (Kattge et al. 2011) |
| rgr | Plant relative growth rate [g/g/day] | Quantitative |  | 27% | Competitiveness. *Omitted due to insufficient data coverage.* | TRY (Kattge et al. 2011) |
| rd | Rooting depth [m] | Quantitative |  | 29.5% | Nutrient acquisition strategy, stress tolerance. *Omitted due to insufficient data coverage.* | TRY (Kattge et al. 2011) |
| nit | Leaf nitrogen (N) content per leaf dry mass [mg/g] | Quantitative |  | 50% | Nutrient acquisition strategy, decomposition of litter | TRY (Kattge et al. 2011) |
| pho | Leaf phosphorus (P) content per leaf dry mass [mg/g] | Quantitative |  | 36% | Nutrient acquisition strategy, decomposition of litter | TRY (Kattge et al. 2011) |
| h | hummocks forming | Binary: 0-no, 1-yes |  | 100% | Reproduction & competitiveness (space occupation) | Moraczewski et al. 2000 |
| n | nitrogen fixation | Binary: 0-no, 1-yes |  | 59.5% | Nutrient acquisition strategy | TRY (Kattge et al. 2011) |
| MStatusO | Always mycorrhizal | Binary |  | 66% | Nutrient acquisition strategy | MycoFlor^#^ (Hempel et al. 2013; Akhmetzhanova et al. 2012; Veselkin et al. 2014) |
| MStatusN | Always non-mycorrhizal | Binary |  | 66% | Nutrient acquisition strategy, Tolerance to and ability to grow in anoxic conditions | MycoFlor^#^ (Hempel et al. 2013; Akhmetzhanova et al. 2012; Veselkin et al. 2014) |
| MFlexi | Flexible: sometimes mycorrhizal, sometimes non-mycorrhizal | Binary |  | 66% | Nutrient acquisition strategy | MycoFlor^#^ (Hempel et al. 2013; Akhmetzhanova et al. 2012; Veselkin et al. 2014; Moora, 2014) |
| **Traits indicating dispersal and recruitment potential** | | | | | | |
| sm /sm.s | seed mass [mg] | Quantitative | log-transformed,  scaled into 0-1 | 79% | Reproduction, colonisation & dispersal, competitiveness (of seedlings) | TRY (Kattge et al. 2011) |
| snb /snb.s | Seed number per ramet | Ordinal (reclassified): 0 - <100, 0.5 - 101-10000, 1 - >10 000 seeds |  | 60% | Reproduction, colonisation & dispersal | based on LEDA (Kleyer et al. 2008) |
| ge | Seed germination rate (germination efficiency) [%] | Quantitative |  | 48% | Reproduction, colonisation. *Omitted due to insufficient data coverage.* | TRY (Kattge et al. 2011) |
| seed buoyancy | Floating capacity of diaspores on water [%] | Quantitative | scaled into 0-1 | 50% | Dispersal | based on LEDA (Kleyer et al. 2008) |
| Dispersal syndromes | |  |  |  |  |  |
| autochor | (self) | Binary for each dispersal category / syndrome |  | 67% | Dispersal | based on LEDA (Kleyer et al. 2008) |
| bythisochor | (water moving) |  |  | 67% | Dispersal |  |
| chamaechor | (wind whole plant) |  |  | 67% | Dispersal |  |
| hemerochor | (antropo) |  |  | 67% | Dispersal |  |
| meteorochor | (wind) |  |  | 67% | Dispersal |  |
| nautochor | (water) |  |  | 67% | Dispersal |  |
| ombrochor | (rain drops) |  |  | 67% | Dispersal |  |
| other | (other) |  |  | 67% | Dispersal |  |
| zoochor | (animals) |  |  | 67% | Dispersal |  |
| inverb | (invertebrates) |  |  | 67% | Dispersal |  |
| mammals | (mammals) |  |  | 67% | Dispersal |  |
| diversity /diversity.s | number of syndromes | Quantitative | scaled into 0-1 | 67% | Dispersal |  |
| **Functional groups, life strategies** | | | | | | |
| PO, CY, FO, PT  BM, SPH | Ecological groups: grasses, sedges & rushes, forbs, ferns and spore plants, brown mosses en *Sphagnum* mosses | Binary: 0-no, 1-yes |  | 100% | Life strategy, e.g. tolerance to anoxic conditions (CY), competitiveness (PO) | based on taxonomy |
| pls | plant lifespan | Binary: 0-perennial, 1-annual/biennial |  | 92% | Reproduction, Nutrient acquisition strategy | Rothmaler 2009; Rutkowski 2011 |
| pha | Phanerophyte | Binary: 0-no, 1-yes |  | 100% | Competitiveness for light | based on taxonomy |
| bry | bryophyte | Binary: 0-no, 1-yes |  | 100% | Competitiveness, decomposition of litter, Tolerance to and ability to grow in anoxic conditions | based on taxonomy |
| st_c | C – competitor | strategy *sensu* Grime, fuzzy coding relative to contribution: e.g. ‘CS’= 0.5 for each C & S; ‘CSR’ = 0.33 for each strategy |  | 66.5% | Competitiveness | TRY (Kattge et al. 2011) |
| st_r | R – ruderal |  |  | 66.5% | Tolerance to disturbance |  |
| st_s | S – stress tolerator |  |  | 66.5% | Stress – tolerance |  |

^^^without Phanerophyte (trees and shrubs)

^#^Information about mycorrhizal associations (in vascular plants only) was obtained from MycoFlor database (Hempel et al., 2013), cross-checked with data from Akhmetzhanova et al. 2012, and supplemented with data from Veselkin et al. (2014) for Carex spp.

Akhmetzhanova AA, Soudzilovskaia NA, Onipchenko VG, Cornwell WK, Agafonov VA, Selivanov IA, et al. A rediscovered treasure: mycorrhizal intensity database for 3000 vascular plant species across the former Soviet Union. Ecology 2012 Mar;93(3):689–690.

Ellenberg H, et al. Zeigerwerte der Gefäßpflanzen. Scripta Geobotanica 1992;18:9-166.

Hempel S, Götzenberger L, Kühn I, Michalski SG, Rillig MC, Zobel M, et al. Mycorrhizas in the Central European flora: relationships with plant life history traits and ecology. Ecology 2013 Jun;94(6):1389–1399.

Veselkin DV, Konoplenko MA, Betekhtina AA. Means for soil nutrient uptake in sedges with different ecological strategies. Russian Journal of Ecology 2014;45(6):547–554.

Kattge J, Diaz S, Lavorel S, Prentice IC, Leadley P, Bönisch G, et al. TRY - a global database of plant traits. Glob Change Biol 2011 Sep;17(9):2905-2935.doi: 10.1111/j.1365-2486.2011.02451.x

Kleyer M, Bekker RM, Knevel IC, Bakker JP, Thompson K, Sonnenschein M, et al. The LEDA Traitbase: a database of life-history traits of the Northwest European flora. J Ecol 2008 Nov;96(6):1266–1274.doi: 10.1111/j.1365-2745.2008.01430.x

Klimešová J, De Bello F. CLO‐PLA: the database of clonal and bud bank traits of Central European flora. J Veg Sci 2009;20(3): 511-516.

Moora M. Mycorrhizal traits and plant communities: perspectives for integration. J Veg Sci 2014 Sep;25(5):1126–1132. doi: 10.1111/jvs.12177)

Moraczewski IR, Sudnik-Wójcikowska B, Dubielecka B, Rutkowski R, Nowak KA, Borkowski W, Galera H. Flora ojczysta - gatunki pospolite, chronione, ciekawe... (CD-ROM: Atlas roślin, słownik botaniczny i multimedialne klucze do oznaczania) [Polish multimedia key for vascular plants]. Warszawa: Wyd. Stigma; 2000. Polish.

Rothmaler W. Exkursionsflora von Deutschland, Band 3. Gefäßpflanzen: Atlasband., Heidelberg: Spektrum Akademischer Verlag; 2009. German.

Rutkowski L. Klucz do oznaczania roślin naczyniowych Polski niżowej [The key to determining the Polish lowland vascular plants]. 2nd ed.Warszawa: Wydawnictwo Naukowe PWN; 2011. Polish.

Calculation of community means and the FD indices

Using species composition and abundance, the PFT values were established per relevee and aggregated to the community level.

The PFT information per community was expressed as community weighted means (CWMs) or community means (CMs). The mean values for the most of quantitative trait data (numeric data) were standardised into values between 0 and 1, which was indicated with ‘s’ behind the trait code (see S2 Table ).

An estimation of absolute change in trait values provided an accurate indication of an effect and its direction (increase , decrease) of RE and TSR on each trait. In our opinion, an observation of direct values and of by how much these values have changed gives important insight in the data. Therefore, we did not apply a classical effect size calculation with standardization with standard deviation.

The outcome of restoration depends on the scale of degradation, site-specific factors and may differ largely. In assessing the effects of restoration on PFTs we used data from before restoration and after restoration (last available observations).

Community means were calculated with traits of vascular plants, omitting mosses and shrubs or trees (except means for ecological groups bry, pha, BM, SPH). PFTs have a different ecological meaning in mosses and vascular plants, due to different scales and life strategies. Restored vegetation was generally open and often under nature management, but almost always some juvenile shrub or tree were found in the vegetation, even in the reference mires (mostly not managed). PFTs are reflecting adult plant characteristics, therefore including these groups in calculations could result in biased estimates. CWMs were calculated only if trait data were present for species contributing cumulatively to at least 80% cover abundance (Pakeman & Quested 2007), or otherwise replaced with ‘no data’. Consequently, some quantitative PFTs had to be omitted, due to insufficient data (S2 Table ).

For multi-traits metrics of FD used Functional Richness (FRich), Functional Eveness (FEve), Functional Diversity (FDiv), Functional Dispersion (FDis), and Rao index (Mason et al. 2005, 2013). Those FD indices were calculated using the ‘FD package’ (Laliberté and Legendre 2010; Laliberté et al. 2014) in R (R Development Core Team, 2014) with the following PFT: sla, ldmc, snb, sm, w, ch, seed buoyancy, (sla, sm, ch log transformed, all traits rescaled to 0-1 values for the data set). Integrated indices were calculated using the quantitative traits and in general allow a quantification of the values, but are based on the latent factors, which are constructed from the included traits and represent the largest variation in data.

Multivariate data analysis with ordination techniques

Data time series differed largely in length and frequency of collection, so their straightforward comparison was not possible. Furthermore, PFTs are not independent of each other, but co-vary, due to positive relations and trade-offs. To explore those patterns of change the multifunctional analysis of species composition and of PFTs combinations were used. Detrended Correspondence Analysis (DCA) was used to explore the data, to confirm if data covered a variety of species combinations and conditions and later to depict the shifts in vegetation after restoration (plant species composition data used). Principal component analysis (PCA) with CMs (short gradient values) was used for exploring shifts in trait composition (functional trait composition data used). The plot scores were generated for the first two ordination axes. Next, they were combined for site x treatment x time combination and used for a graphical representation of shifts in vegetation (plotted as a shift between observation before and last observation after restoration). For reference fens, mean and standard deviation of scores were used as indication of the range of the reference values.
