# Supplementary material for: Are we restoring functional fens? – The outcomes of restoration projects in fens re-analysed with plant functional traits
Source: PLoS One. 2019 Apr 24;14(4):e0215645. doi: 10.1371/journal.pone.0215645 (PMC6481837; doi:10.1371/journal.pone.0215645)
Supplement: S2 Supplementary Materials — (DOCX) [file pone.0215645.s002.docx]

**S2 Supplementary materials - Results**

Multivariate analysis results

As revealed by a species-based DCA, the main two gradients in the data were related to nutrient availability and a gradient in moisture-inundation, first and second axes, respectively (S1 Fig). A cumulative explained variation on first two axes was low (3.4%), due to a large number of species and diversity of sites. Locality (site included as a supplementary variable) accounted for 22.2% variation (adjusted) in species composition. The ordination graphs illustrating shifts of species composition were presented in S2 Fig . For exploring the vegetation change we generated the plot scores in a DCA analysis with reduced number of species (by omitting species that occurred only once or twice in the data set), which increased cumulative explained variation of first two axes to 8.5%. Species-based ordination revealed a shift towards the reference after restoration. Based on the species ecology and species ordination we observed that after RE, a shift in species composition indicated inundation and eutrophic conditions and after TSR the shift towards reference was more clear but indicating drier conditions in several cases. In about half of the cases these shifts were not directional and vegetation trajectory exhibited looping and set-backs (data not shown).

Multivariate analysis (PCA) with un-weighted community means of PFT as discriminative values in the trait space (traits used as species) were used for exploring the response to restoration in functional spectra. Those two approaches gave a different picture. We found a larger, more consistent shifts towards reference after TSR than after RE, indicating overall change towards sets of PFT indicating more nutrient poor and wetter conditions. This was related to larger shifts along 1st PCA axis, which was mainly reflecting a gradient in nutrient acquisition strategies (see S3 Fig ).

**S1 Fig.** **Results of DCA ordination analysis for species (75 best fitted species plotted).** Ordination with 5476 plots, 837 species. Eigenvalues for first and second ordination axis: 0.88, 0.65 (gradient length 9.0, 7.2 SD units, respectively). Explained variation (cumulative): ca. 2% and 3.4%. Species abbreviation include 4 letters of genus name and 4 letters of species name. When site included as suppl. variable they accounted for 22.2% variation in data (adjusted).

**S2 Fig .** **Graphs of the shift in species composition after restoration with RE and TSR.** Results for RE and TSR were plotted separately for clarity. Only the situation before restoration and the last year of observation were plotted. Arrows connect the two points and indicate the direction of change. Graphs are constructed using scores of samples for first two axes from DCA analysis, with all plots and reduced number of species (species with less than 2 occurrences were excluded, resulting in 727 species). This DCA analysis explained 5.5% and 8.5% cumulative variation on first and second axis (Eigenvalues of 0.87, 0.48, and gradient length 8.2 and 6.6 SD unit, respectively). Scores were aggregated per site x treatment x time combination. For MIRES (reference records), the score average and the standard deviation were plotted (error bars), to indicate a range of values. For site symbols see S1 Table 1 in Supporting materials. The number behind the site letters code indicates number of years since the restoration measure was applied. Stripped arrows and grey letters codes indicate the controls when these were available. For the controls the age was not indicated as it is the same as for corresponding restored sites.

**S3 Fig.** **Graph illustrating the main gradients with the related PFTs and the (expected) shift in the trait space after restoration with RE and TSR.** In the PCA analysis first two axes from explained cumulatively 39.5% and 47.3% of variation in data respectively (Eigenvalues of 0.395, 0.08, short gradient of ca. 1.3 SD unit). For MIRES, the average and the standard deviation were plotted (error bars).

**S4 Fig.** **Results of the comparisons in selected quantitative PFTs.** On the X axis the CM mean value of the trait were plotted, while on the Y axis the mean value of the functional range of the trait were plotted. Error bars indicate 80% confidence interval. An absolute change in values was calculated based on the paired samples, then aggregated per site and later aggregated per treatment. (s) indicates standardized values (between 0 and 1) in this data set.

**S5 Fig .** **Selected results of comparisons in qualitative traits between non-degraded (MIRE), degraded (BEFORE_RE, BEFORE_TSR) and restored fens (after rewetting - RE or topsoil removal - TSR).**

Symbols: Point – Mean; Error bars indicate 80% confidence interval. Letters indicate groups different at p < 0.05 in non-parametric Kruskal-Wallis test. For all PFT: NBEFORE_RE = 18; NREWET = 18; NBEFORE_TSR = 20; NTSR = 19; NMIRE = 37.

(a) Seed output (s), the CWM value (X) v.s. CM value (Y) of the trait were plotted,

(b) Fraction of obligatory mycorrhizal species (MStatusO), the CWM value (X) v.s. CM value (Y) of the trait were plotted. For contribution of species that are flexible in mycorrhizal status there was no differences between non-degraded, degraded or restored fens,

(c) Fraction of non-mycorrhizal species (MStatusN). the CWM value (X) v.s. CM value (Y) of the trait were plotted,

(d) Contribution of C- and S-strategies in the vegetation, on X and Y axis, respectively. Only CWMs have been used. CRS C- and S-strategies were shown as commonly applied indicators, but they are not traits,

(e) Clonal spread (cs) in the vegetation v.s. ‘hummockness’ (h). For clonal spread (cs), an ordinal, semi-quantitative scale of this trait indicated annual plants (no clonal spread), slow lateral spread (e.g. tussocks forming or forming ramets) and fast lateral spreads (e.g. with rhizomes or stolon’s). Trait ‘hummockness’ (h)– a contribution of species forming tussocks and hummocks. Only CWMs have been used. No significant difference between groups were found.

(f) Contribution of Bryophytes (X) v.s. Phanerophytes (trees and shrubs) (Y) in the vegetation. Only CWMs have been used. For pha, no differences between groups were found. For Bry: MIRE differed from BEFORE_R & REWET, test p<0.05.

(g) Contribution of BM – brown mosses (X) v.s. contribution of SPH – Sphagnum mosses (Y) in the vegetation. Only CWMs have been used. For SPH no differences between groups were found. For BM: only MIRE differed significantly (p<0.05) from the other groups.

(h) Contribution of CY – Cyperaceae family (mainly sedges) (X) v.s. contribution of PO – Poaceae family, grasses (Y) in the vegetation. Only CWMs have been used. For CY, ‘MIRE’ differed from the degraded situation (before_re, before_tsr) , but not from restored situation. For PO, reference data differed from all other groups, test p<0.05.

(i) Contribution of FO – forbs (different families) (X) v.s. PT – Pteridophyte –ferns, Equisetum, other spore plants (Y) in the vegetation. Only CWMs have been used. For FO, no differences between groups were found. For PT, reference MIRE differed from site after TSR and from situation before restoration with TSR, but not from sites after RE (or degraded systems before_RE).

(j) N-fixing ability (CWM) in the community.

(k) Contribution of various dispersal syndromes in the vegetation. Only CMs have been used, as these traits have mainly indicative information regarding various dispersal vectors. Categories: nautochor - dispersal by water; hemerochor - dispersal by men (antropogenic aid); zoochor - dispersal by animals (all groups).

(l) Contribution of various dispersal syndromes in the vegetation. Only CMs have been used, as these traits have mainly indicative information regarding various dispersal vectors. Categories: autochor- self-dispersing plants (e.g. mechanical adaptations for discharging seeds); mammals - dispersal by large mammals; meteorochor - dispersal by wind (light seeds with morphological adaptations for flying).
