# Supplementary material for: Are we restoring functional fens? – The outcomes of restoration projects in fens re-analysed with plant functional traits
Source: PLoS One. 2019 Apr 24;14(4):e0215645. doi: 10.1371/journal.pone.0215645 (PMC6481837; doi:10.1371/journal.pone.0215645)
Supplement: S1 Table — Peatland characteristics (system type, presence of gyttja, size, depth of peat, peat type and catchment characteristics) were provided by authors. Climatic characteristics were based on the open access European Environmental Agency data on Precipitation and Evapotranspiration. Column descriptions: max. depth—maximum depth of peat in meters; T–mean annual air temperature in degree Celsius, P—mean annual precipitation in millimetres, T winter—mean winter air temperature in degree Celsius, T summer—mean summer air temperature in degree Celsius. (DOCX) [file pone.0215645.s003.docx]

S1 Table. Site characteristics of the fen restoration projects. Peatland characteristics (system type, presence of gyttja, size, depth of peat, peat type and catchment characteristics) were provided by authors. Climatic characteristics were based on the open access European Environmental Agency data on Precipitation and Evapotranspiration. Column descriptions: max. depth - maximum depth of peat in meters; T – mean annual air temperature in degree Celsius, P - mean annual precipitation in millimetres, T winter - mean winter air temperature in degree Celsius, T summer - mean summer air temperature in degree Celsius.

| No. | code | Site | Coordinates | RE/TSR | System type | Gyttja 0 /1 | max. depth [m] | T | P | T winter | T  Summer | Soil/ peat type | Catchment | References |
| --- | --- | --- | --- | --- | --- | --- | --- | --- | --- | --- | --- | --- | --- | --- |
| 1 | BB | Biesenbrow | 53°07'56"N,  13°59'37"E | REWET | River valley system | 1 | 2 | 7.67 | 551.4 | 0.98 | 15.56 | Week groundwater seepage, not continues gyttja , peat 1-2 m deep, sedge type | long-term deforested, predominantly intensive agriculture use | Timmermann 1999 |
| 2 | CIE | Cieszynka | 53°05'42"N, 16°00'18"E | REWET | Lake basin | 1 | 1 | 7.37 | 585.6 | 0.89 | 15.65 | gyttja 6-8m deep, moss- sedge peat | Forest | Wołejko et al. 2001; Stańko, R.  unpublished data |
| 3 | DU | Dümmer Lake | 52°29'07"N, 8°19'26"E | REWET | Lake basin | 1 | 1.25 | 8.31 | 800.9 | 2.81 | 15.37 | sedge -reed peat | intensive agriculture use | Blüml 2011; Ganzert & Pfadenhauer 1988 |
| 4 | HAV | Havel | 53°27'8.5"N,  12°56'59"E | REWET | Lake basin | 1 | 4 | 7.25 | 571.2 | 1.21 | 15.62 | Heavily degraded peat, moss- sedge peat | predominantly intensive agriculture use | Vegelin, K. 2014, unpublished data |
| 5 | HO | Hohner See | 54°17'54"N,  9°28'03"E | REWET | Lake basin | 0 | 2 | 7.67 | 829.5 | 1.94 | 15.52 | sedge -reed peat | intensive agriculture use and build-up areas | Schrautzer et al. 2013 |
| 6 | KB | Klosterland Benediktbeuern | 47°43'28"N,  11°22'28"E | REWET | River valley system | 1 | 6 | 6.62 | 1002.9 | -1.01 | 14.11 | On peatland - intensive agriculture, gyttja 3 m deep, sedge - 7reed peat | lower Alps, mixed land use: low intensity agriculture and forests | Geiger-Udod 2001;  Quinger 2009 |
| 7 | LA | Landgrobental | 53°40'38"N, 13°43'22"E | REWET | River valley system & Spring area | 0 | 6 | 7.58 | 555.8 | 0.98 | 15.56 | moss- sedge peat, sedge peat | predominantly intensive agriculture use | Vegelin, K. 2014, unpublished data |
| 8 | OOS | Oosterschar | 52°54'57"N,  5°53'45"E | REWET | River valley system | 0 | 1.2 | 7.60 | 785.6 | 5.41 | 12.30 | clay-loam layer under peat, sedge peat | predominantly intensive agriculture use | Jager, H.J. unpublished data |
| 9 | PLI | Kosobudki | 52°14'48"N, 15°10'09"E | REWET | River valley system | 1 | 5 | 7.96 | 562.1 | 0.01 | 15.99 | sedge and woody peat, gyttja present locally | Forest, not transformed | Stańko, R., unpublished data |
| 10 | RR | Randow - Rustow | 53°56'28"N,  N13°04'50"E | REWET | River valley system | 1 | 7 | 7.72 | 557.2 | 1.08 | 15.59 | moss-sedge peat, sedge peat | predominantly intensive agriculture use | Vegelin, K. 2014, unpublished data |
| 11 | SE | Sevenmosses | 60°26'13"N,  17°57'35"E | REWET | River valley system | 0 | 1 | 4.87 | 577.0 | -5.88 | 12.75 | moss-sedge peat | Forest (coniferous) | Hedberg et al. 2012 |
| 12 | ST | Styggkärret | 59°57'35"N,  17°18'20"E | REWET | River valley system | 0 | 2 | 4.84 | 574.9 | -3.82 | 13.11 | moss-sedge peat | Forest (coniferous) | Hedberg et al. 2012 |
| 13 | TA | Taarlo | 53°02'20"N,  6°38'19"E | REWET | River valley system | 0 | 1.2 | 7.68 | 788.0 |  |  | sedge and woody peat | predominantly intensive agriculture use | Bakker, J.P. & De Vries, Y. 2014, unpublished data; Bakker 1989 |
| 14 | TR | Trebeltal | 54°6'50"N,  12°41'28"E | REWET | River valley system | 1 | 9 | 7.64 | 558.1 | 1.28 | 15.64 | moss-sedge peat, sedge peat | Forest and agricultural use | Vegelin, K. 2014, unpublished data |
| 15 | UL | Ultunaviken | 59°57'13"N,  17°18'21"E | REWET | River valley system | 0 | 3 | 4.91 | 573.4 |  |  | moss-sedge peat | Forest (coniferous) | Hedberg et al. 2012 |
| 16 | UZ | Vloweitje Urkhoven | 51°26'15"N,  5°32'17"E | REWET | River valley system | 0 | 1.5 | 8.39 | 775.5 | 4.42 | 15.90 | sedge peat, reed peat and woody peat | predominantly intensive agriculture use and build-up areas | van der Burg, R.F. 2015, unpublished data |
| 17 | ZB | Zwarte Beek | 51°05'43"N, 5°17'16"E | REWET | River valley system | 0 | 3 | 8.27 | 810.9 | 5.01 | 15.87 | moss-sedge peat, woody peat | Forest (coniferous) and agriculture use | Aggenbach, C. unpublished data |
| 18 | CAL | Całowanie | 52°00'40"N, 21°21'00"E | TSR | River valley system & spring area | 0 | 4 | 6.78 | 511.3 | -0.98 | 18.40 | moss-sedge peat | Forest (coniferous) | Klimkowska et al. 2010  & unpublished data |
| 19 | COM | Commissarissenlanden | 51°57'03"N,  4°44'09"E | TSR | River valley system | 0 | 10 | 8.41 | 793.3 | 5.01 | 15.87 | peatland is under low intensity use, sedge - woody peat | predominantly intensive agriculture use | Kerkhof, D., Reinink, W., van Heerden, A. 2013, unpublished data |
| 20 | DE | Delling | 48°03'14"N, 11°14'52"E | TSR | Spring area | 0 | 1.5 | 7.21 | 954.3 | -0.99 | 14.00 | sedge peat | predominantly intensive agriculture use | Sliva 1996; Sliva, J. unpublished data |
| 21 | DO | Donaumoos Dachsholz | 48°42'00"N,  11°11'00"E | TSR | River valley system | 0 | 1.5 | 7.61 | 769.4 | -0.99 | 14.00 | flow-through mire type, sedge peat, reed peat and woody peat | predominantly intensive agriculture use, also on peatland | Wild 1997; Patzelt 1998; Schachtele 2004 |
| 22 | KO | Korenburgerveen | 51°59'13"N,  6°39'53"E | TSR | River valley system | 0 | 1 | 8.29 | 777.9 | 3.84 | 15.92 | edge of bog, former leg zone, transition towards a local brook valley, groundwater 23influence, sedge -sphagnum peat | long-term deforested, predominantly intensive agriculture use | Van der Hoek & Heijmans 2007 |
| 23 | LM | Lage Maden | 53°0'50"N,  6°37'43"E | TSR | River valley system | 0 | 1.2 | 7.63 | 787.5 | 5.41 | 12.30 | sedge peat, and woody peat | predominantly intensive agriculture use | Grootjans, A.P. unpublished data; Klimkowska et al. 2015 |
| 24 | MOS | Mosbeek | 52°26'47"N,  6°52'13"E | TSR | Spring area | 0 | 0.5 | 8.47 | 776.8 | 3.84 | 15.92 | Moss - sedge peat | predominantly intensive agriculture use, forest | van Tweel 2014, unpublished data |
| 25 | NP | Nieuwkoopse Plassen | 52°08'46"N, 04°49'02" E | TSR | Lake basin | 0 | 1 | 8.52 | 799.8 | 5.01 | 15.87 | Intensive drainage (deep polders), sphagnum peat - reed peat | predominantly intensive agriculture use | van Diggelen et al. 2015 |
| 26 | OUD | Oudeland | 51°56'16"N 4°40'43"E | TSR | River valley system | 0 | 4 | 8.77 | 787.6 | 5.01 | 15.87 | peatland is under low intensity use, sedge peat, and woody peat | predominantly intensive agriculture use and build-up areas | Kerkhof, D., Reinink, W., van Heerden, A. 2013, unpublished data |
| 27 | PBG | Polder Berkenwoude Graafkade | 51°57'24"N,  4°41'49"E | TSR | River valley system | 0 | 10 | 8.35 | 785.9 | 5.01 | 15.87 | peatland is under low intensity use, sedge peat, and woody peat | predominantly intensive agriculture use and build-up areas | Kerkhof, D., Reinink, W., van Heerden, A. 2013, unpublished data |
| 28 | PBN | Polder Berkenwoude Nooitgedacht | 51°57'47"N,  4°42'30"E | TSR | River valley system | 0 | 10 | 8.67 | 790.0 | - | - | peatland is under low intensity use, sedge peat, and woody peat | predominantly intensive agriculture use and build-up areas | Kerkhof, D., Reinink, W., van Heerden, A. 2013, unpublished data |
| 29 | PMB | Polder Middelblok | 51°59'21"N, 4°41'28"E | TSR | River valley system | 0 | 10 | 8.05 | 786.9 | - | - | peatland is under low intensity use, sedge peat, and woody peat | predominantly intensive agriculture use and build-up areas | Kerkhof, D., Reinink, W., van Heerden, A. 2013, unpublished data |
| 30 | PVB | Polder Veerstalblok | 51°59'53"N,  4°43'01"E | TSR | River valley system | 0 | 3 | 7.72 | 791.0 | - | - | peatland is under low intensity use, sedge peat, and woody peat | predominantly intensive agriculture use and build-up areas | Kerkhof, D., Reinink, W., van Heerden, A. 2013, unpublished data |
| 31 | VK | Veenkampen | 52°00'26"N, 5°35'43.5"E | TSR / REWET | River valley system | 0 | 1 | 7.91 | 784.5 | 4.13 | 15.91 | peaty soils, with clay intrusion | long-term deforested, predominantly intensive agriculture use | Van der Hoek & Sykora 2006 |
| 32 | VPA | Suikerpot A | 52°13'11.5"N, 5°06'31"E | TSR | River valley system | 0 | 2 | 8.57 | 803.2 | 5.01 | 15.87 | sedge peat, and reed peat | predominantly intensive agriculture use and build-up areas | Beltman et al. 1999; Faasen et al. 2000; Beltman 2002 unpublished data |
| 33 | VPB | Suikerpot B | 52°13'12"N, 5°06'28"E | TSR | River valley system | 0 | 2 | 8.57 | 803.2 | 5.01 | 15.87 | sedge peat, and reed peat | predominantly intensive agriculture use and build-up areas | Beltman et al. 1999; Faasen et al. 2000; Beltman 2002 unpublished data |
| 34 | VPC | het Hol | 52°13'18"N, 5°05'01"E | TSR | River valley system | 0 | 2 | 8.57 | 803.2 | 5.01 | 15.87 | sedge peat, and reed peat | predominantly intensive agriculture use and build-up areas | Beltman et al. 1999; Faasen et al. 2000; Beltman 2002 unpublished data |
| 35 | VPD | Ster | 52°11'29"N,  5°06'28"E | TSR | River valley system | 0 | 2 | 8.59 | 802.5 | 5.01 | 15.87 | sedge peat, and reed peat | predominantly intensive agriculture use and build-up areas | Beltman et al. 1999; Faasen et al. 2000; Beltman 2002 unpublished data |
| 36 | VPE | Weersloot | 52°11'22"N, 5°07'14"E | TSR | River valley system | 0 | 2 | 8.59 | 802.5 | 5.01 | 15.87 | sedge peat, and reed peat | predominantly intensive agriculture use and build-up areas | Beltman et al. 1999; Faasen et al. 2000; Beltman 2002 unpublished data |
| 37 | VPF | Tienhoven | 52°10'09"N, 5°05'37"E | TSR | River valley system | 0 | 2 | 8.62 | 802.6 | 5.01 | 15.87 | sedge peat, and reed peat | predominantly intensive agriculture use and build-up areas | Beltman et al. 1999; Faasen et al. 2000; Beltman 2002 unpublished data |
| 38 | VPG | Molenpolder | 52°08'49"N, 5°05'28"E | TSR | River valley system | 0 | 2 | 8.62 | 802.6 | 5.01 | 15.87 | sedge peat, and reed peat | predominantly intensive agriculture use and build-up areas | Beltman et al. 1999; Faasen et al. 2000; Beltman 2002 unpublished data |

Bakker JP. Nature Management by Grazing and Cutting. Geobotany 1989;14:121-184.

Beltman B, Barendregt T, Broek T, van den Bootsma MC. Effectgerichte maatregelen tegen verzuring, OBN-proefprojekten ILperveld en Vechtstreek. Tussenrapportage 1998. Milieukunde & Hydro-ecologie, Faculteit Biologie, Universiteit Utrecht; 1999.

Blüml V. Langfristige Veränderungen von Flora und Vegetation des Grünlandes in der Dümmerniederung (Niedersachsen) unter dem Einfluss von Naturschutzmaßnahmen. [Dissertation] Unversity of Bremen; 2011.

Faasen T, van den Broek T, Beltman B. Effecten van bekalken, al of niet in combinati met plaggen, in verzuurde trilveenvegetaties in het vechtplassengebied. Leerstoelgroep Landschapoekologie, Universiteit Utrecht; 2000.

Ganzert CJ, Pfadenhauer J. Vegetation und Nutzung des Grunlandes am Dummer. - Naturschutz Landschaftspfl. Niedersachs 1988;16:78s.

Geiger-Udod B. Effizienzkontrolle der Renaturierungsmaßnahmen auf den Flächen des Klosters Benediktbeuern [dissertation]. Lehrstuhl für Vegetationsökologie der TUM Freising-Weihenstephan, Freising; 2001.

Hedberg P, Kotowski W, Saetre P, Malson K, Rydin H, Sundberg S. Vegetation recovery after multiple-site experimental fen restorations. Biolog Conserv 2012;147(1):60–67.

Klimkowska A, van der Elst DJD, Grootjans AP. Understanding long-term effects of topsoil removal in peatlands: overcoming thresholds for fen meadows restoration Appl Veg Sci 2015;18:110–120.

Patzelt A. Vegetationsökologische und populationsbiologische Grundlagen für die Etablierung von Magerwiesen in Niedermooren [dissertation].Dissertationes Botanicae Band 297, Gebrüder Borntraeger, D-14129 Berlin, D-70176 Stuttgart; ISBN 3-443-64209-8. Printed in Germany by Strauss offsetdruck gmbh, D-69509 Mörlenbach; 1998.

Quinger B. Untersuchungen zur Vegetationsentwicklung auf den Grünlandflächen des Klosters Benediktbeuern in den nordöstlichen Loisach-Kochelseemooren mit Empfehlungen zum weiteren Management Zentrum für Umwelt und Kultur Benediktbeuern (ZUK), Heidehoh Stiftung; 2009.

Schachtele M. Einfluss von Bodenabschub und Mahgutubertragung af die langfristige Vegetationsentwicklung neu angelegter Magerwiesen in Kalkflachmooren [dissertation]. Diplomarbeit 2004, Lehrstuhl fur Vegetationsokologie der Technischen Universitat Munchen – Weinhenstephan; 2004.

Schrautzer J, Sival F, Breuera M, Runhaar H, Fichtner A, 2013. Characterizing and evaluating successional pathways of fen degradation and restoration.Ecol Indic 2013;25:108–120.

Sliva J. Untersuchungen zur Renaturierung ehemals landwirtschaftlich genutzter Niedermoorflachen und teilabgetorfter Hochmoore. Techische Universitat Munchen; 1996.

Timmermann T. Anbau von Schilf (Phragmites australis) als ein Weg zur Sanierung von Niedermooren - Eine Fallstudie zu Etablierungsmethoden, Vegetationsentwicklung und Konsequenzen für die Praxis. Archiv für Naturschutz und Landschaftsforschung 1999;38(2-4):111-143.

Van der Hoek D, Heijmans MMPD. Effectiveness of Turf Stripping as a Measure for Restoring Species-Rich Fen Meadows in Suboptimal Hydrological Conditions. Restor Ecol 2007;15(4):627–637.

Van der Hoek D, Sykora KV. Fen-meadow succession in relation to spatial and temporal differences in hydrological and soil conditions. Appl Veg Sci 2006;9:185-194.

van Diggelen J, Bense IHM, Brouwer E, Limpens J, van Schie JMM, Smolders AJP, Lamers LPM. Restoration of acidified and eutrophied rich fens: Long-term effects of traditional management and experimental liming. Ecol Eng 2015;75:208–216.

Wild U. Renaturierung entwässerter Niedermoore am Beispiel des Donaumooses bei Ingolstadt : Vegetationsentwicklung und Stoffhaushalt [dissertation].TU Munchen,Freising-Weihenstephan, München Utz, Wiss. Germany; 1997.

Wołejko L,Grootjans AP, Veeman I, Verschoor A, Stańko R. Development and Degradation of groundwater-fed wetlands in the Drawa National Park, Poland. Water-Environment-Rural Areas 2001;1(1):105-122
