# Supplementary material for: Are we restoring functional fens? – The outcomes of restoration projects in fens re-analysed with plant functional traits
Source: PLoS One. 2019 Apr 24;14(4):e0215645. doi: 10.1371/journal.pone.0215645 (PMC6481837; doi:10.1371/journal.pone.0215645)
Supplement: S2 Table — Log-transformation was applied to adjust the right-skewed frequency distribution. % data stands for data coverage and indicates for how many species the trait estimation was available (in total 828 species). (DOCX) [file pone.0215645.s004.docx]

S2 Table. Characteristics of PFT data used in the analysis. Log-transformation was applied to adjust the right-skewed frequency distribution. % data stands for data coverage and indicates for how many species the trait estimation was available (in total 828 species).

| Short name | Trait name and [unit] | Scale | Standardisation algorithm (.s) | % data | Related to | Source |
| --- | --- | --- | --- | --- | --- | --- |
| **Traits related to stress and competition** | | | | | | |
| ch/ch.s | canopy height [m] | Quantitative | log-transformed,  scaled into 0-1 | 84.5%  78.5%^ | Competitiveness for light | TRY (Kattge et al. 2011) |
| rh/ rh.s | releasing height [m] | Quantitative | log-transformed,  scaled into 0-1 | 65% | *Strongly correlated with ch, omitted* | TRY (Kattge et al. 2011; Rutkowski 2011) |
| sla /sla.s | specific leaf area [mm2/mg] | Quantitative | log-transformed,  scaled into 0-1 | 70% | Nutrient acquisition strategy, competitiveness, Tolerance to anoxic conditions | TRY (Kattge et al. 2011) |
| ldmc / ldmc.s | leaf dry matter content [g/g] | Quantitative | scaled into 0-1 | 64.5% | Nutrient acquisition strategy, decomposition of litter | LEDA (Kleyer et al. 2008) |
| cs | lateral spread | Ordinal scale: 0-annual plant, 0.3-lateral spread <0.1m/yr; 0.6-lateral spread 0.1-0.25m/yr; 1-lateral spread >0.25m/yr |  | 72% | Reproduction & competitiveness (space occupation) | CLO-PLA (Klimešová & De Bello 2009) |
| fl/ fl.s | start of flowering in months | Ordinal | scaled into 0-1 | 59% | Reproduction, adaptation to competition (for light) | Rutkowski 2011; Moraczewski et al. 2000 |
| w | Ellenberg moisture value | Ordinal | scaled into 0-1 | 81% | Tolerance to and ability to grow in anoxic conditions, not ‘functional trait’ *sensu stricto,* used commonly as indicator of adaptation to survive and grow under anoxia | Ellenberg et al. 1992 |
| cn | Leaf carbon/nitrogen (C/N) ratio [g/g] | Quantitative |  | 33% | Nutrient acquisition strategy, decomposition of litter. *Omitted due to insufficient data coverage.* | TRY (Kattge et al. 2011) |
| rgr | Plant relative growth rate [g/g/day] | Quantitative |  | 27% | Competitiveness. *Omitted due to insufficient data coverage.* | TRY (Kattge et al. 2011) |
| rd | Rooting depth [m] | Quantitative |  | 29.5% | Nutrient acquisition strategy, stress tolerance. *Omitted due to insufficient data coverage.* | TRY (Kattge et al. 2011) |
| nit | Leaf nitrogen (N) content per leaf dry mass [mg/g] | Quantitative |  | 50% | Nutrient acquisition strategy, decomposition of litter | TRY (Kattge et al. 2011) |
| pho | Leaf phosphorus (P) content per leaf dry mass [mg/g] | Quantitative |  | 36% | Nutrient acquisition strategy, decomposition of litter | TRY (Kattge et al. 2011) |
| h | hummocks forming | Binary: 0-no, 1-yes |  | 100% | Reproduction & competitiveness (space occupation) | Moraczewski et al. 2000 |
| n | nitrogen fixation | Binary: 0-no, 1-yes |  | 59.5% | Nutrient acquisition strategy | TRY (Kattge et al. 2011) |
| MStatusO | Always mycorrhizal | Binary |  | 66% | Nutrient acquisition strategy | MycoFlor^#^ (Hempel et al. 2013; Akhmetzhanova et al. 2012; Veselkin et al. 2014) |
| MStatusN | Always non-mycorrhizal | Binary |  | 66% | Nutrient acquisition strategy, Tolerance to and ability to grow in anoxic conditions | MycoFlor^#^ (Hempel et al. 2013; Akhmetzhanova et al. 2012; Veselkin et al. 2014) |
| MFlexi | Flexible: sometimes mycorrhizal, sometimes non-mycorrhizal | Binary |  | 66% | Nutrient acquisition strategy | MycoFlor^#^ (Hempel et al. 2013; Akhmetzhanova et al. 2012; Veselkin et al. 2014; Moora, 2014) |
| **Traits indicating dispersal and recruitment potential** | | | | | | |
| sm /sm.s | seed mass [mg] | Quantitative | log-transformed,  scaled into 0-1 | 79% | Reproduction, colonisation & dispersal, competitiveness (of seedlings) | TRY (Kattge et al. 2011) |
| snb /snb.s | Seed number per ramet | Ordinal (reclassified): 0 - <100, 0.5 - 101-10000, 1 - >10 000 seeds |  | 60% | Reproduction, colonisation & dispersal | based on LEDA (Kleyer et al. 2008) |
| ge | Seed germination rate (germination efficiency) [%] | Quantitative |  | 48% | Reproduction, colonisation. *Omitted due to insufficient data coverage.* | TRY (Kattge et al. 2011) |
| seed buoyancy | Floating capacity of diaspores on water [%] | Quantitative | scaled into 0-1 | 50% | Dispersal | based on LEDA (Kleyer et al. 2008) |
| Dispersal syndromes | |  |  |  |  |  |
| autochor | (self) | Binary for each dispersal category / syndrome |  | 67% | Dispersal | based on LEDA (Kleyer et al. 2008) |
| bythisochor | (water moving) |  |  | 67% | Dispersal |  |
| chamaechor | (wind whole plant) |  |  | 67% | Dispersal |  |
| hemerochor | (antropo) |  |  | 67% | Dispersal |  |
| meteorochor | (wind) |  |  | 67% | Dispersal |  |
| nautochor | (water) |  |  | 67% | Dispersal |  |
| ombrochor | (rain drops) |  |  | 67% | Dispersal |  |
| other | (other) |  |  | 67% | Dispersal |  |
| zoochor | (animals) |  |  | 67% | Dispersal |  |
| inverb | (invertebrates) |  |  | 67% | Dispersal |  |
| mammals | (mammals) |  |  | 67% | Dispersal |  |
| diversity /diversity.s | number of syndromes | Quantitative | scaled into 0-1 | 67% | Dispersal |  |
| **Functional groups, life strategies** | | | | | | |
| PO, CY, FO, PT  BM, SPH | Ecological groups: grasses, sedges & rushes, forbs, ferns and spore plants, brown mosses en *Sphagnum* mosses | Binary: 0-no, 1-yes |  | 100% | Life strategy, e.g. tolerance to anoxic conditions (CY), competitiveness (PO) | based on taxonomy |
| pls | plant lifespan | Binary: 0-perennial, 1-annual/biennial |  | 92% | Reproduction, Nutrient acquisition strategy | Rothmaler 2009; Rutkowski 2011 |
| pha | Phanerophyte | Binary: 0-no, 1-yes |  | 100% | Competitiveness for light | based on taxonomy |
| bry | bryophyte | Binary: 0-no, 1-yes |  | 100% | Competitiveness, decomposition of litter, Tolerance to and ability to grow in anoxic conditions | based on taxonomy |
| st_c | C – competitor | strategy *sensu* Grime, fuzzy coding relative to contribution: e.g. ‘CS’= 0.5 for each C & S; ‘CSR’ = 0.33 for each strategy |  | 66.5% | Competitiveness | TRY (Kattge et al. 2011) |
| st_r | R – ruderal |  |  | 66.5% | Tolerance to disturbance |  |
| st_s | S – stress tolerator |  |  | 66.5% | Stress – tolerance |  |

^^^without Phanerophyte (trees and shrubs)

^#^Information about mycorrhizal associations (in vascular plants only) was obtained from MycoFlor database (Hempel et al., 2013), cross-checked with data from Akhmetzhanova et al. 2012, and supplemented with data from Veselkin et al. (2014) for Carex spp.

Akhmetzhanova AA, Soudzilovskaia NA, Onipchenko VG, Cornwell WK, Agafonov VA, Selivanov IA, et al. A rediscovered treasure: mycorrhizal intensity database for 3000 vascular plant species across the former Soviet Union. Ecology 2012 Mar;93(3):689–690.

Ellenberg H, et al. Zeigerwerte der Gefäßpflanzen. Scripta Geobotanica 1992;18:9-166.

Hempel S, Götzenberger L, Kühn I, Michalski SG, Rillig MC, Zobel M, et al. Mycorrhizas in the Central European flora: relationships with plant life history traits and ecology. Ecology 2013 Jun;94(6):1389–1399.

Veselkin DV, Konoplenko MA, Betekhtina AA. Means for soil nutrient uptake in sedges with different ecological strategies. Russian Journal of Ecology 2014;45(6):547–554.

Kattge J, Diaz S, Lavorel S, Prentice IC, Leadley P, Bönisch G, et al. TRY - a global database of plant traits. Glob Change Biol 2011 Sep;17(9):2905-2935.doi: 10.1111/j.1365-2486.2011.02451.x

Kleyer M, Bekker RM, Knevel IC, Bakker JP, Thompson K, Sonnenschein M, et al. The LEDA Traitbase: a database of life-history traits of the Northwest European flora. J Ecol 2008 Nov;96(6):1266–1274.doi: 10.1111/j.1365-2745.2008.01430.x

Klimešová J, De Bello F. CLO‐PLA: the database of clonal and bud bank traits of Central European flora. J Veg Sci 2009;20(3): 511-516.

Moora M. Mycorrhizal traits and plant communities: perspectives for integration. J Veg Sci 2014 Sep;25(5):1126–1132. doi: 10.1111/jvs.12177)

Moraczewski IR, Sudnik-Wójcikowska B, Dubielecka B, Rutkowski R, Nowak KA, Borkowski W, Galera H. Flora ojczysta - gatunki pospolite, chronione, ciekawe... (CD-ROM: Atlas roślin, słownik botaniczny i multimedialne klucze do oznaczania) [Polish multimedia key for vascular plants]. Warszawa: Wyd. Stigma; 2000. Polish.

Rothmaler W. Exkursionsflora von Deutschland, Band 3. Gefäßpflanzen: Atlasband., Heidelberg: Spektrum Akademischer Verlag; 2009. German.

Rutkowski L. Klucz do oznaczania roślin naczyniowych Polski niżowej [The key to determining the Polish lowland vascular plants]. 2nd ed.Warszawa: Wydawnictwo Naukowe PWN; 2011. Polish.
