# Supplementary figures and images for: Are we restoring functional fens? – The outcomes of restoration projects in fens re-analysed with plant functional traits
Source: PLoS One. 2019 Apr 24;14(4):e0215645. doi: 10.1371/journal.pone.0215645 (PMC6481837; doi:10.1371/journal.pone.0215645)

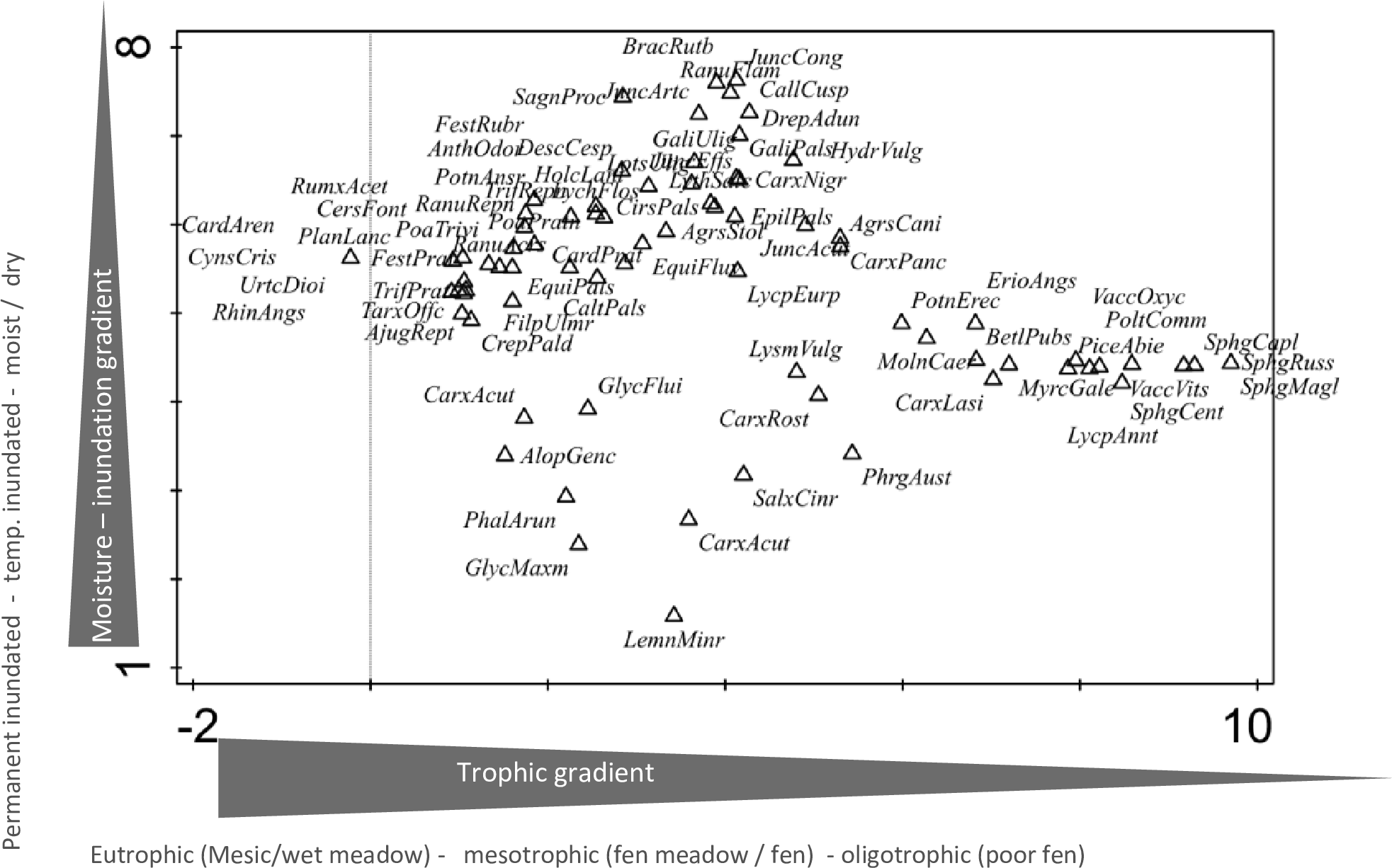

Supplement: S1 Fig — (TIF) [file pone.0215645.s005.tif]

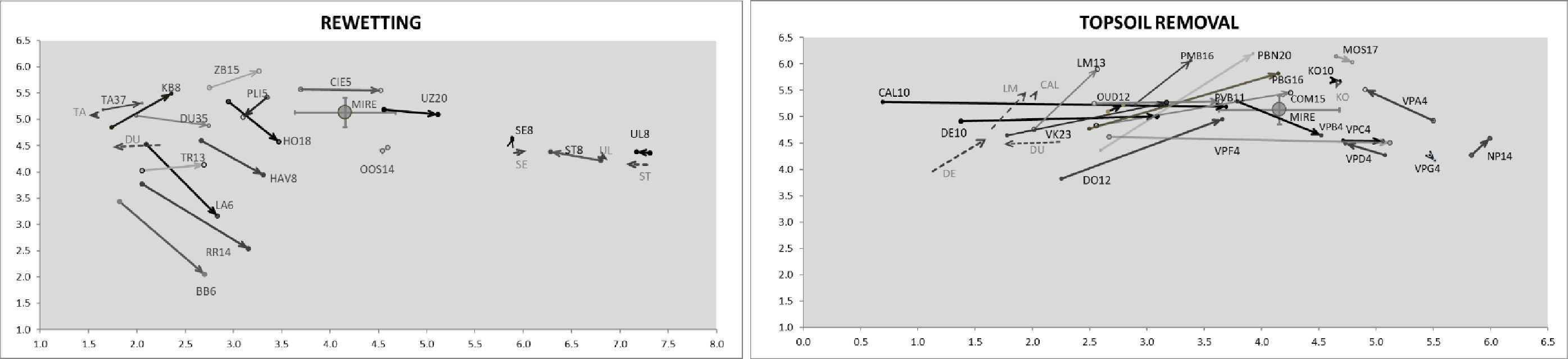

Supplement: S2 Fig — (TIF) [file pone.0215645.s006.tif]

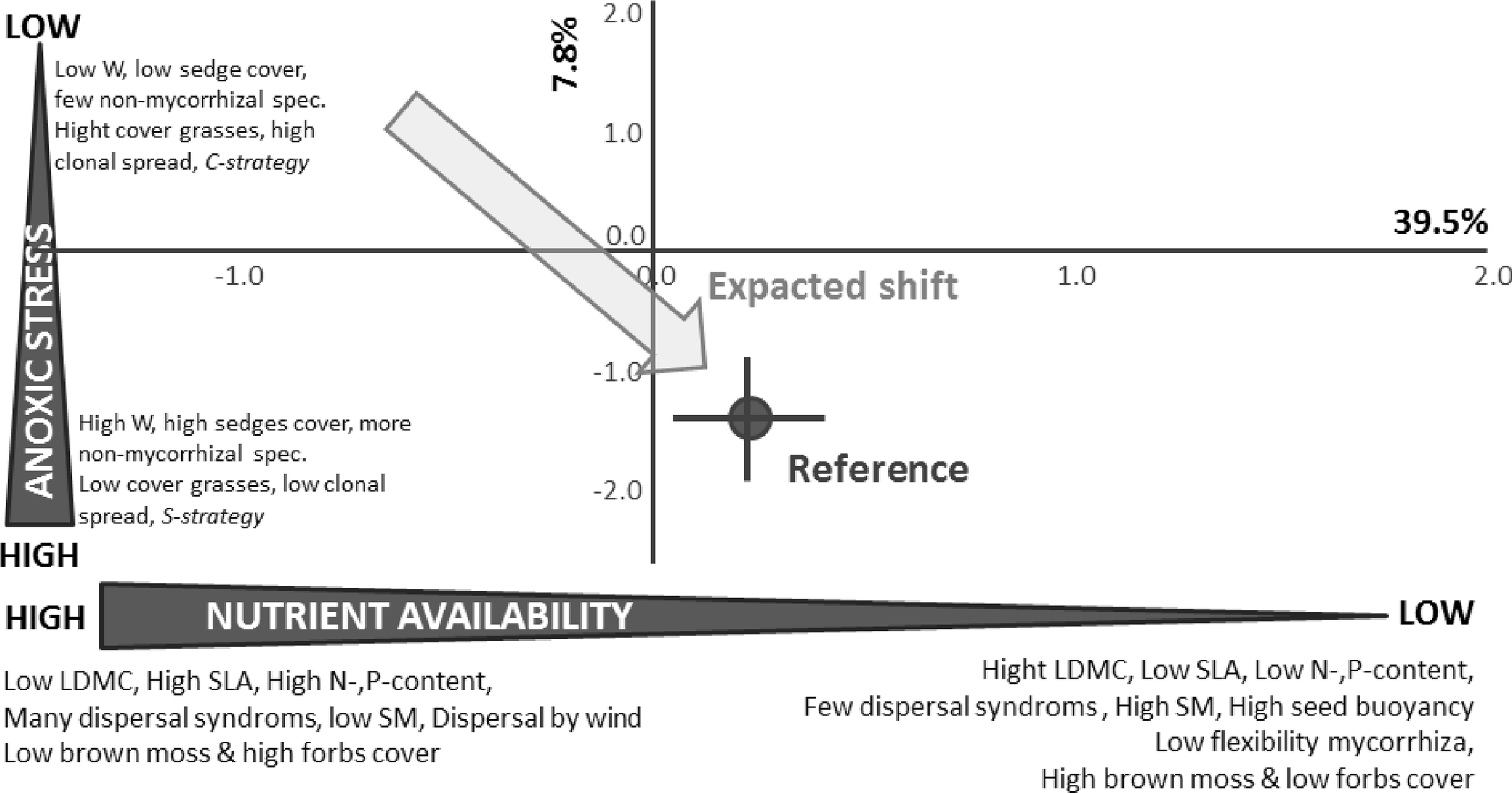

Supplement: S3 Fig — (TIF) [file pone.0215645.s007.tif]

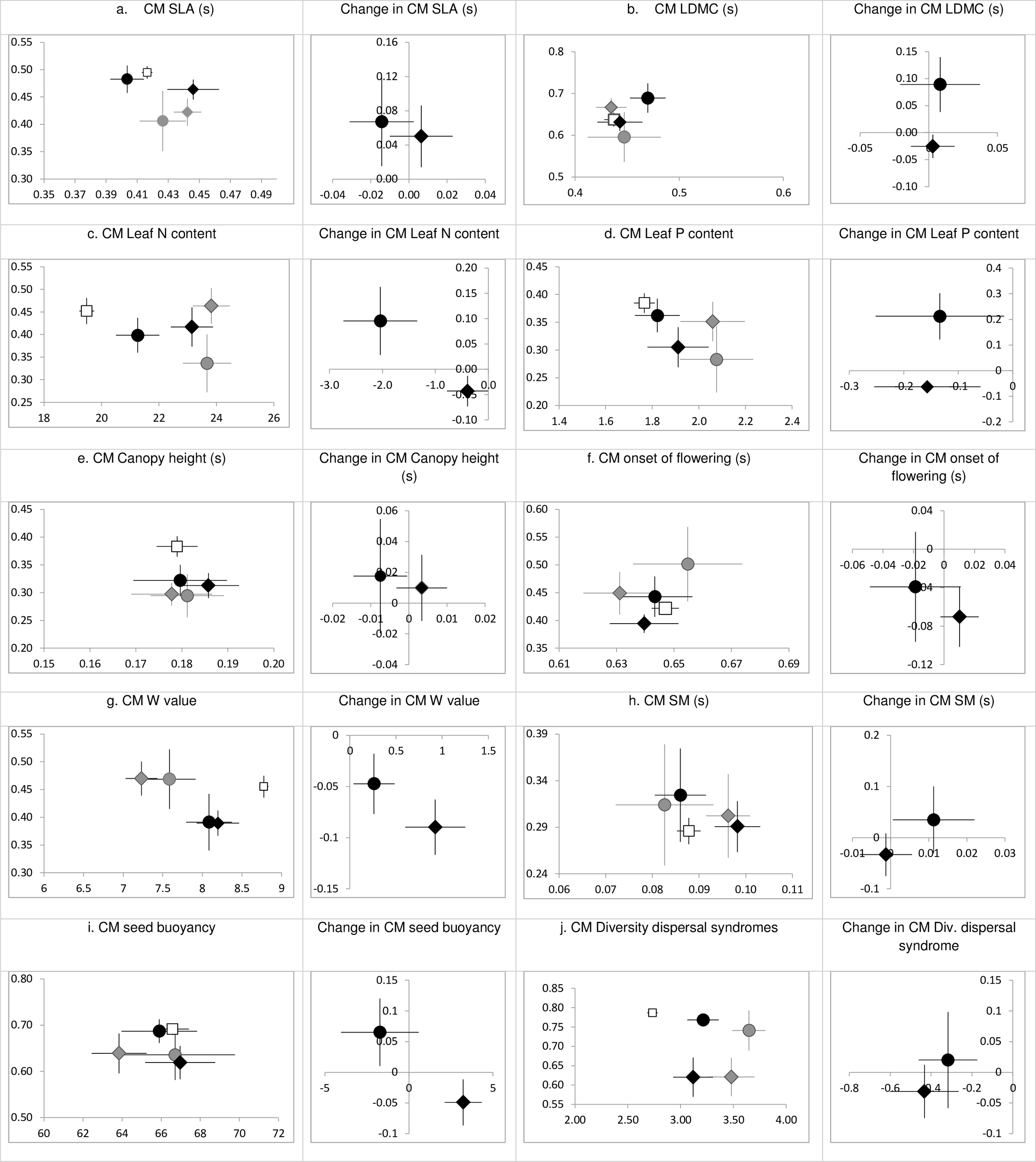

Supplement: S4 Fig — (TIF) [file pone.0215645.s008.tif]

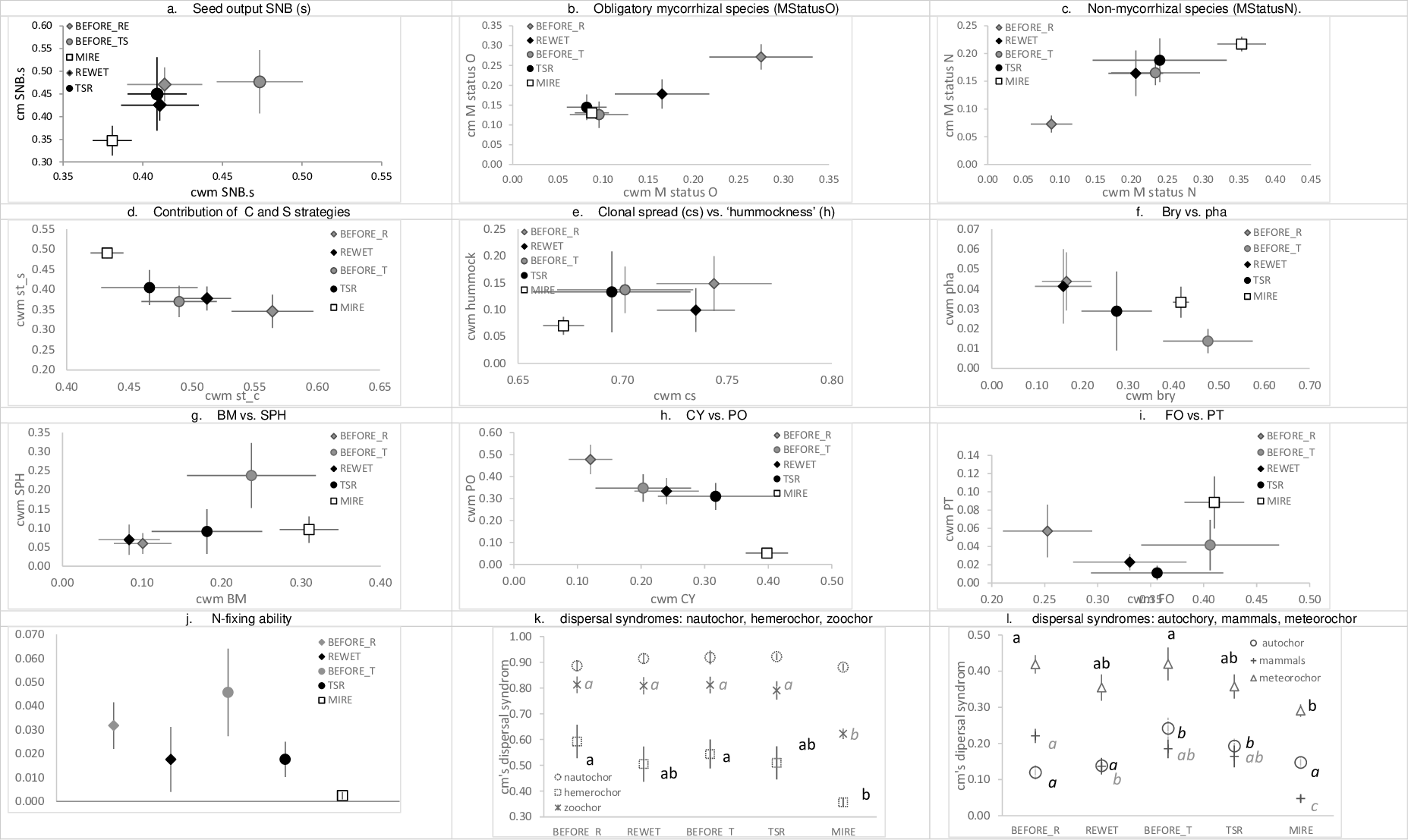

Supplement: S5 Fig — (TIF) [file pone.0215645.s009.tif]
