# Supplementary material for: Are we restoring functional fens? – The outcomes of restoration projects in fens re-analysed with plant functional traits
Source: PLoS One. 2019 Apr 24;14(4):e0215645. doi: 10.1371/journal.pone.0215645 (PMC6481837; doi:10.1371/journal.pone.0215645)
Supplement: S2 File — (PDF) [file pone.0215645.s011.pdf]

| RELEVE_NR | YEAR1 | SITE3 | TREATMENT | 1 ch | 1 ch.s | 2 sla | 2 sla.s | 3 ldmc | 3 ldmc.s | 4 cs | 5 fl | 5 fl.s | 6 w | 6 w.s | 7 nit | 8 pho | 9 h_0 non hu | 9 h_1 humm |
|---|---|---|---|---|---|---|---|---|---|---|---|---|---|---|---|---|---|---|
| 501 | 2011 | MIRE1 | MIRE | 0.4259 | 0.1614 | 23.1603 | 0.4357 | 0.2312 | 0.4341 | 0.6578 | 5.3289 | 0.6184 | 8.9533 | 0.6953 | 20.6904 | 2.3308 | 0.9145 | 0.0855 |
| 508 | 2011 | MIRE3 | MIRE | 0.4348 | 0.1658 | 19.6508 | 0.3909 | 0.2304 | 0.4321 | 0.6441 | 5.0902 | 0.5843 | 9.3689 | 0.7369 | 19.9193 | 2.4273 | 0.8770 | 0.1230 |
| 512 | 2011 | MIRE5 | MIRE | 1.8400 | 0.4477 | 15.1185 | 0.2314 | 0.2082 | 0.3798 | 0.8615 | 5.8413 | 0.6916 | 9.2222 | 0.7222 | 28.7884 | 3.9473 | 1.0000 | 0.0000 |
| 517 | 2011 | MIRE8 | MIRE | 0.5141 | 0.1941 | 19.0723 | 0.3784 | 0.2286 | 0.4278 | 0.6852 | 5.1528 | 0.5933 | 9.3188 | 0.7319 | 19.2063 | 2.3477 | 0.9583 | 0.0417 |
| 518 | 2011 | MIRE8 | MIRE | 0.4832 | 0.1828 | 19.8949 | 0.3884 | 0.2364 | 0.4462 | 0.6877 | 5.0842 | 0.5835 | 9.2211 | 0.7221 | 20.0569 | 2.4757 | 0.9789 | 0.0211 |
| 521 | 2011 | MIRE9 | MIRE | 0.3621 | 0.1391 | 21.1035 | 0.4023 | 0.2230 | 0.4147 | 0.6667 | 5.2182 | 0.6026 | 9.7545 | 0.7755 | 21.0002 | 2.6162 | 0.9727 | 0.0273 |
| 522 | 2011 | MIRE9 | MIRE | 0.4432 | 0.1614 | 19.4652 | 0.3868 | 0.2335 | 0.4395 | 0.6824 | 5.0455 | 0.5779 | 9.2818 | 0.7282 | 20.9492 | 2.5496 | 0.9818 | 0.0182 |
| 523 | 2011 | MIRE10 | MIRE | 0.6323 | 0.2162 | 15.6892 | 0.3171 | 0.3406 | 0.6915 | 0.7016 | 5.6947 | 0.6707 | 8.8387 | 0.6839 | 17.7061 | 1.4393 | 0.9789 | 0.0211 |
| 524 | 2011 | MIRE10 | MIRE | 0.5223 | 0.1864 | 15.1985 | 0.3133 | 0.3543 | 0.7237 | 0.6667 | 5.8333 | 0.6905 | 9.2292 | 0.7229 | 17.1429 | 1.4220 | 0.9479 | 0.0521 |
| 525 | 2011 | MIRE11 | MIRE | 0.4665 | 0.1781 | 20.0803 | 0.3909 | 0.2271 | 0.4244 | 0.5566 | 5.0943 | 0.5849 | 9.0680 | 0.7068 | 18.1206 | 1.8205 | 0.6415 | 0.3585 |
| 530 | 2011 | MIRE12 | MIRE | 0.4115 | 0.1532 | 25.3031 | 0.4669 | 0.2327 | 0.4375 | 0.6637 | 5.2389 | 0.6056 | 8.8407 | 0.6841 | 22.9075 | 2.8261 | 0.9646 | 0.0354 |
| 537 | 2011 | MIRE15 | MIRE | 0.5794 | 0.2083 | 17.0311 | 0.3371 | 0.2902 | 0.5729 | 0.6630 | 5.5106 | 0.6444 | 9.0426 | 0.7043 | 18.8370 | 1.8903 | 0.9362 | 0.0638 |
| 551 | 2012 | MIRE23 | MIRE | 0.4082 | 0.1539 | 19.2601 | 0.3726 | 0.2387 | 0.4517 | 0.6810 | 5.3290 | 0.6184 | 9.2968 | 0.7297 | 19.2803 | 2.0585 | 0.8968 | 0.1032 |
| 553 | 2012 | MIRE24 | MIRE | 0.3549 | 0.1335 | 21.1581 | 0.3971 | 0.2467 | 0.4705 | 0.6822 | 5.7333 | 0.6762 | 8.5282 | 0.6528 | 18.5006 | 1.4290 | 0.9804 | 0.0196 |
| 554 | 2012 | MIRE24 | MIRE | 0.3867 | 0.1474 | 21.4241 | 0.4082 | 0.2545 | 0.4889 | 0.6491 | 5.6250 | 0.6607 | 8.4865 | 0.6486 | 17.2875 | 1.2381 | 0.9535 | 0.0465 |
| 556 | 2012 | MIRE25 | MIRE | 0.6359 | 0.2271 | 19.0483 | 0.3752 | 0.2361 | 0.4455 | 0.6705 | 5.3448 | 0.6207 | 9.4253 | 0.7425 | 18.4987 | 2.1426 | 0.9310 | 0.0690 |
| 559 | 2012 | MIRE28 | MIRE | 0.2885 | 0.1145 | 21.0368 | 0.3883 | 0.2514 | 0.4815 | 0.6167 | 5.7238 | 0.6748 | 8.7553 | 0.6755 | 17.9424 | 1.3490 | 0.9429 | 0.0571 |
| 561 | 2012 | MIRE29 | MIRE | 0.3743 | 0.1447 | 17.8471 | 0.3494 | 0.2594 | 0.5004 | 0.6458 | 5.3678 | 0.6240 | 8.9880 | 0.6988 | 19.4124 | 1.8256 | 0.9326 | 0.0674 |
| 564 | 2012 | MIRE30 | MIRE | 1.4984 | 0.3806 | 13.8558 | 0.2490 | 0.2922 | 0.5776 | 0.8436 | 6.0568 | 0.7224 | 8.9792 | 0.6979 | 27.4271 | 3.1060 | 0.9778 | 0.0222 |
| 565 | 2012 | MIRE31 | MIRE | 0.4604 | 0.1740 | 21.1881 | 0.4100 | 0.2160 | 0.3982 | 0.7013 | 5.1280 | 0.5897 | 9.2276 | 0.7228 | 20.6382 | 2.4881 | 1.0000 | 0.0000 |
| 566 | 2012 | MIRE31 | MIRE | 0.3938 | 0.1508 | 22.9859 | 0.4357 | 0.2017 | 0.3646 | 0.6790 | 5.1704 | 0.5958 | 9.0075 | 0.7008 | 22.5823 | 2.8167 | 1.0000 | 0.0000 |
| 567 | 2012 | MIRE32 | MIRE | 0.2471 | 0.0998 | 16.0833 | 0.3156 | 0.2923 | 0.5778 | 0.6559 | 5.5258 | 0.6465 | 8.4615 | 0.6462 | 17.1458 | 1.2643 | 0.8660 | 0.1340 |
| 568 | 2012 | MIRE32 | MIRE | 0.2413 | 0.0965 | 15.1633 | 0.2920 | 0.2814 | 0.5521 | 0.6610 | 5.4262 | 0.6323 | 8.8803 | 0.6880 | 18.6396 | 1.3195 | 0.9758 | 0.0242 |
| 569 | 2012 | MIRE33 | MIRE | 0.4416 | 0.1643 | 20.5940 | 0.4001 | 0.2548 | 0.4895 | 0.6874 | 5.2595 | 0.6085 | 8.9211 | 0.6921 | 18.6317 | 1.9107 | 1.0000 | 0.0000 |
| 571 | 2012 | MIRE34 | MIRE | 0.5018 | 0.1547 | 16.6393 | 0.3026 | 0.2596 | 0.5008 | 0.6886 | 5.4610 | 0.6373 | 8.8540 | 0.6854 | 20.9352 | 1.9151 | 0.9610 | 0.0390 |
| 572 | 2012 | MIRE34 | MIRE | 0.4427 | 0.1638 | 26.4445 | 0.4671 | 0.1897 | 0.3363 | 0.6748 | 6.1496 | 0.7357 | 8.7500 | 0.6750 | 20.1185 | 1.3781 | 0.9846 | 0.0154 |
| 573 | 2012 | MIRE35 | MIRE | 0.4836 | 0.1817 | 21.4399 | 0.4070 | 0.2233 | 0.4155 | 0.6701 | 5.3402 | 0.6200 | 8.6598 | 0.6660 | 19.2202 | 2.0211 | 0.8660 | 0.1340 |
| 574 | 2012 | MIRE35 | MIRE | 0.4050 | 0.1562 | 24.3073 | 0.4453 | 0.2365 | 0.4464 | 0.6825 | 5.4408 | 0.6344 | 8.7039 | 0.6704 | 20.4889 | 2.0482 | 0.9671 | 0.0329 |
| 575 | 2012 | MIRE35 | MIRE | 0.4252 | 0.1610 | 20.5891 | 0.3869 | 0.2577 | 0.4963 | 0.7041 | 5.2100 | 0.6014 | 8.6500 | 0.6650 | 19.0249 | 1.8642 | 0.9400 | 0.0600 |
| 576 | 2012 | MIRE36 | MIRE | 0.5254 | 0.1935 | 20.3545 | 0.3959 | 0.2018 | 0.3649 | 0.6214 | 5.3398 | 0.6200 | 8.9417 | 0.6942 | 21.5976 | 2.3208 | 0.9126 | 0.0874 |
| 578 | 2012 | MIRE37 | MIRE | 0.4750 | 0.1793 | 20.0488 | 0.3941 | 0.2378 | 0.4494 | 0.6771 | 5.5859 | 0.6551 | 9.3434 | 0.7343 | 20.0817 | 1.8236 | 0.9495 | 0.0505 |
| 582 | 2012 | MIRE39 | MIRE | 0.3656 | 0.1410 | 20.4038 | 0.3837 | 0.2599 | 0.5015 | 0.6375 | 5.5126 | 0.6447 | 8.7479 | 0.6748 | 18.6275 | 1.5117 | 0.9748 | 0.0252 |
| 584 | 2012 | MIRE40 | MIRE | 0.4845 | 0.1797 | 20.8022 | 0.4047 | 0.2187 | 0.4045 | 0.6558 | 5.1883 | 0.5983 | 9.0130 | 0.7013 | 21.5711 | 2.5653 | 0.8961 | 0.1039 |
| 586 | 2012 | MIRE41 | MIRE | 0.4771 | 0.1804 | 19.0170 | 0.3695 | 0.2404 | 0.4556 | 0.6993 | 5.0652 | 0.5807 | 9.1739 | 0.7174 | 20.1485 | 2.2483 | 1.0000 | 0.0000 |
| 590 | 2012 | MIRE44 | MIRE | 0.5768 | 0.2007 | 21.1400 | 0.4020 | 0.2535 | 0.4866 | 0.6756 | 5.2720 | 0.6103 | 9.4480 | 0.7448 | 18.0799 | 1.3585 | 0.6308 | 0.3308 |
| 592 | 2012 | MIRE45 | MIRE | 0.5596 | 0.2025 | 19.5175 | 0.3687 | 0.2512 | 0.4811 | 0.6867 | 4.9699 | 0.5671 | 9.3233 | 0.7323 | 19.0125 | 1.9971 | 0.8647 | 0.1353 |
| 601 | 2013 | MIRE23 | MIRE | 0.4504 | 0.1691 | 21.7264 | 0.4170 | 0.2271 | 0.4243 | 0.6911 | 5.4312 | 0.6330 | 8.9358 | 0.6936 | 20.6176 | 2.4290 | 0.9817 | 0.0183 |
| 602 | 2013 | MIRE23 | MIRE | 0.4296 | 0.1601 | 21.9905 | 0.4177 | 0.2232 | 0.4151 | 0.6795 | 5.3019 | 0.6146 | 8.8868 | 0.6887 | 20.6300 | 2.3568 | 0.9717 | 0.0283 |
| 606 | 2013 | MIRE46 | MIRE | 0.7077 | 0.2445 | 23.2862 | 0.4092 | 0.2675 | 0.5194 | 0.8542 | 4.6250 | 0.5179 | 8.8203 | 0.6820 | 14.0429 | 0.9422 | 0.9766 | 0.0234 |
| 616 | 2013 | MIRE7 | MIRE | 0.4235 | 0.1613 | 17.0763 | 0.3367 | 0.3131 | 0.6267 | 0.6460 | 5.5826 | 0.6547 | 8.7857 | 0.6786 | 17.2939 | 1.2667 | 0.9304 | 0.0696 |
| 617 | 2013 | MIRE2 | MIRE | 0.5725 | 0.2100 | 26.5365 | 0.4666 | 0.1967 | 0.3528 | 0.6781 | 6.5470 | 0.7924 | 8.3675 | 0.6368 | 15.2173 | 1.2618 | 1.0000 | 0.0000 |
| 619 | 2013 | MIRE2 | MIRE | 0.6521 | 0.2347 | 28.1444 | 0.4990 | 0.1746 | 0.3007 | 0.6632 | 6.5368 | 0.7910 | 8.2211 | 0.6221 | 17.3382 | 1.7919 | 0.9684 | 0.0316 |
| 621 | 2013 | MIRE49 | MIRE | 0.5243 | 0.1925 | 25.5629 | 0.4633 | 0.2231 | 0.4149 | 0.6957 | 5.8957 | 0.6994 | 8.6000 | 0.6600 | 19.3059 | 1.8512 | 0.9826 | 0.0174 |
| 627 | 2013 | MIRE51 | MIRE | 0.5654 | 0.2020 | 15.3555 | 0.3046 | 0.3296 | 0.6655 | 0.7674 | 5.8922 | 0.6989 | 9.3333 | 0.7333 | 19.2735 | 1.5020 | 0.9706 | 0.0294 |
| 628 | 2013 | MIRE51 | MIRE | 0.3079 | 0.1205 | 17.0068 | 0.3302 | 0.2918 | 0.5766 | 0.6552 | 5.3694 | 0.6242 | 9.1911 | 0.7191 | 19.2260 | 1.7899 | 0.9554 | 0.0446 |
| 639 | 2013 | MIRE52 | MIRE | 0.4831 | 0.1733 | 22.5552 | 0.4201 | 0.2167 | 0.4000 | 0.6667 | 5.4074 | 0.6296 | 9.2500 | 0.7250 | 21.6575 | 2.4762 | 0.9815 | 0.0185 |
| 641 | 2013 | MIRE53 | MIRE | 0.6352 | 0.2250 | 24.1180 | 0.4373 | 0.2074 | 0.3779 | 0.6667 | 6.0882 | 0.7269 | 8.6324 | 0.6632 | 17.7689 | 1.8089 | 0.9559 | 0.0441 |
| 647 | 2013 | MIRE39 | MIRE | 0.5714 | 0.2074 | 21.0870 | 0.3887 | 0.1907 | 0.3388 | 0.7146 | 6.0523 | 0.7218 | 8.3333 | 0.6333 | 23.7606 | 1.3384 | 0.9477 | 0.0523 |
| 650 | 2013 | MIRE44 | MIRE | 0.5835 | 0.2066 | 22.8173 | 0.4222 | 0.2387 | 0.4516 | 0.6590 | 5.5169 | 0.6453 | 8.8876 | 0.6888 | 17.4862 | 1.2012 | 0.7528 | 0.2472 |
| 652 | 2013 | MIRE44 | MIRE | 0.6407 | 0.2266 | 17.7560 | 0.3324 | 0.3095 | 0.6182 | 0.6951 | 5.6190 | 0.6599 | 9.1429 | 0.7143 | 17.8574 | 1.6706 | 0.9286 | 0.0714 |

| | | | | | | | | | | | | | | | | | | |
|---|---|---|---|---|---|---|---|---|---|---|---|---|---|---|---|---|---|---|
| 654 | 2013 | MIRE42 | MIRE | 0.4131 | 0.1602 | 20.5249 | 0.3836 | 0.2664 | 0.5169 | 0.6597 | 5.4966 | 0.6424 | 8.7063 | 0.6706 | 19.5162 | 1.5591 | 0.9793 | 0.0207 |
| 655 | 2013 | MIRE41 | MIRE | 0.5732 | 0.2120 | 17.5028 | 0.3584 | 0.2463 | 0.4695 | 0.6794 | 5.1333 | 0.5905 | 9.4369 | 0.7437 | 19.0280 | 2.3056 | 0.9810 | 0.0190 |
| 657 | 2013 | MIRE54 | MIRE | 0.5242 | 0.1926 | 23.6373 | 0.4321 | 0.2410 | 0.4570 | 0.7218 | 5.8760 | 0.6966 | 8.5537 | 0.6554 | 20.0738 | 1.6621 | 0.9752 | 0.0248 |
| 659 | 2013 | MIRE25 | MIRE | 0.7051 | 0.2449 | 20.2444 | 0.3893 | 0.2416 | 0.4584 | 0.5183 | 5.7603 | 0.6800 | 8.7113 | 0.6711 | 23.1141 | 2.1699 | 0.5205 | 0.4795 |
| 660 | 2013 | MIRE28 | MIRE | 0.3182 | 0.1256 | 21.8418 | 0.4178 | 0.2743 | 0.5354 | 0.6417 | 5.5421 | 0.6489 | 8.3878 | 0.6388 | 17.6255 | 1.3976 | 0.9720 | 0.0280 |
| 661 | 2013 | MIRE28 | MIRE | 0.3932 | 0.1515 | 18.7630 | 0.3617 | 0.2833 | 0.5566 | 0.6485 | 5.6577 | 0.6654 | 8.5486 | 0.6549 | 17.4717 | 1.3630 | 0.9799 | 0.0201 |
| 662 | 2013 | MIRE31 | MIRE | 0.5421 | 0.1996 | 20.8444 | 0.3952 | 0.2176 | 0.4019 | 0.5648 | 5.3056 | 0.6151 | 8.5146 | 0.6515 | 18.7144 | 1.4981 | 0.6481 | 0.3519 |
| 663 | 2013 | MIRE31 | MIRE | 0.6027 | 0.2163 | 20.5554 | 0.3901 | 0.2466 | 0.4702 | 0.6962 | 5.1304 | 0.5901 | 8.6339 | 0.6634 | 18.4466 | 1.5684 | 0.8435 | 0.1565 |
| 664 | 2013 | MIRE31 | MIRE | 0.5200 | 0.1923 | 18.9805 | 0.3789 | 0.2604 | 0.5027 | 0.7025 | 5.2231 | 0.6033 | 8.7155 | 0.6716 | 19.4504 | 2.0837 | 0.9587 | 0.0413 |
| 672 | 2014 | MIRE56 | MIRE | 0.4068 | 0.1569 | 21.4190 | 0.4101 | 0.2376 | 0.4490 | 0.6980 | 5.2235 | 0.6034 | 8.4268 | 0.6427 | 20.6458 | 2.1523 | 0.9647 | 0.0353 |
| BB_02a | 2002 | BB | BB_REWET | 1.5626 | 0.4424 | 18.0106 | 0.3751 | 0.2998 | 0.5954 | 0.9963 | 6.0787 | 0.7255 | 9.9944 | 0.7994 | 18.7088 | 2.1302 | 1.0000 | 0.0000 |
| BB_02b | 2002 | BB | BB_REWET | 1.5489 | 0.4338 | 21.2874 | 0.4029 | 0.2785 | 0.5453 | 0.9606 | 5.9113 | 0.7016 | 10.0739 | 0.8074 | 18.5448 | 2.1916 | 1.0000 | 0.0000 |
| BB_02c | 2002 | BB | BB_REWET | 1.2286 | 0.3641 | 22.7071 | 0.4061 | 0.2857 | 0.5623 | 0.9441 | 5.1156 | 0.5879 | 9.6168 | 0.7617 | 18.7495 | 1.7630 | 1.0000 | 0.0000 |
| BB_02d | 2002 | BB | BB_REWET | 0.7605 | 0.2285 | 46.3659 | 0.5827 | 0.1965 | 0.3522 | 0.7113 | 5.1464 | 0.5923 | 9.3178 | 0.7318 | 24.6345 | 2.2813 | 1.0000 | 0.0000 |
| BB_02e | 2002 | BB | BB_REWET | 1.6362 | 0.4583 | 18.3822 | 0.3816 | 0.2871 | 0.5656 | 0.9961 | 5.9767 | 0.7110 | 10.0000 | 0.8000 | 18.0169 | 2.1906 | 1.0000 | 0.0000 |
| BB_02f | 2002 | BB | BB_REWET | 0.5288 | 0.1480 | 58.4564 | 0.6651 | 0.1712 | 0.2927 | 0.5470 | 4.8782 | 0.5540 | 10.7981 | 0.8798 | 24.6913 | 2.2000 | 1.0000 | 0.0000 |
| BB_02g | 2002 | BB | BB_REWET | 1.0899 | 0.3051 | 38.0919 | 0.5215 | 0.2299 | 0.4309 | 0.7737 | 5.4495 | 0.6356 | 10.4037 | 0.8404 | 21.3413 | 2.2000 | 1.0000 | 0.0000 |
| BB_96a | 1996 | BB | BB_BEFORE | 1.1026 | 0.3431 | 21.0488 | 0.4193 | 0.2581 | 0.4972 | 0.9569 | 5.9010 | 0.7001 | 7.8832 | 0.5883 | 23.8771 | 2.3694 | 0.9949 | 0.0051 |
| BB_96b | 1996 | BB | BB_BEFORE | 1.1099 | 0.3451 | 22.2469 | 0.4336 | 0.2259 | 0.4216 | 0.9067 | 5.6927 | 0.6704 | 7.7196 | 0.5720 | 23.0181 | 2.4678 | 0.9908 | 0.0092 |
| BB_96c | 1996 | BB | BB_BEFORE | 1.1750 | 0.3622 | 21.7687 | 0.4287 | 0.2544 | 0.4886 | 0.9196 | 5.8344 | 0.6906 | 7.6567 | 0.5657 | 23.3479 | 2.5499 | 1.0000 | 0.0000 |
| BB_96d | 1996 | BB | BB_BEFORE | 0.8462 | 0.2799 | 23.5337 | 0.4500 | 0.2306 | 0.4326 | 0.8004 | 5.4412 | 0.6345 | 6.6111 | 0.4611 | 24.9038 | 2.5509 | 0.9958 | 0.0042 |
| BB_96e | 1996 | BB | BB_BEFORE | 1.2561 | 0.3771 | 20.9879 | 0.4176 | 0.2595 | 0.5006 | 0.9190 | 5.8336 | 0.6905 | 8.2707 | 0.6271 | 22.1916 | 2.4733 | 0.9963 | 0.0037 |
| BB_96f | 1996 | BB | BB_BEFORE | 1.0861 | 0.3321 | 20.3111 | 0.4112 | 0.2489 | 0.4755 | 0.9861 | 5.8281 | 0.6897 | 7.7757 | 0.5776 | 22.8296 | 2.4044 | 0.9896 | 0.0052 |
| BB_96g | 1996 | BB | BB_BEFORE | 0.9221 | 0.2976 | 22.7660 | 0.4398 | 0.2347 | 0.4423 | 0.8800 | 5.6279 | 0.6611 | 7.1951 | 0.5195 | 24.4354 | 2.5866 | 0.9963 | 0.0037 |
| CAL_14_115 | 2014 | CAL | CAL_TSR | 0.6466 | 0.2072 | 51.1391 | 0.4909 | 0.1875 | 0.3312 | 0.7963 | 5.5833 | 0.6548 | 10.4167 | 0.8417 | 21.2318 | 1.7489 | 1.0000 | 0.0000 |
| CAL_14_119 | 2014 | CAL | CAL_TSR | 0.6097 | 0.2183 | 17.7495 | 0.3562 | 0.2611 | 0.5043 | 0.7083 | 5.3750 | 0.6250 | 9.3750 | 0.7375 | 18.1436 | 1.8342 | 0.9375 | 0.0625 |
| CAL_14_123 | 2014 | CAL | CAL_TSR | 0.5222 | 0.1941 | 21.2523 | 0.4146 | 0.3068 | 0.6118 | 0.5366 | 5.4390 | 0.6341 | 9.1463 | 0.7146 | 20.7195 | 1.6520 | 1.0000 | 0.0000 |
| CAL_14_127 | 2014 | CAL | CAL_TSR | 0.8098 | 0.2676 | 15.7067 | 0.3199 | 0.3085 | 0.6159 | 0.8000 | 5.6000 | 0.6571 | 9.6000 | 0.7600 | 13.3667 | 1.0839 | 1.0000 | 0.0000 |
| CAL_14_148 | 2014 | CAL | CAL_TSR | 0.4399 | 0.1547 | 20.0835 | 0.3949 | 0.3189 | 0.6403 | 0.6667 | 5.3415 | 0.6202 | 8.1951 | 0.6195 | 17.3359 | 1.6011 | 0.9512 | 0.0488 |
| CAL_14_152 | 2014 | CAL | CAL_TSR | 0.3517 | 0.1362 | 20.4124 | 0.4029 | 0.3193 | 0.6414 | 0.6667 | 5.3256 | 0.6179 | 8.2326 | 0.6233 | 17.6048 | 1.5798 | 0.9535 | 0.0465 |
| CAL_14_156 | 2014 | CAL | CAL_TSR | 0.3895 | 0.1490 | 20.5134 | 0.4050 | 0.3008 | 0.5979 | 0.6608 | 5.2807 | 0.6115 | 8.6491 | 0.6649 | 18.1489 | 1.8936 | 0.9649 | 0.0351 |
| CAL_14_160 | 2014 | CAL | CAL_TSR | 0.4694 | 0.1711 | 20.4917 | 0.3945 | 0.2849 | 0.5602 | 0.6111 | 5.7917 | 0.6845 | 8.5417 | 0.6542 | 18.0165 | 2.2256 | 0.8750 | 0.1250 |
| CAL_14_177 | 2014 | CAL | CAL_TSR | 0.7155 | 0.2453 | 22.2122 | 0.4052 | 0.2193 | 0.4060 | 0.6333 | 5.9000 | 0.7000 | 8.9000 | 0.6900 | 17.7405 | 1.7335 | 1.0000 | 0.0000 |
| CAL_14_181 | 2014 | CAL | CAL_TSR | 0.6731 | 0.2375 | 24.0113 | 0.4406 | 0.2278 | 0.4259 | 0.6333 | 6.0000 | 0.7143 | 8.7500 | 0.6750 | 17.7000 | 1.7894 | 1.0000 | 0.0000 |
| CAL_14_185 | 2014 | CAL | CAL_TSR | 0.5140 | 0.1911 | 20.8335 | 0.3857 | 0.2765 | 0.5407 | 0.5877 | 5.6316 | 0.6617 | 8.1316 | 0.6132 | 20.2568 | 1.7551 | 0.7895 | 0.2105 |
| CAL_14_189 | 2014 | CAL | CAL_TSR | 0.4796 | 0.1785 | 22.5742 | 0.4138 | 0.2648 | 0.5131 | 0.6083 | 5.4500 | 0.6357 | 8.5500 | 0.6550 | 21.0359 | 1.8188 | 0.8000 | 0.2000 |
| CAL_14_19 | 2014 | CAL | CAL_TSR | 0.5606 | 0.2005 | 21.4919 | 0.4039 | 0.2597 | 0.5012 | 0.6250 | 5.8750 | 0.6964 | 8.8125 | 0.6813 | 18.9801 | 2.5254 | 0.8125 | 0.1875 |
| CAL_14_193 | 2014 | CAL | CAL_TSR | 0.6372 | 0.2275 | 21.0526 | 0.3595 | 0.2398 | 0.4542 | 0.7027 | 5.1053 | 0.5865 | 9.9211 | 0.7921 | 16.1624 | 1.9599 | 1.0000 | 0.0000 |
| CAL_14_197 | 2014 | CAL | CAL_TSR | 0.6152 | 0.2237 | 16.7605 | 0.3492 | 0.2509 | 0.4804 | 0.6979 | 5.1563 | 0.5938 | 9.8125 | 0.7813 | 18.0295 | 2.2805 | 1.0000 | 0.0000 |
| CAL_14_201 | 2014 | CAL | CAL_TSR | 0.5878 | 0.2155 | 18.6527 | 0.3754 | 0.2536 | 0.4867 | 0.6250 | 5.2250 | 0.6036 | 9.6500 | 0.7650 | 18.1869 | 2.3225 | 1.0000 | 0.0000 |
| CAL_14_205 | 2014 | CAL | CAL_TSR | 0.7296 | 0.2457 | 19.1507 | 0.3611 | 0.2557 | 0.4917 | 0.7083 | 6.1250 | 0.7321 | 9.1250 | 0.7125 | 20.3074 | 1.5133 | 1.0000 | 0.0000 |
| CAL_14_228 | 2014 | CAL | CAL_TSR | 0.6744 | 0.2422 | 16.5574 | 0.3442 | 0.3222 | 0.6482 | 0.7931 | 6.0000 | 0.7143 | 9.8448 | 0.7845 | 21.1834 | 1.9258 | 1.0000 | 0.0000 |
| CAL_14_23 | 2014 | CAL | CAL_TSR | 0.6549 | 0.2343 | 13.9799 | 0.2425 | 0.2628 | 0.5083 | 0.6074 | 5.8000 | 0.6857 | 7.7556 | 0.5756 | 16.9877 | 1.8106 | 0.4222 | 0.5778 |
| CAL_14_232 | 2014 | CAL | CAL_TSR | 0.6232 | 0.2244 | 17.1284 | 0.3471 | 0.2406 | 0.4560 | 0.6937 | 5.2703 | 0.6100 | 9.6486 | 0.7649 | 17.5760 | 2.2481 | 0.9730 | 0.0270 |
| CAL_14_236 | 2014 | CAL | CAL_TSR | 0.6474 | 0.2333 | 17.9436 | 0.3622 | 0.2614 | 0.5051 | 0.7143 | 5.5238 | 0.6463 | 9.7143 | 0.7714 | 19.1715 | 2.1514 | 0.9762 | 0.0238 |
| CAL_14_240 | 2014 | CAL | CAL_TSR | 0.6880 | 0.2418 | 18.7855 | 0.3677 | 0.2893 | 0.5707 | 0.7500 | 6.0000 | 0.7143 | 9.5500 | 0.7550 | 20.8441 | 1.8954 | 0.9500 | 0.0500 |
| CAL_14_257 | 2014 | CAL | CAL_TSR | 0.4788 | 0.1781 | 23.5539 | 0.4280 | 0.2554 | 0.4910 | 0.6296 | 5.9630 | 0.7090 | 8.3704 | 0.6370 | 20.4969 | 2.8228 | 0.8704 | 0.1296 |
| CAL_14_261 | 2014 | CAL | CAL_TSR | 0.6503 | 0.2305 | 15.6981 | 0.2699 | 0.2565 | 0.4936 | 0.6400 | 5.9400 | 0.7057 | 7.7000 | 0.5700 | 18.3400 | 2.1372 | 0.4800 | 0.5200 |
| CAL_14_265 | 2014 | CAL | CAL_TSR | 0.7218 | 0.2512 | 22.4869 | 0.4275 | 0.2297 | 0.4305 | 0.8527 | 5.2791 | 0.6113 | 7.9070 | 0.5907 | 20.4602 | 1.8663 | 0.9767 | 0.0233 |
| CAL_14_269 | 2014 | CAL | CAL_TSR | 0.5694 | 0.2034 | 22.7832 | 0.4252 | 0.2276 | 0.4256 | 0.7193 | 5.6579 | 0.6654 | 8.2105 | 0.6211 | 19.9123 | 2.1779 | 0.9474 | 0.0526 |
| CAL_14_27 | 2014 | CAL | CAL_TSR | 0.5925 | 0.2178 | 17.7329 | 0.3519 | 0.2531 | 0.4854 | 0.6111 | 5.5000 | 0.6429 | 9.1111 | 0.7111 | 17.5324 | 1.9494 | 0.7778 | 0.2222 |

| | | | | | | | | | | | | | | | | | | |
|---|---|---|---|---|---|---|---|---|---|---|---|---|---|---|---|---|---|---|
| CAL_14_292 | 2014 | CAL | CAL_TSR | 0.5663 | 0.2045 | 23.5778 | 0.4387 | 0.2452 | 0.4668 | 0.6458 | 5.5625 | 0.6518 | 8.4688 | 0.6469 | 20.5332 | 2.2797 | 0.9688 | 0.0313 |
| CAL_14_296 | 2014 | CAL | CAL_TSR | 0.5431 | 0.1983 | 17.8665 | 0.3223 | 0.2673 | 0.5190 | 0.6715 | 5.8986 | 0.6998 | 8.0000 | 0.6000 | 18.8102 | 2.4760 | 0.6232 | 0.3768 |
| CAL_14_300 | 2014 | CAL | CAL_TSR | 0.6339 | 0.2264 | 22.0390 | 0.4176 | 0.2447 | 0.4658 | 0.6667 | 5.5625 | 0.6518 | 8.0938 | 0.6094 | 23.5260 | 2.1231 | 0.9375 | 0.0625 |
| CAL_14_304 | 2014 | CAL | CAL_TSR | 0.6472 | 0.2316 | 21.5392 | 0.4182 | 0.2533 | 0.4860 | 0.7356 | 5.3276 | 0.6182 | 7.9655 | 0.5966 | 21.7015 | 1.8898 | 0.8966 | 0.1034 |
| CAL_14_31 | 2014 | CAL | CAL_TSR | 0.6321 | 0.2269 | 16.0469 | 0.2843 | 0.2702 | 0.5258 | 0.6257 | 5.7895 | 0.6842 | 7.7368 | 0.5737 | 19.2869 | 1.7940 | 0.5263 | 0.4737 |
| CAL_14_339 | 2014 | CAL | CAL_TSR | 0.8532 | 0.2870 | 15.4009 | 0.3231 | 0.3115 | 0.6229 | 0.6587 | 4.5476 | 0.5068 | 9.7381 | 0.7738 | 17.0480 | 1.0147 | 0.3810 | 0.6190 |
| CAL_14_343 | 2014 | CAL | CAL_TSR | 0.6378 | 0.2293 | 17.2333 | 0.3551 | 0.2402 | 0.4553 | 0.5614 | 5.3684 | 0.6241 | 9.2632 | 0.7263 | 17.0885 | 1.8523 | 0.6842 | 0.3158 |
| CAL_14_347 | 2014 | CAL | CAL_TSR | 0.5141 | 0.1829 | 25.9596 | 0.3749 | 0.2554 | 0.4910 | 0.7273 | 5.3043 | 0.6149 | 8.9565 | 0.6957 | 20.5711 | 1.6009 | 0.9130 | 0.0870 |
| CAL_14_351 | 2014 | CAL | CAL_TSR | 0.5676 | 0.2053 | 23.7172 | 0.3877 | 0.2485 | 0.4746 | 0.6237 | 5.3125 | 0.6161 | 9.3438 | 0.7344 | 17.8875 | 1.6318 | 0.7813 | 0.2188 |
| CAL_14_370 | 2014 | CAL | CAL_TSR | 0.6723 | 0.2336 | 20.8156 | 0.3834 | 0.2339 | 0.4403 | 0.5667 | 5.8000 | 0.6857 | 8.7000 | 0.6700 | 18.5747 | 1.9517 | 0.8000 | 0.2000 |
| CAL_14_374 | 2014 | CAL | CAL_TSR | 0.5833 | 0.2166 | 15.7197 | 0.3308 | 0.2428 | 0.4614 | 0.4530 | 5.0769 | 0.5824 | 9.1538 | 0.7154 | 15.1190 | 1.3027 | 0.3590 | 0.6410 |
| CAL_14_378 | 2014 | CAL | CAL_TSR | 0.4299 | 0.1649 | 19.0792 | 0.3764 | 0.2926 | 0.5785 | 0.5897 | 5.5000 | 0.6429 | 8.6923 | 0.6692 | 17.7148 | 2.0225 | 0.7308 | 0.2692 |
| CAL_14_382 | 2014 | CAL | CAL_TSR | 0.5121 | 0.1902 | 18.1160 | 0.3661 | 0.2723 | 0.5308 | 0.6437 | 5.2069 | 0.6010 | 8.7931 | 0.6793 | 16.5837 | 1.8161 | 0.7586 | 0.2414 |
| CAL_14_403 | 2014 | CAL | CAL_TSR | 0.3333 | 0.1308 | 14.8471 | 0.2812 | 0.2972 | 0.5894 | 0.8478 | 4.8043 | 0.5435 | 8.7609 | 0.6761 | 20.8079 | 1.9527 | 0.9783 | 0.0217 |
| CAL_14_407 | 2014 | CAL | CAL_TSR | 0.4279 | 0.1650 | 25.1570 | 0.4694 | 0.2202 | 0.4081 | 0.5763 | 5.6102 | 0.6586 | 7.9286 | 0.5929 | 28.4227 | 2.4619 | 0.9831 | 0.0169 |
| CAL_14_411 | 2014 | CAL | CAL_TSR | 0.4014 | 0.1558 | 23.1908 | 0.4408 | 0.2471 | 0.4715 | 0.5444 | 5.7000 | 0.6714 | 7.9500 | 0.5950 | 25.6643 | 2.3787 | 0.9000 | 0.1000 |
| CAL_14_415 | 2014 | CAL | CAL_TSR | 0.3625 | 0.1412 | 21.6959 | 0.4229 | 0.2864 | 0.5639 | 0.6204 | 5.2083 | 0.6012 | 8.1667 | 0.6167 | 20.2286 | 2.0899 | 0.8750 | 0.1250 |
| CAL_14_434 | 2014 | CAL | CAL_TSR | 0.3983 | 0.1560 | 23.5243 | 0.4492 | 0.2868 | 0.5648 | 0.5355 | 5.5410 | 0.6487 | 8.8689 | 0.6869 | 20.4832 | 3.1570 | 0.9836 | 0.0164 |
| CAL_14_438 | 2014 | CAL | CAL_TSR | 0.3987 | 0.1562 | 21.7129 | 0.4118 | 0.2638 | 0.5107 | 0.5632 | 5.3103 | 0.6158 | 7.2545 | 0.5255 | 20.7966 | 1.7971 | 0.7011 | 0.2989 |
| CAL_14_442 | 2014 | CAL | CAL_TSR | 0.3935 | 0.1510 | 23.6071 | 0.4424 | 0.2685 | 0.5218 | 0.6894 | 5.7955 | 0.6851 | 8.6279 | 0.6628 | 19.9164 | 2.9096 | 0.9773 | 0.0227 |
| CAL_14_446 | 2014 | CAL | CAL_TSR | 0.4879 | 0.1794 | 22.4273 | 0.4238 | 0.2601 | 0.5021 | 0.6825 | 5.5714 | 0.6531 | 8.4762 | 0.6476 | 19.4107 | 2.3265 | 0.9524 | 0.0476 |
| CAL_14_449 | 2014 | CAL | CAL_TSR | 0.5409 | 0.1947 | 21.4983 | 0.3908 | 0.2670 | 0.5181 | 0.6410 | 5.4231 | 0.6319 | 8.9231 | 0.6923 | 18.8364 | 1.9411 | 0.9615 | 0.0385 |
| CAL_14_453 | 2014 | CAL | CAL_TSR | 0.3937 | 0.1529 | 21.4715 | 0.4169 | 0.2771 | 0.5421 | 0.6437 | 5.3448 | 0.6207 | 8.6897 | 0.6690 | 18.9441 | 2.3948 | 0.7931 | 0.2069 |
| CAL_14_457 | 2014 | CAL | CAL_TSR | 0.4255 | 0.1624 | 21.2272 | 0.4117 | 0.2922 | 0.5775 | 0.5769 | 5.4615 | 0.6374 | 8.4615 | 0.6462 | 18.5250 | 2.1216 | 0.7692 | 0.2308 |
| CAL_14_461 | 2014 | CAL | CAL_TSR | 0.4124 | 0.1610 | 20.2087 | 0.3999 | 0.2758 | 0.5390 | 0.4974 | 5.5077 | 0.6440 | 8.9231 | 0.6923 | 20.0212 | 2.8915 | 0.5077 | 0.4923 |
| CAL_14_484 | 2014 | CAL | CAL_TSR | 0.4359 | 0.1699 | 16.9886 | 0.3557 | 0.2689 | 0.5227 | 0.4615 | 5.1795 | 0.5971 | 8.8974 | 0.6897 | 18.4220 | 1.6426 | 0.3333 | 0.6667 |
| CAL_14_488 | 2014 | CAL | CAL_TSR | 0.3768 | 0.1409 | 21.8606 | 0.4298 | 0.3131 | 0.6267 | 0.6882 | 5.4286 | 0.6327 | 8.4921 | 0.6492 | 18.5526 | 2.4048 | 0.9841 | 0.0159 |
| CAL_14_492 | 2014 | CAL | CAL_TSR | 0.3455 | 0.1360 | 21.4162 | 0.4184 | 0.3074 | 0.6134 | 0.6715 | 5.5507 | 0.6501 | 8.5942 | 0.6594 | 18.8316 | 2.3445 | 0.9710 | 0.0290 |
| CAL_14_496 | 2014 | CAL | CAL_TSR | 0.4975 | 0.1850 | 18.9051 | 0.3767 | 0.2970 | 0.5888 | 0.5600 | 5.2400 | 0.6057 | 8.5200 | 0.6520 | 19.5897 | 1.5534 | 0.7600 | 0.2400 |
| CAL_14_50 | 2014 | CAL | CAL_TSR | 0.5352 | 0.1926 | 25.0949 | 0.4443 | 0.2511 | 0.4809 | 0.6897 | 5.6897 | 0.6700 | 8.2586 | 0.6259 | 25.4851 | 1.7483 | 0.9828 | 0.0172 |
| CAL_14_54 | 2014 | CAL | CAL_TSR | 0.5077 | 0.1878 | 19.8608 | 0.3640 | 0.2417 | 0.4586 | 0.6167 | 5.7000 | 0.6714 | 8.2000 | 0.6200 | 21.3411 | 1.8608 | 0.7000 | 0.3000 |
| CAL_14_58 | 2014 | CAL | CAL_TSR | 0.6250 | 0.2231 | 17.5716 | 0.3279 | 0.2530 | 0.4853 | 0.6914 | 5.5185 | 0.6455 | 8.5185 | 0.6519 | 17.9528 | 2.0682 | 0.7037 | 0.2963 |
| CAL_14_62 | 2014 | CAL | CAL_TSR | 0.5925 | 0.2141 | 19.8882 | 0.3835 | 0.2843 | 0.5588 | 0.5625 | 5.8125 | 0.6875 | 8.8125 | 0.6813 | 22.2031 | 1.8355 | 0.7813 | 0.2188 |
| CAL_14_82 | 2014 | CAL | CAL_TSR | 0.7268 | 0.2295 | 47.3998 | 0.4670 | 0.1873 | 0.3305 | 0.7879 | 5.5714 | 0.6531 | 10.2857 | 0.8286 | 18.3869 | 1.2258 | 0.9643 | 0.0357 |
| CAL_14_86 | 2014 | CAL | CAL_TSR | 0.7625 | 0.2497 | 25.7762 | 0.3895 | 0.2323 | 0.4365 | 0.7576 | 5.4783 | 0.6398 | 9.6522 | 0.7652 | 18.5248 | 1.8384 | 1.0000 | 0.0000 |
| CAL_14_90 | 2014 | CAL | CAL_TSR | 0.8467 | 0.2767 | 16.7624 | 0.3341 | 0.2426 | 0.4609 | 0.7619 | 5.5714 | 0.6531 | 9.5238 | 0.7524 | 17.3893 | 1.8759 | 0.9524 | 0.0476 |
| CAL_14_94 | 2014 | CAL | CAL_TSR | 0.7761 | 0.2663 | 18.2972 | 0.3505 | 0.3217 | 0.6469 | 0.8095 | 6.3571 | 0.7653 | 9.2143 | 0.7214 | 22.6638 | 1.3845 | 0.9286 | 0.0714 |
| CAL_D04_1 | 2004 | CAL | CAL_BEFORI | 0.3194 | 0.1260 | 23.5078 | 0.4460 | 0.1918 | 0.3413 | 0.7588 | 5.1711 | 0.5959 | 6.1129 | 0.4113 | 23.9002 | 2.6692 | 1.0000 | 0.0000 |
| CAL_D04_10 | 2004 | CAL | CAL_BEFORI | 0.4301 | 0.1679 | 18.2343 | 0.3646 | 0.2495 | 0.4770 | 0.8500 | 5.7000 | 0.6714 | 6.2703 | 0.4270 | 18.5363 | 1.9062 | 0.9750 | 0.0250 |
| CAL_D04_11 | 2004 | CAL | CAL_BEFORI | 0.3562 | 0.1415 | 21.3345 | 0.4209 | 0.2303 | 0.4319 | 0.6776 | 5.4754 | 0.6393 | 8.9508 | 0.6951 | 22.5300 | 2.2889 | 0.9836 | 0.0164 |
| CAL_D04_12 | 2004 | CAL | CAL_BEFORI | 0.3160 | 0.1260 | 25.7154 | 0.4702 | 0.2034 | 0.3686 | 0.3333 | 4.8269 | 0.5467 | 5.0600 | 0.3060 | 24.4659 | 1.8971 | 1.0000 | 0.0000 |
| CAL_D04_13 | 2004 | CAL | CAL_BEFORI | 0.4054 | 0.1594 | 17.9793 | 0.3622 | 0.2441 | 0.4643 | 0.8175 | 5.6905 | 0.6701 | 6.2000 | 0.4200 | 18.5371 | 1.8472 | 0.9524 | 0.0238 |
| CAL_D04_2 | 2004 | CAL | CAL_BEFORI | 0.6297 | 0.2241 | 26.9019 | 0.4845 | 0.2040 | 0.3699 | 0.7322 | 5.8033 | 0.6862 | 6.5000 | 0.4500 | 32.3112 | 3.1924 | 0.9672 | 0.0164 |
| CAL_D04_3 | 2004 | CAL | CAL_BEFORI | 0.6096 | 0.2193 | 27.0728 | 0.4898 | 0.2054 | 0.3732 | 0.7862 | 5.6604 | 0.6658 | 6.1800 | 0.4180 | 33.9802 | 3.3664 | 0.9623 | 0.0189 |
| CAL_D04_4 | 2004 | CAL | CAL_BEFORI | 0.3278 | 0.1311 | 23.1914 | 0.4328 | 0.2304 | 0.4322 | 0.7176 | 5.4583 | 0.6369 | 5.6087 | 0.3609 | 21.0166 | 2.0409 | 1.0000 | 0.0000 |
| CAL_D04_5 | 2004 | CAL | CAL_BEFORI | 0.3185 | 0.1258 | 23.7069 | 0.4484 | 0.1923 | 0.3424 | 0.7521 | 5.1667 | 0.5952 | 6.0625 | 0.4063 | 24.3206 | 2.6547 | 1.0000 | 0.0000 |
| CAL_D04_6 | 2004 | CAL | CAL_BEFORI | 0.4650 | 0.1750 | 29.1968 | 0.5119 | 0.2177 | 0.4021 | 0.5833 | 5.2353 | 0.6050 | 6.3833 | 0.4383 | 25.6190 | 2.9075 | 1.0000 | 0.0000 |
| CAL_D04_7 | 2004 | CAL | CAL_BEFORI | 0.3690 | 0.1466 | 19.1970 | 0.3800 | 0.2393 | 0.4530 | 0.7518 | 5.4468 | 0.6353 | 5.9302 | 0.3930 | 18.3825 | 1.8676 | 0.9787 | 0.0213 |
| CAL_D04_8 | 2004 | CAL | CAL_BEFORI | 0.4221 | 0.1654 | 17.2133 | 0.3510 | 0.2558 | 0.4918 | 0.8421 | 5.8158 | 0.6880 | 6.3889 | 0.4389 | 17.5206 | 1.7160 | 0.9737 | 0.0263 |
| CAL_D04_9 | 2004 | CAL | CAL_BEFORI | 0.2607 | 0.1074 | 23.6763 | 0.4525 | 0.1902 | 0.3375 | 0.7609 | 5.0000 | 0.5714 | 5.8636 | 0.3864 | 24.0035 | 2.9050 | 1.0000 | 0.0000 |
| CAL_FM04_1 | 2004 | CAL | CAL_REF | 0.5233 | 0.1933 | 17.4126 | 0.3487 | 0.2638 | 0.5107 | 0.6993 | 5.1569 | 0.5938 | 9.1176 | 0.7118 | 18.6432 | 2.1576 | 0.9804 | 0.0196 |

| | | | | | | | | | | | | | | | | | | |
|---|---|---|---|---|---|---|---|---|---|---|---|---|---|---|---|---|---|---|
| CAL_FM04_10 | 2004 | CAL | CAL_REF | 0.5709 | 0.2109 | 18.0855 | 0.3660 | 0.2425 | 0.4606 | 0.7083 | 5.3878 | 0.6268 | 8.6250 | 0.6625 | 19.7734 | 2.2171 | 1.0000 | 0.0000 |
| CAL_FM04_11 | 2004 | CAL | CAL_REF | 0.5208 | 0.1954 | 17.4332 | 0.3509 | 0.2528 | 0.4847 | 0.8235 | 5.7843 | 0.6835 | 7.0204 | 0.5020 | 18.5441 | 1.8638 | 0.9608 | 0.0392 |
| CAL_FM04_2 | 2004 | CAL | CAL_REF | 0.4205 | 0.1592 | 14.3248 | 0.2774 | 0.2825 | 0.5547 | 0.8366 | 4.8627 | 0.5518 | 8.8039 | 0.6804 | 20.4825 | 1.6941 | 1.0000 | 0.0000 |
| CAL_FM04_3 | 2004 | CAL | CAL_REF | 0.5036 | 0.1762 | 12.7605 | 0.2499 | 0.2797 | 0.5480 | 0.7943 | 4.9362 | 0.5623 | 8.8936 | 0.6894 | 20.1883 | 1.6020 | 0.9787 | 0.0213 |
| CAL_FM04_4 | 2004 | CAL | CAL_REF | 0.4623 | 0.1763 | 18.9229 | 0.3726 | 0.2656 | 0.5150 | 0.7185 | 5.6667 | 0.6667 | 8.8000 | 0.6800 | 19.9530 | 1.5955 | 1.0000 | 0.0000 |
| CAL_FM04_5 | 2004 | CAL | CAL_REF | 0.4439 | 0.1718 | 20.6887 | 0.3904 | 0.2504 | 0.4790 | 0.8061 | 5.7091 | 0.6727 | 6.6981 | 0.4698 | 17.5112 | 1.8443 | 0.9636 | 0.0364 |
| CAL_FM04_6 | 2004 | CAL | CAL_REF | 0.4663 | 0.1781 | 21.9525 | 0.4108 | 0.2339 | 0.4404 | 0.7778 | 5.6087 | 0.6584 | 7.0667 | 0.5067 | 19.7403 | 1.8284 | 1.0000 | 0.0000 |
| CAL_FM04_7 | 2004 | CAL | CAL_REF | 0.4875 | 0.1840 | 18.9450 | 0.3808 | 0.2441 | 0.4644 | 0.4830 | 5.2245 | 0.6035 | 8.6735 | 0.6673 | 20.7150 | 2.0493 | 0.4898 | 0.5102 |
| CAL_FM04_8 | 2004 | CAL | CAL_REF | 0.6377 | 0.2276 | 16.4539 | 0.3284 | 0.2661 | 0.5162 | 0.7172 | 5.6970 | 0.6710 | 8.2727 | 0.6273 | 20.5527 | 1.8026 | 0.9697 | 0.0303 |
| CAL_FM04_9 | 2004 | CAL | CAL_REF | 0.4893 | 0.1856 | 16.4163 | 0.3289 | 0.2654 | 0.5145 | 0.7976 | 5.7143 | 0.6735 | 7.1964 | 0.5196 | 17.9318 | 1.8189 | 0.8750 | 0.1250 |
| CAL_FM14_1 | 2014 | CAL | CAL_REF | 0.4826 | 0.1846 | 19.9082 | 0.3998 | 0.2573 | 0.4955 | 0.6458 | 5.9375 | 0.7054 | 9.0000 | 0.7000 | 16.4497 | 1.5524 | 0.9688 | 0.0313 |
| CAL_FM14_2 | 2014 | CAL | CAL_REF | 0.2828 | 0.1139 | 24.7181 | 0.4535 | 0.1930 | 0.3441 | 0.6667 | 5.0000 | 0.5714 | 9.1000 | 0.7100 | 23.3222 | 3.0081 | 1.0000 | 0.0000 |
| CAL_FM14_3 | 2014 | CAL | CAL_REF | 0.7051 | 0.2428 | 16.5328 | 0.3132 | 0.2700 | 0.5254 | 0.8205 | 5.1346 | 0.5907 | 9.2308 | 0.7231 | 10.5190 | 1.0243 | 0.9808 | 0.0192 |
| CAL_FM14_4 | 2014 | CAL | CAL_REF | 0.4558 | 0.1733 | 21.6399 | 0.4103 | 0.2609 | 0.5039 | 0.6452 | 5.6452 | 0.6636 | 7.5806 | 0.5581 | 21.2549 | 1.7615 | 0.9677 | 0.0323 |
| CAL_FM14_5 | 2014 | CAL | CAL_REF | 0.7972 | 0.2713 | 17.0010 | 0.3344 | 0.2151 | 0.3962 | 0.8679 | 5.6038 | 0.6577 | 9.6226 | 0.7623 | 14.4473 | 1.6897 | 1.0000 | 0.0000 |
| CAL_FM14_6 | 2014 | CAL | CAL_REF | 0.4569 | 0.1752 | 20.2821 | 0.3937 | 0.2449 | 0.4662 | 0.6167 | 5.4000 | 0.6286 | 8.2564 | 0.6256 | 20.8944 | 2.0321 | 0.8250 | 0.1750 |
| CAL_FM14_7 | 2014 | CAL | CAL_REF | 0.4160 | 0.1596 | 21.3634 | 0.4141 | 0.2887 | 0.5694 | 0.6882 | 5.2581 | 0.6083 | 7.9355 | 0.5935 | 19.0247 | 1.9285 | 0.9839 | 0.0161 |
| CAL_FM14_8 | 2014 | CAL | CAL_REF | 0.7614 | 0.2637 | 21.8389 | 0.4088 | 0.2384 | 0.4510 | 0.8824 | 5.1176 | 0.5882 | 8.1765 | 0.6176 | 18.8209 | 1.7449 | 0.9412 | 0.0588 |
| CIE1 | 1998 | CIE | CIE_BEFORE | 0.4513 | 0.1647 | 21.1163 | 0.4119 | 0.2354 | 0.4439 | 0.8248 | 5.9065 | 0.7009 | 7.3608 | 0.5361 | 21.2372 | 1.6827 | 0.9953 | 0.0047 |
| CIE2 | 1998 | CIE | CIE_BEFORE | 0.4685 | 0.1788 | 20.4977 | 0.4004 | 0.2291 | 0.4291 | 0.6492 | 5.0729 | 0.5818 | 9.0228 | 0.7023 | 20.0120 | 2.4628 | 0.9431 | 0.0569 |
| CIE3 | 2013 | CIE | CIE_REWET | 0.3800 | 0.1496 | 20.4897 | 0.4126 | 0.2959 | 0.5861 | 0.6292 | 5.7650 | 0.6807 | 7.5547 | 0.5555 | 21.0933 | 1.4606 | 0.7006 | 0.2994 |
| CIE4 | 2013 | CIE | CIE_REWET | 0.3682 | 0.1447 | 15.6240 | 0.3085 | 0.2620 | 0.5064 | 0.7864 | 4.6841 | 0.5263 | 9.3448 | 0.7345 | 20.7475 | 2.3438 | 1.0000 | 0.0000 |
| CIE5 | 2013 | CIE | CIE_REWET | 0.2670 | 0.1104 | 12.8934 | 0.2384 | 0.2976 | 0.5903 | 0.9056 | 4.4552 | 0.4936 | 9.0860 | 0.7086 | 20.1889 | 1.9322 | 1.0000 | 0.0000 |
| DE_D3_E_85 | 1985 | DE | DE_BEFORE | 0.4582 | 0.1762 | 25.9557 | 0.4817 | 0.2696 | 0.5243 | 0.7235 | 5.1881 | 0.5983 | 5.5579 | 0.3558 | 22.5272 | 2.5009 | 0.9986 | 0.0000 |
| DE_D3_E_95 | 1995 | DE | DE_TSR | 0.5725 | 0.2098 | 21.6687 | 0.4267 | 0.3454 | 0.7028 | 0.8829 | 6.0294 | 0.7185 | 4.3703 | 0.2370 | 21.6026 | 1.3266 | 0.3293 | 0.0526 |
| DE_D3_W_85 | 1985 | DE | DE_BEFORE | 0.4514 | 0.1742 | 27.5617 | 0.4986 | 0.2541 | 0.4879 | 0.6743 | 5.0698 | 0.5814 | 6.0260 | 0.4026 | 23.0524 | 2.9409 | 0.9977 | 0.0011 |
| DE_D3_W_95 | 1995 | DE | DE_TSR | 0.4950 | 0.1833 | 20.0060 | 0.4009 | 0.3189 | 0.6404 | 0.7631 | 5.9439 | 0.7063 | 5.5321 | 0.3532 | 18.2437 | 1.3691 | 0.7951 | 0.0239 |
| DO_D10_03 | 2003 | DO | DO_TSR | 0.4242 | 0.1647 | 21.0717 | 0.4156 | 0.3027 | 0.6022 | 0.5835 | 5.6255 | 0.6608 | 8.0888 | 0.6089 | 15.0798 | 1.0505 | 0.8450 | 0.1550 |
| DO_D11_03 | 2003 | DO | DO_TSR | 0.5363 | 0.2005 | 24.0412 | 0.4448 | 0.2690 | 0.5229 | 0.8243 | 5.6262 | 0.6609 | 6.0710 | 0.4071 | 24.6414 | 2.0144 | 0.9752 | 0.0248 |
| DO_D12_03 | 2003 | DO | DO_TSR | 0.4487 | 0.1716 | 20.6973 | 0.4090 | 0.2897 | 0.5718 | 0.7398 | 6.0473 | 0.7210 | 6.0498 | 0.4050 | 18.4906 | 1.4553 | 0.9378 | 0.0155 |
| DO_D25_03 | 2003 | DO | DO_TSR | 0.4494 | 0.1727 | 19.4142 | 0.3866 | 0.2352 | 0.4433 | 0.5607 | 5.8379 | 0.6911 | 8.4220 | 0.6422 | 14.8354 | 1.2036 | 0.9592 | 0.0408 |
| DO_D26_03 | 2003 | DO | DO_TSR | 0.4322 | 0.1649 | 19.4989 | 0.3880 | 0.2677 | 0.5199 | 0.6260 | 5.6620 | 0.6660 | 8.4979 | 0.6498 | 15.4572 | 1.2278 | 0.9226 | 0.0761 |
| DO_D29_03 | 2003 | DO | DO_TSR | 0.9846 | 0.3117 | 11.5991 | 0.2296 | 0.2733 | 0.5330 | 0.9168 | 6.3388 | 0.7627 | 9.5409 | 0.7541 | 20.4151 | 1.4663 | 0.9990 | 0.0010 |
| DO_D30_03 | 2003 | DO | DO_TSR | 0.7036 | 0.2472 | 14.5520 | 0.2779 | 0.2942 | 0.5823 | 0.7349 | 5.5926 | 0.6561 | 8.2854 | 0.6285 | 20.4076 | 1.6762 | 0.6528 | 0.3472 |
| DO_D8_91 | 1991 | DO | DO_BEFORE | 0.4238 | 0.1647 | 29.4929 | 0.5008 | 0.1576 | 0.2607 | 0.0341 | 5.7904 | 0.6843 | 5.0059 | 0.3006 | 30.0534 | 2.9274 | 0.9788 | 0.0000 |
| DO_D8_92 | 1992 | DO | DO_BEFORE | 0.5147 | 0.1914 | 36.7896 | 0.5578 | 0.1346 | 0.2067 | 0.4628 | 4.5932 | 0.5133 | 6.8275 | 0.4827 | 33.4462 | 3.8102 | 0.9010 | 0.0000 |
| DO_donorHH_1 | 1993 | DO | DO_REF | 0.5106 | 0.1871 | 21.7112 | 0.4267 | 0.2494 | 0.4768 | 0.6690 | 5.7891 | 0.6842 | 6.8637 | 0.4864 | 22.3298 | 1.7283 | 0.8229 | 0.0220 |
| DO_donorHH_2 | 1993 | DO | DO_REF | 0.7555 | 0.2460 | 22.7753 | 0.4405 | 0.2454 | 0.4673 | 0.7569 | 5.7108 | 0.6730 | 6.6610 | 0.4661 | 22.2461 | 2.0524 | 0.9131 | 0.0370 |
| DO_donorLI_1 | 1993 | DO | DO_REF | 0.6055 | 0.2166 | 24.8588 | 0.4610 | 0.2429 | 0.4615 | 0.7431 | 5.5141 | 0.6449 | 8.2646 | 0.6265 | 22.2720 | 1.6959 | 0.9443 | 0.0278 |
| DO_donorLI_2 | 1993 | DO | DO_REF | 0.5805 | 0.2061 | 23.0180 | 0.4433 | 0.2402 | 0.4552 | 0.6880 | 5.7928 | 0.6847 | 7.0336 | 0.5034 | 25.3071 | 1.9245 | 0.8813 | 0.0000 |
| DO_donorZE_1 | 1993 | DO | DO_REF | 0.8269 | 0.2808 | 19.6163 | 0.3941 | 0.3182 | 0.6388 | 0.8353 | 6.0384 | 0.7198 | 8.1845 | 0.6184 | 22.4787 | 1.6865 | 0.9565 | 0.0435 |
| DO_donorZE_2 | 1993 | DO | DO_REF | 0.7112 | 0.2470 | 20.0974 | 0.3967 | 0.2824 | 0.5545 | 0.7616 | 5.3737 | 0.6248 | 8.3978 | 0.6398 | 19.3823 | 1.4706 | 0.9036 | 0.0723 |
| DO_donorZE_3 | 1993 | DO | DO_REF | 0.7447 | 0.2562 | 23.1654 | 0.4092 | 0.2589 | 0.4991 | 0.7935 | 5.7533 | 0.6790 | 7.4676 | 0.5468 | 23.2876 | 2.6351 | 0.9874 | 0.0126 |
| DO_donorZS_1 | 1993 | DO | DO_REF | 0.4212 | 0.1522 | 19.5717 | 0.3965 | 0.3006 | 0.5974 | 0.5636 | 5.5291 | 0.6470 | 5.6762 | 0.3676 | 20.0769 | 1.5913 | 0.7509 | 0.1494 |
| DO_donorZS_2 | 1993 | DO | DO_REF | 0.4722 | 0.1792 | 19.7484 | 0.3985 | 0.2689 | 0.5227 | 0.5562 | 5.9478 | 0.7068 | 5.7085 | 0.3708 | 20.1336 | 1.4856 | 0.6667 | 0.1105 |
| DO_donorZS_3 | 1993 | DO | DO_REF | 0.5408 | 0.1880 | 20.0369 | 0.4042 | 0.3048 | 0.6072 | 0.5950 | 6.2443 | 0.7492 | 7.3079 | 0.5308 | 17.6166 | 1.3777 | 0.4941 | 0.3686 |
| DO_donorZS_4 | 1993 | DO | DO_REF | 0.6914 | 0.2136 | 19.8774 | 0.3919 | 0.2375 | 0.4489 | 0.7301 | 5.2944 | 0.6135 | 6.7740 | 0.4774 | 20.5950 | 1.6840 | 0.9135 | 0.0014 |
| DO_donorZS_5 | 1993 | DO | DO_REF | 0.9709 | 0.3077 | 16.6472 | 0.3364 | 0.3153 | 0.6319 | 0.6921 | 5.0878 | 0.5840 | 9.0744 | 0.7074 | 18.4849 | 1.2015 | 0.6145 | 0.3855 |
| DO_donorZS_6 | 1993 | DO | DO_REF | 0.8147 | 0.2755 | 18.1848 | 0.3679 | 0.2449 | 0.4662 | 0.6957 | 5.3182 | 0.6169 | 8.6522 | 0.6652 | 19.5642 | 1.4339 | 0.7391 | 0.2174 |
| DO_M1_03 | 2003 | DO | DO_TSR | 0.5989 | 0.2153 | 18.8351 | 0.3860 | 0.3319 | 0.6709 | 0.7397 | 5.4825 | 0.6404 | 7.8170 | 0.5817 | 14.4413 | 0.9491 | 0.5887 | 0.3766 |
| DO_M10_03 | 2003 | DO | DO_TSR | 1.0332 | 0.3106 | 14.0424 | 0.2835 | 0.3468 | 0.7059 | 0.7452 | 5.9600 | 0.7086 | 9.2964 | 0.7296 | 19.2337 | 1.3883 | 0.9455 | 0.0545 |

| | | | | | | | | | | | | | | | | | |
|---|---|---|---|---|---|---|---|---|---|---|---|---|---|---|---|---|---|
| DO_M11_03 | 2003 | DO | DO_TSR | 0.5391 | 0.1957 | 16.1469 | 0.3162 | 0.2785 | 0.5452 | 0.7391 | 5.8862 | 0.6980 | 8.5919 | 0.6592 | 20.1042 | 1.6382 | 0.8661 | 0.1339 |
| DO_M12_03 | 2003 | DO | DO_TSR | 0.8341 | 0.2777 | 14.1972 | 0.2945 | 0.3520 | 0.7183 | 0.9141 | 6.3119 | 0.7588 | 9.4195 | 0.7419 | 22.7922 | 1.4796 | 0.9638 | 0.0362 |
| DO_M13_03 | 2003 | DO | DO_TSR | 0.9280 | 0.3004 | 14.0454 | 0.2931 | 0.3710 | 0.7629 | 0.8752 | 6.0678 | 0.7240 | 9.5338 | 0.7534 | 22.7775 | 1.4276 | 0.8843 | 0.1157 |
| DO_M14_03 | 2003 | DO | DO_TSR | 0.8660 | 0.2876 | 13.9260 | 0.2967 | 0.3707 | 0.7623 | 0.8085 | 5.7709 | 0.6816 | 9.6866 | 0.7687 | 20.5721 | 1.2055 | 0.7084 | 0.2916 |
| DO_M2_03 | 2003 | DO | DO_TSR | 0.6384 | 0.2291 | 19.4615 | 0.3939 | 0.3107 | 0.6211 | 0.7245 | 5.5392 | 0.6485 | 7.9327 | 0.5933 | 13.9085 | 0.8850 | 0.6310 | 0.3528 |
| DO_M3_03 | 2003 | DO | DO_TSR | 0.4758 | 0.1812 | 20.2744 | 0.3979 | 0.3032 | 0.6035 | 0.6602 | 6.1443 | 0.7349 | 7.4069 | 0.5407 | 15.3287 | 1.0416 | 0.5565 | 0.4222 |
| DO_M4_03 | 2003 | DO | DO_TSR | 0.4659 | 0.1783 | 17.3329 | 0.3457 | 0.3163 | 0.6344 | 0.6575 | 6.1884 | 0.7412 | 7.4290 | 0.5429 | 15.2185 | 1.0598 | 0.5137 | 0.4856 |
| DO_M5_03 | 2003 | DO | DO_TSR | 0.5125 | 0.1813 | 19.2121 | 0.3816 | 0.3206 | 0.6443 | 0.6606 | 5.9557 | 0.7080 | 7.7777 | 0.5778 | 16.5582 | 1.1105 | 0.6343 | 0.3647 |
| DO_M6_03 | 2003 | DO | DO_TSR | 0.4878 | 0.1833 | 17.8265 | 0.3631 | 0.3499 | 0.7134 | 0.6971 | 6.2753 | 0.7536 | 8.1368 | 0.6137 | 17.0995 | 1.0861 | 0.6295 | 0.3705 |
| DO_M8_03 | 2003 | DO | DO_TSR | 0.7073 | 0.2311 | 18.2763 | 0.3667 | 0.3211 | 0.6457 | 0.7723 | 6.0970 | 0.7281 | 9.0546 | 0.7055 | 20.8954 | 1.4005 | 0.9342 | 0.0658 |
| DO_M9_03 | 2003 | DO | DO_TSR | 0.6451 | 0.2244 | 15.6768 | 0.3203 | 0.3369 | 0.6828 | 0.8315 | 6.0904 | 0.7272 | 8.9535 | 0.6954 | 22.4929 | 1.4457 | 0.9640 | 0.0360 |
| DU_1 | 1995 | DU | DU_BEFORE | 0.4414 | 0.1702 | 28.5939 | 0.5078 | 0.2745 | 0.5358 | 0.6581 | 5.5897 | 0.6557 | 6.0052 | 0.4005 | 24.3335 | 1.8437 | 0.9949 | 0.0051 |
| DU_132 | 2010 | DU | DU_REWET | 0.7101 | 0.2495 | 21.6093 | 0.4208 | 0.2880 | 0.5678 | 0.8856 | 5.0392 | 0.5770 | 8.5147 | 0.6515 | 23.0505 | 2.2762 | 0.9902 | 0.0098 |
| DU_138 | 2010 | DU | DU_REWET | 0.3054 | 0.1233 | 25.0024 | 0.4641 | 0.2817 | 0.5528 | 0.6825 | 4.9429 | 0.5633 | 7.3960 | 0.5396 | 20.5590 | 1.7449 | 0.9573 | 0.0427 |
| DU_167 | 2010 | DU | DU_REWET | 0.4251 | 0.1631 | 24.5935 | 0.4571 | 0.2342 | 0.4411 | 0.7206 | 4.9915 | 0.5702 | 7.8485 | 0.5848 | 26.9770 | 2.3896 | 0.9400 | 0.0600 |
| DU_172 | 2010 | DU | DU_REWET | 0.7152 | 0.2502 | 19.3878 | 0.3857 | 0.2856 | 0.5620 | 0.8917 | 4.4000 | 0.4857 | 8.9550 | 0.6955 | 26.2801 | 1.9373 | 1.0000 | 0.0000 |
| DU_173 | 1987 | DU | DU_BEFORE | 0.4442 | 0.1668 | 24.3419 | 0.4597 | 0.2335 | 0.4395 | 0.6460 | 5.4643 | 0.6378 | 6.6259 | 0.4626 | 22.9606 | 2.8048 | 0.9788 | 0.0204 |
| DU_174 | 1987 | DU | DU_BEFORE | 0.4701 | 0.1814 | 27.8014 | 0.5013 | 0.2691 | 0.5231 | 0.6819 | 5.6192 | 0.6599 | 7.4164 | 0.5416 | 32.7789 | 2.0451 | 0.9982 | 0.0018 |
| DU_175 | 1987 | DU | DU_BEFORE | 0.3473 | 0.1376 | 21.8989 | 0.4218 | 0.2527 | 0.4847 | 0.5734 | 5.6656 | 0.6665 | 7.2102 | 0.5210 | 22.9415 | 2.4316 | 0.6977 | 0.3023 |
| DU_2 | 1997 | DU | DU_BEFORE | 0.4757 | 0.1804 | 27.0715 | 0.4911 | 0.2967 | 0.5882 | 0.6633 | 5.4020 | 0.6289 | 5.8636 | 0.3864 | 22.2397 | 1.9545 | 0.9950 | 0.0050 |
| DU_72 | 2010 | DU | DU_REWET | 0.6269 | 0.2209 | 21.2612 | 0.4162 | 0.2856 | 0.5620 | 0.8507 | 4.7645 | 0.5378 | 8.0652 | 0.6065 | 25.2505 | 2.1568 | 0.9885 | 0.0115 |
| DU_78 | 2010 | DU | DU_REWET | 0.3791 | 0.1487 | 22.3598 | 0.4264 | 0.2646 | 0.5125 | 0.6637 | 5.3632 | 0.6233 | 7.3462 | 0.5346 | 21.0150 | 1.9556 | 0.8161 | 0.1839 |
| DU_83 | 2010 | DU | DU_REWET | 0.6351 | 0.2231 | 20.0873 | 0.3989 | 0.2798 | 0.5484 | 0.8984 | 4.6476 | 0.5211 | 8.1683 | 0.6168 | 25.5418 | 2.1717 | 0.9763 | 0.0237 |
| DU_84 | 2010 | DU | DU_REWET | 0.5966 | 0.2119 | 21.3706 | 0.4162 | 0.2727 | 0.5316 | 0.8799 | 4.6306 | 0.5187 | 8.1019 | 0.6102 | 25.0650 | 2.1832 | 0.9821 | 0.0179 |
| HAV_1_2002 | 2002 | HAV | HAV_BEFOR | 0.7960 | 0.2763 | 16.7976 | 0.3276 | 0.2572 | 0.4952 | 0.8610 | 5.3149 | 0.6164 | 7.6752 | 0.5675 | 20.1513 | 1.8279 | 0.7362 | 0.2638 |
| HAV_10_2002 | 2002 | HAV | HAV_BEFOR | 0.3829 | 0.1506 | 19.9740 | 0.4058 | 0.3601 | 0.7373 | 0.6354 | 6.5714 | 0.7959 | 7.2260 | 0.5226 | 14.3932 | 0.8244 | 0.2547 | 0.7453 |
| HAV_100_2010 | 2010 | HAV | HAV_REWET | 0.5482 | 0.1998 | 22.0615 | 0.4186 | 0.3174 | 0.6369 | 0.6588 | 6.4643 | 0.7806 | 8.0714 | 0.6071 | 16.5399 | 1.0521 | 0.4118 | 0.5765 |
| HAV_101_2010 | 2010 | HAV | HAV_REWET | 1.7985 | 0.4820 | 9.7490 | 0.1584 | 0.3778 | 0.7791 | 0.6782 | 6.0455 | 0.7208 | 9.9545 | 0.7955 | 15.9193 | 1.5073 | 0.9072 | 0.0000 |
| HAV_102_2010 | 2010 | HAV | HAV_REWET | 0.4932 | 0.1792 | 21.1040 | 0.4036 | 0.3583 | 0.7331 | 0.7121 | 5.4425 | 0.6346 | 8.5089 | 0.6509 | 18.0552 | 1.1020 | 0.9153 | 0.0508 |
| HAV_103_2010 | 2010 | HAV | HAV_REWET | 0.4285 | 0.1631 | 23.5958 | 0.4447 | 0.2661 | 0.5160 | 0.7769 | 5.1529 | 0.5933 | 6.8465 | 0.4846 | 26.0330 | 2.1162 | 1.0000 | 0.0000 |
| HAV_104_2010 | 2010 | HAV | HAV_REWET | 0.3854 | 0.1529 | 23.2347 | 0.4452 | 0.2370 | 0.4475 | 0.6450 | 5.1667 | 0.5952 | 5.7662 | 0.3766 | 26.5620 | 2.6004 | 0.9675 | 0.0325 |
| HAV_105_2010 | 2010 | HAV | HAV_REWET | 0.4211 | 0.1648 | 19.1991 | 0.3845 | 0.2637 | 0.5105 | 0.5782 | 5.6377 | 0.6625 | 6.3622 | 0.4362 | 22.4206 | 2.1020 | 0.6019 | 0.3791 |
| HAV_106_2010 | 2010 | HAV | HAV_REWET | 0.3434 | 0.1385 | 24.6691 | 0.4581 | 0.2303 | 0.4318 | 0.7277 | 5.5000 | 0.6429 | 7.3376 | 0.5338 | 23.6970 | 1.6608 | 1.0000 | 0.0000 |
| HAV_107_2010 | 2010 | HAV | HAV_REWET | 0.8706 | 0.2949 | 16.3464 | 0.3495 | 0.3439 | 0.6993 | 0.9773 | 4.6515 | 0.5216 | 9.1439 | 0.7144 | 13.9344 | 0.8169 | 1.0000 | 0.0000 |
| HAV_108_2010 | 2010 | HAV | HAV_REWET | 0.8618 | 0.2929 | 17.4069 | 0.3493 | 0.3510 | 0.7159 | 0.9868 | 5.0236 | 0.5748 | 9.3622 | 0.7362 | 15.9946 | 0.9061 | 1.0000 | 0.0000 |
| HAV_11_2002 | 2002 | HAV | HAV_BEFOR | 0.4369 | 0.1704 | 19.9889 | 0.3958 | 0.2713 | 0.5283 | 0.8273 | 5.6761 | 0.6680 | 6.5547 | 0.4555 | 21.3039 | 1.4022 | 0.9838 | 0.0162 |
| HAV_12_2002 | 2002 | HAV | HAV_BEFOR | 0.3539 | 0.1412 | 23.6272 | 0.4505 | 0.2266 | 0.4232 | 0.6616 | 4.9887 | 0.5698 | 5.7213 | 0.3721 | 28.0906 | 2.3242 | 0.9962 | 0.0038 |
| HAV_13_2002 | 2002 | HAV | HAV_BEFOR | 0.4135 | 0.1615 | 18.5526 | 0.3731 | 0.2633 | 0.5096 | 0.7287 | 5.6695 | 0.6671 | 5.9780 | 0.3978 | 19.8519 | 2.0053 | 0.8057 | 0.1619 |
| HAV_14_2002 | 2002 | HAV | HAV_BEFOR | 0.4151 | 0.1630 | 21.6166 | 0.4197 | 0.2556 | 0.4915 | 0.7516 | 5.3628 | 0.6233 | 6.1210 | 0.4121 | 23.5476 | 1.8954 | 0.9717 | 0.0252 |
| HAV_15_2002 | 2002 | HAV | HAV_BEFOR | 0.5501 | 0.2026 | 20.8349 | 0.4035 | 0.2724 | 0.5310 | 0.8220 | 5.2294 | 0.6042 | 7.2079 | 0.5208 | 20.7982 | 2.0129 | 0.9319 | 0.0681 |
| HAV_16_2002 | 2002 | HAV | HAV_BEFOR | 0.9984 | 0.3232 | 14.2513 | 0.2952 | 0.2980 | 0.5912 | 0.7834 | 4.6497 | 0.5214 | 9.5606 | 0.7561 | 16.2509 | 0.7598 | 0.4904 | 0.5096 |
| HAV_17_2002 | 2002 | HAV | HAV_BEFOR | 0.3642 | 0.1434 | 25.9256 | 0.4795 | 0.2493 | 0.4765 | 0.6820 | 5.4043 | 0.6292 | 6.0868 | 0.4087 | 25.2826 | 2.2549 | 0.9833 | 0.0000 |
| HAV_18_2002 | 2002 | HAV | HAV_BEFOR | 0.5359 | 0.1976 | 17.9806 | 0.3582 | 0.2621 | 0.5068 | 0.7394 | 5.9491 | 0.7070 | 7.7778 | 0.5778 | 21.7821 | 1.4158 | 1.0000 | 0.0000 |
| HAV_19_2002 | 2002 | HAV | HAV_BEFOR | 0.6308 | 0.2279 | 20.0384 | 0.3819 | 0.2470 | 0.4712 | 0.6961 | 6.0643 | 0.7235 | 8.0769 | 0.6077 | 19.2489 | 1.4785 | 0.9679 | 0.0321 |
| HAV_2_2002 | 2002 | HAV | HAV_BEFOR | 0.3967 | 0.1567 | 18.0262 | 0.3763 | 0.3733 | 0.7685 | 0.6922 | 5.9949 | 0.7136 | 6.0625 | 0.4063 | 14.4287 | 1.1459 | 1.0000 | 0.0000 |
| HAV_20_2002 | 2002 | HAV | HAV_BEFOR | 0.9158 | 0.3072 | 16.3177 | 0.3402 | 0.2972 | 0.5893 | 0.9685 | 4.5473 | 0.5068 | 9.1014 | 0.7101 | 10.6674 | 0.6760 | 1.0000 | 0.0000 |
| HAV_21_2002 | 2002 | HAV | HAV_BEFOR | 0.3687 | 0.1457 | 29.1082 | 0.5124 | 0.2155 | 0.3970 | 0.7281 | 5.4255 | 0.6322 | 6.0772 | 0.4077 | 29.6159 | 2.4319 | 0.9716 | 0.0284 |
| HAV_22_2002 | 2002 | HAV | HAV_BEFOR | 0.4039 | 0.1573 | 28.5742 | 0.5053 | 0.2227 | 0.4139 | 0.7247 | 5.5365 | 0.6481 | 6.1107 | 0.4111 | 28.1637 | 2.3654 | 0.9715 | 0.0253 |
| HAV_23_2002 | 2002 | HAV | HAV_BEFOR | 2.7029 | 0.5094 | 20.1451 | 0.3864 | 0.2579 | 0.4969 | 0.5960 | 6.1692 | 0.7385 | 6.8167 | 0.4817 | 21.0128 | 1.6094 | 0.7099 | 0.2901 |
| HAV_24_2002 | 2002 | HAV | HAV_BEFOR | 0.7130 | 0.2473 | 20.8787 | 0.3976 | 0.3065 | 0.6113 | 0.8047 | 6.6768 | 0.8110 | 9.1313 | 0.7131 | 22.5433 | 1.7815 | 1.0000 | 0.0000 |
| HAV_25_2002 | 2002 | HAV | HAV_BEFOR | 1.7256 | 0.4637 | 9.1721 | 0.1629 | 0.3706 | 0.7622 | 0.6684 | 6.1171 | 0.7310 | 9.9171 | 0.7917 | 15.5479 | 1.4095 | 1.0000 | 0.0000 |

| | | | | | | | | | | | | | | | | | | |
|---|---|---|---|---|---|---|---|---|---|---|---|---|---|---|---|---|---|---|
| HAV_26_2002 | 2002 | HAV | HAV_BEFOR | 0.5139 | 0.1949 | 29.5651 | 0.5193 | 0.2364 | 0.4462 | 0.7022 | 5.2546 | 0.6078 | 6.5737 | 0.4574 | 28.1230 | 2.9903 | 1.0000 | 0.0000 |
| HAV_27_2002 | 2002 | HAV | HAV_BEFOR | 0.4959 | 0.1869 | 25.3539 | 0.4731 | 0.2632 | 0.5092 | 0.7488 | 5.2624 | 0.6089 | 5.5612 | 0.3561 | 28.1017 | 2.6698 | 0.9859 | 0.0070 |
| HAV_28_2002 | 2002 | HAV | HAV_BEFOR | 0.3746 | 0.1462 | 26.6243 | 0.4885 | 0.2315 | 0.4348 | 0.8025 | 5.1862 | 0.5980 | 5.2699 | 0.3270 | 32.8554 | 2.7958 | 0.9894 | 0.0053 |
| HAV_29_2002 | 2002 | HAV | HAV_BEFOR | 0.5030 | 0.1886 | 24.8052 | 0.4693 | 0.2596 | 0.5009 | 0.8137 | 5.4622 | 0.6375 | 5.1797 | 0.3180 | 26.1147 | 2.3242 | 0.9825 | 0.0000 |
| HAV_3_2002 | 2002 | HAV | HAV_BEFOR | 0.8036 | 0.2770 | 17.0164 | 0.3462 | 0.3194 | 0.6416 | 0.8283 | 4.6168 | 0.5167 | 8.1867 | 0.6187 | 14.2423 | 0.9762 | 0.7964 | 0.2036 |
| HAV_30_2002 | 2002 | HAV | HAV_BEFOR | 0.5361 | 0.1995 | 24.5711 | 0.4654 | 0.2777 | 0.5434 | 0.5793 | 5.5348 | 0.6478 | 5.4386 | 0.3439 | 21.8451 | 2.4931 | 0.9894 | 0.0053 |
| HAV_31_2002 | 2002 | HAV | HAV_BEFOR | 0.4268 | 0.1667 | 26.6637 | 0.4886 | 0.2633 | 0.5095 | 0.6920 | 5.5640 | 0.6520 | 6.0641 | 0.4064 | 28.2138 | 2.1939 | 0.9801 | 0.0159 |
| HAV_32_2002 | 2002 | HAV | HAV_BEFOR | 0.5765 | 0.2059 | 32.4408 | 0.5050 | 0.2255 | 0.4206 | 0.2763 | 6.3562 | 0.7652 | 6.8194 | 0.4819 | 28.5611 | 2.0516 | 0.6234 | 0.3377 |
| HAV_4_2002 | 2002 | HAV | HAV_BEFOR | 0.7071 | 0.2505 | 22.5005 | 0.4085 | 0.2421 | 0.4596 | 0.8320 | 5.9339 | 0.7048 | 6.3620 | 0.4362 | 31.9720 | 3.0002 | 0.7314 | 0.2686 |
| HAV_5_2002 | 2002 | HAV | HAV_BEFOR | 0.5046 | 0.1864 | 21.2206 | 0.4169 | 0.2564 | 0.4932 | 0.7719 | 5.2257 | 0.6037 | 6.6906 | 0.4691 | 22.9582 | 2.3114 | 0.8186 | 0.1814 |
| HAV_6_2002 | 2002 | HAV | HAV_BEFOR | 0.7246 | 0.2574 | 19.8116 | 0.3860 | 0.3073 | 0.6131 | 0.7506 | 6.5481 | 0.7926 | 9.1111 | 0.7111 | 24.4672 | 1.5270 | 0.8148 | 0.1852 |
| HAV_7_2002 | 2002 | HAV | HAV_BEFOR | 0.6848 | 0.2439 | 21.5242 | 0.4078 | 0.2628 | 0.5085 | 0.6667 | 6.1257 | 0.7322 | 8.8743 | 0.6874 | 23.9975 | 1.7715 | 0.7801 | 0.2199 |
| HAV_73_2010 | 2010 | HAV | HAV_REWE | 0.7755 | 0.2665 | 22.2739 | 0.4050 | 0.2444 | 0.4650 | 0.9217 | 5.1061 | 0.5866 | 8.4773 | 0.6477 | 27.1300 | 1.7262 | 0.9394 | 0.0606 |
| HAV_74_2010 | 2010 | HAV | HAV_REWE | 0.4003 | 0.1582 | 19.3948 | 0.3908 | 0.3590 | 0.7346 | 0.5894 | 6.1014 | 0.7288 | 5.0556 | 0.3056 | 14.6666 | 0.9615 | 1.0000 | 0.0000 |
| HAV_75_2010 | 2010 | HAV | HAV_REWE | 0.8793 | 0.2948 | 19.3789 | 0.3760 | 0.3115 | 0.6231 | 0.9633 | 4.0826 | 0.4404 | 9.1009 | 0.7101 | 12.2878 | 0.6520 | 1.0000 | 0.0000 |
| HAV_76_2010 | 2010 | HAV | HAV_REWE | 0.6093 | 0.2220 | 19.7853 | 0.3442 | 0.2250 | 0.4193 | 0.4157 | 6.2530 | 0.7504 | 8.1078 | 0.6108 | 16.9974 | 1.9556 | 0.6287 | 0.3653 |
| HAV_77_2010 | 2010 | HAV | HAV_REWE | 0.5092 | 0.1705 | 39.1207 | 0.5339 | 0.2394 | 0.4532 | 0.6983 | 4.5520 | 0.5074 | 10.1810 | 0.8181 | 18.5778 | 0.6093 | 1.0000 | 0.0000 |
| HAV_78_2010 | 2010 | HAV | HAV_REWE | 0.2679 | 0.1112 | 22.8173 | 0.4334 | 0.2116 | 0.3878 | 0.7810 | 5.2343 | 0.6049 | 6.3532 | 0.4353 | 25.0422 | 2.9694 | 0.9958 | 0.0042 |
| HAV_79_2010 | 2010 | HAV | HAV_REWE | 0.7854 | 0.2709 | 15.4517 | 0.2866 | 0.2497 | 0.4775 | 0.9396 | 5.1477 | 0.5925 | 8.5839 | 0.6584 | 16.1174 | 0.9561 | 1.0000 | 0.0000 |
| HAV_8_2002 | 2002 | HAV | HAV_BEFOR | 0.3624 | 0.1418 | 20.5025 | 0.4131 | 0.3478 | 0.7084 | 0.5907 | 6.4365 | 0.7766 | 7.1560 | 0.5156 | 15.4971 | 0.9963 | 0.3370 | 0.6630 |
| HAV_80_2010 | 2010 | HAV | HAV_REWE | 0.5150 | 0.1773 | 44.2669 | 0.6044 | 0.1635 | 0.2746 | 0.5568 | 5.3130 | 0.6161 | 10.7244 | 0.8724 | 30.6088 | 3.2000 | 0.8397 | 0.0000 |
| HAV_81_2010 | 2010 | HAV | HAV_REWE | 0.6914 | 0.2259 | 57.2922 | 0.7132 | 0.1529 | 0.2496 | 0.3333 | 5.9512 | 0.7073 | 11.9877 | 0.9988 | 32.9122 | NA | 0.5062 | 0.0000 |
| HAV_82_2010 | 2010 | HAV | HAV_REWE | 0.3317 | 0.1323 | 25.3210 | 0.4662 | 0.2387 | 0.4516 | 0.7993 | 5.4785 | 0.6398 | 6.6000 | 0.4600 | 30.4841 | 2.4480 | 0.8602 | 0.1398 |
| HAV_83_2010 | 2010 | HAV | HAV_REWE | 0.3930 | 0.1531 | 24.5187 | 0.4527 | 0.2535 | 0.4865 | 0.7060 | 5.5991 | 0.6570 | 6.8199 | 0.4820 | 27.4728 | 2.3242 | 0.7925 | 0.2075 |
| HAV_84_2010 | 2010 | HAV | HAV_REWE | 0.6111 | 0.2129 | 28.4052 | 0.4934 | 0.2568 | 0.4941 | 0.6158 | 6.2500 | 0.7500 | 7.5160 | 0.5516 | 25.8299 | 1.6652 | 0.8333 | 0.1667 |
| HAV_85_2010 | 2010 | HAV | HAV_REWE | 0.7287 | 0.2552 | 22.8488 | 0.4263 | 0.2659 | 0.5157 | 0.7837 | 6.7136 | 0.8162 | 8.7707 | 0.6771 | 23.1586 | 1.8362 | 0.9854 | 0.0146 |
| HAV_86_2010 | 2010 | HAV | HAV_REWE | 0.8978 | 0.2386 | 78.7033 | 0.5753 | 0.2137 | 0.3927 | 0.6370 | 5.9123 | 0.7018 | 11.0058 | 0.9006 | 24.8971 | 1.4255 | 1.0000 | 0.0000 |
| HAV_87_2010 | 2010 | HAV | HAV_REWE | 0.3028 | 0.1214 | 26.0642 | 0.4822 | 0.2314 | 0.4344 | 0.8577 | 5.4512 | 0.6359 | 6.9268 | 0.4927 | 28.9825 | 2.5310 | 1.0000 | 0.0000 |
| HAV_88_2010 | 2010 | HAV | HAV_REWE | 0.4092 | 0.1597 | 29.8553 | 0.5184 | 0.2290 | 0.4287 | 0.7000 | 6.2333 | 0.7476 | 8.1533 | 0.6153 | 23.8670 | 1.7446 | 1.0000 | 0.0000 |
| HAV_89_2010 | 2010 | HAV | HAV_REWE | 0.6257 | 0.2226 | 24.6979 | 0.4429 | 0.1927 | 0.3433 | 0.2395 | 6.8370 | 0.8339 | 9.1333 | 0.7133 | 16.5394 | 1.4572 | 1.0000 | 0.0000 |
| HAV_9_2002 | 2002 | HAV | HAV_BEFOR | 0.6267 | 0.2103 | 17.8492 | 0.3623 | 0.3709 | 0.7629 | 0.6606 | 6.6951 | 0.8136 | 7.5938 | 0.5594 | 13.8208 | 0.7394 | 0.2683 | 0.7317 |
| HAV_90_2010 | 2010 | HAV | HAV_REWE | 0.8472 | 0.2828 | 28.1597 | 0.5002 | 0.2180 | 0.4028 | 0.7824 | 5.9163 | 0.7023 | 6.9559 | 0.4956 | 31.3407 | 2.5682 | 0.9954 | 0.0000 |
| HAV_91_2010 | 2010 | HAV | HAV_REWE | 1.3309 | 0.4005 | 19.9432 | 0.4072 | 0.2606 | 0.5032 | 0.9931 | 6.0139 | 0.7163 | 7.9930 | 0.5993 | 22.5289 | 2.3157 | 1.0000 | 0.0000 |
| HAV_92_2010 | 2010 | HAV | HAV_REWE | 0.5068 | 0.1866 | 22.3150 | 0.4263 | 0.2572 | 0.4952 | 0.7814 | 5.6651 | 0.6664 | 6.7400 | 0.4740 | 24.6530 | 1.9740 | 0.8632 | 0.1368 |
| HAV_93_2010 | 2010 | HAV | HAV_REWE | 0.5259 | 0.1950 | 34.5117 | 0.5180 | 0.2390 | 0.4524 | 0.3560 | 6.0809 | 0.7258 | 6.8435 | 0.4844 | 27.4151 | 2.2715 | 0.6135 | 0.3742 |
| HAV_94_2010 | 2010 | HAV | HAV_REWE | 0.3964 | 0.1581 | 17.8919 | 0.3607 | 0.3760 | 0.7748 | 0.6603 | 6.3269 | 0.7610 | 7.3158 | 0.5316 | 14.2506 | 0.8588 | 0.0481 | 0.9519 |
| HAV_95_2010 | 2010 | HAV | HAV_REWE | 0.4678 | 0.1795 | 17.5494 | 0.3549 | 0.3637 | 0.7459 | 0.6439 | 6.4786 | 0.7827 | 7.5752 | 0.5575 | 15.8223 | 0.9680 | 0.1795 | 0.8205 |
| HAV_96_2010 | 2010 | HAV | HAV_REWE | 0.4036 | 0.1604 | 17.3573 | 0.3519 | 0.3801 | 0.7845 | 0.6667 | 6.3608 | 0.7658 | 7.3505 | 0.5351 | 13.7123 | 0.8642 | 0.0206 | 0.9794 |
| HAV_97_2010 | 2010 | HAV | HAV_REWE | 0.4614 | 0.1772 | 17.9546 | 0.3388 | 0.3619 | 0.7415 | 0.6667 | 5.8000 | 0.6857 | 8.0303 | 0.6030 | 18.0899 | 1.2040 | 0.3143 | 0.6857 |
| HAV_98_2010 | 2010 | HAV | HAV_REWE | 0.5267 | 0.1844 | 22.0318 | 0.3999 | 0.3148 | 0.6308 | 0.9517 | 6.5379 | 0.7911 | 10.2576 | 0.8258 | 29.7575 | 1.1429 | 0.7442 | 0.0233 |
| HAV_99_2010 | 2010 | HAV | HAV_REWE | 0.6675 | 0.2379 | 22.0439 | 0.4101 | 0.2657 | 0.5152 | 0.7589 | 6.5448 | 0.7921 | 9.0069 | 0.7007 | 24.9041 | 1.6813 | 0.9448 | 0.0552 |
| HO_1_04 | 2004 | HO | HO_REWET | 0.5096 | 0.1871 | 17.7811 | 0.3459 | 0.2595 | 0.5006 | 0.6479 | 5.3283 | 0.6183 | 8.8702 | 0.6870 | 19.8143 | 1.5522 | 0.9568 | 0.0432 |
| HO_1_87 | 1987 | HO | HO_BEFORE | 0.3676 | 0.1445 | 26.6253 | 0.4781 | 0.2328 | 0.4378 | 0.6839 | 5.5077 | 0.6440 | 7.0730 | 0.5073 | 23.5002 | 1.9254 | 0.9617 | 0.0383 |
| HO_3_04 | 2004 | HO | HO_REWET | 0.8252 | 0.2766 | 17.7695 | 0.3685 | 0.3018 | 0.6001 | 0.8735 | 4.6000 | 0.5143 | 8.8000 | 0.6800 | 24.6990 | 1.8964 | 1.0000 | 0.0000 |
| KB_1994_10 | 1994 | KB | KB_BEFORE | 0.3271 | 0.1299 | 25.1235 | 0.4632 | 0.2571 | 0.4950 | 0.7022 | 4.7976 | 0.5425 | 7.0466 | 0.5047 | 20.5891 | 1.8921 | 0.9589 | 0.0122 |
| KB_1994_13 | 1994 | KB | KB_BEFORE | 0.3705 | 0.1444 | 27.5288 | 0.4972 | 0.2297 | 0.4306 | 0.6395 | 4.7980 | 0.5426 | 6.0995 | 0.4099 | 26.5858 | 2.9948 | 0.9503 | 0.0000 |
| KB_1994_17 | 1994 | KB | KB_BEFORE | 0.4414 | 0.1668 | 26.2722 | 0.4808 | 0.2278 | 0.4261 | 0.6808 | 5.2277 | 0.6040 | 6.2666 | 0.4267 | 24.8534 | 2.5657 | 0.9830 | 0.0088 |
| KB_1994_18 | 1994 | KB | KB_BEFORE | 0.3536 | 0.1394 | 23.1700 | 0.4358 | 0.2609 | 0.5038 | 0.6275 | 5.2393 | 0.6056 | 6.6721 | 0.4672 | 21.0406 | 1.8023 | 0.8455 | 0.0611 |
| KB_1994_19 | 1994 | KB | KB_BEFORE | 0.3588 | 0.1403 | 26.4224 | 0.4827 | 0.2296 | 0.4303 | 0.8399 | 5.0727 | 0.5818 | 6.0155 | 0.4016 | 31.5243 | 2.6316 | 0.9975 | 0.0025 |
| KB_1994_20 | 1994 | KB | KB_BEFORE | 0.4781 | 0.1808 | 28.1362 | 0.5010 | 0.2319 | 0.4356 | 0.5670 | 4.6497 | 0.5214 | 5.8875 | 0.3887 | 26.4846 | 2.8513 | 0.9973 | 0.0000 |
| KB_1994_23 | 1994 | KB | KB_BEFORE | 0.3730 | 0.1467 | 26.3587 | 0.4840 | 0.2437 | 0.4634 | 0.6865 | 5.0641 | 0.5806 | 5.9528 | 0.3953 | 25.3644 | 2.6462 | 0.9985 | 0.0015 |

| | | | | | | | | | | | | | | | | | | |
|---|---|---|---|---|---|---|---|---|---|---|---|---|---|---|---|---|---|---|
| KB_1994_7 | 1994 | KB | KB_BEFORE | 0.3753 | 0.1479 | 23.6830 | 0.4419 | 0.2492 | 0.4763 | 0.6859 | 5.3762 | 0.6252 | 6.3224 | 0.4322 | 22.0819 | 1.9852 | 0.9643 | 0.0264 |
| KB_1994_8 | 1994 | KB | KB_BEFORE | 0.3880 | 0.1505 | 27.5802 | 0.4957 | 0.2324 | 0.4368 | 0.6969 | 4.9524 | 0.5646 | 6.5072 | 0.4507 | 24.2422 | 2.7176 | 0.9886 | 0.0077 |
| KB_1997_7a | 1997 | KB | KB_BEFORE | 0.3016 | 0.1230 | 32.3537 | 0.5389 | 0.2010 | 0.3629 | 0.5824 | 4.2075 | 0.4582 | 7.2987 | 0.5299 | 23.9568 | 1.9008 | 0.9896 | 0.0000 |
| KB_1997_8a | 1997 | KB | KB_BEFORE | 0.4472 | 0.1694 | 34.5494 | 0.5606 | 0.1798 | 0.3130 | 0.3704 | 4.7886 | 0.5412 | 6.2073 | 0.4207 | 28.6296 | 3.4586 | 0.9899 | 0.0000 |
| KB_2008_10 | 2008 | KB | KB_REWET | 0.2979 | 0.1219 | 23.6868 | 0.4501 | 0.2812 | 0.5517 | 0.6686 | 5.1274 | 0.5896 | 7.2041 | 0.5204 | 20.4618 | 1.9346 | 1.0000 | 0.0000 |
| KB_2008_13 | 2008 | KB | KB_REWET | 0.2934 | 0.1208 | 27.9393 | 0.5014 | 0.2330 | 0.4381 | 0.6719 | 5.2291 | 0.6042 | 6.1301 | 0.4130 | 23.6229 | 2.2463 | 1.0000 | 0.0000 |
| KB_2008_17 | 2008 | KB | KB_REWET | 0.4152 | 0.1592 | 22.4570 | 0.4019 | 0.2435 | 0.4630 | 0.6587 | 5.1025 | 0.5861 | 5.9331 | 0.3933 | 24.1951 | 1.8992 | 0.6564 | 0.1714 |
| KB_2008_18 | 2008 | KB | KB_REWET | 0.3939 | 0.1507 | 19.9163 | 0.3830 | 0.2971 | 0.5890 | 0.5584 | 5.2704 | 0.6101 | 7.5302 | 0.5530 | 17.9697 | 1.4718 | 0.7789 | 0.2211 |
| KB_2008_19 | 2008 | KB | KB_REWET | 0.5215 | 0.1934 | 21.8293 | 0.4160 | 0.2256 | 0.4209 | 0.8118 | 5.3646 | 0.6235 | 6.7137 | 0.4714 | 24.7913 | 2.0935 | 0.9903 | 0.0081 |
| KB_2008_20 | 2008 | KB | KB_REWET | 0.2509 | 0.1050 | 27.6726 | 0.4999 | 0.2458 | 0.4682 | 0.7448 | 4.5389 | 0.5056 | 6.7995 | 0.4800 | 21.3865 | 2.0978 | 0.9972 | 0.0028 |
| KB_2008_23 | 2008 | KB | KB_REWET | 0.3443 | 0.1390 | 24.8922 | 0.4627 | 0.2400 | 0.4547 | 0.6858 | 5.3885 | 0.6269 | 6.7500 | 0.4750 | 19.6179 | 2.0122 | 0.9956 | 0.0044 |
| KB_2008_7 | 2008 | KB | KB_REWET | 0.4089 | 0.1613 | 21.4570 | 0.4050 | 0.2308 | 0.4330 | 0.8263 | 5.8831 | 0.6976 | 6.8224 | 0.4822 | 17.7064 | 2.0527 | 0.9973 | 0.0027 |
| KB_2008_7a | 2008 | KB | KB_REWET | 0.3208 | 0.1293 | 20.2849 | 0.4076 | 0.3031 | 0.6031 | 0.6405 | 5.1275 | 0.5896 | 7.2093 | 0.5209 | 20.4159 | 1.9566 | 0.9177 | 0.0823 |
| KB_2008_8 | 2008 | KB | KB_REWET | 0.4600 | 0.1752 | 24.1818 | 0.4372 | 0.2572 | 0.4951 | 0.6271 | 5.0633 | 0.5805 | 5.9853 | 0.3985 | 22.5642 | 2.4419 | 0.8682 | 0.1318 |
| KB_2008_8a | 2008 | KB | KB_REWET | 0.4842 | 0.1792 | 23.5475 | 0.4284 | 0.2451 | 0.4667 | 0.7614 | 5.3644 | 0.6235 | 6.7021 | 0.4702 | 25.8826 | 2.0452 | 0.9505 | 0.0450 |
| KO_A1_12 | 2001 | KO | KO_TSR | 0.2272 | 0.0910 | 20.9214 | 0.4016 | 0.2714 | 0.5286 | 0.7330 | 6.3232 | 0.7605 | 8.7997 | 0.6800 | 18.2674 | 1.6475 | 0.8664 | 0.1323 |
| KO_A2_13 | 1991 | KO | KO_BEFORE | 0.3738 | 0.1477 | 18.6218 | 0.3857 | 0.3518 | 0.7179 | 0.6552 | 6.1671 | 0.7382 | 7.3758 | 0.5376 | 14.2621 | 0.9955 | 0.4849 | 0.5151 |
| KO_A3_23 | 2001 | KO | KO_TSR | 0.2614 | 0.1042 | 21.5894 | 0.4187 | 0.3269 | 0.6593 | 0.7182 | 5.5760 | 0.6537 | 8.5903 | 0.6590 | 18.0490 | 1.3987 | 0.9988 | 0.0012 |
| KO_A4_24 | 1991 | KO | KO_BEFORE | 0.3453 | 0.1378 | 18.6689 | 0.3870 | 0.3575 | 0.7313 | 0.6667 | 5.9956 | 0.7137 | 7.5062 | 0.5506 | 14.4018 | 1.0851 | 0.5573 | 0.4427 |
| KO_B1_35 | 2001 | KO | KO_TSR | 0.4002 | 0.1481 | 18.2725 | 0.3716 | 0.3424 | 0.6956 | 0.8667 | 5.9018 | 0.7003 | 9.2957 | 0.7296 | 20.0458 | 1.5166 | 0.9987 | 0.0000 |
| KO_B2_36 | 1991 | KO | KO_BEFORE | 0.4115 | 0.1607 | 17.3146 | 0.3606 | 0.3123 | 0.6248 | 0.6338 | 5.9944 | 0.7135 | 7.5665 | 0.5567 | 17.8312 | 0.8949 | 0.6241 | 0.3759 |
| KO_B3_47 | 2001 | KO | KO_TSR | 0.2858 | 0.1148 | 23.0289 | 0.4324 | 0.3098 | 0.6189 | 0.6662 | 5.3679 | 0.6240 | 8.4249 | 0.6425 | 18.1343 | 1.4982 | 0.9270 | 0.0730 |
| KO_B4_48 | 1991 | KO | KO_BEFORE | 0.4444 | 0.1705 | 18.4438 | 0.3669 | 0.2753 | 0.5377 | 0.6961 | 5.7208 | 0.6744 | 7.8030 | 0.5803 | 18.5376 | 1.4466 | 0.8198 | 0.1802 |
| KO_C_1 | 1991 | KO | KO_REF | 0.2763 | 0.1110 | 23.2070 | 0.4358 | 0.2770 | 0.5417 | 0.6762 | 5.1514 | 0.5931 | 7.5936 | 0.5594 | 19.5693 | 2.0229 | 0.9985 | 0.0000 |
| KO_C_2 | 1991 | KO | KO_REF | 0.2894 | 0.1181 | 21.1483 | 0.4111 | 0.2746 | 0.5361 | 0.7253 | 5.4067 | 0.6295 | 7.1989 | 0.5199 | 20.4701 | 2.3175 | 0.9974 | 0.0013 |
| KO_C_3 | 1991 | KO | KO_REF | 0.2733 | 0.1118 | 21.8662 | 0.4240 | 0.2579 | 0.4969 | 0.7075 | 5.4635 | 0.6376 | 7.3650 | 0.5365 | 20.3924 | 2.2602 | 0.9978 | 0.0011 |
| KO_C_4 | 2001 | KO | KO_REF | 0.2564 | 0.1067 | 23.6760 | 0.4474 | 0.2656 | 0.5149 | 0.6388 | 5.2976 | 0.6139 | 7.3448 | 0.5345 | 20.4624 | 2.2124 | 0.9990 | 0.0010 |
| KO_C_5 | 2001 | KO | KO_REF | 0.2696 | 0.1115 | 21.7185 | 0.4250 | 0.2933 | 0.5802 | 0.6573 | 5.4797 | 0.6400 | 8.0186 | 0.6019 | 17.9889 | 1.3742 | 0.9480 | 0.0520 |
| KO_C_6 | 2001 | KO | KO_REF | 0.2763 | 0.1141 | 21.1054 | 0.4171 | 0.3000 | 0.5959 | 0.6755 | 5.5033 | 0.6433 | 7.8686 | 0.5869 | 17.8493 | 1.3482 | 0.9451 | 0.0549 |
| KR_900001 | 1998 | OUD | OUD_TSR | 0.3701 | 0.1412 | 20.7158 | 0.3875 | 0.2254 | 0.4203 | 0.7855 | 5.2706 | 0.6101 | 7.3252 | 0.5325 | 23.2130 | 2.6795 | 0.5549 | 0.0862 |
| KR_900006 | 1996 | PBG | PBG_TSR | 0.3078 | 0.1195 | 23.5020 | 0.4287 | 0.2167 | 0.3999 | 0.7109 | 4.9178 | 0.5597 | 7.1693 | 0.5169 | 25.8487 | 2.8049 | 0.8858 | 0.1142 |
| KR_900013 | 1997 | PBG | PBG_TSR | 0.3544 | 0.1361 | 19.9586 | 0.3670 | 0.2127 | 0.3905 | 0.6019 | 5.2816 | 0.6117 | 7.3441 | 0.5344 | 25.1755 | 2.4742 | 0.8382 | 0.1618 |
| KR_900019 | 1996 | PBG | PBG_TSR | 0.4151 | 0.1576 | 23.9062 | 0.4236 | 0.2067 | 0.3762 | 0.3195 | 5.5069 | 0.6438 | 7.6252 | 0.5625 | 22.0883 | 2.5517 | 0.8453 | 0.1531 |
| KR_900026 | 1997 | PBG | PBG_TSR | 0.3666 | 0.1413 | 16.2801 | 0.2852 | 0.2214 | 0.4110 | 0.5165 | 5.9115 | 0.7016 | 8.5801 | 0.6580 | 17.9455 | 1.6901 | 0.8473 | 0.1512 |
| KR_900032 | 1997 | PBG | PBG_TSR | 0.5020 | 0.1841 | 31.0740 | 0.4986 | 0.1588 | 0.2636 | 0.3761 | 5.6767 | 0.6681 | 9.4659 | 0.7466 | 25.2763 | 2.1583 | 0.9604 | 0.0396 |
| KR_900038 | 1997 | PBG | PBG_TSR | 0.3014 | 0.1165 | 20.4606 | 0.3760 | 0.2117 | 0.3880 | 0.7672 | 5.2329 | 0.6047 | 6.9024 | 0.4902 | 27.3720 | 2.5614 | 0.8488 | 0.1512 |
| KR_900048 | 1994 | PBN | PBN_TSR | 0.4635 | 0.1718 | 27.7159 | 0.4873 | 0.2105 | 0.3853 | 0.5835 | 5.8175 | 0.6882 | 7.2689 | 0.5269 | 26.9693 | 2.5300 | 0.9841 | 0.0153 |
| KR_900057 | 1994 | PBN | PBN_TSR | 0.3179 | 0.1233 | 28.9895 | 0.4420 | 0.2002 | 0.3611 | 0.4519 | 5.9492 | 0.7070 | 9.3732 | 0.7373 | 22.4111 | 1.7062 | 0.8740 | 0.0431 |
| KR_900070 | 1994 | PBN | PBN_TSR | 0.3834 | 0.1466 | 26.6010 | 0.4748 | 0.2537 | 0.4870 | 0.7351 | 5.6571 | 0.6653 | 7.8552 | 0.5855 | 26.0365 | 2.4469 | 0.9547 | 0.0444 |
| KR_900079 | 1994 | PBN | PBN_TSR | 0.5075 | 0.1903 | 24.3698 | 0.4341 | 0.3188 | 0.6402 | 0.7001 | 5.9592 | 0.7085 | 8.7085 | 0.6708 | 24.7509 | 2.0151 | 0.9216 | 0.0784 |
| KR_900104 | 1998 | PMB | PMB_TSR | 0.5949 | 0.2137 | 13.3304 | 0.2245 | 0.2481 | 0.4738 | 0.6750 | 5.4978 | 0.6425 | 7.5344 | 0.5534 | 22.7565 | 1.9948 | 0.5400 | 0.4600 |
| KR_900106 | 1999 | PMB | PMB_TSR | 0.4822 | 0.1751 | 15.8106 | 0.2732 | 0.2304 | 0.4321 | 0.8143 | 5.1049 | 0.5864 | 6.9441 | 0.4944 | 24.8362 | 2.9849 | 0.5650 | 0.4350 |
| KR_900109 | 1999 | PMB | PMB_TSR | 0.5931 | 0.2110 | 13.4100 | 0.2191 | 0.2521 | 0.4832 | 0.7601 | 5.1600 | 0.5943 | 7.0279 | 0.5028 | 23.4092 | 2.8211 | 0.4228 | 0.5772 |
| KR_900112 | 1997 | COM | COM_TSR | 0.3549 | 0.1383 | 24.0757 | 0.4359 | 0.2341 | 0.4407 | 0.7662 | 5.5486 | 0.6498 | 7.0261 | 0.5026 | 23.8756 | 2.3684 | 0.8143 | 0.0929 |
| KR_900118 | 1997 | COM | COM_TSR | 0.3412 | 0.1331 | 21.2120 | 0.3925 | 0.2368 | 0.4473 | 0.6567 | 5.6086 | 0.6584 | 6.8036 | 0.4804 | 24.4012 | 2.1133 | 0.8990 | 0.1000 |
| KR_900124 | 1997 | COM | COM_TSR | 0.2711 | 0.1101 | 26.0310 | 0.4678 | 0.2144 | 0.3944 | 0.8550 | 5.5176 | 0.6454 | 6.9906 | 0.4991 | 26.2947 | 2.6838 | 0.9472 | 0.0518 |
| KR_900130 | 1997 | COM | COM_TSR | 0.5587 | 0.1917 | 13.7971 | 0.2640 | 0.2416 | 0.4584 | 0.7410 | 6.0556 | 0.7222 | 8.3506 | 0.6351 | 17.3198 | 1.8738 | 0.7403 | 0.2584 |
| KR_900136 | 1998 | PVB | PVB_TSR | 0.2625 | 0.1068 | 15.3517 | 0.3172 | 0.2273 | 0.4248 | 0.6332 | 5.6604 | 0.6658 | 7.9584 | 0.5958 | 21.6918 | 2.0186 | 0.9987 | 0.0013 |
| KR_900140 | 1998 | PVB | PVB_TSR | 0.3373 | 0.1275 | 16.9771 | 0.3252 | 0.2564 | 0.4932 | 0.7550 | 6.3575 | 0.7654 | 8.4329 | 0.6433 | 19.1547 | 1.7440 | 0.8062 | 0.1929 |
| KR_900144 | 1998 | PVB | PVB_TSR | 0.4973 | 0.1797 | 16.9423 | 0.3099 | 0.2708 | 0.5273 | 0.7398 | 6.0122 | 0.7160 | 8.0218 | 0.6022 | 22.9536 | 1.7315 | 0.7394 | 0.2606 |
| KR_900190 | 2007 | PBG | PBG_TSR | 0.3499 | 0.1368 | 21.6086 | 0.4129 | 0.3027 | 0.6022 | 0.6877 | 5.2420 | 0.6060 | 8.2875 | 0.6288 | 19.4756 | 1.4804 | 0.9605 | 0.0395 |

| | | | | | | | | | | | | | | | | | | |
|---|---|---|---|---|---|---|---|---|---|---|---|---|---|---|---|---|---|---|
| KR_900192 | 2007 | PBG | PBG_TSR | 0.3648 | 0.1385 | 22.1244 | 0.4176 | 0.2963 | 0.5871 | 0.7403 | 5.8106 | 0.6872 | 8.6529 | 0.6653 | 19.2047 | 1.0532 | 0.9612 | 0.0388 |
| KR_900193 | 2007 | PBG | PBG_TSR | 0.4453 | 0.1620 | 20.2177 | 0.3889 | 0.2998 | 0.5954 | 0.9236 | 5.7613 | 0.6802 | 9.4738 | 0.7474 | 21.7194 | 1.1789 | 0.9411 | 0.0023 |
| KR_900194 | 2007 | PBG | PBG_TSR | 0.3509 | 0.1292 | 18.9381 | 0.3748 | 0.2931 | 0.5795 | 0.9460 | 5.9660 | 0.7094 | 9.6122 | 0.7612 | 19.0589 | 1.2097 | 0.9516 | 0.0000 |
| KR_900204 | 2007 | PVB | PVB_TSR | 0.6099 | 0.2192 | 20.2667 | 0.3831 | 0.2855 | 0.5618 | 0.7773 | 6.3118 | 0.7588 | 8.3522 | 0.6352 | 23.0256 | 1.4970 | 0.9293 | 0.0707 |
| KR_900218 | 2008 | COM | COM_TSR | 0.4449 | 0.1710 | 22.6130 | 0.4134 | 0.2309 | 0.4333 | 0.6619 | 5.4571 | 0.6367 | 7.2744 | 0.5274 | 25.1896 | 2.0856 | 0.8579 | 0.1421 |
| KR_900219 | 2008 | COM | COM_TSR | 0.5142 | 0.1894 | 19.9261 | 0.3953 | 0.3639 | 0.7462 | 0.7943 | 5.8289 | 0.6898 | 8.7277 | 0.6728 | 21.3433 | 1.2123 | 0.9984 | 0.0016 |
| KR_900220 | 2008 | COM | COM_TSR | 0.6177 | 0.2182 | 18.4517 | 0.3648 | 0.2979 | 0.5909 | 0.7821 | 6.3132 | 0.7590 | 9.0074 | 0.7007 | 23.5620 | 1.6036 | 0.9448 | 0.0552 |
| KR_900225 | 2010 | OUD | OUD_TSR | 0.4699 | 0.1730 | 21.5550 | 0.3900 | 0.2364 | 0.4462 | 0.7945 | 5.2057 | 0.6008 | 7.2941 | 0.5294 | 24.9327 | 2.4342 | 0.8379 | 0.1621 |
| KR_900226 | 2011 | PBN | PBN_TSR | 0.3496 | 0.1377 | 23.4255 | 0.4314 | 0.2367 | 0.4468 | 0.7511 | 4.9528 | 0.5647 | 7.1085 | 0.5109 | 22.6474 | 1.8295 | 0.9273 | 0.0727 |
| KR_900227 | 2011 | PMB | PMB_TSR | 0.6950 | 0.2453 | 16.7869 | 0.3051 | 0.2737 | 0.5341 | 0.7085 | 6.0600 | 0.7229 | 8.1015 | 0.6102 | 22.5095 | 1.8963 | 0.7400 | 0.2600 |
| KR_900228 | 2011 | COM | COM_TSR | 0.6143 | 0.2159 | 18.1306 | 0.3647 | 0.3162 | 0.6340 | 0.7674 | 6.6781 | 0.8112 | 9.1656 | 0.7166 | 21.6131 | 1.6711 | 0.9425 | 0.0575 |
| KR_900230 | 2012 | PBG | PBG_TSR | 0.4277 | 0.1606 | 20.7892 | 0.3883 | 0.2927 | 0.5787 | 0.7746 | 5.3389 | 0.6198 | 8.6804 | 0.6680 | 20.3627 | 1.4007 | 0.9455 | 0.0545 |
| KR_900231 | 2012 | PBG | PBG_TSR | 0.3774 | 0.1446 | 24.0885 | 0.4292 | 0.2326 | 0.4374 | 0.7059 | 5.0601 | 0.5800 | 8.6230 | 0.6623 | 20.2947 | 1.4859 | 0.9364 | 0.0636 |
| KR_900232 | 2012 | PBG | PBG_TSR | 0.3651 | 0.1395 | 22.6777 | 0.4304 | 0.2636 | 0.5101 | 0.7591 | 5.3646 | 0.6235 | 8.7819 | 0.6782 | 21.4314 | 1.5295 | 0.9990 | 0.0010 |
| KR_900233 | 2012 | PBN | PBN_TSR | 0.4151 | 0.1596 | 19.9650 | 0.3654 | 0.2315 | 0.4348 | 0.6566 | 5.2839 | 0.6120 | 7.7956 | 0.5796 | 21.7096 | 1.8774 | 0.8809 | 0.1191 |
| KR_900234 | 2012 | PBN | PBN_TSR | 0.4521 | 0.1713 | 20.4475 | 0.3773 | 0.2237 | 0.4163 | 0.7470 | 5.5614 | 0.6516 | 7.3621 | 0.5362 | 24.3721 | 1.9971 | 0.9299 | 0.0701 |
| KR_900235 | 2012 | PBN | PBN_TSR | 0.3978 | 0.1498 | 17.4070 | 0.3549 | 0.2369 | 0.4474 | 0.7444 | 5.5505 | 0.6501 | 9.3158 | 0.7316 | 18.4631 | 2.1106 | 0.9631 | 0.0369 |
| KR_900236 | 2012 | PBN | PBN_TSR | 0.2619 | 0.1058 | 23.3413 | 0.4427 | 0.2607 | 0.5033 | 0.6854 | 5.3150 | 0.6164 | 8.5021 | 0.6502 | 19.5027 | 1.1689 | 0.9798 | 0.0202 |
| KR_900237 | 2012 | PMB | PMB_TSR | 0.5266 | 0.1895 | 24.7911 | 0.4601 | 0.2579 | 0.4969 | 0.6660 | 5.4880 | 0.6411 | 8.1366 | 0.6137 | 23.4485 | 2.8115 | 0.9626 | 0.0374 |
| KR_900238 | 2012 | PMB | PMB_TSR | 0.3058 | 0.1224 | 23.1826 | 0.4409 | 0.2938 | 0.5812 | 0.7057 | 5.2958 | 0.6137 | 8.0482 | 0.6048 | 19.7822 | 1.8492 | 0.9549 | 0.0451 |
| KR_900239 | 2013 | PBN | PBN_TSR | 0.3056 | 0.1168 | 21.5829 | 0.3989 | 0.2332 | 0.4388 | 0.6647 | 5.3171 | 0.6167 | 8.4284 | 0.6428 | 19.7674 | 1.5353 | 0.8675 | 0.1325 |
| KR_900240 | 2013 | PBN | PBN_TSR | 0.2998 | 0.1201 | 21.3900 | 0.4060 | 0.3122 | 0.6246 | 0.6852 | 5.0075 | 0.5725 | 8.2910 | 0.6291 | 18.5373 | 1.3036 | 0.9520 | 0.0480 |
| KR_900241 | 2013 | PBN | PBN_TSR | 0.2758 | 0.1111 | 21.2556 | 0.4147 | 0.3106 | 0.6208 | 0.6976 | 5.1487 | 0.5927 | 8.3529 | 0.6353 | 17.5050 | 1.3974 | 0.9747 | 0.0253 |
| KR_900272 | 2011 | PBN | PBN_TSR | 0.3994 | 0.1558 | 22.3770 | 0.4220 | 0.2492 | 0.4762 | 0.7392 | 5.0232 | 0.5747 | 7.5272 | 0.5527 | 23.0823 | 1.7575 | 0.9606 | 0.0394 |
| KR_900273 | 2011 | PBN | PBN_TSR | 0.5618 | 0.2035 | 20.4863 | 0.3716 | 0.2628 | 0.5083 | 0.7122 | 5.2444 | 0.6063 | 8.0391 | 0.6039 | 22.0652 | 1.6065 | 0.8996 | 0.1004 |
| LA_1_2007 | 2007 | LA | LA_BEFORE | 0.5621 | 0.2081 | 25.9969 | 0.4810 | 0.3016 | 0.5996 | 0.7446 | 5.5458 | 0.6494 | 5.9573 | 0.3957 | 26.8891 | 2.0632 | 1.0000 | 0.0000 |
| LA_10_2007 | 2007 | LA | LA_BEFORE | 0.3933 | 0.1506 | 18.9212 | 0.3357 | 0.2195 | 0.4065 | 0.7037 | 6.1111 | 0.7302 | 8.4615 | 0.6462 | 24.3610 | 2.3568 | 0.9286 | 0.0357 |
| LA_11_2007 | 2007 | LA | LA_BEFORE | 0.4676 | 0.1767 | 26.6337 | 0.4874 | 0.2342 | 0.4410 | 0.8129 | 5.6288 | 0.6613 | 6.4508 | 0.4451 | 29.8475 | 2.6347 | 1.0000 | 0.0000 |
| LA_12_2007 | 2007 | LA | LA_BEFORE | 0.6671 | 0.2354 | 22.1857 | 0.4142 | 0.2097 | 0.3833 | 0.8705 | 6.2346 | 0.7478 | 5.9421 | 0.3942 | 20.5810 | 1.7582 | 0.9692 | 0.0308 |
| LA_13_2007 | 2007 | LA | LA_BEFORE | 0.4036 | 0.1550 | 28.0225 | 0.4984 | 0.2199 | 0.4073 | 0.7407 | 5.3345 | 0.6192 | 6.4563 | 0.4456 | 26.1379 | 2.6621 | 0.9928 | 0.0036 |
| LA_14_2007 | 2007 | LA | LA_BEFORE | 0.3283 | 0.1228 | 28.1067 | 0.4942 | 0.1857 | 0.3269 | 0.7516 | 5.5137 | 0.6448 | 7.2913 | 0.5291 | 23.3774 | 2.2770 | 0.9686 | 0.0314 |
| LA_15_2007 | 2007 | LA | LA_BEFORE | 0.4076 | 0.1562 | 18.8437 | 0.3714 | 0.2644 | 0.5121 | 0.5511 | 5.9380 | 0.7054 | 7.2161 | 0.5216 | 19.6392 | 1.7634 | 0.4891 | 0.5109 |
| LA_16_2007 | 2007 | LA | LA_BEFORE | 0.7031 | 0.2398 | 21.3582 | 0.4151 | 0.2435 | 0.4628 | 0.6402 | 6.0076 | 0.7154 | 7.0379 | 0.5038 | 19.8374 | 1.9456 | 0.6174 | 0.3826 |
| LA_19_2007 | 2007 | LA | LA_BEFORE | 0.3063 | 0.1224 | 25.9111 | 0.4757 | 0.2365 | 0.4465 | 0.7528 | 5.1723 | 0.5960 | 6.3274 | 0.4327 | 22.1235 | 2.2714 | 0.9403 | 0.0597 |
| LA_2_2007 | 2007 | LA | LA_BEFORE | 0.8291 | 0.2650 | 24.0030 | 0.4537 | 0.2842 | 0.5588 | 0.8003 | 5.7585 | 0.6798 | 7.5990 | 0.5599 | 23.2325 | 2.1273 | 0.9952 | 0.0048 |
| LA_20_2007 | 2007 | LA | LA_BEFORE | 0.4109 | 0.1609 | 20.3712 | 0.3963 | 0.2690 | 0.5229 | 0.8142 | 5.5099 | 0.6443 | 5.9048 | 0.3905 | 18.3853 | 2.1776 | 0.9368 | 0.0632 |
| LA_21_2007 | 2007 | LA | LA_BEFORE | 0.4270 | 0.1661 | 21.0172 | 0.4079 | 0.2901 | 0.5727 | 0.5744 | 5.5502 | 0.6500 | 6.5675 | 0.4567 | 21.6401 | 2.2734 | 0.5848 | 0.4152 |
| LA_22_2007 | 2007 | LA | LA_BEFORE | 0.4560 | 0.1752 | 20.9887 | 0.4046 | 0.2928 | 0.5791 | 0.5580 | 5.7236 | 0.6748 | 6.6861 | 0.4686 | 21.5963 | 1.9802 | 0.5632 | 0.4332 |
| LA_3_2007 | 2007 | LA | LA_BEFORE | 0.4346 | 0.1657 | 29.0060 | 0.5126 | 0.2044 | 0.3709 | 0.7686 | 5.9784 | 0.7112 | 6.0669 | 0.4067 | 29.4887 | 2.6818 | 0.9964 | 0.0036 |
| LA_59_2013 | 2013 | LA | LA_REWET | 0.2090 | 0.0630 | 63.7614 | 0.6952 | 0.1220 | 0.1770 | 0.4281 | 4.6919 | 0.5274 | 10.7820 | 0.8782 | 28.8508 | 1.4318 | 1.0000 | 0.0000 |
| LA_6_2007 | 2007 | LA | LA_BEFORE | 0.4121 | 0.1594 | 27.9064 | 0.5003 | 0.2237 | 0.4163 | 0.8105 | 5.8733 | 0.6962 | 6.2464 | 0.4246 | 26.4280 | 2.0041 | 0.9966 | 0.0034 |
| LA_60_2013 | 2013 | LA | LA_REWET | 0.5145 | 0.1707 | 60.3015 | 0.7141 | 0.1448 | 0.2307 | 0.4116 | 5.5157 | 0.6451 | 11.6780 | 0.9678 | 35.7746 | NA | 1.0000 | 0.0000 |
| LA_61_2013 | 2013 | LA | LA_REWET | 0.7151 | 0.2303 | 58.2391 | 0.6955 | 0.1492 | 0.2409 | 0.5622 | 5.4444 | 0.6349 | 11.5903 | 0.9590 | 37.8789 | 5.6100 | 1.0000 | 0.0000 |
| LA_62_2013 | 2013 | LA | LA_REWET | 0.8010 | 0.2622 | 61.5830 | 0.7301 | 0.1394 | 0.2179 | 0.3810 | 5.6063 | 0.6580 | 11.7874 | 0.9787 | 39.2922 | 5.6100 | 1.0000 | 0.0000 |
| LA_63_2013 | 2013 | LA | LA_REWET | 0.3558 | 0.1398 | 20.2878 | 0.3984 | 0.2852 | 0.5610 | 0.6378 | 5.9068 | 0.7010 | 7.6357 | 0.5636 | 17.2846 | 1.1686 | 0.8102 | 0.1356 |
| LA_64_2013 | 2013 | LA | LA_REWET | 0.8613 | 0.2812 | 17.2802 | 0.2828 | 0.2830 | 0.5559 | 0.8966 | 6.1264 | 0.7323 | 9.4195 | 0.7420 | 20.6177 | 1.4594 | 0.9483 | 0.0517 |
| LA_65_2013 | 2013 | LA | LA_REWET | 0.5786 | 0.2106 | 26.9363 | 0.4823 | 0.2490 | 0.4759 | 0.6695 | 5.8983 | 0.6998 | 7.5714 | 0.5571 | 28.4840 | 2.2619 | 0.9958 | 0.0042 |
| LA_66_2013 | 2013 | LA | LA_REWET | 0.8664 | 0.2697 | 20.9936 | 0.4155 | 0.2569 | 0.4945 | 0.8140 | 6.0670 | 0.7239 | 8.7220 | 0.6722 | 19.8615 | 1.8961 | 0.9643 | 0.0357 |
| LA_67_2013 | 2013 | LA | LA_REWET | 1.0007 | 0.3143 | 23.0503 | 0.4390 | 0.2248 | 0.4190 | 0.6000 | 6.3813 | 0.7688 | 8.6938 | 0.6694 | 18.7282 | 2.2060 | 0.9750 | 0.0250 |
| LA_68_2013 | 2013 | LA | LA_REWET | 1.2632 | 0.3820 | 20.5454 | 0.4086 | 0.2519 | 0.4827 | 0.9304 | 6.0087 | 0.7155 | 8.1826 | 0.6183 | 21.7303 | 2.2819 | 0.9913 | 0.0087 |
| LA_69_2013 | 2013 | LA | LA_REWET | 0.8745 | 0.2861 | 16.9371 | 0.3248 | 0.2549 | 0.4897 | 0.7579 | 6.0357 | 0.7194 | 7.9821 | 0.5982 | 19.9676 | 2.0064 | 0.7143 | 0.2857 |

| | | | | | | | | | | | | | | | | | | |
|---|---|---|---|---|---|---|---|---|---|---|---|---|---|---|---|---|---|---|
| LA_70_2013 | 2013 | LA | LA_REWET | 0.8055 | 0.2428 | 32.1454 | 0.4963 | 0.2379 | 0.4497 | 0.7121 | 5.7727 | 0.6818 | 9.3939 | 0.7394 | 27.8188 | 2.1850 | 0.9697 | 0.0303 |
| LA_71_2013 | 2013 | LA | LA_REWET | 0.4243 | 0.1641 | 23.2428 | 0.4313 | 0.2563 | 0.4930 | 0.6256 | 5.5222 | 0.6460 | 6.8866 | 0.4887 | 22.6522 | 2.3173 | 0.6847 | 0.3153 |
| LA_72_2013 | 2013 | LA | LA_REWET | 0.3250 | 0.1281 | 23.4438 | 0.4403 | 0.2212 | 0.4105 | 0.8591 | 5.2762 | 0.6109 | 6.7155 | 0.4715 | 23.3673 | 2.8022 | 0.9331 | 0.0669 |
| LA_73_2013 | 2013 | LA | LA_REWET | 0.5401 | 0.2040 | 26.6118 | 0.4796 | 0.2665 | 0.5170 | 0.6542 | 5.5270 | 0.6467 | 6.9352 | 0.4935 | 27.5177 | 2.6230 | 0.8340 | 0.1660 |
| LA_74_2013 | 2013 | LA | LA_REWET | 0.4118 | 0.1608 | 24.7918 | 0.4539 | 0.2313 | 0.4341 | 0.8811 | 6.0526 | 0.7218 | 9.0819 | 0.7082 | 32.4429 | 2.3439 | 0.9709 | 0.0291 |
| LA_9_2007 | 2007 | LA | LA_BEFORE | 0.4341 | 0.1649 | 18.5686 | 0.3628 | 0.2595 | 0.5005 | 0.7158 | 5.9545 | 0.7078 | 7.6374 | 0.5637 | 18.1688 | 1.0989 | 0.8448 | 0.1414 |
| LM_101 | 2005 | LM | LM_TSR | 0.3466 | 0.1369 | 19.7321 | 0.3784 | 0.2362 | 0.4456 | 0.6067 | 5.2549 | 0.6078 | 8.0323 | 0.6032 | 19.2429 | 1.7211 | 0.9804 | 0.0196 |
| LM_102 | 2005 | LM | LM_TSR | 0.6165 | 0.2236 | 17.5381 | 0.3469 | 0.1959 | 0.3509 | 0.7230 | 5.2817 | 0.6117 | 9.3429 | 0.7343 | 23.6090 | 2.1020 | 0.9437 | 0.0563 |
| LM_104 | 2005 | LM | LM_TSR | 0.4082 | 0.1576 | 18.9808 | 0.3723 | 0.2043 | 0.3706 | 0.4857 | 5.6667 | 0.6667 | 7.8438 | 0.5844 | 18.6921 | 1.7649 | 1.0000 | 0.0000 |
| LM_105 | 2005 | LM | LM_TSR | 0.4781 | 0.1742 | 17.4998 | 0.3410 | 0.2346 | 0.4420 | 0.7688 | 5.2258 | 0.6037 | 8.3600 | 0.6360 | 17.1277 | 1.4796 | 1.0000 | 0.0000 |
| LM_112 | 2008 | LM | LM_TSR | 0.3838 | 0.1478 | 20.5024 | 0.4007 | 0.1870 | 0.3300 | 0.6667 | 5.5636 | 0.6519 | 7.4400 | 0.5440 | 19.5647 | 1.9687 | 0.9636 | 0.0364 |
| LM_113 | 2008 | LM | LM_TSR | 0.6805 | 0.2378 | 18.6664 | 0.3663 | 0.2526 | 0.4843 | 0.7597 | 5.5057 | 0.6437 | 8.2987 | 0.6299 | 16.9236 | 1.5122 | 1.0000 | 0.0000 |
| LM_114 | 2008 | LM | LM_TSR | 0.3554 | 0.1416 | 14.3820 | 0.2860 | 0.2137 | 0.3927 | 0.7833 | 5.8250 | 0.6893 | 8.3467 | 0.6347 | 21.0965 | 1.4719 | 1.0000 | 0.0000 |
| LM_115 | 2008 | LM | LM_TSR | 0.5011 | 0.1846 | 18.0148 | 0.3584 | 0.2038 | 0.3696 | 0.6286 | 5.7500 | 0.6786 | 8.1379 | 0.6138 | 20.0171 | 1.8261 | 0.9722 | 0.0278 |
| LM_116 | 2008 | LM | LM_TSR | 0.8103 | 0.2788 | 20.2315 | 0.4074 | 0.3184 | 0.6392 | 0.7174 | 5.6954 | 0.6708 | 7.9400 | 0.5940 | 27.2933 | 2.1003 | 0.9934 | 0.0066 |
| LM_117 | 2008 | LM | LM_TSR | 0.3700 | 0.1453 | 23.9950 | 0.4385 | 0.2205 | 0.4088 | 0.6977 | 5.5116 | 0.6445 | 7.3373 | 0.5337 | 23.7752 | 2.3526 | 0.9767 | 0.0233 |
| LM_118 | 2008 | LM | LM_TSR | 0.3816 | 0.1503 | 21.2436 | 0.4203 | 0.1806 | 0.3149 | 0.5238 | 5.7821 | 0.6832 | 6.5200 | 0.4520 | 22.0452 | 2.2359 | 0.9872 | 0.0128 |
| LM_119 | 2008 | LM | LM_TSR | 0.4225 | 0.1620 | 22.6261 | 0.4312 | 0.1839 | 0.3227 | 0.5840 | 5.4915 | 0.6416 | 6.5900 | 0.4590 | 24.5100 | 2.2551 | 0.9661 | 0.0339 |
| LM_120 | 2008 | LM | LM_TSR | 0.6670 | 0.2329 | 19.5788 | 0.3942 | 0.3322 | 0.6716 | 0.6788 | 5.7182 | 0.6740 | 8.0741 | 0.6074 | 22.2363 | 1.9373 | 1.0000 | 0.0000 |
| LM_121 | 2008 | LM | LM_TSR | 0.4209 | 0.1628 | 20.9844 | 0.4056 | 0.2141 | 0.3936 | 0.6483 | 5.5182 | 0.6455 | 7.2826 | 0.5283 | 23.6116 | 2.1546 | 0.9909 | 0.0091 |
| LM_122 | 2008 | LM | LM_TSR | 0.5923 | 0.2104 | 19.7993 | 0.4012 | 0.3091 | 0.6173 | 0.7760 | 5.2800 | 0.6114 | 7.9180 | 0.5918 | 20.7002 | 1.8177 | 0.9920 | 0.0080 |
| LM_123 | 2008 | LM | LM_TSR | 0.6804 | 0.2369 | 18.4028 | 0.3549 | 0.2658 | 0.5154 | 0.7561 | 5.4553 | 0.6365 | 8.4417 | 0.6442 | 17.6685 | 1.3514 | 1.0000 | 0.0000 |
| LM_124 | 1995 | LM | LM_BEFORE | 0.3994 | 0.1553 | 28.6036 | 0.5083 | 0.2423 | 0.4601 | 0.7380 | 5.4650 | 0.6379 | 7.0426 | 0.5043 | 25.7509 | 2.6254 | 1.0000 | 0.0000 |
| LM_125 | 1995 | LM | LM_BEFORE | 0.4237 | 0.1642 | 29.2960 | 0.5162 | 0.2350 | 0.4430 | 0.7213 | 5.4310 | 0.6330 | 6.8087 | 0.4809 | 26.8577 | 2.7102 | 1.0000 | 0.0000 |
| LM_126 | 1995 | LM | LM_BEFORE | 0.5743 | 0.2086 | 25.0416 | 0.4659 | 0.2342 | 0.4409 | 0.8014 | 5.1443 | 0.5920 | 7.2541 | 0.5254 | 25.9736 | 2.5761 | 0.9948 | 0.0052 |
| LM_127 | 1995 | LM | LM_BEFORE | 0.4357 | 0.1658 | 25.1427 | 0.4624 | 0.2288 | 0.4283 | 0.7309 | 5.3472 | 0.6210 | 6.7262 | 0.4726 | 23.8200 | 2.4104 | 0.9585 | 0.0415 |
| LM_128 | 1995 | LM | LM_BEFORE | 0.3340 | 0.1328 | 26.6078 | 0.4864 | 0.2287 | 0.4280 | 0.7041 | 5.1573 | 0.5939 | 6.6308 | 0.4631 | 23.4932 | 2.3551 | 1.0000 | 0.0000 |
| LM_129 | 1995 | LM | LM_BEFORE | 0.3358 | 0.1333 | 25.6264 | 0.4684 | 0.2184 | 0.4038 | 0.7121 | 5.1909 | 0.5987 | 6.7012 | 0.4701 | 23.3606 | 2.5219 | 0.9636 | 0.0364 |
| LM_130 | 1995 | LM | LM_BEFORE | 0.3835 | 0.1505 | 28.0044 | 0.4994 | 0.2185 | 0.4040 | 0.7046 | 5.3270 | 0.6181 | 6.6125 | 0.4613 | 24.2762 | 2.4780 | 0.9953 | 0.0047 |
| LM_131 | 1995 | LM | LM_BEFORE | 0.3371 | 0.1340 | 25.1544 | 0.4613 | 0.2288 | 0.4282 | 0.7404 | 5.3526 | 0.6218 | 6.7018 | 0.4702 | 23.2445 | 2.4560 | 0.9000 | 0.1000 |
| MOS_1 | 1999 | MOS | MOS_TSR | 0.1334 | 0.0579 | 12.2242 | 0.2415 | 0.2847 | 0.5598 | 0.6650 | 5.3242 | 0.6177 | 8.9350 | 0.6935 | 16.2270 | 0.8578 | 0.9817 | 0.0183 |
| MOS_4 | 2012 | MOS | MOS_TSR | 0.2200 | 0.0897 | 20.5429 | 0.3942 | 0.2751 | 0.5373 | 0.5667 | 6.1186 | 0.7312 | 8.6667 | 0.6667 | 15.4309 | 1.0847 | 0.9672 | 0.0328 |
| NP_1 | 2007 | NP | NP_TSR | 0.9505 | 0.3011 | 14.3363 | 0.2947 | 0.3637 | 0.7458 | 0.8744 | 5.7477 | 0.6782 | 8.9907 | 0.6991 | 22.0502 | 1.1526 | 0.8224 | 0.1776 |
| NP_11 | 2007 | NP | NP_TSR | 1.0644 | 0.3250 | 14.0056 | 0.2878 | 0.3526 | 0.7197 | 0.7861 | 5.3448 | 0.6207 | 8.5586 | 0.6559 | 20.8009 | 0.9935 | 0.7379 | 0.2621 |
| NP_14 | 2007 | NP | NP_TSR | 1.1478 | 0.3447 | 13.2574 | 0.2738 | 0.3486 | 0.7104 | 0.7883 | 4.9950 | 0.5707 | 8.6634 | 0.6663 | 21.9040 | 1.0287 | 0.8119 | 0.1881 |
| NP_16 | 2007 | NP | NP_TSR | 0.9025 | 0.2869 | 14.7825 | 0.3064 | 0.3583 | 0.7330 | 0.7778 | 5.7788 | 0.6827 | 8.3846 | 0.6385 | 19.7908 | 0.9780 | 0.6346 | 0.3654 |
| NP_19 | 2007 | NP | NP_TSR | 0.9933 | 0.3087 | 14.3644 | 0.2947 | 0.3561 | 0.7280 | 0.8351 | 5.4971 | 0.6424 | 8.7368 | 0.6737 | 21.5040 | 1.0629 | 0.7778 | 0.2222 |
| NP_21 | 2007 | NP | NP_TSR | 1.0127 | 0.3116 | 14.9081 | 0.3014 | 0.3581 | 0.7327 | 0.8211 | 5.4177 | 0.6311 | 8.7848 | 0.6785 | 21.4166 | 1.0822 | 0.7848 | 0.2152 |
| NP_24 | 2007 | NP | NP_TSR | 1.0665 | 0.3248 | 14.3796 | 0.2960 | 0.3560 | 0.7276 | 0.7552 | 5.2929 | 0.6133 | 8.5786 | 0.6579 | 21.0769 | 0.9962 | 0.7286 | 0.2714 |
| NP_26 | 2007 | NP | NP_TSR | 0.8900 | 0.2865 | 13.6217 | 0.2667 | 0.3115 | 0.6229 | 0.9167 | 5.7538 | 0.6791 | 8.8154 | 0.6815 | 21.2876 | 1.2030 | 0.9231 | 0.0769 |
| NP_29 | 2007 | NP | NP_TSR | 1.0757 | 0.3309 | 13.0900 | 0.2689 | 0.3422 | 0.6951 | 0.8956 | 5.3522 | 0.6217 | 8.9057 | 0.6906 | 22.5388 | 1.1138 | 0.8931 | 0.1069 |
| NP_4 | 2007 | NP | NP_TSR | 0.9056 | 0.2872 | 15.0527 | 0.3117 | 0.3656 | 0.7504 | 0.7857 | 5.7921 | 0.6846 | 8.4109 | 0.6411 | 19.8627 | 0.9818 | 0.6238 | 0.3762 |
| NP_6 | 2007 | NP | NP_TSR | 0.7417 | 0.2437 | 16.9437 | 0.3455 | 0.3667 | 0.7528 | 0.7222 | 6.0786 | 0.7255 | 8.0217 | 0.6022 | 17.7598 | 0.9181 | 0.4571 | 0.5429 |
| NP_9 | 2007 | NP | NP_TSR | 0.9155 | 0.2893 | 15.2259 | 0.3159 | 0.3692 | 0.7586 | 0.7751 | 5.7889 | 0.6841 | 8.4171 | 0.6417 | 19.8793 | 0.9779 | 0.6181 | 0.3819 |
| OOS_1 | 2000 | OOS | OOS_REWET | 0.3460 | 0.1395 | 18.7969 | 0.3843 | 0.3854 | 0.7970 | 0.6473 | 6.4923 | 0.7846 | 7.1532 | 0.5153 | 14.0668 | 0.9735 | 0.3075 | 0.6925 |
| OOS_10 | 2000 | OOS | OOS_REWET | 0.3951 | 0.1565 | 29.0837 | 0.5100 | 0.2311 | 0.4336 | 0.6617 | 5.6782 | 0.6683 | 6.3723 | 0.4372 | 25.0082 | 1.9747 | 0.9839 | 0.0161 |
| OOS_11 | 2000 | OOS | OOS_REWET | 1.1336 | 0.3409 | 13.4985 | 0.2854 | 0.3592 | 0.7353 | 0.6993 | 5.1422 | 0.5917 | 8.3635 | 0.6363 | 20.9374 | 0.9213 | 0.6650 | 0.3350 |
| OOS_12 | 2000 | OOS | OOS_REWET | 0.4606 | 0.1752 | 18.7707 | 0.3827 | 0.3583 | 0.7331 | 0.7266 | 6.6892 | 0.8127 | 7.3568 | 0.5357 | 15.4437 | 1.1027 | 0.2810 | 0.7190 |
| OOS_13 | 2000 | OOS | OOS_REWET | 0.6534 | 0.2331 | 29.4740 | 0.4758 | 0.2047 | 0.3716 | 0.6428 | 6.4593 | 0.7799 | 8.6795 | 0.6679 | 25.1142 | 1.3457 | 0.8063 | 0.1937 |
| OOS_14 | 2000 | OOS | OOS_REWET | 0.7558 | 0.2634 | 17.9282 | 0.3598 | 0.3255 | 0.6560 | 0.8176 | 6.1903 | 0.7415 | 8.8801 | 0.6880 | 25.5420 | 1.5323 | 1.0000 | 0.0000 |
| OOS_15 | 2000 | OOS | OOS_REWET | 0.3688 | 0.1480 | 16.4788 | 0.3384 | 0.3813 | 0.7872 | 0.7328 | 6.4513 | 0.7788 | 7.4638 | 0.5464 | 14.0463 | 0.8087 | 0.2780 | 0.7220 |

| | | | | | | | | | | | | | | | | | | |
|---|---|---|---|---|---|---|---|---|---|---|---|---|---|---|---|---|---|---|
| OOS_2 | 2000 | OOS | OOS_REWET | 0.4100 | 0.1628 | 14.7299 | 0.3083 | 0.4246 | 0.8890 | 0.6667 | 6.9976 | 0.8568 | 7.5032 | 0.5503 | 11.9411 | 0.5871 | 0.5012 | 0.4988 |
| OOS_3 | 2000 | OOS | OOS_REWET | 0.5338 | 0.1965 | 13.6260 | 0.2839 | 0.3722 | 0.7659 | 0.7339 | 6.0000 | 0.7143 | 8.1996 | 0.6200 | 15.8949 | 1.1882 | 0.6008 | 0.3992 |
| OOS_32 | 2004 | OOS | OOS_REWET | 0.5192 | 0.1901 | 32.2483 | 0.5409 | 0.1845 | 0.3240 | 0.4996 | 6.3662 | 0.7666 | 8.5742 | 0.6574 | 23.0597 | 1.9124 | 1.0000 | 0.0000 |
| OOS_4 | 2000 | OOS | OOS_REWET | 0.6447 | 0.2309 | 13.3926 | 0.2559 | 0.2573 | 0.4953 | 0.7725 | 5.9605 | 0.7086 | 8.5633 | 0.6563 | 20.6329 | 1.4481 | 0.6798 | 0.3186 |
| OOS_5 | 2000 | OOS | OOS_REWET | 0.3733 | 0.1498 | 29.0125 | 0.5150 | 0.2581 | 0.4972 | 0.6673 | 5.7885 | 0.6841 | 6.9775 | 0.4978 | 20.9457 | 2.1044 | 1.0000 | 0.0000 |
| OOS_50 | 2014 | OOS | OOS_REWET | 0.3533 | 0.1425 | 19.6026 | 0.3981 | 0.3801 | 0.7844 | 0.6654 | 6.3717 | 0.7674 | 7.2917 | 0.5292 | 14.2875 | 0.7925 | 0.2906 | 0.7094 |
| OOS_51 | 2014 | OOS | OOS_REWET | 0.3640 | 0.1463 | 18.1145 | 0.3698 | 0.3939 | 0.8168 | 0.6654 | 6.4722 | 0.7817 | 7.4106 | 0.5411 | 13.8009 | 0.7501 | 0.4022 | 0.5978 |
| OOS_52 | 2014 | OOS | OOS_REWET | 0.3500 | 0.1415 | 20.0873 | 0.4078 | 0.3851 | 0.7962 | 0.6523 | 6.2446 | 0.7492 | 7.2132 | 0.5213 | 14.0572 | 0.7321 | 0.3383 | 0.6617 |
| OOS_53 | 2014 | OOS | OOS_REWET | 0.4843 | 0.1843 | 17.7129 | 0.3677 | 0.3798 | 0.7836 | 0.6667 | 6.7968 | 0.8281 | 7.4064 | 0.5406 | 13.5406 | 0.8536 | 0.2032 | 0.7968 |
| OOS_54 | 2014 | OOS | OOS_REWET | 0.4160 | 0.1643 | 17.9856 | 0.3727 | 0.3938 | 0.8167 | 0.6795 | 7.0000 | 0.8571 | 7.2333 | 0.5233 | 12.9006 | 0.6691 | 0.1667 | 0.8333 |
| OOS_55 | 2014 | OOS | OOS_REWET | 0.7584 | 0.2668 | 12.5138 | 0.2664 | 0.2969 | 0.5887 | 0.6438 | 5.4749 | 0.6393 | 8.8018 | 0.6802 | 20.3811 | 1.7789 | 0.4710 | 0.5290 |
| OOS_56 | 2014 | OOS | OOS_REWET | 0.3690 | 0.1465 | 26.0133 | 0.4656 | 0.2506 | 0.4796 | 0.6962 | 5.6413 | 0.6630 | 6.5037 | 0.4504 | 20.7997 | 1.9934 | 0.9114 | 0.0886 |
| OOS_57 | 2014 | OOS | OOS_REWET | 1.0960 | 0.3334 | 13.9120 | 0.2927 | 0.3576 | 0.7314 | 0.6963 | 5.2709 | 0.6101 | 8.4422 | 0.6442 | 21.2154 | 0.9349 | 0.6514 | 0.3486 |
| OOS_58 | 2014 | OOS | OOS_REWET | 0.5294 | 0.1932 | 19.6047 | 0.3557 | 0.2909 | 0.5746 | 0.7205 | 5.4034 | 0.6291 | 8.0432 | 0.6043 | 20.3118 | 2.2181 | 0.7024 | 0.2976 |
| OOS_59 | 2014 | OOS | OOS_REWET | 0.9073 | 0.3055 | 16.3914 | 0.3433 | 0.3220 | 0.6476 | 0.8273 | 6.1311 | 0.7330 | 8.6409 | 0.6641 | 24.0537 | 1.8777 | 0.9987 | 0.0013 |
| OOS_6 | 2000 | OOS | OOS_REWET | 1.2401 | 0.3682 | 12.6533 | 0.2684 | 0.3571 | 0.7302 | 0.7266 | 4.9785 | 0.5684 | 8.5943 | 0.6594 | 22.2152 | 0.9554 | 0.7288 | 0.2712 |
| OOS_60 | 2014 | OOS | OOS_REWET | 0.2133 | 0.0743 | 42.1691 | 0.5820 | 0.1645 | 0.2769 | 0.7333 | 5.6000 | 0.6571 | 11.3333 | 0.9333 | 23.5067 | 1.2374 | 0.3000 | 0.0000 |
| OOS_61 | 2014 | OOS | OOS_REWET | 1.0712 | 0.3360 | 21.0177 | 0.4183 | 0.2533 | 0.4860 | 0.7972 | 5.8747 | 0.6964 | 8.0268 | 0.6027 | 22.0583 | 2.4300 | 0.9991 | 0.0009 |
| OOS_62 | 2014 | OOS | OOS_REWET | 1.3418 | 0.3930 | 11.7251 | 0.2518 | 0.3537 | 0.7222 | 0.7616 | 4.6017 | 0.5145 | 8.8035 | 0.6803 | 23.4121 | 0.9902 | 0.8734 | 0.1266 |
| OOS_63 | 2014 | OOS | OOS_REWET | 0.4862 | 0.1844 | 17.1812 | 0.3494 | 0.3694 | 0.7592 | 0.7198 | 6.7345 | 0.8192 | 7.5279 | 0.5528 | 15.2503 | 1.0650 | 0.2382 | 0.7618 |
| OOS_64 | 2014 | OOS | OOS_REWET | 0.6611 | 0.2355 | 25.0968 | 0.4349 | 0.2739 | 0.5344 | 0.7528 | 6.5521 | 0.7932 | 9.1533 | 0.7153 | 25.6064 | 1.3733 | 0.5420 | 0.0637 |
| OOS_65 | 2014 | OOS | OOS_REWET | 0.7684 | 0.2653 | 18.5964 | 0.3679 | 0.2673 | 0.5190 | 0.7355 | 5.5172 | 0.6453 | 8.7854 | 0.6785 | 24.2831 | 2.4271 | 0.7955 | 0.2045 |
| OOS_7 | 2000 | OOS | OOS_REWET | 0.5437 | 0.1988 | 17.1984 | 0.3144 | 0.2828 | 0.5554 | 0.6931 | 5.8819 | 0.6974 | 8.1375 | 0.6138 | 20.1074 | 2.8034 | 0.6301 | 0.3699 |
| OOS_8 | 2000 | OOS | OOS_REWET | 0.9570 | 0.3128 | 17.3215 | 0.3542 | 0.3095 | 0.6183 | 0.8144 | 5.4654 | 0.6379 | 8.4229 | 0.6423 | 23.4043 | 2.0578 | 0.9991 | 0.0009 |
| OOS_9 | 2000 | OOS | OOS_REWET | 0.0017 | 0.0006 | 144.2485 | 0.9801 | 0.0509 | 0.0097 | 0.4641 | 5.8700 | 0.6957 | 11.9423 | 0.9942 | 33.3845 | 1.4207 | 1.0000 | 0.0000 |
| PLI_B1_14 | 2014 | PLI | PLI_REWET | 0.6527 | 0.2337 | 27.1823 | 0.4762 | 0.2395 | 0.4536 | 0.7723 | 5.8558 | 0.6937 | 8.1074 | 0.6107 | 15.7528 | 1.1929 | 1.0000 | 0.0000 |
| PLI_B1_95 | 1995 | PLI | PLI_BEFORE | 0.4300 | 0.1637 | 25.0792 | 0.4591 | 0.2319 | 0.4355 | 0.6672 | 5.8566 | 0.6938 | 7.2473 | 0.5247 | 22.3076 | 1.7467 | 0.8489 | 0.0504 |
| PLI_B2_14 | 2014 | PLI | PLI_REWET | 0.3210 | 0.1271 | 21.2224 | 0.4053 | 0.2556 | 0.4914 | 0.6724 | 5.0838 | 0.5834 | 8.6318 | 0.6632 | 20.9812 | 2.3523 | 0.9994 | 0.0006 |
| PLI_B2_95 | 1995 | PLI | PLI_BEFORE | 0.4064 | 0.1557 | 22.6381 | 0.4325 | 0.2503 | 0.4788 | 0.7166 | 5.3093 | 0.6156 | 7.7949 | 0.5795 | 20.8570 | 1.6708 | 0.9989 | 0.0011 |
| PLI_B3_14 | 2014 | PLI | PLI_REWET | 0.3773 | 0.1470 | 25.4900 | 0.4618 | 0.2137 | 0.3928 | 0.7319 | 5.4014 | 0.6288 | 7.8440 | 0.5844 | 20.0639 | 2.1918 | 0.9993 | 0.0007 |
| PLI_B3_95 | 1995 | PLI | PLI_BEFORE | 0.3632 | 0.1420 | 23.5840 | 0.4487 | 0.2784 | 0.5451 | 0.6376 | 5.3405 | 0.6201 | 7.5811 | 0.5581 | 19.9430 | 1.4619 | 0.9766 | 0.0234 |
| PLI_B4_14 | 2014 | PLI | PLI_REWET | 0.4120 | 0.1548 | 21.5473 | 0.4206 | 0.2324 | 0.4367 | 0.7628 | 5.5504 | 0.6501 | 7.6357 | 0.5636 | 20.8996 | 1.9463 | 0.9994 | 0.0006 |
| PLI_B4_95 | 1995 | PLI | PLI_BEFORE | 0.3104 | 0.1259 | 20.7839 | 0.4026 | 0.2689 | 0.5227 | 0.6745 | 5.5111 | 0.6444 | 7.9628 | 0.5963 | 17.9974 | 1.5330 | 0.9738 | 0.0252 |
| PLI_B5_14 | 2014 | PLI | PLI_REWET | 0.6086 | 0.2113 | 25.8732 | 0.4691 | 0.2188 | 0.4048 | 0.7630 | 5.7585 | 0.6798 | 8.2163 | 0.6216 | 25.3656 | 2.2159 | 0.8773 | 0.1227 |
| PLI_B5_95 | 1995 | PLI | PLI_BEFORE | 0.5698 | 0.2087 | 19.5559 | 0.3923 | 0.2754 | 0.5381 | 0.7564 | 5.4470 | 0.6353 | 8.1746 | 0.6175 | 19.6387 | 1.9972 | 0.9990 | 0.0010 |
| PLI_B6_14 | 2014 | PLI | PLI_REWET | 0.4368 | 0.1450 | 51.2130 | 0.6064 | 0.1893 | 0.3354 | 0.5977 | 4.3459 | 0.4780 | 10.0756 | 0.8076 | 22.1940 | 1.1581 | 0.9959 | 0.0041 |
| PLI_B6_95 | 1995 | PLI | PLI_BEFORE | 0.6031 | 0.2201 | 18.6409 | 0.3500 | 0.2571 | 0.4950 | 0.4883 | 5.1048 | 0.5864 | 9.2454 | 0.7245 | 24.1227 | 1.9139 | 0.3642 | 0.6358 |
| RR_1_1998 | 1998 | RR | RR_BEFORE | 0.3096 | 0.1237 | 25.0966 | 0.4711 | 0.2057 | 0.3739 | 0.5698 | 4.6919 | 0.5274 | 5.5150 | 0.3515 | 23.9012 | 2.8156 | 0.9651 | 0.0349 |
| RR_10_1998 | 1998 | RR | RR_BEFORE | 0.2694 | 0.1089 | 24.9917 | 0.4692 | 0.2110 | 0.3863 | 0.8854 | 5.0402 | 0.5772 | 6.7883 | 0.4788 | 24.3320 | 3.1469 | 0.9911 | 0.0089 |
| RR_100_1998 | 1998 | RR | RR_BEFORE | 0.5554 | 0.2020 | 24.6068 | 0.4664 | 0.2787 | 0.5457 | 0.7961 | 5.3981 | 0.6283 | 5.7329 | 0.3733 | 22.3863 | 2.2493 | 1.0000 | 0.0000 |
| RR_117_1998 | 1998 | RR | RR_BEFORE | 0.8394 | 0.2794 | 23.4969 | 0.4378 | 0.2550 | 0.4900 | 0.5293 | 6.2000 | 0.7429 | 6.9070 | 0.4907 | 27.6479 | 2.6705 | 0.6718 | 0.3206 |
| RR_124_1998 | 1998 | RR | RR_BEFORE | 0.5691 | 0.2017 | 25.9695 | 0.4774 | 0.2582 | 0.4974 | 0.7855 | 5.6294 | 0.6613 | 6.3115 | 0.4311 | 22.8011 | 2.0274 | 0.9930 | 0.0070 |
| RR_133_1998 | 1998 | RR | RR_BEFORE | 0.7419 | 0.2426 | 26.5935 | 0.4831 | 0.2451 | 0.4666 | 0.9953 | 5.9860 | 0.7123 | 7.9930 | 0.5993 | 22.5019 | 2.3339 | 1.0000 | 0.0000 |
| RR_142_1998 | 1998 | RR | RR_BEFORE | 0.3455 | 0.1361 | 27.8347 | 0.4965 | 0.2244 | 0.4180 | 0.8893 | 5.6183 | 0.6598 | 7.3167 | 0.5317 | 23.9131 | 3.1202 | 0.9834 | 0.0166 |
| RR_151_1998 | 1998 | RR | RR_BEFORE | 0.4099 | 0.1575 | 27.1584 | 0.4923 | 0.2337 | 0.4399 | 0.8321 | 5.2692 | 0.6099 | 6.8248 | 0.4825 | 25.4099 | 3.0068 | 0.9962 | 0.0038 |
| RR_160_1998 | 1998 | RR | RR_BEFORE | 0.4849 | 0.1814 | 24.7926 | 0.4680 | 0.2372 | 0.4481 | 0.9187 | 5.5072 | 0.6439 | 6.9633 | 0.4963 | 27.8889 | 2.4872 | 1.0000 | 0.0000 |
| RR_169_1998 | 1998 | RR | RR_BEFORE | 0.5207 | 0.1863 | 24.4063 | 0.4589 | 0.2203 | 0.4083 | 0.7886 | 6.4878 | 0.7840 | 7.8943 | 0.5894 | 26.8526 | 2.2231 | 0.9837 | 0.0163 |
| RR_177_1998 | 1998 | RR | RR_BEFORE | 0.7943 | 0.2757 | 15.8150 | 0.3322 | 0.3811 | 0.7868 | 0.9243 | 6.6432 | 0.8062 | 9.6595 | 0.7659 | 23.8573 | 1.6081 | 0.8919 | 0.1081 |
| RR_185_1998 | 1998 | RR | RR_BEFORE | 1.5364 | 0.4356 | 18.2170 | 0.3675 | 0.2833 | 0.5566 | 0.9608 | 5.9559 | 0.7080 | 9.7794 | 0.7779 | 18.1573 | 2.1661 | 0.9412 | 0.0588 |
| RR_19_1998 | 1998 | RR | RR_BEFORE | 0.6575 | 0.2236 | 24.5408 | 0.4591 | 0.2475 | 0.4722 | 0.8216 | 5.3816 | 0.6259 | 6.8955 | 0.4895 | 23.6141 | 2.7738 | 0.9649 | 0.0351 |
| RR_194_1998 | 1998 | RR | RR_BEFORE | 0.9952 | 0.3192 | 16.4050 | 0.3217 | 0.2708 | 0.5272 | 0.8333 | 5.7759 | 0.6823 | 7.9483 | 0.5948 | 20.7261 | 2.1157 | 0.7644 | 0.2356 |

| | | | | | | | | | | | | | | | | | | |
|---|---|---|---|---|---|---|---|---|---|---|---|---|---|---|---|---|---|---|
| RR_203_1998 | 1998 | RR | RR_BEFORE | 1.1330 | 0.3519 | 18.8155 | 0.3870 | 0.2782 | 0.5445 | 0.8894 | 5.7426 | 0.6775 | 7.9453 | 0.5945 | 22.3570 | 2.1950 | 0.8713 | 0.1287 |
| RR_212_1998 | 1998 | RR | RR_BEFORE | 0.6817 | 0.2442 | 15.9191 | 0.3416 | 0.2816 | 0.5526 | 0.7632 | 4.7697 | 0.5385 | 9.5395 | 0.7539 | 21.1677 | 2.2337 | 0.9868 | 0.0132 |
| RR_221_1998 | 1998 | RR | RR_BEFORE | 0.3333 | 0.1306 | 25.1812 | 0.4692 | 0.2221 | 0.4127 | 0.8246 | 5.0803 | 0.5829 | 6.5992 | 0.4599 | 24.1305 | 3.1060 | 0.9839 | 0.0161 |
| RR_230_1998 | 1998 | RR | RR_BEFORE | 0.4799 | 0.1796 | 24.9281 | 0.4690 | 0.2443 | 0.4648 | 0.8876 | 5.4659 | 0.6380 | 6.5973 | 0.4597 | 26.7106 | 2.4395 | 0.9960 | 0.0040 |
| RR_239_1998 | 1998 | RR | RR_BEFORE | 0.8528 | 0.2823 | 23.2477 | 0.4312 | 0.2512 | 0.4810 | 0.7718 | 5.9225 | 0.7032 | 7.3231 | 0.5323 | 26.9071 | 2.5735 | 0.8244 | 0.1679 |
| RR_246_2012 | 2012 | RR | RR_REWET | 0.8351 | 0.2813 | 15.6777 | 0.2813 | 0.2742 | 0.5351 | 0.7505 | 6.0000 | 0.7143 | 7.3626 | 0.5363 | 22.2386 | 1.9006 | 0.5263 | 0.4737 |
| RR_247_2012 | 2012 | RR | RR_REWET | 0.9106 | 0.2948 | 20.3745 | 0.3943 | 0.2930 | 0.5794 | 0.8235 | 5.5941 | 0.6563 | 8.5588 | 0.6559 | 24.2026 | 2.2371 | 0.9941 | 0.0059 |
| RR_249_2012 | 2012 | RR | RR_REWET | 0.2711 | 0.0895 | 61.1696 | 0.6755 | 0.1360 | 0.2100 | 0.3732 | 4.8478 | 0.5497 | 10.3913 | 0.8391 | 24.4085 | 1.6707 | 0.9891 | 0.0109 |
| RR_250_2012 | 2012 | RR | RR_REWET | 0.7689 | 0.2410 | 26.5147 | 0.4455 | 0.2204 | 0.4087 | 0.7384 | 5.7234 | 0.6748 | 9.9787 | 0.7979 | 22.8282 | 1.8429 | 0.9149 | 0.0851 |
| RR_251_2012 | 2012 | RR | RR_REWET | 0.9134 | 0.3072 | 16.7611 | 0.3573 | 0.3189 | 0.6403 | 0.9841 | 4.0816 | 0.4402 | 8.9660 | 0.6966 | 11.2957 | 0.6301 | 0.9932 | 0.0068 |
| RR_252_2012 | 2012 | RR | RR_REWET | 0.7106 | 0.2530 | 15.4421 | 0.3301 | 0.3162 | 0.6341 | 0.8130 | 5.8415 | 0.6916 | 9.7805 | 0.7780 | 20.6654 | 1.9579 | 0.9878 | 0.0122 |
| RR_253_2012 | 2012 | RR | RR_REWET | 0.6141 | 0.1787 | 44.0480 | 0.5322 | 0.1660 | 0.2806 | 0.6094 | 5.3612 | 0.6230 | 10.7414 | 0.8741 | 22.5761 | 1.6500 | 1.0000 | 0.0000 |
| RR_254_2012 | 2012 | RR | RR_REWET | 0.6802 | 0.2410 | 77.6310 | 0.7891 | 0.1456 | 0.2326 | 0.6789 | 5.9184 | 0.7026 | 11.8163 | 0.9816 | 36.6003 | 3.0550 | 0.2174 | 0.0000 |
| RR_255_2012 | 2012 | RR | RR_REWET | 0.7246 | 0.2293 | 29.0945 | 0.4285 | 0.1929 | 0.3439 | 0.6339 | 5.4203 | 0.6315 | 10.2681 | 0.8268 | 23.4484 | 0.5064 | 0.9928 | 0.0072 |
| RR_256_2012 | 2012 | RR | RR_REWET | 0.4771 | 0.1571 | 58.5035 | 0.6959 | 0.1532 | 0.2505 | 0.4516 | 4.9829 | 0.5690 | 11.4861 | 0.9486 | 32.0882 | 1.3348 | 0.6736 | 0.0023 |
| RR_257_2012 | 2012 | RR | RR_REWET | 1.2933 | 0.3906 | 20.4268 | 0.4118 | 0.2545 | 0.4888 | 0.9635 | 5.9766 | 0.7109 | 8.0469 | 0.6047 | 22.2580 | 2.3022 | 0.9922 | 0.0078 |
| RR_258_2012 | 2012 | RR | RR_REWET | 0.5772 | 0.2149 | 23.5651 | 0.4375 | 0.3394 | 0.6887 | 0.6735 | 5.5670 | 0.6524 | 8.6701 | 0.6670 | 28.7158 | 2.0876 | 0.9948 | 0.0052 |
| RR_259_2012 | 2012 | RR | RR_REWET | 0.2029 | 0.0696 | 46.9828 | 0.6137 | 0.1554 | 0.2555 | 0.6114 | 5.1356 | 0.5908 | 10.9313 | 0.8931 | 25.6649 | 1.2708 | 0.9956 | 0.0022 |
| RR_260_2012 | 2012 | RR | RR_REWET | 0.7463 | 0.2147 | 35.0946 | 0.5125 | 0.1988 | 0.3577 | 0.6619 | 5.9680 | 0.7097 | 10.8683 | 0.8868 | 23.4405 | 1.8719 | 1.0000 | 0.0000 |
| RR_261_2012 | 2012 | RR | RR_REWET | 0.4081 | 0.1410 | 33.7822 | 0.5014 | 0.1983 | 0.3567 | 0.7556 | 5.7333 | 0.6762 | 11.2000 | 0.9200 | 34.1095 | 5.6100 | 0.0816 | 0.0000 |
| RR_262_2012 | 2012 | RR | RR_REWET | 0.4220 | 0.1647 | 24.4809 | 0.4516 | 0.2393 | 0.4529 | 0.8758 | 6.0196 | 0.7171 | 9.2451 | 0.7245 | 33.5747 | 2.0017 | 1.0000 | 0.0000 |
| RR_263_2012 | 2012 | RR | RR_REWET | 0.4920 | 0.1814 | 25.7813 | 0.4353 | 0.2335 | 0.4394 | 0.8475 | 5.6809 | 0.6687 | 9.6915 | 0.7691 | 30.4705 | 1.4857 | 0.9894 | 0.0106 |
| RR_264_2012 | 2012 | RR | RR_REWET | 0.5817 | 0.2077 | 26.2541 | 0.4677 | 0.2055 | 0.3734 | 0.6287 | 6.3418 | 0.7631 | 8.8354 | 0.6835 | 22.8268 | 1.6886 | 1.0000 | 0.0000 |
| RR_265_2012 | 2012 | RR | RR_REWET | 0.4841 | 0.1583 | 65.8810 | 0.7330 | 0.1302 | 0.1963 | 0.4650 | 5.5364 | 0.6481 | 11.7471 | 0.9747 | 32.5691 | 0.8456 | 0.6471 | 0.0000 |
| RR_266_2012 | 2012 | RR | RR_REWET | 0.3341 | 0.1093 | 66.2232 | 0.7386 | 0.1318 | 0.2000 | 0.4253 | 5.4414 | 0.6345 | 11.6865 | 0.9686 | 31.4007 | 0.8456 | 0.7838 | 0.0000 |
| RR_267_2012 | 2012 | RR | RR_REWET | 0.2893 | 0.0947 | 60.7350 | 0.7278 | 0.1481 | 0.2384 | 0.3333 | 5.6667 | 0.6667 | 11.8571 | 0.9857 | 30.0667 | NA | 0.8571 | 0.0000 |
| RR_268_2012 | 2012 | RR | RR_REWET | 0.5612 | 0.1899 | 47.8447 | 0.5700 | 0.2132 | 0.3915 | 0.7556 | 5.1351 | 0.5907 | 10.4872 | 0.8487 | 27.3537 | 1.5173 | 0.9487 | 0.0000 |
| RR_269_2012 | 2012 | RR | RR_REWET | 0.5275 | 0.1700 | 60.6066 | 0.7053 | 0.1479 | 0.2379 | 0.4000 | 5.8947 | 0.6992 | 11.8500 | 0.9850 | 33.3316 | 1.5759 | 0.9500 | 0.0000 |
| RR_270_2012 | 2012 | RR | RR_REWET | 0.1535 | 0.0528 | 93.4405 | 0.7787 | 0.1366 | 0.2114 | 0.5977 | 5.7774 | 0.6825 | 11.4630 | 0.9463 | 31.0753 | 1.4411 | 0.9815 | 0.0000 |
| RR_271_2012 | 2012 | RR | RR_REWET | 0.3197 | 0.1278 | 21.7493 | 0.4226 | 0.3324 | 0.6723 | 0.6916 | 5.4023 | 0.6289 | 8.2471 | 0.6247 | 20.7236 | 1.6853 | 0.9885 | 0.0115 |
| RR_272_2012 | 2012 | RR | RR_REWET | 0.9954 | 0.3141 | 23.6865 | 0.4494 | 0.2406 | 0.4561 | 0.8209 | 6.1194 | 0.7313 | 8.1791 | 0.6179 | 22.8010 | 2.2565 | 1.0000 | 0.0000 |
| RR_273_2012 | 2012 | RR | RR_REWET | 0.2442 | 0.0844 | 53.4645 | 0.6331 | 0.1903 | 0.3377 | 0.5312 | 5.4136 | 0.6305 | 10.7906 | 0.8791 | 27.2918 | 1.4882 | 0.9738 | 0.0262 |
| RR_28_1998 | 1998 | RR | RR_BEFORE | 0.4845 | 0.1768 | 27.0806 | 0.4904 | 0.2220 | 0.4123 | 0.7911 | 5.2627 | 0.6090 | 6.9856 | 0.4986 | 26.1900 | 3.0165 | 0.9816 | 0.0184 |
| RR_37_1998 | 1998 | RR | RR_BEFORE | 0.4546 | 0.1720 | 25.2057 | 0.4722 | 0.2407 | 0.4563 | 0.8875 | 5.5063 | 0.6438 | 6.5111 | 0.4511 | 26.5864 | 2.3127 | 0.9958 | 0.0042 |
| RR_46_1998 | 1998 | RR | RR_BEFORE | 0.4202 | 0.1644 | 29.9811 | 0.5181 | 0.2262 | 0.4223 | 0.6497 | 5.6939 | 0.6706 | 6.4227 | 0.4423 | 24.0209 | 2.1056 | 0.9490 | 0.0510 |
| RR_55_1998 | 1998 | RR | RR_BEFORE | 0.5233 | 0.1955 | 24.9041 | 0.4333 | 0.2370 | 0.4476 | 0.6810 | 5.5345 | 0.6478 | 6.8793 | 0.4879 | 24.0158 | 2.5984 | 0.7586 | 0.2414 |
| RR_64_1998 | 1998 | RR | RR_BEFORE | 0.5649 | 0.2099 | 24.4916 | 0.4652 | 0.2690 | 0.5230 | 0.8865 | 5.6489 | 0.6641 | 5.8235 | 0.3824 | 26.6201 | 2.0145 | 0.9947 | 0.0053 |
| RR_73_1998 | 1998 | RR | RR_BEFORE | 1.0626 | 0.3315 | 22.2175 | 0.4345 | 0.2640 | 0.5112 | 0.9110 | 5.7443 | 0.6778 | 7.4651 | 0.5465 | 22.5863 | 2.4538 | 1.0000 | 0.0000 |
| RR_82_1998 | 1998 | RR | RR_BEFORE | 0.3704 | 0.1472 | 29.8405 | 0.5217 | 0.2433 | 0.4624 | 0.8725 | 5.6103 | 0.6586 | 7.2273 | 0.5227 | 23.3946 | 2.8314 | 1.0000 | 0.0000 |
| RR_91_1998 | 1998 | RR | RR_BEFORE | 1.5321 | 0.4340 | 20.1275 | 0.3954 | 0.2767 | 0.5411 | 0.9559 | 5.9552 | 0.7079 | 9.8971 | 0.7897 | 19.3631 | 2.2961 | 0.9706 | 0.0147 |
| SE_A1_2010 | 2010 | SE | SE_REWET | 0.5314 | 0.1762 | 18.1385 | 0.3717 | 0.3737 | 0.7693 | 0.6202 | 6.0696 | 0.7242 | 7.4215 | 0.5422 | 15.4859 | 0.9082 | 0.4845 | 0.5155 |
| SE_A1a_2010 | 2010 | SE | SE_REWET | 0.3682 | 0.1459 | 19.0090 | 0.3870 | 0.3800 | 0.7842 | 0.6474 | 6.1538 | 0.7363 | 7.6875 | 0.5688 | 15.3260 | 0.8420 | 0.5385 | 0.4615 |
| SE_A1b_2010 | 2010 | SE | SE_REWET | 0.3771 | 0.1510 | 19.4113 | 0.3975 | 0.3795 | 0.7829 | 0.6520 | 6.6264 | 0.8038 | 7.2048 | 0.5205 | 13.5774 | 0.7527 | 0.2308 | 0.7692 |
| SE_A2_2010 | 2010 | SE | SE_REWET | 0.5435 | 0.1898 | 14.2815 | 0.2966 | 0.3460 | 0.7042 | 0.5972 | 5.7143 | 0.6735 | 8.4231 | 0.6423 | 18.1136 | 0.9954 | 0.7857 | 0.2143 |
| SE_A2a_2010 | 2010 | SE | SE_REWET | 0.4592 | 0.1671 | 14.8613 | 0.3063 | 0.3493 | 0.7118 | 0.5315 | 5.8125 | 0.6875 | 8.1493 | 0.6149 | 16.8542 | 1.0870 | 0.7500 | 0.2500 |
| SE_A2b_2010 | 2010 | SE | SE_REWET | 0.6675 | 0.2170 | 14.4780 | 0.2978 | 0.3505 | 0.7148 | 0.5397 | 5.2069 | 0.6010 | 8.5417 | 0.6542 | 20.6196 | 1.1424 | 0.8966 | 0.1034 |
| SE_A3_2010 | 2010 | SE | SE_REWET | 0.3867 | 0.1527 | 17.3791 | 0.3562 | 0.3662 | 0.7518 | 0.6449 | 6.4348 | 0.7764 | 7.7619 | 0.5762 | 14.8525 | 0.8684 | 0.4348 | 0.5652 |
| SE_A3a_2010 | 2010 | SE | SE_REWET | 0.3139 | 0.1281 | 16.3605 | 0.3350 | 0.3576 | 0.7314 | 0.5385 | 5.9341 | 0.7049 | 7.5862 | 0.5586 | 14.3154 | 0.7944 | 0.5604 | 0.4396 |
| SE_A3b_2010 | 2010 | SE | SE_REWET | 0.3335 | 0.1326 | 15.4418 | 0.3164 | 0.3481 | 0.7091 | 0.5876 | 5.8814 | 0.6973 | 8.2642 | 0.6264 | 16.3971 | 0.9966 | 0.7288 | 0.2712 |
| SE_A4_2010 | 2010 | SE | SE_REWET | 0.6770 | 0.2214 | 12.7049 | 0.2651 | 0.3556 | 0.7268 | 0.5608 | 5.1839 | 0.5977 | 8.5301 | 0.6530 | 19.6486 | 0.9852 | 0.9080 | 0.0920 |
| SE_A4a_2010 | 2010 | SE | SE_REWET | 0.8708 | 0.2724 | 14.0862 | 0.2952 | 0.3646 | 0.7478 | 0.6800 | 5.3023 | 0.6146 | 8.4419 | 0.6442 | 19.9485 | 0.9042 | 0.7209 | 0.2791 |

| | | | | | | | | | | | | | | | | | | |
|---|---|---|---|---|---|---|---|---|---|---|---|---|---|---|---|---|---|---|
| SE_A4b_2010 | 2010 | SE | SE_REWET | 0.2841 | 0.1138 | 15.4219 | 0.3125 | 0.3391 | 0.6880 | 0.5082 | 5.6066 | 0.6581 | 8.3556 | 0.6356 | 18.4821 | 1.2758 | 0.8361 | 0.1639 |
| SE_A5_2010 | 2010 | SE | SE_REWET | 0.3317 | 0.1343 | 17.0399 | 0.3487 | 0.3609 | 0.7393 | 0.5946 | 6.2838 | 0.7548 | 7.5147 | 0.5515 | 13.8905 | 0.7808 | 0.4667 | 0.5333 |
| SE_A5a_2010 | 2010 | SE | SE_REWET | 0.3337 | 0.1337 | 17.5947 | 0.3589 | 0.3533 | 0.7213 | 0.6095 | 6.2857 | 0.7551 | 7.7053 | 0.5705 | 14.5340 | 0.8364 | 0.5472 | 0.4528 |
| SE_A5b_2010 | 2010 | SE | SE_REWET | 0.5126 | 0.1767 | 13.1510 | 0.2713 | 0.3582 | 0.7329 | 0.5413 | 5.4000 | 0.6286 | 8.1405 | 0.6140 | 17.3058 | 0.8705 | 0.7705 | 0.2295 |
| SE_B1_2010 | 2010 | SE | SE_REWET | 0.4157 | 0.1637 | 18.6664 | 0.3871 | 0.3846 | 0.7949 | 0.6860 | 6.8551 | 0.8364 | 7.1803 | 0.5180 | 13.3213 | 0.7410 | 0.1884 | 0.8116 |
| SE_B1a_2010 | 2010 | SE | SE_REWET | 0.4054 | 0.1591 | 17.6124 | 0.3658 | 0.3904 | 0.8087 | 0.7222 | 6.6667 | 0.8095 | 7.0000 | 0.5000 | 13.4500 | 0.8035 | 0.3333 | 0.6667 |
| SE_B1b_2010 | 2010 | SE | SE_REWET | 0.4217 | 0.1650 | 19.0771 | 0.3940 | 0.3751 | 0.7726 | 0.6842 | 6.9158 | 0.8451 | 7.3261 | 0.5326 | 13.9565 | 0.7585 | 0.1579 | 0.8421 |
| SE_B2_2010 | 2010 | SE | SE_REWET | 0.4149 | 0.1625 | 18.9836 | 0.3908 | 0.3835 | 0.7923 | 0.7014 | 6.8333 | 0.8333 | 7.1915 | 0.5191 | 14.1514 | 0.8459 | 0.1667 | 0.8333 |
| SE_B2a_2010 | 2010 | SE | SE_REWET | 0.3481 | 0.1381 | 17.1335 | 0.3505 | 0.3684 | 0.7569 | 0.5793 | 5.9784 | 0.7112 | 7.6605 | 0.5660 | 15.2462 | 0.8452 | 0.5671 | 0.4329 |
| SE_B2b_2010 | 2010 | SE | SE_REWET | 0.5693 | 0.1965 | 17.8777 | 0.3651 | 0.3769 | 0.7769 | 0.6614 | 5.9067 | 0.7010 | 7.9701 | 0.5970 | 17.7361 | 0.9585 | 0.5733 | 0.4267 |
| SE_B3_2010 | 2010 | SE | SE_REWET | 0.6174 | 0.2031 | 17.3574 | 0.3525 | 0.3550 | 0.7253 | 0.5652 | 5.5506 | 0.6501 | 7.8025 | 0.5802 | 18.2288 | 0.9624 | 0.6129 | 0.3871 |
| SE_B3a_2010 | 2010 | SE | SE_REWET | 0.2869 | 0.1182 | 15.1160 | 0.3103 | 0.3502 | 0.7140 | 0.5000 | 5.7471 | 0.6782 | 7.6667 | 0.5667 | 14.2824 | 0.8024 | 0.6364 | 0.3636 |
| SE_B3b_2010 | 2010 | SE | SE_REWET | 0.2856 | 0.1171 | 17.3809 | 0.3503 | 0.3488 | 0.7107 | 0.5230 | 5.5263 | 0.6466 | 7.8333 | 0.5833 | 15.8207 | 0.9184 | 0.7966 | 0.2034 |
| SE_B4_2010 | 2010 | SE | SE_REWET | 0.3506 | 0.1384 | 16.5728 | 0.3369 | 0.3752 | 0.7729 | 0.6410 | 5.8077 | 0.6868 | 8.2340 | 0.6234 | 16.6049 | 0.9271 | 0.8621 | 0.1379 |
| SE_B4a_2010 | 2010 | SE | SE_REWET | 0.8012 | 0.2544 | 12.9188 | 0.2734 | 0.3385 | 0.6865 | 0.5636 | 5.6207 | 0.6601 | 8.3913 | 0.6391 | 18.8126 | 0.9917 | 0.8182 | 0.1818 |
| SE_B4b_2010 | 2010 | SE | SE_REWET | 0.2908 | 0.1192 | 19.7986 | 0.3961 | 0.3716 | 0.7644 | 0.5862 | 5.8889 | 0.6984 | 7.4615 | 0.5462 | 15.2533 | 0.8700 | 0.5932 | 0.4068 |
| SE_B5_2010 | 2010 | SE | SE_REWET | 0.2912 | 0.1182 | 15.0539 | 0.3075 | 0.3470 | 0.7064 | 0.5220 | 5.6415 | 0.6631 | 8.1333 | 0.6133 | 16.5389 | 1.0553 | 0.7736 | 0.2264 |
| SE_B5a_2010 | 2010 | SE | SE_REWET | 0.5356 | 0.1872 | 15.7344 | 0.3240 | 0.3626 | 0.7433 | 0.6202 | 6.0800 | 0.7257 | 7.7556 | 0.5756 | 15.7833 | 0.8447 | 0.5294 | 0.4706 |
| SE_B5b_2010 | 2010 | SE | SE_REWET | 0.3658 | 0.1467 | 18.2817 | 0.3758 | 0.3685 | 0.7572 | 0.6095 | 6.6286 | 0.8041 | 7.1818 | 0.5182 | 13.4213 | 0.7515 | 0.2222 | 0.7778 |
| SE_C1_2010 | 2010 | SE | SE_REWET | 0.4601 | 0.1745 | 18.8366 | 0.3888 | 0.3789 | 0.7816 | 0.6753 | 6.7917 | 0.8274 | 7.3190 | 0.5319 | 14.4996 | 0.7933 | 0.1667 | 0.8333 |
| SE_C1a_2010 | 2010 | SE | SE_REWET | 0.6949 | 0.2280 | 17.1341 | 0.3529 | 0.3633 | 0.7449 | 0.6316 | 5.8462 | 0.6923 | 7.9524 | 0.5952 | 18.4508 | 1.0084 | 0.5385 | 0.4615 |
| SE_C1b_2010 | 2010 | SE | SE_REWET | 0.4288 | 0.1669 | 19.0851 | 0.3936 | 0.3836 | 0.7927 | 0.6895 | 6.8630 | 0.8376 | 7.3714 | 0.5371 | 14.5536 | 0.8021 | 0.1781 | 0.8219 |
| SE_C2_2010 | 2010 | SE | SE_REWET | 0.3616 | 0.1449 | 19.7900 | 0.4036 | 0.3709 | 0.7628 | 0.6277 | 6.7128 | 0.8161 | 6.9878 | 0.4988 | 13.9687 | 0.8272 | 0.1489 | 0.8511 |
| SE_C2a_2010 | 2010 | SE | SE_REWET | 0.5201 | 0.1847 | 17.3487 | 0.3569 | 0.3780 | 0.7795 | 0.6944 | 6.2593 | 0.7513 | 7.3913 | 0.5391 | 16.3644 | 0.9621 | 0.4444 | 0.5556 |
| SE_C2b_2010 | 2010 | SE | SE_REWET | 0.4344 | 0.1676 | 18.8291 | 0.3873 | 0.3870 | 0.8006 | 0.7027 | 6.6622 | 0.8089 | 7.6667 | 0.5667 | 15.6595 | 0.8763 | 0.3514 | 0.6486 |
| SE_C3_2010 | 2010 | SE | SE_REWET | 0.3337 | 0.1343 | 18.3843 | 0.3735 | 0.3710 | 0.7629 | 0.5848 | 6.1786 | 0.7398 | 7.4340 | 0.5434 | 14.3574 | 0.8274 | 0.4386 | 0.5614 |
| SE_C3a_2010 | 2010 | SE | SE_REWET | 0.3316 | 0.1335 | 15.6025 | 0.3218 | 0.3354 | 0.6792 | 0.5946 | 6.6000 | 0.8000 | 7.6087 | 0.5609 | 14.2095 | 0.9584 | 0.7297 | 0.2703 |
| SE_C3b_2010 | 2010 | SE | SE_REWET | 0.2502 | 0.1041 | 15.0815 | 0.2989 | 0.3748 | 0.7719 | 0.5797 | 5.5435 | 0.6491 | 8.1842 | 0.6184 | 15.8264 | 0.9843 | 0.7391 | 0.2609 |
| SE_C4_2010 | 2010 | SE | SE_REWET | 0.4078 | 0.1558 | 18.7754 | 0.3843 | 0.3726 | 0.7668 | 0.6230 | 6.4368 | 0.7767 | 7.3293 | 0.5329 | 14.2666 | 0.7876 | 0.2809 | 0.7191 |
| SE_C4a_2010 | 2010 | SE | SE_REWET | 0.6709 | 0.2241 | 16.3582 | 0.3383 | 0.3735 | 0.7689 | 0.6627 | 6.0450 | 0.7207 | 7.4545 | 0.5455 | 15.9045 | 0.7876 | 0.3514 | 0.6486 |
| SE_C4b_2010 | 2010 | SE | SE_REWET | 1.1760 | 0.3474 | 11.7844 | 0.2500 | 0.3457 | 0.7036 | 0.5359 | 4.6000 | 0.5143 | 8.6442 | 0.6644 | 22.7657 | 0.9768 | 0.8830 | 0.1170 |
| SE_C5_2010 | 2010 | SE | SE_REWET | 0.3176 | 0.1284 | 19.2859 | 0.3898 | 0.3596 | 0.7361 | 0.5895 | 6.4043 | 0.7720 | 7.2535 | 0.5254 | 15.1008 | 0.9637 | 0.3684 | 0.6316 |
| SE_C5a_2010 | 2010 | SE | SE_REWET | 0.2507 | 0.1041 | 17.1835 | 0.3442 | 0.3123 | 0.6248 | 0.5051 | 5.6327 | 0.6618 | 8.0141 | 0.6014 | 17.3965 | 1.2766 | 0.8384 | 0.1616 |
| SE_C5b_2010 | 2010 | SE | SE_REWET | 1.0306 | 0.3122 | 12.9251 | 0.2726 | 0.3418 | 0.6943 | 0.6066 | 5.2000 | 0.6000 | 8.4690 | 0.6469 | 20.6763 | 1.0039 | 0.8298 | 0.1702 |
| SE_D1_2010 | 2010 | SE | SE_REWET | 0.4482 | 0.1620 | 19.4306 | 0.3930 | 0.3616 | 0.7408 | 0.6000 | 6.1429 | 0.7347 | 7.6232 | 0.5623 | 17.6021 | 1.0876 | 0.4839 | 0.5161 |
| SE_D1a_2010 | 2010 | SE | SE_REWET | 0.3453 | 0.1391 | 18.6932 | 0.3814 | 0.3711 | 0.7633 | 0.6158 | 6.6000 | 0.8000 | 7.1724 | 0.5172 | 13.0649 | 0.6974 | 0.2542 | 0.7458 |
| SE_D1b_2010 | 2010 | SE | SE_REWET | 0.4648 | 0.1768 | 18.5052 | 0.3832 | 0.3896 | 0.8068 | 0.7284 | 6.8846 | 0.8407 | 7.7200 | 0.5720 | 15.8915 | 0.8956 | 0.3333 | 0.6667 |
| SE_D2_2010 | 2010 | SE | SE_REWET | 0.4195 | 0.1596 | 14.3926 | 0.3006 | 0.3665 | 0.7524 | 0.6370 | 5.9556 | 0.7079 | 8.3409 | 0.6341 | 19.0972 | 1.0915 | 0.8222 | 0.1778 |
| SE_D2a_2010 | 2010 | SE | SE_REWET | 0.2932 | 0.1186 | 11.5593 | 0.2399 | 0.3460 | 0.7041 | 0.4928 | 5.3913 | 0.6273 | 8.2609 | 0.6261 | 16.9795 | 0.9332 | 0.9130 | 0.0870 |
| SE_D2b_2010 | 2010 | SE | SE_REWET | 0.3185 | 0.1276 | 16.3599 | 0.3335 | 0.3605 | 0.7383 | 0.5625 | 6.0455 | 0.7208 | 7.6304 | 0.5630 | 15.0547 | 0.8486 | 0.5833 | 0.4167 |
| SE_D3_2010 | 2010 | SE | SE_REWET | 0.3233 | 0.1311 | 14.9247 | 0.3066 | 0.3852 | 0.7964 | 0.7007 | 5.8723 | 0.6960 | 6.5417 | 0.4542 | 14.2618 | 0.8634 | 0.5918 | 0.4082 |
| SE_D3a_2010 | 2010 | SE | SE_REWET | 0.3797 | 0.1519 | 15.2051 | 0.3209 | 0.3817 | 0.7882 | 0.7733 | 6.0000 | 0.7143 | 6.3913 | 0.4391 | 15.2724 | 0.8747 | 0.6538 | 0.3462 |
| SE_D3b_2010 | 2010 | SE | SE_REWET | 0.3299 | 0.1339 | 16.1783 | 0.3324 | 0.3658 | 0.7508 | 0.5753 | 6.2535 | 0.7505 | 7.4384 | 0.5438 | 13.0504 | 0.6888 | 0.3973 | 0.6027 |
| SE_D4_2010 | 2010 | SE | SE_REWET | 0.2788 | 0.1144 | 17.6632 | 0.3562 | 0.3510 | 0.7160 | 0.5161 | 5.9333 | 0.7048 | 7.4400 | 0.5440 | 15.6507 | 1.0086 | 0.5484 | 0.4516 |
| SE_D4a_2010 | 2010 | SE | SE_REWET | 0.7200 | 0.2345 | 15.3228 | 0.3186 | 0.3605 | 0.7383 | 0.5926 | 5.7179 | 0.6740 | 7.8026 | 0.5803 | 17.4146 | 0.8238 | 0.4937 | 0.5063 |
| SE_D4b_2010 | 2010 | SE | SE_REWET | 0.2403 | 0.1011 | 12.9662 | 0.2653 | 0.3332 | 0.6740 | 0.4242 | 5.3256 | 0.6179 | 7.9211 | 0.5921 | 16.4352 | 1.0943 | 0.9091 | 0.0909 |
| SE_D5_2010 | 2010 | SE | SE_REWET | 0.2835 | 0.1165 | 16.2473 | 0.3302 | 0.3508 | 0.7155 | 0.5072 | 5.8889 | 0.6984 | 7.5250 | 0.5525 | 15.0074 | 0.9316 | 0.5652 | 0.4348 |
| SE_D5a_2010 | 2010 | SE | SE_REWET | 0.2921 | 0.1200 | 14.5598 | 0.2980 | 0.3585 | 0.7336 | 0.5333 | 5.8235 | 0.6891 | 7.7879 | 0.5788 | 14.0596 | 0.7994 | 0.6571 | 0.3429 |
| SE_D5b_2010 | 2010 | SE | SE_REWET | 0.3163 | 0.1275 | 14.6027 | 0.3004 | 0.3627 | 0.7435 | 0.5429 | 5.7353 | 0.6765 | 7.9143 | 0.5914 | 15.1075 | 0.8035 | 0.7143 | 0.2857 |
| SE_E1_2002 | 2002 | SE | SE_BEFORE | 0.3286 | 0.1324 | 20.5725 | 0.4156 | 0.3612 | 0.7398 | 0.5833 | 6.5000 | 0.7857 | 7.0000 | 0.5000 | 15.1700 | 0.9676 | 0.2500 | 0.7500 |
| SE_E2_2002 | 2002 | SE | SE_BEFORE | 0.4407 | 0.1689 | 17.6238 | 0.3697 | 0.3872 | 0.8010 | 0.7056 | 6.4213 | 0.7745 | 8.1176 | 0.6118 | 15.6829 | 1.0074 | 0.6853 | 0.3147 |

| | | | | | | | | | | | | | | | | | | |
|---|---|---|---|---|---|---|---|---|---|---|---|---|---|---|---|---|---|---|
| SE_E3_2002 | 2002 | SE | SE_BEFORE | 0.3916 | 0.1563 | 19.5235 | 0.4017 | 0.3782 | 0.7800 | 0.6575 | 6.9315 | 0.8474 | 7.0000 | 0.5000 | 12.9611 | 0.7022 | 0.0411 | 0.9589 |
| SE_E4_2002 | 2002 | SE | SE_BEFORE | 0.3890 | 0.1552 | 19.5684 | 0.4022 | 0.3774 | 0.7780 | 0.6541 | 6.9057 | 0.8437 | 7.0085 | 0.5009 | 13.1232 | 0.7199 | 0.0566 | 0.9434 |
| SE_E5_2002 | 2002 | SE | SE_BEFORE | 0.4724 | 0.1737 | 19.2646 | 0.3941 | 0.3680 | 0.7559 | 0.6053 | 6.5541 | 0.7934 | 7.2137 | 0.5214 | 14.9751 | 0.9008 | 0.2424 | 0.7576 |
| SE_F1_2002 | 2002 | SE | SE_BEFORE | 0.3286 | 0.1324 | 20.5725 | 0.4156 | 0.3612 | 0.7398 | 0.5833 | 6.5000 | 0.7857 | 7.0000 | 0.5000 | 15.1700 | 0.9676 | 0.2500 | 0.7500 |
| SE_F2_2002 | 2002 | SE | SE_BEFORE | 0.3789 | 0.1514 | 19.7685 | 0.4050 | 0.3746 | 0.7714 | 0.6420 | 6.8519 | 0.8360 | 7.0000 | 0.5000 | 13.4319 | 0.7554 | 0.0741 | 0.9259 |
| SE_F3_2002 | 2002 | SE | SE_BEFORE | 0.2867 | 0.1171 | 14.2875 | 0.2896 | 0.4050 | 0.8431 | 0.8148 | 6.1111 | 0.7302 | 5.6667 | 0.3667 | 11.8556 | 0.8611 | 0.4444 | 0.5556 |
| SE_F4_2002 | 2002 | SE | SE_BEFORE | 0.4307 | 0.1681 | 19.1771 | 0.3961 | 0.3819 | 0.7886 | 0.6707 | 6.9756 | 0.8537 | 7.1728 | 0.5173 | 13.5495 | 0.7507 | 0.0854 | 0.9146 |
| SE_F5_2002 | 2002 | SE | SE_BEFORE | 0.4059 | 0.1606 | 19.6596 | 0.4036 | 0.3774 | 0.7781 | 0.6548 | 6.8571 | 0.8367 | 6.8519 | 0.4852 | 13.6600 | 0.9365 | 0.0357 | 0.9643 |
| SE_G1_2002 | 2002 | SE | SE_BEFORE | 0.3803 | 0.1441 | 21.3803 | 0.4240 | 0.3489 | 0.7111 | 0.5395 | 5.6579 | 0.6654 | 5.8293 | 0.3829 | 21.3895 | 2.5340 | 0.5395 | 0.4605 |
| SE_G2_2002 | 2002 | SE | SE_BEFORE | 0.3081 | 0.1248 | 19.5474 | 0.3851 | 0.3675 | 0.7549 | 0.6667 | 6.3289 | 0.7613 | 6.5104 | 0.4510 | 14.3196 | 0.9065 | 0.3355 | 0.6645 |
| SE_G3_2002 | 2002 | SE | SE_BEFORE | 0.3801 | 0.1519 | 19.7488 | 0.4047 | 0.3749 | 0.7722 | 0.6434 | 6.8605 | 0.8372 | 7.0000 | 0.5000 | 13.3893 | 0.7502 | 0.0698 | 0.9302 |
| SE_G4_2002 | 2002 | SE | SE_BEFORE | 0.4091 | 0.1598 | 19.3375 | 0.3956 | 0.3761 | 0.7751 | 0.6699 | 6.7194 | 0.8171 | 7.2015 | 0.5202 | 15.6219 | 0.8661 | 0.2206 | 0.7794 |
| SE_G5_2002 | 2002 | SE | SE_BEFORE | 0.4160 | 0.1615 | 19.4445 | 0.3988 | 0.3758 | 0.7743 | 0.6484 | 6.7801 | 0.8257 | 7.2886 | 0.5289 | 14.9207 | 0.8884 | 0.2167 | 0.7833 |
| SE_H1_2002 | 2002 | SE | SE_BEFORE | 0.5245 | 0.1935 | 18.2530 | 0.3776 | 0.3880 | 0.8030 | 0.7971 | 6.6087 | 0.8012 | 8.0000 | 0.6000 | 19.0421 | 1.4931 | 0.5652 | 0.4348 |
| SE_H2_2002 | 2002 | SE | SE_BEFORE | 0.3121 | 0.1232 | 23.7425 | 0.4443 | 0.3357 | 0.6800 | 0.8990 | 5.8485 | 0.6926 | 6.8667 | 0.4867 | 19.0955 | 2.5651 | 0.6970 | 0.3030 |
| SE_H3_2002 | 2002 | SE | SE_BEFORE | 0.2612 | 0.1072 | 16.6239 | 0.3333 | 0.3308 | 0.6684 | 0.5741 | 5.7831 | 0.6833 | 6.9505 | 0.4951 | 16.2380 | 1.2743 | 0.8830 | 0.1170 |
| SE_H4_2002 | 2002 | SE | SE_BEFORE | 0.4159 | 0.1598 | 18.8913 | 0.3868 | 0.3739 | 0.7699 | 0.6537 | 6.6839 | 0.8120 | 7.1601 | 0.5160 | 14.5815 | 0.9746 | 0.2424 | 0.7576 |
| SE_H5_2002 | 2002 | SE | SE_BEFORE | 0.3781 | 0.1512 | 19.5209 | 0.4007 | 0.3755 | 0.7736 | 0.6569 | 6.7647 | 0.8235 | 6.9051 | 0.4905 | 13.6912 | 0.7704 | 0.1202 | 0.8798 |
| SE_I1_2010 | 2010 | SE | SE_REWET | 0.3520 | 0.1428 | 20.3469 | 0.4136 | 0.2400 | 0.4548 | 0.1333 | 5.4000 | 0.6286 | 7.0000 | 0.5000 | 20.1400 | 2.4532 | 0.8182 | 0.1818 |
| SE_I2_2010 | 2010 | SE | SE_REWET | 0.3900 | 0.1560 | 19.6210 | 0.4033 | 0.3510 | 0.7159 | 0.5556 | 6.6667 | 0.8095 | 7.0000 | 0.5000 | 14.2500 | 1.0383 | 0.3750 | 0.6250 |
| SE_I3_2010 | 2010 | SE | SE_REWET | 0.3490 | 0.1404 | 17.1159 | 0.3506 | 0.3914 | 0.8110 | 0.7333 | 6.6000 | 0.8000 | 6.4000 | 0.4400 | 12.3200 | 0.7538 | 0.2000 | 0.8000 |
| SE_I4_2010 | 2010 | SE | SE_REWET | 0.6752 | 0.2409 | 14.1259 | 0.3069 | 0.3697 | 0.7599 | 0.8642 | 6.1852 | 0.7407 | 9.5385 | 0.7538 | 21.7660 | 1.7562 | 1.0000 | 0.0000 |
| SE_I5_2010 | 2010 | SE | SE_REWET | 0.3833 | 0.1531 | 20.5913 | 0.4096 | 0.3704 | 0.7616 | 0.6858 | 6.8851 | 0.8407 | 6.9639 | 0.4964 | 12.9885 | 0.6450 | 0.0787 | 0.9213 |
| SE_J1_2010 | 2010 | SE | SE_REWET | 0.2560 | 0.0987 | 17.3318 | 0.3666 | 0.3559 | 0.7273 | 1.0000 | 6.2000 | 0.7429 | 6.0000 | 0.4000 | 20.0166 | 1.4217 | 1.0000 | 0.0000 |
| SE_J2_2010 | 2010 | SE | SE_REWET | 0.4571 | 0.1765 | 18.6284 | 0.3868 | 0.3877 | 0.8022 | 0.7143 | 7.0000 | 0.8571 | 7.4286 | 0.5429 | 14.4571 | 0.7740 | 0.1429 | 0.8571 |
| SE_J3_2010 | 2010 | SE | SE_REWET | 0.3400 | 0.1387 | 20.5761 | 0.4168 | 0.2050 | 0.3723 | 0.0000 | 5.0000 | 0.5714 | NA | NA | 22.0000 | 2.9000 | 1.0000 | 0.0000 |
| SE_J4_2010 | 2010 | SE | SE_REWET | 0.3935 | 0.1570 | 19.2790 | 0.3970 | 0.3805 | 0.7854 | 0.6694 | 6.9512 | 0.8502 | 6.9508 | 0.4951 | 12.7494 | 0.6830 | 0.0244 | 0.9756 |
| SE_J5_2010 | 2010 | SE | SE_REWET | 0.4000 | 0.1594 | 19.4300 | 0.4005 | 0.3802 | 0.7847 | 0.6667 | 7.0000 | 0.8571 | 7.0000 | 0.5000 | 12.7000 | 0.6660 | 0.0099 | 0.9901 |
| SE_K2_2010 | 2010 | SE | SE_REWET | 0.4000 | 0.1594 | 19.4300 | 0.4005 | 0.3802 | 0.7847 | 0.6667 | 7.0000 | 0.8571 | 7.0000 | 0.5000 | 12.7000 | 0.6660 | 0.0000 | 1.0000 |
| SE_K3_2010 | 2010 | SE | SE_REWET | 0.3484 | 0.1402 | 18.9009 | 0.3889 | 0.3784 | 0.7803 | 0.6923 | 6.5000 | 0.7857 | 7.0500 | 0.5050 | 12.8867 | 0.7820 | 0.3462 | 0.6538 |
| SE_K4_2010 | 2010 | SE | SE_REWET | 0.1450 | 0.0642 | 7.8595 | 0.1510 | 0.4361 | 0.9162 | 1.0000 | 5.0000 | 0.5714 | 4.0000 | 0.2000 | 10.8000 | 1.1050 | 1.0000 | 0.0000 |
| SE_K5_2010 | 2010 | SE | SE_REWET | 0.2867 | 0.1171 | 14.2875 | 0.2896 | 0.4050 | 0.8431 | 0.8148 | 6.1111 | 0.7302 | 5.6667 | 0.3667 | 11.8556 | 0.8611 | 0.5000 | 0.5000 |
| SE_L1_2010 | 2010 | SE | SE_REWET | 0.5103 | 0.1902 | 17.6787 | 0.3709 | 0.3856 | 0.7975 | 0.7471 | 6.9310 | 0.8473 | 7.7931 | 0.5793 | 16.3307 | 0.9719 | 0.4000 | 0.6000 |
| SE_L2_2010 | 2010 | SE | SE_REWET | 0.3487 | 0.1413 | 17.2861 | 0.3659 | 0.3834 | 0.7921 | 0.6800 | 6.0400 | 0.7200 | 6.7500 | 0.4750 | 12.0380 | 0.9170 | 1.0000 | 0.0000 |
| SE_L3_2010 | 2010 | SE | SE_REWET | 0.3995 | 0.1593 | 19.3983 | 0.4000 | 0.3803 | 0.7849 | 0.6667 | 6.9863 | 0.8552 | 7.0000 | 0.5000 | 12.6904 | 0.6691 | 0.0137 | 0.9863 |
| SE_L4_2010 | 2010 | SE | SE_REWET | 0.3982 | 0.1588 | 19.4584 | 0.4009 | 0.3797 | 0.7836 | 0.6646 | 6.9876 | 0.8554 | 7.0000 | 0.5000 | 12.7614 | 0.6735 | 0.0062 | 0.9938 |
| SE_L5_2010 | 2010 | SE | SE_REWET | 0.4000 | 0.1594 | 19.4300 | 0.4005 | 0.3802 | 0.7847 | 0.6667 | 7.0000 | 0.8571 | 7.0000 | 0.5000 | 12.7000 | 0.6660 | 0.0000 | 1.0000 |
| SE_M1_2002 | 2002 | SE | SE_BEFORE | 0.1525 | 0.0672 | 21.7382 | 0.4190 | 0.3590 | 0.7348 | 1.0000 | 5.0000 | 0.5714 | 5.3750 | 0.3375 | 19.3126 | 3.9631 | 1.0000 | 0.0000 |
| SE_M2_2002 | 2002 | SE | SE_BEFORE | 0.3490 | 0.1404 | 17.1159 | 0.3506 | 0.3914 | 0.8110 | 0.7333 | 6.6000 | 0.8000 | 6.4000 | 0.4400 | 12.3200 | 0.7538 | 0.2000 | 0.8000 |
| SE_M3_2002 | 2002 | SE | SE_BEFORE | 0.1750 | 0.0764 | 24.4491 | 0.4662 | 0.3180 | 0.6383 | 1.0000 | 5.0000 | 0.5714 | 6.0000 | 0.4000 | 21.5500 | 4.5000 | 1.0000 | 0.0000 |
| SE_M4_2002 | 2002 | SE | SE_BEFORE | 0.1450 | 0.0642 | 7.8595 | 0.1510 | 0.4361 | 0.9162 | 1.0000 | 5.0000 | 0.5714 | 4.0000 | 0.2000 | 10.8000 | 1.1050 | 1.0000 | 0.0000 |
| SE_M5_2002 | 2002 | SE | SE_BEFORE | 0.6250 | 0.2218 | 12.6673 | 0.2726 | 0.4308 | 0.9036 | 0.9841 | 6.5238 | 0.7891 | 8.4286 | 0.6429 | 21.0333 | 1.3103 | 0.9524 | 0.0476 |
| SE_N1_2002 | 2002 | SE | SE_BEFORE | 0.1450 | 0.0642 | 7.8595 | 0.1510 | 0.4361 | 0.9162 | 1.0000 | 5.0000 | 0.5714 | 4.0000 | 0.2000 | 10.8000 | 1.1050 | 1.0000 | 0.0000 |
| SE_N2_2002 | 2002 | SE | SE_BEFORE | 0.8000 | 0.2785 | 13.8191 | 0.3046 | 0.4324 | 0.9074 | 1.0000 | 7.0000 | 0.8571 | 10.0000 | 0.8000 | 25.0000 | 1.4217 | 1.0000 | 0.0000 |
| SE_N3_2002 | 2002 | SE | SE_BEFORE | 0.7173 | 0.2536 | 19.5993 | 0.3725 | 0.4039 | 0.8403 | 0.9359 | 6.9048 | 0.8435 | 8.8000 | 0.6800 | 25.4034 | 1.4244 | 0.8077 | 0.0000 |
| SE_N4_2002 | 2002 | SE | SE_BEFORE | 0.8000 | 0.2785 | 13.8191 | 0.3046 | 0.4324 | 0.9074 | 1.0000 | 7.0000 | 0.8571 | 10.0000 | 0.8000 | 25.0000 | 1.4217 | 1.0000 | 0.0000 |
| SE_N5_2002 | 2002 | SE | SE_BEFORE | 0.2661 | 0.1097 | 15.7559 | 0.3036 | 0.3918 | 0.8120 | 0.8217 | 5.7059 | 0.6723 | 4.8824 | 0.2882 | 12.2553 | 0.9031 | 0.8140 | 0.1744 |
| SE_O1_2002 | 2002 | SE | SE_BEFORE | 0.6526 | 0.1928 | 20.1815 | 0.3994 | 0.3869 | 0.8003 | 0.8438 | 5.5588 | 0.6513 | 6.2941 | 0.4294 | 20.3176 | 2.6311 | 0.8056 | 0.1389 |
| SE_O2_2002 | 2002 | SE | SE_BEFORE | 0.4648 | 0.1787 | 18.5862 | 0.3858 | 0.3485 | 0.7100 | 0.7216 | 6.4396 | 0.7771 | 7.7912 | 0.5791 | 19.5734 | 1.1887 | 0.4505 | 0.5495 |
| SE_O3_2002 | 2002 | SE | SE_BEFORE | 0.3333 | 0.1360 | 23.1067 | 0.4481 | 0.3207 | 0.6447 | 0.6667 | 6.3333 | 0.7619 | 8.3333 | 0.6333 | 18.3533 | 2.5873 | 0.6667 | 0.3333 |
| SE_O4_2002 | 2002 | SE | SE_BEFORE | 0.7846 | 0.2740 | 14.0349 | 0.3083 | 0.4303 | 0.9027 | 0.9872 | 7.0000 | 0.8571 | 9.8846 | 0.7885 | 24.5269 | 1.3926 | 0.9615 | 0.0385 |

| | | | | | | | | | | | | | | | | | | |
|---|---|---|---|---|---|---|---|---|---|---|---|---|---|---|---|---|---|---|
| SE_O5_2002 | 2002 | SE | SE_BEFORE | 0.5165 | 0.1946 | 20.6814 | 0.4045 | 0.3872 | 0.8012 | 0.7500 | 7.0000 | 0.8571 | 7.3000 | 0.5300 | 18.1012 | 1.0100 | 0.2500 | 0.6000 |
| SE_P1_2002 | 2002 | SE | SE_BEFORE | 0.6769 | 0.2392 | 14.6949 | 0.3027 | 0.4258 | 0.8920 | 0.9744 | 6.6667 | 0.8095 | 8.6154 | 0.6615 | 23.0621 | 1.3944 | 0.9231 | 0.0000 |
| SE_P2_2002 | 2002 | SE | SE_BEFORE | 0.2565 | 0.1036 | 22.9945 | 0.3996 | 0.3600 | 0.7371 | 0.6667 | 5.0000 | 0.5714 | 4.0000 | 0.2000 | 20.5293 | 1.5592 | 0.6667 | 0.0000 |
| SE_P4_2002 | 2002 | SE | SE_BEFORE | NA | NA | NA | NA | NA | NA | NA | NA | NA | NA | NA | NA | NA | NA | NA |
| SE_P5_2002 | 2002 | SE | SE_BEFORE | 0.2839 | 0.1150 | 28.5632 | 0.4688 | 0.2441 | 0.4644 | 0.6581 | 5.5714 | 0.6531 | 6.7021 | 0.4702 | 19.2961 | 1.1240 | 0.7436 | 0.2137 |
| ST_A1_2010 | 2010 | ST | ST_REWET | 0.6169 | 0.2156 | 23.3952 | 0.4430 | 0.3184 | 0.6392 | 0.7447 | 6.2021 | 0.7432 | 9.0957 | 0.7096 | 21.8909 | 3.0369 | 1.0000 | 0.0000 |
| ST_A1a_2010 | 2010 | ST | ST_REWET | 0.3960 | 0.1540 | 22.7488 | 0.4389 | 0.3177 | 0.6375 | 0.7285 | 6.1550 | 0.7364 | 9.1672 | 0.7167 | 21.8632 | 3.1309 | 1.0000 | 0.0000 |
| ST_A1b_2010 | 2010 | ST | ST_REWET | 0.7295 | 0.2276 | 23.1662 | 0.4385 | 0.2978 | 0.5906 | 0.6957 | 5.7843 | 0.6835 | 9.0850 | 0.7085 | 21.1101 | 2.8861 | 0.9585 | 0.0415 |
| ST_A2_2010 | 2010 | ST | ST_REWET | 0.3062 | 0.1211 | 23.9205 | 0.4531 | 0.3378 | 0.6849 | 0.8606 | 6.4957 | 0.7851 | 7.7421 | 0.5742 | 16.3197 | 2.1138 | 1.0000 | 0.0000 |
| ST_A2a_2010 | 2010 | ST | ST_REWET | 0.7212 | 0.2140 | 18.4753 | 0.3760 | 0.4012 | 0.8342 | 0.8786 | 6.1654 | 0.7379 | 8.5940 | 0.6594 | 19.6771 | 1.2319 | 1.0000 | 0.0000 |
| ST_A2b_2010 | 2010 | ST | ST_REWET | 0.4575 | 0.1746 | 19.5119 | 0.3882 | 0.2828 | 0.5555 | 0.7006 | 5.4746 | 0.6392 | 9.0678 | 0.7068 | 17.9970 | 2.5117 | 0.9661 | 0.0339 |
| ST_A3_2010 | 2010 | ST | ST_REWET | 0.6621 | 0.2340 | 12.1930 | 0.2629 | 0.4073 | 0.8485 | 1.0000 | 6.2230 | 0.7461 | 9.6115 | 0.7612 | 22.7712 | 1.3820 | 1.0000 | 0.0000 |
| ST_A3a_2010 | 2010 | ST | ST_REWET | 0.5176 | 0.1883 | 10.7886 | 0.2265 | 0.3849 | 0.7957 | 1.0000 | 5.4898 | 0.6414 | 9.5102 | 0.7510 | 22.3143 | 1.4331 | 1.0000 | 0.0000 |
| ST_A3b_2010 | 2010 | ST | ST_REWET | 0.5869 | 0.2116 | 12.8804 | 0.2772 | 0.3807 | 0.7858 | 0.9048 | 5.9524 | 0.7075 | 8.5714 | 0.6571 | 19.6220 | 1.6387 | 1.0000 | 0.0000 |
| ST_A4_2010 | 2010 | ST | ST_REWET | 0.5656 | 0.2061 | 13.2418 | 0.2847 | 0.3514 | 0.7169 | 0.8519 | 5.6667 | 0.6667 | 8.6667 | 0.6667 | 18.7379 | 1.8061 | 1.0000 | 0.0000 |
| ST_A4a_2010 | 2010 | ST | ST_REWET | 0.6501 | 0.2355 | 15.6620 | 0.3340 | 0.3050 | 0.6077 | 0.7748 | 5.6757 | 0.6680 | 9.9730 | 0.7973 | 20.1148 | 2.0957 | 1.0000 | 0.0000 |
| ST_A4b_2010 | 2010 | ST | ST_REWET | 0.1579 | 0.0693 | 9.1851 | 0.1802 | 0.4368 | 0.9179 | 1.0000 | 5.0000 | 0.5714 | 4.0000 | 0.2000 | 11.6596 | 1.1289 | 1.0000 | 0.0000 |
| ST_A5_2010 | 2010 | ST | ST_REWET | 0.6645 | 0.2388 | 11.3481 | 0.2473 | 0.3868 | 0.8002 | 0.7849 | 6.2903 | 0.7558 | 9.3548 | 0.7355 | 18.2613 | 1.1699 | 1.0000 | 0.0000 |
| ST_A5a_2010 | 2010 | ST | ST_REWET | 0.6753 | 0.2398 | 12.5983 | 0.2694 | 0.3895 | 0.8066 | 0.8235 | 6.3208 | 0.7601 | 9.4528 | 0.7453 | 20.4548 | 1.1991 | 1.0000 | 0.0000 |
| ST_A5b_2010 | 2010 | ST | ST_REWET | 0.6250 | 0.2277 | 11.6704 | 0.2546 | 0.3947 | 0.8187 | 0.8108 | 6.4324 | 0.7761 | 9.4324 | 0.7432 | 19.5338 | 1.1451 | 1.0000 | 0.0000 |
| ST_B1_2010 | 2010 | ST | ST_REWET | 0.4177 | 0.1627 | 24.9621 | 0.4664 | 0.2679 | 0.5204 | 0.6851 | 5.5853 | 0.6550 | 9.0184 | 0.7018 | 23.8973 | 2.5406 | 0.9819 | 0.0181 |
| ST_B1a_2010 | 2010 | ST | ST_REWET | 1.1598 | 0.2545 | 22.0501 | 0.4218 | 0.2985 | 0.5923 | 0.6799 | 5.5906 | 0.6558 | 8.4678 | 0.6468 | 21.4227 | 3.0099 | 0.9372 | 0.0628 |
| ST_B1b_2010 | 2010 | ST | ST_REWET | 0.5690 | 0.2014 | 23.5174 | 0.4395 | 0.2529 | 0.4850 | 0.7071 | 5.5152 | 0.6450 | 9.0606 | 0.7061 | 22.8398 | 2.3764 | 0.9714 | 0.0286 |
| ST_B2_2010 | 2010 | ST | ST_REWET | 0.7568 | 0.2488 | 13.9430 | 0.2886 | 0.3578 | 0.7320 | 0.7701 | 5.5385 | 0.6484 | 9.2051 | 0.7205 | 21.4308 | 1.6296 | 1.0000 | 0.0000 |
| ST_B2a_2010 | 2010 | ST | ST_REWET | 0.8519 | 0.2719 | 14.5300 | 0.2909 | 0.3556 | 0.7267 | 0.7702 | 5.2387 | 0.6055 | 9.1419 | 0.7142 | 21.3871 | 1.0824 | 1.0000 | 0.0000 |
| ST_B2b_2010 | 2010 | ST | ST_REWET | 0.8100 | 0.2812 | 12.5714 | 0.2764 | 0.4113 | 0.8578 | 0.8667 | 6.6000 | 0.8000 | 9.6000 | 0.7600 | 21.7000 | 1.4730 | 1.0000 | 0.0000 |
| ST_B3_2010 | 2010 | ST | ST_REWET | 0.5012 | 0.1910 | 24.9181 | 0.4645 | 0.2569 | 0.4944 | 0.7132 | 5.2907 | 0.6130 | 9.1279 | 0.7128 | 26.3103 | 1.6490 | 1.0000 | 0.0000 |
| ST_B3a_2010 | 2010 | ST | ST_REWET | 0.5545 | 0.2058 | 17.9728 | 0.3707 | 0.3640 | 0.7466 | 0.7879 | 5.7273 | 0.6753 | 8.9091 | 0.6909 | 25.6000 | 1.5145 | 1.0000 | 0.0000 |
| ST_B3b_2010 | 2010 | ST | ST_REWET | 0.6650 | 0.2389 | 18.4676 | 0.3665 | 0.3384 | 0.6863 | 0.8000 | 6.0000 | 0.7143 | 9.4000 | 0.7400 | 24.0700 | 1.5587 | 1.0000 | 0.0000 |
| ST_B4_2010 | 2010 | ST | ST_REWET | 0.7714 | 0.2691 | 13.4064 | 0.2944 | 0.4301 | 0.9022 | 0.9848 | 6.8636 | 0.8377 | 9.6818 | 0.7682 | 23.9795 | 1.4132 | 1.0000 | 0.0000 |
| ST_B4a_2010 | 2010 | ST | ST_REWET | 0.8036 | 0.2795 | 13.3735 | 0.2945 | 0.4248 | 0.8897 | 0.9524 | 6.8571 | 0.8367 | 9.8571 | 0.7857 | 23.8214 | 1.4400 | 1.0000 | 0.0000 |
| ST_B4b_2010 | 2010 | ST | ST_REWET | 0.8000 | 0.2785 | 13.8191 | 0.3046 | 0.4324 | 0.9074 | 1.0000 | 7.0000 | 0.8571 | 10.0000 | 0.8000 | 25.0000 | 1.4217 | 1.0000 | 0.0000 |
| ST_B5_2010 | 2010 | ST | ST_REWET | 0.4458 | 0.1695 | 15.5000 | 0.3145 | 0.2984 | 0.5922 | 0.6667 | 5.6000 | 0.6571 | 9.0000 | 0.7000 | 19.1882 | 1.9515 | 1.0000 | 0.0000 |
| ST_B5a_2010 | 2010 | ST | ST_REWET | 0.7013 | 0.2499 | 12.6011 | 0.2763 | 0.4110 | 0.8571 | 0.8927 | 6.6780 | 0.8111 | 9.6780 | 0.7678 | 21.9008 | 1.2658 | 1.0000 | 0.0000 |
| ST_B5b_2010 | 2010 | ST | ST_REWET | 0.8250 | 0.2851 | 10.7000 | 0.2340 | 0.3797 | 0.7835 | 0.6667 | 6.0000 | 0.7143 | 9.0000 | 0.7000 | 16.7500 | 1.5500 | 1.0000 | 0.0000 |
| ST_C1_2010 | 2010 | ST | ST_REWET | 0.3474 | 0.1374 | 23.1861 | 0.4284 | 0.2877 | 0.5669 | 0.7274 | 5.5890 | 0.6556 | 9.0720 | 0.7072 | 21.2597 | 2.9954 | 1.0000 | 0.0000 |
| ST_C1a_2010 | 2010 | ST | ST_REWET | 0.4143 | 0.1591 | 22.4651 | 0.4244 | 0.3143 | 0.6294 | 0.7305 | 5.9569 | 0.7081 | 9.1019 | 0.7102 | 21.1795 | 2.8981 | 1.0000 | 0.0000 |
| ST_C1b_2010 | 2010 | ST | ST_REWET | 0.3791 | 0.1493 | 24.2213 | 0.4495 | 0.2868 | 0.5648 | 0.7012 | 5.7387 | 0.6770 | 9.0495 | 0.7050 | 21.5989 | 2.9996 | 1.0000 | 0.0000 |
| ST_C2_2010 | 2010 | ST | ST_REWET | 0.6232 | 0.2062 | 22.3020 | 0.4358 | 0.3360 | 0.6806 | 1.0000 | 5.2759 | 0.6108 | 9.1579 | 0.7158 | 22.0850 | 1.1574 | 1.0000 | 0.0000 |
| ST_C2a_2010 | 2010 | ST | ST_REWET | 0.6337 | 0.2208 | 17.7605 | 0.3662 | 0.3873 | 0.8014 | 0.9677 | 6.3871 | 0.7696 | 9.6452 | 0.7645 | 24.5017 | 1.6991 | 1.0000 | 0.0000 |
| ST_C2b_2010 | 2010 | ST | ST_REWET | 0.6096 | 0.2131 | 19.8732 | 0.4002 | 0.3289 | 0.6638 | 0.8546 | 6.0106 | 0.7158 | 9.3011 | 0.7301 | 24.8086 | 1.4583 | 1.0000 | 0.0000 |
| ST_C3_2010 | 2010 | ST | ST_REWET | 0.5510 | 0.2020 | 18.8716 | 0.3738 | 0.3644 | 0.7475 | 0.8263 | 6.1018 | 0.7288 | 9.1761 | 0.7176 | 22.9860 | 1.8619 | 1.0000 | 0.0000 |
| ST_C3a_2010 | 2010 | ST | ST_REWET | 0.5620 | 0.2081 | 13.7486 | 0.2897 | 0.3804 | 0.7850 | 0.7826 | 6.1304 | 0.7329 | 9.2174 | 0.7217 | 20.0606 | 1.2423 | 1.0000 | 0.0000 |
| ST_C3b_2010 | 2010 | ST | ST_REWET | 0.6756 | 0.2395 | 18.1757 | 0.3595 | 0.3791 | 0.7821 | 0.9236 | 6.4514 | 0.7788 | 9.7059 | 0.7706 | 24.7596 | 1.4084 | 1.0000 | 0.0000 |
| ST_C4_2010 | 2010 | ST | ST_REWET | 0.6585 | 0.2302 | 14.9690 | 0.3117 | 0.3869 | 0.8003 | 0.9031 | 5.9573 | 0.7082 | 8.9655 | 0.6966 | 21.3649 | 1.4080 | 0.8669 | 0.1331 |
| ST_C4a_2010 | 2010 | ST | ST_REWET | 0.6136 | 0.2174 | 14.9989 | 0.3071 | 0.3997 | 0.8306 | 0.9564 | 6.2710 | 0.7530 | 8.6038 | 0.6604 | 21.6859 | 1.4105 | 1.0000 | 0.0000 |
| ST_C4b_2010 | 2010 | ST | ST_REWET | 0.6003 | 0.2162 | 17.5361 | 0.3631 | 0.3711 | 0.7631 | 0.7436 | 5.5455 | 0.6494 | 8.5775 | 0.6577 | 21.7508 | 1.3886 | 1.0000 | 0.0000 |
| ST_C5_2010 | 2010 | ST | ST_REWET | 0.6377 | 0.2256 | 17.7266 | 0.3523 | 0.3590 | 0.7348 | 0.7658 | 5.6892 | 0.6699 | 8.9452 | 0.6945 | 20.8321 | 1.4532 | 1.0000 | 0.0000 |
| ST_C5a_2010 | 2010 | ST | ST_REWET | 0.5002 | 0.1871 | 18.1559 | 0.3678 | 0.3619 | 0.7416 | 0.7395 | 5.5077 | 0.6440 | 8.6408 | 0.6641 | 22.1316 | 1.6889 | 1.0000 | 0.0000 |
| ST_C5b_2010 | 2010 | ST | ST_REWET | 0.7029 | 0.2441 | 17.5642 | 0.3469 | 0.3674 | 0.7545 | 0.8582 | 6.1469 | 0.7353 | 9.4605 | 0.7461 | 22.3925 | 1.5247 | 1.0000 | 0.0000 |
| ST_D1_2010 | 2010 | ST | ST_REWET | 1.1863 | 0.3173 | 16.8790 | 0.3493 | 0.3679 | 0.7556 | 0.8414 | 5.5631 | 0.6519 | 8.6757 | 0.6676 | 23.2986 | 1.5855 | 1.0000 | 0.0000 |

| | | | | | | | | | | | | | | | | | | |
|---|---|---|---|---|---|---|---|---|---|---|---|---|---|---|---|---|---|---|
| ST_D1a_2010 | 2010 | ST | ST_REWET | 0.3961 | 0.1427 | 20.0947 | 0.4017 | 0.3614 | 0.7404 | 0.9232 | 5.7853 | 0.6836 | 9.2000 | 0.7200 | 23.0987 | 1.6935 | 1.0000 | 0.0000 |
| ST_D1b_2010 | 2010 | ST | ST_REWET | 0.5179 | 0.1861 | 22.8558 | 0.4260 | 0.3174 | 0.6369 | 0.8026 | 5.8628 | 0.6947 | 9.2780 | 0.7278 | 22.8790 | 1.8523 | 1.0000 | 0.0000 |
| ST_D2_2010 | 2010 | ST | ST_REWET | 0.5799 | 0.2094 | 20.7913 | 0.4018 | 0.3399 | 0.6898 | 0.8116 | 6.2298 | 0.7471 | 9.3913 | 0.7391 | 22.8812 | 2.2100 | 1.0000 | 0.0000 |
| ST_D2a_2010 | 2010 | ST | ST_REWET | 0.3524 | 0.1315 | 25.0682 | 0.4590 | 0.2962 | 0.5870 | 0.7924 | 5.4706 | 0.6387 | 9.1626 | 0.7163 | 22.9044 | 2.5815 | 1.0000 | 0.0000 |
| ST_D2b_2010 | 2010 | ST | ST_REWET | 0.5063 | 0.1834 | 19.2623 | 0.3893 | 0.3447 | 0.7012 | 0.8633 | 6.0415 | 0.7202 | 9.3134 | 0.7313 | 23.6532 | 1.7685 | 1.0000 | 0.0000 |
| ST_D3_2010 | 2010 | ST | ST_REWET | 0.4138 | 0.1516 | 24.8336 | 0.4530 | 0.3091 | 0.6174 | 0.7778 | 5.5161 | 0.6452 | 9.0115 | 0.7011 | 20.3933 | 2.2552 | 1.0000 | 0.0000 |
| ST_D3a_2010 | 2010 | ST | ST_REWET | 0.6731 | 0.2375 | 17.2012 | 0.3483 | 0.3630 | 0.7441 | 0.8312 | 6.3450 | 0.7636 | 9.5242 | 0.7524 | 22.7679 | 1.7686 | 0.9905 | 0.0095 |
| ST_D3b_2010 | 2010 | ST | ST_REWET | 0.6058 | 0.2096 | 23.4624 | 0.4236 | 0.3008 | 0.5977 | 0.7076 | 5.2105 | 0.6015 | 8.8545 | 0.6855 | 17.4229 | 1.4876 | 1.0000 | 0.0000 |
| ST_D4_2010 | 2010 | ST | ST_REWET | 0.4096 | 0.1511 | 24.8623 | 0.4439 | 0.3038 | 0.6049 | 0.8041 | 5.3947 | 0.6278 | 9.2385 | 0.7239 | 21.7711 | 1.5430 | 1.0000 | 0.0000 |
| ST_D4a_2010 | 2010 | ST | ST_REWET | 0.4075 | 0.1491 | 22.7735 | 0.4212 | 0.3313 | 0.6696 | 0.8820 | 5.5528 | 0.6504 | 9.3145 | 0.7314 | 23.2502 | 1.4125 | 1.0000 | 0.0000 |
| ST_D4b_2010 | 2010 | ST | ST_REWET | 0.6325 | 0.2205 | 19.7593 | 0.3851 | 0.3554 | 0.7264 | 0.8841 | 6.1630 | 0.7376 | 9.4348 | 0.7435 | 23.2983 | 1.5807 | 1.0000 | 0.0000 |
| ST_D5_2010 | 2010 | ST | ST_REWET | 0.8197 | 0.2820 | 15.6658 | 0.3293 | 0.3935 | 0.8160 | 0.9098 | 6.7176 | 0.8168 | 9.7294 | 0.7729 | 23.7443 | 1.4806 | 1.0000 | 0.0000 |
| ST_D5a_2010 | 2010 | ST | ST_REWET | 0.6289 | 0.2252 | 16.7024 | 0.3471 | 0.4131 | 0.8620 | 0.8599 | 6.2826 | 0.7547 | 9.2899 | 0.7290 | 21.5638 | 1.3290 | 1.0000 | 0.0000 |
| ST_D5b_2010 | 2010 | ST | ST_REWET | 0.7096 | 0.2506 | 18.6223 | 0.3742 | 0.3451 | 0.7021 | 0.8211 | 6.1870 | 0.7410 | 9.5203 | 0.7520 | 24.0534 | 1.4983 | 1.0000 | 0.0000 |
| ST_E1_2002 | 2002 | ST | ST_BEFORE | 0.2199 | 0.0937 | 8.2340 | 0.1553 | 0.3405 | 0.6913 | 0.9967 | 4.0294 | 0.4328 | 9.0000 | 0.7000 | 20.4529 | 1.4868 | 1.0000 | 0.0000 |
| ST_E2_2002 | 2002 | ST | ST_BEFORE | 0.2078 | 0.0859 | 21.1147 | 0.4033 | 0.3639 | 0.7463 | 0.9789 | 6.4121 | 0.7732 | 6.8427 | 0.4843 | 13.6916 | 1.3011 | 1.0000 | 0.0000 |
| ST_E3_2002 | 2002 | ST | ST_BEFORE | 0.3250 | 0.1218 | 11.5014 | 0.2012 | 0.3919 | 0.8122 | 0.9130 | 5.2609 | 0.6087 | 5.4286 | 0.3429 | 15.4758 | 1.1207 | 1.0000 | 0.0000 |
| ST_E4_2002 | 2002 | ST | ST_BEFORE | 0.4991 | 0.1788 | 13.0678 | 0.2498 | 0.3929 | 0.8145 | 0.8824 | 5.8235 | 0.6891 | 7.3333 | 0.5333 | 19.7172 | 1.2771 | 1.0000 | 0.0000 |
| ST_E5_2002 | 2002 | ST | ST_BEFORE | 0.1000 | 0.0452 | 21.8400 | 0.4339 | 0.3481 | 0.7091 | 1.0000 | 5.0000 | 0.5714 | 9.0000 | 0.7000 | NA | NA | 1.0000 | 0.0000 |
| ST_F1_2002 | 2002 | ST | ST_BEFORE | 0.1635 | 0.0715 | 10.4632 | 0.1952 | 0.3849 | 0.7957 | 0.9275 | 4.6739 | 0.5248 | 5.8750 | 0.3875 | 15.4315 | 1.2954 | 1.0000 | 0.0000 |
| ST_F2_2002 | 2002 | ST | ST_BEFORE | 0.2802 | 0.1101 | 18.9982 | 0.3698 | 0.3773 | 0.7779 | 0.9336 | 6.0359 | 0.7194 | 6.9268 | 0.4927 | 14.1677 | 1.3240 | 1.0000 | 0.0000 |
| ST_F3_2002 | 2002 | ST | ST_BEFORE | 0.1866 | 0.0779 | 22.8624 | 0.4347 | 0.3686 | 0.7574 | 0.9535 | 6.6932 | 0.8133 | 6.0638 | 0.4064 | 12.0235 | 1.2620 | 1.0000 | 0.0000 |
| ST_F4_2002 | 2002 | ST | ST_BEFORE | 0.3100 | 0.1242 | 8.9297 | 0.1833 | 0.4007 | 0.8328 | 0.8333 | 5.5000 | 0.6429 | 6.5000 | 0.4500 | 13.0500 | 1.0043 | 1.0000 | 0.0000 |
| ST_F5_2002 | 2002 | ST | ST_BEFORE | NA | NA | NA | NA | NA | NA | NA | NA | NA | NA | NA | NA | NA | NA | NA |
| ST_G1_2002 | 2002 | ST | ST_BEFORE | 0.1827 | 0.0789 | 22.5546 | 0.4370 | 0.3852 | 0.7965 | 1.0000 | 6.3478 | 0.7640 | 6.1672 | 0.4167 | 12.9485 | 1.2542 | 1.0000 | 0.0000 |
| ST_G2_2002 | 2002 | ST | ST_BEFORE | 0.1498 | 0.0662 | 24.2937 | 0.4599 | 0.3670 | 0.7535 | 1.0000 | 6.9277 | 0.8468 | 5.9277 | 0.3928 | 10.5590 | 1.2183 | 1.0000 | 0.0000 |
| ST_G3_2002 | 2002 | ST | ST_BEFORE | 0.8134 | 0.2511 | 14.9602 | 0.3031 | 0.3771 | 0.7774 | 1.0000 | 5.3784 | 0.6255 | 7.7681 | 0.5768 | 19.1432 | 1.1608 | 1.0000 | 0.0000 |
| ST_G4_2002 | 2002 | ST | ST_BEFORE | 0.3667 | 0.1370 | 21.2130 | 0.4159 | 0.3870 | 0.8007 | 1.0000 | 7.0000 | 0.8571 | 7.3333 | 0.5333 | 15.3667 | 1.2889 | 1.0000 | 0.0000 |
| ST_G5_2002 | 2002 | ST | ST_BEFORE | 0.4779 | 0.1733 | 19.2929 | 0.3868 | 0.3982 | 0.8270 | 0.9972 | 6.9917 | 0.8560 | 8.0083 | 0.6008 | 17.7665 | 1.3240 | 1.0000 | 0.0000 |
| ST_H1_2002 | 2002 | ST | ST_BEFORE | 0.1500 | 0.0662 | 24.9100 | 0.4715 | 0.3644 | 0.7474 | 1.0000 | 7.0000 | 0.8571 | 6.0000 | 0.4000 | 10.5500 | 1.2225 | 1.0000 | 0.0000 |
| ST_H2_2002 | 2002 | ST | ST_BEFORE | 0.1500 | 0.0662 | 24.9100 | 0.4715 | 0.3644 | 0.7474 | 1.0000 | 7.0000 | 0.8571 | 6.0000 | 0.4000 | 10.5500 | 1.2225 | 1.0000 | 0.0000 |
| ST_H3_2002 | 2002 | ST | ST_BEFORE | 0.6129 | 0.2173 | 12.1163 | 0.2607 | 0.4334 | 0.9099 | 1.0000 | 6.4286 | 0.7755 | 8.2857 | 0.6286 | 20.9429 | 1.3312 | 1.0000 | 0.0000 |
| ST_H4_2002 | 2002 | ST | ST_BEFORE | 0.2150 | 0.0923 | 7.8159 | 0.1495 | 0.3420 | 0.6947 | 1.0000 | 4.0000 | 0.4286 | 9.0000 | 0.7000 | 20.5000 | 1.5000 | 1.0000 | 0.0000 |
| ST_H5_2002 | 2002 | ST | ST_BEFORE | 0.1500 | 0.0662 | 24.9100 | 0.4715 | 0.3644 | 0.7474 | 1.0000 | 7.0000 | 0.8571 | 6.0000 | 0.4000 | 10.5500 | 1.2225 | 1.0000 | 0.0000 |
| ST_I1_2010 | 2010 | ST | ST_REWET | 0.4772 | 0.1776 | 18.5069 | 0.3784 | 0.3213 | 0.6461 | 0.8421 | 6.0526 | 0.7218 | 8.5965 | 0.6596 | 16.6189 | 1.8365 | 1.0000 | 0.0000 |
| ST_I2_2010 | 2010 | ST | ST_REWET | 0.2891 | 0.1145 | 18.8978 | 0.3615 | 0.3333 | 0.6743 | 0.8623 | 5.7438 | 0.6777 | 8.1322 | 0.6132 | 17.3899 | 2.1110 | 1.0000 | 0.0000 |
| ST_I3_2010 | 2010 | ST | ST_REWET | 0.3916 | 0.1430 | 19.8700 | 0.3935 | 0.3944 | 0.8181 | 0.9968 | 6.5421 | 0.7917 | 7.2796 | 0.5280 | 15.8874 | 1.2751 | 1.0000 | 0.0000 |
| ST_I4_2010 | 2010 | ST | ST_REWET | 0.1889 | 0.0809 | 29.9594 | 0.5134 | 0.2930 | 0.5793 | 0.8889 | 6.2222 | 0.7460 | 7.2857 | 0.5286 | 16.0711 | 1.7482 | 1.0000 | 0.0000 |
| ST_I5_2010 | 2010 | ST | ST_REWET | NA | NA | NA | NA | NA | NA | NA | NA | NA | NA | NA | NA | NA | NA | NA |
| ST_J1_2010 | 2010 | ST | ST_REWET | 0.6763 | 0.2425 | 21.4322 | 0.3657 | 0.2818 | 0.5532 | 0.6667 | 5.4684 | 0.6383 | 9.1266 | 0.7127 | 23.9812 | 1.7021 | 1.0000 | 0.0000 |
| ST_J2_2010 | 2010 | ST | ST_REWET | 0.5843 | 0.2112 | 25.4264 | 0.4515 | 0.2981 | 0.5914 | 0.7319 | 5.9710 | 0.7101 | 9.1838 | 0.7184 | 22.1671 | 2.1560 | 1.0000 | 0.0000 |
| ST_J3_2010 | 2010 | ST | ST_REWET | 0.6735 | 0.2377 | 15.9589 | 0.3121 | 0.3636 | 0.7457 | 0.7440 | 6.0536 | 0.7219 | 7.8200 | 0.5820 | 22.8061 | 2.0283 | 0.7500 | 0.2500 |
| ST_J4_2010 | 2010 | ST | ST_REWET | 0.6257 | 0.2242 | 18.1067 | 0.3653 | 0.3769 | 0.7770 | 0.8806 | 6.5461 | 0.7923 | 9.6441 | 0.7644 | 23.6282 | 2.0582 | 1.0000 | 0.0000 |
| ST_J5_2010 | 2010 | ST | ST_REWET | 0.1120 | 0.0503 | 38.7160 | 0.5974 | 0.1735 | 0.2982 | 0.7333 | 4.2000 | 0.4571 | 9.0000 | 0.7000 | 19.4500 | 0.1000 | 1.0000 | 0.0000 |
| ST_K1_2010 | 2010 | ST | ST_REWET | 0.3915 | 0.1471 | 18.2444 | 0.3675 | 0.3182 | 0.6388 | 0.8685 | 5.2441 | 0.6063 | 9.4641 | 0.7464 | 19.9096 | 2.0554 | 1.0000 | 0.0000 |
| ST_K2_2010 | 2010 | ST | ST_REWET | 0.3614 | 0.1348 | 22.1561 | 0.4104 | 0.3159 | 0.6334 | 0.8063 | 5.4874 | 0.6411 | 9.1208 | 0.7121 | 19.2514 | 1.9257 | 1.0000 | 0.0000 |
| ST_K3_2010 | 2010 | ST | ST_REWET | 0.7309 | 0.2295 | 18.4720 | 0.3576 | 0.3355 | 0.6794 | 0.7506 | 5.1121 | 0.5874 | 9.1297 | 0.7130 | 23.4062 | 1.5645 | 1.0000 | 0.0000 |
| ST_K4_2010 | 2010 | ST | ST_REWET | 0.3499 | 0.1346 | 15.2889 | 0.2908 | 0.3682 | 0.7565 | 0.8119 | 5.7430 | 0.6776 | 7.3669 | 0.5367 | 15.0524 | 1.1874 | 1.0000 | 0.0000 |
| ST_K5_2010 | 2010 | ST | ST_REWET | 0.9890 | 0.3040 | 13.5577 | 0.2820 | 0.3780 | 0.7794 | 0.9404 | 5.4629 | 0.6376 | 8.7600 | 0.6760 | 21.5726 | 1.1542 | 1.0000 | 0.0000 |
| ST_L1_2010 | 2010 | ST | ST_REWET | 0.5234 | 0.1922 | 23.1665 | 0.4208 | 0.3122 | 0.6246 | 0.7481 | 5.8034 | 0.6862 | 8.3831 | 0.6383 | 28.5422 | 1.7203 | 1.0000 | 0.0000 |
| ST_L2_2010 | 2010 | ST | ST_REWET | 0.6299 | 0.2269 | 15.5681 | 0.3157 | 0.3790 | 0.7818 | 0.8560 | 6.0041 | 0.7149 | 8.4606 | 0.6461 | 20.4801 | 1.5236 | 1.0000 | 0.0000 |

| | | | | | | | | | | | | | | | | | | |
|---|---|---|---|---|---|---|---|---|---|---|---|---|---|---|---|---|---|---|
| ST_L3_2010 | 2010 | ST | ST_REWET | 0.7609 | 0.2653 | 12.6552 | 0.2770 | 0.4199 | 0.8781 | 0.9195 | 6.6207 | 0.8030 | 9.3448 | 0.7345 | 22.0293 | 1.4308 | 1.0000 | 0.0000 |
| ST_L4_2010 | 2010 | ST | ST_REWET | 0.6691 | 0.2355 | 12.8484 | 0.2779 | 0.4276 | 0.8961 | 0.9810 | 6.5429 | 0.7918 | 8.8857 | 0.6886 | 22.0088 | 1.3734 | 1.0000 | 0.0000 |
| ST_L5_2010 | 2010 | ST | ST_REWET | 0.2781 | 0.1019 | 14.2938 | 0.2860 | 0.3817 | 0.7883 | 0.9460 | 5.0796 | 0.5828 | 7.2301 | 0.5230 | 13.7469 | 1.0494 | 1.0000 | 0.0000 |
| ST_M1_2002 | 2002 | ST | ST_BEFORE | 0.1571 | 0.0685 | 24.7379 | 0.4687 | 0.3644 | 0.7476 | 0.9964 | 6.9781 | 0.8540 | 6.0492 | 0.4049 | 10.6181 | 1.2261 | 1.0000 | 0.0000 |
| ST_M2_2002 | 2002 | ST | ST_BEFORE | 0.2459 | 0.0981 | 21.7492 | 0.4188 | 0.3751 | 0.7727 | 0.9907 | 6.6978 | 0.8140 | 6.5810 | 0.4581 | 13.0649 | 1.2519 | 1.0000 | 0.0000 |
| ST_M3_2002 | 2002 | ST | ST_BEFORE | 0.4177 | 0.1375 | 12.6624 | 0.2249 | 0.3787 | 0.7812 | 1.0000 | 4.7805 | 0.5401 | 5.5714 | 0.3571 | 15.7207 | 1.0355 | 1.0000 | 0.0000 |
| ST_M4_2002 | 2002 | ST | ST_BEFORE | 0.7906 | 0.2412 | 9.3341 | 0.1941 | 0.3560 | 0.7277 | 0.9333 | 4.1111 | 0.4444 | 8.4444 | 0.6444 | 21.5375 | 1.2006 | 1.0000 | 0.0000 |
| ST_M5_2002 | 2002 | ST | ST_BEFORE | 0.1450 | 0.0642 | 7.8595 | 0.1510 | 0.4361 | 0.9162 | 1.0000 | 5.0000 | 0.5714 | 4.0000 | 0.2000 | 10.8000 | 1.1050 | 1.0000 | 0.0000 |
| ST_N1_2002 | 2002 | ST | ST_BEFORE | 0.1504 | 0.0664 | 24.8073 | 0.4697 | 0.3650 | 0.7489 | 1.0000 | 6.9824 | 0.8546 | 5.9912 | 0.3991 | 10.5830 | 1.2223 | 1.0000 | 0.0000 |
| ST_N2_2002 | 2002 | ST | ST_BEFORE | 0.1551 | 0.0682 | 24.4483 | 0.4644 | 0.3681 | 0.7563 | 0.9993 | 6.8908 | 0.8415 | 5.9868 | 0.3987 | 10.8729 | 1.2271 | 1.0000 | 0.0000 |
| ST_N3_2002 | 2002 | ST | ST_BEFORE | 0.2664 | 0.0997 | 20.9722 | 0.4018 | 0.3653 | 0.7497 | 1.0000 | 6.2143 | 0.7449 | 6.6923 | 0.4692 | 13.5214 | 1.2518 | 1.0000 | 0.0000 |
| ST_N4_2002 | 2002 | ST | ST_BEFORE | 0.1668 | 0.0728 | 13.2058 | 0.2861 | 0.3071 | 0.6127 | 0.3922 | 5.0588 | 0.5798 | 4.3125 | 0.2313 | 15.0104 | 1.1940 | 0.1176 | 0.8824 |
| ST_N5_2002 | 2002 | ST | ST_BEFORE | NA | NA | NA | NA | NA | NA | NA | NA | NA | NA | NA | NA | NA | NA | NA |
| ST_O1_2002 | 2002 | ST | ST_BEFORE | NA | NA | NA | NA | NA | NA | NA | NA | NA | NA | NA | NA | NA | NA | NA |
| ST_O2_2002 | 2002 | ST | ST_BEFORE | 0.1775 | 0.0752 | 24.0888 | 0.4574 | 0.3662 | 0.7516 | 0.9865 | 6.9272 | 0.8467 | 6.0946 | 0.4095 | 10.8236 | 1.2344 | 1.0000 | 0.0000 |
| ST_O3_2002 | 2002 | ST | ST_BEFORE | 0.2620 | 0.1028 | 22.6058 | 0.4354 | 0.3777 | 0.7788 | 1.0000 | 6.9540 | 0.8506 | 6.6437 | 0.4644 | 13.0471 | 1.2541 | 1.0000 | 0.0000 |
| ST_O4_2002 | 2002 | ST | ST_BEFORE | 0.2080 | 0.0891 | 14.3362 | 0.2938 | 0.4396 | 0.9245 | 1.0000 | 5.0000 | 0.5714 | 4.0000 | 0.2000 | 15.0000 | 1.2220 | 1.0000 | 0.0000 |
| ST_O5_2002 | 2002 | ST | ST_BEFORE | 0.1225 | 0.0547 | 27.7197 | 0.4051 | 0.2946 | 0.5832 | 1.0000 | 5.0000 | 0.5714 | 4.0000 | 0.2000 | 15.1250 | 0.6025 | 1.0000 | 0.0000 |
| ST_P1_2002 | 2002 | ST | ST_BEFORE | 0.1535 | 0.0676 | 24.6905 | 0.4686 | 0.3671 | 0.7538 | 1.0000 | 6.9298 | 0.8471 | 6.0000 | 0.4000 | 10.8044 | 1.2252 | 1.0000 | 0.0000 |
| ST_P2_2002 | 2002 | ST | ST_BEFORE | 0.1786 | 0.0775 | 23.1226 | 0.4479 | 0.3866 | 0.7996 | 1.0000 | 6.4286 | 0.7755 | 6.0000 | 0.4000 | 12.6214 | 1.2446 | 1.0000 | 0.0000 |
| ST_P3_2002 | 2002 | ST | ST_BEFORE | 0.3964 | 0.1410 | 18.7822 | 0.3709 | 0.3945 | 0.8184 | 1.0000 | 6.2262 | 0.7466 | 6.8472 | 0.4847 | 15.4000 | 1.2366 | 1.0000 | 0.0000 |
| ST_P4_2002 | 2002 | ST | ST_BEFORE | 0.2247 | 0.0887 | 23.0651 | 0.4399 | 0.3699 | 0.7603 | 0.9867 | 6.7746 | 0.8249 | 6.2533 | 0.4253 | 12.0171 | 1.2249 | 1.0000 | 0.0000 |
| ST_P5_2002 | 2002 | ST | ST_BEFORE | NA | NA | NA | NA | NA | NA | NA | NA | NA | NA | NA | NA | NA | NA | NA |
| TA_279 | 1972 | TA | TA_BEFORE | 0.4244 | 0.1612 | 23.1030 | 0.4366 | 0.2256 | 0.4209 | 0.6897 | 5.2921 | 0.6132 | 6.5181 | 0.4518 | 22.9404 | 2.0720 | 1.0000 | 0.0000 |
| TA_280 | 1972 | TA | TA_BEFORE | 0.4751 | 0.1794 | 25.7981 | 0.4650 | 0.2248 | 0.4189 | 0.6727 | 5.4518 | 0.6360 | 6.5506 | 0.4551 | 25.0441 | 2.2464 | 1.0000 | 0.0000 |
| TA_281 | 1972 | TA | TA_BEFORE | 0.8600 | 0.2718 | 21.2689 | 0.4121 | 0.2455 | 0.4677 | 0.7814 | 5.5726 | 0.6532 | 7.8393 | 0.5839 | 21.3114 | 2.1195 | 1.0000 | 0.0000 |
| TA_282 | 1972 | TA | TA_BEFORE | 0.5564 | 0.1963 | 23.9945 | 0.4448 | 0.2209 | 0.4097 | 0.7040 | 5.3394 | 0.6199 | 6.9158 | 0.4916 | 21.9634 | 2.1473 | 1.0000 | 0.0000 |
| TA_283 | 1972 | TA | TA_BEFORE | 0.7772 | 0.2602 | 18.1379 | 0.3533 | 0.2440 | 0.4642 | 0.7977 | 5.2650 | 0.6093 | 8.2136 | 0.6214 | 16.1581 | 1.6786 | 1.0000 | 0.0000 |
| TA_284 | 1972 | TA | TA_BEFORE | 0.8342 | 0.2772 | 15.7231 | 0.3091 | 0.2495 | 0.4771 | 0.8974 | 5.0692 | 0.5813 | 8.7500 | 0.6750 | 12.5406 | 1.0995 | 1.0000 | 0.0000 |
| TA_285 | 1972 | TA | TA_BEFORE | 0.6080 | 0.2164 | 22.4129 | 0.4310 | 0.2691 | 0.5231 | 0.6389 | 5.5833 | 0.6548 | 7.1373 | 0.5137 | 23.6824 | 2.2385 | 1.0000 | 0.0000 |
| TA_286 | 1972 | TA | TA_BEFORE | 0.4859 | 0.1797 | 22.7233 | 0.4327 | 0.2178 | 0.4024 | 0.7044 | 5.0660 | 0.5809 | 7.2128 | 0.5213 | 22.4831 | 2.2345 | 1.0000 | 0.0000 |
| TA_287 | 1972 | TA | TA_BEFORE | 0.5726 | 0.2060 | 20.2091 | 0.4024 | 0.2655 | 0.5148 | 0.7371 | 5.0423 | 0.5775 | 7.9839 | 0.5984 | 19.6920 | 1.7015 | 1.0000 | 0.0000 |
| TA_288 | 1972 | TA | TA_BEFORE | 0.6071 | 0.2161 | 20.5980 | 0.4050 | 0.2525 | 0.4841 | 0.7807 | 4.8584 | 0.5512 | 7.7143 | 0.5714 | 18.6526 | 1.5904 | 0.9912 | 0.0000 |
| TA_289 | 1972 | TA | TA_BEFORE | 0.6392 | 0.2256 | 20.5471 | 0.4037 | 0.2651 | 0.5137 | 0.7884 | 4.8684 | 0.5526 | 7.7619 | 0.5762 | 18.7712 | 1.6257 | 0.9913 | 0.0000 |
| TA_290 | 1972 | TA | TA_BEFORE | 0.5493 | 0.1983 | 21.3657 | 0.4166 | 0.2331 | 0.4384 | 0.7550 | 4.6333 | 0.5190 | 8.1852 | 0.6185 | 22.6561 | 2.1310 | 0.9934 | 0.0000 |
| TA_291 | 1972 | TA | TA_BEFORE | 0.5348 | 0.1957 | 23.4109 | 0.4346 | 0.2269 | 0.4238 | 0.6627 | 5.2530 | 0.6076 | 7.3733 | 0.5373 | 21.7627 | 1.7422 | 0.9765 | 0.0000 |
| TA_292 | 1972 | TA | TA_BEFORE | 0.4700 | 0.1764 | 23.2994 | 0.4374 | 0.2244 | 0.4180 | 0.6364 | 5.1944 | 0.5992 | 7.4574 | 0.5457 | 23.5952 | 2.1222 | 0.9818 | 0.0000 |
| TA_293 | 1972 | TA | TA_BEFORE | 0.5080 | 0.1874 | 23.6167 | 0.4416 | 0.2268 | 0.4237 | 0.6027 | 5.4184 | 0.6312 | 7.0370 | 0.5037 | 23.5711 | 2.1907 | 0.9899 | 0.0000 |
| TA_294 | 1972 | TA | TA_BEFORE | 0.7151 | 0.2436 | 23.6174 | 0.4474 | 0.2783 | 0.5448 | 0.6667 | 5.3545 | 0.6221 | 7.5319 | 0.5532 | 24.5377 | 2.3223 | 1.0000 | 0.0000 |
| TA_295 | 1972 | TA | TA_BEFORE | 0.9868 | 0.3067 | 21.9849 | 0.4246 | 0.2536 | 0.4868 | 0.8006 | 5.4554 | 0.6365 | 8.3048 | 0.6305 | 22.1656 | 2.3260 | 0.9821 | 0.0179 |
| TA_296 | 1972 | TA | TA_BEFORE | 0.6882 | 0.2340 | 24.1489 | 0.4525 | 0.2356 | 0.4444 | 0.6517 | 5.0787 | 0.5827 | 7.7867 | 0.5787 | 24.3623 | 2.2725 | 1.0000 | 0.0000 |
| TA_297 | 1972 | TA | TA_BEFORE | 0.9269 | 0.2904 | 23.0942 | 0.4398 | 0.2526 | 0.4845 | 0.7672 | 5.2500 | 0.6071 | 8.3143 | 0.6314 | 21.5442 | 2.2792 | 1.0000 | 0.0000 |
| TA_298 | 1972 | TA | TA_BEFORE | 0.5808 | 0.2099 | 24.9806 | 0.4633 | 0.2179 | 0.4028 | 0.7022 | 5.1067 | 0.5867 | 7.8841 | 0.5884 | 26.3836 | 2.6151 | 1.0000 | 0.0000 |
| TA_299 | 1972 | TA | TA_BEFORE | 0.7466 | 0.2529 | 21.2901 | 0.4194 | 0.2839 | 0.5580 | 0.7333 | 5.4174 | 0.6311 | 7.8447 | 0.5845 | 23.3630 | 2.1201 | 1.0000 | 0.0000 |
| TA_300 | 1972 | TA | TA_BEFORE | 0.4076 | 0.1518 | 25.8754 | 0.4758 | 0.2187 | 0.4045 | 0.7562 | 5.3605 | 0.6229 | 6.5865 | 0.4586 | 23.2332 | 2.2752 | 0.9866 | 0.0000 |
| TA_301 | 1972 | TA | TA_BEFORE | 0.4614 | 0.1707 | 24.1876 | 0.4539 | 0.2097 | 0.3835 | 0.6337 | 5.2828 | 0.6118 | 6.3294 | 0.4329 | 21.8478 | 2.2151 | 0.9802 | 0.0000 |
| TA_302 | 1972 | TA | TA_BEFORE | 0.4761 | 0.1745 | 23.1168 | 0.4406 | 0.2288 | 0.4282 | 0.6884 | 5.0326 | 0.5761 | 6.5750 | 0.4575 | 20.7983 | 2.2485 | 1.0000 | 0.0000 |
| TA_303 | 1972 | TA | TA_BEFORE | 0.3411 | 0.1324 | 22.9402 | 0.4390 | 0.2051 | 0.3726 | 0.8196 | 5.1019 | 0.5860 | 6.7802 | 0.4780 | 22.2203 | 2.5073 | 0.9908 | 0.0000 |
| TA_304 | 1972 | TA | TA_BEFORE | 0.3839 | 0.1462 | 23.9693 | 0.4522 | 0.2119 | 0.3885 | 0.7749 | 5.1826 | 0.5975 | 6.8990 | 0.4899 | 23.5973 | 2.6606 | 0.9829 | 0.0000 |
| TA_305 | 1972 | TA | TA_BEFORE | 0.5884 | 0.2090 | 21.4641 | 0.4174 | 0.2691 | 0.5232 | 0.7227 | 5.4701 | 0.6386 | 7.2079 | 0.5208 | 22.9668 | 2.2626 | 0.9832 | 0.0000 |
| TA_306 | 1972 | TA | TA_BEFORE | 0.4566 | 0.1704 | 23.7408 | 0.4464 | 0.2172 | 0.4010 | 0.6667 | 5.3465 | 0.6209 | 6.6071 | 0.4607 | 21.5378 | 2.2528 | 0.9902 | 0.0000 |

| | | | | | | | | | | | | | | | | | | |
|---|---|---|---|---|---|---|---|---|---|---|---|---|---|---|---|---|---|---|
| TA_307 | 1972 | TA | TA_BEFORE | 0.4393 | 0.1663 | 22.0286 | 0.4274 | 0.2371 | 0.4479 | 0.4565 | 5.7182 | 0.6740 | 6.6337 | 0.4634 | 19.4426 | 2.0820 | 0.9910 | 0.0000 |
| TA_308 | 1972 | TA | TA_BEFORE | 0.4802 | 0.1778 | 23.3884 | 0.4390 | 0.2136 | 0.3925 | 0.6667 | 5.0482 | 0.5783 | 7.3188 | 0.5319 | 19.8924 | 1.9272 | 0.9765 | 0.0000 |
| TA_937 | 2009 | TA | TA_REWET | 0.6423 | 0.2311 | 21.6544 | 0.4172 | 0.2926 | 0.5786 | 0.6817 | 5.6826 | 0.6689 | 8.1845 | 0.6184 | 25.6423 | 2.1669 | 0.9222 | 0.0778 |
| TA_938 | 2009 | TA | TA_REWET | 0.6755 | 0.2396 | 20.9685 | 0.4117 | 0.2754 | 0.5381 | 0.6996 | 5.2548 | 0.6078 | 7.9252 | 0.5925 | 23.7615 | 2.1366 | 0.9011 | 0.0989 |
| TA_939 | 2009 | TA | TA_REWET | 0.5894 | 0.2134 | 22.3344 | 0.4313 | 0.2510 | 0.4807 | 0.6199 | 5.5387 | 0.6484 | 7.8542 | 0.5854 | 25.5873 | 2.0303 | 0.9410 | 0.0590 |
| TA_940 | 2009 | TA | TA_REWET | 0.3994 | 0.1523 | 19.3967 | 0.3889 | 0.2157 | 0.3976 | 0.8309 | 5.6015 | 0.6574 | 7.0162 | 0.5016 | 20.3346 | 2.2942 | 0.9963 | 0.0037 |
| TA_941 | 2009 | TA | TA_REWET | 0.4647 | 0.1718 | 20.0112 | 0.3999 | 0.2243 | 0.4178 | 0.7733 | 5.6544 | 0.6649 | 7.1913 | 0.5191 | 21.2735 | 2.3429 | 1.0000 | 0.0000 |
| TA_942 | 2009 | TA | TA_REWET | 0.5120 | 0.1865 | 18.3608 | 0.3721 | 0.2419 | 0.4591 | 0.7830 | 5.6484 | 0.6641 | 7.4570 | 0.5457 | 20.2770 | 2.0432 | 0.9688 | 0.0313 |
| TA_943 | 2009 | TA | TA_REWET | 0.4190 | 0.1577 | 22.4587 | 0.4336 | 0.2230 | 0.4146 | 0.7792 | 5.3593 | 0.6228 | 7.3618 | 0.5362 | 25.1612 | 2.6482 | 0.9913 | 0.0087 |
| TA_944 | 2009 | TA | TA_REWET | 0.4439 | 0.1669 | 21.5109 | 0.4171 | 0.2359 | 0.4450 | 0.8129 | 5.3684 | 0.6241 | 7.1554 | 0.5155 | 23.7213 | 2.5473 | 0.9912 | 0.0088 |
| TA_945 | 2009 | TA | TA_REWET | 0.4536 | 0.1673 | 20.9016 | 0.4099 | 0.2395 | 0.4535 | 0.7437 | 5.5168 | 0.6453 | 7.4560 | 0.5456 | 20.9171 | 2.1741 | 0.9916 | 0.0084 |
| TA_946 | 2009 | TA | TA_REWET | 0.6153 | 0.2204 | 16.7870 | 0.3308 | 0.2496 | 0.4772 | 0.8667 | 5.2875 | 0.6125 | 8.3506 | 0.6351 | 20.4034 | 1.0929 | 1.0000 | 0.0000 |
| TA_947 | 2009 | TA | TA_REWET | 0.5885 | 0.2129 | 21.5032 | 0.4208 | 0.2716 | 0.5291 | 0.6395 | 5.3288 | 0.6184 | 8.2714 | 0.6271 | 28.1572 | 2.3828 | 1.0000 | 0.0000 |
| TA_948 | 2009 | TA | TA_REWET | 0.6529 | 0.2328 | 22.2750 | 0.4275 | 0.2753 | 0.5377 | 0.6315 | 5.4441 | 0.6349 | 8.1892 | 0.6189 | 27.1206 | 2.1938 | 0.9936 | 0.0064 |
| TA_949 | 2009 | TA | TA_REWET | 0.6191 | 0.2219 | 21.7250 | 0.4234 | 0.2660 | 0.5159 | 0.6882 | 5.1565 | 0.5938 | 8.3171 | 0.6317 | 26.8128 | 2.1298 | 0.9966 | 0.0034 |
| TA_950 | 2009 | TA | TA_REWET | 0.5830 | 0.2109 | 21.9937 | 0.4262 | 0.2627 | 0.5082 | 0.6439 | 5.2313 | 0.6045 | 8.3176 | 0.6318 | 27.2356 | 2.1791 | 0.9967 | 0.0033 |
| TA_951 | 2009 | TA | TA_REWET | 0.5226 | 0.1941 | 19.1791 | 0.3805 | 0.2362 | 0.4458 | 0.6970 | 5.0710 | 0.5816 | 8.4561 | 0.6456 | 28.0305 | 2.6132 | 0.9936 | 0.0000 |
| TA_952 | 2009 | TA | TA_REWET | 0.7695 | 0.2647 | 17.2869 | 0.3461 | 0.3072 | 0.6129 | 0.7801 | 5.5344 | 0.6478 | 8.4837 | 0.6484 | 21.4284 | 1.7317 | 0.9947 | 0.0053 |
| TA_953 | 2009 | TA | TA_REWET | 0.6548 | 0.2333 | 21.6815 | 0.4181 | 0.2851 | 0.5609 | 0.5937 | 5.8066 | 0.6867 | 8.1260 | 0.6126 | 26.7660 | 1.9045 | 0.9964 | 0.0036 |
| TA_954 | 2009 | TA | TA_REWET | 0.7336 | 0.2543 | 18.8321 | 0.3725 | 0.2889 | 0.5697 | 0.7484 | 5.3472 | 0.6210 | 8.0000 | 0.6000 | 19.7390 | 1.7397 | 0.9962 | 0.0038 |
| TA_955 | 2009 | TA | TA_REWET | 0.7230 | 0.2517 | 18.9600 | 0.3653 | 0.2605 | 0.5029 | 0.7410 | 5.5019 | 0.6431 | 8.6540 | 0.6654 | 19.5798 | 1.6169 | 0.9963 | 0.0037 |
| TA_956 | 2009 | TA | TA_REWET | 0.7394 | 0.2562 | 18.8948 | 0.3718 | 0.2929 | 0.5791 | 0.7425 | 5.4776 | 0.6397 | 8.4198 | 0.6420 | 21.0876 | 1.7934 | 1.0000 | 0.0000 |
| TA_957 | 2009 | TA | TA_REWET | 0.6856 | 0.2415 | 20.4622 | 0.3987 | 0.2917 | 0.5764 | 0.6804 | 5.6027 | 0.6575 | 8.0896 | 0.6090 | 25.5973 | 2.1530 | 0.9909 | 0.0091 |
| TA_958 | 2009 | TA | TA_REWET | 0.5355 | 0.1993 | 21.2773 | 0.4047 | 0.2537 | 0.4870 | 0.5900 | 5.6467 | 0.6638 | 8.2085 | 0.6208 | 27.5441 | 1.7603 | 0.9967 | 0.0033 |
| TA_959 | 2009 | TA | TA_REWET | 0.5405 | 0.1979 | 21.4530 | 0.4142 | 0.2951 | 0.5844 | 0.6350 | 5.3306 | 0.6187 | 8.2385 | 0.6238 | 24.8332 | 2.1812 | 0.9917 | 0.0083 |
| TA_960 | 2009 | TA | TA_REWET | 0.6295 | 0.2259 | 19.9081 | 0.3850 | 0.2939 | 0.5816 | 0.7329 | 5.8723 | 0.6960 | 7.9464 | 0.5946 | 25.7864 | 2.0745 | 0.9965 | 0.0035 |
| TA_961 | 2009 | TA | TA_REWET | 0.6299 | 0.2273 | 21.0114 | 0.4012 | 0.2845 | 0.5594 | 0.7196 | 5.8265 | 0.6895 | 7.9121 | 0.5912 | 27.0388 | 2.2631 | 0.9971 | 0.0029 |
| TA_962 | 2009 | TA | TA_REWET | 0.5814 | 0.2099 | 23.0036 | 0.4306 | 0.2891 | 0.5701 | 0.6179 | 5.6516 | 0.6645 | 7.9509 | 0.5951 | 23.9738 | 1.7447 | 0.9965 | 0.0000 |
| TA_963 | 2009 | TA | TA_REWET | 0.5453 | 0.1982 | 21.9876 | 0.4229 | 0.2821 | 0.5537 | 0.6769 | 5.3436 | 0.6205 | 7.9018 | 0.5902 | 21.7417 | 1.4141 | 1.0000 | 0.0000 |
| TA_964 | 2009 | TA | TA_REWET | 0.6155 | 0.2204 | 22.5175 | 0.4273 | 0.2713 | 0.5284 | 0.6543 | 5.4007 | 0.6287 | 8.0171 | 0.6017 | 22.9430 | 1.3976 | 1.0000 | 0.0000 |
| TA_965 | 2009 | TA | TA_REWET | 0.5948 | 0.2136 | 19.7641 | 0.3809 | 0.2788 | 0.5459 | 0.6946 | 5.5133 | 0.6448 | 8.0820 | 0.6082 | 21.3010 | 1.5872 | 0.9962 | 0.0038 |
| TA_966 | 2009 | TA | TA_REWET | 0.5763 | 0.2120 | 27.3856 | 0.4746 | 0.2049 | 0.3720 | 0.4963 | 5.7370 | 0.6767 | 8.1353 | 0.6135 | 24.9366 | 1.1003 | 0.9926 | 0.0074 |
| TR_1_1998 | 1998 | TR | TR_BEFORE | 0.4165 | 0.1604 | 28.4512 | 0.5075 | 0.2220 | 0.4123 | 0.8710 | 5.3277 | 0.6182 | 6.7041 | 0.4704 | 31.4902 | 2.9848 | 1.0000 | 0.0000 |
| TR_10_1998 | 1998 | TR | TR_BEFORE | 0.2957 | 0.1189 | 28.3525 | 0.5064 | 0.2065 | 0.3758 | 0.9247 | 5.2312 | 0.6045 | 6.6566 | 0.4657 | 32.6156 | 3.0939 | 1.0000 | 0.0000 |
| TR_102_2008 | 2008 | TR | TR_REWET | 0.7829 | 0.2705 | 22.5982 | 0.4024 | 0.2452 | 0.4670 | 0.7821 | 5.9121 | 0.7017 | 8.5604 | 0.6560 | 18.4482 | 1.4520 | 0.9890 | 0.0110 |
| TR_103_1995 | 1995 | TR | TR_BEFORE | 0.4161 | 0.1588 | 19.4235 | 0.3900 | 0.2367 | 0.4469 | 0.5525 | 5.0137 | 0.5734 | 9.0205 | 0.7021 | 22.8235 | 2.5220 | 0.5890 | 0.4110 |
| TR_109_2008 | 2008 | TR | TR_REWET | 0.3896 | 0.1470 | 25.1397 | 0.4403 | 0.2031 | 0.3679 | 0.6782 | 4.9792 | 0.5685 | 9.1111 | 0.7111 | 23.7731 | 2.6888 | 0.9653 | 0.0347 |
| TR_110_1995 | 1995 | TR | TR_BEFORE | 0.5594 | 0.2046 | 18.4448 | 0.3724 | 0.2416 | 0.4586 | 0.4919 | 4.9709 | 0.5673 | 8.9126 | 0.6913 | 24.3296 | 2.4589 | 0.5097 | 0.4903 |
| TR_116_2008 | 2008 | TR | TR_REWET | 0.6193 | 0.2238 | 16.2573 | 0.3311 | 0.2706 | 0.5267 | 0.5798 | 5.0424 | 0.5775 | 9.0303 | 0.7030 | 18.8849 | 1.5796 | 0.6303 | 0.3697 |
| TR_117_1995 | 1995 | TR | TR_BEFORE | 0.9005 | 0.3028 | 13.0338 | 0.2646 | 0.2900 | 0.5723 | 0.6560 | 5.8782 | 0.6969 | 9.2564 | 0.7256 | 16.4205 | 0.9623 | 0.9487 | 0.0513 |
| TR_12_2008 | 2008 | TR | TR_REWET | 0.6454 | 0.2263 | 28.5344 | 0.5025 | 0.2408 | 0.4566 | 0.7343 | 5.6129 | 0.6590 | 6.9585 | 0.4959 | 26.2498 | 2.6188 | 0.9816 | 0.0184 |
| TR_123_2008 | 2008 | TR | TR_REWET | 0.8761 | 0.2966 | 11.0252 | 0.2279 | 0.3107 | 0.6211 | 0.7690 | 5.4339 | 0.6334 | 9.2751 | 0.7275 | 16.4812 | 1.0829 | 0.8995 | 0.1005 |
| TR_124_1995 | 1995 | TR | TR_BEFORE | 0.5758 | 0.2142 | 13.8825 | 0.2925 | 0.3155 | 0.6324 | 0.6723 | 5.1864 | 0.5981 | 8.6780 | 0.6678 | 17.5730 | 1.8455 | 0.8588 | 0.1412 |
| TR_13_1998 | 1998 | TR | TR_BEFORE | 0.3680 | 0.1429 | 27.6796 | 0.4950 | 0.2188 | 0.4047 | 0.8701 | 5.3220 | 0.6174 | 7.2983 | 0.5298 | 26.2276 | 3.3590 | 0.9729 | 0.0271 |
| TR_130_2008 | 2008 | TR | TR_REWET | 0.7258 | 0.2503 | 22.3584 | 0.3892 | 0.2633 | 0.5095 | 0.7825 | 4.8205 | 0.5458 | 9.5169 | 0.7517 | 22.8301 | 2.1665 | 0.9746 | 0.0169 |
| TR_131_1995 | 1995 | TR | TR_BEFORE | 0.6220 | 0.2166 | 25.6020 | 0.3357 | 0.2492 | 0.4763 | 0.6531 | 5.6327 | 0.6618 | 8.2041 | 0.6204 | 19.8951 | 1.8237 | 0.4694 | 0.5306 |
| TR_137_2008 | 2008 | TR | TR_REWET | 0.6532 | 0.2302 | 22.7827 | 0.3837 | 0.2432 | 0.4623 | 0.7270 | 4.9389 | 0.5627 | 9.8626 | 0.7863 | 21.3664 | 2.2200 | 0.9695 | 0.0305 |
| TR_138_1995 | 1995 | TR | TR_BEFORE | 1.1306 | 0.3418 | 22.7909 | 0.4008 | 0.2471 | 0.4715 | 0.8440 | 5.2447 | 0.6064 | 9.5851 | 0.7585 | 19.7868 | 2.2346 | 0.7340 | 0.2660 |
| TR_144_2008 | 2008 | TR | TR_REWET | 0.6479 | 0.2222 | 39.4163 | 0.4665 | 0.1663 | 0.2812 | 0.6996 | 5.1333 | 0.5905 | 9.6981 | 0.7698 | 21.1618 | 2.2581 | 0.4245 | 0.5660 |
| TR_145_1995 | 1995 | TR | TR_BEFORE | 0.8442 | 0.2842 | 17.7241 | 0.3617 | 0.3011 | 0.5985 | 0.8777 | 4.5872 | 0.5125 | 8.9541 | 0.6954 | 26.0312 | 1.9428 | 1.0000 | 0.0000 |
| TR_15_2008 | 2008 | TR | TR_REWET | 0.6813 | 0.2439 | 12.3839 | 0.2340 | 0.2647 | 0.5127 | 0.6628 | 5.5988 | 0.6570 | 8.1453 | 0.6145 | 17.9348 | 2.0373 | 0.4884 | 0.5116 |

| | | | | | | | | | | | | | | | | | | |
|---|---|---|---|---|---|---|---|---|---|---|---|---|---|---|---|---|---|---|
| TR_151_2008 | 2008 | TR | TR_REWET | 0.7945 | 0.2745 | 16.5976 | 0.3444 | 0.2980 | 0.5913 | 0.8532 | 4.4771 | 0.4967 | 9.2936 | 0.7294 | 24.5659 | 2.0521 | 0.9633 | 0.0367 |
| TR_152_1995 | 1995 | TR | TR_BEFORE | 1.1472 | 0.3401 | 22.8154 | 0.4149 | 0.2817 | 0.5528 | 0.9059 | 5.3306 | 0.6187 | 9.8306 | 0.7831 | 22.7029 | 2.0611 | 1.0000 | 0.0000 |
| TR_158_2008 | 2008 | TR | TR_REWET | 1.1109 | 0.3344 | 32.7040 | 0.4569 | 0.2375 | 0.4488 | 0.8796 | 5.5467 | 0.6495 | 9.9474 | 0.7947 | 22.2080 | 2.0552 | 0.9737 | 0.0132 |
| TR_159_1995 | 1995 | TR | TR_BEFORE | 0.5116 | 0.1788 | 24.7402 | 0.4653 | 0.2142 | 0.3939 | 0.8804 | 5.1928 | 0.5990 | 7.0465 | 0.5047 | 25.7008 | 3.1500 | 1.0000 | 0.0000 |
| TR_16_1998 | 1998 | TR | TR_BEFORE | 0.4521 | 0.1751 | 28.0781 | 0.5044 | 0.2573 | 0.4953 | 0.6633 | 5.0498 | 0.5785 | 6.0700 | 0.4070 | 23.7522 | 2.8774 | 1.0000 | 0.0000 |
| TR_165_2008 | 2008 | TR | TR_REWET | 0.9417 | 0.3073 | 21.4164 | 0.4206 | 0.2766 | 0.5409 | 0.8768 | 5.7818 | 0.6831 | 7.9441 | 0.5944 | 22.4257 | 2.1977 | 0.9697 | 0.0303 |
| TR_166_1995 | 1995 | TR | TR_BEFORE | 0.8633 | 0.2709 | 22.9183 | 0.4355 | 0.2391 | 0.4525 | 0.8686 | 5.4373 | 0.6339 | 8.0466 | 0.6047 | 23.2862 | 2.8711 | 0.9534 | 0.0466 |
| TR_172_2008 | 2008 | TR | TR_REWET | 1.1708 | 0.3624 | 20.5387 | 0.4054 | 0.2520 | 0.4830 | 0.9456 | 5.7143 | 0.6735 | 8.0816 | 0.6082 | 22.7892 | 2.2219 | 0.9660 | 0.0340 |
| TR_173_1995 | 1995 | TR | TR_BEFORE | 0.8159 | 0.2826 | 27.6040 | 0.5001 | 0.2100 | 0.3840 | 0.8821 | 5.8559 | 0.6937 | 5.8145 | 0.3814 | 39.0614 | 3.9512 | 1.0000 | 0.0000 |
| TR_179_2008 | 2008 | TR | TR_REWET | 0.5042 | 0.1903 | 24.4132 | 0.4653 | 0.2708 | 0.5272 | 0.8325 | 5.5217 | 0.6460 | 5.1308 | 0.3131 | 24.5007 | 2.1060 | 1.0000 | 0.0000 |
| TR_18_2008 | 2008 | TR | TR_REWET | 0.3502 | 0.1393 | 24.6593 | 0.4556 | 0.2356 | 0.4442 | 0.8026 | 5.6447 | 0.6635 | 6.5022 | 0.4502 | 24.4008 | 2.1533 | 0.9649 | 0.0351 |
| TR_180_1995 | 1995 | TR | TR_BEFORE | 0.5521 | 0.2032 | 23.7359 | 0.4480 | 0.2651 | 0.5138 | 0.7786 | 5.4363 | 0.6338 | 5.8920 | 0.3892 | 25.0292 | 2.3243 | 0.9961 | 0.0039 |
| TR_186_2008 | 2008 | TR | TR_REWET | 0.4324 | 0.1658 | 25.5333 | 0.4714 | 0.2349 | 0.4426 | 0.7481 | 5.1389 | 0.5913 | 6.2987 | 0.4299 | 25.6650 | 2.7751 | 1.0000 | 0.0000 |
| TR_187_1995 | 1995 | TR | TR_BEFORE | 0.3578 | 0.1422 | 25.3286 | 0.4744 | 0.2596 | 0.5008 | 0.7279 | 5.0136 | 0.5734 | 5.6500 | 0.3650 | 20.9115 | 2.6623 | 1.0000 | 0.0000 |
| TR_19_1998 | 1998 | TR | TR_BEFORE | 0.4232 | 0.1624 | 28.3420 | 0.5063 | 0.2060 | 0.3747 | 0.7258 | 4.7581 | 0.5369 | 6.6532 | 0.4653 | 26.1985 | 3.5421 | 1.0000 | 0.0000 |
| TR_193_2008 | 2008 | TR | TR_REWET | 0.5611 | 0.2041 | 24.0325 | 0.4509 | 0.2556 | 0.4914 | 0.8078 | 5.0368 | 0.5767 | 7.1355 | 0.5135 | 20.2704 | 1.8077 | 0.9939 | 0.0061 |
| TR_194_1995 | 1995 | TR | TR_BEFORE | 0.4977 | 0.1882 | 23.3982 | 0.4474 | 0.2640 | 0.5111 | 0.8414 | 5.5388 | 0.6484 | 5.6855 | 0.3685 | 23.9308 | 2.1030 | 1.0000 | 0.0000 |
| TR_200_2008 | 2008 | TR | TR_REWET | 0.5496 | 0.2000 | 22.4182 | 0.4331 | 0.2621 | 0.5068 | 0.8049 | 5.1932 | 0.5990 | 7.3684 | 0.5368 | 22.1803 | 2.0259 | 0.9773 | 0.0227 |
| TR_201_1995 | 1995 | TR | TR_BEFORE | 0.5812 | 0.2081 | 27.6642 | 0.4891 | 0.2127 | 0.3904 | 0.7398 | 5.6898 | 0.6700 | 6.3901 | 0.4390 | 27.3449 | 2.1620 | 0.9947 | 0.0053 |
| TR_207_2008 | 2008 | TR | TR_REWET | 0.4836 | 0.1803 | 22.4239 | 0.4248 | 0.2273 | 0.4248 | 0.7924 | 5.5965 | 0.6566 | 6.7688 | 0.4769 | 21.8040 | 2.1141 | 1.0000 | 0.0000 |
| TR_208_1995 | 1995 | TR | TR_BEFORE | 0.6289 | 0.2208 | 20.6018 | 0.4061 | 0.2641 | 0.5115 | 0.8818 | 4.3880 | 0.4840 | 8.3103 | 0.6310 | 26.8562 | 2.3793 | 1.0000 | 0.0000 |
| TR_21_2008 | 2008 | TR | TR_REWET | 0.3278 | 0.1317 | 23.0570 | 0.4384 | 0.2426 | 0.4608 | 0.8014 | 4.9125 | 0.5589 | 6.1650 | 0.4165 | 22.9005 | 2.4998 | 1.0000 | 0.0000 |
| TR_214_2008 | 2008 | TR | TR_REWET | 0.9962 | 0.3238 | 18.8544 | 0.3836 | 0.2875 | 0.5665 | 0.9541 | 4.9694 | 0.5671 | 8.3854 | 0.6385 | 25.6029 | 2.1382 | 0.9745 | 0.0255 |
| TR_215_1995 | 1995 | TR | TR_BEFORE | 0.4542 | 0.1761 | 29.2904 | 0.5176 | 0.2738 | 0.5342 | 0.7487 | 5.9733 | 0.7105 | 7.2086 | 0.5209 | 32.1272 | 1.8118 | 1.0000 | 0.0000 |
| TR_22_1998 | 1998 | TR | TR_BEFORE | 0.3639 | 0.1415 | 27.0732 | 0.4923 | 0.2093 | 0.3824 | 0.8143 | 5.0295 | 0.5756 | 6.8667 | 0.4867 | 26.3177 | 3.3380 | 1.0000 | 0.0000 |
| TR_221_2008 | 2008 | TR | TR_REWET | 0.7771 | 0.2587 | 22.4063 | 0.4244 | 0.2438 | 0.4636 | 0.8607 | 5.8307 | 0.6901 | 7.4419 | 0.5442 | 25.3519 | 2.1495 | 0.9206 | 0.0794 |
| TR_222_1995 | 1995 | TR | TR_BEFORE | 0.5202 | 0.1945 | 27.7718 | 0.4999 | 0.2628 | 0.5082 | 0.7230 | 5.6280 | 0.6611 | 7.0443 | 0.5044 | 30.0077 | 2.3534 | 0.9952 | 0.0048 |
| TR_228_2008 | 2008 | TR | TR_REWET | 0.9187 | 0.3081 | 16.9259 | 0.3596 | 0.3192 | 0.6411 | 0.9784 | 4.1753 | 0.4536 | 8.8377 | 0.6838 | 12.4093 | 0.7758 | 0.9740 | 0.0260 |
| TR_229_1995 | 1995 | TR | TR_BEFORE | 0.4186 | 0.1560 | 38.5245 | 0.5896 | 0.2018 | 0.3649 | 0.4675 | 4.7593 | 0.5370 | 5.4783 | 0.3478 | 31.0176 | 2.2631 | 0.6883 | 0.0130 |
| TR_23_2008 | 2008 | TR | TR_REWET | 0.5996 | 0.2200 | 16.5798 | 0.3436 | 0.2443 | 0.4648 | 0.6514 | 5.2519 | 0.6074 | 9.4733 | 0.7473 | 19.0634 | 2.2905 | 0.9389 | 0.0611 |
| TR_235_2008 | 2008 | TR | TR_REWET | 0.6220 | 0.2218 | 41.4539 | 0.6117 | 0.2011 | 0.3631 | 0.3829 | 6.1622 | 0.7375 | 5.9429 | 0.3943 | 31.8630 | 1.8680 | 0.6033 | 0.0083 |
| TR_236_1995 | 1995 | TR | TR_BEFORE | 0.2664 | 0.1081 | 24.3599 | 0.4634 | 0.2232 | 0.4151 | 0.8674 | 5.0739 | 0.5820 | 6.3551 | 0.4355 | 22.9902 | 2.9237 | 1.0000 | 0.0000 |
| TR_24_1998 | 1998 | TR | TR_BEFORE | 0.4047 | 0.1545 | 24.5399 | 0.4588 | 0.2647 | 0.5129 | 0.7597 | 5.2853 | 0.6122 | 6.6718 | 0.4672 | 27.0379 | 2.3346 | 0.9755 | 0.0245 |
| TR_242_2008 | 2008 | TR | TR_REWET | 0.3900 | 0.1506 | 26.3035 | 0.4817 | 0.2080 | 0.3794 | 0.8106 | 5.2775 | 0.6111 | 6.7342 | 0.4734 | 27.2097 | 3.0482 | 1.0000 | 0.0000 |
| TR_243_1995 | 1995 | TR | TR_BEFORE | 0.5780 | 0.1965 | 23.7636 | 0.4546 | 0.2458 | 0.4682 | 0.8544 | 5.3686 | 0.6241 | 6.5361 | 0.4536 | 22.0866 | 2.5387 | 1.0000 | 0.0000 |
| TR_249_2008 | 2008 | TR | TR_REWET | 0.3005 | 0.1176 | 24.4610 | 0.4620 | 0.1979 | 0.3555 | 0.9070 | 5.1931 | 0.5990 | 6.7403 | 0.4740 | 25.3908 | 3.0444 | 0.9957 | 0.0043 |
| TR_250_1995 | 1995 | TR | TR_BEFORE | 0.2539 | 0.1051 | 24.5610 | 0.4631 | 0.2117 | 0.3881 | 0.8680 | 5.2508 | 0.6073 | 6.3725 | 0.4372 | 24.0144 | 2.6545 | 1.0000 | 0.0000 |
| TR_256_2008 | 2008 | TR | TR_REWET | 0.3053 | 0.1218 | 25.0868 | 0.4673 | 0.2044 | 0.3709 | 0.8618 | 5.1951 | 0.5993 | 6.8259 | 0.4826 | 25.4711 | 3.0348 | 0.9903 | 0.0049 |
| TR_257_1995 | 1995 | TR | TR_BEFORE | 0.2615 | 0.1075 | 25.1081 | 0.4709 | 0.2177 | 0.4023 | 0.8665 | 5.2527 | 0.6075 | 6.4367 | 0.4437 | 24.3260 | 2.6580 | 1.0000 | 0.0000 |
| TR_26_2008 | 2008 | TR | TR_REWET | 0.3799 | 0.1460 | 18.8491 | 0.3807 | 0.3165 | 0.6347 | 0.6819 | 5.1813 | 0.5973 | 8.4400 | 0.6440 | 18.1818 | 1.8102 | 0.9886 | 0.0114 |
| TR_263_2008 | 2008 | TR | TR_REWET | 0.4796 | 0.1753 | 23.7573 | 0.4436 | 0.2283 | 0.4272 | 0.8457 | 5.5037 | 0.6434 | 6.8851 | 0.4885 | 23.9827 | 2.4320 | 1.0000 | 0.0000 |
| TR_264_1995 | 1995 | TR | TR_BEFORE | 0.3388 | 0.1339 | 26.4558 | 0.4862 | 0.2310 | 0.4335 | 0.8166 | 5.3391 | 0.6199 | 6.8667 | 0.4867 | 26.5630 | 2.7479 | 1.0000 | 0.0000 |
| TR_27_1998 | 1998 | TR | TR_BEFORE | 0.7367 | 0.2470 | 24.6451 | 0.4633 | 0.2414 | 0.4580 | 0.7687 | 5.6619 | 0.6660 | 6.4908 | 0.4491 | 26.3777 | 2.6680 | 1.0000 | 0.0000 |
| TR_270_2008 | 2008 | TR | TR_REWET | 0.4950 | 0.1819 | 24.3015 | 0.4500 | 0.2302 | 0.4316 | 0.7937 | 5.2597 | 0.6085 | 7.2687 | 0.5269 | 23.5885 | 2.6851 | 0.9827 | 0.0173 |
| TR_271_1995 | 1995 | TR | TR_BEFORE | 0.4353 | 0.1691 | 27.5126 | 0.4956 | 0.2579 | 0.4968 | 0.7014 | 5.5792 | 0.6542 | 6.6864 | 0.4686 | 27.5236 | 2.3390 | 0.9667 | 0.0333 |
| TR_277_2008 | 2008 | TR | TR_REWET | 0.5035 | 0.1902 | 20.0563 | 0.3864 | 0.2560 | 0.4923 | 0.8353 | 5.7888 | 0.6841 | 6.5992 | 0.4599 | 20.6719 | 2.1469 | 0.9522 | 0.0478 |
| TR_278_1995 | 1995 | TR | TR_BEFORE | 0.4310 | 0.1669 | 26.9485 | 0.4913 | 0.2691 | 0.5233 | 0.7135 | 5.7479 | 0.6783 | 7.1983 | 0.5198 | 30.0837 | 2.0581 | 0.9835 | 0.0165 |
| TR_284_2008 | 2008 | TR | TR_REWET | 0.7334 | 0.2552 | 24.2015 | 0.4506 | 0.3072 | 0.6129 | 0.7812 | 5.3015 | 0.6145 | 7.6107 | 0.5611 | 28.8648 | 2.1501 | 0.9662 | 0.0188 |
| TR_285_1995 | 1995 | TR | TR_BEFORE | 1.1106 | 0.3457 | 20.0260 | 0.4040 | 0.2695 | 0.5242 | 0.9216 | 5.5294 | 0.6471 | 7.9679 | 0.5968 | 23.3735 | 2.2666 | 1.0000 | 0.0000 |
| TR_29_2008 | 2008 | TR | TR_REWET | 0.6887 | 0.2304 | 19.7482 | 0.4008 | 0.2281 | 0.4266 | 0.7701 | 5.3388 | 0.6198 | 7.4026 | 0.5403 | 24.4183 | 2.4365 | 0.9967 | 0.0000 |
| TR_291_2008 | 2008 | TR | TR_REWET | 0.5400 | 0.1975 | 28.6067 | 0.5051 | 0.2523 | 0.4836 | 0.8037 | 5.8712 | 0.6959 | 7.9080 | 0.5908 | 29.6625 | 1.8317 | 0.9939 | 0.0000 |

| | | | | | | | | | | | | | | | | | | |
|---|---|---|---|---|---|---|---|---|---|---|---|---|---|---|---|---|---|---|
| TR_3_2008 | 2008 | TR | TR_REWET | 0.3693 | 0.1442 | 28.5813 | 0.5037 | 0.2553 | 0.4907 | 0.8560 | 5.5377 | 0.6482 | 7.3709 | 0.5371 | 30.5025 | 2.6016 | 0.9813 | 0.0187 |
| TR_30_1998 | 1998 | TR | TR_BEFORE | 0.8518 | 0.2800 | 24.3632 | 0.4589 | 0.2513 | 0.4814 | 0.8143 | 5.6287 | 0.6612 | 7.0411 | 0.5041 | 24.9418 | 2.6279 | 1.0000 | 0.0000 |
| TR_32_2008 | 2008 | TR | TR_REWET | 0.5512 | 0.1906 | 22.0321 | 0.4303 | 0.2054 | 0.3732 | 0.7161 | 5.6844 | 0.6692 | 7.0570 | 0.5057 | 24.2165 | 2.5261 | 0.9962 | 0.0038 |
| TR_33_1998 | 1998 | TR | TR_BEFORE | 0.3638 | 0.1437 | 26.5631 | 0.4836 | 0.2263 | 0.4225 | 0.7885 | 5.4619 | 0.6374 | 6.6564 | 0.4656 | 29.5982 | 2.5958 | 0.9949 | 0.0051 |
| TR_35_2008 | 2008 | TR | TR_REWET | 0.4793 | 0.1786 | 20.9180 | 0.3809 | 0.2278 | 0.4259 | 0.7558 | 5.7219 | 0.6746 | 7.4785 | 0.5478 | 25.4219 | 2.2739 | 0.8663 | 0.1337 |
| TR_36_1998 | 1998 | TR | TR_BEFORE | 0.4587 | 0.1752 | 28.3651 | 0.5041 | 0.2266 | 0.4232 | 0.7281 | 5.0855 | 0.5836 | 6.4150 | 0.4415 | 29.1362 | 3.1814 | 0.9474 | 0.0526 |
| TR_38_2008 | 2008 | TR | TR_REWET | 0.3389 | 0.1342 | 25.1615 | 0.4673 | 0.2118 | 0.3883 | 0.8299 | 5.2510 | 0.6073 | 6.7572 | 0.4757 | 25.0726 | 3.1313 | 0.9671 | 0.0329 |
| TR_39_1998 | 1998 | TR | TR_BEFORE | 0.4148 | 0.1621 | 25.4656 | 0.4613 | 0.2569 | 0.4944 | 0.6959 | 5.6912 | 0.6702 | 7.0892 | 0.5089 | 23.7981 | 2.4621 | 0.7235 | 0.2765 |
| TR_4_1998 | 1998 | TR | TR_BEFORE | 0.5382 | 0.2004 | 26.5242 | 0.4862 | 0.2388 | 0.4519 | 0.9075 | 5.4806 | 0.6401 | 5.9917 | 0.3992 | 32.7077 | 2.3028 | 1.0000 | 0.0000 |
| TR_41_2008 | 2008 | TR | TR_REWET | 0.4486 | 0.1746 | 28.4475 | 0.5029 | 0.2741 | 0.5349 | 0.7103 | 6.0238 | 0.7177 | 7.3631 | 0.5363 | 29.7234 | 1.8447 | 0.9107 | 0.0893 |
| TR_42_1998 | 1998 | TR | TR_BEFORE | 0.7010 | 0.2348 | 26.2423 | 0.4808 | 0.2291 | 0.4289 | 0.8669 | 5.4241 | 0.6320 | 7.2019 | 0.5202 | 27.3203 | 3.0734 | 1.0000 | 0.0000 |
| TR_44_2008 | 2008 | TR | TR_REWET | 0.3463 | 0.1018 | 45.1825 | 0.5919 | 0.1363 | 0.2105 | 0.8254 | 4.5738 | 0.5105 | 10.7131 | 0.8713 | 31.1610 | 1.5850 | 1.0000 | 0.0000 |
| TR_45_2008 | 2008 | TR | TR_REWET | 0.7622 | 0.2581 | 24.1747 | 0.4006 | 0.2885 | 0.5689 | 0.8189 | 4.5197 | 0.5028 | 9.1626 | 0.7163 | 15.1982 | 0.8272 | 0.9055 | 0.0945 |
| TR_46_2008 | 2008 | TR | TR_REWET | 0.1409 | 0.0588 | 22.6827 | 0.4376 | 0.1750 | 0.3016 | 0.5875 | 5.7178 | 0.6740 | 7.8832 | 0.5883 | 22.9205 | 2.0523 | 0.9950 | 0.0050 |
| TR_47_1995 | 1995 | TR | TR_BEFORE | 0.4395 | 0.1695 | 21.2684 | 0.4062 | 0.2487 | 0.4752 | 0.8577 | 5.6285 | 0.6612 | 6.2419 | 0.4242 | 20.9110 | 2.2098 | 1.0000 | 0.0000 |
| TR_53_2008 | 2008 | TR | TR_REWET | 0.8881 | 0.2893 | 22.9233 | 0.4348 | 0.2524 | 0.4840 | 0.8211 | 5.7740 | 0.6820 | 7.5371 | 0.5537 | 23.2247 | 2.5777 | 0.9153 | 0.0847 |
| TR_54_1995 | 1995 | TR | TR_BEFORE | 0.7803 | 0.2711 | 18.4357 | 0.3556 | 0.2732 | 0.5329 | 0.7505 | 4.8563 | 0.5509 | 8.7006 | 0.6701 | 26.2952 | 1.7307 | 0.9042 | 0.0958 |
| TR_6_2008 | 2008 | TR | TR_REWET | 0.5420 | 0.2013 | 26.0896 | 0.4816 | 0.2376 | 0.4491 | 0.8855 | 5.5283 | 0.6469 | 6.8286 | 0.4829 | 29.1031 | 2.5829 | 1.0000 | 0.0000 |
| TR_60_2008 | 2008 | TR | TR_REWET | 0.8467 | 0.2880 | 16.0826 | 0.2819 | 0.3242 | 0.6529 | 0.8210 | 5.1111 | 0.5873 | 9.1373 | 0.7137 | 24.5053 | 1.6284 | 0.9739 | 0.0261 |
| TR_67_2008 | 2008 | TR | TR_REWET | 0.8023 | 0.2682 | 24.4943 | 0.4490 | 0.2689 | 0.5228 | 0.7732 | 5.7945 | 0.6849 | 7.4338 | 0.5434 | 26.8042 | 2.3272 | 0.9132 | 0.0868 |
| TR_68_1995 | 1995 | TR | TR_BEFORE | 1.2122 | 0.3253 | 23.4650 | 0.4320 | 0.2517 | 0.4821 | 0.6142 | 5.3708 | 0.6244 | 7.5730 | 0.5573 | 21.0800 | 1.9972 | 0.8502 | 0.1498 |
| TR_7_1998 | 1998 | TR | TR_BEFORE | 0.4702 | 0.1779 | 27.1866 | 0.4937 | 0.2305 | 0.4323 | 0.8814 | 5.4151 | 0.6307 | 6.9240 | 0.4924 | 29.6275 | 2.7763 | 1.0000 | 0.0000 |
| TR_74_2008 | 2008 | TR | TR_REWET | 0.9642 | 0.3072 | 17.7857 | 0.3690 | 0.3013 | 0.5989 | 0.8375 | 5.1240 | 0.5891 | 8.5620 | 0.6562 | 19.0488 | 1.4137 | 0.7934 | 0.2066 |
| TR_75_1995 | 1995 | TR | TR_BEFORE | 0.4589 | 0.1712 | 23.1357 | 0.4415 | 0.2512 | 0.4811 | 0.7895 | 5.4115 | 0.6302 | 6.2536 | 0.4254 | 23.0465 | 2.1392 | 0.9809 | 0.0191 |
| TR_81_2008 | 2008 | TR | TR_REWET | 0.7396 | 0.2526 | 21.4607 | 0.4147 | 0.2555 | 0.4913 | 0.7634 | 5.8951 | 0.6993 | 7.1667 | 0.5167 | 22.6323 | 2.0753 | 0.7531 | 0.2469 |
| TR_82_1995 | 1995 | TR | TR_BEFORE | 0.3018 | 0.1188 | 25.2675 | 0.4728 | 0.2107 | 0.3856 | 0.8648 | 5.1732 | 0.5962 | 6.8622 | 0.4862 | 25.6371 | 3.0355 | 0.9961 | 0.0039 |
| TR_88_2008 | 2008 | TR | TR_REWET | 0.5316 | 0.1976 | 16.8609 | 0.3443 | 0.2717 | 0.5292 | 0.8609 | 5.9528 | 0.7075 | 6.3701 | 0.4370 | 18.1386 | 1.8393 | 0.8819 | 0.1181 |
| TR_89_1995 | 1995 | TR | TR_BEFORE | 0.5057 | 0.1743 | 24.6217 | 0.4643 | 0.2250 | 0.4194 | 0.8785 | 5.4513 | 0.6359 | 6.9348 | 0.4935 | 24.5392 | 2.5882 | 1.0000 | 0.0000 |
| TR_9_2008 | 2008 | TR | TR_REWET | 0.3422 | 0.1352 | 28.6097 | 0.5080 | 0.2304 | 0.4322 | 0.8509 | 5.6423 | 0.6632 | 7.0909 | 0.5091 | 25.9837 | 2.4253 | 1.0000 | 0.0000 |
| TR_95_2008 | 2008 | TR | TR_REWET | 0.5950 | 0.2155 | 23.5484 | 0.4326 | 0.2385 | 0.4511 | 0.8184 | 5.8144 | 0.6878 | 6.8788 | 0.4879 | 24.2827 | 2.1605 | 0.9760 | 0.0240 |
| TR_96_1995 | 1995 | TR | TR_BEFORE | 0.7600 | 0.2642 | 20.2976 | 0.3718 | 0.3051 | 0.6080 | 0.6758 | 5.6164 | 0.6595 | 8.0091 | 0.6009 | 27.2086 | 2.5385 | 0.9954 | 0.0046 |
| UL_A1_2010 | 2010 | UL | UL_REWET | 0.4428 | 0.1646 | 21.4817 | 0.3895 | 0.2869 | 0.5652 | 0.6667 | 5.2752 | 0.6107 | 9.1009 | 0.7101 | 17.6887 | 2.0229 | 1.0000 | 0.0000 |
| UL_A1a_2010 | 2010 | UL | UL_REWET | 0.6233 | 0.2205 | 11.6832 | 0.2421 | 0.3407 | 0.6918 | 0.6780 | 5.3367 | 0.6195 | 9.1649 | 0.7165 | 17.2720 | 1.3094 | 0.9388 | 0.0612 |
| UL_A1b_2010 | 2010 | UL | UL_REWET | 0.6283 | 0.2259 | 14.1648 | 0.2894 | 0.3268 | 0.6589 | 0.7051 | 5.5000 | 0.6429 | 9.0800 | 0.7080 | 17.2925 | 1.8127 | 1.0000 | 0.0000 |
| UL_A2_2010 | 2010 | UL | UL_REWET | 0.8965 | 0.2815 | 11.8260 | 0.2496 | 0.3513 | 0.7165 | 0.6858 | 4.9000 | 0.5571 | 8.9426 | 0.6943 | 20.3101 | 1.0693 | 1.0000 | 0.0000 |
| UL_A2a_2010 | 2010 | UL | UL_REWET | 0.8658 | 0.2776 | 11.9379 | 0.2549 | 0.3524 | 0.7193 | 0.6667 | 5.0390 | 0.5770 | 8.8571 | 0.6857 | 19.8189 | 1.0590 | 1.0000 | 0.0000 |
| UL_A2b_2010 | 2010 | UL | UL_REWET | 1.0810 | 0.3337 | 11.5794 | 0.2505 | 0.3328 | 0.6732 | 0.6784 | 4.7699 | 0.5386 | 9.2124 | 0.7212 | 21.0094 | 1.3897 | 1.0000 | 0.0000 |
| UL_A3_2010 | 2010 | UL | UL_REWET | 0.3445 | 0.1324 | 12.3530 | 0.2571 | 0.3554 | 0.7262 | 0.6667 | 5.0909 | 0.5844 | 9.0000 | 0.7000 | 15.3877 | 1.1399 | 1.0000 | 0.0000 |
| UL_A3a_2010 | 2010 | UL | UL_REWET | 0.4143 | 0.1533 | 12.1509 | 0.2490 | 0.3606 | 0.7384 | 0.7246 | 5.1594 | 0.5942 | 8.7101 | 0.6710 | 16.0491 | 1.3567 | 1.0000 | 0.0000 |
| UL_A3b_2010 | 2010 | UL | UL_REWET | 0.4017 | 0.1555 | 12.3427 | 0.2599 | 0.3729 | 0.7675 | 0.7778 | 5.3175 | 0.6168 | 7.6508 | 0.5651 | 16.5152 | 1.0556 | 1.0000 | 0.0000 |
| UL_A4_2010 | 2010 | UL | UL_REWET | 0.5525 | 0.1951 | 7.9326 | 0.1468 | 0.3809 | 0.7862 | 0.6737 | 3.9091 | 0.4156 | 8.9273 | 0.6927 | 18.1150 | 1.3818 | 0.4545 | 0.5455 |
| UL_A4a_2010 | 2010 | UL | UL_REWET | 0.7574 | 0.2466 | 9.6329 | 0.1951 | 0.3633 | 0.7448 | 0.6995 | 4.4454 | 0.4922 | 8.9488 | 0.6949 | 19.3651 | 1.2553 | 0.7611 | 0.2389 |
| UL_A4b_2010 | 2010 | UL | UL_REWET | 0.4056 | 0.1515 | 9.4639 | 0.1969 | 0.3790 | 0.7819 | 0.6667 | 5.1111 | 0.5873 | 9.0000 | 0.7000 | 15.9620 | 0.9602 | 0.9444 | 0.0556 |
| UL_A5_2010 | 2010 | UL | UL_REWET | 0.3903 | 0.1517 | 13.9628 | 0.2476 | 0.3431 | 0.6973 | 0.7284 | 4.8765 | 0.5538 | 8.4444 | 0.6444 | 16.2821 | 1.3666 | 0.7037 | 0.2963 |
| UL_A5a_2010 | 2010 | UL | UL_REWET | 0.2465 | 0.1000 | 11.5441 | 0.2041 | 0.3636 | 0.7457 | 0.7750 | 4.7407 | 0.5344 | 8.7468 | 0.6747 | 16.2247 | 1.2139 | 0.9259 | 0.0741 |
| UL_A5b_2010 | 2010 | UL | UL_REWET | 0.8445 | 0.2659 | 9.8862 | 0.2000 | 0.3642 | 0.7469 | 0.7041 | 4.3704 | 0.4815 | 8.7593 | 0.6759 | 19.9802 | 1.1666 | 0.8025 | 0.1975 |
| UL_B1_2010 | 2010 | UL | UL_REWET | 0.5400 | 0.2030 | 18.1761 | 0.3578 | 0.2711 | 0.5279 | 0.6904 | 5.2089 | 0.6013 | 9.4089 | 0.7409 | 17.4351 | 2.0526 | 0.9022 | 0.0978 |
| UL_B1a_2010 | 2010 | UL | UL_REWET | 0.4153 | 0.1597 | 19.2572 | 0.3552 | 0.2951 | 0.5845 | 0.6667 | 5.0833 | 0.5833 | 9.1667 | 0.7167 | 16.3911 | 1.5212 | 0.9907 | 0.0093 |
| UL_B1b_2010 | 2010 | UL | UL_REWET | 0.5674 | 0.2113 | 18.0525 | 0.3603 | 0.2915 | 0.5758 | 0.6987 | 5.3760 | 0.6251 | 8.8720 | 0.6872 | 17.1135 | 1.8905 | 0.8720 | 0.1280 |
| UL_B2_2010 | 2010 | UL | UL_REWET | 0.5022 | 0.1898 | 12.5075 | 0.2683 | 0.3814 | 0.7876 | 0.8376 | 5.4615 | 0.6374 | 6.9675 | 0.4968 | 17.3711 | 1.1114 | 1.0000 | 0.0000 |
| UL_B2a_2010 | 2010 | UL | UL_REWET | 0.4306 | 0.1679 | 13.9524 | 0.2953 | 0.3792 | 0.7823 | 0.8913 | 5.2265 | 0.6038 | 6.2346 | 0.4235 | 18.0543 | 1.0625 | 0.9890 | 0.0110 |

| | | | | | | | | | | | | | | | | | | |
|---|---|---|---|---|---|---|---|---|---|---|---|---|---|---|---|---|---|---|
| UL_B2b_2010 | 2010 | UL | UL_REWET | 0.5191 | 0.1962 | 11.9318 | 0.2561 | 0.3679 | 0.7556 | 0.6667 | 5.7647 | 0.6807 | 8.7647 | 0.6765 | 15.6346 | 1.0528 | 1.0000 | 0.0000 |
| UL_B3_2010 | 2010 | UL | UL_REWET | 0.4685 | 0.1792 | 17.9175 | 0.3714 | 0.3806 | 0.7855 | 0.6703 | 6.8087 | 0.8298 | 7.2842 | 0.5284 | 13.3661 | 0.8079 | 0.1694 | 0.8306 |
| UL_B3a_2010 | 2010 | UL | UL_REWET | 0.4847 | 0.1804 | 18.7059 | 0.3865 | 0.3776 | 0.7784 | 0.6667 | 6.7749 | 0.8250 | 7.1571 | 0.5157 | 13.6152 | 0.6917 | 0.0785 | 0.9215 |
| UL_B3b_2010 | 2010 | UL | UL_REWET | 0.3674 | 0.1453 | 17.0382 | 0.3437 | 0.3679 | 0.7557 | 0.7806 | 5.7722 | 0.6817 | 7.0676 | 0.5068 | 14.9433 | 0.7588 | 0.5949 | 0.4051 |
| UL_B4_2010 | 2010 | UL | UL_REWET | 0.3956 | 0.1569 | 13.9361 | 0.2933 | 0.3797 | 0.7835 | 0.9238 | 5.1857 | 0.5980 | 6.0429 | 0.4043 | 17.8717 | 1.0676 | 1.0000 | 0.0000 |
| UL_B4a_2010 | 2010 | UL | UL_REWET | 0.3204 | 0.1283 | 11.6311 | 0.2512 | 0.4015 | 0.8348 | 0.9266 | 4.9492 | 0.5642 | 5.6441 | 0.3644 | 16.2549 | 1.0573 | 1.0000 | 0.0000 |
| UL_B4b_2010 | 2010 | UL | UL_REWET | 0.4095 | 0.1620 | 12.3873 | 0.2684 | 0.3878 | 0.8025 | 0.8808 | 5.2847 | 0.6121 | 6.4453 | 0.4445 | 17.4644 | 1.0031 | 1.0000 | 0.0000 |
| UL_B5_2010 | 2010 | UL | UL_REWET | 0.3476 | 0.1394 | 8.1513 | 0.1554 | 0.3903 | 0.8084 | 0.6686 | 4.7751 | 0.5393 | 8.9822 | 0.6982 | 15.7562 | 1.1038 | 0.7160 | 0.2840 |
| UL_B5a_2010 | 2010 | UL | UL_REWET | 0.3856 | 0.1527 | 12.0067 | 0.2614 | 0.3808 | 0.7860 | 0.8293 | 5.1280 | 0.5897 | 7.0720 | 0.5072 | 17.3065 | 1.0305 | 0.9680 | 0.0320 |
| UL_B5b_2010 | 2010 | UL | UL_REWET | 0.3868 | 0.1534 | 12.7239 | 0.2730 | 0.3706 | 0.7621 | 0.8272 | 5.2698 | 0.6100 | 7.0582 | 0.5058 | 17.5708 | 1.1354 | 0.9947 | 0.0053 |
| UL_C1_2010 | 2010 | UL | UL_REWET | 1.3317 | 0.3908 | 10.4258 | 0.2258 | 0.3451 | 0.7021 | 0.6970 | 4.3137 | 0.4734 | 9.0392 | 0.7039 | 23.3594 | 1.0931 | 1.0000 | 0.0000 |
| UL_C1a_2010 | 2010 | UL | UL_REWET | 1.2984 | 0.3847 | 10.9184 | 0.2367 | 0.3380 | 0.6853 | 0.6726 | 4.4087 | 0.4870 | 9.1154 | 0.7115 | 22.8857 | 1.2045 | 1.0000 | 0.0000 |
| UL_C1b_2010 | 2010 | UL | UL_REWET | 1.1500 | 0.3503 | 12.3092 | 0.2670 | 0.3069 | 0.6120 | 0.6733 | 4.4232 | 0.4890 | 9.3983 | 0.7398 | 22.0696 | 1.5839 | 1.0000 | 0.0000 |
| UL_C2_2010 | 2010 | UL | UL_REWET | 0.4316 | 0.1635 | 11.8686 | 0.2504 | 0.3480 | 0.7088 | 0.6711 | 5.3816 | 0.6259 | 9.0395 | 0.7039 | 15.7998 | 1.2192 | 1.0000 | 0.0000 |
| UL_C2a_2010 | 2010 | UL | UL_REWET | 0.4431 | 0.1641 | 15.2518 | 0.2956 | 0.3491 | 0.7115 | 0.7500 | 5.7500 | 0.6786 | 8.6875 | 0.6688 | 15.0753 | 1.2463 | 1.0000 | 0.0000 |
| UL_C2b_2010 | 2010 | UL | UL_REWET | 0.5012 | 0.1919 | 10.7229 | 0.2314 | 0.3551 | 0.7255 | 0.6667 | 5.8837 | 0.6977 | 9.0698 | 0.7070 | 15.6272 | 1.0812 | 1.0000 | 0.0000 |
| UL_C3_2010 | 2010 | UL | UL_REWET | 1.1479 | 0.3388 | 9.9206 | 0.2122 | 0.3574 | 0.7311 | 0.8288 | 4.3248 | 0.4750 | 8.4872 | 0.6487 | 22.0986 | 1.0329 | 1.0000 | 0.0000 |
| UL_C3a_2010 | 2010 | UL | UL_REWET | 1.0942 | 0.3317 | 11.5271 | 0.2517 | 0.3605 | 0.7383 | 0.9451 | 4.4308 | 0.4901 | 7.6872 | 0.5687 | 22.3322 | 1.0636 | 1.0000 | 0.0000 |
| UL_C3b_2010 | 2010 | UL | UL_REWET | 0.4090 | 0.1618 | 8.5957 | 0.1590 | 0.3793 | 0.7826 | 0.7421 | 3.7075 | 0.3868 | 8.1792 | 0.6179 | 17.7220 | 1.5791 | 0.3396 | 0.6604 |
| UL_C4_2010 | 2010 | UL | UL_REWET | 0.8332 | 0.2563 | 9.2485 | 0.1888 | 0.3702 | 0.7611 | 0.7168 | 4.5917 | 0.5131 | 9.0000 | 0.7000 | 19.8320 | 0.9770 | 1.0000 | 0.0000 |
| UL_C4a_2010 | 2010 | UL | UL_REWET | 0.5624 | 0.1833 | 12.3374 | 0.2591 | 0.3474 | 0.7074 | 0.7578 | 4.5935 | 0.5134 | 8.5871 | 0.6587 | 20.3132 | 1.0830 | 1.0000 | 0.0000 |
| UL_C4b_2010 | 2010 | UL | UL_REWET | 0.5984 | 0.2062 | 11.6939 | 0.2512 | 0.3736 | 0.7690 | 0.9214 | 4.8477 | 0.5497 | 7.0530 | 0.5053 | 19.4598 | 1.0829 | 1.0000 | 0.0000 |
| UL_C5_2010 | 2010 | UL | UL_REWET | 0.3197 | 0.1247 | 12.7315 | 0.2415 | 0.3533 | 0.7214 | 0.8995 | 4.8769 | 0.5538 | 7.5538 | 0.5554 | 17.4479 | 1.2350 | 1.0000 | 0.0000 |
| UL_C5a_2010 | 2010 | UL | UL_REWET | 0.6499 | 0.2131 | 11.9246 | 0.2395 | 0.3561 | 0.7280 | 0.8374 | 4.8393 | 0.5485 | 8.0179 | 0.6018 | 18.9390 | 1.0986 | 1.0000 | 0.0000 |
| UL_C5b_2010 | 2010 | UL | UL_REWET | 1.1542 | 0.3405 | 9.6301 | 0.2032 | 0.3607 | 0.7387 | 0.6812 | 4.2667 | 0.4667 | 9.0000 | 0.7000 | 22.2344 | 0.9881 | 0.9733 | 0.0267 |
| UL_D1_2010 | 2010 | UL | UL_REWET | 0.8316 | 0.2568 | 15.0043 | 0.3033 | 0.3501 | 0.7139 | 0.8744 | 5.3140 | 0.6163 | 7.9174 | 0.5917 | 18.0170 | 1.1799 | 1.0000 | 0.0000 |
| UL_D1a_2010 | 2010 | UL | UL_REWET | 0.6128 | 0.1968 | 17.2612 | 0.3403 | 0.3563 | 0.7285 | 0.9057 | 5.7770 | 0.6824 | 7.4662 | 0.5466 | 15.8195 | 1.1740 | 1.0000 | 0.0000 |
| UL_D1b_2010 | 2010 | UL | UL_REWET | 1.0253 | 0.3122 | 9.8799 | 0.2104 | 0.3510 | 0.7158 | 0.7873 | 4.5880 | 0.5126 | 8.9614 | 0.6961 | 21.4313 | 1.0572 | 1.0000 | 0.0000 |
| UL_D2_2010 | 2010 | UL | UL_REWET | 0.5336 | 0.1803 | 14.5989 | 0.2779 | 0.3694 | 0.7592 | 0.8402 | 5.0397 | 0.5771 | 7.7845 | 0.5785 | 16.2313 | 1.2909 | 0.7086 | 0.2914 |
| UL_D2a_2010 | 2010 | UL | UL_REWET | 0.6781 | 0.2265 | 12.3460 | 0.2585 | 0.3666 | 0.7527 | 0.7886 | 5.4754 | 0.6393 | 8.0317 | 0.6032 | 17.4815 | 0.9994 | 1.0000 | 0.0000 |
| UL_D2b_2010 | 2010 | UL | UL_REWET | 0.3235 | 0.1287 | 14.9730 | 0.2973 | 0.3700 | 0.7607 | 0.8250 | 6.0750 | 0.7250 | 7.6433 | 0.5643 | 13.7684 | 1.1134 | 1.0000 | 0.0000 |
| UL_D3_2010 | 2010 | UL | UL_REWET | 0.6454 | 0.2151 | 13.6259 | 0.2905 | 0.3699 | 0.7604 | 0.9278 | 4.8861 | 0.5552 | 7.2025 | 0.5203 | 19.8996 | 1.0451 | 1.0000 | 0.0000 |
| UL_D3a_2010 | 2010 | UL | UL_REWET | 0.4090 | 0.1615 | 13.3104 | 0.2917 | 0.3818 | 0.7884 | 0.9598 | 4.9655 | 0.5665 | 5.7069 | 0.3707 | 18.4064 | 1.2115 | 1.0000 | 0.0000 |
| UL_D3b_2010 | 2010 | UL | UL_REWET | 0.6932 | 0.2222 | 11.9178 | 0.2300 | 0.3471 | 0.7066 | 0.8095 | 4.9216 | 0.5602 | 8.3137 | 0.6314 | 18.2825 | 1.1254 | 1.0000 | 0.0000 |
| UL_D4_2010 | 2010 | UL | UL_REWET | 0.3858 | 0.1395 | 9.8608 | 0.1987 | 0.3840 | 0.7937 | 0.6667 | 5.0286 | 0.5755 | 8.9429 | 0.6943 | 15.9611 | 0.8798 | 0.9714 | 0.0286 |
| UL_D4b_2010 | 2010 | UL | UL_REWET | 0.7292 | 0.2423 | 11.7529 | 0.2483 | 0.3535 | 0.7219 | 0.6957 | 5.4274 | 0.6325 | 8.5484 | 0.6548 | 17.4502 | 1.0705 | 0.8871 | 0.1129 |
| UL_D5_2010 | 2010 | UL | UL_REWET | 0.4540 | 0.1643 | 8.4548 | 0.1651 | 0.3838 | 0.7932 | 0.6667 | 4.5064 | 0.5009 | 9.0000 | 0.7000 | 16.9407 | 1.1285 | 0.7253 | 0.2747 |
| UL_D5a_2010 | 2010 | UL | UL_REWET | 0.7316 | 0.2433 | 12.5336 | 0.2593 | 0.3566 | 0.7290 | 0.6667 | 5.6175 | 0.6596 | 8.6503 | 0.6650 | 17.3269 | 0.9206 | 0.8142 | 0.1858 |
| UL_D5b_2010 | 2010 | UL | UL_REWET | 0.4140 | 0.1629 | 8.4061 | 0.1592 | 0.3704 | 0.7615 | 0.6882 | 4.2258 | 0.4608 | 8.9032 | 0.6903 | 16.8400 | 1.4577 | 0.4839 | 0.5161 |
| UL_E1_2002 | 2002 | UL | UL_BEFORE | 0.7098 | 0.2181 | 16.0978 | 0.3173 | 0.3649 | 0.7486 | 0.9829 | 5.4427 | 0.6347 | 6.9948 | 0.4995 | 16.5044 | 1.1488 | 1.0000 | 0.0000 |
| UL_E2_2002 | 2002 | UL | UL_BEFORE | 0.3870 | 0.1339 | 16.8781 | 0.3294 | 0.3734 | 0.7687 | 0.9472 | 5.8203 | 0.6886 | 6.5686 | 0.4569 | 13.5432 | 1.1789 | 1.0000 | 0.0000 |
| UL_E3_2002 | 2002 | UL | UL_BEFORE | 0.5832 | 0.1931 | 9.1392 | 0.1884 | 0.3770 | 0.7772 | 0.8769 | 4.8588 | 0.5513 | 7.5714 | 0.5571 | 17.3200 | 1.1001 | 1.0000 | 0.0000 |
| UL_E4_2002 | 2002 | UL | UL_BEFORE | 0.5272 | 0.1909 | 18.3148 | 0.3788 | 0.3762 | 0.7752 | 0.6667 | 6.6593 | 0.8085 | 7.2418 | 0.5242 | 14.0802 | 0.7053 | 0.1209 | 0.8791 |
| UL_E5_2002 | 2002 | UL | UL_BEFORE | 0.4966 | 0.1826 | 14.4619 | 0.3012 | 0.3743 | 0.7708 | 0.7018 | 6.1935 | 0.7419 | 7.6452 | 0.5645 | 14.5889 | 0.8472 | 0.5161 | 0.4839 |
| UL_F1_2002 | 2002 | UL | UL_BEFORE | 0.2008 | 0.0804 | 22.3817 | 0.4250 | 0.3657 | 0.7505 | 1.0000 | 6.5798 | 0.7971 | 6.2269 | 0.4227 | 11.7271 | 1.2278 | 1.0000 | 0.0000 |
| UL_F2_2002 | 2002 | UL | UL_BEFORE | 0.1540 | 0.0678 | 24.0551 | 0.4558 | 0.3645 | 0.7476 | 0.9986 | 6.8449 | 0.8350 | 6.1398 | 0.4140 | 11.0606 | 1.2333 | 1.0000 | 0.0000 |
| UL_F3_2002 | 2002 | UL | UL_BEFORE | 0.3386 | 0.1175 | 21.0070 | 0.4024 | 0.3701 | 0.7609 | 0.9968 | 6.3558 | 0.7651 | 6.2362 | 0.4236 | 12.6705 | 1.1811 | 1.0000 | 0.0000 |
| UL_F4_2002 | 2002 | UL | UL_BEFORE | 1.3721 | 0.3998 | 11.0866 | 0.2324 | 0.3432 | 0.6976 | 0.6667 | 4.3370 | 0.4767 | 9.0000 | 0.7000 | 23.3244 | 1.0436 | 1.0000 | 0.0000 |
| UL_F5_2002 | 2002 | UL | UL_BEFORE | 0.4081 | 0.1610 | 18.6075 | 0.3818 | 0.3692 | 0.7588 | 0.6667 | 6.7803 | 0.8258 | 7.3333 | 0.5333 | 13.5226 | 0.8001 | 0.1667 | 0.8333 |
| UL_G1_2002 | 2002 | UL | UL_BEFORE | 0.1819 | 0.0780 | 22.0589 | 0.4231 | 0.3749 | 0.7723 | 0.9542 | 6.5332 | 0.7905 | 5.9114 | 0.3911 | 11.7995 | 1.2537 | 0.9314 | 0.0686 |
| UL_G2_2002 | 2002 | UL | UL_BEFORE | 0.1515 | 0.0668 | 22.4097 | 0.4242 | 0.3732 | 0.7681 | 0.9968 | 6.6671 | 0.8096 | 5.7922 | 0.3792 | 10.7870 | 1.2205 | 1.0000 | 0.0000 |

| | | | | | | | | | | | | | | | | | | |
|---|---|---|---|---|---|---|---|---|---|---|---|---|---|---|---|---|---|---|
| UL_G3_2002 | 2002 | UL | UL_BEFORE | 0.6740 | 0.2194 | 18.9318 | 0.3665 | 0.3221 | 0.6480 | 0.7357 | 5.4881 | 0.6412 | 8.6294 | 0.6629 | 21.3533 | 2.3226 | 1.0000 | 0.0000 |
| UL_G4_2002 | 2002 | UL | UL_BEFORE | 0.4289 | 0.1590 | 22.2151 | 0.4254 | 0.2980 | 0.5911 | 0.6667 | 5.7872 | 0.6839 | 9.0157 | 0.7016 | 21.3642 | 3.1049 | 1.0000 | 0.0000 |
| UL_G5_2002 | 2002 | UL | UL_BEFORE | 0.4478 | 0.1728 | 18.8351 | 0.3899 | 0.3671 | 0.7539 | 0.6732 | 6.7395 | 0.8199 | 7.3908 | 0.5391 | 13.6837 | 0.8580 | 0.1379 | 0.8621 |
| UL_H1_2002 | 2002 | UL | UL_BEFORE | 0.6025 | 0.2094 | 18.3128 | 0.3559 | 0.3529 | 0.7205 | 0.8320 | 5.9481 | 0.7069 | 9.1567 | 0.7157 | 21.8812 | 1.8454 | 1.0000 | 0.0000 |
| UL_H2_2002 | 2002 | UL | UL_BEFORE | 0.5685 | 0.2059 | 18.5288 | 0.3617 | 0.3471 | 0.7067 | 0.7807 | 6.2554 | 0.7508 | 9.3131 | 0.7313 | 23.4353 | 2.3504 | 1.0000 | 0.0000 |
| UL_H3_2002 | 2002 | UL | UL_BEFORE | 0.4352 | 0.1667 | 21.2581 | 0.4145 | 0.2757 | 0.5386 | 0.5919 | 5.9609 | 0.7087 | 9.2736 | 0.7274 | 20.5998 | 2.5946 | 0.7865 | 0.2135 |
| UL_H4_2002 | 2002 | UL | UL_BEFORE | 0.5366 | 0.1899 | 21.5946 | 0.4142 | 0.3131 | 0.6267 | 0.7127 | 5.8781 | 0.6969 | 9.1493 | 0.7149 | 22.0959 | 2.8364 | 1.0000 | 0.0000 |
| UL_H5_2002 | 2002 | UL | UL_BEFORE | 0.5444 | 0.2015 | 17.5329 | 0.3632 | 0.3898 | 0.8072 | 0.7822 | 6.7990 | 0.8284 | 8.2063 | 0.6206 | 17.6433 | 1.0820 | 0.4801 | 0.5199 |
| UL_I1_2010 | 2010 | UL | UL_REWET | 0.2983 | 0.1178 | 10.6449 | 0.2146 | 0.3378 | 0.6849 | 0.8889 | 4.2500 | 0.4643 | 8.9583 | 0.6958 | 18.6235 | 1.3471 | 1.0000 | 0.0000 |
| UL_I2_2010 | 2010 | UL | UL_REWET | 1.1994 | 0.3512 | 10.8591 | 0.2257 | 0.3419 | 0.6945 | 0.8718 | 4.1088 | 0.4441 | 9.0000 | 0.7000 | 23.8991 | 1.1421 | 1.0000 | 0.0000 |
| UL_I3_2010 | 2010 | UL | UL_REWET | 0.4619 | 0.1667 | 12.4269 | 0.2636 | 0.3796 | 0.7832 | 0.9519 | 4.8416 | 0.5488 | 6.6600 | 0.4660 | 18.7784 | 1.1220 | 0.9604 | 0.0396 |
| UL_I4_2010 | 2010 | UL | UL_REWET | 0.9255 | 0.2880 | 15.0467 | 0.3151 | 0.3649 | 0.7486 | 0.6942 | 5.4980 | 0.6426 | 7.9918 | 0.5992 | 18.6758 | 0.8432 | 0.5181 | 0.4819 |
| UL_I5_2010 | 2010 | UL | UL_REWET | 0.6586 | 0.2139 | 16.5173 | 0.3286 | 0.3669 | 0.7533 | 0.8688 | 5.9098 | 0.7014 | 7.5164 | 0.5516 | 15.7008 | 1.2422 | 1.0000 | 0.0000 |
| UL_J1_2010 | 2010 | UL | UL_REWET | 0.3511 | 0.1209 | 19.0907 | 0.3840 | 0.3721 | 0.7655 | 0.9869 | 5.0847 | 0.5835 | 8.5495 | 0.6549 | 18.0677 | 1.1853 | 1.0000 | 0.0000 |
| UL_J2_2010 | 2010 | UL | UL_REWET | 0.7094 | 0.2200 | 8.7908 | 0.1785 | 0.3673 | 0.7543 | 0.9971 | 4.2678 | 0.4668 | 7.6940 | 0.5694 | 19.6634 | 1.2103 | 1.0000 | 0.0000 |
| UL_J3_2010 | 2010 | UL | UL_REWET | 0.5055 | 0.1894 | 10.0880 | 0.2150 | 0.3748 | 0.7720 | 0.7009 | 5.8077 | 0.6868 | 8.3595 | 0.6359 | 15.3429 | 1.1222 | 1.0000 | 0.0000 |
| UL_J4_2010 | 2010 | UL | UL_REWET | 0.4723 | 0.1782 | 13.4826 | 0.2872 | 0.3053 | 0.6085 | 0.7644 | 5.0133 | 0.5733 | 8.3425 | 0.6342 | 16.0651 | 2.0452 | 1.0000 | 0.0000 |
| UL_J5_2010 | 2010 | UL | UL_REWET | NA | NA | NA | NA | NA | NA | NA | NA | NA | NA | NA | NA | NA | NA | NA |
| UL_K1_2010 | 2010 | UL | UL_REWET | 0.1945 | 0.0810 | 19.3712 | 0.3938 | 0.3691 | 0.7586 | 0.9927 | 5.0220 | 0.5746 | 7.9880 | 0.5988 | 18.2952 | 1.1056 | 1.0000 | 0.0000 |
| UL_K2_2010 | 2010 | UL | UL_REWET | 0.2402 | 0.1016 | 17.4401 | 0.3628 | 0.4396 | 0.9244 | 0.9919 | 4.9855 | 0.5694 | 5.0000 | 0.3000 | 17.1052 | 1.2820 | 1.0000 | 0.0000 |
| UL_K3_2010 | 2010 | UL | UL_REWET | 0.1875 | 0.0808 | 15.2270 | 0.3249 | 0.4020 | 0.8360 | 0.8333 | 4.5000 | 0.5000 | 9.0000 | 0.7000 | 17.8000 | 1.3000 | 1.0000 | 0.0000 |
| UL_K4_2010 | 2010 | UL | UL_REWET | NA | NA | NA | NA | NA | NA | NA | NA | NA | NA | NA | NA | NA | 1.0000 | 0.0000 |
| UL_K5_2010 | 2010 | UL | UL_REWET | NA | NA | NA | NA | NA | NA | NA | NA | NA | NA | NA | NA | NA | NA | NA |
| UL_L1_2010 | 2010 | UL | UL_REWET | 0.1717 | 0.0740 | 21.3579 | 0.4052 | 0.3647 | 0.7481 | 0.9939 | 6.4495 | 0.7785 | 6.4037 | 0.4404 | 12.1358 | 1.2639 | 1.0000 | 0.0000 |
| UL_L2_2010 | 2010 | UL | UL_REWET | 0.1924 | 0.0777 | 24.4650 | 0.4640 | 0.3638 | 0.7460 | 1.0000 | 6.9091 | 0.8442 | 6.0909 | 0.4091 | 10.9879 | 1.2158 | 1.0000 | 0.0000 |
| UL_L3_2010 | 2010 | UL | UL_REWET | 0.7411 | 0.2349 | 9.6183 | 0.2030 | 0.3747 | 0.7718 | 0.7500 | 4.8649 | 0.5521 | 8.1892 | 0.6189 | 18.1441 | 0.9674 | 1.0000 | 0.0000 |
| UL_L4_2010 | 2010 | UL | UL_REWET | NA | NA | NA | NA | NA | NA | NA | NA | NA | NA | NA | NA | NA | NA | NA |
| UL_L5_2010 | 2010 | UL | UL_REWET | NA | NA | NA | NA | NA | NA | NA | NA | NA | NA | NA | NA | NA | 1.0000 | 0.0000 |
| UL_M1_2002 | 2002 | UL | UL_BEFORE | 0.1626 | 0.0703 | 24.4288 | 0.4639 | 0.3676 | 0.7551 | 0.9975 | 6.8864 | 0.8409 | 6.0276 | 0.4028 | 10.9313 | 1.2272 | 0.9962 | 0.0038 |
| UL_M2_2002 | 2002 | UL | UL_BEFORE | 0.1938 | 0.0794 | 21.2170 | 0.4058 | 0.3791 | 0.7821 | 0.9862 | 6.4613 | 0.7802 | 5.8750 | 0.3875 | 11.4024 | 1.2100 | 1.0000 | 0.0000 |
| UL_M3_2002 | 2002 | UL | UL_BEFORE | 0.1494 | 0.0656 | 8.7815 | 0.1708 | 0.4279 | 0.8969 | 0.9850 | 5.0000 | 0.5714 | 4.5056 | 0.2506 | 10.9098 | 1.1001 | 1.0000 | 0.0000 |
| UL_M4_2002 | 2002 | UL | UL_BEFORE | 0.3723 | 0.1471 | 9.9215 | 0.2116 | 0.3657 | 0.7506 | 0.7000 | 5.4000 | 0.6286 | 9.0000 | 0.7000 | 15.8760 | 0.9693 | 1.0000 | 0.0000 |
| UL_M5_2002 | 2002 | UL | UL_BEFORE | 0.2150 | 0.0923 | 7.8159 | 0.1495 | 0.3420 | 0.6947 | 1.0000 | 4.0000 | 0.4286 | 9.0000 | 0.7000 | 20.5000 | 1.5000 | 1.0000 | 0.0000 |
| UL_N1_2002 | 2002 | UL | UL_BEFORE | 0.1648 | 0.0700 | 8.1224 | 0.1566 | 0.4280 | 0.8973 | 0.9962 | 4.9432 | 0.5633 | 4.4205 | 0.2420 | 11.5178 | 1.1278 | 1.0000 | 0.0000 |
| UL_N2_2002 | 2002 | UL | UL_BEFORE | 0.6854 | 0.2152 | 10.9484 | 0.2256 | 0.3852 | 0.7965 | 0.9685 | 4.4670 | 0.4953 | 7.7389 | 0.5739 | 19.2615 | 1.1771 | 1.0000 | 0.0000 |
| UL_N3_2002 | 2002 | UL | UL_BEFORE | 0.2234 | 0.0855 | 23.0239 | 0.4405 | 0.3628 | 0.7438 | 0.9767 | 6.5385 | 0.7912 | 6.5275 | 0.4527 | 11.4932 | 1.2039 | 1.0000 | 0.0000 |
| UL_N4_2002 | 2002 | UL | UL_BEFORE | 0.3417 | 0.1365 | 10.7352 | 0.2210 | 0.3591 | 0.7349 | 0.7980 | 5.3030 | 0.6147 | 8.7692 | 0.6769 | 16.6566 | 1.1318 | 1.0000 | 0.0000 |
| UL_N5_2002 | 2002 | UL | UL_BEFORE | 0.6625 | 0.2391 | 12.6098 | 0.2747 | 0.3449 | 0.7016 | 0.7292 | 5.8750 | 0.6964 | 9.5000 | 0.7500 | 18.3282 | 1.6440 | 1.0000 | 0.0000 |
| UL_O1_2002 | 2002 | UL | UL_BEFORE | 0.1469 | 0.0648 | 23.1035 | 0.4428 | 0.3700 | 0.7605 | 1.0000 | 6.4444 | 0.7778 | 6.4118 | 0.4412 | 11.0500 | 1.2198 | 1.0000 | 0.0000 |
| UL_O2_2002 | 2002 | UL | UL_BEFORE | 0.3029 | 0.1130 | 20.8470 | 0.4075 | 0.3882 | 0.8035 | 1.0000 | 6.2500 | 0.7500 | 6.5472 | 0.4547 | 14.2191 | 1.2401 | 1.0000 | 0.0000 |
| UL_O3_2002 | 2002 | UL | UL_BEFORE | 0.2153 | 0.0869 | 23.8720 | 0.4557 | 0.3698 | 0.7602 | 1.0000 | 6.9752 | 0.8536 | 6.3554 | 0.4355 | 11.8636 | 1.2371 | 1.0000 | 0.0000 |
| UL_O4_2002 | 2002 | UL | UL_BEFORE | 0.2390 | 0.0915 | 21.0619 | 0.4025 | 0.3779 | 0.7793 | 0.9965 | 6.4901 | 0.7843 | 5.9797 | 0.3980 | 11.8837 | 1.1974 | 1.0000 | 0.0000 |
| UL_O5_2002 | 2002 | UL | UL_BEFORE | 0.3372 | 0.1266 | 9.6212 | 0.1960 | 0.4258 | 0.8919 | 0.9605 | 5.4359 | 0.6337 | 5.8533 | 0.3853 | 14.4936 | 1.1425 | 1.0000 | 0.0000 |
| UL_P1_2002 | 2002 | UL | UL_BEFORE | 0.3066 | 0.1161 | 20.1249 | 0.3939 | 0.3725 | 0.7665 | 0.9417 | 6.3250 | 0.7607 | 7.3250 | 0.5325 | 13.2766 | 1.3055 | 1.0000 | 0.0000 |
| UL_P2_2002 | 2002 | UL | UL_BEFORE | 0.2799 | 0.1086 | 22.3129 | 0.4310 | 0.3796 | 0.7831 | 1.0000 | 6.9556 | 0.8508 | 6.7556 | 0.4756 | 13.4456 | 1.2597 | 1.0000 | 0.0000 |
| UL_P3_2002 | 2002 | UL | UL_BEFORE | 0.2097 | 0.0857 | 23.7873 | 0.4547 | 0.3707 | 0.7623 | 0.9990 | 6.9527 | 0.8504 | 6.4107 | 0.4411 | 11.9500 | 1.2418 | 1.0000 | 0.0000 |
| UL_P4_2002 | 2002 | UL | UL_BEFORE | 0.1627 | 0.0705 | 24.2521 | 0.4600 | 0.3660 | 0.7513 | 1.0000 | 6.9237 | 0.8462 | 6.1043 | 0.4104 | 11.0002 | 1.2300 | 1.0000 | 0.0000 |
| UL_P5_2002 | 2002 | UL | UL_BEFORE | 0.2033 | 0.0876 | 7.8232 | 0.1497 | 0.3577 | 0.7316 | 1.0000 | 4.1667 | 0.4524 | 8.1667 | 0.6167 | 18.8833 | 1.4342 | 1.0000 | 0.0000 |
| UZ_101 | 1982 | UZ | UZ_BEFORE | 0.4134 | 0.1587 | 27.2027 | 0.4796 | 0.2298 | 0.4308 | 0.6560 | 6.0211 | 0.7173 | 6.4674 | 0.4467 | 24.2337 | 1.6831 | 0.9474 | 0.0526 |
| UZ_102 | 1982 | UZ | UZ_BEFORE | 0.3808 | 0.1502 | 25.3883 | 0.4689 | 0.2801 | 0.5491 | 0.6566 | 6.0896 | 0.7271 | 7.6053 | 0.5605 | 20.6354 | 2.1263 | 0.8507 | 0.1493 |
| UZ_103 | 1982 | UZ | UZ_BEFORE | 0.3919 | 0.1533 | 21.9519 | 0.4228 | 0.3120 | 0.6242 | 0.6737 | 6.2188 | 0.7455 | 8.0588 | 0.6059 | 18.8885 | 2.2712 | 0.6875 | 0.3125 |

| | | | | | | | | | | | | | | | | | | |
|---|---|---|---|---|---|---|---|---|---|---|---|---|---|---|---|---|---|---|
| UZ_104 | 1982 | UZ | UZ_BEFORE | 0.7824 | 0.2711 | 12.3476 | 0.2485 | 0.3397 | 0.6893 | 0.6966 | 6.1737 | 0.7391 | 8.7785 | 0.6778 | 24.2469 | 1.5724 | 0.9162 | 0.0838 |
| UZ_105 | 1982 | UZ | UZ_BEFORE | 0.7065 | 0.2485 | 16.6311 | 0.3253 | 0.2777 | 0.5435 | 0.7246 | 5.9554 | 0.7079 | 8.4370 | 0.6437 | 25.1934 | 1.8265 | 0.9444 | 0.0309 |
| UZ_106 | 1982 | UZ | UZ_BEFORE | 0.4699 | 0.1690 | 22.0498 | 0.4225 | 0.2444 | 0.4651 | 0.6247 | 6.0840 | 0.7263 | 8.4583 | 0.6458 | 20.5863 | 2.3131 | 0.9313 | 0.0687 |
| UZ_107 | 1982 | UZ | UZ_BEFORE | 0.1553 | 0.0601 | 25.8797 | 0.4768 | 0.1479 | 0.2378 | 0.6696 | 5.9397 | 0.7057 | 8.7717 | 0.6772 | 21.8704 | 1.2136 | 0.9957 | 0.0043 |
| UZ_108 | 1982 | UZ | UZ_BEFORE | 0.5295 | 0.1954 | 24.2781 | 0.4364 | 0.2551 | 0.4902 | 0.6667 | 5.9794 | 0.7113 | 7.0920 | 0.5092 | 23.7483 | 1.7016 | 0.9394 | 0.0606 |
| UZ_109 | 1982 | UZ | UZ_BEFORE | 0.1589 | 0.0617 | 25.5413 | 0.4732 | 0.1679 | 0.2849 | 0.6610 | 6.0924 | 0.7275 | 8.6828 | 0.6683 | 20.3902 | 1.1200 | 0.8870 | 0.1130 |
| UZ_110 | 1982 | UZ | UZ_BEFORE | 0.3732 | 0.1351 | 23.4269 | 0.4374 | 0.2123 | 0.3894 | 0.6536 | 6.1674 | 0.7382 | 8.3242 | 0.6324 | 20.7532 | 1.2967 | 0.8504 | 0.1496 |
| UZ_111 | 1982 | UZ | UZ_BEFORE | 0.6305 | 0.2247 | 20.2894 | 0.3967 | 0.2779 | 0.5438 | 0.7306 | 6.1558 | 0.7365 | 8.1818 | 0.6182 | 22.1772 | 2.2630 | 0.9397 | 0.0603 |
| UZ_112 | 1982 | UZ | UZ_BEFORE | 0.5852 | 0.2132 | 18.1802 | 0.3678 | 0.3439 | 0.6992 | 0.7216 | 6.6513 | 0.8073 | 7.9379 | 0.5938 | 18.8072 | 1.2010 | 0.5879 | 0.4121 |
| UZ_113 | 1982 | UZ | UZ_BEFORE | 0.6796 | 0.2413 | 15.7373 | 0.3266 | 0.3520 | 0.7183 | 0.7665 | 6.5395 | 0.7914 | 8.5423 | 0.6542 | 20.8718 | 1.3728 | 0.7371 | 0.2629 |
| UZ_114 | 1982 | UZ | UZ_BEFORE | 0.7460 | 0.2570 | 19.1498 | 0.3689 | 0.2895 | 0.5713 | 0.7701 | 5.9744 | 0.7106 | 8.0818 | 0.6082 | 23.9545 | 1.8739 | 0.9661 | 0.0339 |
| UZ_115 | 1982 | UZ | UZ_BEFORE | 0.3760 | 0.1483 | 22.9202 | 0.4405 | 0.3187 | 0.6399 | 0.6667 | 6.4182 | 0.7740 | 7.4444 | 0.5444 | 16.5769 | 1.3789 | 0.5120 | 0.4880 |
| UZ_116 | 1982 | UZ | UZ_BEFORE | 0.5651 | 0.2044 | 16.4840 | 0.3292 | 0.2880 | 0.5676 | 0.6774 | 6.1076 | 0.7297 | 8.5259 | 0.6526 | 20.9103 | 1.5399 | 0.8365 | 0.1635 |
| UZ_117 | 1982 | UZ | UZ_BEFORE | 0.6901 | 0.2430 | 16.8496 | 0.3244 | 0.3239 | 0.6521 | 0.7409 | 6.3067 | 0.7581 | 8.6244 | 0.6624 | 24.5883 | 1.5573 | 0.9454 | 0.0546 |
| UZ_118 | 1982 | UZ | UZ_BEFORE | 0.4315 | 0.1669 | 20.5778 | 0.4124 | 0.3511 | 0.7162 | 0.6715 | 6.6967 | 0.8138 | 7.5604 | 0.5560 | 15.7572 | 1.3632 | 0.3302 | 0.6698 |
| UZ_119 | 1982 | UZ | UZ_BEFORE | 0.4478 | 0.1726 | 19.3486 | 0.3968 | 0.3675 | 0.7548 | 0.6737 | 6.8646 | 0.8378 | 7.2766 | 0.5277 | 14.0291 | 0.8263 | 0.1563 | 0.8438 |
| UZ_120 | 1982 | UZ | UZ_BEFORE | 0.4365 | 0.1672 | 20.6841 | 0.4129 | 0.3424 | 0.6957 | 0.6814 | 6.5756 | 0.7965 | 7.6468 | 0.5647 | 16.4335 | 1.6702 | 0.4299 | 0.5701 |
| UZ_121 | 1982 | UZ | UZ_BEFORE | 0.4151 | 0.1618 | 19.5835 | 0.3998 | 0.3674 | 0.7545 | 0.6585 | 6.8113 | 0.8302 | 7.2212 | 0.5221 | 13.9711 | 0.9092 | 0.1455 | 0.8498 |
| UZ_122 | 1982 | UZ | UZ_BEFORE | 0.3534 | 0.1378 | 19.4778 | 0.3962 | 0.3630 | 0.7442 | 0.6610 | 6.0440 | 0.7206 | 7.5214 | 0.5521 | 15.8938 | 1.1799 | 0.5464 | 0.4481 |
| UZ_123 | 1982 | UZ | UZ_BEFORE | 0.7808 | 0.2704 | 12.9566 | 0.2624 | 0.3353 | 0.6789 | 0.6930 | 6.2212 | 0.7459 | 8.8241 | 0.6824 | 24.1291 | 1.4784 | 0.9266 | 0.0734 |
| UZ_124 | 1982 | UZ | UZ_BEFORE | 0.3708 | 0.1462 | 22.8038 | 0.4422 | 0.3193 | 0.6414 | 0.6691 | 6.2555 | 0.7508 | 8.2370 | 0.6237 | 18.7033 | 2.4940 | 0.6957 | 0.2971 |
| UZ_125 | 1982 | UZ | UZ_BEFORE | 0.3976 | 0.1570 | 19.5412 | 0.3967 | 0.3670 | 0.7536 | 0.6633 | 6.7736 | 0.8248 | 7.3725 | 0.5373 | 14.2655 | 1.1023 | 0.2336 | 0.7570 |
| UZ_126 | 1982 | UZ | UZ_BEFORE | 0.6797 | 0.2262 | 18.0765 | 0.3667 | 0.3473 | 0.7073 | 0.6523 | 6.0000 | 0.7143 | 7.8581 | 0.5858 | 17.6921 | 1.2663 | 0.4873 | 0.5063 |
| UZ_127 | 1982 | UZ | UZ_BEFORE | 0.4518 | 0.1707 | 20.0500 | 0.4016 | 0.3460 | 0.7043 | 0.6573 | 6.4966 | 0.7852 | 7.7862 | 0.5786 | 16.9023 | 1.6065 | 0.4533 | 0.5467 |
| UZ_128 | 1982 | UZ | UZ_BEFORE | 0.4203 | 0.1642 | 19.3951 | 0.3974 | 0.3719 | 0.7652 | 0.6603 | 6.8671 | 0.8382 | 7.1097 | 0.5110 | 13.4228 | 0.7928 | 0.1180 | 0.8758 |
| UZ_129 | 1982 | UZ | UZ_BEFORE | 0.4172 | 0.1623 | 19.5570 | 0.3994 | 0.3669 | 0.7533 | 0.6625 | 6.7964 | 0.8281 | 7.2561 | 0.5256 | 14.0640 | 0.9531 | 0.1607 | 0.8333 |
| UZ_130 | 1982 | UZ | UZ_BEFORE | 0.4451 | 0.1678 | 19.0319 | 0.3834 | 0.3629 | 0.7440 | 0.6608 | 6.5652 | 0.7950 | 7.6370 | 0.5637 | 15.2910 | 1.2393 | 0.4173 | 0.5755 |
| UZ_131 | 1982 | UZ | UZ_BEFORE | 0.4088 | 0.1570 | 20.7850 | 0.4117 | 0.3397 | 0.6894 | 0.6578 | 6.4000 | 0.7714 | 7.8435 | 0.5843 | 16.7127 | 1.7543 | 0.4917 | 0.5083 |
| UZ_132 | 1982 | UZ | UZ_BEFORE | 0.5755 | 0.2084 | 18.3103 | 0.3674 | 0.3065 | 0.6111 | 0.6984 | 6.2045 | 0.7435 | 7.7353 | 0.5735 | 17.3447 | 1.5049 | 0.6222 | 0.3778 |
| UZ_133 | 1982 | UZ | UZ_BEFORE | 0.6882 | 0.2439 | 18.3393 | 0.3633 | 0.3240 | 0.6523 | 0.7789 | 6.4896 | 0.7842 | 8.6538 | 0.6654 | 23.4044 | 1.6436 | 0.9479 | 0.0521 |
| UZ_134 | 1982 | UZ | UZ_BEFORE | 0.4744 | 0.1751 | 18.8671 | 0.3854 | 0.3508 | 0.7154 | 0.6607 | 6.5041 | 0.7863 | 7.3364 | 0.5336 | 16.3491 | 1.1258 | 0.3798 | 0.6202 |
| UZ_135 | 1982 | UZ | UZ_BEFORE | 0.3377 | 0.1340 | 20.9467 | 0.4155 | 0.3202 | 0.6434 | 0.6649 | 5.9740 | 0.7106 | 8.2889 | 0.6289 | 18.0863 | 2.2496 | 0.7938 | 0.2062 |
| UZ_136 | 1982 | UZ | UZ_BEFORE | 0.4016 | 0.1530 | 22.0435 | 0.4287 | 0.3162 | 0.6341 | 0.6558 | 6.1437 | 0.7348 | 8.3476 | 0.6348 | 19.1042 | 2.5091 | 0.7515 | 0.2426 |
| UZ_137 | 1982 | UZ | UZ_BEFORE | 0.4246 | 0.1616 | 20.7182 | 0.4132 | 0.3449 | 0.7015 | 0.6630 | 6.4211 | 0.7744 | 7.8424 | 0.5842 | 16.6061 | 1.6727 | 0.4740 | 0.5208 |
| UZ_138 | 1982 | UZ | UZ_BEFORE | 0.5973 | 0.2138 | 17.4375 | 0.3557 | 0.2668 | 0.5178 | 0.7292 | 6.0538 | 0.7220 | 8.2874 | 0.6287 | 24.8884 | 2.1539 | 0.9692 | 0.0308 |
| UZ_139 | 1982 | UZ | UZ_BEFORE | 0.4376 | 0.1672 | 20.8725 | 0.4162 | 0.3341 | 0.6761 | 0.6382 | 6.5065 | 0.7866 | 7.7292 | 0.5729 | 17.4068 | 1.6450 | 0.4937 | 0.5063 |
| UZ_140 | 1982 | UZ | UZ_BEFORE | 0.4034 | 0.1568 | 20.3183 | 0.4090 | 0.3521 | 0.7185 | 0.6539 | 6.5266 | 0.7895 | 7.5257 | 0.5526 | 15.5729 | 1.3843 | 0.3598 | 0.6402 |
| UZ_141 | 1982 | UZ | UZ_BEFORE | 0.3862 | 0.1528 | 20.6656 | 0.4151 | 0.3560 | 0.7276 | 0.6571 | 6.6215 | 0.8031 | 7.5353 | 0.5535 | 15.3356 | 1.3910 | 0.3258 | 0.6742 |
| UZ_142 | 1982 | UZ | UZ_BEFORE | 0.6312 | 0.2217 | 20.7388 | 0.4136 | 0.3111 | 0.6221 | 0.7839 | 6.0109 | 0.7158 | 8.0000 | 0.6000 | 20.7453 | 2.0560 | 0.8280 | 0.1720 |
| UZ_143 | 1982 | UZ | UZ_BEFORE | 0.3094 | 0.1221 | 16.8877 | 0.3496 | 0.3207 | 0.6445 | 0.5121 | 5.7987 | 0.6855 | 6.0222 | 0.4022 | 15.6782 | 1.2796 | 0.3548 | 0.6452 |
| UZ_144 | 1982 | UZ | UZ_BEFORE | 0.4500 | 0.1716 | 19.6590 | 0.3999 | 0.3572 | 0.7306 | 0.6423 | 6.6867 | 0.8124 | 7.3226 | 0.5323 | 14.6723 | 1.1049 | 0.2941 | 0.7059 |
| UZ_145 | 1982 | UZ | UZ_BEFORE | 0.4715 | 0.1784 | 19.9964 | 0.4057 | 0.3498 | 0.7132 | 0.6463 | 6.7365 | 0.8195 | 7.5070 | 0.5507 | 15.3592 | 1.1880 | 0.3221 | 0.6779 |
| UZ_146 | 1982 | UZ | UZ_BEFORE | 0.6420 | 0.2298 | 15.2455 | 0.3099 | 0.2915 | 0.5759 | 0.6582 | 6.0881 | 0.7269 | 8.5954 | 0.6595 | 22.5628 | 1.8133 | 0.9239 | 0.0761 |
| UZ_147 | 1982 | UZ | UZ_BEFORE | 0.6356 | 0.2260 | 20.5795 | 0.4020 | 0.2695 | 0.5242 | 0.7539 | 5.9800 | 0.7114 | 7.8444 | 0.5844 | 22.1638 | 2.1289 | 0.9600 | 0.0400 |
| UZ_148 | 1982 | UZ | UZ_BEFORE | 0.4276 | 0.1656 | 19.7906 | 0.4022 | 0.3519 | 0.7180 | 0.6524 | 6.7212 | 0.8173 | 7.3046 | 0.5305 | 14.3786 | 1.0590 | 0.2771 | 0.7229 |
| UZ_149 | 1982 | UZ | UZ_BEFORE | 0.4290 | 0.1646 | 20.3698 | 0.4089 | 0.3356 | 0.6796 | 0.6468 | 6.5045 | 0.7864 | 7.6804 | 0.5680 | 16.4826 | 1.6580 | 0.4737 | 0.5263 |
| UZ_150 | 1982 | UZ | UZ_BEFORE | 0.6266 | 0.2244 | 16.5235 | 0.3343 | 0.3015 | 0.5994 | 0.6208 | 6.2722 | 0.7532 | 8.0500 | 0.6050 | 20.2889 | 1.4296 | 0.7647 | 0.2353 |
| UZ_151 | 1982 | UZ | UZ_BEFORE | 0.5171 | 0.1931 | 18.3918 | 0.3763 | 0.3517 | 0.7175 | 0.6723 | 6.7514 | 0.8216 | 7.4311 | 0.5431 | 15.2938 | 0.9077 | 0.3202 | 0.6798 |
| UZ_152 | 1982 | UZ | UZ_BEFORE | 0.6038 | 0.2175 | 20.3952 | 0.3885 | 0.2645 | 0.5123 | 0.7006 | 6.1000 | 0.7286 | 7.3419 | 0.5342 | 22.1000 | 1.6896 | 0.8882 | 0.1118 |
| UZ_153 | 1982 | UZ | UZ_BEFORE | 0.4659 | 0.1774 | 21.3756 | 0.4144 | 0.2977 | 0.5905 | 0.6578 | 6.3646 | 0.7664 | 6.9296 | 0.4930 | 17.6704 | 1.2581 | 0.5833 | 0.4167 |
| UZ_154 | 1982 | UZ | UZ_BEFORE | 0.5022 | 0.1874 | 22.1176 | 0.4194 | 0.2563 | 0.4931 | 0.6364 | 6.0752 | 0.7250 | 7.0476 | 0.5048 | 20.9978 | 1.7165 | 0.8872 | 0.1128 |

| | | | | | | | | | | | | | | | | | | |
|---|---|---|---|---|---|---|---|---|---|---|---|---|---|---|---|---|---|---|
| UZ_155 | 1982 | UZ | UZ_BEFORE | 0.5316 | 0.1937 | 20.5676 | 0.3994 | 0.2398 | 0.4541 | 0.6582 | 6.2167 | 0.7452 | 7.8182 | 0.5818 | 20.6951 | 1.4094 | 0.8760 | 0.1240 |
| UZ_401 | 2013 | UZ | UZ_REWET | 0.2646 | 0.1030 | 22.8924 | 0.4435 | 0.2883 | 0.5684 | 0.4740 | 5.3401 | 0.6200 | 8.1064 | 0.6106 | 21.6266 | 1.8807 | 0.9335 | 0.0665 |
| UZ_402 | 2013 | UZ | UZ_REWET | 0.2661 | 0.1062 | 22.3442 | 0.4355 | 0.2778 | 0.5435 | 0.4422 | 5.3430 | 0.6204 | 7.6457 | 0.5646 | 20.8101 | 1.7500 | 0.9151 | 0.0849 |
| UZ_403 | 2013 | UZ | UZ_REWET | 0.4886 | 0.1780 | 18.8832 | 0.3824 | 0.3031 | 0.6032 | 0.6103 | 5.9711 | 0.7102 | 7.4759 | 0.5476 | 17.1007 | 1.1442 | 0.6163 | 0.3837 |
| UZ_404 | 2013 | UZ | UZ_REWET | 0.5299 | 0.1878 | 19.8566 | 0.3974 | 0.3151 | 0.6313 | 0.7215 | 6.0797 | 0.7257 | 8.5765 | 0.6577 | 21.1605 | 1.8077 | 0.8405 | 0.1595 |
| UZ_405 | 2013 | UZ | UZ_REWET | 0.5442 | 0.1961 | 17.6208 | 0.3593 | 0.3195 | 0.6417 | 0.8180 | 6.4190 | 0.7741 | 9.0380 | 0.7038 | 20.8584 | 1.7880 | 0.8853 | 0.1147 |
| UZ_406 | 2013 | UZ | UZ_REWET | 0.7031 | 0.2443 | 17.8436 | 0.3682 | 0.3014 | 0.5992 | 0.8428 | 5.9989 | 0.7141 | 8.4650 | 0.6465 | 22.3321 | 1.8760 | 1.0000 | 0.0000 |
| UZ_407 | 2013 | UZ | UZ_REWET | 0.3459 | 0.1334 | 21.3353 | 0.4201 | 0.3053 | 0.6084 | 0.6470 | 5.5109 | 0.6444 | 8.1830 | 0.6183 | 17.8804 | 1.1546 | 0.9261 | 0.0739 |
| UZ_408 | 2013 | UZ | UZ_REWET | 0.4526 | 0.1657 | 20.9635 | 0.4108 | 0.2778 | 0.5437 | 0.6758 | 6.0262 | 0.7180 | 8.4071 | 0.6407 | 21.1356 | 1.5493 | 0.8862 | 0.1138 |
| UZ_409 | 2013 | UZ | UZ_REWET | 0.5570 | 0.1925 | 17.8099 | 0.3638 | 0.2838 | 0.5578 | 0.6233 | 5.8005 | 0.6858 | 8.1071 | 0.6107 | 20.2533 | 1.2961 | 0.8860 | 0.1140 |
| UZ_410 | 2013 | UZ | UZ_REWET | 1.0045 | 0.3030 | 15.0696 | 0.3088 | 0.3279 | 0.6617 | 0.7920 | 5.2165 | 0.6024 | 8.8217 | 0.6822 | 21.8642 | 1.2259 | 0.8563 | 0.1422 |
| UZ_411 | 2013 | UZ | UZ_REWET | 0.6495 | 0.2226 | 25.6616 | 0.4014 | 0.3101 | 0.6197 | 0.8663 | 6.2722 | 0.7532 | 9.2475 | 0.7248 | 23.5600 | 1.5713 | 0.8752 | 0.0416 |
| UZ_412 | 2013 | UZ | UZ_REWET | 0.7489 | 0.2461 | 22.3412 | 0.3763 | 0.3348 | 0.6779 | 0.7819 | 5.8284 | 0.6898 | 8.8166 | 0.6817 | 22.1337 | 1.2841 | 0.8387 | 0.1075 |
| UZ_413 | 2013 | UZ | UZ_REWET | 0.5886 | 0.2093 | 18.1605 | 0.3727 | 0.3573 | 0.7308 | 0.8427 | 6.1929 | 0.7418 | 9.0966 | 0.7097 | 21.9819 | 1.5790 | 0.9723 | 0.0277 |
| UZ_414 | 2013 | UZ | UZ_REWET | 0.2936 | 0.1157 | 20.6468 | 0.4102 | 0.3235 | 0.6512 | 0.6670 | 5.2888 | 0.6127 | 8.2123 | 0.6212 | 17.8810 | 1.7268 | 0.9756 | 0.0244 |
| UZ_415 | 2013 | UZ | UZ_REWET | 0.9227 | 0.2957 | 16.8480 | 0.3436 | 0.3113 | 0.6224 | 0.8225 | 5.6087 | 0.6584 | 8.4156 | 0.6416 | 23.0227 | 1.5874 | 0.9377 | 0.0623 |
| UZ_416 | 2013 | UZ | UZ_REWET | 0.7173 | 0.2499 | 16.7815 | 0.3503 | 0.3580 | 0.7323 | 0.8921 | 6.3877 | 0.7697 | 9.0175 | 0.7018 | 22.8127 | 1.7042 | 0.9662 | 0.0338 |
| UZ_417 | 2013 | UZ | UZ_REWET | 0.8270 | 0.2665 | 16.4594 | 0.3375 | 0.3373 | 0.6838 | 0.7874 | 5.4668 | 0.6381 | 8.7493 | 0.6749 | 21.6246 | 1.4231 | 0.9654 | 0.0346 |
| UZ_418 | 2013 | UZ | UZ_REWET | 0.9421 | 0.2952 | 14.9520 | 0.3114 | 0.3572 | 0.7305 | 0.7913 | 5.4104 | 0.6301 | 8.8371 | 0.6837 | 22.0040 | 1.1491 | 0.8976 | 0.1024 |
| UZ_419 | 2013 | UZ | UZ_REWET | 0.8323 | 0.2628 | 16.5548 | 0.3376 | 0.3699 | 0.7604 | 0.7272 | 5.0293 | 0.5756 | 8.6519 | 0.6652 | 20.9802 | 1.0426 | 0.9713 | 0.0281 |
| UZ_420 | 2013 | UZ | UZ_REWET | 0.6220 | 0.2187 | 11.9782 | 0.2629 | 0.2897 | 0.5716 | 0.6559 | 6.9468 | 0.8495 | 7.6238 | 0.5624 | 15.5349 | 1.1043 | 0.9744 | 0.0256 |
| UZ_421 | 2013 | UZ | UZ_REWET | 0.6410 | 0.2194 | 13.7090 | 0.2862 | 0.3697 | 0.7600 | 0.6306 | 6.3986 | 0.7712 | 7.8191 | 0.5819 | 16.0870 | 0.8659 | 0.5250 | 0.4741 |
| UZ_422 | 2013 | UZ | UZ_REWET | 0.7486 | 0.2470 | 12.4309 | 0.2615 | 0.3743 | 0.7708 | 0.6133 | 5.8000 | 0.6857 | 8.2195 | 0.6220 | 17.2928 | 0.8132 | 0.7818 | 0.2182 |
| UZ_423 | 2013 | UZ | UZ_REWET | 0.9122 | 0.2865 | 13.8570 | 0.2919 | 0.3705 | 0.7617 | 0.6923 | 5.5596 | 0.6514 | 8.3096 | 0.6310 | 19.2503 | 0.8928 | 0.7459 | 0.2541 |
| UZ_424 | 2013 | UZ | UZ_REWET | 0.6265 | 0.2151 | 11.9989 | 0.2581 | 0.3777 | 0.7789 | 0.6287 | 6.2694 | 0.7528 | 7.8803 | 0.5880 | 14.9854 | 0.8170 | 0.8626 | 0.1374 |
| UZ_425 | 2013 | UZ | UZ_REWET | 0.5643 | 0.1993 | 14.4237 | 0.2963 | 0.3832 | 0.7917 | 0.6353 | 6.0971 | 0.7282 | 7.9497 | 0.5950 | 16.5202 | 0.8572 | 0.5997 | 0.4003 |
| UZ_426 | 2013 | UZ | UZ_REWET | 0.7233 | 0.2387 | 14.6493 | 0.3055 | 0.3510 | 0.7160 | 0.6652 | 5.7367 | 0.6767 | 8.1503 | 0.6150 | 18.2827 | 0.8944 | 0.8327 | 0.1660 |
| UZ_427 | 2013 | UZ | UZ_REWET | 0.6388 | 0.2174 | 13.6184 | 0.2861 | 0.3985 | 0.8277 | 0.6471 | 6.1500 | 0.7357 | 8.0000 | 0.6000 | 16.0073 | 0.7986 | 0.7750 | 0.2250 |
| UZ_428 | 2013 | UZ | UZ_REWET | 0.4774 | 0.1799 | 14.8028 | 0.3103 | 0.3503 | 0.7142 | 0.6656 | 6.3738 | 0.7677 | 8.4902 | 0.6490 | 16.7074 | 1.0359 | 1.0000 | 0.0000 |
| UZ_429 | 2013 | UZ | UZ_REWET | 0.4840 | 0.1801 | 14.6960 | 0.3059 | 0.4050 | 0.8429 | 0.7020 | 6.3155 | 0.7594 | 7.9988 | 0.5999 | 14.5064 | 0.7014 | 0.8861 | 0.1139 |
| UZ_430 | 2013 | UZ | UZ_REWET | 0.4869 | 0.1801 | 12.0238 | 0.2500 | 0.4077 | 0.8494 | 0.5995 | 6.1883 | 0.7412 | 8.0000 | 0.6000 | 15.0800 | 0.7570 | 0.8441 | 0.1559 |
| UZ_431 | 2013 | UZ | UZ_REWET | 0.4850 | 0.1827 | 12.1931 | 0.2603 | 0.4158 | 0.8683 | 0.6648 | 6.8950 | 0.8421 | 7.8246 | 0.5825 | 12.4224 | 0.6232 | 0.8037 | 0.1963 |
| UZ_432 | 2013 | UZ | UZ_REWET | 0.4372 | 0.1661 | 17.7080 | 0.3672 | 0.3613 | 0.7403 | 0.6522 | 6.5374 | 0.7911 | 7.2116 | 0.5212 | 15.2468 | 0.7910 | 0.3151 | 0.6842 |
| UZ_433 | 2013 | UZ | UZ_REWET | 0.7071 | 0.2412 | 16.1920 | 0.3322 | 0.3140 | 0.6287 | 0.6800 | 5.8930 | 0.6990 | 8.5814 | 0.6581 | 20.0897 | 1.3652 | 0.7863 | 0.2137 |
| UZ_434 | 2013 | UZ | UZ_REWET | 0.5170 | 0.1822 | 15.4215 | 0.3170 | 0.3794 | 0.7829 | 0.5842 | 6.1388 | 0.7341 | 7.9960 | 0.5996 | 16.1142 | 0.9212 | 0.8584 | 0.1416 |
| UZ_435 | 2013 | UZ | UZ_REWET | 0.5385 | 0.1905 | 17.5803 | 0.3614 | 0.3519 | 0.7180 | 0.6667 | 5.9401 | 0.7057 | 7.8571 | 0.5857 | 16.9855 | 0.8893 | 0.7503 | 0.2497 |
| UZ_436 | 2013 | UZ | UZ_REWET | 0.5711 | 0.1982 | 16.9805 | 0.3519 | 0.3377 | 0.6847 | 0.6655 | 5.6927 | 0.6704 | 7.6672 | 0.5667 | 17.5148 | 0.8904 | 0.7706 | 0.2294 |
| UZ_437 | 2013 | UZ | UZ_REWET | 0.4697 | 0.1747 | 15.4541 | 0.3246 | 0.3321 | 0.6713 | 0.6661 | 6.4440 | 0.7777 | 7.6879 | 0.5688 | 16.6367 | 0.8195 | 0.7780 | 0.2220 |
| UZ_438 | 2013 | UZ | UZ_REWET | 0.5864 | 0.2056 | 16.6489 | 0.3488 | 0.3004 | 0.5967 | 0.6662 | 6.2019 | 0.7431 | 7.6650 | 0.5665 | 17.3032 | 0.9313 | 0.7334 | 0.2653 |
| UZ_439 | 2013 | UZ | UZ_REWET | 0.5999 | 0.2042 | 17.3007 | 0.3547 | 0.3400 | 0.6901 | 0.6667 | 5.5366 | 0.6481 | 8.0541 | 0.6054 | 18.1501 | 0.9136 | 0.9512 | 0.0488 |
| UZ_440 | 2013 | UZ | UZ_REWET | 0.5955 | 0.2106 | 16.1332 | 0.3379 | 0.3031 | 0.6032 | 0.6663 | 5.8273 | 0.6896 | 8.0000 | 0.6000 | 17.9040 | 0.9405 | 0.9002 | 0.0998 |
| UZ_441 | 2013 | UZ | UZ_REWET | 0.6235 | 0.2107 | 17.3531 | 0.3544 | 0.3607 | 0.7387 | 0.6662 | 5.4109 | 0.6301 | 8.1816 | 0.6182 | 18.3787 | 0.9123 | 0.8975 | 0.1025 |
| UZ_442 | 2013 | UZ | UZ_REWET | 0.4939 | 0.1862 | 16.5743 | 0.3458 | 0.2723 | 0.5307 | 0.6193 | 6.2031 | 0.7433 | 7.7030 | 0.5703 | 16.3623 | 0.9615 | 0.9139 | 0.0861 |
| UZ_443 | 2013 | UZ | UZ_REWET | 0.6341 | 0.2160 | 16.8906 | 0.3492 | 0.2967 | 0.5881 | 0.6297 | 5.6799 | 0.6686 | 8.0025 | 0.6002 | 18.9047 | 1.0019 | 0.9096 | 0.0904 |
| UZ_444 | 2013 | UZ | UZ_REWET | 0.8951 | 0.2790 | 14.7081 | 0.3078 | 0.3179 | 0.6380 | 0.6125 | 5.1565 | 0.5938 | 8.1034 | 0.6103 | 20.3490 | 0.9671 | 0.8530 | 0.1459 |
| UZ_445 | 2013 | UZ | UZ_REWET | 0.4859 | 0.1769 | 18.8026 | 0.3717 | 0.3139 | 0.6285 | 0.6667 | 5.9642 | 0.7092 | 8.0868 | 0.6087 | 16.6378 | 0.9793 | 0.9311 | 0.0689 |
| UZ_446 | 2013 | UZ | UZ_REWET | 0.7767 | 0.2544 | 15.6417 | 0.3205 | 0.2879 | 0.5675 | 0.6811 | 5.3493 | 0.6213 | 8.5892 | 0.6589 | 20.6105 | 1.0550 | 0.9679 | 0.0321 |
| UZ_447 | 2013 | UZ | UZ_REWET | 0.4540 | 0.1659 | 20.1076 | 0.3973 | 0.2931 | 0.5797 | 0.6445 | 5.7509 | 0.6787 | 8.0765 | 0.6076 | 17.9267 | 0.9665 | 0.8744 | 0.1244 |
| UZ_448 | 2013 | UZ | UZ_REWET | 0.5862 | 0.2122 | 15.6135 | 0.3299 | 0.2739 | 0.5345 | 0.6434 | 6.0577 | 0.7225 | 7.6667 | 0.5667 | 15.6696 | 0.7731 | 0.8798 | 0.1202 |
| UZ_449 | 2013 | UZ | UZ_REWET | 0.3846 | 0.1501 | 18.2058 | 0.3656 | 0.2466 | 0.4701 | 0.6237 | 6.3589 | 0.7656 | 7.3349 | 0.5335 | 15.1667 | 0.8915 | 0.8840 | 0.0696 |
| UZ_450 | 2013 | UZ | UZ_REWET | 0.5992 | 0.2047 | 17.4825 | 0.3521 | 0.3036 | 0.6045 | 0.6462 | 5.7349 | 0.6764 | 8.0557 | 0.6056 | 18.1567 | 1.0187 | 0.9124 | 0.0859 |

| | | | | | | | | | | | | | | | | | | |
|---|---|---|---|---|---|---|---|---|---|---|---|---|---|---|---|---|---|---|
| UZ_451 | 2013 | UZ | UZ_REWET | 0.4307 | 0.1601 | 22.0815 | 0.4151 | 0.2887 | 0.5693 | 0.7071 | 5.6909 | 0.6701 | 8.4664 | 0.6466 | 17.5971 | 1.0945 | 0.8765 | 0.0617 |
| UZ_452 | 2013 | UZ | UZ_REWET | 0.3521 | 0.1396 | 19.9678 | 0.3992 | 0.2528 | 0.4847 | 0.6329 | 6.0245 | 0.7178 | 7.6087 | 0.5609 | 16.7319 | 0.9440 | 0.9490 | 0.0510 |
| UZ_453 | 2013 | UZ | UZ_REWET | 0.4496 | 0.1690 | 16.5194 | 0.3479 | 0.2420 | 0.4594 | 0.6512 | 6.2882 | 0.7555 | 7.5470 | 0.5547 | 17.5335 | 0.9486 | 0.9792 | 0.0208 |
| UZ_454 | 2013 | UZ | UZ_REWET | 0.3820 | 0.1487 | 19.6123 | 0.3931 | 0.2974 | 0.5897 | 0.6664 | 5.7081 | 0.6726 | 7.9404 | 0.5940 | 16.9956 | 0.9524 | 0.9763 | 0.0237 |
| UZ_455 | 2013 | UZ | UZ_REWET | 0.3484 | 0.1340 | 18.1140 | 0.3627 | 0.2702 | 0.5258 | 0.6837 | 5.4040 | 0.6291 | 8.3889 | 0.6389 | 19.4028 | 0.9686 | 1.0000 | 0.0000 |
| VK_A10_1 | 2008 | VK | VK_TSR | 0.3401 | 0.1331 | 22.6728 | 0.4383 | 0.2987 | 0.5928 | 0.5684 | 5.5011 | 0.6430 | 8.3958 | 0.6396 | 20.8115 | 2.2395 | 1.0000 | 0.0000 |
| VK_A10_2 | 2008 | VK | VK_TSR | 0.3325 | 0.1320 | 22.6229 | 0.4329 | 0.3109 | 0.6217 | 0.6043 | 5.5093 | 0.6442 | 8.1667 | 0.6167 | 21.1243 | 2.1941 | 0.9785 | 0.0215 |
| VK_A10_3 | 2008 | VK | VK_TSR | 0.2996 | 0.1199 | 23.8194 | 0.4530 | 0.3097 | 0.6187 | 0.5436 | 5.3267 | 0.6181 | 7.6805 | 0.5681 | 23.0576 | 1.6562 | 1.0000 | 0.0000 |
| VK_A10_4 | 2008 | VK | VK_TSR | 0.4334 | 0.1646 | 21.9014 | 0.4228 | 0.2911 | 0.5749 | 0.6226 | 5.4340 | 0.6334 | 7.8571 | 0.5857 | 20.7839 | 1.5545 | 0.9811 | 0.0189 |
| VK_A10_5 | 2008 | VK | VK_TSR | 0.4760 | 0.1795 | 20.7498 | 0.3977 | 0.2895 | 0.5711 | 0.6308 | 5.5124 | 0.6446 | 7.7482 | 0.5748 | 21.8187 | 1.6197 | 0.9462 | 0.0538 |
| VK_A5_1 | 2008 | VK | VK_TSR | 0.4440 | 0.1716 | 24.7160 | 0.4583 | 0.2704 | 0.5263 | 0.6429 | 5.5709 | 0.6530 | 7.5510 | 0.5551 | 26.0465 | 1.8146 | 0.9990 | 0.0010 |
| VK_A5_2 | 2008 | VK | VK_TSR | 0.4342 | 0.1680 | 23.2008 | 0.4392 | 0.3071 | 0.6126 | 0.6664 | 5.4894 | 0.6413 | 7.5737 | 0.5574 | 24.5188 | 1.3548 | 0.9991 | 0.0009 |
| VK_A5_3 | 2008 | VK | VK_TSR | 0.3677 | 0.1447 | 23.4233 | 0.4397 | 0.2965 | 0.5877 | 0.6469 | 5.2955 | 0.6136 | 7.8521 | 0.5852 | 22.1333 | 1.9312 | 0.9609 | 0.0391 |
| VK_A5_4 | 2008 | VK | VK_TSR | 0.5320 | 0.1984 | 23.4777 | 0.4440 | 0.2585 | 0.4981 | 0.6048 | 5.5278 | 0.6468 | 7.5185 | 0.5518 | 22.0828 | 1.5855 | 0.9990 | 0.0010 |
| VK_A5_5 | 2008 | VK | VK_TSR | 0.4519 | 0.1736 | 22.9392 | 0.4366 | 0.2972 | 0.5893 | 0.6674 | 5.5102 | 0.6443 | 7.6254 | 0.5625 | 24.0076 | 1.7826 | 1.0000 | 0.0000 |
| VK_A85_1 | 2008 | VK | VK_TSR | 0.5664 | 0.2119 | 17.9548 | 0.3586 | 0.3028 | 0.6026 | 0.6925 | 5.3150 | 0.6164 | 8.5527 | 0.6553 | 28.0422 | 2.8832 | 1.0000 | 0.0000 |
| VK_AM_1 | 2008 | VK | VK_REWET | 0.4416 | 0.1708 | 25.7917 | 0.4665 | 0.2481 | 0.4737 | 0.6733 | 5.2205 | 0.6029 | 7.2377 | 0.5238 | 27.2330 | 1.8968 | 0.9992 | 0.0008 |
| VK_AM_2 | 2008 | VK | VK_REWET | 0.4259 | 0.1644 | 24.7688 | 0.4495 | 0.2454 | 0.4673 | 0.6516 | 5.0688 | 0.5813 | 7.4079 | 0.5408 | 25.4403 | 1.9505 | 0.9547 | 0.0453 |
| VK_AM_3 | 2008 | VK | VK_REWET | 0.4765 | 0.1826 | 22.3324 | 0.4143 | 0.2322 | 0.4364 | 0.7068 | 5.4843 | 0.6406 | 7.2667 | 0.5267 | 28.1535 | 1.8603 | 0.9988 | 0.0012 |
| VK_AM_4 | 2008 | VK | VK_REWET | 0.4479 | 0.1727 | 22.8546 | 0.4319 | 0.2294 | 0.4296 | 0.6724 | 5.2318 | 0.6045 | 7.6630 | 0.5663 | 27.3823 | 2.2657 | 1.0000 | 0.0000 |
| VK_AM_5 | 2008 | VK | VK_REWET | 0.5042 | 0.1915 | 23.3580 | 0.4324 | 0.2425 | 0.4605 | 0.6805 | 5.6705 | 0.6672 | 7.1392 | 0.5139 | 28.6577 | 2.0998 | 1.0000 | 0.0000 |
| VK_BM_1 | 2008 | VK | VK_REF | 0.2863 | 0.1174 | 19.7735 | 0.3956 | 0.3628 | 0.7436 | 0.6442 | 5.4350 | 0.6336 | 8.0704 | 0.6070 | 16.4497 | 1.0524 | 0.8268 | 0.1732 |
| VK_BM_10 | 2008 | VK | VK_REF | 0.5620 | 0.2004 | 15.3383 | 0.3235 | 0.3356 | 0.6797 | 0.8039 | 4.9190 | 0.5599 | 8.6281 | 0.6628 | 15.2308 | 1.1858 | 1.0000 | 0.0000 |
| VK_BM_11 | 2008 | VK | VK_REF | 0.3266 | 0.1329 | 20.8032 | 0.4106 | 0.3695 | 0.7594 | 0.6753 | 5.7731 | 0.6819 | 7.7217 | 0.5722 | 15.4328 | 0.8883 | 0.6272 | 0.3728 |
| VK_BM_12 | 2008 | VK | VK_REF | 0.3020 | 0.1229 | 21.1300 | 0.4144 | 0.3539 | 0.7227 | 0.6340 | 5.5112 | 0.6445 | 8.1261 | 0.6126 | 16.4237 | 1.1163 | 0.8052 | 0.1948 |
| VK_BM_2 | 2008 | VK | VK_REF | 0.3073 | 0.1259 | 19.4415 | 0.3969 | 0.3713 | 0.7636 | 0.6323 | 5.8144 | 0.6878 | 7.7112 | 0.5711 | 14.9704 | 0.9632 | 0.6094 | 0.3906 |
| VK_BM_3 | 2008 | VK | VK_REF | 0.2949 | 0.1203 | 19.4781 | 0.3921 | 0.3483 | 0.7095 | 0.6034 | 5.5223 | 0.6460 | 8.0996 | 0.6100 | 16.8086 | 1.1826 | 0.8093 | 0.1907 |
| VK_BM_4 | 2008 | VK | VK_REF | 0.3294 | 0.1334 | 18.7297 | 0.3749 | 0.3666 | 0.7528 | 0.6738 | 5.6676 | 0.6668 | 7.9085 | 0.5909 | 16.0682 | 0.8991 | 0.6678 | 0.3322 |
| VK_BM_5 | 2008 | VK | VK_REF | 0.2963 | 0.1207 | 17.6256 | 0.3592 | 0.3352 | 0.6788 | 0.5785 | 5.6645 | 0.6664 | 8.1317 | 0.6132 | 16.4552 | 1.0497 | 0.6745 | 0.3255 |
| VK_BM_6 | 2008 | VK | VK_REF | 0.3116 | 0.1274 | 19.3096 | 0.3904 | 0.3562 | 0.7282 | 0.6047 | 5.7907 | 0.6844 | 7.9025 | 0.5903 | 15.6100 | 1.0095 | 0.6515 | 0.3485 |
| VK_BM_7 | 2008 | VK | VK_REF | 0.3037 | 0.1247 | 18.5571 | 0.3755 | 0.3534 | 0.7216 | 0.6180 | 5.4850 | 0.6407 | 8.1205 | 0.6120 | 15.9452 | 0.9446 | 0.7664 | 0.2336 |
| VK_BM_8 | 2008 | VK | VK_REF | 0.3250 | 0.1315 | 19.7998 | 0.3915 | 0.3503 | 0.7144 | 0.6670 | 5.4743 | 0.6392 | 8.0265 | 0.6027 | 16.3251 | 0.9641 | 0.8000 | 0.2000 |
| VK_BM_9 | 2008 | VK | VK_REF | 0.3237 | 0.1297 | 18.4481 | 0.3680 | 0.3386 | 0.6868 | 0.7183 | 5.4374 | 0.6339 | 8.1896 | 0.6190 | 17.0428 | 1.1536 | 0.8761 | 0.1239 |
| VK_E5_1 | 2008 | VK | VK_TSR | 0.3825 | 0.1501 | 22.8958 | 0.4254 | 0.3339 | 0.6756 | 0.5952 | 5.1919 | 0.5988 | 7.4651 | 0.5465 | 20.5364 | 1.3337 | 0.8928 | 0.1072 |
| VK_E5_2 | 2008 | VK | VK_TSR | 0.5103 | 0.1914 | 20.3211 | 0.3925 | 0.2937 | 0.5812 | 0.6667 | 5.5319 | 0.6474 | 7.6343 | 0.5634 | 20.9122 | 1.2987 | 0.9557 | 0.0443 |
| VK_E5_3 | 2008 | VK | VK_TSR | 0.4116 | 0.1576 | 19.0223 | 0.3749 | 0.2940 | 0.5819 | 0.6767 | 5.2711 | 0.6102 | 7.9967 | 0.5997 | 18.1609 | 1.5407 | 0.9398 | 0.0602 |
| VK_E5_4 | 2008 | VK | VK_TSR | 0.2437 | 0.1023 | 21.4765 | 0.4234 | 0.3397 | 0.6892 | 0.6670 | 4.9769 | 0.5681 | 7.9042 | 0.5904 | 17.2920 | 1.6207 | 0.9991 | 0.0009 |
| VK_E5_5 | 2008 | VK | VK_TSR | 0.4591 | 0.1762 | 25.4995 | 0.4589 | 0.2538 | 0.4871 | 0.7397 | 5.6372 | 0.6625 | 7.3525 | 0.5352 | 29.3867 | 2.0511 | 0.9447 | 0.0553 |
| VK_E85_1 | 2008 | VK | VK_TSR | 0.3438 | 0.1354 | 20.2201 | 0.4060 | 0.3402 | 0.6904 | 0.6414 | 5.1534 | 0.5933 | 7.7844 | 0.5784 | 16.7181 | 1.4120 | 1.0000 | 0.0000 |
| VK_EM_1 | 2008 | VK | VK_REWET | 0.5516 | 0.2048 | 23.3999 | 0.4337 | 0.2430 | 0.4618 | 0.6969 | 5.2949 | 0.6136 | 6.7493 | 0.4749 | 30.8782 | 3.1601 | 0.9546 | 0.0454 |
| VK_EM_2 | 2008 | VK | VK_REWET | 0.5778 | 0.2144 | 21.5701 | 0.4055 | 0.2350 | 0.4430 | 0.6670 | 5.5325 | 0.6475 | 6.7406 | 0.4741 | 32.2411 | 2.7224 | 0.9536 | 0.0464 |
| VK_EM_3 | 2008 | VK | VK_REWET | 0.5299 | 0.1995 | 26.4104 | 0.4685 | 0.2190 | 0.4053 | 0.6733 | 5.1807 | 0.5972 | 6.8857 | 0.4886 | 31.9574 | 3.1422 | 0.9201 | 0.0799 |
| VK_EM_4 | 2008 | VK | VK_REWET | 0.4973 | 0.1853 | 22.8503 | 0.4202 | 0.2691 | 0.5232 | 0.7463 | 5.2152 | 0.6022 | 6.8889 | 0.4889 | 28.0436 | 2.5137 | 0.8929 | 0.1071 |
| VK_EM_5 | 2008 | VK | VK_REWET | 0.4811 | 0.1811 | 23.7659 | 0.4169 | 0.2616 | 0.5056 | 0.6742 | 4.9350 | 0.5621 | 7.1210 | 0.5121 | 26.4313 | 2.0269 | 0.8265 | 0.1735 |
| VK_G10_1 | 2008 | VK | VK_TSR | 0.5719 | 0.2114 | 20.3596 | 0.3879 | 0.2647 | 0.5128 | 0.6174 | 5.5713 | 0.6530 | 7.0345 | 0.5035 | 27.1314 | 2.1920 | 0.9363 | 0.0637 |
| VK_G10_2 | 2008 | VK | VK_TSR | 0.3828 | 0.1496 | 21.5162 | 0.4120 | 0.3232 | 0.6505 | 0.5983 | 5.1196 | 0.5885 | 7.4003 | 0.5400 | 19.9248 | 1.3166 | 0.9414 | 0.0586 |
| VK_G10_3 | 2008 | VK | VK_TSR | 0.4744 | 0.1804 | 22.3963 | 0.4117 | 0.2833 | 0.5566 | 0.5888 | 5.3785 | 0.6255 | 7.4808 | 0.5481 | 25.6561 | 1.3500 | 0.8832 | 0.1168 |
| VK_G10_4 | 2008 | VK | VK_TSR | 0.4880 | 0.1842 | 21.3547 | 0.3858 | 0.3017 | 0.5998 | 0.6936 | 5.3201 | 0.6172 | 7.3355 | 0.5336 | 25.7453 | 1.3795 | 0.8415 | 0.1585 |
| VK_G10_5 | 2008 | VK | VK_TSR | 0.5754 | 0.2134 | 18.5429 | 0.3672 | 0.2223 | 0.4131 | 0.6381 | 5.7256 | 0.6751 | 7.1401 | 0.5140 | 25.7275 | 2.0062 | 1.0000 | 0.0000 |
| VK_G5_1 | 2008 | VK | VK_TSR | 0.5202 | 0.1935 | 28.0870 | 0.4991 | 0.2645 | 0.5124 | 0.7091 | 5.1584 | 0.5941 | 6.3376 | 0.4338 | 26.4372 | 2.8654 | 0.9842 | 0.0158 |
| VK_G5_2 | 2008 | VK | VK_TSR | 0.5628 | 0.2077 | 21.8595 | 0.4015 | 0.2737 | 0.5339 | 0.6589 | 5.2189 | 0.6027 | 6.5718 | 0.4572 | 24.7659 | 2.2331 | 0.9027 | 0.0973 |

| | | | | | | | | | | | | | | | | | | |
|---|---|---|---|---|---|---|---|---|---|---|---|---|---|---|---|---|---|---|
| VK_G5_3 | 2008 | VK | VK_TSR | 0.4181 | 0.1605 | 25.2375 | 0.4621 | 0.2570 | 0.4946 | 0.7484 | 4.9558 | 0.5651 | 6.9659 | 0.4966 | 25.5569 | 2.4200 | 1.0000 | 0.0000 |
| VK_G5_4 | 2008 | VK | VK_TSR | 0.4201 | 0.1602 | 26.5522 | 0.4781 | 0.2373 | 0.4483 | 0.7193 | 4.9609 | 0.5658 | 6.7854 | 0.4785 | 25.9725 | 3.2865 | 0.9601 | 0.0399 |
| VK_G5_5 | 2008 | VK | VK_TSR | 0.5797 | 0.2126 | 22.0110 | 0.3873 | 0.2780 | 0.5441 | 0.6913 | 5.0758 | 0.5823 | 6.8290 | 0.4829 | 26.7560 | 2.4431 | 0.8004 | 0.1996 |
| VK_G85_1 | 2008 | VK | VK_TSR | 0.3716 | 0.1457 | 22.9317 | 0.4317 | 0.3148 | 0.6306 | 0.7043 | 5.0668 | 0.5810 | 7.4716 | 0.5472 | 20.4225 | 1.5034 | 0.9548 | 0.0452 |
| VK_GM_1 | 2008 | VK | VK_BEFORE | 0.5301 | 0.1994 | 28.0151 | 0.4988 | 0.2538 | 0.4871 | 0.6765 | 5.2206 | 0.6029 | 6.5333 | 0.4533 | 28.2557 | 2.9213 | 1.0000 | 0.0000 |
| VK_GM_2 | 2008 | VK | VK_BEFORE | 0.5066 | 0.1909 | 27.8461 | 0.5012 | 0.2648 | 0.5131 | 0.6667 | 4.9483 | 0.5640 | 6.4995 | 0.4500 | 27.4130 | 2.7676 | 1.0000 | 0.0000 |
| VK_GM_3 | 2008 | VK | VK_BEFORE | 0.4964 | 0.1876 | 27.6875 | 0.4961 | 0.2494 | 0.4769 | 0.7047 | 5.2291 | 0.6042 | 6.6643 | 0.4664 | 28.1628 | 2.9383 | 1.0000 | 0.0000 |
| VK_GM_4 | 2008 | VK | VK_BEFORE | 0.5672 | 0.2100 | 27.2770 | 0.4946 | 0.2855 | 0.5618 | 0.6746 | 5.0219 | 0.5746 | 6.5261 | 0.4526 | 28.4567 | 3.1900 | 1.0000 | 0.0000 |
| VK_GM_5 | 2008 | VK | VK_BEFORE | 0.5099 | 0.1917 | 27.1660 | 0.4890 | 0.2758 | 0.5390 | 0.6848 | 5.0356 | 0.5765 | 6.5562 | 0.4556 | 26.7079 | 2.6019 | 1.0000 | 0.0000 |
| VP_ACM_99 | 1999 | VPA | VPA_BEFOR | 0.4875 | 0.1882 | 24.8700 | 0.4711 | 0.2324 | 0.4368 | 0.3333 | 7.0000 | 0.8571 | NA | NA | 24.0000 | NA | 0.0000 | 1.0000 |
| VP_AP_02 | 2002 | VPA | VPA_TSR | 0.5010 | 0.1894 | 22.2948 | 0.4051 | 0.2491 | 0.4761 | 0.6180 | 4.8539 | 0.5506 | 8.6340 | 0.6634 | 25.0574 | 1.6587 | 0.7191 | 0.2809 |
| VP_BCM_99 | 1999 | VPB | VPB_BEFOR | 0.5377 | 0.1972 | 22.6136 | 0.4377 | 0.2743 | 0.5354 | 0.6111 | 5.8667 | 0.6952 | 8.4091 | 0.6409 | 21.7180 | 2.4998 | 0.6667 | 0.3333 |
| VP_BP_02 | 2002 | VPB | VPB_TSR | 0.6371 | 0.2335 | 14.1798 | 0.3115 | 0.3007 | 0.5976 | 0.3381 | 5.0016 | 0.5717 | 9.0016 | 0.7002 | 23.1680 | 1.4752 | 0.0111 | 0.9889 |
| VP_CCM_99 | 1999 | VPC | VPC_BEFOR | 0.6500 | 0.2298 | 16.4590 | 0.2769 | 0.3038 | 0.6048 | 0.7619 | 6.1429 | 0.7347 | 8.1429 | 0.6143 | 21.0681 | 1.6424 | 0.5714 | 0.4286 |
| VP_CP_02 | 2002 | VPC | VPC_TSR | 0.4720 | 0.1768 | 19.6439 | 0.3909 | 0.2881 | 0.5680 | 0.5736 | 5.9419 | 0.7060 | 9.2353 | 0.7235 | 23.2541 | 1.9763 | 0.9767 | 0.0233 |
| VP_DCM_99 | 1999 | VPD | VPD_BEFOR | 0.6883 | 0.2440 | 16.9258 | 0.3341 | 0.3202 | 0.6434 | 0.7333 | 5.8000 | 0.6857 | 9.0667 | 0.7067 | 23.8067 | 1.6372 | 0.8667 | 0.1333 |
| VP_DP_02 | 2002 | VPD | VPD_TSR | 0.7982 | 0.2770 | 12.9434 | 0.2729 | 0.3460 | 0.7041 | 0.8998 | 6.6776 | 0.8111 | 9.1421 | 0.7142 | 22.0713 | 1.4415 | 1.0000 | 0.0000 |
| VP_FCM_99 | 1999 | VPF | VPF_BEFORI | 0.4632 | 0.1781 | 23.8657 | 0.4381 | 0.2654 | 0.5144 | 0.6894 | 5.7727 | 0.6818 | 7.9302 | 0.5930 | 26.7411 | 1.6599 | 1.0000 | 0.0000 |
| VP_FP_02 | 2002 | VPF | VPF_TSR | 0.5141 | 0.1914 | 16.0821 | 0.3224 | 0.3056 | 0.6092 | 0.8272 | 5.2804 | 0.6115 | 9.4722 | 0.7472 | 22.2218 | 1.7362 | 0.9907 | 0.0093 |
| VP_GCM_99 | 1999 | VPG | VPG_BEFOR | 0.5260 | 0.1925 | 21.5920 | 0.4118 | 0.3492 | 0.7116 | 0.8333 | 5.5000 | 0.6429 | 10.0000 | 0.8000 | 22.6949 | 1.4609 | 1.0000 | 0.0000 |
| VP_GP_02 | 2002 | VPG | VPG_TSR | 0.3067 | 0.1113 | 32.1573 | 0.5135 | 0.2360 | 0.4452 | 0.7727 | 6.2922 | 0.7560 | 9.3333 | 0.7333 | 16.4408 | 1.6780 | 1.0000 | 0.0000 |
| ZB_B3_1992 | 1992 | ZB | ZB_BEFORE | 0.4728 | 0.1776 | 18.0628 | 0.3703 | 0.2886 | 0.5690 | 0.6794 | 5.2228 | 0.6033 | 8.9667 | 0.6967 | 17.1071 | 1.7985 | 0.9946 | 0.0054 |
| ZB_B3_1998 | 1998 | ZB | ZB_REWET | 0.4787 | 0.1817 | 19.3108 | 0.3892 | 0.2700 | 0.5253 | 0.6640 | 5.3200 | 0.6171 | 9.0488 | 0.7049 | 19.1405 | 1.8746 | 0.9920 | 0.0080 |
| ZB_B4_1992 | 1992 | ZB | ZB_BEFORE | 0.6216 | 0.2264 | 14.1219 | 0.2807 | 0.2523 | 0.4836 | 0.6644 | 5.6200 | 0.6600 | 8.4037 | 0.6404 | 17.9844 | 2.0909 | 0.7333 | 0.2667 |
| ZB_B4_1998 | 1998 | ZB | ZB_REWET | 0.4655 | 0.1781 | 23.7923 | 0.4491 | 0.2404 | 0.4556 | 0.6585 | 5.1301 | 0.5900 | 8.7686 | 0.6769 | 22.9481 | 1.7965 | 0.9919 | 0.0000 |
| ZB_B5_1992 | 1992 | ZB | ZB_BEFORE | 0.5378 | 0.2009 | 17.0174 | 0.3553 | 0.2590 | 0.4993 | 0.6622 | 5.1554 | 0.5936 | 9.4392 | 0.7439 | 18.3346 | 2.2976 | 0.9730 | 0.0270 |
| ZB_B5_1998 | 1998 | ZB | ZB_REWET | 0.2866 | 0.1156 | 19.5801 | 0.3999 | 0.3346 | 0.6773 | 0.6580 | 5.2092 | 0.6013 | 8.2171 | 0.6217 | 16.9884 | 1.5818 | 0.9935 | 0.0065 |
| ZB_B6_1992 | 1992 | ZB | ZB_BEFORE | 0.2999 | 0.1212 | 19.5640 | 0.3981 | 0.3282 | 0.6622 | 0.6730 | 5.2370 | 0.6053 | 8.2079 | 0.6208 | 17.3630 | 1.7832 | 1.0000 | 0.0000 |
| ZB_B6_1998 | 1998 | ZB | ZB_REWET | 0.5404 | 0.2024 | 17.0708 | 0.3561 | 0.2386 | 0.4513 | 0.6761 | 5.3546 | 0.6221 | 9.2105 | 0.7211 | 20.2077 | 2.3028 | 1.0000 | 0.0000 |
| ZB_B7_1992 | 1992 | ZB | ZB_BEFORE | 0.3697 | 0.1463 | 24.1224 | 0.4476 | 0.2813 | 0.5519 | 0.6684 | 5.9737 | 0.7105 | 8.5000 | 0.6500 | 21.9624 | 3.1476 | 0.9211 | 0.0789 |
| ZB_B7_1998 | 1998 | ZB | ZB_REWET | 0.9633 | 0.3061 | 21.2776 | 0.4166 | 0.2688 | 0.5225 | 0.8521 | 5.8767 | 0.6967 | 7.7895 | 0.5789 | 23.9684 | 2.1712 | 0.9660 | 0.0272 |
| ZB_B8_1992 | 1992 | ZB | ZB_BEFORE | 0.3510 | 0.1407 | 23.5115 | 0.4496 | 0.2811 | 0.5515 | 0.6647 | 5.8988 | 0.6998 | 9.0629 | 0.7063 | 20.9578 | 3.4188 | 0.9940 | 0.0060 |
| ZB_B8_1998 | 1998 | ZB | ZB_REWET | 0.5105 | 0.1909 | 17.9448 | 0.3497 | 0.2665 | 0.5169 | 0.6568 | 5.7624 | 0.6803 | 8.9368 | 0.6937 | 20.3080 | 2.8416 | 0.8416 | 0.1584 |
| ZB_C1_1988 | 1988 | ZB | ZB_BEFORE | 0.5742 | 0.2072 | 24.2054 | 0.4438 | 0.2234 | 0.4155 | 0.7095 | 5.6055 | 0.6579 | 7.7944 | 0.5794 | 24.4863 | 1.9475 | 0.9725 | 0.0275 |
| ZB_C1_1999 | 1999 | ZB | ZB_REWET | 0.6266 | 0.2228 | 21.6142 | 0.4191 | 0.2503 | 0.4790 | 0.7863 | 5.2203 | 0.6029 | 8.1491 | 0.6149 | 22.6705 | 1.7542 | 1.0000 | 0.0000 |
| ZB_C2_1988 | 1988 | ZB | ZB_BEFORE | 0.7183 | 0.2521 | 21.0407 | 0.4077 | 0.2226 | 0.4138 | 0.8182 | 5.4041 | 0.6292 | 7.7672 | 0.5767 | 24.6470 | 1.9230 | 0.9795 | 0.0205 |
| ZB_C2_2006 | 2006 | ZB | ZB_REWET | 0.5885 | 0.2143 | 19.6675 | 0.3877 | 0.2353 | 0.4437 | 0.7133 | 5.4330 | 0.6333 | 7.8182 | 0.5818 | 23.9068 | 1.7142 | 1.0000 | 0.0000 |
| ZB_C6_1992 | 1992 | ZB | ZB_BEFORE | 0.6908 | 0.2422 | 21.4106 | 0.4205 | 0.2425 | 0.4606 | 0.8705 | 5.0777 | 0.5825 | 7.7622 | 0.5762 | 19.5785 | 1.7528 | 1.0000 | 0.0000 |
| ZB_C6_1999 | 1999 | ZB | ZB_REWET | 0.3593 | 0.1369 | 19.4971 | 0.3920 | 0.3271 | 0.6597 | 0.7166 | 5.0946 | 0.5849 | 8.2238 | 0.6224 | 16.0845 | 1.4223 | 1.0000 | 0.0000 |
| ZB_C9_1988 | 1988 | ZB | ZB_BEFORE | 0.7369 | 0.2582 | 19.0780 | 0.3912 | 0.2555 | 0.4911 | 0.8660 | 5.0882 | 0.5840 | 8.2424 | 0.6242 | 32.6526 | 2.0472 | 1.0000 | 0.0000 |
| ZB_C9_2006 | 2006 | ZB | ZB_REWET | 0.6062 | 0.2211 | 19.4047 | 0.3849 | 0.2444 | 0.4649 | 0.6820 | 5.4082 | 0.6297 | 8.3974 | 0.6397 | 28.3149 | 1.8423 | 1.0000 | 0.0000 |

| 10 n_0 no N | 10 n_1 N fixi | 11 MStatusC | 12 MStatusN | 13 MFlexi_1 | 14 sm | 14 sm.s | 15 snb | 15 snb.s | 16 seed buoy | 17 autochor_ | 18 bythisoch | 19 chamaech | 20 hemeroch | 21 meteoroc | 22 nautocho | 23 ombrocho | 24 other_1 | 25 zoochor_ |
|---|---|---|---|---|---|---|---|---|---|---|---|---|---|---|---|---|---|---|
| 1.0000 | 0.0000 | 0.0526 | 0.4408 | 0.5066 | 1.3507 | 0.1365 | 1.6186 | 0.3093 | 61.5912 | 0.0263 | 0.0000 | 0.0000 | 0.1447 | 0.1118 | 0.8816 | 0.0000 | 0.0592 | 0.7303 |
| 1.0000 | 0.0000 | 0.0164 | 0.5164 | 0.4344 | 1.4549 | 0.1527 | 1.8922 | 0.4461 | 59.7020 | 0.0820 | 0.0000 | 0.0000 | 0.0902 | 0.0574 | 0.9426 | 0.0000 | 0.0574 | 0.5984 |
| 0.4154 | 0.0000 | 0.0462 | 0.1231 | 0.8000 | 1.1985 | 0.1367 | 2.1364 | 0.5682 | 71.3855 | 0.0462 | 0.0000 | 0.0000 | 0.1538 | 0.1538 | 0.3846 | 0.0000 | 0.0769 | 0.2769 |
| 1.0000 | 0.0000 | 0.0556 | 0.5694 | 0.3333 | 0.9049 | 0.1137 | 1.9444 | 0.4722 | 80.4655 | 0.0694 | 0.0000 | 0.0000 | 0.2778 | 0.2361 | 1.0000 | 0.0278 | 0.2083 | 0.2917 |
| 1.0000 | 0.0000 | 0.0632 | 0.5263 | 0.3895 | 1.1626 | 0.1322 | 1.8387 | 0.4194 | 71.7031 | 0.0526 | 0.0000 | 0.0000 | 0.2000 | 0.1368 | 0.9579 | 0.0211 | 0.1158 | 0.4211 |
| 1.0000 | 0.0000 | 0.0000 | 0.5455 | 0.4364 | 1.6636 | 0.1714 | 1.9205 | 0.4602 | 60.0790 | 0.1727 | 0.0000 | 0.0000 | 0.0909 | 0.0545 | 0.8455 | 0.0000 | 0.0455 | 0.5909 |
| 0.9909 | 0.0000 | 0.0182 | 0.4545 | 0.5273 | 1.5418 | 0.1593 | 1.8846 | 0.4423 | 63.8658 | 0.1000 | 0.0000 | 0.0000 | 0.1273 | 0.1000 | 0.9455 | 0.0273 | 0.0727 | 0.5455 |
| 1.0000 | 0.0000 | 0.0842 | 0.0947 | 0.8000 | 3.6089 | 0.2305 | 1.3721 | 0.1860 | 51.4405 | 0.1368 | 0.0000 | 0.0211 | 0.2000 | 0.1684 | 0.8632 | 0.0211 | 0.0421 | 0.8737 |
| 1.0000 | 0.0000 | 0.0625 | 0.1563 | 0.7188 | 3.6765 | 0.2436 | 1.2051 | 0.1026 | 50.6041 | 0.2292 | 0.0000 | 0.0000 | 0.1563 | 0.0938 | 0.8125 | 0.0000 | 0.0417 | 0.9688 |
| 1.0000 | 0.0000 | 0.0755 | 0.6792 | 0.2453 | 0.9336 | 0.1089 | 1.6970 | 0.3485 | 55.3313 | 0.0283 | 0.0000 | 0.0000 | 0.1321 | 0.1321 | 0.9811 | 0.0000 | 0.0472 | 0.7453 |
| 1.0000 | 0.0000 | 0.0531 | 0.4159 | 0.5310 | 1.5393 | 0.1550 | 1.5851 | 0.2926 | 65.2569 | 0.0354 | 0.0000 | 0.0000 | 0.0973 | 0.1150 | 0.8673 | 0.0177 | 0.0354 | 0.7611 |
| 0.9787 | 0.0000 | 0.0426 | 0.1809 | 0.7447 | 3.6328 | 0.2341 | 1.4023 | 0.2011 | 50.6337 | 0.0638 | 0.0000 | 0.0000 | 0.0745 | 0.0957 | 0.8936 | 0.0213 | 0.0000 | 0.7021 |
| 0.9806 | 0.0000 | 0.1226 | 0.3355 | 0.5290 | 1.2404 | 0.1324 | 1.7227 | 0.3613 | 51.1808 | 0.1484 | 0.0000 | 0.0000 | 0.1871 | 0.1806 | 0.7806 | 0.0129 | 0.0710 | 0.6903 |
| 0.9542 | 0.0000 | 0.1046 | 0.3268 | 0.5490 | 0.6322 | 0.0781 | 1.5069 | 0.2535 | 67.7849 | 0.2484 | 0.0000 | 0.0261 | 0.1438 | 0.1765 | 0.9542 | 0.0261 | 0.0654 | 0.6013 |
| 0.8605 | 0.0000 | 0.1860 | 0.3953 | 0.3488 | 0.6721 | 0.0829 | 1.7576 | 0.3788 | 74.0159 | 0.0698 | 0.0000 | 0.0465 | 0.2093 | 0.3023 | 0.7674 | 0.0698 | 0.0465 | 0.5581 |
| 1.0000 | 0.0000 | 0.0230 | 0.6207 | 0.3218 | 0.9344 | 0.1134 | 1.9211 | 0.4605 | 74.0161 | 0.1034 | 0.0000 | 0.0230 | 0.2644 | 0.1264 | 0.9770 | 0.0230 | 0.1149 | 0.3908 |
| 0.9238 | 0.0000 | 0.1143 | 0.2381 | 0.6000 | 0.6063 | 0.0786 | 1.4388 | 0.2194 | 71.9211 | 0.2571 | 0.0000 | 0.0190 | 0.2762 | 0.2476 | 0.9143 | 0.0571 | 0.1619 | 0.7714 |
| 0.9326 | 0.0000 | 0.0674 | 0.3596 | 0.4944 | 0.8229 | 0.1017 | 1.5897 | 0.2949 | 74.6861 | 0.0899 | 0.0000 | 0.0000 | 0.2135 | 0.2247 | 0.8764 | 0.0225 | 0.2472 | 0.6517 |
| 0.5111 | 0.0000 | 0.0444 | 0.1556 | 0.7556 | 0.9451 | 0.1117 | 2.0455 | 0.5227 | 74.2244 | 0.0222 | 0.0000 | 0.0000 | 0.1556 | 0.2778 | 0.4778 | 0.0222 | 0.1000 | 0.4556 |
| 1.0000 | 0.0000 | 0.0240 | 0.4160 | 0.5600 | 1.7802 | 0.1570 | 1.9180 | 0.4590 | 62.4792 | 0.0560 | 0.0000 | 0.0000 | 0.2080 | 0.1040 | 0.9760 | 0.0000 | 0.1120 | 0.5360 |
| 1.0000 | 0.0000 | 0.0222 | 0.1704 | 0.8074 | 2.1222 | 0.1837 | 1.9466 | 0.4733 | 49.8381 | 0.0296 | 0.0000 | 0.0000 | 0.2519 | 0.0963 | 0.9852 | 0.0000 | 0.0741 | 0.7630 |
| 0.9691 | 0.0000 | 0.1959 | 0.1443 | 0.6598 | 0.8294 | 0.1054 | 1.3846 | 0.1923 | 80.9952 | 0.0825 | 0.0000 | 0.0206 | 0.3299 | 0.2680 | 0.9278 | 0.0206 | 0.4536 | 0.8763 |
| 0.9677 | 0.0000 | 0.0645 | 0.1855 | 0.7339 | 0.8323 | 0.1073 | 1.2328 | 0.1164 | 83.6754 | 0.0565 | 0.0000 | 0.0000 | 0.1452 | 0.1371 | 0.9194 | 0.0000 | 0.5806 | 0.7903 |
| 0.9873 | 0.0000 | 0.0633 | 0.3671 | 0.5696 | 1.2307 | 0.1263 | 1.6618 | 0.3309 | 59.0077 | 0.0759 | 0.0000 | 0.0127 | 0.1709 | 0.1899 | 0.7911 | 0.0253 | 0.0759 | 0.6646 |
| 0.8896 | 0.0000 | 0.0519 | 0.1753 | 0.7597 | 0.8687 | 0.1044 | 1.2385 | 0.1192 | 82.3756 | 0.1039 | 0.0000 | 0.0130 | 0.1688 | 0.1688 | 0.8701 | 0.0519 | 0.5000 | 0.6818 |
| 0.9615 | 0.0000 | 0.0538 | 0.3385 | 0.5538 | 0.3853 | 0.0536 | 1.3125 | 0.1563 | 66.0082 | 0.0846 | 0.0000 | 0.0000 | 0.1462 | 0.1385 | 0.6538 | 0.0154 | 0.1462 | 0.3000 |
| 1.0000 | 0.0000 | 0.0722 | 0.3918 | 0.5361 | 0.8812 | 0.1005 | 1.7949 | 0.3974 | 60.1255 | 0.0928 | 0.0000 | 0.0000 | 0.2680 | 0.2062 | 0.9381 | 0.0000 | 0.1340 | 0.5464 |
| 1.0000 | 0.0000 | 0.0395 | 0.4605 | 0.4803 | 0.9134 | 0.1042 | 1.4656 | 0.2328 | 77.2394 | 0.1382 | 0.0000 | 0.0132 | 0.1842 | 0.1908 | 0.8618 | 0.0132 | 0.0592 | 0.6776 |
| 1.0000 | 0.0000 | 0.0700 | 0.3100 | 0.5900 | 0.7473 | 0.0936 | 1.5778 | 0.2889 | 65.4883 | 0.1000 | 0.0000 | 0.0200 | 0.3800 | 0.3100 | 0.9600 | 0.0200 | 0.1200 | 0.5400 |
| 1.0000 | 0.0000 | 0.0485 | 0.2913 | 0.6311 | 1.0860 | 0.1229 | 1.9462 | 0.4731 | 57.3538 | 0.0777 | 0.0000 | 0.0000 | 0.2136 | 0.1165 | 0.9806 | 0.0291 | 0.2330 | 0.6699 |
| 1.0000 | 0.0000 | 0.0202 | 0.2929 | 0.6869 | 0.7299 | 0.0942 | 2.0112 | 0.5056 | 58.9512 | 0.0707 | 0.0000 | 0.0303 | 0.4949 | 0.0707 | 0.9697 | 0.0303 | 0.1919 | 0.7778 |
| 0.9664 | 0.0168 | 0.1261 | 0.2773 | 0.5966 | 0.7878 | 0.0912 | 1.5481 | 0.2740 | 72.9214 | 0.2017 | 0.0000 | 0.0168 | 0.2185 | 0.2269 | 0.7899 | 0.0504 | 0.1597 | 0.7479 |
| 1.0000 | 0.0000 | 0.0455 | 0.4870 | 0.4675 | 1.3235 | 0.1412 | 1.8881 | 0.4440 | 61.5884 | 0.0390 | 0.0000 | 0.0130 | 0.1364 | 0.1818 | 0.9870 | 0.0325 | 0.0714 | 0.5390 |
| 0.9674 | 0.0326 | 0.0870 | 0.5000 | 0.4130 | 0.8536 | 0.1067 | 1.7528 | 0.3764 | 69.7719 | 0.1196 | 0.0000 | 0.0217 | 0.3696 | 0.2391 | 1.0000 | 0.0000 | 0.0652 | 0.2935 |
| 0.9615 | 0.0000 | 0.0308 | 0.2231 | 0.6846 | 0.8696 | 0.1023 | 1.8495 | 0.4247 | 76.1952 | 0.1462 | 0.0000 | 0.0154 | 0.1615 | 0.1231 | 0.7615 | 0.0462 | 0.0615 | 0.6923 |
| 1.0000 | 0.0000 | 0.0376 | 0.3910 | 0.5489 | 1.1792 | 0.1267 | 1.7917 | 0.3958 | 70.9902 | 0.0301 | 0.0000 | 0.0000 | 0.2782 | 0.1429 | 0.9774 | 0.0150 | 0.0451 | 0.5188 |
| 1.0000 | 0.0000 | 0.0734 | 0.2385 | 0.6881 | 1.2814 | 0.1294 | 2.0426 | 0.5213 | 56.1835 | 0.0367 | 0.0000 | 0.0000 | 0.1927 | 0.2110 | 0.9266 | 0.0367 | 0.0459 | 0.5963 |
| 0.9717 | 0.0000 | 0.1321 | 0.2358 | 0.6321 | 1.3651 | 0.1373 | 1.8315 | 0.4157 | 52.0066 | 0.0566 | 0.0000 | 0.0000 | 0.1981 | 0.2358 | 0.8868 | 0.0377 | 0.0755 | 0.7170 |
| 1.0000 | 0.0000 | 0.0703 | 0.6875 | 0.2422 | 0.9142 | 0.1137 | 1.9455 | 0.4727 | 78.4832 | 0.0625 | 0.0000 | 0.0000 | 0.1406 | 0.1484 | 0.9609 | 0.0156 | 0.0781 | 0.8047 |
| 1.0000 | 0.0000 | 0.4174 | 0.1652 | 0.3913 | 0.9999 | 0.1036 | 1.2936 | 0.1468 | 67.2333 | 0.0696 | 0.0000 | 0.0000 | 0.1739 | 0.2261 | 0.6261 | 0.0174 | 0.0174 | 0.8261 |
| 1.0000 | 0.0000 | 0.1880 | 0.1026 | 0.7094 | 0.1947 | 0.0277 | 1.4286 | 0.2143 | 70.5611 | 0.0427 | 0.0000 | 0.0000 | 0.0684 | 0.0940 | 0.2650 | 0.0256 | 0.0513 | 0.2564 |
| 1.0000 | 0.0000 | 0.0211 | 0.0842 | 0.8632 | 0.1693 | 0.0232 | 1.7826 | 0.3913 | 71.3916 | 0.0211 | 0.0000 | 0.0000 | 0.1684 | 0.0211 | 0.2842 | 0.0211 | 0.0211 | 0.2211 |
| 0.9826 | 0.0000 | 0.0522 | 0.3130 | 0.6348 | 1.0002 | 0.0910 | 1.5634 | 0.2817 | 68.0491 | 0.0522 | 0.0000 | 0.0000 | 0.1739 | 0.1043 | 0.5826 | 0.0174 | 0.0174 | 0.4435 |
| 1.0000 | 0.0000 | 0.1765 | 0.0784 | 0.7157 | 1.8071 | 0.1324 | 1.6818 | 0.3409 | 57.6823 | 0.0784 | 0.0000 | 0.0000 | 0.1275 | 0.3039 | 0.6863 | 0.0196 | 0.0000 | 0.7941 |
| 1.0000 | 0.0000 | 0.1401 | 0.4331 | 0.3949 | 0.9869 | 0.1074 | 1.2483 | 0.1241 | 69.3097 | 0.0573 | 0.0000 | 0.0000 | 0.0828 | 0.2994 | 0.7580 | 0.0191 | 0.1274 | 0.6879 |
| 0.9815 | 0.0000 | 0.0278 | 0.3056 | 0.6667 | 1.5722 | 0.1496 | 2.0787 | 0.5393 | 52.8620 | 0.0648 | 0.0000 | 0.0000 | 0.3333 | 0.1574 | 0.9537 | 0.0000 | 0.0926 | 0.7500 |
| 0.9706 | 0.0000 | 0.0588 | 0.2794 | 0.6618 | 0.4886 | 0.0601 | 1.9792 | 0.4896 | 73.6068 | 0.1471 | 0.0000 | 0.0000 | 0.2647 | 0.2353 | 0.7353 | 0.0441 | 0.0882 | 0.4118 |
| 0.9542 | 0.0261 | 0.0915 | 0.3987 | 0.4967 | 0.5554 | 0.0587 | 1.9365 | 0.4683 | 60.0286 | 0.0654 | 0.0000 | 0.0000 | 0.2092 | 0.1373 | 0.4902 | 0.0261 | 0.0392 | 0.3595 |
| 1.0000 | 0.0000 | 0.1011 | 0.1573 | 0.7191 | 0.6678 | 0.0744 | 1.7049 | 0.3525 | 75.5687 | 0.0449 | 0.0000 | 0.0225 | 0.2135 | 0.1798 | 0.7191 | 0.0674 | 0.0449 | 0.5955 |
| 1.0000 | 0.0000 | 0.0595 | 0.1548 | 0.7500 | 3.6940 | 0.2289 | 1.4000 | 0.2000 | 50.9889 | 0.0714 | 0.0000 | 0.0238 | 0.1905 | 0.0952 | 0.9524 | 0.0238 | 0.0714 | 0.8452 |

| | | | | | | | | | | | | | | | | | | |
|---|---|---|---|---|---|---|---|---|---|---|---|---|---|---|---|---|---|---|
| 0.9655 | 0.0207 | 0.3517 | 0.3310 | 0.3172 | 0.7298 | 0.0895 | 1.4511 | 0.2256 | 67.9623 | 0.0621 | 0.0000 | 0.0276 | 0.2069 | 0.1793 | 0.6759 | 0.0414 | 0.0897 | 0.7586 |
| 0.9810 | 0.0190 | 0.0571 | 0.7619 | 0.1810 | 0.9736 | 0.1195 | 1.9479 | 0.4740 | 79.2876 | 0.0571 | 0.0000 | 0.0000 | 0.2000 | 0.1143 | 0.9524 | 0.0286 | 0.0476 | 0.2286 |
| 0.9669 | 0.0000 | 0.0826 | 0.2810 | 0.6364 | 0.6101 | 0.0689 | 1.4951 | 0.2476 | 58.2061 | 0.2314 | 0.0000 | 0.0000 | 0.2810 | 0.0909 | 0.7851 | 0.0165 | 0.0579 | 0.5289 |
| 0.9726 | 0.0000 | 0.0137 | 0.5274 | 0.4452 | 0.5743 | 0.0751 | 2.0323 | 0.5161 | 51.7665 | 0.0274 | 0.0000 | 0.0000 | 0.1507 | 0.0616 | 0.7123 | 0.0000 | 0.0479 | 0.6438 |
| 0.9626 | 0.0000 | 0.1682 | 0.1495 | 0.6822 | 0.6573 | 0.0833 | 1.4952 | 0.2476 | 65.5982 | 0.1028 | 0.0000 | 0.0000 | 0.4019 | 0.3271 | 0.9720 | 0.0374 | 0.0748 | 0.7850 |
| 0.9866 | 0.0000 | 0.3289 | 0.1342 | 0.5369 | 0.6535 | 0.0802 | 1.4028 | 0.2014 | 64.2580 | 0.1141 | 0.0000 | 0.0000 | 0.3020 | 0.2550 | 0.7315 | 0.0268 | 0.1074 | 0.8389 |
| 0.9722 | 0.0278 | 0.0741 | 0.5556 | 0.3704 | 0.6253 | 0.0810 | 1.7031 | 0.3516 | 51.7000 | 0.0833 | 0.0000 | 0.0185 | 0.3519 | 0.2037 | 0.9722 | 0.0278 | 0.1296 | 0.7870 |
| 0.9826 | 0.0174 | 0.0783 | 0.4957 | 0.4261 | 0.7385 | 0.0933 | 1.7667 | 0.3833 | 61.7595 | 0.0696 | 0.0000 | 0.0000 | 0.4000 | 0.2174 | 0.9652 | 0.0261 | 0.1391 | 0.7043 |
| 0.9669 | 0.0331 | 0.0826 | 0.4298 | 0.4876 | 0.8075 | 0.1009 | 1.7521 | 0.3761 | 68.3894 | 0.0826 | 0.0000 | 0.0000 | 0.3884 | 0.2562 | 0.9835 | 0.0000 | 0.1157 | 0.5702 |
| 1.0000 | 0.0000 | 0.0588 | 0.3176 | 0.6235 | 1.0192 | 0.1110 | 1.8933 | 0.4467 | 59.3381 | 0.0471 | 0.0000 | 0.0000 | 0.3647 | 0.2941 | 0.9412 | 0.0706 | 0.1765 | 0.7176 |
| 1.0000 | 0.0000 | 0.0000 | 0.0056 | 0.9944 | 0.1369 | 0.0237 | 2.0904 | 0.5452 | 79.9360 | 0.0000 | 0.0000 | 0.0000 | 0.0056 | 0.0955 | 1.0000 | 0.0000 | 0.0056 | 1.0000 |
| 1.0000 | 0.0000 | 0.0000 | 0.0640 | 0.9360 | 0.1400 | 0.0241 | 2.0000 | 0.5000 | 79.4650 | 0.0000 | 0.0000 | 0.0000 | 0.0000 | 0.0000 | 1.0000 | 0.0000 | 0.0000 | 1.0000 |
| 1.0000 | 0.0000 | 0.0000 | 0.4467 | 0.5533 | 0.5705 | 0.0748 | 2.0000 | 0.5000 | 75.5767 | 0.0000 | 0.0000 | 0.0000 | 0.0544 | 0.0544 | 1.0000 | 0.0000 | 0.0544 | 1.0000 |
| 1.0000 | 0.0000 | 0.0000 | 0.4393 | 0.5607 | 0.7043 | 0.0988 | 2.0000 | 0.5000 | 75.5106 | 0.0000 | 0.0000 | 0.0000 | 0.5607 | 0.5607 | 1.0000 | 0.0000 | 0.5607 | 1.0000 |
| 1.0000 | 0.0000 | 0.0000 | 0.0116 | 0.9884 | 0.1406 | 0.0241 | 2.0000 | 0.5000 | 80.5287 | 0.0000 | 0.0000 | 0.0000 | 0.0000 | 0.0000 | 1.0000 | 0.0000 | 0.0000 | 1.0000 |
| 1.0000 | 0.0000 | 0.0000 | 0.6795 | 0.3205 | 0.1350 | 0.0235 | 2.0000 | 0.5000 | 61.1382 | 0.0000 | 0.0000 | 0.0000 | 0.0000 | 0.0000 | 1.0000 | 0.0000 | 0.0000 | 1.0000 |
| 1.0000 | 0.0000 | 0.0000 | 0.3394 | 0.6606 | 0.1350 | 0.0235 | 2.0000 | 0.5000 | 71.6212 | 0.0000 | 0.0000 | 0.0000 | 0.0000 | 0.0000 | 1.0000 | 0.0000 | 0.0000 | 1.0000 |
| 0.9949 | 0.0000 | 0.1244 | 0.0000 | 0.8756 | 0.9393 | 0.1102 | 1.9569 | 0.4784 | 82.1495 | 0.0000 | 0.0000 | 0.0000 | 0.9492 | 0.8858 | 0.9949 | 0.0990 | 0.8401 | 0.9949 |
| 0.9954 | 0.0000 | 0.0367 | 0.0046 | 0.9587 | 2.5194 | 0.1590 | 1.8096 | 0.4048 | 77.1266 | 0.0046 | 0.0000 | 0.0000 | 0.9495 | 0.6881 | 0.9954 | 0.0849 | 0.7408 | 0.9954 |
| 1.0000 | 0.0000 | 0.0545 | 0.0000 | 0.9455 | 0.6445 | 0.0889 | 1.9958 | 0.4979 | 82.3928 | 0.0000 | 0.0000 | 0.0000 | 0.9832 | 0.8218 | 1.0000 | 0.1614 | 0.9119 | 1.0000 |
| 0.9916 | 0.0000 | 0.0630 | 0.0210 | 0.9160 | 0.8547 | 0.1071 | 1.9790 | 0.4895 | 64.2879 | 0.0000 | 0.0000 | 0.0042 | 0.9748 | 0.7920 | 0.9916 | 0.1954 | 0.5924 | 0.9958 |
| 0.9927 | 0.0000 | 0.0146 | 0.0146 | 0.9707 | 0.5568 | 0.0729 | 2.0183 | 0.5091 | 81.4061 | 0.0000 | 0.0000 | 0.0037 | 0.7185 | 0.5558 | 0.9963 | 0.1554 | 0.6746 | 1.0000 |
| 1.0000 | 0.0000 | 0.1321 | 0.0052 | 0.8627 | 0.9342 | 0.1137 | 1.8782 | 0.4391 | 85.8886 | 0.0104 | 0.0000 | 0.0000 | 0.9482 | 0.8368 | 0.9948 | 0.0907 | 0.8005 | 0.9948 |
| 0.9963 | 0.0000 | 0.1262 | 0.0000 | 0.8738 | 0.8853 | 0.1018 | 2.0170 | 0.5085 | 73.0182 | 0.0037 | 0.0000 | 0.0000 | 0.9492 | 0.7503 | 0.9963 | 0.2247 | 0.7357 | 0.9963 |
| 1.0000 | 0.0000 | 0.0000 | 0.3750 | 0.3750 | 0.5342 | 0.0681 | 2.4375 | 0.7188 | 83.5141 | 0.2917 | 0.0000 | 0.0000 | 0.2917 | 0.2917 | 0.7083 | 0.0000 | 0.0000 | 0.4167 |
| 1.0000 | 0.0000 | 0.0625 | 0.5625 | 0.3750 | 0.6343 | 0.0816 | 2.0769 | 0.5385 | 77.7353 | 0.0625 | 0.0000 | 0.0000 | 0.1875 | 0.2500 | 0.9375 | 0.0000 | 0.0625 | 0.4375 |
| 1.0000 | 0.0000 | 0.0244 | 0.7317 | 0.2439 | 0.5385 | 0.0755 | 2.4667 | 0.7333 | 50.4340 | 0.0244 | 0.0000 | 0.0244 | 0.6829 | 0.2195 | 0.9756 | 0.0000 | 0.0244 | 0.8780 |
| 1.0000 | 0.0000 | 0.0000 | 0.6000 | 0.4000 | 0.2763 | 0.0410 | 2.6667 | 0.8333 | 62.8975 | 0.0000 | 0.0000 | 0.0000 | 0.4000 | 0.4000 | 0.8000 | 0.0000 | 0.0000 | 0.4000 |
| 0.9756 | 0.0000 | 0.0488 | 0.0732 | 0.8780 | 0.6067 | 0.0821 | 1.4615 | 0.2308 | 54.3587 | 0.0488 | 0.0000 | 0.0244 | 0.2195 | 0.1463 | 0.9512 | 0.0244 | 0.0976 | 0.9024 |
| 0.9767 | 0.0000 | 0.0698 | 0.0930 | 0.8140 | 0.6565 | 0.0882 | 1.3750 | 0.1875 | 56.1288 | 0.0233 | 0.0000 | 0.0000 | 0.2326 | 0.1395 | 0.9767 | 0.0465 | 0.0698 | 0.9070 |
| 0.9825 | 0.0000 | 0.0351 | 0.2632 | 0.6842 | 0.6205 | 0.0834 | 1.4444 | 0.2222 | 52.2387 | 0.0175 | 0.0000 | 0.0000 | 0.2105 | 0.1754 | 0.9825 | 0.0000 | 0.0351 | 0.7018 |
| 0.9583 | 0.0000 | 0.0833 | 0.1250 | 0.7500 | 0.4858 | 0.0606 | 1.7727 | 0.3864 | 57.8761 | 0.0833 | 0.0000 | 0.0417 | 0.5417 | 0.4167 | 0.9583 | 0.0000 | 0.0417 | 0.8750 |
| 0.9000 | 0.0000 | 0.2000 | 0.2000 | 0.6000 | 0.4463 | 0.0529 | 2.4286 | 0.7143 | 71.9350 | 0.1000 | 0.0000 | 0.0000 | 0.5000 | 0.4000 | 0.9000 | 0.0000 | 0.2000 | 0.7000 |
| 0.9000 | 0.0000 | 0.4000 | 0.1000 | 0.5000 | 0.3966 | 0.0524 | 2.0000 | 0.5000 | 68.7623 | 0.0500 | 0.0000 | 0.0000 | 0.2500 | 0.2000 | 0.9500 | 0.0500 | 0.1500 | 0.9000 |
| 0.9737 | 0.0000 | 0.0526 | 0.2895 | 0.6579 | 0.4597 | 0.0609 | 1.7931 | 0.3966 | 63.8214 | 0.1842 | 0.0000 | 0.0263 | 0.5789 | 0.2368 | 0.9737 | 0.0263 | 0.2105 | 0.8947 |
| 0.9750 | 0.0000 | 0.0750 | 0.2750 | 0.5000 | 0.6299 | 0.0807 | 1.6000 | 0.3000 | 76.6156 | 0.0500 | 0.0000 | 0.0250 | 0.3750 | 0.2250 | 0.9750 | 0.0250 | 0.1750 | 0.7750 |
| 0.9375 | 0.0000 | 0.0625 | 0.1875 | 0.6875 | 0.4235 | 0.0513 | 2.0000 | 0.5000 | 56.9913 | 0.0625 | 0.0000 | 0.0000 | 0.7500 | 0.4375 | 0.9375 | 0.0000 | 0.0625 | 0.8125 |
| 0.9737 | 0.0000 | 0.0000 | 0.8684 | 0.1053 | 0.9042 | 0.1112 | 2.0333 | 0.5167 | 85.4168 | 0.0263 | 0.0000 | 0.0000 | 0.0789 | 0.0526 | 0.8158 | 0.0000 | 0.0263 | 0.1316 |
| 1.0000 | 0.0000 | 0.0000 | 0.8750 | 0.1250 | 0.9939 | 0.1225 | 2.0323 | 0.5161 | 85.0716 | 0.0000 | 0.0000 | 0.0313 | 0.0938 | 0.0938 | 0.9688 | 0.0000 | 0.0313 | 0.1250 |
| 0.9750 | 0.0000 | 0.0250 | 0.8500 | 0.1250 | 0.9653 | 0.1192 | 2.0606 | 0.5303 | 76.0538 | 0.0000 | 0.0000 | 0.0250 | 0.2250 | 0.1000 | 0.9750 | 0.0000 | 0.0250 | 0.2750 |
| 0.8750 | 0.0000 | 0.1250 | 0.3750 | 0.5000 | 0.5602 | 0.0591 | 2.1429 | 0.5714 | 85.4129 | 0.1250 | 0.0000 | 0.1250 | 0.3750 | 0.3750 | 0.8750 | 0.0000 | 0.1250 | 0.6250 |
| 0.9655 | 0.0000 | 0.0172 | 0.4828 | 0.5000 | 0.6573 | 0.0826 | 2.4386 | 0.7193 | 79.6719 | 0.0000 | 0.0000 | 0.0172 | 0.0345 | 0.4655 | 0.9828 | 0.0000 | 0.0172 | 0.5172 |
| 0.9778 | 0.0000 | 0.0222 | 0.2444 | 0.7333 | 0.3309 | 0.0414 | 1.9474 | 0.4737 | 65.3070 | 0.5778 | 0.0000 | 0.0222 | 0.8222 | 0.0667 | 0.9778 | 0.0222 | 0.0667 | 0.9111 |
| 1.0000 | 0.0000 | 0.0270 | 0.7838 | 0.1892 | 0.8901 | 0.1105 | 2.0571 | 0.5286 | 82.5067 | 0.0541 | 0.0000 | 0.0270 | 0.1351 | 0.1081 | 0.9730 | 0.0000 | 0.0541 | 0.1892 |
| 0.9762 | 0.0000 | 0.0238 | 0.6429 | 0.3095 | 0.8676 | 0.1064 | 2.1463 | 0.5732 | 81.8237 | 0.0238 | 0.0000 | 0.0238 | 0.0952 | 0.2381 | 0.9762 | 0.0000 | 0.0714 | 0.3095 |
| 0.9500 | 0.0000 | 0.1000 | 0.4000 | 0.4500 | 0.6481 | 0.0780 | 2.3158 | 0.6579 | 83.1447 | 0.0000 | 0.0000 | 0.0500 | 0.2000 | 0.4500 | 0.9500 | 0.0000 | 0.0500 | 0.5500 |
| 0.9630 | 0.0185 | 0.0370 | 0.1296 | 0.8333 | 0.3520 | 0.0406 | 2.0192 | 0.5096 | 48.2896 | 0.1481 | 0.0000 | 0.0185 | 0.8889 | 0.5741 | 0.9815 | 0.0370 | 0.1111 | 0.8889 |
| 0.9600 | 0.0200 | 0.0600 | 0.1200 | 0.8200 | 0.3495 | 0.0372 | 2.0000 | 0.5000 | 63.0488 | 0.5200 | 0.0000 | 0.0200 | 0.8800 | 0.2200 | 0.9800 | 0.0400 | 0.0600 | 0.9000 |
| 0.9535 | 0.0233 | 0.0698 | 0.6744 | 0.2558 | 0.3091 | 0.0385 | 1.9762 | 0.4881 | 76.7188 | 0.0233 | 0.0000 | 0.0233 | 0.3488 | 0.1628 | 0.9767 | 0.0233 | 0.1163 | 0.9070 |
| 0.9737 | 0.0000 | 0.0526 | 0.3684 | 0.5526 | 0.5309 | 0.0594 | 1.9429 | 0.4714 | 62.3503 | 0.1053 | 0.0000 | 0.0263 | 0.5789 | 0.3158 | 0.9737 | 0.0263 | 0.1579 | 0.8421 |
| 0.9444 | 0.0000 | 0.0000 | 0.5000 | 0.4444 | 0.6723 | 0.0869 | 2.0000 | 0.5000 | 76.4365 | 0.1111 | 0.0000 | 0.0000 | 0.2778 | 0.0556 | 0.9444 | 0.0556 | 0.1111 | 0.6111 |

| | | | | | | | | | | | | | | | | | | |
|---|---|---|---|---|---|---|---|---|---|---|---|---|---|---|---|---|---|---|
| 0.9375 | 0.0313 | 0.0625 | 0.5000 | 0.4375 | 0.5324 | 0.0617 | 2.0000 | 0.5000 | 57.6748 | 0.0625 | 0.0000 | 0.0000 | 0.6563 | 0.3125 | 0.9688 | 0.0000 | 0.0938 | 0.8750 |
| 0.9855 | 0.0145 | 0.0435 | 0.0870 | 0.8696 | 0.2044 | 0.0250 | 1.9552 | 0.4776 | 54.1059 | 0.3913 | 0.0000 | 0.0145 | 0.9130 | 0.4203 | 0.9855 | 0.0145 | 0.0580 | 0.9420 |
| 0.9375 | 0.0313 | 0.0938 | 0.4375 | 0.4688 | 0.7079 | 0.0821 | 2.0400 | 0.5200 | 62.9903 | 0.0938 | 0.0000 | 0.0000 | 0.6250 | 0.2188 | 0.9688 | 0.0625 | 0.1563 | 0.9375 |
| 0.9655 | 0.0172 | 0.0345 | 0.6034 | 0.3621 | 0.4418 | 0.0562 | 1.9412 | 0.4706 | 68.4038 | 0.0345 | 0.0000 | 0.0000 | 0.4483 | 0.1034 | 0.9828 | 0.0345 | 0.0690 | 0.9138 |
| 0.9825 | 0.0000 | 0.0175 | 0.2105 | 0.7719 | 0.3225 | 0.0400 | 1.9375 | 0.4688 | 66.0230 | 0.4737 | 0.0000 | 0.0175 | 0.7895 | 0.1754 | 0.9825 | 0.0175 | 0.1579 | 0.9123 |
| 0.9762 | 0.0000 | 0.0476 | 0.2143 | 0.7381 | 0.7778 | 0.1020 | 1.9737 | 0.4868 | 87.7185 | 0.0000 | 0.0000 | 0.0000 | 0.1429 | 0.0714 | 0.9762 | 0.0000 | 0.0000 | 0.7857 |
| 0.9474 | 0.0000 | 0.0000 | 0.6316 | 0.3684 | 0.6746 | 0.0893 | 2.1538 | 0.5769 | 62.5542 | 0.0526 | 0.0000 | 0.0000 | 0.3158 | 0.1579 | 1.0000 | 0.0000 | 0.0526 | 0.6316 |
| 0.9565 | 0.0000 | 0.1739 | 0.1739 | 0.6087 | 0.4596 | 0.0603 | 1.7500 | 0.3750 | 62.1450 | 0.1304 | 0.0000 | 0.0000 | 0.6522 | 0.4783 | 0.9130 | 0.0000 | 0.0870 | 0.6087 |
| 1.0000 | 0.0000 | 0.0313 | 0.4688 | 0.4688 | 0.5760 | 0.0790 | 2.0000 | 0.5000 | 59.2217 | 0.0938 | 0.0000 | 0.0000 | 0.3750 | 0.1250 | 0.9375 | 0.0000 | 0.0313 | 0.6563 |
| 0.9000 | 0.0000 | 0.2000 | 0.3000 | 0.5000 | 0.7118 | 0.0831 | 2.2500 | 0.6250 | 70.0760 | 0.1000 | 0.0000 | 0.0000 | 0.6000 | 0.3000 | 1.0000 | 0.0000 | 0.1000 | 0.7000 |
| 1.0000 | 0.0000 | 0.0000 | 0.8718 | 0.1282 | 0.5643 | 0.0795 | 2.0000 | 0.5000 | 46.5087 | 0.0513 | 0.0000 | 0.0000 | 0.1026 | 0.0256 | 0.9744 | 0.0000 | 0.0256 | 0.7692 |
| 1.0000 | 0.0000 | 0.0385 | 0.2692 | 0.6923 | 0.4040 | 0.0576 | 1.8000 | 0.4000 | 48.8008 | 0.0769 | 0.0000 | 0.0000 | 0.3846 | 0.3077 | 1.0000 | 0.0000 | 0.0385 | 0.9231 |
| 1.0000 | 0.0000 | 0.0345 | 0.5172 | 0.4483 | 0.6680 | 0.0872 | 1.7727 | 0.3864 | 59.4104 | 0.1034 | 0.0000 | 0.0000 | 0.2759 | 0.1379 | 0.9655 | 0.0000 | 0.1034 | 0.7241 |
| 0.9783 | 0.0217 | 0.0435 | 0.0652 | 0.8913 | 0.4578 | 0.0651 | 1.4667 | 0.2333 | 54.0164 | 0.0217 | 0.0000 | 0.0000 | 0.9348 | 0.8043 | 1.0000 | 0.0217 | 0.1087 | 0.3913 |
| 0.5763 | 0.4237 | 0.4576 | 0.1695 | 0.3729 | 0.6108 | 0.0778 | 1.5088 | 0.2544 | 53.8926 | 0.4237 | 0.0000 | 0.0169 | 0.8305 | 0.3220 | 1.0000 | 0.0339 | 0.1695 | 0.9661 |
| 0.5833 | 0.4167 | 0.4500 | 0.0500 | 0.5000 | 0.6547 | 0.0838 | 1.4828 | 0.2414 | 54.1647 | 0.4167 | 0.0000 | 0.0000 | 0.8500 | 0.2667 | 0.9833 | 0.0333 | 0.1167 | 0.9333 |
| 0.9167 | 0.0833 | 0.1111 | 0.2361 | 0.6528 | 0.7103 | 0.0916 | 1.4000 | 0.2000 | 52.0594 | 0.1111 | 0.0000 | 0.0000 | 0.4861 | 0.2083 | 0.9167 | 0.0417 | 0.1111 | 0.8611 |
| 0.9836 | 0.0164 | 0.0328 | 0.4590 | 0.5082 | 0.4306 | 0.0588 | 1.9714 | 0.4857 | 38.6052 | 0.0328 | 0.0000 | 0.0164 | 0.9180 | 0.4754 | 0.9836 | 0.0000 | 0.0328 | 0.9672 |
| 0.9195 | 0.0690 | 0.1609 | 0.0460 | 0.7931 | 0.6008 | 0.0802 | 1.6118 | 0.3059 | 75.7904 | 0.0805 | 0.0000 | 0.0000 | 0.9425 | 0.4368 | 0.9885 | 0.0920 | 0.1839 | 0.9310 |
| 1.0000 | 0.0000 | 0.0455 | 0.0682 | 0.8864 | 0.2750 | 0.0370 | 1.9286 | 0.4643 | 46.7681 | 0.0227 | 0.0000 | 0.0000 | 0.8864 | 0.7500 | 0.9773 | 0.0455 | 0.1136 | 0.9091 |
| 0.9524 | 0.0476 | 0.0476 | 0.2857 | 0.6667 | 0.4338 | 0.0564 | 1.8421 | 0.4211 | 55.4353 | 0.0476 | 0.0000 | 0.0000 | 0.7619 | 0.4762 | 0.9524 | 0.0000 | 0.1429 | 0.8095 |
| 0.9231 | 0.0385 | 0.1154 | 0.3846 | 0.5000 | 0.7058 | 0.0881 | 1.8261 | 0.4130 | 66.6740 | 0.0769 | 0.0000 | 0.0000 | 0.3462 | 0.1538 | 0.9615 | 0.0000 | 0.0385 | 0.6538 |
| 1.0000 | 0.0000 | 0.0345 | 0.1034 | 0.6552 | 0.6228 | 0.0816 | 1.5714 | 0.2857 | 61.1438 | 0.0345 | 0.0000 | 0.0000 | 0.3793 | 0.3448 | 1.0000 | 0.0000 | 0.1034 | 0.8621 |
| 0.9615 | 0.0000 | 0.0769 | 0.3077 | 0.6154 | 0.5776 | 0.0732 | 1.7368 | 0.3684 | 48.7385 | 0.0000 | 0.0000 | 0.0000 | 0.4231 | 0.3077 | 1.0000 | 0.0385 | 0.0385 | 0.9615 |
| 0.9846 | 0.0000 | 0.0154 | 0.1077 | 0.4923 | 0.4956 | 0.0665 | 1.5862 | 0.2931 | 59.0906 | 0.0308 | 0.0000 | 0.0000 | 0.4615 | 0.4154 | 1.0000 | 0.0000 | 0.0154 | 0.9846 |
| 1.0000 | 0.0000 | 0.0000 | 0.0769 | 0.2821 | 0.7565 | 0.1017 | 1.3158 | 0.1579 | 79.6992 | 0.0256 | 0.0000 | 0.0000 | 0.2821 | 0.0513 | 1.0000 | 0.0000 | 0.0256 | 0.9487 |
| 1.0000 | 0.0000 | 0.0317 | 0.1429 | 0.8254 | 0.4040 | 0.0571 | 1.6230 | 0.3115 | 44.1290 | 0.0000 | 0.0000 | 0.0000 | 0.4603 | 0.4286 | 0.9841 | 0.0000 | 0.0000 | 0.9524 |
| 1.0000 | 0.0000 | 0.0145 | 0.0580 | 0.9275 | 0.4046 | 0.0576 | 1.6418 | 0.3209 | 43.4130 | 0.0145 | 0.0000 | 0.0000 | 0.5507 | 0.4203 | 1.0000 | 0.0145 | 0.0435 | 0.9275 |
| 0.9600 | 0.0000 | 0.0400 | 0.4800 | 0.4800 | 0.8018 | 0.0955 | 1.6667 | 0.3333 | 52.3312 | 0.0000 | 0.0000 | 0.0000 | 0.3200 | 0.1200 | 1.0000 | 0.0000 | 0.0400 | 0.8800 |
| 1.0000 | 0.0000 | 0.0172 | 0.1897 | 0.7759 | 0.2503 | 0.0359 | 2.0545 | 0.5273 | 81.9779 | 0.1034 | 0.0000 | 0.0172 | 0.6724 | 0.5517 | 0.9828 | 0.0000 | 0.4310 | 0.7414 |
| 0.9500 | 0.0250 | 0.0750 | 0.1500 | 0.6250 | 0.4128 | 0.0525 | 1.7368 | 0.3684 | 76.9849 | 0.3250 | 0.0000 | 0.0250 | 0.5250 | 0.2250 | 0.9750 | 0.0500 | 0.2250 | 0.8750 |
| 1.0000 | 0.0000 | 0.0370 | 0.3704 | 0.5556 | 0.5336 | 0.0676 | 2.0000 | 0.5000 | 75.1631 | 0.2222 | 0.0000 | 0.0000 | 0.5926 | 0.1852 | 0.9630 | 0.0741 | 0.0741 | 0.6296 |
| 0.9375 | 0.0000 | 0.0313 | 0.2500 | 0.5313 | 0.7325 | 0.0855 | 2.1200 | 0.5600 | 70.5868 | 0.0625 | 0.0000 | 0.0000 | 0.4375 | 0.3750 | 0.9688 | 0.0313 | 0.1250 | 0.9063 |
| 0.9643 | 0.0000 | 0.0000 | 0.3929 | 0.3929 | 0.3369 | 0.0420 | 2.5385 | 0.7692 | 73.2123 | 0.2143 | 0.0000 | 0.0000 | 0.2857 | 0.2857 | 0.5714 | 0.0000 | 0.0714 | 0.4643 |
| 0.9565 | 0.0000 | 0.0870 | 0.4348 | 0.4348 | 0.5135 | 0.0668 | 2.3333 | 0.6667 | 80.8510 | 0.0435 | 0.0000 | 0.0000 | 0.3913 | 0.4348 | 0.9130 | 0.0000 | 0.0435 | 0.3913 |
| 1.0000 | 0.0000 | 0.0476 | 0.4286 | 0.4762 | 0.5052 | 0.0653 | 2.3333 | 0.6667 | 84.0170 | 0.0000 | 0.0000 | 0.0476 | 0.4762 | 0.4762 | 0.9524 | 0.0000 | 0.0952 | 0.3810 |
| 0.9286 | 0.0000 | 0.1429 | 0.1429 | 0.7143 | 0.3691 | 0.0434 | 2.5833 | 0.7917 | 77.9577 | 0.0714 | 0.0000 | 0.0000 | 0.3571 | 0.6429 | 0.9286 | 0.0000 | 0.0714 | 0.7857 |
| 0.9737 | 0.0000 | 0.5000 | 0.0526 | 0.4474 | 0.9139 | 0.1074 | 1.6400 | 0.3200 | 61.2016 | 0.0526 | 0.0000 | 0.0395 | 0.9342 | 0.3947 | 0.9737 | 0.5132 | 0.2632 | 0.9737 |
| 0.9750 | 0.0250 | 0.1000 | 0.0250 | 0.8750 | 0.8552 | 0.1091 | 1.9500 | 0.4750 | 64.5293 | 0.0750 | 0.0000 | 0.0250 | 0.9500 | 0.7750 | 0.9750 | 0.0500 | 0.7750 | 0.9500 |
| 1.0000 | 0.0000 | 0.0164 | 0.0656 | 0.9180 | 1.3701 | 0.1383 | 1.9828 | 0.4914 | 43.7752 | 0.0000 | 0.0000 | 0.0164 | 0.4918 | 0.0820 | 0.9836 | 0.0000 | 0.0328 | 0.9344 |
| 0.9808 | 0.0192 | 0.5962 | 0.1538 | 0.2500 | 0.8208 | 0.0808 | 1.9412 | 0.4706 | 73.6270 | 0.5192 | 0.0000 | 0.4808 | 0.9615 | 0.2500 | 0.5000 | 0.0577 | 0.2308 | 0.9423 |
| 0.9524 | 0.0238 | 0.1905 | 0.0238 | 0.7619 | 0.9388 | 0.1129 | 1.8500 | 0.4250 | 64.1914 | 0.0714 | 0.0000 | 0.0476 | 0.8810 | 0.6905 | 0.9286 | 0.0952 | 0.6905 | 0.9048 |
| 0.9672 | 0.0000 | 0.1967 | 0.1639 | 0.6230 | 0.7352 | 0.0804 | 1.9153 | 0.4576 | 72.8818 | 0.0328 | 0.0000 | 0.0492 | 0.9016 | 0.5902 | 0.9672 | 0.2787 | 0.1639 | 0.9344 |
| 0.9623 | 0.0189 | 0.1321 | 0.0566 | 0.8113 | 0.4151 | 0.0518 | 1.9423 | 0.4712 | 77.8832 | 0.0566 | 0.0000 | 0.0377 | 0.9434 | 0.6226 | 0.9623 | 0.2075 | 0.2830 | 0.9811 |
| 0.9722 | 0.0278 | 0.1250 | 0.0139 | 0.8611 | 1.0960 | 0.1057 | 1.5915 | 0.2958 | 60.3080 | 0.0417 | 0.0000 | 0.0139 | 0.6111 | 0.4167 | 0.9722 | 0.0417 | 0.4167 | 0.9444 |
| 0.9615 | 0.0128 | 0.5000 | 0.0513 | 0.4487 | 0.9802 | 0.1105 | 1.6364 | 0.3182 | 60.8293 | 0.0513 | 0.0000 | 0.0385 | 0.9359 | 0.3846 | 0.9744 | 0.5128 | 0.2692 | 0.9744 |
| 0.9706 | 0.0147 | 0.1176 | 0.1176 | 0.7647 | 0.5519 | 0.0680 | 1.8529 | 0.4265 | 60.8653 | 0.0588 | 0.0000 | 0.0588 | 0.8824 | 0.2206 | 0.9853 | 0.6324 | 0.4706 | 0.9853 |
| 1.0000 | 0.0000 | 0.2340 | 0.0213 | 0.7447 | 0.8903 | 0.1068 | 1.9149 | 0.4574 | 67.5493 | 0.1702 | 0.0000 | 0.1277 | 0.9574 | 0.6809 | 0.8511 | 0.0851 | 0.6170 | 0.9574 |
| 0.9737 | 0.0263 | 0.1579 | 0.0526 | 0.7895 | 0.8613 | 0.1103 | 1.9459 | 0.4730 | 66.7864 | 0.0526 | 0.0000 | 0.0263 | 0.8947 | 0.7895 | 0.9474 | 0.0263 | 0.7632 | 0.9211 |
| 0.9783 | 0.0217 | 0.7609 | 0.0435 | 0.1957 | 0.7434 | 0.0928 | 1.3778 | 0.1889 | 56.8424 | 0.1957 | 0.0000 | 0.1304 | 0.9348 | 0.1304 | 0.8478 | 0.5870 | 0.1304 | 0.9348 |
| 1.0000 | 0.0000 | 0.0392 | 0.5686 | 0.3922 | 0.9713 | 0.1180 | 1.7708 | 0.3854 | 75.1655 | 0.0000 | 0.0000 | 0.0000 | 0.2941 | 0.2549 | 0.9804 | 0.0196 | 0.0588 | 0.3333 |

| | | | | | | | | | | | | | | | | | | |
|---|---|---|---|---|---|---|---|---|---|---|---|---|---|---|---|---|---|---|
| 0.9796 | 0.0204 | 0.1020 | 0.5510 | 0.3265 | 0.9999 | 0.1223 | 1.9574 | 0.4787 | 79.5200 | 0.0204 | 0.0000 | 0.0000 | 0.3469 | 0.3469 | 0.9796 | 0.0000 | 0.1633 | 0.4286 |
| 0.9804 | 0.0196 | 0.0784 | 0.1765 | 0.7451 | 0.8888 | 0.1121 | 1.9200 | 0.4600 | 70.7879 | 0.0196 | 0.0000 | 0.0000 | 0.7647 | 0.6863 | 0.9608 | 0.0196 | 0.5294 | 0.8039 |
| 0.9804 | 0.0196 | 0.0588 | 0.1765 | 0.7647 | 0.6774 | 0.0806 | 1.5714 | 0.2857 | 62.2849 | 0.0196 | 0.0000 | 0.0000 | 0.7647 | 0.7255 | 0.9804 | 0.0000 | 0.1961 | 0.3137 |
| 1.0000 | 0.0000 | 0.0213 | 0.2128 | 0.7660 | 0.8164 | 0.0918 | 1.4872 | 0.2436 | 61.0065 | 0.0000 | 0.0000 | 0.0000 | 0.7872 | 0.7021 | 0.9787 | 0.0000 | 0.1489 | 0.3830 |
| 0.9778 | 0.0000 | 0.0222 | 0.0889 | 0.8889 | 0.6616 | 0.0787 | 1.8409 | 0.4205 | 51.6891 | 0.0000 | 0.0000 | 0.0000 | 0.8222 | 0.2667 | 0.9778 | 0.0000 | 0.1333 | 0.7778 |
| 0.9818 | 0.0182 | 0.0545 | 0.0909 | 0.8545 | 0.7604 | 0.0981 | 1.8519 | 0.4259 | 58.6489 | 0.0182 | 0.0000 | 0.0000 | 0.8909 | 0.5818 | 0.9636 | 0.1273 | 0.5273 | 0.8727 |
| 0.9855 | 0.0145 | 0.0435 | 0.1304 | 0.8261 | 0.8339 | 0.1060 | 1.8235 | 0.4118 | 65.7670 | 0.0290 | 0.0000 | 0.0000 | 0.8551 | 0.6087 | 0.9855 | 0.0290 | 0.4638 | 0.7681 |
| 0.9592 | 0.0408 | 0.0612 | 0.2041 | 0.2245 | 0.9042 | 0.1136 | 1.3830 | 0.1915 | 81.0600 | 0.0408 | 0.0000 | 0.0000 | 0.2449 | 0.1224 | 0.9796 | 0.0000 | 0.0816 | 0.7755 |
| 0.9697 | 0.0303 | 0.0909 | 0.2727 | 0.6364 | 0.8193 | 0.0972 | 1.8667 | 0.4333 | 74.9215 | 0.1212 | 0.0000 | 0.0000 | 0.4848 | 0.3333 | 0.9697 | 0.0000 | 0.2727 | 0.7273 |
| 0.9821 | 0.0179 | 0.0714 | 0.1250 | 0.8036 | 0.8614 | 0.1085 | 1.8545 | 0.4273 | 68.8055 | 0.1250 | 0.0000 | 0.0000 | 0.7321 | 0.5714 | 1.0000 | 0.0000 | 0.5000 | 0.8571 |
| 0.9688 | 0.0000 | 0.0625 | 0.0938 | 0.8438 | 0.5313 | 0.0739 | 2.0000 | 0.5000 | 49.7203 | 0.0000 | 0.0000 | 0.0000 | 0.8438 | 0.0313 | 0.9688 | 0.0000 | 0.0000 | 0.9063 |
| 1.0000 | 0.0000 | 0.0000 | 0.1000 | 0.9000 | 2.3851 | 0.2178 | 2.0357 | 0.5179 | 38.9872 | 0.0000 | 0.0000 | 0.0000 | 0.0667 | 0.0333 | 0.9667 | 0.0000 | 0.0333 | 0.9333 |
| 0.9808 | 0.0192 | 0.0385 | 0.6538 | 0.3077 | 0.4026 | 0.0514 | 1.6923 | 0.3462 | 65.7300 | 0.0385 | 0.0000 | 0.0000 | 0.2115 | 0.1154 | 0.5192 | 0.0192 | 0.0769 | 0.3462 |
| 0.8065 | 0.1935 | 0.2258 | 0.1290 | 0.6452 | 0.7641 | 0.0951 | 1.5333 | 0.2667 | 54.5221 | 0.2258 | 0.0000 | 0.0000 | 0.7097 | 0.2903 | 0.9677 | 0.0323 | 0.2581 | 0.8065 |
| 1.0000 | 0.0000 | 0.1321 | 0.2642 | 0.6038 | 0.9285 | 0.1116 | 1.9750 | 0.4875 | 76.1729 | 0.0000 | 0.0000 | 0.0000 | 0.1132 | 0.0755 | 0.8868 | 0.0000 | 0.0377 | 0.2264 |
| 0.8500 | 0.1500 | 0.1750 | 0.2500 | 0.4250 | 0.8012 | 0.1032 | 1.5897 | 0.2949 | 71.0445 | 0.2000 | 0.0000 | 0.0000 | 0.5500 | 0.3000 | 0.9750 | 0.0250 | 0.2250 | 0.7750 |
| 0.9839 | 0.0161 | 0.0806 | 0.1613 | 0.7581 | 0.7114 | 0.0943 | 1.5085 | 0.2542 | 56.4102 | 0.0323 | 0.0000 | 0.0000 | 0.4032 | 0.2097 | 0.9839 | 0.0968 | 0.2742 | 0.8387 |
| 0.9706 | 0.0294 | 0.0588 | 0.8529 | 0.0882 | 0.1742 | 0.0278 | 1.9375 | 0.4688 | 79.0161 | 0.0588 | 0.0000 | 0.0000 | 0.1176 | 0.0294 | 0.9706 | 0.0000 | 0.0000 | 0.9118 |
| 0.9369 | 0.0631 | 0.0748 | 0.0070 | 0.9159 | 0.5999 | 0.0794 | 1.9155 | 0.4577 | 51.5868 | 0.2967 | 0.0000 | 0.0023 | 0.6869 | 0.3832 | 0.9977 | 0.0070 | 0.3084 | 0.9883 |
| 0.9977 | 0.0023 | 0.0046 | 0.5740 | 0.4214 | 1.3083 | 0.1432 | 1.8692 | 0.4346 | 64.8111 | 0.0023 | 0.0000 | 0.0000 | 0.1868 | 0.0661 | 0.9954 | 0.0569 | 0.1207 | 0.5353 |
| 0.9596 | 0.0389 | 0.3428 | 0.0778 | 0.5778 | 0.9469 | 0.1180 | 1.8060 | 0.4030 | 70.2145 | 0.0763 | 0.0000 | 0.0000 | 0.7275 | 0.7246 | 0.9955 | 0.0015 | 0.0419 | 0.9162 |
| 0.9982 | 0.0018 | 0.0451 | 0.3628 | 0.5921 | 1.1333 | 0.1284 | 1.6823 | 0.3412 | 63.7095 | 0.0036 | 0.0000 | 0.0000 | 0.4134 | 0.4061 | 1.0000 | 0.0018 | 0.0018 | 0.1877 |
| 1.0000 | 0.0000 | 0.0896 | 0.0932 | 0.8172 | 0.6874 | 0.0868 | 1.3656 | 0.1828 | 56.0818 | 0.0036 | 0.0000 | 0.0000 | 0.7276 | 0.8065 | 1.0000 | 0.0000 | 0.0036 | 0.1004 |
| 0.9917 | 0.0041 | 0.2583 | 0.0014 | 0.7390 | 0.7818 | 0.0858 | 1.7950 | 0.3975 | 46.0193 | 0.0041 | 0.0000 | 0.0014 | 0.9558 | 0.2238 | 0.9613 | 0.7334 | 0.2169 | 0.9613 |
| 0.9155 | 0.0052 | 0.1603 | 0.0181 | 0.8190 | 2.1452 | 0.1901 | 1.3653 | 0.1827 | 74.1598 | 0.5647 | 0.0172 | 0.0207 | 0.3276 | 0.8871 | 0.4034 | 0.0578 | 0.0603 | 0.9922 |
| 0.9931 | 0.0023 | 0.0971 | 0.0023 | 0.8994 | 0.3918 | 0.0531 | 1.9737 | 0.4868 | 51.3320 | 0.0034 | 0.0000 | 0.0011 | 0.9371 | 0.0971 | 0.9680 | 0.8629 | 0.4651 | 0.9680 |
| 0.8766 | 0.0029 | 0.0931 | 0.0588 | 0.8469 | 1.0150 | 0.1038 | 1.6204 | 0.3102 | 77.2068 | 0.1211 | 0.0000 | 0.0017 | 0.4144 | 0.4296 | 0.8213 | 0.2014 | 0.0640 | 0.9383 |
| 0.9993 | 0.0000 | 0.1980 | 0.0000 | 0.7308 | 1.1909 | 0.1324 | 1.4708 | 0.2354 | 77.5049 | 0.0860 | 0.0000 | 0.0000 | 0.3700 | 0.2269 | 0.9845 | 0.0155 | 0.0853 | 0.9140 |
| 0.8750 | 0.1238 | 0.2760 | 0.0248 | 0.6980 | 2.5435 | 0.1728 | 1.7449 | 0.3725 | 45.9949 | 0.1238 | 0.0000 | 0.0000 | 0.6980 | 0.2735 | 0.9740 | 0.3725 | 0.1250 | 0.9715 |
| 0.9845 | 0.0000 | 0.1865 | 0.0319 | 0.7817 | 0.7756 | 0.0740 | 2.1709 | 0.5855 | 86.1286 | 0.0163 | 0.0000 | 0.0000 | 0.4522 | 0.3419 | 1.0000 | 0.0785 | 0.2805 | 1.0000 |
| 0.9990 | 0.0000 | 0.1437 | 0.0010 | 0.8542 | 0.6773 | 0.0851 | 1.6779 | 0.3389 | 82.4036 | 0.2446 | 0.0000 | 0.0000 | 0.3109 | 0.2273 | 0.9990 | 0.0204 | 0.1651 | 0.9572 |
| 1.0000 | 0.0000 | 0.0546 | 0.0013 | 0.9442 | 0.6761 | 0.0793 | 1.6818 | 0.3409 | 77.2997 | 0.1777 | 0.0000 | 0.0000 | 0.2094 | 0.1332 | 0.9480 | 0.0025 | 0.2551 | 0.9454 |
| 1.0000 | 0.0000 | 0.0021 | 0.4055 | 0.5924 | 0.7554 | 0.0875 | 2.3931 | 0.6965 | 64.3019 | 0.1242 | 0.0000 | 0.0000 | 0.0842 | 0.4066 | 0.9979 | 0.0010 | 0.0411 | 0.9979 |
| 1.0000 | 0.0000 | 0.0000 | 0.2972 | 0.7028 | 0.4143 | 0.0540 | 2.0796 | 0.5398 | 61.1315 | 0.4213 | 0.0000 | 0.0194 | 0.5898 | 0.1676 | 1.0000 | 0.0009 | 0.0935 | 0.9815 |
| 0.9402 | 0.0000 | 0.0589 | 0.3146 | 0.6265 | 3.7564 | 0.2253 | 2.2622 | 0.6311 | 42.1549 | 0.0193 | 0.0000 | 0.2024 | 0.9420 | 0.2236 | 0.7958 | 0.2971 | 0.2420 | 0.9807 |
| 0.8998 | 0.0006 | 0.1735 | 0.0373 | 0.7892 | 2.3050 | 0.1326 | 2.0000 | 0.5000 | 50.3222 | 0.0006 | 0.0000 | 0.0000 | 0.7092 | 0.1973 | 0.9383 | 0.3048 | 0.4252 | 0.9389 |
| 0.8303 | 0.0882 | 0.4251 | 0.0738 | 0.4273 | 1.6261 | 0.1189 | 1.8018 | 0.4009 | 57.9574 | 0.1694 | 0.0000 | 0.0004 | 0.6025 | 0.3299 | 0.9846 | 0.0738 | 0.1620 | 0.8299 |
| 0.9131 | 0.0370 | 0.5915 | 0.0246 | 0.2606 | 1.1251 | 0.0998 | 1.8541 | 0.4271 | 63.6861 | 0.0499 | 0.0000 | 0.0000 | 0.7400 | 0.4800 | 0.8509 | 0.0006 | 0.1738 | 0.8262 |
| 0.9709 | 0.0253 | 0.0835 | 0.0506 | 0.7139 | 1.4339 | 0.1225 | 1.9480 | 0.4740 | 60.4905 | 0.1785 | 0.0000 | 0.0013 | 0.7684 | 0.1291 | 0.9734 | 0.0278 | 0.1038 | 0.7696 |
| 0.7629 | 0.1184 | 0.2800 | 0.1169 | 0.4846 | 2.5003 | 0.1720 | 1.6818 | 0.3409 | 57.9468 | 0.2368 | 0.0000 | 0.0000 | 0.5625 | 0.1779 | 0.9230 | 0.0710 | 0.2362 | 0.8754 |
| 0.9710 | 0.0290 | 0.2038 | 0.0305 | 0.6642 | 1.7759 | 0.1138 | 2.2362 | 0.6181 | 66.5014 | 0.1305 | 0.0000 | 0.0000 | 0.6686 | 0.6352 | 0.9703 | 0.0007 | 0.2321 | 0.8687 |
| 0.9747 | 0.0012 | 0.0729 | 0.1929 | 0.6974 | 2.0547 | 0.1185 | 1.9747 | 0.4873 | 65.0890 | 0.0615 | 0.0000 | 0.0000 | 0.8065 | 0.2785 | 0.9873 | 0.0000 | 0.1573 | 0.9271 |
| 0.8357 | 0.0379 | 0.2908 | 0.0259 | 0.6075 | 2.0873 | 0.1282 | 2.0000 | 0.5000 | 61.0268 | 0.2023 | 0.0000 | 0.0000 | 0.9362 | 0.3293 | 0.9867 | 0.0253 | 0.1776 | 0.8982 |
| 0.9228 | 0.0511 | 0.4247 | 0.0498 | 0.5255 | 1.1463 | 0.1214 | 1.5147 | 0.2573 | 81.7467 | 0.3001 | 0.0000 | 0.0000 | 0.8244 | 0.3250 | 0.9004 | 0.0249 | 0.0000 | 0.9502 |
| 0.9263 | 0.0000 | 0.4438 | 0.0737 | 0.4088 | 1.7994 | 0.1562 | 1.6685 | 0.3343 | 62.1647 | 0.0755 | 0.0000 | 0.0000 | 0.7035 | 0.3333 | 0.8527 | 0.0387 | 0.1123 | 0.9263 |
| 0.8627 | 0.0392 | 0.6029 | 0.0402 | 0.2196 | 0.8838 | 0.1062 | 2.0211 | 0.5106 | 72.6938 | 0.0990 | 0.0000 | 0.0000 | 0.8029 | 0.6039 | 0.9794 | 0.0000 | 0.1176 | 0.7431 |
| 0.8851 | 0.0298 | 0.7092 | 0.0014 | 0.2043 | 0.7927 | 0.0931 | 2.1190 | 0.5595 | 84.0133 | 0.1433 | 0.0000 | 0.0000 | 0.5149 | 0.3702 | 0.8851 | 0.0000 | 0.0000 | 0.6582 |
| 0.8473 | 0.0000 | 0.0840 | 0.0000 | 0.9160 | 0.9281 | 0.1099 | 2.0045 | 0.5023 | 83.8329 | 0.2290 | 0.0000 | 0.0000 | 0.4618 | 0.0840 | 1.0000 | 0.0000 | 0.0763 | 0.8435 |
| 0.8696 | 0.0000 | 0.0435 | 0.0870 | 0.8696 | 0.4672 | 0.0613 | 2.4500 | 0.7250 | 75.0441 | 0.1304 | 0.0000 | 0.0000 | 0.6522 | 0.4348 | 1.0000 | 0.0000 | 0.5217 | 0.9130 |
| 0.9654 | 0.0000 | 0.3602 | 0.3602 | 0.2442 | 0.8993 | 0.1122 | 1.8377 | 0.4188 | 83.6046 | 0.0874 | 0.0000 | 0.0000 | 0.5541 | 0.3602 | 0.9827 | 0.0173 | 0.0190 | 0.9636 |
| 0.6227 | 0.0000 | 0.0373 | 0.1282 | 0.8345 | 2.0501 | 0.1380 | 2.0814 | 0.5407 | 74.4410 | 0.0545 | 0.0000 | 0.0000 | 0.1318 | 0.2391 | 0.9991 | 0.0182 | 0.1109 | 0.6400 |

| | | | | | | | | | | | | | | | | | | |
|---|---|---|---|---|---|---|---|---|---|---|---|---|---|---|---|---|---|---|
| 1.0000 | 0.0000 | 0.0295 | 0.1352 | 0.8353 | 0.3888 | 0.0488 | 2.0608 | 0.5304 | 68.3230 | 0.4297 | 0.0000 | 0.0268 | 0.3548 | 0.2195 | 0.9987 | 0.0535 | 0.1084 | 0.9679 |
| 1.0000 | 0.0000 | 0.0181 | 0.1989 | 0.7830 | 0.3827 | 0.0499 | 2.6041 | 0.8021 | 69.9238 | 0.1637 | 0.0000 | 0.0000 | 0.1817 | 0.6013 | 0.9819 | 0.0000 | 0.0181 | 0.9810 |
| 0.9174 | 0.0000 | 0.0174 | 0.1488 | 0.8339 | 0.5580 | 0.0681 | 2.6037 | 0.8019 | 72.0589 | 0.0826 | 0.0000 | 0.0000 | 0.1504 | 0.5512 | 0.9669 | 0.0000 | 0.0182 | 0.9000 |
| 0.9297 | 0.0000 | 0.0007 | 0.0562 | 0.9431 | 0.5413 | 0.0684 | 2.4419 | 0.7210 | 79.9336 | 0.1124 | 0.0000 | 0.0000 | 0.0857 | 0.4406 | 0.9993 | 0.0000 | 0.0014 | 0.9290 |
| 0.9838 | 0.0000 | 0.3366 | 0.3220 | 0.2441 | 0.9170 | 0.1164 | 1.9002 | 0.4501 | 79.8926 | 0.0333 | 0.0000 | 0.0000 | 0.5823 | 0.3366 | 0.9676 | 0.0333 | 0.0333 | 0.8865 |
| 0.9563 | 0.0010 | 0.4863 | 0.0417 | 0.3693 | 0.8415 | 0.1041 | 1.7941 | 0.3970 | 72.8683 | 0.0844 | 0.0000 | 0.0000 | 0.7314 | 0.4232 | 0.9573 | 0.0610 | 0.0610 | 0.8759 |
| 0.9324 | 0.0000 | 0.4742 | 0.0141 | 0.5097 | 0.7233 | 0.0913 | 1.7713 | 0.3856 | 75.3616 | 0.2157 | 0.0000 | 0.0000 | 0.6624 | 0.4735 | 0.9712 | 0.0676 | 0.0670 | 0.9839 |
| 0.9621 | 0.0000 | 0.4054 | 0.0000 | 0.5937 | 0.7519 | 0.0901 | 1.8455 | 0.4227 | 78.0529 | 0.1302 | 0.0000 | 0.0000 | 0.5559 | 0.4958 | 0.9612 | 0.0369 | 0.1108 | 0.9234 |
| 0.9812 | 0.0000 | 0.3565 | 0.0206 | 0.6220 | 0.6774 | 0.0833 | 1.9644 | 0.4822 | 78.1800 | 0.1698 | 0.0000 | 0.0000 | 0.5094 | 0.5797 | 0.9981 | 0.0197 | 0.0394 | 0.9944 |
| 0.9342 | 0.0000 | 0.1332 | 0.1332 | 0.7336 | 0.4141 | 0.0543 | 2.2286 | 0.6143 | 69.4050 | 0.0378 | 0.0000 | 0.0000 | 0.2418 | 0.4967 | 1.0000 | 0.0000 | 0.2336 | 0.9309 |
| 0.8947 | 0.0000 | 0.0711 | 0.1939 | 0.7351 | 0.4504 | 0.0578 | 2.2939 | 0.6469 | 60.8347 | 0.1412 | 0.0000 | 0.0000 | 0.2316 | 0.4351 | 1.0000 | 0.0000 | 0.2114 | 0.9825 |
| 0.9949 | 0.0051 | 0.2154 | 0.0051 | 0.7795 | 0.4703 | 0.0669 | 2.5445 | 0.7723 | 67.8424 | 0.0103 | 0.0000 | 0.0000 | 0.9949 | 0.8513 | 0.9949 | 0.1077 | 0.6821 | 0.9949 |
| 0.9951 | 0.0000 | 0.0049 | 0.1415 | 0.8488 | 0.8340 | 0.1075 | 1.9951 | 0.4975 | 67.3649 | 0.0098 | 0.0000 | 0.0000 | 0.3805 | 0.1707 | 0.9951 | 0.0780 | 0.2390 | 0.9707 |
| 0.9763 | 0.0142 | 0.0332 | 0.0190 | 0.9384 | 0.7388 | 0.0957 | 1.5000 | 0.2500 | 69.9604 | 0.0190 | 0.0000 | 0.0000 | 0.6872 | 0.5592 | 0.9953 | 0.0284 | 0.2701 | 0.9905 |
| 0.9400 | 0.0000 | 0.0040 | 0.1200 | 0.8160 | 0.7489 | 0.0976 | 2.1239 | 0.5620 | 66.3175 | 0.0080 | 0.0000 | 0.0000 | 0.7920 | 0.3400 | 0.9360 | 0.0160 | 0.3360 | 0.6840 |
| 0.9950 | 0.0000 | 0.0050 | 0.6368 | 0.3532 | 1.0466 | 0.1298 | 1.9095 | 0.4548 | 47.0326 | 0.0199 | 0.0000 | 0.0000 | 0.2637 | 0.0498 | 0.9900 | 0.0050 | 0.1343 | 0.9652 |
| 0.9961 | 0.0016 | 0.3409 | 0.0784 | 0.5807 | 0.9696 | 0.1065 | 1.9543 | 0.4772 | 66.4629 | 0.0603 | 0.0000 | 0.0000 | 0.9553 | 0.7516 | 0.9984 | 0.0635 | 0.4945 | 0.9397 |
| 0.9991 | 0.0009 | 0.0273 | 0.0660 | 0.9068 | 0.2428 | 0.0340 | 1.9739 | 0.4870 | 88.0592 | 0.0229 | 0.0000 | 0.0000 | 0.9534 | 0.7740 | 0.9991 | 0.0026 | 0.7942 | 0.8452 |
| 0.9992 | 0.0008 | 0.1254 | 0.0024 | 0.8722 | 0.6703 | 0.0830 | 2.0748 | 0.5374 | 74.5377 | 0.0032 | 0.0000 | 0.0000 | 0.9751 | 0.5265 | 0.9992 | 0.0217 | 0.2645 | 0.8778 |
| 0.9899 | 0.0050 | 0.3166 | 0.0101 | 0.6683 | 0.4568 | 0.0675 | 2.4061 | 0.7030 | 62.6449 | 0.0101 | 0.0000 | 0.0000 | 0.9849 | 0.7286 | 0.9899 | 0.2362 | 0.4673 | 0.9849 |
| 0.9885 | 0.0077 | 0.0154 | 0.3769 | 0.6038 | 1.3436 | 0.1346 | 1.9228 | 0.4614 | 59.4062 | 0.0154 | 0.0000 | 0.0000 | 0.6038 | 0.4923 | 0.9962 | 0.0231 | 0.4346 | 0.9615 |
| 0.9193 | 0.0135 | 0.0314 | 0.0314 | 0.8700 | 0.7901 | 0.0974 | 1.7432 | 0.3716 | 76.4728 | 0.0135 | 0.0000 | 0.0000 | 0.8161 | 0.5785 | 0.9955 | 0.0224 | 0.3408 | 0.9507 |
| 0.9953 | 0.0000 | 0.0190 | 0.5213 | 0.4502 | 1.2616 | 0.1437 | 2.0286 | 0.5143 | 55.4610 | 0.0142 | 0.0000 | 0.0000 | 0.4455 | 0.3223 | 0.9953 | 0.0190 | 0.3223 | 0.9668 |
| 0.9955 | 0.0000 | 0.0179 | 0.4395 | 0.5336 | 1.1905 | 0.1370 | 1.9685 | 0.4842 | 58.1947 | 0.0090 | 0.0000 | 0.0000 | 0.5157 | 0.3632 | 0.9955 | 0.0359 | 0.3363 | 0.9507 |
| 0.9830 | 0.0170 | 0.0596 | 0.6043 | 0.3362 | 0.1799 | 0.0253 | 1.9655 | 0.4828 | 74.9036 | 0.2723 | 0.0000 | 0.0000 | 0.3745 | 0.0511 | 1.0000 | 0.0511 | 0.0426 | 0.9957 |
| 0.9689 | 0.0000 | 0.7578 | 0.0373 | 0.2050 | 0.7497 | 0.0990 | 1.8205 | 0.4103 | 83.6351 | 0.0994 | 0.0000 | 0.0000 | 0.8696 | 0.8012 | 0.9689 | 0.0062 | 0.0062 | 0.9317 |
| 0.9765 | 0.0000 | 0.5412 | 0.1529 | 0.3059 | 1.2379 | 0.0967 | 2.0143 | 0.5071 | 70.2381 | 0.0824 | 0.0000 | 0.0118 | 0.6000 | 0.5647 | 0.9882 | 0.0118 | 0.0588 | 0.8824 |
| 0.0825 | 0.0000 | 0.0206 | 0.0000 | 0.8763 | 2.0674 | 0.2032 | 2.3333 | 0.6667 | 91.2264 | 0.0206 | 0.0000 | 0.0000 | 0.0206 | 0.0309 | 0.8969 | 0.0000 | 0.0206 | 0.0722 |
| 0.8814 | 0.0000 | 0.0508 | 0.0678 | 0.8220 | 1.8851 | 0.1527 | 1.3978 | 0.1989 | 66.5421 | 0.0847 | 0.0000 | 0.0000 | 0.1186 | 0.1102 | 0.8644 | 0.0000 | 0.0254 | 0.8559 |
| 0.7355 | 0.2149 | 0.2231 | 0.1240 | 0.6529 | 0.7337 | 0.0929 | 1.5622 | 0.2811 | 57.7481 | 0.0165 | 0.0000 | 0.0041 | 0.6405 | 0.2479 | 0.9628 | 0.4091 | 0.5579 | 0.9628 |
| 0.8049 | 0.1951 | 0.4919 | 0.0000 | 0.5081 | 1.3348 | 0.1484 | 1.6098 | 0.3049 | 48.0918 | 0.0041 | 0.0000 | 0.0000 | 0.8902 | 0.6220 | 0.9837 | 0.5122 | 0.5407 | 0.9837 |
| 0.9573 | 0.0427 | 0.3934 | 0.0000 | 0.6066 | 0.7986 | 0.0957 | 1.7820 | 0.3910 | 71.5776 | 0.0095 | 0.0000 | 0.0000 | 0.9668 | 0.4408 | 0.9953 | 0.2038 | 0.4976 | 0.9953 |
| 0.9542 | 0.0458 | 0.2061 | 0.3053 | 0.4885 | 1.0496 | 0.1202 | 1.3027 | 0.1513 | 54.5701 | 0.0191 | 0.0000 | 0.0038 | 0.4389 | 0.3740 | 1.0000 | 0.2099 | 0.3321 | 0.6908 |
| 1.0000 | 0.0000 | 0.0076 | 0.7652 | 0.2273 | 0.8871 | 0.1126 | 2.1591 | 0.5795 | 88.4714 | 0.0000 | 0.0000 | 0.0000 | 0.0152 | 0.2045 | 1.0000 | 0.0000 | 0.0000 | 0.9924 |
| 1.0000 | 0.0000 | 0.0000 | 0.6457 | 0.3465 | 0.7684 | 0.0984 | 2.3120 | 0.6560 | 85.7844 | 0.0236 | 0.0000 | 0.0000 | 0.0157 | 0.3150 | 0.9921 | 0.0000 | 0.0000 | 0.9764 |
| 0.9838 | 0.0121 | 0.2753 | 0.1012 | 0.6235 | 1.4672 | 0.1568 | 1.6189 | 0.3094 | 52.6679 | 0.0364 | 0.0000 | 0.0000 | 0.5870 | 0.5385 | 0.9919 | 0.2753 | 0.3117 | 0.8907 |
| 0.8113 | 0.1887 | 0.5132 | 0.0038 | 0.4830 | 1.1440 | 0.1289 | 1.5660 | 0.2830 | 48.8511 | 0.0075 | 0.0000 | 0.0000 | 0.5811 | 0.3434 | 0.7736 | 0.3962 | 0.4453 | 0.7698 |
| 0.9838 | 0.0162 | 0.2955 | 0.0000 | 0.7045 | 0.7958 | 0.1009 | 1.9555 | 0.4777 | 75.4912 | 0.0040 | 0.0000 | 0.0000 | 0.8623 | 0.6437 | 0.8988 | 0.0891 | 0.4980 | 0.8988 |
| 0.9969 | 0.0031 | 0.3270 | 0.0252 | 0.6478 | 1.1344 | 0.1258 | 1.7029 | 0.3514 | 58.3374 | 0.0094 | 0.0000 | 0.0000 | 0.6509 | 0.5220 | 0.8742 | 0.2767 | 0.4591 | 0.8459 |
| 0.9964 | 0.0036 | 0.0573 | 0.1577 | 0.7849 | 0.8186 | 0.1016 | 1.8993 | 0.4496 | 72.5286 | 0.0036 | 0.0000 | 0.0000 | 0.7204 | 0.4839 | 0.9964 | 0.2186 | 0.6810 | 0.9785 |
| 1.0000 | 0.0000 | 0.0127 | 0.3567 | 0.6306 | 0.9175 | 0.1153 | 2.0256 | 0.5128 | 84.5342 | 0.0191 | 0.0000 | 0.0000 | 0.0764 | 0.0637 | 1.0000 | 0.0000 | 0.0382 | 0.9745 |
| 0.9958 | 0.0042 | 0.2385 | 0.0042 | 0.7573 | 0.8373 | 0.0924 | 2.0255 | 0.5128 | 70.7551 | 0.0209 | 0.0000 | 0.0084 | 0.9623 | 0.6192 | 0.9833 | 0.3389 | 0.4979 | 0.9833 |
| 0.7818 | 0.2182 | 0.2436 | 0.1164 | 0.6364 | 0.5934 | 0.0796 | 1.7904 | 0.3952 | 62.3257 | 0.2509 | 0.0000 | 0.0000 | 0.5818 | 0.3491 | 0.9927 | 0.0036 | 0.4727 | 0.9709 |
| 0.8394 | 0.1606 | 0.1767 | 0.1124 | 0.7108 | 1.5526 | 0.1137 | 1.5123 | 0.2562 | 44.3631 | 0.1767 | 0.0000 | 0.0000 | 0.4378 | 0.1928 | 0.6546 | 0.0040 | 0.1847 | 0.6426 |
| 1.0000 | 0.0000 | 0.0000 | 0.0000 | 1.0000 | 0.0697 | 0.0125 | 2.0000 | 0.5000 | 99.1640 | 0.0000 | 0.0000 | 0.0000 | 0.0816 | 0.0765 | 1.0000 | 0.0051 | 0.0051 | 1.0000 |
| 1.0000 | 0.0000 | 0.0068 | 0.8514 | 0.1419 | 0.8315 | 0.1039 | 2.0082 | 0.5041 | 89.5593 | 0.0338 | 0.0000 | 0.0000 | 0.0676 | 0.0405 | 0.8243 | 0.0000 | 0.0608 | 0.8108 |
| 0.7837 | 0.2128 | 0.2979 | 0.0035 | 0.6986 | 0.4931 | 0.0670 | 2.1495 | 0.5747 | 57.0330 | 0.0177 | 0.0000 | 0.0000 | 0.9823 | 0.4397 | 0.9823 | 0.4965 | 0.8830 | 0.9823 |
| 0.8703 | 0.1266 | 0.2785 | 0.0032 | 0.7152 | 0.5755 | 0.0739 | 2.2315 | 0.6158 | 60.2484 | 0.0190 | 0.0000 | 0.0032 | 0.9715 | 0.5823 | 0.9684 | 0.3703 | 0.8038 | 0.9715 |
| 0.9695 | 0.0000 | 0.7099 | 0.0000 | 0.2824 | 3.0774 | 0.2189 | 2.0323 | 0.5161 | 82.7828 | 0.0153 | 0.0000 | 0.0076 | 0.3893 | 0.3130 | 0.9389 | 0.0229 | 0.0916 | 0.9466 |
| 0.9596 | 0.0000 | 0.0303 | 0.0101 | 0.9596 | 0.2467 | 0.0317 | 2.5942 | 0.7971 | 65.9342 | 0.0808 | 0.0000 | 0.0000 | 0.2323 | 0.5657 | 0.7374 | 0.0404 | 0.0101 | 0.6566 |
| 0.1220 | 0.0000 | 0.0000 | 0.0390 | 0.9610 | 1.9932 | 0.1964 | 2.5000 | 0.7500 | 93.3419 | 0.0390 | 0.0000 | 0.0000 | 0.0049 | 0.0049 | 0.8878 | 0.0000 | 0.0098 | 0.0488 |

| | | | | | | | | | | | | | | | | | | |
|---|---|---|---|---|---|---|---|---|---|---|---|---|---|---|---|---|---|---|
| 0.9769 | 0.0185 | 0.1759 | 0.0000 | 0.8241 | 0.5533 | 0.0654 | 1.9814 | 0.4907 | 52.6269 | 0.0000 | 0.0000 | 0.0000 | 0.9815 | 0.3194 | 0.9769 | 0.6667 | 0.7685 | 0.9815 |
| 0.9648 | 0.0282 | 0.8521 | 0.0000 | 0.1479 | 1.5469 | 0.1604 | 1.7254 | 0.3627 | 44.2078 | 0.0000 | 0.0000 | 0.0000 | 0.9859 | 0.8099 | 0.9859 | 0.4507 | 0.1549 | 0.9930 |
| 0.6772 | 0.3175 | 0.9048 | 0.0000 | 0.0952 | 1.2459 | 0.1378 | 1.5585 | 0.2793 | 43.7181 | 0.0053 | 0.0000 | 0.0000 | 0.9894 | 0.5926 | 0.9894 | 0.6984 | 0.3915 | 0.9947 |
| 0.9825 | 0.0175 | 0.6681 | 0.0000 | 0.3319 | 1.7310 | 0.1646 | 1.5590 | 0.2795 | 39.9072 | 0.0000 | 0.0000 | 0.0000 | 0.9651 | 0.6594 | 0.9825 | 0.4978 | 0.0524 | 0.9825 |
| 0.9880 | 0.0060 | 0.1018 | 0.6347 | 0.2635 | 0.8045 | 0.1033 | 1.9568 | 0.4784 | 83.7970 | 0.1018 | 0.0000 | 0.0000 | 0.3473 | 0.1078 | 1.0000 | 0.0299 | 0.0299 | 0.9940 |
| 0.9894 | 0.0053 | 0.4840 | 0.0000 | 0.5106 | 0.5501 | 0.0691 | 1.9189 | 0.4595 | 50.5284 | 0.0053 | 0.0000 | 0.0106 | 0.9894 | 0.5851 | 0.9894 | 0.4096 | 0.1862 | 0.9894 |
| 0.9363 | 0.0598 | 0.3785 | 0.0000 | 0.6175 | 0.7323 | 0.0815 | 1.9036 | 0.4518 | 65.3925 | 0.0040 | 0.0000 | 0.0080 | 0.9880 | 0.7012 | 0.9920 | 0.4343 | 0.5020 | 0.9920 |
| 0.8961 | 0.0000 | 0.0649 | 0.0000 | 0.9221 | 2.9476 | 0.1962 | 1.6316 | 0.3158 | 72.1344 | 0.3896 | 0.0000 | 0.0000 | 0.5065 | 0.1558 | 0.9481 | 0.0130 | 0.0130 | 0.9610 |
| 1.0000 | 0.0000 | 0.1157 | 0.0000 | 0.8802 | 0.3770 | 0.0459 | 2.0041 | 0.5021 | 79.7000 | 0.1653 | 0.0000 | 0.0000 | 0.9917 | 0.6777 | 0.9959 | 0.0496 | 0.1281 | 0.9917 |
| 0.9558 | 0.0398 | 0.2478 | 0.2832 | 0.4646 | 0.5878 | 0.0732 | 1.8438 | 0.4219 | 69.8642 | 0.0044 | 0.0000 | 0.0177 | 0.7035 | 0.2035 | 0.9912 | 0.3805 | 0.1903 | 0.9912 |
| 1.0000 | 0.0000 | 0.0148 | 0.2000 | 0.7852 | 0.2702 | 0.0374 | 2.8986 | 0.9493 | 64.9795 | 0.0074 | 0.0000 | 0.0000 | 0.0444 | 0.4741 | 0.7037 | 0.0074 | 0.0296 | 0.6963 |
| 1.0000 | 0.0000 | 0.0105 | 0.2880 | 0.6963 | 0.3916 | 0.0527 | 2.5366 | 0.7683 | 60.0917 | 0.0000 | 0.0000 | 0.0000 | 0.1571 | 0.2670 | 0.6806 | 0.0209 | 0.0471 | 0.6073 |
| 1.0000 | 0.0000 | 0.0000 | 0.8636 | 0.1364 | 0.1836 | 0.0292 | 1.9832 | 0.4916 | 75.7137 | 0.0303 | 0.0000 | 0.0000 | 0.0455 | 0.0455 | 1.0000 | 0.0606 | 0.0076 | 0.9924 |
| 1.0000 | 0.0000 | 0.1087 | 0.0000 | 0.8913 | 0.5946 | 0.0483 | 2.0000 | 0.5000 | 98.9627 | 0.0000 | 0.0000 | 0.0000 | 0.1304 | 0.0145 | 0.8913 | 0.0000 | 0.0000 | 1.0000 |
| 1.0000 | 0.0000 | 0.0275 | 0.9633 | 0.0092 | 1.0622 | 0.1333 | 2.0096 | 0.5048 | 91.6407 | 0.0000 | 0.0000 | 0.0000 | 0.0092 | 0.0275 | 1.0000 | 0.0000 | 0.0000 | 0.9725 |
| 0.6287 | 0.0060 | 0.0359 | 0.0958 | 0.8623 | 0.5200 | 0.0680 | 2.0467 | 0.5233 | 76.8370 | 0.3653 | 0.0000 | 0.0000 | 0.5689 | 0.0898 | 0.9940 | 0.0120 | 0.0719 | 0.9641 |
| 1.0000 | 0.0000 | 0.0000 | 0.9955 | 0.0045 | 1.0815 | 0.1357 | 2.0083 | 0.5041 | 82.9393 | 0.0000 | 0.0000 | 0.0000 | 0.0045 | 0.0045 | 1.0000 | 0.0000 | 0.0000 | 0.9955 |
| 0.9372 | 0.0628 | 0.5523 | 0.0042 | 0.4435 | 1.4428 | 0.1502 | 1.9351 | 0.4675 | 62.2138 | 0.1715 | 0.0000 | 0.0000 | 0.8243 | 0.6862 | 0.9958 | 0.5146 | 0.3515 | 0.9958 |
| 1.0000 | 0.0000 | 0.0067 | 0.4564 | 0.1342 | 0.5059 | 0.0643 | 2.0000 | 0.5000 | 64.1392 | 0.4362 | 0.0000 | 0.0000 | 0.0604 | 0.4295 | 0.9597 | 0.0000 | 0.0134 | 0.9262 |
| 0.9890 | 0.0000 | 0.6851 | 0.0331 | 0.2818 | 0.6945 | 0.0940 | 1.7374 | 0.3687 | 84.3020 | 0.2265 | 0.0000 | 0.0000 | 0.9282 | 0.7403 | 0.9890 | 0.0055 | 0.0276 | 0.9613 |
| 0.5769 | 0.0000 | 0.0000 | 0.6090 | 0.3846 | 2.7000 | 0.2426 | 2.0000 | 0.5000 | 42.0572 | 0.4167 | 0.0000 | 0.0000 | 0.0000 | 0.0000 | 1.0000 | 0.0000 | 0.0000 | 0.8397 |
| 0.3210 | 0.0000 | 0.0000 | 1.0000 | 0.0000 | 2.7448 | 0.2449 | NA | NA | 50.0000 | 0.6790 | 0.0000 | 0.0000 | 0.0000 | 0.0000 | 1.0000 | 0.0000 | 0.0000 | 0.5062 |
| 0.7849 | 0.2151 | 0.3548 | 0.0000 | 0.6452 | 0.8125 | 0.0920 | 1.7661 | 0.3830 | 74.8058 | 0.0108 | 0.0000 | 0.0000 | 0.9785 | 0.5054 | 1.0000 | 0.3118 | 0.6989 | 1.0000 |
| 0.9292 | 0.0708 | 0.1981 | 0.0000 | 0.8019 | 0.6322 | 0.0723 | 1.9746 | 0.4873 | 81.0857 | 0.0236 | 0.0000 | 0.0000 | 0.9811 | 0.5283 | 0.9811 | 0.1745 | 0.6792 | 0.9811 |
| 0.9583 | 0.0000 | 0.2135 | 0.0417 | 0.7448 | 0.8506 | 0.0785 | 1.9704 | 0.4852 | 80.2805 | 0.1667 | 0.0000 | 0.0052 | 0.6875 | 0.5260 | 0.9115 | 0.0260 | 0.3646 | 0.8906 |
| 0.9757 | 0.0000 | 0.0388 | 0.0194 | 0.9417 | 0.2301 | 0.0290 | 2.5630 | 0.7815 | 63.9658 | 0.0825 | 0.0000 | 0.0049 | 0.2136 | 0.3883 | 0.6068 | 0.0243 | 0.0631 | 0.5728 |
| 0.5322 | 0.0000 | 0.0000 | 0.0585 | 0.4737 | 2.1667 | 0.2127 | 3.0000 | 1.0000 | 89.6543 | 0.4737 | 0.0000 | 0.0000 | 0.0058 | 0.0058 | 0.5263 | 0.0000 | 0.0000 | 0.5263 |
| 0.9350 | 0.0610 | 0.0935 | 0.0000 | 0.9065 | 1.1870 | 0.1193 | 1.9370 | 0.4685 | 88.9319 | 0.0041 | 0.0000 | 0.0000 | 0.9919 | 0.8943 | 1.0000 | 0.0650 | 0.9593 | 1.0000 |
| 0.7800 | 0.0000 | 0.5333 | 0.0067 | 0.4600 | 0.4923 | 0.0645 | 2.0725 | 0.5362 | 87.5220 | 0.0267 | 0.0000 | 0.0000 | 0.7200 | 0.2000 | 1.0000 | 0.0000 | 0.1800 | 0.9933 |
| 0.2593 | 0.0000 | 0.0074 | 0.1185 | 0.8741 | 0.9916 | 0.1235 | 2.1203 | 0.5602 | 88.6900 | 0.0000 | 0.0000 | 0.0000 | 0.0222 | 0.1111 | 1.0000 | 0.0000 | 0.0148 | 0.9926 |
| 0.8415 | 0.0000 | 0.7439 | 0.0122 | 0.2439 | 0.9586 | 0.1170 | 1.9420 | 0.4710 | 84.1428 | 0.0305 | 0.0000 | 0.0000 | 0.7805 | 0.7683 | 0.9939 | 0.0061 | 0.0183 | 0.8232 |
| 0.9769 | 0.0046 | 0.1157 | 0.0000 | 0.8843 | 1.1823 | 0.1211 | 2.0833 | 0.5417 | 69.5016 | 0.0000 | 0.0000 | 0.0000 | 0.9722 | 0.8935 | 1.0000 | 0.0926 | 0.5000 | 1.0000 |
| 0.9931 | 0.0000 | 0.0069 | 0.0000 | 0.9931 | 0.7072 | 0.0989 | 2.0139 | 0.5069 | 93.9556 | 0.0000 | 0.0000 | 0.0000 | 0.9861 | 0.9861 | 1.0000 | 0.0000 | 0.9792 | 1.0000 |
| 0.9858 | 0.0047 | 0.2311 | 0.0000 | 0.7689 | 1.0052 | 0.1086 | 1.6923 | 0.3462 | 67.7469 | 0.0236 | 0.0000 | 0.0000 | 0.7736 | 0.4717 | 0.9953 | 0.3585 | 0.4009 | 1.0000 |
| 0.9816 | 0.0000 | 0.0368 | 0.0000 | 0.9571 | 2.0500 | 0.1628 | 1.7222 | 0.3611 | 76.9342 | 0.3497 | 0.0000 | 0.0000 | 0.6994 | 0.0368 | 0.9816 | 0.0245 | 0.0307 | 0.9939 |
| 1.0000 | 0.0000 | 0.8077 | 0.0000 | 0.1923 | 0.7129 | 0.0994 | 2.0000 | 0.5000 | 78.8009 | 0.0096 | 0.0000 | 0.0000 | 0.8173 | 0.9135 | 0.9615 | 0.0000 | 0.0000 | 0.8173 |
| 1.0000 | 0.0000 | 0.6838 | 0.0342 | 0.2821 | 0.6116 | 0.0857 | 2.0000 | 0.5000 | 78.7454 | 0.0000 | 0.0000 | 0.0000 | 0.7265 | 0.7863 | 0.9573 | 0.0000 | 0.0000 | 0.8889 |
| 1.0000 | 0.0000 | 0.8247 | 0.0000 | 0.1753 | 0.7124 | 0.0993 | 2.0000 | 0.5000 | 78.3336 | 0.0000 | 0.0000 | 0.0000 | 0.8351 | 0.9897 | 1.0000 | 0.0000 | 0.0000 | 0.8454 |
| 0.9714 | 0.0000 | 0.4571 | 0.0857 | 0.4571 | 0.7218 | 0.0893 | 2.0294 | 0.5147 | 71.7068 | 0.0286 | 0.0000 | 0.0000 | 0.4857 | 0.8000 | 0.9714 | 0.0000 | 0.0000 | 0.7143 |
| 0.7674 | 0.0000 | 0.0058 | 0.0581 | 0.4651 | 0.1847 | 0.0298 | 2.3361 | 0.6680 | 64.3206 | 0.0058 | 0.0000 | 0.0000 | 0.0233 | 0.4709 | 0.7616 | 0.0000 | 0.0000 | 0.7616 |
| 0.9724 | 0.0000 | 0.0138 | 0.1310 | 0.8276 | 0.2496 | 0.0326 | 2.5915 | 0.7958 | 65.5849 | 0.0276 | 0.0000 | 0.0000 | 0.0828 | 0.2966 | 0.5586 | 0.0000 | 0.0138 | 0.5724 |
| 0.9136 | 0.0432 | 0.0000 | 0.2376 | 0.6760 | 1.2780 | 0.0912 | 1.7754 | 0.3877 | 56.3620 | 0.1890 | 0.0000 | 0.0000 | 0.4276 | 0.1026 | 0.8704 | 0.0000 | 0.1026 | 0.6598 |
| 0.9681 | 0.0287 | 0.0990 | 0.0830 | 0.8148 | 0.7533 | 0.0783 | 2.1538 | 0.5769 | 58.5232 | 0.0383 | 0.0000 | 0.0255 | 0.8404 | 0.6162 | 0.9713 | 0.0511 | 0.5747 | 0.9585 |
| 1.0000 | 0.0000 | 0.0000 | 0.5224 | 0.4776 | 1.7986 | 0.1498 | 1.8245 | 0.4122 | 41.2471 | 0.0122 | 0.0000 | 0.0000 | 0.3265 | 0.0367 | 1.0000 | 0.0000 | 0.0449 | 1.0000 |
| 0.8340 | 0.0469 | 0.1976 | 0.0367 | 0.7388 | 1.1178 | 0.1108 | 1.3099 | 0.1549 | 68.3196 | 0.0733 | 0.0000 | 0.0041 | 0.6417 | 0.4838 | 0.8784 | 0.0682 | 0.1640 | 0.9430 |
| 0.8504 | 0.0792 | 0.3091 | 0.0000 | 0.6422 | 0.8219 | 0.0954 | 1.8468 | 0.4234 | 59.2261 | 0.0497 | 0.0000 | 0.0276 | 0.9257 | 0.5118 | 0.9980 | 0.5290 | 0.4966 | 0.9405 |
| 0.9085 | 0.0296 | 0.2927 | 0.0015 | 0.7005 | 1.3258 | 0.1207 | 1.8935 | 0.4468 | 60.1471 | 0.0704 | 0.0000 | 0.0249 | 0.9660 | 0.5607 | 0.9862 | 0.3159 | 0.4708 | 0.9496 |
| 0.8083 | 0.0659 | 0.1834 | 0.0353 | 0.6860 | 1.0872 | 0.1179 | 1.5002 | 0.2501 | 65.4441 | 0.1199 | 0.0000 | 0.0376 | 0.5602 | 0.2728 | 0.9083 | 0.1458 | 0.2352 | 0.8859 |
| 0.6558 | 0.3270 | 0.4317 | 0.0062 | 0.5610 | 0.9837 | 0.1109 | 1.5223 | 0.2612 | 52.6121 | 0.0283 | 0.0000 | 0.0062 | 0.8553 | 0.2863 | 0.9926 | 0.6706 | 0.6927 | 0.9791 |
| 0.9365 | 0.0027 | 0.3364 | 0.0000 | 0.6636 | 0.7427 | 0.0831 | 1.8855 | 0.4428 | 44.6633 | 0.0594 | 0.0000 | 0.0581 | 0.9824 | 0.3776 | 0.9851 | 0.5798 | 0.3979 | 0.9595 |
| 0.8247 | 0.0834 | 0.4193 | 0.0000 | 0.5761 | 0.9489 | 0.1098 | 1.7259 | 0.3630 | 56.6411 | 0.0232 | 0.0000 | 0.0185 | 0.9537 | 0.5290 | 0.9846 | 0.4672 | 0.4208 | 0.9598 |

| | | | | | | | | | | | | | | | | | | |
|---|---|---|---|---|---|---|---|---|---|---|---|---|---|---|---|---|---|---|
| 0.8958 | 0.0422 | 0.2304 | 0.0000 | 0.7510 | 0.8791 | 0.1023 | 1.7274 | 0.3637 | 63.7346 | 0.0515 | 0.0000 | 0.0031 | 0.8113 | 0.5416 | 0.9935 | 0.2325 | 0.4805 | 0.8661 |
| 0.9524 | 0.0061 | 0.1648 | 0.0061 | 0.8291 | 0.8832 | 0.0983 | 1.8656 | 0.4328 | 65.9427 | 0.0430 | 0.0000 | 0.0323 | 0.9677 | 0.5326 | 0.9908 | 0.3853 | 0.5421 | 0.9800 |
| 0.5778 | 0.0104 | 0.1058 | 0.0000 | 0.8942 | 0.6308 | 0.0714 | 1.5716 | 0.2858 | 59.1212 | 0.3579 | 0.0000 | 0.0519 | 0.9585 | 0.4948 | 0.9668 | 0.0726 | 0.0954 | 0.6421 |
| 0.6596 | 0.0077 | 0.2509 | 0.0039 | 0.7410 | 1.2087 | 0.0759 | 2.0535 | 0.5267 | 49.2005 | 0.1007 | 0.0000 | 0.0862 | 0.9923 | 0.0329 | 0.8194 | 0.5521 | 0.5695 | 0.9357 |
| 0.9154 | 0.0551 | 0.1331 | 0.0019 | 0.8650 | 0.7690 | 0.0935 | 1.4272 | 0.2136 | 62.6476 | 0.0580 | 0.0000 | 0.0000 | 0.5732 | 0.4021 | 0.9677 | 0.1150 | 0.1217 | 0.9696 |
| 0.9148 | 0.0140 | 0.1229 | 0.0009 | 0.8736 | 0.8510 | 0.1020 | 1.9727 | 0.4864 | 75.0997 | 0.0000 | 0.0000 | 0.0000 | 0.8850 | 0.8323 | 0.9649 | 0.0588 | 0.5461 | 0.9210 |
| 0.9597 | 0.0009 | 0.1337 | 0.1757 | 0.5193 | 1.1480 | 0.1147 | 2.0446 | 0.5223 | 75.9768 | 0.1722 | 0.0000 | 0.0000 | 0.7489 | 0.6118 | 0.8029 | 0.1337 | 0.3033 | 0.7601 |
| 0.9694 | 0.0111 | 0.0893 | 0.0196 | 0.8912 | 1.2062 | 0.1201 | 1.3688 | 0.1844 | 74.7715 | 0.2857 | 0.0000 | 0.0026 | 0.5765 | 0.1165 | 0.9566 | 0.0459 | 0.0578 | 0.9617 |
| 0.8743 | 0.0868 | 0.1549 | 0.3487 | 0.4882 | 0.7989 | 0.0824 | 2.0036 | 0.5018 | 65.5070 | 0.1217 | 0.0000 | 0.0000 | 0.5929 | 0.3317 | 0.8865 | 0.1760 | 0.2482 | 0.8735 |
| 0.9706 | 0.0000 | 0.0364 | 0.0000 | 0.9636 | 0.9024 | 0.1054 | 1.3991 | 0.1996 | 89.2449 | 0.0010 | 0.0000 | 0.0000 | 0.9512 | 0.8991 | 0.9718 | 0.0497 | 0.2641 | 0.9811 |
| 0.7235 | 0.0009 | 0.0210 | 0.0044 | 0.7122 | 0.6069 | 0.0837 | 1.5599 | 0.2800 | 74.7002 | 0.0044 | 0.0000 | 0.0000 | 0.6142 | 0.7577 | 0.9956 | 0.1190 | 0.1409 | 0.9300 |
| 0.8894 | 0.0000 | 0.1556 | 0.0027 | 0.7338 | 0.7373 | 0.0974 | 1.9766 | 0.4883 | 78.9532 | 0.0054 | 0.0000 | 0.0000 | 0.7212 | 0.9128 | 1.0000 | 0.0459 | 0.5270 | 0.8444 |
| 1.0000 | 0.0000 | 0.0082 | 0.0082 | 0.9837 | 0.7365 | 0.1000 | 1.6449 | 0.3225 | 54.6431 | 0.0156 | 0.0000 | 0.0000 | 0.4715 | 0.2142 | 1.0000 | 0.1623 | 0.0578 | 0.9918 |
| 0.9886 | 0.0026 | 0.1248 | 0.0176 | 0.8576 | 0.5084 | 0.0695 | 1.9772 | 0.4886 | 68.3774 | 0.1344 | 0.0000 | 0.0000 | 0.8770 | 0.4315 | 1.0000 | 0.3199 | 0.1793 | 0.8770 |
| 0.9946 | 0.0009 | 0.2178 | 0.1440 | 0.6382 | 1.0300 | 0.1109 | 2.0281 | 0.5140 | 69.6628 | 0.0459 | 0.0000 | 0.0000 | 0.8533 | 0.7435 | 0.8524 | 0.0630 | 0.3519 | 0.8569 |
| 0.6653 | 0.0000 | 0.1323 | 0.0688 | 0.7989 | 0.3375 | 0.0423 | 1.9297 | 0.4649 | 74.2150 | 0.4643 | 0.0000 | 0.0000 | 0.3347 | 0.6627 | 0.9974 | 0.2646 | 0.0000 | 0.9312 |
| 0.8288 | 0.0000 | 0.5151 | 0.0370 | 0.4479 | 0.6220 | 0.0867 | 1.6589 | 0.3295 | 75.7797 | 0.1740 | 0.0000 | 0.0000 | 0.6849 | 0.6521 | 1.0000 | 0.0000 | 0.0000 | 1.0000 |
| 0.7942 | 0.0000 | 0.0012 | 0.0907 | 0.8791 | 1.1753 | 0.1185 | 1.4704 | 0.2352 | 67.9211 | 0.2047 | 0.0000 | 0.0000 | 0.1814 | 0.3244 | 0.9419 | 0.1163 | 0.0023 | 0.8826 |
| 0.8899 | 0.0000 | 0.4405 | 0.0022 | 0.5573 | 0.6576 | 0.0912 | 1.5595 | 0.2797 | 68.3035 | 0.1145 | 0.0000 | 0.0000 | 0.5529 | 0.5529 | 1.0000 | 0.0000 | 0.0000 | 1.0000 |
| 0.5834 | 0.0013 | 0.0013 | 0.3860 | 0.6127 | 0.5545 | 0.0616 | 2.0394 | 0.5197 | 70.5927 | 0.1631 | 0.0000 | 0.0000 | 0.1338 | 0.3834 | 0.7439 | 0.0318 | 0.0013 | 0.7108 |
| 0.5771 | 0.0000 | 0.3778 | 0.3308 | 0.2914 | 0.5790 | 0.0794 | 1.7061 | 0.3531 | 85.6889 | 0.2368 | 0.0000 | 0.0000 | 0.6165 | 0.5733 | 0.9530 | 0.0000 | 0.0019 | 0.9041 |
| 0.9971 | 0.0015 | 0.0745 | 0.0409 | 0.8847 | 1.4871 | 0.1343 | 1.3500 | 0.1750 | 61.6005 | 0.0759 | 0.0000 | 0.0000 | 0.4102 | 0.2219 | 0.9635 | 0.0000 | 0.0015 | 0.9606 |
| 0.6431 | 0.0000 | 0.1802 | 0.0989 | 0.7208 | 0.3092 | 0.0443 | 1.7054 | 0.3527 | 82.1615 | 0.3640 | 0.0000 | 0.0000 | 0.8057 | 0.8021 | 0.9117 | 0.0000 | 0.0035 | 0.9046 |
| 0.9608 | 0.0000 | 0.0058 | 0.0407 | 0.9535 | 0.8570 | 0.1053 | 1.3575 | 0.1787 | 65.3435 | 0.1105 | 0.0000 | 0.0363 | 0.6265 | 0.3706 | 0.9622 | 0.0392 | 0.1497 | 0.9593 |
| 0.9935 | 0.0013 | 0.0078 | 0.0699 | 0.9224 | 1.1376 | 0.1274 | 1.3721 | 0.1860 | 59.8500 | 0.0039 | 0.0000 | 0.0323 | 0.4709 | 0.3687 | 0.9340 | 0.0660 | 0.1345 | 0.8991 |
| 0.9955 | 0.0000 | 0.0605 | 0.0582 | 0.8802 | 1.1153 | 0.1270 | 1.4510 | 0.2255 | 58.9764 | 0.0034 | 0.0000 | 0.0280 | 0.4860 | 0.3483 | 0.9698 | 0.1131 | 0.1713 | 0.9127 |
| 0.9739 | 0.0000 | 0.0502 | 0.1952 | 0.7546 | 1.1778 | 0.1288 | 1.3089 | 0.1545 | 65.1107 | 0.0271 | 0.0000 | 0.0966 | 0.5372 | 0.2966 | 0.9758 | 0.1449 | 0.0734 | 0.9024 |
| 0.9729 | 0.0000 | 0.2102 | 0.1301 | 0.6597 | 1.1203 | 0.1271 | 1.3878 | 0.1939 | 64.9420 | 0.0271 | 0.0000 | 0.0010 | 0.3996 | 0.2966 | 0.9740 | 0.0541 | 0.0552 | 0.9199 |
| 0.9725 | 0.0000 | 0.2209 | 0.1110 | 0.6681 | 1.1090 | 0.1270 | 1.3774 | 0.1887 | 66.3699 | 0.0286 | 0.0000 | 0.0000 | 0.3934 | 0.3637 | 0.9725 | 0.0560 | 0.0857 | 0.9176 |
| 0.5675 | 0.0144 | 0.0304 | 0.0879 | 0.5221 | 0.9457 | 0.0991 | 1.9568 | 0.4784 | 79.2714 | 0.1447 | 0.0000 | 0.0000 | 0.5101 | 0.2763 | 0.6404 | 0.0735 | 0.3205 | 0.6381 |
| 0.9075 | 0.0297 | 0.0616 | 0.0616 | 0.8756 | 1.2327 | 0.1278 | 1.8846 | 0.4423 | 75.6419 | 0.1495 | 0.0000 | 0.0011 | 0.9053 | 0.6096 | 0.9977 | 0.2089 | 0.6107 | 0.9954 |
| 0.8039 | 0.0324 | 0.1010 | 0.1955 | 0.7036 | 0.7897 | 0.0837 | 2.1526 | 0.5763 | 78.0872 | 0.3411 | 0.0000 | 0.0000 | 0.6395 | 0.4110 | 0.9994 | 0.1644 | 0.4718 | 0.9981 |
| 0.5666 | 0.0046 | 0.0521 | 0.1194 | 0.8285 | 0.6248 | 0.0769 | 1.9584 | 0.4792 | 76.5711 | 0.2741 | 0.0000 | 0.0000 | 0.6064 | 0.2067 | 0.9969 | 0.2389 | 0.2083 | 0.9908 |
| 0.7695 | 0.0015 | 0.0075 | 0.2260 | 0.7290 | 0.1942 | 0.0256 | 2.1139 | 0.5569 | 85.9466 | 0.7126 | 0.0000 | 0.0000 | 0.0569 | 0.0105 | 0.9985 | 0.0075 | 0.0075 | 0.9955 |
| 0.9572 | 0.0000 | 0.0016 | 0.1601 | 0.8384 | 0.4207 | 0.0597 | 2.0430 | 0.5215 | 74.5382 | 0.1189 | 0.0000 | 0.0000 | 0.1252 | 0.0824 | 1.0000 | 0.0808 | 0.0792 | 0.9984 |
| 0.6038 | 0.2275 | 0.2305 | 0.2450 | 0.5245 | 0.7220 | 0.0786 | 1.8771 | 0.4386 | 75.3491 | 0.3648 | 0.0000 | 0.0000 | 0.6624 | 0.2807 | 0.9988 | 0.2607 | 0.5191 | 0.9819 |
| 0.9321 | 0.0159 | 0.0814 | 0.2632 | 0.6542 | 1.1971 | 0.1264 | 2.0449 | 0.5224 | 67.0420 | 0.0789 | 0.0000 | 0.0006 | 0.9002 | 0.4657 | 0.9982 | 0.4027 | 0.5245 | 0.9963 |
| 0.7413 | 0.0033 | 0.0879 | 0.1327 | 0.6949 | 0.5580 | 0.0558 | 1.7874 | 0.3937 | 79.0156 | 0.3781 | 0.0000 | 0.0000 | 0.1111 | 0.0547 | 0.9138 | 0.0066 | 0.0149 | 0.8209 |
| 0.9272 | 0.0453 | 0.0080 | 0.0905 | 0.9015 | 1.3602 | 0.1463 | 1.7430 | 0.3715 | 76.4211 | 0.0701 | 0.0000 | 0.0000 | 0.9042 | 0.7622 | 0.9547 | 0.0728 | 0.4499 | 0.9308 |
| 0.9577 | 0.0000 | 0.0031 | 0.0423 | 0.9545 | 0.8570 | 0.1009 | 1.3777 | 0.1889 | 75.6913 | 0.2759 | 0.0000 | 0.0000 | 0.7962 | 0.6708 | 1.0000 | 0.0047 | 0.0862 | 0.9969 |
| 0.9935 | 0.0011 | 0.0108 | 0.4071 | 0.5821 | 0.6464 | 0.0363 | 2.4854 | 0.7427 | 78.5659 | 0.6771 | 0.0000 | 0.0000 | 0.3186 | 0.2300 | 0.9978 | 0.0594 | 0.1706 | 0.9957 |
| 0.9805 | 0.0014 | 0.0208 | 0.4343 | 0.5441 | 1.0472 | 0.1046 | 2.5007 | 0.7503 | 88.5194 | 0.4364 | 0.0000 | 0.0000 | 0.5636 | 0.5413 | 0.9986 | 0.0368 | 0.5240 | 0.9979 |
| 1.0000 | 0.0000 | 0.0207 | 0.4984 | 0.4809 | 0.7662 | 0.0778 | 2.4745 | 0.7373 | 86.4116 | 0.5788 | 0.0000 | 0.0000 | 0.5000 | 0.3790 | 1.0000 | 0.0406 | 0.3591 | 1.0000 |
| 0.8059 | 0.0019 | 0.2869 | 0.0966 | 0.6147 | 0.5982 | 0.0641 | 2.0284 | 0.5142 | 83.8187 | 0.1653 | 0.0000 | 0.0009 | 0.8533 | 0.4048 | 0.9053 | 0.0548 | 0.4689 | 0.9034 |
| 0.6930 | 0.1010 | 0.1580 | 0.2010 | 0.6410 | 0.2185 | 0.0278 | 1.9462 | 0.4731 | 78.6601 | 0.3050 | 0.0000 | 0.0000 | 0.7940 | 0.2840 | 0.9980 | 0.1550 | 0.4320 | 0.9970 |
| 0.7907 | 0.1036 | 0.5233 | 0.1047 | 0.3710 | 0.6853 | 0.0789 | 1.9182 | 0.4591 | 78.9970 | 0.0808 | 0.0000 | 0.0010 | 0.9699 | 0.2394 | 0.9969 | 0.1876 | 0.4187 | 0.9969 |
| 0.7959 | 0.0013 | 0.0439 | 0.1667 | 0.7881 | 0.1971 | 0.0218 | 2.1354 | 0.5677 | 83.7108 | 0.6473 | 0.0000 | 0.0000 | 0.5672 | 0.2080 | 0.9987 | 0.0401 | 0.1059 | 0.8669 |
| 0.6952 | 0.0334 | 0.0441 | 0.2714 | 0.6845 | 0.1743 | 0.0226 | 1.8340 | 0.4170 | 75.8355 | 0.5067 | 0.0000 | 0.0000 | 0.3543 | 0.0160 | 0.9987 | 0.2045 | 0.0775 | 0.9960 |
| 0.6104 | 0.0000 | 0.0251 | 0.3645 | 0.6085 | 0.0622 | 0.0089 | 2.0029 | 0.5015 | 88.7549 | 0.7473 | 0.0000 | 0.0000 | 0.4156 | 0.1986 | 0.9971 | 0.0251 | 0.1948 | 0.9961 |
| 0.8236 | 0.0009 | 0.0087 | 0.3510 | 0.6403 | 0.0622 | 0.0083 | 2.1969 | 0.5984 | 90.1524 | 0.6099 | 0.0000 | 0.0009 | 0.4257 | 0.3553 | 0.9974 | 0.0052 | 0.3310 | 0.9939 |
| 0.8984 | 0.0016 | 0.0023 | 0.1186 | 0.8016 | 1.0064 | 0.1084 | 1.5770 | 0.2885 | 69.1181 | 0.0992 | 0.0000 | 0.0000 | 0.3364 | 0.2543 | 0.9225 | 0.0008 | 0.0628 | 0.8829 |

| | | | | | | | | | | | | | | | | | | |
|---|---|---|---|---|---|---|---|---|---|---|---|---|---|---|---|---|---|---|
| 0.7321 | 0.0008 | 0.1537 | 0.0594 | 0.7108 | 0.8747 | 0.0923 | 1.8481 | 0.4241 | 74.1107 | 0.0791 | 0.0000 | 0.0000 | 0.1035 | 0.2131 | 0.9239 | 0.0000 | 0.1164 | 0.9231 |
| 0.3488 | 0.0000 | 0.1133 | 0.0566 | 0.3488 | 0.2610 | 0.0345 | 2.4190 | 0.7095 | 80.2916 | 0.0872 | 0.0000 | 0.0000 | 0.0340 | 0.2288 | 0.5187 | 0.0000 | 0.0045 | 0.5187 |
| 0.2965 | 0.0000 | 0.0969 | 0.1957 | 0.2946 | 0.1834 | 0.0240 | 2.2950 | 0.6475 | 86.5626 | 0.2432 | 0.0000 | 0.0000 | 0.0271 | 0.1948 | 0.5872 | 0.0000 | 0.0029 | 0.5872 |
| 0.9284 | 0.0236 | 0.0961 | 0.1178 | 0.7861 | 0.4004 | 0.0500 | 2.5156 | 0.7578 | 71.7089 | 0.1659 | 0.0000 | 0.0019 | 0.3827 | 0.6418 | 1.0000 | 0.0028 | 0.3308 | 0.9746 |
| 0.8556 | 0.1148 | 0.2439 | 0.0569 | 0.6850 | 0.5691 | 0.0727 | 1.7259 | 0.3630 | 63.4337 | 0.2990 | 0.0000 | 0.0000 | 0.8846 | 0.4434 | 0.9852 | 0.0307 | 0.3570 | 0.9710 |
| 0.7960 | 0.0217 | 0.0241 | 0.0032 | 0.8120 | 1.0075 | 0.1053 | 1.9751 | 0.4876 | 72.2297 | 0.0651 | 0.0000 | 0.0000 | 0.1703 | 0.4096 | 0.8378 | 0.0217 | 0.0851 | 0.8369 |
| 0.9437 | 0.0531 | 0.0817 | 0.0308 | 0.8875 | 0.3526 | 0.0455 | 2.4543 | 0.7271 | 65.0632 | 0.1369 | 0.0000 | 0.0000 | 0.4098 | 0.6412 | 0.9968 | 0.0000 | 0.1369 | 0.9936 |
| 0.8193 | 0.0721 | 0.0764 | 0.0914 | 0.7607 | 0.7468 | 0.0816 | 1.8798 | 0.4399 | 73.6651 | 0.2350 | 0.0000 | 0.0007 | 0.7093 | 0.2757 | 0.9279 | 0.1100 | 0.4164 | 0.9250 |
| 0.7427 | 0.0378 | 0.1490 | 0.0218 | 0.6839 | 0.5705 | 0.0768 | 1.4368 | 0.2184 | 70.0077 | 0.1308 | 0.0000 | 0.0000 | 0.7376 | 0.4593 | 0.8539 | 0.1308 | 0.1693 | 0.8503 |
| 0.9850 | 0.0000 | 0.0150 | 0.0150 | 0.9700 | 0.4291 | 0.0274 | 1.9949 | 0.4975 | 72.5462 | 0.2600 | 0.0000 | 0.0050 | 0.5900 | 0.3000 | 0.9900 | 0.0250 | 0.2850 | 0.9800 |
| 0.9945 | 0.0011 | 0.0343 | 0.0044 | 0.9602 | 0.4716 | 0.0511 | 2.4102 | 0.7051 | 62.7409 | 0.0631 | 0.0000 | 0.0000 | 0.2954 | 0.6737 | 0.9978 | 0.0022 | 0.1460 | 0.9934 |
| 0.4882 | 0.0021 | 0.1079 | 0.0556 | 0.4348 | 0.5757 | 0.0648 | 1.9435 | 0.4717 | 73.7186 | 0.0823 | 0.0000 | 0.0000 | 0.1688 | 0.2179 | 0.5983 | 0.0011 | 0.0321 | 0.5972 |
| 0.4468 | 0.0012 | 0.2460 | 0.2472 | 0.2607 | 0.5415 | 0.0646 | 1.8149 | 0.4075 | 84.5611 | 0.0673 | 0.0000 | 0.0000 | 0.1652 | 0.1285 | 0.7540 | 0.0306 | 0.0661 | 0.6304 |
| 0.5047 | 0.0031 | 0.0538 | 0.1086 | 0.4488 | 0.4966 | 0.0677 | 1.9020 | 0.4510 | 69.3915 | 0.0062 | 0.0000 | 0.0000 | 0.1892 | 0.2358 | 0.6112 | 0.0010 | 0.0589 | 0.5584 |
| 0.6427 | 0.0221 | 0.0229 | 0.1585 | 0.5229 | 0.5195 | 0.0603 | 1.9093 | 0.4546 | 71.8722 | 0.2579 | 0.0000 | 0.0000 | 0.4629 | 0.2035 | 0.7043 | 0.0213 | 0.1861 | 0.6435 |
| 0.7391 | 0.1395 | 0.2096 | 0.0729 | 0.5094 | 0.7059 | 0.0760 | 1.7539 | 0.3770 | 59.9328 | 0.3317 | 0.0000 | 0.0173 | 0.6662 | 0.4712 | 0.9306 | 0.1221 | 0.2658 | 0.8931 |
| 0.5902 | 0.0000 | 0.0000 | 0.4112 | 0.3156 | 0.6347 | 0.0701 | 1.9981 | 0.4991 | 80.1922 | 0.2404 | 0.0000 | 0.0000 | 0.1776 | 0.1052 | 0.7268 | 0.0000 | 0.0697 | 0.4522 |
| 0.8813 | 0.0209 | 0.0225 | 0.1567 | 0.8014 | 1.0632 | 0.1196 | 1.5024 | 0.2512 | 65.7666 | 0.0784 | 0.0000 | 0.0000 | 0.2413 | 0.0830 | 0.9806 | 0.0008 | 0.0434 | 0.9395 |
| 0.9790 | 0.0022 | 0.3593 | 0.0561 | 0.5659 | 0.8791 | 0.1018 | 2.0908 | 0.5454 | 59.3366 | 0.0576 | 0.0000 | 0.0000 | 0.8481 | 0.7522 | 0.9805 | 0.0763 | 0.0973 | 0.9805 |
| 0.9540 | 0.0009 | 0.0938 | 0.0009 | 0.8602 | 1.1283 | 0.1188 | 1.4325 | 0.2162 | 59.8732 | 0.0469 | 0.0000 | 0.0000 | 0.5248 | 0.3408 | 0.9540 | 0.0911 | 0.0234 | 0.9522 |
| 0.8622 | 0.0035 | 0.0071 | 0.2235 | 0.6802 | 0.5877 | 0.0775 | 1.8225 | 0.4113 | 65.4054 | 0.1378 | 0.0000 | 0.0009 | 0.2208 | 0.1652 | 0.9099 | 0.0044 | 0.0336 | 0.8171 |
| 0.8090 | 0.0019 | 0.0038 | 0.1204 | 0.6877 | 1.3212 | 0.1340 | 1.4148 | 0.2074 | 69.1692 | 0.0960 | 0.0000 | 0.0000 | 0.2211 | 0.1938 | 0.8119 | 0.0009 | 0.0056 | 0.7168 |
| 0.6707 | 0.0019 | 0.0019 | 0.1656 | 0.5987 | 1.0976 | 0.1195 | 1.4151 | 0.2076 | 66.8174 | 0.0739 | 0.0000 | 0.0000 | 0.1310 | 0.0992 | 0.7661 | 0.0019 | 0.0318 | 0.7175 |
| 0.8447 | 0.0394 | 0.1368 | 0.0587 | 0.6499 | 0.8867 | 0.0983 | 1.4261 | 0.2130 | 69.7977 | 0.1383 | 0.0000 | 0.0008 | 0.7241 | 0.5046 | 0.8833 | 0.0417 | 0.2944 | 0.8617 |
| 0.4676 | 0.0343 | 0.0423 | 0.0740 | 0.3884 | 0.5513 | 0.0606 | 1.9160 | 0.4580 | 76.3715 | 0.1413 | 0.0000 | 0.0000 | 0.3236 | 0.2774 | 0.5020 | 0.0066 | 0.0793 | 0.4954 |
| 1.0000 | 0.0000 | 0.4780 | 0.0034 | 0.5186 | 0.9734 | 0.1058 | 1.9966 | 0.4983 | 59.6119 | 0.0000 | 0.0000 | 0.0000 | 0.9966 | 0.8441 | 0.9864 | 0.1492 | 0.2983 | 0.9864 |
| 0.9643 | 0.0000 | 0.0357 | 0.0357 | 0.8929 | 0.3802 | 0.0450 | 2.0769 | 0.5385 | 72.5315 | 0.6071 | 0.0000 | 0.0000 | 0.3929 | 0.2857 | 0.9643 | 0.2143 | 0.0714 | 0.9286 |
| 1.0000 | 0.0000 | 0.3190 | 0.0123 | 0.6687 | 1.4057 | 0.1311 | 1.7569 | 0.3785 | 67.5820 | 0.0031 | 0.0000 | 0.0000 | 0.9908 | 0.7853 | 0.9202 | 0.1748 | 0.4571 | 0.9202 |
| 0.9962 | 0.0038 | 0.1000 | 0.0038 | 0.8962 | 1.1500 | 0.1259 | 2.1462 | 0.5731 | 52.7670 | 0.0077 | 0.0000 | 0.0000 | 0.9923 | 0.8462 | 1.0000 | 0.0154 | 0.7038 | 1.0000 |
| 0.9283 | 0.0000 | 0.0609 | 0.0143 | 0.9211 | 0.7438 | 0.0839 | 2.0827 | 0.5414 | 69.4875 | 0.0108 | 0.0000 | 0.0036 | 0.9498 | 0.6237 | 0.9928 | 0.3118 | 0.6129 | 0.9928 |
| 0.9020 | 0.0000 | 0.1059 | 0.0000 | 0.8941 | 0.8012 | 0.0913 | 2.0157 | 0.5078 | 68.6424 | 0.0235 | 0.0000 | 0.0039 | 0.6784 | 0.4353 | 0.9961 | 0.2000 | 0.5020 | 1.0000 |
| 0.9854 | 0.0000 | 0.0146 | 0.0000 | 0.9854 | 0.3997 | 0.0592 | 2.0292 | 0.5146 | 75.4777 | 0.0000 | 0.0000 | 0.0000 | 0.7810 | 0.2117 | 1.0000 | 0.0584 | 0.2336 | 1.0000 |
| 1.0000 | 0.0000 | 0.3371 | 0.0000 | 0.6629 | 0.9285 | 0.1039 | 1.9621 | 0.4811 | 65.3216 | 0.0076 | 0.0000 | 0.0000 | 0.8902 | 0.6970 | 0.9015 | 0.1174 | 0.6894 | 0.9924 |
| 0.9925 | 0.0037 | 0.0299 | 0.0000 | 0.9664 | 0.9538 | 0.1071 | 2.0637 | 0.5318 | 76.6183 | 0.0224 | 0.0000 | 0.0149 | 0.9813 | 0.7164 | 0.9813 | 0.1866 | 0.5933 | 0.9963 |
| 1.0000 | 0.0000 | 0.1353 | 0.0048 | 0.8599 | 0.2515 | 0.0373 | 1.9792 | 0.4896 | 76.9288 | 0.0048 | 0.0000 | 0.0000 | 0.6812 | 0.4106 | 1.0000 | 0.2126 | 0.3527 | 0.9952 |
| 0.9842 | 0.0000 | 0.0158 | 0.0000 | 0.9842 | 0.7687 | 0.0955 | 2.0316 | 0.5158 | 63.4842 | 0.0119 | 0.0000 | 0.0000 | 0.9960 | 0.5534 | 1.0000 | 0.3755 | 0.5929 | 1.0000 |
| 1.0000 | 0.0000 | 0.0035 | 0.0000 | 0.9965 | 0.4988 | 0.0610 | 2.0035 | 0.5017 | 78.1560 | 0.0069 | 0.0000 | 0.0035 | 1.0000 | 0.2837 | 0.9965 | 0.2976 | 0.3668 | 1.0000 |
| 0.9856 | 0.0036 | 0.0975 | 0.0036 | 0.8917 | 0.5726 | 0.0696 | 2.1927 | 0.5964 | 76.9134 | 0.0072 | 0.0000 | 0.0000 | 0.9603 | 0.4404 | 0.9928 | 0.1083 | 0.3646 | 0.9892 |
| 0.9964 | 0.0000 | 0.0755 | 0.0000 | 0.9245 | 0.6158 | 0.0773 | 2.5547 | 0.7774 | 66.9501 | 0.0072 | 0.0000 | 0.0000 | 0.9928 | 0.8813 | 0.9964 | 0.2338 | 0.5180 | 0.9964 |
| 0.9810 | 0.0000 | 0.1185 | 0.6635 | 0.1801 | 0.2165 | 0.0324 | 1.9577 | 0.4789 | 57.8812 | 0.0000 | 0.0000 | 0.0000 | 0.1374 | 0.1185 | 1.0000 | 0.0000 | 0.0190 | 0.7393 |
| 0.9966 | 0.0000 | 0.2911 | 0.0000 | 0.7089 | 1.3161 | 0.1350 | 2.2639 | 0.6319 | 59.4048 | 0.0068 | 0.0000 | 0.0000 | 0.9966 | 0.9692 | 0.9966 | 0.0548 | 0.6370 | 0.9966 |
| 0.5157 | 0.0000 | 0.0194 | 0.9201 | 0.0605 | 2.5139 | 0.2282 | 1.0000 | 0.0000 | 48.7692 | 0.4843 | 0.0000 | 0.0000 | 0.0000 | 0.0000 | 1.0000 | 0.0605 | 0.0000 | 0.9806 |
| 0.3882 | 0.0000 | 0.0263 | 0.8750 | 0.0987 | 2.6683 | 0.2318 | 1.0000 | 0.0000 | 48.3784 | 0.5592 | 0.0000 | 0.0000 | 0.0000 | 0.0000 | 1.0000 | 0.0987 | 0.0000 | 0.9737 |
| 0.2109 | 0.0000 | 0.0078 | 0.9844 | 0.0078 | 2.7525 | 0.2444 | 1.0000 | 0.0000 | 49.8740 | 0.7813 | 0.0000 | 0.0000 | 0.0000 | 0.0000 | 1.0000 | 0.0078 | 0.0000 | 0.9922 |
| 0.9763 | 0.0034 | 0.4136 | 0.0441 | 0.4508 | 1.1160 | 0.1227 | 1.6007 | 0.3003 | 77.4799 | 0.2068 | 0.0000 | 0.0000 | 0.5186 | 0.5254 | 0.9627 | 0.0034 | 0.0169 | 0.9085 |
| 1.0000 | 0.0000 | 0.0057 | 0.1149 | 0.6494 | 0.1068 | 0.0173 | 2.6623 | 0.8312 | 65.0120 | 0.2759 | 0.0000 | 0.0000 | 0.3276 | 0.8161 | 1.0000 | 0.0000 | 0.0057 | 0.7644 |
| 0.9283 | 0.0000 | 0.0591 | 0.0042 | 0.9156 | 0.2709 | 0.0369 | 2.0476 | 0.5238 | 81.2313 | 0.0042 | 0.0000 | 0.0000 | 0.8228 | 0.6456 | 0.9789 | 0.2363 | 0.7257 | 0.8945 |
| 0.9821 | 0.0000 | 0.0268 | 0.0357 | 0.9375 | 0.3622 | 0.0462 | 1.9375 | 0.4688 | 65.0346 | 0.0000 | 0.0000 | 0.0000 | 0.2143 | 0.0848 | 1.0000 | 0.1786 | 0.4286 | 0.9911 |
| 0.6250 | 0.0000 | 0.0000 | 0.0000 | 1.0000 | 0.7237 | 0.0960 | 1.9938 | 0.4969 | 91.2071 | 0.0313 | 0.0000 | 0.0000 | 0.4250 | 0.3875 | 1.0000 | 0.0125 | 0.4188 | 0.9938 |
| 0.9652 | 0.0000 | 0.0000 | 0.0087 | 0.9913 | 0.6895 | 0.0959 | 2.0351 | 0.5175 | 93.3836 | 0.0000 | 0.0000 | 0.0000 | 0.9217 | 0.9130 | 1.0000 | 0.0000 | 0.8783 | 0.9652 |
| 0.9524 | 0.0000 | 0.0238 | 0.0060 | 0.9702 | 0.4649 | 0.0624 | 2.0123 | 0.5061 | 83.4000 | 0.3274 | 0.0000 | 0.0000 | 0.8036 | 0.4821 | 1.0000 | 0.0060 | 0.4583 | 0.9881 |

| | | | | | | | | | | | | | | | | | | |
|---|---|---|---|---|---|---|---|---|---|---|---|---|---|---|---|---|---|---|
| 0.9848 | 0.0000 | 0.2424 | 0.1212 | 0.6364 | 0.5303 | 0.0752 | 1.7368 | 0.3684 | 82.0202 | 0.0303 | 0.0000 | 0.0000 | 0.4697 | 0.4091 | 1.0000 | 0.0000 | 0.3939 | 0.7576 |
| 1.0000 | 0.0000 | 0.0099 | 0.0148 | 0.9754 | 0.5571 | 0.0703 | 2.1188 | 0.5594 | 72.8644 | 0.0296 | 0.0000 | 0.0000 | 0.9458 | 0.3941 | 1.0000 | 0.2414 | 0.5567 | 0.9951 |
| 1.0000 | 0.0000 | 0.0042 | 0.0000 | 0.9958 | 1.3493 | 0.1400 | 2.1046 | 0.5523 | 77.4312 | 0.0377 | 0.0000 | 0.0000 | 1.0000 | 0.7238 | 1.0000 | 0.2008 | 0.8954 | 0.9958 |
| 0.9959 | 0.0000 | 0.1245 | 0.0041 | 0.8714 | 0.4464 | 0.0524 | 1.8945 | 0.4473 | 66.4633 | 0.0041 | 0.0000 | 0.0000 | 0.9959 | 0.3610 | 1.0000 | 0.4481 | 0.6722 | 1.0000 |
| 0.9128 | 0.0000 | 0.4709 | 0.0000 | 0.5233 | 0.5291 | 0.0744 | 1.3736 | 0.1868 | 70.2934 | 0.0058 | 0.0000 | 0.0000 | 0.5581 | 0.0523 | 0.9942 | 0.3547 | 0.0523 | 0.9826 |
| 0.9862 | 0.0069 | 0.3069 | 0.1552 | 0.3276 | 0.8900 | 0.1003 | 1.6540 | 0.3270 | 73.5383 | 0.2690 | 0.0000 | 0.0000 | 0.4621 | 0.7448 | 0.9793 | 0.0000 | 0.0345 | 0.8414 |
| 0.9608 | 0.0196 | 0.1373 | 0.0588 | 0.8039 | 0.6063 | 0.0736 | 1.6327 | 0.3163 | 75.3153 | 0.3725 | 0.0000 | 0.0000 | 0.6471 | 0.3922 | 0.9412 | 0.0784 | 0.1961 | 0.8235 |
| 0.9859 | 0.0000 | 0.0423 | 0.5775 | 0.3803 | 0.8377 | 0.1041 | 2.1212 | 0.5606 | 78.6016 | 0.1690 | 0.0000 | 0.0141 | 0.3521 | 0.0986 | 0.9437 | 0.0563 | 0.1831 | 0.3239 |
| 0.8095 | 0.0381 | 0.0952 | 0.0952 | 0.8095 | 0.5394 | 0.0606 | 1.9136 | 0.4568 | 67.4311 | 0.4381 | 0.0000 | 0.0095 | 0.6571 | 0.2095 | 0.9238 | 0.0000 | 0.1619 | 0.6286 |
| 0.9194 | 0.0806 | 0.1613 | 0.2581 | 0.5806 | 0.5155 | 0.0605 | 1.7234 | 0.3617 | 74.1417 | 0.2742 | 0.0000 | 0.0000 | 0.5161 | 0.3226 | 0.7581 | 0.1290 | 0.3226 | 0.6935 |
| 0.9091 | 0.0364 | 0.5818 | 0.1455 | 0.2727 | 0.9844 | 0.1136 | 1.8750 | 0.4375 | 56.3583 | 0.0909 | 0.0000 | 0.0000 | 0.8364 | 0.2182 | 0.8727 | 0.4909 | 0.6182 | 0.8364 |
| 0.9770 | 0.0115 | 0.2414 | 0.3218 | 0.4368 | 0.6054 | 0.0718 | 1.9833 | 0.4917 | 63.4888 | 0.0460 | 0.0000 | 0.0000 | 0.5977 | 0.4483 | 0.7011 | 0.1379 | 0.5172 | 0.6437 |
| 0.9750 | 0.0000 | 0.1000 | 0.3875 | 0.5125 | 0.3658 | 0.0421 | 1.9811 | 0.4906 | 76.1475 | 0.3250 | 0.0000 | 0.0000 | 0.2250 | 0.1375 | 0.6625 | 0.0625 | 0.2125 | 0.6000 |
| 0.9722 | 0.0000 | 0.1944 | 0.1389 | 0.6667 | 0.5894 | 0.0689 | 2.0938 | 0.5469 | 71.8222 | 0.3056 | 0.0000 | 0.0000 | 0.6111 | 0.3889 | 0.9167 | 0.1111 | 0.3611 | 0.8333 |
| 0.8940 | 0.0993 | 0.1126 | 0.2715 | 0.6159 | 0.5854 | 0.0803 | 1.9000 | 0.4500 | 69.2162 | 0.1126 | 0.0000 | 0.0000 | 0.7219 | 0.5894 | 0.9868 | 0.0066 | 0.5629 | 0.9868 |
| 0.9535 | 0.0116 | 0.0698 | 0.0349 | 0.8953 | 0.6370 | 0.0725 | 2.1566 | 0.5783 | 72.5404 | 0.2093 | 0.0000 | 0.0000 | 0.7558 | 0.4767 | 0.9767 | 0.1860 | 0.6163 | 0.9302 |
| 0.9103 | 0.0641 | 0.6282 | 0.0385 | 0.3333 | 1.4286 | 0.1557 | 1.7237 | 0.3618 | 50.1327 | 0.0897 | 0.0000 | 0.0000 | 0.9615 | 0.2949 | 0.9615 | 0.3590 | 0.6154 | 0.7436 |
| 0.7966 | 0.1610 | 0.6780 | 0.0508 | 0.2712 | 1.1098 | 0.1228 | 1.6903 | 0.3451 | 59.3156 | 0.0932 | 0.0000 | 0.0000 | 0.9153 | 0.4237 | 0.9237 | 0.2712 | 0.5254 | 0.6949 |
| 0.9727 | 0.0000 | 0.0545 | 0.0909 | 0.8545 | 0.6442 | 0.0870 | 1.8333 | 0.4167 | 61.4615 | 0.0273 | 0.0000 | 0.0000 | 0.6818 | 0.6000 | 0.9182 | 0.0273 | 0.7000 | 0.9000 |
| 0.8364 | 0.1455 | 0.5364 | 0.0545 | 0.4091 | 0.8630 | 0.1017 | 1.6190 | 0.3095 | 58.1690 | 0.3000 | 0.0000 | 0.0000 | 0.8000 | 0.3909 | 0.9455 | 0.2273 | 0.6091 | 0.9273 |
| 0.9760 | 0.0160 | 0.0480 | 0.3280 | 0.6240 | 0.5424 | 0.0746 | 1.6341 | 0.3171 | 64.1794 | 0.0320 | 0.0000 | 0.0000 | 0.3120 | 0.2560 | 0.9840 | 0.0240 | 0.2560 | 0.9840 |
| 0.8537 | 0.1301 | 0.1626 | 0.3659 | 0.4715 | 0.4690 | 0.0604 | 1.7778 | 0.3889 | 63.2858 | 0.1545 | 0.0000 | 0.0000 | 0.5528 | 0.2846 | 0.6667 | 0.0813 | 0.3577 | 0.6179 |
| 1.0000 | 0.0000 | 0.0329 | 0.0041 | 0.9630 | 1.0076 | 0.1126 | 2.0617 | 0.5309 | 69.5182 | 0.0082 | 0.0000 | 0.0000 | 1.0000 | 0.7078 | 1.0000 | 0.2675 | 0.7819 | 0.9835 |
| 0.9957 | 0.0043 | 0.0172 | 0.0043 | 0.9784 | 0.6790 | 0.0798 | 2.1853 | 0.5927 | 67.8149 | 0.0086 | 0.0000 | 0.0000 | 0.9957 | 0.6121 | 0.9957 | 0.3707 | 0.9009 | 0.9914 |
| 1.0000 | 0.0000 | 0.0773 | 0.3144 | 0.6031 | 0.6480 | 0.0746 | 1.9637 | 0.4819 | 70.0704 | 0.0155 | 0.0000 | 0.0000 | 0.6804 | 0.3093 | 0.9897 | 0.3351 | 0.5464 | 0.9691 |
| 1.0000 | 0.0000 | 0.1865 | 0.0829 | 0.7254 | 1.0685 | 0.1151 | 2.0417 | 0.5208 | 69.1187 | 0.0518 | 0.0000 | 0.0000 | 0.9119 | 0.5699 | 0.9896 | 0.2487 | 0.6062 | 0.9482 |
| 0.9944 | 0.0000 | 0.1685 | 0.0056 | 0.8258 | 1.1265 | 0.1251 | 1.8371 | 0.4185 | 73.3839 | 0.0112 | 0.0000 | 0.0000 | 0.9888 | 0.7472 | 0.9888 | 0.1854 | 0.6292 | 0.9494 |
| 1.0000 | 0.0000 | 0.1182 | 0.0091 | 0.8727 | 1.1493 | 0.1244 | 1.8950 | 0.4475 | 71.2500 | 0.0455 | 0.0000 | 0.0000 | 0.9864 | 0.6182 | 0.9909 | 0.2545 | 0.6636 | 0.9227 |
| 0.9905 | 0.0000 | 0.1469 | 0.0095 | 0.8436 | 0.7591 | 0.0912 | 2.1524 | 0.5762 | 64.7907 | 0.0142 | 0.0000 | 0.0000 | 0.9905 | 0.5687 | 0.9905 | 0.4028 | 0.8246 | 0.9905 |
| 0.9947 | 0.0000 | 0.0474 | 0.0053 | 0.9474 | 1.0723 | 0.1173 | 2.0947 | 0.5474 | 75.4031 | 0.0368 | 0.0000 | 0.0000 | 0.9895 | 0.6947 | 0.9947 | 0.1579 | 0.7421 | 0.9737 |
| 0.7991 | 0.0000 | 0.0228 | 0.0046 | 0.9041 | 0.8313 | 0.1069 | 1.1961 | 0.0980 | 91.5335 | 0.1416 | 0.0000 | 0.0000 | 0.0411 | 0.1553 | 0.9315 | 0.1142 | 0.7306 | 0.9315 |
| 0.6557 | 0.0000 | 0.0820 | 0.2131 | 0.6393 | 0.6093 | 0.0723 | 1.5221 | 0.2611 | 85.2451 | 0.3443 | 0.0000 | 0.0000 | 0.2049 | 0.3934 | 0.9262 | 0.2131 | 0.1230 | 0.8852 |
| 0.6449 | 0.3551 | 0.1589 | 0.0187 | 0.7757 | 0.6681 | 0.0838 | 2.7048 | 0.8524 | 80.2387 | 0.0935 | 0.0000 | 0.0000 | 0.1776 | 0.6262 | 1.0000 | 0.0467 | 0.0000 | 0.5794 |
| 0.4759 | 0.5241 | 0.2759 | 0.0000 | 0.6897 | 0.9228 | 0.1131 | 2.6690 | 0.8345 | 78.3797 | 0.0828 | 0.0000 | 0.0000 | 0.2759 | 0.4759 | 1.0000 | 0.0345 | 0.0000 | 0.4276 |
| 0.3762 | 0.6238 | 0.1980 | 0.0000 | 0.7772 | 1.0115 | 0.1223 | 2.7000 | 0.8500 | 81.3363 | 0.0594 | 0.0000 | 0.0000 | 0.2327 | 0.3663 | 0.9901 | 0.0248 | 0.0000 | 0.3168 |
| 0.6346 | 0.3654 | 0.3846 | 0.0000 | 0.5673 | 0.8448 | 0.1047 | 2.5096 | 0.7548 | 78.4070 | 0.0673 | 0.0000 | 0.0000 | 0.3846 | 0.6346 | 1.0000 | 0.0192 | 0.0000 | 0.5962 |
| 0.5556 | 0.4444 | 0.2339 | 0.0000 | 0.7368 | 0.8086 | 0.1002 | 2.6527 | 0.8263 | 85.7263 | 0.0585 | 0.0000 | 0.0000 | 0.2515 | 0.5439 | 0.9883 | 0.0292 | 0.0000 | 0.4737 |
| 0.5190 | 0.4810 | 0.2152 | 0.0000 | 0.7848 | 0.8511 | 0.1050 | 2.6709 | 0.8354 | 86.4208 | 0.0633 | 0.0000 | 0.0000 | 0.2405 | 0.5190 | 1.0000 | 0.0633 | 0.0000 | 0.4304 |
| 0.4571 | 0.5429 | 0.2857 | 0.0000 | 0.7143 | 0.9991 | 0.1212 | 2.6500 | 0.8250 | 63.2174 | 0.0357 | 0.0000 | 0.0000 | 0.2857 | 0.4571 | 1.0000 | 0.0357 | 0.0000 | 0.4071 |
| 0.7077 | 0.2615 | 0.0769 | 0.0000 | 0.6615 | 0.4780 | 0.0599 | 2.5231 | 0.7615 | 79.6267 | 0.3385 | 0.0000 | 0.0000 | 0.1077 | 0.7077 | 0.9692 | 0.0769 | 0.0000 | 0.6000 |
| 0.5094 | 0.4780 | 0.1195 | 0.0000 | 0.7736 | 0.7773 | 0.0946 | 2.7261 | 0.8631 | 83.2090 | 0.1384 | 0.0000 | 0.0000 | 0.1195 | 0.5094 | 0.9874 | 0.0314 | 0.0000 | 0.4528 |
| 0.6238 | 0.3762 | 0.3762 | 0.0000 | 0.5990 | 0.8124 | 0.1026 | 2.5545 | 0.7772 | 76.8883 | 0.0495 | 0.0000 | 0.0000 | 0.3861 | 0.6238 | 1.0000 | 0.0248 | 0.0000 | 0.5891 |
| 0.7286 | 0.2714 | 0.5429 | 0.0000 | 0.4571 | 0.7871 | 0.1022 | 2.3643 | 0.6821 | 88.5374 | 0.0500 | 0.0000 | 0.0000 | 0.5714 | 0.7143 | 1.0000 | 0.0357 | 0.0000 | 0.6786 |
| 0.6080 | 0.3819 | 0.3819 | 0.0000 | 0.6181 | 0.8327 | 0.1052 | 2.5729 | 0.7864 | 72.5311 | 0.0251 | 0.0000 | 0.0000 | 0.3920 | 0.6080 | 0.9899 | 0.0251 | 0.0000 | 0.5729 |
| 0.9989 | 0.0000 | 0.8308 | 0.0277 | 0.1416 | 0.7133 | 0.0947 | 1.9137 | 0.4569 | 78.2965 | 0.0564 | 0.0000 | 0.0000 | 0.9148 | 0.7765 | 0.9989 | 0.0000 | 0.0000 | 1.0000 |
| 0.9994 | 0.0006 | 0.1618 | 0.0000 | 0.8382 | 0.5736 | 0.0757 | 2.3520 | 0.6760 | 79.8501 | 0.0328 | 0.0000 | 0.0006 | 0.9839 | 0.8690 | 0.9839 | 0.0816 | 0.8529 | 0.9833 |
| 0.3840 | 0.6127 | 0.3333 | 0.0033 | 0.6618 | 1.1056 | 0.1342 | 2.6579 | 0.8289 | 83.2585 | 0.0016 | 0.0000 | 0.0000 | 0.3301 | 0.3693 | 0.9951 | 0.0000 | 0.0000 | 0.3824 |
| 0.9447 | 0.0000 | 0.7190 | 0.0000 | 0.2810 | 0.5801 | 0.0824 | 1.9712 | 0.4856 | 77.5623 | 0.1681 | 0.0000 | 0.0000 | 0.9148 | 0.8042 | 1.0000 | 0.0011 | 0.0000 | 0.9712 |
| 1.0000 | 0.0000 | 0.0048 | 0.2186 | 0.7287 | 0.2759 | 0.0289 | 2.6061 | 0.8030 | 66.4967 | 0.0268 | 0.0000 | 0.0010 | 0.0077 | 0.1246 | 0.3758 | 0.0010 | 0.0019 | 0.3720 |
| 1.0000 | 0.0000 | 0.1903 | 0.3568 | 0.4529 | 0.9915 | 0.1077 | 2.4520 | 0.7260 | 88.5147 | 0.0000 | 0.0000 | 0.0000 | 0.0010 | 0.5480 | 1.0000 | 0.0000 | 0.0010 | 1.0000 |
| 1.0000 | 0.0000 | 0.8123 | 0.0000 | 0.1877 | 0.6009 | 0.0852 | 1.9097 | 0.4549 | 83.0579 | 0.0939 | 0.0000 | 0.0000 | 0.9964 | 0.9964 | 1.0000 | 0.0000 | 0.0000 | 0.8195 |

| | | | | | | | | | | | | | | | | | | |
|---|---|---|---|---|---|---|---|---|---|---|---|---|---|---|---|---|---|---|
| 1.0000 | 0.0000 | 0.9976 | 0.0000 | 0.0024 | 0.3638 | 0.0512 | 2.4988 | 0.7494 | 63.0714 | 0.4996 | 0.0000 | 0.0000 | 0.9992 | 0.9992 | 1.0000 | 0.0000 | 0.0000 | 1.0000 |
| 1.0000 | 0.0000 | 0.3992 | 0.0000 | 0.6008 | 3.2104 | 0.2087 | 1.4012 | 0.2006 | 79.2531 | 0.0020 | 0.0000 | 0.0000 | 0.6008 | 0.5988 | 1.0000 | 0.0000 | 0.0000 | 0.8004 |
| 0.7635 | 0.0000 | 0.3932 | 0.2085 | 0.3714 | 0.8798 | 0.1018 | 2.2112 | 0.6056 | 53.7691 | 0.0010 | 0.0000 | 0.0000 | 0.6297 | 0.0571 | 1.0000 | 0.1338 | 0.1328 | 0.7915 |
| 0.9984 | 0.0000 | 0.0000 | 0.1577 | 0.5252 | 0.9743 | 0.0532 | 2.1429 | 0.5714 | 86.2054 | 0.5126 | 0.0000 | 0.0016 | 0.2035 | 0.5158 | 0.9968 | 0.0016 | 0.0000 | 0.9558 |
| 0.9991 | 0.0009 | 0.0241 | 0.0232 | 0.9527 | 0.1474 | 0.0222 | 1.9527 | 0.4763 | 66.1893 | 0.0232 | 0.0000 | 0.0000 | 1.0000 | 0.8822 | 1.0000 | 0.0946 | 0.1642 | 1.0000 |
| 0.9981 | 0.0000 | 0.7113 | 0.0038 | 0.2849 | 0.9876 | 0.1195 | 1.7151 | 0.3575 | 84.3211 | 0.0491 | 0.0000 | 0.0000 | 0.8075 | 0.7585 | 1.0000 | 0.0000 | 0.0000 | 0.9528 |
| 0.9984 | 0.0000 | 0.7552 | 0.0032 | 0.2417 | 0.8337 | 0.1010 | 1.9173 | 0.4587 | 88.1613 | 0.1987 | 0.0000 | 0.0000 | 0.8378 | 0.7981 | 1.0000 | 0.0000 | 0.0000 | 0.9603 |
| 0.9983 | 0.0000 | 0.6221 | 0.0033 | 0.3746 | 1.2332 | 0.1389 | 1.6271 | 0.3135 | 66.2049 | 0.0033 | 0.0000 | 0.0000 | 0.6650 | 0.6650 | 1.0000 | 0.0000 | 0.0000 | 0.9983 |
| 1.0000 | 0.0000 | 0.7968 | 0.0000 | 0.2032 | 1.9849 | 0.1571 | 1.8008 | 0.4004 | 59.4394 | 0.0000 | 0.0000 | 0.0000 | 0.8008 | 0.8008 | 1.0000 | 0.0000 | 0.0000 | 1.0000 |
| 1.0000 | 0.0000 | 0.9667 | 0.0000 | 0.0333 | 0.6022 | 0.0844 | 2.1667 | 0.5833 | 89.4246 | 0.1333 | 0.0000 | 0.0000 | 0.9667 | 1.0000 | 1.0000 | 0.0000 | 0.0000 | 1.0000 |
| 1.0000 | 0.0000 | 0.0013 | 0.5290 | 0.4037 | 7.1573 | 0.1847 | 1.9982 | 0.4991 | 65.9733 | 0.2005 | 0.0000 | 0.0000 | 0.2665 | 0.2005 | 0.9987 | 0.0000 | 0.0000 | 0.9974 |
| 1.0000 | 0.0000 | 0.0886 | 0.0009 | 0.9105 | 0.4700 | 0.0548 | 1.9105 | 0.4553 | 86.4743 | 0.0895 | 0.0000 | 0.0000 | 1.0000 | 0.9088 | 1.0000 | 0.0018 | 0.2675 | 1.0000 |
| 0.4262 | 0.5723 | 0.3059 | 0.0015 | 0.6925 | 1.0574 | 0.1294 | 2.6778 | 0.8389 | 90.0941 | 0.0015 | 0.0000 | 0.0000 | 0.3059 | 0.3805 | 0.9604 | 0.0000 | 0.0000 | 0.3881 |
| 0.9987 | 0.0013 | 0.0344 | 0.1323 | 0.8333 | 0.2303 | 0.0352 | 1.8981 | 0.4491 | 76.5819 | 0.3995 | 0.0000 | 0.0000 | 0.8320 | 0.5979 | 1.0000 | 0.0000 | 0.0013 | 1.0000 |
| 1.0000 | 0.0000 | 0.1298 | 0.1285 | 0.7416 | 0.8172 | 0.0851 | 2.1289 | 0.5644 | 65.7139 | 0.4833 | 0.0000 | 0.0000 | 0.4833 | 0.2571 | 0.9974 | 0.0000 | 0.0000 | 0.9961 |
| 0.2722 | 0.6710 | 0.2165 | 0.0011 | 0.7278 | 1.1492 | 0.1383 | 2.7662 | 0.8831 | 69.5238 | 0.0011 | 0.0000 | 0.0000 | 0.2154 | 0.2680 | 0.9443 | 0.0000 | 0.0000 | 0.2744 |
| 0.3000 | 0.0000 | 0.0000 | 0.0400 | 0.2400 | 0.1242 | 0.0217 | 1.6154 | 0.3077 | 49.8371 | 0.0000 | 0.0000 | 0.0000 | 0.0000 | 0.0800 | 0.3000 | 0.0000 | 0.0000 | 0.1400 |
| 0.9972 | 0.0000 | 0.0037 | 0.1161 | 0.8802 | 0.8282 | 0.1063 | 1.9060 | 0.4530 | 69.3492 | 0.0940 | 0.0000 | 0.0009 | 0.9751 | 0.7871 | 0.9991 | 0.0931 | 0.7650 | 1.0000 |
| 0.2246 | 0.7754 | 0.1278 | 0.0323 | 0.8400 | 1.2398 | 0.1455 | 2.8385 | 0.9193 | 50.4608 | 0.0012 | 0.0000 | 0.0000 | 0.1253 | 0.1861 | 1.0000 | 0.0000 | 0.0000 | 0.2246 |
| 1.0000 | 0.0000 | 0.6821 | 0.0000 | 0.3179 | 0.5078 | 0.0720 | 2.1040 | 0.5520 | 74.4101 | 0.1584 | 0.0000 | 0.0000 | 0.8930 | 0.8657 | 0.9990 | 0.0000 | 0.0000 | 0.9727 |
| 0.6057 | 0.0000 | 0.0019 | 0.0959 | 0.4921 | 0.1812 | 0.0262 | 2.8152 | 0.9076 | 66.0799 | 0.0000 | 0.0000 | 0.0000 | 0.0164 | 0.2391 | 0.3685 | 0.0000 | 0.0013 | 0.3678 |
| 1.0000 | 0.0000 | 0.0020 | 0.3806 | 0.6174 | 10.7641 | 0.2522 | 2.0000 | 0.5000 | 70.3283 | 0.0030 | 0.0000 | 0.0000 | 0.3117 | 0.1043 | 1.0000 | 0.1022 | 0.2065 | 0.9970 |
| 0.9790 | 0.0016 | 0.0031 | 0.0979 | 0.8990 | 0.1431 | 0.0191 | 2.0567 | 0.5284 | 69.4556 | 0.4499 | 0.0000 | 0.0000 | 0.8827 | 0.5105 | 1.0000 | 0.0000 | 0.0039 | 1.0000 |
| 1.0000 | 0.0000 | 0.0887 | 0.5541 | 0.3573 | 3.7061 | 0.1869 | 2.0213 | 0.5106 | 62.7294 | 0.4211 | 0.0000 | 0.0000 | 0.4238 | 0.0230 | 1.0000 | 0.0000 | 0.0009 | 0.9113 |
| 1.0000 | 0.0000 | 0.0000 | 0.0545 | 0.0273 | 0.1211 | 0.0212 | 1.0714 | 0.0357 | 71.9473 | 0.9172 | 0.0000 | 0.0000 | 0.0000 | 0.0010 | 0.0828 | 0.0000 | 0.0000 | 0.9727 |
| 0.9986 | 0.0007 | 0.0014 | 0.2308 | 0.7678 | 0.3159 | 0.0437 | 1.8281 | 0.4141 | 65.7285 | 0.0724 | 0.0000 | 0.0178 | 0.4837 | 0.2678 | 0.6619 | 0.0362 | 0.1065 | 0.6428 |
| 0.8484 | 0.0504 | 0.1275 | 0.1139 | 0.7587 | 1.1152 | 0.1013 | 1.6690 | 0.3345 | 64.2988 | 0.1139 | 0.0000 | 0.0000 | 0.4690 | 0.4055 | 0.7350 | 0.0126 | 0.1889 | 0.6579 |
| 0.9994 | 0.0000 | 0.0170 | 0.4266 | 0.5564 | 1.1520 | 0.1271 | 1.3741 | 0.1871 | 58.5380 | 0.0649 | 0.0000 | 0.0000 | 0.1607 | 0.1771 | 0.7473 | 0.0013 | 0.1430 | 0.8406 |
| 0.9454 | 0.0546 | 0.1115 | 0.3585 | 0.5290 | 0.9729 | 0.1145 | 1.7196 | 0.3598 | 70.6359 | 0.0339 | 0.0000 | 0.0000 | 0.4164 | 0.4984 | 0.8885 | 0.0295 | 0.3005 | 0.8557 |
| 0.9986 | 0.0007 | 0.0396 | 0.2180 | 0.7424 | 1.1921 | 0.1258 | 1.6475 | 0.3238 | 65.3447 | 0.0209 | 0.0000 | 0.0007 | 0.3993 | 0.4000 | 0.9266 | 0.0029 | 0.1799 | 0.7626 |
| 0.9766 | 0.0234 | 0.1581 | 0.1599 | 0.6595 | 1.0291 | 0.1186 | 1.4908 | 0.2454 | 53.7156 | 0.0916 | 0.0000 | 0.0009 | 0.4780 | 0.5193 | 0.9982 | 0.0027 | 0.2264 | 0.8598 |
| 0.9833 | 0.0161 | 0.1291 | 0.1933 | 0.6776 | 0.8153 | 0.0940 | 1.8216 | 0.4108 | 63.0736 | 0.2087 | 0.0000 | 0.0006 | 0.4836 | 0.5157 | 0.9981 | 0.0488 | 0.2415 | 0.7572 |
| 0.9960 | 0.0040 | 0.1541 | 0.1803 | 0.6657 | 0.5489 | 0.0726 | 1.7857 | 0.3929 | 77.1186 | 0.1541 | 0.0000 | 0.0020 | 0.2890 | 0.5096 | 0.8983 | 0.0534 | 0.2548 | 0.7694 |
| 0.9961 | 0.0019 | 0.0019 | 0.1710 | 0.8271 | 0.8708 | 0.1012 | 2.1966 | 0.5983 | 48.8950 | 0.0039 | 0.0000 | 0.0000 | 0.2705 | 0.1952 | 0.8773 | 0.0966 | 0.4116 | 0.8522 |
| 0.9745 | 0.0255 | 0.0511 | 0.3743 | 0.5747 | 0.9645 | 0.1149 | 1.9212 | 0.4606 | 84.3689 | 0.2485 | 0.0000 | 0.0000 | 0.4754 | 0.2515 | 0.9980 | 0.0246 | 0.2240 | 0.6277 |
| 0.9986 | 0.0000 | 0.0014 | 0.8135 | 0.1514 | 1.0749 | 0.1188 | 2.0831 | 0.5415 | 78.6851 | 0.0014 | 0.0000 | 0.0000 | 0.0797 | 0.0068 | 0.9311 | 0.0014 | 0.0068 | 0.8946 |
| 0.9990 | 0.0010 | 0.0010 | 0.8159 | 0.1831 | 0.7971 | 0.1070 | 2.2410 | 0.6205 | 88.5394 | 0.0264 | 0.0000 | 0.0000 | 0.0326 | 0.0020 | 0.9990 | 0.0010 | 0.0275 | 0.7935 |
| 0.9942 | 0.0058 | 0.5407 | 0.0000 | 0.4593 | 0.8030 | 0.0980 | 1.9940 | 0.4970 | 88.4832 | 0.0116 | 0.0000 | 0.0000 | 0.5349 | 0.2500 | 0.5349 | 0.2442 | 0.2267 | 0.5349 |
| 0.9955 | 0.0000 | 0.0580 | 0.0000 | 0.9420 | 1.5458 | 0.1553 | 1.9861 | 0.4931 | 85.9017 | 0.0089 | 0.0000 | 0.0000 | 0.9911 | 0.6518 | 0.9955 | 0.2946 | 0.8170 | 0.9955 |
| 1.0000 | 0.0000 | 0.3155 | 0.0000 | 0.6845 | 1.0201 | 0.1072 | 1.7024 | 0.3512 | 90.3574 | 0.0000 | 0.0000 | 0.0000 | 0.9951 | 0.4029 | 0.9951 | 0.5874 | 0.1990 | 0.9951 |
| 0.6947 | 0.0000 | 0.0229 | 0.0153 | 0.9542 | 1.8051 | 0.1236 | 2.0000 | 0.5000 | 69.6134 | 0.0153 | 0.0000 | 0.0000 | 0.6336 | 0.5267 | 0.9771 | 0.0687 | 0.0916 | 0.9695 |
| 0.9930 | 0.0000 | 0.1748 | 0.0070 | 0.8182 | 0.7663 | 0.0895 | 2.1439 | 0.5719 | 81.3748 | 0.0070 | 0.0000 | 0.0000 | 0.9790 | 0.6503 | 1.0000 | 0.3007 | 0.5245 | 1.0000 |
| 1.0000 | 0.0000 | 0.5594 | 0.0000 | 0.4406 | 0.4817 | 0.0705 | 1.9841 | 0.4921 | 49.8140 | 0.0000 | 0.0000 | 0.0000 | 1.0000 | 0.4336 | 1.0000 | 0.0070 | 0.4336 | 1.0000 |
| 1.0000 | 0.0000 | 0.5975 | 0.0000 | 0.4025 | 0.8066 | 0.0917 | 1.9835 | 0.4917 | 87.8636 | 0.0249 | 0.0000 | 0.0041 | 0.9959 | 0.3568 | 1.0000 | 0.1203 | 0.3776 | 0.9959 |
| 0.9962 | 0.0038 | 0.2654 | 0.0000 | 0.7346 | 0.9680 | 0.1032 | 1.8773 | 0.4386 | 89.5354 | 0.0115 | 0.0000 | 0.0000 | 0.9923 | 0.3577 | 0.9962 | 0.4731 | 0.5731 | 0.9962 |
| 1.0000 | 0.0000 | 0.4976 | 0.0000 | 0.5024 | 2.0189 | 0.1899 | 1.5192 | 0.2596 | 82.3126 | 0.0000 | 0.0000 | 0.0000 | 0.9952 | 0.7847 | 0.9952 | 0.2105 | 0.4928 | 0.9952 |
| 0.9919 | 0.0000 | 0.0325 | 0.0000 | 0.9675 | 0.5507 | 0.0421 | 1.9832 | 0.4916 | 49.7600 | 0.0163 | 0.0000 | 0.0081 | 0.4228 | 0.3902 | 1.0000 | 0.0081 | 0.5041 | 0.9756 |
| 0.9946 | 0.0054 | 0.0216 | 0.0108 | 0.9568 | 5.6481 | 0.1108 | 2.6818 | 0.8409 | 53.4229 | 0.0162 | 0.0000 | 0.0000 | 0.2054 | 0.8270 | 0.9946 | 0.0000 | 0.0162 | 0.9730 |
| 1.0000 | 0.0000 | 0.0000 | 0.0294 | 0.9706 | 0.1448 | 0.0242 | 2.0149 | 0.5075 | 69.1063 | 0.0735 | 0.0000 | 0.0000 | 0.0882 | 0.0147 | 1.0000 | 0.0000 | 0.0147 | 0.9853 |
| 1.0000 | 0.0000 | 0.0395 | 0.0000 | 0.9605 | 0.8050 | 0.0937 | 1.9912 | 0.4956 | 64.2339 | 0.0175 | 0.0000 | 0.0000 | 1.0000 | 0.5175 | 1.0000 | 0.4386 | 0.7412 | 1.0000 |
| 0.9885 | 0.0057 | 0.0230 | 0.1954 | 0.7816 | 0.7342 | 0.0899 | 1.9767 | 0.4884 | 67.4937 | 0.2356 | 0.0000 | 0.0057 | 0.7759 | 0.5000 | 1.0000 | 0.0402 | 0.5057 | 0.9655 |

| | | | | | | | | | | | | | | | | | | |
|---|---|---|---|---|---|---|---|---|---|---|---|---|---|---|---|---|---|---|
| 1.0000 | 0.0000 | 0.0050 | 0.1089 | 0.8861 | 0.7548 | 0.0977 | 1.9851 | 0.4925 | 71.7974 | 0.0050 | 0.0000 | 0.0000 | 0.8762 | 0.7178 | 1.0000 | 0.0347 | 0.7129 | 0.9950 |
| 1.0000 | 0.0000 | 0.0000 | 0.8092 | 0.1908 | 1.4136 | 0.1404 | 1.9733 | 0.4867 | 48.9320 | 0.0066 | 0.0000 | 0.0000 | 0.1776 | 0.0132 | 1.0000 | 0.0066 | 0.0066 | 0.4605 |
| 0.9960 | 0.0040 | 0.0723 | 0.0000 | 0.9277 | 1.2548 | 0.1297 | 1.9959 | 0.4980 | 75.7389 | 0.0201 | 0.0000 | 0.0000 | 0.9920 | 0.5462 | 0.9960 | 0.4177 | 0.7470 | 0.9960 |
| 0.9960 | 0.0040 | 0.4257 | 0.0000 | 0.5743 | 1.7310 | 0.1657 | 1.6048 | 0.3024 | 76.8300 | 0.0080 | 0.0000 | 0.0000 | 0.9960 | 0.7149 | 0.9960 | 0.2731 | 0.4739 | 0.9960 |
| 0.9771 | 0.0000 | 0.0687 | 0.0229 | 0.9008 | 0.7915 | 0.0815 | 1.9365 | 0.4683 | 54.0032 | 0.0611 | 0.0000 | 0.0000 | 0.8550 | 0.5954 | 0.9771 | 0.0382 | 0.4122 | 0.9695 |
| 0.9883 | 0.0058 | 0.0175 | 0.0000 | 0.9825 | 0.2380 | 0.0335 | 1.9766 | 0.4883 | 92.6090 | 0.4737 | 0.0000 | 0.0000 | 0.9708 | 0.4971 | 1.0000 | 0.0351 | 0.4854 | 0.9942 |
| 1.0000 | 0.0000 | 0.0000 | 0.0294 | 0.9706 | 0.7163 | 0.0989 | 1.8916 | 0.4458 | 79.1156 | 0.0059 | 0.0000 | 0.0000 | 0.9471 | 0.5882 | 1.0000 | 0.0235 | 0.5000 | 0.9941 |
| 0.8370 | 0.0000 | 0.0109 | 0.6630 | 0.3152 | 0.6483 | 0.0843 | 2.2333 | 0.6167 | 48.6667 | 0.0109 | 0.0000 | 0.0000 | 0.1413 | 0.1087 | 1.0000 | 0.0109 | 0.0217 | 0.9130 |
| 0.8936 | 0.0000 | 0.0106 | 0.2660 | 0.5638 | 4.9038 | 0.1252 | 1.8333 | 0.4167 | 54.3423 | 0.0000 | 0.0000 | 0.0000 | 0.1170 | 0.1170 | 0.9894 | 0.0000 | 0.0213 | 0.8404 |
| 1.0000 | 0.0000 | 0.0000 | 0.9592 | 0.0340 | 1.0588 | 0.1329 | 2.0204 | 0.5102 | 93.8353 | 0.0000 | 0.0000 | 0.0068 | 0.0340 | 0.0272 | 0.9932 | 0.0068 | 0.0272 | 0.9864 |
| 0.9939 | 0.0000 | 0.0122 | 0.5183 | 0.4695 | 0.9580 | 0.0886 | 2.4051 | 0.7025 | 57.4771 | 0.0549 | 0.0000 | 0.0061 | 0.0915 | 0.3902 | 0.9756 | 0.0061 | 0.0305 | 0.4878 |
| 0.9772 | 0.0000 | 0.0152 | 0.5551 | 0.3992 | 0.1367 | 0.0200 | 2.7778 | 0.8889 | 62.8851 | 0.0000 | 0.0000 | 0.0000 | 0.3992 | 0.3840 | 1.0000 | 0.0038 | 0.0038 | 0.6198 |
| 0.0348 | 0.0000 | 0.0000 | 0.1913 | 0.0217 | 1.4279 | 0.1460 | 1.1429 | 0.0714 | 83.1843 | 0.1739 | 0.0000 | 0.0000 | 0.0000 | 0.0000 | 0.6522 | 0.0174 | 0.0000 | 0.6522 |
| 0.8043 | 0.0000 | 0.0000 | 0.3188 | 0.6522 | 4.1705 | 0.2131 | 2.0319 | 0.5160 | 73.9214 | 0.0000 | 0.0000 | 0.0000 | 0.5072 | 0.0652 | 0.9928 | 0.0000 | 0.0072 | 0.5072 |
| 0.3981 | 0.0000 | 0.0000 | 0.9282 | 0.0625 | 2.5044 | 0.1653 | 2.6250 | 0.8125 | 38.3636 | 0.4167 | 0.0000 | 0.0000 | 0.0023 | 0.0579 | 1.0000 | 0.0000 | 0.0000 | 0.6759 |
| 0.9688 | 0.0000 | 0.0391 | 0.0000 | 0.9609 | 1.0412 | 0.0994 | 1.9922 | 0.4961 | 67.4516 | 0.0000 | 0.0000 | 0.0000 | 0.9453 | 0.9453 | 1.0000 | 0.0078 | 0.9453 | 0.9922 |
| 1.0000 | 0.0000 | 0.0000 | 0.0000 | 1.0000 | 0.8062 | 0.1046 | 1.5876 | 0.2938 | 61.9004 | 0.0052 | 0.0000 | 0.0000 | 1.0000 | 0.5619 | 1.0000 | 0.0206 | 0.1701 | 1.0000 |
| 0.9313 | 0.0000 | 0.0022 | 0.6253 | 0.1818 | 0.5844 | 0.0735 | 1.4033 | 0.2016 | 76.2227 | 0.0244 | 0.0000 | 0.0000 | 0.0288 | 0.0044 | 0.9956 | 0.0022 | 0.0067 | 0.8027 |
| 0.9929 | 0.0000 | 0.0036 | 0.4413 | 0.5018 | 0.2735 | 0.0378 | 2.1338 | 0.5669 | 50.3153 | 0.0000 | 0.0000 | 0.0000 | 0.1459 | 0.1423 | 1.0000 | 0.0142 | 0.0000 | 0.8577 |
| 0.0714 | 0.0000 | 0.0000 | 0.0357 | 0.0459 | 2.7608 | 0.1640 | 1.1000 | 0.0500 | 50.0000 | 0.0000 | 0.0000 | 0.0000 | 0.0000 | 0.0000 | 0.0816 | 0.0408 | 0.0000 | 0.0816 |
| 0.9118 | 0.0000 | 0.3922 | 0.0196 | 0.5882 | 0.5552 | 0.0781 | 1.2742 | 0.1371 | 61.7304 | 0.0294 | 0.0000 | 0.0000 | 0.5098 | 0.0980 | 1.0000 | 0.3922 | 0.0686 | 1.0000 |
| 0.9468 | 0.0000 | 0.1596 | 0.1809 | 0.6596 | 0.6351 | 0.0862 | 1.4085 | 0.2042 | 54.3630 | 0.0638 | 0.0000 | 0.0000 | 0.2979 | 0.1064 | 1.0000 | 0.4255 | 0.1170 | 1.0000 |
| 0.7089 | 0.0000 | 0.1899 | 0.0380 | 0.7722 | 0.7020 | 0.0870 | 1.8387 | 0.4194 | 82.8864 | 0.1139 | 0.0000 | 0.0000 | 0.3797 | 0.1646 | 1.0000 | 0.1013 | 0.2785 | 1.0000 |
| 0.5235 | 0.0000 | 0.0000 | 0.8588 | 0.0059 | 1.5991 | 0.1487 | 1.1250 | 0.0625 | 56.2263 | 0.5176 | 0.0000 | 0.0000 | 0.0000 | 0.0059 | 0.9529 | 0.0000 | 0.0000 | 0.6471 |
| 0.6703 | 0.0000 | 0.0000 | 0.8703 | 0.0054 | 1.5991 | 0.1487 | 1.1250 | 0.0625 | 56.8332 | 0.3676 | 0.0000 | 0.0000 | 0.0000 | 0.0054 | 0.9568 | 0.0000 | 0.0000 | 0.7838 |
| 0.7143 | 0.0000 | 0.0000 | 1.0000 | 0.0000 | 2.7448 | 0.2449 | NA | NA | 88.8082 | 0.2857 | 0.0000 | 0.0000 | 0.0000 | 0.0000 | 1.0000 | 0.0000 | 0.0000 | 0.8571 |
| 0.8205 | 0.0000 | 0.0000 | 0.7949 | 0.0769 | 1.2806 | 0.1442 | 1.9000 | 0.4500 | 58.8744 | 0.2564 | 0.0000 | 0.0000 | 0.0256 | 0.0000 | 0.8974 | 0.0256 | 0.0256 | 0.9744 |
| 0.5500 | 0.0000 | 0.0000 | 0.9000 | 0.0500 | 2.4465 | 0.2188 | 3.0000 | 1.0000 | 69.0270 | 0.5000 | 0.0000 | 0.0000 | 0.0500 | 0.0500 | 0.9500 | 0.0000 | 0.0000 | 0.9000 |
| 0.9778 | 0.0000 | 0.0000 | 0.4074 | 0.0926 | 0.5256 | 0.0669 | 2.1695 | 0.5847 | 79.5909 | 0.4667 | 0.0000 | 0.0000 | 0.0037 | 0.0926 | 0.5556 | 0.0000 | 0.0000 | 0.9704 |
| 0.9943 | 0.0000 | 0.0172 | 0.0057 | 0.9770 | 0.9961 | 0.0991 | 1.2093 | 0.1047 | 75.9567 | 0.0632 | 0.0000 | 0.0000 | 0.2931 | 0.2586 | 1.0000 | 0.0517 | 0.1437 | 0.9943 |
| 0.8507 | 0.0000 | 0.1269 | 0.0000 | 0.8731 | 0.7966 | 0.0986 | 1.9412 | 0.4706 | 74.8395 | 0.0000 | 0.0000 | 0.0000 | 0.8060 | 0.6716 | 1.0000 | 0.0373 | 0.6716 | 0.9925 |
| 1.0000 | 0.0000 | 0.0105 | 0.6754 | 0.2513 | 3.4607 | 0.1005 | 2.1935 | 0.5968 | 78.4588 | 0.0471 | 0.0000 | 0.0000 | 0.0838 | 0.1361 | 0.9738 | 0.0052 | 0.0157 | 0.9843 |
| 0.9908 | 0.0046 | 0.0922 | 0.0000 | 0.9078 | 0.9641 | 0.1056 | 2.0613 | 0.5307 | 81.9651 | 0.0046 | 0.0000 | 0.0000 | 0.9770 | 0.5438 | 0.9816 | 0.3871 | 0.8756 | 0.9770 |
| 1.0000 | 0.0000 | 0.4262 | 0.0000 | 0.5738 | 1.8124 | 0.1735 | 1.6864 | 0.3432 | 89.1189 | 0.0000 | 0.0000 | 0.0000 | 1.0000 | 0.7975 | 1.0000 | 0.1983 | 0.4768 | 1.0000 |
| 0.9796 | 0.0000 | 0.0408 | 0.0102 | 0.9490 | 0.3342 | 0.0517 | 2.5833 | 0.7917 | 77.6499 | 0.0306 | 0.0000 | 0.0000 | 0.9694 | 0.6531 | 1.0000 | 0.2143 | 0.8469 | 0.9898 |
| 0.9828 | 0.0000 | 0.0517 | 0.0172 | 0.9224 | 0.3889 | 0.0509 | 2.2143 | 0.6071 | 77.8053 | 0.1897 | 0.0000 | 0.0000 | 0.9741 | 0.3190 | 1.0000 | 0.3534 | 0.6466 | 0.9828 |
| 1.0000 | 0.0000 | 0.6489 | 0.0000 | 0.3511 | 1.8608 | 0.1741 | 1.3583 | 0.1791 | 66.9041 | 0.0000 | 0.0000 | 0.0000 | 0.9947 | 0.6649 | 0.9947 | 0.3191 | 0.1330 | 0.9947 |
| 1.0000 | 0.0000 | 0.0455 | 0.0000 | 0.9545 | 0.6175 | 0.0853 | 1.9651 | 0.4826 | 49.3333 | 0.0000 | 0.0000 | 0.0000 | 0.9943 | 0.7159 | 1.0000 | 0.2614 | 0.8011 | 1.0000 |
| 0.9853 | 0.0074 | 0.6324 | 0.0074 | 0.3603 | 0.3625 | 0.0527 | 1.8929 | 0.4464 | 74.1683 | 0.0147 | 0.0000 | 0.0000 | 0.9853 | 0.0441 | 0.9926 | 0.3456 | 0.1691 | 0.9926 |
| 0.9706 | 0.0000 | 0.0147 | 0.0441 | 0.9412 | 1.0633 | 0.0453 | 2.0000 | 0.5000 | 59.2787 | 0.0147 | 0.0000 | 0.0000 | 0.0441 | 0.0147 | 1.0000 | 0.0000 | 0.0000 | 1.0000 |
| 0.9098 | 0.0129 | 0.6031 | 0.0258 | 0.3711 | 0.8285 | 0.1023 | 1.7655 | 0.3827 | 84.3703 | 0.0773 | 0.0000 | 0.0000 | 0.6675 | 0.7062 | 0.9253 | 0.0000 | 0.0000 | 0.9124 |
| 0.9231 | 0.0000 | 0.4808 | 0.0000 | 0.5192 | 1.0068 | 0.1146 | 1.6731 | 0.3365 | 90.7688 | 0.0385 | 0.0000 | 0.0000 | 0.5192 | 0.6346 | 0.9808 | 0.0192 | 0.0000 | 0.9808 |
| 1.0000 | 0.0000 | 0.8132 | 0.0000 | 0.1868 | 0.8193 | 0.1052 | 1.8132 | 0.4066 | 89.0072 | 0.0440 | 0.0000 | 0.0000 | 0.8132 | 0.7692 | 0.9560 | 0.0000 | 0.0000 | 1.0000 |
| 0.6786 | 0.1429 | 0.5000 | 0.0179 | 0.4821 | 0.5677 | 0.0763 | 1.7857 | 0.3929 | 94.0920 | 0.0714 | 0.0000 | 0.0000 | 0.2857 | 0.5536 | 0.7857 | 0.0000 | 0.0000 | 0.7679 |
| 0.7500 | 0.0750 | 0.5250 | 0.0125 | 0.4625 | 0.5858 | 0.0809 | 1.4625 | 0.2313 | 84.1959 | 0.1500 | 0.0000 | 0.0000 | 0.4500 | 0.4875 | 0.7250 | 0.0000 | 0.0000 | 0.9125 |
| 0.5517 | 0.2759 | 0.2414 | 0.0000 | 0.7586 | 0.7837 | 0.0971 | 1.7931 | 0.3966 | 89.2195 | 0.1724 | 0.0000 | 0.0000 | 0.2759 | 0.3448 | 0.8621 | 0.0000 | 0.0000 | 0.7241 |
| 0.9783 | 0.0000 | 0.8261 | 0.0000 | 0.1739 | 0.5866 | 0.0820 | 1.7727 | 0.3864 | 83.5482 | 0.0870 | 0.0000 | 0.0000 | 0.6522 | 0.6739 | 0.8261 | 0.0000 | 0.0000 | 0.9565 |
| 0.6703 | 0.0000 | 0.4835 | 0.0000 | 0.5165 | 0.7281 | 0.0909 | 1.4505 | 0.2253 | 82.8376 | 0.0440 | 0.0000 | 0.0000 | 0.4945 | 0.7802 | 0.9560 | 0.0110 | 0.0000 | 0.9890 |
| 0.7966 | 0.0000 | 0.5085 | 0.0678 | 0.4237 | 0.4862 | 0.0664 | 1.5614 | 0.2807 | 86.7431 | 0.1017 | 0.0000 | 0.0000 | 0.3729 | 0.6780 | 0.8644 | 0.0000 | 0.0000 | 0.8644 |
| 0.4943 | 0.2759 | 0.2989 | 0.0115 | 0.6897 | 0.8215 | 0.1000 | 1.7529 | 0.3765 | 83.7545 | 0.0460 | 0.0000 | 0.0000 | 0.1379 | 0.3793 | 0.8161 | 0.0000 | 0.0000 | 0.7126 |
| 0.5349 | 0.4186 | 0.3023 | 0.0698 | 0.6279 | 1.0336 | 0.1230 | 2.2558 | 0.6279 | 83.6111 | 0.0000 | 0.0000 | 0.0000 | 0.2791 | 0.4651 | 0.9767 | 0.0000 | 0.0000 | 0.5116 |

| | | | | | | | | | | | | | | | | | | |
|---|---|---|---|---|---|---|---|---|---|---|---|---|---|---|---|---|---|---|
| 0.7377 | 0.0000 | 0.3279 | 0.0656 | 0.6066 | 0.3591 | 0.0552 | 1.3220 | 0.1610 | 85.1710 | 0.2623 | 0.0000 | 0.0000 | 0.4426 | 0.5738 | 0.8689 | 0.0164 | 0.0000 | 0.9180 |
| 0.8267 | 0.0000 | 0.6533 | 0.0133 | 0.3200 | 0.7050 | 0.0893 | 1.6571 | 0.3286 | 87.0260 | 0.0267 | 0.0000 | 0.0000 | 0.6400 | 0.7867 | 0.9200 | 0.0267 | 0.0000 | 0.9467 |
| 0.9057 | 0.0000 | 0.5943 | 0.0472 | 0.3491 | 0.7632 | 0.0920 | 1.6970 | 0.3485 | 85.7598 | 0.0189 | 0.0000 | 0.0000 | 0.6321 | 0.7453 | 0.9057 | 0.0755 | 0.0000 | 0.8774 |
| 0.4918 | 0.1639 | 0.3852 | 0.0000 | 0.6066 | 0.6560 | 0.0827 | 1.7647 | 0.3824 | 85.1141 | 0.0000 | 0.0000 | 0.0082 | 0.2459 | 0.6066 | 0.9836 | 0.0164 | 0.0000 | 0.8033 |
| 1.0000 | 0.0000 | 0.8261 | 0.0000 | 0.1739 | 0.6094 | 0.0856 | 2.0435 | 0.5217 | 85.9641 | 0.0000 | 0.0000 | 0.0000 | 0.8116 | 0.8696 | 0.9855 | 0.0000 | 0.0000 | 1.0000 |
| 1.0000 | 0.0000 | 0.7500 | 0.0000 | 0.2500 | 0.5213 | 0.0742 | 2.0000 | 0.5000 | 81.8629 | 0.0000 | 0.0000 | 0.0000 | 0.6667 | 0.7500 | 1.0000 | 0.0000 | 0.0000 | 1.0000 |
| 1.0000 | 0.0000 | 0.8632 | 0.0000 | 0.1368 | 0.6190 | 0.0871 | 2.0947 | 0.5474 | 83.6980 | 0.0105 | 0.0000 | 0.0000 | 0.8737 | 0.9684 | 1.0000 | 0.0211 | 0.0000 | 0.9579 |
| 1.0000 | 0.0000 | 0.8750 | 0.0000 | 0.1250 | 0.8828 | 0.1013 | 2.0000 | 0.5000 | 83.5705 | 0.0208 | 0.0000 | 0.0000 | 0.8542 | 0.9167 | 1.0000 | 0.0000 | 0.0000 | 1.0000 |
| 0.7316 | 0.0087 | 0.4372 | 0.0000 | 0.5628 | 0.8061 | 0.0970 | 1.5739 | 0.2870 | 87.6435 | 0.0519 | 0.0000 | 0.0000 | 0.4848 | 0.7446 | 1.0000 | 0.0000 | 0.0000 | 0.9913 |
| 0.8267 | 0.1600 | 0.4533 | 0.0000 | 0.5467 | 0.9552 | 0.1136 | 1.9863 | 0.4932 | 88.6149 | 0.1067 | 0.0000 | 0.0000 | 0.5333 | 0.5467 | 1.0000 | 0.0000 | 0.0000 | 0.8400 |
| 0.6129 | 0.2581 | 0.4301 | 0.0000 | 0.5699 | 0.9355 | 0.1146 | 1.9551 | 0.4775 | 87.8634 | 0.1290 | 0.0000 | 0.0430 | 0.5269 | 0.4839 | 0.9570 | 0.0108 | 0.0000 | 0.6882 |
| 0.5341 | 0.0000 | 0.3864 | 0.0227 | 0.5909 | 0.6023 | 0.0781 | 1.3678 | 0.1839 | 92.4335 | 0.0455 | 0.0000 | 0.0114 | 0.4091 | 0.8409 | 0.9773 | 0.0000 | 0.0000 | 0.9659 |
| 0.6271 | 0.0000 | 0.2881 | 0.0000 | 0.6949 | 0.9310 | 0.1034 | 1.3684 | 0.1842 | 87.0080 | 0.0678 | 0.0000 | 0.0169 | 0.2881 | 0.6271 | 0.9661 | 0.0000 | 0.0000 | 0.8983 |
| 0.7586 | 0.0000 | 0.2931 | 0.0172 | 0.5862 | 0.9988 | 0.1067 | 1.5208 | 0.2604 | 88.4517 | 0.0172 | 0.0000 | 0.0000 | 0.2414 | 0.4828 | 0.8103 | 0.0172 | 0.0000 | 0.8621 |
| 0.4886 | 0.3636 | 0.4205 | 0.0568 | 0.5114 | 0.7818 | 0.0968 | 2.1928 | 0.5964 | 84.5886 | 0.0227 | 0.0000 | 0.0000 | 0.4205 | 0.5000 | 0.7955 | 0.0114 | 0.0000 | 0.6023 |
| 0.7797 | 0.0000 | 0.4746 | 0.0000 | 0.5085 | 1.0773 | 0.1221 | 1.4444 | 0.2222 | 83.5316 | 0.1017 | 0.0000 | 0.0678 | 0.5085 | 0.5424 | 0.9153 | 0.0000 | 0.0000 | 0.9153 |
| 0.6981 | 0.0000 | 0.3774 | 0.0755 | 0.5472 | 0.5460 | 0.0729 | 1.2642 | 0.1321 | 84.0753 | 0.1509 | 0.0000 | 0.0000 | 0.3774 | 0.6226 | 0.8491 | 0.0000 | 0.0000 | 0.9245 |
| 0.7451 | 0.1569 | 0.6863 | 0.0000 | 0.3137 | 0.7241 | 0.0937 | 1.9565 | 0.4783 | 83.8631 | 0.0392 | 0.0000 | 0.0196 | 0.5882 | 0.6275 | 0.8627 | 0.0000 | 0.0000 | 0.8235 |
| 0.8611 | 0.0000 | 0.8056 | 0.0000 | 0.1667 | 0.6311 | 0.0895 | 1.8000 | 0.4000 | 83.5736 | 0.0556 | 0.0000 | 0.0000 | 0.8333 | 0.8889 | 0.9444 | 0.0000 | 0.0000 | 0.9722 |
| 0.9667 | 0.0333 | 0.8417 | 0.0000 | 0.1583 | 0.7083 | 0.0972 | 2.0500 | 0.5250 | 82.3959 | 0.0333 | 0.0000 | 0.0000 | 0.8833 | 0.9167 | 0.9917 | 0.0000 | 0.0000 | 0.9667 |
| 0.7308 | 0.2692 | 0.4615 | 0.0000 | 0.5385 | 0.7662 | 0.1001 | 2.1923 | 0.5962 | 88.8973 | 0.1538 | 0.0000 | 0.0000 | 0.6154 | 0.5385 | 1.0000 | 0.0000 | 0.0000 | 0.7308 |
| 1.0000 | 0.0000 | 0.8219 | 0.0000 | 0.1781 | 0.6799 | 0.0930 | 2.0411 | 0.5205 | 90.8621 | 0.0411 | 0.0000 | 0.0000 | 0.8630 | 0.9315 | 1.0000 | 0.0000 | 0.0000 | 1.0000 |
| 1.0000 | 0.0000 | 0.8723 | 0.0000 | 0.1277 | 0.6631 | 0.0937 | 1.8511 | 0.4255 | 87.6496 | 0.1277 | 0.0000 | 0.0000 | 0.9787 | 0.8511 | 0.9894 | 0.0000 | 0.0000 | 1.0000 |
| 0.8889 | 0.1111 | 0.6296 | 0.0000 | 0.3704 | 0.6264 | 0.0859 | 2.0370 | 0.5185 | 84.8206 | 0.1111 | 0.0000 | 0.0000 | 0.6667 | 0.6667 | 1.0000 | 0.0000 | 0.0000 | 0.8889 |
| 1.0000 | 0.0000 | 0.6757 | 0.0000 | 0.3243 | 0.7573 | 0.0960 | 2.0000 | 0.5000 | 88.4804 | 0.0541 | 0.0000 | 0.0000 | 0.7162 | 0.8243 | 0.9865 | 0.0000 | 0.0000 | 1.0000 |
| 0.8246 | 0.0000 | 0.6140 | 0.0175 | 0.3684 | 0.8554 | 0.1053 | 1.6111 | 0.3056 | 86.1176 | 0.0702 | 0.0000 | 0.0175 | 0.6491 | 0.7193 | 0.9825 | 0.0000 | 0.0000 | 0.9825 |
| 0.7838 | 0.0000 | 0.6757 | 0.0270 | 0.2973 | 0.4831 | 0.0610 | 1.6471 | 0.3235 | 84.6819 | 0.0541 | 0.0000 | 0.0541 | 0.6486 | 0.7838 | 0.6216 | 0.0000 | 0.0000 | 0.9189 |
| 0.9130 | 0.0000 | 0.6304 | 0.0217 | 0.3478 | 0.5543 | 0.0726 | 1.4000 | 0.2000 | 83.4350 | 0.1739 | 0.0000 | 0.0000 | 0.4348 | 0.3696 | 0.9783 | 0.0000 | 0.0000 | 0.9783 |
| 0.8764 | 0.0449 | 0.7640 | 0.0562 | 0.1685 | 0.8034 | 0.1056 | 1.8372 | 0.4186 | 88.3290 | 0.0674 | 0.0000 | 0.0112 | 0.7865 | 0.7416 | 0.9551 | 0.0000 | 0.0000 | 0.9213 |
| 0.7477 | 0.2523 | 0.7207 | 0.0000 | 0.2793 | 0.9246 | 0.1189 | 2.1532 | 0.5766 | 84.8848 | 0.0090 | 0.0000 | 0.0000 | 0.6577 | 0.6486 | 0.9279 | 0.0000 | 0.0000 | 0.7477 |
| 0.1988 | 0.7018 | 0.1579 | 0.0117 | 0.8304 | 1.1123 | 0.1330 | 2.5714 | 0.7857 | 92.2332 | 0.0234 | 0.0000 | 0.0058 | 0.1637 | 0.2456 | 0.9708 | 0.0000 | 0.0000 | 0.2807 |
| 0.9684 | 0.0000 | 0.7263 | 0.0211 | 0.2526 | 0.6019 | 0.0840 | 1.7111 | 0.3556 | 87.8188 | 0.2105 | 0.0000 | 0.0105 | 0.8842 | 0.7158 | 0.9474 | 0.0000 | 0.0000 | 0.9684 |
| 0.7273 | 0.0000 | 0.4646 | 0.0303 | 0.5051 | 0.3480 | 0.0501 | 1.5745 | 0.2872 | 84.5274 | 0.2424 | 0.0000 | 0.0101 | 0.4444 | 0.6364 | 0.9293 | 0.0000 | 0.0000 | 0.7980 |
| 0.3972 | 0.5674 | 0.3404 | 0.0000 | 0.6596 | 0.9575 | 0.1160 | 2.4783 | 0.7391 | 83.7295 | 0.0567 | 0.0000 | 0.0071 | 0.3688 | 0.3404 | 0.8511 | 0.0000 | 0.0000 | 0.4255 |
| 0.8817 | 0.0860 | 0.5376 | 0.0000 | 0.4624 | 0.6569 | 0.0890 | 1.8791 | 0.4396 | 84.4871 | 0.2581 | 0.0000 | 0.0215 | 0.7742 | 0.6129 | 0.9785 | 0.0000 | 0.0000 | 0.8925 |
| 0.7966 | 0.0000 | 0.8136 | 0.0000 | 0.1864 | 0.6947 | 0.0945 | 1.8000 | 0.4000 | 87.1391 | 0.0169 | 0.0000 | 0.0678 | 0.7627 | 0.8814 | 0.9322 | 0.0000 | 0.0000 | 0.9322 |
| 0.9630 | 0.0000 | 0.7037 | 0.0000 | 0.2963 | 0.5398 | 0.0768 | 2.1923 | 0.5962 | 86.2284 | 0.0370 | 0.0000 | 0.0370 | 0.7037 | 0.8889 | 0.9630 | 0.0000 | 0.0000 | 0.9630 |
| 0.6444 | 0.0000 | 0.2667 | 0.0000 | 0.7333 | 0.4669 | 0.0599 | 1.8000 | 0.4000 | 84.8411 | 0.0222 | 0.0000 | 0.0000 | 0.2000 | 0.8444 | 0.9778 | 0.0000 | 0.0000 | 0.9556 |
| 0.3913 | 0.0000 | 0.2609 | 0.0000 | 0.7391 | 0.3305 | 0.0466 | 1.4000 | 0.2000 | 83.7312 | 0.0000 | 0.0000 | 0.0000 | 0.0870 | 0.8261 | 1.0000 | 0.0000 | 0.0000 | 0.9565 |
| 0.5833 | 0.0000 | 0.5208 | 0.0000 | 0.4792 | 0.5380 | 0.0735 | 1.6047 | 0.3023 | 81.9460 | 0.0417 | 0.0000 | 0.0833 | 0.4583 | 0.8125 | 0.9167 | 0.0000 | 0.0000 | 0.9167 |
| 0.8776 | 0.0000 | 0.8980 | 0.0000 | 0.1020 | 0.5979 | 0.0837 | 1.4348 | 0.2174 | 86.6663 | 0.0204 | 0.0000 | 0.0408 | 0.4286 | 0.4898 | 0.5306 | 0.0000 | 0.0000 | 0.9592 |
| 0.9231 | 0.0000 | 0.8846 | 0.0192 | 0.0577 | 0.5427 | 0.0735 | 1.4490 | 0.2245 | 84.7887 | 0.0000 | 0.0000 | 0.0000 | 0.4231 | 0.4423 | 0.4423 | 0.0000 | 0.0000 | 0.9615 |
| 0.6986 | 0.0000 | 0.6986 | 0.0000 | 0.3014 | 0.5872 | 0.0814 | 1.6567 | 0.3284 | 86.9645 | 0.0000 | 0.0000 | 0.0274 | 0.6027 | 0.8767 | 0.9589 | 0.0000 | 0.0000 | 0.9726 |
| 0.7097 | 0.0000 | 0.5161 | 0.0000 | 0.4839 | 0.5472 | 0.0763 | 1.4828 | 0.2414 | 91.3474 | 0.1935 | 0.0000 | 0.0323 | 0.6452 | 0.7097 | 0.9677 | 0.0000 | 0.0000 | 0.9677 |
| 0.5570 | 0.3038 | 0.5190 | 0.0000 | 0.4684 | 0.8574 | 0.1099 | 2.1429 | 0.5714 | 92.3300 | 0.0253 | 0.0000 | 0.0000 | 0.5316 | 0.6329 | 0.9873 | 0.0000 | 0.0000 | 0.6835 |
| 0.3409 | 0.0000 | 0.2045 | 0.0000 | 0.7955 | 0.4123 | 0.0554 | 1.1463 | 0.0732 | 86.7933 | 0.0909 | 0.0000 | 0.0227 | 0.2273 | 0.7727 | 0.9318 | 0.0000 | 0.0000 | 0.9773 |
| 0.6304 | 0.0000 | 0.5000 | 0.0000 | 0.5000 | 0.5028 | 0.0713 | 1.4651 | 0.2326 | 89.5842 | 0.1304 | 0.0000 | 0.0217 | 0.5652 | 0.7826 | 0.9783 | 0.0000 | 0.0000 | 0.9783 |
| 0.6000 | 0.0000 | 0.5429 | 0.0286 | 0.4286 | 0.5510 | 0.0735 | 1.4333 | 0.2167 | 85.6500 | 0.0286 | 0.0000 | 0.0286 | 0.4000 | 0.7143 | 0.9143 | 0.0000 | 0.0000 | 0.9714 |
| 0.5429 | 0.0000 | 0.4000 | 0.0286 | 0.5714 | 0.6419 | 0.0803 | 1.4375 | 0.2188 | 88.8044 | 0.0000 | 0.0000 | 0.0286 | 0.2857 | 0.7429 | 0.9429 | 0.0000 | 0.0000 | 0.9714 |
| 1.0000 | 0.0000 | 0.7500 | 0.0000 | 0.2500 | 0.6269 | 0.0893 | 1.7500 | 0.3750 | 87.6986 | 0.2500 | 0.0000 | 0.0000 | 1.0000 | 0.7500 | 1.0000 | 0.0000 | 0.0000 | 1.0000 |
| 1.0000 | 0.0000 | 0.3046 | 0.0000 | 0.6954 | 0.3000 | 0.0444 | 2.1168 | 0.5584 | 84.8042 | 0.0761 | 0.0000 | 0.0000 | 0.3909 | 0.5178 | 1.0000 | 0.0000 | 0.0000 | 1.0000 |

| | | | | | | | | | | | | | | | | | | |
|---|---|---|---|---|---|---|---|---|---|---|---|---|---|---|---|---|---|---|
| 1.0000 | 0.0000 | 0.9589 | 0.0000 | 0.0411 | 0.6971 | 0.0977 | 1.9726 | 0.4863 | 83.8831 | 0.0274 | 0.0000 | 0.0000 | 0.9863 | 0.9589 | 1.0000 | 0.0000 | 0.0000 | 1.0000 |
| 1.0000 | 0.0000 | 0.9434 | 0.0000 | 0.0566 | 0.6910 | 0.0970 | 1.9623 | 0.4811 | 85.5515 | 0.0404 | 0.0000 | 0.0000 | 0.9838 | 0.9461 | 1.0000 | 0.0000 | 0.0000 | 1.0000 |
| 0.9259 | 0.0673 | 0.7609 | 0.0000 | 0.2391 | 0.7614 | 0.1028 | 1.9459 | 0.4730 | 86.3333 | 0.1178 | 0.0000 | 0.0034 | 0.9259 | 0.8114 | 0.9966 | 0.0000 | 0.0000 | 0.9293 |
| 1.0000 | 0.0000 | 0.7500 | 0.0000 | 0.2500 | 0.6269 | 0.0893 | 1.7500 | 0.3750 | 83.7377 | 0.2500 | 0.0000 | 0.0000 | 1.0000 | 0.7500 | 1.0000 | 0.0000 | 0.0000 | 1.0000 |
| 1.0000 | 0.0000 | 0.9259 | 0.0000 | 0.0741 | 0.6894 | 0.0969 | 1.9259 | 0.4630 | 71.2730 | 0.0741 | 0.0000 | 0.0000 | 1.0000 | 0.9259 | 1.0000 | 0.0000 | 0.0000 | 1.0000 |
| 1.0000 | 0.0000 | 1.0000 | 0.0000 | 0.0000 | 0.5223 | 0.0760 | 1.5556 | 0.2778 | 82.6569 | 0.0000 | 0.0000 | 0.0000 | 0.5556 | 0.5556 | 1.0000 | 0.0000 | 0.0000 | 1.0000 |
| 1.0000 | 0.0000 | 0.9146 | 0.0000 | 0.0854 | 0.7042 | 0.0976 | 2.0366 | 0.5183 | 84.9847 | 0.0122 | 0.0000 | 0.0000 | 0.9512 | 0.9878 | 1.0000 | 0.0000 | 0.0000 | 1.0000 |
| 1.0000 | 0.0000 | 0.9643 | 0.0000 | 0.0357 | 0.6841 | 0.0963 | 1.9643 | 0.4821 | 81.8842 | 0.0357 | 0.0000 | 0.0000 | 0.9286 | 0.9643 | 0.9286 | 0.0000 | 0.0000 | 1.0000 |
| 1.0000 | 0.0000 | 0.4737 | 0.0000 | 0.5263 | 0.4022 | 0.0620 | 1.5921 | 0.2961 | 80.1430 | 0.4605 | 0.0000 | 0.0000 | 0.5263 | 0.5263 | 0.6053 | 0.0000 | 0.0000 | 1.0000 |
| 1.0000 | 0.0000 | 0.8007 | 0.0000 | 0.1993 | 0.5933 | 0.0851 | 1.6667 | 0.3333 | 83.7377 | 0.1661 | 0.0000 | 0.0000 | 0.8306 | 0.6645 | 1.0000 | 0.0000 | 0.0000 | 0.9668 |
| 1.0000 | 0.0000 | 0.9302 | 0.0000 | 0.0698 | 0.6910 | 0.0971 | 1.9302 | 0.4651 | 83.6321 | 0.0698 | 0.0000 | 0.0000 | 1.0000 | 0.9302 | 1.0000 | 0.0000 | 0.0000 | 1.0000 |
| 1.0000 | 0.0000 | 0.7914 | 0.0024 | 0.2062 | 0.6341 | 0.0893 | 1.9976 | 0.4988 | 82.7690 | 0.0600 | 0.0000 | 0.0000 | 0.8897 | 0.8801 | 0.9880 | 0.0000 | 0.0000 | 0.9976 |
| 0.9974 | 0.0000 | 0.7885 | 0.0000 | 0.2115 | 0.6550 | 0.0914 | 1.9686 | 0.4843 | 74.7581 | 0.1044 | 0.0000 | 0.0026 | 0.9164 | 0.8877 | 0.9948 | 0.0000 | 0.0000 | 0.9974 |
| 1.0000 | 0.0000 | 0.5435 | 0.0000 | 0.4565 | 1.5385 | 0.1090 | 2.1739 | 0.5870 | 62.6248 | 0.0435 | 0.0000 | 0.0000 | 0.5435 | 0.8261 | 1.0000 | 0.0000 | 0.0652 | 1.0000 |
| 1.0000 | 0.0000 | 0.7879 | 0.0000 | 0.2121 | 5.2801 | 0.2588 | 1.5455 | 0.2727 | 85.3726 | 0.0000 | 0.0000 | 0.0000 | 0.3030 | 0.4242 | 1.0000 | 0.0000 | 0.0000 | 0.9091 |
| 0.7807 | 0.0000 | 0.4678 | 0.0058 | 0.5263 | 0.3402 | 0.0501 | 1.4348 | 0.2174 | 85.9808 | 0.2339 | 0.0000 | 0.0292 | 0.4971 | 0.4591 | 0.8099 | 0.0000 | 0.0000 | 0.9649 |
| 0.9242 | 0.0253 | 0.8333 | 0.0000 | 0.1667 | 0.6692 | 0.0929 | 1.9973 | 0.4987 | 84.0616 | 0.0379 | 0.0000 | 0.0253 | 0.8586 | 0.8586 | 0.9495 | 0.0000 | 0.0000 | 0.9495 |
| 0.9971 | 0.0000 | 0.9267 | 0.0000 | 0.0733 | 0.6678 | 0.0941 | 1.8824 | 0.4412 | 67.9058 | 0.0733 | 0.0000 | 0.0029 | 0.9531 | 0.8798 | 0.9531 | 0.0000 | 0.0000 | 0.9971 |
| 0.9091 | 0.0000 | 0.1818 | 0.0000 | 0.7273 | 4.3832 | 0.2931 | 1.2000 | 0.1000 | 82.3172 | 0.0000 | 0.0000 | 0.0000 | 0.1818 | 0.1818 | 0.9091 | 0.0000 | 0.0000 | 0.9091 |
| 0.7500 | 0.0000 | 0.6250 | 0.0000 | 0.1250 | 1.4798 | 0.1403 | 1.8333 | 0.4167 | 45.5500 | 0.0000 | 0.0000 | 0.0000 | 0.6250 | 0.6250 | 0.7500 | 0.0000 | 0.0000 | 0.7500 |
| 1.0000 | 0.0000 | 1.0000 | 0.0000 | 0.0000 | 0.6287 | 0.0893 | 1.8000 | 0.4000 | 83.1355 | 0.0000 | 0.0000 | 0.0000 | 0.8000 | 0.8000 | 1.0000 | 0.0000 | 0.0000 | 1.0000 |
| 1.0000 | 0.0000 | 0.0741 | 0.2963 | 0.6296 | 0.6443 | 0.0719 | 2.4815 | 0.7407 | 83.3900 | 0.0000 | 0.0000 | 0.0000 | 0.0000 | 0.5926 | 1.0000 | 0.0000 | 0.0000 | 0.7037 |
| 0.9775 | 0.0000 | 0.9326 | 0.0000 | 0.0449 | 0.7055 | 0.0989 | 1.9425 | 0.4713 | 79.1036 | 0.0000 | 0.0000 | 0.0000 | 0.9213 | 0.9213 | 0.9775 | 0.0000 | 0.0000 | 0.9326 |
| 0.8333 | 0.0000 | 0.0000 | 0.0000 | 0.8333 | 0.0250 | 0.0044 | 2.2000 | 0.6000 NA | | 0.0000 | 0.0000 | 0.0000 | 0.0000 | 0.8333 | 0.1667 | 0.0000 | 0.0000 | 0.1667 |
| 1.0000 | 0.0000 | 0.8571 | 0.0000 | 0.1429 | 0.6308 | 0.0888 | 2.1429 | 0.5714 | 82.9339 | 0.0000 | 0.0000 | 0.0000 | 0.8571 | 1.0000 | 1.0000 | 0.0000 | 0.0000 | 1.0000 |
| 0.5000 | 0.0000 | 0.0000 | 0.0000 | 0.5000 | 5.3000 | 0.3413 | 1.0000 | 0.0000 | 84.2788 | 0.0000 | 0.0000 | 0.0000 | 0.0000 | 0.0000 | 0.5000 | 0.0000 | 0.0000 | 0.5000 |
| 1.0000 | 0.0000 | 0.9919 | 0.0000 | 0.0081 | 0.7058 | 0.0989 | 1.9756 | 0.4878 | 83.3900 | 0.0081 | 0.0000 | 0.0000 | 0.9837 | 0.9756 | 1.0000 | 0.0000 | 0.0000 | 1.0000 |
| 0.9901 | 0.0000 | 0.9901 | 0.0000 | 0.0000 | 0.7158 | 0.1001 | 2.0000 | 0.5000 | 85.0689 | 0.0000 | 0.0000 | 0.0000 | 0.9901 | 0.9901 | 0.9901 | 0.0000 | 0.0000 | 0.9901 |
| 1.0000 | 0.0000 | 1.0000 | 0.0000 | 0.0000 | 0.7158 | 0.1001 | 2.0000 | 0.5000 | 44.7700 | 0.0000 | 0.0000 | 0.0000 | 1.0000 | 1.0000 | 1.0000 | 0.0000 | 0.0000 | 1.0000 |
| 1.0000 | 0.0000 | 0.6923 | 0.0769 | 0.2308 | 0.6428 | 0.0863 | 1.8462 | 0.4231 | 67.8098 | 0.0385 | 0.0000 | 0.0000 | 0.6923 | 0.6538 | 0.9231 | 0.0000 | 0.0000 | 0.9231 |
| 1.0000 | 0.0000 | 1.0000 | 0.0000 | 0.0000 | 0.2805 | 0.0458 | 1.0000 | 0.0000 | 81.5040 | 0.0000 | 0.0000 | 0.0000 | 0.0000 | 0.0000 | 1.0000 | 0.0000 | 0.0000 | 1.0000 |
| 0.9000 | 0.0000 | 0.9000 | 0.0000 | 0.0000 | 0.5223 | 0.0760 | 1.5556 | 0.2778 | 100.0000 | 0.0000 | 0.0000 | 0.0000 | 0.5000 | 0.5000 | 0.9000 | 0.0000 | 0.0000 | 0.9000 |
| 0.9667 | 0.0000 | 0.6667 | 0.0000 | 0.3000 | 0.5334 | 0.0742 | 2.2963 | 0.6481 | 84.4511 | 0.0000 | 0.0000 | 0.0000 | 0.6333 | 0.9667 | 0.9000 | 0.0000 | 0.0000 | 0.9000 |
| 1.0000 | 0.0000 | 0.0400 | 0.0000 | 0.9600 | 0.0712 | 0.0125 | 1.9787 | 0.4894 | 83.4260 | 0.0000 | 0.0000 | 0.0000 | 0.0000 | 0.0600 | 0.9200 | 0.0000 | 0.0000 | 0.9400 |
| 1.0000 | 0.0000 | 0.9863 | 0.0000 | 0.0137 | 0.7069 | 0.0989 | 2.0000 | 0.5000 | 83.3900 | 0.0000 | 0.0000 | 0.0000 | 0.9863 | 0.9863 | 1.0000 | 0.0000 | 0.0000 | 1.0000 |
| 1.0000 | 0.0000 | 0.9938 | 0.0000 | 0.0062 | 0.7136 | 0.0998 | 1.9938 | 0.4969 | 48.9540 | 0.0062 | 0.0000 | 0.0000 | 1.0000 | 0.9938 | 1.0000 | 0.0000 | 0.0000 | 1.0000 |
| 1.0000 | 0.0000 | 1.0000 | 0.0000 | 0.0000 | 0.7158 | 0.1001 | 2.0000 | 0.5000 | 70.5167 | 0.0000 | 0.0000 | 0.0000 | 1.0000 | 1.0000 | 1.0000 | 0.0000 | 0.0000 | 1.0000 |
| 1.0000 | 0.0000 | 0.6250 | 0.0000 | 0.3750 | 5.3018 | 0.2494 | 1.3750 | 0.1875 | 68.9075 | 0.0000 | 0.0000 | 0.0000 | 0.3750 | 0.0000 | 0.6250 | 0.0000 | 0.0000 | 1.0000 |
| 1.0000 | 0.0000 | 1.0000 | 0.0000 | 0.0000 | 0.6287 | 0.0893 | 1.8000 | 0.4000 | 64.2647 | 0.0000 | 0.0000 | 0.0000 | 0.8000 | 0.8000 | 1.0000 | 0.0000 | 0.0000 | 1.0000 |
| 1.0000 | 0.0000 | 1.0000 | 0.0000 | 0.0000 | 10.3000 | 0.4497 | 1.0000 | 0.0000 | 44.7700 | 0.0000 | 0.0000 | 0.0000 | 0.0000 | 0.0000 | 1.0000 | 0.0000 | 0.0000 | 1.0000 |
| 1.0000 | 0.0000 | 1.0000 | 0.0000 | 0.0000 | 0.2805 | 0.0458 | 1.0000 | 0.0000 | 44.7700 | 0.0000 | 0.0000 | 0.0000 | 0.0000 | 0.0000 | 1.0000 | 0.0000 | 0.0000 | 1.0000 |
| 1.0000 | 0.0000 | 0.2857 | 0.0000 | 0.7143 | 0.1873 | 0.0308 | 2.4762 | 0.7381 | 73.9600 | 0.0000 | 0.0000 | 0.0000 | 0.0476 | 0.7619 | 1.0000 | 0.0000 | 0.0000 | 1.0000 |
| 1.0000 | 0.0000 | 1.0000 | 0.0000 | 0.0000 | 0.2805 | 0.0458 | 1.0000 | 0.0000 | 75.1286 | 0.0000 | 0.0000 | 0.0000 | 0.0000 | 0.0000 | 1.0000 | 0.0000 | 0.0000 | 1.0000 |
| 0.9375 | 0.0000 | 0.0000 | 0.0000 | 0.9375 | 0.1210 | 0.0212 | 3.0000 | 1.0000 | 83.3900 | 0.0000 | 0.0000 | 0.0000 | 0.0000 | 0.9375 | 0.9375 | 0.0000 | 0.0000 | 0.9375 |
| 1.0000 | 0.0000 | 0.0000 | 0.0000 | 1.0000 | 0.5564 | 0.0621 | 2.5385 | 0.7692 | 90.9522 | 0.0000 | 0.0000 | 0.0000 | 0.0000 | 0.9615 | 0.8077 | 0.0000 | 0.0000 | 0.9615 |
| 1.0000 | 0.0000 | 0.0000 | 0.0000 | 1.0000 | 0.1210 | 0.0212 | 3.0000 | 1.0000 | 76.0355 | 0.0000 | 0.0000 | 0.0000 | 0.0000 | 1.0000 | 1.0000 | 0.0000 | 0.0000 | 1.0000 |
| 1.0000 | 0.0000 | 0.5814 | 0.0000 | 0.4186 | 0.3228 | 0.0478 | 1.5233 | 0.2616 | 80.6957 | 0.0000 | 0.0000 | 0.0000 | 0.1744 | 0.1860 | 0.9884 | 0.0000 | 0.0000 | 0.9419 |
| 1.0000 | 0.0000 | 0.2778 | 0.0000 | 0.7222 | 0.5432 | 0.0714 | 2.1111 | 0.5556 | 74.3227 | 0.0556 | 0.0000 | 0.0000 | 0.7500 | 0.5000 | 0.3889 | 0.0000 | 0.0000 | 0.8889 |
| 1.0000 | 0.0000 | 0.5495 | 0.0000 | 0.4505 | 0.7250 | 0.0972 | 2.1648 | 0.5824 | 80.6165 | 0.0000 | 0.0000 | 0.0000 | 0.8352 | 1.0000 | 1.0000 | 0.0000 | 0.0000 | 1.0000 |
| 1.0000 | 0.0000 | 0.3333 | 0.0000 | 0.6667 | 0.2786 | 0.0406 | 2.0000 | 0.5000 | 69.0950 | 0.0000 | 0.0000 | 0.0000 | 1.0000 | 1.0000 | 1.0000 | 0.0000 | 0.0000 | 1.0000 |
| 1.0000 | 0.0000 | 0.0385 | 0.0000 | 0.9615 | 0.1439 | 0.0242 | 2.9615 | 0.9808 | 68.6000 | 0.0000 | 0.0000 | 0.0000 | 0.0385 | 1.0000 | 1.0000 | 0.0000 | 0.0000 | 1.0000 |

| | | | | | | | | | | | | | | | | | | |
|---|---|---|---|---|---|---|---|---|---|---|---|---|---|---|---|---|---|---|
| 1.0000 | 0.0000 | 0.6000 | 0.0000 | 0.4000 | 0.8031 | 0.0985 | 2.1000 | 0.5500 | 75.9286 | 0.0000 | 0.0000 | 0.0000 | 0.6000 | 1.0000 | 0.8500 | 0.0000 | 0.0000 | 1.0000 |
| 1.0000 | 0.0000 | 0.1538 | 0.0000 | 0.8462 | 0.3123 | 0.0403 | 2.5385 | 0.7692 | 44.7700 | 0.0000 | 0.0000 | 0.0000 | 0.0000 | 0.8462 | 0.9231 | 0.0000 | 0.0000 | 1.0000 |
| 1.0000 | 0.0000 | 0.3333 | 0.0000 | 0.6667 | 0.9766 | 0.1079 | 1.0000 | 0.0000 | NA | 0.3333 | 0.0000 | 0.0000 | 0.3333 | 0.3333 | 0.6667 | 0.0000 | 0.0000 | 1.0000 |
| NA | NA | NA | NA | NA | NA | NA | NA | NA | 81.7057 | NA | NA | NA | NA | NA | NA | NA | NA | NA |
| 1.0000 | 0.0000 | 0.2564 | 0.0855 | 0.6581 | 1.8290 | 0.1505 | 1.2710 | 0.1355 | 75.4894 | 0.0000 | 0.0000 | 0.0000 | 0.2137 | 0.2735 | 0.8718 | 0.0000 | 0.0000 | 0.5726 |
| 1.0000 | 0.0000 | 0.0075 | 0.2985 | 0.6940 | 0.0860 | 0.0151 | 2.1364 | 0.5682 | 42.5600 | 0.0000 | 0.0000 | 0.0000 | 0.5299 | 0.6791 | 0.9851 | 0.0000 | 0.0000 | 0.6791 |
| 0.9818 | 0.0000 | 0.0000 | 0.0274 | 0.9726 | 0.0862 | 0.0147 | 2.1585 | 0.5793 | 44.7700 | 0.0000 | 0.0000 | 0.0000 | 0.7903 | 0.9726 | 0.9970 | 0.0000 | 0.0000 | 0.9909 |
| 1.0000 | 0.0000 | 0.0207 | 0.3161 | 0.6632 | 0.2168 | 0.0306 | 2.0208 | 0.5104 | 44.7700 | 0.1036 | 0.0000 | 0.0000 | 0.4767 | 0.5389 | 0.9741 | 0.0000 | 0.0000 | 0.5596 |
| 0.9885 | 0.0000 | 0.0000 | 0.0315 | 0.9685 | 0.2186 | 0.0250 | 2.0851 | 0.5426 | 83.3619 | 0.0458 | 0.0000 | 0.0000 | 0.3438 | 0.9169 | 0.5330 | 0.0000 | 0.0000 | 0.4756 |
| 0.9699 | 0.0000 | 0.0376 | 0.0526 | 0.9098 | 0.9213 | 0.0951 | 2.1802 | 0.5901 | 50.0618 | 0.0000 | 0.0000 | 0.0000 | 0.0376 | 0.6466 | 0.8045 | 0.0000 | 0.0000 | 0.7895 |
| 1.0000 | 0.0000 | 0.0000 | 0.5424 | 0.4576 | 0.8900 | 0.1049 | 1.9434 | 0.4717 | 59.9763 | 0.0508 | 0.0000 | 0.0000 | 0.2712 | 0.3729 | 0.8983 | 0.0000 | 0.0000 | 0.3729 |
| 1.0000 | 0.0000 | 0.0288 | 0.0360 | 0.9353 | 0.1923 | 0.0314 | 2.4925 | 0.7463 | 70.3152 | 0.0000 | 0.0000 | 0.0000 | 0.2158 | 0.9353 | 0.9640 | 0.0000 | 0.0000 | 0.7482 |
| 1.0000 | 0.0000 | 0.0000 | 0.0204 | 0.9796 | 0.2797 | 0.0441 | 2.0000 | 0.5000 | 71.7665 | 0.0000 | 0.0000 | 0.0000 | 0.4898 | 0.9796 | 0.9796 | 0.0000 | 0.0000 | 0.4898 |
| 1.0000 | 0.0000 | 0.2381 | 0.2857 | 0.4762 | 0.4673 | 0.0628 | 2.2381 | 0.6190 | 36.1845 | 0.0000 | 0.0000 | 0.0000 | 0.0000 | 0.4762 | 1.0000 | 0.0000 | 0.0000 | 0.7143 |
| 1.0000 | 0.0000 | 0.2222 | 0.4444 | 0.3333 | 0.6360 | 0.0822 | 2.1111 | 0.5556 | 30.0000 | 0.0000 | 0.0000 | 0.0000 | 0.0000 | 0.3333 | 1.0000 | 0.0000 | 0.0000 | 0.5556 |
| 1.0000 | 0.0000 | 0.0000 | 0.6486 | 0.3514 | 0.8181 | 0.1018 | 2.3243 | 0.6622 | 52.3267 | 0.0270 | 0.0000 | 0.0000 | 0.0000 | 0.3514 | 1.0000 | 0.0270 | 0.0000 | 0.3243 |
| 1.0000 | 0.0000 | 1.0000 | 0.0000 | 0.0000 | 0.2792 | 0.0456 | 1.1228 | 0.0614 | 38.8362 | 0.0000 | 0.0000 | 0.0000 | 0.1228 | 0.0000 | 1.0000 | 0.0000 | 0.0000 | 1.0000 |
| 1.0000 | 0.0000 | 0.4516 | 0.0323 | 0.5161 | 1.6246 | 0.1141 | 1.6667 | 0.3333 | 76.0036 | 0.0000 | 0.0000 | 0.0000 | 0.0000 | 0.3226 | 0.5161 | 0.0000 | 0.0000 | 0.9677 |
| 0.9623 | 0.0377 | 0.4151 | 0.0189 | 0.5660 | 0.7245 | 0.0702 | 1.9811 | 0.4906 | 76.7400 | 0.0000 | 0.0000 | 0.0000 | 0.0189 | 0.4528 | 0.5849 | 0.0000 | 0.0000 | 0.9434 |
| 0.9737 | 0.0000 | 0.5263 | 0.0000 | 0.4474 | 0.4972 | 0.0581 | 1.8649 | 0.4324 | 73.9600 | 0.0000 | 0.0000 | 0.0000 | 0.0000 | 0.4211 | 0.4474 | 0.0000 | 0.0000 | 0.9737 |
| 1.0000 | 0.0000 | 0.0000 | 0.4706 | 0.5294 | 0.1979 | 0.0321 | 1.5973 | 0.2986 | 73.9600 | 0.0181 | 0.0000 | 0.0000 | 0.4751 | 0.5068 | 1.0000 | 0.0000 | 0.0000 | 0.5294 |
| 0.9948 | 0.0000 | 0.1047 | 0.1204 | 0.7749 | 0.1726 | 0.0224 | 2.0733 | 0.5366 | 73.9600 | 0.0681 | 0.0000 | 0.0000 | 0.8063 | 0.7644 | 0.8953 | 0.0000 | 0.0052 | 0.7801 |
| 1.0000 | 0.0000 | 0.0000 | 0.5143 | 0.4857 | 0.1937 | 0.0315 | 1.6571 | 0.3286 | 34.8900 | 0.0286 | 0.0000 | 0.0000 | 0.4857 | 0.4571 | 1.0000 | 0.0000 | 0.0000 | 0.3714 |
| 0.7436 | 0.2564 | 0.2564 | 0.0513 | 0.6923 | 0.5540 | 0.0724 | 2.1282 | 0.5641 | 36.6700 | 0.0000 | 0.0000 | 0.0000 | 0.2308 | 0.4359 | 0.7436 | 0.0000 | 0.0000 | 0.7179 |
| 0.6645 | 0.3355 | 0.2581 | 0.0129 | 0.7290 | 0.9390 | 0.1084 | 2.0903 | 0.5452 | 51.8717 | 0.0000 | 0.0000 | 0.0000 | 0.1097 | 0.2129 | 0.7419 | 0.0000 | 0.0000 | 0.6645 |
| 1.0000 | 0.0000 | 0.0000 | 0.0000 | 1.0000 | 2.9126 | 0.1679 | 2.2000 | 0.6000 | 94.5833 | 0.0000 | 0.0000 | 0.0000 | 0.0000 | 0.6000 | 1.0000 | 0.0000 | 0.0000 | 1.0000 |
| 1.0000 | 0.0000 | 0.0000 | 0.8372 | 0.1628 | 0.4194 | 0.0566 | 1.2791 | 0.1395 | 36.8514 | 0.0000 | 0.0000 | 0.0000 | 0.0000 | 0.1395 | 1.0000 | 0.0000 | 0.0000 | 0.1628 |
| 0.5455 | 0.0000 | 0.0000 | 0.6364 | 0.3636 | 0.4713 | 0.0674 | 1.7273 | 0.3636 | NA | 0.0000 | 0.0000 | 0.0000 | 0.0000 | 0.3636 | 1.0000 | 0.0000 | 0.0000 | 0.8182 |
| 1.0000 | 0.0000 | 0.0000 | 0.4000 | 0.6000 | 1.6084 | 0.1083 | 1.8000 | 0.4000 | 98.5000 | 0.0000 | 0.0000 | 0.0000 | 0.0000 | 0.4000 | 1.0000 | 0.0000 | 0.0000 | 0.6000 |
| 1.0000 | 0.0000 | 0.0455 | 0.0000 | 0.9545 | 0.4455 | 0.0390 | 2.8182 | 0.9091 | 44.4620 | 0.0000 | 0.0000 | 0.0000 | 0.0000 | 0.9091 | 1.0000 | 0.0000 | 0.0000 | 1.0000 |
| 1.0000 | 0.0000 | 0.0000 | 0.0000 | 1.0000 | 1.1180 | 0.0736 | 2.7143 | 0.8571 | 63.8750 | 0.0000 | 0.0000 | 0.0000 | 0.0000 | 0.8571 | 1.0000 | 0.0000 | 0.0000 | 1.0000 |
| 1.0000 | 0.0000 | 0.0000 | 0.0000 | 1.0000 | 0.1210 | 0.0212 | 3.0000 | 1.0000 | 41.4242 | 0.0000 | 0.0000 | 0.0000 | 0.0000 | 1.0000 | 1.0000 | 0.0000 | 0.0000 | 1.0000 |
| 1.0000 | 0.0000 | 0.4000 | 0.0000 | 0.6000 | 2.6792 | 0.2027 | 1.4000 | 0.2000 | 48.0000 | 0.0000 | 0.0000 | 0.0000 | 0.0000 | 0.0000 | 0.6000 | 0.0000 | 0.0000 | 1.0000 |
| 1.0000 | 0.0000 | 0.3051 | 0.0000 | 0.6949 | 0.3452 | 0.0426 | 2.3559 | 0.6780 | 44.7700 | 0.0000 | 0.0000 | 0.0000 | 0.0000 | 0.6780 | 0.6949 | 0.0000 | 0.0000 | 1.0000 |
| 1.0000 | 0.0000 | 0.0000 | 0.0000 | 1.0000 | 7.1000 | 0.3879 | 1.0000 | 0.0000 | 53.1531 | 0.0000 | 0.0000 | 0.0000 | 0.0000 | 0.0000 | 1.0000 | 0.0000 | 0.0000 | 1.0000 |
| 1.0000 | 0.0000 | 0.0254 | 0.0042 | 0.9703 | 0.3493 | 0.0442 | 1.8596 | 0.4298 | 81.5711 | 0.0085 | 0.0000 | 0.0000 | 0.8898 | 0.8220 | 0.9958 | 0.0000 | 0.0000 | 0.8517 |
| 0.9677 | 0.0000 | 0.0000 | 0.0404 | 0.9596 | 0.7633 | 0.0545 | 1.9108 | 0.4554 | 74.8054 | 0.0377 | 0.0000 | 0.0000 | 0.6846 | 0.8086 | 0.9919 | 0.0000 | 0.0000 | 0.9003 |
| 0.9910 | 0.0000 | 0.0045 | 0.1036 | 0.8919 | 0.5113 | 0.0452 | 1.8145 | 0.4072 | 73.3347 | 0.0450 | 0.0000 | 0.0000 | 0.7477 | 0.7342 | 0.9955 | 0.0000 | 0.0000 | 0.8108 |
| 1.0000 | 0.0000 | 0.0000 | 0.9151 | 0.0849 | 0.8716 | 0.0966 | 1.6095 | 0.3048 | 52.9168 | 0.0000 | 0.0000 | 0.0000 | 0.0000 | 0.0755 | 0.5377 | 0.0000 | 0.0000 | 0.0755 |
| 1.0000 | 0.0000 | 0.0000 | 0.3429 | 0.6571 | 0.4953 | 0.0586 | 2.3429 | 0.6714 | 71.1412 | 0.0000 | 0.0000 | 0.0000 | 0.0857 | 0.6571 | 0.7714 | 0.0000 | 0.0000 | 0.6571 |
| 1.0000 | 0.0000 | 0.0000 | 0.4262 | 0.5738 | 0.5558 | 0.0697 | 2.0164 | 0.5082 | 66.7658 | 0.0000 | 0.0000 | 0.0000 | 0.3361 | 0.2295 | 0.8033 | 0.0000 | 0.0000 | 0.5656 |
| 0.7633 | 0.0000 | 0.0473 | 0.2722 | 0.6805 | 0.3607 | 0.0491 | 2.0592 | 0.5296 | 73.3972 | 0.0000 | 0.0000 | 0.0000 | 0.2012 | 0.6154 | 0.9527 | 0.0059 | 0.0000 | 0.9172 |
| 0.8841 | 0.0000 | 0.4348 | 0.1739 | 0.3913 | 0.3862 | 0.0566 | 1.7463 | 0.3731 | 55.5903 | 0.0000 | 0.0000 | 0.0000 | 0.0290 | 0.3768 | 0.5942 | 0.0000 | 0.0000 | 0.9420 |
| 0.9444 | 0.0000 | 0.0069 | 0.1667 | 0.8264 | 0.2960 | 0.0400 | 2.4653 | 0.7326 | 57.9803 | 0.0000 | 0.0000 | 0.0000 | 0.0417 | 0.7431 | 1.0000 | 0.0000 | 0.0000 | 0.8333 |
| 0.9667 | 0.0000 | 0.1331 | 0.0765 | 0.7903 | 0.6198 | 0.0675 | 2.3078 | 0.6539 | 59.4701 | 0.0000 | 0.0000 | 0.0000 | 0.0166 | 0.5324 | 0.9834 | 0.0000 | 0.0000 | 0.9484 |
| 0.9820 | 0.0000 | 0.1982 | 0.0721 | 0.7297 | 0.4562 | 0.0507 | 2.3333 | 0.6667 | 50.6199 | 0.0000 | 0.0000 | 0.0000 | 0.0541 | 0.6126 | 0.9640 | 0.0000 | 0.0000 | 0.9369 |
| 0.4601 | 0.0000 | 0.0000 | 0.6626 | 0.3374 | 1.2354 | 0.1106 | 1.5153 | 0.2577 | 54.8216 | 0.0000 | 0.0000 | 0.0000 | 0.0123 | 0.1963 | 1.0000 | 0.0000 | 0.0000 | 0.8712 |
| 0.7073 | 0.0000 | 0.0000 | 0.3902 | 0.6098 | 2.0970 | 0.1460 | 1.5854 | 0.2927 | 60.7662 | 0.0000 | 0.0000 | 0.0000 | 0.1098 | 0.2561 | 1.0000 | 0.0000 | 0.0000 | 0.8780 |
| 0.4359 | 0.0000 | 0.0040 | 0.5641 | 0.4319 | 1.2372 | 0.1132 | 1.4714 | 0.2357 | 54.2610 | 0.0000 | 0.0000 | 0.0000 | 0.1128 | 0.2619 | 1.0000 | 0.0000 | 0.0000 | 0.9871 |
| 0.9429 | 0.0000 | 0.0057 | 0.1769 | 0.8174 | 1.5377 | 0.1056 | 2.0674 | 0.5337 | 43.3541 | 0.0000 | 0.0000 | 0.0000 | 0.1598 | 0.5308 | 0.9943 | 0.0000 | 0.0000 | 0.8276 |
| 1.0000 | 0.0000 | 0.2568 | 0.2703 | 0.4730 | 1.5102 | 0.1308 | 2.1757 | 0.5878 | 74.2108 | 0.0000 | 0.0000 | 0.0000 | 0.3829 | 0.6351 | 0.5315 | 0.0000 | 0.0000 | 0.4775 |

| | | | | | | | | | | | | | | | | | | |
|---|---|---|---|---|---|---|---|---|---|---|---|---|---|---|---|---|---|---|
| 1.0000 | 0.0000 | 0.0311 | 0.5803 | 0.3886 | 1.2532 | 0.1356 | 1.6995 | 0.3497 | 64.3208 | 0.0052 | 0.0000 | 0.0104 | 0.0984 | 0.3938 | 0.4301 | 0.0104 | 0.0000 | 0.3990 |
| 0.9859 | 0.0000 | 0.0177 | 0.1731 | 0.8092 | 0.6342 | 0.0787 | 2.0638 | 0.5319 | 82.5498 | 0.0000 | 0.0000 | 0.0035 | 0.4240 | 0.5406 | 0.8693 | 0.0035 | 0.0000 | 0.8127 |
| 0.9883 | 0.0000 | 0.0058 | 0.0936 | 0.8889 | 0.6186 | 0.0536 | 2.1775 | 0.5888 | 53.1911 | 0.0936 | 0.0000 | 0.0000 | 0.3743 | 0.6667 | 0.9415 | 0.0000 | 0.0000 | 0.8012 |
| 1.0000 | 0.0000 | 0.0687 | 0.2887 | 0.6426 | 0.9472 | 0.1054 | 1.6220 | 0.3110 | 50.8750 | 0.0412 | 0.0000 | 0.0069 | 0.4296 | 0.4433 | 0.7251 | 0.0069 | 0.0000 | 0.6701 |
| 0.9913 | 0.0000 | 0.0131 | 0.3231 | 0.6638 | 0.7998 | 0.0864 | 2.0131 | 0.5066 | 66.0877 | 0.0175 | 0.0000 | 0.0000 | 0.3144 | 0.4236 | 0.7380 | 0.0000 | 0.0000 | 0.6638 |
| 1.0000 | 0.0000 | 0.0000 | 0.2524 | 0.7476 | 1.2175 | 0.1056 | 1.5922 | 0.2961 | 61.9932 | 0.0194 | 0.0000 | 0.0000 | 0.4175 | 0.3981 | 0.8447 | 0.0000 | 0.0000 | 0.6699 |
| 1.0000 | 0.0000 | 0.0317 | 0.0396 | 0.9287 | 1.6821 | 0.1192 | 2.2560 | 0.6280 | 69.1099 | 0.0317 | 0.0000 | 0.0000 | 0.1109 | 0.6260 | 0.9588 | 0.0000 | 0.0000 | 0.9192 |
| 1.0000 | 0.0000 | 0.0000 | 0.2603 | 0.7397 | 1.8638 | 0.1408 | 1.4225 | 0.2113 | NA | 0.0548 | 0.0000 | 0.0000 | 0.3562 | 0.1918 | 0.9589 | 0.0000 | 0.0000 | 0.6027 |
| 0.9825 | 0.0000 | 0.0263 | 0.1930 | 0.7807 | 1.2812 | 0.1175 | 1.5789 | 0.2895 | 60.0975 | 0.1053 | 0.0000 | 0.0000 | 0.3158 | 0.2982 | 0.8158 | 0.0000 | 0.0000 | 0.7105 |
| 1.0000 | 0.0000 | 0.0248 | 0.3230 | 0.6522 | 1.1592 | 0.1171 | 1.6646 | 0.3323 | 56.0218 | 0.0745 | 0.0000 | 0.0000 | 0.2050 | 0.3416 | 0.6522 | 0.0000 | 0.0000 | 0.5901 |
| 0.9904 | 0.0000 | 0.0962 | 0.3077 | 0.5962 | 0.8918 | 0.0845 | 2.0673 | 0.5337 | 53.5163 | 0.0577 | 0.0000 | 0.0000 | 0.0865 | 0.5192 | 0.7692 | 0.0000 | 0.0000 | 0.6250 |
| 0.9794 | 0.0000 | 0.0309 | 0.1856 | 0.7835 | 1.0597 | 0.0739 | 2.4845 | 0.7423 | 44.7700 | 0.0000 | 0.0000 | 0.0000 | 0.0000 | 0.6907 | 1.0000 | 0.0000 | 0.0000 | 0.8247 |
| 1.0000 | 0.0000 | 0.0072 | 0.0000 | 0.9928 | 1.3682 | 0.1117 | 2.2029 | 0.6014 | 66.9373 | 0.0000 | 0.0000 | 0.0000 | 0.0362 | 0.6159 | 1.0000 | 0.0000 | 0.0000 | 0.9928 |
| 1.0000 | 0.0000 | 0.0074 | 0.3556 | 0.6370 | 0.7719 | 0.0659 | 2.0889 | 0.5444 | 67.1560 | 0.0000 | 0.0000 | 0.0000 | 0.0444 | 0.4815 | 1.0000 | 0.0000 | 0.0000 | 0.6444 |
| 1.0000 | 0.0000 | 0.0000 | 0.0000 | 1.0000 | 0.5167 | 0.0722 | 1.0000 | 0.0000 | 44.7700 | 0.0000 | 0.0000 | 0.0000 | 0.9804 | 0.9804 | 1.0000 | 0.0000 | 0.0000 | 0.0098 |
| 1.0000 | 0.0000 | 0.0000 | 0.0000 | 1.0000 | 0.3945 | 0.0585 | 1.5155 | 0.2577 | 81.0757 | 0.0288 | 0.0000 | 0.0000 | 0.2017 | 0.9654 | 0.2795 | 0.0000 | 0.0000 | 0.1066 |
| 1.0000 | 0.0000 | 0.6522 | 0.0000 | 0.3478 | 0.2556 | 0.0411 | 1.2609 | 0.1304 | 73.9600 | 0.0000 | 0.0000 | 0.0000 | 0.0000 | 0.0000 | 1.0000 | 0.0000 | 0.0000 | 0.9130 |
| 1.0000 | 0.0000 | 0.3529 | 0.0000 | 0.6471 | 0.2095 | 0.0342 | 1.8235 | 0.4118 | 73.3487 | 0.0588 | 0.0000 | 0.0000 | 0.0588 | 0.2941 | 1.0000 | 0.0000 | 0.0000 | 0.9412 |
| 1.0000 | 0.0000 | 0.0000 | 1.0000 | 0.0000 | 1.7920 | 0.1904 | 1.0000 | 0.0000 | NA | 0.0000 | 0.0000 | 0.0000 | 0.0000 | 0.0000 | 0.0000 | 0.0000 | 0.0000 | 0.0000 |
| 1.0000 | 0.0000 | 0.5435 | 0.0000 | 0.4565 | 0.3514 | 0.0555 | 1.0000 | 0.0000 | 60.2686 | 0.1087 | 0.0000 | 0.0000 | 0.4348 | 0.3261 | 1.0000 | 0.0000 | 0.0000 | 0.6522 |
| 1.0000 | 0.0000 | 0.1992 | 0.0000 | 0.8008 | 2.4881 | 0.1602 | 1.3175 | 0.1587 | 55.7163 | 0.0199 | 0.0000 | 0.0000 | 0.2988 | 0.6175 | 0.5020 | 0.0000 | 0.0000 | 0.3785 |
| 1.0000 | 0.0000 | 0.0797 | 0.0000 | 0.9203 | 0.9475 | 0.0786 | 1.3922 | 0.1961 | 50.0000 | 0.0598 | 0.0000 | 0.0000 | 0.0598 | 0.8367 | 0.2032 | 0.0000 | 0.0000 | 0.1992 |
| 1.0000 | 0.0000 | 1.0000 | 0.0000 | 0.0000 | 0.3743 | 0.0585 | 1.0000 | 0.0000 | NA | 0.0000 | 0.0000 | 0.0000 | 0.0000 | 0.0000 | 0.5000 | 0.0000 | 0.0000 | 1.0000 |
| NA | NA | NA | NA | NA | NA | NA | NA | NA | NA | NA | NA | NA | NA | NA | NA | NA | NA | NA |
| 1.0000 | 0.0000 | 0.2746 | 0.0000 | 0.7254 | 0.2875 | 0.0467 | 1.9051 | 0.4526 | 54.5602 | 0.0000 | 0.0000 | 0.0000 | 0.3089 | 0.7254 | 0.3135 | 0.0000 | 0.0000 | 0.2792 |
| 1.0000 | 0.0000 | 0.0361 | 0.0000 | 0.9639 | 0.2805 | 0.0458 | 1.0000 | 0.0000 | 72.8773 | 0.0000 | 0.0000 | 0.0000 | 0.0000 | 0.9639 | 0.0361 | 0.0000 | 0.0000 | 0.0361 |
| 0.5946 | 0.4054 | 0.1622 | 0.0000 | 0.8378 | 0.8611 | 0.1054 | 2.5741 | 0.7870 | 72.9382 | 0.0000 | 0.0000 | 0.0000 | 0.0946 | 0.4324 | 0.7297 | 0.0000 | 0.0000 | 0.2973 |
| 1.0000 | 0.0000 | 0.0000 | 0.0000 | 1.0000 | 0.1210 | 0.0212 | 3.0000 | 1.0000 | NA | 0.0000 | 0.0000 | 0.0000 | 0.0000 | 1.0000 | 0.3333 | 0.0000 | 0.0000 | 0.3333 |
| 1.0000 | 0.0000 | 0.0000 | 0.0000 | 1.0000 | 0.2354 | 0.0272 | 2.9672 | 0.9836 | 66.2092 | 0.0000 | 0.0000 | 0.0000 | 0.0000 | 0.9917 | 0.5041 | 0.0000 | 0.0000 | 0.5041 |
| 1.0000 | 0.0000 | 0.0000 | 0.0000 | 1.0000 | NA | NA | NA | NA | 55.5441 | 0.0000 | 0.0000 | 0.0000 | 0.0000 | 1.0000 | 0.0000 | 0.0000 | 0.0000 | 0.0000 |
| 1.0000 | 0.0000 | 0.0000 | 0.0000 | 1.0000 | NA | NA | NA | NA | 83.7667 | 0.0000 | 0.0000 | 0.0000 | 0.0000 | 1.0000 | 0.0000 | 0.0000 | 0.0000 | 0.0000 |
| 1.0000 | 0.0000 | 0.2857 | 0.0000 | 0.7143 | 0.1666 | 0.0282 | 2.4286 | 0.7143 | 60.8402 | 0.0000 | 0.0000 | 0.0000 | 0.0000 | 0.7143 | 1.0000 | 0.0000 | 0.0000 | 1.0000 |
| 1.0000 | 0.0000 | 0.0000 | 0.0000 | 1.0000 | 0.4500 | 0.0689 | 1.0000 | 0.0000 | 79.7000 | 0.0000 | 0.0000 | 0.0000 | 1.0000 | 1.0000 | 1.0000 | 0.0000 | 0.0000 | 0.0000 |
| 1.0000 | 0.0000 | 0.0000 | 0.0000 | 1.0000 | NA | NA | NA | NA | 74.9879 | 0.0000 | 0.0000 | 0.0000 | 0.0000 | 1.0000 | 0.0000 | 0.0000 | 0.0000 | 0.0000 |
| 1.0000 | 0.0000 | 0.0000 | 0.4737 | 0.5263 | 0.9084 | 0.1124 | 2.2703 | 0.6351 | 50.2602 | 0.0000 | 0.0000 | 0.0000 | 0.0000 | 0.5263 | 0.6491 | 0.0000 | 0.0000 | 0.1754 |
| 1.0000 | 0.0000 | 0.0000 | 0.0165 | 0.9835 | 0.8844 | 0.0703 | 1.5412 | 0.2706 | 72.1866 | 0.0000 | 0.0000 | 0.0000 | 0.6116 | 0.9174 | 0.7025 | 0.0000 | 0.0000 | 0.4215 |
| 0.9626 | 0.0374 | 0.1682 | 0.0000 | 0.8318 | 0.4092 | 0.0475 | 2.5294 | 0.7647 | 89.0016 | 0.0000 | 0.0000 | 0.0000 | 0.1308 | 0.7850 | 0.4766 | 0.0000 | 0.0000 | 0.4393 |
| 1.0000 | 0.0000 | 0.0000 | 0.0000 | 1.0000 | 0.2760 | 0.0413 | 1.6000 | 0.3000 | 48.8952 | 0.0000 | 0.0000 | 0.0000 | 0.3333 | 0.7778 | 0.5556 | 0.0000 | 0.0000 | 0.3333 |
| NA | NA | NA | NA | NA | NA | NA | NA | NA | 44.3034 | NA | NA | NA | NA | NA | NA | NA | NA | NA |
| 1.0000 | 0.0000 | 0.0000 | 0.1266 | 0.8734 | 0.4665 | 0.0501 | 1.5823 | 0.2911 | 58.4721 | 0.4051 | 0.0000 | 0.0000 | 0.0000 | 0.0000 | 1.0000 | 0.0000 | 0.0000 | 0.4684 |
| 1.0000 | 0.0000 | 0.0000 | 0.0072 | 0.9928 | 0.4182 | 0.0530 | 1.9270 | 0.4635 | 62.5075 | 0.2899 | 0.0000 | 0.0000 | 0.3841 | 0.6087 | 0.9928 | 0.0000 | 0.0000 | 0.6884 |
| 1.0000 | 0.0000 | 0.3571 | 0.0000 | 0.6429 | 0.3315 | 0.0450 | 2.0566 | 0.5283 | 51.2576 | 0.0000 | 0.0000 | 0.0000 | 0.0893 | 0.4464 | 0.6964 | 0.0000 | 0.0000 | 0.9107 |
| 1.0000 | 0.0000 | 0.0000 | 0.0035 | 0.9965 | 0.2933 | 0.0313 | 2.5780 | 0.7890 | 70.4375 | 0.0000 | 0.0000 | 0.0000 | 0.3333 | 0.9220 | 1.0000 | 0.0000 | 0.0000 | 0.9929 |
| 1.0000 | 0.0000 | 0.0000 | 0.0000 | 1.0000 | 0.7680 | 0.1054 | 1.0000 | 0.0000 | 70.8116 | 0.0000 | 0.0000 | 0.0000 | 0.8000 | 0.0000 | 1.0000 | 0.0000 | 0.0000 | 0.8000 |
| 1.0000 | 0.0000 | 0.0000 | 0.6995 | 0.3005 | 1.2698 | 0.1377 | 1.6415 | 0.3208 | 78.5899 | 0.0000 | 0.0000 | 0.0000 | 0.1315 | 0.2441 | 0.6197 | 0.0000 | 0.0000 | 0.2066 |
| 1.0000 | 0.0000 | 0.1155 | 0.3213 | 0.5632 | 1.9997 | 0.1566 | 1.4188 | 0.2094 | 56.9550 | 0.0289 | 0.0000 | 0.0000 | 0.2599 | 0.2455 | 0.6245 | 0.0000 | 0.0000 | 0.6101 |
| 0.6771 | 0.3229 | 0.0179 | 0.1076 | 0.8744 | 0.8230 | 0.0978 | 2.1480 | 0.5740 | 44.7721 | 0.1794 | 0.0000 | 0.0000 | 0.3229 | 0.3049 | 0.8924 | 0.0179 | 0.0000 | 0.5426 |
| 0.9553 | 0.0000 | 0.5587 | 0.0447 | 0.3966 | 0.7004 | 0.0727 | 1.3006 | 0.1503 | 44.7700 | 0.0894 | 0.0000 | 0.0000 | 0.0950 | 0.2514 | 0.6201 | 0.0447 | 0.0000 | 0.8547 |
| 0.5429 | 0.4571 | 0.0743 | 0.0000 | 0.9257 | 1.0111 | 0.1092 | 2.9248 | 0.9624 | 65.8184 | 0.0000 | 0.0000 | 0.0000 | 0.0000 | 0.4457 | 0.8229 | 0.0000 | 0.0000 | 0.3714 |
| 0.9972 | 0.0000 | 0.0000 | 0.0983 | 0.9017 | 0.3361 | 0.0432 | 1.9379 | 0.4689 | 59.2239 | 0.0169 | 0.0000 | 0.0000 | 0.5562 | 0.5730 | 0.9045 | 0.0000 | 0.3820 | 0.8848 |
| 1.0000 | 0.0000 | 0.2469 | 0.0000 | 0.7531 | 2.0092 | 0.1245 | 1.7449 | 0.3724 | 61.9594 | 0.0658 | 0.0000 | 0.0000 | 0.1029 | 0.4280 | 0.7613 | 0.0000 | 0.0000 | 0.9095 |

| | | | | | | | | | | | | | | | | | | |
|---|---|---|---|---|---|---|---|---|---|---|---|---|---|---|---|---|---|---|
| 1.0000 | 0.0000 | 0.0690 | 0.0000 | 0.9310 | 1.8166 | 0.1114 | 2.3793 | 0.6897 | 49.1373 | 0.0000 | 0.0000 | 0.0000 | 0.0000 | 0.6897 | 1.0000 | 0.0000 | 0.0000 | 1.0000 |
| 1.0000 | 0.0000 | 0.1714 | 0.0286 | 0.8000 | 0.5949 | 0.0512 | 2.4857 | 0.7429 | 70.0680 | 0.0000 | 0.0000 | 0.0000 | 0.0000 | 0.7429 | 0.9714 | 0.0000 | 0.0000 | 0.9714 |
| 0.9292 | 0.0708 | 0.4956 | 0.4248 | 0.0796 | 1.0865 | 0.1219 | 1.1416 | 0.0708 | 69.3929 | 0.0000 | 0.0000 | 0.0000 | 0.0000 | 0.0000 | 0.4336 | 0.0000 | 0.0000 | 0.5044 |
| 1.0000 | 0.0000 | 0.0000 | 0.0055 | 0.9945 | 5.3307 | 0.3221 | 1.0000 | 0.0000 | 66.3150 | 0.0000 | 0.0000 | 0.0000 | 0.0000 | 0.9836 | 0.0109 | 0.0000 | 0.0000 | 0.0109 |
| 1.0000 | 0.0000 | 0.0746 | 0.0093 | 0.9160 | 0.7293 | 0.0624 | 1.9559 | 0.4779 | 57.7536 | 0.0000 | 0.0000 | 0.0000 | 0.0560 | 0.9049 | 0.2257 | 0.0000 | 0.0000 | 0.2052 |
| 0.8049 | 0.1951 | 0.6341 | 0.0000 | 0.3659 | 0.5414 | 0.0745 | 1.4390 | 0.2195 | 76.7400 | 0.0000 | 0.0000 | 0.0000 | 0.0732 | 0.0732 | 1.0000 | 0.0000 | 0.0000 | 0.6341 |
| 0.5556 | 0.4444 | 0.1111 | 0.1111 | 0.7778 | 0.8639 | 0.1097 | 1.8889 | 0.4444 | 87.8600 | 0.0000 | 0.0000 | 0.0000 | 0.3333 | 0.3333 | 1.0000 | 0.0000 | 0.0000 | 0.2222 |
| 1.0000 | 0.0000 | 1.0000 | 0.0000 | 0.0000 | 0.2805 | 0.0458 | 1.0000 | 0.0000 | 73.9272 | 0.0000 | 0.0000 | 0.0000 | 0.0000 | 0.0000 | 1.0000 | 0.0000 | 0.0000 | 1.0000 |
| 1.0000 | 0.0000 | 0.0088 | 0.0000 | 0.9912 | 0.2753 | 0.0451 | 1.5000 | 0.2500 | 67.8691 | 0.0000 | 0.0000 | 0.0000 | 0.0044 | 0.9912 | 0.0088 | 0.0000 | 0.0000 | 0.0088 |
| 1.0000 | 0.0000 | 0.0525 | 0.0021 | 0.9454 | 0.3078 | 0.0485 | 1.8077 | 0.4038 | 62.6173 | 0.0000 | 0.0000 | 0.0000 | 0.0420 | 0.9454 | 0.0546 | 0.0000 | 0.0000 | 0.0525 |
| 0.9286 | 0.0714 | 0.0714 | 0.0000 | 0.9286 | 0.6348 | 0.0855 | 1.7500 | 0.3750 | 71.3407 | 0.0000 | 0.0000 | 0.0000 | 0.2143 | 0.8571 | 0.2857 | 0.0000 | 0.0000 | 0.0714 |
| 1.0000 | 0.0000 | 0.0588 | 0.0000 | 0.9412 | 0.3679 | 0.0577 | 1.1176 | 0.0588 | 61.6088 | 0.0000 | 0.0000 | 0.0000 | 1.0000 | 0.9412 | 1.0000 | 0.0000 | 0.0000 | 1.0000 |
| NA | NA | NA | NA | NA | NA | NA | NA | NA | 75.7927 | NA | NA | NA | NA | NA | NA | NA | NA | NA |
| NA | NA | NA | NA | NA | NA | NA | NA | NA | 68.5229 | NA | NA | NA | NA | NA | NA | NA | NA | NA |
| 1.0000 | 0.0000 | 0.0162 | 0.0000 | 0.9838 | 5.1511 | 0.2901 | 1.0476 | 0.0238 | 72.2043 | 0.0000 | 0.0000 | 0.0000 | 0.0027 | 0.9434 | 0.0566 | 0.0000 | 0.0000 | 0.0566 |
| 1.0000 | 0.0000 | 0.0230 | 0.0000 | 0.9770 | 0.1398 | 0.0241 | 2.7647 | 0.8824 | 57.7608 | 0.0000 | 0.0000 | 0.0000 | 0.0000 | 0.9770 | 0.1954 | 0.0000 | 0.0000 | 0.1954 |
| 1.0000 | 0.0000 | 1.0000 | 0.0000 | 0.0000 | 0.2742 | 0.0449 | 1.6000 | 0.3000 | 59.1693 | 0.0000 | 0.0000 | 0.0000 | 0.6000 | 0.0000 | 1.0000 | 0.0000 | 0.0000 | 1.0000 |
| 1.0000 | 0.0000 | 0.5000 | 0.0000 | 0.5000 | 0.4403 | 0.0665 | 1.0000 | 0.0000 | 54.6743 | 0.0000 | 0.0000 | 0.0000 | 0.0000 | 0.0000 | 1.0000 | 0.0000 | 0.0000 | 0.5000 |
| 1.0000 | 0.0000 | 0.0351 | 0.0000 | 0.9649 | 0.2700 | 0.0443 | 2.0000 | 0.5000 | 66.9800 | 0.0000 | 0.0000 | 0.0000 | 0.0351 | 0.9649 | 0.0351 | 0.0000 | 0.0000 | 0.0351 |
| 1.0000 | 0.0000 | 0.2857 | 0.0000 | 0.7143 | 0.2700 | 0.0443 | 2.0000 | 0.5000 | 58.6654 | 0.0000 | 0.0000 | 0.0000 | 0.2857 | 0.7143 | 0.2857 | 0.0000 | 0.0000 | 0.2857 |
| 0.9167 | 0.0833 | 0.2619 | 0.0000 | 0.7381 | 0.3965 | 0.0552 | 2.2727 | 0.6364 | 56.6867 | 0.0000 | 0.0000 | 0.0000 | 0.1429 | 0.6548 | 0.5238 | 0.0000 | 0.0000 | 0.4405 |
| 0.9741 | 0.0259 | 0.0648 | 0.0000 | 0.9352 | 0.4329 | 0.0600 | 2.0656 | 0.5328 | 72.7153 | 0.0130 | 0.0000 | 0.0000 | 0.0259 | 0.8938 | 0.1451 | 0.0000 | 0.0000 | 0.1295 |
| NA | NA | NA | NA | NA | NA | NA | NA | NA | 67.6596 | NA | NA | NA | NA | NA | NA | NA | NA | NA |
| 0.8989 | 0.0674 | 0.2809 | 0.0899 | 0.6067 | 0.9919 | 0.1124 | 2.0000 | 0.5000 | 59.5520 | 0.0787 | 0.0000 | 0.0000 | 0.7753 | 0.6292 | 0.8876 | 0.1348 | 0.5843 | 0.8876 |
| 0.8916 | 0.0723 | 0.1566 | 0.0783 | 0.7651 | 0.6305 | 0.0787 | 2.1623 | 0.5812 | 63.5005 | 0.0241 | 0.0000 | 0.0120 | 0.8554 | 0.5602 | 0.9157 | 0.2831 | 0.7108 | 0.9096 |
| 0.8952 | 0.0484 | 0.1694 | 0.0726 | 0.7419 | 0.5795 | 0.0719 | 1.9558 | 0.4779 | 57.1811 | 0.0242 | 0.0000 | 0.0161 | 0.5645 | 0.3952 | 0.9032 | 0.1129 | 0.3871 | 0.8790 |
| 0.8532 | 0.0734 | 0.2202 | 0.0917 | 0.6697 | 0.7243 | 0.0888 | 1.9010 | 0.4505 | 61.6891 | 0.1193 | 0.0000 | 0.0183 | 0.7064 | 0.4771 | 0.8899 | 0.1376 | 0.4587 | 0.8532 |
| 0.9744 | 0.0000 | 0.1624 | 0.4017 | 0.4359 | 0.8124 | 0.0810 | 1.9589 | 0.4795 | 54.3673 | 0.0598 | 0.0000 | 0.0085 | 0.5128 | 0.3419 | 0.6068 | 0.1111 | 0.3333 | 0.5983 |
| 0.9846 | 0.0000 | 0.0615 | 0.6231 | 0.3154 | 0.3810 | 0.0445 | 1.9167 | 0.4583 | 68.7628 | 0.0462 | 0.0000 | 0.0000 | 0.2615 | 0.1615 | 0.4462 | 0.0462 | 0.1769 | 0.4385 |
| 0.9583 | 0.0000 | 0.1417 | 0.0667 | 0.7917 | 1.0211 | 0.1172 | 1.9912 | 0.4956 | 59.2076 | 0.0250 | 0.0000 | 0.0000 | 0.8333 | 0.6750 | 0.9000 | 0.0333 | 0.5500 | 0.8250 |
| 0.7925 | 0.1132 | 0.3208 | 0.1321 | 0.5472 | 0.9978 | 0.1196 | 1.7353 | 0.3676 | 63.5717 | 0.1415 | 0.0000 | 0.0000 | 0.7170 | 0.3962 | 0.8396 | 0.0377 | 0.3491 | 0.7736 |
| 0.9014 | 0.0704 | 0.2113 | 0.3239 | 0.4648 | 1.0556 | 0.1257 | 1.8235 | 0.4118 | 60.9319 | 0.0845 | 0.0000 | 0.0000 | 0.5634 | 0.2676 | 0.9296 | 0.0704 | 0.2676 | 0.8028 |
| 0.9123 | 0.0439 | 0.2105 | 0.3860 | 0.3947 | 1.1506 | 0.1270 | 1.8818 | 0.4409 | 59.5195 | 0.0702 | 0.0000 | 0.0000 | 0.5175 | 0.3158 | 0.9123 | 0.0877 | 0.3158 | 0.8684 |
| 0.9391 | 0.0174 | 0.1565 | 0.3826 | 0.4522 | 1.2179 | 0.1262 | 1.9099 | 0.4550 | 50.3250 | 0.0609 | 0.0000 | 0.0000 | 0.4957 | 0.3739 | 0.9043 | 0.0522 | 0.2783 | 0.8609 |
| 0.9536 | 0.0132 | 0.1457 | 0.3046 | 0.5430 | 1.2475 | 0.1320 | 1.9103 | 0.4552 | 62.1062 | 0.0331 | 0.0000 | 0.0000 | 0.6291 | 0.2119 | 0.9470 | 0.0861 | 0.2185 | 0.6556 |
| 0.8706 | 0.0706 | 0.2000 | 0.2235 | 0.5529 | 1.2509 | 0.1210 | 1.7975 | 0.3987 | 68.2218 | 0.1059 | 0.0000 | 0.0000 | 0.6353 | 0.5059 | 0.8824 | 0.0588 | 0.2824 | 0.8000 |
| 0.8545 | 0.0727 | 0.2182 | 0.1364 | 0.6273 | 1.0264 | 0.1203 | 1.8019 | 0.4009 | 58.3855 | 0.0909 | 0.0000 | 0.0000 | 0.6909 | 0.4273 | 0.9364 | 0.1091 | 0.3364 | 0.8182 |
| 0.8990 | 0.0606 | 0.2727 | 0.0909 | 0.6263 | 1.0344 | 0.1146 | 1.9149 | 0.4574 | 62.9373 | 0.0404 | 0.0000 | 0.0000 | 0.7273 | 0.5556 | 0.9192 | 0.1414 | 0.4646 | 0.8687 |
| 0.9455 | 0.0182 | 0.1273 | 0.0364 | 0.8364 | 0.9074 | 0.0941 | 2.0275 | 0.5138 | 67.8122 | 0.0545 | 0.0000 | 0.0364 | 0.8364 | 0.6000 | 0.9818 | 0.1273 | 0.6727 | 0.9636 |
| 0.9732 | 0.0179 | 0.0714 | 0.0804 | 0.8482 | 0.5979 | 0.0655 | 1.9459 | 0.4730 | 59.6292 | 0.0804 | 0.0000 | 0.0089 | 0.5268 | 0.3125 | 0.9732 | 0.0893 | 0.3839 | 0.9196 |
| 0.8539 | 0.1124 | 0.2360 | 0.1348 | 0.6292 | 0.6723 | 0.0869 | 1.9205 | 0.4602 | 65.0579 | 0.1798 | 0.0000 | 0.0225 | 0.7191 | 0.3708 | 0.9663 | 0.1124 | 0.4494 | 0.9551 |
| 0.9741 | 0.0000 | 0.1034 | 0.1293 | 0.7672 | 0.5966 | 0.0748 | 1.9561 | 0.4781 | 72.7704 | 0.0517 | 0.0000 | 0.0172 | 0.4914 | 0.2328 | 0.9828 | 0.1207 | 0.3276 | 0.9052 |
| 0.9867 | 0.0000 | 0.0933 | 0.1333 | 0.7733 | 0.8247 | 0.1015 | 1.9452 | 0.4726 | 64.0068 | 0.0533 | 0.0000 | 0.0000 | 0.8267 | 0.2933 | 0.9733 | 0.1600 | 0.4800 | 0.6267 |
| 0.9478 | 0.0348 | 0.1565 | 0.1391 | 0.7043 | 1.2728 | 0.1150 | 1.9469 | 0.4735 | 62.3130 | 0.0696 | 0.0000 | 0.0174 | 0.7565 | 0.5652 | 0.9652 | 0.0696 | 0.5913 | 0.8957 |
| 0.9597 | 0.0268 | 0.1074 | 0.0268 | 0.8523 | 1.5054 | 0.1344 | 2.1409 | 0.5705 | 63.2978 | 0.0537 | 0.0000 | 0.0000 | 0.9060 | 0.7584 | 0.9866 | 0.0537 | 0.7047 | 0.9463 |
| 0.9208 | 0.0594 | 0.2178 | 0.0594 | 0.7030 | 1.6953 | 0.1393 | 1.9278 | 0.4639 | 66.7458 | 0.1386 | 0.0000 | 0.0000 | 0.8614 | 0.4455 | 0.9604 | 0.1386 | 0.5644 | 0.8416 |
| 0.9674 | 0.0000 | 0.1413 | 0.0652 | 0.7935 | 1.0877 | 0.1201 | 1.8667 | 0.4333 | 62.1704 | 0.1522 | 0.0000 | 0.0217 | 0.8587 | 0.5217 | 0.9457 | 0.0652 | 0.6196 | 0.8913 |
| 0.9817 | 0.0000 | 0.1560 | 0.0734 | 0.7615 | 1.3938 | 0.1494 | 1.9346 | 0.4673 | 63.4556 | 0.0734 | 0.0000 | 0.0000 | 0.8991 | 0.6697 | 0.9358 | 0.1284 | 0.7339 | 0.9174 |
| 0.9658 | 0.0000 | 0.1538 | 0.0427 | 0.7863 | 1.3434 | 0.1445 | 1.9561 | 0.4781 | 58.0340 | 0.0513 | 0.0000 | 0.0000 | 0.9316 | 0.6410 | 0.9402 | 0.1709 | 0.7436 | 0.8974 |
| 0.9664 | 0.0000 | 0.1345 | 0.0504 | 0.7983 | 0.9170 | 0.1113 | 1.9652 | 0.4826 | 63.2536 | 0.0504 | 0.0000 | 0.0000 | 0.8824 | 0.6891 | 0.9160 | 0.1176 | 0.7395 | 0.9076 |
| 0.9510 | 0.0196 | 0.1863 | 0.0784 | 0.7255 | 1.3165 | 0.1259 | 1.9896 | 0.4948 | 59.8317 | 0.0784 | 0.0000 | 0.0000 | 0.8627 | 0.4608 | 0.9118 | 0.2059 | 0.5686 | 0.8333 |

| | | | | | | | | | | | | | | | | | | |
|---|---|---|---|---|---|---|---|---|---|---|---|---|---|---|---|---|---|---|
| 0.9369 | 0.0180 | 0.1081 | 0.0721 | 0.8108 | 1.6307 | 0.1539 | 1.8037 | 0.4019 | 61.8240 | 0.0541 | 0.0000 | 0.0000 | 0.7748 | 0.2432 | 0.9099 | 0.0811 | 0.2432 | 0.5405 |
| 0.8588 | 0.0471 | 0.2588 | 0.2000 | 0.5176 | 0.9016 | 0.1085 | 1.8272 | 0.4136 | 62.4902 | 0.1176 | 0.0000 | 0.0235 | 0.7059 | 0.3059 | 0.9059 | 0.1412 | 0.4235 | 0.8235 |
| 0.8174 | 0.1796 | 0.2216 | 0.1257 | 0.6497 | 0.5560 | 0.0749 | 1.9880 | 0.4940 | 67.7794 | 0.1826 | 0.0000 | 0.0000 | 0.6766 | 0.3802 | 0.9910 | 0.2036 | 0.3413 | 0.9760 |
| 0.9392 | 0.0570 | 0.1141 | 0.2319 | 0.6540 | 0.8059 | 0.1043 | 1.9389 | 0.4695 | 72.7235 | 0.0646 | 0.0000 | 0.0000 | 0.7567 | 0.4563 | 0.9886 | 0.1255 | 0.3954 | 0.9772 |
| 0.6900 | 0.2952 | 0.3985 | 0.1513 | 0.4502 | 0.8825 | 0.1100 | 1.7000 | 0.3500 | 73.5481 | 0.3026 | 0.0000 | 0.0000 | 0.8339 | 0.3616 | 0.9815 | 0.0886 | 0.3284 | 0.9668 |
| 0.9815 | 0.0037 | 0.3247 | 0.0627 | 0.6089 | 1.3370 | 0.1481 | 1.9765 | 0.4882 | 60.4679 | 0.0037 | 0.0000 | 0.0000 | 0.9188 | 0.5498 | 0.9262 | 0.3542 | 0.8672 | 0.9336 |
| 0.9412 | 0.0037 | 0.3603 | 0.0515 | 0.5882 | 1.3035 | 0.1477 | 1.9470 | 0.4735 | 54.9665 | 0.0037 | 0.0000 | 0.0000 | 0.9007 | 0.5625 | 0.9154 | 0.3235 | 0.7941 | 0.9596 |
| 0.9648 | 0.0313 | 0.2734 | 0.1211 | 0.6016 | 1.1899 | 0.1350 | 1.9435 | 0.4717 | 72.7193 | 0.0391 | 0.0000 | 0.0000 | 0.8750 | 0.5742 | 0.8945 | 0.2344 | 0.7344 | 0.8945 |
| 0.8745 | 0.1126 | 0.2121 | 0.0563 | 0.7316 | 1.3060 | 0.1425 | 1.8855 | 0.4427 | 68.3904 | 0.1212 | 0.0000 | 0.0000 | 0.9351 | 0.6494 | 0.9784 | 0.1342 | 0.6840 | 0.9524 |
| 0.9561 | 0.0351 | 0.1053 | 0.0570 | 0.8377 | 1.1715 | 0.1319 | 1.9182 | 0.4591 | 64.8102 | 0.0439 | 0.0000 | 0.0000 | 0.9079 | 0.7018 | 0.9605 | 0.1447 | 0.7061 | 0.9518 |
| 0.9412 | 0.0210 | 0.2479 | 0.0756 | 0.6765 | 1.1516 | 0.1312 | 1.8924 | 0.4462 | 77.3348 | 0.0294 | 0.0000 | 0.0000 | 0.7899 | 0.5420 | 0.9034 | 0.1891 | 0.6345 | 0.9076 |
| 0.9500 | 0.0125 | 0.0250 | 0.6625 | 0.3125 | 0.5288 | 0.0677 | 1.9615 | 0.4808 | 61.2170 | 0.0500 | 0.0125 | 0.0000 | 0.2875 | 0.1750 | 0.6750 | 0.0375 | 0.1875 | 0.6500 |
| 0.7017 | 0.2068 | 0.2203 | 0.0949 | 0.6847 | 0.7139 | 0.0959 | 1.7440 | 0.3720 | 38.1691 | 0.2915 | 0.0000 | 0.0000 | 0.8305 | 0.4136 | 0.9898 | 0.0068 | 0.3085 | 0.7864 |
| 0.7476 | 0.2013 | 0.2141 | 0.1374 | 0.6486 | 0.8170 | 0.1014 | 1.7910 | 0.3955 | 55.4572 | 0.2556 | 0.0000 | 0.0000 | 0.7604 | 0.5048 | 0.9904 | 0.0064 | 0.3642 | 0.8594 |
| 0.7517 | 0.2245 | 0.2415 | 0.2143 | 0.5442 | 0.8556 | 0.1081 | 1.7911 | 0.3955 | 55.0476 | 0.2245 | 0.0000 | 0.0000 | 0.7517 | 0.3027 | 0.9898 | 0.0204 | 0.2687 | 0.7551 |
| 0.6938 | 0.2704 | 0.2834 | 0.1661 | 0.5505 | 0.7776 | 0.0997 | 1.6711 | 0.3356 | 37.9941 | 0.2932 | 0.0000 | 0.0000 | 0.7655 | 0.2801 | 0.9674 | 0.0098 | 0.2280 | 0.7590 |
| 0.6731 | 0.0577 | 0.0769 | 0.0769 | 0.8397 | 0.5873 | 0.0766 | 1.9048 | 0.4524 | 37.9391 | 0.2949 | 0.0000 | 0.0000 | 0.8782 | 0.3462 | 0.9359 | 0.0385 | 0.0962 | 0.6795 |
| 0.9630 | 0.0053 | 0.0265 | 0.3122 | 0.6561 | 0.5002 | 0.0670 | 2.0153 | 0.5076 | 40.4395 | 0.0370 | 0.0053 | 0.0000 | 0.6296 | 0.5185 | 0.6931 | 0.0265 | 0.4974 | 0.6190 |
| 0.6314 | 0.3650 | 0.3978 | 0.1022 | 0.5000 | 0.6251 | 0.0860 | 1.6008 | 0.3004 | 59.5611 | 0.3723 | 0.0000 | 0.0000 | 0.8796 | 0.4745 | 0.9015 | 0.0182 | 0.4343 | 0.8905 |
| 0.9585 | 0.0377 | 0.1132 | 0.2340 | 0.6528 | 0.6342 | 0.0763 | 1.9706 | 0.4853 | 49.5198 | 0.1321 | 0.0000 | 0.0000 | 0.7472 | 0.5396 | 0.7660 | 0.0604 | 0.6189 | 0.7509 |
| 0.9331 | 0.0595 | 0.1413 | 0.3048 | 0.5539 | 0.5934 | 0.0726 | 1.9787 | 0.4894 | 75.7177 | 0.0669 | 0.0000 | 0.0000 | 0.5688 | 0.4981 | 0.6952 | 0.0706 | 0.3755 | 0.6394 |
| 0.9478 | 0.0336 | 0.1157 | 0.2500 | 0.6343 | 0.6975 | 0.0856 | 1.9372 | 0.4686 | 54.3792 | 0.0410 | 0.0000 | 0.0000 | 0.6455 | 0.5672 | 0.7575 | 0.0187 | 0.4813 | 0.6716 |
| 0.9452 | 0.0411 | 0.1279 | 0.1233 | 0.7489 | 0.7960 | 0.0976 | 1.9439 | 0.4719 | 55.2971 | 0.0639 | 0.0000 | 0.0000 | 0.7854 | 0.6667 | 0.8904 | 0.0228 | 0.5753 | 0.7717 |
| 0.7133 | 0.2800 | 0.3533 | 0.1433 | 0.5033 | 0.8026 | 0.0896 | 1.6592 | 0.3296 | 63.3455 | 0.2833 | 0.0000 | 0.0000 | 0.7767 | 0.2800 | 0.8867 | 0.0533 | 0.2267 | 0.8600 |
| 0.9132 | 0.0661 | 0.1033 | 0.0455 | 0.8512 | 0.7387 | 0.0999 | 1.7595 | 0.3797 | 58.4665 | 0.0744 | 0.0000 | 0.0000 | 0.6488 | 0.4091 | 0.9628 | 0.0083 | 0.2851 | 0.8678 |
| 0.9113 | 0.0567 | 0.1738 | 0.1631 | 0.6631 | 0.5400 | 0.0716 | 2.0581 | 0.5290 | 66.2014 | 0.0638 | 0.0000 | 0.0142 | 0.6418 | 0.5177 | 0.8262 | 0.2128 | 0.5035 | 0.8369 |
| 0.9118 | 0.0765 | 0.1618 | 0.1294 | 0.7088 | 0.5545 | 0.0736 | 2.0201 | 0.5100 | 65.8591 | 0.0882 | 0.0000 | 0.0029 | 0.7265 | 0.5412 | 0.8765 | 0.2265 | 0.5588 | 0.8676 |
| 0.8819 | 0.1146 | 0.1424 | 0.1215 | 0.7361 | 1.0667 | 0.1049 | 1.6468 | 0.3234 | 80.1279 | 0.1181 | 0.0000 | 0.0035 | 0.4479 | 0.5417 | 0.9306 | 0.0069 | 0.3056 | 0.9306 |
| 0.7533 | 0.2423 | 0.2599 | 0.2026 | 0.5374 | 1.6673 | 0.1288 | 1.4775 | 0.2387 | 63.0159 | 0.2467 | 0.0000 | 0.0000 | 0.4185 | 0.2731 | 0.9780 | 0.0044 | 0.1498 | 0.9648 |
| 0.7441 | 0.2525 | 0.3030 | 0.2593 | 0.4377 | 1.4468 | 0.1206 | 1.5730 | 0.2865 | 72.9903 | 0.2525 | 0.0000 | 0.0000 | 0.4714 | 0.3300 | 0.9461 | 0.0135 | 0.1818 | 0.9360 |
| 0.9696 | 0.0190 | 0.0837 | 0.2281 | 0.6882 | 0.8978 | 0.0954 | 1.8361 | 0.4180 | 55.9326 | 0.1749 | 0.0000 | 0.0000 | 0.3688 | 0.4715 | 0.9278 | 0.0190 | 0.3004 | 0.9202 |
| 0.6889 | 0.3000 | 0.3370 | 0.1889 | 0.4741 | 0.5860 | 0.0770 | 1.6582 | 0.3291 | 73.6342 | 0.3074 | 0.0000 | 0.0000 | 0.4370 | 0.4741 | 0.8741 | 0.0074 | 0.0778 | 0.8556 |
| 0.8305 | 0.1695 | 0.5452 | 0.0000 | 0.4548 | 0.9839 | 0.1059 | 1.5884 | 0.2942 | 82.0384 | 0.0000 | 0.0000 | 0.0000 | 0.9887 | 0.3051 | 0.9887 | 0.5367 | 0.6215 | 0.9887 |
| 0.7312 | 0.2688 | 0.5108 | 0.0000 | 0.4892 | 0.9394 | 0.1056 | 1.6267 | 0.3134 | 65.9403 | 0.0027 | 0.0000 | 0.0000 | 0.9973 | 0.2930 | 0.9973 | 0.4866 | 0.7581 | 0.9973 |
| 0.9945 | 0.0055 | 0.0165 | 0.2582 | 0.7253 | 0.2547 | 0.0322 | 1.9783 | 0.4891 | 75.1544 | 0.1484 | 0.0000 | 0.0055 | 0.2363 | 0.0440 | 0.5275 | 0.0549 | 0.0769 | 0.5110 |
| 1.0000 | 0.0000 | 0.0068 | 0.0959 | 0.6233 | 1.5358 | 0.1564 | 1.6691 | 0.3346 | 77.1620 | 0.0137 | 0.0000 | 0.0000 | 0.0685 | 0.0137 | 0.9589 | 0.0000 | 0.0205 | 0.9110 |
| 1.0000 | 0.0000 | 0.0208 | 0.1944 | 0.7778 | 1.4944 | 0.1471 | 1.9402 | 0.4701 | 80.6885 | 0.0139 | 0.0000 | 0.0069 | 0.2500 | 0.0347 | 0.8403 | 0.0139 | 0.0556 | 0.6528 |
| 1.0000 | 0.0000 | 0.0728 | 0.4903 | 0.4369 | 1.2940 | 0.1173 | 1.4579 | 0.2289 | 42.7137 | 0.0049 | 0.0000 | 0.0000 | 0.0340 | 0.0922 | 0.4369 | 0.0049 | 0.0243 | 0.4369 |
| 1.0000 | 0.0000 | 0.0364 | 0.4545 | 0.5030 | 0.9673 | 0.0942 | 1.4928 | 0.2464 | 58.6276 | 0.0121 | 0.0000 | 0.0061 | 0.1394 | 0.0182 | 0.4970 | 0.0121 | 0.0061 | 0.4788 |
| 1.0000 | 0.0000 | 0.2564 | 0.3077 | 0.4359 | 0.8081 | 0.0769 | 1.8966 | 0.4483 | 65.8312 | 0.0256 | 0.0000 | 0.0000 | 0.0128 | 0.2692 | 0.6923 | 0.0064 | 0.0192 | 0.6667 |
| 0.9908 | 0.0046 | 0.0276 | 0.0000 | 0.9724 | 0.3866 | 0.0565 | 2.2441 | 0.6221 | 59.4105 | 0.0230 | 0.0000 | 0.0000 | 1.0000 | 0.5853 | 1.0000 | 0.3779 | 0.9124 | 1.0000 |
| 1.0000 | 0.0000 | 0.0106 | 0.4444 | 0.5450 | 1.5814 | 0.0606 | 1.7734 | 0.3867 | 53.8991 | 0.0265 | 0.0000 | 0.0000 | 0.0688 | 0.0212 | 0.6032 | 0.0053 | 0.0159 | 0.5979 |
| 1.0000 | 0.0000 | 0.0113 | 0.0169 | 0.9718 | 0.6086 | 0.0847 | 2.0000 | 0.5000 | 60.5093 | 0.1469 | 0.0000 | 0.0000 | 0.9661 | 0.0226 | 1.0000 | 0.0113 | 0.0282 | 0.9887 |
| 1.0000 | 0.0000 | 0.2712 | 0.0000 | 0.7288 | 0.9365 | 0.1014 | 1.9953 | 0.4977 | 44.1511 | 0.0169 | 0.0000 | 0.0000 | 0.9966 | 0.3593 | 1.0000 | 0.3390 | 0.6949 | 1.0000 |
| 0.9915 | 0.0000 | 0.0169 | 0.7797 | 0.2034 | 1.4817 | 0.1337 | 2.0189 | 0.5094 | 54.8425 | 0.0508 | 0.0000 | 0.0000 | 0.1441 | 0.0508 | 0.9915 | 0.0085 | 0.0169 | 0.5678 |
| 1.0000 | 0.0000 | 0.1020 | 0.1633 | 0.7347 | 1.4297 | 0.0521 | 1.8611 | 0.4306 | 60.4185 | 0.5102 | 0.0000 | 0.0000 | 0.7755 | 0.1224 | 1.0000 | 0.0000 | 0.0408 | 0.9796 |
| 1.0000 | 0.0000 | 0.0076 | 0.8626 | 0.0687 | 2.7528 | 0.1632 | 1.9237 | 0.4619 | 56.8602 | 0.0458 | 0.0000 | 0.0000 | 0.0458 | 0.0000 | 0.9695 | 0.0000 | 0.0000 | 0.3740 |
| 1.0000 | 0.0000 | 0.0106 | 0.2553 | 0.7340 | 14.3065 | 0.2545 | 1.6941 | 0.3471 | 60.2442 | 0.0106 | 0.0000 | 0.0000 | 0.3085 | 0.0213 | 1.0000 | 0.0000 | 0.0106 | 0.9787 |
| 0.9906 | 0.0000 | 0.0094 | 0.2170 | 0.5943 | 34.1770 | 0.5421 | 1.2353 | 0.1176 | 52.9928 | 0.1698 | 0.0000 | 0.0000 | 0.5849 | 0.0094 | 0.8585 | 0.0000 | 0.0000 | 0.8868 |
| 1.0000 | 0.0000 | 0.0183 | 0.5688 | 0.4128 | 1.7220 | 0.1466 | 1.9346 | 0.4673 | 69.6899 | 0.0000 | 0.0000 | 0.0000 | 0.3211 | 0.0275 | 1.0000 | 0.0092 | 0.0183 | 0.9725 |
| 0.9942 | 0.0058 | 0.0291 | 0.3488 | 0.6221 | 0.5466 | 0.0667 | 1.9593 | 0.4797 | 58.2605 | 0.5000 | 0.0000 | 0.0000 | 0.6163 | 0.0872 | 1.0000 | 0.0233 | 0.1047 | 0.6512 |

| | | | | | | | | | | | | | | | | | | |
|---|---|---|---|---|---|---|---|---|---|---|---|---|---|---|---|---|---|---|
| 1.0000 | 0.0000 | 0.0000 | 0.9266 | 0.0642 | 3.0934 | 0.1776 | 1.9352 | 0.4676 | 87.7627 | 0.0092 | 0.0000 | 0.0000 | 0.0367 | 0.0000 | 1.0000 | 0.0092 | 0.0000 | 0.7523 |
| 1.0000 | 0.0000 | 0.0081 | 0.4516 | 0.5323 | 0.5763 | 0.0742 | 1.9907 | 0.4953 | 73.2469 | 0.0000 | 0.0000 | 0.0000 | 0.0484 | 0.0403 | 1.0000 | 0.0000 | 0.0403 | 1.0000 |
| 0.9868 | 0.0000 | 0.0000 | 0.3947 | 0.5526 | 0.6345 | 0.0769 | 2.0476 | 0.5238 | 80.3896 | 0.0789 | 0.0000 | 0.0000 | 0.1184 | 0.1053 | 0.9342 | 0.0000 | 0.0000 | 0.6974 |
| 0.9955 | 0.0000 | 0.1345 | 0.0045 | 0.8610 | 1.1469 | 0.1260 | 1.9013 | 0.4507 | 80.8003 | 0.0045 | 0.0000 | 0.0000 | 0.9955 | 0.6099 | 1.0000 | 0.3812 | 0.8296 | 1.0000 |
| 1.0000 | 0.0000 | 0.0249 | 0.0000 | 0.9751 | 0.2379 | 0.0380 | 2.0149 | 0.5075 | 72.9422 | 0.0398 | 0.0000 | 0.0000 | 0.9801 | 0.1443 | 0.9801 | 0.8010 | 0.5771 | 0.9801 |
| 0.9697 | 0.0303 | 0.1273 | 0.0182 | 0.8242 | 2.0306 | 0.0985 | 1.9177 | 0.4589 | 83.7216 | 0.4061 | 0.0000 | 0.0061 | 0.8909 | 0.3636 | 0.9697 | 0.0303 | 0.3091 | 0.9576 |
| 1.0000 | 0.0000 | 0.0215 | 0.0108 | 0.9677 | 1.3830 | 0.0888 | 1.9640 | 0.4820 | 74.0673 | 0.0358 | 0.0000 | 0.0036 | 0.7025 | 0.3369 | 1.0000 | 0.2903 | 0.6308 | 0.9964 |
| 1.0000 | 0.0000 | 0.0000 | 0.1088 | 0.8912 | 1.6127 | 0.1218 | 1.9452 | 0.4726 | 71.0296 | 0.0544 | 0.0000 | 0.0068 | 0.8503 | 0.7007 | 1.0000 | 0.0136 | 0.7211 | 1.0000 |
| 1.0000 | 0.0000 | 0.0348 | 0.0000 | 0.9609 | 0.8845 | 0.0771 | 2.0349 | 0.5175 | 71.8136 | 0.0000 | 0.0000 | 0.0000 | 0.9957 | 0.9913 | 0.9957 | 0.0043 | 0.0348 | 0.9957 |
| 0.9952 | 0.0048 | 0.5266 | 0.0000 | 0.4734 | 1.6537 | 0.1583 | 1.5266 | 0.2633 | 73.3853 | 0.0000 | 0.0000 | 0.0000 | 1.0000 | 0.6039 | 1.0000 | 0.4493 | 0.0290 | 1.0000 |
| 0.9781 | 0.0219 | 0.0482 | 0.0044 | 0.9474 | 0.9347 | 0.1050 | 2.2368 | 0.6184 | 70.8071 | 0.0132 | 0.0000 | 0.0000 | 0.9737 | 0.8860 | 0.9825 | 0.0439 | 0.9211 | 0.9825 |
| 0.9846 | 0.0000 | 0.3861 | 0.0000 | 0.6139 | 1.1041 | 0.1219 | 1.9073 | 0.4537 | 75.9482 | 0.0077 | 0.0000 | 0.0000 | 0.9421 | 0.7220 | 0.9421 | 0.2085 | 0.3089 | 0.9421 |
| 0.9944 | 0.0056 | 0.1889 | 0.0222 | 0.7889 | 0.8378 | 0.1011 | 2.0782 | 0.5391 | 70.1622 | 0.0500 | 0.0000 | 0.0000 | 0.9611 | 0.6611 | 0.9722 | 0.2667 | 0.5500 | 0.9722 |
| 0.9932 | 0.0000 | 0.1837 | 0.0068 | 0.8095 | 0.6514 | 0.0801 | 1.9048 | 0.4524 | 55.9569 | 0.0068 | 0.0000 | 0.0000 | 0.9728 | 0.2109 | 0.9728 | 0.7551 | 0.2517 | 0.9728 |
| 0.9919 | 0.0081 | 0.0847 | 0.0000 | 0.9153 | 0.7592 | 0.0839 | 1.9960 | 0.4980 | 67.5701 | 0.1048 | 0.0000 | 0.0000 | 0.9395 | 0.2540 | 0.9395 | 0.5766 | 0.9153 | 0.9395 |
| 0.9939 | 0.0061 | 0.1043 | 0.3620 | 0.5337 | 0.6460 | 0.0848 | 2.1728 | 0.5864 | 70.8089 | 0.0368 | 0.0000 | 0.0000 | 0.6564 | 0.3681 | 1.0000 | 0.2577 | 0.4724 | 1.0000 |
| 0.9951 | 0.0049 | 0.4369 | 0.0000 | 0.5631 | 1.4720 | 0.1467 | 1.6019 | 0.3010 | 69.9947 | 0.0000 | 0.0000 | 0.0000 | 0.9757 | 0.5874 | 0.9806 | 0.3932 | 0.2573 | 0.9806 |
| 0.9943 | 0.0057 | 0.1136 | 0.3409 | 0.5455 | 0.5918 | 0.0748 | 1.7714 | 0.3857 | 65.8697 | 0.0057 | 0.0000 | 0.0000 | 0.4261 | 0.2386 | 1.0000 | 0.1818 | 0.3807 | 1.0000 |
| 0.9947 | 0.0053 | 0.0802 | 0.0000 | 0.9198 | 1.3831 | 0.1434 | 2.1765 | 0.5882 | 70.8449 | 0.0053 | 0.0000 | 0.0000 | 0.9572 | 0.8235 | 0.9786 | 0.1283 | 0.4706 | 0.9733 |
| 0.9912 | 0.0088 | 0.1974 | 0.0746 | 0.7281 | 0.8473 | 0.1060 | 2.1278 | 0.5639 | 73.1727 | 0.0307 | 0.0000 | 0.0000 | 0.8947 | 0.5526 | 0.9956 | 0.3202 | 0.7544 | 0.9868 |
| 0.9465 | 0.0033 | 0.0569 | 0.6221 | 0.3211 | 1.2408 | 0.1412 | 1.9097 | 0.4548 | 67.5451 | 0.0870 | 0.0000 | 0.0000 | 0.4080 | 0.2441 | 1.0000 | 0.0803 | 0.3612 | 0.9967 |
| 0.9500 | 0.0500 | 0.1000 | 0.0000 | 0.9000 | 0.7872 | 0.0866 | 2.0150 | 0.5075 | 76.0645 | 0.0375 | 0.0000 | 0.0000 | 0.8125 | 0.4917 | 0.8167 | 0.3000 | 0.4917 | 0.8167 |
| 1.0000 | 0.0000 | 0.0459 | 0.5102 | 0.4439 | 1.3713 | 0.1346 | 1.9792 | 0.4896 | 79.9399 | 0.0051 | 0.0000 | 0.0000 | 0.4796 | 0.3776 | 1.0000 | 0.0204 | 0.3571 | 1.0000 |
| 1.0000 | 0.0000 | 0.2353 | 0.0000 | 0.7647 | 0.1483 | 0.0232 | 1.9660 | 0.4830 | 77.8645 | 0.0000 | 0.0000 | 0.0000 | 1.0000 | 0.7647 | 1.0000 | 0.0214 | 0.7594 | 1.0000 |
| 0.9815 | 0.0185 | 0.0406 | 0.0037 | 0.9557 | 1.1658 | 0.1212 | 2.0000 | 0.5000 | 76.0409 | 0.0185 | 0.0000 | 0.0000 | 0.9963 | 0.4871 | 0.9963 | 0.4797 | 0.9483 | 0.9926 |
| 1.0000 | 0.0000 | 0.1323 | 0.0053 | 0.8624 | 1.2378 | 0.1069 | 1.8201 | 0.4101 | 79.0545 | 0.0794 | 0.0000 | 0.0000 | 0.9153 | 0.7302 | 1.0000 | 0.0794 | 0.6614 | 0.9894 |
| 1.0000 | 0.0000 | 0.0773 | 0.0193 | 0.9034 | 0.3008 | 0.0401 | 1.9212 | 0.4606 | 90.1928 | 0.0000 | 0.0000 | 0.0000 | 0.9758 | 0.7005 | 1.0000 | 0.2657 | 0.8599 | 1.0000 |
| 1.0000 | 0.0000 | 0.0000 | 0.9091 | 0.0909 | 1.0297 | 0.1299 | 2.0000 | 0.5000 | 67.4267 | 0.0000 | 0.0000 | 0.0000 | 0.0909 | 0.0584 | 1.0000 | 0.0000 | 0.0325 | 1.0000 |
| 0.5584 | 0.0000 | 0.0000 | 0.0000 | 1.0000 | 3.2106 | 0.2444 | 1.3896 | 0.1948 | 74.4514 | 0.2987 | 0.0000 | 0.0000 | 0.6883 | 0.2208 | 0.9870 | 0.0000 | 0.0000 | 0.9870 |
| 0.9695 | 0.0305 | 0.0458 | 0.7710 | 0.1832 | 1.0100 | 0.1240 | 1.9690 | 0.4845 | 71.6972 | 0.0992 | 0.0000 | 0.0000 | 0.1908 | 0.0840 | 1.0000 | 0.0076 | 0.0458 | 0.2290 |
| 0.8926 | 0.0000 | 0.0248 | 0.0000 | 0.9669 | 2.8717 | 0.2202 | 1.5833 | 0.2917 | 64.5971 | 0.4628 | 0.0000 | 0.0000 | 0.5785 | 0.4298 | 0.9835 | 0.0000 | 0.0000 | 0.9752 |
| 0.9894 | 0.0035 | 0.0458 | 0.0000 | 0.9542 | 1.4401 | 0.1468 | 2.0106 | 0.5053 | 76.9689 | 0.0000 | 0.0000 | 0.0035 | 0.9965 | 0.6303 | 1.0000 | 0.3592 | 0.6514 | 1.0000 |
| 0.8006 | 0.1994 | 0.4049 | 0.0123 | 0.5828 | 0.9147 | 0.1061 | 1.5881 | 0.2940 | 71.2649 | 0.0307 | 0.0000 | 0.0000 | 0.7485 | 0.3221 | 0.9847 | 0.4080 | 0.4969 | 0.9847 |
| 0.9956 | 0.0044 | 0.3128 | 0.0176 | 0.6652 | 0.8421 | 0.0970 | 1.7523 | 0.3761 | 82.2043 | 0.0132 | 0.0000 | 0.0044 | 0.9471 | 0.4141 | 0.9956 | 0.5595 | 0.6079 | 0.9912 |
| 1.0000 | 0.0000 | 0.0922 | 0.0000 | 0.9078 | 0.8608 | 0.1021 | 2.0000 | 0.5000 | 74.0241 | 0.0034 | 0.0000 | 0.0000 | 0.9932 | 0.5973 | 0.9966 | 0.3925 | 0.6246 | 0.9966 |
| 1.0000 | 0.0000 | 0.2918 | 0.0000 | 0.7039 | 1.3444 | 0.1423 | 1.8238 | 0.4119 | 71.4690 | 0.0086 | 0.0043 | 0.0000 | 0.9528 | 0.5923 | 0.9957 | 0.3948 | 0.6180 | 0.9871 |
| 0.9934 | 0.0066 | 0.2244 | 0.0000 | 0.7756 | 1.2682 | 0.1362 | 1.9703 | 0.4851 | 81.5156 | 0.0198 | 0.0000 | 0.0000 | 0.9571 | 0.6502 | 0.9967 | 0.3267 | 0.6436 | 0.9967 |
| 0.9951 | 0.0000 | 0.2282 | 0.0049 | 0.7621 | 1.1945 | 0.1283 | 1.8535 | 0.4268 | 69.2271 | 0.0291 | 0.0049 | 0.0000 | 0.9369 | 0.5583 | 0.9903 | 0.3981 | 0.6845 | 0.9903 |
| 1.0000 | 0.0000 | 0.1613 | 0.0000 | 0.8387 | 1.2304 | 0.1305 | 1.9973 | 0.4987 | 49.2256 | 0.0108 | 0.0000 | 0.0000 | 0.9973 | 0.7070 | 1.0000 | 0.2823 | 0.7231 | 1.0000 |
| 0.9486 | 0.0286 | 0.0571 | 0.2571 | 0.6857 | 0.8397 | 0.1046 | 1.4035 | 0.2018 | 87.0185 | 0.0571 | 0.0000 | 0.0000 | 0.1371 | 0.0971 | 0.9771 | 0.0171 | 0.0629 | 0.7200 |
| 0.9852 | 0.0148 | 0.0667 | 0.0741 | 0.8556 | 0.9235 | 0.1062 | 2.1004 | 0.5502 | 85.9263 | 0.0185 | 0.0037 | 0.0000 | 0.9259 | 0.7074 | 0.9667 | 0.1963 | 0.8333 | 0.9630 |
| 1.0000 | 0.0000 | 0.0311 | 0.0035 | 0.9654 | 1.0943 | 0.1140 | 2.0464 | 0.5232 | 82.6579 | 0.0035 | 0.0000 | 0.0000 | 0.9965 | 0.7716 | 1.0000 | 0.2180 | 0.8547 | 0.9965 |
| 0.8541 | 0.1423 | 0.5018 | 0.0000 | 0.4982 | 0.6676 | 0.0869 | 1.8534 | 0.4267 | 72.2254 | 0.0000 | 0.0000 | 0.0000 | 0.9822 | 0.6228 | 0.9964 | 0.3381 | 0.6335 | 0.9964 |
| 1.0000 | 0.0000 | 0.1082 | 0.1126 | 0.6710 | 0.8269 | 0.0939 | 1.9646 | 0.4823 | 69.0887 | 0.0216 | 0.0000 | 0.0000 | 0.7403 | 0.4242 | 0.8918 | 0.3290 | 0.6234 | 0.8874 |
| 1.0000 | 0.0000 | 0.0167 | 0.0083 | 0.9750 | 0.4260 | 0.0525 | 2.0833 | 0.5417 | 67.3178 | 0.0042 | 0.0000 | 0.0000 | 0.9917 | 0.6625 | 1.0000 | 0.2875 | 0.8208 | 0.9958 |
| 0.9960 | 0.0040 | 0.0996 | 0.0000 | 0.9004 | 0.6939 | 0.0918 | 2.0782 | 0.5391 | 64.2748 | 0.0199 | 0.0000 | 0.0000 | 0.9482 | 0.7052 | 1.0000 | 0.2072 | 0.8327 | 0.9841 |
| 0.9917 | 0.0000 | 0.0248 | 0.0620 | 0.9132 | 0.4989 | 0.0550 | 1.9829 | 0.4915 | 76.6780 | 0.0041 | 0.0000 | 0.0000 | 0.9174 | 0.8678 | 1.0000 | 0.0289 | 0.8430 | 0.9339 |
| 0.9850 | 0.0000 | 0.1654 | 0.2293 | 0.5865 | 0.6067 | 0.0788 | 1.9535 | 0.4767 | 72.3082 | 0.0038 | 0.0000 | 0.0000 | 0.7105 | 0.5789 | 0.9850 | 0.1241 | 0.4925 | 0.9474 |
| 0.9840 | 0.0000 | 0.0107 | 0.2246 | 0.7647 | 1.0187 | 0.1180 | 1.9947 | 0.4973 | 77.7696 | 0.0000 | 0.0000 | 0.0000 | 0.7594 | 0.6684 | 1.0000 | 0.0695 | 0.6952 | 0.9947 |
| 0.7934 | 0.0033 | 0.2754 | 0.2098 | 0.5082 | 0.7403 | 0.0951 | 1.6612 | 0.3306 | 73.0825 | 0.1967 | 0.0000 | 0.0000 | 0.7770 | 0.2787 | 0.9934 | 0.3115 | 0.3016 | 0.9869 |
| 0.9390 | 0.0000 | 0.3659 | 0.1159 | 0.5122 | 0.4195 | 0.0549 | 1.9612 | 0.4806 | 74.1718 | 0.0000 | 0.0000 | 0.0000 | 0.7805 | 0.4146 | 0.9939 | 0.0000 | 0.4268 | 0.9695 |

| | | | | | | | | | | | | | | | | | | |
|---|---|---|---|---|---|---|---|---|---|---|---|---|---|---|---|---|---|---|
| 0.8084 | 0.1869 | 0.3879 | 0.0000 | 0.6075 | 0.9645 | 0.1125 | 1.4419 | 0.2209 | 65.3863 | 0.0327 | 0.0000 | 0.0000 | 0.9813 | 0.5140 | 0.9953 | 0.2617 | 0.4860 | 0.9907 |
| 0.9837 | 0.0130 | 0.1922 | 0.0000 | 0.8078 | 0.7243 | 0.0897 | 1.9406 | 0.4703 | 69.0412 | 0.0000 | 0.0000 | 0.0000 | 0.9479 | 0.6417 | 0.9967 | 0.3550 | 0.7590 | 0.9967 |
| 0.7614 | 0.0000 | 0.3636 | 0.0000 | 0.6326 | 0.5285 | 0.0708 | 1.5798 | 0.2899 | 74.5573 | 0.2348 | 0.0000 | 0.0000 | 0.9583 | 0.3523 | 0.9962 | 0.3258 | 0.3750 | 0.9886 |
| 0.9137 | 0.0812 | 0.3959 | 0.0000 | 0.6041 | 0.5333 | 0.0671 | 1.7582 | 0.3791 | 72.7725 | 0.0355 | 0.0000 | 0.0102 | 0.9695 | 0.4315 | 0.9949 | 0.4569 | 0.6497 | 0.9949 |
| 0.8877 | 0.1070 | 0.2674 | 0.0053 | 0.7273 | 0.3567 | 0.0471 | 1.7797 | 0.3898 | 36.7205 | 0.3583 | 0.0000 | 0.0000 | 0.6631 | 0.2299 | 0.9947 | 0.3476 | 0.3690 | 0.9733 |
| 0.8355 | 0.1645 | 0.2763 | 0.0000 | 0.7237 | 0.5057 | 0.0647 | 1.8041 | 0.4020 | 43.0503 | 0.0329 | 0.0000 | 0.0000 | 0.9934 | 0.1382 | 0.9934 | 0.7500 | 0.7697 | 0.9934 |
| 0.9712 | 0.0041 | 0.4979 | 0.0000 | 0.5021 | 0.8623 | 0.1019 | 1.6250 | 0.3125 | 55.4197 | 0.0247 | 0.0041 | 0.0000 | 0.9547 | 0.2305 | 0.9959 | 0.5802 | 0.4280 | 0.9959 |
| 0.9816 | 0.0184 | 0.3318 | 0.0000 | 0.6682 | 0.4580 | 0.0612 | 2.1338 | 0.5669 | 59.7225 | 0.0000 | 0.0000 | 0.0000 | 1.0000 | 0.2442 | 1.0000 | 0.2028 | 0.3917 | 1.0000 |
| 0.8065 | 0.1935 | 0.7806 | 0.0000 | 0.2194 | 1.7365 | 0.1664 | 1.2548 | 0.1274 | 41.6145 | 0.0000 | 0.0000 | 0.0000 | 0.9968 | 0.5839 | 0.9968 | 0.4129 | 0.4161 | 0.9968 |
| 0.9940 | 0.0000 | 0.2619 | 0.0000 | 0.7381 | 0.1665 | 0.0264 | 2.0079 | 0.5039 | 51.9176 | 0.0060 | 0.0060 | 0.0000 | 0.9583 | 0.6488 | 1.0000 | 0.0060 | 0.6071 | 0.9762 |
| 0.9226 | 0.0774 | 0.1734 | 0.0000 | 0.8266 | 0.6792 | 0.0851 | 1.9128 | 0.4564 | 70.7626 | 0.0000 | 0.0000 | 0.0000 | 1.0000 | 0.4582 | 1.0000 | 0.4768 | 0.8854 | 1.0000 |
| 0.3361 | 0.0000 | 0.0082 | 0.7213 | 0.2377 | 0.1779 | 0.0262 | 2.6364 | 0.8182 | 57.6443 | 0.0000 | 0.0000 | 0.0000 | 0.2213 | 0.2131 | 1.0000 | 0.0000 | 0.0082 | 0.7869 |
| 0.9921 | 0.0079 | 0.0236 | 0.8189 | 0.1260 | 0.9674 | 0.1215 | 2.0700 | 0.5350 | 43.6361 | 0.0394 | 0.0000 | 0.0000 | 0.0945 | 0.1024 | 0.9685 | 0.0079 | 0.0079 | 0.9370 |
| 0.9802 | 0.0198 | 0.4307 | 0.0248 | 0.5446 | 0.6456 | 0.0729 | 1.6318 | 0.3159 | 50.2760 | 0.0545 | 0.0000 | 0.0000 | 0.3614 | 0.2970 | 1.0000 | 0.0050 | 0.2871 | 0.9752 |
| 0.9960 | 0.0040 | 0.0316 | 0.0000 | 0.9684 | 0.8839 | 0.1084 | 2.1423 | 0.5711 | 58.3516 | 0.0040 | 0.0000 | 0.0000 | 0.9842 | 0.7866 | 0.9842 | 0.1937 | 0.9051 | 0.9802 |
| 1.0000 | 0.0000 | 0.0678 | 0.0113 | 0.9209 | 0.5156 | 0.0727 | 2.0462 | 0.5231 | 89.7782 | 0.0000 | 0.0000 | 0.0000 | 0.9209 | 0.6723 | 1.0000 | 0.2260 | 0.8192 | 0.9492 |
| 0.9940 | 0.0000 | 0.0299 | 0.4551 | 0.5150 | 2.8309 | 0.1634 | 1.6627 | 0.3313 | 93.3613 | 0.0898 | 0.0000 | 0.0060 | 0.1677 | 0.0240 | 0.9042 | 0.0060 | 0.0060 | 0.8144 |
| 0.9849 | 0.0151 | 0.4830 | 0.0000 | 0.5170 | 1.6697 | 0.1594 | 1.5811 | 0.2906 | 80.8528 | 0.0000 | 0.0000 | 0.0000 | 1.0000 | 0.6792 | 1.0000 | 0.3208 | 0.5170 | 1.0000 |
| 1.0000 | 0.0000 | 0.0065 | 0.4510 | 0.5163 | 2.3511 | 0.1160 | 1.9078 | 0.4539 | 81.4479 | 0.0327 | 0.0000 | 0.0000 | 0.0523 | 0.0196 | 0.9216 | 0.0065 | 0.0065 | 0.9281 |
| 1.0000 | 0.0000 | 0.0274 | 0.0046 | 0.9635 | 0.3032 | 0.0442 | 1.9907 | 0.4953 | 59.3014 | 0.0685 | 0.0000 | 0.0000 | 0.9589 | 0.6575 | 1.0000 | 0.1872 | 0.8265 | 0.9909 |
| 1.0000 | 0.0000 | 0.0562 | 0.4045 | 0.5393 | 2.9020 | 0.2029 | 1.8160 | 0.4080 | 70.4125 | 0.0037 | 0.0000 | 0.0000 | 0.5693 | 0.1273 | 1.0000 | 0.0337 | 0.1273 | 0.9963 |
| 0.9326 | 0.0674 | 0.5094 | 0.0000 | 0.4906 | 1.3889 | 0.1375 | 1.5680 | 0.2840 | 63.3758 | 0.0000 | 0.0000 | 0.0000 | 0.9892 | 0.4879 | 0.9892 | 0.3962 | 0.5553 | 0.9892 |
| 0.9917 | 0.0083 | 0.0165 | 0.5537 | 0.4298 | 0.8105 | 0.0977 | 2.0105 | 0.5053 | 76.7424 | 0.3388 | 0.0000 | 0.0083 | 0.4380 | 0.0579 | 0.9917 | 0.0413 | 0.0083 | 0.9752 |
| 1.0000 | 0.0000 | 0.1818 | 0.0000 | 0.8182 | 1.3674 | 0.1445 | 1.9091 | 0.4545 | 82.8829 | 0.0048 | 0.0000 | 0.0000 | 0.7656 | 0.6316 | 0.9617 | 0.3014 | 0.4689 | 0.9617 |
| 0.9753 | 0.0247 | 0.2099 | 0.0062 | 0.7593 | 0.9139 | 0.1055 | 1.8704 | 0.4352 | 81.0292 | 0.0309 | 0.0000 | 0.0000 | 0.9630 | 0.7346 | 1.0000 | 0.1049 | 0.7716 | 0.9506 |
| 1.0000 | 0.0000 | 0.0354 | 0.0000 | 0.9646 | 1.5499 | 0.1544 | 2.0315 | 0.5157 | 75.4923 | 0.0039 | 0.0000 | 0.0000 | 0.9961 | 0.8110 | 0.9961 | 0.1732 | 0.9291 | 0.9921 |
| 1.0000 | 0.0000 | 0.0394 | 0.0000 | 0.9606 | 0.8559 | 0.1080 | 2.0317 | 0.5159 | 90.7443 | 0.0000 | 0.0000 | 0.0000 | 0.9528 | 0.8031 | 1.0000 | 0.0787 | 0.7402 | 0.9685 |
| 1.0000 | 0.0000 | 0.0144 | 0.0000 | 0.9856 | 1.2656 | 0.1343 | 2.1444 | 0.5722 | 49.7833 | 0.0000 | 0.0000 | 0.0000 | 0.9856 | 0.9025 | 0.9856 | 0.0830 | 0.9134 | 0.9856 |
| 0.9837 | 0.0163 | 0.2967 | 0.0041 | 0.6992 | 0.8923 | 0.1009 | 2.1774 | 0.5887 | 54.2956 | 0.0122 | 0.0000 | 0.0000 | 0.9959 | 0.6504 | 1.0000 | 0.0976 | 0.6504 | 1.0000 |
| 1.0000 | 0.0000 | 0.1018 | 0.0060 | 0.8862 | 0.9679 | 0.1098 | 1.9740 | 0.4870 | 66.8088 | 0.0359 | 0.0000 | 0.0000 | 0.9401 | 0.7545 | 0.9940 | 0.1497 | 0.7066 | 0.9880 |
| 1.0000 | 0.0000 | 0.0046 | 0.0228 | 0.9726 | 0.3415 | 0.0464 | 1.9862 | 0.4931 | 64.9354 | 0.0228 | 0.0000 | 0.0046 | 0.5890 | 0.1918 | 0.9863 | 0.3699 | 0.5571 | 0.9817 |
| 1.0000 | 0.0000 | 0.1468 | 0.1009 | 0.7523 | 2.4065 | 0.1681 | 1.2661 | 0.1330 | 74.2464 | 0.0000 | 0.0000 | 0.0000 | 0.4771 | 0.1651 | 0.8532 | 0.0000 | 0.0000 | 0.8991 |
| 0.8980 | 0.1020 | 0.4898 | 0.1735 | 0.3367 | 1.5207 | 0.1346 | 1.4286 | 0.2143 | 80.0679 | 0.0000 | 0.0000 | 0.0000 | 0.0408 | 0.0816 | 0.5102 | 0.0000 | 0.0000 | 0.6429 |
| 0.9615 | 0.0000 | 0.0769 | 0.3462 | 0.5769 | 3.7484 | 0.2391 | 1.3462 | 0.1731 | 70.0382 | 0.0000 | 0.0000 | 0.0000 | 0.0769 | 0.0769 | 0.9615 | 0.0000 | 0.0000 | 0.6154 |
| 0.4857 | 0.4190 | 0.3429 | 0.1905 | 0.4667 | 1.1226 | 0.1257 | 1.9257 | 0.4629 | 41.5720 | 0.0048 | 0.0000 | 0.0000 | 0.0095 | 0.0143 | 0.6667 | 0.0048 | 0.0000 | 0.5048 |
| 0.4545 | 0.3636 | 0.3636 | 0.2273 | 0.4091 | 1.1414 | 0.1253 | 1.7727 | 0.3864 | 52.1469 | 0.0065 | 0.0000 | 0.0000 | 0.0000 | 0.0065 | 0.6364 | 0.0065 | 0.0000 | 0.5909 |
| 0.5044 | 0.4956 | 0.1150 | 0.2301 | 0.6549 | 2.1476 | 0.1833 | 2.2143 | 0.6071 | 85.9727 | 0.0000 | 0.0000 | 0.0000 | 0.0000 | 0.0000 | 0.8761 | 0.0000 | 0.0000 | 0.2743 |
| 1.0000 | 0.0000 | 0.3636 | 0.4545 | 0.1818 | 1.4427 | 0.1220 | 1.0455 | 0.0227 | 81.6447 | 0.0455 | 0.0000 | 0.0000 | 0.0000 | 0.0455 | 0.6364 | 0.0455 | 0.0000 | 0.9545 |
| 1.0000 | 0.0000 | 0.2319 | 0.3188 | 0.4493 | 2.6468 | 0.1779 | 1.0435 | 0.0217 | 83.6745 | 0.0435 | 0.0000 | 0.0000 | 0.0870 | 0.1304 | 0.7971 | 0.0435 | 0.0000 | 0.8406 |
| 1.0000 | 0.0000 | 0.7143 | 0.1587 | 0.1270 | 1.0268 | 0.0905 | 1.0328 | 0.0164 | 76.6983 | 0.0317 | 0.0000 | 0.0000 | 0.0000 | 0.0317 | 0.3333 | 0.0317 | 0.0000 | 0.9683 |
| 0.8636 | 0.1364 | 0.2545 | 0.0273 | 0.7182 | 0.9739 | 0.1102 | 1.9184 | 0.4592 | 87.0700 | 0.0000 | 0.0000 | 0.0000 | 0.0000 | 0.5455 | 0.8545 | 0.0000 | 0.0000 | 0.3182 |
| 0.7099 | 0.2901 | 0.2321 | 0.0768 | 0.6911 | 1.5013 | 0.1396 | 1.8276 | 0.4138 | 51.8740 | 0.0085 | 0.0000 | 0.0000 | 0.0512 | 0.2986 | 0.7696 | 0.0085 | 0.0000 | 0.4027 |
| 0.9444 | 0.0556 | 0.6944 | 0.1667 | 0.1389 | 0.6785 | 0.0805 | 1.2308 | 0.1154 | 74.7904 | 0.0000 | 0.0000 | 0.0000 | 0.0000 | 0.0556 | 0.5833 | 0.0000 | 0.0000 | 0.8889 |
| 1.0000 | 0.0000 | 0.4321 | 0.0000 | 0.5679 | 0.9510 | 0.0905 | 1.4444 | 0.2222 | 81.4985 | 0.1481 | 0.0000 | 0.0000 | 0.0494 | 0.4938 | 0.5802 | 0.1481 | 0.0000 | 0.5062 |
| 0.9877 | 0.0123 | 0.5185 | 0.0247 | 0.4568 | 0.5019 | 0.0593 | 1.3673 | 0.1837 | 51.4673 | 0.0988 | 0.0000 | 0.0000 | 0.2716 | 0.4198 | 0.9506 | 0.0988 | 0.0000 | 0.5679 |
| 0.5988 | 0.3951 | 0.2778 | 0.0494 | 0.6728 | 1.1820 | 0.1243 | 2.0867 | 0.5433 | 63.4657 | 0.0185 | 0.0000 | 0.0000 | 0.0123 | 0.2284 | 0.8148 | 0.0185 | 0.0000 | 0.3827 |
| 1.0000 | 0.0000 | 0.1422 | 0.6044 | 0.2533 | 1.2834 | 0.1400 | 1.7067 | 0.3533 | 70.9097 | 0.1422 | 0.0000 | 0.0000 | 0.1733 | 0.1733 | 0.9556 | 0.0000 | 0.0000 | 0.1822 |
| 1.0000 | 0.0000 | 0.3056 | 0.3704 | 0.3241 | 1.0183 | 0.1121 | 1.2037 | 0.1019 | 81.5807 | 0.1852 | 0.0000 | 0.0000 | 0.1111 | 0.0185 | 0.7037 | 0.0000 | 0.0000 | 0.6296 |
| 1.0000 | 0.0000 | 0.2240 | 0.5200 | 0.2560 | 1.8822 | 0.1624 | 1.6400 | 0.3200 | 64.7560 | 0.1280 | 0.0000 | 0.0000 | 0.1280 | 0.1280 | 0.9040 | 0.0000 | 0.0000 | 0.3600 |
| 0.9936 | 0.0000 | 0.7564 | 0.0256 | 0.2179 | 1.7176 | 0.1204 | 1.0128 | 0.0064 | 49.8892 | 0.0000 | 0.0000 | 0.0000 | 0.0000 | 0.0000 | 0.2564 | 0.0000 | 0.0000 | 0.9679 |
| 1.0000 | 0.0000 | 0.8619 | 0.0110 | 0.1271 | 0.7572 | 0.0704 | 1.0112 | 0.0056 | 57.4924 | 0.0000 | 0.0000 | 0.0000 | 0.0497 | 0.0055 | 0.1271 | 0.0000 | 0.0000 | 0.9779 |

| | | | | | | | | | | | | | | | | | | |
|---|---|---|---|---|---|---|---|---|---|---|---|---|---|---|---|---|---|---|
| 0.7647 | 0.0000 | 0.5882 | 0.2353 | 0.1765 | 1.7165 | 0.1360 | 1.0000 | 0.0000 | 82.5275 | 0.0000 | 0.0000 | 0.0000 | 0.0000 | 0.0000 | 0.4118 | 0.0000 | 0.0000 | 1.0000 |
| 0.9945 | 0.0055 | 0.8415 | 0.0000 | 0.1585 | 1.6914 | 0.1439 | 1.8415 | 0.4208 | 66.4462 | 0.0000 | 0.0000 | 0.0000 | 0.8306 | 0.8306 | 1.0000 | 0.0000 | 0.0000 | 0.9945 |
| 0.9267 | 0.0733 | 0.9267 | 0.0000 | 0.0733 | 0.7624 | 0.1043 | 2.0681 | 0.5340 | 66.6716 | 0.0000 | 0.0000 | 0.0000 | 0.9215 | 0.9215 | 0.9948 | 0.0000 | 0.0000 | 0.9267 |
| 0.9873 | 0.0000 | 0.7722 | 0.1772 | 0.0506 | 0.5300 | 0.0767 | 1.4286 | 0.2143 | 58.3414 | 0.0000 | 0.0000 | 0.0000 | 0.4177 | 0.4051 | 0.6203 | 0.0000 | 0.0000 | 0.9241 |
| 1.0000 | 0.0000 | 0.9143 | 0.0143 | 0.0714 | 0.2744 | 0.0438 | 1.0571 | 0.0286 | 89.5792 | 0.0429 | 0.0000 | 0.0000 | 0.0286 | 0.0714 | 0.0857 | 0.0429 | 0.0000 | 0.9143 |
| 1.0000 | 0.0000 | 0.8305 | 0.1356 | 0.0339 | 0.5409 | 0.0605 | 1.0000 | 0.0000 | 73.3934 | 0.0000 | 0.0000 | 0.0000 | 0.0000 | 0.0000 | 0.4068 | 0.0000 | 0.0000 | 1.0000 |
| 1.0000 | 0.0000 | 0.9781 | 0.0146 | 0.0073 | 0.3004 | 0.0475 | 1.0233 | 0.0116 | 67.4415 | 0.0073 | 0.0000 | 0.0000 | 0.0000 | 0.0073 | 0.0803 | 0.0073 | 0.0000 | 0.9781 |
| 1.0000 | 0.0000 | 0.7101 | 0.0000 | 0.2899 | 0.4550 | 0.0671 | 1.4615 | 0.2308 | 55.5368 | 0.0000 | 0.0000 | 0.0000 | 0.0000 | 0.2899 | 0.6627 | 0.0000 | 0.0000 | 0.7101 |
| 1.0000 | 0.0000 | 0.8000 | 0.1600 | 0.0400 | 0.3989 | 0.0604 | 1.0484 | 0.0242 | 57.3307 | 0.0000 | 0.0000 | 0.0000 | 0.0000 | 0.0400 | 0.1920 | 0.0000 | 0.0000 | 0.9440 |
| 1.0000 | 0.0000 | 0.8413 | 0.0847 | 0.0741 | 0.5062 | 0.0675 | 1.0741 | 0.0370 | 89.1473 | 0.0053 | 0.0000 | 0.0000 | 0.0000 | 0.0106 | 0.1746 | 0.0053 | 0.0000 | 0.9894 |
| 0.2157 | 0.7843 | 0.0784 | 0.0588 | 0.8627 | 1.5972 | 0.1628 | 2.6078 | 0.8039 | 73.1119 | 0.0000 | 0.0000 | 0.0000 | 0.0196 | 0.0196 | 0.9216 | 0.0000 | 0.0000 | 0.1569 |
| 0.2692 | 0.7308 | 0.0673 | 0.1250 | 0.8077 | 1.7285 | 0.1698 | 2.5769 | 0.7885 | 70.2707 | 0.0000 | 0.0000 | 0.0000 | 0.0000 | 0.0000 | 0.9279 | 0.0000 | 0.0000 | 0.1490 |
| 0.4191 | 0.5809 | 0.0041 | 0.3983 | 0.5975 | 1.3380 | 0.1552 | 2.5602 | 0.7801 | 69.7400 | 0.0000 | 0.0000 | 0.0000 | 0.0083 | 0.0083 | 0.9959 | 0.0000 | 0.0000 | 0.0124 |
| 0.9868 | 0.0132 | 0.5395 | 0.3421 | 0.1184 | 1.1135 | 0.1087 | 1.1579 | 0.0789 | 69.1243 | 0.0263 | 0.0000 | 0.0000 | 0.0000 | 0.0263 | 0.4737 | 0.0263 | 0.0000 | 0.8553 |
| 1.0000 | 0.0000 | 0.4375 | 0.1875 | 0.3750 | 2.5274 | 0.1746 | 1.0938 | 0.0469 | 63.3980 | 0.0938 | 0.0000 | 0.0000 | 0.0000 | 0.0938 | 0.4375 | 0.0938 | 0.0000 | 0.7188 |
| 0.9767 | 0.0000 | 0.8372 | 0.1163 | 0.0465 | 0.8523 | 0.0938 | 1.0930 | 0.0465 | 51.5715 | 0.0000 | 0.0000 | 0.0000 | 0.0000 | 0.0000 | 0.1628 | 0.0000 | 0.0000 | 0.9070 |
| 0.3162 | 0.6838 | 0.2051 | 0.0513 | 0.7436 | 1.1310 | 0.1331 | 2.3675 | 0.6838 | 70.6259 | 0.0000 | 0.0000 | 0.0000 | 0.0513 | 0.0513 | 0.8974 | 0.0000 | 0.0000 | 0.2650 |
| 0.4051 | 0.5949 | 0.3641 | 0.0410 | 0.5949 | 0.9502 | 0.1154 | 2.2308 | 0.6154 | 71.8167 | 0.0000 | 0.0000 | 0.0000 | 0.0000 | 0.0000 | 0.6359 | 0.0000 | 0.0000 | 0.3641 |
| 1.0000 | 0.0000 | 0.2547 | 0.0755 | 0.6698 | 0.6770 | 0.0932 | 1.7358 | 0.3679 | 55.2251 | 0.0000 | 0.0000 | 0.0000 | 0.0094 | 0.6698 | 0.7736 | 0.0000 | 0.0000 | 0.2547 |
| 0.5503 | 0.4497 | 0.4615 | 0.0000 | 0.5385 | 0.7656 | 0.0960 | 2.4486 | 0.7243 | 94.2148 | 0.0059 | 0.0000 | 0.0000 | 0.0947 | 0.1006 | 0.9053 | 0.0059 | 0.0000 | 0.4615 |
| 0.6387 | 0.2452 | 0.2710 | 0.3613 | 0.3677 | 0.7261 | 0.0965 | 1.5097 | 0.2548 | 86.1018 | 0.0194 | 0.0000 | 0.0000 | 0.1032 | 0.1226 | 0.7290 | 0.0194 | 0.0000 | 0.6323 |
| 0.8146 | 0.1854 | 0.6623 | 0.0132 | 0.3245 | 0.5143 | 0.0706 | 1.3960 | 0.1980 | 79.1221 | 0.0066 | 0.0000 | 0.0000 | 0.1325 | 0.1391 | 0.3510 | 0.0066 | 0.0000 | 0.6623 |
| 0.9692 | 0.0308 | 0.5385 | 0.0000 | 0.4615 | 0.3572 | 0.0543 | 1.1613 | 0.0806 | 90.6405 | 0.0923 | 0.0000 | 0.0000 | 0.3385 | 0.4308 | 0.6000 | 0.0923 | 0.0000 | 0.5385 |
| 0.7321 | 0.2679 | 0.5000 | 0.0357 | 0.4643 | 0.6259 | 0.0830 | 1.6111 | 0.3056 | 77.6809 | 0.0536 | 0.0000 | 0.0000 | 0.1429 | 0.1964 | 0.5893 | 0.0536 | 0.0000 | 0.5357 |
| 0.3067 | 0.6933 | 0.2400 | 0.0267 | 0.7333 | 1.0562 | 0.1273 | 2.7377 | 0.8689 | 73.7384 | 0.0000 | 0.0000 | 0.0000 | 0.0133 | 0.0400 | 0.9467 | 0.0000 | 0.0000 | 0.2667 |
| 0.5702 | 0.4298 | 0.1818 | 0.0826 | 0.7355 | 1.0996 | 0.1333 | 2.3571 | 0.6786 | 64.4832 | 0.0000 | 0.0000 | 0.0000 | 0.0000 | 0.3058 | 0.5620 | 0.0000 | 0.0000 | 0.1818 |
| 0.7162 | 0.2838 | 0.1824 | 0.0541 | 0.7635 | 1.0024 | 0.1231 | 2.2432 | 0.6216 | 62.4672 | 0.0000 | 0.0000 | 0.0000 | 0.0068 | 0.4797 | 0.4054 | 0.0000 | 0.0000 | 0.1824 |
| 0.4464 | 0.5494 | 0.2790 | 0.0043 | 0.7167 | 1.0202 | 0.1226 | 2.1034 | 0.5517 | 84.4046 | 0.0000 | 0.0000 | 0.0000 | 0.1545 | 0.1588 | 0.7210 | 0.0000 | 0.0000 | 0.2918 |
| 0.8013 | 0.1987 | 0.1258 | 0.0000 | 0.8742 | 0.8866 | 0.1143 | 2.1211 | 0.5605 | 78.5899 | 0.0000 | 0.0000 | 0.0000 | 0.0298 | 0.6755 | 0.5364 | 0.0000 | 0.0000 | 0.1258 |
| 0.7606 | 0.2394 | 0.6092 | 0.0000 | 0.3908 | 0.8041 | 0.0957 | 1.5574 | 0.2787 | 60.1999 | 0.0000 | 0.0000 | 0.0000 | 0.0070 | 0.1338 | 0.2887 | 0.0000 | 0.0000 | 0.6268 |
| 1.0000 | 0.0000 | 0.5625 | 0.0250 | 0.4125 | 0.6992 | 0.0796 | 1.0714 | 0.0357 | 53.7571 | 0.0000 | 0.0000 | 0.0000 | 0.0688 | 0.3875 | 0.2375 | 0.0000 | 0.0000 | 0.5875 |
| 0.7595 | 0.2405 | 0.5823 | 0.1709 | 0.2468 | 0.7730 | 0.0951 | 1.5127 | 0.2563 | 70.7212 | 0.0063 | 0.0000 | 0.0000 | 0.0000 | 0.0063 | 0.2785 | 0.0063 | 0.0000 | 0.5886 |
| 1.0000 | 0.0000 | 0.8448 | 0.1207 | 0.0345 | 0.3483 | 0.0515 | 1.1207 | 0.0603 | 87.8120 | 0.0000 | 0.0000 | 0.0000 | 0.0345 | 0.0345 | 0.1897 | 0.0000 | 0.0000 | 0.8448 |
| 0.6863 | 0.3137 | 0.4510 | 0.0000 | 0.5490 | 0.6868 | 0.0898 | 1.7059 | 0.3529 | 74.3249 | 0.0784 | 0.0000 | 0.0000 | 0.1569 | 0.2353 | 0.6863 | 0.0784 | 0.0000 | 0.4510 |
| 0.9000 | 0.1000 | 0.7286 | 0.1571 | 0.1143 | 0.4600 | 0.0655 | 1.4722 | 0.2361 | 69.8083 | 0.0143 | 0.0000 | 0.0000 | 0.0286 | 0.0429 | 0.7857 | 0.0143 | 0.0000 | 0.8857 |
| 0.7419 | 0.2581 | 0.6371 | 0.0968 | 0.2661 | 0.7815 | 0.1021 | 1.7398 | 0.3699 | 46.1378 | 0.0081 | 0.0000 | 0.0000 | 0.1129 | 0.1210 | 0.5484 | 0.0081 | 0.0000 | 0.6371 |
| 0.8970 | 0.1030 | 0.5408 | 0.0815 | 0.3777 | 0.5823 | 0.0808 | 1.6871 | 0.3436 | 64.4461 | 0.0000 | 0.0000 | 0.0000 | 0.0000 | 0.2747 | 0.7597 | 0.0000 | 0.0000 | 0.6223 |
| 0.7377 | 0.2623 | 0.6995 | 0.0000 | 0.3005 | 0.7388 | 0.0980 | 1.7377 | 0.3689 | 42.8155 | 0.0273 | 0.0000 | 0.0000 | 0.1749 | 0.2131 | 0.4754 | 0.0273 | 0.0000 | 0.6995 |
| 1.0000 | 0.0000 | 0.3548 | 0.0968 | 0.5484 | 0.6761 | 0.0943 | 1.5806 | 0.2903 | 80.6027 | 0.0000 | 0.0000 | 0.0000 | 0.0323 | 0.5484 | 0.6774 | 0.0000 | 0.0000 | 0.3871 |
| 0.6094 | 0.3906 | 0.1615 | 0.0260 | 0.8125 | 1.0538 | 0.1272 | 2.3839 | 0.6920 | 81.1629 | 0.0000 | 0.0000 | 0.0000 | 0.0052 | 0.4219 | 0.5781 | 0.0000 | 0.0000 | 0.1615 |
| 0.8633 | 0.1367 | 0.3359 | 0.0977 | 0.5664 | 0.7918 | 0.0992 | 1.5548 | 0.2774 | 59.9634 | 0.0000 | 0.0000 | 0.0000 | 0.0039 | 0.4297 | 0.4141 | 0.0000 | 0.0000 | 0.3359 |
| 0.7647 | 0.2353 | 0.5765 | 0.0000 | 0.4235 | 0.6214 | 0.0842 | 1.4824 | 0.2412 | 70.3948 | 0.0000 | 0.0000 | 0.0000 | 0.2000 | 0.1882 | 0.7176 | 0.0000 | 0.0000 | 0.5765 |
| 0.8901 | 0.1099 | 0.8901 | 0.0000 | 0.1099 | 0.7849 | 0.1064 | 2.0989 | 0.5495 | 50.7006 | 0.0000 | 0.0000 | 0.0000 | 0.8791 | 0.8791 | 0.9890 | 0.0000 | 0.0000 | 0.8901 |
| 0.9194 | 0.0806 | 0.8871 | 0.0161 | 0.0968 | 0.6570 | 0.0915 | 1.6613 | 0.3306 | 55.7764 | 0.0000 | 0.0000 | 0.0000 | 0.5000 | 0.5000 | 0.6774 | 0.0000 | 0.0000 | 0.8871 |
| 0.9664 | 0.0336 | 0.0504 | 0.0084 | 0.9412 | 0.6606 | 0.0872 | 1.4211 | 0.2105 | 69.1574 | 0.0000 | 0.0000 | 0.0000 | 0.0672 | 0.9076 | 0.1513 | 0.0000 | 0.0000 | 0.0504 |
| 1.0000 | 0.0000 | 0.0147 | 0.0000 | 0.9853 | 0.3981 | 0.0617 | 1.2000 | 0.1000 | 51.8164 | 0.0000 | 0.0000 | 0.0000 | 0.0524 | 0.9853 | 0.0566 | 0.0000 | 0.0000 | 0.0147 |
| 0.8682 | 0.1318 | 0.1236 | 0.0000 | 0.8764 | 0.8427 | 0.1050 | 2.0828 | 0.5414 | 86.2969 | 0.0000 | 0.0000 | 0.0000 | 0.0198 | 0.7446 | 0.2504 | 0.0000 | 0.0000 | 0.1236 |
| 0.1713 | 0.8287 | 0.0884 | 0.0000 | 0.9116 | 1.5673 | 0.1602 | 2.6944 | 0.8472 | 80.2425 | 0.0276 | 0.0000 | 0.0000 | 0.0000 | 0.0276 | 0.9171 | 0.0276 | 0.0000 | 0.1436 |
| 0.9924 | 0.0076 | 0.9470 | 0.0000 | 0.0530 | 0.7617 | 0.1019 | 1.8939 | 0.4470 | 83.6048 | 0.0076 | 0.0000 | 0.0000 | 0.8333 | 0.8409 | 0.8864 | 0.0076 | 0.0000 | 0.9848 |
| 1.0000 | 0.0000 | 0.1831 | 0.0114 | 0.8055 | 0.3366 | 0.0507 | 1.5983 | 0.2991 | 43.7381 | 0.0000 | 0.0000 | 0.0000 | 0.1602 | 0.7323 | 0.2563 | 0.0000 | 0.0000 | 0.2517 |
| 1.0000 | 0.0000 | 0.1442 | 0.0069 | 0.8488 | 0.3491 | 0.0524 | 1.0579 | 0.0289 | 89.6372 | 0.0000 | 0.0000 | 0.0000 | 0.0166 | 0.8460 | 0.1581 | 0.0000 | 0.0000 | 0.1512 |

| | | | | | | | | | | | | | | | | | | |
|---|---|---|---|---|---|---|---|---|---|---|---|---|---|---|---|---|---|---|
| 0.7211 | 0.2789 | 0.0070 | 0.0000 | 0.9930 | 0.4885 | 0.0603 | 2.2716 | 0.6358 | 64.1103 | 0.0000 | 0.0000 | 0.0000 | 0.5160 | 0.6276 | 0.8884 | 0.0000 | 0.0000 | 0.5788 |
| 0.9218 | 0.0782 | 0.0469 | 0.0156 | 0.9374 | 0.3393 | 0.0380 | 2.0172 | 0.5086 | 62.6488 | 0.0031 | 0.0000 | 0.0000 | 0.7887 | 0.7856 | 0.9531 | 0.0000 | 0.0000 | 0.9030 |
| 0.9808 | 0.0192 | 0.8621 | 0.0958 | 0.0421 | 0.7637 | 0.1042 | 2.0345 | 0.5172 | 73.2951 | 0.0000 | 0.0000 | 0.0000 | 0.8659 | 0.8812 | 1.0000 | 0.0000 | 0.0000 | 0.8851 |
| 0.9111 | 0.0889 | 0.1185 | 0.0222 | 0.8593 | 0.5825 | 0.0615 | 2.1818 | 0.5909 | 83.8841 | 0.0000 | 0.0000 | 0.0000 | 0.3259 | 0.6148 | 0.8667 | 0.0000 | 0.0000 | 0.8593 |
| 1.0000 | 0.0000 | 0.0033 | 0.0000 | 0.9967 | 0.1157 | 0.0191 | 2.2931 | 0.6466 | 72.4324 | 0.0000 | 0.0000 | 0.0000 | 0.4508 | 0.7546 | 0.9933 | 0.0000 | 0.0000 | 0.9850 |
| 1.0000 | 0.0000 | 0.0071 | 0.2847 | 0.7082 | 0.5172 | 0.0663 | 2.0588 | 0.5294 | 95.9300 | 0.0534 | 0.0000 | 0.0000 | 0.3523 | 0.3701 | 0.7331 | 0.0000 | 0.0000 | 0.4164 |
| 0.9005 | 0.0995 | 0.0373 | 0.0050 | 0.9577 | 0.5140 | 0.0500 | 2.1567 | 0.5784 | NA | 0.0249 | 0.0000 | 0.0000 | 0.6592 | 0.8209 | 1.0000 | 0.0000 | 0.0000 | 0.8632 |
| 1.0000 | 0.0000 | 0.5373 | 0.0000 | 0.4627 | 0.7609 | 0.0818 | 2.2652 | 0.6326 | 45.7376 | 0.0173 | 0.0000 | 0.0000 | 0.5927 | 0.8925 | 1.0000 | 0.0000 | 0.0000 | 1.0000 |
| 0.8077 | 0.0000 | 0.0000 | 0.3846 | 0.5385 | 0.4885 | 0.0719 | 1.0000 | 0.0000 | 79.1730 | 0.0000 | 0.0000 | 0.0000 | 0.5385 | 0.5385 | 0.8462 | 0.0000 | 0.0000 | 0.2692 |
| 0.2653 | 0.7347 | 0.0136 | 0.0544 | 0.9320 | 1.1741 | 0.1381 | 2.4966 | 0.7483 | NA | 0.0000 | 0.0000 | 0.0000 | 0.1769 | 0.1769 | 0.9864 | 0.0000 | 0.0000 | 0.1020 |
| 0.8713 | 0.1089 | 0.5842 | 0.0594 | 0.3564 | 0.5277 | 0.0687 | 1.2784 | 0.1392 | 68.6115 | 0.0000 | 0.0000 | 0.0000 | 0.2475 | 0.2772 | 0.4653 | 0.0000 | 0.0000 | 0.6535 |
| 0.5341 | 0.4659 | 0.5141 | 0.0201 | 0.4659 | 1.0238 | 0.1277 | 2.4378 | 0.7189 | 64.0458 | 0.0000 | 0.0000 | 0.0000 | 0.5060 | 0.4819 | 0.9799 | 0.0000 | 0.0000 | 0.5181 |
| 0.7705 | 0.2295 | 0.0738 | 0.0082 | 0.9180 | 3.8497 | 0.2500 | 1.8000 | 0.4000 | 47.7794 | 0.0000 | 0.0000 | 0.0000 | 0.0000 | 0.4262 | 0.5410 | 0.0000 | 0.0000 | 0.3443 |
| 0.8644 | 0.1356 | 0.2712 | 0.4746 | 0.2542 | 1.2510 | 0.1404 | 1.5566 | 0.2783 | 86.9206 | 0.0000 | 0.0000 | 0.0000 | 0.2458 | 0.1186 | 0.3898 | 0.0000 | 0.0000 | 0.2712 |
| 0.6141 | 0.3804 | 0.2609 | 0.0054 | 0.7283 | 0.7612 | 0.0981 | 1.7705 | 0.3852 | 50.0000 | 0.0000 | 0.0000 | 0.0000 | 0.3478 | 0.3478 | 0.9946 | 0.0000 | 0.0000 | 0.2609 |
| 1.0000 | 0.0000 | 0.7372 | 0.0000 | 0.2628 | 2.0612 | 0.1454 | 1.0000 | 0.0000 | 85.7538 | 0.0000 | 0.0000 | 0.0000 | 0.0128 | 0.0128 | 0.3974 | 0.0000 | 0.0000 | 0.9872 |
| 0.9934 | 0.0000 | 0.2914 | 0.6887 | 0.0132 | 1.0087 | 0.1199 | 1.7200 | 0.3600 | 67.1752 | 0.0000 | 0.0000 | 0.0000 | 0.0265 | 0.0000 | 0.9934 | 0.0000 | 0.0000 | 0.3046 |
| NA | NA | NA | NA | NA | NA | NA | NA | NA | 76.6786 | NA | NA | NA | NA | NA | NA | NA | NA | NA |
| 1.0000 | 0.0000 | 0.3297 | 0.6593 | 0.0110 | 1.3392 | 0.1430 | 1.0879 | 0.0440 | 89.0909 | 0.0000 | 0.0000 | 0.0000 | 0.0879 | 0.0000 | 0.0989 | 0.0000 | 0.0000 | 0.3407 |
| 1.0000 | 0.0000 | 0.9758 | 0.0193 | 0.0048 | 0.3117 | 0.0471 | 1.8792 | 0.4396 | 74.4360 | 0.0000 | 0.0000 | 0.0000 | 0.8792 | 0.0000 | 1.0000 | 0.0000 | 0.0000 | 1.0000 |
| 0.8889 | 0.0000 | 0.4444 | 0.4444 | 0.0000 | 0.4690 | 0.0696 | 1.5000 | 0.2500 | 52.6045 | 0.0000 | 0.0000 | 0.0000 | 0.4444 | 0.0000 | 0.8889 | 0.0000 | 0.0000 | 0.8889 |
| 0.0000 | 0.0000 | 0.0000 | 0.0000 | 0.0000 | NA | NA | NA | NA | 86.7023 | 0.0000 | 0.0000 | 0.0000 | 0.0000 | 0.0000 | 0.0000 | 0.0000 | 0.0000 | 0.0000 |
| NA | NA | NA | NA | NA | NA | NA | NA | NA | 72.0140 | NA | NA | NA | NA | NA | NA | NA | NA | NA |
| 0.9646 | 0.0000 | 0.0442 | 0.0000 | 0.9204 | 0.9914 | 0.0916 | 1.0000 | 0.0000 | 64.4488 | 0.0000 | 0.0000 | 0.0000 | 0.1416 | 0.9027 | 0.2035 | 0.0000 | 0.0000 | 0.0619 |
| 0.9697 | 0.0303 | 0.0000 | 0.0000 | 1.0000 | 1.3692 | 0.1599 | 3.0000 | 1.0000 | 69.2689 | 0.0000 | 0.0000 | 0.0000 | 0.0000 | 0.9697 | 0.0303 | 0.0000 | 0.0000 | 0.0000 |
| 0.6486 | 0.3514 | 0.5676 | 0.0811 | 0.3514 | 0.7415 | 0.0961 | 1.7879 | 0.3939 | 60.6640 | 0.0000 | 0.0000 | 0.0000 | 0.0000 | 0.0000 | 0.7027 | 0.0000 | 0.0000 | 0.6486 |
| NA | NA | NA | NA | NA | NA | NA | NA | NA | 71.3064 | NA | NA | NA | NA | NA | NA | NA | NA | NA |
| 0.0000 | 0.0000 | 0.0000 | 0.0000 | 0.0000 | NA | NA | NA | NA | 79.2885 | 0.0000 | 0.0000 | 0.0000 | 0.0000 | 0.0000 | 0.0000 | 0.0000 | 0.0000 | 0.0000 |
| 0.9962 | 0.0038 | 0.0417 | 0.0000 | 0.9583 | 0.8736 | 0.0817 | 1.9286 | 0.4643 | 60.7100 | 0.0000 | 0.0000 | 0.0000 | 0.0379 | 0.9508 | 0.0530 | 0.0000 | 0.0000 | 0.0455 |
| 0.9815 | 0.0185 | 0.2066 | 0.0185 | 0.7749 | 0.8651 | 0.0814 | 1.3521 | 0.1761 | 49.1283 | 0.0000 | 0.0000 | 0.0000 | 0.0554 | 0.7380 | 0.2583 | 0.0000 | 0.0000 | 0.2435 |
| 1.0000 | 0.0000 | 0.9213 | 0.0787 | 0.0000 | 0.3783 | 0.0556 | 1.0000 | 0.0000 | 67.9469 | 0.0000 | 0.0000 | 0.0000 | 0.0000 | 0.0000 | 0.9213 | 0.0000 | 0.0000 | 0.9438 |
| 1.0000 | 0.0000 | 0.7333 | 0.1667 | 0.1000 | 0.4817 | 0.0724 | 1.0000 | 0.0000 | 58.1869 | 0.0000 | 0.0000 | 0.0000 | 0.1000 | 0.1000 | 0.3333 | 0.0000 | 0.0000 | 0.9000 |
| 1.0000 | 0.0000 | 0.0000 | 0.0000 | 1.0000 | 0.4500 | 0.0689 | 1.0000 | 0.0000 | 62.2654 | 0.0000 | 0.0000 | 0.0000 | 1.0000 | 1.0000 | 1.0000 | 0.0000 | 0.0000 | 0.0000 |
| 0.9886 | 0.0114 | 0.9091 | 0.0114 | 0.0795 | 0.3072 | 0.0490 | 1.0230 | 0.0115 | 79.1089 | 0.0000 | 0.0000 | 0.0000 | 0.0568 | 0.0682 | 0.9886 | 0.0000 | 0.0000 | 0.9205 |
| 0.6447 | 0.3553 | 0.4569 | 0.0102 | 0.5330 | 0.7006 | 0.0906 | 1.9733 | 0.4866 | 79.4286 | 0.0000 | 0.0000 | 0.0000 | 0.3807 | 0.1777 | 1.0000 | 0.0000 | 0.0000 | 0.4569 |
| 0.9451 | 0.0549 | 0.0110 | 0.1099 | 0.8791 | 1.2091 | 0.1412 | 1.6667 | 0.3333 | 76.7454 | 0.0000 | 0.0000 | 0.0000 | 0.0000 | 0.8242 | 0.1209 | 0.0000 | 0.0000 | 0.0659 |
| 1.0000 | 0.0000 | 0.5455 | 0.0758 | 0.3788 | 0.4752 | 0.0719 | 1.0164 | 0.0082 | 64.9194 | 0.0000 | 0.0000 | 0.0000 | 0.3182 | 0.3788 | 0.3939 | 0.0000 | 0.0000 | 0.6212 |
| 1.0000 | 0.0000 | 0.2500 | 0.3125 | 0.4375 | 2.2897 | 0.1644 | 1.6875 | 0.3438 | 50.9069 | 0.0000 | 0.0000 | 0.0000 | 0.0000 | 0.1875 | 0.7500 | 0.0000 | 0.0000 | 0.6875 |
| 1.0000 | 0.0000 | 0.1111 | 0.1667 | 0.7222 | 1.1853 | 0.1323 | 1.2000 | 0.1000 | 71.0034 | 0.0000 | 0.0000 | 0.0000 | 0.0556 | 0.7222 | 0.1111 | 0.0000 | 0.0000 | 0.1111 |
| 0.9412 | 0.0588 | 0.2647 | 0.0000 | 0.7353 | 0.4080 | 0.0577 | 2.1786 | 0.5893 | 79.5823 | 0.0000 | 0.0000 | 0.0000 | 0.2353 | 0.6765 | 0.4118 | 0.0000 | 0.0000 | 0.3382 |
| 0.9917 | 0.0083 | 0.0000 | 0.0000 | 1.0000 | 0.2345 | 0.0338 | 3.0000 | 1.0000 | 79.4128 | 0.0000 | 0.0000 | 0.0000 | 0.0000 | 0.9917 | 0.0909 | 0.0000 | 0.0000 | 0.0826 |
| 0.9505 | 0.0495 | 0.1832 | 0.0000 | 0.8168 | 0.4756 | 0.0655 | 1.6863 | 0.3431 | 75.8058 | 0.0000 | 0.0000 | 0.0000 | 0.0248 | 0.7673 | 0.2525 | 0.0000 | 0.0000 | 0.2079 |
| 0.9744 | 0.0256 | 0.7949 | 0.0000 | 0.2051 | 0.2976 | 0.0467 | 1.4545 | 0.2273 | 56.5381 | 0.0000 | 0.0000 | 0.0000 | 0.0385 | 0.1795 | 0.8974 | 0.0000 | 0.0000 | 0.9744 |
| 1.0000 | 0.0000 | 0.0500 | 0.2000 | 0.7500 | 3.2480 | 0.2197 | 1.3000 | 0.1500 | 65.9481 | 0.0000 | 0.0000 | 0.0000 | 0.0000 | 0.5750 | 0.3000 | 0.0000 | 0.0000 | 0.3000 |
| 1.0000 | 0.0000 | 0.0222 | 0.0000 | 0.9778 | 0.1370 | 0.0236 | 2.8000 | 0.9000 | 54.8220 | 0.0000 | 0.0000 | 0.0000 | 0.0000 | 0.9778 | 0.2222 | 0.0000 | 0.0000 | 0.2222 |
| 1.0000 | 0.0000 | 0.0074 | 0.0148 | 0.9778 | 0.3466 | 0.0445 | 2.6494 | 0.8247 | 82.0932 | 0.0000 | 0.0000 | 0.0000 | 0.0074 | 0.9749 | 0.0990 | 0.0000 | 0.0000 | 0.0990 |
| 1.0000 | 0.0000 | 0.0121 | 0.0000 | 0.9879 | 0.2834 | 0.0451 | 1.8148 | 0.4074 | 82.4712 | 0.0000 | 0.0000 | 0.0000 | 0.0208 | 0.9879 | 0.0468 | 0.0000 | 0.0000 | 0.0295 |
| 1.0000 | 0.0000 | 0.1667 | 0.0000 | 0.8333 | 0.4218 | 0.0650 | 1.0000 | 0.0000 | 81.3720 | 0.0000 | 0.0000 | 0.0000 | 0.8333 | 0.8333 | 1.0000 | 0.0000 | 0.0000 | 0.1667 |
| 0.8947 | 0.0842 | 0.0947 | 0.0105 | 0.8842 | 0.4103 | 0.0603 | 2.5106 | 0.7553 | 81.9607 | 0.1895 | 0.0000 | 0.0000 | 0.9158 | 0.7158 | 0.9895 | 0.0000 | 0.6316 | 0.9789 |
| 0.9701 | 0.0000 | 0.1343 | 0.0000 | 0.8507 | 0.2605 | 0.0359 | 2.0303 | 0.5152 | 61.6188 | 0.0448 | 0.0000 | 0.0000 | 0.9552 | 0.9403 | 0.9851 | 0.0000 | 0.1194 | 0.9851 |
| 0.9792 | 0.0000 | 0.2813 | 0.0104 | 0.6979 | 0.3356 | 0.0464 | 2.0105 | 0.5053 | 74.9018 | 0.0625 | 0.0000 | 0.0000 | 0.8750 | 0.8750 | 0.9792 | 0.0000 | 0.0208 | 0.9792 |

| | | | | | | | | | | | | | | | | | | |
|---|---|---|---|---|---|---|---|---|---|---|---|---|---|---|---|---|---|---|
| 0.9521 | 0.0000 | 0.1018 | 0.0060 | 0.8922 | 0.2875 | 0.0352 | 2.1145 | 0.5572 | 75.9739 | 0.1257 | 0.0000 | 0.0000 | 0.2395 | 0.2994 | 0.9940 | 0.0000 | 0.0299 | 0.9880 |
| 0.6667 | 0.0556 | 0.0988 | 0.0309 | 0.8395 | 0.3139 | 0.0396 | 2.0375 | 0.5188 | 78.4640 | 0.4815 | 0.0000 | 0.0000 | 0.5926 | 0.4074 | 0.9877 | 0.0123 | 0.0617 | 0.9815 |
| 0.9237 | 0.0076 | 0.0687 | 0.0382 | 0.8626 | 0.5460 | 0.0673 | 2.0476 | 0.5238 | 79.8213 | 0.1069 | 0.0000 | 0.0000 | 0.6641 | 0.6336 | 0.9542 | 0.0153 | 0.0305 | 0.9466 |
| 0.9270 | 0.0300 | 0.0558 | 0.0129 | 0.9227 | 0.4922 | 0.0661 | 2.0175 | 0.5087 | 77.4999 | 0.0815 | 0.0000 | 0.0000 | 0.1459 | 0.1245 | 0.9828 | 0.0129 | 0.0515 | 0.9700 |
| 0.8990 | 0.0505 | 0.1010 | 0.0101 | 0.8586 | 0.3697 | 0.0517 | 2.4105 | 0.7053 | 78.0750 | 0.1919 | 0.0000 | 0.0000 | 0.7879 | 0.6768 | 0.9697 | 0.0101 | 0.4949 | 0.9293 |
| 0.9874 | 0.0042 | 0.1130 | 0.0042 | 0.8745 | 0.4728 | 0.0697 | 2.0211 | 0.5105 | 74.6826 | 0.0377 | 0.0000 | 0.0000 | 0.2050 | 0.1925 | 0.9916 | 0.0000 | 0.0209 | 0.9791 |
| 0.9487 | 0.0000 | 0.2137 | 0.0342 | 0.7308 | 0.6592 | 0.0800 | 2.1718 | 0.5859 | 79.5643 | 0.1111 | 0.0000 | 0.0043 | 0.3504 | 0.4359 | 0.9744 | 0.0043 | 0.1154 | 0.9701 |
| 0.7940 | 0.0050 | 0.1256 | 0.0201 | 0.8543 | 0.3857 | 0.0460 | 2.2261 | 0.6131 | 81.3526 | 0.4271 | 0.0000 | 0.0000 | 0.8090 | 0.7538 | 1.0000 | 0.0000 | 0.1256 | 0.9849 |
| 0.9146 | 0.0000 | 0.4724 | 0.0050 | 0.4975 | 0.5095 | 0.0670 | 2.2902 | 0.6451 | 78.1354 | 0.1055 | 0.0000 | 0.0000 | 0.6181 | 0.8492 | 0.9648 | 0.0050 | 0.0854 | 0.9598 |
| 0.8879 | 0.0000 | 0.3017 | 0.0043 | 0.6724 | 0.3750 | 0.0496 | 2.2876 | 0.6438 | 80.6660 | 0.1767 | 0.0000 | 0.0000 | 0.4957 | 0.7241 | 0.9741 | 0.0000 | 0.0172 | 0.9698 |
| 0.9492 | 0.0085 | 0.1017 | 0.0085 | 0.8729 | 0.4591 | 0.0571 | 2.1130 | 0.5565 | 75.7018 | 0.3983 | 0.0000 | 0.0000 | 0.6186 | 0.3051 | 0.9661 | 0.0169 | 0.1610 | 0.9576 |
| 0.9578 | 0.0060 | 0.5000 | 0.0060 | 0.4639 | 0.4767 | 0.0662 | 2.0250 | 0.5125 | 79.4676 | 0.0301 | 0.0000 | 0.0000 | 0.8916 | 0.8735 | 0.9639 | 0.0000 | 0.0301 | 0.9578 |
| 0.7358 | 0.0063 | 0.1761 | 0.0063 | 0.8050 | 0.2836 | 0.0395 | 2.0127 | 0.5064 | 74.0130 | 0.2704 | 0.0000 | 0.0000 | 0.5786 | 0.5597 | 0.9874 | 0.0000 | 0.0063 | 0.9748 |
| 0.9664 | 0.0000 | 0.0462 | 0.0168 | 0.9370 | 0.2674 | 0.0362 | 2.4478 | 0.7239 | 79.7437 | 0.0882 | 0.0000 | 0.0042 | 0.2773 | 0.5210 | 0.9664 | 0.0084 | 0.1765 | 0.9622 |
| 0.9811 | 0.0094 | 0.6934 | 0.0047 | 0.2925 | 0.5805 | 0.0791 | 2.0625 | 0.5313 | 76.2922 | 0.0377 | 0.0000 | 0.0000 | 0.9104 | 0.9434 | 0.9858 | 0.0047 | 0.0236 | 0.9717 |
| 0.9792 | 0.0052 | 0.8542 | 0.0052 | 0.1354 | 0.7025 | 0.0953 | 2.0476 | 0.5238 | 77.0777 | 0.0417 | 0.0000 | 0.0000 | 0.9115 | 0.9323 | 0.9896 | 0.0000 | 0.0052 | 0.9844 |
| 0.9860 | 0.0000 | 0.5748 | 0.0047 | 0.3738 | 0.4931 | 0.0687 | 2.0147 | 0.5074 | 57.6084 | 0.0607 | 0.0000 | 0.0000 | 0.9346 | 0.8879 | 0.9533 | 0.0000 | 0.0140 | 0.9486 |
| 0.9624 | 0.0188 | 0.8638 | 0.0047 | 0.1268 | 0.6824 | 0.0939 | 2.0237 | 0.5118 | 81.7628 | 0.0188 | 0.0000 | 0.0000 | 0.9437 | 0.9437 | 0.9859 | 0.0000 | 0.0047 | 0.9671 |
| 0.9563 | 0.0219 | 0.6776 | 0.0109 | 0.3060 | 1.0108 | 0.1196 | 1.6796 | 0.3398 | 83.4744 | 0.0273 | 0.0000 | 0.0000 | 0.5628 | 0.5792 | 0.9891 | 0.0000 | 0.0055 | 0.9617 |
| 0.9174 | 0.0046 | 0.1514 | 0.0092 | 0.8303 | 0.3667 | 0.0428 | 2.1343 | 0.5671 | 79.2208 | 0.0963 | 0.0000 | 0.0000 | 0.2110 | 0.3624 | 0.9862 | 0.0046 | 0.0459 | 0.9771 |
| 0.9710 | 0.0072 | 0.3261 | 0.0000 | 0.6667 | 0.3488 | 0.0475 | 1.9927 | 0.4964 | 66.1034 | 0.0435 | 0.0000 | 0.0000 | 0.9493 | 0.9203 | 0.9855 | 0.0072 | 0.0145 | 0.9855 |
| 0.9626 | 0.0093 | 0.8131 | 0.0000 | 0.1776 | 0.5904 | 0.0820 | 2.0476 | 0.5238 | 70.5555 | 0.0467 | 0.0000 | 0.0000 | 0.9439 | 0.9533 | 0.9626 | 0.0000 | 0.0000 | 0.9720 |
| 0.7215 | 0.2532 | 0.5380 | 0.0063 | 0.4494 | 0.7816 | 0.0999 | 2.2628 | 0.6314 | 62.8724 | 0.0190 | 0.0000 | 0.0000 | 0.7089 | 0.7152 | 0.9873 | 0.0000 | 0.0063 | 0.7342 |
| 0.9467 | 0.0333 | 0.5533 | 0.0133 | 0.4200 | 0.5513 | 0.0742 | 2.0338 | 0.5169 | 64.1687 | 0.0400 | 0.0000 | 0.0000 | 0.8733 | 0.8800 | 0.9867 | 0.0000 | 0.0067 | 0.9533 |
| 0.9752 | 0.0062 | 0.8944 | 0.0062 | 0.0807 | 0.7134 | 0.0973 | 2.0063 | 0.5032 | 68.2728 | 0.0373 | 0.0000 | 0.0000 | 0.9379 | 0.9317 | 0.9752 | 0.0000 | 0.0062 | 0.9689 |
| 0.9524 | 0.0238 | 0.8512 | 0.0060 | 0.1369 | 0.6596 | 0.0913 | 2.0240 | 0.5120 | 88.5870 | 0.0238 | 0.0000 | 0.0000 | 0.9524 | 0.9464 | 0.9821 | 0.0000 | 0.0000 | 0.9643 |
| 0.9137 | 0.0576 | 0.6978 | 0.0072 | 0.2878 | 0.5517 | 0.0746 | 2.1594 | 0.5797 | 83.2074 | 0.1367 | 0.0000 | 0.0000 | 0.8993 | 0.8993 | 0.9784 | 0.0072 | 0.0072 | 0.9209 |
| 0.9417 | 0.0333 | 0.5333 | 0.0167 | 0.4417 | 0.5205 | 0.0700 | 2.0336 | 0.5168 | 70.2911 | 0.0500 | 0.0000 | 0.0000 | 0.9000 | 0.8917 | 0.9917 | 0.0083 | 0.0083 | 0.9417 |
| 0.7778 | 0.0222 | 0.3778 | 0.0222 | 0.5556 | 0.4718 | 0.0613 | 2.0465 | 0.5233 | 65.1197 | 0.3333 | 0.0000 | 0.0000 | 0.8000 | 0.6889 | 0.9556 | 0.0000 | 0.0444 | 0.8889 |
| 0.8438 | 0.0000 | 0.0625 | 0.0104 | 0.9271 | 0.8426 | 0.0521 | 2.5851 | 0.7926 | 58.0701 | 0.1979 | 0.0000 | 0.0000 | 0.5208 | 0.8854 | 0.9896 | 0.0104 | 0.1771 | 0.9792 |
| 0.9225 | 0.0310 | 0.6744 | 0.0465 | 0.2093 | 0.6807 | 0.0921 | 1.9748 | 0.4874 | 65.0185 | 0.0930 | 0.0000 | 0.0000 | 0.7984 | 0.7364 | 0.9225 | 0.0078 | 0.0078 | 0.8760 |
| 0.9124 | 0.0052 | 0.4330 | 0.0000 | 0.5515 | 0.5587 | 0.0696 | 1.7853 | 0.3927 | 64.3324 | 0.0876 | 0.0000 | 0.0000 | 0.7371 | 0.7371 | 0.9845 | 0.0000 | 0.0103 | 0.9742 |
| 0.9290 | 0.0473 | 0.2899 | 0.0059 | 0.6923 | 0.3400 | 0.0459 | 2.0602 | 0.5301 | 53.3104 | 0.0473 | 0.0000 | 0.0000 | 0.8994 | 0.8994 | 0.9822 | 0.0000 | 0.0059 | 0.9231 |
| 0.9427 | 0.0417 | 0.5677 | 0.0000 | 0.4219 | 0.5806 | 0.0764 | 2.0159 | 0.5079 | 61.0973 | 0.0208 | 0.0000 | 0.0000 | 0.8698 | 0.8854 | 0.9844 | 0.0000 | 0.0000 | 0.9375 |
| 0.5615 | 0.0077 | 0.0615 | 0.1154 | 0.8154 | 0.4025 | 0.0513 | 2.1860 | 0.5930 | 57.4147 | 0.4308 | 0.0000 | 0.0000 | 0.7231 | 0.7308 | 0.9923 | 0.0077 | 0.0692 | 0.9769 |
| 0.9114 | 0.0570 | 0.5759 | 0.0000 | 0.3924 | 0.5385 | 0.0733 | 1.9281 | 0.4641 | 65.2089 | 0.1329 | 0.0000 | 0.0000 | 0.9304 | 0.8354 | 0.9684 | 0.0000 | 0.0063 | 0.9557 |
| 0.9524 | 0.0212 | 0.6614 | 0.0000 | 0.3280 | 0.6008 | 0.0811 | 1.9624 | 0.4812 | 66.0725 | 0.0476 | 0.0000 | 0.0000 | 0.9101 | 0.8942 | 0.9894 | 0.0000 | 0.0053 | 0.9577 |
| 0.9831 | 0.0056 | 0.6798 | 0.0000 | 0.3090 | 0.5904 | 0.0804 | 1.9659 | 0.4830 | 77.4933 | 0.0337 | 0.0000 | 0.0000 | 0.9438 | 0.9213 | 0.9888 | 0.0000 | 0.0056 | 0.9775 |
| 0.9785 | 0.0000 | 0.1828 | 0.0108 | 0.7849 | 0.3893 | 0.0551 | 1.9121 | 0.4560 | 71.6462 | 0.5376 | 0.0000 | 0.0000 | 0.9247 | 0.3871 | 0.9785 | 0.0108 | 0.0323 | 0.9677 |
| 0.9548 | 0.0129 | 0.3613 | 0.0000 | 0.6258 | 0.6117 | 0.0813 | 1.5229 | 0.2614 | 52.9756 | 0.0710 | 0.0000 | 0.0000 | 0.8903 | 0.8516 | 0.9871 | 0.0000 | 0.0065 | 0.9742 |
| 0.9588 | 0.0059 | 0.7294 | 0.0118 | 0.2294 | 0.6799 | 0.0918 | 1.9758 | 0.4879 | 79.2064 | 0.0588 | 0.0000 | 0.0000 | 0.9353 | 0.8882 | 0.9706 | 0.0000 | 0.0176 | 0.9588 |
| 0.9799 | 0.0067 | 0.7315 | 0.0000 | 0.2550 | 1.0572 | 0.0986 | 2.0068 | 0.5034 | 86.0711 | 0.0671 | 0.0000 | 0.0000 | 0.8993 | 0.9060 | 0.9866 | 0.0067 | 0.0134 | 0.9866 |
| 0.6853 | 0.0051 | 0.1523 | 0.0000 | 0.8426 | 0.3308 | 0.0386 | 1.9796 | 0.4898 | 82.4105 | 0.3503 | 0.0000 | 0.0000 | 0.5990 | 0.6244 | 0.9949 | 0.0000 | 0.0254 | 0.9746 |
| 0.7200 | 0.0067 | 0.0467 | 0.0133 | 0.9333 | 0.8065 | 0.0432 | 2.0872 | 0.5436 | 81.5081 | 0.5800 | 0.0000 | 0.0000 | 0.9533 | 0.6400 | 0.9933 | 0.0067 | 0.1200 | 0.9800 |
| 0.9398 | 0.0060 | 0.7590 | 0.0000 | 0.2289 | 0.6688 | 0.0902 | 1.9817 | 0.4909 | 73.9031 | 0.0843 | 0.0000 | 0.0000 | 0.9518 | 0.9337 | 0.9880 | 0.0060 | 0.0060 | 0.9759 |
| 0.9254 | 0.0044 | 0.5439 | 0.0000 | 0.4167 | 0.5041 | 0.0691 | 1.9680 | 0.4840 | 80.5938 | 0.1096 | 0.0000 | 0.0000 | 0.9298 | 0.8947 | 0.9561 | 0.0000 | 0.0044 | 0.9474 |
| 0.7471 | 0.0059 | 0.3412 | 0.0000 | 0.6471 | 0.5082 | 0.0595 | 2.0119 | 0.5060 | 83.4990 | 0.2941 | 0.0000 | 0.0000 | 0.6471 | 0.6765 | 0.9824 | 0.0059 | 0.0588 | 0.9765 |
| 0.9494 | 0.0000 | 0.7528 | 0.0112 | 0.2247 | 0.6623 | 0.0869 | 2.0227 | 0.5114 | 80.8781 | 0.1011 | 0.0000 | 0.0000 | 0.7978 | 0.8315 | 0.9888 | 0.0000 | 0.0056 | 0.9775 |
| 0.7081 | 0.0124 | 0.1429 | 0.0186 | 0.8261 | 0.8788 | 0.0554 | 2.2675 | 0.6338 | 79.7688 | 0.3851 | 0.0000 | 0.0062 | 0.8261 | 0.7081 | 0.9814 | 0.0062 | 0.2733 | 0.9627 |
| 0.7865 | 0.0000 | 0.4531 | 0.0052 | 0.5365 | 0.4726 | 0.0662 | 2.1885 | 0.5942 | 68.2911 | 0.2708 | 0.0000 | 0.0052 | 0.9635 | 0.9010 | 0.9948 | 0.0104 | 0.2240 | 0.9844 |
| 0.6917 | 0.0000 | 0.1504 | 0.0075 | 0.8346 | 0.3963 | 0.0520 | 2.2424 | 0.6212 | 77.1123 | 0.3910 | 0.0000 | 0.0000 | 0.9323 | 0.8571 | 0.9925 | 0.0075 | 0.3233 | 0.9774 |

| | | | | | | | | | | | | | | | | | | |
|---|---|---|---|---|---|---|---|---|---|---|---|---|---|---|---|---|---|---|
| 0.6529 | 0.0083 | 0.2479 | 0.0083 | 0.7273 | 0.4700 | 0.0580 | 2.1597 | 0.5798 | 76.4034 | 0.3802 | 0.0000 | 0.0083 | 0.7025 | 0.7686 | 0.9835 | 0.0165 | 0.1488 | 0.9752 |
| 0.8823 | 0.0007 | 0.1017 | 0.0007 | 0.8477 | 0.3302 | 0.0513 | 1.3870 | 0.1935 | 77.8516 | 0.7161 | 0.0000 | 0.0000 | 0.8830 | 0.2673 | 0.9501 | 0.0000 | 0.0000 | 0.9169 |
| 0.8302 | 0.0000 | 0.2139 | 0.0008 | 0.7003 | 0.3694 | 0.0545 | 1.4666 | 0.2333 | 59.3500 | 0.6163 | 0.0000 | 0.0000 | 0.8294 | 0.3837 | 0.9143 | 0.0000 | 0.0000 | 0.8302 |
| 0.6777 | 0.0614 | 0.4156 | 0.0153 | 0.5384 | 0.4806 | 0.0666 | 1.9221 | 0.4611 | 77.3909 | 0.3837 | 0.0000 | 0.0000 | 0.8146 | 0.7084 | 0.9693 | 0.0000 | 0.0006 | 0.8772 |
| 0.8804 | 0.0399 | 0.1627 | 0.0000 | 0.8373 | 0.3326 | 0.0489 | 2.0399 | 0.5199 | 83.4291 | 0.3987 | 0.0000 | 0.0000 | 0.7177 | 0.5614 | 1.0000 | 0.0000 | 0.0016 | 0.9585 |
| 0.7706 | 0.0000 | 0.0780 | 0.0765 | 0.8456 | 0.2478 | 0.0335 | 2.2661 | 0.6330 | 73.6879 | 0.3471 | 0.0000 | 0.0000 | 0.5000 | 0.6896 | 1.0000 | 0.0000 | 0.0000 | 0.9969 |
| 0.7780 | 0.0000 | 0.0000 | 0.0000 | 1.0000 | 0.3267 | 0.0438 | 1.9989 | 0.4994 | 74.4724 | 0.6382 | 0.0000 | 0.0000 | 0.6948 | 0.3885 | 1.0000 | 0.0000 | 0.0000 | 1.0000 |
| 0.8877 | 0.0007 | 0.1492 | 0.0007 | 0.8131 | 1.2286 | 0.1208 | 1.5391 | 0.2696 | 68.4771 | 0.1484 | 0.0000 | 0.0000 | 0.2600 | 0.2784 | 0.9631 | 0.0000 | 0.0000 | 0.8892 |
| 0.8282 | 0.0580 | 0.2571 | 0.0000 | 0.7429 | 0.4981 | 0.0666 | 1.8294 | 0.4147 | 82.7683 | 0.4005 | 0.0000 | 0.0000 | 0.6837 | 0.4835 | 1.0000 | 0.0000 | 0.0000 | 0.9977 |
| 0.6579 | 0.1140 | 0.2303 | 0.0000 | 0.7697 | 0.5964 | 0.0757 | 1.9133 | 0.4567 | 87.0445 | 0.5131 | 0.0000 | 0.0000 | 0.7412 | 0.5143 | 1.0000 | 0.0000 | 0.0000 | 0.8860 |
| 0.3940 | 0.5334 | 0.1437 | 0.0711 | 0.7838 | 0.8916 | 0.1088 | 2.6017 | 0.8009 | 61.1243 | 0.1095 | 0.0000 | 0.0000 | 0.2504 | 0.2859 | 0.9986 | 0.0000 | 0.0000 | 0.4666 |
| 0.6672 | 0.0832 | 0.0416 | 0.0832 | 0.7920 | 0.3765 | 0.0482 | 2.2928 | 0.6464 | 79.5199 | 0.3328 | 0.0000 | 0.0000 | 0.2928 | 0.5424 | 0.9168 | 0.0000 | 0.0017 | 0.9168 |
| 0.6763 | 0.2151 | 0.1613 | 0.0280 | 0.7570 | 0.8190 | 0.0938 | 2.2691 | 0.6346 | 73.2397 | 0.2161 | 0.0000 | 0.0000 | 0.2968 | 0.4591 | 0.9452 | 0.0011 | 0.0022 | 0.7828 |
| 0.9169 | 0.0000 | 0.0288 | 0.0554 | 0.9157 | 0.3978 | 0.0525 | 2.2031 | 0.6015 | 64.6948 | 0.1940 | 0.0000 | 0.0000 | 0.2772 | 0.5266 | 0.9446 | 0.0000 | 0.0277 | 0.9446 |
| 0.9096 | 0.0000 | 0.0235 | 0.0009 | 0.9304 | 0.5686 | 0.0797 | 1.3381 | 0.1690 | 72.8994 | 0.1374 | 0.0000 | 0.0000 | 0.3192 | 0.1826 | 0.9548 | 0.0000 | 0.0226 | 0.9548 |
| 0.7196 | 0.2492 | 0.0947 | 0.0000 | 0.9053 | 0.7951 | 0.0864 | 2.2012 | 0.6006 | 73.1923 | 0.4673 | 0.0000 | 0.0000 | 0.5626 | 0.1732 | 1.0000 | 0.0000 | 0.0156 | 0.7657 |
| 0.9655 | 0.0007 | 0.0345 | 0.0000 | 0.9655 | 0.3930 | 0.0451 | 2.3559 | 0.6779 | 56.4448 | 0.3045 | 0.0000 | 0.0000 | 0.4066 | 0.5758 | 1.0000 | 0.0000 | 0.0169 | 0.9993 |
| 0.6676 | 0.2660 | 0.0332 | 0.0000 | 0.9668 | 1.3424 | 0.1196 | 2.1662 | 0.5831 | 61.2737 | 0.1995 | 0.0000 | 0.0000 | 0.3019 | 0.3005 | 1.0000 | 0.0000 | 0.0013 | 0.7340 |
| 0.5906 | 0.3838 | 0.1024 | 0.0000 | 0.8976 | 1.2703 | 0.1259 | 2.4084 | 0.7042 | 61.0525 | 0.1024 | 0.0000 | 0.0000 | 0.2057 | 0.3337 | 1.0000 | 0.0000 | 0.0000 | 0.6162 |
| 0.6050 | 0.3517 | 0.0281 | 0.0281 | 0.9432 | 1.6062 | 0.1599 | 2.0573 | 0.5287 | 58.7166 | 0.0709 | 0.0000 | 0.0000 | 0.0985 | 0.1553 | 0.9719 | 0.0011 | 0.0000 | 0.6201 |
| 0.7178 | 0.1278 | 0.6912 | 0.0000 | 0.2566 | 0.2277 | 0.0289 | 2.1361 | 0.5680 | 63.9363 | 0.1278 | 0.0000 | 0.0000 | 0.8180 | 0.8200 | 0.3088 | 0.0000 | 0.0000 | 0.8190 |
| 0.7994 | 0.1996 | 0.6747 | 0.0000 | 0.3253 | 0.5902 | 0.0772 | 2.3581 | 0.6790 | 72.0300 | 0.0998 | 0.0000 | 0.0000 | 0.6737 | 0.6986 | 0.6996 | 0.0000 | 0.0000 | 0.7006 |
| 0.5818 | 0.2727 | 0.4364 | 0.0000 | 0.5636 | 0.6025 | 0.0729 | 2.5490 | 0.7745 | 71.0650 | 0.4364 | 0.0000 | 0.0000 | 0.6000 | 0.6000 | 0.9091 | 0.0000 | 0.0000 | 0.6545 |
| 0.5427 | 0.4234 | 0.4065 | 0.0000 | 0.5935 | 0.8908 | 0.1074 | 2.5583 | 0.7791 | NA | 0.2033 | 0.0000 | 0.0000 | 0.4743 | 0.5081 | 1.0000 | 0.0000 | 0.0000 | 0.5766 |
| 0.7288 | 0.1808 | 0.6329 | 0.0000 | 0.3219 | 0.3944 | 0.0499 | 2.4744 | 0.7372 | 92.1300 | 0.4087 | 0.0000 | 0.0000 | 0.7251 | 0.7251 | 0.7722 | 0.0000 | 0.0000 | 0.7722 |
| 0.8338 | 0.1330 | 0.4987 | 0.0000 | 0.5013 | 0.7876 | 0.0927 | 2.2270 | 0.6135 | 87.4489 | 0.1676 | 0.0000 | 0.0000 | 0.5346 | 0.5997 | 0.8005 | 0.0000 | 0.0000 | 0.7340 |
| 0.6667 | 0.2656 | 0.4024 | 0.0000 | 0.5976 | 0.9865 | 0.1125 | 2.3091 | 0.6545 | 71.0650 | 0.2324 | 0.0000 | 0.0000 | 0.4993 | 0.5339 | 0.9641 | 0.0000 | 0.0000 | 0.6999 |
| 0.7750 | 0.2000 | 0.6000 | 0.0000 | 0.4000 | 0.5727 | 0.0696 | 2.5000 | 0.7500 | 69.9733 | 0.4500 | 0.0000 | 0.0000 | 0.7000 | 0.7000 | 0.9750 | 0.0000 | 0.0000 | 0.8000 |
| 0.7500 | 0.0000 | 0.3738 | 0.0025 | 0.6238 | 0.3661 | 0.0400 | 2.1238 | 0.5619 | 64.0433 | 0.6238 | 0.0000 | 0.0000 | 0.7475 | 0.7450 | 0.8762 | 0.0000 | 0.0000 | 0.9975 |
| 0.8861 | 0.0569 | 0.5991 | 0.0000 | 0.4009 | 0.7797 | 0.0827 | 2.3121 | 0.6560 | 52.3390 | 0.4863 | 0.0000 | 0.0000 | 0.6583 | 0.7130 | 0.9989 | 0.0000 | 0.0000 | 0.9419 |
| 0.8753 | 0.0623 | 0.5312 | 0.0000 | 0.4688 | 0.5321 | 0.0594 | 2.4972 | 0.7486 | 48.9075 | 0.5312 | 0.0000 | 0.0000 | 0.6259 | 0.6870 | 0.8741 | 0.0000 | 0.0000 | 0.8130 |
| 0.9028 | 0.0486 | 0.8989 | 0.0000 | 0.1011 | 0.2205 | 0.0297 | 2.6537 | 0.8268 | 93.6900 | 0.6560 | 0.0000 | 0.0000 | 0.9485 | 0.9485 | 0.9018 | 0.0000 | 0.0000 | 0.9504 |
| 0.8698 | 0.0370 | 0.7226 | 0.0370 | 0.2219 | 0.6812 | 0.0907 | 1.9992 | 0.4996 | 79.9400 | 0.1109 | 0.0000 | 0.0000 | 0.8328 | 0.8328 | 0.9438 | 0.0000 | 0.0000 | 0.9445 |
| 0.8219 | 0.0712 | 0.2151 | 0.0000 | 0.7493 | 4.7462 | 0.1576 | 1.8892 | 0.4446 | 44.9928 | 0.1781 | 0.0000 | 0.0000 | 0.3932 | 0.3590 | 0.9644 | 0.0000 | 0.0014 | 0.8917 |
| 0.8555 | 0.1416 | 0.5694 | 0.0028 | 0.4278 | 0.6913 | 0.0789 | 2.1388 | 0.5694 | 56.2292 | 0.4249 | 0.0000 | 0.0000 | 0.7110 | 0.5694 | 0.8584 | 0.0000 | 0.0000 | 0.8584 |
| 0.8127 | 0.1248 | 0.4382 | 0.0000 | 0.5618 | 1.1320 | 0.1257 | 1.9376 | 0.4688 | 48.1224 | 0.1873 | 0.0000 | 0.0000 | 0.5630 | 0.5630 | 0.9988 | 0.0000 | 0.0000 | 0.8752 |
| 0.7691 | 0.1529 | 0.3089 | 0.0000 | 0.6896 | 1.2812 | 0.1391 | 1.8469 | 0.4234 | 74.6553 | 0.0780 | 0.0000 | 0.0000 | 0.4618 | 0.5382 | 0.9969 | 0.0000 | 0.0000 | 0.8440 |
| 0.8335 | 0.0555 | 0.6670 | 0.0555 | 0.2775 | 0.7466 | 0.0904 | 2.2231 | 0.6115 | 41.7195 | 0.3330 | 0.0000 | 0.0000 | 0.7769 | 0.7780 | 1.0000 | 0.0000 | 0.0000 | 0.9434 |
| 0.7321 | 0.1326 | 0.5345 | 0.0013 | 0.4642 | 0.9271 | 0.1109 | 2.0663 | 0.5332 | 59.3373 | 0.2003 | 0.0000 | 0.0000 | 0.7321 | 0.7321 | 0.9973 | 0.0000 | 0.0000 | 0.8674 |
| 0.7561 | 0.1951 | 0.2927 | 0.0000 | 0.7073 | 1.4435 | 0.1528 | 1.8780 | 0.4390 | 70.2628 | 0.1463 | 0.0000 | 0.0000 | 0.3902 | 0.3902 | 1.0000 | 0.0000 | 0.0000 | 0.8049 |
| 0.5259 | 0.0998 | 0.2265 | 0.0000 | 0.7735 | 0.8368 | 0.0911 | 1.9261 | 0.4631 | 67.0702 | 0.4491 | 0.0000 | 0.0000 | 0.6497 | 0.6507 | 0.9990 | 0.0000 | 0.0000 | 0.8992 |
| 0.6926 | 0.2049 | 0.2059 | 0.0000 | 0.7941 | 1.3421 | 0.1411 | 1.8730 | 0.4365 | 45.1636 | 0.2049 | 0.0000 | 0.0000 | 0.3586 | 0.3596 | 1.0000 | 0.0000 | 0.0000 | 0.7941 |
| 0.6489 | 0.0034 | 0.4320 | 0.0017 | 0.5645 | 0.5472 | 0.0632 | 2.0414 | 0.5207 | 76.5648 | 0.5198 | 0.0000 | 0.0000 | 0.7797 | 0.8176 | 0.9983 | 0.0000 | 0.0000 | 0.9105 |
| 0.6365 | 0.1808 | 0.3183 | 0.0018 | 0.6799 | 1.0238 | 0.1142 | 2.0000 | 0.5000 | 76.9228 | 0.2731 | 0.0000 | 0.0000 | 0.5461 | 0.5443 | 1.0000 | 0.0000 | 0.0000 | 0.7740 |
| 0.4434 | 0.4376 | 0.2380 | 0.0012 | 0.7608 | 1.1511 | 0.1321 | 2.2345 | 0.6173 | 69.1417 | 0.1785 | 0.0000 | 0.0000 | 0.4411 | 0.3839 | 0.9977 | 0.0012 | 0.0000 | 0.5589 |
| 0.7920 | 0.0689 | 0.3444 | 0.0014 | 0.6543 | 0.9988 | 0.1058 | 1.9311 | 0.4656 | 75.6841 | 0.3099 | 0.0000 | 0.0000 | 0.4821 | 0.6198 | 0.9656 | 0.0689 | 0.0000 | 0.8278 |
| 0.4846 | 0.2564 | 0.1615 | 0.0013 | 0.8372 | 1.3177 | 0.1225 | 2.0013 | 0.5006 | 63.7400 | 0.2910 | 0.0000 | 0.0000 | 0.4500 | 0.4833 | 1.0000 | 0.0013 | 0.0000 | 0.6782 |
| 0.8109 | 0.0622 | 0.3109 | 0.0012 | 0.6866 | 1.0330 | 0.1141 | 1.8122 | 0.4061 | 81.0214 | 0.1891 | 0.0000 | 0.0000 | 0.5000 | 0.4975 | 1.0000 | 0.0012 | 0.0000 | 0.8744 |
| 0.4880 | 0.0601 | 0.3618 | 0.0012 | 0.6370 | 0.6216 | 0.0693 | 2.0590 | 0.5295 | 85.0863 | 0.5144 | 0.0000 | 0.0000 | 0.7548 | 0.8137 | 1.0000 | 0.0000 | 0.0000 | 0.8774 |
| 0.7672 | 0.0000 | 0.5816 | 0.0009 | 0.4174 | 0.9739 | 0.1077 | 1.8386 | 0.4193 | 51.1881 | 0.3711 | 0.0000 | 0.0000 | 0.7430 | 0.7904 | 0.9527 | 0.0464 | 0.0000 | 0.9063 |
| 0.6512 | 0.1718 | 0.3041 | 0.0017 | 0.6924 | 0.9797 | 0.1093 | 1.9983 | 0.4991 | 82.6218 | 0.3454 | 0.0000 | 0.0000 | 0.5619 | 0.5601 | 0.9966 | 0.0430 | 0.0000 | 0.7801 |

| | | | | | | | | | | | | | | | | | | |
|---|---|---|---|---|---|---|---|---|---|---|---|---|---|---|---|---|---|---|
| 0.8704 | 0.0025 | 0.1889 | 0.0049 | 0.7444 | 1.9111 | 0.1552 | 1.4438 | 0.2219 | 76.3720 | 0.1877 | 0.0000 | 0.0000 | 0.2494 | 0.3728 | 0.9988 | 0.1235 | 0.0012 | 0.8099 |
| 0.8704 | 0.0255 | 0.4847 | 0.0010 | 0.5133 | 1.1731 | 0.1316 | 1.6431 | 0.3216 | 72.1075 | 0.1806 | 0.0000 | 0.0000 | 0.6653 | 0.5622 | 0.9980 | 0.0000 | 0.0000 | 0.9714 |
| 0.8123 | 0.0415 | 0.6030 | 0.0623 | 0.3339 | 1.0977 | 0.1267 | 1.9584 | 0.4792 | 53.2621 | 0.1470 | 0.0000 | 0.0000 | 0.6860 | 0.7284 | 0.9992 | 0.0008 | 0.0000 | 0.9145 |
| 0.8078 | 0.0000 | 0.2386 | 0.0009 | 0.7595 | 1.2684 | 0.1332 | 1.5280 | 0.2640 | 79.3578 | 0.2396 | 0.0000 | 0.0000 | 0.4981 | 0.4773 | 0.9991 | 0.0019 | 0.0000 | 0.9735 |
| 0.7255 | 0.0000 | 0.2505 | 0.2246 | 0.5250 | 1.4158 | 0.1487 | 1.3743 | 0.1871 | 83.6360 | 0.1497 | 0.0000 | 0.0000 | 0.2754 | 0.3752 | 1.0000 | 0.0000 | 0.0250 | 0.8004 |
| 0.8507 | 0.0015 | 0.0015 | 0.0007 | 0.9239 | 0.6238 | 0.0707 | 1.5178 | 0.2589 | 79.0613 | 0.4265 | 0.0000 | 0.0000 | 0.8145 | 0.4619 | 0.9993 | 0.0000 | 0.0185 | 0.9254 |
| 0.9163 | 0.0008 | 0.0215 | 0.0431 | 0.9138 | 0.5803 | 0.0661 | 1.5525 | 0.2762 | 60.1068 | 0.2925 | 0.0000 | 0.0000 | 0.6686 | 0.4979 | 0.9586 | 0.0000 | 0.0837 | 0.9785 |
| 0.8697 | 0.0434 | 0.0434 | 0.0000 | 0.9557 | 0.6544 | 0.0770 | 1.3484 | 0.1742 | 69.0608 | 0.4561 | 0.0000 | 0.0000 | 0.7394 | 0.3692 | 1.0000 | 0.0000 | 0.1955 | 0.9991 |
| 0.7547 | 0.0943 | 0.0943 | 0.0755 | 0.6792 | 1.0066 | 0.0995 | 1.4528 | 0.2264 | 62.7780 | 0.3019 | 0.0000 | 0.0000 | 0.6038 | 0.2830 | 1.0000 | 0.0000 | 0.0755 | 0.8491 |
| 0.7309 | 0.0538 | 0.0538 | 0.1615 | 0.7836 | 1.0897 | 0.0889 | 1.5841 | 0.2920 | 64.3721 | 0.3509 | 0.0000 | 0.0000 | 0.6233 | 0.4855 | 0.9731 | 0.0000 | 0.1367 | 0.9709 |
| 0.7859 | 0.0238 | 0.0238 | 0.0723 | 0.9039 | 0.3753 | 0.0452 | 1.7393 | 0.3696 | 52.6149 | 0.2388 | 0.0000 | 0.0000 | 0.9039 | 0.7850 | 1.0000 | 0.0000 | 0.4282 | 0.9762 |
| 0.8218 | 0.0009 | 0.0009 | 0.0452 | 0.9317 | 0.8889 | 0.0909 | 1.5703 | 0.2851 | 64.0950 | 0.2234 | 0.0000 | 0.0000 | 0.6445 | 0.5771 | 0.9778 | 0.0000 | 0.3555 | 0.9557 |
| 0.8827 | 0.0195 | 0.0399 | 0.1181 | 0.8225 | 0.6820 | 0.0793 | 1.5485 | 0.2743 | 63.0611 | 0.1767 | 0.0000 | 0.0000 | 0.6083 | 0.5098 | 0.9797 | 0.0000 | 0.1767 | 0.9797 |
| 0.7639 | 0.0262 | 0.0262 | 0.0787 | 0.6852 | 0.7765 | 0.0903 | 1.6579 | 0.3290 | 71.4400 | 0.2644 | 0.0000 | 0.0000 | 0.7901 | 0.4470 | 1.0000 | 0.0000 | 0.1836 | 0.7639 |
| 0.7721 | 0.0000 | 0.0590 | 0.0306 | 0.9093 | 0.6769 | 0.0728 | 1.7128 | 0.3564 | 64.5420 | 0.2562 | 0.0000 | 0.0000 | 0.7698 | 0.7381 | 0.9977 | 0.0000 | 0.2846 | 0.9977 |
| 0.9020 | 0.0196 | 0.1371 | 0.0392 | 0.8041 | 0.6443 | 0.0888 | 1.9600 | 0.4800 | 84.4535 | 0.1176 | 0.0000 | 0.0000 | 0.9804 | 0.1575 | 0.9804 | 0.0000 | 0.0588 | 0.9608 |
| 0.8201 | 0.0199 | 0.0215 | 0.0605 | 0.9180 | 0.2796 | 0.0416 | 1.6658 | 0.3329 | 78.9039 | 0.1998 | 0.0000 | 0.0000 | 0.9594 | 0.8185 | 0.9594 | 0.0796 | 0.4196 | 0.9602 |
| 0.8188 | 0.0000 | 0.0000 | 0.1132 | 0.8859 | 0.3161 | 0.0467 | 1.6603 | 0.3302 | 61.3633 | 0.2500 | 0.0000 | 0.0000 | 0.9547 | 0.8623 | 1.0000 | 0.0226 | 0.2509 | 0.9991 |
| 0.7578 | 0.0012 | 0.0313 | 0.1819 | 0.7855 | 0.2521 | 0.0357 | 1.8301 | 0.4151 | 33.9298 | 0.3048 | 0.0000 | 0.0000 | 0.8771 | 0.7542 | 0.8795 | 0.0602 | 0.4530 | 0.8783 |
| 0.7325 | 0.0000 | 0.0007 | 0.0357 | 0.9636 | 0.6500 | 0.0771 | 1.7081 | 0.3540 | 36.0691 | 0.2860 | 0.0000 | 0.0000 | 0.8395 | 0.7853 | 0.9822 | 0.0357 | 0.1434 | 0.9822 |
| 0.6949 | 0.0000 | 0.0008 | 0.0610 | 0.9373 | 0.1613 | 0.0247 | 1.9779 | 0.4890 | 34.8059 | 0.3263 | 0.0000 | 0.0000 | 0.9593 | 0.8560 | 0.9593 | 0.0814 | 0.4906 | 0.9585 |
| 0.8701 | 0.0000 | 0.1957 | 0.1325 | 0.6719 | 1.2875 | 0.1420 | 1.2896 | 0.1448 | 36.1790 | 0.0866 | 0.0000 | 0.0000 | 0.2823 | 0.2623 | 0.9775 | 0.0000 | 0.0009 | 0.9100 |
| 1.0000 | 0.0000 | 0.0000 | 0.3736 | 0.6264 | 1.8116 | 0.1479 | 1.4379 | 0.2190 | 35.6533 | 0.0826 | 0.0000 | 0.0000 | 0.0479 | 0.0413 | 0.9587 | 0.0000 | 0.0000 | 0.9157 |
| 1.0000 | 0.0000 | 0.3748 | 0.0268 | 0.5984 | 1.2980 | 0.1416 | 1.4108 | 0.2054 | 36.6096 | 0.0994 | 0.0000 | 0.0000 | 0.4264 | 0.4006 | 0.9751 | 0.0000 | 0.0000 | 0.8996 |
| 0.9021 | 0.0010 | 0.1957 | 0.0998 | 0.7045 | 1.4712 | 0.1493 | 1.3191 | 0.1596 | 36.3143 | 0.0988 | 0.0000 | 0.0000 | 0.2936 | 0.2674 | 0.9990 | 0.0000 | 0.0000 | 0.9244 |
| 0.8958 | 0.0000 | 0.4177 | 0.1063 | 0.4750 | 1.1181 | 0.1315 | 1.4223 | 0.2112 | 35.5620 | 0.0281 | 0.0000 | 0.0000 | 0.4740 | 0.4458 | 0.9990 | 0.0000 | 0.0010 | 0.9698 |
| 0.8102 | 0.0000 | 0.2144 | 0.2154 | 0.5702 | 1.0822 | 0.1260 | 1.3188 | 0.1594 | 60.9867 | 0.0958 | 0.0000 | 0.0000 | 0.3330 | 0.2865 | 0.9763 | 0.0000 | 0.0237 | 0.9269 |
| 0.9114 | 0.0000 | 0.3339 | 0.1337 | 0.5323 | 1.3782 | 0.1426 | 1.3726 | 0.1863 | 77.8150 | 0.0664 | 0.0000 | 0.0000 | 0.4455 | 0.4234 | 0.9557 | 0.0000 | 0.0009 | 0.8441 |
| 0.6542 | 0.0000 | 0.3255 | 0.4085 | 0.2661 | 0.8874 | 0.1114 | 1.4241 | 0.2120 | 65.8975 | 0.0618 | 0.0000 | 0.0000 | 0.4288 | 0.4076 | 0.9593 | 0.0000 | 0.0000 | 0.8967 |
| 0.8141 | 0.0000 | 0.3727 | 0.2091 | 0.4182 | 1.0760 | 0.1257 | 1.4282 | 0.2141 | 43.1775 | 0.0929 | 0.0000 | 0.0000 | 0.4647 | 0.4191 | 0.9768 | 0.0000 | 0.0000 | 0.9294 |
| 0.7652 | 0.0000 | 0.2640 | 0.2640 | 0.4720 | 1.2326 | 0.1386 | 1.2744 | 0.1372 | 50.0755 | 0.0596 | 0.0000 | 0.0000 | 0.2979 | 0.3236 | 0.9708 | 0.0000 | 0.0000 | 0.8540 |
| 0.9502 | 0.0000 | 0.2488 | 0.0756 | 0.6746 | 1.5535 | 0.1549 | 1.2602 | 0.1301 | 62.1957 | 0.1254 | 0.0000 | 0.0000 | 0.2776 | 0.3005 | 0.9751 | 0.0000 | 0.0010 | 0.8249 |
| 0.9988 | 0.0012 | 0.1264 | 0.0632 | 0.8104 | 1.4132 | 0.1334 | 1.2721 | 0.1361 | 59.0759 | 0.0942 | 0.0000 | 0.0000 | 0.2553 | 0.1896 | 0.9380 | 0.0000 | 0.0012 | 0.8439 |
| 0.9979 | 0.0000 | 0.0000 | 0.0825 | 0.9175 | 1.1399 | 0.1201 | 1.4614 | 0.2307 | 64.2601 | 0.1361 | 0.0000 | 0.0000 | 0.3280 | 0.1640 | 0.9989 | 0.0536 | 0.2433 | 0.7846 |
| 0.6676 | 0.0000 | 0.0000 | 0.0665 | 0.8449 | 0.9510 | 0.0968 | 1.7340 | 0.3670 | 51.2480 | 0.3989 | 0.0000 | 0.0000 | 0.6232 | 0.4663 | 1.0000 | 0.0443 | 0.2004 | 0.9105 |
| 0.7590 | 0.0000 | 0.0000 | 0.0904 | 0.8795 | 0.5055 | 0.0689 | 1.5157 | 0.2578 | 50.2822 | 0.3325 | 0.0000 | 0.0000 | 0.4867 | 0.3337 | 1.0000 | 0.0012 | 0.0928 | 0.9699 |
| 1.0000 | 0.0000 | 0.0009 | 0.0472 | 0.9519 | 0.6923 | 0.0966 | 1.1426 | 0.0713 | 50.9152 | 0.0472 | 0.0000 | 0.0000 | 0.1657 | 0.0713 | 1.0000 | 0.0463 | 0.1398 | 1.0000 |
| 0.9447 | 0.0000 | 0.0011 | 0.0564 | 0.9425 | 0.4996 | 0.0597 | 1.9967 | 0.4983 | 60.5062 | 0.1117 | 0.0000 | 0.0000 | 0.9436 | 0.7478 | 1.0000 | 0.0277 | 0.6659 | 0.9989 |
| 0.8466 | 0.0000 | 0.0383 | 0.0008 | 0.9225 | 0.8770 | 0.1054 | 1.2523 | 0.1262 | 62.6800 | 0.1541 | 0.0000 | 0.0000 | 0.2906 | 0.2324 | 1.0000 | 0.0192 | 0.0215 | 0.8850 |
| 0.8185 | 0.0000 | 0.1824 | 0.0454 | 0.7722 | 0.5523 | 0.0693 | 2.0672 | 0.5336 | 62.6038 | 0.2269 | 0.0000 | 0.0000 | 0.9546 | 0.7495 | 1.0000 | 0.1815 | 0.3875 | 0.9991 |
| 0.6521 | 0.0000 | 0.0937 | 0.0473 | 0.8590 | 0.2699 | 0.0387 | 2.0445 | 0.5223 | 54.2754 | 0.3952 | 0.0000 | 0.0000 | 0.9536 | 0.8599 | 1.0000 | 0.0928 | 0.3043 | 1.0000 |
| 1.0000 | 0.0000 | 0.0799 | 0.0999 | 0.8193 | 0.4333 | 0.0605 | 2.1000 | 0.5500 | 77.4600 | 0.0999 | 0.0000 | 0.0000 | 0.9193 | 0.5995 | 0.9992 | 0.2998 | 0.5396 | 0.9992 |
| 1.0000 | 0.0000 | 0.2152 | 0.1884 | 0.5953 | 0.7618 | 0.0867 | 2.0782 | 0.5391 | 65.5680 | 0.1638 | 0.0000 | 0.0000 | 0.8640 | 0.7259 | 0.9722 | 0.0535 | 0.5910 | 0.9454 |
| 0.9566 | 0.0000 | 0.0442 | 0.1951 | 0.7606 | 0.4198 | 0.0580 | 1.8031 | 0.4016 | 83.0450 | 0.2385 | 0.0000 | 0.0000 | 0.8265 | 0.7598 | 1.0000 | 0.0434 | 0.2836 | 0.9991 |
| 0.4907 | 0.1698 | 0.3608 | 0.0645 | 0.5747 | 0.4150 | 0.0546 | 1.8031 | 0.4016 | 73.4600 | 0.5526 | 0.0000 | 0.0000 | 0.8514 | 0.6154 | 0.9576 | 0.0424 | 0.1087 | 0.9779 |
| 0.9402 | 0.0012 | 0.0610 | 0.1172 | 0.8218 | 0.8918 | 0.1021 | 1.4251 | 0.2126 | 80.0167 | 0.1477 | 0.0000 | 0.0000 | 0.3259 | 0.2649 | 0.9707 | 0.0000 | 0.1489 | 0.7069 |
| 0.7348 | 0.2336 | 0.2944 | 0.1764 | 0.5280 | 0.8154 | 0.0920 | 1.5776 | 0.2888 | 67.2367 | 0.4100 | 0.0000 | 0.0012 | 0.5876 | 0.2932 | 0.9696 | 0.0000 | 0.2056 | 0.9404 |
| 0.9984 | 0.0016 | 0.0824 | 0.3170 | 0.5990 | 0.5703 | 0.0649 | 1.8657 | 0.4329 | 80.5567 | 0.2393 | 0.0000 | 0.0000 | 0.5642 | 0.4802 | 0.9208 | 0.0000 | 0.4390 | 0.9192 |
| 0.2693 | 0.0858 | 0.0926 | 0.0034 | 0.9039 | 0.1422 | 0.0218 | 1.8643 | 0.4321 | 71.0650 | 0.7307 | 0.0000 | 0.0000 | 0.9931 | 0.8199 | 0.9949 | 0.0429 | 0.1750 | 0.9537 |
| 0.9366 | 0.0000 | 0.3340 | 0.0317 | 0.6343 | 0.6204 | 0.0818 | 2.1103 | 0.5551 | 86.4984 | 0.0323 | 0.0000 | 0.0000 | 0.9202 | 0.6343 | 0.9366 | 0.2693 | 0.5716 | 0.9994 |
| 0.7811 | 0.0000 | 0.2198 | 0.1459 | 0.6342 | 0.3849 | 0.0544 | 1.9741 | 0.4870 | 75.6329 | 0.3161 | 0.0000 | 0.0000 | 0.7811 | 0.6586 | 0.9514 | 0.0973 | 0.2704 | 0.9504 |

| | | | | | | | | | | | | | | | | | | |
|---|---|---|---|---|---|---|---|---|---|---|---|---|---|---|---|---|---|---|
| 0.9991 | 0.0000 | 0.1796 | 0.1549 | 0.6655 | 0.7519 | 0.0900 | 1.7786 | 0.3893 | 76.1589 | 0.0451 | 0.0000 | 0.0000 | 0.9097 | 0.6655 | 0.9106 | 0.1770 | 0.4885 | 0.9106 |
| 0.6810 | 0.1595 | 0.4801 | 0.0598 | 0.4601 | 0.8884 | 0.1052 | 1.7007 | 0.3504 | 78.6573 | 0.2392 | 0.0000 | 0.0000 | 0.7990 | 0.4402 | 0.8397 | 0.1595 | 0.4195 | 0.9992 |
| 0.9990 | 0.0010 | 0.2016 | 0.2745 | 0.5240 | 0.3608 | 0.0505 | 2.1292 | 0.5646 | 61.9383 | 0.2255 | 0.0000 | 0.0000 | 0.7505 | 0.5000 | 0.9501 | 0.1996 | 0.4251 | 0.9251 |
| 0.9982 | 0.0009 | 0.0930 | 0.1807 | 0.7254 | 1.2376 | 0.1307 | 1.5714 | 0.2857 | 60.0586 | 0.1373 | 0.0000 | 0.0000 | 0.5023 | 0.3857 | 0.9539 | 0.0226 | 0.3415 | 0.9530 |
| 1.0000 | 0.0000 | 0.2206 | 0.0441 | 0.7353 | 0.4113 | 0.0611 | 2.2121 | 0.6061 | 56.7743 | 0.0294 | 0.0000 | 0.0000 | 0.9706 | 0.5735 | 0.9706 | 0.3676 | 0.6324 | 0.9706 |
| 1.0000 | 0.0000 | 0.2591 | 0.0345 | 0.7064 | 0.4903 | 0.0716 | 1.9649 | 0.4824 | 59.7539 | 0.0517 | 0.0000 | 0.0000 | 1.0000 | 0.6899 | 1.0000 | 0.2584 | 0.4659 | 1.0000 |
| 0.9991 | 0.0000 | 0.1874 | 0.0464 | 0.7662 | 0.5691 | 0.0737 | 2.1652 | 0.5826 | 78.2363 | 0.0241 | 0.0000 | 0.0009 | 0.9768 | 0.6039 | 0.9768 | 0.3479 | 0.6976 | 0.9768 |
| 1.0000 | 0.0000 | 0.3568 | 0.0238 | 0.6194 | 0.5266 | 0.0742 | 2.0238 | 0.5119 | 60.6987 | 0.0247 | 0.0000 | 0.0000 | 1.0000 | 0.6185 | 1.0000 | 0.3568 | 0.5005 | 1.0000 |
| 1.0000 | 0.0000 | 0.2729 | 0.0544 | 0.6727 | 0.4899 | 0.0698 | 1.9797 | 0.4898 | 59.5937 | 0.0189 | 0.0000 | 0.0000 | 0.9637 | 0.6727 | 0.9637 | 0.2721 | 0.4731 | 0.9637 |
| 1.0000 | 0.0000 | 0.0000 | 0.0000 | 1.0000 | NA | NA | NA | NA | 65.1881 | 0.0000 | 0.0000 | 0.0000 | 0.0000 | 0.0000 | 0.0000 | 0.0000 | 0.0000 | 0.0000 |
| 1.0000 | 0.0000 | 0.0000 | 0.8483 | 0.1517 | 0.4049 | 0.0605 | 1.3399 | 0.1699 | 78.0983 | 0.1461 | 0.0000 | 0.0000 | 0.1404 | 0.1404 | 0.9944 | 0.0000 | 0.0000 | 0.4382 |
| 1.0000 | 0.0000 | 0.0333 | 0.2333 | 0.7333 | 0.5214 | 0.0660 | 2.0500 | 0.5250 | 33.4889 | 0.1667 | 0.0000 | 0.0000 | 0.4000 | 0.3667 | 0.7000 | 0.0000 | 0.0333 | 0.7000 |
| 0.9984 | 0.0016 | 0.0016 | 0.9937 | 0.0047 | 0.8178 | 0.1107 | 1.6667 | 0.3333 | 60.4610 | 0.0016 | 0.0000 | 0.0000 | 0.0032 | 0.0032 | 0.9984 | 0.0000 | 0.0000 | 0.9968 |
| 1.0000 | 0.0000 | 0.0000 | 0.1429 | 0.8571 | 0.0539 | 0.0096 | 2.4286 | 0.7143 | 78.9725 | 0.5714 | 0.0000 | 0.0000 | 0.4286 | 0.5714 | 1.0000 | 0.1429 | 0.1429 | 0.8571 |
| 0.9884 | 0.0000 | 0.0116 | 0.3256 | 0.6512 | 0.4832 | 0.0616 | 2.2588 | 0.6294 | 47.6808 | 0.0465 | 0.0000 | 0.0000 | 0.3488 | 0.6395 | 0.9884 | 0.0116 | 0.0000 | 0.6744 |
| 1.0000 | 0.0000 | 0.0000 | 0.2000 | 0.8000 | 3.6926 | 0.0913 | 1.8571 | 0.4286 | 60.3707 | 0.1333 | 0.0000 | 0.0000 | 0.2000 | 0.2000 | 1.0000 | 0.0000 | 0.0000 | 0.8000 |
| 1.0000 | 0.0000 | 0.0000 | 0.0109 | 0.7158 | 0.3379 | 0.0400 | 2.5440 | 0.7720 | 51.7777 | 0.2896 | 0.0000 | 0.0000 | 0.1475 | 0.9672 | 0.9945 | 0.0055 | 0.0055 | 0.9836 |
| 0.9773 | 0.0227 | 0.0227 | 0.0682 | 0.8636 | 0.4430 | 0.0624 | 2.0238 | 0.5119 | NA | 0.0682 | 0.0000 | 0.0000 | 0.8182 | 0.5682 | 0.9545 | 0.0000 | 0.3864 | 0.8864 |
| 1.0000 | 0.0000 | 0.0093 | 0.4815 | 0.5000 | 0.5588 | 0.0743 | 1.7642 | 0.3821 | NA | 0.0093 | 0.0000 | 0.0000 | 0.2407 | 0.4722 | 0.9907 | 0.0000 | 0.0093 | 0.2870 |
| 1.0000 | 0.0000 | 0.0000 | 0.0000 | 1.0000 | 0.3285 | 0.0504 | 2.0000 | 0.5000 | NA | 0.0000 | 0.0000 | 0.0000 | 0.5000 | 1.0000 | 1.0000 | 0.0000 | 0.0000 | 1.0000 |
| 1.0000 | 0.0000 | 0.0000 | 0.0195 | 0.9805 | 0.0682 | 0.0113 | 2.3137 | 0.6569 | NA | 0.6494 | 0.0000 | 0.0000 | 0.0130 | 0.9805 | 1.0000 | 0.6494 | 0.0000 | 0.3377 |
| 0.9293 | 0.0435 | 0.0543 | 0.4511 | 0.4946 | 0.7381 | 0.0966 | 1.5621 | 0.2811 | NA | 0.0707 | 0.0000 | 0.0000 | 0.2174 | 0.0761 | 0.9185 | 0.0109 | 0.0163 | 0.5435 |
| 0.9520 | 0.0320 | 0.0400 | 0.4800 | 0.4800 | 0.8466 | 0.0987 | 1.6612 | 0.3306 | NA | 0.0480 | 0.0000 | 0.0000 | 0.3120 | 0.0560 | 0.9680 | 0.0080 | 0.0320 | 0.5200 |
| 0.7267 | 0.0067 | 0.0133 | 0.3000 | 0.6867 | 0.4357 | 0.0550 | 1.9200 | 0.4600 | NA | 0.5467 | 0.0000 | 0.0000 | 0.6400 | 0.3333 | 1.0000 | 0.0067 | 0.0267 | 0.6933 |
| 0.9194 | 0.0645 | 0.0726 | 0.6532 | 0.2661 | 0.5197 | 0.0729 | 1.3025 | 0.1513 | NA | 0.0887 | 0.0000 | 0.0000 | 0.2097 | 0.0645 | 0.9597 | 0.0081 | 0.0726 | 0.3065 |
| 0.9932 | 0.0068 | 0.0135 | 0.7027 | 0.2838 | 1.1043 | 0.1323 | 1.9048 | 0.4524 | NA | 0.0473 | 0.0000 | 0.0000 | 0.1486 | 0.0405 | 0.9932 | 0.0135 | 0.0270 | 0.3041 |
| 0.9869 | 0.0065 | 0.0131 | 0.0196 | 0.9673 | 2.3248 | 0.1456 | 1.2288 | 0.1144 | NA | 0.0196 | 0.0000 | 0.0000 | 0.0915 | 0.0523 | 1.0000 | 0.0065 | 0.0261 | 0.9804 |
| 0.9431 | 0.0190 | 0.0237 | 0.0806 | 0.8957 | 1.0047 | 0.1036 | 1.3223 | 0.1611 | NA | 0.0569 | 0.0000 | 0.0047 | 0.2322 | 0.1754 | 1.0000 | 0.0095 | 0.0521 | 0.9147 |
| 0.7872 | 0.0284 | 0.0284 | 0.4468 | 0.5248 | 1.7144 | 0.1265 | 1.9143 | 0.4571 | NA | 0.2057 | 0.0000 | 0.0000 | 0.4468 | 0.2695 | 0.9929 | 0.0071 | 0.0851 | 0.5106 |
| 0.9842 | 0.0053 | 0.0211 | 0.0053 | 0.9737 | 0.1206 | 0.0180 | 2.0421 | 0.5211 | NA | 0.0842 | 0.0000 | 0.0000 | 0.9895 | 0.8947 | 0.9947 | 0.0000 | 0.1368 | 0.9947 |
| 0.9252 | 0.0068 | 0.0408 | 0.0068 | 0.9456 | 0.5464 | 0.0759 | 1.9384 | 0.4692 | NA | 0.0884 | 0.0000 | 0.0000 | 0.8844 | 0.8435 | 0.9932 | 0.0000 | 0.7279 | 0.9796 |
| 0.9524 | 0.0000 | 0.0060 | 0.0893 | 0.9048 | 0.1859 | 0.0253 | 2.0000 | 0.5000 | NA | 0.0536 | 0.0000 | 0.0000 | 0.9048 | 0.8988 | 1.0000 | 0.0000 | 0.0060 | 0.9107 |
| 0.9505 | 0.0000 | 0.0495 | 0.2574 | 0.6931 | 0.4629 | 0.0549 | 1.9800 | 0.4900 | NA | 0.2772 | 0.0000 | 0.0000 | 0.6139 | 0.4851 | 0.9901 | 0.0000 | 0.0000 | 0.7327 |
| 0.8899 | 0.1009 | 0.1009 | 0.2018 | 0.6972 | 0.7959 | 0.0933 | 2.0099 | 0.5050 | NA | 0.2202 | 0.0000 | 0.0000 | 0.8440 | 0.3670 | 0.9266 | 0.0275 | 0.3028 | 0.8440 |
| 0.8559 | 0.1271 | 0.1356 | 0.4237 | 0.4322 | 0.4169 | 0.0585 | 1.6881 | 0.3440 | NA | 0.2288 | 0.0000 | 0.0000 | 0.4322 | 0.0763 | 0.9153 | 0.0085 | 0.0763 | 0.8729 |
| 0.8767 | 0.0205 | 0.0411 | 0.4863 | 0.4726 | 0.4007 | 0.0504 | 2.1087 | 0.5543 | NA | 0.1986 | 0.0000 | 0.0000 | 0.5342 | 0.3562 | 0.9452 | 0.0000 | 0.1301 | 0.9041 |
| 0.5052 | 0.0773 | 0.0928 | 0.2216 | 0.6856 | 0.2547 | 0.0370 | 1.7617 | 0.3808 | NA | 0.4948 | 0.0000 | 0.0000 | 0.7371 | 0.6186 | 0.9897 | 0.0000 | 0.0670 | 0.9845 |
| 1.0000 | 0.0000 | 0.0052 | 0.7461 | 0.2487 | 0.2687 | 0.0392 | 1.9297 | 0.4649 | NA | 0.0052 | 0.0000 | 0.0000 | 0.3005 | 0.1140 | 0.9585 | 0.0777 | 0.0777 | 0.9378 |
| 0.9595 | 0.0270 | 0.0270 | 0.1554 | 0.8108 | 0.6175 | 0.0852 | 1.1742 | 0.0871 | NA | 0.0676 | 0.0000 | 0.0000 | 0.1689 | 0.0473 | 0.8919 | 0.0000 | 0.0405 | 0.8649 |
| 1.0000 | 0.0000 | 0.0000 | 0.5922 | 0.3981 | 0.3716 | 0.0533 | 1.9901 | 0.4950 | NA | 0.0000 | 0.0000 | 0.0000 | 0.3981 | 0.1165 | 0.9806 | 0.0000 | 0.0874 | 0.8932 |
| 0.5918 | 0.2041 | 0.2092 | 0.2704 | 0.5204 | 0.3624 | 0.0525 | 1.7713 | 0.3856 | NA | 0.4286 | 0.0000 | 0.0102 | 0.7296 | 0.2449 | 0.9592 | 0.0102 | 0.0612 | 0.9082 |

| 26 inverb_1 | 27 mammals | 28 diversity | 28 diversity.s | 29 gr_CY | 29 gr_FO | 29 gr_PO | 29 gr_PT | 29 gr_SPH | 29 gr_BM | 30 pls_0 pere | 30 pls_1 ann | 31 pha_1 | 32 bry_1 | 33 st_c | 34 st_r | 35 st_s | 36 FDis | 36 FDiv |
|---|---|---|---|---|---|---|---|---|---|---|---|---|---|---|---|---|---|---|
| 0.0000 | 0.0000 | 2.1838 | 0.2368 | 0.4079 | 0.4408 | 0.0197 | 0.1053 | 0.0000 | 0.3347 | 1.0000 | 0.0000 | 0.0167 | 0.3473 | 0.3333 | 0.0716 | 0.5951 | 0.1121 | 0.7264 |
| 0.0000 | 0.0000 | 1.8739 | 0.1748 | 0.5082 | 0.4426 | 0.0246 | 0.0246 | 0.0000 | 0.4344 | 1.0000 | 0.0000 | 0.0136 | 0.4344 | 0.4477 | 0.0621 | 0.4901 | 0.0975 | 0.7725 |
| 0.0000 | 0.0000 | 2.8400 | 0.3680 | 0.6154 | 0.3077 | 0.0769 | 0.0000 | 0.0000 | 0.3500 | 1.0000 | 0.0000 | 0.0000 | 0.4583 | 0.4692 | 0.0769 | 0.4538 | 0.1372 | 0.7729 |
| 0.0000 | 0.0417 | 2.1111 | 0.2222 | 0.6389 | 0.2500 | 0.0833 | 0.0000 | 0.0000 | 0.5283 | 1.0000 | 0.0000 | 0.0000 | 0.5472 | 0.4977 | 0.0602 | 0.4421 | 0.0788 | 0.8589 |
| 0.0000 | 0.0316 | 1.9462 | 0.1892 | 0.5053 | 0.3474 | 0.0842 | 0.0211 | 0.0146 | 0.5146 | 1.0000 | 0.0000 | 0.0000 | 0.5388 | 0.4684 | 0.0632 | 0.4684 | 0.1001 | 0.8627 |
| 0.0000 | 0.0000 | 1.8333 | 0.1667 | 0.4000 | 0.5545 | 0.0273 | 0.0182 | 0.0000 | 0.2903 | 1.0000 | 0.0000 | 0.0000 | 0.2903 | 0.4860 | 0.0386 | 0.4754 | 0.0996 | 0.7563 |
| 0.0000 | 0.0182 | 1.9537 | 0.1907 | 0.4727 | 0.4636 | 0.0273 | 0.0182 | 0.0097 | 0.4320 | 1.0000 | 0.0000 | 0.0243 | 0.4417 | 0.4502 | 0.0436 | 0.5062 | 0.1023 | 0.7302 |
| 0.0211 | 0.0526 | 2.4286 | 0.2857 | 0.6211 | 0.2737 | 0.0211 | 0.0421 | 0.0000 | 0.5222 | 1.0000 | 0.0000 | 0.0099 | 0.5222 | 0.4611 | 0.0889 | 0.4500 | 0.1323 | 0.4882 |
| 0.0000 | 0.0417 | 2.3021 | 0.2604 | 0.6458 | 0.3125 | 0.0417 | 0.0000 | 0.0000 | 0.4628 | 1.0000 | 0.0000 | 0.0266 | 0.4628 | 0.4583 | 0.0708 | 0.4708 | 0.1225 | 0.4579 |
| 0.0000 | 0.0000 | 2.1058 | 0.2212 | 0.6321 | 0.3019 | 0.0189 | 0.0189 | 0.0000 | 0.4755 | 1.0000 | 0.0000 | 0.0049 | 0.4755 | 0.3553 | 0.1667 | 0.4780 | 0.0953 | 0.6839 |
| 0.0000 | 0.0000 | 2.1800 | 0.2360 | 0.4248 | 0.4071 | 0.0177 | 0.1150 | 0.0000 | 0.4364 | 1.0000 | 0.0000 | 0.0763 | 0.4449 | 0.3171 | 0.0295 | 0.6534 | 0.1117 | 0.5489 |
| 0.0000 | 0.0213 | 2.0233 | 0.2047 | 0.6064 | 0.3191 | 0.0000 | 0.0532 | 0.0000 | 0.4817 | 0.9681 | 0.0319 | 0.0262 | 0.4817 | 0.4529 | 0.0290 | 0.5181 | 0.1291 | 0.7940 |
| 0.0129 | 0.0129 | 2.1118 | 0.2224 | 0.3097 | 0.5484 | 0.0258 | 0.0194 | 0.0094 | 0.2956 | 1.0000 | 0.0000 | 0.0881 | 0.4245 | 0.4560 | 0.0667 | 0.4774 | 0.1113 | 0.7175 |
| 0.0719 | 0.2614 | 2.3493 | 0.2699 | 0.2614 | 0.6471 | 0.0458 | 0.0000 | 0.0000 | 0.2780 | 0.9804 | 0.0000 | 0.0179 | 0.2960 | 0.2800 | 0.1533 | 0.5667 | 0.1202 | 0.5755 |
| 0.0930 | 0.0698 | 2.5429 | 0.3086 | 0.3488 | 0.3953 | 0.0465 | 0.0465 | 0.0000 | 0.5556 | 0.9302 | 0.0000 | 0.0427 | 0.5897 | 0.3792 | 0.1417 | 0.4792 | 0.1123 | 0.7189 |
| 0.0230 | 0.0000 | 2.0706 | 0.2141 | 0.5632 | 0.3908 | 0.0230 | 0.0230 | 0.0000 | 0.4506 | 1.0000 | 0.0000 | 0.0123 | 0.4506 | 0.4702 | 0.0357 | 0.4940 | 0.0938 | 0.4362 |
| 0.1333 | 0.1429 | 2.8119 | 0.3624 | 0.3048 | 0.6095 | 0.0667 | 0.0000 | 0.0260 | 0.3646 | 0.9714 | 0.0286 | 0.0625 | 0.3906 | 0.3163 | 0.1582 | 0.5255 | 0.1158 | 0.7767 |
| 0.0899 | 0.0225 | 2.4940 | 0.2988 | 0.5506 | 0.3708 | 0.0337 | 0.0225 | 0.0000 | 0.5189 | 0.9775 | 0.0000 | 0.0000 | 0.5189 | 0.4518 | 0.1084 | 0.4398 | 0.1186 | 0.7372 |
| 0.0667 | 0.0000 | 2.8333 | 0.3667 | 0.5333 | 0.2556 | 0.1667 | 0.0000 | 0.0000 | 0.4866 | 0.9778 | 0.0000 | 0.0321 | 0.4866 | 0.4960 | 0.0437 | 0.4603 | 0.1444 | 0.8689 |
| 0.0160 | 0.0000 | 2.0410 | 0.2082 | 0.3680 | 0.5840 | 0.0240 | 0.0240 | 0.0000 | 0.3611 | 1.0000 | 0.0000 | 0.0000 | 0.4213 | 0.4840 | 0.0400 | 0.4760 | 0.1014 | 0.6601 |
| 0.0000 | 0.0000 | 2.2331 | 0.2466 | 0.1407 | 0.8222 | 0.0222 | 0.0148 | 0.0000 | 0.2100 | 1.0000 | 0.0000 | 0.0000 | 0.3836 | 0.4889 | 0.0370 | 0.4741 | 0.0849 | 0.7732 |
| 0.1031 | 0.0515 | 3.0745 | 0.4149 | 0.5464 | 0.2887 | 0.1340 | 0.0000 | 0.0000 | 0.4681 | 1.0000 | 0.0000 | 0.0160 | 0.4681 | 0.3842 | 0.1895 | 0.4263 | 0.1111 | 0.5481 |
| 0.0726 | 0.0323 | 2.7627 | 0.3525 | 0.6371 | 0.2581 | 0.0484 | 0.0161 | 0.0000 | 0.3774 | 0.9839 | 0.0000 | 0.0283 | 0.3868 | 0.3500 | 0.2250 | 0.4250 | 0.0942 | 0.5939 |
| 0.0570 | 0.0316 | 2.2971 | 0.2594 | 0.3481 | 0.4810 | 0.0253 | 0.1013 | 0.0000 | 0.3114 | 0.9873 | 0.0127 | 0.1099 | 0.3114 | 0.4494 | 0.0823 | 0.4684 | 0.1186 | 0.5669 |
| 0.0714 | 0.0000 | 2.8971 | 0.3794 | 0.5909 | 0.3247 | 0.0260 | 0.0325 | 0.0000 | 0.2819 | 0.9870 | 0.0130 | 0.0308 | 0.2907 | 0.3700 | 0.2000 | 0.4300 | 0.1143 | 0.3818 |
| 0.0462 | 0.0000 | 2.1685 | 0.2337 | 0.1308 | 0.4846 | 0.0385 | 0.2923 | 0.0000 | 0.2857 | 0.9769 | 0.0000 | 0.0265 | 0.2857 | 0.3278 | 0.0634 | 0.6088 | 0.0912 | 0.5971 |
| 0.0206 | 0.0515 | 2.3297 | 0.2659 | 0.3505 | 0.5052 | 0.0619 | 0.0619 | 0.0000 | 0.5324 | 1.0000 | 0.0000 | 0.0093 | 0.5417 | 0.4364 | 0.1271 | 0.4364 | 0.1105 | 0.6237 |
| 0.0263 | 0.0987 | 2.3897 | 0.2779 | 0.3553 | 0.5066 | 0.0263 | 0.1053 | 0.0000 | 0.3672 | 1.0000 | 0.0000 | 0.0273 | 0.3789 | 0.3222 | 0.1022 | 0.5756 | 0.1091 | 0.7065 |
| 0.0200 | 0.0600 | 2.5000 | 0.3000 | 0.4700 | 0.4300 | 0.0600 | 0.0200 | 0.0000 | 0.4757 | 1.0000 | 0.0000 | 0.0291 | 0.4854 | 0.4133 | 0.1020 | 0.4847 | 0.1256 | 0.7790 |
| 0.0000 | 0.0583 | 2.3663 | 0.2733 | 0.3398 | 0.5340 | 0.0874 | 0.0194 | 0.0000 | 0.4579 | 1.0000 | 0.0000 | 0.0000 | 0.5187 | 0.4806 | 0.0777 | 0.4417 | 0.1045 | 0.8622 |
| 0.0202 | 0.0000 | 2.6364 | 0.3273 | 0.3333 | 0.6465 | 0.0202 | 0.0000 | 0.0153 | 0.4541 | 1.0000 | 0.0000 | 0.0102 | 0.4847 | 0.4752 | 0.0496 | 0.4752 | 0.0756 | 0.7650 |
| 0.0504 | 0.1261 | 2.5625 | 0.3125 | 0.3950 | 0.4706 | 0.0168 | 0.0420 | 0.0213 | 0.4638 | 1.0000 | 0.0000 | 0.0085 | 0.4851 | 0.4181 | 0.1724 | 0.4095 | 0.1207 | 0.7030 |
| 0.0130 | 0.0325 | 2.0263 | 0.2053 | 0.3701 | 0.5390 | 0.0649 | 0.0130 | 0.0000 | 0.2684 | 1.0000 | 0.0000 | 0.0087 | 0.3247 | 0.4784 | 0.0758 | 0.4459 | 0.1063 | 0.8496 |
| 0.0435 | 0.0435 | 2.1087 | 0.2217 | 0.5543 | 0.3913 | 0.0435 | 0.0000 | 0.0000 | 0.4503 | 1.0000 | 0.0000 | 0.0117 | 0.4503 | 0.4837 | 0.0761 | 0.4402 | 0.1097 | 0.8289 |
| 0.0154 | 0.0154 | 2.3304 | 0.2661 | 0.3923 | 0.4077 | 0.0462 | 0.1000 | 0.0000 | 0.4132 | 0.9615 | 0.0000 | 0.0413 | 0.4215 | 0.4539 | 0.0476 | 0.4985 | 0.1208 | 0.8547 |
| 0.0000 | 0.0150 | 2.0538 | 0.2108 | 0.5414 | 0.3609 | 0.0752 | 0.0226 | 0.0000 | 0.3799 | 1.0000 | 0.0000 | 0.0306 | 0.3886 | 0.4925 | 0.0376 | 0.4699 | 0.1105 | 0.6488 |
| 0.0000 | 0.0459 | 2.2079 | 0.2416 | 0.2110 | 0.5413 | 0.1468 | 0.0734 | 0.4335 | 0.0246 | 1.0000 | 0.0000 | 0.0049 | 0.4581 | 0.4908 | 0.0459 | 0.4633 | 0.1072 | 0.6886 |
| 0.0000 | 0.0283 | 2.2718 | 0.2544 | 0.1604 | 0.6226 | 0.1132 | 0.0283 | 0.4314 | 0.0000 | 1.0000 | 0.0000 | 0.0490 | 0.4314 | 0.4471 | 0.0673 | 0.4856 | 0.1033 | 0.6239 |
| 0.0234 | 0.0781 | 2.3008 | 0.2602 | 0.5703 | 0.2969 | 0.0781 | 0.0391 | 0.0000 | 0.1216 | 1.0000 | 0.0000 | 0.0000 | 0.1351 | 0.5313 | 0.0703 | 0.3984 | 0.1154 | 0.8734 |
| 0.0957 | 0.0000 | 2.0089 | 0.2018 | 0.3217 | 0.2348 | 0.0435 | 0.0261 | 0.3060 | 0.0819 | 1.0000 | 0.0000 | 0.1164 | 0.3879 | 0.4507 | 0.0551 | 0.4942 | 0.1053 | 0.6374 |
| 0.0171 | 0.0171 | 1.9184 | 0.1837 | 0.0342 | 0.1966 | 0.0171 | 0.5812 | 0.4337 | 0.0321 | 1.0000 | 0.0000 | 0.0643 | 0.4659 | 0.4416 | 0.0228 | 0.5356 | 0.0788 | 0.3137 |
| 0.0421 | 0.0211 | 2.6667 | 0.3333 | 0.0947 | 0.1474 | 0.0421 | 0.7158 | 0.0000 | 0.4343 | 1.0000 | 0.0000 | 0.0229 | 0.4343 | 0.4964 | 0.0072 | 0.4964 | 0.0486 | 0.3276 |
| 0.0174 | 0.0000 | 2.3188 | 0.2638 | 0.2696 | 0.3304 | 0.0174 | 0.3652 | 0.3586 | 0.0000 | 1.0000 | 0.0000 | 0.0606 | 0.3586 | 0.3522 | 0.0348 | 0.6130 | 0.1021 | 0.6474 |
| 0.0000 | 0.0000 | 2.1809 | 0.2362 | 0.3333 | 0.2353 | 0.1765 | 0.0784 | 0.4492 | 0.0085 | 1.0000 | 0.0000 | 0.1102 | 0.4576 | 0.4792 | 0.0000 | 0.5208 | 0.1432 | 0.5222 |
| 0.0000 | 0.0000 | 2.1409 | 0.2282 | 0.5796 | 0.2293 | 0.0255 | 0.0510 | 0.3359 | 0.0078 | 1.0000 | 0.0000 | 0.0430 | 0.3438 | 0.4052 | 0.0523 | 0.5425 | 0.1048 | 0.3729 |
| 0.0000 | 0.0000 | 2.3962 | 0.2792 | 0.1852 | 0.7593 | 0.0370 | 0.0185 | 0.0000 | 0.3571 | 1.0000 | 0.0000 | 0.0000 | 0.3571 | 0.4777 | 0.0550 | 0.4674 | 0.1051 | 0.6414 |
| 0.0588 | 0.0735 | 2.6200 | 0.3240 | 0.2500 | 0.4559 | 0.0294 | 0.2647 | 0.5116 | 0.0174 | 0.9706 | 0.0294 | 0.0756 | 0.5291 | 0.4495 | 0.0556 | 0.4949 | 0.0968 | 0.5212 |
| 0.0523 | 0.0131 | 2.7067 | 0.3413 | 0.0654 | 0.3791 | 0.0261 | 0.5098 | 0.0000 | 0.3633 | 1.0000 | 0.0000 | 0.0000 | 0.3755 | 0.4499 | 0.1264 | 0.4237 | 0.0855 | 0.7632 |
| 0.0562 | 0.0000 | 2.3662 | 0.2732 | 0.3146 | 0.3596 | 0.0449 | 0.2022 | 0.4231 | 0.0625 | 1.0000 | 0.0000 | 0.0769 | 0.4952 | 0.4061 | 0.0613 | 0.5326 | 0.1154 | 0.8265 |
| 0.0238 | 0.0000 | 2.3293 | 0.2659 | 0.6071 | 0.3095 | 0.0595 | 0.0238 | 0.0000 | 0.3669 | 1.0000 | 0.0000 | 0.0288 | 0.3669 | 0.4797 | 0.0528 | 0.4675 | 0.1316 | 0.7437 |

| | | | | | | | | | | | | | | | | | | |
|---|---|---|---|---|---|---|---|---|---|---|---|---|---|---|---|---|---|---|
| 0.0138 | 0.0483 | 2.1449 | 0.2290 | 0.2345 | 0.3379 | 0.0759 | 0.0483 | 0.4030 | 0.0380 | 1.0000 | 0.0000 | 0.0076 | 0.4411 | 0.3839 | 0.0943 | 0.5218 | 0.1169 | 0.7674 |
| 0.0190 | 0.0286 | 1.7100 | 0.1420 | 0.6952 | 0.1810 | 0.0762 | 0.0476 | 0.0000 | 0.3312 | 1.0000 | 0.0000 | 0.0000 | 0.3312 | 0.5111 | 0.0444 | 0.4444 | 0.0683 | 0.9098 |
| 0.0413 | 0.0496 | 2.3398 | 0.2680 | 0.2479 | 0.5207 | 0.0165 | 0.1488 | 0.3534 | 0.0241 | 0.9835 | 0.0165 | 0.1285 | 0.3855 | 0.3567 | 0.0551 | 0.5882 | 0.1095 | 0.7751 |
| 0.0137 | 0.0000 | 2.3077 | 0.2615 | 0.5411 | 0.1849 | 0.0137 | 0.2603 | 0.0000 | 0.3304 | 1.0000 | 0.0000 | 0.0261 | 0.3391 | 0.4883 | 0.0093 | 0.5023 | 0.0854 | 0.3791 |
| 0.0935 | 0.0935 | 2.7009 | 0.3402 | 0.3271 | 0.4673 | 0.1402 | 0.0000 | 0.4115 | 0.0265 | 1.0000 | 0.0000 | 0.0885 | 0.4381 | 0.3146 | 0.1184 | 0.5670 | 0.1206 | 0.8027 |
| 0.0940 | 0.1007 | 2.3758 | 0.2752 | 0.2349 | 0.4362 | 0.0604 | 0.0000 | 0.3527 | 0.0233 | 1.0000 | 0.0000 | 0.0465 | 0.3760 | 0.3870 | 0.0917 | 0.5213 | 0.1146 | 0.8219 |
| 0.0185 | 0.0741 | 2.6476 | 0.3295 | 0.4444 | 0.4074 | 0.1204 | 0.0278 | 0.0000 | 0.4512 | 1.0000 | 0.0000 | 0.0093 | 0.4884 | 0.4352 | 0.1944 | 0.3704 | 0.0973 | 0.8086 |
| 0.0000 | 0.0783 | 2.5664 | 0.3133 | 0.4522 | 0.4261 | 0.1043 | 0.0174 | 0.0000 | 0.3438 | 1.0000 | 0.0000 | 0.0156 | 0.3854 | 0.4867 | 0.1150 | 0.3982 | 0.1110 | 0.8113 |
| 0.0331 | 0.1074 | 2.4370 | 0.2874 | 0.5785 | 0.2975 | 0.1074 | 0.0165 | 0.3982 | 0.0452 | 1.0000 | 0.0000 | 0.0090 | 0.4434 | 0.4421 | 0.0744 | 0.4835 | 0.1136 | 0.8607 |
| 0.0000 | 0.1412 | 2.7750 | 0.3550 | 0.2118 | 0.4471 | 0.2235 | 0.0588 | 0.0000 | 0.4588 | 1.0000 | 0.0000 | 0.0294 | 0.4706 | 0.5039 | 0.0980 | 0.3980 | 0.1142 | 0.6855 |
| 0.0000 | 0.0000 | 2.1067 | 0.2213 | 0.0000 | 0.0056 | 0.9944 | 0.0000 | 0.0000 | 0.0000 | 1.0000 | 0.0000 | 0.0000 | 0.0000 | 0.5028 | 0.0028 | 0.4944 | 0.0295 | 0.6667 |
| 0.0000 | 0.0000 | 2.0000 | 0.2000 | 0.0049 | 0.0591 | 0.9360 | 0.0000 | 0.0000 | 0.0000 | 1.0000 | 0.0000 | 0.0000 | 0.0000 | 0.5000 | 0.0296 | 0.4704 | 0.0381 | 0.9726 |
| 0.0000 | 0.0000 | 2.1633 | 0.2327 | 0.3628 | 0.0839 | 0.5533 | 0.0000 | 0.0000 | 0.0000 | 1.0000 | 0.0000 | 0.0000 | 0.0000 | 0.5272 | 0.0420 | 0.4308 | 0.0850 | 0.7982 |
| 0.0000 | 0.0000 | 3.6822 | 0.5364 | 0.0062 | 0.4330 | 0.5607 | 0.0000 | 0.0000 | 0.0000 | 1.0000 | 0.0000 | 0.0000 | 0.0000 | 0.7804 | 0.2165 | 0.0031 | 0.1904 | 0.7324 |
| 0.0000 | 0.0000 | 2.0000 | 0.2000 | 0.0058 | 0.0058 | 0.9884 | 0.0000 | 0.0000 | 0.0000 | 1.0000 | 0.0000 | 0.0000 | 0.0000 | 0.5000 | 0.0029 | 0.4971 | 0.0044 | 0.7543 |
| 0.0000 | 0.0000 | 2.0000 | 0.2000 | 0.0000 | 0.6795 | 0.3205 | 0.0000 | 0.0000 | 0.0000 | 1.0000 | 0.0000 | 0.0000 | 0.0000 | 0.5000 | 0.3397 | 0.1603 | 0.1525 | 0.5631 |
| 0.0000 | 0.0000 | 2.0000 | 0.2000 | 0.0000 | 0.3394 | 0.6606 | 0.0000 | 0.0000 | 0.0000 | 1.0000 | 0.0000 | 0.0000 | 0.0000 | 0.5000 | 0.1697 | 0.3303 | 0.1545 | 0.7811 |
| 0.0152 | 0.0000 | 4.7883 | 0.7577 | 0.0000 | 0.0914 | 0.9086 | 0.0000 | 0.0000 | 0.0000 | 1.0000 | 0.0000 | 0.0000 | 0.0000 | 0.8861 | 0.0468 | 0.0672 | 0.0816 | 0.8394 |
| 0.1858 | 0.0046 | 4.4793 | 0.6959 | 0.0046 | 0.2362 | 0.7592 | 0.0000 | 0.0000 | 0.0000 | 1.0000 | 0.0000 | 0.0000 | 0.0000 | 0.8925 | 0.0445 | 0.0630 | 0.1137 | 0.7616 |
| 0.0210 | 0.0335 | 4.8784 | 0.7757 | 0.0000 | 0.0252 | 0.9748 | 0.0000 | 0.0000 | 0.0000 | 1.0000 | 0.0000 | 0.0000 | 0.0000 | 0.8728 | 0.0636 | 0.0636 | 0.0616 | 0.8607 |
| 0.0378 | 0.0420 | 4.5654 | 0.7131 | 0.0000 | 0.1534 | 0.8466 | 0.0000 | 0.0000 | 0.0000 | 0.9706 | 0.0294 | 0.0000 | 0.0000 | 0.8038 | 0.1034 | 0.0928 | 0.0886 | 0.7810 |
| 0.0110 | 0.0037 | 4.1042 | 0.6208 | 0.0037 | 0.0658 | 0.9305 | 0.0000 | 0.0000 | 0.0000 | 0.9817 | 0.0183 | 0.0000 | 0.0000 | 0.7374 | 0.0646 | 0.1980 | 0.0686 | 0.8971 |
| 0.0959 | 0.0104 | 4.6762 | 0.7352 | 0.0000 | 0.2176 | 0.7824 | 0.0000 | 0.0000 | 0.0000 | 0.9896 | 0.0104 | 0.0000 | 0.0000 | 0.8429 | 0.0668 | 0.0903 | 0.0741 | 0.8770 |
| 0.0787 | 0.0037 | 4.6734 | 0.7347 | 0.0000 | 0.2016 | 0.7984 | 0.0000 | 0.0000 | 0.0000 | 1.0000 | 0.0000 | 0.0000 | 0.0000 | 0.7880 | 0.0996 | 0.1125 | 0.1007 | 0.8202 |
| 0.0000 | 0.0417 | 2.0870 | 0.2174 | 0.2500 | 0.6667 | 0.0417 | 0.0417 | 0.0000 | 0.2000 | 1.0000 | 0.0000 | 0.0000 | 0.2000 | 0.4815 | 0.0648 | 0.4537 | 0.1850 | 0.8732 |
| 0.0000 | 0.0625 | 2.0667 | 0.2133 | 0.5000 | 0.3125 | 0.1250 | 0.0625 | 0.0000 | 0.2264 | 1.0000 | 0.0000 | 0.4717 | 0.2264 | 0.4271 | 0.0833 | 0.4896 | 0.0906 | 0.7646 |
| 0.0244 | 0.0244 | 2.9000 | 0.3800 | 0.6585 | 0.1463 | 0.1707 | 0.0244 | 0.0000 | 0.3731 | 1.0000 | 0.0000 | 0.0149 | 0.3731 | 0.4583 | 0.0833 | 0.4583 | 0.0863 | 0.6478 |
| 0.0000 | 0.0000 | 2.5000 | 0.3000 | 0.2000 | 0.4000 | 0.2000 | 0.2000 | 0.0000 | 0.6757 | 1.0000 | 0.0000 | 0.1892 | 0.6757 | 0.4583 | 0.0833 | 0.4583 | 0.1011 | 0.7073 |
| 0.0488 | 0.0488 | 2.4750 | 0.2950 | 0.6341 | 0.2195 | 0.0976 | 0.0244 | 0.0000 | 0.3514 | 0.9756 | 0.0244 | 0.0946 | 0.3514 | 0.2083 | 0.0458 | 0.7458 | 0.1225 | 0.4329 |
| 0.0233 | 0.0465 | 2.4524 | 0.2905 | 0.6512 | 0.2326 | 0.0930 | 0.0233 | 0.0000 | 0.0984 | 0.9767 | 0.0233 | 0.0984 | 0.1967 | 0.2183 | 0.0516 | 0.7302 | 0.1053 | 0.7707 |
| 0.0175 | 0.1228 | 2.1607 | 0.2321 | 0.7018 | 0.1404 | 0.1404 | 0.0175 | 0.0000 | 0.3218 | 0.9825 | 0.0175 | 0.0115 | 0.3333 | 0.2411 | 0.0982 | 0.6607 | 0.1117 | 0.8454 |
| 0.0833 | 0.3333 | 3.0870 | 0.4174 | 0.3333 | 0.3333 | 0.2917 | 0.0417 | 0.0000 | 0.3289 | 0.9583 | 0.0417 | 0.3421 | 0.3421 | 0.3768 | 0.1377 | 0.4855 | 0.1253 | 0.7273 |
| 0.0000 | 0.1000 | 3.1111 | 0.4222 | 0.1000 | 0.6000 | 0.2000 | 0.1000 | 0.0000 | 0.4032 | 0.9000 | 0.1000 | 0.4355 | 0.4032 | 0.4444 | 0.1667 | 0.3889 | 0.1103 | 0.6988 |
| 0.0500 | 0.0500 | 2.6842 | 0.3368 | 0.1000 | 0.4000 | 0.4500 | 0.0500 | 0.0000 | 0.3562 | 0.9000 | 0.1000 | 0.3562 | 0.3699 | 0.4649 | 0.1228 | 0.4123 | 0.1003 | 0.6890 |
| 0.0789 | 0.1842 | 3.2162 | 0.4432 | 0.3684 | 0.3947 | 0.2105 | 0.0263 | 0.0000 | 0.2708 | 0.9737 | 0.0263 | 0.3229 | 0.2813 | 0.4740 | 0.1302 | 0.3958 | 0.1134 | 0.6496 |
| 0.0750 | 0.0500 | 2.6923 | 0.3385 | 0.4750 | 0.3000 | 0.2000 | 0.0250 | 0.0000 | 0.3425 | 0.9750 | 0.0250 | 0.0959 | 0.3562 | 0.4231 | 0.1026 | 0.4744 | 0.1159 | 0.7296 |
| 0.0625 | 0.4375 | 3.2667 | 0.4533 | 0.1250 | 0.3750 | 0.4375 | 0.0625 | 0.0000 | 0.3731 | 0.9375 | 0.0625 | 0.3881 | 0.3731 | 0.4889 | 0.1889 | 0.3222 | 0.1035 | 0.9009 |
| 0.0000 | 0.0000 | 1.3438 | 0.0688 | 0.6579 | 0.1579 | 0.0263 | 0.1579 | 0.0000 | 0.3906 | 0.9737 | 0.0263 | 0.0156 | 0.3906 | 0.4910 | 0.0450 | 0.4640 | 0.0598 | 0.8111 |
| 0.0313 | 0.0000 | 1.3871 | 0.0774 | 0.7813 | 0.1250 | 0.0625 | 0.0313 | 0.0000 | 0.4310 | 1.0000 | 0.0000 | 0.0172 | 0.4310 | 0.4896 | 0.0208 | 0.4896 | 0.0420 | 0.7550 |
| 0.0250 | 0.0000 | 1.6667 | 0.1333 | 0.7750 | 0.1500 | 0.0500 | 0.0250 | 0.0000 | 0.3788 | 0.9750 | 0.0250 | 0.0152 | 0.3788 | 0.4853 | 0.0441 | 0.4706 | 0.0765 | 0.7843 |
| 0.1250 | 0.1250 | 3.0000 | 0.4000 | 0.1250 | 0.5000 | 0.2500 | 0.1250 | 0.0000 | 0.6341 | 0.8750 | 0.1250 | 0.1707 | 0.6341 | 0.4583 | 0.1458 | 0.3958 | 0.1130 | 0.6929 |
| 0.0172 | 0.0000 | 2.0702 | 0.2140 | 0.4310 | 0.1034 | 0.4483 | 0.0172 | 0.0000 | 0.2976 | 0.9655 | 0.0345 | 0.0119 | 0.2976 | 0.4914 | 0.0345 | 0.4741 | 0.1018 | 0.8254 |
| 0.0667 | 0.5778 | 3.5455 | 0.5091 | 0.1778 | 0.7556 | 0.0444 | 0.0222 | 0.0000 | 0.3523 | 0.9778 | 0.0222 | 0.0682 | 0.4205 | 0.8205 | 0.0641 | 0.1154 | 0.0970 | 0.8071 |
| 0.0541 | 0.0541 | 1.5833 | 0.1167 | 0.7027 | 0.2432 | 0.0270 | 0.0270 | 0.0000 | 0.3714 | 1.0000 | 0.0000 | 0.1000 | 0.3714 | 0.4954 | 0.0370 | 0.4676 | 0.0618 | 0.7871 |
| 0.0476 | 0.0000 | 1.7805 | 0.1561 | 0.6190 | 0.1905 | 0.1667 | 0.0238 | 0.0000 | 0.3676 | 0.9762 | 0.0238 | 0.0147 | 0.3676 | 0.4921 | 0.0278 | 0.4802 | 0.0828 | 0.6306 |
| 0.0500 | 0.0000 | 2.3684 | 0.2737 | 0.3500 | 0.2500 | 0.3500 | 0.0500 | 0.0000 | 0.5435 | 0.9500 | 0.0500 | 0.0217 | 0.5435 | 0.4833 | 0.0583 | 0.4583 | 0.1140 | 0.7238 |
| 0.0556 | 0.5926 | 3.7170 | 0.5434 | 0.0185 | 0.4444 | 0.5185 | 0.0185 | 0.0000 | 0.3750 | 0.9444 | 0.0556 | 0.0114 | 0.3750 | 0.4906 | 0.2358 | 0.2736 | 0.0987 | 0.9363 |
| 0.0400 | 0.6200 | 3.6939 | 0.5388 | 0.0200 | 0.7800 | 0.1800 | 0.0200 | 0.0000 | 0.3690 | 0.9600 | 0.0400 | 0.0238 | 0.3810 | 0.7313 | 0.1088 | 0.1599 | 0.1078 | 0.8591 |
| 0.0465 | 0.0465 | 2.6429 | 0.3286 | 0.5814 | 0.3023 | 0.0930 | 0.0233 | 0.0000 | 0.4286 | 0.9767 | 0.0233 | 0.0130 | 0.4286 | 0.5155 | 0.0736 | 0.4109 | 0.0745 | 0.9193 |
| 0.1053 | 0.2632 | 3.1081 | 0.4216 | 0.2105 | 0.5263 | 0.2368 | 0.0263 | 0.0000 | 0.4063 | 0.9474 | 0.0526 | 0.0000 | 0.4063 | 0.5000 | 0.1622 | 0.3378 | 0.1127 | 0.8272 |
| 0.0556 | 0.1111 | 2.2941 | 0.2588 | 0.5000 | 0.2778 | 0.1667 | 0.0556 | 0.0000 | 0.4902 | 0.9444 | 0.0556 | 0.1373 | 0.5098 | 0.5294 | 0.0882 | 0.3824 | 0.0878 | 0.6514 |

| | | | | | | | | | | | | | | | | | | |
|---|---|---|---|---|---|---|---|---|---|---|---|---|---|---|---|---|---|---|
| 0.0313 | 0.2188 | 3.0645 | 0.4129 | 0.3750 | 0.4063 | 0.1875 | 0.0313 | 0.0000 | 0.4407 | 0.9375 | 0.0625 | 0.0169 | 0.4407 | 0.4679 | 0.1795 | 0.3526 | 0.1048 | 0.8774 |
| 0.0435 | 0.7246 | 3.7941 | 0.5588 | 0.0435 | 0.5652 | 0.3768 | 0.0145 | 0.0000 | 0.0732 | 0.9855 | 0.0145 | 0.0732 | 0.0854 | 0.6152 | 0.1593 | 0.2255 | 0.1036 | 0.9441 |
| 0.0938 | 0.1250 | 3.1613 | 0.4323 | 0.3750 | 0.4375 | 0.1563 | 0.0313 | 0.0000 | 0.4407 | 0.9375 | 0.0625 | 0.0000 | 0.4576 | 0.5513 | 0.1282 | 0.3205 | 0.1039 | 0.8084 |
| 0.0000 | 0.0690 | 2.6316 | 0.3263 | 0.5690 | 0.2414 | 0.1724 | 0.0172 | 0.0000 | 0.2941 | 0.9655 | 0.0345 | 0.0235 | 0.2941 | 0.5577 | 0.0769 | 0.3654 | 0.0865 | 0.8870 |
| 0.0351 | 0.4561 | 3.5893 | 0.5179 | 0.1754 | 0.6491 | 0.1579 | 0.0175 | 0.0000 | 0.3059 | 0.9825 | 0.0175 | 0.0235 | 0.3059 | 0.7059 | 0.1078 | 0.1863 | 0.1054 | 0.8391 |
| 0.0000 | 0.0000 | 2.0244 | 0.2049 | 0.8333 | 0.1190 | 0.0238 | 0.0238 | 0.0000 | 0.3951 | 0.9762 | 0.0238 | 0.0864 | 0.3951 | 0.4837 | 0.0203 | 0.4959 | 0.0650 | 0.7787 |
| 0.0000 | 0.0526 | 2.2105 | 0.2421 | 0.6316 | 0.3158 | 0.0526 | 0.0000 | 0.0000 | 0.5192 | 0.9474 | 0.0526 | 0.1154 | 0.5192 | 0.4386 | 0.1491 | 0.4123 | 0.0889 | 0.6906 |
| 0.0000 | 0.0435 | 3.0000 | 0.4000 | 0.3913 | 0.4783 | 0.0870 | 0.0435 | 0.0000 | 0.6375 | 0.9565 | 0.0435 | 0.0750 | 0.6375 | 0.4762 | 0.0952 | 0.4286 | 0.1445 | 0.7333 |
| 0.0000 | 0.0000 | 2.2903 | 0.2581 | 0.5000 | 0.4375 | 0.0313 | 0.0313 | 0.0000 | 0.3529 | 1.0000 | 0.0000 | 0.0196 | 0.3529 | 0.4444 | 0.0944 | 0.4611 | 0.1073 | 0.5059 |
| 0.0000 | 0.1000 | 2.8000 | 0.3600 | 0.4000 | 0.6000 | 0.0000 | 0.0000 | 0.0000 | 0.5882 | 0.9000 | 0.1000 | 0.2941 | 0.5882 | 0.4630 | 0.1296 | 0.4074 | 0.1282 | 0.6083 |
| 0.0000 | 0.0256 | 2.0000 | 0.2000 | 0.8205 | 0.1538 | 0.0000 | 0.0256 | 0.0000 | 0.3939 | 1.0000 | 0.0000 | 0.0152 | 0.3939 | 0.3816 | 0.2368 | 0.3816 | 0.0578 | 0.6586 |
| 0.0000 | 0.3077 | 2.7308 | 0.3462 | 0.5000 | 0.2308 | 0.2692 | 0.0000 | 0.0000 | 0.5333 | 1.0000 | 0.0000 | 0.0333 | 0.5333 | 0.3141 | 0.1795 | 0.5064 | 0.0941 | 0.8458 |
| 0.0000 | 0.0690 | 2.3929 | 0.2786 | 0.6552 | 0.2759 | 0.0345 | 0.0345 | 0.0000 | 0.4706 | 1.0000 | 0.0000 | 0.1029 | 0.4706 | 0.3563 | 0.1149 | 0.5287 | 0.1034 | 0.6432 |
| 0.0217 | 0.1739 | 3.2826 | 0.4565 | 0.5870 | 0.2174 | 0.1957 | 0.0000 | 0.0000 | 0.4524 | 1.0000 | 0.0000 | 0.0000 | 0.4524 | 0.4778 | 0.0889 | 0.4333 | 0.1112 | 0.5959 |
| 0.0169 | 0.1695 | 3.7627 | 0.5525 | 0.1356 | 0.6610 | 0.1864 | 0.0000 | 0.0000 | 0.1757 | 1.0000 | 0.0000 | 0.0270 | 0.1757 | 0.4167 | 0.2529 | 0.3305 | 0.0996 | 0.6640 |
| 0.0167 | 0.1500 | 3.6610 | 0.5322 | 0.1333 | 0.6167 | 0.2333 | 0.0167 | 0.0000 | 0.3441 | 1.0000 | 0.0000 | 0.0108 | 0.3441 | 0.4040 | 0.2260 | 0.3701 | 0.1089 | 0.6477 |
| 0.0278 | 0.1250 | 2.9848 | 0.3970 | 0.5694 | 0.3056 | 0.1250 | 0.0000 | 0.0000 | 0.2673 | 1.0000 | 0.0000 | 0.0099 | 0.2772 | 0.2778 | 0.1263 | 0.5960 | 0.0986 | 0.6627 |
| 0.0328 | 0.4262 | 3.4833 | 0.4967 | 0.4426 | 0.1311 | 0.4098 | 0.0164 | 0.0000 | 0.3168 | 1.0000 | 0.0000 | 0.0792 | 0.3168 | 0.3843 | 0.2593 | 0.3565 | 0.0505 | 0.7452 |
| 0.0230 | 0.1149 | 3.6977 | 0.5395 | 0.0345 | 0.2874 | 0.6667 | 0.0115 | 0.0000 | 0.2368 | 0.9770 | 0.0230 | 0.0000 | 0.2368 | 0.6143 | 0.1841 | 0.2016 | 0.1143 | 0.5861 |
| 0.0682 | 0.6136 | 3.7907 | 0.5581 | 0.0682 | 0.2500 | 0.6591 | 0.0227 | 0.0000 | 0.4231 | 1.0000 | 0.0000 | 0.0128 | 0.4231 | 0.4109 | 0.2481 | 0.3411 | 0.0913 | 0.9051 |
| 0.0000 | 0.3333 | 3.3500 | 0.4700 | 0.2381 | 0.3333 | 0.3810 | 0.0476 | 0.0000 | 0.7073 | 1.0000 | 0.0000 | 0.0244 | 0.7195 | 0.4667 | 0.1667 | 0.3667 | 0.1111 | 0.8168 |
| 0.0000 | 0.0769 | 2.3200 | 0.2640 | 0.5385 | 0.3462 | 0.0769 | 0.0385 | 0.0000 | 0.3810 | 0.9615 | 0.0385 | 0.3095 | 0.3810 | 0.3800 | 0.0800 | 0.5400 | 0.1293 | 0.7681 |
| 0.0345 | 0.2069 | 2.7241 | 0.3448 | 0.5862 | 0.2069 | 0.2069 | 0.0000 | 0.0000 | 0.5862 | 1.0000 | 0.0000 | 0.0805 | 0.5862 | 0.3452 | 0.0952 | 0.5595 | 0.1159 | 0.6430 |
| 0.0000 | 0.2692 | 2.7692 | 0.3538 | 0.5385 | 0.1538 | 0.3077 | 0.0000 | 0.0000 | 0.5781 | 0.9615 | 0.0385 | 0.0156 | 0.5781 | 0.3067 | 0.2067 | 0.4867 | 0.0961 | 0.7224 |
| 0.0000 | 0.4000 | 2.9077 | 0.3815 | 0.5077 | 0.0923 | 0.4000 | 0.0000 | 0.0000 | 0.3232 | 0.9846 | 0.0154 | 0.0202 | 0.3232 | 0.4167 | 0.1745 | 0.4089 | 0.1042 | 0.8646 |
| 0.0000 | 0.0256 | 2.3333 | 0.2667 | 0.7692 | 0.2308 | 0.0000 | 0.0000 | 0.0000 | 0.4348 | 1.0000 | 0.0000 | 0.2261 | 0.4348 | 0.5000 | 0.0000 | 0.5000 | 0.0757 | 0.7516 |
| 0.0000 | 0.3968 | 2.8254 | 0.3651 | 0.5397 | 0.0476 | 0.3968 | 0.0000 | 0.0000 | 0.3643 | 1.0000 | 0.0000 | 0.1857 | 0.3643 | 0.2339 | 0.1452 | 0.6210 | 0.0959 | 0.9531 |
| 0.0145 | 0.3768 | 2.9710 | 0.3942 | 0.4348 | 0.1739 | 0.3913 | 0.0000 | 0.0000 | 0.2692 | 1.0000 | 0.0000 | 0.0673 | 0.2692 | 0.2525 | 0.1422 | 0.6054 | 0.0941 | 0.8998 |
| 0.0000 | 0.0400 | 2.3600 | 0.2720 | 0.6800 | 0.2400 | 0.0800 | 0.0000 | 0.0000 | 0.6265 | 0.9200 | 0.0800 | 0.0723 | 0.6265 | 0.3194 | 0.1528 | 0.5278 | 0.0975 | 0.7908 |
| 0.0172 | 0.1034 | 3.5614 | 0.5123 | 0.0690 | 0.4828 | 0.4310 | 0.0172 | 0.0000 | 0.2941 | 1.0000 | 0.0000 | 0.0118 | 0.3059 | 0.3889 | 0.2310 | 0.3801 | 0.0973 | 0.8528 |
| 0.0750 | 0.3000 | 3.3077 | 0.4615 | 0.2250 | 0.5750 | 0.1750 | 0.0250 | 0.0000 | 0.3514 | 0.9750 | 0.0250 | 0.0946 | 0.3649 | 0.5085 | 0.1752 | 0.3162 | 0.1106 | 0.8173 |
| 0.0370 | 0.2222 | 2.8462 | 0.3692 | 0.2963 | 0.5556 | 0.1111 | 0.0370 | 0.0000 | 0.1970 | 1.0000 | 0.0000 | 0.3788 | 0.2121 | 0.6346 | 0.0769 | 0.2885 | 0.1126 | 0.8431 |
| 0.0313 | 0.0625 | 3.0000 | 0.4000 | 0.3750 | 0.3438 | 0.2500 | 0.0313 | 0.0000 | 0.3939 | 0.9063 | 0.0938 | 0.1061 | 0.4091 | 0.5064 | 0.1218 | 0.3718 | 0.1295 | 0.5113 |
| 0.0000 | 0.0000 | 2.4091 | 0.2818 | 0.1071 | 0.6429 | 0.0357 | 0.2143 | 0.0000 | 0.4630 | 0.9643 | 0.0357 | 0.0185 | 0.4630 | 0.4773 | 0.1136 | 0.4091 | 0.1858 | 0.8447 |
| 0.0000 | 0.0435 | 2.3182 | 0.2636 | 0.3478 | 0.5217 | 0.0870 | 0.0435 | 0.0000 | 0.3425 | 1.0000 | 0.0000 | 0.3425 | 0.3425 | 0.4603 | 0.0556 | 0.4841 | 0.1284 | 0.8604 |
| 0.0476 | 0.0000 | 2.5500 | 0.3100 | 0.3810 | 0.4762 | 0.0952 | 0.0476 | 0.0000 | 0.4717 | 1.0000 | 0.0000 | 0.1321 | 0.4717 | 0.4917 | 0.0167 | 0.4917 | 0.1036 | 0.6697 |
| 0.0000 | 0.0714 | 3.0769 | 0.4154 | 0.0000 | 0.4286 | 0.5000 | 0.0714 | 0.0000 | 0.3846 | 0.9286 | 0.0714 | 0.4000 | 0.3846 | 0.5119 | 0.0833 | 0.4048 | 0.1174 | 0.6940 |
| 0.3816 | 0.1316 | 4.2000 | 0.6400 | 0.0000 | 0.8421 | 0.1447 | 0.0132 | 0.0000 | 0.0000 | 0.8816 | 0.1184 | 0.0000 | 0.0000 | 0.4781 | 0.2939 | 0.2281 | 0.1094 | 0.5917 |
| 0.0000 | 0.7000 | 4.5750 | 0.7150 | 0.0250 | 0.2500 | 0.7250 | 0.0000 | 0.0000 | 0.0000 | 0.9750 | 0.0250 | 0.0000 | 0.0000 | 0.8125 | 0.0875 | 0.1000 | 0.0680 | 0.8190 |
| 0.0164 | 0.0000 | 2.5833 | 0.3167 | 0.0492 | 0.9180 | 0.0164 | 0.0164 | 0.0000 | 0.0000 | 1.0000 | 0.0000 | 0.0000 | 0.0000 | 0.4809 | 0.0219 | 0.4973 | 0.0611 | 0.6755 |
| 0.0192 | 0.2115 | 4.0196 | 0.6039 | 0.0000 | 0.8077 | 0.1731 | 0.0192 | 0.0000 | 0.0000 | 0.4038 | 0.5962 | 0.0189 | 0.0000 | 0.4904 | 0.2788 | 0.2308 | 0.0769 | 0.6468 |
| 0.0476 | 0.6905 | 4.4146 | 0.6829 | 0.0000 | 0.3095 | 0.6667 | 0.0238 | 0.0000 | 0.0000 | 0.9762 | 0.0238 | 0.0233 | 0.0000 | 0.7857 | 0.1071 | 0.1071 | 0.0784 | 0.7330 |
| 0.1148 | 0.4918 | 3.9833 | 0.5967 | 0.0000 | 0.8689 | 0.1148 | 0.0164 | 0.0000 | 0.0000 | 0.8197 | 0.1803 | 0.0000 | 0.0000 | 0.7240 | 0.1667 | 0.1093 | 0.1010 | 0.8702 |
| 0.0377 | 0.5849 | 4.0943 | 0.6189 | 0.0000 | 0.8113 | 0.1887 | 0.0000 | 0.0000 | 0.0000 | 0.9245 | 0.0755 | 0.0000 | 0.0000 | 0.7170 | 0.1509 | 0.1321 | 0.0796 | 0.8833 |
| 0.3472 | 0.7361 | 3.5070 | 0.5014 | 0.0000 | 0.5278 | 0.4583 | 0.0139 | 0.0000 | 0.0000 | 0.9028 | 0.0972 | 0.0137 | 0.0000 | 0.6181 | 0.2083 | 0.1736 | 0.1056 | 0.8701 |
| 0.3718 | 0.1410 | 4.1948 | 0.6390 | 0.0000 | 0.8333 | 0.1538 | 0.0128 | 0.0000 | 0.0000 | 0.8718 | 0.1282 | 0.0000 | 0.0000 | 0.4765 | 0.2970 | 0.2265 | 0.1094 | 0.6039 |
| 0.1324 | 0.2794 | 4.2941 | 0.6588 | 0.0000 | 0.4412 | 0.5588 | 0.0000 | 0.0000 | 0.0000 | 0.8382 | 0.1618 | 0.0000 | 0.0000 | 0.4608 | 0.3137 | 0.2255 | 0.0929 | 0.8311 |
| 0.0426 | 0.6170 | 4.4468 | 0.6894 | 0.0000 | 0.3830 | 0.6170 | 0.0000 | 0.0000 | 0.0000 | 0.8723 | 0.1277 | 0.0000 | 0.0000 | 0.7553 | 0.1170 | 0.1277 | 0.0761 | 0.8324 |
| 0.0000 | 0.7105 | 4.5405 | 0.7081 | 0.0263 | 0.2105 | 0.7368 | 0.0263 | 0.0000 | 0.0000 | 0.9737 | 0.0263 | 0.0256 | 0.0000 | 0.8202 | 0.0702 | 0.1096 | 0.0730 | 0.8015 |
| 0.5652 | 0.0870 | 3.9778 | 0.5956 | 0.0000 | 0.9130 | 0.0652 | 0.0217 | 0.0000 | 0.0000 | 0.8478 | 0.1522 | 0.0000 | 0.0000 | 0.3696 | 0.3261 | 0.3043 | 0.0833 | 0.5958 |
| 0.0196 | 0.0392 | 1.9800 | 0.1960 | 0.7255 | 0.2157 | 0.0392 | 0.0196 | 0.0000 | 0.0000 | 0.9804 | 0.0196 | 0.0192 | 0.0000 | 0.4804 | 0.0294 | 0.4902 | 0.1068 | 0.7430 |

| | | | | | | | | | | | | | | | | | | |
|---|---|---|---|---|---|---|---|---|---|---|---|---|---|---|---|---|---|---|
| 0.0612 | 0.1633 | 2.3333 | 0.2667 | 0.5306 | 0.2857 | 0.1633 | 0.0204 | 0.0000 | 0.0000 | 1.0000 | 0.0000 | 0.0000 | 0.0000 | 0.6122 | 0.0408 | 0.3469 | 0.0841 | 0.7840 |
| 0.0392 | 0.5490 | 3.9388 | 0.5878 | 0.1569 | 0.2549 | 0.5686 | 0.0196 | 0.0000 | 0.0000 | 1.0000 | 0.0000 | 0.0000 | 0.0000 | 0.8268 | 0.0327 | 0.1405 | 0.0801 | 0.5350 |
| 0.0392 | 0.0196 | 3.0600 | 0.4120 | 0.6275 | 0.3137 | 0.0392 | 0.0196 | 0.0000 | 0.0000 | 1.0000 | 0.0000 | 0.0192 | 0.0000 | 0.5098 | 0.0392 | 0.4510 | 0.1306 | 0.5300 |
| 0.0000 | 0.0000 | 3.0652 | 0.4130 | 0.5957 | 0.3617 | 0.0213 | 0.0213 | 0.0000 | 0.0000 | 1.0000 | 0.0000 | 0.0000 | 0.0000 | 0.5000 | 0.0217 | 0.4783 | 0.1309 | 0.5150 |
| 0.0222 | 0.0444 | 3.0455 | 0.4091 | 0.1778 | 0.7556 | 0.0444 | 0.0222 | 0.0000 | 0.0000 | 0.9778 | 0.0222 | 0.0217 | 0.0000 | 0.5000 | 0.0556 | 0.4444 | 0.0738 | 0.6259 |
| 0.0182 | 0.5818 | 4.1321 | 0.6264 | 0.0727 | 0.3091 | 0.6000 | 0.0182 | 0.0000 | 0.0000 | 1.0000 | 0.0000 | 0.0179 | 0.0000 | 0.8545 | 0.0364 | 0.1091 | 0.0930 | 0.5619 |
| 0.0290 | 0.4203 | 3.7941 | 0.5588 | 0.1014 | 0.3913 | 0.4928 | 0.0145 | 0.0000 | 0.0000 | 0.9855 | 0.0145 | 0.0143 | 0.0000 | 0.7609 | 0.0797 | 0.1594 | 0.1021 | 0.6748 |
| 0.0204 | 0.0612 | 2.2917 | 0.2583 | 0.6735 | 0.2857 | 0.0204 | 0.0204 | 0.0000 | 0.0000 | 1.0000 | 0.0000 | 0.0000 | 0.0000 | 0.5136 | 0.0544 | 0.4320 | 0.0936 | 0.6322 |
| 0.0303 | 0.2424 | 3.0000 | 0.4000 | 0.2121 | 0.3939 | 0.3636 | 0.0303 | 0.0000 | 0.0000 | 1.0000 | 0.0000 | 0.0000 | 0.0000 | 0.6212 | 0.0303 | 0.3485 | 0.1034 | 0.6696 |
| 0.0179 | 0.5893 | 3.7857 | 0.5571 | 0.2143 | 0.3214 | 0.4643 | 0.0000 | 0.0000 | 0.0000 | 1.0000 | 0.0000 | 0.0000 | 0.0000 | 0.7411 | 0.0357 | 0.2232 | 0.0872 | 0.7675 |
| 0.0000 | 0.0000 | 2.8387 | 0.3677 | 0.0938 | 0.8750 | 0.0000 | 0.0313 | 0.0000 | 0.0588 | 0.9688 | 0.0313 | 0.0000 | 0.0588 | 0.4740 | 0.0365 | 0.4896 | 0.0401 | 0.2915 |
| 0.0000 | 0.0000 | 2.1034 | 0.2207 | 0.0333 | 0.9333 | 0.0000 | 0.0333 | 0.0000 | 0.0882 | 1.0000 | 0.0000 | 0.0294 | 0.0882 | 0.5000 | 0.0167 | 0.4833 | 0.0440 | 0.4797 |
| 0.0192 | 0.0192 | 2.5556 | 0.3111 | 0.2500 | 0.2115 | 0.0577 | 0.4808 | 0.0000 | 0.1290 | 1.0000 | 0.0000 | 0.0323 | 0.1290 | 0.4487 | 0.0545 | 0.4968 | 0.0981 | 0.7883 |
| 0.0323 | 0.1935 | 3.4000 | 0.4800 | 0.2581 | 0.4516 | 0.2581 | 0.0323 | 0.0000 | 0.0263 | 0.9677 | 0.0323 | 0.1579 | 0.0263 | 0.5108 | 0.1237 | 0.3656 | 0.1152 | 0.7725 |
| 0.0000 | 0.0000 | 1.5106 | 0.1021 | 0.1509 | 0.6226 | 0.1132 | 0.1132 | 0.0000 | 0.0185 | 1.0000 | 0.0000 | 0.0000 | 0.0185 | 0.4874 | 0.0346 | 0.4780 | 0.0691 | 0.8658 |
| 0.0250 | 0.2500 | 3.1282 | 0.4256 | 0.3500 | 0.4000 | 0.2250 | 0.0250 | 0.0000 | 0.4118 | 1.0000 | 0.0000 | 0.0000 | 0.4118 | 0.5375 | 0.1250 | 0.3375 | 0.1058 | 0.7719 |
| 0.0161 | 0.1290 | 2.8852 | 0.3770 | 0.5161 | 0.2258 | 0.2419 | 0.0161 | 0.0000 | 0.3922 | 1.0000 | 0.0000 | 0.0000 | 0.3922 | 0.3495 | 0.0753 | 0.5753 | 0.1138 | 0.7674 |
| 0.0000 | 0.0294 | 2.1515 | 0.2303 | 0.7647 | 0.1765 | 0.0294 | 0.0294 | 0.0000 | 0.0000 | 1.0000 | 0.0000 | 0.0000 | 0.0000 | 0.5098 | 0.0539 | 0.4363 | 0.0582 | 0.9735 |
| 0.0093 | 0.3014 | 3.6792 | 0.5358 | 0.0607 | 0.6262 | 0.3084 | 0.0023 | 0.0767 | 0.4413 | 1.0000 | 0.0000 | 0.1535 | 0.5180 | 0.5222 | 0.0304 | 0.4474 | 0.0994 | 0.6872 |
| 0.0046 | 0.0615 | 1.9680 | 0.1936 | 0.5194 | 0.3599 | 0.1185 | 0.0023 | 0.0000 | 0.5559 | 1.0000 | 0.0000 | 0.0008 | 0.6382 | 0.4875 | 0.0820 | 0.4305 | 0.0953 | 0.8826 |
| 0.0434 | 0.3398 | 3.4992 | 0.4998 | 0.1871 | 0.4716 | 0.3383 | 0.0030 | 0.0000 | 0.4305 | 0.9985 | 0.0000 | 0.0000 | 0.4305 | 0.3773 | 0.1419 | 0.4808 | 0.0902 | 0.7045 |
| 0.0000 | 0.0018 | 2.0144 | 0.2029 | 0.7220 | 0.2762 | 0.0018 | 0.0000 | 0.0000 | 0.5801 | 1.0000 | 0.0000 | 0.0000 | 0.5983 | 0.4853 | 0.0313 | 0.4835 | 0.1232 | 0.9164 |
| 0.0000 | 0.0000 | 2.6416 | 0.3283 | 0.8065 | 0.1935 | 0.0000 | 0.0000 | 0.0000 | 0.3488 | 1.0000 | 0.0000 | 0.0000 | 0.5206 | 0.4833 | 0.0335 | 0.4833 | 0.1075 | 0.7905 |
| 0.0083 | 0.5262 | 4.2092 | 0.6418 | 0.0014 | 0.0649 | 0.9337 | 0.0000 | 0.0000 | 0.0000 | 0.9959 | 0.0028 | 0.0014 | 0.0000 | 0.8451 | 0.0775 | 0.0775 | 0.0731 | 0.6165 |
| 0.0621 | 0.1086 | 3.3368 | 0.4674 | 0.0207 | 0.1371 | 0.8233 | 0.0000 | 0.0000 | 0.0000 | 0.9767 | 0.0034 | 0.0000 | 0.0000 | 0.5914 | 0.0501 | 0.3585 | 0.1071 | 0.7875 |
| 0.0343 | 0.4354 | 4.4346 | 0.6869 | 0.0011 | 0.1097 | 0.8891 | 0.0000 | 0.0000 | 0.0000 | 0.9943 | 0.0046 | 0.0011 | 0.0000 | 0.6747 | 0.1635 | 0.1618 | 0.0492 | 0.7826 |
| 0.1566 | 0.0850 | 3.1787 | 0.4357 | 0.2346 | 0.1810 | 0.5832 | 0.0000 | 0.0000 | 0.0000 | 0.9959 | 0.0023 | 0.0000 | 0.0000 | 0.7897 | 0.0706 | 0.1397 | 0.1209 | 0.8269 |
| 0.0437 | 0.0860 | 2.6822 | 0.3364 | 0.5292 | 0.2995 | 0.1712 | 0.0000 | 0.0000 | 0.0000 | 1.0000 | 0.0000 | 0.0000 | 0.0000 | 0.4434 | 0.2147 | 0.3419 | 0.1069 | 0.6518 |
| 0.0025 | 0.1980 | 3.6282 | 0.5256 | 0.2475 | 0.3552 | 0.3725 | 0.0248 | 0.0000 | 0.0000 | 0.9752 | 0.0248 | 0.0000 | 0.0000 | 0.9468 | 0.0260 | 0.0272 | 0.1261 | 0.6824 |
| 0.0622 | 0.1873 | 3.1694 | 0.4339 | 0.0155 | 0.3590 | 0.6255 | 0.0000 | 0.0000 | 0.0000 | 0.9526 | 0.0474 | 0.0000 | 0.0000 | 0.9163 | 0.0462 | 0.0374 | 0.1221 | 0.8242 |
| 0.1437 | 0.2243 | 2.9246 | 0.3849 | 0.4027 | 0.5148 | 0.0826 | 0.0000 | 0.0000 | 0.0000 | 1.0000 | 0.0000 | 0.0000 | 0.0000 | 0.4130 | 0.2168 | 0.3702 | 0.1004 | 0.4914 |
| 0.0520 | 0.1802 | 2.8142 | 0.3628 | 0.3807 | 0.4365 | 0.1294 | 0.0508 | 0.0000 | 0.0000 | 1.0000 | 0.0000 | 0.0000 | 0.0000 | 0.4678 | 0.1953 | 0.3369 | 0.1096 | 0.5793 |
| 0.0000 | 0.1037 | 2.6557 | 0.3311 | 0.4271 | 0.1869 | 0.3850 | 0.0000 | 0.0000 | 0.0000 | 1.0000 | 0.0000 | 0.0000 | 0.0000 | 0.4806 | 0.0420 | 0.4774 | 0.1474 | 0.8425 |
| 0.0194 | 0.3843 | 3.2741 | 0.4548 | 0.3148 | 0.5537 | 0.1315 | 0.0000 | 0.0000 | 0.0000 | 1.0000 | 0.0000 | 0.0000 | 0.0000 | 0.6711 | 0.0415 | 0.2874 | 0.1082 | 0.7731 |
| 0.0754 | 0.4443 | 3.7758 | 0.5552 | 0.0000 | 0.9963 | 0.0037 | 0.0000 | 0.0000 | 0.0000 | 0.0405 | 0.9402 | 0.0000 | 0.0000 | 0.4160 | 0.5828 | 0.0012 | 0.1037 | 0.6223 |
| 0.3653 | 0.2553 | 3.7430 | 0.5486 | 0.0000 | 0.8290 | 0.1710 | 0.0000 | 0.0000 | 0.0000 | 0.6090 | 0.3653 | 0.0000 | 0.0000 | 0.6494 | 0.2085 | 0.1420 | 0.1496 | 0.8004 |
| 0.2994 | 0.0812 | 3.1536 | 0.4307 | 0.1543 | 0.7421 | 0.0224 | 0.0004 | 0.0000 | 0.0000 | 0.9993 | 0.0004 | 0.0358 | 0.0000 | 0.5853 | 0.1179 | 0.2968 | 0.1215 | 0.6977 |
| 0.2693 | 0.0012 | 3.1233 | 0.4247 | 0.0123 | 0.7646 | 0.1848 | 0.0006 | 0.0000 | 0.0000 | 0.9877 | 0.0123 | 0.0580 | 0.0000 | 0.6484 | 0.0610 | 0.2906 | 0.1016 | 0.6639 |
| 0.0785 | 0.0253 | 2.9519 | 0.3904 | 0.1519 | 0.7190 | 0.0785 | 0.0000 | 0.0000 | 0.0000 | 0.9987 | 0.0013 | 0.1134 | 0.0000 | 0.6242 | 0.0637 | 0.3121 | 0.1009 | 0.7612 |
| 0.2436 | 0.0598 | 3.0829 | 0.4166 | 0.1288 | 0.7481 | 0.1169 | 0.0000 | 0.0000 | 0.0000 | 1.0000 | 0.0000 | 0.0000 | 0.0000 | 0.6617 | 0.1172 | 0.2211 | 0.1313 | 0.6800 |
| 0.0725 | 0.0007 | 3.5087 | 0.5017 | 0.0442 | 0.3488 | 0.6062 | 0.0007 | 0.0000 | 0.0000 | 1.0000 | 0.0000 | 0.0000 | 0.0000 | 0.7230 | 0.0341 | 0.2429 | 0.1340 | 0.6712 |
| 0.0247 | 0.0006 | 3.2575 | 0.4515 | 0.3466 | 0.4002 | 0.2411 | 0.0121 | 0.0000 | 0.0000 | 0.9759 | 0.0000 | 0.0006 | 0.0000 | 0.5673 | 0.1077 | 0.3250 | 0.1081 | 0.6313 |
| 0.0379 | 0.1391 | 3.5579 | 0.5116 | 0.0506 | 0.6707 | 0.2781 | 0.0006 | 0.0000 | 0.0000 | 1.0000 | 0.0000 | 0.0000 | 0.0000 | 0.8453 | 0.0173 | 0.1374 | 0.1145 | 0.6859 |
| 0.3001 | 0.0249 | 3.3250 | 0.4650 | 0.1743 | 0.5255 | 0.2752 | 0.0000 | 0.0000 | 0.0000 | 1.0000 | 0.0000 | 0.0695 | 0.0000 | 0.4814 | 0.1718 | 0.3468 | 0.1163 | 0.8444 |
| 0.0405 | 0.1123 | 3.0424 | 0.4085 | 0.0368 | 0.5212 | 0.4420 | 0.0000 | 0.0000 | 0.0000 | 1.0000 | 0.0000 | 0.0995 | 0.0000 | 0.6023 | 0.0798 | 0.3179 | 0.1212 | 0.5333 |
| 0.0784 | 0.0392 | 3.3461 | 0.4692 | 0.0402 | 0.5118 | 0.3696 | 0.0000 | 0.0000 | 0.0000 | 1.0000 | 0.0000 | 0.0192 | 0.0000 | 0.5301 | 0.0987 | 0.3712 | 0.1075 | 0.8043 |
| 0.1135 | 0.0284 | 2.5753 | 0.3151 | 0.0582 | 0.6539 | 0.0312 | 0.0014 | 0.0000 | 0.0000 | 1.0000 | 0.0000 | 0.0550 | 0.0000 | 0.4714 | 0.2005 | 0.3281 | 0.1255 | 0.6563 |
| 0.0763 | 0.0000 | 2.6947 | 0.3389 | 0.5344 | 0.4580 | 0.0038 | 0.0000 | 0.0000 | 0.0000 | 1.0000 | 0.0000 | 0.0113 | 0.0000 | 0.4866 | 0.0267 | 0.4866 | 0.0998 | 0.7720 |
| 0.0000 | 0.0000 | 3.6522 | 0.5304 | 0.3043 | 0.6957 | 0.0000 | 0.0000 | 0.0000 | 0.0000 | 1.0000 | 0.0000 | 0.3429 | 0.0000 | 0.4710 | 0.0580 | 0.4710 | 0.1110 | 0.8362 |
| 0.0355 | 0.0346 | 2.9844 | 0.3969 | 0.4113 | 0.2294 | 0.3593 | 0.0000 | 0.0000 | 0.0000 | 0.9991 | 0.0009 | 0.0170 | 0.0000 | 0.5146 | 0.0587 | 0.4267 | 0.0817 | 0.7817 |
| 0.0000 | 0.0373 | 2.1936 | 0.2387 | 0.5600 | 0.2200 | 0.2191 | 0.0000 | 0.0000 | 0.0000 | 1.0000 | 0.0000 | 0.0187 | 0.0000 | 0.5005 | 0.0373 | 0.4623 | 0.1379 | 0.7822 |

| | | | | | | | | | | | | | | | | | | |
|---|---|---|---|---|---|---|---|---|---|---|---|---|---|---|---|---|---|---|
| 0.0268 | 0.4016 | 3.1593 | 0.4319 | 0.2155 | 0.5944 | 0.1874 | 0.0000 | 0.0000 | 0.0000 | 1.0000 | 0.0000 | 0.0000 | 0.0000 | 0.5481 | 0.1330 | 0.3190 | 0.1163 | 0.5443 |
| 0.0000 | 0.1266 | 2.9277 | 0.3855 | 0.1989 | 0.1998 | 0.5832 | 0.0000 | 0.0000 | 0.0000 | 1.0000 | 0.0000 | 0.0000 | 0.0000 | 0.5092 | 0.0378 | 0.4529 | 0.1158 | 0.5270 |
| 0.0000 | 0.0661 | 2.6694 | 0.3339 | 0.3314 | 0.1182 | 0.5331 | 0.0000 | 0.0000 | 0.0000 | 1.0000 | 0.0000 | 0.0000 | 0.0000 | 0.5172 | 0.0177 | 0.4651 | 0.1075 | 0.3830 |
| 0.0000 | 0.0984 | 2.5685 | 0.3137 | 0.4322 | 0.1279 | 0.4392 | 0.0000 | 0.0000 | 0.0000 | 1.0000 | 0.0000 | 0.0000 | 0.0000 | 0.4955 | 0.0386 | 0.4659 | 0.1009 | 0.7506 |
| 0.0000 | 0.0162 | 2.8727 | 0.3745 | 0.4015 | 0.2620 | 0.3366 | 0.0000 | 0.0000 | 0.0000 | 0.9838 | 0.0162 | 0.0322 | 0.0000 | 0.5650 | 0.0411 | 0.3939 | 0.0870 | 0.7294 |
| 0.0437 | 0.0824 | 3.1943 | 0.4389 | 0.1841 | 0.4140 | 0.4018 | 0.0000 | 0.0000 | 0.0000 | 0.9990 | 0.0000 | 0.0000 | 0.0000 | 0.5967 | 0.0572 | 0.3462 | 0.1003 | 0.7434 |
| 0.0154 | 0.2284 | 3.4437 | 0.4887 | 0.2284 | 0.3523 | 0.4186 | 0.0000 | 0.0000 | 0.0000 | 0.9993 | 0.0000 | 0.0007 | 0.0000 | 0.5831 | 0.0767 | 0.3403 | 0.0981 | 0.7135 |
| 0.0194 | 0.1293 | 3.3375 | 0.4675 | 0.2410 | 0.2641 | 0.4386 | 0.0185 | 0.0000 | 0.0000 | 1.0000 | 0.0000 | 0.0853 | 0.0000 | 0.5103 | 0.1207 | 0.3690 | 0.1103 | 0.6138 |
| 0.0188 | 0.1313 | 3.3136 | 0.4627 | 0.2251 | 0.2148 | 0.5582 | 0.0000 | 0.0000 | 0.0000 | 0.9991 | 0.0009 | 0.0009 | 0.0000 | 0.4703 | 0.1200 | 0.4097 | 0.1109 | 0.5931 |
| 0.0362 | 0.0329 | 2.9408 | 0.3882 | 0.1974 | 0.3076 | 0.4293 | 0.0000 | 0.0000 | 0.0000 | 1.0000 | 0.0000 | 0.0000 | 0.0000 | 0.5285 | 0.0450 | 0.4265 | 0.1427 | 0.7163 |
| 0.0175 | 0.1412 | 3.0018 | 0.4004 | 0.2281 | 0.3719 | 0.3825 | 0.0000 | 0.0000 | 0.0000 | 1.0000 | 0.0000 | 0.0000 | 0.0000 | 0.5475 | 0.0295 | 0.4230 | 0.1260 | 0.6965 |
| 0.0051 | 0.6410 | 4.6598 | 0.7320 | 0.0000 | 0.0513 | 0.9487 | 0.0000 | 0.0000 | 0.0000 | 0.9744 | 0.0256 | 0.0000 | 0.0000 | 0.8986 | 0.0584 | 0.0430 | 0.0886 | 0.8797 |
| 0.0195 | 0.0488 | 2.8578 | 0.3716 | 0.6049 | 0.1463 | 0.2488 | 0.0000 | 0.0000 | 0.0000 | 0.9951 | 0.0000 | 0.0000 | 0.0000 | 0.5090 | 0.0703 | 0.4208 | 0.0695 | 0.9528 |
| 0.0237 | 0.1611 | 3.5667 | 0.5133 | 0.2986 | 0.1848 | 0.5166 | 0.0000 | 0.0000 | 0.0000 | 0.9905 | 0.0047 | 0.0047 | 0.0000 | 0.3913 | 0.1484 | 0.4603 | 0.1189 | 0.7386 |
| 0.0600 | 0.2640 | 3.3248 | 0.4650 | 0.2000 | 0.4280 | 0.3680 | 0.0040 | 0.0000 | 0.0000 | 0.9400 | 0.0000 | 0.0000 | 0.0000 | 0.5858 | 0.1433 | 0.2709 | 0.1080 | 0.8582 |
| 0.0398 | 0.0100 | 2.4523 | 0.2905 | 0.7065 | 0.2388 | 0.0498 | 0.0050 | 0.0000 | 0.0000 | 0.9950 | 0.0000 | 0.0000 | 0.0000 | 0.4808 | 0.0483 | 0.4708 | 0.0795 | 0.9334 |
| 0.0039 | 0.3589 | 4.2700 | 0.6540 | 0.0000 | 0.4875 | 0.5125 | 0.0000 | 0.0000 | 0.0000 | 0.9765 | 0.0227 | 0.0000 | 0.0000 | 0.5698 | 0.2204 | 0.2098 | 0.0956 | 0.9196 |
| 0.0009 | 0.0035 | 4.3952 | 0.6790 | 0.0220 | 0.1829 | 0.7951 | 0.0000 | 0.0000 | 0.0000 | 1.0000 | 0.0000 | 0.0000 | 0.0000 | 0.3408 | 0.3237 | 0.3355 | 0.0489 | 0.9265 |
| 0.1230 | 0.2460 | 3.6710 | 0.5342 | 0.0217 | 0.3730 | 0.6053 | 0.0000 | 0.0000 | 0.0000 | 1.0000 | 0.0000 | 0.0000 | 0.0000 | 0.6259 | 0.1766 | 0.1975 | 0.1004 | 0.9300 |
| 0.0101 | 0.6231 | 4.4467 | 0.6893 | 0.0000 | 0.0503 | 0.9447 | 0.0050 | 0.0000 | 0.0000 | 0.9849 | 0.0151 | 0.0000 | 0.0000 | 0.9520 | 0.0278 | 0.0202 | 0.0929 | 0.8362 |
| 0.0038 | 0.2192 | 3.5405 | 0.5081 | 0.3923 | 0.4192 | 0.1885 | 0.0000 | 0.0000 | 0.0000 | 0.9962 | 0.0000 | 0.0038 | 0.0000 | 0.5708 | 0.1075 | 0.3218 | 0.1170 | 0.9293 |
| 0.0807 | 0.2063 | 3.7342 | 0.5468 | 0.1076 | 0.3587 | 0.5291 | 0.0045 | 0.0000 | 0.0000 | 0.9327 | 0.0673 | 0.0000 | 0.0000 | 0.5859 | 0.1510 | 0.2631 | 0.1101 | 0.8515 |
| 0.0095 | 0.0995 | 3.1000 | 0.4200 | 0.5403 | 0.3175 | 0.1422 | 0.0000 | 0.0000 | 0.0000 | 0.9953 | 0.0000 | 0.0000 | 0.0000 | 0.5317 | 0.0937 | 0.3746 | 0.1054 | 0.9556 |
| 0.0045 | 0.0852 | 3.2207 | 0.4441 | 0.4753 | 0.3094 | 0.2152 | 0.0000 | 0.0000 | 0.0000 | 0.9955 | 0.0000 | 0.0000 | 0.0000 | 0.5098 | 0.1156 | 0.3746 | 0.1134 | 0.9262 |
| 0.0000 | 0.2553 | 2.7872 | 0.3574 | 0.6000 | 0.3191 | 0.0809 | 0.0000 | 0.0000 | 0.0000 | 0.9957 | 0.0043 | 0.0000 | 0.0000 | 0.6383 | 0.0234 | 0.3383 | 0.0560 | 0.9442 |
| 0.0932 | 0.0062 | 3.7771 | 0.5554 | 0.0683 | 0.1677 | 0.7578 | 0.0000 | 0.0000 | 0.2784 | 1.0000 | 0.0000 | 0.0902 | 0.2784 | 0.4710 | 0.0642 | 0.4648 | 0.0591 | 0.3357 |
| 0.0235 | 0.0000 | 3.2381 | 0.4476 | 0.1176 | 0.3176 | 0.5647 | 0.0000 | 0.0000 | 0.0000 | 0.9882 | 0.0000 | 0.5503 | 0.0000 | 0.4940 | 0.0357 | 0.4702 | 0.1067 | 0.4466 |
| 0.0103 | 0.0000 | 1.1705 | 0.0341 | 0.8247 | 0.0619 | 0.0206 | 0.0000 | 0.0000 | 0.0000 | 0.9072 | 0.0000 | 0.0300 | 0.0000 | 0.5000 | 0.0000 | 0.5000 | 0.0440 | 0.7653 |
| 0.0085 | 0.0000 | 2.3143 | 0.2629 | 0.6186 | 0.2373 | 0.1102 | 0.0000 | 0.0000 | 0.0000 | 0.9576 | 0.0000 | 0.2848 | 0.0000 | 0.3899 | 0.1835 | 0.4266 | 0.1120 | 0.6664 |
| 0.0455 | 0.3430 | 3.9485 | 0.5897 | 0.3223 | 0.2975 | 0.3760 | 0.0041 | 0.0000 | 0.0000 | 1.0000 | 0.0000 | 0.0000 | 0.0000 | 0.4552 | 0.1556 | 0.3891 | 0.1137 | 0.5900 |
| 0.1707 | 0.3943 | 4.6116 | 0.7223 | 0.0935 | 0.3984 | 0.5081 | 0.0000 | 0.0000 | 0.0000 | 1.0000 | 0.0000 | 0.0000 | 0.0000 | 0.7893 | 0.0744 | 0.1364 | 0.0850 | 0.6997 |
| 0.0379 | 0.3886 | 4.1286 | 0.6257 | 0.0047 | 0.3697 | 0.6256 | 0.0000 | 0.0000 | 0.0000 | 1.0000 | 0.0000 | 0.0000 | 0.0000 | 0.8455 | 0.0785 | 0.0761 | 0.1047 | 0.8660 |
| 0.0382 | 0.3550 | 3.0687 | 0.4137 | 0.2405 | 0.5496 | 0.2099 | 0.0000 | 0.0000 | 0.0000 | 1.0000 | 0.0000 | 0.0000 | 0.0000 | 0.4924 | 0.0649 | 0.4427 | 0.1051 | 0.5053 |
| 0.0076 | 0.0000 | 2.2121 | 0.2424 | 0.7879 | 0.0227 | 0.1894 | 0.0000 | 0.0000 | 0.0000 | 1.0000 | 0.0000 | 0.0000 | 0.0000 | 0.4861 | 0.0051 | 0.5088 | 0.0638 | 0.6987 |
| 0.0000 | 0.0000 | 2.3228 | 0.2646 | 0.6378 | 0.0472 | 0.3150 | 0.0000 | 0.0000 | 0.0000 | 1.0000 | 0.0000 | 0.0000 | 0.0000 | 0.5000 | 0.0040 | 0.4960 | 0.0875 | 0.9804 |
| 0.0040 | 0.2955 | 3.6612 | 0.5322 | 0.2632 | 0.1862 | 0.5506 | 0.0000 | 0.0000 | 0.0000 | 1.0000 | 0.0000 | 0.0000 | 0.0000 | 0.8123 | 0.0379 | 0.1497 | 0.0931 | 0.7228 |
| 0.1019 | 0.3434 | 4.2878 | 0.6576 | 0.1849 | 0.5547 | 0.2604 | 0.0000 | 0.0000 | 0.0000 | 1.0000 | 0.0000 | 0.0000 | 0.0000 | 0.7195 | 0.1195 | 0.1610 | 0.1010 | 0.7588 |
| 0.0688 | 0.6073 | 4.3333 | 0.6667 | 0.0000 | 0.2470 | 0.7530 | 0.0000 | 0.0000 | 0.0000 | 1.0000 | 0.0000 | 0.0000 | 0.0000 | 0.8949 | 0.0537 | 0.0514 | 0.0636 | 0.9067 |
| 0.0094 | 0.3428 | 4.1619 | 0.6324 | 0.1918 | 0.2704 | 0.5377 | 0.0000 | 0.0000 | 0.0000 | 0.9969 | 0.0031 | 0.0000 | 0.0000 | 0.8141 | 0.0794 | 0.1065 | 0.0933 | 0.8030 |
| 0.0000 | 0.3297 | 4.0971 | 0.6194 | 0.2581 | 0.1147 | 0.6272 | 0.0000 | 0.0000 | 0.0000 | 1.0000 | 0.0000 | 0.0000 | 0.0000 | 0.5791 | 0.1259 | 0.2950 | 0.0826 | 0.9019 |
| 0.0000 | 0.0064 | 2.1720 | 0.2344 | 0.8535 | 0.0892 | 0.0573 | 0.0000 | 0.0000 | 0.0000 | 1.0000 | 0.0000 | 0.0000 | 0.0000 | 0.4915 | 0.0170 | 0.4915 | 0.0800 | 0.9537 |
| 0.0962 | 0.3138 | 4.4894 | 0.6979 | 0.0042 | 0.3431 | 0.6527 | 0.0000 | 0.0000 | 0.0000 | 0.9498 | 0.0502 | 0.0000 | 0.0000 | 0.6089 | 0.2107 | 0.1804 | 0.0900 | 0.8540 |
| 0.0000 | 0.3091 | 3.6484 | 0.5297 | 0.0909 | 0.4436 | 0.4582 | 0.0073 | 0.0036 | 0.0000 | 1.0000 | 0.0000 | 0.0000 | 0.0036 | 0.6224 | 0.0824 | 0.2952 | 0.1055 | 0.6584 |
| 0.0040 | 0.1687 | 3.5031 | 0.5006 | 0.1968 | 0.2530 | 0.2048 | 0.3454 | 0.0000 | 0.0000 | 1.0000 | 0.0000 | 0.0000 | 0.0000 | 0.5649 | 0.0750 | 0.3601 | 0.1162 | 0.5512 |
| 0.0000 | 0.0765 | 2.1684 | 0.2337 | 0.0000 | 0.0765 | 0.9235 | 0.0000 | 0.0000 | 0.0000 | 1.0000 | 0.0000 | 0.0000 | 0.0000 | 0.9966 | 0.0017 | 0.0017 | 0.0201 | 0.4943 |
| 0.0811 | 0.0000 | 2.2295 | 0.2459 | 0.6757 | 0.1419 | 0.0068 | 0.1757 | 0.0000 | 0.0000 | 1.0000 | 0.0000 | 0.0000 | 0.0000 | 0.5113 | 0.0045 | 0.4842 | 0.0507 | 0.6744 |
| 0.0035 | 0.6135 | 4.8525 | 0.7705 | 0.0000 | 0.3262 | 0.6738 | 0.0000 | 0.0000 | 0.0000 | 0.9894 | 0.0106 | 0.0000 | 0.0000 | 0.6427 | 0.1841 | 0.1733 | 0.1043 | 0.7798 |
| 0.0095 | 0.5570 | 4.8274 | 0.7655 | 0.0000 | 0.2500 | 0.7500 | 0.0000 | 0.0000 | 0.0000 | 0.9842 | 0.0127 | 0.0000 | 0.0000 | 0.7041 | 0.1520 | 0.1439 | 0.1025 | 0.7371 |
| 0.0458 | 0.0687 | 2.8790 | 0.3758 | 0.0000 | 0.6031 | 0.3511 | 0.0458 | 0.0040 | 0.0000 | 0.9771 | 0.0153 | 0.4783 | 0.0040 | 0.8128 | 0.0244 | 0.1628 | 0.1437 | 0.8224 |
| 0.0101 | 0.0808 | 3.1081 | 0.4216 | 0.0505 | 0.2020 | 0.4848 | 0.2525 | 0.5926 | 0.0000 | 1.0000 | 0.0000 | 0.0407 | 0.5926 | 0.4694 | 0.0306 | 0.5000 | 0.1264 | 0.7056 |
| 0.0049 | 0.0000 | 1.0737 | 0.0147 | 0.8780 | 0.0488 | 0.0000 | 0.0732 | 0.0000 | 0.0000 | 1.0000 | 0.0000 | 0.0000 | 0.0000 | 0.5000 | 0.0000 | 0.5000 | 0.0622 | 0.8453 |

| | | | | | | | | | | | | | | | | | | |
|---|---|---|---|---|---|---|---|---|---|---|---|---|---|---|---|---|---|---|
| 0.0139 | 0.2083 | 4.7830 | 0.7566 | 0.0000 | 0.0509 | 0.9491 | 0.0000 | 0.0000 | 0.0000 | 0.9907 | 0.0093 | 0.0000 | 0.0000 | 0.5495 | 0.2288 | 0.2217 | 0.0633 | 0.8162 |
| 0.0986 | 0.1268 | 4.4113 | 0.6823 | 0.0000 | 0.1549 | 0.8451 | 0.0000 | 0.0000 | 0.0000 | 0.9507 | 0.0493 | 0.0000 | 0.0000 | 0.8821 | 0.0750 | 0.0429 | 0.1072 | 0.7949 |
| 0.0317 | 0.3704 | 4.6862 | 0.7372 | 0.0000 | 0.3704 | 0.6296 | 0.0000 | 0.0000 | 0.0000 | 0.9841 | 0.0159 | 0.0000 | 0.0000 | 0.7228 | 0.1453 | 0.1319 | 0.0898 | 0.6584 |
| 0.0262 | 0.3231 | 4.2133 | 0.6427 | 0.0000 | 0.1004 | 0.8996 | 0.0000 | 0.0000 | 0.0000 | 0.9956 | 0.0044 | 0.0000 | 0.0000 | 0.9676 | 0.0173 | 0.0151 | 0.0930 | 0.3923 |
| 0.0000 | 0.0958 | 2.6108 | 0.3222 | 0.6287 | 0.1497 | 0.2216 | 0.0000 | 0.0000 | 0.0000 | 1.0000 | 0.0000 | 0.0000 | 0.0000 | 0.6287 | 0.0180 | 0.3533 | 0.0714 | 0.8722 |
| 0.0266 | 0.6649 | 4.2097 | 0.6419 | 0.0000 | 0.0585 | 0.9415 | 0.0000 | 0.0000 | 0.0000 | 0.9734 | 0.0213 | 0.0000 | 0.0000 | 0.8575 | 0.0780 | 0.0645 | 0.0663 | 0.7113 |
| 0.0239 | 0.2510 | 4.6586 | 0.7317 | 0.0040 | 0.1155 | 0.8805 | 0.0000 | 0.0000 | 0.0000 | 0.9801 | 0.0159 | 0.0000 | 0.0000 | 0.6533 | 0.1774 | 0.1693 | 0.0946 | 0.7032 |
| 0.0779 | 0.0390 | 3.0667 | 0.4133 | 0.0000 | 0.5844 | 0.3896 | 0.0130 | 0.0000 | 0.0000 | 0.5714 | 0.4026 | 0.6908 | 0.0000 | 0.5325 | 0.2208 | 0.2468 | 0.1253 | 0.8467 |
| 0.0165 | 0.7231 | 4.0166 | 0.6033 | 0.0000 | 0.7231 | 0.2769 | 0.0000 | 0.0000 | 0.0000 | 0.9959 | 0.0041 | 0.0000 | 0.0000 | 0.9511 | 0.0255 | 0.0234 | 0.0849 | 0.8816 |
| 0.1327 | 0.1637 | 3.5134 | 0.5027 | 0.2876 | 0.2611 | 0.4513 | 0.0000 | 0.0000 | 0.0000 | 0.9956 | 0.0000 | 0.0000 | 0.0000 | 0.6443 | 0.1042 | 0.2515 | 0.0987 | 0.8512 |
| 0.0148 | 0.0000 | 2.7895 | 0.3579 | 0.1926 | 0.0593 | 0.4519 | 0.2963 | 0.0000 | 0.0000 | 1.0000 | 0.0000 | 0.2819 | 0.0000 | 0.5037 | 0.0075 | 0.4888 | 0.1088 | 0.6953 |
| 0.0419 | 0.0000 | 2.6154 | 0.3231 | 0.2932 | 0.1623 | 0.2304 | 0.3141 | 0.0000 | 0.0000 | 1.0000 | 0.0000 | 0.0255 | 0.0000 | 0.4954 | 0.0282 | 0.4763 | 0.1062 | 0.6190 |
| 0.0000 | 0.0303 | 2.1818 | 0.2364 | 0.7955 | 0.1742 | 0.0303 | 0.0000 | 0.0000 | 0.0000 | 1.0000 | 0.0000 | 0.0000 | 0.0000 | 0.5152 | 0.0341 | 0.4508 | 0.0747 | 0.4477 |
| 0.0000 | 0.0145 | 2.0362 | 0.2072 | 0.0000 | 0.1304 | 0.8696 | 0.0000 | 0.0000 | 0.0000 | 0.8841 | 0.1159 | 0.0000 | 0.0000 | 0.9420 | 0.0580 | 0.0000 | 0.0514 | 0.2979 |
| 0.0000 | 0.0000 | 2.0092 | 0.2018 | 0.9266 | 0.0734 | 0.0000 | 0.0000 | 0.0000 | 0.0000 | 1.0000 | 0.0000 | 0.0000 | 0.0000 | 0.4985 | 0.0216 | 0.4799 | 0.0405 | 0.9640 |
| 0.0240 | 0.3892 | 3.0659 | 0.4132 | 0.1138 | 0.8623 | 0.0240 | 0.0000 | 0.0000 | 0.0000 | 0.6347 | 0.3593 | 0.0000 | 0.0000 | 0.7018 | 0.2346 | 0.0636 | 0.1113 | 0.8969 |
| 0.0000 | 0.0000 | 2.0045 | 0.2009 | 0.5430 | 0.4570 | 0.0000 | 0.0000 | 0.0000 | 0.0000 | 1.0000 | 0.0000 | 0.0000 | 0.0000 | 0.5000 | 0.2262 | 0.2738 | 0.1781 | 0.8086 |
| 0.0084 | 0.2427 | 4.5588 | 0.7118 | 0.0042 | 0.5188 | 0.4770 | 0.0000 | 0.0000 | 0.0000 | 1.0000 | 0.0000 | 0.0000 | 0.0000 | 0.6261 | 0.1870 | 0.1870 | 0.0904 | 0.8298 |
| 0.0067 | 0.0000 | 2.9441 | 0.3888 | 0.4027 | 0.5302 | 0.0268 | 0.0403 | 0.0000 | 0.0000 | 1.0000 | 0.0000 | 0.0000 | 0.0000 | 0.4978 | 0.0246 | 0.4776 | 0.1093 | 0.7654 |
| 0.2320 | 0.0221 | 3.9000 | 0.5800 | 0.0166 | 0.2983 | 0.6851 | 0.0000 | 0.0000 | 0.1263 | 1.0000 | 0.0000 | 0.2386 | 0.1263 | 0.4705 | 0.0866 | 0.4429 | 0.0728 | 0.4889 |
| 0.0000 | 0.0000 | 2.2564 | 0.2513 | 0.0000 | 0.8397 | 0.0000 | 0.0000 | 0.0000 | 0.0000 | 1.0000 | 0.0000 | 0.0000 | 0.0000 | 0.5000 | 0.1648 | 0.3352 | 0.1051 | 0.4872 |
| 0.0000 | 0.0000 | 2.1852 | 0.2370 | 0.0000 | 0.5062 | 0.0000 | 0.0000 | 0.0000 | 0.0000 | 1.0000 | 0.0000 | 0.0000 | 0.0000 | 0.5000 | 0.5000 | 0.0000 | 0.0739 | 0.8712 |
| 0.0000 | 0.2527 | 4.5054 | 0.7011 | 0.0000 | 0.4677 | 0.5323 | 0.0000 | 0.0000 | 0.0000 | 1.0000 | 0.0000 | 0.0000 | 0.0000 | 0.4848 | 0.2536 | 0.2616 | 0.1043 | 0.8321 |
| 0.0047 | 0.1368 | 4.4327 | 0.6865 | 0.0000 | 0.3255 | 0.6745 | 0.0000 | 0.0000 | 0.0000 | 1.0000 | 0.0000 | 0.0000 | 0.0000 | 0.5168 | 0.2428 | 0.2404 | 0.0822 | 0.9140 |
| 0.0260 | 0.0313 | 3.9034 | 0.5807 | 0.0365 | 0.3542 | 0.5260 | 0.0833 | 0.0000 | 0.0000 | 0.8698 | 0.1302 | 0.1899 | 0.0000 | 0.4433 | 0.1946 | 0.3621 | 0.0922 | 0.5592 |
| 0.0194 | 0.0437 | 3.1984 | 0.4397 | 0.0388 | 0.2184 | 0.3544 | 0.3883 | 0.2128 | 0.0000 | 0.9951 | 0.0049 | 0.0567 | 0.2128 | 0.4902 | 0.0319 | 0.4779 | 0.1155 | 0.7883 |
| 0.0000 | 0.0000 | 1.5380 | 0.1076 | 0.4678 | 0.5322 | 0.0000 | 0.0000 | 0.0000 | 0.0000 | 1.0000 | 0.0000 | 0.0000 | 0.0000 | 0.5000 | 0.0500 | 0.4500 | 0.2904 | 0.9491 |
| 0.0000 | 0.0650 | 4.9146 | 0.7829 | 0.0000 | 0.5569 | 0.4431 | 0.0000 | 0.0000 | 0.0000 | 0.9959 | 0.0041 | 0.0000 | 0.0000 | 0.3340 | 0.3340 | 0.3320 | 0.0587 | 0.8377 |
| 0.0000 | 0.0267 | 3.1200 | 0.4240 | 0.0067 | 0.2667 | 0.7267 | 0.0000 | 0.0000 | 0.0000 | 0.7733 | 0.2267 | 0.0000 | 0.0000 | 0.3725 | 0.3624 | 0.2651 | 0.0571 | 0.9044 |
| 0.0000 | 0.0000 | 2.1407 | 0.2281 | 0.1259 | 0.7556 | 0.1185 | 0.0000 | 0.0000 | 0.0000 | 0.2519 | 0.7481 | 0.0000 | 0.0000 | 0.4938 | 0.3818 | 0.1244 | 0.0839 | 0.6286 |
| 0.0305 | 0.0061 | 3.4417 | 0.4883 | 0.1768 | 0.0732 | 0.7439 | 0.0000 | 0.0000 | 0.3684 | 1.0000 | 0.0000 | 0.0561 | 0.3684 | 0.4909 | 0.0244 | 0.4848 | 0.0669 | 0.3637 |
| 0.2917 | 0.1944 | 4.4583 | 0.6917 | 0.0000 | 0.4954 | 0.5046 | 0.0000 | 0.0000 | 0.0000 | 0.9769 | 0.0231 | 0.0000 | 0.0000 | 0.8457 | 0.0829 | 0.0713 | 0.1016 | 0.8420 |
| 0.0000 | 0.0069 | 4.9514 | 0.7903 | 0.0000 | 0.0278 | 0.9722 | 0.0000 | 0.0000 | 0.0000 | 0.9931 | 0.0069 | 0.0000 | 0.0000 | 0.9965 | 0.0035 | 0.0000 | 0.0074 | 0.9922 |
| 0.1840 | 0.0708 | 4.0236 | 0.6047 | 0.1887 | 0.3349 | 0.4764 | 0.0000 | 0.0000 | 0.0000 | 0.9670 | 0.0330 | 0.0000 | 0.0000 | 0.6950 | 0.1596 | 0.1454 | 0.1261 | 0.6566 |
| 0.0368 | 0.0123 | 3.1358 | 0.4272 | 0.0000 | 0.5767 | 0.4110 | 0.0061 | 0.0000 | 0.0000 | 0.4908 | 0.3497 | 0.4825 | 0.0000 | 0.5453 | 0.1903 | 0.2644 | 0.1163 | 0.8541 |
| 0.0385 | 0.0385 | 3.6600 | 0.5320 | 0.1442 | 0.0096 | 0.7692 | 0.0385 | 0.0214 | 0.0000 | 0.9904 | 0.0096 | 0.4225 | 0.0214 | 0.4984 | 0.0032 | 0.4984 | 0.0348 | 0.9455 |
| 0.0000 | 0.0342 | 3.5089 | 0.5018 | 0.1026 | 0.0085 | 0.8462 | 0.0427 | 0.3788 | 0.0000 | 1.0000 | 0.0000 | 0.0303 | 0.3788 | 0.4943 | 0.0114 | 0.4943 | 0.0545 | 0.5790 |
| 0.0000 | 0.0103 | 3.6701 | 0.5340 | 0.1546 | 0.0000 | 0.8454 | 0.0000 | 0.5000 | 0.0000 | 1.0000 | 0.0000 | 0.0992 | 0.5000 | 0.4983 | 0.0034 | 0.4983 | 0.0344 | 0.3966 |
| 0.0571 | 0.0571 | 3.0882 | 0.4176 | 0.2857 | 0.0571 | 0.6000 | 0.0286 | 0.1172 | 0.0000 | 0.9429 | 0.0571 | 0.6414 | 0.1172 | 0.4714 | 0.0429 | 0.4857 | 0.0980 | 0.7537 |
| 0.0000 | 0.0000 | 2.6364 | 0.3273 | 0.0523 | 0.2500 | 0.4651 | 0.0000 | 0.0000 | 0.0000 | 0.7674 | 0.0000 | 0.1443 | 0.0000 | 0.4462 | 0.1115 | 0.4423 | 0.1681 | 0.8102 |
| 0.0069 | 0.0000 | 2.6471 | 0.3294 | 0.0966 | 0.1586 | 0.3310 | 0.4138 | 0.0000 | 0.0000 | 1.0000 | 0.0000 | 0.1317 | 0.0000 | 0.4975 | 0.0086 | 0.4939 | 0.1201 | 0.6284 |
| 0.0443 | 0.1458 | 2.5745 | 0.3149 | 0.2808 | 0.4924 | 0.1836 | 0.0432 | 0.4180 | 0.0000 | 0.8920 | 0.1080 | 0.0000 | 0.4180 | 0.4421 | 0.1147 | 0.4432 | 0.1060 | 0.7487 |
| 0.0900 | 0.5619 | 4.1828 | 0.6366 | 0.1213 | 0.3391 | 0.5140 | 0.0255 | 0.1903 | 0.0917 | 1.0000 | 0.0000 | 0.0000 | 0.2820 | 0.6776 | 0.0694 | 0.2530 | 0.1295 | 0.8024 |
| 0.0857 | 0.0000 | 2.4204 | 0.2841 | 0.7551 | 0.1306 | 0.1143 | 0.0000 | 0.0000 | 0.0000 | 1.0000 | 0.0000 | 0.0000 | 0.0000 | 0.4891 | 0.0585 | 0.4524 | 0.0791 | 0.6710 |
| 0.1261 | 0.1186 | 3.3311 | 0.4662 | 0.2282 | 0.3267 | 0.4390 | 0.0061 | 0.0000 | 0.0000 | 0.9817 | 0.0122 | 0.0012 | 0.0000 | 0.3901 | 0.2039 | 0.4060 | 0.1099 | 0.5447 |
| 0.1329 | 0.4232 | 4.4877 | 0.6975 | 0.0049 | 0.3091 | 0.6860 | 0.0000 | 0.0000 | 0.0078 | 0.8863 | 0.1127 | 0.0000 | 0.0078 | 0.5264 | 0.2648 | 0.2088 | 0.0925 | 0.8080 |
| 0.1003 | 0.2312 | 4.3509 | 0.6702 | 0.0117 | 0.4386 | 0.5482 | 0.0000 | 0.0000 | 0.0000 | 0.9516 | 0.0402 | 0.0000 | 0.0000 | 0.5860 | 0.2160 | 0.1980 | 0.0993 | 0.8861 |
| 0.2451 | 0.3015 | 3.3303 | 0.4661 | 0.2669 | 0.4403 | 0.2857 | 0.0000 | 0.0000 | 0.1474 | 0.9972 | 0.0000 | 0.0000 | 0.1474 | 0.4887 | 0.1647 | 0.3467 | 0.1214 | 0.8469 |
| 0.0172 | 0.4230 | 4.5447 | 0.7089 | 0.1349 | 0.5160 | 0.3479 | 0.0012 | 0.0000 | 0.0000 | 0.9791 | 0.0209 | 0.0000 | 0.0000 | 0.5176 | 0.2425 | 0.2400 | 0.1060 | 0.7224 |
| 0.1783 | 0.5073 | 4.4663 | 0.6933 | 0.0000 | 0.2293 | 0.7707 | 0.0000 | 0.0000 | 0.0000 | 0.7930 | 0.2056 | 0.0000 | 0.0000 | 0.6521 | 0.2482 | 0.0997 | 0.0875 | 0.8525 |
| 0.0873 | 0.3305 | 4.3907 | 0.6781 | 0.0216 | 0.3768 | 0.6015 | 0.0000 | 0.0000 | 0.0015 | 0.9753 | 0.0247 | 0.0077 | 0.0015 | 0.6162 | 0.1831 | 0.2006 | 0.0975 | 0.7672 |

| | | | | | | | | | | | | | | | | | | |
|---|---|---|---|---|---|---|---|---|---|---|---|---|---|---|---|---|---|---|
| 0.0651 | 0.3937 | 3.9802 | 0.5960 | 0.1620 | 0.2796 | 0.5581 | 0.0000 | 0.0000 | 0.0212 | 0.9718 | 0.0251 | 0.0000 | 0.0212 | 0.5951 | 0.1797 | 0.2252 | 0.1142 | 0.7469 |
| 0.0569 | 0.2258 | 4.5085 | 0.7017 | 0.0184 | 0.3275 | 0.6540 | 0.0000 | 0.0000 | 0.0000 | 0.9302 | 0.0682 | 0.0000 | 0.0000 | 0.5036 | 0.2676 | 0.2288 | 0.1005 | 0.8027 |
| 0.1162 | 0.1680 | 3.6782 | 0.5356 | 0.0311 | 0.5674 | 0.4015 | 0.0000 | 0.0000 | 0.0000 | 0.5571 | 0.4326 | 0.0000 | 0.0000 | 0.3735 | 0.3609 | 0.2655 | 0.1262 | 0.6065 |
| 0.3545 | 0.2015 | 4.1048 | 0.6210 | 0.0000 | 0.5147 | 0.4853 | 0.0000 | 0.0000 | 0.0000 | 0.5085 | 0.4857 | 0.0000 | 0.0000 | 0.3514 | 0.4743 | 0.1744 | 0.0729 | 0.6492 |
| 0.0627 | 0.2757 | 3.2103 | 0.4421 | 0.3679 | 0.2728 | 0.3584 | 0.0010 | 0.0000 | 0.2755 | 1.0000 | 0.0000 | 0.0000 | 0.2755 | 0.3043 | 0.1812 | 0.5144 | 0.1153 | 0.8441 |
| 0.0781 | 0.3775 | 4.2453 | 0.6491 | 0.0702 | 0.2757 | 0.6541 | 0.0000 | 0.0000 | 0.0173 | 0.9464 | 0.0536 | 0.0000 | 0.0173 | 0.5853 | 0.2089 | 0.2058 | 0.1193 | 0.6578 |
| 0.0403 | 0.1997 | 3.5330 | 0.5066 | 0.0471 | 0.5296 | 0.4233 | 0.0000 | 0.0000 | 0.0489 | 0.8286 | 0.0000 | 0.0000 | 0.0489 | 0.5959 | 0.1950 | 0.2091 | 0.1469 | 0.7185 |
| 0.2406 | 0.1811 | 3.0292 | 0.4058 | 0.3980 | 0.4634 | 0.1301 | 0.0085 | 0.0000 | 0.1807 | 0.9991 | 0.0009 | 0.0021 | 0.1807 | 0.4762 | 0.1828 | 0.3410 | 0.1175 | 0.7471 |
| 0.0300 | 0.1873 | 3.5786 | 0.5157 | 0.2806 | 0.3763 | 0.2449 | 0.0973 | 0.0000 | 0.0464 | 0.9903 | 0.0089 | 0.0000 | 0.0464 | 0.6384 | 0.1190 | 0.2426 | 0.1173 | 0.8699 |
| 0.0276 | 0.1330 | 4.1201 | 0.6240 | 0.0193 | 0.2674 | 0.7132 | 0.0000 | 0.0000 | 0.0000 | 0.9991 | 0.0009 | 0.0009 | 0.0000 | 0.3517 | 0.3241 | 0.3241 | 0.0745 | 0.7792 |
| 0.2940 | 0.2406 | 3.5617 | 0.5123 | 0.1137 | 0.3202 | 0.5661 | 0.0000 | 0.0000 | 0.0255 | 0.7113 | 0.2887 | 0.0026 | 0.0255 | 0.4988 | 0.2281 | 0.2731 | 0.1064 | 0.7544 |
| 0.1079 | 0.5099 | 4.0567 | 0.6113 | 0.0360 | 0.3273 | 0.6367 | 0.0000 | 0.0000 | 0.1836 | 0.8921 | 0.1079 | 0.0000 | 0.1836 | 0.6971 | 0.1418 | 0.1611 | 0.1067 | 0.7800 |
| 0.0000 | 0.1950 | 2.9133 | 0.3827 | 0.5271 | 0.3410 | 0.1319 | 0.0000 | 0.0000 | 0.0218 | 1.0000 | 0.0000 | 0.0000 | 0.0218 | 0.3457 | 0.0640 | 0.5903 | 0.1232 | 0.6007 |
| 0.0026 | 0.2276 | 3.8190 | 0.5638 | 0.1054 | 0.4042 | 0.4903 | 0.0000 | 0.0000 | 0.0657 | 1.0000 | 0.0000 | 0.0000 | 0.0657 | 0.6963 | 0.1497 | 0.1541 | 0.1440 | 0.9071 |
| 0.1107 | 0.1890 | 4.3685 | 0.6737 | 0.0045 | 0.4077 | 0.4527 | 0.1350 | 0.0000 | 0.0177 | 0.9829 | 0.0126 | 0.0000 | 0.0177 | 0.6844 | 0.1580 | 0.1576 | 0.1214 | 0.8788 |
| 0.0053 | 0.3307 | 3.6596 | 0.5319 | 0.1336 | 0.5344 | 0.3307 | 0.0013 | 0.3310 | 0.0009 | 0.9987 | 0.0013 | 0.0009 | 0.3319 | 0.2669 | 0.1338 | 0.5993 | 0.1098 | 0.7519 |
| 0.0356 | 0.1384 | 3.5110 | 0.5022 | 0.3096 | 0.1740 | 0.5164 | 0.0000 | 0.1850 | 0.0925 | 1.0000 | 0.0000 | 0.0472 | 0.2775 | 0.3521 | 0.0233 | 0.6247 | 0.0833 | 0.4826 |
| 0.0058 | 0.2035 | 2.8173 | 0.3635 | 0.5547 | 0.2686 | 0.1477 | 0.0000 | 0.0943 | 0.0943 | 0.9988 | 0.0012 | 0.0000 | 0.1887 | 0.2782 | 0.2288 | 0.4930 | 0.1271 | 0.8449 |
| 0.0022 | 0.1101 | 3.2203 | 0.4441 | 0.4427 | 0.1145 | 0.4427 | 0.0000 | 0.3112 | 0.3112 | 1.0000 | 0.0000 | 0.0008 | 0.6224 | 0.2801 | 0.0015 | 0.7184 | 0.0920 | 0.4907 |
| 0.0318 | 0.1924 | 2.9144 | 0.3829 | 0.4497 | 0.2306 | 0.3185 | 0.0013 | 0.0013 | 0.0025 | 1.0000 | 0.0000 | 0.0000 | 0.0038 | 0.3567 | 0.1584 | 0.4849 | 0.1467 | 0.8448 |
| 0.0959 | 0.1936 | 3.4477 | 0.4895 | 0.2350 | 0.3327 | 0.3835 | 0.0470 | 0.0969 | 0.3634 | 0.9060 | 0.0940 | 0.0242 | 0.4603 | 0.4207 | 0.1115 | 0.4677 | 0.0920 | 0.7630 |
| 0.1839 | 0.1825 | 2.7333 | 0.3467 | 0.4409 | 0.3022 | 0.2204 | 0.0365 | 0.4596 | 0.0368 | 0.9985 | 0.0015 | 0.0000 | 0.4963 | 0.2616 | 0.1849 | 0.5535 | 0.1176 | 0.7184 |
| 0.0035 | 0.4452 | 4.1589 | 0.6318 | 0.0954 | 0.3675 | 0.4452 | 0.0883 | 0.3630 | 0.3630 | 1.0000 | 0.0000 | 0.0000 | 0.7260 | 0.3963 | 0.1225 | 0.4812 | 0.1083 | 0.8211 |
| 0.3299 | 0.1846 | 3.3771 | 0.4754 | 0.3285 | 0.4157 | 0.2180 | 0.0363 | 0.2250 | 0.0011 | 0.9622 | 0.0378 | 0.0000 | 0.2261 | 0.2936 | 0.1235 | 0.5828 | 0.1173 | 0.5667 |
| 0.1695 | 0.1345 | 3.1107 | 0.4221 | 0.3907 | 0.3441 | 0.1992 | 0.0647 | 0.1028 | 0.1028 | 0.8680 | 0.1320 | 0.0000 | 0.2055 | 0.3275 | 0.1548 | 0.5177 | 0.1174 | 0.5473 |
| 0.1445 | 0.1165 | 3.1234 | 0.4247 | 0.3382 | 0.4591 | 0.1725 | 0.0280 | 0.0908 | 0.0954 | 0.8869 | 0.1131 | 0.0018 | 0.1871 | 0.3137 | 0.1517 | 0.5346 | 0.1199 | 0.5516 |
| 0.2454 | 0.0986 | 3.1297 | 0.4259 | 0.3140 | 0.5623 | 0.0986 | 0.0242 | 0.0191 | 0.1907 | 0.9024 | 0.0976 | 0.0008 | 0.2098 | 0.3193 | 0.2425 | 0.4382 | 0.1165 | 0.5354 |
| 0.1592 | 0.0552 | 2.8002 | 0.3600 | 0.4422 | 0.3985 | 0.1322 | 0.0260 | 0.1468 | 0.1468 | 0.9990 | 0.0010 | 0.0007 | 0.2937 | 0.2759 | 0.1661 | 0.5579 | 0.1131 | 0.5896 |
| 0.1396 | 0.0846 | 2.8972 | 0.3794 | 0.4670 | 0.3110 | 0.1945 | 0.0275 | 0.1555 | 0.1361 | 0.9989 | 0.0011 | 0.0008 | 0.2916 | 0.2903 | 0.1755 | 0.5342 | 0.1126 | 0.8548 |
| 0.0293 | 0.1884 | 4.0655 | 0.6131 | 0.0293 | 0.5095 | 0.1022 | 0.0000 | 0.0000 | 0.0817 | 0.6399 | 0.0011 | 0.0011 | 0.0817 | 0.4468 | 0.2428 | 0.3105 | 0.0950 | 0.8883 |
| 0.1484 | 0.3231 | 4.4886 | 0.6977 | 0.0011 | 0.7660 | 0.2329 | 0.0000 | 0.0000 | 0.1022 | 0.8231 | 0.1769 | 0.0020 | 0.1022 | 0.3844 | 0.3518 | 0.2637 | 0.0950 | 0.7725 |
| 0.1159 | 0.3741 | 4.0278 | 0.6056 | 0.0000 | 0.8518 | 0.1482 | 0.0000 | 0.0000 | 0.0895 | 0.8045 | 0.1955 | 0.0012 | 0.0895 | 0.4624 | 0.3368 | 0.2008 | 0.1164 | 0.8928 |
| 0.1991 | 0.4671 | 3.5330 | 0.5066 | 0.0000 | 0.8040 | 0.1960 | 0.0000 | 0.0000 | 0.1334 | 0.4196 | 0.5789 | 0.0040 | 0.1334 | 0.4220 | 0.4895 | 0.0884 | 0.1018 | 0.9037 |
| 0.0030 | 0.6033 | 2.7931 | 0.3586 | 0.0374 | 0.9551 | 0.0075 | 0.0000 | 0.0000 | 0.1313 | 0.8069 | 0.1916 | 0.0000 | 0.1313 | 0.4543 | 0.3391 | 0.2066 | 0.0802 | 0.8120 |
| 0.0412 | 0.1204 | 2.4849 | 0.2970 | 0.0000 | 0.8811 | 0.1189 | 0.0000 | 0.0000 | 0.0734 | 0.8796 | 0.1204 | 0.0000 | 0.0734 | 0.3811 | 0.3590 | 0.2599 | 0.0695 | 0.5107 |
| 0.0351 | 0.5626 | 4.0708 | 0.6142 | 0.0006 | 0.9510 | 0.0478 | 0.0000 | 0.0000 | 0.2142 | 0.9226 | 0.0774 | 0.0009 | 0.2142 | 0.4226 | 0.3182 | 0.2592 | 0.1230 | 0.6549 |
| 0.0031 | 0.2454 | 4.3752 | 0.6750 | 0.0012 | 0.6885 | 0.3103 | 0.0000 | 0.0000 | 0.1095 | 0.7344 | 0.2650 | 0.0000 | 0.1095 | 0.4929 | 0.3115 | 0.1957 | 0.1089 | 0.9273 |
| 0.0033 | 0.3748 | 2.5172 | 0.3034 | 0.0000 | 0.8657 | 0.0498 | 0.0017 | 0.0000 | 0.0398 | 0.7396 | 0.1774 | 0.0000 | 0.0398 | 0.4385 | 0.3526 | 0.2089 | 0.1292 | 0.6354 |
| 0.0027 | 0.1810 | 4.3411 | 0.6682 | 0.0000 | 0.5288 | 0.4268 | 0.0444 | 0.0000 | 0.2620 | 0.8838 | 0.1154 | 0.0000 | 0.2620 | 0.4803 | 0.1851 | 0.3346 | 0.1349 | 0.7859 |
| 0.0392 | 0.2759 | 3.8307 | 0.5661 | 0.0000 | 0.3715 | 0.6285 | 0.0000 | 0.0000 | 0.0016 | 0.9922 | 0.0078 | 0.0000 | 0.0016 | 0.4851 | 0.1113 | 0.4036 | 0.1080 | 0.2990 |
| 0.0054 | 0.4093 | 3.4530 | 0.4906 | 0.0000 | 0.7775 | 0.2214 | 0.0000 | 0.0000 | 0.2292 | 0.9957 | 0.0043 | 0.0017 | 0.2292 | 0.7632 | 0.1043 | 0.1324 | 0.1231 | 0.9037 |
| 0.0014 | 0.0723 | 4.1044 | 0.6209 | 0.0007 | 0.9104 | 0.0890 | 0.0000 | 0.0000 | 0.3195 | 1.0000 | 0.0000 | 0.0005 | 0.3195 | 0.6863 | 0.1525 | 0.1612 | 0.1181 | 0.9844 |
| 0.0008 | 0.1202 | 3.8575 | 0.5715 | 0.0008 | 0.8790 | 0.1202 | 0.0000 | 0.0000 | 0.1067 | 1.0000 | 0.0000 | 0.0000 | 0.1067 | 0.7520 | 0.1139 | 0.1342 | 0.1185 | 0.9746 |
| 0.0065 | 0.2841 | 4.1497 | 0.6299 | 0.0009 | 0.5562 | 0.4429 | 0.0000 | 0.0000 | 0.2830 | 0.9016 | 0.0056 | 0.0000 | 0.2830 | 0.3879 | 0.3591 | 0.2530 | 0.0740 | 0.9203 |
| 0.0050 | 0.6530 | 3.9729 | 0.5946 | 0.0000 | 0.6490 | 0.3510 | 0.0000 | 0.0000 | 0.2855 | 0.9960 | 0.0030 | 0.0000 | 0.2862 | 0.4381 | 0.2819 | 0.2799 | 0.0916 | 0.8498 |
| 0.0031 | 0.3150 | 3.9033 | 0.5807 | 0.0010 | 0.5295 | 0.4694 | 0.0000 | 0.0000 | 0.2804 | 0.9959 | 0.0031 | 0.0000 | 0.2804 | 0.3875 | 0.3081 | 0.3044 | 0.0787 | 0.8599 |
| 0.0052 | 0.6835 | 3.4386 | 0.4877 | 0.0000 | 0.8992 | 0.1008 | 0.0000 | 0.0000 | 0.2437 | 0.9612 | 0.0375 | 0.0019 | 0.2437 | 0.5392 | 0.2059 | 0.2550 | 0.0893 | 0.7323 |
| 0.0013 | 0.8088 | 3.1580 | 0.4316 | 0.0000 | 0.9599 | 0.0401 | 0.0000 | 0.0000 | 0.5098 | 0.9987 | 0.0013 | 0.0000 | 0.5098 | 0.3639 | 0.2822 | 0.3539 | 0.0883 | 0.6743 |
| 0.0010 | 0.7483 | 3.5851 | 0.5170 | 0.0010 | 0.8042 | 0.1938 | 0.0000 | 0.0000 | 0.1617 | 0.9499 | 0.0492 | 0.0000 | 0.1617 | 0.4591 | 0.2886 | 0.2523 | 0.0758 | 0.3167 |
| 0.0052 | 0.4361 | 3.7258 | 0.5452 | 0.0009 | 0.6273 | 0.3701 | 0.0009 | 0.0000 | 0.1479 | 0.9983 | 0.0017 | 0.0007 | 0.1479 | 0.5178 | 0.2283 | 0.2539 | 0.0878 | 0.8768 |
| 0.1178 | 0.1775 | 2.7739 | 0.3548 | 0.4465 | 0.2814 | 0.2721 | 0.0000 | 0.0801 | 0.2292 | 1.0000 | 0.0000 | 0.0016 | 0.3093 | 0.3807 | 0.2193 | 0.4000 | 0.1351 | 0.6768 |

| | | | | | | | | | | | | | | | | | | |
|---|---|---|---|---|---|---|---|---|---|---|---|---|---|---|---|---|---|---|
| 0.0396 | 0.0799 | 2.5535 | 0.3107 | 0.3813 | 0.4460 | 0.1728 | 0.0000 | 0.1200 | 0.0756 | 1.0000 | 0.0000 | 0.0006 | 0.2107 | 0.4073 | 0.1878 | 0.4049 | 0.1457 | 0.5187 |
| 0.0294 | 0.0872 | 2.6834 | 0.3367 | 0.4541 | 0.2616 | 0.2276 | 0.0000 | 0.0000 | 0.1017 | 0.9434 | 0.0000 | 0.0000 | 0.1017 | 0.3916 | 0.1645 | 0.4440 | 0.1385 | 0.8390 |
| 0.0242 | 0.2432 | 2.7970 | 0.3594 | 0.3895 | 0.3672 | 0.1948 | 0.0000 | 0.0000 | 0.0237 | 0.9516 | 0.0000 | 0.0000 | 0.0237 | 0.3893 | 0.2215 | 0.3893 | 0.1221 | 0.7171 |
| 0.0971 | 0.3073 | 3.5005 | 0.5001 | 0.0000 | 0.4326 | 0.5674 | 0.0000 | 0.1163 | 0.1477 | 0.9991 | 0.0009 | 0.1180 | 0.2651 | 0.6021 | 0.0569 | 0.3410 | 0.1386 | 0.8892 |
| 0.1160 | 0.4417 | 4.0306 | 0.6061 | 0.0569 | 0.6583 | 0.2848 | 0.0000 | 0.0000 | 0.2773 | 1.0000 | 0.0000 | 0.0000 | 0.2773 | 0.6326 | 0.1615 | 0.2059 | 0.1245 | 0.6425 |
| 0.0843 | 0.0667 | 2.8965 | 0.3793 | 0.4618 | 0.1743 | 0.3631 | 0.0008 | 0.0000 | 0.2176 | 1.0000 | 0.0000 | 0.0016 | 0.3261 | 0.4465 | 0.1616 | 0.3919 | 0.1427 | 0.5518 |
| 0.0807 | 0.1359 | 3.3223 | 0.4645 | 0.0011 | 0.5159 | 0.4798 | 0.0021 | 0.3015 | 0.1814 | 1.0000 | 0.0000 | 0.0502 | 0.4954 | 0.4702 | 0.0643 | 0.4655 | 0.1367 | 0.8790 |
| 0.0743 | 0.3786 | 3.8799 | 0.5760 | 0.1793 | 0.6407 | 0.1800 | 0.0000 | 0.0000 | 0.2531 | 0.9986 | 0.0014 | 0.0011 | 0.2531 | 0.5018 | 0.1950 | 0.3032 | 0.1091 | 0.8622 |
| 0.2754 | 0.2391 | 3.9021 | 0.5804 | 0.1475 | 0.4688 | 0.3837 | 0.0000 | 0.0000 | 0.0690 | 0.9985 | 0.0015 | 0.0007 | 0.0690 | 0.5330 | 0.2101 | 0.2569 | 0.1087 | 0.5705 |
| 0.1400 | 0.3900 | 3.4697 | 0.4939 | 0.0000 | 0.4600 | 0.5300 | 0.0100 | 0.0000 | 0.1487 | 0.9950 | 0.0050 | 0.6531 | 0.1487 | 0.5975 | 0.0975 | 0.3050 | 0.0966 | 0.9365 |
| 0.1427 | 0.1715 | 3.1785 | 0.4357 | 0.0044 | 0.4657 | 0.5288 | 0.0011 | 0.2139 | 0.3150 | 1.0000 | 0.0000 | 0.0035 | 0.5415 | 0.5109 | 0.0408 | 0.4483 | 0.1322 | 0.8844 |
| 0.0556 | 0.0865 | 2.8375 | 0.3675 | 0.5342 | 0.2756 | 0.1891 | 0.0000 | 0.2163 | 0.1081 | 1.0000 | 0.0000 | 0.0007 | 0.3244 | 0.4193 | 0.2113 | 0.3693 | 0.1522 | 0.7980 |
| 0.0343 | 0.0367 | 2.4432 | 0.2886 | 0.4908 | 0.3525 | 0.1554 | 0.0000 | 0.3706 | 0.1248 | 1.0000 | 0.0000 | 0.0000 | 0.4954 | 0.4253 | 0.1932 | 0.3815 | 0.1274 | 0.6182 |
| 0.0558 | 0.0827 | 2.7174 | 0.3435 | 0.5440 | 0.2213 | 0.2337 | 0.0000 | 0.1463 | 0.2909 | 1.0000 | 0.0000 | 0.0000 | 0.4384 | 0.3525 | 0.1309 | 0.5166 | 0.1443 | 0.6533 |
| 0.0812 | 0.2405 | 3.5207 | 0.5041 | 0.3352 | 0.4677 | 0.1972 | 0.0000 | 0.0000 | 0.2510 | 0.9598 | 0.0402 | 0.0000 | 0.2510 | 0.5476 | 0.1898 | 0.2626 | 0.1303 | 0.7586 |
| 0.0888 | 0.2970 | 3.9739 | 0.5948 | 0.0701 | 0.6523 | 0.2776 | 0.0000 | 0.0000 | 0.3190 | 0.9653 | 0.0347 | 0.0000 | 0.3190 | 0.5246 | 0.1939 | 0.2815 | 0.1237 | 0.7004 |
| 0.0342 | 0.2732 | 2.4380 | 0.2876 | 0.5464 | 0.3839 | 0.0697 | 0.0000 | 0.0000 | 0.1200 | 0.9986 | 0.0014 | 0.0012 | 0.1200 | 0.4063 | 0.1422 | 0.4514 | 0.0813 | 0.6558 |
| 0.0776 | 0.0613 | 2.4138 | 0.2828 | 0.4282 | 0.4926 | 0.0791 | 0.0000 | 0.0000 | 0.4369 | 0.9984 | 0.0016 | 0.0000 | 0.4369 | 0.2757 | 0.1938 | 0.5305 | 0.1263 | 0.5531 |
| 0.0397 | 0.3211 | 3.8679 | 0.5736 | 0.0936 | 0.4386 | 0.4678 | 0.0000 | 0.0000 | 0.1298 | 1.0000 | 0.0000 | 0.0032 | 0.1298 | 0.5807 | 0.2047 | 0.2146 | 0.1390 | 0.8175 |
| 0.1578 | 0.2480 | 3.0747 | 0.4149 | 0.3841 | 0.3201 | 0.2958 | 0.0000 | 0.0000 | 0.0632 | 1.0000 | 0.0000 | 0.0017 | 0.0632 | 0.3830 | 0.2606 | 0.3565 | 0.1243 | 0.8705 |
| 0.0044 | 0.1175 | 2.5165 | 0.3033 | 0.3551 | 0.4850 | 0.1590 | 0.0000 | 0.0000 | 0.3737 | 0.9982 | 0.0018 | 0.0006 | 0.3737 | 0.3138 | 0.0878 | 0.5984 | 0.1396 | 0.7502 |
| 0.0480 | 0.0969 | 2.5203 | 0.3041 | 0.6359 | 0.2211 | 0.1430 | 0.0000 | 0.0000 | 0.2052 | 1.0000 | 0.0000 | 0.0015 | 0.2052 | 0.3602 | 0.2240 | 0.4158 | 0.1267 | 0.5699 |
| 0.0009 | 0.0973 | 2.3773 | 0.2755 | 0.7025 | 0.2002 | 0.0973 | 0.0000 | 0.0000 | 0.3495 | 0.9991 | 0.0009 | 0.0018 | 0.3495 | 0.2709 | 0.1567 | 0.5724 | 0.1210 | 0.5491 |
| 0.1955 | 0.2334 | 3.9046 | 0.5809 | 0.2141 | 0.4560 | 0.3300 | 0.0000 | 0.0000 | 0.1481 | 1.0000 | 0.0000 | 0.0000 | 0.1481 | 0.5206 | 0.2218 | 0.2577 | 0.1148 | 0.6650 |
| 0.1044 | 0.0779 | 3.6368 | 0.5274 | 0.4954 | 0.2695 | 0.2338 | 0.0013 | 0.0000 | 0.4885 | 0.9987 | 0.0013 | 0.0007 | 0.4892 | 0.5962 | 0.1448 | 0.2590 | 0.1543 | 0.7048 |
| 0.0068 | 0.4068 | 4.2610 | 0.6522 | 0.0000 | 0.0983 | 0.9017 | 0.0000 | 0.0000 | 0.0000 | 0.9898 | 0.0102 | 0.0000 | 0.0000 | 0.9299 | 0.0367 | 0.0333 | 0.1163 | 0.7502 |
| 0.0000 | 0.5714 | 3.5926 | 0.5185 | 0.0000 | 0.8571 | 0.1429 | 0.0000 | 0.0000 | 0.0000 | 0.9643 | 0.0000 | 0.0000 | 0.0000 | 0.5062 | 0.2099 | 0.2840 | 0.1199 | 0.3930 |
| 0.1503 | 0.1411 | 4.2646 | 0.6529 | 0.0000 | 0.4847 | 0.5153 | 0.0000 | 0.0000 | 0.0000 | 0.9601 | 0.0399 | 0.0000 | 0.0000 | 0.6267 | 0.1974 | 0.1759 | 0.1183 | 0.7261 |
| 0.3308 | 0.4385 | 4.5654 | 0.7131 | 0.0038 | 0.4962 | 0.5000 | 0.0000 | 0.0000 | 0.0000 | 0.9808 | 0.0192 | 0.0000 | 0.0000 | 0.8628 | 0.1321 | 0.0051 | 0.1163 | 0.8502 |
| 0.0036 | 0.2760 | 4.5144 | 0.7029 | 0.0143 | 0.4516 | 0.5341 | 0.0000 | 0.0000 | 0.0000 | 0.9892 | 0.0072 | 0.0036 | 0.0000 | 0.6133 | 0.1799 | 0.2068 | 0.0930 | 0.8071 |
| 0.0000 | 0.0980 | 3.8392 | 0.5678 | 0.0039 | 0.6784 | 0.3176 | 0.0000 | 0.0000 | 0.0000 | 0.9961 | 0.0039 | 0.0000 | 0.0000 | 0.3908 | 0.1516 | 0.4575 | 0.0929 | 0.7774 |
| 0.0000 | 0.1934 | 3.2847 | 0.4569 | 0.0000 | 0.2518 | 0.7482 | 0.0000 | 0.0000 | 0.0000 | 1.0000 | 0.0000 | 0.0000 | 0.0000 | 0.7445 | 0.0182 | 0.2372 | 0.0868 | 0.6800 |
| 0.1023 | 0.2197 | 4.3118 | 0.6624 | 0.0152 | 0.2538 | 0.7311 | 0.0000 | 0.0000 | 0.0000 | 0.9053 | 0.0947 | 0.0000 | 0.0000 | 0.8619 | 0.0672 | 0.0710 | 0.1340 | 0.7580 |
| 0.0037 | 0.4030 | 4.5094 | 0.7019 | 0.0000 | 0.3507 | 0.6455 | 0.0000 | 0.0000 | 0.0000 | 0.9851 | 0.0149 | 0.0000 | 0.0000 | 0.6461 | 0.1704 | 0.1835 | 0.1055 | 0.8225 |
| 0.0000 | 0.2415 | 3.6570 | 0.5314 | 0.0048 | 0.0483 | 0.9469 | 0.0000 | 0.0000 | 0.0000 | 1.0000 | 0.0000 | 0.0000 | 0.0000 | 0.5866 | 0.1278 | 0.2856 | 0.0868 | 0.7998 |
| 0.0000 | 0.7589 | 4.5296 | 0.7059 | 0.0000 | 0.1344 | 0.8656 | 0.0000 | 0.0000 | 0.0000 | 1.0000 | 0.0000 | 0.0000 | 0.0000 | 0.8900 | 0.0560 | 0.0540 | 0.0593 | 0.7385 |
| 0.0000 | 0.2145 | 3.9550 | 0.5910 | 0.0000 | 0.1522 | 0.8478 | 0.0000 | 0.0000 | 0.0000 | 0.9965 | 0.0035 | 0.0000 | 0.0000 | 0.7537 | 0.1240 | 0.1223 | 0.0678 | 0.7597 |
| 0.0072 | 0.2816 | 3.8909 | 0.5782 | 0.0000 | 0.1588 | 0.8412 | 0.0000 | 0.0000 | 0.0000 | 0.9819 | 0.0108 | 0.0000 | 0.0000 | 0.8824 | 0.0515 | 0.0661 | 0.0985 | 0.7898 |
| 0.0216 | 0.7122 | 4.6426 | 0.7285 | 0.0000 | 0.4676 | 0.5324 | 0.0000 | 0.0000 | 0.0000 | 0.9964 | 0.0036 | 0.0000 | 0.0000 | 0.9248 | 0.0385 | 0.0367 | 0.0941 | 0.6890 |
| 0.0000 | 0.0000 | 2.0142 | 0.2028 | 0.0000 | 1.0000 | 0.0000 | 0.0000 | 0.0000 | 0.0000 | 0.9621 | 0.0379 | 0.0000 | 0.0000 | 0.4785 | 0.4194 | 0.1022 | 0.0947 | 0.9342 |
| 0.0103 | 0.5034 | 4.6735 | 0.7347 | 0.0000 | 0.2260 | 0.7740 | 0.0000 | 0.0000 | 0.0000 | 0.9966 | 0.0034 | 0.0000 | 0.0000 | 0.8912 | 0.0561 | 0.0527 | 0.1382 | 0.7390 |
| 0.0000 | 0.0000 | 2.5254 | 0.3051 | 0.0000 | 1.0000 | 0.0000 | 0.0000 | 0.0000 | 0.0000 | 1.0000 | 0.0000 | 0.0000 | 0.0000 | 0.5000 | 0.4390 | 0.0610 | 0.0678 | 0.7603 |
| 0.0000 | 0.0000 | 2.6316 | 0.3263 | 0.0000 | 1.0000 | 0.0000 | 0.0000 | 0.0000 | 0.0000 | 1.0000 | 0.0000 | 0.0000 | 0.0000 | 0.5000 | 0.3810 | 0.1190 | 0.0748 | 0.7150 |
| 0.0000 | 0.0000 | 2.7813 | 0.3563 | 0.0000 | 1.0000 | 0.0000 | 0.0000 | 0.0000 | 0.0000 | 1.0000 | 0.0000 | 0.0000 | 0.0000 | 0.5000 | 0.4815 | 0.0185 | 0.0428 | 0.8550 |
| 0.2983 | 0.0068 | 3.1747 | 0.4349 | 0.2915 | 0.5424 | 0.1593 | 0.0000 | 0.0000 | 0.0000 | 0.9661 | 0.0271 | 0.0000 | 0.0000 | 0.4007 | 0.1644 | 0.4349 | 0.1177 | 0.5682 |
| 0.0000 | 0.0460 | 3.1897 | 0.4379 | 0.0517 | 0.6034 | 0.3448 | 0.0000 | 0.0000 | 0.0000 | 1.0000 | 0.0000 | 0.0000 | 0.0000 | 0.5186 | 0.0539 | 0.4275 | 0.1449 | 0.7217 |
| 0.0549 | 0.0084 | 4.4009 | 0.6802 | 0.0000 | 0.3080 | 0.6920 | 0.0000 | 0.0000 | 0.0000 | 0.9072 | 0.0886 | 0.0000 | 0.0000 | 0.3826 | 0.2897 | 0.3277 | 0.0842 | 0.5988 |
| 0.0357 | 0.0179 | 2.8973 | 0.3795 | 0.1027 | 0.3616 | 0.5357 | 0.0000 | 0.0000 | 0.0000 | 0.9821 | 0.0179 | 0.0000 | 0.0000 | 0.5506 | 0.0551 | 0.3943 | 0.0974 | 0.5507 |
| 0.0250 | 0.0313 | 3.2688 | 0.4538 | 0.0063 | 0.4500 | 0.5438 | 0.0000 | 0.0000 | 0.0000 | 0.6188 | 0.3813 | 0.0000 | 0.0000 | 0.6958 | 0.1990 | 0.1052 | 0.0686 | 0.7642 |
| 0.0000 | 0.0000 | 4.6783 | 0.7357 | 0.0000 | 0.1217 | 0.8783 | 0.0000 | 0.0000 | 0.0000 | 0.9652 | 0.0348 | 0.0086 | 0.0000 | 0.9319 | 0.0362 | 0.0319 | 0.0314 | 0.9464 |
| 0.0238 | 0.3274 | 4.0655 | 0.6131 | 0.0060 | 0.5060 | 0.4881 | 0.0000 | 0.0000 | 0.0000 | 0.9524 | 0.0476 | 0.0059 | 0.0000 | 0.7954 | 0.0798 | 0.1248 | 0.0883 | 0.7796 |

| | | | | | | | | | | | | | | | | | | |
|---|---|---|---|---|---|---|---|---|---|---|---|---|---|---|---|---|---|---|
| 0.0152 | 0.0303 | 3.0606 | 0.4121 | 0.0152 | 0.4394 | 0.5455 | 0.0000 | 0.0000 | 0.0000 | 0.9848 | 0.0152 | 0.0000 | 0.0000 | 0.7516 | 0.1144 | 0.1340 | 0.1657 | 0.9242 |
| 0.0049 | 0.2611 | 4.1626 | 0.6325 | 0.0099 | 0.2167 | 0.7734 | 0.0000 | 0.0000 | 0.0000 | 1.0000 | 0.0000 | 0.0000 | 0.0000 | 0.7053 | 0.1289 | 0.1658 | 0.0996 | 0.9055 |
| 0.0000 | 0.2803 | 4.8536 | 0.7707 | 0.0000 | 0.5439 | 0.4561 | 0.0000 | 0.0000 | 0.0000 | 1.0000 | 0.0000 | 0.0000 | 0.0000 | 0.5460 | 0.2280 | 0.2259 | 0.0777 | 0.8853 |
| 0.0000 | 0.0373 | 4.4813 | 0.6963 | 0.0041 | 0.0124 | 0.9834 | 0.0000 | 0.0000 | 0.0000 | 1.0000 | 0.0000 | 0.0000 | 0.0000 | 0.5394 | 0.2303 | 0.2303 | 0.0752 | 0.7460 |
| 0.0000 | 0.0058 | 3.0175 | 0.4035 | 0.0000 | 0.4884 | 0.5116 | 0.0000 | 0.0000 | 0.0000 | 0.9012 | 0.0930 | 0.0000 | 0.0000 | 0.4341 | 0.2132 | 0.3527 | 0.1046 | 0.7685 |
| 0.2000 | 0.0207 | 3.3426 | 0.4685 | 0.1793 | 0.6172 | 0.2000 | 0.0000 | 0.0000 | 0.0000 | 0.9828 | 0.0138 | 0.0000 | 0.0000 | 0.4521 | 0.1338 | 0.4141 | 0.1250 | 0.6390 |
| 0.0196 | 0.3725 | 3.6667 | 0.5333 | 0.0392 | 0.6471 | 0.2941 | 0.0196 | 0.0000 | 0.7292 | 0.9216 | 0.0784 | 0.0052 | 0.7292 | 0.4014 | 0.2891 | 0.3095 | 0.1154 | 0.6681 |
| 0.0141 | 0.0845 | 2.2687 | 0.2537 | 0.3662 | 0.5352 | 0.0282 | 0.0563 | 0.0000 | 0.6962 | 0.9437 | 0.0563 | 0.0042 | 0.6962 | 0.5117 | 0.0892 | 0.3991 | 0.1059 | 0.8604 |
| 0.0190 | 0.4381 | 3.2784 | 0.4557 | 0.0190 | 0.7333 | 0.1333 | 0.0762 | 0.0000 | 0.5021 | 0.7143 | 0.2857 | 0.0622 | 0.5021 | 0.3524 | 0.3667 | 0.2810 | 0.1110 | 0.5356 |
| 0.0323 | 0.3871 | 3.9787 | 0.5957 | 0.0161 | 0.5806 | 0.1452 | 0.2419 | 0.0000 | 0.7328 | 0.9839 | 0.0161 | 0.0162 | 0.7328 | 0.4355 | 0.2177 | 0.3468 | 0.1154 | 0.7069 |
| 0.0545 | 0.1273 | 4.4490 | 0.6898 | 0.0182 | 0.7455 | 0.1273 | 0.0909 | 0.0000 | 0.7373 | 0.9818 | 0.0182 | 0.0092 | 0.7373 | 0.4167 | 0.2685 | 0.3148 | 0.1049 | 0.5786 |
| 0.0575 | 0.2529 | 4.4098 | 0.6820 | 0.0230 | 0.5747 | 0.0920 | 0.2874 | 0.0000 | 0.6742 | 0.9885 | 0.0115 | 0.0000 | 0.6742 | 0.6066 | 0.1066 | 0.2868 | 0.1144 | 0.8078 |
| 0.0125 | 0.3375 | 3.2963 | 0.4593 | 0.0500 | 0.5500 | 0.0625 | 0.3250 | 0.0000 | 0.6897 | 0.9875 | 0.0125 | 0.0038 | 0.6897 | 0.3750 | 0.2750 | 0.3500 | 0.0701 | 0.7417 |
| 0.1111 | 0.3333 | 3.8485 | 0.5697 | 0.0556 | 0.8056 | 0.0556 | 0.0556 | 0.0000 | 0.7921 | 0.9722 | 0.0278 | 0.0297 | 0.7921 | 0.5286 | 0.1857 | 0.2857 | 0.1036 | 0.6804 |
| 0.0331 | 0.5497 | 3.9933 | 0.5987 | 0.2649 | 0.6954 | 0.0331 | 0.0066 | 0.0000 | 0.5145 | 1.0000 | 0.0000 | 0.0000 | 0.5145 | 0.7704 | 0.0453 | 0.1843 | 0.0682 | 0.8070 |
| 0.0000 | 0.3837 | 4.2500 | 0.6500 | 0.0116 | 0.5116 | 0.4419 | 0.0233 | 0.0000 | 0.6195 | 0.9767 | 0.0233 | 0.0000 | 0.6195 | 0.4922 | 0.2364 | 0.2713 | 0.0994 | 0.8270 |
| 0.0513 | 0.2564 | 4.1316 | 0.6263 | 0.0128 | 0.9231 | 0.0385 | 0.0128 | 0.0000 | 0.6695 | 0.8077 | 0.1923 | 0.0042 | 0.6695 | 0.5130 | 0.2403 | 0.2468 | 0.0831 | 0.4833 |
| 0.0847 | 0.3983 | 4.0177 | 0.6035 | 0.0085 | 0.7966 | 0.0339 | 0.0339 | 0.0000 | 0.6020 | 0.8729 | 0.1271 | 0.0033 | 0.6020 | 0.5399 | 0.2236 | 0.2365 | 0.1192 | 0.6531 |
| 0.0182 | 0.5909 | 4.1569 | 0.6314 | 0.1364 | 0.7545 | 0.0273 | 0.0727 | 0.0000 | 0.5217 | 0.9818 | 0.0182 | 0.0000 | 0.5217 | 0.7000 | 0.0455 | 0.2545 | 0.0906 | 0.9111 |
| 0.0182 | 0.5000 | 4.4000 | 0.6800 | 0.0182 | 0.7727 | 0.1636 | 0.0364 | 0.0000 | 0.5926 | 0.9909 | 0.0091 | 0.0000 | 0.5926 | 0.5780 | 0.1972 | 0.2248 | 0.1100 | 0.6819 |
| 0.0240 | 0.2400 | 2.8710 | 0.3742 | 0.6400 | 0.3280 | 0.0240 | 0.0080 | 0.0000 | 0.5575 | 0.9920 | 0.0080 | 0.0070 | 0.5575 | 0.4427 | 0.0307 | 0.5267 | 0.1009 | 0.8088 |
| 0.0081 | 0.3740 | 4.0732 | 0.6146 | 0.0163 | 0.6098 | 0.0325 | 0.3333 | 0.0000 | 0.5303 | 0.9675 | 0.0325 | 0.0038 | 0.5303 | 0.5650 | 0.1098 | 0.3252 | 0.1152 | 0.8288 |
| 0.0000 | 0.2551 | 4.7490 | 0.7498 | 0.0000 | 0.2881 | 0.7119 | 0.0000 | 0.0000 | 0.0000 | 0.9835 | 0.0165 | 0.0000 | 0.0000 | 0.5398 | 0.1900 | 0.2702 | 0.1071 | 0.7656 |
| 0.0000 | 0.2845 | 4.8961 | 0.7792 | 0.0000 | 0.2026 | 0.7974 | 0.0000 | 0.0000 | 0.0000 | 0.9957 | 0.0043 | 0.0000 | 0.0000 | 0.5354 | 0.2172 | 0.2475 | 0.0876 | 0.8115 |
| 0.0052 | 0.0619 | 3.8854 | 0.5771 | 0.3093 | 0.2113 | 0.4794 | 0.0000 | 0.0000 | 0.0000 | 0.9794 | 0.0206 | 0.0000 | 0.0000 | 0.4592 | 0.1884 | 0.3524 | 0.0780 | 0.9083 |
| 0.0052 | 0.1813 | 4.3717 | 0.6743 | 0.0777 | 0.3679 | 0.5544 | 0.0000 | 0.0000 | 0.0000 | 0.9585 | 0.0415 | 0.0000 | 0.0000 | 0.5637 | 0.1998 | 0.2365 | 0.0908 | 0.8709 |
| 0.0281 | 0.2135 | 4.5254 | 0.7051 | 0.0000 | 0.4270 | 0.5730 | 0.0000 | 0.0000 | 0.0000 | 0.9551 | 0.0449 | 0.0000 | 0.0000 | 0.5075 | 0.2420 | 0.2505 | 0.1082 | 0.8493 |
| 0.0091 | 0.1727 | 4.5229 | 0.7046 | 0.0000 | 0.4955 | 0.5000 | 0.0045 | 0.0000 | 0.0000 | 0.9318 | 0.0682 | 0.0000 | 0.0000 | 0.4627 | 0.2686 | 0.2686 | 0.1030 | 0.8953 |
| 0.0095 | 0.3033 | 4.8048 | 0.7610 | 0.0000 | 0.2749 | 0.7204 | 0.0047 | 0.0000 | 0.0000 | 0.9953 | 0.0047 | 0.0000 | 0.0000 | 0.5537 | 0.2220 | 0.2243 | 0.0996 | 0.6963 |
| 0.0105 | 0.2842 | 4.6138 | 0.7228 | 0.0000 | 0.4211 | 0.5789 | 0.0000 | 0.0000 | 0.0000 | 0.9789 | 0.0211 | 0.0000 | 0.0000 | 0.5820 | 0.2037 | 0.2143 | 0.0997 | 0.9304 |
| 0.0000 | 0.0228 | 3.2696 | 0.4539 | 0.8219 | 0.1553 | 0.0183 | 0.0000 | 0.0179 | 0.0000 | 1.0000 | 0.0000 | 0.0000 | 0.0179 | 0.2917 | 0.2696 | 0.4387 | 0.0613 | 0.6568 |
| 0.0246 | 0.0656 | 3.3070 | 0.4614 | 0.5164 | 0.4098 | 0.0328 | 0.0000 | 0.4353 | 0.0345 | 1.0000 | 0.0000 | 0.0000 | 0.4741 | 0.1871 | 0.1082 | 0.7047 | 0.1148 | 0.7726 |
| 0.0000 | 0.0000 | 2.5234 | 0.3047 | 0.0374 | 0.0935 | 0.5140 | 0.0000 | 0.5506 | 0.0000 | 1.0000 | 0.0000 | 0.0162 | 0.5506 | 0.4766 | 0.0000 | 0.5234 | 0.1088 | 0.8113 |
| 0.0000 | 0.0000 | 2.2966 | 0.2593 | 0.0000 | 0.0828 | 0.3793 | 0.0000 | 0.4806 | 0.0000 | 1.0000 | 0.0000 | 0.0000 | 0.4876 | 0.4828 | 0.0000 | 0.5172 | 0.1051 | 0.8023 |
| 0.0000 | 0.0099 | 2.0100 | 0.2020 | 0.0248 | 0.0495 | 0.2822 | 0.0099 | 0.3574 | 0.0000 | 1.0000 | 0.0000 | 0.0000 | 0.3934 | 0.4860 | 0.0033 | 0.5107 | 0.0979 | 0.8958 |
| 0.0000 | 0.0000 | 2.7019 | 0.3404 | 0.0192 | 0.0865 | 0.5288 | 0.0000 | 0.0000 | 0.0370 | 1.0000 | 0.0000 | 0.0000 | 0.0370 | 0.4904 | 0.0000 | 0.5096 | 0.1165 | 0.6728 |
| 0.0000 | 0.0000 | 2.3728 | 0.2746 | 0.0292 | 0.0585 | 0.4444 | 0.0117 | 0.0272 | 0.0435 | 1.0000 | 0.0000 | 0.0000 | 0.0707 | 0.4834 | 0.0039 | 0.5127 | 0.1117 | 0.7752 |
| 0.0000 | 0.0000 | 2.3165 | 0.2633 | 0.0253 | 0.0633 | 0.4304 | 0.0000 | 0.0444 | 0.0778 | 1.0000 | 0.0000 | 0.0000 | 0.1222 | 0.4684 | 0.0000 | 0.5316 | 0.1064 | 0.7183 |
| 0.0000 | 0.0000 | 2.2214 | 0.2443 | 0.0143 | 0.0500 | 0.3929 | 0.0000 | 0.0443 | 0.0570 | 1.0000 | 0.0000 | 0.0000 | 0.1139 | 0.4821 | 0.0000 | 0.5179 | 0.1031 | 0.7304 |
| 0.0000 | 0.0000 | 2.8889 | 0.3778 | 0.0308 | 0.3385 | 0.3385 | 0.0308 | 0.0000 | 0.0000 | 1.0000 | 0.0000 | 0.0299 | 0.0000 | 0.4769 | 0.0000 | 0.5231 | 0.1440 | 0.5152 |
| 0.0000 | 0.0000 | 2.2675 | 0.2535 | 0.0126 | 0.1384 | 0.3459 | 0.0126 | 0.0400 | 0.0400 | 1.0000 | 0.0000 | 0.0000 | 0.0914 | 0.4885 | 0.0042 | 0.5073 | 0.1127 | 0.7346 |
| 0.0000 | 0.0000 | 2.6733 | 0.3347 | 0.0099 | 0.0495 | 0.5644 | 0.0000 | 0.3833 | 0.0000 | 1.0000 | 0.0000 | 0.0000 | 0.4179 | 0.4876 | 0.0000 | 0.5124 | 0.1104 | 0.6613 |
| 0.0143 | 0.0000 | 3.0500 | 0.4100 | 0.0143 | 0.0500 | 0.6643 | 0.0000 | 0.3532 | 0.0213 | 1.0000 | 0.0000 | 0.0000 | 0.4043 | 0.4798 | 0.0048 | 0.5155 | 0.1038 | 0.4186 |
| 0.0000 | 0.0000 | 2.6396 | 0.3279 | 0.0101 | 0.0251 | 0.5729 | 0.0101 | 0.3833 | 0.0173 | 1.0000 | 0.0000 | 0.0058 | 0.4207 | 0.4925 | 0.0000 | 0.5075 | 0.1074 | 0.5690 |
| 0.0575 | 0.0852 | 3.7467 | 0.5493 | 0.0830 | 0.0299 | 0.8595 | 0.0000 | 0.0000 | 0.4365 | 1.0000 | 0.0000 | 0.0006 | 0.4365 | 0.4720 | 0.0560 | 0.4720 | 0.0594 | 0.9369 |
| 0.0019 | 0.7399 | 4.8662 | 0.7732 | 0.0000 | 0.2126 | 0.7874 | 0.0000 | 0.0000 | 0.0615 | 1.0000 | 0.0000 | 0.0000 | 0.0615 | 0.8374 | 0.0691 | 0.0936 | 0.1010 | 0.8711 |
| 0.0000 | 0.0049 | 2.0887 | 0.2177 | 0.0033 | 0.0065 | 0.3709 | 0.0049 | 0.3904 | 0.0020 | 0.9984 | 0.0016 | 0.3624 | 0.3928 | 0.5022 | 0.0014 | 0.4965 | 0.0939 | 0.7844 |
| 0.0011 | 0.1106 | 3.8595 | 0.5719 | 0.0277 | 0.1980 | 0.7467 | 0.0000 | 0.1312 | 0.2787 | 1.0000 | 0.0000 | 0.0000 | 0.4099 | 0.5085 | 0.0096 | 0.4819 | 0.0679 | 0.8550 |
| 0.0029 | 0.0000 | 2.2782 | 0.2556 | 0.1927 | 0.1112 | 0.0959 | 0.6002 | 0.0009 | 0.0465 | 1.0000 | 0.0000 | 0.0009 | 0.0474 | 0.4980 | 0.0211 | 0.4808 | 0.0940 | 0.3285 |
| 0.1903 | 0.0000 | 2.5500 | 0.3100 | 0.3568 | 0.2854 | 0.3578 | 0.0000 | 0.0000 | 0.0000 | 1.0000 | 0.0000 | 0.0000 | 0.0000 | 0.6432 | 0.0000 | 0.3568 | 0.1188 | 0.6637 |
| 0.0036 | 0.0000 | 3.9061 | 0.5812 | 0.1805 | 0.0036 | 0.7256 | 0.0000 | 0.7432 | 0.0220 | 1.0000 | 0.0000 | 0.0000 | 0.7653 | 0.4994 | 0.0012 | 0.4994 | 0.0766 | 0.2348 |

| | | | | | | | | | | | | | | | | | | |
|---|---|---|---|---|---|---|---|---|---|---|---|---|---|---|---|---|---|---|
| 0.0008 | 0.0008 | 4.4980 | 0.6996 | 0.0000 | 0.0008 | 0.5004 | 0.0000 | 0.0000 | 0.0391 | 1.0000 | 0.0000 | 0.0000 | 0.0391 | 0.4997 | 0.0005 | 0.4997 | 0.0864 | 0.6336 |
| 0.0000 | 0.0000 | 3.0020 | 0.4004 | 0.5988 | 0.0020 | 0.3992 | 0.0000 | 0.0017 | 0.1675 | 1.0000 | 0.0000 | 0.0000 | 0.1692 | 0.5000 | 0.0000 | 0.5000 | 0.1207 | 0.5236 |
| 0.0021 | 0.0010 | 2.7459 | 0.3492 | 0.0000 | 0.5581 | 0.4419 | 0.0000 | 0.0000 | 0.0000 | 0.5550 | 0.4450 | 0.0000 | 0.0000 | 0.3827 | 0.3676 | 0.2497 | 0.0893 | 0.9387 |
| 0.0016 | 0.1972 | 3.1978 | 0.4396 | 0.1577 | 0.6404 | 0.1987 | 0.0016 | 0.0000 | 0.0379 | 0.9984 | 0.0000 | 0.0000 | 0.0379 | 0.5342 | 0.0137 | 0.4521 | 0.1100 | 0.7198 |
| 0.0000 | 0.8599 | 4.1642 | 0.6328 | 0.0000 | 0.0482 | 0.9518 | 0.0000 | 0.0000 | 0.0009 | 1.0000 | 0.0000 | 0.0000 | 0.0009 | 0.3649 | 0.3176 | 0.3176 | 0.0371 | 0.9068 |
| 0.0509 | 0.0038 | 3.5679 | 0.5136 | 0.2377 | 0.0491 | 0.7113 | 0.0000 | 0.0000 | 0.6360 | 0.9981 | 0.0019 | 0.0000 | 0.6360 | 0.4597 | 0.0805 | 0.4597 | 0.0648 | 0.7204 |
| 0.0429 | 0.0048 | 3.7949 | 0.5590 | 0.2003 | 0.0413 | 0.5994 | 0.0000 | 0.0623 | 0.5452 | 0.9984 | 0.0016 | 0.0006 | 0.6075 | 0.4658 | 0.0684 | 0.4658 | 0.0802 | 0.6618 |
| 0.0050 | 0.0429 | 3.3317 | 0.4663 | 0.3300 | 0.0083 | 0.6617 | 0.0000 | 0.0000 | 0.5269 | 0.9983 | 0.0017 | 0.0000 | 0.5269 | 0.4381 | 0.1254 | 0.4365 | 0.0707 | 0.6114 |
| 0.0000 | 0.0040 | 3.6016 | 0.5203 | 0.1992 | 0.0000 | 0.8008 | 0.0000 | 0.7127 | 0.0011 | 1.0000 | 0.0000 | 0.0000 | 0.7138 | 0.4993 | 0.0013 | 0.4993 | 0.0810 | 0.5611 |
| 0.0000 | 0.0000 | 4.1000 | 0.6200 | 0.0000 | 0.0000 | 0.8667 | 0.0000 | 0.1175 | 0.0012 | 1.0000 | 0.0000 | 0.0000 | 0.1187 | 0.5000 | 0.0000 | 0.5000 | 0.0483 | 0.8954 |
| 0.0000 | 0.1332 | 2.6671 | 0.3334 | 0.5277 | 0.3364 | 0.1346 | 0.0013 | 0.3558 | 0.0390 | 1.0000 | 0.0000 | 0.0000 | 0.4322 | 0.5651 | 0.0004 | 0.4345 | 0.1033 | 0.6542 |
| 0.0000 | 0.8202 | 4.2675 | 0.6535 | 0.0000 | 0.2675 | 0.7325 | 0.0000 | 0.0000 | 0.0432 | 1.0000 | 0.0000 | 0.0000 | 0.0432 | 0.5117 | 0.2442 | 0.2442 | 0.0761 | 0.7539 |
| 0.0000 | 0.0015 | 2.1204 | 0.2241 | 0.0030 | 0.0030 | 0.3820 | 0.0396 | 0.3552 | 0.1720 | 1.0000 | 0.0000 | 0.1504 | 0.5277 | 0.5015 | 0.0000 | 0.4985 | 0.0984 | 0.9958 |
| 0.0026 | 0.3981 | 3.8307 | 0.5661 | 0.0013 | 0.4008 | 0.5979 | 0.0000 | 0.0000 | 0.5688 | 1.0000 | 0.0000 | 0.0011 | 0.5688 | 0.5443 | 0.1772 | 0.2784 | 0.1332 | 0.8896 |
| 0.0000 | 0.0000 | 3.2255 | 0.4451 | 0.1285 | 0.6118 | 0.2571 | 0.0026 | 0.0509 | 0.1536 | 1.0000 | 0.0000 | 0.0010 | 0.2075 | 0.5006 | 0.0000 | 0.4994 | 0.0824 | 0.4138 |
| 0.0011 | 0.0021 | 1.8016 | 0.1603 | 0.0011 | 0.0054 | 0.2680 | 0.0547 | 0.1579 | 0.1141 | 0.9979 | 0.0021 | 0.2833 | 0.2959 | 0.5006 | 0.0017 | 0.4977 | 0.0781 | 0.8673 |
| 0.0000 | 0.0000 | 1.7333 | 0.1467 | 0.0000 | 0.2200 | 0.0800 | 0.0000 | 0.0000 | 0.0000 | 0.3000 | 0.0000 | 0.0000 | 0.0000 | 0.4762 | 0.1905 | 0.3333 | 0.1724 | 0.8733 |
| 0.1862 | 0.0286 | 4.7143 | 0.7429 | 0.0230 | 0.2848 | 0.6922 | 0.0000 | 0.0000 | 0.0028 | 0.9991 | 0.0009 | 0.0000 | 0.0028 | 0.8464 | 0.0713 | 0.0823 | 0.0605 | 0.9260 |
| 0.0000 | 0.0025 | 1.5372 | 0.1074 | 0.0323 | 0.0050 | 0.1861 | 0.0000 | 0.3569 | 0.0787 | 1.0000 | 0.0000 | 0.2535 | 0.4356 | 0.5025 | 0.0000 | 0.4975 | 0.0585 | 0.9023 |
| 0.0010 | 0.1574 | 3.8929 | 0.5786 | 0.0262 | 0.1333 | 0.8132 | 0.0010 | 0.1724 | 0.0052 | 1.0000 | 0.0000 | 0.0009 | 0.1776 | 0.5430 | 0.0178 | 0.4391 | 0.0723 | 0.6204 |
| 0.0006 | 0.0000 | 2.6952 | 0.3390 | 0.0637 | 0.0675 | 0.2372 | 0.2372 | 0.0000 | 0.0840 | 0.6057 | 0.0000 | 0.0000 | 0.1120 | 0.4965 | 0.0351 | 0.4684 | 0.1244 | 0.5499 |
| 0.0020 | 0.0010 | 2.7247 | 0.3449 | 0.3806 | 0.4160 | 0.2034 | 0.0000 | 0.0000 | 0.0000 | 1.0000 | 0.0000 | 0.0000 | 0.0000 | 0.5361 | 0.0341 | 0.4298 | 0.1358 | 0.7850 |
| 0.0023 | 0.7988 | 3.8469 | 0.5694 | 0.0194 | 0.4918 | 0.4887 | 0.0000 | 0.0000 | 0.2897 | 1.0000 | 0.0000 | 0.0000 | 0.2897 | 0.6149 | 0.1627 | 0.2225 | 0.0954 | 0.9593 |
| 0.0009 | 0.0000 | 2.7801 | 0.3560 | 0.5541 | 0.4238 | 0.0222 | 0.0000 | 0.0000 | 0.0018 | 1.0000 | 0.0000 | 0.0000 | 0.0018 | 0.5439 | 0.0000 | 0.4561 | 0.0808 | 0.4496 |
| 0.0000 | 0.0000 | 1.9738 | 0.1948 | 0.0000 | 0.9990 | 0.0010 | 0.0000 | 0.0000 | 0.0000 | 1.0000 | 0.0000 | 0.0000 | 0.0000 | 0.4969 | 0.4784 | 0.0247 | 0.0157 | 0.9327 |
| 0.0021 | 0.1065 | 3.4544 | 0.4909 | 0.1420 | 0.2713 | 0.2486 | 0.3374 | 0.0000 | 0.0651 | 1.0000 | 0.0000 | 0.0020 | 0.0814 | 0.6031 | 0.0246 | 0.3723 | 0.1002 | 0.6147 |
| 0.1778 | 0.1773 | 3.4549 | 0.4910 | 0.1385 | 0.3431 | 0.2650 | 0.2020 | 0.0881 | 0.0348 | 1.0000 | 0.0000 | 0.0022 | 0.1230 | 0.5208 | 0.1100 | 0.3692 | 0.1196 | 0.6737 |
| 0.0649 | 0.0170 | 2.1690 | 0.2338 | 0.4417 | 0.4934 | 0.0328 | 0.0158 | 0.1410 | 0.2644 | 1.0000 | 0.0000 | 0.0352 | 0.4054 | 0.4788 | 0.1218 | 0.3994 | 0.1116 | 0.6155 |
| 0.0579 | 0.2743 | 3.0296 | 0.4059 | 0.2754 | 0.4197 | 0.2743 | 0.0022 | 0.0000 | 0.4069 | 0.9989 | 0.0011 | 0.0013 | 0.4069 | 0.5849 | 0.1018 | 0.3133 | 0.1259 | 0.5614 |
| 0.0194 | 0.1986 | 2.9038 | 0.3808 | 0.0921 | 0.6173 | 0.1986 | 0.0727 | 0.2692 | 0.0725 | 1.0000 | 0.0000 | 0.0828 | 0.3416 | 0.6194 | 0.0856 | 0.2950 | 0.1167 | 0.5647 |
| 0.0907 | 0.2058 | 3.1827 | 0.4365 | 0.3378 | 0.3684 | 0.2695 | 0.0018 | 0.3215 | 0.1055 | 1.0000 | 0.0000 | 0.0005 | 0.4270 | 0.4999 | 0.1584 | 0.3417 | 0.1227 | 0.4572 |
| 0.0970 | 0.1766 | 3.2585 | 0.4517 | 0.0803 | 0.6769 | 0.1606 | 0.0013 | 0.1750 | 0.1400 | 1.0000 | 0.0000 | 0.1400 | 0.3150 | 0.5536 | 0.1182 | 0.3282 | 0.1191 | 0.6196 |
| 0.0050 | 0.3303 | 2.9335 | 0.3867 | 0.3787 | 0.2417 | 0.2518 | 0.0010 | 0.3482 | 0.0986 | 0.9980 | 0.0010 | 0.0000 | 0.4468 | 0.4282 | 0.1210 | 0.4508 | 0.1300 | 0.5779 |
| 0.0261 | 0.0483 | 3.0825 | 0.4165 | 0.1217 | 0.6106 | 0.1459 | 0.1208 | 0.0000 | 0.0000 | 1.0000 | 0.0000 | 0.4581 | 0.0000 | 0.5775 | 0.0895 | 0.3330 | 0.1058 | 0.7139 |
| 0.0049 | 0.2230 | 2.8553 | 0.3711 | 0.4686 | 0.2593 | 0.2701 | 0.0020 | 0.1317 | 0.1982 | 1.0000 | 0.0000 | 0.0000 | 0.3298 | 0.5984 | 0.0517 | 0.3499 | 0.1057 | 0.8432 |
| 0.0027 | 0.0027 | 2.0639 | 0.2128 | 0.2730 | 0.6541 | 0.0378 | 0.0351 | 0.0000 | 0.0000 | 0.9662 | 0.0338 | 0.4583 | 0.0000 | 0.5135 | 0.2747 | 0.2117 | 0.1731 | 0.9495 |
| 0.0000 | 0.0020 | 1.8839 | 0.1768 | 0.7630 | 0.2340 | 0.0020 | 0.0010 | 0.0000 | 0.0000 | 1.0000 | 0.0000 | 0.2767 | 0.0000 | 0.4968 | 0.0349 | 0.4683 | 0.0720 | 0.6037 |
| 0.0000 | 0.2965 | 4.3696 | 0.6739 | 0.0000 | 0.6105 | 0.3895 | 0.0000 | 0.0000 | 0.0000 | 0.9942 | 0.0058 | 0.0000 | 0.0000 | 0.8007 | 0.1051 | 0.0942 | 0.0627 | 0.7163 |
| 0.0045 | 0.1205 | 4.7758 | 0.7552 | 0.0000 | 0.6518 | 0.3482 | 0.0000 | 0.0000 | 0.0000 | 0.9955 | 0.0045 | 0.0000 | 0.0000 | 0.4275 | 0.2885 | 0.2840 | 0.0677 | 0.9459 |
| 0.0049 | 0.4903 | 4.1951 | 0.6390 | 0.0000 | 0.0097 | 0.9903 | 0.0000 | 0.0000 | 0.0000 | 1.0000 | 0.0000 | 0.0000 | 0.0000 | 0.9252 | 0.0374 | 0.0374 | 0.0925 | 0.5584 |
| 0.0153 | 0.2137 | 3.3594 | 0.4719 | 0.0153 | 0.2824 | 0.6947 | 0.0076 | 0.0000 | 0.0000 | 0.9771 | 0.0229 | 0.4449 | 0.0000 | 0.7608 | 0.0318 | 0.2074 | 0.0941 | 0.7496 |
| 0.0070 | 0.5734 | 4.4615 | 0.6923 | 0.0070 | 0.0559 | 0.9371 | 0.0000 | 0.0000 | 0.0000 | 0.9860 | 0.0140 | 0.0000 | 0.0000 | 0.9254 | 0.0373 | 0.0373 | 0.1168 | 0.6985 |
| 0.0000 | 0.0000 | 3.8741 | 0.5748 | 0.0000 | 0.0070 | 0.9930 | 0.0000 | 0.0000 | 0.0000 | 1.0000 | 0.0000 | 0.0000 | 0.0000 | 0.6142 | 0.1911 | 0.1946 | 0.0495 | 0.9916 |
| 0.0041 | 0.1162 | 3.8755 | 0.5751 | 0.0000 | 0.2905 | 0.7095 | 0.0000 | 0.0000 | 0.0000 | 1.0000 | 0.0000 | 0.0000 | 0.0000 | 0.4149 | 0.2925 | 0.2925 | 0.0694 | 0.9720 |
| 0.0038 | 0.1692 | 4.4170 | 0.6834 | 0.0000 | 0.2615 | 0.7385 | 0.0000 | 0.0000 | 0.0000 | 1.0000 | 0.0000 | 0.0000 | 0.0000 | 0.5199 | 0.2400 | 0.2400 | 0.0862 | 0.8638 |
| 0.0000 | 0.0048 | 4.4952 | 0.6990 | 0.0000 | 0.3014 | 0.6986 | 0.0000 | 0.0000 | 0.0000 | 0.9952 | 0.0048 | 0.0000 | 0.0000 | 0.6651 | 0.1675 | 0.1675 | 0.1178 | 0.7397 |
| 0.0407 | 0.3252 | 3.3252 | 0.4650 | 0.0081 | 0.9350 | 0.0569 | 0.0000 | 0.0000 | 0.0000 | 1.0000 | 0.0000 | 0.5341 | 0.0000 | 0.6870 | 0.0081 | 0.3049 | 0.0874 | 0.5646 |
| 0.0054 | 0.0108 | 3.0324 | 0.4065 | 0.0000 | 0.2000 | 0.8000 | 0.0000 | 0.0000 | 0.0000 | 1.0000 | 0.0000 | 0.0054 | 0.0000 | 0.5144 | 0.0090 | 0.4766 | 0.1028 | 0.9533 |
| 0.0000 | 0.0588 | 2.1765 | 0.2353 | 0.0147 | 0.1029 | 0.8824 | 0.0000 | 0.0000 | 0.0000 | 1.0000 | 0.0000 | 0.0000 | 0.0000 | 0.5294 | 0.0074 | 0.4632 | 0.0284 | 0.9210 |
| 0.0044 | 0.2281 | 4.7149 | 0.7430 | 0.0000 | 0.1974 | 0.8026 | 0.0000 | 0.0000 | 0.0000 | 1.0000 | 0.0000 | 0.0000 | 0.0000 | 0.6966 | 0.1506 | 0.1528 | 0.0907 | 0.8335 |
| 0.0115 | 0.2586 | 4.0287 | 0.6057 | 0.1782 | 0.3161 | 0.5057 | 0.0000 | 0.0000 | 0.0000 | 1.0000 | 0.0000 | 0.0000 | 0.0000 | 0.8477 | 0.0230 | 0.1293 | 0.0786 | 0.9674 |

| | | | | | | | | | | | | | | | | | | |
|---|---|---|---|---|---|---|---|---|---|---|---|---|---|---|---|---|---|---|
| 0.0198 | 0.0149 | 4.3416 | 0.6683 | 0.1139 | 0.0446 | 0.8416 | 0.0000 | 0.0000 | 0.0000 | 0.9901 | 0.0099 | 0.0049 | 0.0000 | 0.9175 | 0.0116 | 0.0710 | 0.0586 | 0.9365 |
| 0.0000 | 0.0000 | 1.6711 | 0.1342 | 0.9408 | 0.0461 | 0.0132 | 0.0000 | 0.0000 | 0.0000 | 1.0000 | 0.0000 | 0.0000 | 0.0000 | 0.4801 | 0.0464 | 0.4735 | 0.0715 | 0.7257 |
| 0.0000 | 0.2209 | 4.7339 | 0.7468 | 0.0000 | 0.5221 | 0.4779 | 0.0000 | 0.0000 | 0.0000 | 1.0000 | 0.0000 | 0.0000 | 0.0000 | 0.4933 | 0.2534 | 0.2534 | 0.0794 | 0.9204 |
| 0.0040 | 0.1325 | 4.4758 | 0.6952 | 0.0000 | 0.2610 | 0.7390 | 0.0000 | 0.0000 | 0.0000 | 1.0000 | 0.0000 | 0.0000 | 0.0000 | 0.6996 | 0.1512 | 0.1492 | 0.1175 | 0.8149 |
| 0.0534 | 0.2214 | 4.0000 | 0.6000 | 0.0229 | 0.4046 | 0.5573 | 0.0153 | 0.0000 | 0.0000 | 0.9542 | 0.0458 | 0.4802 | 0.0000 | 0.8385 | 0.0538 | 0.1077 | 0.0924 | 0.9107 |
| 0.0000 | 0.4678 | 4.4561 | 0.6912 | 0.0000 | 0.5146 | 0.4854 | 0.0000 | 0.0000 | 0.0000 | 1.0000 | 0.0000 | 0.0000 | 0.0000 | 0.8177 | 0.0838 | 0.0984 | 0.0754 | 0.4535 |
| 0.0000 | 0.0059 | 4.0588 | 0.6118 | 0.3529 | 0.0647 | 0.5824 | 0.0000 | 0.0000 | 0.0000 | 1.0000 | 0.0000 | 0.0000 | 0.0000 | 0.6745 | 0.1392 | 0.1863 | 0.1040 | 0.7111 |
| 0.0000 | 0.0109 | 2.2065 | 0.2413 | 0.0109 | 0.9674 | 0.0217 | 0.0000 | 0.0000 | 0.0000 | 0.8370 | 0.1630 | 0.0000 | 0.0000 | 0.5055 | 0.4231 | 0.0714 | 0.1343 | 0.8444 |
| 0.0106 | 0.0000 | 2.0851 | 0.2170 | 0.1702 | 0.4681 | 0.3617 | 0.0000 | 0.0000 | 0.0000 | 0.9043 | 0.0957 | 0.0000 | 0.0000 | 0.4764 | 0.1504 | 0.3732 | 0.1466 | 0.7795 |
| 0.0000 | 0.0000 | 2.0959 | 0.2192 | 0.9592 | 0.0408 | 0.0000 | 0.0000 | 0.0000 | 0.0000 | 1.0000 | 0.0000 | 0.0926 | 0.0000 | 0.5023 | 0.0023 | 0.4955 | 0.0139 | 0.9890 |
| 0.0061 | 0.0061 | 2.0938 | 0.2188 | 0.4939 | 0.1098 | 0.3720 | 0.0244 | 0.0000 | 0.0000 | 0.9939 | 0.0061 | 0.0000 | 0.0000 | 0.5051 | 0.0051 | 0.4898 | 0.0953 | 0.5845 |
| 0.0000 | 0.0000 | 2.4106 | 0.2821 | 0.0152 | 0.9772 | 0.0076 | 0.0000 | 0.0000 | 0.0000 | 0.9810 | 0.0190 | 0.0000 | 0.0000 | 0.4961 | 0.2934 | 0.2104 | 0.1719 | 0.9688 |
| 0.0000 | 0.0000 | 2.2933 | 0.2587 | 0.0000 | 0.6522 | 0.0000 | 0.0000 | 0.0000 | 0.0000 | 0.6522 | 0.0000 | 0.0000 | 0.0000 | 0.4985 | 0.4758 | 0.0258 | 0.0510 | 0.8831 |
| 0.0145 | 0.0000 | 2.0797 | 0.2159 | 0.0072 | 0.9928 | 0.0000 | 0.0000 | 0.0000 | 0.0000 | 0.9928 | 0.0072 | 0.0000 | 0.0000 | 0.4951 | 0.1642 | 0.3407 | 0.1574 | 0.8279 |
| 0.0000 | 0.0000 | 2.1528 | 0.2306 | 0.0023 | 0.6157 | 0.0579 | 0.0000 | 0.0000 | 0.0000 | 1.0000 | 0.0000 | 0.0023 | 0.0000 | 0.4974 | 0.4418 | 0.0608 | 0.1058 | 0.7419 |
| 0.0078 | 0.0000 | 4.8359 | 0.7672 | 0.0000 | 0.0625 | 0.9375 | 0.0000 | 0.0000 | 0.0000 | 0.9688 | 0.0313 | 0.0000 | 0.0000 | 0.9674 | 0.0182 | 0.0143 | 0.0177 | 0.9728 |
| 0.0000 | 0.0052 | 3.7577 | 0.5515 | 0.4124 | 0.0052 | 0.5825 | 0.0000 | 0.0000 | 0.0000 | 1.0000 | 0.0000 | 0.0000 | 0.0000 | 0.4192 | 0.1873 | 0.3935 | 0.1041 | 0.5921 |
| 0.0022 | 0.0022 | 1.8689 | 0.1738 | 0.1729 | 0.8137 | 0.0111 | 0.0022 | 0.0000 | 0.0000 | 0.9956 | 0.0022 | 0.1174 | 0.0000 | 0.4744 | 0.3374 | 0.1881 | 0.1284 | 0.8419 |
| 0.0000 | 0.0000 | 2.1601 | 0.2320 | 0.0000 | 0.7153 | 0.2847 | 0.0000 | 0.0000 | 0.0000 | 0.9929 | 0.0071 | 0.0000 | 0.0000 | 0.4911 | 0.2420 | 0.2669 | 0.1644 | 0.9723 |
| 0.0000 | 0.0000 | 2.5000 | 0.3000 | 0.0000 | 0.0816 | 0.0000 | 0.0000 | 0.0000 | 0.0000 | 0.0816 | 0.0000 | 0.0000 | 0.0000 | 0.5000 | 0.2188 | 0.2813 | 0.1135 | 0.5726 |
| 0.0000 | 0.0098 | 3.0980 | 0.4196 | 0.0098 | 0.5098 | 0.4804 | 0.0000 | 0.0000 | 0.0000 | 0.9118 | 0.0882 | 0.0000 | 0.0000 | 0.4232 | 0.1977 | 0.3791 | 0.1088 | 0.7985 |
| 0.0106 | 0.0532 | 3.0106 | 0.4021 | 0.0851 | 0.6596 | 0.2553 | 0.0000 | 0.0000 | 0.0000 | 0.9468 | 0.0532 | 0.0000 | 0.0000 | 0.4823 | 0.1578 | 0.3599 | 0.1304 | 0.7151 |
| 0.1013 | 0.1013 | 3.0380 | 0.4076 | 0.0127 | 0.6329 | 0.3544 | 0.0000 | 0.0000 | 0.0000 | 0.7089 | 0.2911 | 0.0000 | 0.0000 | 0.4915 | 0.2735 | 0.2350 | 0.0994 | 0.8748 |
| 0.0000 | 0.0000 | 2.1235 | 0.2247 | 0.0000 | 0.6412 | 0.0059 | 0.0000 | 0.0000 | 0.0000 | 1.0000 | 0.0000 | 0.1905 | 0.0000 | 0.4695 | 0.4634 | 0.0671 | 0.0899 | 0.7887 |
| 0.0000 | 0.0000 | 2.1135 | 0.2227 | 0.0000 | 0.7784 | 0.0054 | 0.0000 | 0.0000 | 0.0000 | 1.0000 | 0.0000 | 0.0212 | 0.0000 | 0.4786 | 0.4744 | 0.0470 | 0.0833 | 0.7337 |
| 0.0000 | 0.0000 | 2.1429 | 0.2286 | 0.0000 | 0.8571 | 0.0000 | 0.0000 | 0.0000 | 0.0000 | 1.0000 | 0.0000 | 0.0000 | 0.0000 | 0.5000 | 0.5000 | 0.0000 | 0.0598 | 0.8292 |
| 0.0256 | 0.0000 | 2.2051 | 0.2410 | 0.4103 | 0.5641 | 0.0000 | 0.0000 | 0.0000 | 0.0000 | 0.9487 | 0.0256 | 0.0000 | 0.0000 | 0.4944 | 0.1778 | 0.3278 | 0.1690 | 0.8599 |
| 0.0000 | 0.0000 | 2.4500 | 0.2900 | 0.0000 | 0.9500 | 0.0000 | 0.0000 | 0.0000 | 0.0000 | 1.0000 | 0.0000 | 0.0000 | 0.0000 | 0.5000 | 0.4500 | 0.0500 | 0.0710 | 0.6417 |
| 0.0000 | 0.0000 | 2.0889 | 0.2178 | 0.0704 | 0.8222 | 0.0926 | 0.0000 | 0.0000 | 0.0000 | 0.9963 | 0.0000 | 0.0000 | 0.0000 | 0.4828 | 0.3276 | 0.1897 | 0.1346 | 0.8834 |
| 0.0115 | 0.0575 | 2.8046 | 0.3609 | 0.5747 | 0.1897 | 0.2356 | 0.0000 | 0.0000 | 0.0000 | 0.9943 | 0.0057 | 0.0000 | 0.0000 | 0.1820 | 0.0670 | 0.7510 | 0.0897 | 0.4124 |
| 0.0224 | 0.0000 | 4.1791 | 0.6358 | 0.0000 | 0.2239 | 0.7761 | 0.0000 | 0.0000 | 0.0000 | 0.8507 | 0.1493 | 0.0000 | 0.0000 | 0.7711 | 0.1368 | 0.0920 | 0.0653 | 0.9595 |
| 0.0105 | 0.0000 | 2.2579 | 0.2516 | 0.0471 | 0.7801 | 0.1675 | 0.0052 | 0.0000 | 0.0000 | 1.0000 | 0.0000 | 0.3055 | 0.0000 | 0.4982 | 0.3423 | 0.1595 | 0.1498 | 0.7179 |
| 0.0092 | 0.1290 | 4.8357 | 0.7671 | 0.0000 | 0.3272 | 0.6728 | 0.0000 | 0.0000 | 0.0000 | 0.9908 | 0.0092 | 0.0000 | 0.0000 | 0.5180 | 0.2387 | 0.2433 | 0.0888 | 0.9157 |
| 0.0084 | 0.1983 | 4.4726 | 0.6945 | 0.0000 | 0.2616 | 0.7384 | 0.0000 | 0.0000 | 0.0000 | 0.9916 | 0.0084 | 0.0000 | 0.0000 | 0.7518 | 0.1273 | 0.1210 | 0.1274 | 0.7031 |
| 0.0204 | 0.6531 | 4.7041 | 0.7408 | 0.0102 | 0.0816 | 0.9082 | 0.0000 | 0.0000 | 0.0000 | 0.9796 | 0.0204 | 0.0000 | 0.0000 | 0.8010 | 0.1020 | 0.0969 | 0.0768 | 0.6668 |
| 0.0172 | 0.4052 | 4.4655 | 0.6931 | 0.0000 | 0.3190 | 0.6810 | 0.0000 | 0.0000 | 0.0000 | 0.9828 | 0.0172 | 0.0000 | 0.0000 | 0.6695 | 0.1652 | 0.1652 | 0.0880 | 0.8310 |
| 0.0000 | 0.2128 | 4.1230 | 0.6246 | 0.0000 | 0.0266 | 0.9734 | 0.0000 | 0.0000 | 0.0000 | 1.0000 | 0.0000 | 0.0000 | 0.0000 | 0.9109 | 0.0446 | 0.0446 | 0.0888 | 0.4836 |
| 0.0000 | 0.1420 | 4.7727 | 0.7545 | 0.0000 | 0.0057 | 0.9943 | 0.0000 | 0.0000 | 0.0000 | 1.0000 | 0.0000 | 0.0000 | 0.0000 | 0.8996 | 0.0473 | 0.0530 | 0.0723 | 0.8143 |
| 0.0000 | 0.1985 | 3.5704 | 0.5141 | 0.0000 | 0.0368 | 0.9632 | 0.0000 | 0.0000 | 0.0000 | 0.9926 | 0.0074 | 0.0000 | 0.0000 | 0.4864 | 0.2568 | 0.2568 | 0.0713 | 0.8276 |
| 0.0147 | 0.0000 | 2.0735 | 0.2147 | 0.0147 | 0.0882 | 0.8971 | 0.0000 | 0.0000 | 0.0000 | 0.9706 | 0.0147 | 0.6477 | 0.0000 | 0.5147 | 0.0147 | 0.4706 | 0.0442 | 0.9498 |
| 0.0902 | 0.0129 | 3.2887 | 0.4577 | 0.2320 | 0.0902 | 0.5773 | 0.0000 | 0.0000 | 0.2265 | 1.0000 | 0.0000 | 0.1456 | 0.2265 | 0.5082 | 0.0945 | 0.3973 | 0.0900 | 0.4244 |
| 0.0385 | 0.0000 | 3.1731 | 0.4346 | 0.3462 | 0.0577 | 0.5769 | 0.0000 | 0.0000 | 0.0352 | 1.0000 | 0.0000 | 0.7035 | 0.0352 | 0.4519 | 0.1346 | 0.4135 | 0.0890 | 0.7745 |
| 0.0440 | 0.0000 | 3.5824 | 0.5165 | 0.0989 | 0.0440 | 0.8132 | 0.0000 | 0.0000 | 0.0000 | 1.0000 | 0.0000 | 0.0421 | 0.0000 | 0.4982 | 0.0476 | 0.4542 | 0.0465 | 0.9144 |
| 0.0714 | 0.0000 | 2.4643 | 0.2929 | 0.2143 | 0.0714 | 0.2857 | 0.0000 | 0.0000 | 0.3176 | 1.0000 | 0.0000 | 0.0235 | 0.3176 | 0.4435 | 0.1131 | 0.4435 | 0.1242 | 0.6093 |
| 0.2000 | 0.0000 | 2.7250 | 0.3450 | 0.1875 | 0.2000 | 0.2625 | 0.0000 | 0.0000 | 0.0526 | 1.0000 | 0.0000 | 0.1053 | 0.0526 | 0.4771 | 0.1083 | 0.4146 | 0.0962 | 0.5717 |
| 0.1724 | 0.0000 | 2.3793 | 0.2759 | 0.2414 | 0.1724 | 0.1724 | 0.0000 | 0.0000 | 0.3607 | 1.0000 | 0.0000 | 0.1639 | 0.3607 | 0.4310 | 0.1379 | 0.4310 | 0.1308 | 0.8067 |
| 0.0870 | 0.0000 | 3.1957 | 0.4391 | 0.0435 | 0.0870 | 0.6087 | 0.0000 | 0.0000 | 0.0000 | 1.0000 | 0.0000 | 0.0000 | 0.0000 | 0.4710 | 0.0580 | 0.4710 | 0.0870 | 0.5191 |
| 0.0440 | 0.0000 | 3.2747 | 0.4549 | 0.4615 | 0.0549 | 0.4396 | 0.0000 | 0.0000 | 0.2137 | 1.0000 | 0.0000 | 0.0916 | 0.2137 | 0.4139 | 0.1722 | 0.4139 | 0.0885 | 0.7246 |
| 0.1017 | 0.0000 | 2.8814 | 0.3763 | 0.3220 | 0.1017 | 0.3390 | 0.0000 | 0.0000 | 0.3368 | 1.0000 | 0.0000 | 0.0421 | 0.3368 | 0.4294 | 0.1412 | 0.4294 | 0.1085 | 0.6663 |
| 0.0460 | 0.0000 | 2.0920 | 0.2184 | 0.3333 | 0.0460 | 0.1379 | 0.0000 | 0.0000 | 0.1060 | 1.0000 | 0.0000 | 0.3179 | 0.1060 | 0.4387 | 0.1226 | 0.4387 | 0.1227 | 0.7547 |
| 0.0000 | 0.0000 | 2.2326 | 0.2465 | 0.2093 | 0.0000 | 0.3488 | 0.0000 | 0.0000 | 0.4675 | 1.0000 | 0.0000 | 0.0237 | 0.4675 | 0.4767 | 0.0465 | 0.4767 | 0.1259 | 0.6937 |

| | | | | | | | | | | | | | | | | | | |
|---|---|---|---|---|---|---|---|---|---|---|---|---|---|---|---|---|---|---|
| 0.2623 | 0.0000 | 3.0820 | 0.4164 | 0.3279 | 0.2787 | 0.2295 | 0.0000 | 0.0000 | 0.2673 | 1.0000 | 0.0000 | 0.1287 | 0.2673 | 0.4098 | 0.1803 | 0.4098 | 0.0884 | 0.7728 |
| 0.0267 | 0.0533 | 3.3919 | 0.4784 | 0.2800 | 0.0533 | 0.5333 | 0.0000 | 0.0000 | 0.1979 | 0.9867 | 0.0000 | 0.0208 | 0.1979 | 0.4459 | 0.1081 | 0.4459 | 0.0824 | 0.8398 |
| 0.0283 | 0.0755 | 3.2857 | 0.4571 | 0.2736 | 0.1038 | 0.4717 | 0.0000 | 0.0000 | 0.2245 | 0.9906 | 0.0000 | 0.0544 | 0.2245 | 0.4508 | 0.1079 | 0.4413 | 0.0899 | 0.4884 |
| 0.0000 | 0.0000 | 2.6860 | 0.3372 | 0.3934 | 0.0164 | 0.2787 | 0.0082 | 0.0000 | 0.1782 | 0.9918 | 0.0000 | 0.1149 | 0.1839 | 0.4270 | 0.1377 | 0.4353 | 0.1021 | 0.6778 |
| 0.0000 | 0.0000 | 3.6667 | 0.5333 | 0.0000 | 0.0000 | 0.9855 | 0.0000 | 0.0000 | 0.0496 | 1.0000 | 0.0000 | 0.3802 | 0.0496 | 0.5556 | 0.0048 | 0.4396 | 0.0290 | 0.1736 |
| 0.0000 | 0.0833 | 3.4167 | 0.4833 | 0.0000 | 0.0000 | 0.9167 | 0.0000 | 0.0000 | 0.0000 | 1.0000 | 0.0000 | 0.6279 | 0.0000 | 0.5833 | 0.0000 | 0.4167 | 0.0598 | 0.2834 |
| 0.0105 | 0.0000 | 3.8316 | 0.5663 | 0.0000 | 0.0316 | 0.9474 | 0.0000 | 0.0000 | 0.1500 | 1.0000 | 0.0000 | 0.0583 | 0.1500 | 0.5018 | 0.0175 | 0.4807 | 0.0402 | 0.1968 |
| 0.0208 | 0.0417 | 3.7917 | 0.5583 | 0.0208 | 0.0417 | 0.9167 | 0.0000 | 0.0000 | 0.1864 | 1.0000 | 0.0000 | 0.0000 | 0.1864 | 0.4896 | 0.0208 | 0.4896 | 0.0437 | 0.7467 |
| 0.0519 | 0.0000 | 3.2727 | 0.4545 | 0.4329 | 0.0519 | 0.5022 | 0.0000 | 0.0000 | 0.0556 | 1.0000 | 0.0000 | 0.1424 | 0.0556 | 0.4278 | 0.1616 | 0.4105 | 0.0944 | 0.5322 |
| 0.1067 | 0.0000 | 3.0267 | 0.4053 | 0.1733 | 0.1067 | 0.5333 | 0.0000 | 0.0000 | 0.1477 | 1.0000 | 0.0000 | 0.0000 | 0.1477 | 0.4533 | 0.0933 | 0.4533 | 0.1087 | 0.5477 |
| 0.1290 | 0.0000 | 2.8387 | 0.3677 | 0.1720 | 0.1398 | 0.3871 | 0.0430 | 0.0000 | 0.2314 | 1.0000 | 0.0000 | 0.0000 | 0.2314 | 0.4265 | 0.1039 | 0.4695 | 0.1139 | 0.7159 |
| 0.0455 | 0.0000 | 3.2500 | 0.4500 | 0.5682 | 0.0455 | 0.3636 | 0.0114 | 0.0000 | 0.0612 | 1.0000 | 0.0000 | 0.0408 | 0.0612 | 0.3958 | 0.1970 | 0.4072 | 0.0867 | 0.5747 |
| 0.0847 | 0.0000 | 2.9138 | 0.3828 | 0.6102 | 0.0847 | 0.2034 | 0.0169 | 0.0000 | 0.3163 | 0.9831 | 0.0000 | 0.0816 | 0.3163 | 0.3736 | 0.2529 | 0.3736 | 0.0966 | 0.6983 |
| 0.0172 | 0.0690 | 2.7115 | 0.3423 | 0.4655 | 0.0345 | 0.2414 | 0.0000 | 0.0000 | 0.3815 | 0.8966 | 0.0000 | 0.2659 | 0.3988 | 0.4103 | 0.1795 | 0.4103 | 0.1076 | 0.7891 |
| 0.0455 | 0.1818 | 2.3793 | 0.2759 | 0.1477 | 0.0568 | 0.1818 | 0.0000 | 0.0000 | 0.1102 | 0.9886 | 0.0000 | 0.1441 | 0.1102 | 0.4904 | 0.0421 | 0.4674 | 0.1099 | 0.7459 |
| 0.1017 | 0.0000 | 3.1034 | 0.4207 | 0.4068 | 0.1017 | 0.4068 | 0.0678 | 0.0000 | 0.1688 | 0.9831 | 0.0000 | 0.0519 | 0.1818 | 0.3793 | 0.1724 | 0.4483 | 0.0793 | 0.7066 |
| 0.1509 | 0.0000 | 2.9245 | 0.3849 | 0.4528 | 0.1509 | 0.2453 | 0.0000 | 0.0000 | 0.0469 | 1.0000 | 0.0000 | 0.1250 | 0.0469 | 0.4119 | 0.1761 | 0.4119 | 0.0829 | 0.5776 |
| 0.0392 | 0.0784 | 2.9608 | 0.3922 | 0.1176 | 0.0392 | 0.4706 | 0.0196 | 0.0000 | 0.0877 | 1.0000 | 0.0000 | 0.0175 | 0.0877 | 0.4641 | 0.0523 | 0.4837 | 0.0937 | 0.4883 |
| 0.0556 | 0.0000 | 3.8000 | 0.5600 | 0.1111 | 0.0556 | 0.7778 | 0.0000 | 0.0000 | 0.0909 | 0.9722 | 0.0000 | 0.1818 | 0.0909 | 0.4714 | 0.0571 | 0.4714 | 0.0536 | 0.8991 |
| 0.0417 | 0.0083 | 3.7917 | 0.5583 | 0.0083 | 0.0500 | 0.9000 | 0.0000 | 0.0000 | 0.1304 | 1.0000 | 0.0000 | 0.0000 | 0.1304 | 0.4958 | 0.0167 | 0.4875 | 0.0407 | 0.2533 |
| 0.1538 | 0.0000 | 3.0385 | 0.4077 | 0.0000 | 0.1538 | 0.5769 | 0.0000 | 0.0000 | 0.0625 | 1.0000 | 0.0000 | 0.1250 | 0.0625 | 0.4936 | 0.0513 | 0.4551 | 0.1089 | 0.5672 |
| 0.0411 | 0.0000 | 3.8356 | 0.5671 | 0.0274 | 0.0411 | 0.9315 | 0.0000 | 0.0000 | 0.0581 | 1.0000 | 0.0000 | 0.0000 | 0.0581 | 0.4886 | 0.0228 | 0.4886 | 0.0442 | 0.4338 |
| 0.1277 | 0.0106 | 3.9468 | 0.5894 | 0.0000 | 0.1277 | 0.8511 | 0.0000 | 0.0000 | 0.1193 | 1.0000 | 0.0000 | 0.0183 | 0.1193 | 0.4787 | 0.0426 | 0.4787 | 0.0424 | 0.9914 |
| 0.1111 | 0.0741 | 3.3333 | 0.4667 | 0.0000 | 0.1111 | 0.7037 | 0.0000 | 0.0000 | 0.0092 | 1.0000 | 0.0000 | 0.0000 | 0.0092 | 0.5000 | 0.0370 | 0.4630 | 0.0970 | 0.4135 |
| 0.0541 | 0.0135 | 3.5811 | 0.5162 | 0.1081 | 0.0541 | 0.8108 | 0.0000 | 0.0000 | 0.2276 | 1.0000 | 0.0000 | 0.1707 | 0.2276 | 0.4730 | 0.0541 | 0.4730 | 0.0736 | 0.5251 |
| 0.0877 | 0.0000 | 3.4211 | 0.4842 | 0.2982 | 0.0877 | 0.5614 | 0.0175 | 0.0000 | 0.2400 | 1.0000 | 0.0000 | 0.0000 | 0.2400 | 0.4415 | 0.1170 | 0.4415 | 0.0785 | 0.8048 |
| 0.0541 | 0.3243 | 3.0811 | 0.4162 | 0.2703 | 0.0541 | 0.2703 | 0.0541 | 0.0000 | 0.2000 | 1.0000 | 0.0000 | 0.0000 | 0.2211 | 0.4234 | 0.0991 | 0.4775 | 0.0878 | 0.8413 |
| 0.1739 | 0.0000 | 2.9348 | 0.3870 | 0.1957 | 0.1739 | 0.2609 | 0.0000 | 0.0000 | 0.0597 | 1.0000 | 0.0000 | 0.5970 | 0.0597 | 0.4420 | 0.1159 | 0.4420 | 0.0847 | 0.8681 |
| 0.0674 | 0.0000 | 3.5227 | 0.5045 | 0.1124 | 0.0674 | 0.7191 | 0.0112 | 0.0000 | 0.0421 | 0.9888 | 0.0000 | 0.0211 | 0.0421 | 0.4716 | 0.0455 | 0.4830 | 0.0614 | 0.7560 |
| 0.0090 | 0.0000 | 2.9910 | 0.3982 | 0.0180 | 0.0090 | 0.6486 | 0.0000 | 0.0000 | 0.0169 | 1.0000 | 0.0000 | 0.0424 | 0.0169 | 0.4835 | 0.0330 | 0.4835 | 0.0886 | 0.5032 |
| 0.0234 | 0.0234 | 1.6901 | 0.1380 | 0.1170 | 0.0234 | 0.1170 | 0.0058 | 0.0000 | 0.0165 | 1.0000 | 0.0000 | 0.0440 | 0.0165 | 0.4756 | 0.0429 | 0.4815 | 0.0949 | 0.9092 |
| 0.2105 | 0.0421 | 3.7368 | 0.5474 | 0.0632 | 0.2105 | 0.6316 | 0.0105 | 0.0000 | 0.0495 | 1.0000 | 0.0000 | 0.0000 | 0.0594 | 0.4526 | 0.0842 | 0.4632 | 0.0733 | 0.9521 |
| 0.2424 | 0.0606 | 3.0606 | 0.4121 | 0.2929 | 0.2424 | 0.1616 | 0.0101 | 0.0000 | 0.2889 | 1.0000 | 0.0000 | 0.1556 | 0.2944 | 0.3822 | 0.2256 | 0.3923 | 0.1127 | 0.8085 |
| 0.0567 | 0.1489 | 2.0496 | 0.2099 | 0.0355 | 0.0567 | 0.1702 | 0.0071 | 0.0000 | 0.0497 | 1.0000 | 0.0000 | 0.0745 | 0.0497 | 0.4811 | 0.0307 | 0.4882 | 0.1086 | 0.8024 |
| 0.2581 | 0.0000 | 3.5376 | 0.5075 | 0.0323 | 0.2581 | 0.6022 | 0.0215 | 0.0000 | 0.1468 | 1.0000 | 0.0000 | 0.0000 | 0.1468 | 0.4409 | 0.0968 | 0.4624 | 0.0946 | 0.4681 |
| 0.0169 | 0.0000 | 3.5932 | 0.5186 | 0.1695 | 0.0169 | 0.7458 | 0.0678 | 0.0000 | 0.1184 | 1.0000 | 0.0000 | 0.1053 | 0.1184 | 0.4350 | 0.0621 | 0.5028 | 0.0531 | 0.8510 |
| 0.0370 | 0.0000 | 3.5926 | 0.5185 | 0.0000 | 0.0370 | 0.9259 | 0.0370 | 0.0000 | 0.1429 | 1.0000 | 0.0000 | 0.0000 | 0.1429 | 0.4938 | 0.0123 | 0.4938 | 0.0709 | 0.5551 |
| 0.0222 | 0.0222 | 3.0000 | 0.4000 | 0.4444 | 0.0222 | 0.4444 | 0.0000 | 0.0000 | 0.0526 | 1.0000 | 0.0000 | 0.0000 | 0.0526 | 0.4148 | 0.1704 | 0.4148 | 0.1327 | 0.6926 |
| 0.0000 | 0.0000 | 2.8696 | 0.3739 | 0.6522 | 0.0000 | 0.1739 | 0.0000 | 0.0000 | 0.1538 | 1.0000 | 0.0000 | 0.1538 | 0.2564 | 0.3841 | 0.2319 | 0.3841 | 0.0891 | 0.7613 |
| 0.0417 | 0.0000 | 3.2292 | 0.4458 | 0.3750 | 0.0417 | 0.4792 | 0.0833 | 0.0000 | 0.2126 | 1.0000 | 0.0000 | 0.0000 | 0.2441 | 0.3889 | 0.1389 | 0.4722 | 0.0928 | 0.6192 |
| 0.0204 | 0.0000 | 2.4694 | 0.2939 | 0.0816 | 0.0204 | 0.4082 | 0.0408 | 0.0000 | 0.0317 | 1.0000 | 0.0000 | 0.1905 | 0.0317 | 0.4286 | 0.1020 | 0.4694 | 0.0923 | 0.8293 |
| 0.0000 | 0.0769 | 2.3600 | 0.2720 | 0.0769 | 0.0000 | 0.3462 | 0.0000 | 0.0000 | 0.2346 | 0.9615 | 0.0000 | 0.1235 | 0.2346 | 0.4800 | 0.0400 | 0.4800 | 0.0867 | 0.9388 |
| 0.0000 | 0.0000 | 3.4384 | 0.4877 | 0.3014 | 0.0000 | 0.6027 | 0.0274 | 0.0000 | 0.3866 | 1.0000 | 0.0000 | 0.0000 | 0.3866 | 0.4361 | 0.1005 | 0.4635 | 0.0783 | 0.9115 |
| 0.1935 | 0.0000 | 3.5161 | 0.5032 | 0.2903 | 0.1935 | 0.4516 | 0.0323 | 0.0000 | 0.1368 | 1.0000 | 0.0000 | 0.2105 | 0.1368 | 0.4032 | 0.1613 | 0.4355 | 0.0854 | 0.7808 |
| 0.0253 | 0.0000 | 2.8974 | 0.3795 | 0.1392 | 0.0253 | 0.5063 | 0.0000 | 0.0000 | 0.0385 | 0.9873 | 0.0000 | 0.0934 | 0.0385 | 0.4722 | 0.0556 | 0.4722 | 0.1052 | 0.6337 |
| 0.0909 | 0.0455 | 3.0227 | 0.4045 | 0.7045 | 0.0909 | 0.0909 | 0.0227 | 0.0000 | 0.2066 | 1.0000 | 0.0000 | 0.0661 | 0.2066 | 0.3561 | 0.2652 | 0.3788 | 0.0574 | 0.6034 |
| 0.1304 | 0.0000 | 3.4565 | 0.4913 | 0.3696 | 0.1304 | 0.4348 | 0.0217 | 0.0000 | 0.0938 | 1.0000 | 0.0000 | 0.1875 | 0.0938 | 0.4058 | 0.1667 | 0.4275 | 0.0861 | 0.7220 |
| 0.0286 | 0.0286 | 3.0571 | 0.4114 | 0.4286 | 0.0286 | 0.3429 | 0.0286 | 0.0000 | 0.1919 | 1.0000 | 0.0000 | 0.1010 | 0.1919 | 0.4143 | 0.1429 | 0.4429 | 0.0851 | 0.7241 |
| 0.0000 | 0.0000 | 2.9714 | 0.3943 | 0.5429 | 0.0000 | 0.3429 | 0.0286 | 0.0000 | 0.2500 | 1.0000 | 0.0000 | 0.0208 | 0.2500 | 0.4000 | 0.1714 | 0.4286 | 0.0929 | 0.6984 |
| 0.2500 | 0.0000 | 4.0000 | 0.6000 | 0.0000 | 0.2500 | 0.7500 | 0.0000 | 0.0000 | 0.2308 | 1.0000 | 0.0000 | 0.0000 | 0.2308 | 0.4583 | 0.0833 | 0.4583 | 0.0615 | NA |
| 0.0761 | 0.0102 | 2.9848 | 0.3970 | 0.0000 | 0.0761 | 0.9239 | 0.0000 | 0.0000 | 0.0000 | 1.0000 | 0.0000 | 0.0483 | 0.0000 | 0.6887 | 0.0288 | 0.2826 | 0.0750 | 0.5861 |

| | | | | | | | | | | | | | | | | | | |
|---|---|---|---|---|---|---|---|---|---|---|---|---|---|---|---|---|---|---|
| 0.0274 | 0.0000 | 3.9726 | 0.5945 | 0.0000 | 0.0274 | 0.9726 | 0.0000 | 0.0000 | 0.0027 | 1.0000 | 0.0000 | 0.0000 | 0.0027 | 0.5023 | 0.0091 | 0.4886 | 0.0095 | 0.5844 |
| 0.0404 | 0.0000 | 3.9704 | 0.5941 | 0.0000 | 0.0404 | 0.9596 | 0.0000 | 0.0000 | 0.0067 | 1.0000 | 0.0000 | 0.0000 | 0.0067 | 0.5000 | 0.0135 | 0.4865 | 0.0137 | 0.2103 |
| 0.1684 | 0.0000 | 3.7845 | 0.5569 | 0.0034 | 0.1684 | 0.7576 | 0.0034 | 0.0000 | 0.0690 | 1.0000 | 0.0000 | 0.0000 | 0.0690 | 0.5034 | 0.0404 | 0.4562 | 0.0500 | 0.3122 |
| 0.2500 | 0.0000 | 4.0000 | 0.6000 | 0.0000 | 0.2500 | 0.7500 | 0.0000 | 0.0000 | 0.0000 | 1.0000 | 0.0000 | 0.0000 | 0.0000 | 0.4583 | 0.0833 | 0.4583 | 0.0615 | NA |
| 0.0741 | 0.0000 | 4.0000 | 0.6000 | 0.0000 | 0.0741 | 0.9259 | 0.0000 | 0.0000 | 0.0383 | 1.0000 | 0.0000 | 0.0958 | 0.0415 | 0.4877 | 0.0247 | 0.4877 | 0.0225 | NA |
| 0.0000 | 0.4444 | 3.1111 | 0.4222 | 0.0000 | 0.0000 | 0.5556 | 0.0000 | 0.0000 | 0.0110 | 1.0000 | 0.0000 | 0.0000 | 0.0110 | 0.5000 | 0.0000 | 0.5000 | 0.1221 | NA |
| 0.0366 | 0.0000 | 3.9512 | 0.5902 | 0.0000 | 0.0366 | 0.9634 | 0.0000 | 0.0000 | 0.0024 | 1.0000 | 0.0000 | 0.0238 | 0.0024 | 0.5102 | 0.0041 | 0.4858 | 0.0201 | 0.1760 |
| 0.0357 | 0.0000 | 3.8571 | 0.5714 | 0.0000 | 0.0357 | 0.9643 | 0.0000 | 0.0000 | 0.0000 | 1.0000 | 0.0000 | 0.0000 | 0.0000 | 0.5298 | 0.0119 | 0.4583 | 0.0149 | 0.5324 |
| 0.4605 | 0.0132 | 3.1184 | 0.4237 | 0.0000 | 0.4605 | 0.5263 | 0.0000 | 0.0000 | 0.0787 | 1.0000 | 0.0000 | 0.0562 | 0.0899 | 0.6206 | 0.1535 | 0.2259 | 0.1026 | 0.7425 |
| 0.1661 | 0.1329 | 3.6279 | 0.5256 | 0.0000 | 0.1993 | 0.6645 | 0.0000 | 0.0000 | 0.0259 | 1.0000 | 0.0000 | 0.0000 | 0.0259 | 0.4557 | 0.0554 | 0.4889 | 0.0925 | 0.8933 |
| 0.0698 | 0.0000 | 4.0000 | 0.6000 | 0.0000 | 0.0698 | 0.9302 | 0.0000 | 0.0000 | 0.0046 | 1.0000 | 0.0000 | 0.0000 | 0.0046 | 0.4884 | 0.0233 | 0.4884 | 0.0213 | NA |
| 0.1103 | 0.0120 | 3.8153 | 0.5631 | 0.0024 | 0.1103 | 0.8753 | 0.0000 | 0.0000 | 0.0047 | 1.0000 | 0.0000 | 0.0233 | 0.0047 | 0.4832 | 0.0360 | 0.4808 | 0.0509 | 0.6057 |
| 0.1332 | 0.0000 | 3.9034 | 0.5807 | 0.0000 | 0.1332 | 0.8616 | 0.0026 | 0.0000 | 0.0077 | 1.0000 | 0.0000 | 0.0128 | 0.0077 | 0.4939 | 0.0357 | 0.4704 | 0.0447 | 0.3560 |
| 0.0435 | 0.1739 | 3.4783 | 0.4957 | 0.0000 | 0.2174 | 0.7826 | 0.0000 | 0.0000 | 0.0000 | 1.0000 | 0.0000 | 0.0000 | 0.0000 | 0.5181 | 0.0507 | 0.4312 | 0.1076 | 0.6283 |
| 0.0000 | 0.4848 | 2.6364 | 0.3273 | 0.0000 | 0.5758 | 0.4242 | 0.0000 | 0.0000 | 0.0294 | 1.0000 | 0.0000 | 0.0000 | 0.0294 | 0.3737 | 0.1616 | 0.4646 | 0.1450 | 0.7272 |
| 0.2485 | 0.2339 | 2.9942 | 0.3988 | 0.1959 | 0.3070 | 0.2047 | 0.0292 | 0.0000 | 0.0058 | 1.0000 | 0.0000 | 0.0058 | 0.0058 | 0.4464 | 0.1657 | 0.3879 | 0.0976 | 0.7880 |
| 0.1010 | 0.0253 | 3.6793 | 0.5359 | 0.0278 | 0.1010 | 0.7955 | 0.0253 | 0.0000 | 0.0025 | 1.0000 | 0.0000 | 0.0124 | 0.0025 | 0.4912 | 0.0303 | 0.4785 | 0.0431 | 0.2511 |
| 0.0733 | 0.0000 | 3.8592 | 0.5718 | 0.0000 | 0.0733 | 0.8798 | 0.0029 | 0.0000 | 0.0055 | 1.0000 | 0.0000 | 0.0551 | 0.0055 | 0.4863 | 0.0244 | 0.4892 | 0.0327 | 0.9730 |
| 0.7273 | 0.0000 | 2.4000 | 0.2800 | 0.0000 | 0.7273 | 0.1818 | 0.0000 | 0.0000 | 0.1951 | 0.1818 | 0.7273 | 0.5366 | 0.1951 | 0.5000 | 0.4000 | 0.1000 | 0.0847 | NA |
| 0.1250 | 0.0000 | 3.6667 | 0.5333 | 0.0000 | 0.1250 | 0.6250 | 0.0000 | 0.0000 | 0.1778 | 0.6250 | 0.1250 | 0.0889 | 0.2000 | 0.5000 | 0.0833 | 0.4167 | 0.0735 | NA |
| 0.0000 | 0.2000 | 3.6000 | 0.5200 | 0.0000 | 0.0000 | 0.8000 | 0.0000 | 0.0000 | 0.0000 | 1.0000 | 0.0000 | 0.8039 | 0.0000 | 0.5000 | 0.0000 | 0.5000 | 0.0791 | NA |
| 0.0370 | 0.0741 | 2.2963 | 0.2593 | 0.2963 | 0.0370 | 0.5926 | 0.0000 | 0.0000 | 0.2373 | 0.9630 | 0.0370 | 0.3051 | 0.2373 | 0.5000 | 0.0185 | 0.4815 | 0.1148 | 0.8789 |
| 0.0000 | 0.0112 | 3.8391 | 0.5678 | 0.0000 | 0.0449 | 0.9213 | 0.0000 | 0.0000 | 0.1604 | 0.9775 | 0.0000 | 0.0000 | 0.1604 | 0.4770 | 0.0000 | 0.5230 | 0.0268 | 0.9899 |
| 0.0000 | 0.0000 | 1.4000 | 0.0800 | 0.0000 | 0.0000 | 0.1667 | 0.0000 | 0.0000 | 0.1452 | 0.8333 | 0.0000 | 0.7419 | 0.1613 | 0.1000 | 0.0000 | 0.9000 | 0.0826 | NA |
| 0.0000 | 0.0000 | 3.8571 | 0.5714 | 0.0000 | 0.0000 | 1.0000 | 0.0000 | 0.0000 | 0.2192 | 1.0000 | 0.0000 | 0.0000 | 0.2329 | 0.5000 | 0.0000 | 0.5000 | 0.0460 | NA |
| 0.5000 | 0.0000 | 2.0000 | 0.2000 | 0.0000 | 0.5000 | 0.0000 | 0.0000 | 0.0000 | 0.9524 | 0.0000 | 0.5000 | 0.0238 | 0.9524 | 0.5000 | 0.5000 | 0.0000 | 0.0000 | NA |
| 0.0081 | 0.0163 | 3.9675 | 0.5935 | 0.0000 | 0.0081 | 0.9756 | 0.0000 | 0.0000 | 0.0000 | 1.0000 | 0.0000 | 0.0081 | 0.0000 | 0.4986 | 0.0027 | 0.4986 | 0.0101 | 0.9919 |
| 0.0000 | 0.0000 | 4.0000 | 0.6000 | 0.0000 | 0.0000 | 0.9901 | 0.0000 | 0.0000 | 0.0190 | 0.9901 | 0.0000 | 0.0190 | 0.0190 | 0.5000 | 0.0000 | 0.5000 | 0.0000 | NA |
| 0.0000 | 0.0000 | 4.0000 | 0.6000 | 0.0000 | 0.0000 | 1.0000 | 0.0000 | 0.0000 | 0.0536 | 1.0000 | 0.0000 | 0.0089 | 0.0625 | 0.5000 | 0.0000 | 0.5000 | 0.0000 | NA |
| 0.0385 | 0.0385 | 3.5000 | 0.5000 | 0.0769 | 0.0385 | 0.8462 | 0.0000 | 0.0000 | 0.2143 | 1.0000 | 0.0000 | 0.0357 | 0.2214 | 0.5513 | 0.0128 | 0.4359 | 0.0535 | 0.9600 |
| 0.0000 | 1.0000 | 2.0000 | 0.2000 | 0.0000 | 0.0000 | 0.0000 | 0.0000 | 0.0000 | 0.5385 | 1.0000 | 0.0000 | 0.3077 | 0.5385 | 0.5000 | 0.0000 | 0.5000 | 0.0000 | NA |
| 0.0000 | 0.4000 | 3.1111 | 0.4222 | 0.0000 | 0.0000 | 0.5000 | 0.0000 | 0.0000 | 0.0741 | 0.9000 | 0.0000 | 0.1852 | 0.0741 | 0.5000 | 0.0000 | 0.5000 | 0.1221 | NA |
| 0.0333 | 0.0000 | 3.5172 | 0.5034 | 0.0000 | 0.0333 | 0.8667 | 0.0000 | 0.0000 | 0.2881 | 0.9667 | 0.0000 | 0.2034 | 0.2881 | 0.4828 | 0.0000 | 0.5172 | 0.0823 | 0.4979 |
| 0.0000 | 0.0000 | 1.9200 | 0.1840 | 0.0000 | 0.0000 | 0.9200 | 0.0400 | 0.0000 | 0.1062 | 1.0000 | 0.0000 | 0.0088 | 0.1062 | 0.9500 | 0.0000 | 0.0500 | 0.0138 | 0.8504 |
| 0.0000 | 0.0000 | 3.9726 | 0.5945 | 0.0000 | 0.0000 | 1.0000 | 0.0000 | 0.0000 | 0.0644 | 1.0000 | 0.0000 | 0.0337 | 0.0706 | 0.5068 | 0.0000 | 0.4932 | 0.0015 | NA |
| 0.0062 | 0.0000 | 4.0000 | 0.6000 | 0.0000 | 0.0062 | 0.9938 | 0.0000 | 0.0000 | 0.0000 | 1.0000 | 0.0000 | 0.0062 | 0.0000 | 0.4990 | 0.0021 | 0.4990 | 0.0020 | NA |
| 0.0000 | 0.0000 | 4.0000 | 0.6000 | 0.0000 | 0.0000 | 1.0000 | 0.0000 | 0.0000 | 0.0000 | 1.0000 | 0.0000 | 0.0000 | 0.0000 | 0.5000 | 0.0000 | 0.5000 | 0.0000 | NA |
| 0.3750 | 1.0000 | 2.0000 | 0.2000 | 0.0000 | 0.8750 | 0.0000 | 0.0000 | 0.0000 | 0.0164 | 1.0000 | 0.0000 | 0.3279 | 0.0164 | 0.3542 | 0.2917 | 0.3542 | 0.1038 | 0.8681 |
| 0.0000 | 0.2000 | 3.6000 | 0.5200 | 0.0000 | 0.0000 | 0.8000 | 0.0000 | 0.0000 | 0.1389 | 1.0000 | 0.0000 | 0.1667 | 0.1389 | 0.5000 | 0.0000 | 0.5000 | 0.0791 | NA |
| 0.0000 | 1.0000 | 2.0000 | 0.2000 | 0.0000 | 1.0000 | 0.0000 | 0.0000 | 0.0000 | 0.0435 | 1.0000 | 0.0000 | 0.3043 | 0.0435 | 0.3333 | 0.3333 | 0.3333 | 0.0000 | NA |
| 0.0000 | 1.0000 | 2.0000 | 0.2000 | 0.0000 | 0.0000 | 0.0000 | 0.0000 | 0.0000 | 0.5714 | 1.0000 | 0.0000 | 0.0000 | 0.5714 | 0.5000 | 0.0000 | 0.5000 | 0.0000 | NA |
| 0.0000 | 0.2381 | 2.8095 | 0.3619 | 0.0000 | 0.0000 | 0.7619 | 0.0000 | 0.0000 | 0.1186 | 1.0000 | 0.0000 | 0.0000 | 0.2881 | 0.5000 | 0.0000 | 0.5000 | 0.1253 | 0.9445 |
| 0.0000 | 1.0000 | 2.0000 | 0.2000 | 0.0000 | 0.0000 | 0.0000 | 0.0000 | 0.0000 | 0.5833 | 1.0000 | 0.0000 | 0.0000 | 0.5833 | 0.5000 | 0.0000 | 0.5000 | 0.0000 | NA |
| 0.0000 | 0.0000 | 3.0000 | 0.4000 | 0.0000 | 0.0000 | 0.9375 | 0.0000 | 0.0000 | 0.1111 | 0.9375 | 0.0000 | 0.0000 | 0.1111 | 0.5000 | 0.0000 | 0.5000 | 0.0000 | NA |
| 0.1923 | 0.0000 | 2.7308 | 0.3462 | 0.0000 | 0.0385 | 0.9615 | 0.0000 | 0.0000 | 0.1563 | 1.0000 | 0.0000 | 0.0313 | 0.1563 | 0.4808 | 0.0000 | 0.5192 | 0.1425 | 0.8790 |
| 0.0000 | 0.0000 | 3.0000 | 0.4000 | 0.0000 | 0.0000 | 1.0000 | 0.0000 | 0.0000 | 0.5109 | 1.0000 | 0.0000 | 0.3804 | 0.5652 | 0.5000 | 0.0000 | 0.5000 | 0.0000 | NA |
| 0.0116 | 0.4070 | 2.2907 | 0.2581 | 0.0000 | 0.0581 | 0.5349 | 0.0000 | 0.0000 | 0.0169 | 1.0000 | 0.0000 | 0.1695 | 0.1017 | 0.6453 | 0.0000 | 0.3547 | 0.1316 | 0.9387 |
| 0.5833 | 0.4444 | 2.5833 | 0.3167 | 0.0000 | 0.5278 | 0.3333 | 0.0000 | 0.0000 | 0.0217 | 1.0000 | 0.0000 | 0.0000 | 0.0217 | 0.5185 | 0.1574 | 0.3241 | 0.1191 | 0.5641 |
| 0.0000 | 0.2857 | 3.8352 | 0.5670 | 0.0000 | 0.2747 | 0.7253 | 0.0000 | 0.0000 | 0.1302 | 1.0000 | 0.0000 | 0.3254 | 0.1361 | 0.4524 | 0.0952 | 0.4524 | 0.0672 | 0.6109 |
| 0.0000 | 0.6667 | 4.0000 | 0.6000 | 0.0000 | 0.0000 | 1.0000 | 0.0000 | 0.0000 | 0.0625 | 1.0000 | 0.0000 | 0.0000 | 0.0625 | 0.3889 | 0.2222 | 0.3889 | 0.0735 | NA |
| 0.0000 | 0.0000 | 3.0385 | 0.4077 | 0.0000 | 0.0000 | 1.0000 | 0.0000 | 0.0000 | 0.1905 | 1.0000 | 0.0000 | 0.0060 | 0.2202 | 0.5000 | 0.0000 | 0.5000 | 0.0139 | NA |

| | | | | | | | | | | | | | | | | | | |
|---|---|---|---|---|---|---|---|---|---|---|---|---|---|---|---|---|---|---|
| 0.1500 | 0.0000 | 3.4500 | 0.4900 | 0.0000 | 0.0000 | 1.0000 | 0.0000 | 0.0000 | 0.2697 | 1.0000 | 0.0000 | 0.0000 | 0.4382 | 0.5000 | 0.0000 | 0.5000 | 0.0843 | 0.3921 |
| 0.0769 | 0.1538 | 2.7692 | 0.3538 | 0.0000 | 0.0000 | 0.8462 | 0.0000 | 0.0000 | 0.0909 | 1.0000 | 0.0000 | 0.1705 | 0.0909 | 0.5000 | 0.0000 | 0.5000 | 0.1245 | 0.9312 |
| 0.6667 | 0.3333 | 2.6667 | 0.3333 | 0.0000 | 0.3333 | 0.3333 | 0.0000 | 0.0000 | 0.5000 | 1.0000 | 0.0000 | 0.0000 | 0.5000 | 0.4444 | 0.1111 | 0.4444 | 0.0878 | 0.8754 |
| NA | NA | NA | NA | NA | NA | NA | NA | 0.0000 | 0.9444 | NA | NA | 0.0000 | 1.0000 | NA | NA | NA | NA | NA |
| 0.2991 | 0.0427 | 2.1121 | 0.2224 | 0.0000 | 0.5983 | 0.2735 | 0.0855 | 0.0000 | 0.0000 | 0.7436 | 0.2564 | 0.2548 | 0.0000 | 0.3148 | 0.1567 | 0.5285 | 0.1410 | 0.7923 |
| 0.0000 | 0.5299 | 2.9167 | 0.3833 | 0.0075 | 0.0000 | 0.9701 | 0.0149 | 0.0024 | 0.0520 | 1.0000 | 0.0000 | 0.3121 | 0.0544 | 0.3759 | 0.2482 | 0.3759 | 0.0726 | 0.9203 |
| 0.0000 | 0.7903 | 3.7622 | 0.5524 | 0.0243 | 0.0000 | 0.9726 | 0.0030 | 0.0000 | 0.0120 | 1.0000 | 0.0000 | 0.0000 | 0.0120 | 0.3642 | 0.2715 | 0.3642 | 0.0784 | 0.9504 |
| 0.0000 | 0.4145 | 2.6667 | 0.3333 | 0.1036 | 0.1036 | 0.7668 | 0.0052 | 0.0000 | 0.0551 | 1.0000 | 0.0000 | 0.2353 | 0.0551 | 0.4521 | 0.1743 | 0.3736 | 0.0935 | 0.8351 |
| 0.0000 | 0.3438 | 2.3353 | 0.2671 | 0.0344 | 0.0458 | 0.4585 | 0.4613 | 0.0479 | 0.0309 | 1.0000 | 0.0000 | 0.2433 | 0.0827 | 0.4379 | 0.1184 | 0.4436 | 0.0954 | 0.9638 |
| 0.0000 | 0.0075 | 2.3130 | 0.2626 | 0.3383 | 0.0075 | 0.4586 | 0.1654 | 0.5341 | 0.2404 | 1.0000 | 0.0000 | 0.0282 | 0.7745 | 0.4561 | 0.1178 | 0.4261 | 0.1345 | 0.7431 |
| 0.0000 | 0.2712 | 1.9661 | 0.1932 | 0.5763 | 0.0508 | 0.2712 | 0.1017 | 0.0047 | 0.0190 | 1.0000 | 0.0000 | 0.4171 | 0.0237 | 0.4605 | 0.1130 | 0.4266 | 0.1062 | 0.7759 |
| 0.0000 | 0.0288 | 2.9701 | 0.3940 | 0.2158 | 0.0000 | 0.7194 | 0.0360 | 0.2795 | 0.4472 | 1.0000 | 0.0000 | 0.1956 | 0.7267 | 0.5000 | 0.0000 | 0.5000 | 0.1058 | 0.9342 |
| 0.0000 | 0.0000 | 3.0000 | 0.4000 | 0.4898 | 0.0000 | 0.4898 | 0.0204 | 0.4544 | 0.3647 | 1.0000 | 0.0000 | 0.1122 | 0.8191 | 0.5000 | 0.0000 | 0.5000 | 0.1380 | 0.9497 |
| 0.0000 | 0.2381 | 2.1905 | 0.2381 | 0.2857 | 0.0000 | 0.4762 | 0.0000 | 0.3789 | 0.3544 | 1.0000 | 0.0000 | 0.1193 | 0.7333 | 0.5000 | 0.0000 | 0.5000 | 0.1474 | 0.7737 |
| 0.0000 | 0.2222 | 1.8889 | 0.1778 | 0.4444 | 0.0000 | 0.3333 | 0.0000 | 0.5985 | 0.2336 | 1.0000 | 0.0000 | 0.0965 | 0.8340 | 0.5000 | 0.0000 | 0.5000 | 0.1471 | 0.6662 |
| 0.0000 | 0.0000 | 1.7297 | 0.1459 | 0.6486 | 0.0270 | 0.3243 | 0.0000 | 0.3942 | 0.2899 | 1.0000 | 0.0000 | 0.2087 | 0.6841 | 0.4865 | 0.0000 | 0.5135 | 0.0834 | 0.3948 |
| 0.1228 | 1.0000 | 2.1228 | 0.2246 | 0.0000 | 0.0000 | 0.0000 | 0.0000 | 0.2515 | 0.3758 | 1.0000 | 0.0000 | 0.3409 | 0.6273 | 0.5000 | 0.0000 | 0.5000 | 0.0446 | NA |
| 0.0000 | 0.0000 | 1.8667 | 0.1733 | 0.1935 | 0.0000 | 0.3226 | 0.0323 | 0.3649 | 0.2763 | 1.0000 | 0.0000 | 0.2658 | 0.6411 | 0.5000 | 0.0000 | 0.5000 | 0.1164 | 0.9229 |
| 0.0189 | 0.0000 | 2.0000 | 0.2000 | 0.0755 | 0.0189 | 0.4528 | 0.0000 | 0.5499 | 0.1457 | 1.0000 | 0.0000 | 0.1309 | 0.6956 | 0.4874 | 0.0063 | 0.5063 | 0.1313 | 0.7139 |
| 0.0000 | 0.0000 | 1.8919 | 0.1784 | 0.0263 | 0.0000 | 0.4211 | 0.0000 | 0.0079 | 0.6127 | 0.9737 | 0.0000 | 0.3175 | 0.6222 | 0.5000 | 0.0000 | 0.5000 | 0.1233 | 0.9268 |
| 0.0045 | 0.4706 | 2.5294 | 0.3059 | 0.4525 | 0.0226 | 0.5249 | 0.0000 | 0.0000 | 0.1289 | 1.0000 | 0.0000 | 0.0078 | 0.1289 | 0.3533 | 0.3072 | 0.3395 | 0.0748 | 0.8428 |
| 0.0052 | 0.6963 | 3.3194 | 0.4639 | 0.0576 | 0.0733 | 0.7644 | 0.0000 | 0.0067 | 0.2933 | 1.0000 | 0.0000 | 0.0633 | 0.3000 | 0.4630 | 0.2583 | 0.2788 | 0.0921 | 0.7769 |
| 0.0000 | 0.3714 | 2.3429 | 0.2686 | 0.4571 | 0.1429 | 0.4000 | 0.0000 | 0.2360 | 0.1854 | 1.0000 | 0.0000 | 0.1798 | 0.4270 | 0.3737 | 0.2828 | 0.3434 | 0.0990 | 0.8344 |
| 0.0000 | 0.2051 | 2.1282 | 0.2256 | 0.0769 | 0.0000 | 0.4103 | 0.0000 | 0.5315 | 0.0017 | 1.0000 | 0.0000 | 0.3339 | 0.5332 | 0.4658 | 0.0684 | 0.4658 | 0.1380 | 0.8022 |
| 0.1032 | 0.0065 | 1.7290 | 0.1458 | 0.0903 | 0.1032 | 0.2129 | 0.0000 | 0.6655 | 0.0053 | 1.0000 | 0.0000 | 0.0563 | 0.6708 | 0.4366 | 0.0237 | 0.5398 | 0.1407 | 0.7780 |
| 0.0000 | 0.0000 | 2.6000 | 0.3200 | 0.4000 | 0.0000 | 0.6000 | 0.0000 | 0.0544 | 0.8117 | 1.0000 | 0.0000 | 0.0921 | 0.8661 | 0.5000 | 0.0000 | 0.5000 | 0.1408 | NA |
| 0.0000 | 0.0000 | 1.3023 | 0.0605 | 0.8605 | 0.0000 | 0.1395 | 0.0000 | 0.3945 | 0.3364 | 1.0000 | 0.0000 | 0.0061 | 0.7309 | 0.3585 | 0.2829 | 0.3585 | 0.0838 | 0.8215 |
| 0.0000 | 0.0000 | 2.1818 | 0.2364 | 0.6364 | 0.0000 | 0.3636 | 0.0000 | 0.3461 | 0.3855 | 1.0000 | 0.0000 | 0.0171 | 0.7316 | 0.3939 | 0.2121 | 0.3939 | 0.1411 | 0.7116 |
| 0.0000 | 0.0000 | 2.0000 | 0.2000 | 0.6000 | 0.0000 | 0.4000 | 0.0000 | 0.1440 | 0.5506 | 1.0000 | 0.0000 | 0.1479 | 0.6965 | 0.4333 | 0.1333 | 0.4333 | 0.1616 | 0.8245 |
| 0.0000 | 0.0455 | 2.9091 | 0.3818 | 0.0455 | 0.0000 | 0.9091 | 0.0000 | 0.6484 | 0.2051 | 1.0000 | 0.0000 | 0.0659 | 0.8535 | 0.5000 | 0.0000 | 0.5000 | 0.0505 | 0.9564 |
| 0.0000 | 0.0000 | 2.8571 | 0.3714 | 0.1429 | 0.0000 | 0.8571 | 0.0000 | 0.3866 | 0.0905 | 1.0000 | 0.0000 | 0.5086 | 0.4771 | 0.5000 | 0.0000 | 0.5000 | 0.0718 | NA |
| 0.0000 | 0.0000 | 3.0000 | 0.4000 | 0.0000 | 0.0000 | 1.0000 | 0.0000 | 0.6226 | 0.2453 | 1.0000 | 0.0000 | 0.1195 | 0.8679 | 0.5000 | 0.0000 | 0.5000 | 0.0000 | NA |
| 0.0000 | 0.0000 | 1.6000 | 0.1200 | 0.2000 | 0.4000 | 0.0000 | 0.0000 | 0.5325 | 0.3252 | 1.0000 | 0.0000 | 0.1220 | 0.8577 | 0.5000 | 0.0000 | 0.5000 | 0.1128 | 0.9170 |
| 0.0000 | 0.0000 | 2.3729 | 0.2746 | 0.0169 | 0.0000 | 0.6780 | 0.0000 | 0.6667 | 0.1467 | 1.0000 | 0.0000 | 0.0797 | 0.8134 | 0.5000 | 0.0000 | 0.5000 | 0.1096 | 0.9607 |
| 0.0000 | 0.0000 | 2.0000 | 0.2000 | 1.0000 | 0.0000 | 0.0000 | 0.0000 | 0.6925 | 0.0087 | 1.0000 | 0.0000 | 0.2571 | 0.7012 | 0.5000 | 0.0000 | 0.5000 | 0.0000 | NA |
| 0.0593 | 0.5932 | 3.5830 | 0.5166 | 0.1398 | 0.1949 | 0.6610 | 0.0042 | 0.0000 | 0.0541 | 1.0000 | 0.0000 | 0.0000 | 0.0541 | 0.3715 | 0.1977 | 0.4308 | 0.0899 | 0.9005 |
| 0.0027 | 0.6469 | 3.4511 | 0.4902 | 0.1563 | 0.0647 | 0.7763 | 0.0027 | 0.0026 | 0.0158 | 1.0000 | 0.0000 | 0.0026 | 0.0211 | 0.3720 | 0.2264 | 0.4016 | 0.1023 | 0.9401 |
| 0.0090 | 0.6306 | 3.3484 | 0.4697 | 0.1892 | 0.1216 | 0.6847 | 0.0045 | 0.0000 | 0.0045 | 1.0000 | 0.0000 | 0.0000 | 0.0045 | 0.3731 | 0.2447 | 0.3821 | 0.0792 | 0.9292 |
| 0.0000 | 0.0000 | 1.2807 | 0.0561 | 0.4528 | 0.0094 | 0.5283 | 0.0094 | 0.1100 | 0.2435 | 1.0000 | 0.0000 | 0.4383 | 0.3535 | 0.0776 | 0.0000 | 0.9224 | 0.1193 | 0.8066 |
| 0.0000 | 0.0857 | 2.8148 | 0.3630 | 0.2286 | 0.0000 | 0.7714 | 0.0000 | 0.0160 | 0.3930 | 1.0000 | 0.0000 | 0.4011 | 0.4118 | 0.3548 | 0.0323 | 0.6129 | 0.1344 | 0.6582 |
| 0.0082 | 0.0000 | 2.4082 | 0.2816 | 0.1967 | 0.3443 | 0.4590 | 0.0000 | 0.0684 | 0.4085 | 1.0000 | 0.0000 | 0.2736 | 0.4809 | 0.3617 | 0.0000 | 0.6383 | 0.1134 | 0.7043 |
| 0.0059 | 0.1953 | 2.6923 | 0.3385 | 0.2663 | 0.0533 | 0.6331 | 0.0000 | 0.3288 | 0.1474 | 1.0000 | 0.0000 | 0.1315 | 0.4853 | 0.4002 | 0.1517 | 0.4481 | 0.1507 | 0.8435 |
| 0.0000 | 0.0290 | 1.9420 | 0.1884 | 0.1884 | 0.0000 | 0.3768 | 0.0000 | 0.5472 | 0.0210 | 1.0000 | 0.0000 | 0.1426 | 0.5681 | 0.4638 | 0.0725 | 0.4638 | 0.1222 | 0.6805 |
| 0.0278 | 0.0139 | 2.6181 | 0.3236 | 0.1736 | 0.0903 | 0.7361 | 0.0000 | 0.6661 | 0.0035 | 1.0000 | 0.0000 | 0.0787 | 0.6696 | 0.4282 | 0.0602 | 0.5116 | 0.1327 | 0.8003 |
| 0.0166 | 0.1331 | 2.5228 | 0.3046 | 0.1997 | 0.1082 | 0.5591 | 0.0000 | 0.6414 | 0.0687 | 1.0000 | 0.0000 | 0.0933 | 0.7101 | 0.4729 | 0.0114 | 0.5157 | 0.1438 | 0.8877 |
| 0.0180 | 0.1802 | 2.6147 | 0.3229 | 0.0541 | 0.0991 | 0.6486 | 0.0000 | 0.5721 | 0.1758 | 1.0000 | 0.0000 | 0.0680 | 0.7479 | 0.4735 | 0.0062 | 0.5202 | 0.1476 | 0.9385 |
| 0.0123 | 0.0000 | 2.0798 | 0.2160 | 0.6626 | 0.0184 | 0.3190 | 0.0000 | 0.5907 | 0.0056 | 1.0000 | 0.0000 | 0.0972 | 0.5981 | 0.3823 | 0.2145 | 0.4033 | 0.1191 | 0.4642 |
| 0.0976 | 0.0000 | 2.2439 | 0.2488 | 0.5488 | 0.1098 | 0.3415 | 0.0000 | 0.5941 | 0.0032 | 1.0000 | 0.0000 | 0.2706 | 0.5973 | 0.3851 | 0.1081 | 0.5068 | 0.1469 | 0.5136 |
| 0.0645 | 0.0483 | 2.3618 | 0.2724 | 0.6608 | 0.0814 | 0.2579 | 0.0000 | 0.1325 | 0.1616 | 1.0000 | 0.0000 | 0.2868 | 0.3040 | 0.3593 | 0.2041 | 0.4366 | 0.1199 | 0.5282 |
| 0.0913 | 0.0342 | 2.5270 | 0.3054 | 0.2797 | 0.1153 | 0.6050 | 0.0000 | 0.4800 | 0.0920 | 1.0000 | 0.0000 | 0.1119 | 0.5720 | 0.4177 | 0.0344 | 0.5479 | 0.1564 | 0.7545 |
| 0.0946 | 0.0180 | 2.5862 | 0.3172 | 0.3423 | 0.1171 | 0.2883 | 0.0000 | 0.0000 | 0.0833 | 1.0000 | 0.0000 | 0.0317 | 0.0873 | 0.5488 | 0.0240 | 0.4272 | 0.1702 | 0.7807 |

| | | | | | | | | | | | | | | | | | | |
|---|---|---|---|---|---|---|---|---|---|---|---|---|---|---|---|---|---|---|
| 0.0725 | 0.0207 | 3.1325 | 0.4265 | 0.5699 | 0.1295 | 0.3005 | 0.0000 | 0.0000 | 0.0203 | 1.0000 | 0.0000 | 0.0000 | 0.0228 | 0.2391 | 0.0140 | 0.7469 | 0.1363 | 0.7831 |
| 0.2403 | 0.0848 | 3.0528 | 0.4106 | 0.1484 | 0.3746 | 0.4735 | 0.0035 | 0.0000 | 0.0782 | 1.0000 | 0.0000 | 0.0000 | 0.0782 | 0.3658 | 0.0517 | 0.5824 | 0.1478 | 0.8884 |
| 0.0175 | 0.2807 | 3.0559 | 0.4112 | 0.0936 | 0.1930 | 0.7018 | 0.0000 | 0.0000 | 0.3246 | 0.9883 | 0.0000 | 0.0299 | 0.3321 | 0.4224 | 0.0994 | 0.4783 | 0.1399 | 0.8788 |
| 0.2234 | 0.2062 | 3.2038 | 0.4408 | 0.2818 | 0.3402 | 0.3780 | 0.0000 | 0.3393 | 0.0691 | 1.0000 | 0.0000 | 0.0000 | 0.4124 | 0.2157 | 0.0738 | 0.7105 | 0.1298 | 0.8943 |
| 0.0087 | 0.0873 | 2.9231 | 0.3846 | 0.2882 | 0.2576 | 0.4541 | 0.0000 | 0.0000 | 0.1611 | 1.0000 | 0.0000 | 0.0537 | 0.1779 | 0.3487 | 0.0353 | 0.6160 | 0.1343 | 0.8252 |
| 0.1262 | 0.2913 | 2.7816 | 0.3563 | 0.3107 | 0.2039 | 0.4854 | 0.0000 | 0.3024 | 0.0346 | 1.0000 | 0.0000 | 0.2160 | 0.3391 | 0.2545 | 0.1362 | 0.6093 | 0.1321 | 0.8189 |
| 0.0396 | 0.0634 | 2.7603 | 0.3521 | 0.2060 | 0.1902 | 0.5943 | 0.0095 | 0.0317 | 0.0634 | 1.0000 | 0.0000 | 0.1015 | 0.0983 | 0.4587 | 0.0266 | 0.5146 | 0.1548 | 0.8114 |
| 0.2192 | 0.0548 | 2.2571 | 0.2514 | 0.3425 | 0.3288 | 0.3014 | 0.0274 | 0.5731 | 0.0115 | 1.0000 | 0.0000 | 0.0127 | 0.8711 | 0.3684 | 0.0702 | 0.5614 | 0.1399 | 0.6938 |
| 0.2807 | 0.0351 | 2.7234 | 0.3447 | 0.2632 | 0.4123 | 0.3158 | 0.0000 | 0.0022 | 0.0584 | 1.0000 | 0.0000 | 0.6742 | 0.0697 | 0.2763 | 0.0175 | 0.7061 | 0.1510 | 0.7574 |
| 0.1988 | 0.0062 | 2.7523 | 0.3505 | 0.3602 | 0.2857 | 0.3292 | 0.0000 | 0.0568 | 0.1356 | 1.0000 | 0.0000 | 0.2934 | 0.1987 | 0.2443 | 0.0021 | 0.7536 | 0.1517 | 0.7163 |
| 0.0385 | 0.0385 | 2.5476 | 0.3095 | 0.2308 | 0.1538 | 0.5769 | 0.0000 | 0.2319 | 0.2138 | 1.0000 | 0.0000 | 0.1739 | 0.4493 | 0.3659 | 0.0181 | 0.6159 | 0.1575 | 0.8655 |
| 0.0000 | 0.0000 | 2.5155 | 0.3031 | 0.1856 | 0.0309 | 0.7835 | 0.0000 | 0.1298 | 0.3125 | 1.0000 | 0.0000 | 0.3221 | 0.4447 | 0.4863 | 0.0275 | 0.4863 | 0.1185 | 0.8743 |
| 0.0072 | 0.0290 | 2.6449 | 0.3290 | 0.3768 | 0.0145 | 0.6087 | 0.0000 | 0.4159 | 0.0994 | 1.0000 | 0.0000 | 0.1636 | 0.5176 | 0.4420 | 0.1087 | 0.4493 | 0.1307 | 0.7887 |
| 0.0000 | 0.0000 | 2.1704 | 0.2341 | 0.3852 | 0.0519 | 0.5630 | 0.0000 | 0.7239 | 0.0019 | 1.0000 | 0.0000 | 0.0224 | 0.7257 | 0.4431 | 0.1138 | 0.4431 | 0.1546 | 0.8933 |
| 0.0000 | 0.0000 | 2.9706 | 0.3941 | 0.9902 | 0.0098 | 0.0000 | 0.0000 | 0.0000 | 0.0000 | 1.0000 | 0.0000 | 0.1969 | 0.0000 | 0.4951 | 0.0000 | 0.5049 | 0.0053 | 0.1210 |
| 0.0288 | 0.0000 | 1.5821 | 0.1164 | 0.1787 | 0.0288 | 0.0720 | 0.7205 | 0.2418 | 0.3023 | 1.0000 | 0.0000 | 0.0363 | 0.5441 | 0.4942 | 0.0115 | 0.4942 | 0.0690 | 0.8804 |
| 0.0000 | 0.6522 | 1.9130 | 0.1826 | 0.0000 | 0.0870 | 0.2609 | 0.0000 | 0.6714 | 0.0953 | 1.0000 | 0.0000 | 0.0000 | 0.7667 | 0.4565 | 0.0000 | 0.5435 | 0.1284 | 0.6658 |
| 0.0588 | 0.3529 | 2.3529 | 0.2706 | 0.0000 | 0.1176 | 0.5294 | 0.0000 | 0.5249 | 0.0000 | 1.0000 | 0.0000 | 0.0055 | 0.5249 | 0.4608 | 0.0196 | 0.5196 | 0.1649 | 0.8484 |
| 0.0000 | 0.0000 | NA | NA | 1.0000 | 0.0000 | 0.0000 | 0.0000 | 0.4831 | 0.1689 | 1.0000 | 0.0000 | 0.3395 | 0.6520 | 0.0000 | 0.0000 | 1.0000 | 0.0000 | NA |
| 0.1087 | 0.5435 | 2.5217 | 0.3043 | 0.3261 | 0.1304 | 0.0000 | 0.0000 | 0.0000 | 0.0000 | 1.0000 | 0.0000 | 0.3521 | 0.0000 | 0.4710 | 0.0362 | 0.4928 | 0.0748 | 0.9148 |
| 0.1793 | 0.1992 | 1.8167 | 0.1633 | 0.2789 | 0.0239 | 0.0000 | 0.4980 | 0.0000 | 0.1340 | 1.0000 | 0.0000 | 0.0810 | 0.1371 | 0.4947 | 0.0066 | 0.4987 | 0.0923 | 0.9256 |
| 0.0598 | 0.0797 | 1.3586 | 0.0717 | 0.0199 | 0.0637 | 0.0398 | 0.7968 | 0.6038 | 0.0118 | 1.0000 | 0.0000 | 0.0147 | 0.6156 | 0.4880 | 0.0199 | 0.4920 | 0.0405 | 0.9203 |
| 0.0000 | 0.5000 | 1.5000 | 0.1000 | 0.0000 | 0.0000 | 0.0000 | 0.0000 | 0.5510 | 0.1736 | 1.0000 | 0.0000 | 0.0000 | 0.7245 | 0.5000 | 0.0000 | 0.5000 | 0.0731 | NA |
| NA | NA | NA | NA | NA | NA | NA | NA | 0.6209 | 0.3791 | NA | NA | 0.0000 | 1.0000 | NA | NA | NA | NA | NA |
| 0.2746 | 0.2746 | 1.6270 | 0.1254 | 0.0343 | 0.0000 | 0.0046 | 0.6865 | 0.0000 | 0.0337 | 1.0000 | 0.0000 | 0.0421 | 0.0379 | 0.5000 | 0.0000 | 0.5000 | 0.0465 | 0.8568 |
| 0.0000 | 0.0361 | 1.0361 | 0.0072 | 0.0000 | 0.0000 | 0.0000 | 0.9639 | 0.0000 | 0.0435 | 1.0000 | 0.0000 | 0.0543 | 0.0435 | 0.5000 | 0.0000 | 0.5000 | 0.0120 | NA |
| 0.0676 | 0.1622 | 1.5541 | 0.1108 | 0.0270 | 0.0000 | 0.1351 | 0.2703 | 0.0000 | 0.0080 | 1.0000 | 0.0000 | 0.0000 | 0.0107 | 0.5000 | 0.0000 | 0.5000 | 0.1398 | 0.8540 |
| 0.0000 | 0.0000 | 1.6667 | 0.1333 | 0.0000 | 0.0000 | 0.3333 | 0.6667 | 0.4269 | 0.3810 | 1.0000 | 0.0000 | 0.0000 | 0.8079 | 0.5000 | 0.0000 | 0.5000 | 0.1044 | NA |
| 0.0000 | 0.0000 | 2.0000 | 0.2000 | 0.0083 | 0.0000 | 0.4959 | 0.4959 | 0.5346 | 0.2096 | 1.0000 | 0.0000 | 0.0021 | 0.7442 | 0.5000 | 0.0000 | 0.5000 | 0.1183 | 0.9932 |
| 0.0000 | 0.0000 | 1.0000 | 0.0000 | 0.0000 | 0.0000 | 0.0000 | 1.0000 | 0.0000 | 0.0909 | 1.0000 | 0.0000 | 0.2674 | 0.0909 | 0.5000 | 0.0000 | 0.5000 | 0.0000 | NA |
| 0.0000 | 0.0000 | 1.0000 | 0.0000 | 0.0000 | 0.0000 | 0.0000 | 1.0000 | 0.0000 | 0.4286 | 1.0000 | 0.0000 | 0.4762 | 0.4286 | 0.5000 | 0.0000 | 0.5000 | 0.0000 | NA |
| 0.0000 | 0.2857 | 2.7143 | 0.3429 | 0.0000 | 0.0000 | 0.7143 | 0.0000 | 0.0000 | 0.8716 | 1.0000 | 0.0000 | 0.0000 | 0.8716 | 0.5000 | 0.0000 | 0.5000 | 0.1343 | NA |
| 0.0000 | 0.0000 | 3.0000 | 0.4000 | 1.0000 | 0.0000 | 0.0000 | 0.0000 | 0.0000 | 0.9979 | 1.0000 | 0.0000 | 0.0000 | 0.9979 | 0.5000 | 0.0000 | 0.5000 | 0.0000 | NA |
| 0.0000 | 0.0000 | 1.0000 | 0.0000 | 0.0000 | 0.0000 | 0.0000 | 1.0000 | 0.5333 | 0.4381 | 1.0000 | 0.0000 | 0.0000 | 0.9714 | 0.5000 | 0.0000 | 0.5000 | 0.0000 | NA |
| 0.0000 | 0.0000 | 1.3509 | 0.0702 | 0.4737 | 0.0000 | 0.1754 | 0.3509 | 0.0000 | 0.0234 | 1.0000 | 0.0000 | 0.0781 | 0.0313 | 0.5000 | 0.0000 | 0.5000 | 0.1242 | 0.7192 |
| 0.0000 | 0.3306 | 2.6529 | 0.3306 | 0.3471 | 0.0165 | 0.3388 | 0.2975 | 0.2655 | 0.1283 | 1.0000 | 0.0000 | 0.0708 | 0.3938 | 0.4449 | 0.1102 | 0.4449 | 0.1029 | 0.7778 |
| 0.1308 | 0.1682 | 1.8318 | 0.1664 | 0.0093 | 0.0000 | 0.2617 | 0.5234 | 0.3628 | 0.0000 | 1.0000 | 0.0000 | 0.1395 | 0.3628 | 0.5000 | 0.0000 | 0.5000 | 0.1059 | 0.8891 |
| 0.0000 | 0.3333 | 2.0000 | 0.2000 | 0.0000 | 0.2222 | 0.3333 | 0.4444 | 0.5429 | 0.1571 | 1.0000 | 0.0000 | 0.0143 | 0.7286 | 0.3333 | 0.1111 | 0.5556 | 0.1090 | 0.8410 |
| NA | NA | NA | NA | NA | NA | NA | NA | 0.0000 | 1.0000 | NA | NA | 0.0000 | 1.0000 | NA | NA | NA | NA | NA |
| 0.0000 | 0.0000 | 1.8734 | 0.1747 | 0.1392 | 0.4051 | 0.4557 | 0.0000 | 0.0000 | 0.0422 | 1.0000 | 0.0000 | 0.0060 | 0.0422 | 0.5000 | 0.0000 | 0.5000 | 0.1541 | 0.9045 |
| 0.1739 | 0.2029 | 2.9854 | 0.3971 | 0.0000 | 0.4855 | 0.5072 | 0.0072 | 0.5236 | 0.1073 | 1.0000 | 0.0000 | 0.0000 | 0.6387 | 0.4879 | 0.0676 | 0.4444 | 0.1618 | 0.7907 |
| 0.0893 | 0.0714 | 2.1429 | 0.2286 | 0.0000 | 0.0893 | 0.7857 | 0.0536 | 0.0072 | 0.1871 | 1.0000 | 0.0000 | 0.0000 | 0.1942 | 0.5982 | 0.0000 | 0.4018 | 0.0970 | 0.7054 |
| 0.0355 | 0.2837 | 3.2482 | 0.4496 | 0.0248 | 0.0532 | 0.9220 | 0.0000 | 0.0851 | 0.0763 | 1.0000 | 0.0000 | 0.0000 | 0.1725 | 0.4332 | 0.0946 | 0.4722 | 0.1196 | 0.9640 |
| 0.8000 | 0.0000 | 2.6000 | 0.3200 | 0.0000 | 1.0000 | 0.0000 | 0.0000 | 0.3902 | 0.4878 | 1.0000 | 0.0000 | 0.0000 | 0.8780 | 0.0000 | 0.0000 | 1.0000 | 0.0219 | NA |
| 0.0000 | 0.0376 | 1.9394 | 0.1879 | 0.7887 | 0.0376 | 0.1690 | 0.0047 | 0.0000 | 0.0932 | 1.0000 | 0.0000 | 0.0000 | 0.0975 | 0.2966 | 0.0125 | 0.6909 | 0.1218 | 0.7488 |
| 0.1877 | 0.0722 | 2.5926 | 0.3185 | 0.4946 | 0.2599 | 0.1877 | 0.0000 | 0.2555 | 0.0246 | 1.0000 | 0.0000 | 0.0393 | 0.2801 | 0.2280 | 0.0529 | 0.7190 | 0.1263 | 0.6761 |
| 0.2332 | 0.1076 | 2.5327 | 0.3065 | 0.1121 | 0.2601 | 0.2870 | 0.0000 | 0.5369 | 0.0302 | 1.0000 | 0.0000 | 0.0113 | 0.5671 | 0.3550 | 0.0837 | 0.5613 | 0.1621 | 0.8286 |
| 0.0894 | 0.2737 | 1.9553 | 0.1911 | 0.0894 | 0.1453 | 0.1173 | 0.0894 | 0.4673 | 0.0926 | 1.0000 | 0.0000 | 0.0361 | 0.5598 | 0.4339 | 0.0317 | 0.5345 | 0.1224 | 0.6520 |
| 0.0000 | 0.0000 | 1.6400 | 0.1280 | 0.0229 | 0.0000 | 0.2743 | 0.1714 | 0.5929 | 0.0713 | 1.0000 | 0.0000 | 0.0075 | 0.6642 | 0.5000 | 0.0000 | 0.5000 | 0.1023 | 0.9203 |
| 0.0955 | 0.0449 | 3.6677 | 0.5335 | 0.0927 | 0.1489 | 0.7528 | 0.0056 | 0.0000 | 0.0056 | 1.0000 | 0.0000 | 0.0000 | 0.0056 | 0.3343 | 0.1433 | 0.5225 | 0.1174 | 0.6717 |
| 0.0000 | 0.0905 | 2.2675 | 0.2535 | 0.2634 | 0.0782 | 0.4115 | 0.0000 | 0.5164 | 0.0275 | 1.0000 | 0.0000 | 0.0379 | 0.5439 | 0.4822 | 0.0274 | 0.4904 | 0.1553 | 0.7923 |

| | | | | | | | | | | | | | | | | | | |
|---|---|---|---|---|---|---|---|---|---|---|---|---|---|---|---|---|---|---|
| 0.0000 | 0.0690 | 2.6897 | 0.3379 | 0.2414 | 0.0000 | 0.6897 | 0.0000 | 0.0935 | 0.0187 | 1.0000 | 0.0000 | 0.0748 | 0.1121 | 0.5000 | 0.0000 | 0.5000 | 0.1290 | 0.7763 |
| 0.0000 | 0.1714 | 2.7647 | 0.3529 | 0.0857 | 0.0000 | 0.7429 | 0.0000 | 0.6235 | 0.3169 | 1.0000 | 0.0000 | 0.0000 | 0.9404 | 0.4857 | 0.0000 | 0.5143 | 0.1212 | 0.9444 |
| 0.0000 | 0.3540 | 1.6308 | 0.1262 | 0.4336 | 0.0000 | 0.0000 | 0.0000 | 0.7426 | 0.0275 | 1.0000 | 0.0000 | 0.0079 | 0.7701 | 0.2876 | 0.0000 | 0.7124 | 0.1012 | 0.8409 |
| 0.0000 | 0.0000 | 1.0110 | 0.0022 | 0.0164 | 0.0000 | 0.0000 | 0.9836 | 0.0000 | 0.0000 | 1.0000 | 0.0000 | 0.0985 | 0.0000 | 0.4973 | 0.0000 | 0.5027 | 0.0053 | 0.9959 |
| 0.0187 | 0.0466 | 1.3918 | 0.0784 | 0.0653 | 0.0019 | 0.1119 | 0.7463 | 0.0000 | 0.0387 | 1.0000 | 0.0000 | 0.0000 | 0.0563 | 0.4991 | 0.0000 | 0.5009 | 0.0681 | 0.9574 |
| 0.0000 | 0.5854 | 1.7805 | 0.1561 | 0.0732 | 0.0976 | 0.0488 | 0.0000 | 0.0000 | 0.0524 | 1.0000 | 0.0000 | 0.6317 | 0.0524 | 0.4512 | 0.0000 | 0.5488 | 0.1551 | 0.8426 |
| 0.0000 | 0.1111 | 1.8889 | 0.1778 | 0.4444 | 0.0000 | 0.0000 | 0.0000 | 0.0000 | 0.7209 | 1.0000 | 0.0000 | 0.0698 | 0.7209 | 0.5000 | 0.0000 | 0.5000 | 0.1469 | 0.8121 |
| 0.0000 | 1.0000 | 2.0000 | 0.2000 | 0.0000 | 0.0000 | 0.0000 | 0.0000 | 0.0000 | 0.3297 | 1.0000 | 0.0000 | 0.0110 | 0.3297 | 0.5000 | 0.0000 | 0.5000 | 0.0000 | NA |
| 0.0044 | 0.0088 | 1.0132 | 0.0026 | 0.0000 | 0.0000 | 0.0000 | 0.9912 | 0.0000 | 0.4017 | 1.0000 | 0.0000 | 0.1080 | 0.4017 | 0.5000 | 0.0000 | 0.5000 | 0.0021 | 0.9971 |
| 0.0420 | 0.0525 | 1.0945 | 0.0189 | 0.0021 | 0.0000 | 0.0000 | 0.9454 | 0.0000 | 0.0000 | 1.0000 | 0.0000 | 0.0000 | 0.0403 | 0.5000 | 0.0000 | 0.5000 | 0.0111 | 0.9790 |
| 0.0714 | 0.0714 | 1.4286 | 0.0857 | 0.1429 | 0.0000 | 0.0000 | 0.7143 | 0.0000 | 0.0455 | 1.0000 | 0.0000 | 0.0000 | 0.0455 | 0.5000 | 0.0000 | 0.5000 | 0.0679 | 0.8460 |
| 0.0588 | 1.0000 | 3.9412 | 0.5882 | 0.0000 | 0.0000 | 0.9412 | 0.0000 | 0.0000 | 0.2222 | 1.0000 | 0.0000 | 0.5051 | 0.3232 | 0.3431 | 0.3137 | 0.3431 | 0.0453 | 0.6612 |
| NA | NA | NA | NA | NA | NA | NA | NA | 0.0000 | 0.0000 | NA | NA | 1.0000 | 0.0000 | NA | NA | NA | NA | NA |
| NA | NA | NA | NA | NA | NA | NA | NA | 0.0000 | 0.6689 | NA | NA | 0.3311 | 0.6689 | NA | NA | NA | NA | NA |
| 0.0027 | 0.0162 | 1.0593 | 0.0119 | 0.0404 | 0.0000 | 0.0000 | 0.9434 | 0.0000 | 0.0536 | 1.0000 | 0.0000 | 0.0000 | 0.0536 | 0.5000 | 0.0000 | 0.5000 | 0.0187 | 0.9823 |
| 0.0000 | 0.0230 | 1.3678 | 0.0736 | 0.0000 | 0.0000 | 0.1724 | 0.8046 | 0.0000 | 0.0225 | 1.0000 | 0.0000 | 0.0000 | 0.0225 | 0.5000 | 0.0000 | 0.5000 | 0.0698 | 0.9788 |
| 0.6000 | 1.0000 | 2.6000 | 0.3200 | 0.0000 | 0.0000 | 0.0000 | 0.0000 | 0.0000 | 0.7525 | 1.0000 | 0.0000 | 0.0000 | 0.7525 | 0.5000 | 0.0000 | 0.5000 | 0.0993 | NA |
| 0.0000 | 0.5000 | 1.5000 | 0.1000 | 0.0000 | 0.5000 | 0.0000 | 0.0000 | 0.1333 | 0.5600 | 1.0000 | 0.0000 | 0.2167 | 0.7767 | 0.2500 | 0.0000 | 0.7500 | 0.1492 | NA |
| 0.0351 | 0.0351 | 1.0702 | 0.0140 | 0.0000 | 0.0000 | 0.0000 | 0.9649 | 0.0000 | 0.1071 | 1.0000 | 0.0000 | 0.0298 | 0.1220 | 0.5000 | 0.0000 | 0.5000 | 0.0069 | NA |
| 0.2857 | 0.2857 | 1.5714 | 0.1143 | 0.0000 | 0.0000 | 0.0000 | 0.7143 | 0.0275 | 0.1009 | 1.0000 | 0.0000 | 0.2294 | 0.1284 | 0.5000 | 0.0000 | 0.5000 | 0.0415 | NA |
| 0.1429 | 0.2619 | 1.7619 | 0.1524 | 0.0000 | 0.0000 | 0.1786 | 0.4762 | 0.0942 | 0.1149 | 1.0000 | 0.0000 | 0.0000 | 0.2090 | 0.5000 | 0.0000 | 0.5000 | 0.1097 | 0.7945 |
| 0.0259 | 0.0518 | 1.2073 | 0.0415 | 0.0000 | 0.0155 | 0.0518 | 0.8420 | 0.3336 | 0.0382 | 1.0000 | 0.0000 | 0.0000 | 0.3718 | 0.4965 | 0.0043 | 0.4991 | 0.0431 | 0.9655 |
| NA | NA | NA | NA | NA | NA | NA | NA | 0.0000 | 0.4483 | NA | NA | 0.5172 | 0.4828 | NA | NA | NA | NA | NA |
| 0.1124 | 0.3820 | 4.4810 | 0.6962 | 0.0449 | 0.4944 | 0.3933 | 0.0674 | 0.0000 | 0.0000 | 0.9775 | 0.0225 | 0.0000 | 0.0000 | 0.6961 | 0.1490 | 0.1549 | 0.1047 | 0.8688 |
| 0.0964 | 0.4217 | 4.6645 | 0.7329 | 0.0120 | 0.2410 | 0.6747 | 0.0723 | 0.0000 | 0.0000 | 0.9759 | 0.0241 | 0.0000 | 0.0000 | 0.6809 | 0.1626 | 0.1565 | 0.0909 | 0.9064 |
| 0.0645 | 0.2258 | 3.6339 | 0.5268 | 0.0000 | 0.3145 | 0.6210 | 0.0645 | 0.0000 | 0.0000 | 0.9677 | 0.0323 | 0.0000 | 0.0000 | 0.5917 | 0.1333 | 0.2750 | 0.1117 | 0.9044 |
| 0.1835 | 0.3761 | 4.0303 | 0.6061 | 0.0183 | 0.5046 | 0.4220 | 0.0550 | 0.0000 | 0.0000 | 0.9633 | 0.0367 | 0.0000 | 0.0000 | 0.6508 | 0.1556 | 0.1937 | 0.1183 | 0.8489 |
| 0.0171 | 0.2821 | 4.2394 | 0.6479 | 0.0085 | 0.4530 | 0.1624 | 0.3761 | 0.0000 | 0.0000 | 0.9915 | 0.0085 | 0.0000 | 0.0000 | 0.6101 | 0.0928 | 0.2971 | 0.1220 | 0.9182 |
| 0.0385 | 0.1385 | 3.5345 | 0.5069 | 0.0769 | 0.2692 | 0.1154 | 0.5385 | 0.0000 | 0.0000 | 1.0000 | 0.0000 | 0.0000 | 0.0000 | 0.5417 | 0.0807 | 0.3776 | 0.1056 | 0.9408 |
| 0.0583 | 0.4833 | 4.1161 | 0.6232 | 0.0167 | 0.7333 | 0.2000 | 0.0500 | 0.0000 | 0.0000 | 0.9000 | 0.1000 | 0.0000 | 0.0000 | 0.7542 | 0.1102 | 0.1356 | 0.1142 | 0.7990 |
| 0.1604 | 0.2453 | 3.4500 | 0.4900 | 0.0943 | 0.6604 | 0.2075 | 0.0377 | 0.0000 | 0.0000 | 0.9623 | 0.0377 | 0.0000 | 0.0000 | 0.5817 | 0.1827 | 0.2356 | 0.1104 | 0.7387 |
| 0.0282 | 0.1408 | 3.1176 | 0.4235 | 0.3521 | 0.4930 | 0.1127 | 0.0423 | 0.0000 | 0.0000 | 0.9296 | 0.0704 | 0.0000 | 0.0000 | 0.4906 | 0.1385 | 0.3709 | 0.1158 | 0.7736 |
| 0.1316 | 0.1930 | 3.2294 | 0.4459 | 0.3509 | 0.4912 | 0.1228 | 0.0351 | 0.0000 | 0.0000 | 0.9561 | 0.0351 | 0.0000 | 0.0000 | 0.5546 | 0.1298 | 0.3156 | 0.1175 | 0.7798 |
| 0.1391 | 0.1913 | 3.1927 | 0.4385 | 0.3652 | 0.4783 | 0.1217 | 0.0348 | 0.0000 | 0.0000 | 0.9565 | 0.0348 | 0.0000 | 0.0000 | 0.5929 | 0.0973 | 0.3097 | 0.1147 | 0.7836 |
| 0.0861 | 0.1060 | 2.8966 | 0.3793 | 0.2781 | 0.5563 | 0.1258 | 0.0397 | 0.0000 | 0.0000 | 0.9603 | 0.0331 | 0.0000 | 0.0000 | 0.4868 | 0.1755 | 0.3377 | 0.1023 | 0.8546 |
| 0.2706 | 0.1882 | 3.5190 | 0.5038 | 0.1176 | 0.6588 | 0.1529 | 0.0706 | 0.0000 | 0.0000 | 0.9294 | 0.0471 | 0.0000 | 0.0000 | 0.6353 | 0.1412 | 0.2235 | 0.1186 | 0.6829 |
| 0.2273 | 0.2000 | 3.5377 | 0.5075 | 0.1636 | 0.6727 | 0.1273 | 0.0364 | 0.0000 | 0.0000 | 0.9455 | 0.0364 | 0.0000 | 0.0000 | 0.5485 | 0.1848 | 0.2667 | 0.1141 | 0.7365 |
| 0.1515 | 0.2323 | 3.9570 | 0.5914 | 0.0808 | 0.6970 | 0.1818 | 0.0404 | 0.0000 | 0.0000 | 0.8990 | 0.0909 | 0.0000 | 0.0000 | 0.5962 | 0.2045 | 0.1993 | 0.1113 | 0.7470 |
| 0.0909 | 0.5636 | 4.3519 | 0.6704 | 0.0364 | 0.6273 | 0.3364 | 0.0000 | 0.0000 | 0.0000 | 0.9364 | 0.0636 | 0.0000 | 0.0000 | 0.7361 | 0.1343 | 0.1296 | 0.1120 | 0.8967 |
| 0.0446 | 0.2143 | 3.3853 | 0.4771 | 0.0357 | 0.4196 | 0.5357 | 0.0089 | 0.0000 | 0.0000 | 0.9911 | 0.0089 | 0.0000 | 0.0000 | 0.6045 | 0.1000 | 0.2955 | 0.1095 | 0.8651 |
| 0.1124 | 0.2921 | 3.8621 | 0.5724 | 0.1124 | 0.6067 | 0.2809 | 0.0000 | 0.0000 | 0.0000 | 0.9213 | 0.0787 | 0.0000 | 0.0000 | 0.5958 | 0.1705 | 0.2337 | 0.1257 | 0.7961 |
| 0.0603 | 0.2069 | 3.1842 | 0.4368 | 0.0862 | 0.3966 | 0.5172 | 0.0000 | 0.0000 | 0.0000 | 0.9483 | 0.0517 | 0.0000 | 0.0000 | 0.5702 | 0.1316 | 0.2982 | 0.1125 | 0.9328 |
| 0.0000 | 0.2133 | 3.5068 | 0.5014 | 0.0533 | 0.6933 | 0.2267 | 0.0267 | 0.0000 | 0.0000 | 0.9867 | 0.0133 | 0.0000 | 0.0000 | 0.6244 | 0.1711 | 0.2044 | 0.0942 | 0.8538 |
| 0.0783 | 0.4522 | 4.0721 | 0.6144 | 0.0870 | 0.6696 | 0.2261 | 0.0174 | 0.0000 | 0.0000 | 0.9826 | 0.0174 | 0.0000 | 0.0000 | 0.7345 | 0.0973 | 0.1681 | 0.1043 | 0.9038 |
| 0.0805 | 0.3893 | 4.4694 | 0.6939 | 0.0268 | 0.5302 | 0.4430 | 0.0000 | 0.0000 | 0.0000 | 0.9597 | 0.0268 | 0.0000 | 0.0000 | 0.6383 | 0.1723 | 0.1893 | 0.1112 | 0.8051 |
| 0.1386 | 0.3465 | 4.1134 | 0.6227 | 0.0396 | 0.6832 | 0.2574 | 0.0198 | 0.0000 | 0.0000 | 0.8614 | 0.1188 | 0.0000 | 0.0000 | 0.5842 | 0.2155 | 0.2003 | 0.1090 | 0.7580 |
| 0.1087 | 0.4239 | 4.1667 | 0.6333 | 0.0435 | 0.6957 | 0.2609 | 0.0000 | 0.0000 | 0.0000 | 0.9130 | 0.0870 | 0.0000 | 0.0000 | 0.5963 | 0.2296 | 0.1741 | 0.1041 | 0.8144 |
| 0.0734 | 0.2202 | 4.5238 | 0.7048 | 0.0367 | 0.7248 | 0.2202 | 0.0183 | 0.0000 | 0.0000 | 0.9817 | 0.0092 | 0.0000 | 0.0000 | 0.5234 | 0.2336 | 0.2430 | 0.0900 | 0.8703 |
| 0.0598 | 0.2051 | 4.5310 | 0.7062 | 0.0171 | 0.7521 | 0.2137 | 0.0171 | 0.0000 | 0.0000 | 0.9573 | 0.0256 | 0.0000 | 0.0000 | 0.5319 | 0.2362 | 0.2319 | 0.0937 | 0.8722 |
| 0.0840 | 0.5210 | 4.5310 | 0.7062 | 0.0168 | 0.7479 | 0.2017 | 0.0336 | 0.0000 | 0.0000 | 0.9664 | 0.0168 | 0.0000 | 0.0000 | 0.7123 | 0.1311 | 0.1567 | 0.1070 | 0.8284 |
| 0.1078 | 0.2451 | 4.1237 | 0.6247 | 0.0490 | 0.5686 | 0.3431 | 0.0392 | 0.0000 | 0.0000 | 0.8725 | 0.1176 | 0.0000 | 0.0000 | 0.5611 | 0.2046 | 0.2343 | 0.1061 | 0.7262 |

| | | | | | | | | | | | | | | | | | | |
|---|---|---|---|---|---|---|---|---|---|---|---|---|---|---|---|---|---|---|
| 0.1441 | 0.1171 | 3.0095 | 0.4019 | 0.1261 | 0.7207 | 0.1171 | 0.0360 | 0.0000 | 0.0000 | 0.6306 | 0.3604 | 0.0000 | 0.0000 | 0.4358 | 0.2202 | 0.3440 | 0.0995 | 0.6550 |
| 0.1882 | 0.2353 | 3.7089 | 0.5418 | 0.1412 | 0.6706 | 0.1412 | 0.0471 | 0.0000 | 0.0000 | 0.9294 | 0.0471 | 0.0000 | 0.0000 | 0.5181 | 0.2048 | 0.2771 | 0.1135 | 0.7635 |
| 0.0060 | 0.1257 | 3.7855 | 0.5571 | 0.1317 | 0.4192 | 0.4461 | 0.0030 | 0.0000 | 0.0000 | 0.9970 | 0.0030 | 0.0000 | 0.0000 | 0.5653 | 0.1345 | 0.3002 | 0.1267 | 0.6853 |
| 0.0190 | 0.2548 | 3.7931 | 0.5586 | 0.2281 | 0.5399 | 0.2281 | 0.0038 | 0.0000 | 0.0000 | 1.0000 | 0.0000 | 0.0000 | 0.0000 | 0.7385 | 0.0706 | 0.1908 | 0.1029 | 0.7254 |
| 0.0443 | 0.1734 | 3.8778 | 0.5756 | 0.1476 | 0.7122 | 0.1365 | 0.0037 | 0.0000 | 0.0000 | 1.0000 | 0.0000 | 0.0000 | 0.0000 | 0.6101 | 0.1544 | 0.2355 | 0.1173 | 0.7205 |
| 0.0295 | 0.2878 | 4.8392 | 0.7678 | 0.0074 | 0.6421 | 0.2915 | 0.0554 | 0.0000 | 0.0000 | 0.9963 | 0.0037 | 0.0000 | 0.0000 | 0.5580 | 0.2025 | 0.2395 | 0.0829 | 0.7832 |
| 0.1140 | 0.3051 | 4.5947 | 0.7189 | 0.0147 | 0.7647 | 0.1875 | 0.0294 | 0.0000 | 0.0000 | 0.9963 | 0.0037 | 0.0000 | 0.0000 | 0.6078 | 0.1814 | 0.2108 | 0.0926 | 0.7739 |
| 0.0508 | 0.3555 | 4.7261 | 0.7452 | 0.0195 | 0.6875 | 0.1953 | 0.0977 | 0.0000 | 0.0000 | 1.0000 | 0.0000 | 0.0000 | 0.0000 | 0.6529 | 0.1431 | 0.2039 | 0.0972 | 0.8306 |
| 0.0087 | 0.1948 | 4.5330 | 0.7066 | 0.0390 | 0.7749 | 0.1645 | 0.0173 | 0.0000 | 0.0000 | 0.9957 | 0.0043 | 0.0000 | 0.0000 | 0.5325 | 0.2121 | 0.2554 | 0.1005 | 0.8363 |
| 0.0044 | 0.3026 | 4.5773 | 0.7155 | 0.0526 | 0.5833 | 0.3246 | 0.0351 | 0.0000 | 0.0000 | 0.9956 | 0.0044 | 0.0000 | 0.0000 | 0.6177 | 0.1594 | 0.2230 | 0.0949 | 0.8097 |
| 0.1050 | 0.2605 | 4.2646 | 0.6529 | 0.1092 | 0.6639 | 0.1597 | 0.0630 | 0.0000 | 0.0000 | 1.0000 | 0.0000 | 0.0000 | 0.0000 | 0.5518 | 0.1506 | 0.2976 | 0.1141 | 0.7923 |
| 0.0375 | 0.1000 | 3.0741 | 0.4148 | 0.3125 | 0.3125 | 0.0500 | 0.3250 | 0.0000 | 0.0000 | 0.9875 | 0.0125 | 0.0123 | 0.0000 | 0.4833 | 0.1583 | 0.3583 | 0.0997 | 0.8531 |
| 0.0068 | 0.3831 | 3.6519 | 0.5304 | 0.1390 | 0.8034 | 0.0508 | 0.0068 | 0.0000 | 0.0000 | 1.0000 | 0.0000 | 0.0000 | 0.0000 | 0.5531 | 0.1463 | 0.3006 | 0.1082 | 0.8782 |
| 0.0863 | 0.4058 | 3.7653 | 0.5531 | 0.1310 | 0.8211 | 0.0415 | 0.0064 | 0.0000 | 0.0000 | 0.9968 | 0.0032 | 0.0000 | 0.0000 | 0.6645 | 0.1166 | 0.2188 | 0.1092 | 0.8453 |
| 0.0136 | 0.2687 | 3.3356 | 0.4671 | 0.2041 | 0.7143 | 0.0748 | 0.0068 | 0.0000 | 0.0000 | 0.9932 | 0.0068 | 0.0000 | 0.0000 | 0.5612 | 0.1565 | 0.2823 | 0.1082 | 0.9355 |
| 0.0130 | 0.2476 | 3.4027 | 0.4805 | 0.1857 | 0.7394 | 0.0456 | 0.0293 | 0.0000 | 0.0000 | 0.9967 | 0.0033 | 0.0000 | 0.0000 | 0.5157 | 0.1672 | 0.3170 | 0.1122 | 0.9198 |
| 0.0128 | 0.3269 | 3.4694 | 0.4939 | 0.2115 | 0.6859 | 0.0513 | 0.0513 | 0.0000 | 0.0000 | 0.9872 | 0.0064 | 0.0250 | 0.0000 | 0.4242 | 0.1807 | 0.3950 | 0.1061 | 0.9051 |
| 0.0106 | 0.4656 | 4.3008 | 0.6602 | 0.0265 | 0.5926 | 0.0899 | 0.2910 | 0.0000 | 0.0000 | 0.9947 | 0.0053 | 0.0000 | 0.0000 | 0.6791 | 0.0833 | 0.2376 | 0.0954 | 0.8875 |
| 0.0036 | 0.4124 | 4.3871 | 0.6774 | 0.0073 | 0.8029 | 0.0949 | 0.0949 | 0.0000 | 0.0000 | 1.0000 | 0.0000 | 0.0000 | 0.0000 | 0.6484 | 0.1411 | 0.2105 | 0.1131 | 0.7994 |
| 0.0113 | 0.5132 | 4.6961 | 0.7392 | 0.0038 | 0.6679 | 0.0981 | 0.2302 | 0.0000 | 0.0000 | 1.0000 | 0.0000 | 0.0000 | 0.0000 | 0.7164 | 0.0937 | 0.1899 | 0.1099 | 0.9254 |
| 0.1004 | 0.2788 | 4.1702 | 0.6340 | 0.0037 | 0.6059 | 0.0855 | 0.3011 | 0.0000 | 0.0000 | 1.0000 | 0.0000 | 0.0000 | 0.0000 | 0.6822 | 0.0669 | 0.2509 | 0.1148 | 0.8144 |
| 0.0746 | 0.4366 | 4.1208 | 0.6242 | 0.0187 | 0.6642 | 0.0858 | 0.2276 | 0.0000 | 0.0000 | 1.0000 | 0.0000 | 0.0000 | 0.0000 | 0.7245 | 0.0641 | 0.2114 | 0.1056 | 0.9134 |
| 0.0776 | 0.5297 | 4.2194 | 0.6439 | 0.0046 | 0.8128 | 0.0731 | 0.1050 | 0.0000 | 0.0000 | 1.0000 | 0.0000 | 0.0000 | 0.0000 | 0.7717 | 0.0845 | 0.1438 | 0.0997 | 0.8620 |
| 0.1000 | 0.1533 | 3.7828 | 0.5566 | 0.2100 | 0.6467 | 0.0333 | 0.1100 | 0.0000 | 0.0000 | 1.0000 | 0.0000 | 0.0000 | 0.0000 | 0.5156 | 0.2256 | 0.2589 | 0.1068 | 0.6407 |
| 0.1198 | 0.2727 | 3.3249 | 0.4650 | 0.3512 | 0.5868 | 0.0372 | 0.0207 | 0.0000 | 0.0000 | 1.0000 | 0.0000 | 0.0000 | 0.0000 | 0.5420 | 0.1329 | 0.3251 | 0.1075 | 0.7769 |
| 0.0567 | 0.3050 | 4.3038 | 0.6608 | 0.0213 | 0.5000 | 0.3333 | 0.1454 | 0.0000 | 0.0000 | 0.9965 | 0.0035 | 0.0505 | 0.0000 | 0.5947 | 0.1559 | 0.2494 | 0.1144 | 0.7486 |
| 0.0294 | 0.3324 | 4.4214 | 0.6843 | 0.0059 | 0.5353 | 0.3382 | 0.1206 | 0.0000 | 0.0000 | 0.9971 | 0.0029 | 0.0029 | 0.0000 | 0.6324 | 0.1515 | 0.2162 | 0.1106 | 0.8673 |
| 0.2396 | 0.3056 | 3.5167 | 0.5033 | 0.2604 | 0.6354 | 0.0382 | 0.0660 | 0.0000 | 0.0000 | 0.9931 | 0.0069 | 0.0000 | 0.0000 | 0.6440 | 0.0534 | 0.3026 | 0.1126 | 0.7871 |
| 0.1806 | 0.1498 | 3.1036 | 0.4207 | 0.4405 | 0.4493 | 0.0837 | 0.0220 | 0.0000 | 0.0000 | 0.9956 | 0.0044 | 0.0000 | 0.0000 | 0.4934 | 0.0705 | 0.4361 | 0.1197 | 0.7836 |
| 0.1886 | 0.1717 | 3.3096 | 0.4619 | 0.3367 | 0.5623 | 0.0438 | 0.0539 | 0.0000 | 0.0000 | 0.9966 | 0.0034 | 0.0034 | 0.0000 | 0.5735 | 0.0887 | 0.3378 | 0.1206 | 0.8030 |
| 0.1787 | 0.4449 | 3.4303 | 0.4861 | 0.2471 | 0.6274 | 0.0532 | 0.0722 | 0.0000 | 0.0000 | 0.9962 | 0.0038 | 0.0000 | 0.0000 | 0.6527 | 0.0824 | 0.2649 | 0.1121 | 0.6863 |
| 0.3815 | 0.0778 | 3.4557 | 0.4911 | 0.0593 | 0.7741 | 0.0296 | 0.1222 | 0.0000 | 0.0000 | 0.9963 | 0.0037 | 0.0000 | 0.0000 | 0.6642 | 0.1272 | 0.2086 | 0.1005 | 0.6640 |
| 0.0028 | 0.1723 | 4.4800 | 0.6960 | 0.0000 | 0.2966 | 0.7034 | 0.0000 | 0.0000 | 0.0000 | 1.0000 | 0.0000 | 0.0000 | 0.0000 | 0.4648 | 0.2676 | 0.2676 | 0.0960 | 0.8155 |
| 0.0027 | 0.2715 | 4.5445 | 0.7089 | 0.0000 | 0.5457 | 0.4543 | 0.0000 | 0.0000 | 0.0000 | 1.0000 | 0.0000 | 0.0000 | 0.0000 | 0.3482 | 0.3266 | 0.3252 | 0.0912 | 0.7871 |
| 0.0055 | 0.0220 | 3.0417 | 0.4083 | 0.1044 | 0.2473 | 0.1813 | 0.4670 | 0.0000 | 0.0000 | 1.0000 | 0.0000 | 0.1574 | 0.0000 | 0.5064 | 0.0366 | 0.4570 | 0.0999 | 0.5612 |
| 0.0068 | 0.0000 | 2.0714 | 0.2143 | 0.4247 | 0.5068 | 0.0548 | 0.0137 | 0.0000 | 0.0000 | 1.0000 | 0.0000 | 0.3272 | 0.0000 | 0.4783 | 0.0365 | 0.4852 | 0.1103 | 0.6825 |
| 0.0139 | 0.0069 | 2.2231 | 0.2446 | 0.0625 | 0.7708 | 0.0347 | 0.1319 | 0.0000 | 0.0000 | 1.0000 | 0.0000 | 0.5457 | 0.0000 | 0.4688 | 0.1042 | 0.4271 | 0.0885 | 0.7020 |
| 0.0000 | 0.0000 | 2.3667 | 0.2733 | 0.4854 | 0.2961 | 0.1408 | 0.0777 | 0.0000 | 0.0000 | 1.0000 | 0.0000 | 0.0000 | 0.0000 | 0.5089 | 0.0016 | 0.4895 | 0.0915 | 0.6130 |
| 0.0061 | 0.0000 | 2.3537 | 0.2707 | 0.5091 | 0.1758 | 0.1758 | 0.1394 | 0.0000 | 0.0000 | 1.0000 | 0.0000 | 0.0237 | 0.0000 | 0.4899 | 0.0323 | 0.4778 | 0.0941 | 0.7966 |
| 0.0064 | 0.0000 | 2.4444 | 0.2889 | 0.0513 | 0.3013 | 0.3910 | 0.2564 | 0.0000 | 0.0000 | 1.0000 | 0.0000 | 0.0000 | 0.0000 | 0.5032 | 0.0000 | 0.4968 | 0.0912 | 0.8384 |
| 0.0046 | 0.3041 | 4.8986 | 0.7797 | 0.0000 | 0.0461 | 0.9539 | 0.0000 | 0.0000 | 0.0000 | 0.9954 | 0.0046 | 0.0000 | 0.0000 | 0.6667 | 0.1598 | 0.1736 | 0.0886 | 0.8111 |
| 0.0159 | 0.0000 | 2.2193 | 0.2439 | 0.1640 | 0.0899 | 0.4286 | 0.3175 | 0.0000 | 0.0000 | 1.0000 | 0.0000 | 0.0000 | 0.0000 | 0.5106 | 0.0000 | 0.4894 | 0.0700 | 0.7218 |
| 0.0056 | 0.1469 | 3.1638 | 0.4328 | 0.8023 | 0.1808 | 0.0169 | 0.0000 | 0.0000 | 0.0000 | 0.9944 | 0.0056 | 0.0000 | 0.0000 | 0.4416 | 0.2721 | 0.2863 | 0.0516 | 0.8003 |
| 0.0000 | 0.0169 | 4.4068 | 0.6814 | 0.0000 | 0.3559 | 0.6441 | 0.0000 | 0.0000 | 0.0000 | 1.0000 | 0.0000 | 0.0000 | 0.0000 | 0.3610 | 0.3186 | 0.3203 | 0.0667 | 0.8827 |
| 0.0085 | 0.0085 | 1.8462 | 0.1692 | 0.7203 | 0.2542 | 0.0169 | 0.0085 | 0.0000 | 0.0000 | 0.9915 | 0.0000 | 0.0084 | 0.0000 | 0.4887 | 0.0565 | 0.4548 | 0.1033 | 0.8346 |
| 0.0000 | 0.5102 | 3.4286 | 0.4857 | 0.0204 | 0.7347 | 0.2449 | 0.0000 | 0.0000 | 0.0000 | 1.0000 | 0.0000 | 0.0000 | 0.0000 | 0.7449 | 0.1224 | 0.1327 | 0.1452 | 0.7631 |
| 0.0000 | 0.0000 | 1.4351 | 0.0870 | 0.8015 | 0.1832 | 0.0153 | 0.0000 | 0.0000 | 0.0000 | 1.0000 | 0.0000 | 0.0000 | 0.0000 | 0.4948 | 0.0262 | 0.4790 | 0.0906 | 0.8418 |
| 0.0000 | 0.0000 | 2.3298 | 0.2660 | 0.1702 | 0.3723 | 0.4574 | 0.0000 | 0.0000 | 0.0000 | 1.0000 | 0.0000 | 0.0000 | 0.0000 | 0.5035 | 0.0461 | 0.4504 | 0.1426 | 0.8148 |
| 0.0000 | 0.0000 | 2.5094 | 0.3019 | 0.1509 | 0.8491 | 0.0000 | 0.0000 | 0.0000 | 0.0000 | 0.9906 | 0.0000 | 0.0000 | 0.0000 | 0.4908 | 0.0458 | 0.4634 | 0.1585 | 0.5567 |
| 0.0734 | 0.0000 | 2.3486 | 0.2697 | 0.7798 | 0.1284 | 0.0917 | 0.0000 | 0.0000 | 0.0000 | 1.0000 | 0.0000 | 0.0000 | 0.0000 | 0.5000 | 0.0872 | 0.4128 | 0.0633 | 0.6537 |
| 0.0058 | 0.5000 | 2.9826 | 0.3965 | 0.3547 | 0.5174 | 0.1279 | 0.0000 | 0.0000 | 0.0000 | 1.0000 | 0.0000 | 0.0000 | 0.0000 | 0.7568 | 0.0300 | 0.2132 | 0.0818 | 0.9111 |

| | | | | | | | | | | | | | | | | | | |
|---|---|---|---|---|---|---|---|---|---|---|---|---|---|---|---|---|---|---|
| 0.0000 | 0.0000 | 1.8073 | 0.1615 | 0.9174 | 0.0826 | 0.0000 | 0.0000 | 0.0000 | 0.0000 | 1.0000 | 0.0000 | 0.0000 | 0.0000 | 0.4985 | 0.0076 | 0.4939 | 0.0751 | 0.9464 |
| 0.0000 | 0.0000 | 2.1290 | 0.2258 | 0.3226 | 0.1452 | 0.5323 | 0.0000 | 0.0000 | 0.0000 | 1.0000 | 0.0000 | 0.0000 | 0.0000 | 0.5134 | 0.0699 | 0.4167 | 0.1097 | 0.9764 |
| 0.0000 | 0.0000 | 1.9600 | 0.1920 | 0.2368 | 0.4211 | 0.3289 | 0.0132 | 0.0000 | 0.0000 | 0.9868 | 0.0000 | 0.0000 | 0.0000 | 0.5000 | 0.0347 | 0.4653 | 0.1417 | 0.7954 |
| 0.1121 | 0.0179 | 4.8206 | 0.7641 | 0.0000 | 0.5157 | 0.4843 | 0.0000 | 0.0000 | 0.0000 | 0.9955 | 0.0045 | 0.0000 | 0.0000 | 0.5015 | 0.2504 | 0.2481 | 0.0881 | 0.8580 |
| 0.0000 | 0.4428 | 4.5939 | 0.7188 | 0.0000 | 0.0746 | 0.9254 | 0.0000 | 0.0000 | 0.0000 | 1.0000 | 0.0000 | 0.0000 | 0.0000 | 0.6210 | 0.1997 | 0.1794 | 0.0494 | 0.7001 |
| 0.0545 | 0.0121 | 4.0311 | 0.6062 | 0.0061 | 0.6000 | 0.3939 | 0.0000 | 0.0000 | 0.0000 | 1.0000 | 0.0000 | 0.0000 | 0.0000 | 0.6760 | 0.0331 | 0.2909 | 0.0873 | 0.8093 |
| 0.0179 | 0.0430 | 3.9964 | 0.5993 | 0.0000 | 0.3262 | 0.6738 | 0.0000 | 0.0000 | 0.0000 | 1.0000 | 0.0000 | 0.0000 | 0.0000 | 0.4785 | 0.1792 | 0.3423 | 0.0983 | 0.8841 |
| 0.0884 | 0.0340 | 4.3469 | 0.6694 | 0.1020 | 0.2041 | 0.6939 | 0.0000 | 0.0000 | 0.0000 | 0.9932 | 0.0068 | 0.0000 | 0.0000 | 0.8821 | 0.0113 | 0.1066 | 0.0788 | 0.9118 |
| 0.0000 | 0.9609 | 4.0349 | 0.6070 | 0.0000 | 0.9913 | 0.0087 | 0.0000 | 0.0000 | 0.0000 | 0.9957 | 0.0000 | 0.0000 | 0.0000 | 1.0000 | 0.0000 | 0.0000 | 0.0415 | 0.2825 |
| 0.0048 | 0.4155 | 4.0821 | 0.6164 | 0.0000 | 0.0725 | 0.9275 | 0.0000 | 0.0000 | 0.0000 | 1.0000 | 0.0000 | 0.0000 | 0.0000 | 0.9807 | 0.0097 | 0.0097 | 0.0948 | 0.6844 |
| 0.0044 | 0.4649 | 4.8884 | 0.7777 | 0.0000 | 0.3333 | 0.6667 | 0.0000 | 0.0000 | 0.0000 | 1.0000 | 0.0000 | 0.0000 | 0.0000 | 0.6615 | 0.1704 | 0.1682 | 0.0896 | 0.9047 |
| 0.0811 | 0.4363 | 4.3238 | 0.6648 | 0.0000 | 0.2741 | 0.7259 | 0.0000 | 0.0000 | 0.0000 | 0.9923 | 0.0077 | 0.0000 | 0.0000 | 0.9365 | 0.0348 | 0.0287 | 0.1000 | 0.6572 |
| 0.0500 | 0.3333 | 4.5600 | 0.7120 | 0.0056 | 0.4056 | 0.5833 | 0.0056 | 0.0000 | 0.0000 | 1.0000 | 0.0000 | 0.0000 | 0.0000 | 0.7557 | 0.1278 | 0.1165 | 0.0955 | 0.8873 |
| 0.1156 | 0.5850 | 4.2587 | 0.6517 | 0.0000 | 0.2653 | 0.7347 | 0.0000 | 0.0000 | 0.0000 | 0.9932 | 0.0068 | 0.0000 | 0.0000 | 0.7774 | 0.1131 | 0.1096 | 0.0718 | 0.6297 |
| 0.0000 | 0.1089 | 4.9700 | 0.7940 | 0.0000 | 0.4194 | 0.5806 | 0.0000 | 0.0000 | 0.0000 | 1.0000 | 0.0000 | 0.0000 | 0.0000 | 0.3627 | 0.3455 | 0.2918 | 0.0602 | 0.8661 |
| 0.0307 | 0.3804 | 3.7914 | 0.5583 | 0.3374 | 0.1595 | 0.5031 | 0.0000 | 0.0000 | 0.0000 | 1.0000 | 0.0000 | 0.0000 | 0.0000 | 0.6748 | 0.0767 | 0.2485 | 0.1137 | 0.9287 |
| 0.0243 | 0.4320 | 4.2574 | 0.6515 | 0.0049 | 0.1117 | 0.8835 | 0.0000 | 0.0000 | 0.0000 | 0.9951 | 0.0049 | 0.0000 | 0.0000 | 0.9084 | 0.0470 | 0.0446 | 0.0974 | 0.6022 |
| 0.0284 | 0.1875 | 3.2330 | 0.4466 | 0.5739 | 0.1193 | 0.3068 | 0.0000 | 0.0000 | 0.0000 | 1.0000 | 0.0000 | 0.0000 | 0.0000 | 0.4744 | 0.0653 | 0.4602 | 0.1076 | 0.8602 |
| 0.3316 | 0.4385 | 4.4317 | 0.6863 | 0.0107 | 0.4866 | 0.5027 | 0.0000 | 0.0000 | 0.0000 | 0.9947 | 0.0053 | 0.0000 | 0.0000 | 0.9053 | 0.0446 | 0.0501 | 0.1021 | 0.8408 |
| 0.0263 | 0.4474 | 4.5551 | 0.7110 | 0.0658 | 0.3026 | 0.6272 | 0.0044 | 0.0000 | 0.0000 | 1.0000 | 0.0000 | 0.0000 | 0.0000 | 0.6966 | 0.1177 | 0.1857 | 0.0967 | 0.9021 |
| 0.0067 | 0.0234 | 3.1773 | 0.4355 | 0.5351 | 0.3846 | 0.0803 | 0.0000 | 0.0000 | 0.0000 | 0.9431 | 0.0569 | 0.0000 | 0.0000 | 0.4688 | 0.1460 | 0.3852 | 0.1078 | 0.9410 |
| 0.0083 | 0.3875 | 4.6122 | 0.7224 | 0.0000 | 0.3333 | 0.5000 | 0.1667 | 0.0000 | 0.0000 | 1.0000 | 0.0000 | 0.0000 | 0.0000 | 0.6257 | 0.2295 | 0.1448 | 0.0846 | 0.8131 |
| 0.0510 | 0.0255 | 3.2398 | 0.4480 | 0.5102 | 0.1173 | 0.3724 | 0.0000 | 0.0000 | 0.0000 | 1.0000 | 0.0000 | 0.0000 | 0.0000 | 0.7151 | 0.0085 | 0.2764 | 0.0933 | 0.9008 |
| 0.0214 | 0.0000 | 4.5455 | 0.7091 | 0.0000 | 0.0267 | 0.9733 | 0.0000 | 0.0000 | 0.0000 | 1.0000 | 0.0000 | 0.0000 | 0.0000 | 0.3378 | 0.3298 | 0.3324 | 0.0316 | 0.7852 |
| 0.0074 | 0.0775 | 4.9370 | 0.7874 | 0.0000 | 0.4982 | 0.5018 | 0.0000 | 0.0000 | 0.0000 | 0.9963 | 0.0037 | 0.0000 | 0.0000 | 0.3759 | 0.3167 | 0.3074 | 0.0727 | 0.9609 |
| 0.1111 | 0.1058 | 4.4550 | 0.6910 | 0.0212 | 0.3651 | 0.6138 | 0.0000 | 0.0000 | 0.0000 | 0.9947 | 0.0053 | 0.0000 | 0.0000 | 0.7266 | 0.1208 | 0.1526 | 0.1093 | 0.8115 |
| 0.0773 | 0.0000 | 4.8019 | 0.7604 | 0.0193 | 0.1401 | 0.8406 | 0.0000 | 0.0000 | 0.0000 | 1.0000 | 0.0000 | 0.0000 | 0.0000 | 0.3857 | 0.2963 | 0.3180 | 0.0648 | 0.8160 |
| 0.0000 | 0.0325 | 2.1818 | 0.2364 | 0.9091 | 0.0390 | 0.0519 | 0.0000 | 0.0000 | 0.0000 | 1.0000 | 0.0000 | 0.0000 | 0.0000 | 0.5422 | 0.0000 | 0.4578 | 0.0134 | 0.9626 |
| 0.3247 | 0.0130 | 3.1818 | 0.4364 | 0.0000 | 0.7922 | 0.1948 | 0.0130 | 0.0000 | 0.0000 | 0.6494 | 0.3506 | 0.7240 | 0.0000 | 0.2857 | 0.2662 | 0.4481 | 0.1094 | 0.7156 |
| 0.0305 | 0.0687 | 1.6565 | 0.1313 | 0.7634 | 0.1908 | 0.0458 | 0.0000 | 0.0000 | 0.0000 | 1.0000 | 0.0000 | 0.0076 | 0.0000 | 0.5229 | 0.0305 | 0.4466 | 0.0481 | 0.8266 |
| 0.0413 | 0.0744 | 3.4583 | 0.4917 | 0.0000 | 0.6446 | 0.3471 | 0.0083 | 0.0000 | 0.0000 | 0.5289 | 0.4711 | 0.6020 | 0.0000 | 0.3181 | 0.2389 | 0.4431 | 0.1098 | 0.6417 |
| 0.0246 | 0.3380 | 4.6408 | 0.7282 | 0.0000 | 0.6408 | 0.3592 | 0.0000 | 0.0000 | 0.0000 | 0.9859 | 0.0141 | 0.0000 | 0.0000 | 0.5640 | 0.2224 | 0.2136 | 0.0804 | 0.9009 |
| 0.0000 | 0.2485 | 4.0374 | 0.6075 | 0.2117 | 0.3098 | 0.4663 | 0.0123 | 0.0000 | 0.0000 | 0.9969 | 0.0031 | 0.0000 | 0.0000 | 0.4723 | 0.1692 | 0.3585 | 0.1109 | 0.7942 |
| 0.3084 | 0.0705 | 4.5531 | 0.7106 | 0.0000 | 0.5903 | 0.4097 | 0.0000 | 0.0000 | 0.0000 | 0.9736 | 0.0264 | 0.0000 | 0.0000 | 0.4112 | 0.2966 | 0.2922 | 0.0912 | 0.7362 |
| 0.0887 | 0.3686 | 4.6199 | 0.7240 | 0.0034 | 0.3072 | 0.6894 | 0.0000 | 0.0000 | 0.0000 | 0.9966 | 0.0034 | 0.0000 | 0.0000 | 0.7637 | 0.1199 | 0.1164 | 0.1011 | 0.8369 |
| 0.2833 | 0.0944 | 4.5733 | 0.7147 | 0.0043 | 0.7940 | 0.2017 | 0.0000 | 0.0000 | 0.0000 | 1.0000 | 0.0000 | 0.0000 | 0.0000 | 0.4361 | 0.2766 | 0.2874 | 0.0947 | 0.8135 |
| 0.1980 | 0.3135 | 4.6060 | 0.7212 | 0.0264 | 0.6370 | 0.3366 | 0.0000 | 0.0000 | 0.0000 | 1.0000 | 0.0000 | 0.0000 | 0.0000 | 0.5591 | 0.2213 | 0.2196 | 0.1049 | 0.8084 |
| 0.2087 | 0.1068 | 4.6373 | 0.7275 | 0.0049 | 0.6796 | 0.3155 | 0.0000 | 0.0000 | 0.0000 | 0.9903 | 0.0049 | 0.0000 | 0.0000 | 0.4171 | 0.2854 | 0.2976 | 0.0930 | 0.8590 |
| 0.1613 | 0.2823 | 4.7204 | 0.7441 | 0.0027 | 0.6075 | 0.3898 | 0.0000 | 0.0000 | 0.0000 | 1.0000 | 0.0000 | 0.0000 | 0.0000 | 0.5215 | 0.2419 | 0.2366 | 0.0952 | 0.8241 |
| 0.0000 | 0.0229 | 2.1170 | 0.2234 | 0.8000 | 0.1029 | 0.0743 | 0.0229 | 0.0000 | 0.0000 | 1.0000 | 0.0000 | 0.0000 | 0.0000 | 0.2333 | 0.0362 | 0.7305 | 0.1160 | 0.8455 |
| 0.0333 | 0.3148 | 4.7739 | 0.7548 | 0.0296 | 0.3481 | 0.5926 | 0.0296 | 0.0000 | 0.0000 | 1.0000 | 0.0000 | 0.0000 | 0.0000 | 0.6221 | 0.1815 | 0.1964 | 0.0920 | 0.7864 |
| 0.0277 | 0.1488 | 4.8408 | 0.7682 | 0.0000 | 0.4706 | 0.5294 | 0.0000 | 0.0000 | 0.0000 | 1.0000 | 0.0000 | 0.0000 | 0.0000 | 0.4469 | 0.2670 | 0.2860 | 0.0804 | 0.9082 |
| 0.0036 | 0.3879 | 4.5857 | 0.7171 | 0.0142 | 0.1851 | 0.8007 | 0.0000 | 0.0000 | 0.0000 | 1.0000 | 0.0000 | 0.0000 | 0.0000 | 0.7500 | 0.1250 | 0.1250 | 0.1058 | 0.7904 |
| 0.0866 | 0.0563 | 4.3932 | 0.6786 | 0.1082 | 0.4545 | 0.4372 | 0.0000 | 0.0000 | 0.0000 | 1.0000 | 0.0000 | 0.0000 | 0.0000 | 0.4523 | 0.2338 | 0.3139 | 0.0925 | 0.8454 |
| 0.0167 | 0.2292 | 4.7625 | 0.7525 | 0.0000 | 0.1583 | 0.8417 | 0.0000 | 0.0000 | 0.0000 | 1.0000 | 0.0000 | 0.0000 | 0.0000 | 0.5208 | 0.2375 | 0.2417 | 0.0733 | 0.8939 |
| 0.0319 | 0.6096 | 4.6972 | 0.7394 | 0.0359 | 0.1315 | 0.8327 | 0.0000 | 0.0000 | 0.0000 | 1.0000 | 0.0000 | 0.0000 | 0.0000 | 0.7862 | 0.0969 | 0.1169 | 0.0737 | 0.5313 |
| 0.0165 | 0.0083 | 4.5950 | 0.7190 | 0.0000 | 0.2727 | 0.7273 | 0.0000 | 0.0000 | 0.0000 | 0.9876 | 0.0124 | 0.0000 | 0.0000 | 0.3609 | 0.3134 | 0.3258 | 0.0503 | 0.9233 |
| 0.0150 | 0.0038 | 3.9008 | 0.5802 | 0.2256 | 0.0902 | 0.6842 | 0.0000 | 0.0000 | 0.0000 | 0.9850 | 0.0000 | 0.0000 | 0.0000 | 0.5649 | 0.1412 | 0.2939 | 0.0981 | 0.8621 |
| 0.0053 | 0.0214 | 4.1872 | 0.6374 | 0.2139 | 0.0963 | 0.6898 | 0.0000 | 0.0000 | 0.0000 | 0.9412 | 0.0588 | 0.0000 | 0.0000 | 0.8191 | 0.0463 | 0.1346 | 0.0774 | 0.9575 |
| 0.2656 | 0.2000 | 3.8713 | 0.5743 | 0.2098 | 0.4984 | 0.2918 | 0.0000 | 0.0000 | 0.0000 | 0.7770 | 0.2164 | 0.0000 | 0.0000 | 0.4857 | 0.3108 | 0.2035 | 0.1200 | 0.7962 |
| 0.0061 | 0.0000 | 3.6074 | 0.5215 | 0.0976 | 0.1159 | 0.7866 | 0.0000 | 0.0000 | 0.0000 | 0.9390 | 0.0549 | 0.0000 | 0.0000 | 0.3978 | 0.2812 | 0.3211 | 0.0748 | 0.8437 |

| | | | | | | | | | | | | | | | | | | |
|---|---|---|---|---|---|---|---|---|---|---|---|---|---|---|---|---|---|---|
| 0.0047 | 0.2383 | 4.2817 | 0.6563 | 0.0000 | 0.4252 | 0.5748 | 0.0000 | 0.0000 | 0.0000 | 1.0000 | 0.0000 | 0.0000 | 0.0000 | 0.4100 | 0.2246 | 0.3654 | 0.1073 | 0.7274 |
| 0.0033 | 0.1596 | 4.7124 | 0.7425 | 0.0489 | 0.0423 | 0.9088 | 0.0000 | 0.0000 | 0.0000 | 1.0000 | 0.0000 | 0.0000 | 0.0000 | 0.7778 | 0.1111 | 0.1111 | 0.0985 | 0.8199 |
| 0.3182 | 0.2386 | 4.2471 | 0.6494 | 0.0038 | 0.5947 | 0.4015 | 0.0000 | 0.0000 | 0.0000 | 0.7576 | 0.2386 | 0.0000 | 0.0000 | 0.4227 | 0.3999 | 0.1774 | 0.1036 | 0.5892 |
| 0.2183 | 0.1472 | 4.5663 | 0.7133 | 0.0000 | 0.4619 | 0.5381 | 0.0000 | 0.0000 | 0.0000 | 0.9797 | 0.0203 | 0.0000 | 0.0000 | 0.3716 | 0.3206 | 0.3078 | 0.1017 | 0.8452 |
| 0.1711 | 0.4652 | 3.9570 | 0.5914 | 0.0053 | 0.6952 | 0.2941 | 0.0053 | 0.0000 | 0.0000 | 1.0000 | 0.0000 | 0.0000 | 0.0000 | 0.5187 | 0.2219 | 0.2594 | 0.1122 | 0.6694 |
| 0.0066 | 0.2697 | 4.7020 | 0.7404 | 0.0000 | 0.2632 | 0.7368 | 0.0000 | 0.0000 | 0.0000 | 1.0000 | 0.0000 | 0.0000 | 0.0000 | 0.4658 | 0.2737 | 0.2605 | 0.0696 | 0.8109 |
| 0.3374 | 0.0494 | 4.2314 | 0.6463 | 0.0000 | 0.5556 | 0.4444 | 0.0000 | 0.0000 | 0.0000 | 0.9753 | 0.0247 | 0.0000 | 0.0000 | 0.3946 | 0.3099 | 0.2955 | 0.0911 | 0.7338 |
| 0.0000 | 0.1336 | 3.8387 | 0.5677 | 0.0000 | 0.1060 | 0.8940 | 0.0000 | 0.0000 | 0.0000 | 1.0000 | 0.0000 | 0.0000 | 0.0000 | 0.6313 | 0.1843 | 0.1843 | 0.0799 | 0.9255 |
| 0.0258 | 0.2194 | 4.4175 | 0.6835 | 0.0000 | 0.2258 | 0.7742 | 0.0000 | 0.0000 | 0.0000 | 0.9742 | 0.0258 | 0.0000 | 0.0000 | 0.7282 | 0.1424 | 0.1294 | 0.0853 | 0.7022 |
| 0.0060 | 0.0119 | 4.2083 | 0.6417 | 0.0000 | 0.0417 | 0.9583 | 0.0000 | 0.0000 | 0.0000 | 0.9940 | 0.0060 | 0.0000 | 0.0000 | 0.4018 | 0.2946 | 0.3036 | 0.0416 | 0.8007 |
| 0.0124 | 0.0867 | 4.8204 | 0.7641 | 0.0000 | 0.2260 | 0.7740 | 0.0000 | 0.0000 | 0.0000 | 1.0000 | 0.0000 | 0.0000 | 0.0000 | 0.5542 | 0.2229 | 0.2229 | 0.0870 | 0.9174 |
| 0.0000 | 0.0000 | 2.2295 | 0.2459 | 0.0000 | 0.9836 | 0.0164 | 0.0000 | 0.0000 | 0.0000 | 0.9918 | 0.0082 | 0.0000 | 0.0000 | 0.4959 | 0.3811 | 0.1230 | 0.1134 | 0.8034 |
| 0.0394 | 0.0315 | 2.2276 | 0.2455 | 0.7008 | 0.2677 | 0.0315 | 0.0000 | 0.0000 | 0.0000 | 1.0000 | 0.0000 | 0.0929 | 0.0000 | 0.5379 | 0.0664 | 0.3957 | 0.1075 | 0.6993 |
| 0.0248 | 0.0941 | 2.9802 | 0.3960 | 0.0396 | 0.8762 | 0.0842 | 0.0000 | 0.0000 | 0.0000 | 1.0000 | 0.0000 | 0.0818 | 0.0000 | 0.3350 | 0.2285 | 0.4365 | 0.1056 | 0.7457 |
| 0.0000 | 0.6680 | 4.9157 | 0.7831 | 0.0000 | 0.1304 | 0.8696 | 0.0000 | 0.0000 | 0.0000 | 1.0000 | 0.0000 | 0.0000 | 0.0000 | 0.8106 | 0.0937 | 0.0957 | 0.0723 | 0.8945 |
| 0.0000 | 0.0904 | 4.5876 | 0.7175 | 0.0056 | 0.0904 | 0.9040 | 0.0000 | 0.0000 | 0.0000 | 1.0000 | 0.0000 | 0.0000 | 0.0000 | 0.7608 | 0.1111 | 0.1281 | 0.0774 | 0.8952 |
| 0.1557 | 0.0000 | 2.2318 | 0.2464 | 0.4551 | 0.2994 | 0.2395 | 0.0060 | 0.0000 | 0.0000 | 0.9940 | 0.0060 | 0.0000 | 0.0000 | 0.5729 | 0.0070 | 0.4202 | 0.1213 | 0.9180 |
| 0.0038 | 0.0792 | 4.5170 | 0.7034 | 0.0000 | 0.2151 | 0.7849 | 0.0000 | 0.0000 | 0.0000 | 0.9962 | 0.0038 | 0.0000 | 0.0000 | 0.6862 | 0.1579 | 0.1560 | 0.1149 | 0.5279 |
| 0.0065 | 0.0000 | 2.0759 | 0.2152 | 0.4575 | 0.0980 | 0.3922 | 0.0523 | 0.0000 | 0.0000 | 1.0000 | 0.0000 | 0.0000 | 0.0000 | 0.5022 | 0.0022 | 0.4955 | 0.0978 | 0.6881 |
| 0.0046 | 0.0685 | 4.6895 | 0.7379 | 0.0000 | 0.0913 | 0.9087 | 0.0000 | 0.0000 | 0.0000 | 1.0000 | 0.0000 | 0.0000 | 0.0000 | 0.5883 | 0.1933 | 0.2184 | 0.0636 | 0.8873 |
| 0.0000 | 0.0300 | 2.8577 | 0.3715 | 0.3745 | 0.4457 | 0.1798 | 0.0000 | 0.0000 | 0.0000 | 0.7453 | 0.2547 | 0.0000 | 0.0000 | 0.6317 | 0.1373 | 0.2310 | 0.1425 | 0.7850 |
| 0.0027 | 0.0701 | 4.4550 | 0.6910 | 0.0000 | 0.2453 | 0.7547 | 0.0000 | 0.0000 | 0.0000 | 0.9973 | 0.0027 | 0.0000 | 0.0000 | 0.5536 | 0.2239 | 0.2225 | 0.1068 | 0.7111 |
| 0.0000 | 0.0000 | 2.8833 | 0.3767 | 0.5372 | 0.4545 | 0.0000 | 0.0083 | 0.0000 | 0.0000 | 0.9917 | 0.0083 | 0.0082 | 0.0000 | 0.5275 | 0.0152 | 0.4573 | 0.0739 | 0.3953 |
| 0.0383 | 0.3397 | 4.2587 | 0.6517 | 0.1914 | 0.2727 | 0.5359 | 0.0000 | 0.0000 | 0.0000 | 1.0000 | 0.0000 | 0.0000 | 0.0000 | 0.8226 | 0.0837 | 0.0937 | 0.1049 | 0.7838 |
| 0.0247 | 0.1914 | 4.5556 | 0.7111 | 0.0123 | 0.1235 | 0.8642 | 0.0000 | 0.0000 | 0.0000 | 1.0000 | 0.0000 | 0.0000 | 0.0000 | 0.8323 | 0.0854 | 0.0823 | 0.0987 | 0.8028 |
| 0.0157 | 0.0827 | 4.9209 | 0.7842 | 0.0000 | 0.6575 | 0.3425 | 0.0000 | 0.0000 | 0.0000 | 1.0000 | 0.0000 | 0.0000 | 0.0000 | 0.4025 | 0.2997 | 0.2978 | 0.0631 | 0.9291 |
| 0.0315 | 0.7008 | 4.5433 | 0.7087 | 0.0079 | 0.0709 | 0.9213 | 0.0000 | 0.0000 | 0.0000 | 1.0000 | 0.0000 | 0.0000 | 0.0000 | 0.9488 | 0.0236 | 0.0276 | 0.0485 | 0.6199 |
| 0.0144 | 0.1986 | 4.9267 | 0.7853 | 0.0000 | 0.4657 | 0.5343 | 0.0000 | 0.0000 | 0.0000 | 1.0000 | 0.0000 | 0.0000 | 0.0000 | 0.6264 | 0.1868 | 0.1868 | 0.0821 | 0.9105 |
| 0.0163 | 0.2683 | 4.4065 | 0.6813 | 0.0000 | 0.2927 | 0.7073 | 0.0000 | 0.0000 | 0.0000 | 1.0000 | 0.0000 | 0.0000 | 0.0000 | 0.5339 | 0.2188 | 0.2473 | 0.0929 | 0.8914 |
| 0.1497 | 0.4132 | 4.5964 | 0.7193 | 0.0000 | 0.2335 | 0.7665 | 0.0000 | 0.0000 | 0.0000 | 1.0000 | 0.0000 | 0.0060 | 0.0000 | 0.7530 | 0.1114 | 0.1355 | 0.0833 | 0.7624 |
| 0.0046 | 0.1826 | 3.7546 | 0.5509 | 0.0091 | 0.2511 | 0.7306 | 0.0091 | 0.0000 | 0.0000 | 0.9909 | 0.0091 | 0.2369 | 0.0000 | 0.5327 | 0.1309 | 0.3364 | 0.0956 | 0.7106 |
| 0.3119 | 0.1651 | 2.3945 | 0.2789 | 0.3761 | 0.3119 | 0.1651 | 0.0000 | 0.5889 | 0.0197 | 1.0000 | 0.0000 | 0.0000 | 0.6086 | 0.3165 | 0.0550 | 0.6284 | 0.1230 | 0.6814 |
| 0.0204 | 0.0000 | 1.2755 | 0.0551 | 0.3776 | 0.0306 | 0.0000 | 0.0000 | 0.7984 | 0.0060 | 1.0000 | 0.0000 | 0.0000 | 0.8044 | 0.4847 | 0.0000 | 0.5153 | 0.0940 | 0.4556 |
| 0.0000 | 0.0769 | 1.7308 | 0.1462 | 0.8462 | 0.0385 | 0.0385 | 0.0000 | 0.6289 | 0.0692 | 1.0000 | 0.0000 | 0.1384 | 0.6981 | 0.4679 | 0.0256 | 0.5064 | 0.1331 | 0.7887 |
| 0.0000 | 0.0095 | 1.2402 | 0.0480 | 0.2190 | 0.0095 | 0.0095 | 0.0000 | 0.6233 | 0.0051 | 1.0000 | 0.0000 | 0.0169 | 0.6284 | 0.4635 | 0.0349 | 0.5016 | 0.1195 | 0.7416 |
| 0.0000 | 0.0000 | 1.2468 | 0.0494 | 0.2662 | 0.0065 | 0.0000 | 0.0000 | 0.7029 | 0.0018 | 1.0000 | 0.0000 | 0.0163 | 0.7047 | 0.4665 | 0.0606 | 0.4729 | 0.1168 | 0.8194 |
| 0.0000 | 0.0000 | 1.1712 | 0.0342 | 0.3894 | 0.0000 | 0.0000 | 0.0000 | 0.5455 | 0.0000 | 1.0000 | 0.0000 | 0.0594 | 0.5455 | 0.4912 | 0.0000 | 0.5088 | 0.1262 | 0.8110 |
| 0.0000 | 0.0000 | 1.7273 | 0.1455 | 0.5909 | 0.0455 | 0.0000 | 0.0000 | 0.8337 | 0.0737 | 1.0000 | 0.0000 | 0.0000 | 0.9074 | 0.4773 | 0.0000 | 0.5227 | 0.0537 | 0.6853 |
| 0.0000 | 0.0580 | 2.0000 | 0.2000 | 0.7246 | 0.0435 | 0.0000 | 0.0000 | 0.6932 | 0.1017 | 1.0000 | 0.0000 | 0.0776 | 0.7948 | 0.4638 | 0.0000 | 0.5362 | 0.0892 | 0.6186 |
| 0.0000 | 0.0159 | 1.3968 | 0.0794 | 0.2540 | 0.0317 | 0.0000 | 0.0000 | 0.4785 | 0.3203 | 1.0000 | 0.0000 | 0.0781 | 0.7988 | 0.4841 | 0.0000 | 0.5159 | 0.0633 | 0.5157 |
| 0.0000 | 0.0000 | 1.7182 | 0.1436 | 0.6091 | 0.0000 | 0.0000 | 0.0000 | 0.4325 | 0.0150 | 1.0000 | 0.0000 | 0.0000 | 0.4500 | 0.5000 | 0.0000 | 0.5000 | 0.0847 | 0.4992 |
| 0.0000 | 0.0102 | 1.5525 | 0.1105 | 0.4693 | 0.0085 | 0.0000 | 0.0000 | 0.5592 | 0.0073 | 1.0000 | 0.0000 | 0.0037 | 0.5666 | 0.4915 | 0.0000 | 0.5085 | 0.1186 | 0.6776 |
| 0.0000 | 0.0000 | 1.5278 | 0.1056 | 0.2500 | 0.0000 | 0.0000 | 0.0000 | 0.6876 | 0.0180 | 1.0000 | 0.0000 | 0.0359 | 0.7056 | 0.5000 | 0.0000 | 0.5000 | 0.0513 | 0.5949 |
| 0.0000 | 0.0123 | 1.9259 | 0.1852 | 0.4198 | 0.1481 | 0.0000 | 0.0000 | 0.6127 | 0.2309 | 1.0000 | 0.0000 | 0.0037 | 0.8454 | 0.4259 | 0.0000 | 0.5741 | 0.1072 | 0.6371 |
| 0.0247 | 0.0741 | 2.4074 | 0.2815 | 0.3704 | 0.0988 | 0.0000 | 0.0000 | 0.6991 | 0.1486 | 1.0000 | 0.0000 | 0.0037 | 0.8477 | 0.4506 | 0.0000 | 0.5494 | 0.0893 | 0.4510 |
| 0.0000 | 0.0185 | 1.4753 | 0.0951 | 0.3086 | 0.0185 | 0.0000 | 0.0000 | 0.5310 | 0.0901 | 1.0000 | 0.0000 | 0.0750 | 0.6210 | 0.4907 | 0.0000 | 0.5093 | 0.1233 | 0.7783 |
| 0.0000 | 0.0044 | 1.6267 | 0.1253 | 0.7111 | 0.1422 | 0.1022 | 0.0000 | 0.0043 | 0.0383 | 1.0000 | 0.0000 | 0.0000 | 0.0426 | 0.4993 | 0.0015 | 0.4993 | 0.1065 | 0.8278 |
| 0.0926 | 0.0093 | 1.6481 | 0.1296 | 0.4074 | 0.2778 | 0.0185 | 0.0000 | 0.7514 | 0.0019 | 1.0000 | 0.0000 | 0.0385 | 0.7534 | 0.4522 | 0.0031 | 0.5448 | 0.1025 | 0.6856 |
| 0.0000 | 0.0000 | 1.6480 | 0.1296 | 0.6480 | 0.1280 | 0.1280 | 0.0000 | 0.0000 | 0.2000 | 1.0000 | 0.0000 | 0.2000 | 0.2048 | 0.5000 | 0.0000 | 0.5000 | 0.1264 | 0.8578 |
| 0.0000 | 0.0128 | 1.2244 | 0.0449 | 0.2308 | 0.0128 | 0.0000 | 0.0000 | 0.5747 | 0.1228 | 1.0000 | 0.0000 | 0.0249 | 0.6975 | 0.4915 | 0.0043 | 0.5043 | 0.0712 | 0.4709 |
| 0.0442 | 0.0055 | 1.1732 | 0.0346 | 0.0773 | 0.0442 | 0.0055 | 0.0110 | 0.5129 | 0.0155 | 1.0000 | 0.0000 | 0.0000 | 0.5335 | 0.4761 | 0.0037 | 0.5203 | 0.0616 | 0.4837 |

| | | | | | | | | | | | | | | | | | | |
|---|---|---|---|---|---|---|---|---|---|---|---|---|---|---|---|---|---|---|
| 0.0000 | 0.0000 | 1.4118 | 0.0824 | 0.4118 | 0.0000 | 0.0000 | 0.0000 | 0.6533 | 0.1797 | 1.0000 | 0.0000 | 0.0951 | 0.8330 | 0.4608 | 0.0784 | 0.4608 | 0.0487 | 0.6000 |
| 0.0000 | 0.0109 | 3.6557 | 0.5311 | 0.1530 | 0.0000 | 0.8306 | 0.0000 | 0.0000 | 0.0047 | 1.0000 | 0.0000 | 0.1321 | 0.0047 | 0.5000 | 0.0000 | 0.5000 | 0.0696 | 0.1780 |
| 0.0000 | 0.0000 | 3.7644 | 0.5529 | 0.0000 | 0.0000 | 0.9215 | 0.0000 | 0.0851 | 0.0000 | 1.0000 | 0.0000 | 0.1021 | 0.0851 | 0.5000 | 0.0000 | 0.5000 | 0.0282 | 0.2648 |
| 0.0127 | 0.0127 | 2.4286 | 0.2857 | 0.1519 | 0.0506 | 0.4051 | 0.0253 | 0.0000 | 0.6293 | 1.0000 | 0.0000 | 0.1246 | 0.6293 | 0.4726 | 0.0042 | 0.5232 | 0.0924 | 0.7922 |
| 0.0000 | 0.0000 | 1.1857 | 0.0371 | 0.0429 | 0.0429 | 0.0000 | 0.0000 | 0.4060 | 0.2678 | 1.0000 | 0.0000 | 0.0238 | 0.6739 | 0.4786 | 0.0000 | 0.5214 | 0.0582 | 0.7529 |
| 0.0000 | 0.2373 | 1.4068 | 0.0814 | 0.1695 | 0.0000 | 0.0000 | 0.0000 | 0.4428 | 0.3528 | 1.0000 | 0.0000 | 0.0608 | 0.7956 | 0.5000 | 0.0000 | 0.5000 | 0.0489 | 0.6161 |
| 0.0000 | 0.0000 | 1.0803 | 0.0161 | 0.0146 | 0.0073 | 0.0000 | 0.0000 | 0.4195 | 0.1970 | 1.0000 | 0.0000 | 0.0932 | 0.6165 | 0.4964 | 0.0000 | 0.5036 | 0.0552 | 0.8735 |
| 0.0000 | 0.0000 | 1.6627 | 0.1325 | 0.2840 | 0.0000 | 0.0000 | 0.0059 | 0.5066 | 0.0158 | 1.0000 | 0.0000 | 0.0317 | 0.5224 | 0.5000 | 0.0000 | 0.5000 | 0.0582 | 0.4967 |
| 0.0000 | 0.0000 | 1.1760 | 0.0352 | 0.1920 | 0.0000 | 0.0000 | 0.0080 | 0.5566 | 0.0637 | 1.0000 | 0.0000 | 0.0849 | 0.6203 | 0.5000 | 0.0000 | 0.5000 | 0.0610 | 0.3189 |
| 0.0000 | 0.0159 | 1.1852 | 0.0370 | 0.0899 | 0.0688 | 0.0000 | 0.0000 | 0.2661 | 0.2167 | 1.0000 | 0.0000 | 0.1116 | 0.4828 | 0.4974 | 0.0000 | 0.5026 | 0.0707 | 0.5461 |
| 0.0000 | 0.0000 | 1.1176 | 0.0235 | 0.1373 | 0.0000 | 0.0000 | 0.0000 | 0.5730 | 0.0142 | 1.0000 | 0.0000 | 0.0498 | 0.5872 | 0.5000 | 0.0000 | 0.5000 | 0.0814 | 0.9174 |
| 0.0000 | 0.0000 | 1.0821 | 0.0164 | 0.2019 | 0.0000 | 0.0000 | 0.0000 | 0.3532 | 0.0525 | 1.0000 | 0.0000 | 0.0883 | 0.4153 | 0.4976 | 0.0000 | 0.5024 | 0.0885 | 0.8913 |
| 0.0000 | 0.0000 | 1.0249 | 0.0050 | 0.4149 | 0.0000 | 0.0000 | 0.0000 | 0.3053 | 0.0000 | 1.0000 | 0.0000 | 0.0814 | 0.3053 | 0.5000 | 0.0000 | 0.5000 | 0.0885 | 0.8033 |
| 0.0000 | 0.0132 | 1.4079 | 0.0816 | 0.4211 | 0.0263 | 0.0000 | 0.0000 | 0.6821 | 0.0887 | 1.0000 | 0.0000 | 0.0887 | 0.7708 | 0.4868 | 0.0000 | 0.5132 | 0.0554 | 0.4834 |
| 0.0000 | 0.0625 | 1.7692 | 0.1538 | 0.4688 | 0.0938 | 0.0000 | 0.0000 | 0.6088 | 0.2076 | 1.0000 | 0.0000 | 0.0559 | 0.8164 | 0.3594 | 0.0000 | 0.6406 | 0.0786 | 0.6517 |
| 0.0000 | 0.0000 | 1.0698 | 0.0140 | 0.1628 | 0.0000 | 0.0000 | 0.0000 | 0.3086 | 0.5580 | 1.0000 | 0.0000 | 0.0272 | 0.8667 | 0.4961 | 0.0078 | 0.4961 | 0.0328 | 0.7280 |
| 0.0000 | 0.1026 | 1.2650 | 0.0530 | 0.1111 | 0.0000 | 0.0000 | 0.0000 | 0.3873 | 0.3724 | 1.0000 | 0.0000 | 0.0223 | 0.7598 | 0.5000 | 0.0000 | 0.5000 | 0.1272 | 0.9209 |
| 0.0000 | 0.0000 | 1.0000 | 0.0000 | 0.0410 | 0.0000 | 0.0000 | 0.0000 | 0.4586 | 0.1623 | 1.0000 | 0.0000 | 0.0353 | 0.6208 | 0.5000 | 0.0000 | 0.5000 | 0.1538 | 0.9623 |
| 0.0000 | 0.0283 | 1.7075 | 0.1415 | 0.7453 | 0.0000 | 0.0000 | 0.0000 | 0.0030 | 0.3642 | 1.0000 | 0.0000 | 0.0000 | 0.3672 | 0.5000 | 0.0000 | 0.5000 | 0.0815 | 0.8816 |
| 0.0000 | 0.0118 | 1.5740 | 0.1148 | 0.0828 | 0.0059 | 0.0000 | 0.0000 | 0.6265 | 0.0428 | 1.0000 | 0.0000 | 0.0019 | 0.6693 | 0.4970 | 0.0000 | 0.5030 | 0.0831 | 0.6933 |
| 0.0000 | 0.0000 | 1.6258 | 0.1252 | 0.4645 | 0.0194 | 0.0000 | 0.0000 | 0.6751 | 0.0000 | 1.0000 | 0.0000 | 0.0000 | 0.6751 | 0.4323 | 0.0000 | 0.5677 | 0.1046 | 0.8187 |
| 0.0000 | 0.0000 | 1.2980 | 0.0596 | 0.1457 | 0.0066 | 0.0000 | 0.0000 | 0.5914 | 0.1269 | 1.0000 | 0.0000 | 0.0000 | 0.7183 | 0.4967 | 0.0000 | 0.5033 | 0.1129 | 0.9190 |
| 0.0000 | 0.0923 | 2.0923 | 0.2185 | 0.3385 | 0.0923 | 0.0000 | 0.0000 | 0.5624 | 0.2917 | 1.0000 | 0.0000 | 0.0405 | 0.8541 | 0.4538 | 0.0000 | 0.5462 | 0.0916 | 0.5567 |
| 0.0000 | 0.0536 | 1.5714 | 0.1143 | 0.1786 | 0.0536 | 0.0000 | 0.0000 | 0.6209 | 0.1830 | 1.0000 | 0.0000 | 0.0131 | 0.8039 | 0.4732 | 0.0000 | 0.5268 | 0.1288 | 0.7808 |
| 0.0000 | 0.0000 | 1.2667 | 0.0533 | 0.0667 | 0.0000 | 0.0000 | 0.0000 | 0.6261 | 0.0547 | 1.0000 | 0.0000 | 0.0912 | 0.6809 | 0.5000 | 0.0000 | 0.5000 | 0.0729 | 0.8096 |
| 0.0000 | 0.0496 | 1.0496 | 0.0099 | 0.0826 | 0.0000 | 0.0000 | 0.3058 | 0.0033 | 0.0066 | 1.0000 | 0.0000 | 0.1967 | 0.0098 | 0.5000 | 0.0000 | 0.5000 | 0.1361 | 0.8737 |
| 0.0000 | 0.0338 | 1.0743 | 0.0149 | 0.0608 | 0.0000 | 0.0000 | 0.4730 | 0.0000 | 0.0022 | 1.0000 | 0.0000 | 0.3326 | 0.0022 | 0.5000 | 0.0000 | 0.5000 | 0.1249 | 0.9113 |
| 0.0000 | 0.0043 | 1.3262 | 0.0652 | 0.1674 | 0.0000 | 0.0000 | 0.0043 | 0.0065 | 0.0357 | 1.0000 | 0.0000 | 0.1948 | 0.0487 | 0.4993 | 0.0014 | 0.4993 | 0.1252 | 0.7825 |
| 0.0166 | 0.0331 | 1.3675 | 0.0735 | 0.3046 | 0.0000 | 0.0000 | 0.3709 | 0.1592 | 0.0124 | 1.0000 | 0.0000 | 0.0721 | 0.1766 | 0.5000 | 0.0000 | 0.5000 | 0.1221 | 0.9088 |
| 0.0000 | 0.0106 | 1.0563 | 0.0113 | 0.0246 | 0.0000 | 0.0000 | 0.1268 | 0.4351 | 0.0456 | 1.0000 | 0.0000 | 0.0211 | 0.4807 | 0.5000 | 0.0000 | 0.5000 | 0.1106 | 0.6920 |
| 0.0188 | 0.0875 | 1.2813 | 0.0563 | 0.1000 | 0.0000 | 0.0000 | 0.3375 | 0.2131 | 0.1025 | 1.0000 | 0.0000 | 0.0246 | 0.3197 | 0.5000 | 0.0000 | 0.5000 | 0.0909 | 0.7415 |
| 0.0000 | 0.0000 | 1.0294 | 0.0059 | 0.1709 | 0.0063 | 0.0000 | 0.0000 | 0.5665 | 0.0760 | 1.0000 | 0.0000 | 0.0570 | 0.6426 | 0.4272 | 0.0000 | 0.5728 | 0.1292 | 0.9005 |
| 0.0000 | 0.0345 | 1.1034 | 0.0207 | 0.1552 | 0.0000 | 0.0000 | 0.0000 | 0.2011 | 0.5605 | 1.0000 | 0.0000 | 0.0223 | 0.7616 | 0.5000 | 0.0000 | 0.5000 | 0.0608 | 0.4418 |
| 0.0000 | 0.1373 | 1.6863 | 0.1373 | 0.1569 | 0.0784 | 0.0000 | 0.0000 | 0.4825 | 0.2588 | 1.0000 | 0.0000 | 0.1595 | 0.7412 | 0.4608 | 0.0000 | 0.5392 | 0.1372 | 0.6639 |
| 0.0000 | 0.0000 | 1.7714 | 0.1543 | 0.1571 | 0.0143 | 0.0286 | 0.0000 | 0.5984 | 0.0328 | 1.0000 | 0.0000 | 0.0820 | 0.6311 | 0.4929 | 0.0000 | 0.5071 | 0.0578 | 0.6338 |
| 0.0000 | 0.0645 | 1.4355 | 0.0871 | 0.0968 | 0.0081 | 0.1129 | 0.0000 | 0.4269 | 0.2569 | 1.0000 | 0.0000 | 0.0711 | 0.6838 | 0.4960 | 0.0000 | 0.5040 | 0.1190 | 0.8422 |
| 0.0000 | 0.0000 | 1.6567 | 0.1313 | 0.3562 | 0.0000 | 0.0000 | 0.0000 | 0.4761 | 0.0765 | 1.0000 | 0.0000 | 0.0019 | 0.5526 | 0.5000 | 0.0000 | 0.5000 | 0.0710 | 0.4680 |
| 0.0000 | 0.0000 | 1.6175 | 0.1235 | 0.0109 | 0.0273 | 0.1749 | 0.0000 | 0.4917 | 0.1569 | 1.0000 | 0.0000 | 0.0754 | 0.6486 | 0.4863 | 0.0000 | 0.5137 | 0.1122 | 0.8608 |
| 0.0000 | 0.0323 | 1.6452 | 0.1290 | 0.6452 | 0.0000 | 0.0000 | 0.0000 | 0.3753 | 0.5481 | 1.0000 | 0.0000 | 0.0000 | 0.9235 | 0.5000 | 0.0000 | 0.5000 | 0.0719 | 0.5863 |
| 0.0000 | 0.1563 | 1.1667 | 0.0333 | 0.0313 | 0.0000 | 0.0000 | 0.4167 | 0.0256 | 0.0000 | 1.0000 | 0.0000 | 0.1538 | 0.0256 | 0.5000 | 0.0000 | 0.5000 | 0.1412 | 0.8835 |
| 0.0039 | 0.2383 | 1.2573 | 0.0515 | 0.0977 | 0.0000 | 0.0000 | 0.4297 | 0.3935 | 0.0116 | 1.0000 | 0.0000 | 0.0023 | 0.4051 | 0.4707 | 0.0000 | 0.5293 | 0.1156 | 0.7368 |
| 0.0118 | 0.2941 | 1.6824 | 0.1365 | 0.1882 | 0.0000 | 0.0000 | 0.0000 | 0.0000 | 0.0045 | 1.0000 | 0.0000 | 0.0447 | 0.0045 | 0.5000 | 0.0000 | 0.5000 | 0.1214 | 0.7280 |
| 0.0000 | 0.0000 | 3.6374 | 0.5275 | 0.0000 | 0.0000 | 0.8791 | 0.0000 | 0.2436 | 0.0272 | 1.0000 | 0.0000 | 0.0000 | 0.2708 | 0.5000 | 0.0000 | 0.5000 | 0.0410 | 0.2942 |
| 0.0000 | 0.0806 | 2.5645 | 0.3129 | 0.0323 | 0.0000 | 0.4839 | 0.0000 | 0.6113 | 0.1972 | 1.0000 | 0.0000 | 0.0169 | 0.8085 | 0.5000 | 0.0000 | 0.5000 | 0.1003 | 0.4233 |
| 0.0000 | 0.0504 | 1.1864 | 0.0373 | 0.0756 | 0.0000 | 0.0000 | 0.8403 | 0.0000 | 0.0000 | 1.0000 | 0.0000 | 0.0000 | 0.0000 | 0.4958 | 0.0000 | 0.5042 | 0.0436 | 0.9284 |
| 0.0105 | 0.0105 | 1.1090 | 0.0218 | 0.0419 | 0.0000 | 0.0000 | 0.9434 | 0.0000 | 0.0104 | 1.0000 | 0.0000 | 0.0000 | 0.0104 | 0.5000 | 0.0000 | 0.5000 | 0.0162 | 0.9728 |
| 0.0165 | 0.1153 | 1.1384 | 0.0277 | 0.0033 | 0.0000 | 0.0000 | 0.7414 | 0.3308 | 0.0275 | 1.0000 | 0.0000 | 0.1203 | 0.3582 | 0.5000 | 0.0000 | 0.5000 | 0.0682 | 0.9304 |
| 0.0000 | 0.0000 | 1.1436 | 0.0287 | 0.0552 | 0.0276 | 0.0000 | 0.0000 | 0.4491 | 0.1098 | 1.0000 | 0.0000 | 0.0798 | 0.5589 | 0.4862 | 0.0000 | 0.5138 | 0.0707 | 0.9699 |
| 0.0000 | 0.0000 | 3.5606 | 0.5121 | 0.0000 | 0.0455 | 0.8333 | 0.0000 | 0.5188 | 0.0344 | 1.0000 | 0.0000 | 0.0344 | 0.5531 | 0.4962 | 0.0000 | 0.5038 | 0.0443 | 0.2641 |
| 0.0915 | 0.1831 | 1.4167 | 0.0833 | 0.0114 | 0.0046 | 0.0686 | 0.7323 | 0.0000 | 0.0046 | 1.0000 | 0.0000 | 0.0000 | 0.0046 | 0.5263 | 0.0000 | 0.4737 | 0.0465 | 0.9431 |
| 0.0028 | 0.1484 | 1.1802 | 0.0360 | 0.0139 | 0.0028 | 0.0069 | 0.8322 | 0.0000 | 0.2938 | 1.0000 | 0.0000 | 0.0000 | 0.2938 | 0.4940 | 0.0023 | 0.5037 | 0.0454 | 0.9149 |

| | | | | | | | | | | | | | | | | | | |
|---|---|---|---|---|---|---|---|---|---|---|---|---|---|---|---|---|---|---|
| 0.0000 | 0.4951 | 2.6109 | 0.3222 | 0.0279 | 0.0028 | 0.5718 | 0.1116 | 0.0000 | 0.0000 | 1.0000 | 0.0000 | 0.0000 | 0.0000 | 0.4172 | 0.1627 | 0.4200 | 0.1289 | 0.9725 |
| 0.0031 | 0.7825 | 3.4335 | 0.4867 | 0.0266 | 0.0344 | 0.8138 | 0.0000 | 0.0000 | 0.0533 | 1.0000 | 0.0000 | 0.0000 | 0.0533 | 0.3691 | 0.2619 | 0.3691 | 0.0674 | 0.9660 |
| 0.0038 | 0.0000 | 3.6322 | 0.5264 | 0.0958 | 0.0038 | 0.8812 | 0.0000 | 0.0429 | 0.0250 | 1.0000 | 0.0000 | 0.0000 | 0.0679 | 0.4981 | 0.0000 | 0.5019 | 0.0316 | 0.2451 |
| 0.1037 | 0.2519 | 2.7273 | 0.3455 | 0.0519 | 0.1111 | 0.6074 | 0.0222 | 0.0000 | 0.1046 | 1.0000 | 0.0000 | 0.0131 | 0.1046 | 0.3963 | 0.0741 | 0.5296 | 0.1515 | 0.8454 |
| 0.0334 | 0.4174 | 3.1943 | 0.4389 | 0.0000 | 0.0451 | 0.9516 | 0.0033 | 0.0000 | 0.1594 | 1.0000 | 0.0000 | 0.0028 | 0.1594 | 0.4093 | 0.1396 | 0.4511 | 0.1235 | 0.9665 |
| 0.0605 | 0.2847 | 2.6262 | 0.3252 | 0.2918 | 0.1388 | 0.3559 | 0.2135 | 0.1235 | 0.1827 | 1.0000 | 0.0000 | 0.0000 | 0.3062 | 0.4211 | 0.1329 | 0.4460 | 0.0975 | 0.8175 |
| 0.0249 | 0.6219 | 3.3682 | 0.4736 | 0.0423 | 0.0871 | 0.7711 | 0.0000 | 0.0000 | 0.1373 | 1.0000 | 0.0000 | 0.0000 | 0.1373 | 0.3839 | 0.2073 | 0.4088 | 0.1132 | 0.9465 |
| 0.0208 | 0.0433 | 3.5026 | 0.5005 | 0.0433 | 0.0468 | 0.8925 | 0.0000 | 0.0000 | 0.0236 | 1.0000 | 0.0000 | 0.0000 | 0.0270 | 0.4910 | 0.0144 | 0.4945 | 0.1051 | 0.6772 |
| 0.0000 | 0.0000 | 2.5909 | 0.3182 | 0.8462 | 0.0000 | 0.0000 | 0.0769 | 0.0000 | 0.2800 | 0.9231 | 0.0000 | 0.0200 | 0.2867 | 0.4722 | 0.0556 | 0.4722 | 0.0598 | 0.7868 |
| 0.0000 | 0.0272 | 1.4422 | 0.0884 | 0.2109 | 0.0136 | 0.0272 | 0.0000 | 0.7232 | 0.0000 | 1.0000 | 0.0000 | 0.0000 | 0.7232 | 0.4887 | 0.0091 | 0.5023 | 0.1062 | 0.8709 |
| 0.0099 | 0.0495 | 1.6436 | 0.1287 | 0.2673 | 0.0000 | 0.0396 | 0.0396 | 0.7747 | 0.0257 | 1.0000 | 0.0000 | 0.0000 | 0.8004 | 0.4967 | 0.0066 | 0.4967 | 0.1003 | 0.3920 |
| 0.0241 | 0.0281 | 2.5265 | 0.3053 | 0.0201 | 0.0000 | 0.4819 | 0.0000 | 0.0365 | 0.3125 | 1.0000 | 0.0000 | 0.0026 | 0.3490 | 0.4920 | 0.0000 | 0.5080 | 0.1038 | 0.7136 |
| 0.0000 | 0.0410 | 1.3115 | 0.0623 | 0.2705 | 0.0000 | 0.0000 | 0.4262 | 0.5765 | 0.0195 | 1.0000 | 0.0000 | 0.0000 | 0.6026 | 0.5000 | 0.0000 | 0.5000 | 0.1297 | 0.8801 |
| 0.2288 | 0.2373 | 1.9516 | 0.1903 | 0.4915 | 0.0000 | 0.0000 | 0.1017 | 0.1837 | 0.0714 | 1.0000 | 0.0000 | 0.1327 | 0.2653 | 0.2627 | 0.0000 | 0.7373 | 0.1080 | 0.4701 |
| 0.0000 | 0.2609 | 1.9617 | 0.1923 | 0.3533 | 0.0000 | 0.0000 | 0.0000 | 0.0000 | 0.0922 | 0.9946 | 0.0000 | 0.0146 | 0.0922 | 0.5000 | 0.0000 | 0.5000 | 0.1569 | 0.9167 |
| 0.0192 | 0.1154 | 1.4103 | 0.0821 | 0.2436 | 0.0192 | 0.0000 | 0.0000 | 0.0000 | 0.1731 | 0.9808 | 0.0192 | 0.0000 | 0.2500 | 0.5000 | 0.0096 | 0.4904 | 0.0489 | 0.6700 |
| 0.0265 | 0.2914 | 1.3333 | 0.0667 | 0.7020 | 0.0000 | 0.0000 | 0.0000 | 0.1029 | 0.0229 | 0.9934 | 0.0000 | 0.0114 | 0.1257 | 0.5000 | 0.0000 | 0.5000 | 0.1380 | 0.9691 |
| NA | NA | NA | NA | NA | NA | NA | NA | 0.0000 | 0.4217 | NA | NA | 0.5783 | 0.4217 | NA | NA | NA | NA | NA |
| 0.0879 | 0.0879 | 1.5484 | 0.1097 | 0.6703 | 0.0000 | 0.0000 | 0.0000 | 0.7712 | 0.0233 | 1.0000 | 0.0000 | 0.0127 | 0.7945 | 0.1703 | 0.0000 | 0.8297 | 0.0732 | 0.9481 |
| 0.8792 | 0.9758 | 2.8792 | 0.3758 | 0.0242 | 0.0000 | 0.0000 | 0.0000 | 0.0000 | 0.0326 | 1.0000 | 0.0000 | 0.0047 | 0.0326 | 0.5000 | 0.0000 | 0.5000 | 0.0424 | 0.9867 |
| 0.4444 | 0.4444 | 2.5000 | 0.3000 | 0.4444 | 0.0000 | 0.0000 | 0.0000 | 0.0000 | 0.8514 | 0.8889 | 0.0000 | 0.0270 | 0.8514 | 0.5000 | 0.0000 | 0.5000 | 0.0834 | NA |
| 0.0000 | 0.0000 | NA | NA | 0.0000 | 0.0000 | 0.0000 | 0.0000 | 0.0000 | 0.6316 | 0.0000 | 0.0000 | 0.1053 | 0.8421 | NA | NA | NA | NA | NA |
| NA | NA | NA | NA | NA | NA | NA | NA | 0.0000 | 0.9706 | NA | NA | 0.0000 | 1.0000 | NA | NA | NA | NA | NA |
| 0.0000 | 0.0442 | 1.3578 | 0.0716 | 0.1593 | 0.0000 | 0.0000 | 0.7611 | 0.0000 | 0.3843 | 0.9646 | 0.0000 | 0.0926 | 0.3843 | 0.5000 | 0.0000 | 0.5000 | 0.0581 | 0.9233 |
| 0.0000 | 0.0000 | 1.0000 | 0.0000 | 0.0000 | 0.0000 | 0.0000 | 0.9697 | 0.0000 | 0.0556 | 1.0000 | 0.0000 | 0.0139 | 0.0694 | 0.5000 | 0.0000 | 0.5000 | 0.0143 | NA |
| 0.0000 | 0.1622 | 1.3514 | 0.0703 | 0.0811 | 0.0000 | 0.0000 | 0.0000 | 0.9138 | 0.0000 | 1.0000 | 0.0000 | 0.0000 | 0.9138 | 0.5000 | 0.0000 | 0.5000 | 0.1281 | 0.6961 |
| NA | NA | NA | NA | NA | NA | NA | NA | 0.0000 | 0.1176 | NA | NA | 0.8627 | 0.1373 | NA | NA | NA | NA | NA |
| 0.0000 | 0.0000 | NA | NA | 0.0000 | 0.0000 | 0.0000 | 0.0000 | 0.0000 | 0.8846 | 0.0000 | 0.0000 | 0.0385 | 0.9231 | NA | NA | NA | NA | NA |
| 0.0379 | 0.0417 | 1.0871 | 0.0174 | 0.0076 | 0.0000 | 0.0000 | 0.9470 | 0.0000 | 0.0754 | 1.0000 | 0.0000 | 0.1594 | 0.0754 | 0.5000 | 0.0000 | 0.5000 | 0.0109 | 0.9825 |
| 0.0554 | 0.2030 | 1.2952 | 0.0590 | 0.0369 | 0.0000 | 0.0000 | 0.7380 | 0.3529 | 0.0395 | 1.0000 | 0.0000 | 0.1317 | 0.3924 | 0.5000 | 0.0000 | 0.5000 | 0.0575 | 0.8859 |
| 0.0000 | 0.8989 | 1.9762 | 0.1952 | 0.0787 | 0.0000 | 0.0000 | 0.0000 | 0.0707 | 0.8245 | 1.0000 | 0.0000 | 0.0000 | 0.8952 | 0.4719 | 0.0000 | 0.5281 | 0.0331 | 0.9692 |
| 0.0000 | 0.0000 | 1.4333 | 0.0867 | 0.2667 | 0.0000 | 0.0000 | 0.0000 | 0.1061 | 0.0557 | 1.0000 | 0.0000 | 0.0371 | 0.1671 | 0.5000 | 0.0000 | 0.5000 | 0.0226 | 0.3335 |
| 0.0000 | 0.0000 | 3.0000 | 0.4000 | 1.0000 | 0.0000 | 0.0000 | 0.0000 | 0.0000 | 0.0512 | 1.0000 | 0.0000 | 0.2419 | 0.0605 | 0.5000 | 0.0000 | 0.5000 | 0.0000 | NA |
| 0.0000 | 0.9091 | 2.0341 | 0.2068 | 0.0682 | 0.0000 | 0.0000 | 0.0114 | 0.0000 | 0.0112 | 1.0000 | 0.0000 | 0.0000 | 0.0112 | 0.5000 | 0.0000 | 0.5000 | 0.0274 | 0.2902 |
| 0.2030 | 0.4061 | 2.0152 | 0.2030 | 0.1878 | 0.0000 | 0.0000 | 0.0000 | 0.3504 | 0.0916 | 1.0000 | 0.0000 | 0.0270 | 0.4420 | 0.5000 | 0.0000 | 0.5000 | 0.1585 | 0.7850 |
| 0.0000 | 0.0000 | 1.0698 | 0.0140 | 0.1099 | 0.0000 | 0.0000 | 0.8242 | 0.0000 | 0.0648 | 1.0000 | 0.0000 | 0.0926 | 0.0648 | 0.4725 | 0.0000 | 0.5275 | 0.0437 | 0.9183 |
| 0.0152 | 0.0152 | 1.7121 | 0.1424 | 0.3788 | 0.0000 | 0.0000 | 0.0758 | 0.7953 | 0.0504 | 1.0000 | 0.0000 | 0.0129 | 0.8457 | 0.5000 | 0.0000 | 0.5000 | 0.0370 | 0.5424 |
| 0.0000 | 0.0000 | 1.6250 | 0.1250 | 0.5625 | 0.0000 | 0.1875 | 0.0000 | 0.4739 | 0.0047 | 1.0000 | 0.0000 | 0.1422 | 0.4787 | 0.5000 | 0.0000 | 0.5000 | 0.1277 | 0.7170 |
| 0.0556 | 0.1111 | 1.2000 | 0.0400 | 0.1667 | 0.0000 | 0.0000 | 0.7222 | 0.0000 | 0.8154 | 1.0000 | 0.0000 | 0.0000 | 0.8174 | 0.4167 | 0.0000 | 0.5833 | 0.0413 | 0.8764 |
| 0.2206 | 0.2647 | 1.6618 | 0.1324 | 0.0147 | 0.0000 | 0.0735 | 0.5882 | 0.2816 | 0.0870 | 1.0000 | 0.0000 | 0.0512 | 0.3686 | 0.5000 | 0.0000 | 0.5000 | 0.0786 | 0.8453 |
| 0.0000 | 0.0000 | 1.1653 | 0.0331 | 0.0000 | 0.0000 | 0.0826 | 0.9091 | 0.0000 | 0.0472 | 1.0000 | 0.0000 | 0.0000 | 0.0472 | 0.5000 | 0.0000 | 0.5000 | 0.0388 | 0.9637 |
| 0.0248 | 0.1733 | 1.2525 | 0.0505 | 0.0000 | 0.0000 | 0.0248 | 0.7426 | 0.3814 | 0.0060 | 1.0000 | 0.0000 | 0.0060 | 0.3874 | 0.5000 | 0.0000 | 0.5000 | 0.0618 | 0.9078 |
| 0.0385 | 0.6795 | 2.0897 | 0.2179 | 0.0000 | 0.0000 | 0.1795 | 0.0000 | 0.0031 | 0.3882 | 1.0000 | 0.0000 | 0.0031 | 0.3913 | 0.5000 | 0.0000 | 0.5000 | 0.1244 | 0.9352 |
| 0.0000 | 0.0500 | 1.4688 | 0.0938 | 0.3750 | 0.0000 | 0.0750 | 0.5000 | 0.0000 | 0.0000 | 1.0000 | 0.0000 | 0.0000 | 0.0000 | 0.4000 | 0.0000 | 0.6000 | 0.0919 | 0.7731 |
| 0.0000 | 0.0222 | 1.4222 | 0.0844 | 0.0000 | 0.0000 | 0.2000 | 0.7778 | 0.0000 | 0.0044 | 1.0000 | 0.0000 | 0.0044 | 0.0044 | 0.5000 | 0.0000 | 0.5000 | 0.0778 | 0.9794 |
| 0.0074 | 0.0074 | 1.1979 | 0.0396 | 0.0148 | 0.0000 | 0.0916 | 0.8863 | 0.0000 | 0.0000 | 1.0000 | 0.0000 | 0.0000 | 0.0000 | 0.4926 | 0.0000 | 0.5074 | 0.0405 | 0.9838 |
| 0.0035 | 0.0121 | 1.0849 | 0.0170 | 0.0173 | 0.0000 | 0.0173 | 0.9532 | 0.0000 | 0.0000 | 1.0000 | 0.0000 | 0.0000 | 0.0000 | 0.5000 | 0.0000 | 0.5000 | 0.0145 | 0.9827 |
| 0.0000 | 0.1667 | 2.8333 | 0.3667 | 0.8333 | 0.0000 | 0.0000 | 0.0000 | 0.0000 | 0.4194 | 1.0000 | 0.0000 | 0.0806 | 0.4355 | 0.5000 | 0.0000 | 0.5000 | 0.0329 | NA |
| 0.0105 | 0.6947 | 4.4681 | 0.6936 | 0.0105 | 0.3053 | 0.6842 | 0.0000 | 0.0000 | 0.0101 | 1.0000 | 0.0000 | 0.0303 | 0.0101 | 0.7930 | 0.0351 | 0.1719 | 0.1050 | 0.4682 |
| 0.0299 | 0.7164 | 4.0909 | 0.6182 | 0.0000 | 0.1343 | 0.8657 | 0.0000 | 0.0000 | 0.0143 | 1.0000 | 0.0000 | 0.0286 | 0.0143 | 0.4353 | 0.2338 | 0.3308 | 0.0821 | 0.7062 |
| 0.0104 | 0.5729 | 3.8723 | 0.5745 | 0.0417 | 0.1354 | 0.8229 | 0.0000 | 0.0000 | 0.0101 | 1.0000 | 0.0000 | 0.0202 | 0.0101 | 0.4333 | 0.1754 | 0.3912 | 0.0997 | 0.8502 |

| | | | | | | | | | | | | | | | | | | |
|---|---|---|---|---|---|---|---|---|---|---|---|---|---|---|---|---|---|---|
| 0.0120 | 0.1198 | 2.6928 | 0.3386 | 0.0060 | 0.2036 | 0.7844 | 0.0060 | 0.0057 | 0.0230 | 1.0000 | 0.0000 | 0.0057 | 0.0345 | 0.5200 | 0.0140 | 0.4661 | 0.0793 | 0.9126 |
| 0.0247 | 0.2963 | 3.5688 | 0.5138 | 0.0062 | 0.6420 | 0.3395 | 0.0062 | 0.0000 | 0.0181 | 1.0000 | 0.0000 | 0.0000 | 0.0241 | 0.5186 | 0.0404 | 0.4410 | 0.1056 | 0.8713 |
| 0.2137 | 0.3740 | 3.5120 | 0.5024 | 0.0076 | 0.5878 | 0.3969 | 0.0076 | 0.0000 | 0.0219 | 1.0000 | 0.0000 | 0.0073 | 0.0365 | 0.4782 | 0.1244 | 0.3974 | 0.1087 | 0.8496 |
| 0.0258 | 0.0644 | 2.4105 | 0.2821 | 0.0086 | 0.9270 | 0.0558 | 0.0043 | 0.0042 | 0.0042 | 0.9957 | 0.0043 | 0.0042 | 0.0127 | 0.1190 | 0.0303 | 0.8506 | 0.0628 | 0.6765 |
| 0.0707 | 0.5455 | 4.1875 | 0.6375 | 0.0101 | 0.3333 | 0.6364 | 0.0000 | 0.0096 | 0.0192 | 1.0000 | 0.0000 | 0.0096 | 0.0385 | 0.7285 | 0.0739 | 0.1976 | 0.1229 | 0.7010 |
| 0.0377 | 0.0460 | 2.4473 | 0.2895 | 0.0042 | 0.8243 | 0.1590 | 0.0000 | 0.0041 | 0.0000 | 1.0000 | 0.0000 | 0.0083 | 0.0041 | 0.1387 | 0.0168 | 0.8445 | 0.0752 | 0.7026 |
| 0.0128 | 0.1795 | 3.0439 | 0.4088 | 0.0000 | 0.6880 | 0.3034 | 0.0043 | 0.0000 | 0.0084 | 1.0000 | 0.0000 | 0.0042 | 0.0126 | 0.3598 | 0.0186 | 0.6216 | 0.1186 | 0.5632 |
| 0.0151 | 0.5477 | 4.1005 | 0.6201 | 0.0000 | 0.5226 | 0.4673 | 0.0000 | 0.0049 | 0.0098 | 1.0000 | 0.0000 | 0.0098 | 0.0195 | 0.5352 | 0.0804 | 0.3844 | 0.1164 | 0.7804 |
| 0.0352 | 0.1709 | 3.7188 | 0.5438 | 0.0000 | 0.1960 | 0.7789 | 0.0050 | 0.0000 | 0.0000 | 1.0000 | 0.0000 | 0.0100 | 0.0000 | 0.5524 | 0.0137 | 0.4338 | 0.1064 | 0.6744 |
| 0.0129 | 0.1595 | 3.4469 | 0.4894 | 0.0000 | 0.2371 | 0.7414 | 0.0043 | 0.0000 | 0.0000 | 1.0000 | 0.0000 | 0.0085 | 0.0000 | 0.5102 | 0.0168 | 0.4730 | 0.1046 | 0.7791 |
| 0.0678 | 0.1864 | 3.5439 | 0.5088 | 0.0085 | 0.5763 | 0.3983 | 0.0085 | 0.0000 | 0.0165 | 1.0000 | 0.0000 | 0.0083 | 0.0165 | 0.5503 | 0.0201 | 0.4296 | 0.1107 | 0.8403 |
| 0.0181 | 0.3675 | 3.8875 | 0.5775 | 0.0060 | 0.1325 | 0.8494 | 0.0000 | 0.0057 | 0.0343 | 1.0000 | 0.0000 | 0.0057 | 0.0457 | 0.4421 | 0.1159 | 0.4421 | 0.0745 | 0.5438 |
| 0.0377 | 0.3522 | 3.4204 | 0.4841 | 0.0126 | 0.4340 | 0.5346 | 0.0000 | 0.0060 | 0.0241 | 1.0000 | 0.0000 | 0.0120 | 0.0301 | 0.4388 | 0.0464 | 0.5148 | 0.1047 | 0.7683 |
| 0.0210 | 0.2185 | 3.1087 | 0.4217 | 0.0000 | 0.1849 | 0.7815 | 0.0336 | 0.0041 | 0.0164 | 1.0000 | 0.0000 | 0.0000 | 0.0246 | 0.5735 | 0.0126 | 0.4139 | 0.1258 | 0.8208 |
| 0.0094 | 0.2123 | 3.9330 | 0.5866 | 0.0000 | 0.0708 | 0.9104 | 0.0047 | 0.0045 | 0.0181 | 1.0000 | 0.0000 | 0.0136 | 0.0271 | 0.4810 | 0.0711 | 0.4479 | 0.0759 | 0.6575 |
| 0.0156 | 0.0365 | 3.9053 | 0.5811 | 0.0000 | 0.0885 | 0.9010 | 0.0052 | 0.0050 | 0.0201 | 0.9948 | 0.0052 | 0.0050 | 0.0302 | 0.5000 | 0.0156 | 0.4844 | 0.0409 | 0.5149 |
| 0.0093 | 0.2991 | 3.9853 | 0.5971 | 0.0000 | 0.0935 | 0.8645 | 0.0000 | 0.0000 | 0.0089 | 1.0000 | 0.0000 | 0.0313 | 0.0134 | 0.4593 | 0.1057 | 0.4350 | 0.0828 | 0.6506 |
| 0.0188 | 0.0798 | 3.9005 | 0.5801 | 0.0047 | 0.0423 | 0.9296 | 0.0000 | 0.0046 | 0.0046 | 0.9953 | 0.0047 | 0.0046 | 0.0138 | 0.4898 | 0.0297 | 0.4804 | 0.0363 | 0.5415 |
| 0.2404 | 0.0984 | 3.1429 | 0.4286 | 0.1366 | 0.0601 | 0.7705 | 0.0000 | 0.0053 | 0.0000 | 0.9945 | 0.0055 | 0.0107 | 0.0107 | 0.4636 | 0.0838 | 0.4526 | 0.1017 | 0.7733 |
| 0.0367 | 0.0917 | 2.7209 | 0.3442 | 0.0046 | 0.2248 | 0.7615 | 0.0000 | 0.0045 | 0.0045 | 1.0000 | 0.0000 | 0.0045 | 0.0135 | 0.5123 | 0.0123 | 0.4753 | 0.0850 | 0.9152 |
| 0.0290 | 0.5942 | 3.9343 | 0.5869 | 0.0072 | 0.0652 | 0.9130 | 0.0000 | 0.0000 | 0.0207 | 1.0000 | 0.0000 | 0.0207 | 0.0276 | 0.4070 | 0.2077 | 0.3853 | 0.0857 | 0.8741 |
| 0.0093 | 0.1589 | 3.9524 | 0.5905 | 0.0093 | 0.0280 | 0.9065 | 0.0000 | 0.0088 | 0.0263 | 1.0000 | 0.0000 | 0.0175 | 0.0439 | 0.4798 | 0.0498 | 0.4704 | 0.0544 | 0.7189 |
| 0.0253 | 0.1772 | 3.1911 | 0.4382 | 0.0000 | 0.0570 | 0.6772 | 0.0000 | 0.0060 | 0.0181 | 1.0000 | 0.0000 | 0.0181 | 0.0301 | 0.4757 | 0.0612 | 0.4631 | 0.1052 | 0.7154 |
| 0.0467 | 0.2867 | 3.7905 | 0.5581 | 0.0067 | 0.0867 | 0.8733 | 0.0000 | 0.0064 | 0.0382 | 0.9933 | 0.0067 | 0.0000 | 0.0446 | 0.4720 | 0.1029 | 0.4251 | 0.0875 | 0.7428 |
| 0.0248 | 0.0373 | 3.9304 | 0.5861 | 0.0062 | 0.0621 | 0.9130 | 0.0000 | 0.0000 | 0.0000 | 1.0000 | 0.0000 | 0.0062 | 0.0000 | 0.5042 | 0.0168 | 0.4790 | 0.0273 | 0.5300 |
| 0.0179 | 0.1012 | 3.8922 | 0.5784 | 0.0000 | 0.0357 | 0.9286 | 0.0000 | 0.0000 | 0.0174 | 0.9940 | 0.0060 | 0.0058 | 0.0174 | 0.4841 | 0.0377 | 0.4782 | 0.0390 | 0.6200 |
| 0.0216 | 0.2014 | 3.8768 | 0.5754 | 0.0072 | 0.0504 | 0.7698 | 0.0000 | 0.0137 | 0.0205 | 0.9928 | 0.0072 | 0.0068 | 0.0411 | 0.4676 | 0.0719 | 0.4604 | 0.0861 | 0.7795 |
| 0.0333 | 0.3500 | 3.8235 | 0.5647 | 0.0167 | 0.0750 | 0.8667 | 0.0000 | 0.0078 | 0.0313 | 0.9917 | 0.0083 | 0.0156 | 0.0469 | 0.4500 | 0.1250 | 0.4250 | 0.0927 | 0.7783 |
| 0.0444 | 0.3111 | 3.8837 | 0.5767 | 0.0222 | 0.4444 | 0.4889 | 0.0000 | 0.0185 | 0.0926 | 1.0000 | 0.0000 | 0.0370 | 0.1296 | 0.5114 | 0.0682 | 0.4205 | 0.1064 | 0.6700 |
| 0.1146 | 0.3438 | 3.8000 | 0.5600 | 0.0000 | 0.3646 | 0.6250 | 0.0104 | 0.0000 | 0.0000 | 1.0000 | 0.0000 | 0.0202 | 0.0101 | 0.6354 | 0.0208 | 0.3438 | 0.1235 | 0.6991 |
| 0.1085 | 0.0853 | 3.7311 | 0.5462 | 0.0078 | 0.1473 | 0.7364 | 0.0078 | 0.0072 | 0.0362 | 0.9690 | 0.0310 | 0.0145 | 0.0507 | 0.4780 | 0.0523 | 0.4697 | 0.0744 | 0.6792 |
| 0.2216 | 0.4948 | 3.5864 | 0.5173 | 0.0206 | 0.1289 | 0.8351 | 0.0000 | 0.0000 | 0.0248 | 1.0000 | 0.0000 | 0.0149 | 0.0248 | 0.4297 | 0.1510 | 0.4193 | 0.1127 | 0.9152 |
| 0.0355 | 0.6095 | 3.8024 | 0.5605 | 0.0059 | 0.0533 | 0.8521 | 0.0000 | 0.0110 | 0.0442 | 0.9941 | 0.0059 | 0.0055 | 0.0608 | 0.4018 | 0.2143 | 0.3839 | 0.0958 | 0.9095 |
| 0.0417 | 0.3177 | 3.7368 | 0.5474 | 0.0208 | 0.0469 | 0.8750 | 0.0000 | 0.0000 | 0.0000 | 1.0000 | 0.0000 | 0.0103 | 0.0000 | 0.4459 | 0.1187 | 0.4354 | 0.0919 | 0.7635 |
| 0.1923 | 0.4846 | 3.9612 | 0.5922 | 0.0077 | 0.6538 | 0.3308 | 0.0000 | 0.0000 | 0.0643 | 0.8846 | 0.1154 | 0.0000 | 0.0714 | 0.5295 | 0.0872 | 0.3833 | 0.1154 | 0.8594 |
| 0.0633 | 0.2848 | 3.9542 | 0.5908 | 0.0063 | 0.1772 | 0.7848 | 0.0000 | 0.0000 | 0.0355 | 1.0000 | 0.0000 | 0.0178 | 0.0473 | 0.4610 | 0.1169 | 0.4221 | 0.0910 | 0.6848 |
| 0.0476 | 0.2434 | 3.8449 | 0.5690 | 0.0212 | 0.0582 | 0.8889 | 0.0000 | 0.0051 | 0.0255 | 1.0000 | 0.0000 | 0.0000 | 0.0357 | 0.4628 | 0.0904 | 0.4468 | 0.0756 | 0.6874 |
| 0.0337 | 0.2360 | 3.9148 | 0.5830 | 0.0225 | 0.0449 | 0.9213 | 0.0000 | 0.0000 | 0.0000 | 1.0000 | 0.0000 | 0.0056 | 0.0000 | 0.4623 | 0.0923 | 0.4454 | 0.0695 | 0.6107 |
| 0.1183 | 0.1935 | 3.9231 | 0.5846 | 0.0108 | 0.6022 | 0.3763 | 0.0000 | 0.0000 | 0.0294 | 1.0000 | 0.0000 | 0.0294 | 0.0588 | 0.4710 | 0.1014 | 0.4275 | 0.0853 | 0.7896 |
| 0.1290 | 0.5161 | 3.8301 | 0.5660 | 0.0258 | 0.1419 | 0.8129 | 0.0000 | 0.0000 | 0.0000 | 1.0000 | 0.0000 | 0.0127 | 0.0000 | 0.4307 | 0.1840 | 0.3853 | 0.1172 | 0.5280 |
| 0.0824 | 0.1235 | 3.9455 | 0.5891 | 0.0059 | 0.1412 | 0.8235 | 0.0000 | 0.0000 | 0.0284 | 1.0000 | 0.0000 | 0.0000 | 0.0341 | 0.5080 | 0.0472 | 0.4448 | 0.0562 | 0.6485 |
| 0.0537 | 0.1141 | 3.9184 | 0.5837 | 0.0000 | 0.1812 | 0.8121 | 0.0000 | 0.0064 | 0.0255 | 1.0000 | 0.0000 | 0.0127 | 0.0382 | 0.5011 | 0.0450 | 0.4538 | 0.0728 | 0.5422 |
| 0.0406 | 0.4518 | 3.5867 | 0.5173 | 0.0051 | 0.4416 | 0.5533 | 0.0000 | 0.0000 | 0.0100 | 1.0000 | 0.0000 | 0.0000 | 0.0150 | 0.4856 | 0.0541 | 0.4602 | 0.0998 | 0.9289 |
| 0.0400 | 0.5733 | 4.3020 | 0.6604 | 0.0067 | 0.6333 | 0.3600 | 0.0000 | 0.0000 | 0.0321 | 1.0000 | 0.0000 | 0.0064 | 0.0321 | 0.5278 | 0.0844 | 0.3878 | 0.1059 | 0.7336 |
| 0.0783 | 0.1446 | 3.9939 | 0.5988 | 0.0060 | 0.1506 | 0.8313 | 0.0000 | 0.0057 | 0.0230 | 1.0000 | 0.0000 | 0.0115 | 0.0345 | 0.4949 | 0.0404 | 0.4646 | 0.0537 | 0.5895 |
| 0.0614 | 0.3333 | 4.0183 | 0.6037 | 0.0044 | 0.1491 | 0.8070 | 0.0000 | 0.0000 | 0.0254 | 1.0000 | 0.0000 | 0.0042 | 0.0297 | 0.4627 | 0.1065 | 0.4307 | 0.0865 | 0.7218 |
| 0.0588 | 0.3294 | 3.7066 | 0.5413 | 0.0000 | 0.4118 | 0.5765 | 0.0059 | 0.0000 | 0.0225 | 1.0000 | 0.0000 | 0.0169 | 0.0281 | 0.5197 | 0.0375 | 0.4428 | 0.1021 | 0.7314 |
| 0.0393 | 0.0562 | 3.7443 | 0.5489 | 0.0000 | 0.2022 | 0.7921 | 0.0000 | 0.0054 | 0.0269 | 1.0000 | 0.0000 | 0.0054 | 0.0376 | 0.5169 | 0.0113 | 0.4718 | 0.0616 | 0.5560 |
| 0.0559 | 0.5466 | 4.2278 | 0.6456 | 0.0062 | 0.4969 | 0.4907 | 0.0000 | 0.0000 | 0.0234 | 0.9938 | 0.0062 | 0.0234 | 0.0351 | 0.6468 | 0.0335 | 0.3197 | 0.1184 | 0.7768 |
| 0.0521 | 0.4427 | 4.3770 | 0.6754 | 0.0000 | 0.3333 | 0.6667 | 0.0000 | 0.0049 | 0.0294 | 1.0000 | 0.0000 | 0.0196 | 0.0392 | 0.6076 | 0.0295 | 0.3628 | 0.1030 | 0.6104 |
| 0.0902 | 0.6692 | 4.5152 | 0.7030 | 0.0075 | 0.4962 | 0.4962 | 0.0000 | 0.0000 | 0.0211 | 1.0000 | 0.0000 | 0.0282 | 0.0352 | 0.6466 | 0.0526 | 0.3008 | 0.1207 | 0.6195 |

| | | | | | | | | | | | | | | | | | | |
|---|---|---|---|---|---|---|---|---|---|---|---|---|---|---|---|---|---|---|
| 0.0496 | 0.4711 | 4.0504 | 0.6101 | 0.0000 | 0.6860 | 0.3058 | 0.0000 | 0.0000 | 0.0231 | 1.0000 | 0.0000 | 0.0308 | 0.0385 | 0.5278 | 0.0194 | 0.4528 | 0.1163 | 0.6773 |
| 0.5831 | 0.1336 | 3.9293 | 0.5859 | 0.0166 | 0.7832 | 0.1669 | 0.0000 | 0.0007 | 0.0013 | 0.9834 | 0.0000 | 0.0013 | 0.0020 | 0.3786 | 0.2427 | 0.3786 | 0.1017 | 0.5272 |
| 0.5747 | 0.1290 | 3.9054 | 0.5811 | 0.0000 | 0.7445 | 0.1698 | 0.0000 | 0.0016 | 0.0016 | 1.0000 | 0.0000 | 0.0421 | 0.0032 | 0.4257 | 0.2334 | 0.3408 | 0.1108 | 0.4854 |
| 0.1387 | 0.2769 | 3.8727 | 0.5745 | 0.0153 | 0.4463 | 0.4463 | 0.0000 | 0.0006 | 0.0006 | 1.0000 | 0.0000 | 0.0297 | 0.0012 | 0.4568 | 0.0716 | 0.4716 | 0.1057 | 0.6111 |
| 0.1595 | 0.2392 | 3.6380 | 0.5276 | 0.0000 | 0.4785 | 0.4801 | 0.0000 | 0.0032 | 0.0047 | 1.0000 | 0.0000 | 0.0000 | 0.0079 | 0.4075 | 0.1069 | 0.4856 | 0.1267 | 0.8615 |
| 0.0015 | 0.4205 | 3.5336 | 0.5067 | 0.0398 | 0.4235 | 0.5352 | 0.0000 | 0.0030 | 0.0030 | 1.0000 | 0.0000 | 0.0000 | 0.0061 | 0.4348 | 0.0907 | 0.4745 | 0.1291 | 0.8915 |
| 0.0011 | 0.2775 | 3.7214 | 0.5443 | 0.1110 | 0.6948 | 0.1942 | 0.0000 | 0.0022 | 0.0044 | 1.0000 | 0.0000 | 0.0011 | 0.0066 | 0.4258 | 0.0374 | 0.5368 | 0.1022 | 0.7898 |
| 0.0377 | 0.0746 | 2.6365 | 0.3273 | 0.4801 | 0.3161 | 0.1300 | 0.0000 | 0.0014 | 0.0014 | 0.9808 | 0.0007 | 0.0692 | 0.0027 | 0.2864 | 0.1450 | 0.5686 | 0.1338 | 0.6934 |
| 0.2287 | 0.1718 | 3.5654 | 0.5131 | 0.0569 | 0.6564 | 0.2856 | 0.0000 | 0.0011 | 0.0034 | 1.0000 | 0.0000 | 0.0000 | 0.0045 | 0.3479 | 0.0766 | 0.5755 | 0.1321 | 0.6133 |
| 0.3432 | 0.2281 | 3.6545 | 0.5309 | 0.0285 | 0.6853 | 0.1722 | 0.0000 | 0.0023 | 0.0011 | 1.0000 | 0.0000 | 0.0000 | 0.0034 | 0.4287 | 0.0855 | 0.4857 | 0.1268 | 0.5151 |
| 0.0014 | 0.1422 | 2.1110 | 0.2222 | 0.0000 | 0.1821 | 0.2845 | 0.0000 | 0.0014 | 0.0000 | 0.9986 | 0.0000 | 0.0014 | 0.0014 | 0.4404 | 0.0480 | 0.5116 | 0.1235 | 0.7949 |
| 0.0000 | 0.2496 | 3.0033 | 0.4007 | 0.0416 | 0.4176 | 0.4576 | 0.0000 | 0.0017 | 0.0050 | 0.9168 | 0.0000 | 0.0000 | 0.0066 | 0.4168 | 0.0756 | 0.5076 | 0.1649 | 0.8369 |
| 0.0280 | 0.1075 | 2.7061 | 0.3412 | 0.1097 | 0.2720 | 0.3763 | 0.0000 | 0.0011 | 0.0032 | 0.9462 | 0.0000 | 0.0021 | 0.0043 | 0.4325 | 0.0758 | 0.4917 | 0.1621 | 0.8290 |
| 0.0843 | 0.0831 | 3.0857 | 0.4171 | 0.2228 | 0.2772 | 0.5000 | 0.0000 | 0.0011 | 0.0022 | 1.0000 | 0.0000 | 0.0022 | 0.0033 | 0.3826 | 0.0587 | 0.5587 | 0.1400 | 0.7519 |
| 0.0913 | 0.1365 | 2.6932 | 0.3386 | 0.6103 | 0.2532 | 0.1365 | 0.0000 | 0.0018 | 0.0018 | 0.9548 | 0.0000 | 0.0009 | 0.0036 | 0.1338 | 0.0477 | 0.8185 | 0.0964 | 0.7767 |
| 0.0324 | 0.0623 | 2.9844 | 0.3969 | 0.0318 | 0.5464 | 0.1875 | 0.0000 | 0.0018 | 0.0018 | 1.0000 | 0.0000 | 0.0165 | 0.0037 | 0.4656 | 0.0214 | 0.5130 | 0.1177 | 0.7420 |
| 0.0000 | 0.1184 | 3.3031 | 0.4606 | 0.0846 | 0.3566 | 0.5582 | 0.0000 | 0.0020 | 0.0027 | 1.0000 | 0.0000 | 0.0000 | 0.0047 | 0.4464 | 0.0395 | 0.5141 | 0.1199 | 0.7875 |
| 0.0000 | 0.1330 | 2.5372 | 0.3074 | 0.2327 | 0.2686 | 0.2327 | 0.0000 | 0.0040 | 0.0026 | 1.0000 | 0.0000 | 0.0026 | 0.0066 | 0.4169 | 0.0665 | 0.5166 | 0.1494 | 0.8288 |
| 0.0256 | 0.0266 | 2.2579 | 0.2516 | 0.1546 | 0.1535 | 0.3081 | 0.0000 | 0.0020 | 0.0020 | 1.0000 | 0.0000 | 0.0010 | 0.0041 | 0.4524 | 0.0430 | 0.5046 | 0.1355 | 0.8338 |
| 0.0281 | 0.0141 | 1.9734 | 0.1947 | 0.4080 | 0.0996 | 0.1407 | 0.0000 | 0.0006 | 0.0011 | 1.0000 | 0.0000 | 0.0139 | 0.0017 | 0.4200 | 0.1305 | 0.4495 | 0.1503 | 0.5583 |
| 0.0256 | 0.7669 | 3.0529 | 0.4106 | 0.0010 | 0.1789 | 0.0266 | 0.0000 | 0.0009 | 0.0028 | 0.9990 | 0.0000 | 0.0941 | 0.0037 | 0.5212 | 0.0089 | 0.4700 | 0.0536 | 0.4005 |
| 0.0010 | 0.1996 | 3.1907 | 0.4381 | 0.1008 | 0.0010 | 0.3992 | 0.0000 | 0.0009 | 0.0009 | 1.0000 | 0.0000 | 0.0483 | 0.0019 | 0.4993 | 0.0003 | 0.5003 | 0.0949 | 0.7940 |
| 0.0182 | 0.1636 | 3.4510 | 0.4902 | 0.1091 | 0.1636 | 0.1636 | 0.0000 | 0.0015 | 0.0007 | 1.0000 | 0.0000 | 0.0000 | 0.0022 | 0.4909 | 0.0182 | 0.4909 | 0.1117 | 0.8396 |
| 0.0508 | 0.0339 | 2.7622 | 0.3524 | 0.0346 | 0.0847 | 0.3218 | 0.0000 | 0.0020 | 0.0007 | 1.0000 | 0.0000 | 0.0000 | 0.0027 | 0.4887 | 0.0226 | 0.4887 | 0.1109 | 0.7829 |
| 0.0018 | 0.2712 | 3.5712 | 0.5142 | 0.0470 | 0.0922 | 0.1374 | 0.0000 | 0.0023 | 0.0011 | 1.0000 | 0.0000 | 0.3710 | 0.0034 | 0.4922 | 0.0157 | 0.4922 | 0.1121 | 0.7978 |
| 0.0346 | 0.1011 | 3.2715 | 0.4543 | 0.2660 | 0.0678 | 0.3338 | 0.0000 | 0.0038 | 0.0013 | 1.0000 | 0.0000 | 0.0332 | 0.0051 | 0.4774 | 0.0452 | 0.4774 | 0.1098 | 0.8449 |
| 0.1673 | 0.0677 | 3.0302 | 0.4060 | 0.1660 | 0.2337 | 0.1992 | 0.0000 | 0.0026 | 0.0013 | 1.0000 | 0.0000 | 0.0000 | 0.0040 | 0.4719 | 0.0562 | 0.4719 | 0.1310 | 0.8024 |
| 0.0500 | 0.0750 | 3.6250 | 0.5250 | 0.0500 | 0.0750 | 0.2750 | 0.0000 | 0.0020 | 0.0010 | 1.0000 | 0.0000 | 0.0010 | 0.0030 | 0.4792 | 0.0417 | 0.4792 | 0.1141 | 0.7355 |
| 0.1262 | 0.3713 | 3.9901 | 0.5980 | 0.1238 | 0.3812 | 0.1238 | 0.0000 | 0.0022 | 0.0000 | 0.9975 | 0.0025 | 0.1135 | 0.0044 | 0.4579 | 0.0842 | 0.4579 | 0.1345 | 0.7918 |
| 0.0581 | 0.0581 | 3.7984 | 0.5597 | 0.2278 | 0.1162 | 0.1708 | 0.0000 | 0.0011 | 0.0011 | 1.0000 | 0.0000 | 0.0011 | 0.0023 | 0.4617 | 0.0767 | 0.4617 | 0.1258 | 0.8059 |
| 0.0636 | 0.0948 | 4.0342 | 0.6068 | 0.2494 | 0.1259 | 0.0935 | 0.0000 | 0.0012 | 0.0037 | 1.0000 | 0.0000 | 0.0012 | 0.0050 | 0.4738 | 0.0524 | 0.4738 | 0.1066 | 0.8308 |
| 0.0000 | 0.1467 | 4.4095 | 0.6819 | 0.0029 | 0.0486 | 0.1953 | 0.0000 | 0.0010 | 0.0000 | 1.0000 | 0.0000 | 0.0010 | 0.0010 | 0.4992 | 0.0006 | 0.5002 | 0.0843 | 0.7110 |
| 0.0754 | 0.1109 | 3.7340 | 0.5468 | 0.0370 | 0.1309 | 0.7212 | 0.0000 | 0.0007 | 0.0015 | 0.9630 | 0.0370 | 0.0022 | 0.0022 | 0.4845 | 0.0496 | 0.4660 | 0.0668 | 0.5233 |
| 0.0356 | 0.0712 | 2.8907 | 0.3781 | 0.1425 | 0.4302 | 0.3561 | 0.0000 | 0.0045 | 0.0045 | 1.0000 | 0.0000 | 0.3600 | 0.0099 | 0.4644 | 0.0356 | 0.5000 | 0.1417 | 0.6663 |
| 0.1473 | 0.1416 | 3.4221 | 0.4844 | 0.1445 | 0.1473 | 0.1416 | 0.0000 | 0.0022 | 0.0044 | 0.9972 | 0.0028 | 0.2210 | 0.0066 | 0.4509 | 0.0954 | 0.4537 | 0.1287 | 0.7969 |
| 0.1873 | 0.0637 | 3.1873 | 0.4375 | 0.2497 | 0.2497 | 0.3121 | 0.0000 | 0.0000 | 0.0000 | 1.0000 | 0.0000 | 0.0000 | 0.0000 | 0.4480 | 0.1040 | 0.4480 | 0.1189 | 0.6236 |
| 0.1544 | 0.1544 | 2.9234 | 0.3847 | 0.3058 | 0.2324 | 0.3058 | 0.0000 | 0.0030 | 0.0061 | 1.0000 | 0.0000 | 0.0015 | 0.0091 | 0.4365 | 0.1284 | 0.4350 | 0.1311 | 0.6378 |
| 0.3330 | 0.0555 | 3.8313 | 0.5663 | 0.0555 | 0.3885 | 0.2775 | 0.0000 | 0.0011 | 0.0011 | 0.9445 | 0.0555 | 0.0011 | 0.0022 | 0.4721 | 0.0559 | 0.4721 | 0.1152 | 0.5152 |
| 0.3342 | 0.1340 | 3.5292 | 0.5058 | 0.0663 | 0.4668 | 0.3316 | 0.0000 | 0.0013 | 0.0000 | 0.9987 | 0.0013 | 0.0000 | 0.0013 | 0.4775 | 0.0451 | 0.4775 | 0.1046 | 0.5588 |
| 0.2439 | 0.0488 | 2.7317 | 0.3463 | 0.3659 | 0.2927 | 0.0976 | 0.0000 | 0.0019 | 0.0009 | 1.0000 | 0.0000 | 0.0464 | 0.0028 | 0.4309 | 0.1382 | 0.4309 | 0.1406 | 0.7272 |
| 0.1497 | 0.3752 | 3.6477 | 0.5295 | 0.1996 | 0.5240 | 0.1497 | 0.0000 | 0.0020 | 0.0000 | 1.0000 | 0.0000 | 0.0000 | 0.0020 | 0.4583 | 0.0835 | 0.4583 | 0.1273 | 0.6953 |
| 0.1025 | 0.1025 | 2.7172 | 0.3434 | 0.3842 | 0.2049 | 0.1537 | 0.0000 | 0.0031 | 0.0010 | 1.0000 | 0.0000 | 0.0000 | 0.0041 | 0.4273 | 0.1455 | 0.4273 | 0.1404 | 0.8076 |
| 0.2616 | 0.3442 | 4.0328 | 0.6066 | 0.0861 | 0.6110 | 0.1291 | 0.0000 | 0.0031 | 0.0000 | 0.9983 | 0.0017 | 0.0819 | 0.0031 | 0.4564 | 0.0872 | 0.4564 | 0.1214 | 0.7641 |
| 0.2749 | 0.1808 | 3.1374 | 0.4275 | 0.1808 | 0.4557 | 0.1356 | 0.0000 | 0.0072 | 0.0018 | 0.9982 | 0.0018 | 0.0018 | 0.0089 | 0.4461 | 0.1061 | 0.4479 | 0.1365 | 0.7418 |
| 0.1774 | 0.1470 | 2.5613 | 0.3123 | 0.1167 | 0.2964 | 0.1459 | 0.0000 | 0.0023 | 0.0000 | 0.9988 | 0.0012 | 0.0000 | 0.0035 | 0.4646 | 0.0696 | 0.4658 | 0.1355 | 0.8736 |
| 0.1736 | 0.1722 | 3.2741 | 0.4548 | 0.2755 | 0.3802 | 0.1377 | 0.0000 | 0.0027 | 0.0014 | 0.9986 | 0.0014 | 0.0014 | 0.0041 | 0.4079 | 0.1152 | 0.4768 | 0.1405 | 0.7244 |
| 0.1615 | 0.2564 | 2.9038 | 0.3808 | 0.1603 | 0.4859 | 0.0962 | 0.0000 | 0.0038 | 0.0000 | 0.9987 | 0.0013 | 0.0025 | 0.0038 | 0.4342 | 0.0329 | 0.5329 | 0.1554 | 0.8737 |
| 0.2500 | 0.1244 | 3.0622 | 0.4124 | 0.2488 | 0.4403 | 0.1866 | 0.0000 | 0.0062 | 0.0012 | 0.9988 | 0.0012 | 0.0025 | 0.0074 | 0.3750 | 0.1256 | 0.4994 | 0.1382 | 0.4765 |
| 0.1226 | 0.4507 | 3.9603 | 0.5921 | 0.1214 | 0.5757 | 0.1214 | 0.0000 | 0.0048 | 0.0012 | 0.9988 | 0.0012 | 0.0000 | 0.0060 | 0.4685 | 0.0617 | 0.4698 | 0.1125 | 0.5947 |
| 0.4416 | 0.2096 | 3.8098 | 0.5620 | 0.0928 | 0.7430 | 0.0696 | 0.0000 | 0.0028 | 0.0009 | 0.9991 | 0.0009 | 0.0018 | 0.0037 | 0.4301 | 0.0702 | 0.4997 | 0.1106 | 0.8176 |
| 0.2629 | 0.1718 | 3.2926 | 0.4585 | 0.1718 | 0.4828 | 0.1289 | 0.0000 | 0.0051 | 0.0017 | 0.9983 | 0.0017 | 0.0051 | 0.0085 | 0.4336 | 0.0882 | 0.4782 | 0.1358 | 0.7435 |

| | | | | | | | | | | | | | | | | | | |
|---|---|---|---|---|---|---|---|---|---|---|---|---|---|---|---|---|---|---|
| 0.0642 | 0.0617 | 2.7466 | 0.3493 | 0.3741 | 0.5000 | 0.1235 | 0.0000 | 0.0037 | 0.0012 | 0.9988 | 0.0012 | 0.0037 | 0.0049 | 0.3548 | 0.1051 | 0.5402 | 0.1398 | 0.5091 |
| 0.4847 | 0.1020 | 3.3845 | 0.4769 | 0.2561 | 0.6663 | 0.0520 | 0.0000 | 0.0030 | 0.0010 | 1.0000 | 0.0000 | 0.0267 | 0.0040 | 0.3727 | 0.1025 | 0.5249 | 0.1146 | 0.6442 |
| 0.6022 | 0.0831 | 3.4788 | 0.4958 | 0.1453 | 0.6877 | 0.0623 | 0.0000 | 0.0024 | 0.0008 | 0.9585 | 0.0415 | 0.0203 | 0.0041 | 0.4410 | 0.0764 | 0.4826 | 0.1035 | 0.5274 |
| 0.2850 | 0.1894 | 3.1924 | 0.4385 | 0.4025 | 0.5246 | 0.0710 | 0.0000 | 0.0028 | 0.0009 | 0.9991 | 0.0009 | 0.0000 | 0.0038 | 0.3729 | 0.1348 | 0.4923 | 0.1300 | 0.8376 |
| 0.1756 | 0.0499 | 2.6257 | 0.3251 | 0.3992 | 0.5489 | 0.0519 | 0.0000 | 0.0030 | 0.0010 | 0.9750 | 0.0250 | 0.0000 | 0.0040 | 0.3709 | 0.1583 | 0.4707 | 0.1247 | 0.3012 |
| 0.2964 | 0.4250 | 3.6487 | 0.5297 | 0.1848 | 0.5004 | 0.3141 | 0.0007 | 0.0000 | 0.0000 | 1.0000 | 0.0000 | 0.0000 | 0.0000 | 0.4016 | 0.2530 | 0.3454 | 0.1232 | 0.9249 |
| 0.2080 | 0.3944 | 3.5533 | 0.5107 | 0.2693 | 0.2958 | 0.4143 | 0.0207 | 0.0000 | 0.0000 | 1.0000 | 0.0000 | 0.0000 | 0.0000 | 0.3488 | 0.2635 | 0.3877 | 0.1213 | 0.9012 |
| 0.3258 | 0.1520 | 3.7593 | 0.5519 | 0.2606 | 0.4570 | 0.2824 | 0.0000 | 0.0000 | 0.0000 | 1.0000 | 0.0000 | 0.0000 | 0.0000 | 0.3339 | 0.2679 | 0.3982 | 0.1018 | 0.8240 |
| 0.0566 | 0.2075 | 3.1132 | 0.4226 | 0.3774 | 0.4906 | 0.1321 | 0.0000 | 0.0000 | 0.0000 | 1.0000 | 0.0000 | 0.0000 | 0.0000 | 0.4025 | 0.1195 | 0.4780 | 0.1219 | 0.7743 |
| 0.1087 | 0.3229 | 3.6383 | 0.5277 | 0.2960 | 0.4349 | 0.2422 | 0.0269 | 0.0000 | 0.0000 | 1.0000 | 0.0000 | 0.0000 | 0.0000 | 0.4327 | 0.2164 | 0.3509 | 0.1281 | 0.7021 |
| 0.0000 | 0.3092 | 4.3321 | 0.6664 | 0.0951 | 0.3340 | 0.5709 | 0.0000 | 0.0000 | 0.0000 | 1.0000 | 0.0000 | 0.0000 | 0.0000 | 0.3974 | 0.2537 | 0.3489 | 0.0980 | 0.7743 |
| 0.0222 | 0.1782 | 3.8187 | 0.5637 | 0.3324 | 0.2677 | 0.3777 | 0.0222 | 0.0000 | 0.0000 | 1.0000 | 0.0000 | 0.0000 | 0.0000 | 0.3936 | 0.2589 | 0.3475 | 0.1144 | 0.7190 |
| 0.0407 | 0.2549 | 3.4335 | 0.4867 | 0.3127 | 0.2955 | 0.3909 | 0.0008 | 0.0000 | 0.0000 | 1.0000 | 0.0000 | 0.0000 | 0.0000 | 0.3637 | 0.2155 | 0.4208 | 0.1252 | 0.6964 |
| 0.1070 | 0.2634 | 3.4491 | 0.4898 | 0.2099 | 0.5803 | 0.2099 | 0.0000 | 0.0000 | 0.0000 | 1.0000 | 0.0000 | 0.0000 | 0.0000 | 0.5446 | 0.1752 | 0.2802 | 0.1156 | 0.8914 |
| 0.0023 | 0.3696 | 4.0488 | 0.6098 | 0.2279 | 0.2891 | 0.4819 | 0.0011 | 0.0000 | 0.0000 | 1.0000 | 0.0000 | 0.0000 | 0.0000 | 0.4486 | 0.2184 | 0.3330 | 0.1194 | 0.8189 |
| 0.0008 | 0.0784 | 3.3205 | 0.4641 | 0.6857 | 0.2163 | 0.0784 | 0.0196 | 0.0000 | 0.0000 | 1.0000 | 0.0000 | 0.0000 | 0.0000 | 0.4383 | 0.2612 | 0.3004 | 0.0708 | 0.5388 |
| 0.0016 | 0.1807 | 4.5788 | 0.7158 | 0.0000 | 0.2635 | 0.6967 | 0.0398 | 0.0000 | 0.0000 | 1.0000 | 0.0000 | 0.0000 | 0.0000 | 0.4046 | 0.2529 | 0.3425 | 0.0888 | 0.6388 |
| 0.0009 | 0.2500 | 4.3397 | 0.6679 | 0.0000 | 0.3886 | 0.6114 | 0.0000 | 0.0000 | 0.0000 | 1.0000 | 0.0000 | 0.0000 | 0.0000 | 0.4706 | 0.2197 | 0.3098 | 0.1045 | 0.5903 |
| 0.0000 | 0.3325 | 4.7836 | 0.7567 | 0.0000 | 0.3964 | 0.4831 | 0.1205 | 0.0000 | 0.0000 | 1.0000 | 0.0000 | 0.0000 | 0.0000 | 0.4759 | 0.2018 | 0.3223 | 0.0939 | 0.6236 |
| 0.0007 | 0.3039 | 4.1278 | 0.6256 | 0.1427 | 0.5542 | 0.2853 | 0.0178 | 0.0000 | 0.0000 | 1.0000 | 0.0000 | 0.0000 | 0.0000 | 0.5686 | 0.1488 | 0.2826 | 0.1313 | 0.7557 |
| 0.0000 | 0.4288 | 4.8278 | 0.7656 | 0.0000 | 0.3694 | 0.5899 | 0.0407 | 0.0000 | 0.0000 | 1.0000 | 0.0000 | 0.0000 | 0.0000 | 0.4668 | 0.1905 | 0.3427 | 0.0834 | 0.8772 |
| 0.0649 | 0.0225 | 2.5775 | 0.3155 | 0.6710 | 0.1333 | 0.1948 | 0.0009 | 0.0000 | 0.0000 | 0.9991 | 0.0009 | 0.0000 | 0.0000 | 0.3305 | 0.1407 | 0.5288 | 0.0901 | 0.7250 |
| 0.0017 | 0.0826 | 2.1345 | 0.2269 | 0.8694 | 0.0893 | 0.0000 | 0.0413 | 0.0000 | 0.0000 | 0.9983 | 0.0017 | 0.0000 | 0.0000 | 0.3135 | 0.0424 | 0.6441 | 0.1168 | 0.7530 |
| 0.0268 | 0.0258 | 2.8726 | 0.3745 | 0.4980 | 0.1034 | 0.3738 | 0.0249 | 0.0000 | 0.0000 | 1.0000 | 0.0000 | 0.0000 | 0.0000 | 0.3752 | 0.1501 | 0.4747 | 0.0905 | 0.6724 |
| 0.0252 | 0.0727 | 2.5858 | 0.3172 | 0.6056 | 0.1512 | 0.2422 | 0.0010 | 0.0000 | 0.0000 | 0.9990 | 0.0010 | 0.0000 | 0.0000 | 0.3506 | 0.1544 | 0.4950 | 0.0991 | 0.6123 |
| 0.0531 | 0.0031 | 2.9208 | 0.3842 | 0.5469 | 0.0604 | 0.3917 | 0.0010 | 0.0000 | 0.0000 | 1.0000 | 0.0000 | 0.0254 | 0.0000 | 0.3564 | 0.0799 | 0.5637 | 0.0846 | 0.7283 |
| 0.0712 | 0.0712 | 2.7065 | 0.3413 | 0.5930 | 0.1698 | 0.2135 | 0.0237 | 0.0000 | 0.0000 | 1.0000 | 0.0000 | 0.0009 | 0.0000 | 0.3540 | 0.1031 | 0.5429 | 0.0946 | 0.7467 |
| 0.0230 | 0.0230 | 2.8628 | 0.3726 | 0.5536 | 0.0700 | 0.3322 | 0.0443 | 0.0000 | 0.0000 | 1.0000 | 0.0000 | 0.0225 | 0.0000 | 0.4191 | 0.1405 | 0.4404 | 0.0937 | 0.7832 |
| 0.0415 | 0.0203 | 2.8711 | 0.3742 | 0.4882 | 0.1253 | 0.3458 | 0.0407 | 0.0000 | 0.0000 | 1.0000 | 0.0000 | 0.0000 | 0.0000 | 0.4182 | 0.0618 | 0.5199 | 0.0980 | 0.7612 |
| 0.0697 | 0.0465 | 2.9515 | 0.3903 | 0.4879 | 0.1171 | 0.3717 | 0.0232 | 0.0000 | 0.0000 | 1.0000 | 0.0000 | 0.0000 | 0.0000 | 0.4032 | 0.1007 | 0.4961 | 0.0927 | 0.6786 |
| 0.0035 | 0.0304 | 2.5812 | 0.3162 | 0.6437 | 0.0923 | 0.2348 | 0.0292 | 0.0000 | 0.0000 | 1.0000 | 0.0000 | 0.0023 | 0.0000 | 0.3921 | 0.0989 | 0.5090 | 0.0891 | 0.6926 |
| 0.0259 | 0.0517 | 2.5684 | 0.3137 | 0.5970 | 0.1781 | 0.2000 | 0.0249 | 0.0000 | 0.0000 | 1.0000 | 0.0000 | 0.0020 | 0.0000 | 0.3726 | 0.1582 | 0.4692 | 0.0946 | 0.6775 |
| 0.0322 | 0.0644 | 2.4756 | 0.2951 | 0.6208 | 0.1921 | 0.1252 | 0.0620 | 0.0000 | 0.0000 | 1.0000 | 0.0000 | 0.0000 | 0.0000 | 0.2935 | 0.1355 | 0.5710 | 0.0995 | 0.8291 |
| 0.0021 | 0.1083 | 2.7114 | 0.3423 | 0.6174 | 0.1661 | 0.2154 | 0.0011 | 0.0000 | 0.0000 | 0.9989 | 0.0011 | 0.0000 | 0.0000 | 0.4452 | 0.2699 | 0.2849 | 0.1114 | 0.3498 |
| 0.0000 | 0.3768 | 3.6436 | 0.5287 | 0.3324 | 0.4902 | 0.1773 | 0.0000 | 0.0000 | 0.0000 | 1.0000 | 0.0000 | 0.0000 | 0.0000 | 0.5077 | 0.1628 | 0.3295 | 0.1354 | 0.8358 |
| 0.0313 | 0.2410 | 3.2169 | 0.4434 | 0.4530 | 0.4554 | 0.0916 | 0.0000 | 0.0000 | 0.0000 | 1.0000 | 0.0000 | 0.0012 | 0.0000 | 0.2940 | 0.0518 | 0.6542 | 0.1378 | 0.4833 |
| 0.0009 | 0.0472 | 2.4704 | 0.2941 | 0.8102 | 0.0741 | 0.1157 | 0.0000 | 0.0000 | 0.0000 | 1.0000 | 0.0000 | 0.0000 | 0.0000 | 0.0961 | 0.0466 | 0.8573 | 0.0544 | 0.9520 |
| 0.1106 | 0.1670 | 4.4956 | 0.6991 | 0.0011 | 0.3905 | 0.6084 | 0.0000 | 0.0000 | 0.0000 | 0.9989 | 0.0011 | 0.0011 | 0.0000 | 0.4723 | 0.2223 | 0.3053 | 0.0976 | 0.7966 |
| 0.0000 | 0.1541 | 2.6028 | 0.3206 | 0.7094 | 0.1940 | 0.0966 | 0.0000 | 0.0000 | 0.0000 | 1.0000 | 0.0000 | 0.0000 | 0.0000 | 0.2513 | 0.0964 | 0.6524 | 0.1041 | 0.3500 |
| 0.0000 | 0.2051 | 4.4991 | 0.6998 | 0.0000 | 0.5236 | 0.4764 | 0.0000 | 0.0000 | 0.0000 | 1.0000 | 0.0000 | 0.0000 | 0.0000 | 0.6553 | 0.1213 | 0.2234 | 0.1020 | 0.7274 |
| 0.0000 | 0.3720 | 4.6058 | 0.7212 | 0.0000 | 0.5826 | 0.4174 | 0.0000 | 0.0000 | 0.0000 | 1.0000 | 0.0000 | 0.0000 | 0.0000 | 0.6238 | 0.1011 | 0.2750 | 0.0998 | 0.7816 |
| 0.0000 | 0.0400 | 4.4600 | 0.6920 | 0.0000 | 0.4205 | 0.5795 | 0.0000 | 0.0000 | 0.0000 | 1.0000 | 0.0000 | 0.0000 | 0.0000 | 0.6667 | 0.1667 | 0.1667 | 0.0938 | 0.6773 |
| 0.0043 | 0.0300 | 4.4394 | 0.6879 | 0.0000 | 0.4636 | 0.5096 | 0.0268 | 0.0000 | 0.0000 | 1.0000 | 0.0000 | 0.0000 | 0.0000 | 0.6034 | 0.1977 | 0.1988 | 0.1052 | 0.8733 |
| 0.0009 | 0.0659 | 4.1509 | 0.6302 | 0.0000 | 0.3495 | 0.6505 | 0.0000 | 0.0000 | 0.0000 | 1.0000 | 0.0000 | 0.0000 | 0.0000 | 0.5652 | 0.1957 | 0.2391 | 0.1290 | 0.6322 |
| 0.0433 | 0.4032 | 4.1951 | 0.6390 | 0.0637 | 0.5968 | 0.3183 | 0.0212 | 0.0000 | 0.0000 | 1.0000 | 0.0000 | 0.0008 | 0.0000 | 0.5845 | 0.1282 | 0.2874 | 0.1276 | 0.8612 |
| 0.0012 | 0.0879 | 2.6425 | 0.3285 | 0.5862 | 0.2087 | 0.1758 | 0.0293 | 0.0000 | 0.0000 | 1.0000 | 0.0000 | 0.0000 | 0.0000 | 0.4220 | 0.2157 | 0.3623 | 0.1169 | 0.3641 |
| 0.0339 | 0.0607 | 3.5102 | 0.5020 | 0.2629 | 0.4731 | 0.2348 | 0.0292 | 0.0000 | 0.0000 | 0.9988 | 0.0012 | 0.0000 | 0.0000 | 0.4947 | 0.2453 | 0.2599 | 0.1364 | 0.6095 |
| 0.0016 | 0.0412 | 3.8692 | 0.5738 | 0.1981 | 0.2472 | 0.4754 | 0.0792 | 0.0000 | 0.0000 | 1.0000 | 0.0000 | 0.0000 | 0.0000 | 0.5082 | 0.2261 | 0.2657 | 0.1213 | 0.6937 |
| 0.0034 | 0.6878 | 4.7263 | 0.7453 | 0.0017 | 0.7804 | 0.2161 | 0.0017 | 0.0000 | 0.0000 | 1.0000 | 0.0000 | 0.0000 | 0.0000 | 0.4728 | 0.1025 | 0.4247 | 0.0878 | 0.9667 |
| 0.0951 | 0.2389 | 4.3638 | 0.6728 | 0.0000 | 0.1762 | 0.8238 | 0.0000 | 0.0000 | 0.0000 | 1.0000 | 0.0000 | 0.0000 | 0.0000 | 0.7352 | 0.1326 | 0.1322 | 0.1040 | 0.6737 |
| 0.0730 | 0.2928 | 4.1256 | 0.6251 | 0.0730 | 0.3677 | 0.5350 | 0.0243 | 0.0000 | 0.0000 | 1.0000 | 0.0000 | 0.0000 | 0.0000 | 0.6420 | 0.1304 | 0.2276 | 0.1220 | 0.7645 |

| | | | | | | | | | | | | | | | | | | |
|---|---|---|---|---|---|---|---|---|---|---|---|---|---|---|---|---|---|---|
| 0.0009 | 0.0460 | 4.5058 | 0.7012 | 0.0000 | 0.2912 | 0.6204 | 0.0885 | 0.0000 | 0.0000 | 1.0000 | 0.0000 | 0.0000 | 0.0000 | 0.5117 | 0.2444 | 0.2440 | 0.1088 | 0.7616 |
| 0.1595 | 0.0606 | 3.8994 | 0.5799 | 0.0000 | 0.5797 | 0.4203 | 0.0000 | 0.0000 | 0.0000 | 0.9992 | 0.0008 | 0.0000 | 0.0000 | 0.5104 | 0.2498 | 0.2398 | 0.1158 | 0.7470 |
| 0.0000 | 0.0509 | 4.1849 | 0.6370 | 0.0000 | 0.3014 | 0.6487 | 0.0499 | 0.0000 | 0.0000 | 1.0000 | 0.0000 | 0.0000 | 0.0000 | 0.6500 | 0.1750 | 0.1750 | 0.1191 | 0.8419 |
| 0.0235 | 0.1147 | 3.4522 | 0.4904 | 0.4065 | 0.2322 | 0.3162 | 0.0452 | 0.0000 | 0.0000 | 1.0000 | 0.0000 | 0.0000 | 0.0000 | 0.4708 | 0.2422 | 0.2870 | 0.1245 | 0.8115 |
| 0.0000 | 0.2353 | 4.6515 | 0.7303 | 0.0000 | 0.1471 | 0.8235 | 0.0294 | 0.0000 | 0.0000 | 1.0000 | 0.0000 | 0.0000 | 0.0000 | 0.7083 | 0.1495 | 0.1422 | 0.0832 | 0.5528 |
| 0.0000 | 0.1558 | 4.4659 | 0.6932 | 0.0000 | 0.1902 | 0.8098 | 0.0000 | 0.0000 | 0.0000 | 1.0000 | 0.0000 | 0.0000 | 0.0000 | 0.6927 | 0.1579 | 0.1493 | 0.1056 | 0.6307 |
| 0.0009 | 0.1883 | 4.7142 | 0.7428 | 0.0000 | 0.2115 | 0.7653 | 0.0232 | 0.0000 | 0.0000 | 0.9991 | 0.0009 | 0.0000 | 0.0000 | 0.6430 | 0.1792 | 0.1778 | 0.0863 | 0.7486 |
| 0.0000 | 0.0961 | 4.5005 | 0.7001 | 0.0000 | 0.1437 | 0.8563 | 0.0000 | 0.0000 | 0.0000 | 1.0000 | 0.0000 | 0.0000 | 0.0000 | 0.6982 | 0.1511 | 0.1507 | 0.0889 | 0.8138 |
| 0.0000 | 0.1466 | 4.4910 | 0.6982 | 0.0000 | 0.1103 | 0.8534 | 0.0363 | 0.0000 | 0.0000 | 1.0000 | 0.0000 | 0.0000 | 0.0000 | 0.6610 | 0.1697 | 0.1693 | 0.1056 | 0.6359 |
| 0.0000 | 0.0000 | NA | NA | 0.0000 | 0.0000 | 0.0000 | 1.0000 | 0.0849 | 0.8962 | 1.0000 | 0.0000 | 0.0094 | 0.9811 | 0.5000 | 0.0000 | 0.5000 | 0.0000 | NA |
| 0.0056 | 0.0056 | 1.8596 | 0.1719 | 0.7135 | 0.1461 | 0.1404 | 0.0000 | 0.0017 | 0.6885 | 1.0000 | 0.0000 | 0.0034 | 0.6902 | 0.4504 | 0.2341 | 0.3155 | 0.1057 | 0.3155 |
| 0.0667 | 0.3333 | 3.2273 | 0.4455 | 0.2000 | 0.2000 | 0.3333 | 0.2667 | 0.4960 | 0.2480 | 1.0000 | 0.0000 | 0.0160 | 0.7440 | 0.4611 | 0.1111 | 0.4278 | 0.0754 | 0.7235 |
| 0.0000 | 0.0000 | 2.0063 | 0.2013 | 0.9937 | 0.0016 | 0.0032 | 0.0016 | 0.0000 | 0.0409 | 1.0000 | 0.0000 | 0.0015 | 0.0409 | 0.4987 | 0.0011 | 0.5003 | 0.0019 | 0.2658 |
| 0.0000 | 0.2857 | 3.7143 | 0.5429 | 0.0000 | 0.5714 | 0.4286 | 0.0000 | 0.9252 | 0.0093 | 1.0000 | 0.0000 | 0.0000 | 0.9346 | 0.6190 | 0.0476 | 0.3333 | 0.1212 | 0.6130 |
| 0.0233 | 0.3140 | 2.7412 | 0.3482 | 0.0233 | 0.3605 | 0.6047 | 0.0116 | 0.7175 | 0.0063 | 0.7093 | 0.2907 | 0.0032 | 0.7238 | 0.4457 | 0.1085 | 0.4457 | 0.1250 | 0.9412 |
| 0.0000 | 0.0000 | 2.3333 | 0.2667 | 0.2667 | 0.2667 | 0.4667 | 0.0000 | 0.8056 | 0.0463 | 1.0000 | 0.0000 | 0.0093 | 0.8519 | 0.4444 | 0.0444 | 0.5111 | 0.1409 | 0.8025 |
| 0.1421 | 0.0109 | 3.4121 | 0.4824 | 0.0109 | 0.4317 | 0.5519 | 0.0055 | 0.4891 | 0.0633 | 1.0000 | 0.0000 | 0.0024 | 0.5523 | 0.5638 | 0.0036 | 0.4326 | 0.1240 | 0.6674 |
| 0.0227 | 0.1818 | 3.8571 | 0.5714 | 0.1364 | 0.3182 | 0.5000 | 0.0455 | 0.6224 | 0.0559 | 0.9773 | 0.0227 | 0.0140 | 0.6783 | 0.5189 | 0.1212 | 0.3598 | 0.1168 | 0.7518 |
| 0.0000 | 0.0000 | 2.0280 | 0.2056 | 0.7130 | 0.0463 | 0.2315 | 0.0093 | 0.0091 | 0.0091 | 1.0000 | 0.0000 | 0.0000 | 0.0182 | 0.4552 | 0.0802 | 0.4645 | 0.1401 | 0.9495 |
| 0.0000 | 0.0000 | 3.5000 | 0.5000 | 0.0000 | 0.0000 | 1.0000 | 0.0000 | 0.0000 | 0.9490 | 1.0000 | 0.0000 | 0.0306 | 0.9490 | 0.4167 | 0.1667 | 0.4167 | 0.1687 | NA |
| 0.0000 | 0.0000 | 3.6299 | 0.5260 | 0.0195 | 0.6494 | 0.3312 | 0.0000 | 0.2165 | 0.1126 | 1.0000 | 0.0000 | 0.0043 | 0.3290 | 0.1710 | 0.0044 | 0.8246 | 0.1335 | 0.4217 |
| 0.0109 | 0.0652 | 2.0178 | 0.2036 | 0.6957 | 0.1902 | 0.0326 | 0.0815 | 0.0000 | 0.4007 | 1.0000 | 0.0000 | 0.0000 | 0.4007 | 0.3306 | 0.0399 | 0.6295 | 0.1116 | 0.8868 |
| 0.0080 | 0.0400 | 2.0083 | 0.2017 | 0.6400 | 0.3040 | 0.0240 | 0.0320 | 0.0000 | 0.2500 | 1.0000 | 0.0000 | 0.0060 | 0.2500 | 0.3853 | 0.0613 | 0.5533 | 0.0996 | 0.8166 |
| 0.0067 | 0.5733 | 3.2467 | 0.4493 | 0.3467 | 0.6133 | 0.0400 | 0.0000 | 0.0000 | 0.2944 | 1.0000 | 0.0000 | 0.0047 | 0.2944 | 0.6044 | 0.0311 | 0.3644 | 0.0931 | 0.9012 |
| 0.0161 | 0.0323 | 1.7815 | 0.1563 | 0.7258 | 0.2097 | 0.0242 | 0.0403 | 0.0000 | 0.2470 | 1.0000 | 0.0000 | 0.0060 | 0.2470 | 0.3441 | 0.2191 | 0.4368 | 0.0910 | 0.7042 |
| 0.0068 | 0.0541 | 1.5850 | 0.1170 | 0.7770 | 0.1959 | 0.0203 | 0.0068 | 0.0000 | 0.3565 | 0.9932 | 0.0068 | 0.0000 | 0.3565 | 0.4707 | 0.0146 | 0.5146 | 0.0766 | 0.8726 |
| 0.0261 | 0.0196 | 2.1765 | 0.2353 | 0.7908 | 0.1961 | 0.0131 | 0.0000 | 0.0000 | 0.0941 | 1.0000 | 0.0000 | 0.0059 | 0.0941 | 0.1253 | 0.0109 | 0.8638 | 0.0769 | 0.9444 |
| 0.0095 | 0.1611 | 2.4455 | 0.2891 | 0.7346 | 0.1469 | 0.1185 | 0.0000 | 0.0000 | 0.0746 | 1.0000 | 0.0000 | 0.0000 | 0.0746 | 0.1793 | 0.0395 | 0.7812 | 0.0930 | 0.9032 |
| 0.0142 | 0.2482 | 2.5357 | 0.3071 | 0.4823 | 0.4397 | 0.0709 | 0.0071 | 0.0000 | 0.3155 | 1.0000 | 0.0000 | 0.0000 | 0.3155 | 0.4953 | 0.0378 | 0.4669 | 0.0804 | 0.8629 |
| 0.0158 | 0.8632 | 4.0947 | 0.6189 | 0.0000 | 0.1421 | 0.8579 | 0.0000 | 0.0000 | 0.0404 | 1.0000 | 0.0000 | 0.0000 | 0.0404 | 0.4298 | 0.2825 | 0.2877 | 0.0632 | 0.9382 |
| 0.0000 | 0.0884 | 4.5479 | 0.7096 | 0.1020 | 0.1769 | 0.7143 | 0.0068 | 0.0000 | 0.0000 | 1.0000 | 0.0000 | 0.0000 | 0.0000 | 0.7324 | 0.0658 | 0.2018 | 0.0770 | 0.7205 |
| 0.0060 | 0.8929 | 3.7738 | 0.5548 | 0.0893 | 0.0714 | 0.8393 | 0.0000 | 0.0171 | 0.2650 | 1.0000 | 0.0000 | 0.0000 | 0.2821 | 0.3671 | 0.2778 | 0.3552 | 0.0473 | 0.9849 |
| 0.0099 | 0.5842 | 3.1300 | 0.4260 | 0.2574 | 0.3366 | 0.3960 | 0.0099 | 0.3137 | 0.0261 | 1.0000 | 0.0000 | 0.0000 | 0.3399 | 0.4934 | 0.1617 | 0.3449 | 0.0941 | 0.9382 |
| 0.0734 | 0.2661 | 3.8119 | 0.5624 | 0.0734 | 0.6789 | 0.1743 | 0.0734 | 0.0000 | 0.0000 | 0.9725 | 0.0275 | 0.0000 | 0.0000 | 0.6376 | 0.0963 | 0.2661 | 0.1092 | 0.8025 |
| 0.0169 | 0.0424 | 2.8257 | 0.3651 | 0.4661 | 0.4237 | 0.0424 | 0.0678 | 0.0000 | 0.5261 | 1.0000 | 0.0000 | 0.0000 | 0.5261 | 0.4202 | 0.0869 | 0.4929 | 0.0993 | 0.7955 |
| 0.0205 | 0.2260 | 3.2464 | 0.4493 | 0.4110 | 0.4315 | 0.1027 | 0.0548 | 0.0000 | 0.0000 | 0.9795 | 0.0205 | 0.0000 | 0.0000 | 0.6358 | 0.0263 | 0.3379 | 0.0871 | 0.7439 |
| 0.0206 | 0.4742 | 3.9119 | 0.5824 | 0.2474 | 0.5670 | 0.1804 | 0.0052 | 0.0229 | 0.4155 | 1.0000 | 0.0000 | 0.0029 | 0.4413 | 0.4751 | 0.0808 | 0.4442 | 0.0996 | 0.8509 |
| 0.0155 | 0.1192 | 2.5784 | 0.3157 | 0.6425 | 0.1969 | 0.1192 | 0.0415 | 0.0000 | 0.0853 | 1.0000 | 0.0000 | 0.0000 | 0.0853 | 0.5535 | 0.0432 | 0.4033 | 0.0695 | 0.8137 |
| 0.0203 | 0.0270 | 2.3333 | 0.2667 | 0.7027 | 0.1689 | 0.0270 | 0.1014 | 0.0000 | 0.4559 | 1.0000 | 0.0000 | 0.0000 | 0.4559 | 0.1531 | 0.0340 | 0.8129 | 0.0797 | 0.8362 |
| 0.0194 | 0.0777 | 2.5248 | 0.3050 | 0.7282 | 0.2524 | 0.0097 | 0.0097 | 0.0000 | 0.0000 | 0.9903 | 0.0000 | 0.0000 | 0.0000 | 0.5163 | 0.0850 | 0.3987 | 0.0640 | 0.9470 |
| 0.0153 | 0.2296 | 3.4947 | 0.4989 | 0.4133 | 0.5153 | 0.0306 | 0.0408 | 0.0000 | 0.0481 | 1.0000 | 0.0000 | 0.0096 | 0.0481 | 0.4286 | 0.1684 | 0.4031 | 0.1075 | 0.7682 |

| 36 FEve | 36 FRic | 36 RaoQ |
|---|---|---|
| 0.6858 | 0.0329 | 0.0138 |
| 0.6191 | 0.0312 | 0.0105 |
| 0.8873 | 0.0050 | 0.0233 |
| 0.8582 | 0.0019 | 0.0087 |
| 0.8364 | 0.0154 | 0.0118 |
| 0.5395 | 0.0052 | 0.0109 |
| 0.7588 | 0.0357 | 0.0121 |
| 0.8838 | 0.0087 | 0.0204 |
| 0.7952 | 0.0074 | 0.0165 |
| 0.6372 | 0.0022 | 0.0108 |
| 0.6487 | 0.0019 | 0.0139 |
| 0.8697 | 0.0172 | 0.0181 |
| 0.7475 | 0.0361 | 0.0137 |
| 0.7852 | 0.0095 | 0.0150 |
| 0.9191 | 0.0021 | 0.0127 |
| 0.8777 | 0.0038 | 0.0110 |
| 0.8558 | 0.0740 | 0.0139 |
| 0.8859 | 0.0053 | 0.0156 |
| 0.8904 | 0.0078 | 0.0225 |
| 0.7892 | 0.0187 | 0.0115 |
| 0.7033 | 0.0242 | 0.0098 |
| 0.8774 | 0.0117 | 0.0136 |
| 0.8733 | 0.0059 | 0.0116 |
| 0.7534 | 0.0106 | 0.0146 |
| 0.8337 | 0.0156 | 0.0161 |
| 0.6959 | 0.0083 | 0.0109 |
| 0.8103 | 0.0187 | 0.0134 |
| 0.8332 | 0.0285 | 0.0128 |
| 0.8300 | 0.0363 | 0.0169 |
| 0.8166 | 0.0250 | 0.0125 |
| 0.6917 | 0.0329 | 0.0077 |
| 0.8913 | 0.0078 | 0.0158 |
| 0.7627 | 0.0236 | 0.0126 |
| 0.8493 | 0.0599 | 0.0137 |
| 0.8133 | 0.0329 | 0.0154 |
| 0.7202 | 0.0043 | 0.0143 |
| 0.7750 | 0.0207 | 0.0138 |
| 0.9038 | 0.0331 | 0.0125 |
| 0.8630 | 0.0034 | 0.0176 |
| 0.8155 | 0.0196 | 0.0134 |
| 0.5060 | 0.0026 | 0.0092 |
| 0.8821 | 0.0027 | 0.0046 |
| 0.6752 | 0.0225 | 0.0121 |
| 0.7762 | 0.0088 | 0.0220 |
| 0.7160 | 0.0069 | 0.0129 |
| 0.7459 | 0.0368 | 0.0141 |
| 0.7870 | 0.0020 | 0.0118 |
| 0.7791 | 0.0245 | 0.0087 |
| 0.8254 | 0.0329 | 0.0142 |
| 0.8871 | 0.0275 | 0.0194 |

| | | |
|---|---|---|
| 0.8586 | 0.0650 | 0.0152 |
| 0.8692 | 0.0154 | 0.0070 |
| 0.6795 | 0.0020 | 0.0133 |
| 0.6215 | 0.0037 | 0.0087 |
| 0.8475 | 0.0257 | 0.0154 |
| 0.7915 | 0.0211 | 0.0145 |
| 0.8595 | 0.0705 | 0.0119 |
| 0.8447 | 0.0244 | 0.0136 |
| 0.8113 | 0.0547 | 0.0142 |
| 0.8239 | 0.0183 | 0.0146 |
| 0.9695 | 0.0012 | 0.0030 |
| 0.1602 | 0.0032 | 0.0065 |
| 0.6352 | 0.0036 | 0.0128 |
| 0.9014 | 0.0026 | 0.0370 |
| 0.9886 | 0.0004 | 0.0007 |
| 0.8606 | 0.0028 | 0.0263 |
| 0.5244 | 0.0028 | 0.0267 |
| 0.5257 | 0.0093 | 0.0110 |
| 0.5608 | 0.0107 | 0.0174 |
| 0.5148 | 0.0101 | 0.0058 |
| 0.5931 | 0.0097 | 0.0086 |
| 0.6570 | 0.0111 | 0.0067 |
| 0.5088 | 0.0105 | 0.0089 |
| 0.5064 | 0.0820 | 0.0120 |
| 0.6538 | 0.0308 | 0.0396 |
| 0.7876 | 0.0204 | 0.0104 |
| 0.7574 | 0.0653 | 0.0099 |
| 0.9955 | 0.0126 | 0.0120 |
| 0.9388 | 0.0096 | 0.0186 |
| 0.8924 | 0.0186 | 0.0143 |
| 0.6830 | 0.0209 | 0.0142 |
| 0.8871 | 0.0656 | 0.0178 |
| 0.9991 | 0.0011 | 0.0121 |
| 0.7284 | 0.0017 | 0.0097 |
| 0.7597 | 0.0598 | 0.0138 |
| 0.7364 | 0.0653 | 0.0153 |
| 0.8523 | 0.0169 | 0.0124 |
| 0.6600 | 0.0308 | 0.0093 |
| 0.5630 | 0.0653 | 0.0046 |
| 0.5984 | 0.0653 | 0.0082 |
| 0.9957 | 0.0065 | 0.0155 |
| 0.6664 | 0.0621 | 0.0115 |
| 0.8405 | 0.0072 | 0.0112 |
| 0.7543 | 0.0732 | 0.0067 |
| 0.7021 | 0.0067 | 0.0109 |
| 0.7085 | 0.0067 | 0.0151 |
| 0.8626 | 0.0657 | 0.0120 |
| 0.8352 | 0.0717 | 0.0137 |
| 0.7874 | 0.0657 | 0.0089 |
| 0.8251 | 0.0717 | 0.0142 |
| 0.8221 | 0.0013 | 0.0091 |

| | | |
|---|---|---|
| 0.7176 | 0.0564 | 0.0125 |
| 0.8563 | 0.0657 | 0.0121 |
| 0.7810 | 0.0544 | 0.0121 |
| 0.7283 | 0.0524 | 0.0093 |
| 0.7539 | 0.0714 | 0.0127 |
| 0.7533 | 0.0011 | 0.0067 |
| 0.7382 | 0.0110 | 0.0086 |
| 0.9561 | 0.0050 | 0.0257 |
| 0.7452 | 0.0050 | 0.0168 |
| 0.9988 | 0.0014 | 0.0182 |
| 0.5608 | 0.0153 | 0.0049 |
| 0.7175 | 0.0189 | 0.0115 |
| 0.7348 | 0.0523 | 0.0126 |
| 0.8940 | 0.0563 | 0.0148 |
| 0.7049 | 0.0602 | 0.0108 |
| 0.7075 | 0.0563 | 0.0134 |
| 0.7062 | 0.0563 | 0.0121 |
| 0.6720 | 0.0618 | 0.0051 |
| 0.7173 | 0.0100 | 0.0140 |
| 0.9490 | 0.0563 | 0.0113 |
| 0.9504 | 0.0563 | 0.0140 |
| 0.8568 | 0.0209 | 0.0195 |
| 0.7886 | 0.0510 | 0.0148 |
| 0.7874 | 0.0544 | 0.0121 |
| 0.6582 | 0.0192 | 0.0117 |
| 0.6771 | 0.0012 | 0.0073 |
| 0.6426 | 0.0237 | 0.0109 |
| 0.7222 | 0.0203 | 0.0109 |
| 0.7619 | 0.0191 | 0.0124 |
| 0.6640 | 0.0653 | 0.0122 |
| 0.8524 | 0.0108 | 0.0136 |
| 0.8258 | 0.0561 | 0.0143 |
| 0.8658 | 0.0094 | 0.0182 |
| 0.7168 | 0.0386 | 0.0396 |
| 0.8214 | 0.0308 | 0.0226 |
| 0.7874 | 0.0069 | 0.0125 |
| 0.9019 | 0.0112 | 0.0148 |
| 0.8106 | 0.0885 | 0.0135 |
| 0.8696 | 0.0707 | 0.0075 |
| 0.6720 | 0.0615 | 0.0057 |
| 0.6911 | 0.0270 | 0.0080 |
| 0.8857 | 0.0235 | 0.0087 |
| 0.8132 | 0.2949 | 0.0119 |
| 0.7785 | 0.0783 | 0.0084 |
| 0.7521 | 0.0023 | 0.0118 |
| 0.7957 | 0.0885 | 0.0135 |
| 0.7436 | 0.0813 | 0.0110 |
| 0.7706 | 0.0701 | 0.0076 |
| 0.8482 | 0.0174 | 0.0083 |
| 0.7054 | 0.0707 | 0.0085 |
| 0.7844 | 0.0615 | 0.0138 |

| | | |
|---|---|---|
| 0.8263 | 0.0031 | 0.0099 |
| 0.8308 | 0.0039 | 0.0097 |
| 0.7562 | 0.0522 | 0.0187 |
| 0.7512 | 0.0121 | 0.0191 |
| 0.6667 | 0.0521 | 0.0096 |
| 0.8214 | 0.0522 | 0.0109 |
| 0.6750 | 0.0651 | 0.0125 |
| 0.8263 | 0.0228 | 0.0113 |
| 0.7678 | 0.0127 | 0.0125 |
| 0.7380 | 0.0069 | 0.0105 |
| 0.7014 | 0.0011 | 0.0044 |
| 0.5612 | 0.0013 | 0.0052 |
| 0.7192 | 0.0522 | 0.0124 |
| 0.7327 | 0.0019 | 0.0144 |
| 0.5996 | 0.0526 | 0.0077 |
| 0.6979 | 0.0510 | 0.0120 |
| 0.8045 | 0.0021 | 0.0137 |
| 0.5653 | 0.0017 | 0.0079 |
| 0.6077 | 0.0107 | 0.0109 |
| 0.4239 | 0.0174 | 0.0101 |
| 0.5139 | 0.0114 | 0.0108 |
| 0.2536 | 0.0122 | 0.0162 |
| 0.4197 | 0.0122 | 0.0164 |
| 0.5777 | 0.0409 | 0.0072 |
| 0.6285 | 0.1057 | 0.0132 |
| 0.5935 | 0.0911 | 0.0035 |
| 0.5623 | 0.1063 | 0.0153 |
| 0.4011 | 0.0037 | 0.0122 |
| 0.3501 | 0.0026 | 0.0184 |
| 0.7663 | 0.0812 | 0.0177 |
| 0.4662 | 0.0106 | 0.0113 |
| 0.2796 | 0.0112 | 0.0134 |
| 0.6988 | 0.0045 | 0.0220 |
| 0.3993 | 0.0085 | 0.0129 |
| 0.3682 | 0.0034 | 0.0124 |
| 0.3034 | 0.0035 | 0.0234 |
| 0.3703 | 0.0100 | 0.0179 |
| 0.6333 | 0.0300 | 0.0152 |
| 0.4178 | 0.0087 | 0.0125 |
| 0.3669 | 0.0092 | 0.0187 |
| 0.6399 | 0.0025 | 0.0215 |
| 0.6617 | 0.0027 | 0.0137 |
| 0.6830 | 0.0102 | 0.0155 |
| 0.7772 | 0.0279 | 0.0164 |
| 0.8432 | 0.0067 | 0.0161 |
| 0.4480 | 0.0275 | 0.0147 |
| 0.4882 | 0.0274 | 0.0197 |
| 0.7409 | 0.0024 | 0.0125 |
| 0.6994 | 0.0021 | 0.0135 |
| 0.3025 | 0.0199 | 0.0082 |
| 0.4217 | 0.0037 | 0.0201 |

| | | |
|---|---|---|
| 0.3216 | 0.0138 | 0.0157 |
| 0.6906 | 0.0019 | 0.0154 |
| 0.7290 | 0.0029 | 0.0132 |
| 0.2731 | 0.0030 | 0.0114 |
| 0.3312 | 0.0177 | 0.0089 |
| 0.3223 | 0.0083 | 0.0113 |
| 0.4200 | 0.0085 | 0.0114 |
| 0.5836 | 0.0293 | 0.0160 |
| 0.3863 | 0.0043 | 0.0148 |
| 0.4196 | 0.0034 | 0.0222 |
| 0.6768 | 0.0034 | 0.0167 |
| 0.4711 | 0.0703 | 0.0086 |
| 0.5622 | 0.0527 | 0.0067 |
| 0.6535 | 0.0787 | 0.0152 |
| 0.5989 | 0.0513 | 0.0134 |
| 0.6425 | 0.0513 | 0.0077 |
| 0.4493 | 0.0806 | 0.0095 |
| 0.5382 | 0.0539 | 0.0040 |
| 0.4082 | 0.0805 | 0.0109 |
| 0.7481 | 0.0787 | 0.0091 |
| 0.5909 | 0.0787 | 0.0153 |
| 0.5947 | 0.0512 | 0.0129 |
| 0.6846 | 0.0500 | 0.0126 |
| 0.5953 | 0.0500 | 0.0143 |
| 0.6353 | 0.0182 | 0.0050 |
| 0.7369 | 0.0028 | 0.0060 |
| 0.7185 | 0.0060 | 0.0139 |
| 0.8611 | 0.0041 | 0.0080 |
| 0.6943 | 0.0105 | 0.0162 |
| 0.6173 | 0.0126 | 0.0139 |
| 0.6387 | 0.0088 | 0.0080 |
| 0.6240 | 0.0767 | 0.0117 |
| 0.4793 | 0.0490 | 0.0123 |
| 0.4725 | 0.0015 | 0.0070 |
| 0.5861 | 0.0033 | 0.0108 |
| 0.6323 | 0.0455 | 0.0103 |
| 0.6140 | 0.0781 | 0.0112 |
| 0.5656 | 0.0800 | 0.0062 |
| 0.4790 | 0.0854 | 0.0103 |
| 0.4662 | 0.0499 | 0.0086 |
| 0.6383 | 0.0230 | 0.0097 |
| 0.6505 | 0.0517 | 0.0099 |
| 0.4883 | 0.0030 | 0.0128 |
| 0.4988 | 0.0030 | 0.0146 |
| 0.1521 | 0.0001 | 0.0014 |
| 0.4765 | 0.0019 | 0.0042 |
| 0.5804 | 0.0781 | 0.0125 |
| 0.6102 | 0.1000 | 0.0124 |
| 0.7390 | 0.0039 | 0.0226 |
| 0.6161 | 0.0146 | 0.0166 |
| 0.6968 | 0.0036 | 0.0100 |

| | | |
|---|---|---|
| 0.4697 | 0.0284 | 0.0079 |
| 0.5932 | 0.0700 | 0.0140 |
| 0.5917 | 0.0231 | 0.0097 |
| 0.5847 | 0.0104 | 0.0095 |
| 0.6034 | 0.0191 | 0.0073 |
| 0.4882 | 0.0182 | 0.0070 |
| 0.5102 | 0.0177 | 0.0109 |
| 0.7920 | 0.0034 | 0.0167 |
| 0.5956 | 0.0062 | 0.0101 |
| 0.4931 | 0.0548 | 0.0107 |
| 0.3717 | 0.0018 | 0.0124 |
| 0.5314 | 0.0296 | 0.0123 |
| 0.5251 | 0.0045 | 0.0115 |
| 0.7233 | 0.0001 | 0.0060 |
| 0.5528 | 0.0024 | 0.0070 |
| 0.6294 | 0.0557 | 0.0132 |
| 0.6432 | 0.0032 | 0.0323 |
| 0.5953 | 0.0747 | 0.0090 |
| 0.6909 | 0.0208 | 0.0137 |
| 0.6283 | 0.0022 | 0.0074 |
| 0.7916 | 0.0048 | 0.0124 |
| 0.7221 | 0.0034 | 0.0051 |
| 0.5355 | 0.0728 | 0.0126 |
| 0.4531 | 0.0787 | 0.0088 |
| 0.6314 | 0.0220 | 0.0112 |
| 0.5149 | 0.0363 | 0.0146 |
| 0.5458 | 0.0051 | 0.0847 |
| 0.2006 | 0.0430 | 0.0048 |
| 0.5091 | 0.0507 | 0.0055 |
| 0.4377 | 0.0139 | 0.0118 |
| 0.7803 | 0.0027 | 0.0078 |
| 0.5048 | 0.0119 | 0.0114 |
| 0.6729 | 0.0005 | 0.0007 |
| 0.5690 | 0.0523 | 0.0170 |
| 0.6486 | 0.0037 | 0.0151 |
| 0.1973 | 0.0002 | 0.0032 |
| 0.5862 | 0.0112 | 0.0048 |
| 0.6139 | 0.0078 | 0.0022 |
| 0.5401 | 0.0217 | 0.0124 |
| 0.4230 | 0.0037 | 0.0319 |
| 0.6090 | 0.0366 | 0.0157 |
| 0.8400 | 0.0214 | 0.0126 |
| 0.6886 | 0.0518 | 0.0174 |
| 0.6336 | 0.0013 | 0.0106 |
| 0.6304 | 0.0874 | 0.0135 |
| 0.5663 | 0.0772 | 0.0100 |
| 0.5554 | 0.0935 | 0.0115 |
| 0.6012 | 0.0901 | 0.0152 |
| 0.6011 | 0.0800 | 0.0120 |
| 0.5176 | 0.0851 | 0.0103 |
| 0.6161 | 0.0804 | 0.0110 |

| | | |
|---|---|---|
| 0.5367 | 0.0829 | 0.0142 |
| 0.6402 | 0.0806 | 0.0116 |
| 0.7734 | 0.0491 | 0.0167 |
| 0.5086 | 0.0082 | 0.0067 |
| 0.4162 | 0.0029 | 0.0144 |
| 0.5497 | 0.0082 | 0.0146 |
| 0.5636 | 0.0550 | 0.0228 |
| 0.5883 | 0.0843 | 0.0148 |
| 0.5176 | 0.0525 | 0.0153 |
| 0.3993 | 0.0724 | 0.0063 |
| 0.5742 | 0.0028 | 0.0117 |
| 0.2947 | 0.0063 | 0.0140 |
| 0.5236 | 0.0069 | 0.0173 |
| 0.3505 | 0.0021 | 0.0220 |
| 0.5654 | 0.0072 | 0.0155 |
| 0.2362 | 0.0198 | 0.0139 |
| 0.2150 | 0.0045 | 0.0097 |
| 0.3966 | 0.0200 | 0.0170 |
| 0.2390 | 0.0011 | 0.0104 |
| 0.3311 | 0.0224 | 0.0217 |
| 0.2702 | 0.0532 | 0.0097 |
| 0.2963 | 0.0190 | 0.0147 |
| 0.3318 | 0.0198 | 0.0129 |
| 0.3668 | 0.0574 | 0.0147 |
| 0.4890 | 0.0547 | 0.0142 |
| 0.3928 | 0.0557 | 0.0146 |
| 0.3319 | 0.0551 | 0.0143 |
| 0.2896 | 0.0547 | 0.0134 |
| 0.2907 | 0.0196 | 0.0134 |
| 0.3886 | 0.0926 | 0.0107 |
| 0.6009 | 0.0880 | 0.0108 |
| 0.3991 | 0.0888 | 0.0159 |
| 0.6530 | 0.0937 | 0.0110 |
| 0.5496 | 0.0608 | 0.0127 |
| 0.3206 | 0.0090 | 0.0103 |
| 0.5148 | 0.1081 | 0.0187 |
| 0.5295 | 0.0898 | 0.0137 |
| 0.6515 | 0.0112 | 0.0197 |
| 0.6806 | 0.0942 | 0.0191 |
| 0.6368 | 0.0095 | 0.0130 |
| 0.5858 | 0.0914 | 0.0174 |
| 0.5247 | 0.0799 | 0.0144 |
| 0.2294 | 0.0047 | 0.0154 |
| 0.6817 | 0.0937 | 0.0064 |
| 0.6585 | 0.0937 | 0.0101 |
| 0.4399 | 0.0855 | 0.0083 |
| 0.5613 | 0.0661 | 0.0099 |
| 0.7787 | 0.0871 | 0.0109 |
| 0.5336 | 0.0092 | 0.0066 |
| 0.7506 | 0.0709 | 0.0090 |
| 0.3187 | 0.0598 | 0.0197 |

| | | |
|---|---|---|
| 0.4161 | 0.0586 | 0.0218 |
| 0.3536 | 0.0011 | 0.0198 |
| 0.3650 | 0.0022 | 0.0163 |
| 0.3195 | 0.0611 | 0.0204 |
| 0.2853 | 0.0895 | 0.0164 |
| 0.5341 | 0.0621 | 0.0208 |
| 0.3625 | 0.0354 | 0.0203 |
| 0.4688 | 0.0881 | 0.0137 |
| 0.3827 | 0.0942 | 0.0133 |
| 0.8341 | 0.0774 | 0.0105 |
| 0.6316 | 0.0983 | 0.0180 |
| 0.3620 | 0.0220 | 0.0238 |
| 0.4295 | 0.0565 | 0.0179 |
| 0.4808 | 0.0565 | 0.0218 |
| 0.3110 | 0.0846 | 0.0186 |
| 0.3182 | 0.0608 | 0.0165 |
| 0.4230 | 0.0527 | 0.0083 |
| 0.3629 | 0.0554 | 0.0172 |
| 0.4166 | 0.0881 | 0.0201 |
| 0.3280 | 0.0187 | 0.0161 |
| 0.5944 | 0.0935 | 0.0212 |
| 0.3877 | 0.0576 | 0.0183 |
| 0.4243 | 0.0527 | 0.0163 |
| 0.3323 | 0.1003 | 0.0142 |
| 0.6107 | 0.0942 | 0.0248 |
| 0.4591 | 0.0562 | 0.0157 |
| 0.7352 | 0.0026 | 0.0186 |
| 0.5188 | 0.0794 | 0.0148 |
| 0.4849 | 0.0281 | 0.0149 |
| 0.4818 | 0.1000 | 0.0098 |
| 0.6340 | 0.0824 | 0.0095 |
| 0.6001 | 0.0013 | 0.0092 |
| 0.5422 | 0.0820 | 0.0195 |
| 0.5714 | 0.0826 | 0.0118 |
| 0.4868 | 0.0519 | 0.0088 |
| 0.3910 | 0.0787 | 0.0046 |
| 0.3370 | 0.0813 | 0.0052 |
| 0.5915 | 0.1000 | 0.0114 |
| 0.5005 | 0.0750 | 0.0110 |
| 0.4914 | 0.0028 | 0.0183 |
| 0.6077 | 0.0787 | 0.0210 |
| 0.5945 | 0.0024 | 0.0057 |
| 0.5867 | 0.0014 | 0.0074 |
| 0.1347 | 0.0014 | 0.0030 |
| 0.5999 | 0.0094 | 0.0152 |
| 0.4576 | 0.0016 | 0.0239 |
| 0.6129 | 0.0100 | 0.0108 |
| 0.6503 | 0.0094 | 0.0109 |
| 0.4682 | 0.0089 | 0.0057 |
| 0.4626 | 0.0062 | 0.0034 |
| 0.6358 | 0.0562 | 0.0090 |

| | | |
|---|---|---|
| 0.5540 | 0.0283 | 0.0300 |
| 0.5806 | 0.0787 | 0.0111 |
| 0.4360 | 0.0787 | 0.0068 |
| 0.4322 | 0.0771 | 0.0079 |
| 0.6138 | 0.0557 | 0.0125 |
| 0.6464 | 0.0081 | 0.0172 |
| 0.6982 | 0.0285 | 0.0142 |
| 0.6169 | 0.0553 | 0.0136 |
| 0.5763 | 0.0801 | 0.0137 |
| 0.6951 | 0.0807 | 0.0146 |
| 0.7913 | 0.0807 | 0.0128 |
| 0.7035 | 0.0807 | 0.0137 |
| 0.7212 | 0.0097 | 0.0066 |
| 0.7383 | 0.0285 | 0.0120 |
| 0.6103 | 0.0311 | 0.0067 |
| 0.7322 | 0.0807 | 0.0108 |
| 0.6626 | 0.0314 | 0.0087 |
| 0.6127 | 0.0786 | 0.0163 |
| 0.8103 | 0.0314 | 0.0103 |
| 0.6472 | 0.0807 | 0.0129 |
| 0.7784 | 0.0312 | 0.0110 |
| 0.6905 | 0.0807 | 0.0142 |
| 0.5837 | 0.0677 | 0.0131 |
| 0.5894 | 0.0677 | 0.0093 |
| 0.4965 | 0.0787 | 0.0072 |
| 0.4323 | 0.0780 | 0.0094 |
| 0.6201 | 0.0737 | 0.0126 |
| 0.5345 | 0.0728 | 0.0118 |
| 0.5904 | 0.0786 | 0.0114 |
| 0.5623 | 0.0800 | 0.0111 |
| 0.5488 | 0.0143 | 0.0065 |
| 0.6469 | 0.0174 | 0.0141 |
| 0.5103 | 0.0012 | 0.0141 |
| 0.7810 | 0.0018 | 0.0128 |
| 0.6228 | 0.0193 | 0.0124 |
| 0.4759 | 0.0018 | 0.0147 |
| 0.5919 | 0.0019 | 0.0144 |
| 0.7680 | 0.0012 | 0.0133 |
| 0.8505 | 0.0018 | 0.0123 |
| 0.8387 | 0.0014 | 0.0217 |
| 0.6296 | 0.0021 | 0.0157 |
| 0.2875 | 0.0012 | 0.0130 |
| 0.2226 | 0.0015 | 0.0122 |
| 0.8949 | 0.0014 | 0.0121 |
| 0.2119 | 0.0572 | 0.0068 |
| 0.3283 | 0.0850 | 0.0120 |
| 0.7522 | 0.0220 | 0.0098 |
| 0.2028 | 0.0620 | 0.0082 |
| 0.5028 | 0.0078 | 0.0135 |
| 0.7652 | 0.0005 | 0.0152 |
| 0.5201 | 0.0011 | 0.0095 |

| | | |
|---|---|---|
| 0.8566 | 0.0190 | 0.0075 |
| 0.6828 | 0.0010 | 0.0149 |
| 0.4209 | 0.0526 | 0.0096 |
| 0.4138 | 0.0188 | 0.0151 |
| 0.5304 | 0.0051 | 0.0032 |
| 0.4339 | 0.0015 | 0.0057 |
| 0.4298 | 0.0170 | 0.0098 |
| 0.2028 | 0.0194 | 0.0058 |
| 0.8842 | 0.0043 | 0.0102 |
| 0.2957 | 0.0003 | 0.0043 |
| 0.2772 | 0.0212 | 0.0142 |
| 0.1724 | 0.0051 | 0.0082 |
| 0.4217 | 0.0011 | 0.0107 |
| 0.1886 | 0.0206 | 0.0192 |
| 0.1951 | 0.0008 | 0.0087 |
| 0.6347 | 0.0083 | 0.0082 |
| 0.6201 | 0.0032 | 0.0386 |
| 0.3910 | 0.0846 | 0.0057 |
| 0.2694 | 0.0025 | 0.0059 |
| 0.2660 | 0.0192 | 0.0081 |
| 0.3213 | 0.0038 | 0.0174 |
| 0.4312 | 0.0173 | 0.0214 |
| 0.5587 | 0.0644 | 0.0108 |
| 0.1005 | 0.0013 | 0.0098 |
| 0.3078 | 0.0032 | 0.0011 |
| 0.2563 | 0.0035 | 0.0116 |
| 0.3789 | 0.0512 | 0.0159 |
| 0.4538 | 0.0034 | 0.0137 |
| 0.3493 | 0.0117 | 0.0165 |
| 0.3089 | 0.0034 | 0.0144 |
| 0.4091 | 0.0098 | 0.0161 |
| 0.3794 | 0.0038 | 0.0151 |
| 0.3816 | 0.0124 | 0.0179 |
| 0.2785 | 0.0103 | 0.0125 |
| 0.3726 | 0.0115 | 0.0130 |
| 0.4858 | 0.0184 | 0.0318 |
| 0.2978 | 0.0031 | 0.0092 |
| 0.4727 | 0.0787 | 0.0062 |
| 0.5952 | 0.0728 | 0.0055 |
| 0.2699 | 0.0168 | 0.0101 |
| 0.7431 | 0.0044 | 0.0104 |
| 0.5859 | 0.0504 | 0.0159 |
| 0.0993 | 0.0504 | 0.0026 |
| 0.4830 | 0.0857 | 0.0062 |
| 0.5550 | 0.0787 | 0.0089 |
| 0.5304 | 0.0504 | 0.0143 |
| 0.8773 | 0.0172 | 0.0087 |
| 0.8153 | 0.0188 | 0.0176 |
| 0.4841 | 0.0015 | 0.0034 |
| 0.4385 | 0.0504 | 0.0092 |
| 0.7920 | 0.0543 | 0.0078 |

| | | |
|---|---|---|
| 0.7472 | 0.0190 | 0.0058 |
| 0.5711 | 0.0016 | 0.0058 |
| 0.5969 | 0.0787 | 0.0068 |
| 0.4656 | 0.0787 | 0.0144 |
| 0.8360 | 0.0659 | 0.0110 |
| 0.5524 | 0.0018 | 0.0071 |
| 0.4236 | 0.0171 | 0.0127 |
| 0.6046 | 0.0228 | 0.0222 |
| 0.5135 | 0.0026 | 0.0244 |
| 0.3602 | 0.0013 | 0.0013 |
| 0.6698 | 0.0015 | 0.0099 |
| 0.5468 | 0.0071 | 0.0308 |
| 0.4568 | 0.0035 | 0.0038 |
| 0.5068 | 0.0067 | 0.0263 |
| 0.6358 | 0.0054 | 0.0136 |
| 0.6096 | 0.0015 | 0.0021 |
| 0.7088 | 0.0010 | 0.0110 |
| 0.5254 | 0.0057 | 0.0224 |
| 0.3507 | 0.0050 | 0.0274 |
| 0.6271 | 0.0024 | 0.0133 |
| 0.7685 | 0.0504 | 0.0126 |
| 0.6389 | 0.0504 | 0.0186 |
| 0.5148 | 0.0155 | 0.0118 |
| 0.7411 | 0.0056 | 0.0093 |
| 0.7544 | 0.0056 | 0.0083 |
| 0.7595 | 0.0034 | 0.0048 |
| 0.6596 | 0.0054 | 0.0313 |
| 0.5897 | 0.0049 | 0.0084 |
| 0.5637 | 0.0059 | 0.0299 |
| 0.5998 | 0.0478 | 0.0107 |
| 0.6201 | 0.0504 | 0.0066 |
| 0.7224 | 0.0348 | 0.0283 |
| 0.6292 | 0.0787 | 0.0097 |
| 0.3415 | 0.0549 | 0.0173 |
| 0.7067 | 0.0253 | 0.0076 |
| 0.6367 | 0.0813 | 0.0092 |
| 0.3983 | 0.0504 | 0.0090 |
| 0.5168 | 0.0134 | 0.0069 |
| 0.5798 | 0.0294 | 0.0063 |
| 0.7265 | 0.0044 | 0.0070 |
| 0.5737 | 0.0035 | 0.0132 |
| 0.6535 | 0.0011 | 0.0097 |
| 0.4862 | 0.0005 | 0.0038 |
| 0.8999 | 0.0022 | 0.0168 |
| 0.7992 | 0.0021 | 0.0105 |
| 0.8238 | 0.0016 | 0.0183 |
| 0.7272 | 0.0011 | 0.0097 |
| 0.7761 | 0.0005 | 0.0079 |
| 0.8120 | 0.0014 | 0.0136 |
| 0.7070 | 0.0020 | 0.0161 |
| 0.6223 | 0.0019 | 0.0168 |

| | | |
|---|---|---|
| 0.8008 | 0.0012 | 0.0101 |
| 0.6515 | 0.0007 | 0.0076 |
| 0.6289 | 0.0018 | 0.0095 |
| 0.5539 | 0.0017 | 0.0127 |
| 0.4257 | 0.0005 | 0.0028 |
| 0.5809 | 0.0004 | 0.0078 |
| 0.6483 | 0.0010 | 0.0044 |
| 0.5381 | 0.0017 | 0.0060 |
| 0.5024 | 0.0017 | 0.0100 |
| 0.6074 | 0.0016 | 0.0146 |
| 0.6230 | 0.0014 | 0.0153 |
| 0.5139 | 0.0005 | 0.0077 |
| 0.6665 | 0.0013 | 0.0110 |
| 0.6928 | 0.0013 | 0.0129 |
| 0.6267 | 0.0018 | 0.0128 |
| 0.7787 | 0.0002 | 0.0067 |
| 0.8488 | 0.0013 | 0.0081 |
| 0.7091 | 0.0014 | 0.0106 |
| 0.6300 | 0.0003 | 0.0046 |
| 0.5582 | 0.0016 | 0.0048 |
| 0.5702 | 0.0017 | 0.0143 |
| 0.2011 | 0.0008 | 0.0052 |
| 0.2267 | 0.0012 | 0.0038 |
| 0.6999 | 0.0026 | 0.0146 |
| 0.3552 | 0.0008 | 0.0087 |
| 0.7107 | 0.0013 | 0.0068 |
| 0.7995 | 0.0006 | 0.0082 |
| 0.5982 | 0.0006 | 0.0075 |
| 0.6670 | 0.0024 | 0.0065 |
| 0.3608 | 0.0020 | 0.0115 |
| 0.5165 | 0.0019 | 0.0118 |
| 0.4998 | 0.0006 | 0.0064 |
| 0.5965 | 0.0020 | 0.0152 |
| 0.4884 | 0.0023 | 0.0133 |
| 0.5007 | 0.0016 | 0.0126 |
| 0.6866 | 0.0002 | 0.0044 |
| 0.4450 | 0.0010 | 0.0077 |
| 0.5343 | 0.0021 | 0.0188 |
| 0.6821 | 0.0008 | 0.0106 |
| 0.6530 | 0.0008 | 0.0098 |
| 0.6491 | 0.0011 | 0.0087 |
| 0.6632 | 0.0010 | 0.0078 |
| 0.6015 | 0.0003 | 0.0067 |
| 0.5487 | 0.0003 | 0.0074 |
| 0.5207 | 0.0014 | 0.0124 |
| 0.6880 | 0.0004 | 0.0048 |
| 0.4596 | 0.0003 | 0.0074 |
| 0.6463 | 0.0006 | 0.0073 |
| 0.7299 | 0.0010 | 0.0097 |
| NA | NA | 0.0050 |
| 0.4402 | 0.0014 | 0.0088 |

| | | |
|---|---|---|
| 0.9978 | 0.0001 | 0.0008 |
| 0.0481 | 0.0010 | 0.0012 |
| 0.6157 | 0.0014 | 0.0058 |
| NA | NA | 0.0050 |
| NA | NA | 0.0018 |
| NA | NA | 0.0151 |
| 0.9903 | 0.0014 | 0.0021 |
| 0.9763 | 0.0002 | 0.0011 |
| 0.5965 | 0.0021 | 0.0121 |
| 0.7441 | 0.0013 | 0.0112 |
| NA | NA | 0.0017 |
| 0.4179 | 0.0029 | 0.0072 |
| 0.5392 | 0.0021 | 0.0057 |
| 0.7159 | 0.0015 | 0.0165 |
| 0.8424 | 0.0010 | 0.0243 |
| 0.6938 | 0.0017 | 0.0108 |
| 0.6966 | 0.0024 | 0.0050 |
| 0.5747 | 0.0007 | 0.0027 |
| NA | NA | 0.0112 |
| NA | NA | 0.0097 |
| NA | NA | 0.0098 |
| 0.4721 | 0.0017 | 0.0176 |
| 0.9829 | 0.0009 | 0.0037 |
| NA | NA | 0.0107 |
| NA | NA | 0.0043 |
| NA | NA | 0.0000 |
| 0.0483 | 0.0009 | 0.0012 |
| NA | NA | 0.0000 |
| NA | NA | 0.0000 |
| 0.4772 | 0.0014 | 0.0054 |
| NA | NA | 0.0000 |
| NA | NA | 0.0151 |
| 0.9112 | 0.0008 | 0.0083 |
| 0.9983 | 0.0019 | 0.0010 |
| NA | NA | 0.0000 |
| NA | NA | 0.0002 |
| NA | NA | 0.0000 |
| 0.8894 | 0.0003 | 0.0110 |
| NA | NA | 0.0098 |
| NA | NA | 0.0000 |
| NA | NA | 0.0000 |
| 0.5434 | 0.0002 | 0.0203 |
| NA | NA | 0.0000 |
| NA | NA | 0.0000 |
| 0.3936 | 0.0008 | 0.0289 |
| NA | NA | 0.0000 |
| 0.5375 | 0.0010 | 0.0181 |
| 0.7327 | 0.0034 | 0.0197 |
| 0.6332 | 0.0069 | 0.0066 |
| NA | NA | 0.0061 |
| NA | NA | 0.0013 |

| | | |
|---|---|---|
| 0.9367 | 0.0003 | 0.0150 |
| 0.4047 | 0.0005 | 0.0225 |
| 0.9975 | 0.0001 | 0.0081 |
| NA | NA | NA |
| 0.7029 | 0.0016 | 0.0210 |
| 0.1642 | 0.0186 | 0.0092 |
| 0.3877 | 0.0164 | 0.0100 |
| 0.5934 | 0.0213 | 0.0141 |
| 0.5337 | 0.0577 | 0.0119 |
| 0.3853 | 0.0639 | 0.0194 |
| 0.5159 | 0.0570 | 0.0129 |
| 0.7115 | 0.0021 | 0.0149 |
| 0.9995 | 0.0009 | 0.0189 |
| 0.9615 | 0.0010 | 0.0251 |
| 0.8272 | 0.0010 | 0.0249 |
| 0.8211 | 0.0003 | 0.0083 |
| NA | NA | 0.0046 |
| 0.8426 | 0.0009 | 0.0154 |
| 0.3086 | 0.0012 | 0.0177 |
| 0.7438 | 0.0004 | 0.0155 |
| 0.4190 | 0.0189 | 0.0083 |
| 0.5143 | 0.0381 | 0.0150 |
| 0.5920 | 0.0184 | 0.0138 |
| 0.8810 | 0.0199 | 0.0193 |
| 0.8032 | 0.0220 | 0.0208 |
| NA | NA | 0.0207 |
| 0.2316 | 0.0008 | 0.0137 |
| 0.8786 | 0.0000 | 0.0215 |
| 0.9941 | 0.0005 | 0.0266 |
| 0.1753 | 0.0015 | 0.0081 |
| NA | NA | 0.0105 |
| NA | NA | 0.0000 |
| 0.9939 | 0.0001 | 0.0131 |
| 0.4990 | 0.0004 | 0.0138 |
| NA | NA | 0.0000 |
| 0.6288 | 0.0204 | 0.0114 |
| 0.4987 | 0.0223 | 0.0156 |
| 0.7864 | 0.0200 | 0.0102 |
| 0.4748 | 0.0016 | 0.0152 |
| 0.6961 | 0.0184 | 0.0196 |
| 0.1883 | 0.0009 | 0.0157 |
| 0.6267 | 0.0214 | 0.0238 |
| 0.5804 | 0.0161 | 0.0164 |
| 0.8361 | 0.0223 | 0.0236 |
| 0.5979 | 0.0030 | 0.0244 |
| 0.7372 | 0.0025 | 0.0256 |
| 0.3829 | 0.0012 | 0.0169 |
| 0.7532 | 0.0010 | 0.0232 |
| 0.5446 | 0.0223 | 0.0182 |
| 0.7210 | 0.0223 | 0.0257 |
| 0.5334 | 0.0260 | 0.0315 |

| | | |
|---|---|---|
| 0.5523 | 0.0227 | 0.0207 |
| 0.5522 | 0.0206 | 0.0226 |
| 0.6633 | 0.0204 | 0.0221 |
| 0.4027 | 0.0204 | 0.0196 |
| 0.3851 | 0.0199 | 0.0197 |
| 0.7714 | 0.0221 | 0.0200 |
| 0.6193 | 0.0207 | 0.0252 |
| 0.7187 | 0.0223 | 0.0204 |
| 0.5693 | 0.0227 | 0.0258 |
| 0.6678 | 0.0221 | 0.0256 |
| 0.5881 | 0.0204 | 0.0258 |
| 0.6686 | 0.0018 | 0.0180 |
| 0.3416 | 0.0191 | 0.0184 |
| 0.5503 | 0.0021 | 0.0247 |
| 0.9941 | 0.0002 | 0.0008 |
| 0.5233 | 0.0038 | 0.0081 |
| 0.9062 | 0.0016 | 0.0201 |
| 0.6149 | 0.0024 | 0.0287 |
| NA | NA | 0.0000 |
| 0.4664 | 0.0003 | 0.0069 |
| 0.4467 | 0.0066 | 0.0114 |
| 0.7022 | 0.0076 | 0.0056 |
| NA | NA | 0.0053 |
| NA | NA | NA |
| 0.2465 | 0.0052 | 0.0034 |
| NA | NA | 0.0010 |
| 0.7406 | 0.0105 | 0.0212 |
| NA | NA | 0.0123 |
| 0.9931 | 0.0003 | 0.0141 |
| NA | NA | 0.0000 |
| NA | NA | 0.0000 |
| NA | NA | 0.0221 |
| NA | NA | 0.0000 |
| NA | NA | 0.0000 |
| 0.8830 | 0.0010 | 0.0159 |
| 0.3495 | 0.0606 | 0.0117 |
| 0.4364 | 0.0105 | 0.0133 |
| 0.9055 | 0.0307 | 0.0153 |
| NA | NA | NA |
| 0.5513 | 0.0003 | 0.0246 |
| 0.5885 | 0.0233 | 0.0293 |
| 0.5790 | 0.0095 | 0.0127 |
| 0.6129 | 0.0216 | 0.0166 |
| NA | NA | 0.0007 |
| 0.6019 | 0.0220 | 0.0164 |
| 0.7167 | 0.0214 | 0.0207 |
| 0.5772 | 0.0243 | 0.0269 |
| 0.6084 | 0.0554 | 0.0196 |
| 0.4660 | 0.0050 | 0.0125 |
| 0.7506 | 0.0233 | 0.0163 |
| 0.4695 | 0.0216 | 0.0256 |

| | | |
|---|---|---|
| 0.5032 | 0.0015 | 0.0201 |
| 0.5260 | 0.0022 | 0.0200 |
| 0.6660 | 0.0022 | 0.0141 |
| 0.0333 | 0.0014 | 0.0005 |
| 0.6127 | 0.0079 | 0.0089 |
| 0.6955 | 0.0017 | 0.0307 |
| 0.9316 | 0.0019 | 0.0233 |
| NA | NA | 0.0000 |
| 0.0174 | 0.0027 | 0.0002 |
| 0.3478 | 0.0082 | 0.0008 |
| 0.6668 | 0.0076 | 0.0083 |
| 0.9992 | 0.0067 | 0.0054 |
| NA | NA | NA |
| NA | NA | NA |
| 0.3534 | 0.0066 | 0.0019 |
| 0.3846 | 0.0076 | 0.0086 |
| NA | NA | 0.0103 |
| NA | NA | 0.0223 |
| NA | NA | 0.0003 |
| NA | NA | 0.0021 |
| 0.8555 | 0.0105 | 0.0155 |
| 0.6565 | 0.0105 | 0.0060 |
| NA | NA | NA |
| 0.8045 | 0.0876 | 0.0122 |
| 0.7633 | 0.0876 | 0.0098 |
| 0.7760 | 0.0805 | 0.0135 |
| 0.8179 | 0.0876 | 0.0155 |
| 0.7542 | 0.0876 | 0.0154 |
| 0.7797 | 0.0876 | 0.0131 |
| 0.7846 | 0.0820 | 0.0142 |
| 0.7483 | 0.0820 | 0.0132 |
| 0.7198 | 0.0820 | 0.0144 |
| 0.7659 | 0.0820 | 0.0153 |
| 0.7994 | 0.0892 | 0.0148 |
| 0.7800 | 0.0905 | 0.0126 |
| 0.8171 | 0.0892 | 0.0155 |
| 0.8340 | 0.0820 | 0.0145 |
| 0.8221 | 0.0868 | 0.0141 |
| 0.7399 | 0.0805 | 0.0141 |
| 0.7846 | 0.0820 | 0.0132 |
| 0.8143 | 0.0820 | 0.0170 |
| 0.8192 | 0.0805 | 0.0139 |
| 0.7292 | 0.0805 | 0.0102 |
| 0.8253 | 0.0805 | 0.0124 |
| 0.8415 | 0.0820 | 0.0135 |
| 0.7765 | 0.0881 | 0.0135 |
| 0.7812 | 0.0881 | 0.0121 |
| 0.7922 | 0.0786 | 0.0095 |
| 0.8098 | 0.0786 | 0.0100 |
| 0.8153 | 0.0820 | 0.0126 |
| 0.7802 | 0.0808 | 0.0129 |

| | | |
|---|---|---|
| 0.7666 | 0.0807 | 0.0122 |
| 0.8464 | 0.0786 | 0.0141 |
| 0.5184 | 0.0085 | 0.0183 |
| 0.5387 | 0.0820 | 0.0116 |
| 0.6036 | 0.0824 | 0.0141 |
| 0.5804 | 0.0106 | 0.0082 |
| 0.5278 | 0.0095 | 0.0099 |
| 0.5426 | 0.0786 | 0.0108 |
| 0.5585 | 0.0513 | 0.0112 |
| 0.4854 | 0.0497 | 0.0103 |
| 0.6485 | 0.0824 | 0.0141 |
| 0.7726 | 0.0200 | 0.0109 |
| 0.5331 | 0.0328 | 0.0128 |
| 0.5856 | 0.0339 | 0.0131 |
| 0.6129 | 0.0211 | 0.0132 |
| 0.6189 | 0.0213 | 0.0133 |
| 0.6444 | 0.0824 | 0.0126 |
| 0.6143 | 0.0837 | 0.0100 |
| 0.6303 | 0.0209 | 0.0132 |
| 0.6542 | 0.0820 | 0.0133 |
| 0.5879 | 0.0522 | 0.0138 |
| 0.6066 | 0.0820 | 0.0125 |
| 0.6459 | 0.0831 | 0.0114 |
| 0.5363 | 0.0529 | 0.0123 |
| 0.5870 | 0.0831 | 0.0126 |
| 0.6215 | 0.0100 | 0.0151 |
| 0.5785 | 0.0891 | 0.0141 |
| 0.5909 | 0.0031 | 0.0134 |
| 0.5421 | 0.0031 | 0.0152 |
| 0.5713 | 0.0030 | 0.0150 |
| 0.6410 | 0.0034 | 0.0135 |
| 0.6328 | 0.0035 | 0.0116 |
| 0.4928 | 0.0400 | 0.0101 |
| 0.2530 | 0.0672 | 0.0089 |
| 0.4632 | 0.0027 | 0.0115 |
| 0.5699 | 0.0191 | 0.0130 |
| 0.6961 | 0.0195 | 0.0108 |
| 0.5196 | 0.0020 | 0.0097 |
| 0.6523 | 0.0024 | 0.0099 |
| 0.4010 | 0.0016 | 0.0098 |
| 0.5985 | 0.0787 | 0.0095 |
| 0.5335 | 0.0031 | 0.0080 |
| 0.8422 | 0.0072 | 0.0050 |
| 0.5715 | 0.0504 | 0.0047 |
| 0.7039 | 0.0236 | 0.0142 |
| 0.7216 | 0.0164 | 0.0261 |
| 0.6072 | 0.0057 | 0.0145 |
| 0.4484 | 0.0164 | 0.0227 |
| 0.6086 | 0.0035 | 0.0306 |
| 0.5201 | 0.0174 | 0.0092 |
| 0.7126 | 0.0250 | 0.0077 |

| | | |
|---|---|---|
| 0.3725 | 0.0010 | 0.0080 |
| 0.5733 | 0.0045 | 0.0168 |
| 0.6728 | 0.0065 | 0.0252 |
| 0.7848 | 0.0089 | 0.0087 |
| 0.3942 | 0.0781 | 0.0030 |
| 0.6815 | 0.0179 | 0.0102 |
| 0.5841 | 0.0639 | 0.0105 |
| 0.6226 | 0.0024 | 0.0103 |
| 0.4335 | 0.0004 | 0.0032 |
| 0.6062 | 0.0476 | 0.0096 |
| 0.5540 | 0.0813 | 0.0088 |
| 0.5285 | 0.0813 | 0.0122 |
| 0.6047 | 0.0769 | 0.0103 |
| 0.5466 | 0.0061 | 0.0072 |
| 0.3869 | 0.0864 | 0.0044 |
| 0.6861 | 0.0843 | 0.0142 |
| 0.5674 | 0.0069 | 0.0100 |
| 0.5997 | 0.0747 | 0.0128 |
| 0.5883 | 0.0481 | 0.0112 |
| 0.5007 | 0.0876 | 0.0115 |
| 0.5456 | 0.0787 | 0.0126 |
| 0.6033 | 0.0780 | 0.0078 |
| 0.7563 | 0.0014 | 0.0106 |
| 0.1485 | 0.0504 | 0.0019 |
| 0.6770 | 0.0780 | 0.0058 |
| 0.5676 | 0.0525 | 0.0148 |
| 0.4020 | 0.0537 | 0.0061 |
| 0.3256 | 0.0003 | 0.0008 |
| 0.4415 | 0.0021 | 0.0127 |
| 0.6874 | 0.0177 | 0.0048 |
| 0.5452 | 0.0025 | 0.0126 |
| 0.5841 | 0.0491 | 0.0073 |
| 0.5170 | 0.0787 | 0.0130 |
| 0.5903 | 0.0723 | 0.0092 |
| 0.4025 | 0.0787 | 0.0109 |
| 0.6107 | 0.0837 | 0.0101 |
| 0.4392 | 0.0681 | 0.0118 |
| 0.6176 | 0.0813 | 0.0096 |
| 0.5115 | 0.0787 | 0.0099 |
| 0.7063 | 0.0026 | 0.0154 |
| 0.5234 | 0.0824 | 0.0094 |
| 0.3908 | 0.0737 | 0.0076 |
| 0.5542 | 0.0504 | 0.0129 |
| 0.5314 | 0.0787 | 0.0094 |
| 0.4090 | 0.0468 | 0.0064 |
| 0.5157 | 0.0021 | 0.0074 |
| 0.5334 | 0.0507 | 0.0041 |
| 0.5206 | 0.0507 | 0.0104 |
| 0.7040 | 0.0507 | 0.0078 |
| 0.4638 | 0.0179 | 0.0148 |
| 0.6453 | 0.0124 | 0.0078 |

| | | |
|---|---|---|
| 0.4938 | 0.0733 | 0.0131 |
| 0.5704 | 0.0504 | 0.0114 |
| 0.4384 | 0.0528 | 0.0118 |
| 0.6146 | 0.0781 | 0.0118 |
| 0.6158 | 0.0332 | 0.0144 |
| 0.5298 | 0.0767 | 0.0069 |
| 0.5346 | 0.0706 | 0.0087 |
| 0.5577 | 0.0434 | 0.0074 |
| 0.2853 | 0.0022 | 0.0090 |
| 0.3865 | 0.0092 | 0.0029 |
| 0.3377 | 0.0521 | 0.0090 |
| 0.4692 | 0.0228 | 0.0186 |
| 0.6332 | 0.0020 | 0.0185 |
| 0.6442 | 0.0749 | 0.0121 |
| 0.5643 | 0.0088 | 0.0064 |
| 0.5470 | 0.0507 | 0.0071 |
| 0.4279 | 0.0021 | 0.0191 |
| 0.4556 | 0.0077 | 0.0144 |
| 0.5089 | 0.0042 | 0.0154 |
| 0.3536 | 0.0534 | 0.0049 |
| 0.6251 | 0.0021 | 0.0239 |
| 0.2755 | 0.0412 | 0.0122 |
| 0.5990 | 0.0043 | 0.0074 |
| 0.6821 | 0.0805 | 0.0133 |
| 0.6165 | 0.0280 | 0.0121 |
| 0.4517 | 0.0787 | 0.0050 |
| 0.5593 | 0.0017 | 0.0050 |
| 0.5678 | 0.0084 | 0.0079 |
| 0.6225 | 0.0787 | 0.0095 |
| 0.6692 | 0.0242 | 0.0084 |
| 0.6637 | 0.0036 | 0.0100 |
| 0.8199 | 0.0145 | 0.0162 |
| 0.4586 | 0.0020 | 0.0122 |
| 0.6535 | 0.0166 | 0.0193 |
| 0.5628 | 0.0243 | 0.0151 |
| 0.5305 | 0.0006 | 0.0145 |
| 0.6250 | 0.0010 | 0.0173 |
| 0.8484 | 0.0002 | 0.0055 |
| 0.7886 | 0.0010 | 0.0097 |
| 0.5497 | 0.0011 | 0.0069 |
| 0.8072 | 0.0021 | 0.0092 |
| 0.5916 | 0.0026 | 0.0157 |
| 0.9178 | 0.0013 | 0.0046 |
| 0.7695 | 0.0012 | 0.0157 |
| 0.5934 | 0.0025 | 0.0117 |
| 0.5593 | 0.0024 | 0.0169 |
| 0.6886 | 0.0126 | 0.0140 |
| 0.8493 | 0.0147 | 0.0125 |
| 0.7526 | 0.0018 | 0.0178 |
| 0.3025 | 0.0017 | 0.0064 |
| 0.5029 | 0.0145 | 0.0058 |

| | | |
|---|---|---|
| 0.9619 | 0.0000 | 0.0024 |
| 0.4223 | 0.0020 | 0.0090 |
| 0.9662 | 0.0010 | 0.0029 |
| 0.7909 | 0.0024 | 0.0095 |
| 0.5271 | 0.0008 | 0.0069 |
| 0.5303 | 0.0007 | 0.0044 |
| 0.3735 | 0.0006 | 0.0042 |
| 0.7479 | 0.0006 | 0.0038 |
| 0.3559 | 0.0071 | 0.0045 |
| 0.4643 | 0.0012 | 0.0077 |
| 0.7314 | 0.0007 | 0.0107 |
| 0.2827 | 0.0005 | 0.0116 |
| 0.3621 | 0.0006 | 0.0085 |
| 0.5896 | 0.0020 | 0.0072 |
| 0.8114 | 0.0010 | 0.0120 |
| 0.3136 | 0.0002 | 0.0034 |
| 0.8077 | 0.0020 | 0.0205 |
| 0.3341 | 0.0013 | 0.0254 |
| 0.4505 | 0.0015 | 0.0090 |
| 0.2201 | 0.0011 | 0.0092 |
| 0.8739 | 0.0028 | 0.0152 |
| 0.3505 | 0.0015 | 0.0173 |
| 0.6558 | 0.0015 | 0.0139 |
| 0.5834 | 0.0020 | 0.0209 |
| 0.6249 | 0.0015 | 0.0070 |
| 0.7292 | 0.0097 | 0.0194 |
| 0.5705 | 0.0097 | 0.0169 |
| 0.1495 | 0.0097 | 0.0162 |
| 0.4431 | 0.0103 | 0.0156 |
| 0.7480 | 0.0098 | 0.0153 |
| 0.5364 | 0.0082 | 0.0092 |
| 0.4853 | 0.0020 | 0.0207 |
| 0.5197 | 0.0009 | 0.0081 |
| 0.8443 | 0.0014 | 0.0239 |
| 0.3140 | 0.0013 | 0.0051 |
| 0.6975 | 0.0018 | 0.0164 |
| 0.9166 | 0.0012 | 0.0070 |
| 0.7161 | 0.0010 | 0.0139 |
| 0.4639 | 0.0018 | 0.0062 |
| 0.3496 | 0.0097 | 0.0230 |
| 0.6072 | 0.0103 | 0.0179 |
| 0.8497 | 0.0020 | 0.0206 |
| 0.9490 | 0.0010 | 0.0042 |
| 0.5370 | 0.0020 | 0.0120 |
| 0.6086 | 0.0097 | 0.0053 |
| 0.3677 | 0.0024 | 0.0015 |
| 0.4069 | 0.0103 | 0.0107 |
| 0.5236 | 0.0008 | 0.0101 |
| 0.1802 | 0.0010 | 0.0047 |
| 0.4059 | 0.0055 | 0.0046 |
| 0.6621 | 0.0428 | 0.0042 |

| | | |
|---|---|---|
| 0.4569 | 0.0604 | 0.0181 |
| 0.7652 | 0.0225 | 0.0087 |
| 0.5764 | 0.0017 | 0.0027 |
| 0.8005 | 0.0604 | 0.0241 |
| 0.3375 | 0.0587 | 0.0163 |
| 0.5283 | 0.0216 | 0.0117 |
| 0.6387 | 0.0229 | 0.0170 |
| 0.6384 | 0.0198 | 0.0134 |
| 0.7538 | 0.0004 | 0.0046 |
| 0.5978 | 0.0242 | 0.0154 |
| 0.6802 | 0.0103 | 0.0138 |
| 0.4165 | 0.0028 | 0.0112 |
| 0.5110 | 0.0097 | 0.0189 |
| 0.4266 | 0.0103 | 0.0139 |
| 0.8468 | 0.0015 | 0.0279 |
| 0.6118 | 0.0010 | 0.0045 |
| 0.6651 | 0.0014 | 0.0237 |
| NA | NA | NA |
| 0.2179 | 0.0011 | 0.0067 |
| 0.3154 | 0.0012 | 0.0046 |
| NA | NA | 0.0070 |
| NA | NA | NA |
| NA | NA | NA |
| 0.6479 | 0.0058 | 0.0054 |
| NA | NA | 0.0017 |
| 0.7606 | 0.0019 | 0.0192 |
| NA | NA | NA |
| NA | NA | NA |
| 0.5761 | 0.0103 | 0.0010 |
| 0.5896 | 0.0103 | 0.0066 |
| 0.5456 | 0.0005 | 0.0031 |
| 0.5524 | 0.0002 | 0.0007 |
| NA | NA | 0.0000 |
| 0.5322 | 0.0097 | 0.0031 |
| 0.8525 | 0.0020 | 0.0259 |
| 0.6695 | 0.0048 | 0.0047 |
| 0.5133 | 0.0025 | 0.0030 |
| 0.9630 | 0.0006 | 0.0174 |
| 0.5676 | 0.0045 | 0.0036 |
| 0.6354 | 0.0105 | 0.0095 |
| 0.1727 | 0.0027 | 0.0046 |
| 0.5349 | 0.0105 | 0.0088 |
| 0.6064 | 0.0020 | 0.0196 |
| 0.7837 | 0.0076 | 0.0114 |
| 0.4376 | 0.0076 | 0.0096 |
| 0.7125 | 0.0076 | 0.0048 |
| 0.6746 | 0.0079 | 0.0017 |
| NA | NA | 0.0019 |
| 0.6823 | 0.0029 | 0.0152 |
| 0.5861 | 0.0203 | 0.0095 |
| 0.6158 | 0.0200 | 0.0115 |

| | | |
|---|---|---|
| 0.6593 | 0.0556 | 0.0093 |
| 0.6281 | 0.0556 | 0.0131 |
| 0.6686 | 0.0556 | 0.0133 |
| 0.8105 | 0.0917 | 0.0083 |
| 0.7331 | 0.0850 | 0.0163 |
| 0.6667 | 0.0227 | 0.0092 |
| 0.5757 | 0.0556 | 0.0159 |
| 0.5452 | 0.0227 | 0.0148 |
| 0.6472 | 0.0212 | 0.0131 |
| 0.4963 | 0.0176 | 0.0122 |
| 0.6786 | 0.0556 | 0.0148 |
| 0.6169 | 0.0571 | 0.0075 |
| 0.6270 | 0.0227 | 0.0125 |
| 0.5316 | 0.0212 | 0.0168 |
| 0.7047 | 0.0227 | 0.0082 |
| 0.7790 | 0.0227 | 0.0047 |
| 0.6142 | 0.0556 | 0.0082 |
| 0.7354 | 0.0372 | 0.0041 |
| 0.7257 | 0.0673 | 0.0126 |
| 0.6854 | 0.0571 | 0.0104 |
| 0.7093 | 0.0673 | 0.0088 |
| 0.6674 | 0.0362 | 0.0061 |
| 0.7135 | 0.0241 | 0.0139 |
| 0.7016 | 0.0673 | 0.0098 |
| 0.8346 | 0.0673 | 0.0033 |
| 0.7466 | 0.0234 | 0.0044 |
| 0.5677 | 0.0234 | 0.0112 |
| 0.7092 | 0.0673 | 0.0105 |
| 0.7310 | 0.0227 | 0.0133 |
| 0.7577 | 0.0216 | 0.0161 |
| 0.6579 | 0.0673 | 0.0090 |
| 0.6879 | 0.0691 | 0.0141 |
| 0.8242 | 0.0660 | 0.0115 |
| 0.7050 | 0.0673 | 0.0105 |
| 0.5564 | 0.0691 | 0.0150 |
| 0.7160 | 0.0673 | 0.0098 |
| 0.6341 | 0.0660 | 0.0083 |
| 0.6498 | 0.0660 | 0.0069 |
| 0.6963 | 0.0655 | 0.0095 |
| 0.6153 | 0.0673 | 0.0148 |
| 0.6682 | 0.1031 | 0.0060 |
| 0.6953 | 0.0219 | 0.0085 |
| 0.6276 | 0.0675 | 0.0119 |
| 0.5772 | 0.0218 | 0.0128 |
| 0.6494 | 0.0673 | 0.0057 |
| 0.5822 | 0.0673 | 0.0090 |
| 0.6353 | 0.0252 | 0.0123 |
| 0.6554 | 0.0212 | 0.0065 |
| 0.6538 | 0.0556 | 0.0152 |
| 0.5848 | 0.0657 | 0.0121 |
| 0.6295 | 0.0212 | 0.0154 |

| | | |
|---|---|---|
| 0.6864 | 0.0212 | 0.0147 |
| 0.1872 | 0.0497 | 0.0130 |
| 0.6880 | 0.0490 | 0.0157 |
| 0.6320 | 0.0593 | 0.0138 |
| 0.7804 | 0.0221 | 0.0181 |
| 0.7573 | 0.0195 | 0.0174 |
| 0.5273 | 0.0189 | 0.0136 |
| 0.6915 | 0.0204 | 0.0198 |
| 0.2510 | 0.0673 | 0.0190 |
| 0.6234 | 0.0091 | 0.0181 |
| 0.2000 | 0.0227 | 0.0172 |
| 0.8070 | 0.0222 | 0.0363 |
| 0.3142 | 0.0227 | 0.0331 |
| 0.7964 | 0.0610 | 0.0206 |
| 0.4057 | 0.0216 | 0.0119 |
| 0.5825 | 0.0229 | 0.0165 |
| 0.6030 | 0.0212 | 0.0155 |
| 0.8193 | 0.0214 | 0.0235 |
| 0.7174 | 0.0225 | 0.0204 |
| 0.5288 | 0.0088 | 0.0231 |
| 0.3989 | 0.0022 | 0.0063 |
| 0.0539 | 0.0362 | 0.0096 |
| 0.6984 | 0.0346 | 0.0137 |
| 0.5210 | 0.0024 | 0.0135 |
| 0.5068 | 0.0346 | 0.0145 |
| 0.1445 | 0.0346 | 0.0136 |
| 0.6458 | 0.0366 | 0.0183 |
| 0.5811 | 0.0026 | 0.0149 |
| 0.2859 | 0.0025 | 0.0195 |
| 0.5610 | 0.0021 | 0.0172 |
| 0.5133 | 0.0346 | 0.0152 |
| 0.1647 | 0.0320 | 0.0082 |
| 0.8134 | 0.0026 | 0.0084 |
| 0.7909 | 0.0030 | 0.0224 |
| 0.1784 | 0.0021 | 0.0180 |
| 0.6515 | 0.0020 | 0.0171 |
| 0.6816 | 0.0021 | 0.0194 |
| 0.6390 | 0.0020 | 0.0153 |
| 0.1692 | 0.0023 | 0.0139 |
| 0.6969 | 0.0020 | 0.0208 |
| 0.5415 | 0.0020 | 0.0184 |
| 0.7018 | 0.0020 | 0.0211 |
| 0.2432 | 0.0024 | 0.0176 |
| 0.7073 | 0.0024 | 0.0206 |
| 0.2288 | 0.0026 | 0.0196 |
| 0.8311 | 0.0020 | 0.0214 |
| 0.2558 | 0.0023 | 0.0254 |
| 0.2074 | 0.0090 | 0.0210 |
| 0.2362 | 0.0031 | 0.0158 |
| 0.6912 | 0.0017 | 0.0158 |
| 0.2706 | 0.0028 | 0.0202 |

| | | |
|---|---|---|
| 0.2610 | 0.0098 | 0.0213 |
| 0.2224 | 0.0016 | 0.0146 |
| 0.2867 | 0.0036 | 0.0146 |
| 0.2550 | 0.0017 | 0.0179 |
| 0.7370 | 0.0056 | 0.0173 |
| 0.2231 | 0.0157 | 0.0155 |
| 0.3060 | 0.0199 | 0.0155 |
| 0.5690 | 0.0155 | 0.0122 |
| 0.7335 | 0.0198 | 0.0164 |
| 0.3777 | 0.0532 | 0.0181 |
| 0.6304 | 0.0190 | 0.0122 |
| 0.6454 | 0.0020 | 0.0145 |
| 0.6398 | 0.0554 | 0.0170 |
| 0.7237 | 0.0174 | 0.0143 |
| 0.3229 | 0.0171 | 0.0159 |
| 0.6624 | 0.0011 | 0.0072 |
| 0.1952 | 0.0086 | 0.0092 |
| 0.2211 | 0.0824 | 0.0137 |
| 0.2341 | 0.0300 | 0.0102 |
| 0.5713 | 0.0154 | 0.0187 |
| 0.6027 | 0.0735 | 0.0089 |
| 0.3716 | 0.0198 | 0.0101 |
| 0.3893 | 0.0152 | 0.0139 |
| 0.3625 | 0.0175 | 0.0096 |
| 0.3011 | 0.0203 | 0.0117 |
| 0.4404 | 0.0161 | 0.0075 |
| 0.3809 | 0.0205 | 0.0101 |
| 0.2745 | 0.0016 | 0.0098 |
| 0.3717 | 0.0203 | 0.0110 |
| 0.6248 | 0.0126 | 0.0094 |
| 0.2703 | 0.0126 | 0.0092 |
| 0.2454 | 0.0205 | 0.0108 |
| 0.2961 | 0.0160 | 0.0115 |
| 0.3014 | 0.0051 | 0.0149 |
| 0.2045 | 0.0017 | 0.0198 |
| 0.7263 | 0.0053 | 0.0204 |
| 0.8255 | 0.0017 | 0.0063 |
| 0.2360 | 0.0054 | 0.0122 |
| 0.6109 | 0.0053 | 0.0137 |
| 0.8925 | 0.0054 | 0.0121 |
| 0.1423 | 0.0057 | 0.0122 |
| 0.8200 | 0.0047 | 0.0122 |
| 0.7701 | 0.0891 | 0.0136 |
| 0.5952 | 0.0057 | 0.0190 |
| 0.6896 | 0.0301 | 0.0176 |
| 0.7625 | 0.0057 | 0.0167 |
| 0.2865 | 0.0570 | 0.0209 |
| 0.2466 | 0.0055 | 0.0175 |
| 0.2921 | 0.0019 | 0.0103 |
| 0.6768 | 0.0830 | 0.0125 |
| 0.5983 | 0.0067 | 0.0165 |

| | | |
|---|---|---|
| 0.1728 | 0.0693 | 0.0127 |
| 0.3351 | 0.0811 | 0.0150 |
| 0.5441 | 0.0055 | 0.0170 |
| 0.2791 | 0.0799 | 0.0168 |
| 0.4674 | 0.0174 | 0.0087 |
| 0.5829 | 0.0198 | 0.0131 |
| 0.1581 | 0.0688 | 0.0092 |
| 0.5718 | 0.0682 | 0.0096 |
| 0.1787 | 0.0693 | 0.0128 |
| NA | NA | 0.0000 |
| 0.2443 | 0.0043 | 0.0167 |
| 0.6965 | 0.0568 | 0.0076 |
| 0.5028 | 0.0011 | 0.0002 |
| 0.8892 | 0.0005 | 0.0160 |
| 0.7708 | 0.0198 | 0.0172 |
| 0.8518 | 0.0022 | 0.0216 |
| 0.5050 | 0.0122 | 0.0163 |
| 0.6665 | 0.0283 | 0.0152 |
| 0.6463 | 0.0013 | 0.0203 |
| NA | NA | 0.0285 |
| 0.5242 | 0.0009 | 0.0201 |
| 0.4832 | 0.0174 | 0.0134 |
| 0.5316 | 0.0174 | 0.0120 |
| 0.7317 | 0.0517 | 0.0108 |
| 0.6864 | 0.0895 | 0.0106 |
| 0.6132 | 0.0176 | 0.0086 |
| 0.8107 | 0.0169 | 0.0102 |
| 0.6393 | 0.0176 | 0.0114 |
| 0.5840 | 0.0172 | 0.0091 |
| 0.6990 | 0.0517 | 0.0065 |
| 0.5530 | 0.0022 | 0.0093 |
| 0.4234 | 0.0198 | 0.0045 |
| 0.5369 | 0.0198 | 0.0092 |
| 0.6242 | 0.0512 | 0.0131 |
| 0.6653 | 0.0786 | 0.0108 |
| 0.5722 | 0.0075 | 0.0097 |
| 0.5716 | 0.0207 | 0.0123 |
| 0.6229 | 0.0500 | 0.0075 |
| 0.8238 | 0.0498 | 0.0089 |
| 0.6365 | 0.0136 | 0.0053 |
| 0.5799 | 0.0589 | 0.0125 |
