# Supplementary material for: Are we restoring functional fens? – The outcomes of restoration projects in fens re-analysed with plant functional traits
Source: PLoS One. 2019 Apr 24;14(4):e0215645. doi: 10.1371/journal.pone.0215645 (PMC6481837; doi:10.1371/journal.pone.0215645)
Supplement: S3 File — (PDF) [file pone.0215645.s012.pdf]

RELEVE_NR	YEAR1	SITE3	TREATMENT	1 ch	1 ch.s	2 sla	2 sla.s	3 ldmc	3 ldmc.s	4 cs	5 fl	5 fl.s
501	2011	MIRE1	MIRE	0.4625	0.1691	21.4973	0.4031	0.2197	0.4068	0.6806	5.4800	0.6400
508	2011	MIRE3	MIRE	0.4108	0.1554	21.8862	0.4092	0.2515	0.4817	0.6875	5.2778	0.6111
512	2011	MIRE5	MIRE	0.7815	0.2413	26.8430	0.4269	0.2013	0.3636	0.6667	5.7692	0.6813
517	2011	MIRE8	MIRE	0.4267	0.1641	22.6851	0.4178	0.2085	0.3806	0.7143	5.3571	0.6224
518	2011	MIRE8	MIRE	0.4646	0.1755	22.3782	0.4122	0.2337	0.4399	0.6984	5.1905	0.5986
521	2011	MIRE9	MIRE	0.3644	0.1368	27.6890	0.4397	0.2261	0.4220	0.6667	5.0833	0.5833
522	2011	MIRE9	MIRE	0.5173	0.1671	20.1434	0.3796	0.2495	0.4771	0.7111	5.1765	0.5966
523	2011	MIRE10	MIRE	0.6175	0.2004	18.3178	0.3571	0.2832	0.5564	0.7193	5.6364	0.6623
524	2011	MIRE10	MIRE	0.4865	0.1765	18.0152	0.3628	0.3027	0.6022	0.6667	5.6471	0.6639
525	2011	MIRE11	MIRE	0.4060	0.1565	24.0693	0.4358	0.2102	0.3846	0.6667	5.2667	0.6095
530	2011	MIRE12	MIRE	0.5763	0.1870	24.6993	0.4417	0.2124	0.3898	0.6410	5.2143	0.6020
537	2011	MIRE15	MIRE	0.4798	0.1776	20.5165	0.3898	0.2398	0.4543	0.6500	5.3000	0.6143
551	2012	MIRE23	MIRE	0.5041	0.1838	22.9453	0.4214	0.2167	0.3999	0.6914	5.4828	0.6404
553	2012	MIRE24	MIRE	0.4835	0.1682	21.8421	0.4120	0.2461	0.4691	0.6923	5.6538	0.6648
554	2012	MIRE24	MIRE	0.3916	0.1486	21.7025	0.4123	0.2496	0.4773	0.6296	5.6667	0.6667
556	2012	MIRE25	MIRE	0.6603	0.2298	21.8150	0.4033	0.2235	0.4159	0.6984	5.6190	0.6599
559	2012	MIRE28	MIRE	0.3311	0.1278	23.5578	0.4292	0.2497	0.4774	0.6000	5.7500	0.6786
561	2012	MIRE29	MIRE	0.3650	0.1421	20.1335	0.3855	0.2490	0.4760	0.6282	5.5357	0.6480
564	2012	MIRE30	MIRE	0.5662	0.1891	21.8928	0.4066	0.2432	0.4622	0.6078	5.8947	0.6992
565	2012	MIRE31	MIRE	0.5292	0.1949	23.6550	0.4330	0.2076	0.3785	0.7424	5.3182	0.6169
566	2012	MIRE31	MIRE	0.5858	0.2093	21.9041	0.4093	0.2054	0.3732	0.6984	5.4762	0.6395
567	2012	MIRE32	MIRE	0.3077	0.1200	21.4165	0.4052	0.2585	0.4983	0.6349	5.5909	0.6558
568	2012	MIRE32	MIRE	0.4134	0.1548	20.6988	0.3953	0.2715	0.5289	0.6316	5.9000	0.7000
569	2012	MIRE33	MIRE	0.4671	0.1705	21.7353	0.4123	0.2547	0.4892	0.6471	5.5000	0.6429
571	2012	MIRE34	MIRE	0.5424	0.1810	23.7485	0.4228	0.2332	0.4388	0.6556	5.7419	0.6774
572	2012	MIRE34	MIRE	0.4676	0.1653	23.0316	0.4231	0.2152	0.3963	0.6842	5.8571	0.6939
573	2012	MIRE35	MIRE	0.4911	0.1832	23.6199	0.4316	0.2204	0.4085	0.7160	5.5185	0.6455
574	2012	MIRE35	MIRE	0.4361	0.1642	24.6218	0.4426	0.2265	0.4229	0.6875	5.4848	0.6407
575	2012	MIRE35	MIRE	0.4958	0.1819	23.5234	0.4284	0.2243	0.4178	0.6778	5.5161	0.6452
576	2012	MIRE36	MIRE	0.5548	0.2001	21.6751	0.4071	0.2039	0.3698	0.6667	5.4400	0.6343
578	2012	MIRE37	MIRE	0.4775	0.1760	24.5534	0.4452	0.2118	0.3883	0.6863	5.5000	0.6429
582	2012	MIRE39	MIRE	0.4234	0.1585	24.0564	0.4333	0.2232	0.4152	0.6162	5.6471	0.6639
584	2012	MIRE40	MIRE	0.5967	0.2110	22.8116	0.4217	0.2094	0.3826	0.7037	5.4815	0.6402
586	2012	MIRE41	MIRE	0.4543	0.1715	24.5568	0.4481	0.2025	0.3665	0.6508	5.3810	0.6259
590	2012	MIRE44	MIRE	0.4548	0.1663	24.4480	0.4418	0.2193	0.4060	0.6806	5.4800	0.6400
592	2012	MIRE45	MIRE	0.5754	0.2050	23.6560	0.4102	0.2351	0.4432	0.6522	5.2609	0.6087
601	2013	MIRE23	MIRE	0.5068	0.1854	22.8893	0.4223	0.2239	0.4168	0.6833	5.5500	0.6500
602	2013	MIRE23	MIRE	0.5046	0.1832	22.1856	0.4106	0.2377	0.4493	0.6795	5.4444	0.6349
606	2013	MIRE46	MIRE	0.5383	0.1942	25.4697	0.4328	0.2083	0.3800	0.7167	5.4000	0.6286
616	2013	MIRE7	MIRE	0.4250	0.1581	20.8216	0.3970	0.2749	0.5369	0.6173	5.5185	0.6455
617	2013	MIRE2	MIRE	0.4741	0.1726	24.6538	0.4360	0.2195	0.4063	0.7143	5.9286	0.7041
619	2013	MIRE2	MIRE	0.6075	0.2136	23.2110	0.4353	0.2329	0.4380	0.6667	5.5385	0.6484
621	2013	MIRE49	MIRE	0.5146	0.1842	22.8920	0.4183	0.2404	0.4556	0.7222	5.5000	0.6429
627	2013	MIRE51	MIRE	0.4764	0.1680	19.1839	0.3569	0.2871	0.5655	0.7436	5.6000	0.6571
628	2013	MIRE51	MIRE	0.4240	0.1568	20.9710	0.3977	0.2856	0.5621	0.6140	5.6667	0.6667
639	2013	MIRE52	MIRE	0.5670	0.1985	24.1096	0.4198	0.2146	0.3949	0.6667	5.6190	0.6599
641	2013	MIRE53	MIRE	0.6252	0.2198	22.9913	0.4220	0.2238	0.4166	0.6500	5.9500	0.7071
647	2013	MIRE39	MIRE	0.6186	0.2157	23.2833	0.4312	0.1897	0.3364	0.6344	5.7419	0.6774
650	2013	MIRE44	MIRE	0.4503	0.1641	23.2933	0.4258	0.2309	0.4333	0.6533	5.5385	0.6484
652	2013	MIRE44	MIRE	0.5100	0.1846	25.1746	0.4293	0.2407	0.4563	0.7222	5.4211	0.6316
654	2013	MIRE42	MIRE	0.4158	0.1594	23.6531	0.4294	0.2215	0.4111	0.6491	5.4211	0.6316
655	2013	MIRE41	MIRE	0.5362	0.1967	21.1947	0.4008	0.2335	0.4394	0.6863	5.4118	0.6303
657	2013	MIRE54	MIRE	0.4677	0.1756	23.4830	0.4228	0.2406	0.4562	0.6806	5.6667	0.6667
659	2013	MIRE25	MIRE	0.7987	0.2582	22.1861	0.4083	0.2180	0.4028	0.6667	5.8824	0.6975
660	2013	MIRE28	MIRE	0.3531	0.1355	22.6519	0.4244	0.2457	0.4680	0.6322	5.6897	0.6700
661	2013	MIRE28	MIRE	0.3973	0.1500	22.3622	0.4144	0.2421	0.4595	0.6263	5.5758	0.6537
662	2013	MIRE31	MIRE	0.5109	0.1874	24.1901	0.4386	0.2204	0.4085	0.6782	5.4138	0.6305
663	2013	MIRE31	MIRE	0.5840	0.2095	24.1313	0.4352	0.2179	0.4028	0.6782	5.5172	0.6453
664	2013	MIRE31	MIRE	0.5357	0.1959	21.0948	0.4021	0.2324	0.4369	0.7037	5.4444	0.6349
672	2014	MIRE56	MIRE	0.4199	0.1611	22.5364	0.4197	0.2354	0.4440	0.6818	5.1818	0.5974
BB_02a	2002	BB	BB_REWET	0.9490	0.2861	33.3995	0.4770	0.2726	0.5314	0.8333	5.7500	0.6786
BB_02b	2002	BB	BB_REWET	0.6438	0.1930	43.1997	0.5653	0.2195	0.4063	0.6667	5.0000	0.5714
BB_02c	2002	BB	BB_REWET	0.8035	0.2467	34.7065	0.5011	0.2454	0.4673	0.7778	5.0000	0.5714
BB_02d	2002	BB	BB_REWET	0.5678	0.1786	43.6275	0.5717	0.2132	0.3916	0.6667	5.0000	0.5714
BB_02e	2002	BB	BB_REWET	0.8583	0.2574	38.8496	0.5176	0.2396	0.4538	0.7778	4.6667	0.5238
BB_02f	2002	BB	BB_REWET	0.5500	0.1539	52.0996	0.6356	0.1847	0.3244	0.5556	5.3333	0.6190
BB_02g	2002	BB	BB_REWET	0.5500	0.1539	52.0996	0.6356	0.1847	0.3244	0.5556	5.3333	0.6190
BB_96a	1996	BB	BB_BEFORE	0.6068	0.2166	22.1436	0.4283	0.2157	0.3974	0.7857	5.6429	0.6633
BB_96b	1996	BB	BB_BEFORE	0.7252	0.2449	23.5591	0.4289	0.2183	0.4036	0.8095	5.5000	0.6429
BB_96c	1996	BB	BB_BEFORE	0.7999	0.2625	22.7806	0.4429	0.2271	0.4243	0.8519	5.6667	0.6667
BB_96d	1996	BB	BB_BEFORE	0.6210	0.2212	22.1498	0.4292	0.2171	0.4008	0.5741	5.6667	0.6667
BB_96e	1996	BB	BB_BEFORE	0.7423	0.2540	21.6895	0.4258	0.2182	0.4034	0.6000	5.8500	0.6929
BB_96f	1996	BB	BB_BEFORE	0.6049	0.2071	21.4055	0.4036	0.2202	0.4081	0.7576	6.0000	0.7143
BB_96g	1996	BB	BB_BEFORE	0.5864	0.2104	22.5717	0.4338	0.2114	0.3874	0.7778	5.4667	0.6381
CAL_14_115	2014	CAL	CAL_TSR	0.5442	0.1852	38.6182	0.4547	0.2181	0.4032	0.7500	5.4444	0.6349
CAL_14_119	2014	CAL	CAL_TSR	0.6141	0.2162	18.7720	0.3651	0.2682	0.5210	0.7273	5.5455	0.6494

CAL_14_123	2014	CAL	CAL_TSR	0.6008	0.2115	21.2139	0.3967	0.2475	0.4722	0.7500	5.6667	0.6667
CAL_14_127	2014	CAL	CAL_TSR	0.8098	0.2676	15.7067	0.3199	0.3085	0.6159	0.8000	5.6000	0.6571
CAL_14_148	2014	CAL	CAL_TSR	0.7672	0.2467	21.1190	0.3887	0.2433	0.4624	0.6667	5.8235	0.6891
CAL_14_152	2014	CAL	CAL_TSR	0.5332	0.1951	21.7544	0.4073	0.2527	0.4846	0.6667	5.7368	0.6767
CAL_14_156	2014	CAL	CAL_TSR	0.5810	0.2071	20.4242	0.3879	0.2417	0.4588	0.6481	5.6111	0.6587
CAL_14_160	2014	CAL	CAL_TSR	0.6232	0.2169	19.3090	0.3651	0.2500	0.4783	0.5714	6.0000	0.7143
CAL_14_177	2014	CAL	CAL_TSR	0.7155	0.2453	22.2122	0.4052	0.2193	0.4060	0.6333	5.9000	0.7000
CAL_14_181	2014	CAL	CAL_TSR	0.5784	0.2106	25.8884	0.4593	0.2278	0.4259	0.6222	6.0000	0.7143
CAL_14_185	2014	CAL	CAL_TSR	0.5538	0.2026	22.5545	0.4137	0.2302	0.4315	0.5926	5.7778	0.6825
CAL_14_189	2014	CAL	CAL_TSR	0.5322	0.1911	25.1187	0.4270	0.2343	0.4413	0.6333	5.6500	0.6643
CAL_14_19	2014	CAL	CAL_TSR	0.6791	0.2351	19.9223	0.3730	0.2441	0.4644	0.6061	5.8182	0.6883
CAL_14_193	2014	CAL	CAL_TSR	0.5350	0.1839	42.3331	0.5094	0.1917	0.3410	0.6250	5.4444	0.6349
CAL_14_197	2014	CAL	CAL_TSR	0.6606	0.2268	20.5422	0.3867	0.2597	0.5010	0.7917	5.6250	0.6607
CAL_14_201	2014	CAL	CAL_TSR	0.6350	0.2216	23.6370	0.4275	0.2390	0.4524	0.6667	5.8182	0.6883
CAL_14_205	2014	CAL	CAL_TSR	0.7296	0.2457	19.1507	0.3611	0.2557	0.4917	0.7083	6.1250	0.7321
CAL_14_228	2014	CAL	CAL_TSR	0.5516	0.2016	25.6672	0.4570	0.2256	0.4207	0.6000	6.0000	0.7143
CAL_14_23	2014	CAL	CAL_TSR	0.5390	0.1965	23.1423	0.4197	0.2206	0.4091	0.6042	5.7500	0.6786
CAL_14_232	2014	CAL	CAL_TSR	0.6660	0.2276	20.1347	0.3663	0.2249	0.4192	0.7436	5.7692	0.6813
CAL_14_236	2014	CAL	CAL_TSR	0.6762	0.2355	24.0411	0.4315	0.2198	0.4072	0.6923	5.9231	0.7033
CAL_14_240	2014	CAL	CAL_TSR	0.6760	0.2329	22.9116	0.4147	0.2383	0.4506	0.6667	6.0000	0.7143
CAL_14_257	2014	CAL	CAL_TSR	0.5903	0.2107	25.7232	0.4551	0.2130	0.3912	0.5867	5.9200	0.7029
CAL_14_261	2014	CAL	CAL_TSR	0.5855	0.2068	24.5456	0.4366	0.2201	0.4078	0.6032	5.8571	0.6939
CAL_14_265	2014	CAL	CAL_TSR	0.5442	0.1962	26.5947	0.4651	0.2104	0.3851	0.6667	5.6316	0.6617
CAL_14_269	2014	CAL	CAL_TSR	0.5651	0.2012	23.0307	0.4218	0.2127	0.3904	0.6786	5.7143	0.6735
CAL_14_27	2014	CAL	CAL_TSR	0.5897	0.2159	18.5917	0.3578	0.2550	0.4900	0.5897	5.6923	0.6703
CAL_14_292	2014	CAL	CAL_TSR	0.5991	0.2123	24.6759	0.4387	0.2113	0.3872	0.6275	5.7647	0.6807
CAL_14_296	2014	CAL	CAL_TSR	0.5502	0.1973	23.3043	0.4262	0.2261	0.4219	0.6825	5.6667	0.6667
CAL_14_300	2014	CAL	CAL_TSR	0.6295	0.2242	22.4988	0.4155	0.2297	0.4304	0.6667	5.8182	0.6883
CAL_14_304	2014	CAL	CAL_TSR	0.5088	0.1899	25.2609	0.4582	0.2321	0.4362	0.6389	5.5833	0.6548
CAL_14_31	2014	CAL	CAL_TSR	0.5514	0.1989	22.2244	0.4101	0.2460	0.4688	0.6377	5.6957	0.6708
CAL_14_339	2014	CAL	CAL_TSR	0.6795	0.2352	18.1614	0.3497	0.2714	0.5286	0.6410	5.3846	0.6264
CAL_14_343	2014	CAL	CAL_TSR	0.6799	0.2366	20.0482	0.3923	0.2352	0.4433	0.6296	5.7778	0.6825
CAL_14_347	2014	CAL	CAL_TSR	0.5972	0.2081	30.9995	0.4376	0.2284	0.4274	0.6471	5.6667	0.6667
CAL_14_351	2014	CAL	CAL_TSR	0.5831	0.2036	30.4117	0.4248	0.2436	0.4631	0.6875	5.2941	0.6134
CAL_14_370	2014	CAL	CAL_TSR	0.6723	0.2336	20.8156	0.3834	0.2339	0.4403	0.5667	5.8000	0.6857
CAL_14_374	2014	CAL	CAL_TSR	0.5349	0.1986	20.1968	0.3869	0.2394	0.4533	0.6333	5.3000	0.6143
CAL_14_378	2014	CAL	CAL_TSR	0.5124	0.1912	18.6440	0.3544	0.2551	0.4901	0.6364	5.7273	0.6753
CAL_14_382	2014	CAL	CAL_TSR	0.5579	0.2028	20.1152	0.3861	0.2443	0.4647	0.7381	5.4286	0.6327
CAL_14_403	2014	CAL	CAL_TSR	0.5101	0.1870	21.8036	0.4111	0.2359	0.4451	0.6863	5.5882	0.6555
CAL_14_407	2014	CAL	CAL_TSR	0.4090	0.1589	24.7091	0.4584	0.2235	0.4158	0.6333	5.3500	0.6214
CAL_14_411	2014	CAL	CAL_TSR	0.4872	0.1788	20.8450	0.4017	0.2502	0.4786	0.7083	5.5000	0.6429
CAL_14_415	2014	CAL	CAL_TSR	0.5238	0.1916	22.7751	0.4239	0.2393	0.4530	0.6667	5.4348	0.6335
CAL_14_434	2014	CAL	CAL_TSR	0.5307	0.1926	22.2018	0.4124	0.2365	0.4465	0.6667	5.6923	0.6703
CAL_14_438	2014	CAL	CAL_TSR	0.4488	0.1703	23.3729	0.4282	0.2218	0.4118	0.6250	5.5000	0.6429
CAL_14_442	2014	CAL	CAL_TSR	0.5058	0.1831	22.0016	0.4071	0.2402	0.4551	0.7167	5.5500	0.6500
CAL_14_446	2014	CAL	CAL_TSR	0.5466	0.1967	21.6405	0.4088	0.2505	0.4794	0.6875	5.4375	0.6339
CAL_14_449	2014	CAL	CAL_TSR	0.6264	0.2188	24.0441	0.4051	0.2408	0.4565	0.6250	5.6875	0.6696
CAL_14_453	2014	CAL	CAL_TSR	0.4912	0.1833	22.8248	0.4269	0.2422	0.4598	0.7381	5.3571	0.6224
CAL_14_457	2014	CAL	CAL_TSR	0.5022	0.1854	23.7212	0.4379	0.2542	0.4882	0.6061	5.6364	0.6623
CAL_14_461	2014	CAL	CAL_TSR	0.5836	0.2119	21.1224	0.3917	0.2579	0.4967	0.5556	5.7500	0.6786
CAL_14_484	2014	CAL	CAL_TSR	0.5152	0.1918	17.5684	0.3468	0.2711	0.5279	0.6667	5.2000	0.6000
CAL_14_488	2014	CAL	CAL_TSR	0.7232	0.2269	21.7965	0.4180	0.2533	0.4861	0.6296	5.3000	0.6143
CAL_14_492	2014	CAL	CAL_TSR	0.5872	0.2106	19.9804	0.3743	0.2492	0.4764	0.6875	5.5625	0.6518
CAL_14_496	2014	CAL	CAL_TSR	0.5598	0.2043	20.4251	0.3927	0.2692	0.5234	0.6000	5.4000	0.6286
CAL_14_50	2014	CAL	CAL_TSR	0.4683	0.1695	27.2717	0.4468	0.2280	0.4265	0.6429	5.4286	0.6327
CAL_14_54	2014	CAL	CAL_TSR	0.5297	0.1917	24.2356	0.4329	0.2157	0.3976	0.6500	5.6500	0.6643
CAL_14_58	2014	CAL	CAL_TSR	0.5868	0.2085	21.5755	0.3976	0.2461	0.4691	0.7059	5.5294	0.6471
CAL_14_62	2014	CAL	CAL_TSR	0.6374	0.2248	21.8574	0.3977	0.2354	0.4439	0.5686	5.9412	0.7059
CAL_14_82	2014	CAL	CAL_TSR	0.5947	0.1991	36.7630	0.4674	0.1967	0.3527	0.6111	5.4615	0.6374
CAL_14_86	2014	CAL	CAL_TSR	0.5322	0.1874	35.9983	0.4823	0.2314	0.4345	0.6944	5.4615	0.6374
CAL_14_90	2014	CAL	CAL_TSR	0.6509	0.2276	20.6488	0.3933	0.2511	0.4808	0.6970	5.6364	0.6623
CAL_14_94	2014	CAL	CAL_TSR	0.7628	0.2594	20.7850	0.3759	0.2525	0.4841	0.7037	6.0000	0.7143
CAL_D04_1	2004	CAL	CAL_BEFORI	0.4381	0.1636	26.1816	0.4723	0.1908	0.3389	0.5313	5.2500	0.6071
CAL_D04_10	2004	CAL	CAL_BEFORI	0.3792	0.1488	25.5609	0.4689	0.2142	0.3940	0.6250	5.2500	0.6071
CAL_D04_11	2004	CAL	CAL_BEFORI	0.4216	0.1597	21.8495	0.4099	0.2422	0.4599	0.7179	5.3846	0.6264
CAL_D04_12	2004	CAL	CAL_BEFORI	0.4186	0.1608	25.2716	0.4619	0.2039	0.3698	0.6852	5.2778	0.6111
CAL_D04_13	2004	CAL	CAL_BEFORI	0.3272	0.1311	24.5148	0.4570	0.2032	0.3681	0.5741	5.2778	0.6111
CAL_D04_2	2004	CAL	CAL_BEFORI	0.4889	0.1811	24.6924	0.4542	0.2076	0.3784	0.5802	5.5556	0.6508
CAL_D04_3	2004	CAL	CAL_BEFORI	0.4009	0.1541	25.2177	0.4640	0.1939	0.3462	0.5972	5.4583	0.6369
CAL_D04_4	2004	CAL	CAL_BEFORI	0.3542	0.1397	25.5383	0.4694	0.1941	0.3468	0.6140	5.4737	0.6391
CAL_D04_5	2004	CAL	CAL_BEFORI	0.4289	0.1608	26.4812	0.4761	0.1919	0.3415	0.5294	5.2353	0.6050
CAL_D04_6	2004	CAL	CAL_BEFORI	0.4014	0.1521	26.2565	0.4767	0.1946	0.3478	0.5694	5.4583	0.6369
CAL_D04_7	2004	CAL	CAL_BEFORI	0.2962	0.1207	24.7937	0.4610	0.2069	0.3767	0.6296	5.1111	0.5873
CAL_D04_8	2004	CAL	CAL_BEFORI	0.3504	0.1392	23.8360	0.4470	0.2239	0.4169	0.5714	5.5000	0.6429
CAL_D04_9	2004	CAL	CAL_BEFORI	0.3607	0.1418	26.0269	0.4762	0.1925	0.3428	0.6471	5.2941	0.6134
CAL_FM04_1	2004	CAL	CAL_REF	0.5984	0.2119	22.3654	0.4094	0.2273	0.4247	0.6667	5.7647	0.6807
CAL_FM04_10	2004	CAL	CAL_REF	0.5626	0.2043	22.5975	0.4215	0.2262	0.4222	0.6842	5.7000	0.6714

CAL_FM04_11	2004	CAL	CAL_REF	0.5649	0.2052	22.3270	0.4150	0.2307	0.4327	0.6667	5.7273	0.6753
CAL_FM04_2	2004	CAL	CAL_REF	0.5462	0.1999	22.4053	0.4160	0.2253	0.4200	0.7059	5.7059	0.6723
CAL_FM04_3	2004	CAL	CAL_REF	0.5393	0.1927	20.7669	0.3909	0.2413	0.4576	0.6410	5.4615	0.6374
CAL_FM04_4	2004	CAL	CAL_REF	0.5580	0.2029	22.2932	0.4103	0.2425	0.4605	0.7083	5.6875	0.6696
CAL_FM04_5	2004	CAL	CAL_REF	0.4557	0.1724	22.8371	0.4235	0.2289	0.4286	0.6508	5.4762	0.6395
CAL_FM04_6	2004	CAL	CAL_REF	0.4686	0.1751	24.1419	0.4478	0.2164	0.3991	0.6533	5.5200	0.6457
CAL_FM04_7	2004	CAL	CAL_REF	0.5644	0.2037	22.9443	0.4270	0.2116	0.3879	0.6167	5.5500	0.6500
CAL_FM04_8	2004	CAL	CAL_REF	0.6374	0.2243	19.7683	0.3781	0.2414	0.4581	0.6667	5.7222	0.6746
CAL_FM04_9	2004	CAL	CAL_REF	0.4896	0.1841	23.2067	0.4265	0.2225	0.4135	0.6275	5.6471	0.6639
CAL_FM14_1	2014	CAL	CAL_REF	0.5805	0.2103	22.6628	0.4170	0.2338	0.4400	0.5833	5.7500	0.6786
CAL_FM14_2	2014	CAL	CAL_REF	0.5063	0.1816	29.9906	0.4533	0.2013	0.3636	0.6667	5.0000	0.5714
CAL_FM14_3	2014	CAL	CAL_REF	0.4791	0.1767	25.5477	0.4353	0.2373	0.4484	0.6667	5.3889	0.6270
CAL_FM14_4	2014	CAL	CAL_REF	0.5364	0.1975	23.1866	0.4224	0.2398	0.4541	0.6250	5.6250	0.6607
CAL_FM14_5	2014	CAL	CAL_REF	0.4762	0.1739	26.1295	0.4338	0.2144	0.3945	0.7381	5.2143	0.6020
CAL_FM14_6	2014	CAL	CAL_REF	0.4384	0.1668	22.4517	0.4172	0.2408	0.4566	0.6500	5.3000	0.6143
CAL_FM14_7	2014	CAL	CAL_REF	0.5477	0.2020	24.3645	0.4458	0.2250	0.4194	0.6522	5.4783	0.6398
CAL_FM14_8	2014	CAL	CAL_REF	0.5187	0.1891	28.0884	0.4354	0.2227	0.4140	0.6000	5.4000	0.6286
CIE1	1998	CIE	CIE_BEFORE	0.4419	0.1669	24.8192	0.4567	0.2240	0.4169	0.7143	5.5429	0.6490
CIE2	1998	CIE	CIE_BEFORE	0.4408	0.1687	24.1226	0.4454	0.2319	0.4356	0.6667	5.3810	0.6259
CIE3	2013	CIE	CIE_REWET	0.4267	0.1606	23.9003	0.4474	0.2137	0.3927	0.6282	5.6296	0.6614
CIE4	2013	CIE	CIE_REWET	0.3481	0.1401	23.7386	0.4367	0.2150	0.3958	0.6667	5.0000	0.5714
CIE5	2013	CIE	CIE_REWET	0.3582	0.1434	25.9449	0.4545	0.1995	0.3593	0.7083	4.8750	0.5536
DE_D3_E_85	1985	DE	DE_BEFORE	0.5494	0.1963	25.5215	0.4721	0.2050	0.3724	0.6410	5.4074	0.6296
DE_D3_E_95	1995	DE	DE_TSR	0.4073	0.1567	22.5126	0.4308	0.2334	0.4393	0.6219	5.4815	0.6402
DE_D3_W_85	1985	DE	DE_BEFORE	0.5531	0.1980	25.7183	0.4731	0.2056	0.3737	0.6207	5.4333	0.6333
DE_D3_W_95	1995	DE	DE_TSR	0.4171	0.1596	22.6694	0.4324	0.2413	0.4577	0.6149	5.5510	0.6501
DO_D10_03	2003	DO	DO_TSR	0.4315	0.1657	23.6794	0.4351	0.2443	0.4649	0.6812	5.8636	0.6948
DO_D11_03	2003	DO	DO_TSR	0.5190	0.1935	24.0876	0.4445	0.2429	0.4615	0.7101	5.6957	0.6708
DO_D12_03	2003	DO	DO_TSR	0.5020	0.1862	24.8342	0.4606	0.2142	0.3940	0.7059	5.6250	0.6607
DO_D25_03	2003	DO	DO_TSR	0.5460	0.1944	22.7362	0.4298	0.2320	0.4359	0.7333	5.9500	0.7071
DO_D26_03	2003	DO	DO_TSR	0.6854	0.2148	19.7952	0.3843	0.2600	0.5018	0.7719	5.5500	0.6500
DO_D29_03	2003	DO	DO_TSR	0.7910	0.2479	17.4320	0.3421	0.2511	0.4808	0.6944	5.5385	0.6484
DO_D30_03	2003	DO	DO_TSR	0.5853	0.2083	22.0922	0.4089	0.2660	0.5159	0.7222	5.8333	0.6905
DO_D8_91	1991	DO	DO_BEFORE	0.5635	0.2052	27.9823	0.4900	0.1608	0.2684	0.2029	5.5714	0.6531
DO_D8_92	1992	DO	DO_BEFORE	0.5947	0.2129	29.1153	0.5040	0.1619	0.2709	0.3939	5.5263	0.6466
DO_donorHH_1	1993	DO	DO_REF	0.5232	0.1837	20.9989	0.4141	0.2543	0.4884	0.6429	5.6154	0.6593
DO_donorHH_2	1993	DO	DO_REF	0.7922	0.2429	21.2240	0.4142	0.2490	0.4759	0.7284	5.6538	0.6648
DO_donorLI_1	1993	DO	DO_REF	0.5140	0.1881	24.0386	0.4507	0.2331	0.4384	0.7200	5.6190	0.6599
DO_donorLI_2	1993	DO	DO_REF	0.4941	0.1831	21.7989	0.4274	0.2378	0.4495	0.6667	5.7692	0.6813
DO_donorZE_1	1993	DO	DO_REF	0.6728	0.2369	20.2812	0.4009	0.2577	0.4962	0.7500	5.7619	0.6803
DO_donorZE_2	1993	DO	DO_REF	0.6748	0.2396	21.2347	0.4121	0.2531	0.4855	0.7101	5.8261	0.6894
DO_donorZE_3	1993	DO	DO_REF	0.6933	0.2412	21.0195	0.3992	0.2337	0.4399	0.7917	5.7826	0.6832
DO_donorZS_1	1993	DO	DO_REF	0.5503	0.1899	19.2524	0.3916	0.2583	0.4977	0.6032	5.6111	0.6587
DO_donorZS_2	1993	DO	DO_REF	0.4418	0.1673	20.2383	0.4048	0.2523	0.4835	0.6032	5.8824	0.6975
DO_donorZS_3	1993	DO	DO_REF	0.5647	0.1956	20.3267	0.4057	0.2536	0.4868	0.5641	5.8400	0.6914
DO_donorZS_4	1993	DO	DO_REF	0.6728	0.2114	17.3097	0.3490	0.2663	0.5166	0.6296	5.5294	0.6471
DO_donorZS_5	1993	DO	DO_REF	0.7269	0.2410	19.4876	0.3834	0.2892	0.5704	0.6667	5.6667	0.6667
DO_donorZS_6	1993	DO	DO_REF	0.7317	0.2442	21.2659	0.3989	0.2357	0.4446	0.7037	5.1250	0.5893
DO_M1_03	2003	DO	DO_TSR	0.5200	0.1925	21.4420	0.4123	0.2622	0.5070	0.6167	5.5263	0.6466
DO_M10_03	2003	DO	DO_TSR	0.7184	0.2318	19.1596	0.3798	0.2946	0.5833	0.6842	5.5500	0.6500
DO_M11_03	2003	DO	DO_TSR	0.8755	0.2687	19.5403	0.3703	0.2453	0.4670	0.7500	5.5909	0.6558
DO_M12_03	2003	DO	DO_TSR	0.9131	0.2834	18.1757	0.3455	0.2744	0.5358	0.8182	5.4167	0.6310
DO_M13_03	2003	DO	DO_TSR	0.9882	0.2944	16.6596	0.3256	0.2798	0.5484	0.7083	5.3889	0.6270
DO_M14_03	2003	DO	DO_TSR	0.8723	0.2699	16.8930	0.3375	0.2997	0.5951	0.6923	5.4286	0.6327
DO_M2_03	2003	DO	DO_TSR	0.5681	0.2083	21.3583	0.4109	0.2518	0.4825	0.6078	5.5000	0.6429
DO_M3_03	2003	DO	DO_TSR	0.4982	0.1864	22.5078	0.4158	0.2530	0.4853	0.7051	5.6667	0.6667
DO_M4_03	2003	DO	DO_TSR	0.6150	0.2085	19.4454	0.3782	0.2673	0.5189	0.6970	5.6667	0.6667
DO_M5_03	2003	DO	DO_TSR	0.6666	0.2165	20.5117	0.3875	0.2666	0.5172	0.6933	5.5200	0.6457
DO_M6_03	2003	DO	DO_TSR	0.6925	0.2226	20.4943	0.3882	0.2645	0.5122	0.6232	5.5417	0.6488
DO_M8_03	2003	DO	DO_TSR	0.6638	0.2230	20.9698	0.4002	0.2446	0.4655	0.6842	5.7500	0.6786
DO_M9_03	2003	DO	DO_TSR	0.6437	0.2166	17.9847	0.3529	0.3000	0.5958	0.6667	5.6842	0.6692
DU_1	1995	DU	DU_BEFORE	0.4186	0.1619	26.4608	0.4780	0.2351	0.4432	0.6222	4.8000	0.5429
DU_132	2010	DU	DU_REWET	0.5579	0.1997	21.7228	0.4166	0.2666	0.5173	0.7500	5.2917	0.6131
DU_138	2010	DU	DU_REWET	0.4971	0.1841	23.2449	0.4380	0.2458	0.4684	0.6989	5.3226	0.6175
DU_167	2010	DU	DU_REWET	0.5307	0.1939	23.0656	0.4369	0.2658	0.5155	0.7600	5.2000	0.6000
DU_172	2010	DU	DU_REWET	0.5204	0.1901	23.7953	0.4439	0.2572	0.4951	0.7619	5.0952	0.5850
DU_173	1987	DU	DU_BEFORE	0.4752	0.1768	27.0417	0.4752	0.2112	0.3868	0.6111	5.5000	0.6429
DU_174	1987	DU	DU_BEFORE	0.5082	0.1840	21.8830	0.4196	0.2400	0.4547	0.7407	5.1667	0.5952
DU_175	1987	DU	DU_BEFORE	0.4707	0.1736	23.4618	0.4385	0.2367	0.4469	0.6897	5.2414	0.6059
DU_2	1997	DU	DU_BEFORE	0.4582	0.1716	25.3262	0.4627	0.2163	0.3989	0.6316	5.0526	0.5789
DU_72	2010	DU	DU_REWET	0.5504	0.1999	24.2519	0.4522	0.2468	0.4707	0.7564	5.1538	0.5934
DU_78	2010	DU	DU_REWET	0.4432	0.1686	23.6280	0.4403	0.2450	0.4664	0.6566	5.4242	0.6320
DU_83	2010	DU	DU_REWET	0.5282	0.1933	23.9236	0.4399	0.2369	0.4473	0.6933	5.4000	0.6286
DU_84	2010	DU	DU_REWET	0.5118	0.1885	25.2798	0.4644	0.2425	0.4605	0.7333	5.2400	0.6057
HAV_1_2002	2002	HAV	HAV_BEFOR	0.5353	0.2002	24.3269	0.4416	0.2334	0.4392	0.6275	5.5294	0.6471
HAV_10_2002	2002	HAV	HAV_BEFOR	0.5330	0.1851	19.0066	0.3800	0.2934	0.5803	0.6222	5.8000	0.6857
HAV_100_2010	2010	HAV	HAV_REWE	0.6043	0.2112	27.2183	0.4692	0.2349	0.4427	0.6528	5.9130	0.7019

HAV_101_2010	2010	HAV	HAV_REWE	0.9072	0.2843	31.7724	0.4141	0.2431	0.4620	0.7917	6.4444	0.7778
HAV_102_2010	2010	HAV	HAV_REWE	0.7147	0.2348	24.2628	0.3845	0.2560	0.4923	0.7121	5.7917	0.6845
HAV_103_2010	2010	HAV	HAV_REWE	0.4077	0.1568	21.9563	0.4226	0.2499	0.4780	0.7101	5.4348	0.6335
HAV_104_2010	2010	HAV	HAV_REWE	0.3402	0.1363	23.0825	0.4417	0.2322	0.4364	0.7059	5.1176	0.5882
HAV_105_2010	2010	HAV	HAV_REWE	0.3412	0.1354	24.0322	0.4541	0.2199	0.4074	0.6515	5.2381	0.6054
HAV_106_2010	2010	HAV	HAV_REWE	0.3417	0.1366	23.8783	0.4471	0.2222	0.4129	0.7167	5.4000	0.6286
HAV_107_2010	2010	HAV	HAV_REWE	0.5870	0.2083	22.4190	0.4243	0.3018	0.6002	0.6667	5.8333	0.6905
HAV_108_2010	2010	HAV	HAV_REWE	0.5754	0.2015	40.9149	0.4991	0.2364	0.4461	0.7917	5.4444	0.6349
HAV_11_2002	2002	HAV	HAV_BEFOR	0.3933	0.1542	22.9275	0.4357	0.2501	0.4785	0.6806	5.5000	0.6429
HAV_12_2002	2002	HAV	HAV_BEFOR	0.3702	0.1456	22.9524	0.4390	0.2400	0.4546	0.6914	5.4444	0.6349
HAV_13_2002	2002	HAV	HAV_BEFOR	0.4006	0.1539	23.6564	0.4501	0.2306	0.4325	0.6825	5.4500	0.6357
HAV_14_2002	2002	HAV	HAV_BEFOR	0.3749	0.1467	23.8780	0.4500	0.2287	0.4282	0.6667	5.3462	0.6209
HAV_15_2002	2002	HAV	HAV_BEFOR	0.4485	0.1699	22.3090	0.4262	0.2567	0.4940	0.6833	5.2500	0.6071
HAV_16_2002	2002	HAV	HAV_BEFOR	0.6610	0.2305	20.6189	0.3912	0.2526	0.4843	0.7407	5.6667	0.6667
HAV_17_2002	2002	HAV	HAV_BEFOR	0.4096	0.1556	25.0930	0.4665	0.2147	0.3952	0.5606	5.4286	0.6327
HAV_18_2002	2002	HAV	HAV_BEFOR	0.5879	0.2118	20.2537	0.3835	0.2228	0.4143	0.7719	5.7368	0.6767
HAV_19_2002	2002	HAV	HAV_BEFOR	0.5677	0.2071	21.5126	0.4107	0.2287	0.4280	0.6933	5.8000	0.6857
HAV_2_2002	2002	HAV	HAV_BEFOR	0.5873	0.2156	25.4346	0.4687	0.2754	0.5379	0.7778	5.6667	0.6667
HAV_20_2002	2002	HAV	HAV_BEFOR	0.6905	0.2424	21.2918	0.3926	0.2246	0.4185	0.7778	5.7500	0.6786
HAV_21_2002	2002	HAV	HAV_BEFOR	0.4433	0.1697	24.8604	0.4616	0.2094	0.3828	0.6032	5.2857	0.6122
HAV_22_2002	2002	HAV	HAV_BEFOR	0.4825	0.1784	24.0682	0.4518	0.2123	0.3896	0.6190	5.3704	0.6243
HAV_23_2002	2002	HAV	HAV_BEFOR	0.8308	0.2554	29.2242	0.4972	0.2228	0.4141	0.6061	6.0435	0.7205
HAV_24_2002	2002	HAV	HAV_BEFOR	0.7084	0.2392	21.6183	0.4030	0.2659	0.5157	0.6410	6.3077	0.7582
HAV_25_2002	2002	HAV	HAV_BEFOR	0.8410	0.2628	20.4615	0.3793	0.2118	0.3882	0.7500	6.6000	0.8000
HAV_26_2002	2002	HAV	HAV_BEFOR	0.3717	0.1477	30.4188	0.5252	0.2065	0.3758	0.6389	4.6667	0.5238
HAV_27_2002	2002	HAV	HAV_BEFOR	0.4043	0.1567	26.3642	0.4790	0.2223	0.4130	0.5098	5.1875	0.5982
HAV_28_2002	2002	HAV	HAV_BEFOR	0.3918	0.1525	26.5290	0.4808	0.2255	0.4204	0.5556	5.1176	0.5882
HAV_29_2002	2002	HAV	HAV_BEFOR	0.5149	0.1849	28.1263	0.5007	0.2055	0.3736	0.6429	5.1538	0.5934
HAV_3_2002	2002	HAV	HAV_BEFOR	0.5428	0.2019	22.4887	0.4119	0.2705	0.5264	0.6471	5.4118	0.6303
HAV_30_2002	2002	HAV	HAV_BEFOR	0.4615	0.1727	25.5914	0.4699	0.2299	0.4309	0.5439	5.1579	0.5940
HAV_31_2002	2002	HAV	HAV_BEFOR	0.4084	0.1592	25.7053	0.4704	0.2218	0.4118	0.5614	5.2632	0.6090
HAV_32_2002	2002	HAV	HAV_BEFOR	0.7415	0.2352	32.9722	0.5319	0.2003	0.3613	0.5263	5.9375	0.7054
HAV_4_2002	2002	HAV	HAV_BEFOR	0.5271	0.1955	23.4868	0.4315	0.2276	0.4256	0.7179	5.7692	0.6813
HAV_5_2002	2002	HAV	HAV_BEFOR	0.4230	0.1614	23.3115	0.4386	0.2404	0.4556	0.7143	5.2273	0.6039
HAV_6_2002	2002	HAV	HAV_BEFOR	0.6923	0.2439	24.9829	0.4579	0.2182	0.4033	0.6410	6.0000	0.7143
HAV_7_2002	2002	HAV	HAV_BEFOR	0.6358	0.2218	23.9106	0.4231	0.2309	0.4333	0.6818	5.5000	0.6429
HAV_73_2010	2010	HAV	HAV_REWE	0.6829	0.2300	25.0058	0.3879	0.2513	0.4812	0.7333	5.5000	0.6429
HAV_74_2010	2010	HAV	HAV_REWE	0.5904	0.2150	29.6808	0.5109	0.2214	0.4109	0.4000	6.0000	0.7143
HAV_75_2010	2010	HAV	HAV_REWE	0.6106	0.2017	33.2450	0.4927	0.2106	0.3855	0.7143	5.2857	0.6122
HAV_76_2010	2010	HAV	HAV_REWE	0.5681	0.2017	27.4085	0.4609	0.2039	0.3698	0.6842	5.6842	0.6692
HAV_77_2010	2010	HAV	HAV_REWE	0.6123	0.1873	41.0638	0.5208	0.2020	0.3653	0.6667	5.0000	0.5714
HAV_78_2010	2010	HAV	HAV_REWE	0.3506	0.1382	22.8840	0.4361	0.2179	0.4027	0.7037	5.3889	0.6270
HAV_79_2010	2010	HAV	HAV_REWE	0.6439	0.2237	24.3196	0.4079	0.2159	0.3980	0.7368	5.6842	0.6692
HAV_8_2002	2002	HAV	HAV_BEFOR	0.6081	0.2070	20.0256	0.3946	0.2720	0.5300	0.5952	5.9286	0.7041
HAV_80_2010	2010	HAV	HAV_REWE	0.5587	0.1852	48.9240	0.6349	0.1489	0.2402	0.5000	5.4000	0.6286
HAV_81_2010	2010	HAV	HAV_REWE	0.5063	0.1657	65.2200	0.7473	0.1372	0.2128	0.3333	5.3333	0.6190
HAV_82_2010	2010	HAV	HAV_REWE	0.4251	0.1635	23.6980	0.4384	0.2454	0.4675	0.7333	5.4000	0.6286
HAV_83_2010	2010	HAV	HAV_REWE	0.4506	0.1678	23.9118	0.4440	0.2376	0.4490	0.7255	5.2941	0.6134
HAV_84_2010	2010	HAV	HAV_REWE	0.8698	0.2579	27.3580	0.4648	0.2281	0.4266	0.6222	5.9677	0.7097
HAV_85_2010	2010	HAV	HAV_REWE	0.6609	0.2264	21.6843	0.3960	0.2469	0.4709	0.6508	6.1364	0.7338
HAV_86_2010	2010	HAV	HAV_REWE	0.5707	0.1572	61.1950	0.5716	0.1846	0.3242	0.5833	5.8333	0.6905
HAV_87_2010	2010	HAV	HAV_REWE	0.3505	0.1393	26.2370	0.4722	0.2168	0.4001	0.7143	5.7143	0.6735
HAV_88_2010	2010	HAV	HAV_REWE	0.4668	0.1764	26.5768	0.4711	0.2181	0.4032	0.5000	6.1000	0.7286
HAV_89_2010	2010	HAV	HAV_REWE	0.5939	0.2104	23.4168	0.4155	0.2094	0.3826	0.5417	6.0000	0.7143
HAV_9_2002	2002	HAV	HAV_BEFOR	0.5600	0.1930	20.1371	0.3964	0.2701	0.5256	0.6667	6.0667	0.7238
HAV_90_2010	2010	HAV	HAV_REWE	0.5930	0.2098	26.9465	0.4881	0.2176	0.4019	0.7544	5.7778	0.6825
HAV_91_2010	2010	HAV	HAV_REWE	0.9102	0.3006	25.8708	0.4727	0.1938	0.3460	0.8000	6.4000	0.7714
HAV_92_2010	2010	HAV	HAV_REWE	0.5605	0.2026	24.8971	0.4512	0.2124	0.3897	0.6528	6.0000	0.7143
HAV_93_2010	2010	HAV	HAV_REWE	0.7101	0.2230	36.2225	0.5548	0.2190	0.4053	0.4706	5.8667	0.6952
HAV_94_2010	2010	HAV	HAV_REWE	0.3625	0.1456	21.1732	0.3878	0.3255	0.6560	0.5333	5.4000	0.6286
HAV_95_2010	2010	HAV	HAV_REWE	0.5201	0.1955	20.2103	0.3758	0.2856	0.5621	0.5833	5.7500	0.6786
HAV_96_2010	2010	HAV	HAV_REWE	0.4875	0.1837	14.8343	0.2858	0.3548	0.7249	0.6667	5.5000	0.6429
HAV_97_2010	2010	HAV	HAV_REWE	0.4864	0.1835	24.5689	0.4239	0.3033	0.6036	0.5758	5.7273	0.6753
HAV_98_2010	2010	HAV	HAV_REWE	0.3922	0.1419	43.2624	0.5337	0.2242	0.4175	0.6667	5.7500	0.6786
HAV_99_2010	2010	HAV	HAV_REWE	0.5534	0.1972	24.4600	0.4224	0.2347	0.4423	0.6875	5.6471	0.6639
HO_1_04	2004	HO	HO_REWET	0.4953	0.1793	20.9465	0.3870	0.2424	0.4604	0.7121	5.3043	0.6149
HO_1_87	1987	HO	HO_BEFORE	0.4690	0.1746	21.7820	0.4131	0.2375	0.4487	0.7083	5.3333	0.6190
HO_3_04	2004	HO	HO_REWET	0.7914	0.2613	21.1843	0.4161	0.2561	0.4925	0.7407	5.2222	0.6032
KB_1994_10	1994	KB	KB_BEFORE	0.4623	0.1744	24.6934	0.4530	0.2167	0.3997	0.6667	5.3143	0.6163
KB_1994_13	1994	KB	KB_BEFORE	0.3332	0.1320	26.7353	0.4815	0.2177	0.4021	0.5632	4.7241	0.5320
KB_1994_17	1994	KB	KB_BEFORE	0.4682	0.1744	25.4933	0.4695	0.2122	0.3892	0.6026	5.2600	0.6086
KB_1994_18	1994	KB	KB_BEFORE	0.3746	0.1455	24.3287	0.4542	0.2177	0.4022	0.6111	5.2143	0.6020
KB_1994_19	1994	KB	KB_BEFORE	0.4027	0.1565	25.1735	0.4583	0.2204	0.4085	0.6333	5.1290	0.5899
KB_1994_20	1994	KB	KB_BEFORE	0.4492	0.1642	28.5199	0.5023	0.1932	0.3446	0.4800	4.8000	0.5429
KB_1994_23	1994	KB	KB_BEFORE	0.3787	0.1476	26.2925	0.4750	0.2072	0.3776	0.6000	5.0000	0.5714
KB_1994_7	1994	KB	KB_BEFORE	0.4241	0.1627	26.6047	0.4798	0.2115	0.3875	0.6667	5.3256	0.6179
KB_1994_8	1994	KB	KB_BEFORE	0.3923	0.1521	28.3610	0.4968	0.2100	0.3840	0.5702	5.0513	0.5788

KB_1997_7a	1997	KB	KB_BEFORE	0.3898	0.1524	28.5335	0.5025	0.2111	0.3867	0.6111	4.7778	0.5397
KB_1997_8a	1997	KB	KB_BEFORE	0.5354	0.1873	32.6572	0.5385	0.1587	0.2633	0.2917	4.7647	0.5378
KB_2008_10	2008	KB	KB_REWET	0.4004	0.1560	23.9213	0.4497	0.2416	0.4585	0.6667	5.3704	0.6243
KB_2008_13	2008	KB	KB_REWET	0.3823	0.1500	25.4165	0.4724	0.2257	0.4211	0.5797	5.3600	0.6229
KB_2008_17	2008	KB	KB_REWET	0.4526	0.1707	24.8330	0.4561	0.2381	0.4501	0.6800	5.2917	0.6131
KB_2008_18	2008	KB	KB_REWET	0.4306	0.1631	22.6425	0.4288	0.2465	0.4700	0.6396	5.2973	0.6139
KB_2008_19	2008	KB	KB_REWET	0.4496	0.1714	22.6750	0.4251	0.2316	0.4350	0.6847	5.4722	0.6389
KB_2008_20	2008	KB	KB_REWET	0.3651	0.1449	25.7380	0.4676	0.2321	0.4361	0.6140	5.2000	0.6000
KB_2008_23	2008	KB	KB_REWET	0.4332	0.1671	24.5818	0.4506	0.2392	0.4528	0.6500	5.2857	0.6122
KB_2008_7	2008	KB	KB_REWET	0.4170	0.1613	23.4304	0.4362	0.2357	0.4445	0.6863	5.5556	0.6508
KB_2008_7a	2008	KB	KB_REWET	0.4520	0.1712	21.9775	0.4091	0.2458	0.4682	0.6667	5.3684	0.6241
KB_2008_8	2008	KB	KB_REWET	0.4904	0.1843	24.4421	0.4497	0.2441	0.4644	0.6667	5.5625	0.6518
KB_2008_8a	2008	KB	KB_REWET	0.5072	0.1890	24.0358	0.4435	0.2189	0.4049	0.5965	5.4211	0.6316
KO_A1_12	2001	KO	KO_TSR	0.2275	0.0916	22.9206	0.4124	0.2593	0.5001	0.7436	5.6429	0.6633
KO_A2_13	1991	KO	KO_BEFORE	0.3950	0.1495	18.0114	0.3575	0.3304	0.6675	0.6296	5.5000	0.6429
KO_A3_23	2001	KO	KO_TSR	0.4060	0.1499	19.7263	0.3895	0.2690	0.5229	0.7222	5.7000	0.6714
KO_A4_24	1991	KO	KO_BEFORE	0.4714	0.1749	16.7184	0.3323	0.3418	0.6943	0.6667	5.7143	0.6735
KO_B1_35	2001	KO	KO_TSR	0.4073	0.1535	18.2801	0.3598	0.2688	0.5225	0.7544	5.7368	0.6767
KO_B2_36	1991	KO	KO_BEFORE	0.3517	0.1374	19.5260	0.3862	0.2583	0.4978	0.6190	5.5333	0.6476
KO_B3_47	2001	KO	KO_TSR	0.3783	0.1459	21.7566	0.4160	0.2586	0.4985	0.6500	5.4000	0.6286
KO_B4_48	1991	KO	KO_BEFORE	0.4356	0.1644	19.3017	0.3739	0.2658	0.5154	0.6905	5.5714	0.6531
KO_C_1	1991	KO	KO_REF	0.3346	0.1309	23.0865	0.4379	0.2166	0.3996	0.6026	5.4231	0.6319
KO_C_2	1991	KO	KO_REF	0.3958	0.1506	22.2460	0.4244	0.2316	0.4350	0.6000	5.5938	0.6563
KO_C_3	1991	KO	KO_REF	0.3994	0.1508	21.7188	0.4157	0.2348	0.4425	0.6429	5.6897	0.6700
KO_C_4	2001	KO	KO_REF	0.3527	0.1370	21.2934	0.4124	0.2351	0.4432	0.6296	5.5517	0.6502
KO_C_5	2001	KO	KO_REF	0.3678	0.1423	22.2992	0.4283	0.2347	0.4421	0.6282	5.6667	0.6667
KO_C_6	2001	KO	KO_REF	0.3929	0.1516	20.8207	0.4071	0.2538	0.4871	0.6667	5.6957	0.6708
KR_900001	1998	OUD	OUD_TSR	0.4785	0.1769	24.5270	0.4387	0.2006	0.3619	0.6667	5.2414	0.6059
KR_900006	1996	PBG	PBG_TSR	0.4119	0.1566	25.5464	0.4611	0.1978	0.3553	0.5586	5.2051	0.6007
KR_900013	1997	PBG	PBG_TSR	0.4463	0.1669	24.2892	0.4409	0.1975	0.3547	0.5856	5.5000	0.6429
KR_900019	1996	PBG	PBG_TSR	0.4251	0.1585	26.0943	0.4655	0.1801	0.3138	0.5789	5.3421	0.6203
KR_900026	1997	PBG	PBG_TSR	0.5352	0.1869	26.2565	0.4405	0.2063	0.3753	0.5758	5.5217	0.6460
KR_900032	1997	PBG	PBG_TSR	0.6102	0.2061	28.1942	0.4528	0.1979	0.3556	0.5111	5.3333	0.6190
KR_900038	1997	PBG	PBG_TSR	0.6152	0.1994	23.5920	0.4310	0.1920	0.3416	0.6167	5.3333	0.6190
KR_900048	1994	PBN	PBN_TSR	0.4727	0.1752	26.6760	0.4745	0.1959	0.3509	0.5903	5.4200	0.6314
KR_900057	1994	PBN	PBN_TSR	0.5644	0.1987	28.3723	0.4749	0.1824	0.3191	0.5619	5.8571	0.6939
KR_900070	1994	PBN	PBN_TSR	0.5103	0.1824	26.2553	0.4659	0.1930	0.3441	0.5833	5.4722	0.6389
KR_900079	1994	PBN	PBN_TSR	0.4866	0.1786	25.0776	0.4442	0.1948	0.3483	0.5333	5.9000	0.7000
KR_900104	1998	PMB	PMB_TSR	0.7648	0.2313	23.6812	0.4322	0.2059	0.3744	0.6571	5.4865	0.6409
KR_900106	1999	PMB	PMB_TSR	0.4484	0.1708	23.4814	0.4286	0.2118	0.3882	0.7037	5.2000	0.6000
KR_900109	1999	PMB	PMB_TSR	0.5012	0.1874	20.3205	0.3782	0.2737	0.5341	0.7179	5.3846	0.6264
KR_900112	1997	COM	COM_TSR	0.4459	0.1662	25.0118	0.4545	0.1834	0.3214	0.5882	5.3889	0.6270
KR_900118	1997	COM	COM_TSR	0.4371	0.1644	24.0613	0.4435	0.1791	0.3115	0.6364	5.3939	0.6277
KR_900124	1997	COM	COM_TSR	0.3454	0.1352	24.9431	0.4538	0.1856	0.3266	0.5909	5.1739	0.5963
KR_900130	1997	COM	COM_TSR	0.5908	0.2077	23.3202	0.4252	0.1869	0.3296	0.6452	5.7576	0.6797
KR_900136	1998	PVB	PVB_TSR	0.5173	0.1865	23.8492	0.4337	0.1992	0.3585	0.6790	5.7500	0.6786
KR_900140	1998	PVB	PVB_TSR	0.5774	0.2022	20.6373	0.3863	0.2149	0.3955	0.5778	5.7500	0.6786
KR_900144	1998	PVB	PVB_TSR	0.6984	0.2196	22.4962	0.4135	0.2002	0.3611	0.7204	5.6970	0.6710
KR_900190	2007	PBG	PBG_TSR	0.4067	0.1533	21.3708	0.4049	0.2474	0.4721	0.6667	5.5938	0.6563
KR_900192	2007	PBG	PBG_TSR	0.4115	0.1553	22.3173	0.4153	0.2361	0.4456	0.6795	5.5556	0.6508
KR_900193	2007	PBG	PBG_TSR	0.4044	0.1517	21.1502	0.4009	0.2444	0.4650	0.7692	5.9286	0.7041
KR_900194	2007	PBG	PBG_TSR	0.3032	0.1205	23.8276	0.4407	0.2394	0.4532	0.7273	5.5385	0.6484
KR_900204	2007	PVB	PVB_TSR	0.4569	0.1725	23.7377	0.4297	0.2184	0.4039	0.6806	5.8261	0.6894
KR_900218	2008	COM	COM_TSR	0.4167	0.1583	22.8170	0.4266	0.2428	0.4613	0.6556	5.2813	0.6116
KR_900219	2008	COM	COM_TSR	0.4915	0.1762	22.0697	0.4146	0.2205	0.4088	0.6774	5.5938	0.6563
KR_900220	2008	COM	COM_TSR	0.7759	0.2234	20.8498	0.3952	0.2279	0.4262	0.6667	5.8333	0.6905
KR_900225	2010	OUD	OUD_TSR	0.5914	0.2101	23.6498	0.4352	0.2160	0.3981	0.6917	5.3810	0.6259
KR_900226	2011	PBN	PBN_TSR	0.4290	0.1610	23.4352	0.4397	0.2111	0.3866	0.6667	5.3333	0.6190
KR_900227	2011	PMB	PMB_TSR	0.6668	0.2308	23.3123	0.4303	0.2294	0.4296	0.6782	5.6000	0.6571
KR_900228	2011	COM	COM_TSR	0.5661	0.1996	22.3287	0.4197	0.2339	0.4404	0.6481	5.8108	0.6873
KR_900230	2012	PBG	PBG_TSR	0.4463	0.1638	20.6393	0.3910	0.2498	0.4778	0.6842	5.6000	0.6571
KR_900231	2012	PBG	PBG_TSR	0.4950	0.1814	21.5650	0.4055	0.2374	0.4485	0.6800	5.5385	0.6484
KR_900232	2012	PBG	PBG_TSR	0.4511	0.1673	22.0717	0.4181	0.2342	0.4410	0.6933	5.5000	0.6429
KR_900233	2012	PBN	PBN_TSR	0.4660	0.1742	22.3900	0.4207	0.2128	0.3906	0.6286	5.5000	0.6429
KR_900234	2012	PBN	PBN_TSR	0.4682	0.1750	22.5006	0.4201	0.2176	0.4019	0.6752	5.6250	0.6607
KR_900235	2012	PBN	PBN_TSR	0.4497	0.1678	19.6953	0.3742	0.2340	0.4407	0.6875	5.6471	0.6639
KR_900236	2012	PBN	PBN_TSR	0.4217	0.1606	22.2427	0.4183	0.2421	0.4597	0.6173	5.3214	0.6173
KR_900237	2012	PMB	PMB_TSR	0.4680	0.1744	23.8217	0.4434	0.2164	0.3992	0.6333	5.1818	0.5974
KR_900238	2012	PMB	PMB_TSR	0.5610	0.1967	25.0913	0.4581	0.2273	0.4248	0.6667	5.4000	0.6286
KR_900239	2013	PBN	PBN_TSR	0.3932	0.1520	23.2733	0.4326	0.2344	0.4414	0.6179	5.2500	0.6071
KR_900240	2013	PBN	PBN_TSR	0.4449	0.1667	21.8713	0.4110	0.2372	0.4481	0.6522	5.3333	0.6190
KR_900241	2013	PBN	PBN_TSR	0.4245	0.1614	21.6182	0.4096	0.2423	0.4602	0.7000	5.3750	0.6250
KR_900272	2011	PBN	PBN_TSR	0.4494	0.1687	22.7291	0.4281	0.2190	0.4054	0.6757	5.2821	0.6117
KR_900273	2011	PBN	PBN_TSR	0.4703	0.1722	22.7334	0.4219	0.2081	0.3797	0.6917	5.4286	0.6327
LA_1_2007	2007	LA	LA_BEFORE	0.5135	0.1917	27.1972	0.4890	0.2337	0.4398	0.6444	5.3333	0.6190
LA_10_2007	2007	LA	LA_BEFORE	0.6119	0.2174	27.3943	0.4253	0.2339	0.4403	0.7667	6.0000	0.7143
LA_11_2007	2007	LA	LA_BEFORE	0.4892	0.1821	28.8115	0.5072	0.1854	0.3263	0.6970	5.4545	0.6364

LA_12_2007	2007	LA	LA_BEFORE	0.5309	0.1968	25.5602	0.4654	0.2097	0.3833	0.6508	5.7619	0.6803
LA_13_2007	2007	LA	LA_BEFORE	0.5230	0.1895	25.8020	0.4638	0.2102	0.3845	0.6667	5.2500	0.6071
LA_14_2007	2007	LA	LA_BEFORE	0.5056	0.1833	24.6100	0.4504	0.2298	0.4306	0.7255	5.4118	0.6303
LA_15_2007	2007	LA	LA_BEFORE	0.5767	0.2050	26.7352	0.4721	0.2212	0.4104	0.7333	5.7000	0.6714
LA_16_2007	2007	LA	LA_BEFORE	0.6364	0.2230	22.7065	0.4284	0.2342	0.4410	0.6667	5.5909	0.6558
LA_19_2007	2007	LA	LA_BEFORE	0.5106	0.1838	24.7914	0.4471	0.2286	0.4279	0.7407	5.3333	0.6190
LA_2_2007	2007	LA	LA_BEFORE	0.6411	0.2180	23.2849	0.4292	0.2772	0.5422	0.7692	5.6154	0.6593
LA_20_2007	2007	LA	LA_BEFORE	0.5550	0.2003	23.7103	0.4228	0.2268	0.4235	0.7436	5.4615	0.6374
LA_21_2007	2007	LA	LA_BEFORE	0.5021	0.1838	25.3530	0.4699	0.2341	0.4408	0.6667	5.1111	0.5873
LA_22_2007	2007	LA	LA_BEFORE	0.5887	0.2136	24.8481	0.4613	0.2264	0.4227	0.6316	5.3889	0.6270
LA_3_2007	2007	LA	LA_BEFORE	0.4183	0.1602	25.7294	0.4623	0.1942	0.3468	0.7059	5.1765	0.5966
LA_59_2013	2013	LA	LA_REWET	0.4411	0.1504	34.9944	0.5166	0.1217	0.1763	0.5000	5.8750	0.6964
LA_6_2007	2007	LA	LA_BEFORE	0.5489	0.1973	27.3723	0.4797	0.1907	0.3387	0.7111	5.2000	0.6000
LA_60_2013	2013	LA	LA_REWET	0.3000	0.1041	50.6100	0.6405	0.1639	0.2756	0.5000	5.6000	0.6571
LA_61_2013	2013	LA	LA_REWET	0.7050	0.2090	46.6580	0.6152	0.1789	0.3109	0.6667	5.5000	0.6429
LA_62_2013	2013	LA	LA_REWET	0.7050	0.2090	46.6580	0.6152	0.1789	0.3109	0.6667	5.5000	0.6429
LA_63_2013	2013	LA	LA_REWET	0.4058	0.1529	22.2500	0.4250	0.2523	0.4837	0.5833	5.6071	0.6582
LA_64_2013	2013	LA	LA_REWET	0.6604	0.2284	22.4764	0.3573	0.2486	0.4749	0.6667	5.7000	0.6714
LA_65_2013	2013	LA	LA_REWET	0.6372	0.2260	24.7011	0.4448	0.1883	0.3330	0.6508	6.0952	0.7279
LA_66_2013	2013	LA	LA_REWET	0.6923	0.2368	23.6438	0.4424	0.2366	0.4468	0.7719	5.9474	0.7068
LA_67_2013	2013	LA	LA_REWET	0.6621	0.2273	23.6397	0.4279	0.2183	0.4037	0.6667	5.9167	0.7024
LA_68_2013	2013	LA	LA_REWET	0.6429	0.2113	29.4257	0.4578	0.2008	0.3623	0.5714	5.7143	0.6735
LA_69_2013	2013	LA	LA_REWET	0.7125	0.2415	20.7178	0.3915	0.2264	0.4228	0.7083	5.9375	0.7054
LA_70_2013	2013	LA	LA_REWET	0.6650	0.2211	25.9751	0.4256	0.2350	0.4429	0.6429	5.9286	0.7041
LA_71_2013	2013	LA	LA_REWET	0.4500	0.1676	22.7957	0.4294	0.2315	0.4346	0.7333	5.1500	0.5929
LA_72_2013	2013	LA	LA_REWET	0.5495	0.1999	22.2499	0.4168	0.2579	0.4968	0.7222	5.3333	0.6190
LA_73_2013	2013	LA	LA_REWET	0.5144	0.1906	27.2368	0.4806	0.2469	0.4710	0.7667	5.2000	0.6000
LA_74_2013	2013	LA	LA_REWET	0.6576	0.2283	23.0509	0.4187	0.2315	0.4346	0.6667	5.8462	0.6923
LA_9_2007	2007	LA	LA_BEFORE	0.4518	0.1677	22.1522	0.4242	0.2509	0.4804	0.6092	5.7586	0.6798
LM_101	2005	LM	LM_TSR	0.5068	0.1867	19.2395	0.3729	0.2276	0.4256	0.6042	5.2353	0.6050
LM_102	2005	LM	LM_TSR	0.5716	0.2053	21.4392	0.3999	0.1944	0.3474	0.6863	5.2353	0.6050
LM_104	2005	LM	LM_TSR	0.4319	0.1653	22.0981	0.4189	0.2007	0.3621	0.6377	5.2174	0.6025
LM_105	2005	LM	LM_TSR	0.4659	0.1737	21.3002	0.4091	0.2199	0.4074	0.6481	5.2222	0.6032
LM_112	2008	LM	LM_TSR	0.4351	0.1641	21.8249	0.4110	0.2042	0.3704	0.6250	5.4000	0.6286
LM_113	2008	LM	LM_TSR	0.5055	0.1882	24.0228	0.4484	0.2120	0.3889	0.6400	5.5385	0.6484
LM_114	2008	LM	LM_TSR	0.4265	0.1636	23.0129	0.4257	0.2085	0.3805	0.6833	5.4500	0.6357
LM_115	2008	LM	LM_TSR	0.4991	0.1854	20.6773	0.3944	0.2014	0.3637	0.6491	5.4000	0.6286
LM_116	2008	LM	LM_TSR	0.5926	0.2141	24.0028	0.4379	0.2340	0.4405	0.6875	5.3750	0.6250
LM_117	2008	LM	LM_TSR	0.4321	0.1654	23.2199	0.4283	0.2180	0.4030	0.5926	5.4815	0.6402
LM_118	2008	LM	LM_TSR	0.4685	0.1748	23.2113	0.4352	0.2000	0.3604	0.6364	5.2609	0.6087
LM_119	2008	LM	LM_TSR	0.4196	0.1595	23.3222	0.4342	0.1976	0.3549	0.6515	5.3043	0.6149
LM_120	2008	LM	LM_TSR	0.4214	0.1610	24.5228	0.4517	0.2044	0.3708	0.6232	5.2609	0.6087
LM_121	2008	LM	LM_TSR	0.4371	0.1661	22.5286	0.4239	0.2186	0.4044	0.6944	5.2400	0.6057
LM_122	2008	LM	LM_TSR	0.4778	0.1777	22.5638	0.4208	0.2227	0.4141	0.6957	5.4783	0.6398
LM_123	2008	LM	LM_TSR	0.4524	0.1705	23.6376	0.4376	0.2077	0.3786	0.6667	5.4138	0.6305
LM_124	1995	LM	LM_BEFORE	0.3839	0.1516	26.3862	0.4845	0.2409	0.4568	0.6389	5.2500	0.6071
LM_125	1995	LM	LM_BEFORE	0.3709	0.1471	26.3631	0.4847	0.2173	0.4013	0.6429	5.2143	0.6020
LM_126	1995	LM	LM_BEFORE	0.4989	0.1834	23.5262	0.4415	0.2393	0.4531	0.6852	5.3158	0.6165
LM_127	1995	LM	LM_BEFORE	0.4768	0.1790	23.5247	0.4414	0.2256	0.4207	0.6275	5.2778	0.6111
LM_128	1995	LM	LM_BEFORE	0.3702	0.1444	25.5374	0.4741	0.2226	0.4138	0.6222	5.2000	0.6000
LM_129	1995	LM	LM_BEFORE	0.3745	0.1473	24.2088	0.4422	0.2059	0.3743	0.6481	5.2778	0.6111
LM_130	1995	LM	LM_BEFORE	0.4899	0.1820	22.7618	0.4252	0.2380	0.4500	0.6667	5.3889	0.6270
LM_131	1995	LM	LM_BEFORE	0.3833	0.1505	24.7263	0.4519	0.2230	0.4147	0.6032	5.2857	0.6122
MOS_1	1999	MOS	MOS_TSR	0.2803	0.1120	17.4531	0.3478	0.2938	0.5814	0.6250	6.1000	0.7286
MOS_4	2012	MOS	MOS_TSR	0.2215	0.0904	22.6585	0.4220	0.2473	0.4718	0.5333	5.8750	0.6964
NP_1	2007	NP	NP_TSR	0.6261	0.2130	16.3774	0.3069	0.3037	0.6046	0.7778	5.5714	0.6531
NP_11	2007	NP	NP_TSR	0.7786	0.2522	15.6951	0.2900	0.3177	0.6377	0.8667	6.1429	0.7347
NP_14	2007	NP	NP_TSR	0.5711	0.1992	18.4563	0.3396	0.3039	0.6051	0.8095	6.0000	0.7143
NP_16	2007	NP	NP_TSR	0.6779	0.2266	17.1317	0.3197	0.2927	0.5788	0.7778	5.8571	0.6939
NP_19	2007	NP	NP_TSR	0.5606	0.1915	20.7246	0.3669	0.2571	0.4949	0.8095	5.7500	0.6786
NP_21	2007	NP	NP_TSR	0.6040	0.1999	18.5912	0.3394	0.3273	0.6601	0.8333	5.6000	0.6571
NP_24	2007	NP	NP_TSR	0.6700	0.2213	18.7276	0.3495	0.3100	0.6195	0.7333	5.8333	0.6905
NP_26	2007	NP	NP_TSR	0.7401	0.2399	16.2945	0.3052	0.2999	0.5957	0.8333	5.7143	0.6735
NP_29	2007	NP	NP_TSR	0.6557	0.2137	18.5871	0.3379	0.2726	0.5315	0.8095	5.6250	0.6607
NP_4	2007	NP	NP_TSR	0.6242	0.2096	16.7520	0.3062	0.3042	0.6058	0.8667	5.6667	0.6667
NP_6	2007	NP	NP_TSR	0.5224	0.1751	19.4926	0.3596	0.3234	0.6509	0.7333	5.5000	0.6429
NP_9	2007	NP	NP_TSR	0.7426	0.2369	17.7510	0.3327	0.3184	0.6392	0.8000	5.6667	0.6667
OOS_1	2000	OOS	OOS_REWE	0.2144	0.0902	19.1645	0.3826	0.3384	0.6863	0.5556	5.3636	0.6234
OOS_10	2000	OOS	OOS_REWE	0.4148	0.1593	25.3871	0.4629	0.2190	0.4052	0.6842	5.4211	0.6316
OOS_11	2000	OOS	OOS_REWE	1.0086	0.2856	16.2854	0.3199	0.3123	0.6248	0.6389	6.0000	0.7143
OOS_12	2000	OOS	OOS_REWE	0.3882	0.1453	20.4578	0.3799	0.2799	0.5486	0.7333	5.9167	0.7024
OOS_13	2000	OOS	OOS_REWE	0.7274	0.2416	33.3931	0.4953	0.1955	0.3500	0.7083	6.1200	0.7314
OOS_14	2000	OOS	OOS_REWE	0.8972	0.2960	21.5662	0.4112	0.2789	0.5462	0.8667	6.2000	0.7429
OOS_15	2000	OOS	OOS_REWE	0.3899	0.1496	15.0130	0.3062	0.3856	0.7973	0.7500	6.0000	0.7143
OOS_2	2000	OOS	OOS_REWE	0.4069	0.1560	18.4388	0.3707	0.3754	0.7733	0.6667	6.4000	0.7714
OOS_3	2000	OOS	OOS_REWE	0.5985	0.2136	14.2490	0.2947	0.3525	0.7196	0.8333	5.7500	0.6786
OOS_32	2004	OOS	OOS_REWE	0.4885	0.1761	30.1258	0.5013	0.1745	0.3006	0.4603	6.3182	0.7597

OOS_4	2000	OOS	OOS_REWE	0.6819	0.2361	21.0294	0.3797	0.2351	0.4431	0.7083	5.9375	0.7054
OOS_5	2000	OOS	OOS_REWE	0.3251	0.1315	27.1974	0.4943	0.2280	0.4264	0.7407	5.0000	0.5714
OOS_50	2014	OOS	OOS_REWE	0.2270	0.0952	15.3773	0.3148	0.3518	0.7178	0.5714	5.3333	0.6190
OOS_51	2014	OOS	OOS_REWE	0.2419	0.1009	17.0034	0.3424	0.3317	0.6706	0.5714	5.4444	0.6349
OOS_52	2014	OOS	OOS_REWE	0.3441	0.1350	17.0067	0.3386	0.2678	0.5200	0.5833	5.6250	0.6607
OOS_53	2014	OOS	OOS_REWE	0.5083	0.1896	18.3583	0.3688	0.3503	0.7143	0.6667	6.3333	0.7619
OOS_54	2014	OOS	OOS_REWE	0.5400	0.2014	14.4164	0.3069	0.4273	0.8955	0.8333	7.0000	0.8571
OOS_55	2014	OOS	OOS_REWE	0.7324	0.2512	17.3684	0.3364	0.2566	0.4938	0.6429	6.0000	0.7143
OOS_56	2014	OOS	OOS_REWE	0.3947	0.1545	24.8147	0.4503	0.2256	0.4209	0.7000	5.1000	0.5857
OOS_57	2014	OOS	OOS_REWE	0.8325	0.2802	16.5252	0.3235	0.3124	0.6251	0.6667	5.9000	0.7000
OOS_58	2014	OOS	OOS_REWE	0.5595	0.2015	18.9599	0.3581	0.3079	0.6145	0.6410	5.8462	0.6923
OOS_59	2014	OOS	OOS_REWE	0.9458	0.3060	20.7568	0.3940	0.2772	0.5422	0.7083	6.3750	0.7679
OOS_6	2000	OOS	OOS_REWE	0.7438	0.2533	25.0997	0.3974	0.2731	0.5325	0.5417	6.1000	0.7286
OOS_60	2014	OOS	OOS_REWE	0.1600	0.0557	47.4948	0.6156	0.1684	0.2862	0.6667	5.6000	0.6571
OOS_61	2014	OOS	OOS_REWE	0.5074	0.1834	26.5386	0.4642	0.2084	0.3804	0.6667	5.1250	0.5893
OOS_62	2014	OOS	OOS_REWE	1.2514	0.3270	14.9521	0.2946	0.3220	0.6477	0.7619	5.6667	0.6667
OOS_63	2014	OOS	OOS_REWE	0.4516	0.1672	17.7258	0.3449	0.3159	0.6332	0.7037	6.1000	0.7286
OOS_64	2014	OOS	OOS_REWE	0.6298	0.2172	26.3659	0.4437	0.2178	0.4024	0.6250	6.1875	0.7411
OOS_65	2014	OOS	OOS_REWE	0.8372	0.2779	25.7140	0.4488	0.2154	0.3967	0.7719	5.8421	0.6917
OOS_7	2000	OOS	OOS_REWE	0.5608	0.2036	20.3233	0.3880	0.2551	0.4902	0.6852	5.7368	0.6767
OOS_8	2000	OOS	OOS_REWE	0.9063	0.2890	21.4961	0.4053	0.2577	0.4963	0.8095	5.5714	0.6531
OOS_9	2000	OOS	OOS_REWE	0.2286	0.0796	60.6792	0.6655	0.1428	0.2258	0.7222	5.7143	0.6735
PLI_B1_14	2014	PLI	PLI_REWET	0.4897	0.1828	27.7368	0.4850	0.2213	0.4108	0.6833	5.6000	0.6571
PLI_B1_95	1995	PLI	PLI_BEFORE	0.4345	0.1635	24.1160	0.4447	0.2317	0.4352	0.6667	5.4857	0.6408
PLI_B2_14	2014	PLI	PLI_REWET	0.3981	0.1509	24.4528	0.4452	0.2277	0.4257	0.6111	5.5417	0.6488
PLI_B2_95	1995	PLI	PLI_BEFORE	0.3955	0.1505	23.5174	0.4399	0.2349	0.4426	0.6162	5.4848	0.6407
PLI_B3_14	2014	PLI	PLI_REWET	0.4030	0.1551	24.6981	0.4514	0.2310	0.4335	0.6437	5.4483	0.6355
PLI_B3_95	1995	PLI	PLI_BEFORE	0.4366	0.1647	23.4882	0.4351	0.2374	0.4485	0.6571	5.3714	0.6245
PLI_B4_14	2014	PLI	PLI_REWET	0.4326	0.1620	23.8902	0.4446	0.2175	0.4016	0.6667	5.6923	0.6703
PLI_B4_95	1995	PLI	PLI_BEFORE	0.3790	0.1463	23.1483	0.4355	0.2319	0.4357	0.5980	5.4242	0.6320
PLI_B5_14	2014	PLI	PLI_REWET	0.6121	0.2169	24.4151	0.4463	0.2127	0.3905	0.6800	5.6800	0.6686
PLI_B5_95	1995	PLI	PLI_BEFORE	0.4445	0.1666	24.0145	0.4429	0.2277	0.4257	0.6667	5.3571	0.6224
PLI_B6_14	2014	PLI	PLI_REWET	0.7186	0.2460	24.6481	0.4292	0.2235	0.4159	0.6377	5.7391	0.6770
PLI_B6_95	1995	PLI	PLI_BEFORE	0.5575	0.2001	25.3571	0.4345	0.2199	0.4073	0.6667	5.3125	0.6161
RR_1_1998	1998	RR	RR_BEFORE	0.4904	0.1813	23.5518	0.4391	0.2264	0.4226	0.6875	5.4375	0.6339
RR_10_1998	1998	RR	RR_BEFORE	0.4451	0.1707	24.9904	0.4556	0.2107	0.3857	0.6889	5.1333	0.5905
RR_100_1998	1998	RR	RR_BEFORE	0.5651	0.2031	25.1499	0.4710	0.2612	0.5046	0.7333	5.4000	0.6286
RR_117_1998	1998	RR	RR_BEFORE	0.9270	0.2859	27.6728	0.4804	0.2352	0.4434	0.6667	5.7143	0.6735
RR_124_1998	1998	RR	RR_BEFORE	0.5499	0.1995	25.6637	0.4539	0.2326	0.4373	0.7179	5.1538	0.5934
RR_133_1998	1998	RR	RR_BEFORE	0.5946	0.2067	27.2950	0.4928	0.2571	0.4948	0.8667	5.6000	0.6571
RR_142_1998	1998	RR	RR_BEFORE	0.4808	0.1779	23.4860	0.4374	0.2314	0.4343	0.7059	5.4706	0.6387
RR_151_1998	1998	RR	RR_BEFORE	0.4385	0.1643	24.7809	0.4513	0.2203	0.4082	0.7451	5.2353	0.6050
RR_160_1998	1998	RR	RR_BEFORE	0.4701	0.1737	25.4329	0.4735	0.2360	0.4453	0.6667	5.5833	0.6548
RR_169_1998	1998	RR	RR_BEFORE	0.8311	0.2741	27.8944	0.4843	0.2149	0.3956	0.7576	6.0455	0.7208
RR_177_1998	1998	RR	RR_BEFORE	0.7477	0.2537	27.1535	0.4817	0.2247	0.4188	0.7333	5.9500	0.7071
RR_185_1998	1998	RR	RR_BEFORE	0.7973	0.2590	26.2579	0.3956	0.2390	0.4523	0.7222	5.5000	0.6429
RR_19_1998	1998	RR	RR_BEFORE	0.5382	0.1964	23.5636	0.4333	0.2655	0.5146	0.7778	5.6667	0.6667
RR_194_1998	1998	RR	RR_BEFORE	0.5313	0.1936	23.9132	0.4377	0.2354	0.4439	0.7586	5.5862	0.6552
RR_203_1998	1998	RR	RR_BEFORE	0.6341	0.2265	21.8423	0.4146	0.2619	0.5063	0.6833	5.3000	0.6143
RR_212_1998	1998	RR	RR_BEFORE	0.7272	0.2526	20.5247	0.4014	0.2654	0.5144	0.7778	5.2667	0.6095
RR_221_1998	1998	RR	RR_BEFORE	0.4850	0.1797	24.3104	0.4513	0.2214	0.4110	0.7193	5.3684	0.6241
RR_230_1998	1998	RR	RR_BEFORE	0.4667	0.1725	24.9117	0.4568	0.2289	0.4286	0.7556	5.2667	0.6095
RR_239_1998	1998	RR	RR_BEFORE	0.6207	0.2209	25.5079	0.4473	0.2328	0.4377	0.6905	5.7561	0.6794
RR_246_2012	2012	RR	RR_REWET	0.6985	0.2447	23.5846	0.4319	0.2314	0.4343	0.6970	6.0000	0.7143
RR_247_2012	2012	RR	RR_REWET	0.5959	0.2101	26.4246	0.4248	0.2612	0.5047	0.7037	5.5556	0.6508
RR_249_2012	2012	RR	RR_REWET	0.6296	0.2134	26.3390	0.4321	0.1962	0.3517	0.6389	5.8333	0.6905
RR_250_2012	2012	RR	RR_REWET	0.6569	0.2228	27.1643	0.4580	0.2013	0.3637	0.5641	5.6429	0.6633
RR_251_2012	2012	RR	RR_REWET	0.6600	0.2359	23.9257	0.4384	0.2500	0.4783	0.7333	5.2000	0.6000
RR_252_2012	2012	RR	RR_REWET	0.7582	0.2624	21.5679	0.3941	0.2354	0.4439	0.7436	5.7692	0.6813
RR_253_2012	2012	RR	RR_REWET	0.6377	0.2019	32.3688	0.4959	0.1799	0.3133	0.5556	5.6923	0.6703
RR_254_2012	2012	RR	RR_REWET	0.5117	0.1617	49.3744	0.6276	0.1526	0.2489	0.7143	5.4286	0.6327
RR_255_2012	2012	RR	RR_REWET	0.6214	0.2045	29.9321	0.4643	0.1638	0.2754	0.5556	5.7143	0.6735
RR_256_2012	2012	RR	RR_REWET	0.5005	0.1712	38.2049	0.5331	0.1887	0.3340	0.6667	5.4444	0.6349
RR_257_2012	2012	RR	RR_REWET	0.7143	0.2462	25.4246	0.4570	0.1659	0.2802	0.7222	6.0000	0.7143
RR_258_2012	2012	RR	RR_REWET	0.7234	0.2520	21.8806	0.3982	0.2937	0.5811	0.7222	5.6667	0.6667
RR_259_2012	2012	RR	RR_REWET	0.5398	0.1890	38.1391	0.5280	0.1971	0.3537	0.6522	5.5833	0.6548
RR_260_2012	2012	RR	RR_REWET	0.5825	0.1892	33.4539	0.5062	0.1671	0.2831	0.5667	5.7000	0.6714
RR_261_2012	2012	RR	RR_REWET	0.5050	0.1519	43.6792	0.5865	0.1648	0.2776	0.6667	5.2000	0.6000
RR_262_2012	2012	RR	RR_REWET	0.4708	0.1741	24.3608	0.4513	0.2485	0.4747	0.6667	5.5455	0.6494
RR_263_2012	2012	RR	RR_REWET	0.5536	0.1978	27.6221	0.4585	0.2222	0.4129	0.6889	5.6667	0.6667
RR_264_2012	2012	RR	RR_REWET	0.5126	0.1852	29.1959	0.4851	0.2219	0.4121	0.6154	5.9231	0.7033
RR_265_2012	2012	RR	RR_REWET	0.3538	0.1180	64.1884	0.6874	0.1572	0.2598	0.6667	5.5714	0.6531
RR_266_2012	2012	RR	RR_REWET	0.3538	0.1180	64.1884	0.6874	0.1572	0.2598	0.6667	5.5714	0.6531
RR_267_2012	2012	RR	RR_REWET	0.5063	0.1657	65.2200	0.7473	0.1372	0.2128	0.3333	5.3333	0.6190
RR_268_2012	2012	RR	RR_REWET	0.5092	0.1765	52.1634	0.6088	0.1656	0.2796	0.7037	5.6000	0.6571
RR_269_2012	2012	RR	RR_REWET	0.5915	0.1836	71.2230	0.6886	0.1354	0.2085	0.5556	5.6000	0.6571
RR_270_2012	2012	RR	RR_REWET	0.4775	0.1644	57.2166	0.6260	0.1850	0.3254	0.7143	5.5000	0.6429

RR_271_2012	2012	RR	RR_REWET	0.4260	0.1628	23.7739	0.4328	0.2164	0.3992	0.7255	5.7059	0.6723
RR_272_2012	2012	RR	RR_REWET	0.6025	0.2138	29.3494	0.5079	0.1943	0.3472	0.6667	5.9286	0.7041
RR_273_2012	2012	RR	RR_REWET	0.6156	0.2145	31.4322	0.4731	0.2209	0.4097	0.7308	5.6667	0.6667
RR_28_1998	1998	RR	RR_BEFORE	0.4392	0.1649	27.1114	0.4860	0.2105	0.3853	0.6471	5.0588	0.5798
RR_37_1998	1998	RR	RR_BEFORE	0.4965	0.1821	27.2711	0.4869	0.2227	0.4141	0.6389	4.7500	0.5357
RR_46_1998	1998	RR	RR_BEFORE	0.4990	0.1875	25.5765	0.4568	0.2210	0.4100	0.6275	5.3529	0.6218
RR_55_1998	1998	RR	RR_BEFORE	0.5704	0.2080	26.4757	0.4699	0.2146	0.3948	0.7059	5.5294	0.6471
RR_64_1998	1998	RR	RR_BEFORE	0.5119	0.1847	24.1136	0.4540	0.2458	0.4682	0.7500	5.3750	0.6250
RR_73_1998	1998	RR	RR_BEFORE	0.6019	0.2160	25.8098	0.4745	0.2745	0.5358	0.8333	5.7500	0.6786
RR_82_1998	1998	RR	RR_BEFORE	0.5038	0.1850	26.8254	0.4866	0.2334	0.4391	0.7273	5.0909	0.5844
RR_91_1998	1998	RR	RR_BEFORE	0.7594	0.2518	33.9505	0.5018	0.2027	0.3670	0.6667	5.6250	0.6607
SE_A1_2010	2010	SE	SE_REWET	0.6596	0.2020	14.3541	0.2991	0.3694	0.7591	0.6364	5.3846	0.6264
SE_A1a_2010	2010	SE	SE_REWET	0.3443	0.1347	17.3811	0.3543	0.3424	0.6956	0.5833	5.8750	0.6964
SE_A1b_2010	2010	SE	SE_REWET	0.3259	0.1315	18.5950	0.3766	0.3694	0.7592	0.6000	5.8000	0.6857
SE_A2_2010	2010	SE	SE_REWET	0.4730	0.1671	15.6221	0.3199	0.3418	0.6944	0.6250	5.5556	0.6508
SE_A2a_2010	2010	SE	SE_REWET	0.5281	0.1818	14.7119	0.3054	0.3461	0.7043	0.5238	5.6250	0.6607
SE_A2b_2010	2010	SE	SE_REWET	0.5492	0.1900	15.6227	0.3207	0.3652	0.7494	0.6111	5.5714	0.6531
SE_A3_2010	2010	SE	SE_REWET	0.3509	0.1377	16.0629	0.3248	0.3532	0.7212	0.6250	5.7500	0.6786
SE_A3a_2010	2010	SE	SE_REWET	0.2658	0.1089	18.0192	0.3608	0.3198	0.6426	0.5000	5.6667	0.6667
SE_A3b_2010	2010	SE	SE_REWET	0.3230	0.1275	15.3303	0.3110	0.3496	0.7127	0.6296	5.6667	0.6667
SE_A4_2010	2010	SE	SE_REWET	0.4605	0.1624	14.0475	0.2876	0.3658	0.7508	0.6250	5.4444	0.6349
SE_A4a_2010	2010	SE	SE_REWET	0.5471	0.1892	13.5470	0.2836	0.3676	0.7550	0.6667	5.5714	0.6531
SE_A4b_2010	2010	SE	SE_REWET	0.3018	0.1183	14.5716	0.2964	0.3346	0.6774	0.5833	5.7500	0.6786
SE_A5_2010	2010	SE	SE_REWET	0.2605	0.1068	15.2256	0.3078	0.3268	0.6591	0.5556	5.7778	0.6825
SE_A5a_2010	2010	SE	SE_REWET	0.3662	0.1395	15.4042	0.3166	0.3387	0.6869	0.5556	5.9167	0.7024
SE_A5b_2010	2010	SE	SE_REWET	0.5097	0.1754	16.6675	0.3360	0.3489	0.7109	0.6250	5.6250	0.6607
SE_B1_2010	2010	SE	SE_REWET	0.4400	0.1675	14.5173	0.3039	0.4032	0.8388	0.7500	6.2500	0.7500
SE_B1a_2010	2010	SE	SE_REWET	0.4263	0.1620	14.5559	0.3052	0.4090	0.8525	0.8333	6.2500	0.7500
SE_B1b_2010	2010	SE	SE_REWET	0.3478	0.1349	19.8223	0.4016	0.3072	0.6128	0.6111	6.1667	0.7381
SE_B2_2010	2010	SE	SE_REWET	0.3191	0.1245	18.6646	0.3708	0.3801	0.7844	0.7778	5.6667	0.6667
SE_B2a_2010	2010	SE	SE_REWET	0.4912	0.1723	15.5091	0.3170	0.3742	0.7706	0.6190	5.5000	0.6429
SE_B2b_2010	2010	SE	SE_REWET	0.5099	0.1761	15.2797	0.3102	0.3723	0.7661	0.6111	5.4286	0.6327
SE_B3_2010	2010	SE	SE_REWET	0.3921	0.1353	19.1728	0.3791	0.3164	0.6346	0.5000	5.3333	0.6190
SE_B3a_2010	2010	SE	SE_REWET	0.2385	0.0982	17.2364	0.3432	0.3500	0.7135	0.5714	5.5000	0.6429
SE_B3b_2010	2010	SE	SE_REWET	0.3439	0.1308	20.1364	0.3996	0.3227	0.6492	0.5238	5.8333	0.6905
SE_B4_2010	2010	SE	SE_REWET	0.3145	0.1240	15.0850	0.3074	0.3374	0.6839	0.6000	5.9000	0.7000
SE_B4a_2010	2010	SE	SE_REWET	0.4422	0.1565	14.4387	0.2977	0.3228	0.6496	0.5000	5.7273	0.6753
SE_B4b_2010	2010	SE	SE_REWET	0.2189	0.0916	20.2370	0.3971	0.3535	0.7218	0.5333	5.5000	0.6429
SE_B5_2010	2010	SE	SE_REWET	0.3421	0.1331	15.5149	0.3178	0.3617	0.7412	0.6190	5.7143	0.6735
SE_B5a_2010	2010	SE	SE_REWET	0.4188	0.1498	15.6506	0.3141	0.3533	0.7213	0.5833	5.6250	0.6607
SE_B5b_2010	2010	SE	SE_REWET	0.3036	0.1228	15.7213	0.3194	0.3423	0.6955	0.5000	5.7500	0.6786
SE_C1_2010	2010	SE	SE_REWET	0.6274	0.2153	17.1761	0.3532	0.3534	0.7216	0.6190	5.7500	0.6786
SE_C1a_2010	2010	SE	SE_REWET	0.6449	0.2157	16.9178	0.3505	0.3698	0.7602	0.6667	5.8000	0.6857
SE_C1b_2010	2010	SE	SE_REWET	0.3986	0.1516	19.9198	0.4019	0.3816	0.7880	0.6667	6.0000	0.7143
SE_C2_2010	2010	SE	SE_REWET	0.2836	0.1148	15.3224	0.3070	0.3714	0.7639	0.6667	5.7500	0.6786
SE_C2a_2010	2010	SE	SE_REWET	0.5616	0.1905	15.4081	0.3172	0.3809	0.7862	0.7333	5.6667	0.6667
SE_C2b_2010	2010	SE	SE_REWET	0.3741	0.1448	16.5208	0.3352	0.3714	0.7640	0.6667	6.1667	0.7381
SE_C3_2010	2010	SE	SE_REWET	0.3181	0.1224	17.8480	0.3565	0.3634	0.7451	0.5000	5.5714	0.6531
SE_C3a_2010	2010	SE	SE_REWET	0.2306	0.0957	16.2626	0.3238	0.3481	0.7091	0.5833	5.7143	0.6735
SE_C3b_2010	2010	SE	SE_REWET	0.2564	0.1057	14.6263	0.2945	0.3593	0.7354	0.5714	5.4286	0.6327
SE_C4_2010	2010	SE	SE_REWET	0.3518	0.1282	14.7440	0.2961	0.3624	0.7428	0.5667	5.2000	0.6000
SE_C4a_2010	2010	SE	SE_REWET	0.5089	0.1716	16.7580	0.3340	0.3704	0.7615	0.5833	5.2000	0.6000
SE_C4b_2010	2010	SE	SE_REWET	0.3772	0.1344	15.5918	0.3125	0.3477	0.7082	0.5833	5.5000	0.6429
SE_C5_2010	2010	SE	SE_REWET	0.2306	0.0957	16.2626	0.3238	0.3481	0.7091	0.5833	5.7143	0.6735
SE_C5a_2010	2010	SE	SE_REWET	0.2598	0.1070	15.8158	0.3187	0.3269	0.6592	0.5897	5.5833	0.6548
SE_C5b_2010	2010	SE	SE_REWET	0.3822	0.1365	15.4128	0.3069	0.3621	0.7421	0.6250	5.5000	0.6429
SE_D1_2010	2010	SE	SE_REWET	0.4921	0.1686	17.8899	0.3588	0.3652	0.7494	0.6111	5.5000	0.6429
SE_D1a_2010	2010	SE	SE_REWET	0.2189	0.0916	20.2370	0.3971	0.3535	0.7218	0.5333	5.5000	0.6429
SE_D1b_2010	2010	SE	SE_REWET	0.3499	0.1339	20.0468	0.4026	0.3760	0.7748	0.6667	6.2500	0.7500
SE_D2_2010	2010	SE	SE_REWET	0.3440	0.1350	16.0954	0.3259	0.3559	0.7274	0.6667	5.7500	0.6786
SE_D2a_2010	2010	SE	SE_REWET	0.3696	0.1444	15.7505	0.3203	0.3595	0.7358	0.6667	5.8333	0.6905
SE_D2b_2010	2010	SE	SE_REWET	0.2992	0.1176	17.5147	0.3475	0.3769	0.7769	0.6190	5.6667	0.6667
SE_D3_2010	2010	SE	SE_REWET	0.2612	0.1072	14.6886	0.2931	0.3711	0.7633	0.6250	5.4286	0.6327
SE_D3a_2010	2010	SE	SE_REWET	0.3200	0.1301	13.1018	0.2723	0.3714	0.7639	0.6296	5.6667	0.6667
SE_D3b_2010	2010	SE	SE_REWET	0.2758	0.1129	15.7973	0.3137	0.3781	0.7796	0.6111	5.6000	0.6571
SE_D4_2010	2010	SE	SE_REWET	0.2158	0.0908	18.1306	0.3546	0.3658	0.7508	0.5556	5.4000	0.6286
SE_D4a_2010	2010	SE	SE_REWET	0.4616	0.1590	15.5231	0.3112	0.3623	0.7426	0.5333	5.1667	0.5952
SE_D4b_2010	2010	SE	SE_REWET	0.2492	0.1030	17.2330	0.3414	0.3527	0.7199	0.5714	5.8333	0.6905
SE_D5_2010	2010	SE	SE_REWET	0.2158	0.0908	18.1306	0.3546	0.3658	0.7508	0.5556	5.4000	0.6286
SE_D5a_2010	2010	SE	SE_REWET	0.2744	0.1123	16.2923	0.3279	0.3541	0.7232	0.5556	5.7500	0.6786
SE_D5b_2010	2010	SE	SE_REWET	0.3381	0.1327	15.5754	0.3166	0.3820	0.7889	0.6250	5.7143	0.6735
SE_E1_2002	2002	SE	SE_BEFORE	0.2573	0.1054	21.7150	0.4307	0.3421	0.6950	0.5000	6.0000	0.7143
SE_E2_2002	2002	SE	SE_BEFORE	0.3654	0.1405	17.2848	0.3595	0.3607	0.7387	0.6000	6.0000	0.7143
SE_E3_2002	2002	SE	SE_BEFORE	0.2915	0.1188	20.1817	0.4087	0.3572	0.7306	0.5556	6.0000	0.7143
SE_E4_2002	2002	SE	SE_BEFORE	0.4186	0.1588	18.5910	0.3827	0.3760	0.7748	0.6667	6.2500	0.7500
SE_E5_2002	2002	SE	SE_BEFORE	0.5608	0.1856	17.8300	0.3575	0.3373	0.6836	0.4667	5.6000	0.6571
SE_F1_2002	2002	SE	SE_BEFORE	0.2573	0.1054	21.7150	0.4307	0.3421	0.6950	0.5000	6.0000	0.7143

SE_F2_2002	2002	SE	SE_BEFORE	0.2573	0.1054	21.7150	0.4307	0.3421	0.6950	0.5000	6.0000	0.7143
SE_F3_2002	2002	SE	SE_BEFORE	0.2725	0.1118	13.6447	0.2757	0.4081	0.8504	0.8333	6.0000	0.7143
SE_F4_2002	2002	SE	SE_BEFORE	0.5786	0.2045	18.8123	0.3862	0.3722	0.7658	0.5833	6.5000	0.7857
SE_F5_2002	2002	SE	SE_BEFORE	0.3798	0.1470	21.2633	0.4251	0.3545	0.7242	0.5556	6.0000	0.7143
SE_G1_2002	2002	SE	SE_BEFORE	0.4169	0.1567	17.0937	0.3462	0.3864	0.7993	0.7333	6.0000	0.7143
SE_G2_2002	2002	SE	SE_BEFORE	0.1919	0.0813	21.2937	0.3627	0.3377	0.6846	0.7333	5.4000	0.6286
SE_G3_2002	2002	SE	SE_BEFORE	0.2573	0.1054	21.7150	0.4307	0.3421	0.6950	0.5000	6.0000	0.7143
SE_G4_2002	2002	SE	SE_BEFORE	0.3674	0.1365	17.8585	0.3545	0.3550	0.7254	0.6250	5.6250	0.6607
SE_G5_2002	2002	SE	SE_BEFORE	0.4378	0.1604	19.8456	0.3923	0.3454	0.7027	0.5714	6.0000	0.7143
SE_H1_2002	2002	SE	SE_BEFORE	0.4562	0.1688	19.6017	0.3980	0.3688	0.7579	0.7222	6.0000	0.7143
SE_H2_2002	2002	SE	SE_BEFORE	0.3688	0.1399	26.3195	0.4577	0.3209	0.6451	0.9167	6.0000	0.7143
SE_H3_2002	2002	SE	SE_BEFORE	0.2341	0.0967	16.7399	0.3331	0.3334	0.6744	0.6389	5.5455	0.6494
SE_H4_2002	2002	SE	SE_BEFORE	0.4693	0.1644	17.7184	0.3550	0.3579	0.7322	0.6000	5.7000	0.6714
SE_H5_2002	2002	SE	SE_BEFORE	0.2474	0.1011	20.6875	0.4102	0.3617	0.7412	0.6667	5.6667	0.6667
SE_I1_2010	2010	SE	SE_REWET	0.3700	0.1491	20.0031	0.4087	0.2926	0.5785	0.3333	6.0000	0.7143
SE_I2_2010	2010	SE	SE_REWET	0.3700	0.1491	20.0031	0.4087	0.2926	0.5785	0.3333	6.0000	0.7143
SE_I3_2010	2010	SE	SE_REWET	0.2725	0.1118	13.6447	0.2757	0.4081	0.8504	0.8333	6.0000	0.7143
SE_I4_2010	2010	SE	SE_REWET	0.4713	0.1760	14.4387	0.3023	0.3304	0.6676	0.6667	5.5000	0.6429
SE_I5_2010	2010	SE	SE_REWET	0.2150	0.0896	24.9565	0.4036	0.3231	0.6503	0.8889	5.6667	0.6667
SE_J1_2010	2010	SE	SE_REWET	0.4600	0.1661	16.0145	0.3434	0.3845	0.7949	1.0000	6.5000	0.7857
SE_J2_2010	2010	SE	SE_REWET	0.6000	0.2190	16.6245	0.3526	0.4063	0.8460	0.8333	7.0000	0.8571
SE_J3_2010	2010	SE	SE_REWET	0.3400	0.1387	20.5761	0.4168	0.2050	0.3723	0.0000	5.0000	0.5714
SE_J4_2010	2010	SE	SE_REWET	0.2198	0.0917	17.0965	0.3374	0.3734	0.7687	0.6667	5.6667	0.6667
SE_J5_2010	2010	SE	SE_REWET	0.4000	0.1594	19.4300	0.4005	0.3802	0.7847	0.6667	7.0000	0.8571
SE_K2_2010	2010	SE	SE_REWET	0.4000	0.1594	19.4300	0.4005	0.3802	0.7847	0.6667	7.0000	0.8571
SE_K3_2010	2010	SE	SE_REWET	0.2239	0.0932	18.0489	0.3622	0.3712	0.7634	0.7333	5.6000	0.6571
SE_K4_2010	2010	SE	SE_REWET	0.1450	0.0642	7.8595	0.1510	0.4361	0.9162	1.0000	5.0000	0.5714
SE_K5_2010	2010	SE	SE_REWET	0.2725	0.1118	13.6447	0.2757	0.4081	0.8504	0.8333	6.0000	0.7143
SE_L1_2010	2010	SE	SE_REWET	0.5750	0.2029	17.1110	0.3625	0.3534	0.7216	0.6667	6.7500	0.8214
SE_L2_2010	2010	SE	SE_REWET	0.2713	0.1103	17.3050	0.3544	0.3412	0.6929	0.7500	6.2500	0.7500
SE_L3_2010	2010	SE	SE_REWET	0.3800	0.1526	18.2725	0.3826	0.3839	0.7933	0.6667	6.5000	0.7857
SE_L4_2010	2010	SE	SE_REWET	0.2573	0.1054	21.7150	0.4307	0.3421	0.6950	0.5000	6.0000	0.7143
SE_L5_2010	2010	SE	SE_REWET	0.4000	0.1594	19.4300	0.4005	0.3802	0.7847	0.6667	7.0000	0.8571
SE_M1_2002	2002	SE	SE_BEFORE	0.1483	0.0655	18.3529	0.3542	0.3807	0.7858	1.0000	5.0000	0.5714
SE_M2_2002	2002	SE	SE_BEFORE	0.2725	0.1118	13.6447	0.2757	0.4081	0.8504	0.8333	6.0000	0.7143
SE_M3_2002	2002	SE	SE_BEFORE	0.1750	0.0764	24.4491	0.4662	0.3180	0.6383	1.0000	5.0000	0.5714
SE_M4_2002	2002	SE	SE_BEFORE	0.1450	0.0642	7.8595	0.1510	0.4361	0.9162	1.0000	5.0000	0.5714
SE_M5_2002	2002	SE	SE_BEFORE	0.4483	0.1674	13.7028	0.2854	0.4162	0.8694	0.8889	6.3333	0.7619
SE_N1_2002	2002	SE	SE_BEFORE	0.1450	0.0642	7.8595	0.1510	0.4361	0.9162	1.0000	5.0000	0.5714
SE_N2_2002	2002	SE	SE_BEFORE	0.8000	0.2785	13.8191	0.3046	0.4324	0.9074	1.0000	7.0000	0.8571
SE_N3_2002	2002	SE	SE_BEFORE	0.4700	0.1730	32.8410	0.5169	0.3085	0.6159	0.8889	6.0000	0.7143
SE_N4_2002	2002	SE	SE_BEFORE	0.8000	0.2785	13.8191	0.3046	0.4324	0.9074	1.0000	7.0000	0.8571
SE_N5_2002	2002	SE	SE_BEFORE	0.3030	0.1220	25.8217	0.4325	0.3394	0.6886	0.8000	5.7500	0.6786
SE_O1_2002	2002	SE	SE_BEFORE	0.7466	0.2245	19.5936	0.3811	0.3800	0.7842	0.7083	5.7500	0.6786
SE_O2_2002	2002	SE	SE_BEFORE	0.4750	0.1804	19.4247	0.3947	0.3351	0.6786	0.7500	6.2500	0.7500
SE_O3_2002	2002	SE	SE_BEFORE	0.3500	0.1419	22.1875	0.4362	0.3356	0.6797	0.6667	6.5000	0.7857
SE_O4_2002	2002	SE	SE_BEFORE	0.6000	0.2190	16.6245	0.3526	0.4063	0.8460	0.8333	7.0000	0.8571
SE_O5_2002	2002	SE	SE_BEFORE	0.5700	0.2111	23.4577	0.4307	0.3842	0.7940	0.7778	7.0000	0.8571
SE_P1_2002	2002	SE	SE_BEFORE	0.4850	0.1793	19.6008	0.3475	0.4028	0.8379	0.8889	6.0000	0.7143
SE_P2_2002	2002	SE	SE_BEFORE	0.2565	0.1036	22.9945	0.3996	0.3600	0.7371	0.6667	5.0000	0.5714
SE_P4_2002	2002	SE	SE_BEFORE									
SE_P5_2002	2002	SE	SE_BEFORE	0.3870	0.1494	22.0998	0.3836	0.3072	0.6129	0.7619	5.8333	0.6905
ST_A1_2010	2010	ST	ST_REWET	0.4717	0.1702	24.7224	0.3849	0.3323	0.6720	0.8667	5.8000	0.6857
ST_A1a_2010	2010	ST	ST_REWET	0.5900	0.2134	18.5698	0.3688	0.3177	0.6374	0.8000	5.6000	0.6571
ST_A1b_2010	2010	ST	ST_REWET	1.5088	0.3368	19.0668	0.3700	0.2962	0.5869	0.7222	5.2857	0.6122
ST_A2_2010	2010	ST	ST_REWET	0.5194	0.1891	19.6532	0.3800	0.3228	0.6496	0.8148	5.6667	0.6667
ST_A2a_2010	2010	ST	ST_REWET	1.2228	0.2668	21.2372	0.4048	0.3060	0.6099	0.7917	5.5556	0.6508
ST_A2b_2010	2010	ST	ST_REWET	0.4442	0.1683	21.7958	0.3910	0.2985	0.5923	0.7222	5.5000	0.6429
ST_A3_2010	2010	ST	ST_REWET	0.5400	0.1909	9.7236	0.2010	0.3715	0.7642	1.0000	5.2500	0.6071
ST_A3a_2010	2010	ST	ST_REWET	0.6717	0.2331	10.3450	0.2176	0.3500	0.7136	1.0000	5.3333	0.6190
ST_A3b_2010	2010	ST	ST_REWET	0.5150	0.1885	12.3928	0.2641	0.3723	0.7660	0.8889	5.6667	0.6667
ST_A4_2010	2010	ST	ST_REWET	0.5150	0.1885	12.3928	0.2641	0.3723	0.7660	0.8889	5.6667	0.6667
ST_A4a_2010	2010	ST	ST_REWET	0.4850	0.1755	23.6616	0.4206	0.2725	0.5312	0.7778	6.0000	0.7143
ST_A4b_2010	2010	ST	ST_REWET	0.1975	0.0850	13.2567	0.2700	0.4390	0.9232	1.0000	5.0000	0.5714
ST_A5_2010	2010	ST	ST_REWET	0.7750	0.2691	10.9798	0.2383	0.3633	0.7448	0.8333	6.0000	0.7143
ST_A5a_2010	2010	ST	ST_REWET	0.7025	0.2365	18.0407	0.3421	0.3208	0.6448	0.7333	5.3333	0.6190
ST_A5b_2010	2010	ST	ST_REWET	0.7000	0.2493	11.5064	0.2514	0.3924	0.8135	0.7778	6.3333	0.7619
ST_B1_2010	2010	ST	ST_REWET	0.5942	0.2100	22.0357	0.3992	0.2864	0.5638	0.7333	5.6000	0.6571
ST_B1a_2010	2010	ST	ST_REWET	1.1795	0.2661	21.4309	0.4018	0.2919	0.5768	0.7083	4.8889	0.5556
ST_B1b_2010	2010	ST	ST_REWET	0.8348	0.2737	18.2740	0.3419	0.2651	0.5138	0.7500	5.7500	0.6786
ST_B2_2010	2010	ST	ST_REWET	0.5775	0.1965	13.1008	0.2707	0.3563	0.7283	0.8000	5.1667	0.5952
ST_B2a_2010	2010	ST	ST_REWET	0.5588	0.1925	17.5524	0.3415	0.3455	0.7029	0.7143	5.2500	0.6071
ST_B2b_2010	2010	ST	ST_REWET	0.8125	0.2818	12.2595	0.2693	0.4060	0.8454	0.8333	6.5000	0.7857
ST_B3_2010	2010	ST	ST_REWET	0.5888	0.2142	18.4873	0.3687	0.3614	0.7405	0.7500	5.7500	0.6786
ST_B3a_2010	2010	ST	ST_REWET	0.5500	0.2047	19.5014	0.3911	0.3405	0.6913	0.7778	5.6667	0.6667
ST_B3b_2010	2010	ST	ST_REWET	0.6917	0.2466	17.1730	0.3444	0.3453	0.7025	0.7778	6.0000	0.7143
ST_B4_2010	2010	ST	ST_REWET	0.5900	0.2093	10.7928	0.2299	0.4160	0.8690	0.8889	6.0000	0.7143

ST_B4a_2010	2010	ST	ST_REWET	0.8125	0.2818	12.2595	0.2693	0.4060	0.8454	0.8333	6.5000	0.7857
ST_B4b_2010	2010	ST	ST_REWET	0.8000	0.2785	13.8191	0.3046	0.4324	0.9074	1.0000	7.0000	0.8571
ST_B5_2010	2010	ST	ST_REWET	0.5090	0.1887	14.7000	0.3011	0.3120	0.6241	0.6667	5.6667	0.6667
ST_B5a_2010	2010	ST	ST_REWET	0.7000	0.2493	11.5064	0.2514	0.3924	0.8135	0.7778	6.3333	0.7619
ST_B5b_2010	2010	ST	ST_REWET	0.8250	0.2851	10.7000	0.2340	0.3797	0.7835	0.6667	6.0000	0.7143
ST_C1_2010	2010	ST	ST_REWET	0.5740	0.2049	21.4850	0.3873	0.2785	0.5452	0.7407	5.4444	0.6349
ST_C1a_2010	2010	ST	ST_REWET	0.4333	0.1605	23.7327	0.4138	0.2863	0.5636	0.8182	5.1818	0.5974
ST_C1b_2010	2010	ST	ST_REWET	0.4901	0.1820	23.2882	0.4131	0.2718	0.5295	0.7576	5.3636	0.6234
ST_C2_2010	2010	ST	ST_REWET	0.6240	0.2107	23.3118	0.4113	0.3044	0.6063	1.0000	5.5000	0.6429
ST_C2a_2010	2010	ST	ST_REWET	0.5800	0.2010	21.1310	0.4176	0.3461	0.7043	0.8889	6.0000	0.7143
ST_C2b_2010	2010	ST	ST_REWET	0.4475	0.1588	27.1082	0.4723	0.2815	0.5524	0.8667	5.4000	0.6286
ST_C3_2010	2010	ST	ST_REWET	0.4927	0.1800	23.7149	0.4268	0.3001	0.5962	0.7407	5.4444	0.6349
ST_C3a_2010	2010	ST	ST_REWET	0.4150	0.1609	17.6397	0.3492	0.3575	0.7313	0.7143	5.5714	0.6531
ST_C3b_2010	2010	ST	ST_REWET	0.4988	0.1811	24.7049	0.4401	0.2810	0.5511	0.7083	5.6250	0.6607
ST_C4_2010	2010	ST	ST_REWET	0.4832	0.1711	21.7189	0.3958	0.3142	0.6293	0.8148	5.1111	0.5873
ST_C4a_2010	2010	ST	ST_REWET	0.4221	0.1550	22.6771	0.4090	0.3074	0.6133	0.8333	5.2000	0.6000
ST_C4b_2010	2010	ST	ST_REWET	0.5200	0.1847	24.6620	0.4364	0.3189	0.6403	0.7778	5.3333	0.6190
ST_C5_2010	2010	ST	ST_REWET	0.5107	0.1812	22.5743	0.3947	0.3092	0.6175	0.8333	5.1667	0.5952
ST_C5a_2010	2010	ST	ST_REWET	0.5057	0.1818	24.3770	0.4323	0.2891	0.5704	0.7143	5.7143	0.6735
ST_C5b_2010	2010	ST	ST_REWET	0.4975	0.1766	22.4215	0.4069	0.3027	0.6022	0.7778	5.4444	0.6349
ST_D1_2010	2010	ST	ST_REWET	0.8001	0.2438	21.7909	0.4138	0.2825	0.5548	0.6667	5.6000	0.6571
ST_D1a_2010	2010	ST	ST_REWET	0.5483	0.1951	29.1936	0.4977	0.2492	0.4763	0.7333	6.0000	0.7143
ST_D1b_2010	2010	ST	ST_REWET	0.5479	0.1952	25.3899	0.4561	0.2556	0.4914	0.6923	5.7692	0.6813
ST_D2_2010	2010	ST	ST_REWET	0.6019	0.2117	23.5363	0.4367	0.2814	0.5521	0.6667	5.8000	0.6857
ST_D2a_2010	2010	ST	ST_REWET	0.5710	0.2014	26.6824	0.4752	0.2711	0.5279	0.7037	5.7778	0.6825
ST_D2b_2010	2010	ST	ST_REWET	0.6068	0.2157	23.1345	0.4321	0.2835	0.5571	0.6667	6.0000	0.7143
ST_D3_2010	2010	ST	ST_REWET	0.5140	0.1824	25.7814	0.4560	0.3006	0.5974	0.7407	5.5556	0.6508
ST_D3a_2010	2010	ST	ST_REWET	0.5681	0.2023	23.8369	0.4436	0.2686	0.5221	0.6111	5.7500	0.6786
ST_D3b_2010	2010	ST	ST_REWET	0.5205	0.1852	23.6712	0.4199	0.2922	0.5776	0.7778	5.3333	0.6190
ST_D4_2010	2010	ST	ST_REWET	0.4523	0.1673	23.4249	0.4213	0.3023	0.6013	0.7273	5.6364	0.6623
ST_D4a_2010	2010	ST	ST_REWET	0.4683	0.1705	24.6071	0.4305	0.2935	0.5807	0.7407	5.6667	0.6667
ST_D4b_2010	2010	ST	ST_REWET	0.5918	0.2092	26.1458	0.4514	0.2730	0.5323	0.7000	5.9000	0.7000
ST_D5_2010	2010	ST	ST_REWET	0.7100	0.2478	21.4377	0.4082	0.2937	0.5811	0.6667	6.1667	0.7381
ST_D5a_2010	2010	ST	ST_REWET	0.4492	0.1679	24.3207	0.4373	0.3016	0.5997	0.7222	5.8333	0.6905
ST_D5b_2010	2010	ST	ST_REWET	0.7350	0.2561	19.3627	0.3885	0.2982	0.5916	0.6111	6.0000	0.7143
ST_E1_2002	2002	ST	ST_BEFORE	0.3800	0.1408	22.0320	0.3476	0.2916	0.5761	0.8889	5.0000	0.5714
ST_E2_2002	2002	ST	ST_BEFORE	0.3119	0.1211	18.5950	0.3656	0.3705	0.7619	0.8000	5.6000	0.6571
ST_E3_2002	2002	ST	ST_BEFORE	0.3650	0.1336	21.3398	0.3282	0.3167	0.6353	0.8889	5.3333	0.6190
ST_E4_2002	2002	ST	ST_BEFORE	0.4019	0.1462	20.3677	0.3500	0.3373	0.6837	0.8000	5.6000	0.6571
ST_E5_2002	2002	ST	ST_BEFORE	0.1000	0.0452	21.8400	0.4339	0.3481	0.7091	1.0000	5.0000	0.5714
ST_F1_2002	2002	ST	ST_BEFORE	0.1436	0.0632	21.8138	0.3551	0.3088	0.6166	0.8333	4.7500	0.5357
ST_F2_2002	2002	ST	ST_BEFORE	0.2571	0.1014	20.2170	0.3593	0.3459	0.7039	0.8571	5.2857	0.6122
ST_F3_2002	2002	ST	ST_BEFORE	0.3558	0.1318	21.4781	0.3802	0.3449	0.7016	0.8333	5.8333	0.6905
ST_F4_2002	2002	ST	ST_BEFORE	0.3100	0.1242	8.9297	0.1833	0.4007	0.8328	0.8333	5.5000	0.6429
ST_F5_2002	2002	ST	ST_BEFORE									
ST_G1_2002	2002	ST	ST_BEFORE	0.3538	0.1357	16.2997	0.3286	0.3952	0.8199	1.0000	5.7500	0.6786
ST_G2_2002	2002	ST	ST_BEFORE	0.1475	0.0652	16.3847	0.3112	0.4002	0.8318	1.0000	6.0000	0.7143
ST_G3_2002	2002	ST	ST_BEFORE	0.5183	0.1751	13.8806	0.2812	0.3936	0.8163	1.0000	5.3333	0.6190
ST_G4_2002	2002	ST	ST_BEFORE	0.4750	0.1724	19.3645	0.3881	0.3984	0.8274	1.0000	7.0000	0.8571
ST_G5_2002	2002	ST	ST_BEFORE	0.5917	0.2100	16.4764	0.3367	0.3921	0.8127	0.8889	6.6667	0.8095
ST_H1_2002	2002	ST	ST_BEFORE	0.1500	0.0662	24.9100	0.4715	0.3644	0.7474	1.0000	7.0000	0.8571
ST_H2_2002	2002	ST	ST_BEFORE	0.1500	0.0662	24.9100	0.4715	0.3644	0.7474	1.0000	7.0000	0.8571
ST_H3_2002	2002	ST	ST_BEFORE	0.4725	0.1714	10.8393	0.2278	0.4342	0.9118	1.0000	6.0000	0.7143
ST_H4_2002	2002	ST	ST_BEFORE	0.2150	0.0923	7.8159	0.1495	0.3420	0.6947	1.0000	4.0000	0.4286
ST_H5_2002	2002	ST	ST_BEFORE	0.1500	0.0662	24.9100	0.4715	0.3644	0.7474	1.0000	7.0000	0.8571
ST_I1_2010	2010	ST	ST_REWET	0.5167	0.1892	18.0764	0.3710	0.3484	0.7097	0.8889	6.3333	0.7619
ST_I2_2010	2010	ST	ST_REWET	0.5627	0.1916	14.7807	0.2991	0.3414	0.6933	0.8571	5.7143	0.6735
ST_I3_2010	2010	ST	ST_REWET	0.6200	0.2072	14.3612	0.2953	0.3999	0.8311	0.9333	5.6667	0.6667
ST_I4_2010	2010	ST	ST_REWET	0.1833	0.0786	32.4783	0.5342	0.2695	0.5241	0.8889	6.0000	0.7143
ST_I5_2010	2010	ST	ST_REWET									
ST_J1_2010	2010	ST	ST_REWET	0.6938	0.2479	18.2150	0.3348	0.2973	0.5896	0.6667	5.5000	0.6429
ST_J2_2010	2010	ST	ST_REWET	0.5640	0.2004	25.5110	0.4349	0.2594	0.5003	0.7407	5.5556	0.6508
ST_J3_2010	2010	ST	ST_REWET	0.4863	0.1747	23.7266	0.4243	0.3168	0.6355	0.7917	5.8750	0.6964
ST_J4_2010	2010	ST	ST_REWET	0.4501	0.1672	25.9627	0.4484	0.2666	0.5173	0.7619	5.4286	0.6327
ST_J5_2010	2010	ST	ST_REWET	0.1075	0.0484	42.0400	0.6206	0.1659	0.2802	0.8333	4.5000	0.5000
ST_K1_2010	2010	ST	ST_REWET	0.4834	0.1761	20.4772	0.3710	0.2948	0.5837	0.8519	5.3333	0.6190
ST_K2_2010	2010	ST	ST_REWET	0.3330	0.1272	23.8714	0.4213	0.2932	0.5799	0.7333	5.5000	0.6429
ST_K3_2010	2010	ST	ST_REWET	0.4105	0.1414	23.9134	0.4140	0.3005	0.5970	0.7778	5.3000	0.6143
ST_K4_2010	2010	ST	ST_REWET	0.3163	0.1214	23.6059	0.4132	0.3097	0.6187	0.7576	5.6364	0.6623
ST_K5_2010	2010	ST	ST_REWET	0.6667	0.2240	12.8755	0.2649	0.3836	0.7927	0.8000	5.8333	0.6905
ST_L1_2010	2010	ST	ST_REWET	0.4615	0.1702	25.4603	0.4416	0.2797	0.5482	0.7879	5.3636	0.6234
ST_L2_2010	2010	ST	ST_REWET	0.4171	0.1589	20.6944	0.3627	0.3184	0.6391	0.8333	5.4000	0.6286
ST_L3_2010	2010	ST	ST_REWET	0.5900	0.2093	10.7928	0.2299	0.4160	0.8690	0.8889	6.0000	0.7143
ST_L4_2010	2010	ST	ST_REWET	0.4675	0.1682	13.5546	0.2809	0.3991	0.8290	0.9167	5.7500	0.6786
ST_L5_2010	2010	ST	ST_REWET	0.6190	0.2044	12.1249	0.2512	0.3748	0.7720	0.8333	5.2000	0.6000
ST_M1_2002	2002	ST	ST_BEFORE	0.3583	0.1322	19.1500	0.3798	0.3641	0.7467	0.8889	6.0000	0.7143
ST_M2_2002	2002	ST	ST_BEFORE	0.3317	0.1269	17.1398	0.3174	0.3635	0.7453	0.9259	5.4444	0.6349

ST_M3_2002	2002	ST	ST_BEFORE	0.4220	0.1381	18.1925	0.3104	0.3217	0.6469	1.0000	4.8000	0.5429
ST_M4_2002	2002	ST	ST_BEFORE	0.5088	0.1640	9.4251	0.1957	0.3713	0.7636	0.8889	4.2500	0.4643
ST_M5_2002	2002	ST	ST_BEFORE	0.1450	0.0642	7.8595	0.1510	0.4361	0.9162	1.0000	5.0000	0.5714
ST_N1_2002	2002	ST	ST_BEFORE	0.1817	0.0787	17.1411	0.3371	0.4142	0.8646	1.0000	5.6667	0.6667
ST_N2_2002	2002	ST	ST_BEFORE	0.2863	0.1147	16.7309	0.3370	0.3727	0.7670	0.9167	5.5000	0.6429
ST_N3_2002	2002	ST	ST_BEFORE	0.5413	0.1770	15.4012	0.3079	0.3733	0.7685	1.0000	5.0000	0.5714
ST_N4_2002	2002	ST	ST_BEFORE	0.2341	0.0991	18.5530	0.3759	0.3441	0.6996	0.6667	5.3333	0.6190
ST_N5_2002	2002	ST	ST_BEFORE									
ST_O1_2002	2002	ST	ST_BEFORE									
ST_O2_2002	2002	ST	ST_BEFORE	0.3425	0.1303	15.5309	0.3114	0.4055	0.8443	0.9167	5.7500	0.6786
ST_O3_2002	2002	ST	ST_BEFORE	0.3650	0.1363	15.5295	0.3090	0.4109	0.8570	1.0000	6.3333	0.7619
ST_O4_2002	2002	ST	ST_BEFORE	0.1975	0.0850	13.2567	0.2700	0.4390	0.9232	1.0000	5.0000	0.5714
ST_O5_2002	2002	ST	ST_BEFORE	0.1225	0.0547	27.7197	0.4051	0.2946	0.5832	1.0000	5.0000	0.5714
ST_P1_2002	2002	ST	ST_BEFORE	0.2000	0.0860	21.7820	0.4302	0.4032	0.8388	1.0000	6.0000	0.7143
ST_P2_2002	2002	ST	ST_BEFORE	0.2000	0.0860	21.7820	0.4302	0.4032	0.8388	1.0000	6.0000	0.7143
ST_P3_2002	2002	ST	ST_BEFORE	0.5790	0.1917	15.0935	0.3075	0.4040	0.8406	1.0000	5.6000	0.6571
ST_P4_2002	2002	ST	ST_BEFORE	0.4481	0.1549	19.6309	0.3592	0.3553	0.7260	0.8571	5.5000	0.6429
ST_P5_2002	2002	ST	ST_BEFORE									
TA_279	1972	TA	TA_BEFORE	0.4603	0.1703	22.8280	0.4322	0.2092	0.3823	0.6282	5.2222	0.6032
TA_280	1972	TA	TA_BEFORE	0.4780	0.1762	23.4477	0.4385	0.2087	0.3810	0.6173	5.1481	0.5926
TA_281	1972	TA	TA_BEFORE	0.4951	0.1815	23.9758	0.4458	0.2098	0.3837	0.6296	5.3214	0.6173
TA_282	1972	TA	TA_BEFORE	0.4941	0.1809	24.6497	0.4546	0.2069	0.3768	0.6465	5.0588	0.5798
TA_283	1972	TA	TA_BEFORE	0.5348	0.1923	24.2993	0.4500	0.2073	0.3778	0.6806	5.0417	0.5774
TA_284	1972	TA	TA_BEFORE	0.5288	0.1903	24.2789	0.4491	0.2021	0.3656	0.7361	5.0000	0.5714
TA_285	1972	TA	TA_BEFORE	0.5460	0.1965	23.9969	0.4466	0.2140	0.3934	0.6400	5.2000	0.6000
TA_286	1972	TA	TA_BEFORE	0.5195	0.1877	22.4114	0.4268	0.2145	0.3946	0.6667	5.2000	0.6000
TA_287	1972	TA	TA_BEFORE	0.4785	0.1788	21.2826	0.4110	0.2352	0.4434	0.7302	5.2857	0.6122
TA_288	1972	TA	TA_BEFORE	0.4845	0.1797	23.4149	0.4364	0.2166	0.3997	0.6667	5.2593	0.6085
TA_289	1972	TA	TA_BEFORE	0.4735	0.1758	22.8606	0.4289	0.2172	0.4009	0.7024	5.1852	0.5979
TA_290	1972	TA	TA_BEFORE	0.5352	0.1929	23.5264	0.4376	0.2204	0.4086	0.6667	5.2414	0.6059
TA_291	1972	TA	TA_BEFORE	0.4704	0.1755	23.6772	0.4406	0.2164	0.3991	0.6552	5.2143	0.6020
TA_292	1972	TA	TA_BEFORE	0.4457	0.1684	23.3194	0.4354	0.2324	0.4368	0.6465	5.1563	0.5938
TA_293	1972	TA	TA_BEFORE	0.5013	0.1846	23.6987	0.4403	0.2254	0.4202	0.6022	5.3000	0.6143
TA_294	1972	TA	TA_BEFORE	0.5389	0.1936	26.3307	0.4776	0.2047	0.3717	0.5873	4.9524	0.5646
TA_295	1972	TA	TA_BEFORE	0.6139	0.2162	23.7999	0.4422	0.2179	0.4026	0.6790	5.1852	0.5979
TA_296	1972	TA	TA_BEFORE	0.5506	0.1962	25.1634	0.4658	0.2070	0.3771	0.6000	5.0400	0.5771
TA_297	1972	TA	TA_BEFORE	0.4670	0.1727	27.1956	0.4889	0.1986	0.3572	0.5897	4.8077	0.5440
TA_298	1972	TA	TA_BEFORE	0.5659	0.2025	24.4469	0.4545	0.2101	0.3843	0.7018	5.0526	0.5789
TA_299	1972	TA	TA_BEFORE	0.5427	0.1944	23.3093	0.4419	0.2139	0.3932	0.6812	5.0870	0.5839
TA_300	1972	TA	TA_BEFORE	0.5979	0.2101	24.0032	0.4504	0.2186	0.4043	0.6667	5.3636	0.6234
TA_301	1972	TA	TA_BEFORE	0.6060	0.2126	23.5757	0.4431	0.2227	0.4139	0.6154	5.3200	0.6171
TA_302	1972	TA	TA_BEFORE	0.5067	0.1812	25.1015	0.4655	0.2120	0.3888	0.6173	5.0000	0.5714
TA_303	1972	TA	TA_BEFORE	0.4536	0.1697	24.7351	0.4558	0.2095	0.3830	0.6667	5.0455	0.5779
TA_304	1972	TA	TA_BEFORE	0.4642	0.1736	24.6560	0.4550	0.2129	0.3908	0.6377	5.0455	0.5779
TA_305	1972	TA	TA_BEFORE	0.5223	0.1872	24.1489	0.4477	0.2100	0.3841	0.6812	5.1364	0.5909
TA_306	1972	TA	TA_BEFORE	0.5006	0.1825	23.8643	0.4467	0.2277	0.4258	0.6667	5.1538	0.5934
TA_307	1972	TA	TA_BEFORE	0.4644	0.1726	24.8794	0.4574	0.2139	0.3934	0.6667	5.0769	0.5824
TA_308	1972	TA	TA_BEFORE	0.4170	0.1586	25.3486	0.4637	0.2067	0.3762	0.6190	4.9630	0.5661
TA_937	2009	TA	TA_REWET	0.5587	0.2035	21.5626	0.4094	0.2398	0.4542	0.6190	5.6364	0.6623
TA_938	2009	TA	TA_REWET	0.5429	0.1985	21.6889	0.4113	0.2342	0.4411	0.6970	5.3182	0.6169
TA_939	2009	TA	TA_REWET	0.5787	0.2087	21.7562	0.4132	0.2324	0.4369	0.7143	5.3810	0.6259
TA_940	2009	TA	TA_REWET	0.4927	0.1826	22.6492	0.4312	0.2334	0.4391	0.6818	5.3478	0.6211
TA_941	2009	TA	TA_REWET	0.5148	0.1898	23.1610	0.4390	0.2231	0.4148	0.6508	5.3333	0.6190
TA_942	2009	TA	TA_REWET	0.4813	0.1793	22.5280	0.4238	0.2363	0.4461	0.6667	5.2500	0.6071
TA_943	2009	TA	TA_REWET	0.4958	0.1847	22.8817	0.4280	0.2256	0.4208	0.6552	5.3793	0.6256
TA_944	2009	TA	TA_REWET	0.4607	0.1741	22.6403	0.4263	0.2375	0.4489	0.6528	5.2917	0.6131
TA_945	2009	TA	TA_REWET	0.5130	0.1894	23.3647	0.4370	0.2227	0.4141	0.6452	5.2581	0.6083
TA_946	2009	TA	TA_REWET	0.5316	0.1955	24.2096	0.4445	0.2087	0.3811	0.6667	5.5385	0.6484
TA_947	2009	TA	TA_REWET	0.5161	0.1917	24.1159	0.4424	0.2418	0.4590	0.7284	5.3333	0.6190
TA_948	2009	TA	TA_REWET	0.5342	0.1973	23.0786	0.4198	0.2376	0.4490	0.7222	5.4000	0.6286
TA_949	2009	TA	TA_REWET	0.5127	0.1908	23.3969	0.4303	0.2273	0.4247	0.6882	5.4839	0.6406
TA_950	2009	TA	TA_REWET	0.5320	0.1961	23.6901	0.4348	0.2320	0.4358	0.7255	5.3529	0.6218
TA_951	2009	TA	TA_REWET	0.5146	0.1896	23.8251	0.4430	0.2411	0.4573	0.7160	5.2857	0.6122
TA_952	2009	TA	TA_REWET	0.5616	0.2007	23.3526	0.4289	0.2396	0.4537	0.7284	5.4643	0.6378
TA_953	2009	TA	TA_REWET	0.5206	0.1925	21.7454	0.4076	0.2516	0.4821	0.7460	5.3333	0.6190
TA_954	2009	TA	TA_REWET	0.4829	0.1808	22.6663	0.4234	0.2287	0.4281	0.7179	5.2692	0.6099
TA_955	2009	TA	TA_REWET	0.5443	0.1999	21.9058	0.4102	0.2266	0.4231	0.7361	5.4167	0.6310
TA_956	2009	TA	TA_REWET	0.4787	0.1792	23.8737	0.4430	0.2234	0.4155	0.7011	5.2759	0.6108
TA_957	2009	TA	TA_REWET	0.5092	0.1890	22.7734	0.4184	0.2221	0.4125	0.6979	5.3438	0.6205
TA_958	2009	TA	TA_REWET	0.5018	0.1864	22.0376	0.4140	0.2444	0.4650	0.7143	5.3214	0.6173
TA_959	2009	TA	TA_REWET	0.4762	0.1786	22.4001	0.4138	0.2361	0.4456	0.6989	5.2258	0.6037
TA_960	2009	TA	TA_REWET	0.4911	0.1826	22.5928	0.4216	0.2299	0.4309	0.6452	5.5161	0.6452
TA_961	2009	TA	TA_REWET	0.4545	0.1723	23.7011	0.4341	0.2169	0.4004	0.7083	5.3125	0.6161
TA_962	2009	TA	TA_REWET	0.5133	0.1892	22.5994	0.4273	0.2515	0.4816	0.6667	5.5455	0.6494
TA_963	2009	TA	TA_REWET	0.5231	0.1931	21.6379	0.4128	0.2460	0.4688	0.6667	5.5714	0.6531
TA_964	2009	TA	TA_REWET	0.5175	0.1913	22.5998	0.4285	0.2389	0.4522	0.6212	5.5000	0.6429
TA_965	2009	TA	TA_REWET	0.5211	0.1928	21.9833	0.4112	0.2339	0.4404	0.6543	5.4074	0.6296

TA_966	2009	TA	TA_REWET	0.4992	0.1861	23.8787	0.4320	0.2309	0.4333	0.6444	5.4667	0.6381
TR_1_1998	1998	TR	TR_BEFORE	0.3306	0.1320	26.2768	0.4845	0.2174	0.4015	0.8148	5.1111	0.5873
TR_10_1998	1998	TR	TR_BEFORE	0.3443	0.1364	26.9914	0.4921	0.2088	0.3812	0.8148	5.0000	0.5714
TR_102_2008	2008	TR	TR_REWET	0.6550	0.2311	27.6922	0.4628	0.2296	0.4302	0.7222	5.5833	0.6548
TR_103_1995	1995	TR	TR_BEFORE	0.5814	0.2097	20.9666	0.4046	0.2363	0.4460	0.6508	5.3810	0.6259
TR_109_2008	2008	TR	TR_REWET	0.5293	0.1927	27.0286	0.4622	0.2185	0.4040	0.6800	5.6400	0.6629
TR_110_1995	1995	TR	TR_BEFORE	0.6919	0.2401	19.8361	0.3848	0.2361	0.4455	0.7179	5.7692	0.6813
TR_116_2008	2008	TR	TR_REWET	0.6432	0.2300	21.1099	0.3987	0.2420	0.4594	0.7000	5.7500	0.6786
TR_117_1995	1995	TR	TR_BEFORE	0.8140	0.2771	19.0696	0.3698	0.2358	0.4447	0.7407	5.7778	0.6825
TR_12_2008	2008	TR	TR_REWET	0.5619	0.2002	27.5422	0.4760	0.2184	0.4039	0.7179	5.6154	0.6593
TR_123_2008	2008	TR	TR_REWET	0.8753	0.2936	19.4462	0.3748	0.2335	0.4394	0.7255	5.8235	0.6891
TR_124_1995	1995	TR	TR_BEFORE	0.6063	0.2163	21.6599	0.4088	0.2243	0.4178	0.7381	5.6429	0.6633
TR_13_1998	1998	TR	TR_BEFORE	0.5573	0.2001	21.7271	0.3989	0.2692	0.5234	0.7407	5.7778	0.6825
TR_130_2008	2008	TR	TR_REWET	0.7048	0.2408	27.0510	0.4295	0.2169	0.4002	0.7143	5.6500	0.6643
TR_131_1995	1995	TR	TR_BEFORE	0.7547	0.2511	28.9544	0.4600	0.2437	0.4634	0.7222	5.6667	0.6667
TR_137_2008	2008	TR	TR_REWET	0.7267	0.2356	38.5611	0.5088	0.1919	0.3416	0.7222	5.5385	0.6484
TR_138_1995	1995	TR	TR_BEFORE	0.8325	0.2707	29.7214	0.4743	0.2487	0.4753	0.8000	5.5000	0.6429
TR_144_2008	2008	TR	TR_REWET	0.5042	0.1763	49.1677	0.5974	0.1661	0.2808	0.7222	5.5833	0.6548
TR_145_1995	1995	TR	TR_BEFORE	0.7680	0.2546	28.2493	0.4634	0.2132	0.3917	0.7500	5.5833	0.6548
TR_15_2008	2008	TR	TR_REWET	0.5541	0.2021	23.3286	0.4258	0.2508	0.4801	0.6250	5.5625	0.6518
TR_151_2008	2008	TR	TR_REWET	0.5861	0.2044	31.0756	0.4763	0.2100	0.3840	0.7407	5.2222	0.6032
TR_152_1995	1995	TR	TR_BEFORE	0.6405	0.2048	34.0681	0.5189	0.2146	0.3949	0.7407	5.4444	0.6349
TR_158_2008	2008	TR	TR_REWET	0.7641	0.2467	40.6089	0.5023	0.2020	0.3653	0.6905	5.5000	0.6429
TR_159_1995	1995	TR	TR_BEFORE	0.4968	0.1804	26.5884	0.4844	0.2129	0.3909	0.7037	5.1111	0.5873
TR_16_1998	1998	TR	TR_BEFORE	0.3852	0.1507	26.6985	0.4872	0.1993	0.3589	0.7037	5.3333	0.6190
TR_165_2008	2008	TR	TR_REWET	0.6892	0.2392	24.8541	0.4493	0.2313	0.4343	0.7067	5.6400	0.6629
TR_166_1995	1995	TR	TR_BEFORE	0.5777	0.2031	22.8261	0.4263	0.2228	0.4141	0.7368	5.4737	0.6391
TR_172_2008	2008	TR	TR_REWET	0.8049	0.2684	26.5699	0.4452	0.2106	0.3854	0.7556	5.5333	0.6476
TR_173_1995	1995	TR	TR_BEFORE	0.6793	0.2421	24.4317	0.4608	0.2189	0.4050	0.7500	5.7500	0.6786
TR_179_2008	2008	TR	TR_REWET	0.4636	0.1735	27.1952	0.4946	0.2060	0.3746	0.8000	5.3000	0.6143
TR_18_2008	2008	TR	TR_REWET	0.4180	0.1591	24.6756	0.4529	0.2141	0.3937	0.7083	5.3750	0.6250
TR_180_1995	1995	TR	TR_BEFORE	0.5657	0.2045	24.9177	0.4542	0.2059	0.3744	0.6984	5.1905	0.5986
TR_186_2008	2008	TR	TR_REWET	0.3929	0.1519	24.2439	0.4529	0.2157	0.3975	0.7843	4.7647	0.5378
TR_187_1995	1995	TR	TR_BEFORE	0.4698	0.1763	27.4084	0.4932	0.2055	0.3734	0.6429	4.9286	0.5612
TR_19_1998	1998	TR	TR_BEFORE	0.4571	0.1667	25.6203	0.4764	0.2231	0.4149	0.6389	5.1667	0.5952
TR_193_2008	2008	TR	TR_REWET	0.4238	0.1622	23.9634	0.4524	0.2266	0.4233	0.7727	5.1364	0.5909
TR_194_1995	1995	TR	TR_BEFORE	0.4138	0.1587	23.8204	0.4515	0.2062	0.3751	0.6863	5.4706	0.6387
TR_200_2008	2008	TR	TR_REWET	0.4377	0.1660	24.0312	0.4524	0.2265	0.4228	0.8039	5.2353	0.6050
TR_201_1995	1995	TR	TR_BEFORE	0.4474	0.1702	24.0439	0.4494	0.2276	0.4254	0.7619	5.4762	0.6395
TR_207_2008	2008	TR	TR_REWET	0.4528	0.1677	24.8813	0.4579	0.2156	0.3973	0.7576	5.2273	0.6039
TR_208_1995	1995	TR	TR_BEFORE	0.4532	0.1693	25.9818	0.4734	0.2036	0.3690	0.6667	4.8125	0.5446
TR_21_2008	2008	TR	TR_REWET	0.4749	0.1778	24.8559	0.4620	0.2157	0.3974	0.7083	5.3125	0.6161
TR_214_2008	2008	TR	TR_REWET	0.7302	0.2558	23.7368	0.4330	0.2324	0.4369	0.7949	6.0000	0.7143
TR_215_1995	1995	TR	TR_BEFORE	0.5173	0.1832	25.9348	0.4783	0.2559	0.4921	0.8889	5.6667	0.6667
TR_22_1998	1998	TR	TR_BEFORE	0.4253	0.1625	25.5930	0.4758	0.2173	0.4013	0.6275	5.4118	0.6303
TR_221_2008	2008	TR	TR_REWET	0.5541	0.2019	24.5021	0.4496	0.1956	0.3502	0.7833	5.8000	0.6857
TR_222_1995	1995	TR	TR_BEFORE	0.5390	0.1944	23.8178	0.4497	0.2570	0.4948	0.7778	5.3333	0.6190
TR_228_2008	2008	TR	TR_REWET	0.8158	0.2760	21.5443	0.4173	0.2849	0.5604	0.7778	5.5000	0.6429
TR_229_1995	1995	TR	TR_BEFORE	0.4878	0.1790	35.5436	0.5535	0.2087	0.3810	0.4583	5.6667	0.6667
TR_23_2008	2008	TR	TR_REWET	0.5950	0.2091	22.3636	0.4178	0.2205	0.4087	0.6667	5.8462	0.6923
TR_235_2008	2008	TR	TR_REWET	0.5023	0.1846	36.7451	0.5661	0.2166	0.3995	0.5208	5.6364	0.6623
TR_236_1995	1995	TR	TR_BEFORE	0.4389	0.1659	28.3454	0.5011	0.1941	0.3466	0.6458	5.3125	0.6161
TR_24_1998	1998	TR	TR_BEFORE	0.4552	0.1715	22.7705	0.4315	0.2618	0.5060	0.6970	5.5000	0.6429
TR_242_2008	2008	TR	TR_REWET	0.4463	0.1686	27.6577	0.4902	0.1800	0.3134	0.6667	5.5455	0.6494
TR_243_1995	1995	TR	TR_BEFORE	0.4547	0.1695	27.3366	0.4914	0.2089	0.3815	0.7381	4.8571	0.5510
TR_249_2008	2008	TR	TR_REWET	0.5470	0.1954	25.4253	0.4633	0.2181	0.4031	0.7200	5.7600	0.6800
TR_250_1995	1995	TR	TR_BEFORE	0.3665	0.1443	24.1435	0.4543	0.2123	0.3895	0.6875	5.2500	0.6071
TR_256_2008	2008	TR	TR_REWET	0.4379	0.1664	24.2939	0.4434	0.2152	0.3963	0.7018	5.7368	0.6767
TR_257_1995	1995	TR	TR_BEFORE	0.4942	0.1809	25.6057	0.4749	0.2236	0.4160	0.8182	5.1818	0.5974
TR_26_2008	2008	TR	TR_REWET	0.6273	0.2218	21.0566	0.3911	0.2610	0.5040	0.7544	5.7222	0.6746
TR_263_2008	2008	TR	TR_REWET	0.5013	0.1835	24.7979	0.4529	0.2037	0.3693	0.7500	5.5500	0.6500
TR_264_1995	1995	TR	TR_BEFORE	0.4538	0.1724	26.8225	0.4895	0.2492	0.4762	0.7619	5.3571	0.6224
TR_27_1998	1998	TR	TR_BEFORE	0.4541	0.1690	24.9591	0.4677	0.2179	0.4027	0.7222	5.5556	0.6508
TR_270_2008	2008	TR	TR_REWET	0.5320	0.1923	24.9208	0.4518	0.1992	0.3587	0.7451	5.5294	0.6471
TR_271_1995	1995	TR	TR_BEFORE	0.4020	0.1564	23.9107	0.4502	0.2404	0.4555	0.7143	5.4286	0.6327
TR_277_2008	2008	TR	TR_REWET	0.5324	0.1951	22.1494	0.4144	0.2536	0.4867	0.6889	5.8000	0.6857
TR_278_1995	1995	TR	TR_BEFORE	0.4983	0.1839	24.2893	0.4524	0.2334	0.4392	0.5686	5.7059	0.6723
TR_284_2008	2008	TR	TR_REWET	0.6365	0.2252	23.6089	0.4310	0.2795	0.5476	0.7556	5.6000	0.6571
TR_285_1995	1995	TR	TR_BEFORE	0.5010	0.1827	25.9702	0.4732	0.2113	0.3870	0.5238	5.7143	0.6735
TR_29_2008	2008	TR	TR_REWET	0.5153	0.1874	27.0807	0.4832	0.1950	0.3487	0.6222	5.9375	0.7054
TR_291_2008	2008	TR	TR_REWET	0.7820	0.2629	25.8473	0.4686	0.2064	0.3756	0.6970	6.0000	0.7143
TR_3_2008	2008	TR	TR_REWET	0.3985	0.1552	24.6410	0.4285	0.2263	0.4225	0.7619	5.5385	0.6484
TR_30_1998	1998	TR	TR_BEFORE	0.4846	0.1785	24.8918	0.4671	0.2237	0.4164	0.7407	5.5000	0.6429
TR_32_2008	2008	TR	TR_REWET	0.5418	0.1962	26.5710	0.4667	0.2049	0.3722	0.6491	5.9474	0.7068
TR_33_1998	1998	TR	TR_BEFORE	0.3753	0.1472	24.5127	0.4497	0.1936	0.3455	0.5833	5.3750	0.6250
TR_35_2008	2008	TR	TR_REWET	0.5993	0.2142	25.3029	0.4533	0.2153	0.3966	0.7308	5.7692	0.6813
TR_36_1998	1998	TR	TR_BEFORE	0.3934	0.1528	25.5434	0.4732	0.2163	0.3989	0.6923	5.0000	0.5714

TR_38_2008	2008	TR	TR_REWET	0.3687	0.1451	26.2935	0.4744	0.2102	0.3846	0.5439	5.5789	0.6541
TR_39_1998	1998	TR	TR_BEFORE	0.4271	0.1649	26.1829	0.4784	0.2490	0.4758	0.7333	5.5000	0.6429
TR_4_1998	1998	TR	TR_BEFORE	0.4652	0.1760	28.3745	0.4976	0.1784	0.3097	0.6667	4.6667	0.5238
TR_41_2008	2008	TR	TR_REWET	0.4238	0.1658	28.4864	0.4909	0.2305	0.4322	0.5667	6.1000	0.7286
TR_42_1998	1998	TR	TR_BEFORE	0.4657	0.1713	25.8208	0.4786	0.2340	0.4407	0.8056	5.6667	0.6667
TR_44_2008	2008	TR	TR_REWET	0.5839	0.1888	37.7867	0.5488	0.1647	0.2776	0.6667	5.7778	0.6825
TR_45_2008	2008	TR	TR_REWET	0.7179	0.2442	24.5396	0.4076	0.2314	0.4345	0.6667	5.9333	0.7048
TR_46_2008	2008	TR	TR_REWET	0.3701	0.1444	24.2828	0.4401	0.2080	0.3794	0.6316	5.5263	0.6466
TR_47_1995	1995	TR	TR_BEFORE	0.4907	0.1804	24.9490	0.4660	0.2292	0.4292	0.7179	5.3077	0.6154
TR_53_2008	2008	TR	TR_REWET	0.5066	0.1868	25.4324	0.4643	0.2285	0.4276	0.7381	5.4286	0.6327
TR_54_1995	1995	TR	TR_BEFORE	0.6475	0.2307	23.5020	0.4235	0.2163	0.3988	0.6667	5.2857	0.6122
TR_6_2008	2008	TR	TR_REWET	0.4849	0.1811	28.4165	0.5001	0.1931	0.3442	0.7917	5.1250	0.5893
TR_60_2008	2008	TR	TR_REWET	0.7259	0.2484	26.0239	0.3847	0.2339	0.4404	0.7381	5.4667	0.6381
TR_67_2008	2008	TR	TR_REWET	0.6273	0.2186	23.6788	0.4387	0.2768	0.5413	0.7556	5.6000	0.6571
TR_68_1995	1995	TR	TR_BEFORE	1.0107	0.2975	23.4173	0.4396	0.2360	0.4454	0.6667	5.6667	0.6667
TR_7_1998	1998	TR	TR_BEFORE	0.3458	0.1371	29.6405	0.5162	0.1803	0.3141	0.7083	4.8750	0.5536
TR_74_2008	2008	TR	TR_REWET	0.9719	0.2848	23.3979	0.4227	0.2370	0.4477	0.7179	5.6923	0.6703
TR_75_1995	1995	TR	TR_BEFORE	0.6178	0.2164	24.2226	0.4536	0.2520	0.4828	0.7059	5.4118	0.6303
TR_81_2008	2008	TR	TR_REWET	0.6335	0.2243	25.1275	0.4603	0.2369	0.4473	0.6852	5.6667	0.6667
TR_82_1995	1995	TR	TR_BEFORE	0.5495	0.1979	25.6120	0.4720	0.2237	0.4163	0.6389	5.2500	0.6071
TR_88_2008	2008	TR	TR_REWET	0.5834	0.2107	25.6853	0.4646	0.2404	0.4557	0.7273	5.8182	0.6883
TR_89_1995	1995	TR	TR_BEFORE	0.5459	0.1947	27.3638	0.4949	0.2102	0.3845	0.7037	5.3333	0.6190
TR_9_2008	2008	TR	TR_REWET	0.4505	0.1678	25.8249	0.4732	0.2369	0.4473	0.8000	5.4000	0.6286
TR_95_2008	2008	TR	TR_REWET	0.5063	0.1875	26.1000	0.4684	0.2134	0.3921	0.7255	5.5882	0.6555
TR_96_1995	1995	TR	TR_BEFORE	0.8544	0.2676	27.3803	0.4690	0.2251	0.4196	0.7193	5.7368	0.6767
UL_A1_2010	2010	UL	UL_REWET	0.4630	0.1736	19.5290	0.3680	0.2926	0.5784	0.6667	5.4000	0.6286
UL_A1a_2010	2010	UL	UL_REWET	0.4894	0.1711	17.3892	0.3062	0.3068	0.6119	0.7500	4.5556	0.5079
UL_A1b_2010	2010	UL	UL_REWET	0.3825	0.1472	17.7607	0.3240	0.3226	0.6491	0.7917	5.2500	0.6071
UL_A2_2010	2010	UL	UL_REWET	0.4300	0.1525	19.9581	0.3609	0.3100	0.6194	0.7333	5.1818	0.5974
UL_A2a_2010	2010	UL	UL_REWET	0.5757	0.1966	16.7965	0.3233	0.3147	0.6304	0.6667	5.1429	0.5918
UL_A2b_2010	2010	UL	UL_REWET	0.6250	0.2112	12.6440	0.2640	0.3502	0.7141	0.7333	5.1667	0.5952
UL_A3_2010	2010	UL	UL_REWET	0.3700	0.1376	18.5415	0.3328	0.3109	0.6216	0.6667	5.5000	0.6429
UL_A3a_2010	2010	UL	UL_REWET	0.2771	0.1074	15.9545	0.2950	0.3386	0.6866	0.8095	5.1429	0.5918
UL_A3b_2010	2010	UL	UL_REWET	0.3179	0.1229	14.7249	0.2744	0.3566	0.7290	0.7619	5.2857	0.6122
UL_A4_2010	2010	UL	UL_REWET	0.5679	0.1963	10.0223	0.2097	0.3793	0.7825	0.7222	4.7143	0.5306
UL_A4a_2010	2010	UL	UL_REWET	0.4322	0.1506	14.2543	0.2648	0.3447	0.7010	0.7917	4.7778	0.5397
UL_A4b_2010	2010	UL	UL_REWET	0.5958	0.2024	9.4510	0.1951	0.3757	0.7740	0.6667	4.6667	0.5238
UL_A5_2010	2010	UL	UL_REWET	0.3593	0.1386	13.9819	0.2520	0.3497	0.7129	0.8095	5.0000	0.5714
UL_A5a_2010	2010	UL	UL_REWET	0.4240	0.1502	13.2701	0.2481	0.3611	0.7397	0.7778	4.8000	0.5429
UL_A5b_2010	2010	UL	UL_REWET	0.4016	0.1426	14.1829	0.2636	0.3407	0.6917	0.7667	5.0000	0.5714
UL_B1_2010	2010	UL	UL_REWET	0.4736	0.1800	18.0673	0.3432	0.3152	0.6318	0.7143	5.5714	0.6531
UL_B1a_2010	2010	UL	UL_REWET	0.4175	0.1594	20.8694	0.3870	0.3007	0.5975	0.6667	5.3750	0.6250
UL_B1b_2010	2010	UL	UL_REWET	0.4750	0.1791	18.1600	0.3539	0.3286	0.6632	0.7222	5.3333	0.6190
UL_B2_2010	2010	UL	UL_REWET	0.4244	0.1620	18.7218	0.3453	0.3216	0.6468	0.7917	5.2500	0.6071
UL_B2a_2010	2010	UL	UL_REWET	0.4566	0.1740	20.3706	0.3879	0.2900	0.5724	0.6667	5.5000	0.6429
UL_B2b_2010	2010	UL	UL_REWET	0.5667	0.2096	12.7950	0.2745	0.3702	0.7610	0.6667	5.6667	0.6667
UL_B3_2010	2010	UL	UL_REWET	0.7300	0.2381	12.0536	0.2518	0.3852	0.7965	0.7778	5.5000	0.6429
UL_B3a_2010	2010	UL	UL_REWET	0.8083	0.2624	13.2183	0.2793	0.3635	0.7453	0.6667	5.6667	0.6667
UL_B3b_2010	2010	UL	UL_REWET	0.3938	0.1497	18.4999	0.3494	0.3431	0.6973	0.8333	5.2500	0.6071
UL_B4_2010	2010	UL	UL_REWET	0.3490	0.1368	17.6864	0.3239	0.2987	0.5928	0.8000	5.2000	0.6000
UL_B4a_2010	2010	UL	UL_REWET	0.3940	0.1497	10.7619	0.2317	0.3888	0.8049	0.8000	5.2000	0.6000
UL_B4b_2010	2010	UL	UL_REWET	0.3460	0.1356	17.6429	0.3224	0.3133	0.6272	0.7333	5.4000	0.6286
UL_B5_2010	2010	UL	UL_REWET	0.3063	0.1241	12.2227	0.2314	0.3829	0.7910	0.7500	5.2500	0.6071
UL_B5a_2010	2010	UL	UL_REWET	0.3583	0.1413	13.6737	0.2797	0.3547	0.7246	0.7778	5.0000	0.5714
UL_B5b_2010	2010	UL	UL_REWET	0.2610	0.1065	16.3654	0.2993	0.3256	0.6561	0.7619	4.8571	0.5510
UL_C1_2010	2010	UL	UL_REWET	0.6317	0.2139	11.0068	0.2364	0.3404	0.6909	0.7333	4.8333	0.5476
UL_C1a_2010	2010	UL	UL_REWET	0.6125	0.2061	13.3442	0.2838	0.3414	0.6933	0.7333	5.0000	0.5714
UL_C1b_2010	2010	UL	UL_REWET	0.7330	0.2456	10.8482	0.2315	0.3361	0.6808	0.7500	5.0000	0.5714
UL_C2_2010	2010	UL	UL_REWET	0.5393	0.1830	15.3929	0.2915	0.3247	0.6541	0.7222	5.1429	0.5918
UL_C2a_2010	2010	UL	UL_REWET	0.3200	0.1208	18.4131	0.3310	0.3332	0.6740	0.8000	5.6000	0.6571
UL_C2b_2010	2010	UL	UL_REWET	0.5750	0.2129	13.4713	0.2901	0.3397	0.6894	0.6667	5.5000	0.6429
UL_C3_2010	2010	UL	UL_REWET	0.5558	0.1875	9.7334	0.2054	0.3717	0.7646	0.8000	4.8333	0.5476
UL_C3a_2010	2010	UL	UL_REWET	0.7563	0.2525	12.2937	0.2678	0.3399	0.6899	0.7778	5.0000	0.5714
UL_C3b_2010	2010	UL	UL_REWET	0.3725	0.1470	10.1679	0.2077	0.3633	0.7449	0.8333	4.6667	0.5238
UL_C4_2010	2010	UL	UL_REWET	0.4908	0.1680	14.8588	0.2685	0.3152	0.6316	0.7333	5.5000	0.6429
UL_C4a_2010	2010	UL	UL_REWET	0.4121	0.1415	16.3438	0.3094	0.3200	0.6429	0.7778	4.8571	0.5510
UL_C4b_2010	2010	UL	UL_REWET	0.4993	0.1734	15.1794	0.2833	0.3219	0.6475	0.7778	5.0000	0.5714
UL_C5_2010	2010	UL	UL_REWET	0.4343	0.1508	14.0879	0.2568	0.3487	0.7106	0.8333	5.0000	0.5714
UL_C5a_2010	2010	UL	UL_REWET	0.3956	0.1389	13.8019	0.2573	0.3504	0.7145	0.8095	4.8750	0.5536
UL_C5b_2010	2010	UL	UL_REWET	0.4942	0.1703	8.9703	0.1810	0.3694	0.7592	0.7333	4.3333	0.4762
UL_D1_2010	2010	UL	UL_REWET	0.5840	0.1962	13.6989	0.2793	0.3518	0.7178	0.8333	5.4000	0.6286
UL_D1a_2010	2010	UL	UL_REWET	0.4764	0.1657	11.9870	0.2411	0.3594	0.7358	0.8333	5.1429	0.5918
UL_D1b_2010	2010	UL	UL_REWET	0.5371	0.1850	12.7422	0.2596	0.3711	0.7633	0.8333	5.2857	0.6122
UL_D2_2010	2010	UL	UL_REWET	0.4550	0.1594	12.2638	0.2421	0.3831	0.7915	0.8889	4.8571	0.5510
UL_D2a_2010	2010	UL	UL_REWET	0.4950	0.1727	11.5699	0.2353	0.3811	0.7867	0.8571	5.2500	0.6071
UL_D2b_2010	2010	UL	UL_REWET	0.3575	0.1384	12.8798	0.2612	0.3741	0.7703	0.8333	5.3750	0.6250
UL_D3_2010	2010	UL	UL_REWET	0.4721	0.1623	17.7830	0.3409	0.3152	0.6317	0.7778	5.0000	0.5714

UL_D3a_2010	2010	UL	UL_REWET	0.3400	0.1347	11.1563	0.2336	0.3569	0.7298	0.9167	4.7500	0.5357
UL_D3b_2010	2010	UL	UL_REWET	0.4880	0.1619	15.5132	0.2716	0.3250	0.6548	0.8333	5.0000	0.5714
UL_D4_2010	2010	UL	UL_REWET	0.4675	0.1591	16.7866	0.3102	0.3340	0.6760	0.6667	5.3333	0.6190
UL_D4b_2010	2010	UL	UL_REWET	0.4893	0.1694	16.0399	0.2983	0.3324	0.6721	0.7222	5.4286	0.6327
UL_D5_2010	2010	UL	UL_REWET	0.5500	0.1859	9.2012	0.1873	0.3749	0.7721	0.6667	4.4000	0.4857
UL_D5a_2010	2010	UL	UL_REWET	0.5760	0.1944	17.5406	0.3110	0.3228	0.6497	0.6667	5.2000	0.6000
UL_D5b_2010	2010	UL	UL_REWET	0.3267	0.1298	9.8929	0.2017	0.3568	0.7295	0.7778	4.5000	0.5000
UL_E1_2002	2002	UL	UL_BEFORE	0.5225	0.1789	12.7184	0.2577	0.3502	0.7140	0.8667	5.1667	0.5952
UL_E2_2002	2002	UL	UL_BEFORE	0.4671	0.1617	15.5698	0.3170	0.3642	0.7469	0.8889	5.2857	0.6122
UL_E3_2002	2002	UL	UL_BEFORE	0.5270	0.1780	10.9109	0.2253	0.3861	0.7985	0.9167	4.8000	0.5429
UL_E4_2002	2002	UL	UL_BEFORE	0.8083	0.2624	13.2183	0.2793	0.3635	0.7453	0.6667	5.6667	0.6667
UL_E5_2002	2002	UL	UL_BEFORE	0.5642	0.1944	11.8051	0.2458	0.3528	0.7202	0.8000	5.1667	0.5952
UL_F1_2002	2002	UL	UL_BEFORE	0.4320	0.1423	14.5301	0.2855	0.3671	0.7538	1.0000	5.0000	0.5714
UL_F2_2002	2002	UL	UL_BEFORE	0.2038	0.0877	14.7447	0.2880	0.3908	0.8097	0.9167	5.2500	0.6071
UL_F3_2002	2002	UL	UL_BEFORE	0.4642	0.1594	13.2440	0.2664	0.3825	0.7900	0.9333	5.1667	0.5952
UL_F4_2002	2002	UL	UL_BEFORE	0.6210	0.2049	16.0379	0.2868	0.3283	0.6626	0.6667	5.4000	0.6286
UL_F5_2002	2002	UL	UL_BEFORE	0.5414	0.1819	20.9442	0.3824	0.2836	0.5574	0.6667	5.6000	0.6571
UL_G1_2002	2002	UL	UL_BEFORE	0.2075	0.0864	22.3830	0.4003	0.3466	0.7056	0.8889	5.5000	0.6429
UL_G2_2002	2002	UL	UL_BEFORE	0.2169	0.0909	20.4505	0.3677	0.3427	0.6965	0.9167	5.3750	0.6250
UL_G3_2002	2002	UL	UL_BEFORE	0.4729	0.1610	18.8450	0.3284	0.3275	0.6606	0.8889	5.2857	0.6122
UL_G4_2002	2002	UL	UL_BEFORE	0.5729	0.1991	15.0184	0.3020	0.3141	0.6292	0.6667	5.2222	0.6032
UL_G5_2002	2002	UL	UL_BEFORE	0.6930	0.2312	19.0948	0.3691	0.3169	0.6357	0.7500	5.4000	0.6286
UL_H1_2002	2002	UL	UL_BEFORE	0.4918	0.1709	19.7235	0.3563	0.3322	0.6718	0.8333	5.5455	0.6494
UL_H2_2002	2002	UL	UL_BEFORE	0.3636	0.1365	26.0013	0.4491	0.2759	0.5392	0.7619	5.5714	0.6531
UL_H3_2002	2002	UL	UL_BEFORE	0.3201	0.1248	24.6410	0.4331	0.2641	0.5115	0.6667	5.7000	0.6714
UL_H4_2002	2002	UL	UL_BEFORE	0.6033	0.2058	19.2550	0.3577	0.3061	0.6102	0.7083	5.2222	0.6032
UL_H5_2002	2002	UL	UL_BEFORE	0.3888	0.1472	21.2436	0.3944	0.3231	0.6503	0.7083	5.6250	0.6607
UL_I1_2010	2010	UL	UL_REWET	0.4380	0.1624	14.7402	0.2956	0.3138	0.6284	0.8000	4.6000	0.5143
UL_I2_2010	2010	UL	UL_REWET	0.5129	0.1758	17.5808	0.3161	0.3197	0.6423	0.7778	5.0000	0.5714
UL_I3_2010	2010	UL	UL_REWET	0.4486	0.1614	13.8663	0.2877	0.3803	0.7849	0.8333	5.2727	0.6104
UL_I4_2010	2010	UL	UL_REWET	0.4350	0.1512	14.2583	0.2961	0.3827	0.7905	0.8333	5.1429	0.5918
UL_I5_2010	2010	UL	UL_REWET	0.5450	0.1832	12.5824	0.2591	0.3754	0.7734	0.8000	5.3333	0.6190
UL_J1_2010	2010	UL	UL_REWET	0.4121	0.1431	14.4720	0.2903	0.3775	0.7784	0.9444	5.1429	0.5918
UL_J2_2010	2010	UL	UL_REWET	0.6275	0.2057	10.3501	0.2147	0.3429	0.6968	0.8889	4.5000	0.5000
UL_J3_2010	2010	UL	UL_REWET	0.3714	0.1443	11.1429	0.2294	0.3634	0.7452	0.7143	5.1429	0.5918
UL_J4_2010	2010	UL	UL_REWET	0.4550	0.1694	13.1784	0.2777	0.3765	0.7760	0.8333	5.2500	0.6071
UL_J5_2010	2010	UL	UL_REWET									
UL_K1_2010	2010	UL	UL_REWET	0.4100	0.1559	14.9288	0.3139	0.3872	0.8012	0.8667	5.4000	0.6286
UL_K2_2010	2010	UL	UL_REWET	0.3363	0.1277	12.2534	0.2587	0.4049	0.8429	0.8333	5.0000	0.5714
UL_K3_2010	2010	UL	UL_REWET	0.1875	0.0808	15.2270	0.3249	0.4020	0.8360	0.8333	4.5000	0.5000
UL_K4_2010	2010	UL	UL_REWET	NA	NA	NA	NA	NA	NA	NA	NA	NA
UL_K5_2010	2010	UL	UL_REWET									
UL_L1_2010	2010	UL	UL_REWET	0.3338	0.1269	12.8213	0.2515	0.3805	0.7854	0.9167	5.5000	0.6429
UL_L2_2010	2010	UL	UL_REWET	0.8500	0.2549	17.5675	0.3466	0.3547	0.7246	1.0000	5.5000	0.6429
UL_L3_2010	2010	UL	UL_REWET	0.4990	0.1668	9.4966	0.1982	0.3847	0.7952	0.7500	4.8000	0.5429
UL_L4_2010	2010	UL	UL_REWET									
UL_L5_2010	2010	UL	UL_REWET	NA	NA	NA	NA	NA	NA	NA	NA	NA
UL_M1_2002	2002	UL	UL_BEFORE	0.5533	0.1874	13.1217	0.2606	0.3924	0.8133	0.8667	5.0000	0.5714
UL_M2_2002	2002	UL	UL_BEFORE	0.5029	0.1721	13.4498	0.2777	0.3849	0.7958	0.8333	5.2857	0.6122
UL_M3_2002	2002	UL	UL_BEFORE	0.2113	0.0873	12.8749	0.2653	0.3779	0.7791	0.8333	5.0000	0.5714
UL_M4_2002	2002	UL	UL_BEFORE	0.2538	0.1047	9.3037	0.1920	0.3711	0.7632	0.7500	4.7500	0.5357
UL_M5_2002	2002	UL	UL_BEFORE	0.2150	0.0923	7.8159	0.1495	0.3420	0.6947	1.0000	4.0000	0.4286
UL_N1_2002	2002	UL	UL_BEFORE	0.4370	0.1444	12.5221	0.2509	0.3699	0.7604	0.9167	4.8000	0.5429
UL_N2_2002	2002	UL	UL_BEFORE	0.4933	0.1692	11.2755	0.2316	0.3714	0.7640	0.8667	4.6667	0.5238
UL_N3_2002	2002	UL	UL_BEFORE	0.4250	0.1394	15.2747	0.3060	0.3669	0.7533	0.8333	5.0000	0.5714
UL_N4_2002	2002	UL	UL_BEFORE	0.2430	0.1009	14.6360	0.2973	0.3751	0.7727	0.8667	5.2000	0.6000
UL_N5_2002	2002	UL	UL_BEFORE	0.6750	0.2426	12.5048	0.2727	0.3564	0.7287	0.7500	6.0000	0.7143
UL_O1_2002	2002	UL	UL_BEFORE	0.1613	0.0703	18.3159	0.3613	0.3976	0.8257	1.0000	5.5000	0.6429
UL_O2_2002	2002	UL	UL_BEFORE	0.5183	0.1751	13.8806	0.2812	0.3936	0.8163	1.0000	5.3333	0.6190
UL_O3_2002	2002	UL	UL_BEFORE	0.8333	0.2628	16.3180	0.3326	0.3806	0.7855	1.0000	6.0000	0.7143
UL_O4_2002	2002	UL	UL_BEFORE	0.5100	0.1756	13.2952	0.2708	0.4000	0.8313	0.8889	5.5714	0.6531
UL_O5_2002	2002	UL	UL_BEFORE	0.5700	0.1938	11.3594	0.2373	0.4060	0.8453	0.8667	5.3333	0.6190
UL_P1_2002	2002	UL	UL_BEFORE	0.4040	0.1478	15.8257	0.3190	0.3921	0.8127	0.9333	6.0000	0.7143
UL_P2_2002	2002	UL	UL_BEFORE	0.3650	0.1363	15.5295	0.3090	0.4109	0.8570	1.0000	6.3333	0.7619
UL_P3_2002	2002	UL	UL_BEFORE	0.4300	0.1574	17.5606	0.3547	0.3896	0.8067	0.9333	6.0000	0.7143
UL_P4_2002	2002	UL	UL_BEFORE	0.3120	0.1214	14.6117	0.2931	0.4034	0.8392	1.0000	5.6000	0.6571
UL_P5_2002	2002	UL	UL_BEFORE	0.1800	0.0782	7.8377	0.1502	0.3890	0.8055	1.0000	4.5000	0.5000
UZ_101	1982	UZ	UZ_BEFORE	0.6252	0.2211	18.9654	0.3601	0.2632	0.5093	0.6111	5.9231	0.7033
UZ_102	1982	UZ	UZ_BEFORE	0.4716	0.1735	20.7694	0.3996	0.2540	0.4876	0.6222	5.9375	0.7054
UZ_103	1982	UZ	UZ_BEFORE	0.5050	0.1849	20.3385	0.3860	0.2500	0.4782	0.6458	5.8235	0.6891
UZ_104	1982	UZ	UZ_BEFORE	0.5839	0.2104	19.6576	0.3707	0.2684	0.5215	0.6863	6.0588	0.7227
UZ_105	1982	UZ	UZ_BEFORE	0.5603	0.2025	21.5940	0.3989	0.2407	0.4564	0.7246	5.8182	0.6883
UZ_106	1982	UZ	UZ_BEFORE	0.4775	0.1751	21.1765	0.3982	0.2443	0.4648	0.6933	5.6154	0.6593
UZ_107	1982	UZ	UZ_BEFORE	0.4663	0.1712	22.7987	0.4312	0.2186	0.4044	0.6452	5.3636	0.6234
UZ_108	1982	UZ	UZ_BEFORE	0.5766	0.2081	21.9162	0.4097	0.2511	0.4809	0.6667	5.7037	0.6720
UZ_109	1982	UZ	UZ_BEFORE	0.6167	0.2154	18.8197	0.3642	0.2737	0.5341	0.6471	5.7500	0.6786
UZ_110	1982	UZ	UZ_BEFORE	0.5777	0.2065	20.7402	0.3879	0.2608	0.5037	0.6833	5.8571	0.6939

UZ_111	1982	UZ	UZ_BEFORE	0.6424	0.2248	20.3935	0.3848	0.2661	0.5161	0.6458	5.9412	0.7059
UZ_112	1982	UZ	UZ_BEFORE	0.6149	0.2189	20.7275	0.3946	0.2506	0.4795	0.6111	5.9474	0.7068
UZ_113	1982	UZ	UZ_BEFORE	0.6360	0.2259	19.5095	0.3718	0.2617	0.5057	0.7111	5.8750	0.6964
UZ_114	1982	UZ	UZ_BEFORE	0.5474	0.1969	21.7222	0.4030	0.2473	0.4718	0.6812	5.7083	0.6726
UZ_115	1982	UZ	UZ_BEFORE	0.5086	0.1783	20.2436	0.3891	0.2758	0.5389	0.6667	5.8000	0.6857
UZ_116	1982	UZ	UZ_BEFORE	0.5855	0.2050	19.4044	0.3706	0.2901	0.5725	0.6458	5.7895	0.6842
UZ_117	1982	UZ	UZ_BEFORE	0.5535	0.2006	23.1995	0.4236	0.2441	0.4644	0.6833	6.1500	0.7357
UZ_118	1982	UZ	UZ_BEFORE	0.6615	0.2293	21.2347	0.3996	0.2595	0.5007	0.5778	5.8824	0.6975
UZ_119	1982	UZ	UZ_BEFORE	0.5986	0.2092	19.9545	0.3855	0.2587	0.4987	0.6000	5.8235	0.6891
UZ_120	1982	UZ	UZ_BEFORE	0.6221	0.2183	18.6832	0.3595	0.2742	0.5351	0.7037	5.9500	0.7071
UZ_121	1982	UZ	UZ_BEFORE	0.4725	0.1674	18.3647	0.3616	0.2903	0.5731	0.5278	5.9375	0.7054
UZ_122	1982	UZ	UZ_BEFORE	0.4446	0.1591	18.8098	0.3679	0.2725	0.5312	0.5490	5.7500	0.6786
UZ_123	1982	UZ	UZ_BEFORE	0.6014	0.2115	21.6705	0.4057	0.2528	0.4848	0.6825	5.6957	0.6708
UZ_124	1982	UZ	UZ_BEFORE	0.5402	0.1901	21.1594	0.4061	0.3125	0.6254	0.6190	5.7500	0.6786
UZ_125	1982	UZ	UZ_BEFORE	0.5083	0.1804	15.7168	0.3158	0.3385	0.6864	0.6190	6.0000	0.7143
UZ_126	1982	UZ	UZ_BEFORE	0.4977	0.1752	20.3724	0.3975	0.2647	0.5129	0.5385	5.9333	0.7048
UZ_127	1982	UZ	UZ_BEFORE	0.6007	0.2085	17.2500	0.3447	0.2959	0.5863	0.5833	5.7368	0.6767
UZ_128	1982	UZ	UZ_BEFORE	0.6076	0.2099	18.1300	0.3585	0.3113	0.6225	0.5952	5.8750	0.6964
UZ_129	1982	UZ	UZ_BEFORE	0.4747	0.1698	16.8501	0.3419	0.3011	0.5985	0.5833	6.0000	0.7143
UZ_130	1982	UZ	UZ_BEFORE	0.4214	0.1535	20.0841	0.3831	0.2915	0.5759	0.6061	5.9286	0.7041
UZ_131	1982	UZ	UZ_BEFORE	0.4834	0.1721	17.9731	0.3495	0.3101	0.6198	0.6000	5.7368	0.6767
UZ_132	1982	UZ	UZ_BEFORE	0.6661	0.2309	18.2365	0.3489	0.2824	0.5544	0.6889	5.7059	0.6723
UZ_133	1982	UZ	UZ_BEFORE	0.6269	0.2237	21.2794	0.3961	0.2598	0.5012	0.6667	6.0455	0.7208
UZ_134	1982	UZ	UZ_BEFORE	0.5055	0.1771	18.2493	0.3606	0.2898	0.5718	0.6250	5.8500	0.6929
UZ_135	1982	UZ	UZ_BEFORE	0.5323	0.1877	20.4817	0.3977	0.2786	0.5456	0.6444	5.8889	0.6984
UZ_136	1982	UZ	UZ_BEFORE	0.5031	0.1790	18.7205	0.3743	0.2798	0.5485	0.5625	5.7895	0.6842
UZ_137	1982	UZ	UZ_BEFORE	0.5446	0.1913	19.3932	0.3845	0.2888	0.5695	0.5476	5.8750	0.6964
UZ_138	1982	UZ	UZ_BEFORE	0.5614	0.1961	21.2608	0.4104	0.2688	0.5224	0.6667	5.8333	0.6905
UZ_139	1982	UZ	UZ_BEFORE	0.6271	0.2172	18.5606	0.3734	0.2980	0.5912	0.6000	5.9412	0.7059
UZ_140	1982	UZ	UZ_BEFORE	0.5515	0.1926	18.2486	0.3652	0.3050	0.6077	0.5641	5.6667	0.6667
UZ_141	1982	UZ	UZ_BEFORE	0.6054	0.2089	17.9616	0.3619	0.3234	0.6509	0.6061	5.7692	0.6813
UZ_142	1982	UZ	UZ_BEFORE	0.5701	0.2047	20.8761	0.4006	0.2784	0.5451	0.6667	5.6667	0.6667
UZ_143	1982	UZ	UZ_BEFORE	0.5216	0.1861	18.9853	0.3763	0.2962	0.5870	0.5686	5.8000	0.6857
UZ_144	1982	UZ	UZ_BEFORE	0.5723	0.2004	19.1685	0.3759	0.2886	0.5690	0.6296	5.5500	0.6500
UZ_145	1982	UZ	UZ_BEFORE	0.6489	0.2282	20.3868	0.3975	0.2580	0.4970	0.6222	6.0000	0.7143
UZ_146	1982	UZ	UZ_BEFORE	0.4899	0.1796	21.3839	0.4125	0.2683	0.5212	0.6481	5.7778	0.6825
UZ_147	1982	UZ	UZ_BEFORE	0.6868	0.2356	22.0597	0.4152	0.2647	0.5128	0.6667	5.7143	0.6735
UZ_148	1982	UZ	UZ_BEFORE	0.5988	0.2068	21.0948	0.4057	0.2857	0.5622	0.6429	5.8125	0.6875
UZ_149	1982	UZ	UZ_BEFORE	0.6044	0.2104	18.9264	0.3790	0.2828	0.5554	0.6042	5.7778	0.6825
UZ_150	1982	UZ	UZ_BEFORE	0.7058	0.2431	20.7706	0.4042	0.2672	0.5188	0.6222	6.0000	0.7143
UZ_151	1982	UZ	UZ_BEFORE	0.5966	0.2125	20.2703	0.3864	0.2717	0.5293	0.6667	6.0588	0.7227
UZ_152	1982	UZ	UZ_BEFORE	0.6543	0.2263	22.1903	0.4108	0.2366	0.4466	0.6905	5.8710	0.6959
UZ_153	1982	UZ	UZ_BEFORE	0.5849	0.2090	23.3162	0.4333	0.2764	0.5403	0.6471	5.8947	0.6992
UZ_154	1982	UZ	UZ_BEFORE	0.5717	0.2063	22.7954	0.4294	0.2798	0.5483	0.6275	5.8333	0.6905
UZ_155	1982	UZ	UZ_BEFORE	0.5868	0.2092	23.8502	0.4412	0.2481	0.4739	0.6349	5.9565	0.7081
UZ_401	2013	UZ	UZ_REWET	0.4015	0.1528	23.8544	0.4500	0.2350	0.4430	0.6111	5.6154	0.6593
UZ_402	2013	UZ	UZ_REWET	0.4129	0.1586	21.0085	0.4120	0.2432	0.4621	0.5667	5.8182	0.6883
UZ_403	2013	UZ	UZ_REWET	0.5186	0.1833	21.2247	0.4132	0.2662	0.5164	0.6190	5.6250	0.6607
UZ_404	2013	UZ	UZ_REWET	0.6100	0.2103	20.2507	0.3991	0.2763	0.5401	0.6667	5.9000	0.7000
UZ_405	2013	UZ	UZ_REWET	0.4188	0.1557	18.9144	0.3777	0.2857	0.5623	0.6667	6.0769	0.7253
UZ_406	2013	UZ	UZ_REWET	0.4966	0.1810	18.4847	0.3722	0.3018	0.6002	0.7778	5.7778	0.6825
UZ_407	2013	UZ	UZ_REWET	0.4696	0.1724	20.2765	0.4006	0.2793	0.5472	0.6190	5.6875	0.6696
UZ_408	2013	UZ	UZ_REWET	0.4787	0.1702	19.6964	0.3892	0.2736	0.5338	0.6222	5.8750	0.6964
UZ_409	2013	UZ	UZ_REWET	0.5778	0.1985	18.3943	0.3755	0.2987	0.5929	0.6667	5.9091	0.7013
UZ_410	2013	UZ	UZ_REWET	0.5904	0.1994	29.5477	0.4455	0.2719	0.5298	0.7083	6.1111	0.7302
UZ_411	2013	UZ	UZ_REWET	0.5938	0.2046	27.6823	0.4260	0.2749	0.5369	0.7778	6.0000	0.7143
UZ_412	2013	UZ	UZ_REWET	0.5388	0.1881	26.2335	0.4281	0.2724	0.5309	0.7143	6.0000	0.7143
UZ_413	2013	UZ	UZ_REWET	0.4641	0.1698	19.8696	0.3965	0.3069	0.6121	0.7222	5.8462	0.6923
UZ_414	2013	UZ	UZ_REWET	0.5403	0.1941	18.9891	0.3609	0.2710	0.5276	0.6875	5.7500	0.6786
UZ_415	2013	UZ	UZ_REWET	0.5618	0.1989	19.7112	0.3875	0.2738	0.5343	0.6471	5.8333	0.6905
UZ_416	2013	UZ	UZ_REWET	0.6232	0.2166	17.9057	0.3583	0.2973	0.5895	0.6905	5.8667	0.6952
UZ_417	2013	UZ	UZ_REWET	0.6416	0.2211	17.6471	0.3613	0.3025	0.6017	0.7273	5.7500	0.6786
UZ_418	2013	UZ	UZ_REWET	0.5581	0.1935	18.5761	0.3743	0.3236	0.6513	0.7000	5.7273	0.6753
UZ_419	2013	UZ	UZ_REWET	0.5280	0.1812	20.5270	0.3949	0.3012	0.5988	0.7000	5.8182	0.6883
UZ_420	2013	UZ	UZ_REWET	0.6356	0.2196	14.9950	0.3108	0.2917	0.5764	0.6111	5.8889	0.6984
UZ_421	2013	UZ	UZ_REWET	0.5285	0.1854	15.1252	0.3032	0.3638	0.7461	0.6111	6.1429	0.7347
UZ_422	2013	UZ	UZ_REWET	0.5530	0.1953	14.5835	0.2993	0.3644	0.7475	0.6190	6.0000	0.7143
UZ_423	2013	UZ	UZ_REWET	0.6058	0.2113	15.5549	0.3244	0.3464	0.7052	0.6667	6.0000	0.7143
UZ_424	2013	UZ	UZ_REWET	0.5057	0.1797	14.0231	0.2919	0.3538	0.7226	0.5833	5.7273	0.6753
UZ_425	2013	UZ	UZ_REWET	0.5027	0.1804	14.5101	0.3011	0.3415	0.6936	0.5926	6.0000	0.7143
UZ_426	2013	UZ	UZ_REWET	0.5064	0.1821	16.6593	0.3342	0.3144	0.6298	0.5667	6.0909	0.7273
UZ_427	2013	UZ	UZ_REWET	0.5366	0.1889	15.2112	0.3181	0.3497	0.7128	0.6190	6.0000	0.7143
UZ_428	2013	UZ	UZ_REWET	0.5106	0.1858	16.1721	0.3379	0.3042	0.6059	0.6250	6.3000	0.7571
UZ_429	2013	UZ	UZ_REWET	0.5337	0.1902	15.7666	0.3302	0.3251	0.6550	0.6250	6.3000	0.7571
UZ_430	2013	UZ	UZ_REWET	0.5129	0.1825	14.0181	0.2911	0.3376	0.6845	0.5833	5.9000	0.7000
UZ_431	2013	UZ	UZ_REWET	0.4914	0.1760	13.8714	0.2883	0.3569	0.7298	0.5714	5.7778	0.6825
UZ_432	2013	UZ	UZ_REWET	0.4579	0.1648	16.2226	0.3328	0.3068	0.6120	0.5926	5.8333	0.6905

UZ_433	2013	UZ	UZ_REWET	0.6861	0.2348	16.9211	0.3478	0.2834	0.5568	0.6667	5.7143	0.6735
UZ_434	2013	UZ	UZ_REWET	0.4431	0.1594	16.1258	0.3341	0.3227	0.6493	0.6111	5.8889	0.6984
UZ_435	2013	UZ	UZ_REWET	0.5641	0.1992	15.6823	0.3275	0.3361	0.6810	0.6667	6.2222	0.7460
UZ_436	2013	UZ	UZ_REWET	0.5409	0.1916	16.5641	0.3440	0.2975	0.5901	0.5926	5.8182	0.6883
UZ_437	2013	UZ	UZ_REWET	0.5392	0.1912	16.3284	0.3382	0.3021	0.6007	0.6190	5.9000	0.7000
UZ_438	2013	UZ	UZ_REWET	0.4856	0.1736	16.6010	0.3388	0.3075	0.6136	0.6250	6.1000	0.7286
UZ_439	2013	UZ	UZ_REWET	0.5784	0.2021	16.1618	0.3356	0.3423	0.6954	0.6667	6.0000	0.7143
UZ_440	2013	UZ	UZ_REWET	0.5677	0.2015	16.3581	0.3389	0.3146	0.6301	0.6250	6.2000	0.7429
UZ_441	2013	UZ	UZ_REWET	0.5808	0.2044	16.8593	0.3474	0.3176	0.6374	0.6190	6.0000	0.7143
UZ_442	2013	UZ	UZ_REWET	0.5307	0.1887	18.9311	0.3736	0.2855	0.5617	0.6333	5.6429	0.6633
UZ_443	2013	UZ	UZ_REWET	0.5006	0.1785	18.1622	0.3603	0.2908	0.5743	0.6250	5.7273	0.6753
UZ_444	2013	UZ	UZ_REWET	0.4007	0.1469	18.6773	0.3647	0.2666	0.5173	0.5152	6.0000	0.7143
UZ_445	2013	UZ	UZ_REWET	0.4914	0.1761	18.0664	0.3553	0.2870	0.5654	0.6296	6.0833	0.7262
UZ_446	2013	UZ	UZ_REWET	0.4748	0.1687	19.5083	0.3847	0.2621	0.5067	0.6667	5.7333	0.6762
UZ_447	2013	UZ	UZ_REWET	0.4640	0.1651	20.7069	0.3988	0.2586	0.4984	0.6389	5.7692	0.6813
UZ_448	2013	UZ	UZ_REWET	0.5046	0.1810	17.5984	0.3598	0.2881	0.5678	0.6061	5.8571	0.6939
UZ_449	2013	UZ	UZ_REWET	0.3067	0.1214	19.9619	0.3855	0.2422	0.4598	0.5556	6.0833	0.7262
UZ_450	2013	UZ	UZ_REWET	0.3978	0.1443	21.0129	0.3974	0.2585	0.4981	0.5833	5.7333	0.6762
UZ_451	2013	UZ	UZ_REWET	0.5300	0.1866	20.9203	0.3967	0.2489	0.4756	0.6667	5.7368	0.6767
UZ_452	2013	UZ	UZ_REWET	0.4059	0.1531	21.3463	0.4166	0.2653	0.5143	0.6154	5.7333	0.6762
UZ_453	2013	UZ	UZ_REWET	0.4653	0.1669	18.7399	0.3672	0.2863	0.5637	0.6389	5.6250	0.6607
UZ_454	2013	UZ	UZ_REWET	0.3162	0.1230	21.5882	0.4093	0.2511	0.4808	0.6410	5.8000	0.6857
UZ_455	2013	UZ	UZ_REWET	0.3791	0.1422	19.6158	0.3856	0.2576	0.4961	0.6923	5.4615	0.6374
VK_A10_1	2008	VK	VK_TSR	0.4547	0.1717	22.5923	0.4298	0.2735	0.5336	0.6389	5.5000	0.6429
VK_A10_2	2008	VK	VK_TSR	0.4526	0.1714	21.4394	0.3985	0.2642	0.5116	0.6111	5.3529	0.6218
VK_A10_3	2008	VK	VK_TSR	0.3848	0.1499	24.7219	0.4621	0.2821	0.5537	0.6000	5.5000	0.6429
VK_A10_4	2008	VK	VK_TSR	0.4503	0.1698	23.5922	0.4371	0.2695	0.5242	0.6000	5.3333	0.6190
VK_A10_5	2008	VK	VK_TSR	0.4736	0.1763	21.9446	0.4146	0.2562	0.4928	0.6296	5.3889	0.6270
VK_A5_1	2008	VK	VK_TSR	0.4266	0.1649	23.9553	0.4372	0.2409	0.4568	0.6111	5.2500	0.6071
VK_A5_2	2008	VK	VK_TSR	0.4445	0.1697	22.3345	0.4093	0.2457	0.4680	0.6389	5.3333	0.6190
VK_A5_3	2008	VK	VK_TSR	0.4129	0.1572	22.7160	0.4228	0.2482	0.4739	0.6111	5.2222	0.6032
VK_A5_4	2008	VK	VK_TSR	0.4717	0.1774	23.8128	0.4429	0.2474	0.4721	0.5625	5.4375	0.6339
VK_A5_5	2008	VK	VK_TSR	0.4365	0.1665	24.0513	0.4473	0.2531	0.4856	0.7083	5.1250	0.5893
VK_A85_1	2008	VK	VK_TSR	0.6114	0.2226	22.5754	0.4188	0.2554	0.4908	0.6667	5.8000	0.6857
VK_AM_1	2008	VK	VK_REWET	0.4410	0.1680	23.9832	0.4359	0.2139	0.3933	0.6667	5.3125	0.6161
VK_AM_2	2008	VK	VK_REWET	0.4735	0.1773	24.3478	0.4435	0.2271	0.4242	0.6429	5.2143	0.6020
VK_AM_3	2008	VK	VK_REWET	0.5013	0.1891	22.5818	0.4131	0.2318	0.4353	0.6905	5.2857	0.6122
VK_AM_4	2008	VK	VK_REWET	0.3825	0.1510	23.9046	0.4441	0.2449	0.4661	0.6667	5.2500	0.6071
VK_AM_5	2008	VK	VK_REWET	0.4151	0.1615	24.4639	0.4516	0.2164	0.3991	0.7179	5.1538	0.5934
VK_BM_1	2008	VK	VK_REF	0.3443	0.1344	18.6871	0.3663	0.2758	0.5390	0.6471	5.5294	0.6471
VK_BM_10	2008	VK	VK_REF	0.4471	0.1699	16.1455	0.3260	0.2717	0.5293	0.7273	5.1818	0.5974
VK_BM_11	2008	VK	VK_REF	0.3609	0.1420	19.4540	0.3775	0.2725	0.5312	0.7143	5.3846	0.6264
VK_BM_12	2008	VK	VK_REF	0.3676	0.1436	19.4141	0.3684	0.2620	0.5065	0.6296	5.4118	0.6303
VK_BM_2	2008	VK	VK_REF	0.3565	0.1394	20.2383	0.3912	0.2705	0.5265	0.6667	5.5625	0.6518
VK_BM_3	2008	VK	VK_REF	0.3635	0.1414	19.7936	0.3740	0.2648	0.5131	0.6667	5.5000	0.6429
VK_BM_4	2008	VK	VK_REF	0.3872	0.1497	18.4720	0.3610	0.2941	0.5819	0.6429	5.5385	0.6484
VK_BM_5	2008	VK	VK_REF	0.3044	0.1218	20.2420	0.3794	0.2709	0.5274	0.6667	5.1875	0.5982
VK_BM_6	2008	VK	VK_REF	0.3162	0.1285	18.7284	0.3652	0.2810	0.5511	0.6667	5.5455	0.6494
VK_BM_7	2008	VK	VK_REF	0.3130	0.1272	18.9877	0.3675	0.2741	0.5349	0.6889	5.4286	0.6327
VK_BM_8	2008	VK	VK_REF	0.4413	0.1669	17.9217	0.3467	0.2933	0.5802	0.6875	5.4667	0.6381
VK_BM_9	2008	VK	VK_REF	0.4239	0.1617	20.0958	0.3870	0.2691	0.5231	0.7222	5.5000	0.6429
VK_E5_1	2008	VK	VK_TSR	0.4240	0.1628	21.2550	0.3916	0.2563	0.4930	0.6471	5.3529	0.6218
VK_E5_2	2008	VK	VK_TSR	0.5140	0.1935	24.2240	0.4372	0.2291	0.4289	0.6667	5.2727	0.6104
VK_E5_3	2008	VK	VK_TSR	0.4182	0.1603	23.1849	0.4296	0.2495	0.4769	0.6905	5.0714	0.5816
VK_E5_4	2008	VK	VK_TSR	0.4146	0.1615	25.5469	0.4507	0.2185	0.4041	0.6970	4.9091	0.5584
VK_E5_5	2008	VK	VK_TSR	0.4501	0.1728	23.3043	0.4284	0.2497	0.4776	0.6667	5.3846	0.6264
VK_E85_1	2008	VK	VK_TSR	0.4150	0.1597	24.5313	0.4606	0.2681	0.5209	0.6667	5.0833	0.5833
VK_EM_1	2008	VK	VK_REWET	0.5089	0.1913	23.5801	0.4294	0.2254	0.4203	0.6970	5.2727	0.6104
VK_EM_2	2008	VK	VK_REWET	0.4735	0.1793	23.4164	0.4282	0.2383	0.4507	0.6970	5.0909	0.5844
VK_EM_3	2008	VK	VK_REWET	0.4865	0.1830	24.2561	0.4348	0.2420	0.4593	0.7083	5.2222	0.6032
VK_EM_4	2008	VK	VK_REWET	0.4558	0.1723	23.7246	0.4355	0.2301	0.4315	0.6667	5.1579	0.5940
VK_EM_5	2008	VK	VK_REWET	0.4806	0.1822	23.5722	0.4341	0.2387	0.4516	0.7143	5.1429	0.5918
VK_G10_1	2008	VK	VK_TSR	0.4725	0.1794	23.2149	0.4186	0.2411	0.4573	0.6852	5.1667	0.5952
VK_G10_2	2008	VK	VK_TSR	0.4343	0.1669	21.7165	0.4086	0.2548	0.4896	0.6667	5.2500	0.6071
VK_G10_3	2008	VK	VK_TSR	0.4572	0.1731	23.6648	0.4328	0.2406	0.4560	0.6491	5.1053	0.5865
VK_G10_4	2008	VK	VK_TSR	0.4286	0.1648	22.3100	0.4167	0.2542	0.4882	0.7111	5.2000	0.6000
VK_G10_5	2008	VK	VK_TSR	0.4428	0.1708	24.7270	0.4561	0.2182	0.4034	0.6444	5.0667	0.5810
VK_G5_1	2008	VK	VK_TSR	0.5021	0.1844	25.7790	0.4658	0.2292	0.4293	0.7593	5.0000	0.5714
VK_G5_2	2008	VK	VK_TSR	0.4518	0.1724	23.1032	0.4250	0.2326	0.4373	0.6875	5.0625	0.5804
VK_G5_3	2008	VK	VK_TSR	0.4050	0.1567	26.0480	0.4737	0.2309	0.4333	0.7333	5.0667	0.5810
VK_G5_4	2008	VK	VK_TSR	0.4302	0.1648	24.9437	0.4535	0.2362	0.4457	0.6458	4.9375	0.5625
VK_G5_5	2008	VK	VK_TSR	0.4575	0.1750	23.3593	0.4262	0.2269	0.4237	0.6923	5.1538	0.5934
VK_G85_1	2008	VK	VK_TSR	0.4286	0.1648	24.0690	0.4429	0.2419	0.4591	0.6833	5.0500	0.5786
VK_GM_1	2008	VK	VK_BEFORE	0.4526	0.1748	25.3843	0.4597	0.2280	0.4264	0.7083	5.0000	0.5714
VK_GM_2	2008	VK	VK_BEFORE	0.4291	0.1668	27.2451	0.4949	0.2318	0.4353	0.6667	4.7778	0.5397
VK_GM_3	2008	VK	VK_BEFORE	0.3921	0.1534	24.8264	0.4575	0.2136	0.3925	0.6667	4.9091	0.5584
VK_GM_4	2008	VK	VK_BEFORE	0.3886	0.1519	27.2663	0.4955	0.2235	0.4159	0.7083	4.6250	0.5179

VK_GM_5	2008	VK	VK_BEFORE	0.4432	0.1695	25.4370	0.4651	0.2317	0.4351	0.7222	5.0000	0.5714
VP_ACM_99	1999	VPA	VPA_BEFOR	0.4875	0.1882	24.8700	0.4711	0.2324	0.4368	0.3333	7.0000	0.8571
VP_AP_02	2002	VPA	VPA_TSR	0.4618	0.1730	19.1068	0.3574	0.2765	0.5405	0.5714	4.7143	0.5306
VP_BCM_99	1999	VPB	VPB_BEFOR	0.5656	0.2036	22.2247	0.4293	0.2728	0.5318	0.6364	5.4545	0.6364
VP_BP_02	2002	VPB	VPB_TSR	0.6044	0.2170	18.0680	0.3634	0.3034	0.6040	0.7083	5.1250	0.5893
VP_CCM_99	1999	VPC	VPC_BEFOR	0.5940	0.2114	19.0727	0.3103	0.2829	0.5555	0.7333	6.0000	0.7143
VP_CP_02	2002	VPC	VPC_TSR	0.4993	0.1822	19.3486	0.3611	0.2639	0.5109	0.6667	5.6429	0.6633
VP_DCM_99	1999	VPD	VPD_BEFOR	0.6361	0.2281	19.2788	0.3761	0.2924	0.5780	0.7037	5.5556	0.6508
VP_DP_02	2002	VPD	VPD_TSR	0.6683	0.2332	18.0290	0.3462	0.2598	0.5012	0.7879	6.0909	0.7273
VP_FCM_99	1999	VPF	VPF_BEFOR	0.4496	0.1714	21.7793	0.4020	0.2468	0.4706	0.7083	5.5625	0.6518
VP_FP_02	2002	VPF	VPF_TSR	0.4968	0.1831	16.4691	0.3306	0.2534	0.4861	0.7778	5.5455	0.6494
VP_GCM_99	1999	VPG	VPG_BEFOR	0.5260	0.1925	21.5920	0.4118	0.3492	0.7116	0.8333	5.5000	0.6429
VP_GP_02	2002	VPG	VPG_TSR	0.4303	0.1628	25.0283	0.4536	0.2692	0.5233	0.6667	5.3333	0.6190
ZB_B3_1992	1992	ZB	ZB_BEFORE	0.5516	0.2028	20.8859	0.3983	0.2498	0.4778	0.6667	5.7778	0.6825
ZB_B3_1998	1998	ZB	ZB_REWET	0.5573	0.2044	22.2878	0.4143	0.2365	0.4465	0.6481	5.7222	0.6746
ZB_B4_1992	1992	ZB	ZB_BEFORE	0.4935	0.1846	23.8406	0.4393	0.2255	0.4206	0.6500	5.6000	0.6571
ZB_B4_1998	1998	ZB	ZB_REWET	0.5060	0.1880	24.1594	0.4479	0.2174	0.4015	0.6812	5.3913	0.6273
ZB_B5_1992	1992	ZB	ZB_BEFORE	0.5666	0.2051	22.1311	0.4157	0.2515	0.4819	0.6296	5.7222	0.6746
ZB_B5_1998	1998	ZB	ZB_REWET	0.5353	0.1969	23.0715	0.4294	0.2340	0.4406	0.6471	5.7059	0.6723
ZB_B6_1992	1992	ZB	ZB_BEFORE	0.4626	0.1757	23.5883	0.4416	0.2319	0.4357	0.6667	5.6190	0.6599
ZB_B6_1998	1998	ZB	ZB_REWET	0.5063	0.1883	22.0325	0.4209	0.2272	0.4246	0.6667	5.4500	0.6357
ZB_B7_1992	1992	ZB	ZB_BEFORE	0.5138	0.1888	25.2647	0.4563	0.2141	0.3937	0.6889	5.6875	0.6696
ZB_B7_1998	1998	ZB	ZB_REWET	0.6453	0.2279	24.0303	0.4357	0.2321	0.4360	0.6667	5.9231	0.7033
ZB_B8_1992	1992	ZB	ZB_BEFORE	0.5282	0.1936	21.7432	0.3994	0.2214	0.4109	0.6250	5.6250	0.6607
ZB_B8_1998	1998	ZB	ZB_REWET	0.5824	0.2111	19.7128	0.3768	0.2371	0.4478	0.6667	5.7273	0.6753
ZB_C1_1988	1988	ZB	ZB_BEFORE	0.5510	0.2011	21.7836	0.4091	0.2266	0.4230	0.7000	5.5500	0.6500
ZB_C1_1999	1999	ZB	ZB_REWET	0.5069	0.1877	24.0342	0.4449	0.2239	0.4167	0.7051	5.2963	0.6138
ZB_C2_1988	1988	ZB	ZB_BEFORE	0.6459	0.2297	22.6767	0.4201	0.2080	0.3793	0.6667	5.6667	0.6667
ZB_C2_2006	2006	ZB	ZB_REWET	0.5067	0.1868	24.4632	0.4514	0.2222	0.4128	0.6957	5.3333	0.6190
ZB_C6_1992	1992	ZB	ZB_BEFORE	0.4971	0.1846	25.3106	0.4631	0.1995	0.3593	0.7193	5.4211	0.6316
ZB_C6_1999	1999	ZB	ZB_REWET	0.4585	0.1724	24.8864	0.4571	0.2112	0.3870	0.7333	5.3077	0.6154
ZB_C9_1988	1988	ZB	ZB_BEFORE	0.5749	0.2107	19.4794	0.3847	0.2185	0.4040	0.7273	5.4545	0.6364
ZB_C9_2006	2006	ZB	ZB_REWET	0.5117	0.1899	25.4205	0.4580	0.2280	0.4265	0.7000	5.4500	0.6357

6 w	6 w.s	7 nit	8 pho	9 h_0 non hu	9 h_1 humm	10 n_0 no N	10 n_1 N fixi	11 MStatusC	12 MStatusN	13 MFlexi_1	14 sm	14 sm.s
9.0417	0.7042	18.6246	1.9073	0.9600	0.0400	1.0000	0.0000	0.1600	0.2400	0.6000	0.8864	0.0901
9.2778	0.7278	18.7011	1.9172	0.8889	0.1111	1.0000	0.0000	0.0556	0.2222	0.6667	0.7904	0.0967
9.3077	0.7308	23.2182	2.5639	1.0000	0.0000	0.9231	0.0000	0.0769	0.2308	0.6154	0.8709	0.0972
8.6923	0.6692	20.3852	2.2141	0.9286	0.0714	1.0000	0.0000	0.1429	0.0714	0.7857	0.5965	0.0790
8.7619	0.6762	20.8833	2.2579	0.9524	0.0476	1.0000	0.0000	0.1429	0.1429	0.7143	0.7713	0.0946
9.6667	0.7667	19.8865	1.6740	0.9167	0.0833	1.0000	0.0000	0.0000	0.3333	0.5833	0.8750	0.1018
8.9412	0.6941	20.0817	1.8515	0.9412	0.0588	1.0000	0.0000	0.0588	0.2353	0.7059	0.6851	0.0823
8.9048	0.6905	18.9911	1.5183	0.9545	0.0455	1.0000	0.0000	0.1818	0.1818	0.5909	1.0605	0.1005
8.9412	0.6941	18.2990	1.5258	0.8824	0.1176	1.0000	0.0000	0.1765	0.1176	0.6471	1.3146	0.1272
8.8571	0.6857	20.4879	2.0241	0.9333	0.0667	1.0000	0.0000	0.2000	0.2000	0.6000	0.6440	0.0785
8.7143	0.6714	20.5374	2.0915	0.8571	0.1429	1.0000	0.0000	0.2143	0.1429	0.6429	0.6369	0.0760
9.1000	0.7100	19.2529	1.9023	0.9000	0.1000	1.0000	0.0000	0.1000	0.2000	0.7000	1.1174	0.1080
9.0000	0.7000	17.4014	1.6720	0.9310	0.0690	1.0000	0.0000	0.1724	0.1724	0.6207	0.7270	0.0868
8.2917	0.6292	19.9686	1.5557	0.9630	0.0370	0.8889	0.0000	0.2593	0.2222	0.4815	0.7029	0.0879
8.6111	0.6611	17.3337	1.2044	0.9474	0.0526	0.8947	0.0000	0.2105	0.3158	0.4211	0.6154	0.0770
9.0476	0.7048	18.7442	1.6990	0.9048	0.0952	1.0000	0.0000	0.0476	0.2857	0.6667	0.7071	0.0864
8.5185	0.6519	18.2053	1.3732	0.9375	0.0625	0.9375	0.0000	0.1563	0.1875	0.6250	0.6269	0.0800
8.9259	0.6926	19.4714	1.6104	0.9310	0.0690	0.8966	0.0000	0.1034	0.2759	0.5517	0.7332	0.0902
8.8947	0.6895	21.5669	1.6374	0.9500	0.0500	0.8000	0.0000	0.1000	0.3000	0.5000	0.6198	0.0792
8.8095	0.6810	20.2217	1.9312	1.0000	0.0000	1.0000	0.0000	0.0455	0.2273	0.7273	1.4016	0.1022
8.8500	0.6850	20.9207	2.1531	1.0000	0.0000	1.0000	0.0000	0.0476	0.2381	0.7143	1.8697	0.1288
8.4000	0.6400	18.5911	1.4862	0.9545	0.0455	0.9545	0.0000	0.1818	0.2273	0.5909	0.7288	0.0893
8.8421	0.6842	18.5661	1.3928	0.9524	0.0476	0.9048	0.0000	0.1429	0.2381	0.5714	0.7033	0.0878
8.6774	0.6677	19.1229	1.4832	1.0000	0.0000	0.9706	0.0000	0.1176	0.2353	0.6471	0.6830	0.0811
8.7241	0.6724	18.7580	1.4578	0.9032	0.0968	0.9355	0.0000	0.1290	0.2258	0.6452	0.7413	0.0778
9.0476	0.7048	20.2600	1.6547	0.9545	0.0455	0.9545	0.0000	0.1364	0.1818	0.5909	0.4226	0.0568
8.4074	0.6407	19.6144	1.9270	0.9630	0.0370	1.0000	0.0000	0.1111	0.1481	0.7407	0.6131	0.0761
8.6364	0.6636	19.9002	1.8414	0.9394	0.0606	1.0000	0.0000	0.0909	0.2121	0.6970	0.6608	0.0833
8.4839	0.6484	19.7031	1.8810	0.9355	0.0645	1.0000	0.0000	0.0968	0.1935	0.7097	0.7475	0.0889
8.6400	0.6640	22.1241	2.0815	0.8800	0.1200	1.0000	0.0000	0.0800	0.2400	0.6800	0.7492	0.0896
9.2222	0.7222	20.9361	1.9156	0.8889	0.1111	1.0000	0.0000	0.0556	0.3333	0.6111	0.8328	0.0998
8.6765	0.6676	19.2066	1.6382	0.9706	0.0294	0.9412	0.0294	0.2059	0.2353	0.5588	0.6368	0.0764
8.6667	0.6667	20.8259	2.0341	0.9259	0.0741	1.0000	0.0000	0.1111	0.2222	0.6667	0.7933	0.0923
8.5238	0.6524	21.5581	2.2266	1.0000	0.0000	0.9524	0.0476	0.1905	0.1905	0.6190	0.6019	0.0767
8.9200	0.6920	18.0561	1.5408	0.8889	0.1111	0.9259	0.0000	0.0741	0.1852	0.6667	0.6331	0.0798
8.8696	0.6870	19.3314	1.8084	0.8696	0.1304	1.0000	0.0000	0.0870	0.2609	0.6522	1.0560	0.1030
8.5500	0.6550	16.8194	1.8847	0.9500	0.0500	1.0000	0.0000	0.2000	0.1000	0.7000	0.4707	0.0597
8.8148	0.6815	18.1151	1.6908	0.9630	0.0370	1.0000	0.0000	0.1852	0.2222	0.5926	0.6989	0.0838
8.6000	0.6600	17.3103	1.5910	0.9500	0.0500	1.0000	0.0000	0.2000	0.2500	0.5500	0.5765	0.0709
8.7308	0.6731	18.0284	1.3584	0.8889	0.1111	1.0000	0.0000	0.2222	0.1852	0.5926	0.8766	0.0902
8.7143	0.6714	15.3524	1.5776	1.0000	0.0000	1.0000	0.0000	0.2143	0.1429	0.6429	0.4422	0.0596
8.6154	0.6615	17.2422	1.7102	0.9231	0.0769	1.0000	0.0000	0.0769	0.1538	0.7692	0.4901	0.0686
8.7917	0.6792	18.3280	1.6581	0.9583	0.0417	0.9583	0.0000	0.1250	0.2500	0.6250	1.2864	0.1068
9.5333	0.7533	18.1503	1.5493	0.9333	0.0667	1.0000	0.0000	0.0667	0.0667	0.8000	1.1993	0.1106
9.0000	0.7000	18.6723	1.4209	0.8571	0.1429	1.0000	0.0000	0.1429	0.1905	0.6190	1.2323	0.1196
9.3810	0.7381	19.9720	1.7823	0.9524	0.0476	0.9524	0.0000	0.0476	0.3810	0.5714	1.2891	0.1174
8.6500	0.6650	18.3346	1.7696	0.9500	0.0500	0.9500	0.0000	0.1000	0.2000	0.7000	0.5928	0.0722
8.5484	0.6548	21.4042	1.6766	0.9677	0.0323	0.9032	0.0323	0.1935	0.2581	0.5161	0.9522	0.1007
8.8077	0.6808	17.9891	1.4288	0.8846	0.1154	1.0000	0.0000	0.1538	0.1923	0.6538	0.8204	0.0849
9.2632	0.7263	18.9913	1.7621	0.8947	0.1053	1.0000	0.0000	0.1053	0.2632	0.6316	1.0317	0.1014
8.4865	0.6486	21.4716	1.8597	0.9737	0.0263	0.9474	0.0263	0.1842	0.2632	0.5526	0.5573	0.0706
8.5000	0.6500	21.6122	2.0699	0.9412	0.0588	0.9412	0.0588	0.1765	0.2941	0.5294	0.6406	0.0796
8.6667	0.6667	19.3233	1.8155	0.9583	0.0417	0.9167	0.0000	0.0833	0.1250	0.7917	0.8632	0.0869
8.9412	0.6941	21.9213	2.1579	0.8824	0.1176	0.9412	0.0000	0.0588	0.2353	0.7059	0.6911	0.0822
8.4231	0.6423	17.7750	1.3523	0.9655	0.0345	1.0000	0.0000	0.2069	0.1379	0.6552	0.5421	0.0694
8.4516	0.6452	18.6715	1.5513	0.9697	0.0303	1.0000	0.0000	0.1515	0.1212	0.7273	0.8561	0.0918
8.2593	0.6259	21.2944	1.8307	0.9655	0.0345	0.9655	0.0345	0.1034	0.2414	0.6552	0.6960	0.0852
8.3929	0.6393	21.3566	1.9680	0.9655	0.0345	0.9655	0.0345	0.1379	0.2069	0.6552	0.7162	0.0868
8.1600	0.6160	21.0645	2.0269	0.9259	0.0741	0.9630	0.0370	0.1481	0.1111	0.7407	0.7180	0.0870
8.3810	0.6381	20.3089	1.8408	0.9545	0.0455	1.0000	0.0000	0.0909	0.1818	0.7273	0.5892	0.0738
9.7500	0.7750	23.2928	1.9739	1.0000	0.0000	1.0000	0.0000	0.0000	0.2500	0.7500	0.3187	0.0477
10.5000	0.8500	21.1250	1.4000	1.0000	0.0000	1.0000	0.0000	0.0000	0.7500	0.2500	0.6125	0.0801
9.8333	0.7833	22.3571	1.7461	1.0000	0.0000	1.0000	0.0000	0.0000	0.5000	0.5000	0.7938	0.1022
10.0000	0.8000	22.1928	1.4500	1.0000	0.0000	1.0000	0.0000	0.0000	0.7500	0.2500	0.8950	0.1175
10.0000	0.8000	18.9667	1.4000	1.0000	0.0000	1.0000	0.0000	0.0000	0.6667	0.3333	0.6125	0.0801
11.0000	0.9000	24.5000	2.2000	1.0000	0.0000	1.0000	0.0000	0.0000	0.6667	0.3333	0.1350	0.0235
11.0000	0.9000	24.5000	2.2000	1.0000	0.0000	1.0000	0.0000	0.0000	0.6667	0.3333	0.1350	0.0235
7.2500	0.5250	25.6583	2.4648	0.9286	0.0714	0.9286	0.0000	0.3571	0.0000	0.6429	1.6575	0.1335
6.9231	0.4923	23.6329	2.5281	0.8571	0.1429	0.9286	0.0000	0.3571	0.0000	0.6429	1.4941	0.1141
6.6250	0.4625	24.0021	2.6600	1.0000	0.0000	1.0000	0.0000	0.3333	0.0000	0.6667	0.9785	0.1117
6.4118	0.4412	25.7020	2.5603	0.9444	0.0556	0.8889	0.0000	0.3333	0.1111	0.5556	0.9503	0.1085
6.5556	0.4556	24.5085	2.2798	0.9500	0.0500	0.9000	0.0000	0.2000	0.2000	0.6000	1.5740	0.1372
7.4545	0.5455	23.4914	2.0486	0.9167	0.0833	1.0000	0.0000	0.1667	0.0833	0.7500	1.1973	0.1100
7.0769	0.5077	24.7462	2.4648	0.9333	0.0667	0.9333	0.0000	0.3333	0.0000	0.6667	1.5555	0.1261
10.0000	0.8000	19.7213	1.4571	1.0000	0.0000	1.0000	0.0000	0.0000	0.3333	0.5556	0.3974	0.0533
9.0909	0.7091	18.2177	1.4525	0.9091	0.0909	1.0000	0.0000	0.0909	0.1818	0.7273	0.3772	0.0522

9.0833	0.7083	17.6620	1.7960	1.0000	0.0000	1.0000	0.0000	0.0833	0.4167	0.5000	0.3296	0.0460
9.6000	0.7600	13.3667	1.0839	1.0000	0.0000	1.0000	0.0000	0.0000	0.6000	0.4000	0.2763	0.0410
8.4706	0.6471	19.6259	1.7878	0.8824	0.1176	0.9412	0.0000	0.1176	0.1765	0.7059	0.3606	0.0463
8.5263	0.6526	20.3559	1.7272	0.8947	0.1053	0.9474	0.0000	0.1579	0.2105	0.6316	0.5259	0.0673
8.9444	0.6944	17.7100	1.8465	0.8889	0.1111	0.9444	0.0000	0.1111	0.2222	0.6667	0.5044	0.0653
8.5714	0.6571	17.4952	1.9273	0.7857	0.2143	0.9286	0.0000	0.1429	0.2143	0.6429	0.5400	0.0627
8.9000	0.6900	17.7405	1.7335	1.0000	0.0000	0.9000	0.0000	0.2000	0.2000	0.6000	0.4463	0.0529
8.6667	0.6667	19.2001	1.8391	1.0000	0.0000	0.8667	0.0000	0.2000	0.1333	0.6667	0.4632	0.0587
8.5556	0.6556	18.9416	1.8720	0.8333	0.1667	0.9444	0.0000	0.1111	0.2222	0.6667	0.5343	0.0671
8.6000	0.6600	19.8207	1.6951	0.8500	0.1500	0.9500	0.0000	0.1500	0.2500	0.6000	0.5227	0.0653
8.7273	0.6727	17.4087	1.6733	0.7273	0.2727	0.9091	0.0000	0.0909	0.2727	0.6364	0.5887	0.0698
9.6667	0.7667	20.3717	1.4911	1.0000	0.0000	0.8889	0.0000	0.0000	0.3333	0.5556	0.5355	0.0703
9.2500	0.7250	18.1220	1.4911	1.0000	0.0000	1.0000	0.0000	0.0000	0.3750	0.6250	0.3755	0.0514
9.1818	0.7182	18.7927	1.8323	1.0000	0.0000	0.9091	0.0000	0.0909	0.3636	0.5455	0.5603	0.0683
9.1250	0.7125	20.3074	1.5133	1.0000	0.0000	0.8750	0.0000	0.1250	0.2500	0.6250	0.5602	0.0591
9.1000	0.7100	18.9772	1.8527	1.0000	0.0000	0.8000	0.0000	0.1000	0.3000	0.6000	0.6421	0.0772
8.5000	0.6500	18.8980	1.9800	0.8750	0.1250	0.9375	0.0000	0.0625	0.3125	0.6250	0.6725	0.0792
9.0000	0.7000	16.4180	1.7231	0.9231	0.0769	1.0000	0.0000	0.0769	0.3077	0.6154	0.3179	0.0447
9.0769	0.7077	19.0301	1.7266	0.9231	0.0769	0.9231	0.0000	0.0769	0.1538	0.7692	0.5139	0.0623
9.1000	0.7100	19.8997	1.8323	0.9000	0.1000	0.9000	0.0000	0.2000	0.2000	0.6000	0.6358	0.0723
8.0400	0.6040	20.8131	2.0282	0.9200	0.0800	0.9200	0.0400	0.0800	0.2800	0.6400	0.6987	0.0765
8.1905	0.6190	21.4178	2.4190	0.9048	0.0952	0.9048	0.0476	0.1429	0.2857	0.5714	0.7949	0.0817
7.7895	0.5789	20.4602	2.0989	0.9474	0.0526	0.8947	0.0526	0.1579	0.2632	0.5789	0.5454	0.0601
8.1071	0.6107	19.5953	1.9162	0.9286	0.0714	0.9643	0.0000	0.0714	0.3214	0.5714	0.6880	0.0749
8.7692	0.6769	17.2935	1.6740	0.6923	0.3077	0.9231	0.0000	0.0000	0.1538	0.8462	0.4694	0.0641
8.2941	0.6294	20.2844	1.9105	0.9412	0.0588	0.8824	0.0588	0.1176	0.3529	0.5294	0.7339	0.0769
8.0000	0.6000	19.4982	1.9611	0.9048	0.0952	0.9524	0.0476	0.1429	0.2857	0.5714	0.5801	0.0657
7.9091	0.5909	23.5260	2.2328	0.9091	0.0909	0.9091	0.0455	0.1364	0.1818	0.6818	0.8360	0.0915
7.9167	0.5917	22.6887	2.1434	0.9583	0.0417	0.9167	0.0417	0.0833	0.2500	0.6667	0.7435	0.0850
8.3913	0.6391	20.1587	1.9233	0.8696	0.1304	0.9565	0.0000	0.0435	0.2609	0.6957	0.6066	0.0708
9.1538	0.7154	18.4718	1.3259	0.8462	0.1538	0.9231	0.0000	0.1538	0.1538	0.6923	0.6732	0.0824
9.0000	0.7000	18.2077	2.0091	0.8889	0.1111	0.8889	0.0000	0.0000	0.0000	1.0000	0.4992	0.0679
8.9444	0.6944	20.5948	1.6397	0.8889	0.1111	0.9444	0.0000	0.2222	0.1667	0.5556	0.4626	0.0577
9.3529	0.7353	19.1048	1.5904	0.8824	0.1176	1.0000	0.0000	0.0588	0.1765	0.7059	0.4450	0.0625
8.7000	0.6700	18.5747	1.9517	0.8000	0.2000	0.9000	0.0000	0.2000	0.1000	0.7000	0.7118	0.0831
9.1000	0.7100	14.7192	1.4017	0.9000	0.1000	1.0000	0.0000	0.0000	0.3000	0.7000	0.4757	0.0656
8.7273	0.6727	18.4727	2.0122	0.8182	0.1818	1.0000	0.0000	0.0909	0.0000	0.9091	0.3708	0.0513
8.5714	0.6571	17.1541	2.0091	0.8571	0.1429	1.0000	0.0000	0.0714	0.2143	0.7143	0.5176	0.0656
8.3529	0.6353	21.2028	2.1589	0.9412	0.0588	0.9412	0.0588	0.1176	0.1176	0.7647	0.5859	0.0757
7.7647	0.5765	23.2257	2.0786	0.9500	0.0500	0.9500	0.0500	0.1500	0.2500	0.6000	0.5865	0.0762
8.1250	0.6125	19.1374	2.1885	0.9375	0.0625	0.9375	0.0625	0.1875	0.1250	0.6875	0.6616	0.0841
8.0870	0.6087	20.6054	2.1739	0.8261	0.1739	0.9565	0.0435	0.1304	0.1739	0.6957	0.5175	0.0690
8.3846	0.6385	18.6252	2.1144	0.9231	0.0769	0.9231	0.0769	0.1538	0.3077	0.5385	0.5621	0.0685
7.6667	0.5667	22.5115	2.0989	0.9167	0.0833	0.9167	0.0417	0.1667	0.1667	0.6667	0.7636	0.0924
8.1579	0.6158	18.2316	2.0083	0.9500	0.0500	1.0000	0.0000	0.1000	0.1500	0.7500	0.5330	0.0684
8.3125	0.6313	18.7302	1.8175	0.9375	0.0625	0.9375	0.0625	0.0625	0.3125	0.6250	0.5506	0.0707
8.8750	0.6875	20.3624	1.9125	0.9375	0.0625	0.8750	0.0625	0.1875	0.2500	0.5625	0.5229	0.0632
8.7143	0.6714	19.3376	2.2512	0.9286	0.0714	1.0000	0.0000	0.0714	0.1429	0.7857	0.6772	0.0852
8.1818	0.6182	20.8968	2.2946	0.9091	0.0909	0.9091	0.0000	0.1818	0.1818	0.6364	0.7813	0.0883
8.5833	0.6583	19.9427	2.0794	0.7500	0.2500	0.9167	0.0000	0.0833	0.0833	0.8333	0.5566	0.0698
8.6000	0.6600	18.4220	1.7317	0.8000	0.2000	1.0000	0.0000	0.0000	0.2000	0.8000	0.5585	0.0763
8.7000	0.6700	18.2531	2.0986	0.9000	0.1000	1.0000	0.0000	0.2000	0.2000	0.6000	0.4856	0.0674
8.7500	0.6750	19.7236	2.0241	0.8750	0.1250	1.0000	0.0000	0.0625	0.1250	0.8125	0.3806	0.0545
8.5333	0.6533	23.8795	1.8368	0.9333	0.0667	0.9333	0.0000	0.0667	0.2667	0.6667	0.9280	0.1005
9.0000	0.7000	17.5182	1.8137	0.9286	0.0714	1.0000	0.0000	0.0714	0.3571	0.5714	0.4252	0.0585
8.4000	0.6400	20.6009	2.0428	0.9000	0.1000	0.9000	0.0500	0.1500	0.2500	0.6000	0.5739	0.0705
8.5294	0.6529	18.7622	2.0525	0.8235	0.1765	1.0000	0.0000	0.0588	0.2353	0.7059	0.4886	0.0633
8.3529	0.6353	21.2043	2.0236	0.8824	0.1176	0.8824	0.0000	0.0588	0.1765	0.7647	0.8617	0.0896
9.8462	0.7846	21.0963	1.4920	0.9231	0.0769	0.9231	0.0000	0.0000	0.3077	0.6154	0.6436	0.0787
9.3846	0.7385	21.4911	1.4825	1.0000	0.0000	0.9231	0.0000	0.1538	0.3077	0.4615	0.4076	0.0561
9.0909	0.7091	19.5810	1.5231	0.9091	0.0909	1.0000	0.0000	0.0909	0.2727	0.6364	0.3918	0.0533
8.7778	0.6778	20.9951	1.3473	0.8889	0.1111	0.8889	0.0000	0.2222	0.2222	0.5556	0.5069	0.0558
6.4286	0.4429	24.9580	2.4211	1.0000	0.0000	0.9375	0.0000	0.2813	0.1250	0.5938	0.8041	0.0892
6.7692	0.4769	22.5236	2.3608	0.9375	0.0625	0.9375	0.0625	0.2500	0.0625	0.6875	0.5736	0.0739
8.7692	0.6769	17.2336	1.9156	0.9231	0.0769	1.0000	0.0000	0.0769	0.2308	0.6923	0.6873	0.0807
6.3750	0.4375	22.2301	1.9818	1.0000	0.0000	0.9444	0.0556	0.3889	0.1667	0.4444	0.8227	0.0914
6.5000	0.4500	22.8107	2.2217	0.9444	0.0556	0.8889	0.0556	0.4444	0.0556	0.4444	0.7916	0.0853
6.7917	0.4792	24.0710	2.1883	0.9630	0.0370	0.9259	0.0000	0.2593	0.1852	0.5185	1.0147	0.1025
6.1905	0.4190	24.8774	2.2890	0.9583	0.0417	0.9167	0.0417	0.2917	0.1250	0.5833	0.7451	0.0848
6.4375	0.4438	23.5751	2.0123	1.0000	0.0000	0.8947	0.1053	0.4737	0.0526	0.4737	0.8836	0.0908
6.3000	0.4300	25.9433	2.4009	1.0000	0.0000	0.9118	0.0294	0.2941	0.1176	0.5882	0.9627	0.0974
5.9524	0.3952	25.3872	2.4464	1.0000	0.0000	0.9167	0.0417	0.3333	0.1250	0.5417	0.7284	0.0828
6.5000	0.4500	22.1239	2.2508	0.9444	0.0556	1.0000	0.0000	0.3333	0.0556	0.6111	0.9089	0.0978
7.1667	0.5167	20.7420	1.8505	0.9286	0.0714	0.9286	0.0714	0.4286	0.1429	0.4286	0.5498	0.0722
6.2667	0.4267	23.1762	2.4530	1.0000	0.0000	0.9412	0.0588	0.3529	0.1176	0.5294	0.7291	0.0798
8.2353	0.6235	20.0092	2.1282	0.9412	0.0588	1.0000	0.0000	0.1176	0.2353	0.6471	0.8640	0.0965
7.5789	0.5579	22.9819	2.0493	1.0000	0.0000	0.9500	0.0500	0.2500	0.1000	0.6000	0.7489	0.0910

7.5000	0.5500	21.5282	1.9806	0.9091	0.0909	0.9545	0.0455	0.1818	0.0909	0.7273	0.6498	0.0820
8.4118	0.6412	20.8906	1.7740	1.0000	0.0000	0.9412	0.0588	0.1765	0.1765	0.6471	1.0274	0.0987
8.6154	0.6615	18.7109	1.8289	0.9231	0.0769	1.0000	0.0000	0.0769	0.3077	0.6154	0.8687	0.0984
8.4375	0.6438	19.7577	1.7756	1.0000	0.0000	0.9375	0.0000	0.0625	0.1875	0.7500	1.0752	0.1004
7.9474	0.5947	20.4027	1.9806	0.9048	0.0952	0.9524	0.0476	0.1429	0.1429	0.7143	0.6994	0.0888
7.3810	0.5381	21.5353	1.9522	1.0000	0.0000	0.9600	0.0400	0.1200	0.1200	0.7600	0.7156	0.0888
7.9500	0.5950	21.5105	1.9080	0.9500	0.0500	0.9000	0.1000	0.1500	0.2000	0.6500	0.8269	0.0981
8.2222	0.6222	21.1403	1.7269	0.9444	0.0556	0.9444	0.0556	0.1667	0.1667	0.6667	0.8594	0.0969
7.8824	0.5882	21.9609	2.0474	0.8824	0.1176	0.9412	0.0588	0.2353	0.0588	0.7059	0.7828	0.0954
9.0000	0.7000	16.4497	1.6902	0.8750	0.1250	0.8750	0.0000	0.2500	0.1250	0.6250	0.8353	0.0965
9.5000	0.7500	18.6505	1.8570	1.0000	0.0000	1.0000	0.0000	0.0000	0.3333	0.6667	0.9693	0.1036
8.3333	0.6333	20.1437	2.0013	0.9444	0.0556	0.9444	0.0556	0.1111	0.2222	0.6667	0.6306	0.0802
7.8125	0.5813	20.5331	1.9127	0.9375	0.0625	0.9375	0.0625	0.1250	0.1875	0.6875	0.7607	0.0865
8.9286	0.6929	19.0526	2.1475	1.0000	0.0000	1.0000	0.0000	0.1429	0.2143	0.6429	0.6843	0.0847
8.2632	0.6263	19.2065	2.0174	0.9000	0.1000	0.9500	0.0500	0.1000	0.2000	0.7000	0.6898	0.0879
8.0435	0.6043	21.0537	1.9174	0.9565	0.0435	0.9565	0.0435	0.2174	0.1304	0.6522	0.5937	0.0770
8.6000	0.6600	18.8209	1.7274	0.8000	0.2000	0.9000	0.1000	0.2000	0.4000	0.4000	0.3135	0.0451
7.0968	0.5097	22.7430	1.8230	0.9429	0.0571	0.9143	0.0857	0.2286	0.0857	0.6571	1.2185	0.1074
8.0000	0.6000	20.3511	2.1119	0.9524	0.0476	0.9524	0.0476	0.0952	0.1429	0.7619	0.6370	0.0782
7.3913	0.5391	22.0462	1.8842	0.9630	0.0370	0.8889	0.0741	0.2222	0.1111	0.6296	1.2585	0.1045
8.1111	0.6111	23.3449	2.6004	1.0000	0.0000	0.8889	0.1111	0.1111	0.1111	0.7778	0.9317	0.1107
9.0000	0.7000	22.5760	2.8766	1.0000	0.0000	1.0000	0.0000	0.1250	0.1250	0.7500	0.9030	0.1070
5.8696	0.3870	26.1211	2.3710	1.0000	0.0000	0.7857	0.1071	0.5000	0.0357	0.4286	0.6228	0.0764
5.1273	0.3127	23.3007	2.0909	0.9296	0.0704	0.7183	0.0845	0.4366	0.0282	0.4930	1.7282	0.1316
5.9231	0.3923	25.3435	2.4588	0.9677	0.0323	0.8065	0.0645	0.4194	0.0645	0.4839	0.8070	0.0886
5.4400	0.3440	23.1892	2.0664	0.9194	0.0806	0.7903	0.0806	0.3871	0.0323	0.5484	1.5580	0.1261
7.3913	0.5391	19.9985	1.6925	0.9130	0.0870	0.9565	0.0000	0.2174	0.0000	0.6957	0.6770	0.0819
6.8000	0.4800	23.0974	2.1621	0.9565	0.0435	0.9130	0.0435	0.3478	0.0435	0.5652	1.3769	0.1164
6.2143	0.4214	23.8876	2.0303	0.9444	0.0556	0.9444	0.0000	0.1111	0.1111	0.7778	1.6960	0.1359
7.8000	0.5800	19.4421	1.6514	0.9500	0.0500	0.9500	0.0000	0.1500	0.0500	0.7500	0.6888	0.0825
7.6111	0.5611	19.3369	1.7109	0.9048	0.0952	1.0000	0.0000	0.1905	0.0476	0.7619	0.4761	0.0590
8.2308	0.6231	20.3098	1.6431	0.9231	0.0769	1.0000	0.0000	0.1538	0.1538	0.6923	0.6065	0.0739
8.7059	0.6706	19.9569	1.4880	0.9444	0.0556	1.0000	0.0000	0.0000	0.1667	0.8333	0.4115	0.0562
5.7500	0.3750	26.4061	2.7835	1.0000	0.0000	0.6800	0.0000	0.2800	0.2400	0.4800	2.7358	0.1473
5.5000	0.3500	30.3854	3.1100	1.0000	0.0000	0.7083	0.0417	0.3333	0.1250	0.5417	2.9879	0.1685
6.6429	0.4643	21.5827	1.6948	0.9355	0.0645	0.8065	0.0645	0.3871	0.0323	0.5161	1.4083	0.1091
6.8519	0.4852	24.1092	2.0318	0.9333	0.0667	0.8000	0.0667	0.4667	0.0333	0.4667	1.9799	0.1390
7.5385	0.5538	21.2554	1.8525	0.8846	0.1154	0.8462	0.0385	0.3077	0.0385	0.5769	3.4568	0.1421
6.6538	0.4654	23.5135	2.1246	1.0000	0.0000	0.8387	0.0645	0.4839	0.0323	0.4194	1.8214	0.1448
7.8000	0.5800	19.8891	1.6839	0.9048	0.0952	0.9524	0.0476	0.1905	0.1905	0.5238	1.8240	0.1115
7.6522	0.5652	21.6729	1.5218	0.8750	0.1250	0.8750	0.0833	0.2083	0.1250	0.5417	4.4333	0.1790
7.4783	0.5478	23.1996	2.1634	0.9583	0.0417	0.8750	0.0833	0.1667	0.0833	0.6667	4.2911	0.1682
5.3889	0.3389	22.0010	1.7274	0.8636	0.1364	0.7727	0.1364	0.5455	0.0000	0.4545	1.5630	0.1467
6.1500	0.4150	21.4622	1.7317	0.8571	0.1429	0.9048	0.0000	0.3333	0.0476	0.5714	1.2617	0.1198
7.2917	0.5292	21.0885	1.8277	0.9286	0.0714	0.8929	0.0714	0.3571	0.0714	0.4643	0.8966	0.1023
6.6471	0.4647	21.7371	1.5395	0.9500	0.0500	0.8000	0.1000	0.4000	0.0500	0.4500	0.9133	0.0998
8.3750	0.6375	18.7094	1.3680	0.7778	0.2222	0.8889	0.0000	0.3333	0.0000	0.6667	0.8214	0.0958
8.7778	0.6778	17.7111	1.5408	0.8889	0.1111	0.6667	0.0000	0.1111	0.2222	0.6667	0.7767	0.0957
7.2632	0.5263	19.0880	1.6020	0.8500	0.1500	0.9500	0.0000	0.2000	0.1500	0.5500	1.2375	0.1178
8.2000	0.6200	20.8821	1.4649	0.9000	0.1000	0.9000	0.0000	0.1000	0.1000	0.8000	3.1898	0.1286
8.5238	0.6524	20.3960	1.8479	0.9545	0.0455	1.0000	0.0000	0.1364	0.1364	0.7273	0.4985	0.0634
8.4167	0.6417	21.1832	1.6806	0.9167	0.0833	1.0000	0.0000	0.0833	0.2500	0.6667	0.5337	0.0684
8.7647	0.6765	20.9388	1.5633	0.8889	0.1111	0.9444	0.0000	0.1111	0.1111	0.7778	0.6857	0.0809
8.7143	0.6714	19.2162	1.3938	0.8571	0.1429	0.9286	0.0000	0.0714	0.0714	0.8571	0.7065	0.0832
7.5294	0.5529	20.6508	1.5899	0.8235	0.1765	0.9412	0.0000	0.1176	0.2353	0.5294	0.8447	0.1038
7.3333	0.5333	18.4780	1.5572	0.8889	0.1111	0.8519	0.0370	0.2963	0.0741	0.5556	1.4258	0.1103
7.3913	0.5391	20.4295	1.4917	0.9167	0.0833	0.9167	0.0000	0.2917	0.0417	0.5417	0.7000	0.0848
7.6250	0.5625	20.5399	1.6039	0.9259	0.0741	0.8889	0.0000	0.2593	0.0000	0.7037	0.6793	0.0812
7.8696	0.5870	21.6948	1.6531	0.9200	0.0800	0.9600	0.0000	0.2400	0.1200	0.6000	0.6793	0.0812
8.2632	0.6263	21.2512	1.4865	0.9500	0.0500	0.9500	0.0000	0.1500	0.1000	0.7500	1.0289	0.0856
8.0556	0.6056	19.5681	1.4369	0.9000	0.1000	0.9000	0.0000	0.2000	0.1000	0.7000	0.6494	0.0779
6.0714	0.4071	27.0855	2.8485	0.9333	0.0667	0.9333	0.0667	0.2000	0.0667	0.7333	0.6348	0.0802
8.3750	0.6375	23.2745	2.0566	0.9200	0.0800	0.9600	0.0000	0.0400	0.1600	0.7600	0.7108	0.0905
7.4483	0.5448	24.2732	2.0817	0.9375	0.0625	0.8438	0.0938	0.1250	0.0938	0.7188	0.7522	0.0943
7.9583	0.5958	21.0368	1.9743	0.9615	0.0385	0.9615	0.0000	0.0385	0.1923	0.7308	0.7112	0.0904
8.3333	0.6333	22.0125	1.9917	1.0000	0.0000	0.9545	0.0000	0.0455	0.2273	0.6818	0.7561	0.0949
7.1667	0.5167	25.3334	2.6328	0.9535	0.0465	0.8837	0.0465	0.3023	0.0930	0.6047	0.6071	0.0771
7.4118	0.5412	27.6765	2.6182	0.8889	0.1111	0.9444	0.0556	0.3889	0.1111	0.5000	0.7053	0.0902
7.4074	0.5407	23.2337	2.3688	0.9310	0.0690	0.9655	0.0345	0.2414	0.0690	0.6897	0.7814	0.0956
6.5000	0.4500	27.0393	2.5980	0.9474	0.0526	0.8947	0.0526	0.2105	0.1053	0.6316	0.5235	0.0682
7.5000	0.5500	23.4668	2.0750	0.8889	0.1111	0.8889	0.0741	0.1481	0.1111	0.7037	3.0577	0.1303
7.6774	0.5677	23.2893	2.0120	0.9394	0.0606	0.8788	0.0909	0.1212	0.0909	0.7576	0.7690	0.0947
7.8696	0.5870	22.5256	2.0715	0.9231	0.0769	0.9615	0.0000	0.0385	0.0769	0.8077	0.6975	0.0872
7.8261	0.5826	22.1550	2.0319	0.9615	0.0385	0.9615	0.0000	0.0385	0.1154	0.7692	0.7127	0.0907
7.6875	0.5688	27.3646	2.1873	0.8235	0.1765	0.9412	0.0588	0.2353	0.1765	0.5882	0.8400	0.0773
8.2143	0.6214	18.5404	1.3067	0.9333	0.0667	0.8667	0.0000	0.2000	0.2000	0.6000	0.8257	0.0938
8.8696	0.6870	20.5593	1.7360	0.8750	0.1250	0.9167	0.0000	0.1667	0.2500	0.5833	2.8535	0.1140

9.5556	0.7556	20.0595	1.5073	1.0000	0.0000	0.7273	0.0000	0.1818	0.0000	0.5455	0.7464	0.0787
9.4000	0.7400	19.1350	1.5025	0.8846	0.1154	0.8462	0.0000	0.1154	0.1538	0.5769	2.9183	0.1182
6.8182	0.4818	24.6616	1.9042	1.0000	0.0000	0.7826	0.1304	0.2609	0.1304	0.6087	0.9521	0.1104
5.9375	0.3938	24.3210	2.3759	0.9412	0.0588	0.8824	0.1176	0.4118	0.0000	0.5882	1.0779	0.1234
5.7619	0.3762	24.5553	2.3504	0.9545	0.0455	0.9091	0.0909	0.4545	0.0000	0.5455	0.8490	0.0995
6.7895	0.4789	24.3889	2.0298	1.0000	0.0000	0.9000	0.1000	0.2500	0.0500	0.7000	0.7913	0.0935
8.6667	0.6667	17.0559	1.6360	1.0000	0.0000	1.0000	0.0000	0.1667	0.3333	0.5000	0.6679	0.0850
9.7778	0.7778	23.2225	1.5844	1.0000	0.0000	1.0000	0.0000	0.0000	0.2222	0.6667	0.7425	0.0954
7.0833	0.5083	26.3842	1.9856	0.9583	0.0417	0.8333	0.1250	0.3750	0.0417	0.5833	0.8081	0.0951
6.3478	0.4348	24.3161	2.0677	0.9630	0.0370	0.9259	0.0741	0.4444	0.0370	0.5185	0.9934	0.1128
5.6667	0.3667	23.8899	2.2503	0.9524	0.0476	0.9524	0.0476	0.5714	0.0000	0.4286	0.8795	0.1034
6.3077	0.4308	23.1981	2.1284	0.9630	0.0370	0.9630	0.0370	0.4074	0.0370	0.5556	0.8656	0.0978
7.3500	0.5350	23.2965	2.0059	0.9000	0.1000	0.9500	0.0500	0.3000	0.1000	0.6000	0.8400	0.1008
9.0000	0.7000	18.9821	1.5266	0.9444	0.0556	1.0000	0.0000	0.1111	0.2778	0.6111	0.5888	0.0740
5.8421	0.3842	29.4388	2.2415	1.0000	0.0000	0.9545	0.0455	0.4545	0.0455	0.5000	0.9406	0.1077
8.1176	0.6118	21.2114	1.7336	1.0000	0.0000	0.9474	0.0526	0.2632	0.2632	0.4211	0.5193	0.0682
8.1304	0.6130	20.9582	1.8314	0.9600	0.0400	0.9600	0.0400	0.2000	0.1600	0.6400	0.9359	0.0923
6.5000	0.4500	27.8722	2.8888	1.0000	0.0000	1.0000	0.0000	0.0000	0.0000	1.0000	0.1313	0.0226
8.7500	0.6750	18.4369	1.6579	1.0000	0.0000	1.0000	0.0000	0.0833	0.2500	0.6667	0.6369	0.0725
6.5556	0.4556	26.6920	2.5904	0.9524	0.0476	0.9048	0.0476	0.3810	0.0476	0.5714	0.7243	0.0870
6.1250	0.4125	26.0736	2.4668	0.9643	0.0357	0.9286	0.0357	0.4286	0.0357	0.5000	0.7298	0.0883
7.0000	0.5000	25.3824	2.1698	0.8333	0.1667	0.8333	0.0000	0.2500	0.0000	0.7083	1.1858	0.1144
8.8462	0.6846	18.5589	2.0595	1.0000	0.0000	0.9231	0.0000	0.2308	0.0769	0.6923	0.6783	0.0755
9.4000	0.7400	18.4073	1.4095	1.0000	0.0000	0.8000	0.0000	0.0000	0.2000	0.8000	0.8016	0.0873
5.9000	0.3900	29.8437	2.9519	1.0000	0.0000	0.8333	0.0833	0.4167	0.0000	0.5833	0.7231	0.0882
5.7333	0.3733	27.4601	2.7635	0.9412	0.0588	0.8824	0.0588	0.5294	0.0000	0.4706	0.7949	0.0962
5.7500	0.3750	28.0337	2.6026	0.9444	0.0556	0.8889	0.0556	0.5000	0.0000	0.5000	0.6644	0.0837
5.9167	0.3917	31.8521	2.9418	1.0000	0.0000	0.9286	0.0714	0.4286	0.0000	0.5714	1.1930	0.1097
7.8235	0.5824	24.1182	1.6851	0.8235	0.1765	0.8824	0.0588	0.1765	0.2353	0.5882	0.5282	0.0680
5.8824	0.3882	28.6160	2.6113	0.9500	0.0500	0.9000	0.0500	0.4500	0.0000	0.5000	0.6892	0.0855
6.1176	0.4118	27.9739	2.3998	0.9500	0.0500	0.9000	0.0500	0.4000	0.0000	0.5500	0.7967	0.0938
6.2778	0.4278	29.1746	2.7717	0.9000	0.1000	0.7500	0.0000	0.1000	0.0000	0.8500	3.7919	0.2231
6.7778	0.4778	25.7442	2.6870	0.8462	0.1538	1.0000	0.0000	0.3846	0.0000	0.5385	0.7959	0.0917
6.4211	0.4421	25.3399	2.3356	0.9091	0.0909	0.8636	0.0909	0.3636	0.0909	0.5000	0.7943	0.0975
8.6154	0.6615	24.3393	2.4148	0.9231	0.0769	1.0000	0.0000	0.1538	0.2308	0.6154	0.7415	0.0871
9.0909	0.7091	23.7503	1.8091	0.8636	0.1364	1.0000	0.0000	0.0909	0.1818	0.6818	0.6863	0.0859
9.5000	0.7500	22.8111	1.4851	0.8000	0.2000	1.0000	0.0000	0.0000	0.5000	0.5000	0.3694	0.0512
5.2500	0.3250	27.7659	2.9859	1.0000	0.0000	1.0000	0.0000	0.2000	0.0000	0.8000	3.3136	0.2102
9.7143	0.7714	23.8095	1.9381	1.0000	0.0000	1.0000	0.0000	0.4286	0.2857	0.2857	0.5123	0.0653
8.0500	0.6050	23.5471	2.4966	0.9000	0.1000	0.8500	0.0500	0.1500	0.1000	0.7000	0.4992	0.0663
10.5000	0.8500	20.0500	1.1650	1.0000	0.0000	1.0000	0.0000	0.0000	0.7500	0.2500	0.5750	0.0737
6.3529	0.4353	24.3530	2.3410	0.9444	0.0556	0.9444	0.0556	0.5000	0.0556	0.4444	0.7247	0.0873
8.8421	0.6842	18.4890	1.4069	1.0000	0.0000	1.0000	0.0000	0.0526	0.3158	0.5789	0.3966	0.0519
8.2308	0.6231	18.2828	1.3446	0.9286	0.0714	0.8571	0.0000	0.1429	0.2143	0.6429	0.9432	0.1059
10.6667	0.8667	31.4847	3.2000	1.0000	0.0000	0.5000	0.0000	0.0000	0.6667	0.1667	2.5116	0.2323
11.7500	0.9750	32.5333	NA	1.0000	0.0000	0.5000	0.0000	0.0000	1.0000	0.0000	2.7448	0.2449
6.6429	0.4643	26.8409	2.3451	0.8667	0.1333	0.9333	0.0667	0.4000	0.0000	0.6000	0.7032	0.0819
6.3125	0.4313	25.5884	2.4666	0.8824	0.1176	0.9412	0.0588	0.4706	0.0000	0.5294	0.7113	0.0847
8.0357	0.6036	24.3078	1.9890	0.8387	0.1613	0.9677	0.0000	0.2258	0.1290	0.6452	1.1505	0.1091
8.7727	0.6773	19.9774	2.0568	0.8636	0.1364	0.9091	0.0000	0.0909	0.1818	0.7273	0.7458	0.0861
11.1667	0.9167	23.7600	1.5759	1.0000	0.0000	0.8333	0.0000	0.0000	0.5000	0.3333	1.2570	0.1324
7.4286	0.5429	27.1862	2.5867	1.0000	0.0000	0.7143	0.1429	0.2857	0.0000	0.7143	0.6411	0.0764
8.7000	0.6700	24.7659	1.9915	1.0000	0.0000	0.8000	0.0000	0.1000	0.1000	0.8000	0.7468	0.0853
9.5000	0.7500	22.2046	1.7059	1.0000	0.0000	0.8750	0.0000	0.1250	0.2500	0.6250	0.6473	0.0850
8.4286	0.6429	18.7580	1.2542	0.9333	0.0667	0.8667	0.0000	0.2000	0.1333	0.6667	0.7441	0.0834
6.4706	0.4471	28.2900	2.4961	1.0000	0.0000	0.8947	0.0526	0.4211	0.0000	0.5789	1.3838	0.1282
7.7500	0.5750	29.6975	2.8603	1.0000	0.0000	0.8000	0.0000	0.2000	0.0000	0.8000	0.9076	0.1134
7.0000	0.5000	23.4906	2.0762	0.9167	0.0833	0.8750	0.0417	0.3750	0.0000	0.6250	0.8305	0.0941
6.3125	0.4313	27.5706	2.3124	0.8333	0.1667	0.8333	0.0000	0.1667	0.0000	0.7778	2.4849	0.1972
8.0000	0.6000	20.8190	1.3915	0.4000	0.6000	1.0000	0.0000	0.4000	0.0000	0.6000	0.7540	0.1004
8.5714	0.6571	20.7876	1.8535	0.5000	0.5000	1.0000	0.0000	0.1250	0.1250	0.7500	0.4807	0.0661
8.5000	0.6500	19.3814	1.8535	0.5000	0.5000	1.0000	0.0000	0.2500	0.0000	0.7500	0.4086	0.0580
8.2222	0.6222	23.7623	1.6908	0.7273	0.2727	0.9091	0.0000	0.1818	0.1818	0.6364	0.8697	0.0823
10.1250	0.8125	27.5224	1.7532	0.8889	0.1111	0.8889	0.0000	0.1111	0.3333	0.2222	0.5283	0.0712
9.3529	0.7353	23.5350	2.1181	0.9412	0.0588	0.9412	0.0000	0.1176	0.2941	0.5294	0.7650	0.0905
8.7727	0.6773	18.3892	1.6132	0.9565	0.0435	0.9130	0.0435	0.0000	0.1304	0.7826	1.2792	0.0896
7.8696	0.5870	21.2391	1.9280	0.8750	0.1250	0.8750	0.0833	0.1250	0.1250	0.7083	3.2960	0.1276
8.5556	0.6556	20.3267	1.9815	1.0000	0.0000	1.0000	0.0000	0.0000	0.1111	0.8889	1.9758	0.1469
6.3636	0.4364	24.3768	2.2796	0.9500	0.0500	0.7250	0.1000	0.2250	0.1000	0.6000	1.8146	0.1206
6.3077	0.4308	24.9642	2.5579	1.0000	0.0000	0.8065	0.0323	0.3548	0.0000	0.6129	0.5898	0.0745
5.8696	0.3870	25.8063	2.2557	0.9821	0.0179	0.7679	0.1071	0.3214	0.0179	0.6071	1.8851	0.1302
6.2500	0.4250	23.9754	2.0111	0.9388	0.0612	0.8163	0.0816	0.2449	0.0408	0.6327	1.1726	0.1120
6.4138	0.4414	23.9151	2.1986	0.9677	0.0323	0.7419	0.0968	0.2903	0.0968	0.5806	1.1165	0.0999
6.0000	0.4000	27.1407	2.6912	1.0000	0.0000	0.7778	0.0741	0.2963	0.0000	0.7037	1.1127	0.1044
5.8286	0.3829	24.8544	2.4429	0.9756	0.0244	0.7805	0.0732	0.4146	0.0000	0.5610	1.0141	0.0985
6.2632	0.4263	24.8449	2.5479	0.9778	0.0222	0.7333	0.1111	0.3778	0.0000	0.5778	1.8374	0.1332
5.9722	0.3972	26.4181	2.4583	0.9762	0.0238	0.8571	0.0476	0.3333	0.0238	0.6429	1.0998	0.1002

6.2000	0.4200	26.3283	2.3473	1.0000	0.0000	0.7368	0.0526	0.2105	0.0000	0.7895	1.3912	0.1127
6.0667	0.4067	30.5888	3.0121	1.0000	0.0000	0.5000	0.0500	0.3500	0.0500	0.5000	3.2245	0.1489
6.5909	0.4591	23.5301	2.2750	1.0000	0.0000	0.8148	0.1111	0.3333	0.0741	0.5926	1.7366	0.1295
6.0952	0.4095	27.8992	2.8246	1.0000	0.0000	0.7600	0.1200	0.3600	0.0400	0.5600	1.0417	0.1168
6.2500	0.4250	26.1404	2.2552	0.9615	0.0385	0.8077	0.0385	0.3462	0.0769	0.5385	1.7200	0.1413
6.6774	0.4677	23.6073	1.7831	0.8919	0.1081	0.8378	0.1081	0.2432	0.0811	0.6757	2.0659	0.1413
6.5588	0.4559	26.8458	2.4456	0.9744	0.0256	0.7436	0.1282	0.3590	0.0769	0.5385	1.1309	0.1086
6.6250	0.4625	23.3883	2.5165	0.9500	0.0500	0.8500	0.0000	0.4000	0.0000	0.6000	0.7254	0.0872
6.2222	0.4222	24.6116	2.7029	0.9524	0.0476	0.8095	0.0476	0.2381	0.0476	0.6667	0.8579	0.1059
7.0667	0.5067	21.8592	2.4073	0.9444	0.0556	0.8889	0.0000	0.1667	0.0556	0.7222	0.6495	0.0837
7.0000	0.5000	22.2369	2.2260	0.8421	0.1579	1.0000	0.0000	0.1053	0.1053	0.7895	0.6509	0.0804
6.5000	0.4500	24.2392	2.3691	0.9375	0.0625	0.8750	0.0625	0.4375	0.0625	0.5000	1.4878	0.1051
6.3750	0.4375	28.4470	2.7931	0.9500	0.0500	0.9000	0.0500	0.3500	0.1000	0.5500	1.7430	0.1243
8.8571	0.6857	20.9800	1.5631	0.9333	0.0667	0.7333	0.0000	0.0667	0.2000	0.7333	0.5124	0.0657
7.5714	0.5571	19.8025	1.2963	0.8000	0.2000	0.8000	0.0000	0.2000	0.3000	0.5000	0.6137	0.0773
8.6667	0.6667	20.7707	1.5931	0.9524	0.0476	0.8095	0.0000	0.0476	0.2381	0.6667	0.5369	0.0695
8.0000	0.6000	18.5232	1.2521	0.7143	0.2857	0.8571	0.0000	0.1429	0.1429	0.7143	0.6153	0.0730
8.9000	0.6900	20.8144	1.6508	1.0000	0.0000	0.8000	0.0500	0.0500	0.2000	0.7500	0.8080	0.0770
7.9167	0.5917	22.4146	1.5089	0.9333	0.0667	0.8000	0.0000	0.1333	0.3333	0.5333	0.5076	0.0696
8.5000	0.6500	21.7179	1.6443	0.9500	0.0500	0.9000	0.0500	0.1000	0.2000	0.7000	1.0197	0.1010
7.9091	0.5909	20.6004	1.6318	0.8571	0.1429	0.8571	0.0000	0.1429	0.2857	0.5714	0.3829	0.0548
7.4091	0.5409	22.5919	2.2571	1.0000	0.0000	0.8889	0.0000	0.1481	0.1481	0.7037	0.9742	0.1103
7.7143	0.5714	23.8190	2.0494	0.9697	0.0303	0.8485	0.0303	0.1818	0.1515	0.6667	1.1025	0.1145
7.7200	0.5720	22.0655	2.0682	0.9677	0.0323	0.8710	0.0000	0.1613	0.1290	0.6774	1.0385	0.1062
7.7083	0.5708	22.2845	2.0547	0.9655	0.0345	0.8966	0.0000	0.1379	0.2069	0.6552	0.7761	0.0921
7.7273	0.5727	21.7323	2.0158	0.9630	0.0370	0.9259	0.0000	0.1852	0.1481	0.6667	0.8021	0.0942
7.7368	0.5737	21.7760	2.0573	0.9565	0.0435	0.9565	0.0000	0.1739	0.1739	0.6522	0.7952	0.0954
7.7778	0.5778	25.4753	2.3299	0.9000	0.0667	0.8000	0.0333	0.1667	0.1667	0.6000	0.3791	0.0511
7.1351	0.5135	26.2118	2.4211	0.9744	0.0256	0.7692	0.0513	0.1538	0.1538	0.6667	0.5540	0.0694
7.3056	0.5306	24.3185	2.1895	0.9474	0.0526	0.7368	0.0526	0.2368	0.1579	0.6053	0.9317	0.0763
7.3333	0.5333	24.6767	2.2753	0.9750	0.0250	0.7000	0.0750	0.2500	0.1500	0.6000	0.5246	0.0667
7.9565	0.5957	24.4103	2.2397	0.9167	0.0833	0.7083	0.0417	0.2083	0.1667	0.5833	0.4419	0.0552
8.3333	0.6333	24.1920	2.2134	0.9333	0.0667	0.8000	0.0000	0.0667	0.2667	0.6667	0.3559	0.0463
7.5128	0.5513	24.0904	2.3779	0.9524	0.0476	0.7619	0.0476	0.1667	0.1667	0.6667	0.8760	0.0736
7.2766	0.5277	26.8147	2.4662	0.9804	0.0196	0.7255	0.0392	0.2353	0.1373	0.5882	1.0748	0.0997
8.6111	0.6611	24.8760	2.1036	0.9211	0.0526	0.7895	0.0526	0.1053	0.1842	0.6579	2.5437	0.1093
7.5946	0.5595	25.4534	2.3500	0.9737	0.0263	0.7632	0.0526	0.2368	0.1316	0.6316	0.7157	0.0807
8.2000	0.6200	23.4657	1.9929	0.9500	0.0500	0.8500	0.0000	0.1000	0.1500	0.7500	1.3662	0.0917
7.4000	0.5400	25.8448	2.5524	0.9189	0.0811	0.8378	0.0270	0.2703	0.0811	0.6486	2.3664	0.1088
6.7647	0.4765	27.1865	2.8102	0.9000	0.1000	0.8000	0.1000	0.3000	0.0500	0.6000	0.5541	0.0739
7.6923	0.5692	23.6685	2.2219	0.8462	0.1538	1.0000	0.0000	0.1538	0.1538	0.6923	0.7121	0.0850
7.0571	0.5057	26.2547	2.6681	0.9730	0.0270	0.7027	0.0541	0.2973	0.1351	0.5135	0.9923	0.0805
7.0625	0.5063	25.0780	2.5646	0.9706	0.0294	0.7353	0.0588	0.2941	0.0588	0.6471	1.3004	0.1054
6.4762	0.4476	26.6743	2.6489	0.9583	0.0417	0.8333	0.0417	0.3333	0.0833	0.5417	0.6004	0.0729
7.8485	0.5848	23.4188	2.1901	0.9706	0.0294	0.6765	0.0294	0.2941	0.1765	0.5000	1.3344	0.1022
7.6538	0.5654	24.8906	2.4099	0.9643	0.0357	0.8214	0.0357	0.3214	0.1429	0.5357	1.2464	0.0965
7.6875	0.5688	22.1831	2.0209	0.9444	0.0556	0.6667	0.0000	0.1111	0.2222	0.5556	0.5355	0.0613
8.1290	0.6129	24.3422	2.1419	0.9394	0.0606	0.8788	0.0303	0.3030	0.1515	0.5455	0.9750	0.0735
8.0690	0.6069	21.4981	1.7765	0.9394	0.0606	0.7576	0.0606	0.0909	0.1818	0.6970	1.0276	0.0804
7.9231	0.5923	21.1380	1.8194	0.9286	0.0714	0.7857	0.0357	0.1071	0.2143	0.6429	0.5040	0.0638
8.6429	0.6643	19.5819	1.3131	0.8750	0.0625	0.7500	0.0000	0.0625	0.0625	0.7500	0.4027	0.0511
8.7500	0.6750	19.6072	1.1758	0.9333	0.0000	0.6667	0.0000	0.0667	0.2000	0.5333	0.4653	0.0600
7.8636	0.5864	23.2023	1.9369	0.9167	0.0833	0.8750	0.0417	0.2083	0.1250	0.6667	0.7151	0.0823
7.1379	0.5138	24.4315	2.1689	0.9375	0.0625	0.7813	0.0938	0.2188	0.0938	0.6563	0.6307	0.0783
7.5862	0.5586	23.4956	2.0086	0.9375	0.0625	0.8125	0.0938	0.1875	0.1250	0.6563	0.5943	0.0724
8.0690	0.6069	20.0747	1.8565	0.8667	0.1333	0.8667	0.0333	0.1333	0.1667	0.7000	0.9641	0.0684
7.6316	0.5632	24.2969	2.4223	0.9048	0.0952	0.8571	0.0476	0.1905	0.1429	0.6429	2.0896	0.1108
7.1351	0.5135	24.4685	2.2483	0.9762	0.0238	0.8095	0.0952	0.2381	0.1190	0.6190	1.1762	0.1048
7.8519	0.5852	24.9709	2.3571	0.9000	0.1000	0.9000	0.0000	0.1000	0.1000	0.8000	2.5529	0.1100
8.1515	0.6152	21.1957	1.8764	0.9189	0.0811	0.8919	0.0270	0.1892	0.1081	0.6757	2.5920	0.1179
7.6842	0.5684	22.0811	1.7684	0.9091	0.0909	0.7273	0.0909	0.0909	0.1364	0.6818	0.4689	0.0582
8.1667	0.6167	21.4218	1.8933	0.8929	0.1071	0.8214	0.0357	0.0714	0.1429	0.7143	2.3260	0.0901
8.1600	0.6160	23.1165	1.7605	0.9643	0.0357	0.7500	0.1071	0.1071	0.1786	0.6429	1.1496	0.0883
7.7273	0.5727	23.7062	2.0085	0.9167	0.0833	0.8056	0.1111	0.1389	0.1667	0.6667	2.5394	0.1134
7.6111	0.5611	22.3682	2.0465	0.9250	0.0750	0.8250	0.0750	0.1750	0.1750	0.6000	2.0635	0.1096
8.3750	0.6375	20.8706	2.2455	0.8235	0.1765	0.8824	0.0000	0.0000	0.1176	0.8235	3.7357	0.1250
7.5600	0.5560	24.2907	2.0778	0.9286	0.0714	0.7857	0.1071	0.1786	0.1429	0.6429	0.8287	0.0967
6.9444	0.4944	27.4402	2.4983	0.9545	0.0455	0.8182	0.1364	0.3182	0.0909	0.5455	0.9526	0.1121
7.5882	0.5588	24.3525	2.4143	0.9500	0.0500	0.9000	0.0500	0.2500	0.0000	0.7000	2.1923	0.1280
7.2895	0.5289	23.0892	2.0984	0.9545	0.0455	0.7955	0.0909	0.1818	0.1136	0.6364	0.7435	0.0900
8.0000	0.6000	23.3420	1.8761	0.9167	0.0833	0.8333	0.0833	0.1667	0.2083	0.5833	2.8140	0.1188
8.0000	0.6000	22.5469	2.1107	0.9063	0.0938	0.8125	0.0625	0.0625	0.1250	0.7500	2.3265	0.1098
7.3529	0.5353	23.4346	2.2653	0.9487	0.0513	0.8718	0.0769	0.1538	0.0513	0.7179	2.4753	0.1187
7.6410	0.5641	23.0292	2.2404	0.9286	0.0714	0.8810	0.0476	0.1905	0.1667	0.6190	1.8389	0.0954
6.6154	0.4615	30.3212	3.2607	1.0000	0.0000	1.0000	0.0000	0.2000	0.0667	0.7333	1.2216	0.1257
8.4444	0.6444	30.6005	2.9023	0.9091	0.0909	0.9091	0.0000	0.0909	0.0909	0.7273	0.5905	0.0680
6.2105	0.4211	28.3466	2.9053	1.0000	0.0000	1.0000	0.0000	0.2727	0.0455	0.6818	1.3393	0.1225

5.8235	0.3824	24.5643	2.1357	0.9048	0.0952	0.9524	0.0476	0.2857	0.0476	0.6667	1.4679	0.1337
6.5909	0.4591	24.1532	2.4125	0.9600	0.0400	0.8800	0.0000	0.1200	0.0400	0.8000	0.5558	0.0665
6.4375	0.4438	23.1175	2.1102	0.9412	0.0588	0.9412	0.0000	0.1765	0.0000	0.8235	0.6840	0.0785
6.5556	0.4556	25.0793	2.4412	0.9000	0.1000	0.9000	0.0000	0.1000	0.0000	0.9000	0.4210	0.0612
6.9545	0.4955	22.3286	2.3114	0.8636	0.1364	1.0000	0.0000	0.2727	0.0000	0.7273	0.6153	0.0742
6.2500	0.4250	24.5058	2.2393	0.8947	0.1053	0.8947	0.0526	0.2632	0.0000	0.6842	0.5957	0.0725
7.6923	0.5692	24.4383	2.0730	0.9231	0.0769	1.0000	0.0000	0.3077	0.0769	0.6154	0.5813	0.0748
6.5000	0.4500	22.9633	2.4918	0.8462	0.1538	0.9231	0.0000	0.0769	0.0000	0.9231	0.4789	0.0612
6.2222	0.4222	26.0152	2.7090	0.8889	0.1111	1.0000	0.0000	0.1111	0.0000	0.8889	0.3909	0.0479
6.8235	0.4824	26.8857	2.5564	0.9500	0.0500	0.8000	0.0500	0.1500	0.0500	0.7000	1.4435	0.1062
6.6000	0.4600	28.6555	2.8462	0.9412	0.0588	0.9412	0.0000	0.2941	0.0000	0.7059	0.7330	0.0874
10.1250	0.8125	30.9611	1.1150	1.0000	0.0000	0.8750	0.0000	0.1250	0.1250	0.6250	0.4129	0.0591
6.5385	0.4538	28.6290	2.8979	0.9333	0.0667	0.9333	0.0000	0.2000	0.0000	0.8000	0.9162	0.1021
11.4000	0.9400	36.7400	NA	1.0000	0.0000	0.8000	0.0000	0.2000	0.6000	0.2000	1.7054	0.1697
11.4000	0.9400	38.3800	5.6100	1.0000	0.0000	0.6000	0.0000	0.2000	0.6000	0.2000	3.0069	0.2299
11.4000	0.9400	38.3800	5.6100	1.0000	0.0000	0.6000	0.0000	0.2000	0.6000	0.2000	3.0069	0.2299
7.7600	0.5760	21.0080	1.7232	0.9667	0.0333	0.8667	0.0333	0.2333	0.1000	0.5667	0.5704	0.0748
9.0000	0.7000	20.2121	1.8722	0.8000	0.2000	1.0000	0.0000	0.1000	0.3000	0.5000	0.2878	0.0414
8.0500	0.6050	25.3465	2.3901	0.9545	0.0455	0.8636	0.0000	0.1818	0.0455	0.6818	0.6480	0.0767
8.1111	0.6111	22.0436	2.0181	0.9474	0.0526	0.9474	0.0000	0.1579	0.0526	0.7895	0.7238	0.0834
8.3333	0.6333	22.2905	2.1221	0.9167	0.0833	0.9167	0.0000	0.0000	0.0000	1.0000	0.7203	0.0842
8.8571	0.6857	20.1923	2.2478	0.8571	0.1429	0.8571	0.0000	0.0000	0.1429	0.8571	0.7899	0.0944
8.5625	0.6563	21.7065	2.1121	0.8750	0.1250	0.9375	0.0000	0.0625	0.0625	0.8750	0.5164	0.0667
9.0714	0.7071	21.9618	1.8270	0.8571	0.1429	0.9286	0.0000	0.1429	0.0714	0.7857	0.4214	0.0590
7.4444	0.5444	21.1267	2.0064	0.9000	0.1000	1.0000	0.0000	0.1000	0.1500	0.7500	0.5805	0.0774
6.5000	0.4500	20.2864	2.1870	0.8333	0.1667	1.0000	0.0000	0.0833	0.0000	0.9167	0.5162	0.0661
6.6667	0.4667	21.7481	2.1404	0.9000	0.1000	0.9000	0.0000	0.3000	0.1000	0.6000	0.7320	0.0809
8.0000	0.6000	24.0370	2.2415	0.8571	0.1429	0.9286	0.0000	0.1429	0.0000	0.7857	0.4698	0.0603
7.6154	0.5615	22.3269	1.5909	0.9333	0.0667	0.8667	0.0667	0.2667	0.1000	0.5667	1.1274	0.1011
7.6000	0.5600	19.5142	1.8592	0.9412	0.0588	0.8824	0.0588	0.2353	0.1765	0.5882	0.6901	0.0834
8.5000	0.6500	21.8417	2.0021	0.9412	0.0588	0.9412	0.0000	0.1765	0.1765	0.6471	0.6761	0.0808
8.0952	0.6095	22.9075	2.0833	1.0000	0.0000	0.8696	0.0435	0.1739	0.1304	0.6957	0.6902	0.0843
7.2500	0.5250	21.6887	1.9148	1.0000	0.0000	0.8889	0.1111	0.2222	0.1111	0.6667	0.8195	0.0949
7.1739	0.5174	21.0320	2.0455	0.9200	0.0800	0.8000	0.0800	0.3200	0.2000	0.4800	0.6279	0.0744
7.6250	0.5625	21.6375	1.8160	1.0000	0.0000	0.9231	0.0385	0.2692	0.1538	0.5769	0.6738	0.0794
7.7778	0.5778	19.8656	1.8728	1.0000	0.0000	0.9000	0.0000	0.2500	0.1500	0.6000	0.6732	0.0803
7.8824	0.5882	20.9313	1.8347	0.9500	0.0500	0.9500	0.0000	0.2000	0.2500	0.5500	0.5756	0.0688
7.4000	0.5400	22.2765	1.9435	0.9375	0.0625	0.8750	0.0625	0.1875	0.1250	0.6875	0.6125	0.0764
7.5833	0.5583	22.6686	1.9243	0.9259	0.0741	0.8519	0.0370	0.2222	0.1111	0.6667	0.5434	0.0670
7.1500	0.5150	22.9905	2.3181	0.9565	0.0435	0.8261	0.0870	0.3478	0.1304	0.5217	0.7218	0.0893
7.0500	0.5050	22.4919	2.2548	0.9565	0.0435	0.8261	0.0870	0.3478	0.1304	0.5217	0.7456	0.0888
7.3810	0.5381	20.5920	2.1639	1.0000	0.0000	0.8696	0.0000	0.2609	0.1304	0.6087	0.5735	0.0712
7.1818	0.5182	22.3450	2.1983	0.9600	0.0400	0.8400	0.0800	0.2800	0.1200	0.6000	0.6984	0.0847
7.5000	0.5500	21.8385	2.0098	0.9565	0.0435	0.8696	0.0870	0.2609	0.0870	0.6522	0.6334	0.0792
7.5385	0.5538	21.8286	1.9679	1.0000	0.0000	0.8621	0.0690	0.2069	0.2069	0.5862	0.6125	0.0753
6.7000	0.4700	22.4851	2.1668	1.0000	0.0000	1.0000	0.0000	0.1667	0.0833	0.7500	0.9589	0.1084
6.4167	0.4417	25.7599	2.4620	1.0000	0.0000	0.9286	0.0714	0.2857	0.0714	0.6429	0.9258	0.1074
6.8750	0.4875	23.3649	2.2600	0.9474	0.0526	1.0000	0.0000	0.2632	0.1053	0.5789	0.8506	0.0997
6.6429	0.4643	23.2783	2.2719	0.9444	0.0556	1.0000	0.0000	0.2778	0.1111	0.5556	0.8714	0.1005
6.5385	0.4538	22.7051	2.4011	1.0000	0.0000	0.9333	0.0000	0.4000	0.0667	0.5333	1.0931	0.1258
6.6667	0.4667	23.3368	2.2630	0.9444	0.0556	1.0000	0.0000	0.3333	0.1111	0.5556	0.8986	0.1052
7.0000	0.5000	20.6575	2.0837	0.9444	0.0556	0.8889	0.0000	0.2222	0.1111	0.6667	0.9637	0.1119
6.5789	0.4579	21.7624	2.0839	0.9048	0.0952	0.9524	0.0000	0.2857	0.0476	0.6667	0.8275	0.0985
8.5000	0.6500	15.4117	0.8755	0.9000	0.1000	0.8000	0.0000	0.2000	0.1000	0.6000	0.5484	0.0660
8.3571	0.6357	17.0581	1.4384	0.9412	0.0588	0.8235	0.0000	0.2353	0.1176	0.5882	0.5304	0.0666
8.7143	0.6714	19.6592	1.2537	0.7143	0.2857	0.8571	0.1429	0.1429	0.1429	0.5714	0.5008	0.0666
8.7143	0.6714	17.1634	1.1389	0.8571	0.1429	0.8571	0.1429	0.2857	0.0000	0.5714	0.3440	0.0452
8.5556	0.6556	18.3405	1.4463	0.8889	0.1111	0.8889	0.1111	0.2222	0.0000	0.6667	0.3065	0.0410
8.7143	0.6714	19.0691	1.2537	0.8571	0.1429	0.8571	0.1429	0.2857	0.0000	0.5714	0.7815	0.0860
8.6250	0.6625	19.0691	1.2537	0.8750	0.1250	0.8750	0.1250	0.2500	0.0000	0.6250	0.3364	0.0446
8.8000	0.6800	19.0405	1.2775	0.8000	0.2000	0.8000	0.2000	0.2000	0.0000	0.8000	0.5345	0.0706
8.8333	0.6833	19.0405	1.2775	0.8333	0.1667	0.8333	0.1667	0.3333	0.0000	0.6667	0.9096	0.1000
8.5714	0.6571	19.0691	1.2537	0.8571	0.1429	0.7143	0.1429	0.1429	0.0000	0.7143	0.4476	0.0592
8.6250	0.6625	19.0691	1.2537	0.8750	0.1250	0.7500	0.1250	0.2500	0.0000	0.6250	0.3843	0.0509
8.6667	0.6667	19.0691	1.2537	0.8333	0.1667	0.8333	0.1667	0.1667	0.0000	0.6667	0.4476	0.0592
8.8000	0.6800	19.6304	1.3767	0.8333	0.1667	0.8333	0.1667	0.1667	0.0000	0.8333	0.5054	0.0684
8.6667	0.6667	19.0405	1.2775	0.8333	0.1667	0.6667	0.1667	0.1667	0.0000	0.8333	0.5345	0.0706
6.4286	0.4429	18.7024	1.6445	0.8182	0.1818	0.9091	0.0000	0.3636	0.0909	0.5455	0.7235	0.0903
6.5714	0.4571	24.1762	2.4621	0.9474	0.0526	0.9474	0.0526	0.2632	0.0000	0.7368	0.7397	0.0898
7.8182	0.5818	22.7975	1.8548	0.6000	0.4000	0.8000	0.0667	0.3333	0.1333	0.4667	1.3248	0.1222
8.4444	0.6444	20.5788	1.8801	0.8333	0.1667	0.9167	0.0000	0.1667	0.0000	0.8333	0.3234	0.0480
8.9583	0.6958	24.2136	1.9954	0.8800	0.1200	1.0000	0.0000	0.2000	0.2000	0.5200	4.2885	0.1440
8.4000	0.6400	27.2416	1.9720	1.0000	0.0000	1.0000	0.0000	0.2000	0.2000	0.6000	0.9410	0.1092
8.5000	0.6500	18.3819	1.1921	0.8000	0.2000	1.0000	0.0000	0.4000	0.0000	0.6000	0.3319	0.0499
8.5000	0.6500	18.5179	1.6017	0.8000	0.2000	1.0000	0.0000	0.4000	0.0000	0.6000	0.2539	0.0383
8.2500	0.6250	18.3188	1.4540	0.7500	0.2500	1.0000	0.0000	0.2500	0.0000	0.7500	2.1325	0.1501
8.4286	0.6429	26.9135	2.1871	1.0000	0.0000	0.7727	0.0000	0.2273	0.1364	0.5455	1.8487	0.1383

8.8667	0.6867	22.1157	1.9984	0.7059	0.2353	0.9412	0.0000	0.0000	0.0588	0.8235	5.0751	0.1473
6.4286	0.4429	25.4713	2.3999	1.0000	0.0000	0.8889	0.1111	0.2222	0.1111	0.6667	0.7327	0.0892
7.0000	0.5000	19.0441	1.2496	0.7778	0.2222	0.8889	0.0000	0.3333	0.2222	0.4444	0.7358	0.0919
7.2500	0.5250	19.7752	1.5431	0.7778	0.2222	0.8889	0.0000	0.2222	0.2222	0.5556	0.7003	0.0865
7.2500	0.5250	19.7570	1.3117	0.6667	0.3333	0.8889	0.0000	0.3333	0.2222	0.4444	0.9408	0.1062
8.3333	0.6333	16.8767	1.9213	0.6667	0.3333	1.0000	0.0000	0.3333	0.0000	0.6667	2.6253	0.1663
8.3333	0.6333	16.2765	0.8626	0.6667	0.3333	1.0000	0.0000	0.6667	0.0000	0.3333	0.2832	0.0412
8.8462	0.6846	21.3986	1.8753	0.7143	0.2857	1.0000	0.0000	0.0714	0.2143	0.6429	4.8285	0.1431
6.7143	0.4714	23.5105	2.4182	0.9000	0.1000	1.0000	0.0000	0.1000	0.1000	0.8000	0.6804	0.0828
8.6250	0.6625	23.5245	1.5932	0.4000	0.6000	0.8000	0.1000	0.2000	0.1000	0.7000	0.6701	0.0853
7.9091	0.5909	21.5684	1.7680	0.7692	0.2308	0.9231	0.0769	0.1538	0.0769	0.7692	0.4150	0.0575
8.4286	0.6429	24.5414	1.6739	0.8750	0.1250	1.0000	0.0000	0.2500	0.1250	0.6250	4.6426	0.1649
7.8571	0.5857	24.2812	1.9350	0.5455	0.4545	0.5455	0.0909	0.2727	0.0909	0.4545	1.0537	0.1163
11.2000	0.9200	28.0800	0.9609	0.8333	0.0000	0.8333	0.0000	0.0000	0.3333	0.3333	0.1370	0.0238
6.8125	0.4813	22.9462	2.6777	0.9375	0.0625	0.8125	0.0000	0.2500	0.1875	0.5625	0.6754	0.0791
7.8750	0.5875	22.4941	1.5711	0.6667	0.3333	0.8889	0.1111	0.3333	0.2222	0.4444	1.3638	0.1293
8.3750	0.6375	19.7643	1.6007	0.7000	0.3000	1.0000	0.0000	0.2000	0.0000	0.8000	0.2705	0.0415
9.0667	0.7067	25.6040	1.7314	0.8235	0.1176	0.9412	0.0000	0.1765	0.2353	0.4706	0.8719	0.0912
8.2632	0.6263	25.0454	2.3948	0.8421	0.1579	1.0000	0.0000	0.1053	0.1053	0.7895	4.8212	0.1536
7.6875	0.5688	24.0946	2.0889	0.8421	0.1579	0.8421	0.1053	0.2105	0.1579	0.6316	1.3875	0.1053
8.4286	0.6429	21.2430	1.9262	0.8571	0.1429	1.0000	0.0000	0.1429	0.1429	0.7143	11.2208	0.2479
11.1429	0.9143	29.0833	1.1145	1.0000	0.0000	1.0000	0.0000	0.0000	0.4286	0.2857	0.1278	0.0222
8.0000	0.6000	22.3161	1.6913	1.0000	0.0000	0.9000	0.0500	0.1000	0.1500	0.7500	0.7243	0.0826
7.9032	0.5903	23.1542	1.9156	0.9722	0.0278	0.9167	0.0278	0.1944	0.1389	0.6667	1.0085	0.1047
8.0000	0.6000	18.4366	1.6195	0.9583	0.0417	0.9583	0.0000	0.1250	0.2083	0.6667	0.7297	0.0870
7.7931	0.5793	20.8554	1.6556	0.9697	0.0303	0.9394	0.0606	0.1818	0.2121	0.6061	0.8055	0.0908
7.6400	0.5640	20.3786	1.5878	0.9655	0.0345	0.9310	0.0345	0.2414	0.1724	0.5862	0.9375	0.1018
7.7742	0.5774	21.5579	1.7508	0.9429	0.0571	0.9429	0.0571	0.1429	0.1714	0.6857	0.8633	0.0991
7.6818	0.5682	20.4320	1.6652	0.9615	0.0385	0.9231	0.0385	0.2308	0.1154	0.6538	0.6961	0.0832
7.7000	0.5700	21.4112	1.6116	0.9706	0.0294	0.8824	0.1176	0.1765	0.2059	0.6176	0.6711	0.0834
8.2609	0.6261	24.0370	1.9099	0.8400	0.1600	0.8400	0.0800	0.0800	0.2000	0.7200	1.1474	0.1012
7.5600	0.5560	20.4309	1.7920	0.9643	0.0357	0.9286	0.0714	0.1429	0.1786	0.6786	1.1513	0.1056
8.8095	0.6810	22.2380	1.7799	0.8696	0.1304	0.9565	0.0000	0.0435	0.1739	0.7391	3.1370	0.1242
8.5333	0.6533	21.3492	1.4969	0.9375	0.0625	0.9375	0.0625	0.0625	0.3125	0.6250	0.5613	0.0761
6.2143	0.4214	24.2060	2.2355	0.8125	0.1875	0.9375	0.0625	0.3125	0.0000	0.6875	0.8982	0.1012
6.5385	0.4538	24.6136	2.5535	0.8667	0.1333	0.9333	0.0000	0.4000	0.0000	0.6000	0.8702	0.0963
6.3333	0.4333	25.0375	2.6447	1.0000	0.0000	1.0000	0.0000	0.6000	0.0000	0.4000	0.6841	0.0847
7.6000	0.5600	26.4260	2.6093	0.8636	0.1364	0.9545	0.0000	0.1364	0.0455	0.7727	4.7231	0.1664
7.5455	0.5545	27.4642	2.5432	0.9231	0.0769	0.9231	0.0000	0.2308	0.0000	0.7692	0.7108	0.0826
7.8000	0.5800	25.9072	2.8335	1.0000	0.0000	1.0000	0.0000	0.2000	0.0000	0.8000	0.9339	0.1092
6.6875	0.4688	22.8374	2.3462	0.9412	0.0588	1.0000	0.0000	0.3529	0.0000	0.6471	0.7604	0.0871
6.6667	0.4667	27.2572	2.5911	0.9412	0.0588	0.9412	0.0588	0.3529	0.0000	0.6471	0.7393	0.0870
6.6364	0.4636	26.0455	2.5768	1.0000	0.0000	1.0000	0.0000	0.4167	0.0000	0.5833	1.0302	0.1137
8.1364	0.6136	24.6084	2.2534	0.9091	0.0909	0.9545	0.0000	0.1818	0.0000	0.8182	2.5499	0.1424
8.1500	0.6150	24.6445	2.4224	0.9500	0.0500	0.9500	0.0500	0.2000	0.1000	0.6000	5.7012	0.1814
9.0000	0.7000	20.8058	2.0308	0.8333	0.1667	1.0000	0.0000	0.0000	0.1667	0.8333	0.3350	0.0454
7.2727	0.5273	24.0152	2.2100	0.8333	0.1667	1.0000	0.0000	0.1667	0.0000	0.8333	0.7320	0.0846
8.0000	0.6000	24.0098	2.0987	0.9310	0.0690	0.9310	0.0345	0.1379	0.1724	0.6897	0.6063	0.0774
7.6842	0.5684	23.6762	2.2519	0.9000	0.1000	1.0000	0.0000	0.0500	0.1000	0.8500	1.2826	0.1120
9.2000	0.7200	21.9991	1.7946	0.8667	0.1333	1.0000	0.0000	0.0000	0.2000	0.8000	3.8790	0.1379
6.4118	0.4412	26.8300	2.4586	0.9474	0.0526	0.9474	0.0526	0.4211	0.0000	0.5789	0.7920	0.0933
6.1429	0.4143	24.8162	2.6009	0.9333	0.0667	0.9333	0.0667	0.4667	0.0000	0.5333	0.7557	0.0886
7.6429	0.5643	24.5521	2.1436	0.9302	0.0698	0.9302	0.0000	0.2093	0.0698	0.6977	1.2092	0.1025
8.0000	0.6000	26.2304	2.3178	0.8182	0.1818	0.8182	0.0909	0.2727	0.0000	0.7273	0.6805	0.0815
8.7778	0.6778	26.7958	1.9136	0.8889	0.1111	1.0000	0.0000	0.0000	0.2222	0.7778	0.4829	0.0666
9.2500	0.7250	24.5078	1.5878	0.9167	0.0833	0.9167	0.0000	0.0833	0.1667	0.6667	0.5138	0.0693
9.3571	0.7357	22.5689	1.8643	0.9286	0.0714	0.7857	0.0000	0.0714	0.2143	0.6429	4.2159	0.1246
8.6000	0.6600	16.3234	1.1415	0.8000	0.2000	1.0000	0.0000	0.0000	0.2000	0.6000	0.7944	0.1012
8.7692	0.6769	19.6910	1.8140	0.8462	0.1538	0.9231	0.0000	0.1538	0.1538	0.6923	4.0885	0.1079
10.0000	0.8000	26.7625	1.6825	1.0000	0.0000	0.7692	0.0000	0.0769	0.3846	0.4615	0.7181	0.0832
11.2500	0.9250	33.8724	3.0550	1.0000	0.0000	0.5000	0.0000	0.0000	0.5000	0.2000	4.4351	0.2259
9.9286	0.7929	23.2819	1.2332	0.9286	0.0714	0.7857	0.0000	0.0000	0.4286	0.5000	6.7137	0.2063
10.7000	0.8700	29.3940	1.4739	0.9000	0.1000	0.7000	0.0000	0.0000	0.6000	0.3000	11.6425	0.2933
9.0000	0.7000	21.9462	2.3685	0.8333	0.1667	0.8333	0.0000	0.3333	0.0000	0.6667	8.2985	0.1633
7.8333	0.5833	25.5481	2.3316	0.8333	0.1667	1.0000	0.0000	0.0000	0.0000	1.0000	0.4785	0.0650
9.5600	0.7560	28.2834	2.5587	0.9600	0.0400	0.8400	0.0000	0.0400	0.2800	0.5600	0.8214	0.0976
10.0000	0.8000	25.9702	1.4767	1.0000	0.0000	0.8000	0.0000	0.1000	0.2000	0.6000	0.5686	0.0740
11.1667	0.9167	31.3087	5.6100	1.0000	0.0000	0.5714	0.0000	0.0000	0.5714	0.2857	7.6487	0.3258
8.2727	0.6273	24.4178	2.0740	1.0000	0.0000	0.8182	0.0000	0.0909	0.1818	0.7273	0.7485	0.0940
8.7333	0.6733	23.1770	1.8008	0.9333	0.0667	0.8667	0.0000	0.0667	0.2000	0.7333	0.8196	0.0975
9.0000	0.7000	24.3806	1.6602	1.0000	0.0000	0.8462	0.0000	0.0769	0.2308	0.6923	0.6673	0.0837
11.3750	0.9375	33.4000	1.1145	1.0000	0.0000	0.6250	0.0000	0.0000	0.6250	0.1250	1.0119	0.0984
11.3750	0.9375	33.4000	1.1145	1.0000	0.0000	0.6250	0.0000	0.0000	0.6250	0.1250	1.0119	0.0984
11.7500	0.9750	32.5333	NA	1.0000	0.0000	0.5000	0.0000	0.0000	1.0000	0.0000	2.7448	0.2449
10.5833	0.8583	31.7308	2.2070	1.0000	0.0000	0.6667	0.0000	0.0000	0.5000	0.3333	0.9249	0.1076
11.5000	0.9500	29.0800	1.5759	1.0000	0.0000	0.6667	0.0000	0.0000	0.6667	0.1667	1.4024	0.1278
10.9000	0.8900	34.2980	2.5621	1.0000	0.0000	0.7000	0.0000	0.0000	0.5000	0.3000	0.9460	0.1040

8.2941	0.6294	22.0078	2.1262	0.8824	0.1176	0.9412	0.0000	0.1765	0.0588	0.7647	3.3231	0.1061
8.3571	0.6357	25.5392	2.2645	1.0000	0.0000	0.7857	0.0000	0.2143	0.0000	0.7857	1.6602	0.1350
9.1111	0.7111	26.8169	1.9505	0.9259	0.0741	1.0000	0.0000	0.0741	0.2593	0.5926	3.2990	0.1378
6.9333	0.4933	26.7234	2.6458	0.9412	0.0588	0.8824	0.0588	0.4118	0.0000	0.5882	0.6592	0.0805
6.5556	0.4556	28.1205	2.8230	0.9167	0.0833	1.0000	0.0000	0.1667	0.0000	0.8333	0.7771	0.0922
7.1875	0.5188	19.3405	2.0719	0.8824	0.1176	0.8824	0.0000	0.2353	0.0588	0.7059	0.4365	0.0591
7.4118	0.5412	24.2669	2.2573	0.8824	0.1176	0.8824	0.0000	0.1765	0.1176	0.6471	0.5859	0.0715
6.7143	0.4714	23.8958	2.6042	0.8750	0.1250	1.0000	0.0000	0.3750	0.0000	0.6250	0.9127	0.1020
7.8571	0.5857	28.1966	2.3037	1.0000	0.0000	1.0000	0.0000	0.2500	0.0000	0.7500	0.7610	0.0898
6.9000	0.4900	27.7219	2.5692	1.0000	0.0000	0.8182	0.0909	0.3636	0.0909	0.5455	0.7250	0.0903
9.2222	0.7222	32.7667	3.4301	0.8889	0.1111	0.7778	0.0000	0.1111	0.3333	0.5556	7.9093	0.2064
8.0909	0.6091	18.2350	1.2136	0.9231	0.0769	0.8462	0.0769	0.3077	0.0769	0.6154	0.6547	0.0810
8.3333	0.6333	17.3719	1.0955	0.8750	0.1250	0.8750	0.0000	0.2500	0.0000	0.7500	0.5375	0.0658
8.0000	0.6000	15.7832	1.0271	0.8000	0.2000	1.0000	0.0000	0.4000	0.0000	0.6000	0.7856	0.0925
8.7500	0.6750	19.4860	1.1107	0.8889	0.1111	0.7778	0.1111	0.3333	0.1111	0.5556	0.6441	0.0785
8.5000	0.6500	17.5951	1.2265	0.8750	0.1250	0.7500	0.1250	0.2500	0.1250	0.6250	0.6277	0.0813
8.5000	0.6500	19.4860	1.1107	0.8571	0.1429	0.7143	0.1429	0.2857	0.0000	0.7143	0.7955	0.0954
8.5714	0.6571	17.7693	1.0823	0.8750	0.1250	0.8750	0.0000	0.5000	0.0000	0.5000	0.5505	0.0678
8.0000	0.6000	16.9207	1.0706	0.8333	0.1667	0.8333	0.0000	0.3333	0.0000	0.6667	0.6859	0.0823
8.7500	0.6750	17.7693	1.0823	0.8889	0.1111	0.8889	0.0000	0.4444	0.1111	0.4444	0.5141	0.0644
8.7500	0.6750	18.8023	1.0706	0.8889	0.1111	0.7778	0.1111	0.3333	0.1111	0.5556	0.6657	0.0821
8.7143	0.6714	18.8672	0.9583	0.8571	0.1429	0.7143	0.1429	0.2857	0.1429	0.5714	0.7759	0.0926
8.7143	0.6714	17.9946	1.1341	0.8750	0.1250	0.8750	0.0000	0.3750	0.1250	0.5000	0.2924	0.0454
8.4286	0.6429	16.1148	1.0404	0.9000	0.1000	0.8000	0.0000	0.4000	0.1000	0.4000	0.5076	0.0633
8.6000	0.6600	17.3105	1.1920	0.9231	0.0769	0.7692	0.0000	0.3077	0.1538	0.4615	0.6308	0.0768
8.3333	0.6333	17.8891	1.0755	0.9000	0.1000	0.6000	0.1000	0.4000	0.0000	0.5000	0.6468	0.0767
7.6667	0.5667	15.4170	0.9953	0.7500	0.2500	1.0000	0.0000	0.5000	0.0000	0.5000	0.4677	0.0646
7.0000	0.5000	15.1250	1.0215	0.7500	0.2500	1.0000	0.0000	0.5000	0.0000	0.5000	0.2953	0.0446
8.5000	0.6500	17.9935	1.1929	0.8333	0.1667	1.0000	0.0000	0.3333	0.0000	0.6667	0.2171	0.0329
7.0000	0.5000	18.1610	1.7275	0.8333	0.1667	1.0000	0.0000	0.5000	0.0000	0.5000	2.3496	0.1494
8.5000	0.6500	18.3308	1.0691	0.8750	0.1250	0.7500	0.1250	0.2500	0.0000	0.7500	0.6706	0.0803
8.5000	0.6500	19.3860	1.0984	0.8571	0.1429	0.7143	0.1429	0.2857	0.0000	0.7143	0.7572	0.0901
7.8333	0.5833	18.8607	1.0899	0.8571	0.1429	0.5714	0.1429	0.2857	0.0000	0.7143	0.8361	0.0971
8.1667	0.6167	16.7289	1.0606	0.8571	0.1429	0.7143	0.0000	0.4286	0.1429	0.4286	0.7169	0.0874
7.8333	0.5833	17.4016	1.3405	0.8750	0.1250	0.6250	0.0000	0.3750	0.0000	0.5000	0.8704	0.0990
8.6250	0.6625	17.2254	1.0881	0.9091	0.0909	0.8182	0.0000	0.3636	0.0909	0.4545	0.4689	0.0591
8.5556	0.6556	17.4323	1.1883	0.9167	0.0833	0.6667	0.0833	0.3333	0.1667	0.4167	0.5907	0.0761
7.5000	0.5500	17.2053	1.1129	0.8333	0.1667	0.5000	0.0000	0.3333	0.0000	0.5000	0.9026	0.1039
8.6667	0.6667	18.3832	1.1328	0.8571	0.1429	0.8571	0.0000	0.2857	0.1429	0.5714	0.6318	0.0779
8.1429	0.6143	17.1594	1.0246	0.8889	0.1111	0.6667	0.1111	0.5556	0.0000	0.4444	0.7097	0.0858
8.0000	0.6000	16.8600	1.1474	0.8000	0.2000	0.6000	0.0000	0.4000	0.0000	0.4000	0.4396	0.0661
8.4286	0.6429	20.6133	1.3359	0.8750	0.1250	0.8750	0.1250	0.2500	0.0000	0.7500	1.0117	0.1188
8.6667	0.6667	19.4560	1.1707	0.8000	0.2000	0.8000	0.2000	0.2000	0.0000	0.8000	0.5260	0.0699
8.3333	0.6333	19.1539	1.1901	0.7500	0.2500	1.0000	0.0000	0.2500	0.0000	0.7500	0.8792	0.1002
6.6667	0.4667	15.3450	1.1368	0.7500	0.2500	1.0000	0.0000	0.7500	0.0000	0.2500	0.4561	0.0685
7.5000	0.5500	18.0133	1.1598	0.8333	0.1667	0.8333	0.1667	0.3333	0.0000	0.6667	0.4851	0.0659
8.5000	0.6500	17.4693	1.1150	0.8333	0.1667	1.0000	0.0000	0.5000	0.0000	0.5000	0.6245	0.0733
8.0000	0.6000	16.8613	1.2384	0.8750	0.1250	0.6250	0.0000	0.3750	0.1250	0.5000	0.9557	0.1113
8.1667	0.6167	15.9632	1.0537	0.8750	0.1250	0.7500	0.0000	0.5000	0.1250	0.3750	0.5805	0.0703
8.5000	0.6500	16.3232	1.0144	0.8571	0.1429	0.8571	0.0000	0.4286	0.1429	0.4286	0.6430	0.0797
8.2000	0.6200	16.9405	1.0103	0.9167	0.0833	0.5833	0.0833	0.4167	0.1667	0.3333	0.8113	0.0996
7.5000	0.5500	17.7167	1.0677	0.8000	0.2000	0.8000	0.2000	0.4000	0.0000	0.6000	1.1470	0.1331
8.2857	0.6286	17.4693	1.0448	0.8889	0.1111	0.6667	0.1111	0.4444	0.1111	0.4444	0.6791	0.0815
8.1667	0.6167	15.9632	1.0537	0.8750	0.1250	0.7500	0.0000	0.5000	0.1250	0.3750	0.5805	0.0703
7.9091	0.5909	14.9913	1.0396	0.9231	0.0769	0.6923	0.0000	0.6154	0.1538	0.2308	0.5081	0.0637
7.4286	0.5429	16.5165	1.0534	0.8889	0.1111	0.6667	0.1111	0.5556	0.0000	0.4444	0.6863	0.0826
8.1667	0.6167	20.3232	1.1521	0.8571	0.1429	0.5714	0.1429	0.2857	0.0000	0.7143	0.8501	0.0995
7.5000	0.5500	17.2053	1.1129	0.8000	0.2000	0.6000	0.0000	0.4000	0.0000	0.6000	0.9026	0.1039
8.0000	0.6000	18.0700	1.2134	0.8000	0.2000	0.8000	0.0000	0.4000	0.0000	0.6000	0.3152	0.0474
7.8571	0.5857	17.1193	1.1282	0.8750	0.1250	0.8750	0.0000	0.5000	0.0000	0.5000	0.5606	0.0693
8.5000	0.6500	17.1839	0.9294	0.8333	0.1667	0.8333	0.0000	0.5000	0.0000	0.5000	0.5961	0.0691
8.1667	0.6167	18.2632	1.1181	0.8571	0.1429	0.7143	0.0000	0.4286	0.0000	0.5714	0.6552	0.0784
7.2857	0.5286	15.9913	1.0487	0.8750	0.1250	0.7500	0.0000	0.7500	0.0000	0.2500	0.4498	0.0658
7.6250	0.5625	14.7434	0.9028	0.9000	0.1000	0.7000	0.0000	0.6000	0.1000	0.2000	0.7123	0.0871
8.0000	0.6000	14.7589	0.7999	0.8333	0.1667	0.6667	0.0000	0.6667	0.0000	0.3333	0.7837	0.0927
7.8000	0.5800	16.5789	1.0421	0.8333	0.1667	0.6667	0.0000	0.5000	0.0000	0.5000	0.7621	0.0899
8.2000	0.6200	18.2632	1.0337	0.8571	0.1429	0.5714	0.1429	0.2857	0.0000	0.5714	0.8633	0.1016
7.8000	0.5800	15.9632	1.0537	0.8571	0.1429	0.7143	0.0000	0.5714	0.0000	0.4286	0.6401	0.0758
7.8000	0.5800	16.5789	1.0421	0.8333	0.1667	0.6667	0.0000	0.5000	0.0000	0.5000	0.7621	0.0899
8.1429	0.6143	15.8526	1.0287	0.8889	0.1111	0.6667	0.0000	0.5556	0.1111	0.3333	0.6976	0.0848
8.3750	0.6375	16.8072	0.9243	0.8750	0.1250	0.6250	0.0000	0.5000	0.1250	0.3750	0.7588	0.0910
7.0000	0.5000	17.6400	1.2693	0.5000	0.5000	1.0000	0.0000	0.5000	0.0000	0.5000	0.5379	0.0786
7.0000	0.5000	17.3386	1.1767	0.6000	0.4000	1.0000	0.0000	0.2000	0.0000	0.8000	0.3312	0.0503
7.0000	0.5000	15.7600	1.1440	0.6667	0.3333	1.0000	0.0000	0.3333	0.0000	0.6667	0.3799	0.0562
8.5000	0.6500	18.0700	1.2134	0.7500	0.2500	1.0000	0.0000	0.2500	0.0000	0.7500	0.3152	0.0474
7.8000	0.5800	19.5677	1.3905	0.8333	0.1667	0.5000	0.1667	0.3333	0.0000	0.6667	0.8534	0.1062
7.0000	0.5000	17.6400	1.2693	0.5000	0.5000	1.0000	0.0000	0.5000	0.0000	0.5000	0.5379	0.0786

7.0000	0.5000	17.6400	1.2693	0.5000	0.5000	1.0000	0.0000	0.5000	0.0000	0.5000	0.5379	0.0786
5.5000	0.3500	11.7500	0.8855	0.5000	0.5000	1.0000	0.0000	1.0000	0.0000	0.0000	0.4982	0.0730
8.3333	0.6333	19.5677	1.4959	0.7500	0.2500	1.0000	0.0000	0.2500	0.0000	0.7500	0.7010	0.0890
6.0000	0.4000	18.8267	2.1295	0.3333	0.6667	1.0000	0.0000	0.6667	0.0000	0.3333	0.5086	0.0753
6.5000	0.4500	18.4560	1.7831	0.6000	0.4000	1.0000	0.0000	0.6000	0.0000	0.4000	0.3855	0.0586
6.6667	0.4667	16.0460	0.9147	0.8000	0.2000	1.0000	0.0000	0.6000	0.0000	0.4000	0.4313	0.0648
7.0000	0.5000	17.6400	1.2693	0.5000	0.5000	1.0000	0.0000	0.5000	0.0000	0.5000	0.5379	0.0786
7.5714	0.5571	21.9184	1.8356	0.8750	0.1250	1.0000	0.0000	0.2500	0.1250	0.6250	0.5554	0.0746
7.5000	0.5500	22.4770	1.3774	0.8571	0.1429	0.8571	0.0000	0.4286	0.0000	0.5714	0.6962	0.0911
7.7500	0.5750	20.1953	1.9423	0.8333	0.1667	1.0000	0.0000	0.3333	0.0000	0.6667	2.0576	0.1245
7.6667	0.5667	19.6750	1.6719	0.7500	0.2500	1.0000	0.0000	0.5000	0.0000	0.5000	2.9342	0.1645
7.5556	0.5556	15.3332	1.1209	0.9167	0.0833	0.7500	0.0000	0.5000	0.0833	0.4167	0.5321	0.0682
7.6000	0.5600	19.9859	1.9348	0.9091	0.0909	0.7273	0.0909	0.3636	0.0000	0.6364	0.7724	0.0934
6.3333	0.4333	17.9933	1.1862	0.7500	0.2500	0.7500	0.0000	0.7500	0.0000	0.2500	0.4319	0.0647
7.0000	0.5000	17.3500	1.7830	0.6667	0.3333	0.6667	0.0000	0.3333	0.0000	0.3333	3.0079	0.2207
7.0000	0.5000	17.3500	1.7830	0.6667	0.3333	0.6667	0.0000	0.3333	0.0000	0.3333	3.0079	0.2207
5.5000	0.3500	11.7500	0.8855	0.5000	0.5000	1.0000	0.0000	1.0000	0.0000	0.0000	0.4982	0.0730
8.0000	0.6000	18.9526	1.9679	1.0000	0.0000	1.0000	0.0000	0.2500	0.0000	0.7500	1.7254	0.1386
5.5000	0.3500	14.3167	0.6237	0.7500	0.2500	0.7500	0.0000	0.5000	0.0000	0.2500	0.5321	0.0777
7.5000	0.5500	21.8854	1.4217	1.0000	0.0000	0.6667	0.0000	0.0000	0.0000	0.6667	0.0610	0.0107
8.5000	0.6500	18.8500	1.0439	0.5000	0.5000	1.0000	0.0000	0.5000	0.0000	0.5000	0.4184	0.0606
NA	NA	22.0000	2.9000	1.0000	0.0000	0.5000	0.0000	0.0000	0.0000	0.5000	5.3000	0.3413
5.5000	0.3500	15.3600	1.2145	0.6667	0.3333	1.0000	0.0000	0.6667	0.0000	0.3333	0.4521	0.0676
7.0000	0.5000	12.7000	0.6660	0.5000	0.5000	0.5000	0.0000	0.5000	0.0000	0.0000	0.7158	0.1001
7.0000	0.5000	12.7000	0.6660	0.0000	1.0000	1.0000	0.0000	1.0000	0.0000	0.0000	0.7158	0.1001
6.6667	0.4667	14.5200	1.1343	0.8000	0.2000	1.0000	0.0000	0.4000	0.0000	0.6000	0.6425	0.0810
4.0000	0.2000	10.8000	1.1050	1.0000	0.0000	1.0000	0.0000	1.0000	0.0000	0.0000	0.2805	0.0458
5.5000	0.3500	11.7500	0.8855	0.6667	0.3333	0.6667	0.0000	0.6667	0.0000	0.0000	0.4982	0.0730
7.7500	0.5750	17.2977	1.3778	0.8000	0.2000	0.8000	0.0000	0.4000	0.0000	0.4000	0.6123	0.0750
7.0000	0.5000	12.8375	1.1049	1.0000	0.0000	1.0000	0.0000	0.5000	0.0000	0.5000	0.1790	0.0279
7.0000	0.5000	12.3500	0.7797	0.5000	0.5000	1.0000	0.0000	0.5000	0.0000	0.5000	0.3899	0.0558
7.0000	0.5000	17.6400	1.2693	0.5000	0.5000	1.0000	0.0000	0.5000	0.0000	0.5000	0.5379	0.0786
7.0000	0.5000	12.7000	0.6660	0.0000	1.0000	1.0000	0.0000	1.0000	0.0000	0.0000	0.7158	0.1001
5.0000	0.3000	17.1723	3.2683	1.0000	0.0000	1.0000	0.0000	0.6667	0.0000	0.3333	3.6306	0.1819
5.5000	0.3500	11.7500	0.8855	0.5000	0.5000	1.0000	0.0000	1.0000	0.0000	0.0000	0.4982	0.0730
6.0000	0.4000	21.5500	4.5000	1.0000	0.0000	1.0000	0.0000	1.0000	0.0000	0.0000	10.3000	0.4497
4.0000	0.2000	10.8000	1.1050	1.0000	0.0000	1.0000	0.0000	1.0000	0.0000	0.0000	0.2805	0.0458
7.0000	0.5000	16.1667	1.0642	0.6667	0.3333	1.0000	0.0000	0.6667	0.0000	0.3333	0.3724	0.0557
4.0000	0.2000	10.8000	1.1050	1.0000	0.0000	1.0000	0.0000	1.0000	0.0000	0.0000	0.2805	0.0458
10.0000	0.8000	25.0000	1.4217	1.0000	0.0000	0.5000	0.0000	0.0000	0.0000	0.5000	0.1210	0.0212
7.0000	0.5000	24.2193	1.0739	1.0000	0.0000	1.0000	0.0000	0.0000	0.0000	1.0000	1.0034	0.1097
10.0000	0.8000	25.0000	1.4217	1.0000	0.0000	1.0000	0.0000	0.0000	0.0000	1.0000	0.1210	0.0212
5.0000	0.3000	16.6316	0.8929	0.8000	0.2000	1.0000	0.0000	0.4000	0.0000	0.6000	0.7899	0.0931
6.2500	0.4250	20.2543	2.0321	0.7778	0.2222	1.0000	0.0000	0.4444	0.0000	0.5556	0.6928	0.0844
8.5000	0.6500	22.2200	1.9089	0.7500	0.2500	1.0000	0.0000	0.2500	0.0000	0.7500	0.5073	0.0681
8.0000	0.6000	16.9400	2.1070	0.5000	0.5000	1.0000	0.0000	0.5000	0.0000	0.5000	0.3879	0.0554
8.5000	0.6500	18.8500	1.0439	0.5000	0.5000	1.0000	0.0000	0.5000	0.0000	0.5000	0.4184	0.0606
7.0000	0.5000	21.9693	1.2626	0.6667	0.3333	1.0000	0.0000	0.3333	0.0000	0.6667	1.0420	0.1140
6.0000	0.4000	21.3359	1.4089	1.0000	0.0000	1.0000	0.0000	0.3333	0.0000	0.6667	0.8969	0.0959
4.0000	0.2000	20.5293	1.5592	1.0000	0.0000	1.0000	0.0000	0.3333	0.0000	0.6667	0.9766	0.1079
6.6000	0.4600	20.5525	1.2274	0.8571	0.1429	1.0000	0.0000	0.2857	0.1429	0.5714	1.3296	0.1166
7.6000	0.5600	19.8802	1.8937	1.0000	0.0000	1.0000	0.0000	0.1667	0.1667	0.6667	0.2023	0.0329
9.2000	0.7200	18.5950	1.6924	1.0000	0.0000	0.8000	0.0000	0.0000	0.6000	0.4000	0.2663	0.0393
8.7143	0.6714	18.4252	1.8929	0.8750	0.1250	1.0000	0.0000	0.1250	0.2500	0.6250	0.2588	0.0360
8.8889	0.6889	15.4817	1.7145	1.0000	0.0000	0.8889	0.0000	0.0000	0.2222	0.7778	1.5819	0.1236
8.3333	0.6333	17.9202	1.7966	1.0000	0.0000	0.8889	0.0000	0.2222	0.3333	0.4444	0.4776	0.0562
8.1667	0.6167	16.5190	2.0039	0.8333	0.1667	1.0000	0.0000	0.0000	0.1667	0.8333	0.8998	0.0957
8.2500	0.6250	14.4250	1.0317	1.0000	0.0000	1.0000	0.0000	0.2500	0.2500	0.5000	0.2130	0.0340
9.6667	0.7667	15.6333	1.0072	1.0000	0.0000	1.0000	0.0000	0.0000	0.3333	0.6667	0.1905	0.0300
8.0000	0.6000	17.9367	1.6572	1.0000	0.0000	1.0000	0.0000	0.3333	0.0000	0.6667	0.5338	0.0711
8.0000	0.6000	17.9367	1.6572	1.0000	0.0000	1.0000	0.0000	0.3333	0.0000	0.6667	0.5338	0.0711
9.6667	0.7667	18.3376	1.8889	1.0000	0.0000	1.0000	0.0000	0.0000	0.0000	1.0000	0.4458	0.0568
4.0000	0.2000	14.3000	1.2025	1.0000	0.0000	1.0000	0.0000	1.0000	0.0000	0.0000	0.2753	0.0451
9.5000	0.7500	14.6125	0.9938	1.0000	0.0000	1.0000	0.0000	0.2500	0.2500	0.5000	1.9224	0.1201
9.1667	0.7167	21.7700	1.3151	1.0000	0.0000	0.8333	0.1667	0.1667	0.1667	0.6667	1.7030	0.1343
9.3333	0.7333	19.0167	1.2918	1.0000	0.0000	0.7500	0.0000	0.2500	0.0000	0.5000	2.5630	0.1601
8.8000	0.6800	22.2332	2.0989	0.8333	0.1667	1.0000	0.0000	0.0000	0.3333	0.6667	0.2435	0.0365
7.8889	0.5889	20.7383	1.8731	0.9000	0.1000	0.9000	0.0000	0.1000	0.3000	0.6000	0.4798	0.0589
8.7500	0.6750	19.4082	2.1760	0.8000	0.2000	1.0000	0.0000	0.0000	0.4000	0.6000	0.1180	0.0197
9.1667	0.7167	21.3960	1.6747	1.0000	0.0000	0.8333	0.1667	0.1667	0.0000	0.8333	0.5227	0.0711
9.0000	0.7000	19.9276	1.5372	1.0000	0.0000	0.8750	0.1250	0.1250	0.0000	0.8750	1.6145	0.1348
9.5000	0.7500	20.8750	1.4859	1.0000	0.0000	1.0000	0.0000	0.0000	0.0000	1.0000	3.6105	0.2045
9.0000	0.7000	21.2214	1.3679	1.0000	0.0000	1.0000	0.0000	0.0000	0.2500	0.7500	2.4728	0.1718
9.0000	0.7000	25.9000	1.5609	1.0000	0.0000	0.6667	0.0000	0.0000	0.6667	0.3333	0.4237	0.0619
9.3333	0.7333	22.8500	1.5572	1.0000	0.0000	1.0000	0.0000	0.0000	0.3333	0.6667	2.5237	0.1549
7.6667	0.5667	17.5167	1.3589	1.0000	0.0000	1.0000	0.0000	0.3333	0.0000	0.6667	2.5005	0.1516

9.5000	0.7500	20.8750	1.4859	1.0000	0.0000	1.0000	0.0000	0.0000	0.0000	1.0000	3.6105	0.2045
10.0000	0.8000	25.0000	1.4217	1.0000	0.0000	1.0000	0.0000	0.0000	0.0000	1.0000	0.1210	0.0212
9.0000	0.7000	18.7818	1.8845	1.0000	0.0000	1.0000	0.0000	0.3333	0.0000	0.6667	3.4160	0.2336
9.3333	0.7333	19.0167	1.2918	1.0000	0.0000	1.0000	0.0000	0.3333	0.0000	0.6667	2.5630	0.1601
9.0000	0.7000	16.7500	1.5500	1.0000	0.0000	1.0000	0.0000	0.0000	0.0000	1.0000	7.1000	0.3879
9.2222	0.7222	16.9660	1.6239	1.0000	0.0000	1.0000	0.0000	0.1111	0.1111	0.7778	1.5296	0.1207
9.1000	0.7100	17.3800	1.3700	1.0000	0.0000	0.9091	0.0000	0.0000	0.1818	0.8182	1.2644	0.1105
9.0909	0.7091	18.1021	1.9082	1.0000	0.0000	0.9091	0.0000	0.0909	0.2727	0.6364	1.0681	0.0898
9.6667	0.7667	15.2833	0.5406	1.0000	0.0000	1.0000	0.0000	0.0000	0.4000	0.6000	0.5227	0.0633
9.3333	0.7333	23.0900	2.4849	1.0000	0.0000	1.0000	0.0000	0.0000	0.2500	0.7500	0.5183	0.0600
9.2500	0.7250	22.2250	1.0132	1.0000	0.0000	1.0000	0.0000	0.0000	0.1667	0.8333	0.6422	0.0821
8.5000	0.6500	20.1436	1.6491	1.0000	0.0000	0.9000	0.0000	0.1000	0.3000	0.6000	1.0681	0.0915
8.8571	0.6857	19.8860	1.5339	1.0000	0.0000	0.8571	0.0000	0.2857	0.2857	0.4286	0.6170	0.0749
9.0000	0.7000	21.8360	1.6639	1.0000	0.0000	0.8750	0.0000	0.1250	0.2500	0.6250	1.5783	0.1286
8.5000	0.6500	18.7583	1.3295	0.9000	0.1000	0.9000	0.0000	0.1000	0.2000	0.7000	1.5030	0.1314
8.0000	0.6000	19.1659	1.3995	1.0000	0.0000	0.9091	0.0000	0.1818	0.1818	0.6364	1.4012	0.1243
8.8000	0.6800	19.3839	0.9679	1.0000	0.0000	0.8571	0.0000	0.0000	0.2857	0.7143	1.6930	0.1365
9.0000	0.7000	20.4250	1.1429	1.0000	0.0000	0.8571	0.0000	0.0000	0.2857	0.7143	1.4259	0.1145
9.0000	0.7000	20.5950	1.6549	1.0000	0.0000	0.8571	0.0000	0.1429	0.1429	0.7143	1.7537	0.1390
9.0000	0.7000	20.5760	1.6239	1.0000	0.0000	0.9000	0.0000	0.1000	0.2000	0.7000	1.4618	0.1250
8.8000	0.6800	22.0607	1.9488	1.0000	0.0000	1.0000	0.0000	0.2000	0.1000	0.7000	1.5635	0.1338
9.0000	0.7000	19.7411	2.1262	1.0000	0.0000	1.0000	0.0000	0.1818	0.0909	0.7273	0.8780	0.0987
8.9231	0.6923	16.7294	1.9044	1.0000	0.0000	0.9286	0.0000	0.1429	0.2857	0.5714	0.7577	0.0882
9.0000	0.7000	20.2302	2.0122	1.0000	0.0000	0.9167	0.0000	0.0833	0.0833	0.7500	1.5715	0.1349
9.0000	0.7000	22.7427	2.1733	1.0000	0.0000	1.0000	0.0000	0.1000	0.2000	0.7000	0.9279	0.1035
8.9091	0.6909	19.2011	2.2665	1.0000	0.0000	0.9167	0.0000	0.1667	0.1667	0.6667	1.4349	0.1238
8.8750	0.6875	19.4511	1.5738	1.0000	0.0000	1.0000	0.0000	0.0000	0.1000	0.9000	1.6123	0.1361
9.0000	0.7000	19.8380	2.1238	0.9231	0.0769	1.0000	0.0000	0.0769	0.0769	0.8462	1.7287	0.1507
9.0000	0.7000	17.3258	1.3804	1.0000	0.0000	1.0000	0.0000	0.0000	0.1538	0.8462	1.3226	0.1179
9.0000	0.7000	18.2800	1.4028	1.0000	0.0000	0.9091	0.0000	0.1818	0.0909	0.7273	1.4600	0.1246
9.0000	0.7000	19.2784	1.5911	1.0000	0.0000	1.0000	0.0000	0.1111	0.0000	0.8889	1.5698	0.1320
8.7000	0.6700	18.6630	2.1923	1.0000	0.0000	0.9091	0.0000	0.3636	0.0909	0.5455	1.3377	0.1081
9.0000	0.7000	20.9088	2.0524	1.0000	0.0000	0.8571	0.0000	0.2857	0.4286	0.2857	1.6193	0.1218
9.0000	0.7000	18.8702	2.1715	1.0000	0.0000	1.0000	0.0000	0.1667	0.0000	0.8333	1.7483	0.1277
9.1667	0.7167	19.5500	1.4379	1.0000	0.0000	1.0000	0.0000	0.1429	0.2857	0.5714	1.7051	0.1327
9.0000	0.7000	18.9000	1.0500	1.0000	0.0000	1.0000	0.0000	0.0000	0.0000	1.0000	2.7167	0.1813
8.2500	0.6250	18.9932	1.3634	1.0000	0.0000	1.0000	0.0000	0.0000	0.0000	1.0000	0.8128	0.0924
6.5000	0.4500	18.6969	0.9017	1.0000	0.0000	1.0000	0.0000	0.3333	0.0000	0.6667	0.3197	0.0490
7.6667	0.5667	20.7341	1.1999	1.0000	0.0000	1.0000	0.0000	0.2000	0.0000	0.8000	0.2880	0.0450
9.0000	0.7000	NA	NA	1.0000	0.0000	1.0000	0.0000	0.0000	0.0000	1.0000	1.7920	0.1904
6.5000	0.4500	18.3325	1.1444	1.0000	0.0000	1.0000	0.0000	0.2500	0.0000	0.7500	0.4226	0.0647
7.0000	0.5000	16.9186	1.2357	1.0000	0.0000	1.0000	0.0000	0.2857	0.0000	0.7143	1.5101	0.1152
7.2500	0.5250	17.5217	1.2120	1.0000	0.0000	1.0000	0.0000	0.1667	0.0000	0.8333	1.6923	0.1198
6.5000	0.4500	13.0500	1.0043	1.0000	0.0000	1.0000	0.0000	1.0000	0.0000	0.0000	0.3743	0.0585
8.3333	0.6333	18.4625	1.3611	1.0000	0.0000	1.0000	0.0000	0.2500	0.0000	0.7500	0.2803	0.0448
5.0000	0.3000	10.6750	1.1638	1.0000	0.0000	1.0000	0.0000	0.5000	0.0000	0.5000	0.2805	0.0458
7.6000	0.5600	18.2750	1.2582	1.0000	0.0000	0.8333	0.1667	0.3333	0.0000	0.6667	0.4981	0.0680
8.0000	0.6000	17.7750	1.3221	1.0000	0.0000	1.0000	0.0000	0.0000	0.0000	1.0000	0.1210	0.0212
8.3333	0.6333	17.4333	1.3981	1.0000	0.0000	1.0000	0.0000	0.0000	0.0000	1.0000	3.6105	0.2045
6.0000	0.4000	10.5500	1.2225	1.0000	0.0000	1.0000	0.0000	0.0000	0.0000	1.0000	NA	NA
6.0000	0.4000	10.5500	1.2225	1.0000	0.0000	1.0000	0.0000	0.0000	0.0000	1.0000	NA	NA
7.0000	0.5000	17.9000	1.2634	1.0000	0.0000	1.0000	0.0000	0.5000	0.0000	0.5000	0.2008	0.0335
9.0000	0.7000	20.5000	1.5000	1.0000	0.0000	1.0000	0.0000	0.0000	0.0000	1.0000	0.4500	0.0689
6.0000	0.4000	10.5500	1.2225	1.0000	0.0000	1.0000	0.0000	0.0000	0.0000	1.0000	NA	NA
8.6667	0.6667	17.8534	1.6964	1.0000	0.0000	1.0000	0.0000	0.0000	0.0000	1.0000	0.6605	0.0837
8.8571	0.6857	17.9467	1.8287	1.0000	0.0000	1.0000	0.0000	0.0000	0.0000	1.0000	1.4098	0.0991
7.6000	0.5600	17.6500	1.2665	1.0000	0.0000	0.8333	0.1667	0.3333	0.0000	0.6667	1.8281	0.1318
7.5000	0.5500	17.0600	1.6235	1.0000	0.0000	1.0000	0.0000	0.0000	0.0000	1.0000	0.3300	0.0490
9.2500	0.7250	20.2003	1.8317	1.0000	0.0000	1.0000	0.0000	0.0000	0.0000	1.0000	2.7928	0.1827
9.1250	0.7125	18.4769	1.4488	1.0000	0.0000	1.0000	0.0000	0.0000	0.1111	0.8889	0.5234	0.0676
8.0000	0.6000	19.9734	1.5657	0.8750	0.1250	1.0000	0.0000	0.3750	0.0000	0.6250	0.7306	0.0846
9.3333	0.7333	20.0780	1.8129	1.0000	0.0000	1.0000	0.0000	0.0000	0.0000	1.0000	1.5430	0.1262
9.0000	0.7000	19.4500	0.1000	1.0000	0.0000	1.0000	0.0000	0.0000	0.0000	1.0000	0.7050	0.0986
9.3750	0.7375	17.4700	1.5235	1.0000	0.0000	1.0000	0.0000	0.0000	0.1111	0.8889	1.3593	0.1147
9.1250	0.7125	19.3350	1.8477	1.0000	0.0000	1.0000	0.0000	0.2000	0.0000	0.8000	1.2058	0.1045
8.5000	0.6500	19.0953	1.5497	1.0000	0.0000	0.9000	0.1000	0.1000	0.0000	0.9000	1.2509	0.1073
8.1111	0.6111	17.0681	1.5026	1.0000	0.0000	0.9091	0.0000	0.1818	0.0909	0.7273	1.0616	0.0903
8.6667	0.6667	17.8833	1.1546	1.0000	0.0000	0.8333	0.1667	0.3333	0.0000	0.6667	1.8516	0.1348
8.9000	0.6900	20.8951	1.4048	1.0000	0.0000	0.9091	0.0000	0.0000	0.1818	0.8182	0.5232	0.0673
8.1111	0.6111	18.4600	1.3935	1.0000	0.0000	1.0000	0.0000	0.3000	0.0000	0.7000	1.1344	0.0944
7.6667	0.5667	17.5167	1.3589	1.0000	0.0000	1.0000	0.0000	0.3333	0.0000	0.6667	2.5005	0.1516
8.0000	0.6000	17.5167	1.3589	1.0000	0.0000	1.0000	0.0000	0.2500	0.0000	0.7500	2.3234	0.1613
8.0000	0.6000	16.9625	1.1397	1.0000	0.0000	0.8000	0.2000	0.4000	0.0000	0.6000	2.2019	0.1711
8.0000	0.6000	13.6500	1.3863	1.0000	0.0000	1.0000	0.0000	0.0000	0.0000	1.0000	4.4460	0.2892
7.5714	0.5571	17.4438	1.1524	1.0000	0.0000	1.0000	0.0000	0.3333	0.1111	0.5556	1.1581	0.0912

7.3333	0.5333	19.7550	1.0445	1.0000	0.0000	0.8000	0.2000	0.4000	0.0000	0.6000	0.6159	0.0843
7.7500	0.5750	18.7667	1.2017	1.0000	0.0000	0.7500	0.2500	0.2500	0.0000	0.7500	0.6919	0.0924
4.0000	0.2000	10.8000	1.1050	1.0000	0.0000	1.0000	0.0000	1.0000	0.0000	0.0000	0.2805	0.0458
5.0000	0.3000	13.0500	1.2092	1.0000	0.0000	1.0000	0.0000	0.6667	0.0000	0.3333	0.2753	0.0451
6.6667	0.4667	14.2901	1.5181	1.0000	0.0000	1.0000	0.0000	0.5000	0.0000	0.5000	0.5835	0.0788
8.0000	0.6000	18.4625	1.2556	1.0000	0.0000	0.7500	0.2500	0.2500	0.0000	0.7500	0.6964	0.0910
6.5000	0.4500	17.7977	1.9593	0.6667	0.3333	1.0000	0.0000	0.3333	0.0000	0.6667	0.2417	0.0389

6.3333	0.4333	13.9750	1.2944	1.0000	0.0000	1.0000	0.0000	0.5000	0.0000	0.5000	2.5502	0.1594
6.6667	0.4667	15.4500	1.2497	1.0000	0.0000	1.0000	0.0000	0.3333	0.0000	0.6667	0.2008	0.0335
4.0000	0.2000	14.3000	1.2025	1.0000	0.0000	1.0000	0.0000	1.0000	0.0000	0.0000	0.2753	0.0451
4.0000	0.2000	15.1250	0.6025	1.0000	0.0000	1.0000	0.0000	0.5000	0.0000	0.5000	0.4403	0.0665
6.0000	0.4000	14.1750	1.2613	1.0000	0.0000	1.0000	0.0000	0.5000	0.0000	0.5000	0.2700	0.0443
6.0000	0.4000	14.1750	1.2613	1.0000	0.0000	1.0000	0.0000	0.5000	0.0000	0.5000	0.2700	0.0443
7.2500	0.5250	17.8300	1.2098	1.0000	0.0000	0.8000	0.2000	0.4000	0.0000	0.6000	0.5102	0.0678
7.6000	0.5600	18.3100	1.1157	1.0000	0.0000	0.8750	0.1250	0.3750	0.0000	0.6250	0.4955	0.0695

6.6250	0.4625	23.5092	2.1601	1.0000	0.0000	0.8519	0.0741	0.2963	0.1111	0.5556	0.7522	0.0913
6.7917	0.4792	22.1201	2.0839	1.0000	0.0000	0.8519	0.0741	0.2963	0.1111	0.5926	0.7604	0.0922
6.8696	0.4870	22.3454	2.0126	1.0000	0.0000	0.8214	0.0714	0.3214	0.1071	0.5357	0.7585	0.0912
6.7143	0.4714	22.6749	2.3131	1.0000	0.0000	0.8235	0.0588	0.2647	0.1176	0.5882	0.7389	0.0901
6.8000	0.4800	22.6332	2.2678	1.0000	0.0000	0.8750	0.0000	0.2500	0.1667	0.5833	0.8406	0.0945
6.8636	0.4864	22.9897	2.4453	1.0000	0.0000	0.9583	0.0000	0.2083	0.1667	0.6250	0.8507	0.0956
6.7273	0.4727	21.0891	2.1677	1.0000	0.0000	0.9200	0.0000	0.2400	0.1200	0.6400	1.1309	0.1168
7.0455	0.5045	22.5704	2.0817	1.0000	0.0000	0.8800	0.0800	0.2400	0.1200	0.6400	0.9191	0.1082
7.3889	0.5389	21.2756	1.9418	1.0000	0.0000	0.9048	0.0476	0.2381	0.1429	0.6190	0.8873	0.1039
6.8400	0.4840	23.6881	2.0755	1.0000	0.0000	0.8214	0.1071	0.2500	0.1071	0.6071	1.3505	0.1168
6.8000	0.4800	22.2476	2.0939	1.0000	0.0000	0.8929	0.0357	0.2143	0.1071	0.6429	1.3036	0.1118
7.1481	0.5148	22.3015	2.1053	1.0000	0.0000	0.8333	0.0333	0.2333	0.1000	0.6333	1.4309	0.1203
7.0000	0.5000	23.4859	1.9517	1.0000	0.0000	0.7931	0.1034	0.2414	0.1724	0.5517	1.3467	0.1185
7.0345	0.5034	23.0533	2.0412	1.0000	0.0000	0.8182	0.0606	0.1818	0.1212	0.6667	0.9201	0.1068
6.6786	0.4679	23.0274	2.1386	1.0000	0.0000	0.8387	0.0645	0.2581	0.1290	0.5806	1.0706	0.1097
6.8750	0.4875	23.5970	2.3549	1.0000	0.0000	0.9048	0.0476	0.2857	0.0476	0.6667	1.5143	0.1226
7.2083	0.5208	24.7285	2.2232	0.9630	0.0370	0.8889	0.0741	0.1852	0.1481	0.6667	1.2724	0.1029
7.1500	0.5150	24.3673	2.4052	1.0000	0.0000	0.8800	0.0400	0.2800	0.0800	0.6400	0.8202	0.0995
7.0952	0.5095	24.2092	2.5618	1.0000	0.0000	0.9231	0.0000	0.1923	0.1538	0.6538	0.8939	0.1023
7.7647	0.5765	22.2647	2.1766	1.0000	0.0000	0.9474	0.0000	0.2105	0.2105	0.5789	0.9984	0.1095
7.4211	0.5421	22.0125	2.1129	1.0000	0.0000	0.9130	0.0435	0.2609	0.1739	0.5652	1.4044	0.1167
6.6500	0.4650	22.8180	2.2901	1.0000	0.0000	0.9130	0.0435	0.2174	0.0435	0.6957	1.6128	0.1297
6.4783	0.4478	23.1436	2.2569	1.0000	0.0000	0.8846	0.0769	0.1923	0.0769	0.6923	1.7821	0.1397
6.4348	0.4435	22.7226	2.4697	1.0000	0.0000	0.9259	0.0000	0.2222	0.0741	0.7037	1.2514	0.1267
6.6500	0.4650	21.8185	2.1940	1.0000	0.0000	0.9130	0.0000	0.2174	0.1304	0.6087	0.9242	0.1093
6.6000	0.4600	21.9002	2.1760	1.0000	0.0000	0.9130	0.0000	0.2174	0.1304	0.6087	0.9980	0.1164
6.8000	0.4800	21.6380	2.2231	1.0000	0.0000	0.9130	0.0000	0.1739	0.0870	0.6957	0.8173	0.0980
6.4583	0.4458	21.6772	2.1562	1.0000	0.0000	0.8889	0.0370	0.2222	0.0741	0.6667	1.4005	0.1207
6.5833	0.4583	22.6296	2.2139	1.0000	0.0000	0.8519	0.0370	0.1852	0.0741	0.7037	1.3778	0.1186
7.1250	0.5125	22.0853	2.2462	1.0000	0.0000	0.8214	0.0357	0.2143	0.1429	0.6071	0.8675	0.1043
7.5789	0.5579	22.5560	2.0360	0.9091	0.0909	0.9091	0.0455	0.2273	0.1364	0.5909	0.8383	0.0982
7.3684	0.5368	21.8764	2.1180	0.9091	0.0909	0.9091	0.0455	0.2727	0.0909	0.6364	0.8482	0.1016
7.2778	0.5278	22.4667	2.0726	0.9048	0.0952	0.9048	0.0476	0.2381	0.0952	0.6667	0.8185	0.0970
7.3158	0.5316	21.6237	2.0793	0.9565	0.0435	0.9130	0.0435	0.2609	0.1304	0.5652	0.9105	0.1072
7.2778	0.5278	22.3125	2.0299	1.0000	0.0000	0.9048	0.0476	0.2381	0.1905	0.5714	1.0172	0.1174
7.3000	0.5300	21.5532	1.8878	0.9167	0.0833	0.8750	0.0833	0.2083	0.1667	0.5833	0.8932	0.1075
7.3600	0.5360	22.9306	2.0654	0.9310	0.0690	0.8276	0.0690	0.2759	0.1034	0.6207	0.8354	0.1005
7.3500	0.5350	21.5420	2.0527	0.9167	0.0833	0.8750	0.0417	0.2917	0.1250	0.5833	0.8615	0.1029
7.3571	0.5357	22.4527	1.9822	0.9355	0.0645	0.8710	0.0645	0.2258	0.1290	0.6452	0.7746	0.0952
8.0000	0.6000	24.5837	2.0828	1.0000	0.0000	0.8462	0.0385	0.0769	0.1923	0.7308	0.5179	0.0674
7.4800	0.5480	23.8589	1.8338	1.0000	0.0000	0.8148	0.0741	0.2222	0.1481	0.6296	0.6331	0.0813
7.4074	0.5407	25.0479	2.0351	0.9333	0.0667	0.8000	0.1333	0.1667	0.1333	0.7000	1.4684	0.1070
7.3571	0.5357	24.0921	1.8846	0.9677	0.0323	0.7419	0.1290	0.2903	0.1290	0.5806	1.1455	0.1006
7.4194	0.5419	25.1750	1.8832	0.9706	0.0294	0.7647	0.1176	0.2353	0.1471	0.6176	1.0109	0.0916
7.1538	0.5154	24.1602	1.9235	1.0000	0.0000	0.7931	0.1034	0.2069	0.0690	0.6897	0.7544	0.0915
7.7692	0.5769	22.2055	1.9201	0.9643	0.0357	0.8571	0.0357	0.1786	0.1071	0.6786	0.5044	0.0632
7.9444	0.5944	22.5096	1.9514	0.9524	0.0476	0.9048	0.0476	0.1905	0.1905	0.6190	0.7122	0.0901
7.1739	0.5174	23.5782	1.9914	0.9615	0.0385	0.8462	0.1154	0.2308	0.1154	0.6538	1.2734	0.1065
7.6000	0.5600	23.7815	2.0675	0.9583	0.0417	0.8333	0.0833	0.2917	0.1250	0.5833	1.3122	0.1066
7.6538	0.5654	23.0545	1.8927	1.0000	0.0000	0.8621	0.0690	0.2759	0.1724	0.5517	1.2033	0.1049
7.5357	0.5536	23.0816	1.8893	0.9375	0.0625	0.8438	0.0625	0.2500	0.1875	0.5625	1.0793	0.0938
7.4000	0.5400	22.8562	1.8236	0.9643	0.0357	0.8571	0.0714	0.2500	0.1786	0.5714	1.2732	0.1095
7.4643	0.5464	21.8699	1.7890	0.9355	0.0645	0.8710	0.0645	0.2581	0.1613	0.5806	0.7302	0.0891
7.3704	0.5370	22.2315	1.9550	0.9677	0.0323	0.8387	0.0645	0.2903	0.1290	0.5806	0.7083	0.0886
7.2857	0.5286	22.2191	2.0493	0.9688	0.0313	0.8125	0.0625	0.2188	0.1563	0.6250	0.7281	0.0895
7.0000	0.5000	22.8015	1.8209	1.0000	0.0000	0.8696	0.0870	0.3043	0.1739	0.5217	1.3356	0.1091
7.3889	0.5389	21.1958	1.5557	1.0000	0.0000	0.8571	0.0952	0.2857	0.1905	0.5238	1.3636	0.1057
6.9000	0.4900	21.0368	1.6145	1.0000	0.0000	0.8636	0.0909	0.3636	0.1818	0.4545	1.3774	0.1115
6.6522	0.4652	23.9316	1.8552	0.9630	0.0370	0.8148	0.0741	0.2222	0.1481	0.6296	1.3196	0.1119

7.0370	0.5037	22.6089	1.8406	0.9333	0.0667	0.8333	0.0667	0.3000	0.2000	0.5000	1.1010	0.0937
6.0000	0.4000	27.7103	2.9295	1.0000	0.0000	0.8889	0.1111	0.6667	0.0000	0.3333	1.0751	0.1195
6.2500	0.4250	28.0953	2.7593	1.0000	0.0000	0.8889	0.1111	0.5556	0.0000	0.4444	0.8618	0.0967
8.5833	0.6583	23.4457	2.1353	0.9167	0.0833	0.9583	0.0417	0.1250	0.2083	0.6667	0.8094	0.0841
8.8095	0.6810	19.2203	1.5561	0.8095	0.1905	1.0000	0.0000	0.0476	0.0952	0.8571	0.7645	0.0892
8.6400	0.6640	22.5166	1.9972	0.9200	0.0800	1.0000	0.0000	0.1200	0.1200	0.7600	0.6621	0.0812
8.9231	0.6923	21.1342	1.7825	0.8462	0.1538	1.0000	0.0000	0.0769	0.0769	0.8462	4.3610	0.1297
8.9000	0.6900	21.8270	1.5765	0.9000	0.1000	1.0000	0.0000	0.1500	0.0500	0.8000	1.0947	0.1055
9.1111	0.7111	21.4223	1.3438	0.8889	0.1111	1.0000	0.0000	0.1111	0.1111	0.7778	0.6446	0.0743
6.5385	0.4538	27.1967	2.6414	0.9231	0.0769	0.8462	0.0769	0.2308	0.0000	0.7692	0.7547	0.0868
8.7647	0.6765	21.1642	1.8888	0.8824	0.1176	1.0000	0.0000	0.1176	0.1176	0.7647	4.9295	0.1840
8.3571	0.6357	21.1077	2.1646	0.9286	0.0714	1.0000	0.0000	0.1429	0.0714	0.7857	0.7990	0.0975
7.6667	0.5667	23.4963	2.2804	0.7778	0.2222	1.0000	0.0000	0.1111	0.0000	0.8889	0.5566	0.0671
9.2381	0.7238	22.9147	2.2937	0.9048	0.0952	0.9524	0.0000	0.0952	0.2381	0.6667	3.0067	0.1095
8.8333	0.6833	21.6780	2.0407	0.8333	0.1667	1.0000	0.0000	0.1667	0.0833	0.7500	4.8789	0.1333
9.7692	0.7769	24.2008	1.8439	0.9231	0.0769	1.0000	0.0000	0.0769	0.1538	0.6154	5.6791	0.1698
9.2000	0.7200	22.4848	2.2185	0.9000	0.1000	1.0000	0.0000	0.1000	0.1000	0.8000	6.0560	0.1712
10.0000	0.8000	29.4992	2.7611	0.9231	0.0769	0.9231	0.0000	0.0769	0.3077	0.4615	5.8575	0.1465
9.1667	0.7167	23.1718	2.2176	1.0000	0.0000	1.0000	0.0000	0.1667	0.1667	0.6667	1.5266	0.1109
7.6875	0.5688	21.8244	1.9963	0.8125	0.1875	0.9375	0.0625	0.1250	0.0000	0.8750	0.6654	0.0823
9.8889	0.7889	27.9803	1.8323	0.8889	0.1111	1.0000	0.0000	0.0000	0.2222	0.6667	7.8364	0.2166
9.5556	0.7556	26.2179	1.7239	1.0000	0.0000	1.0000	0.0000	0.1111	0.2222	0.5556	0.4889	0.0665
10.0667	0.8067	25.7582	2.4973	0.9333	0.0667	0.9333	0.0000	0.0000	0.5333	0.4000	0.9526	0.1029
7.1250	0.5125	26.6968	2.9018	1.0000	0.0000	0.8889	0.0000	0.3333	0.1111	0.5556	0.7302	0.0923
6.0000	0.4000	24.8849	2.7324	1.0000	0.0000	1.0000	0.0000	0.2222	0.0000	0.7778	0.7079	0.0860
8.2400	0.6240	21.2862	2.1407	0.9200	0.0800	0.9200	0.0800	0.1600	0.1200	0.6400	3.6248	0.1314
8.0000	0.6000	23.3916	2.2685	0.8421	0.1579	1.0000	0.0000	0.1579	0.1579	0.6842	3.0073	0.1002
8.1333	0.6133	24.4716	2.4138	0.8667	0.1333	1.0000	0.0000	0.0000	0.0667	0.9333	4.7461	0.1479
5.3333	0.3333	25.8364	3.0831	1.0000	0.0000	1.0000	0.0000	0.2000	0.0000	0.6000	1.9463	0.1627
5.5556	0.3556	30.6363	3.1387	1.0000	0.0000	0.9000	0.1000	0.3000	0.0000	0.7000	1.4824	0.1481
6.8000	0.4800	25.0049	2.2397	0.9375	0.0625	0.8750	0.1250	0.3125	0.0625	0.6250	0.5545	0.0724
6.1176	0.4118	26.0477	2.7749	0.9524	0.0476	0.9524	0.0000	0.2381	0.0000	0.7619	0.9556	0.1105
6.4000	0.4400	26.1485	2.6263	1.0000	0.0000	0.9412	0.0588	0.2353	0.0588	0.7059	0.8292	0.0981
6.5000	0.4500	27.5559	2.8902	1.0000	0.0000	0.9286	0.0000	0.2857	0.0714	0.6429	0.9981	0.1128
6.1667	0.4167	28.2414	2.7539	1.0000	0.0000	0.8333	0.1667	0.5833	0.0000	0.4167	1.1879	0.1109
6.9500	0.4950	25.3946	2.2760	0.9545	0.0455	0.9545	0.0455	0.3636	0.1364	0.5000	0.7844	0.0956
5.9286	0.3929	26.3594	2.4721	1.0000	0.0000	0.9412	0.0588	0.4706	0.0000	0.5294	1.2000	0.1319
6.5333	0.4533	25.2355	2.2716	0.9412	0.0588	0.9412	0.0588	0.4118	0.0588	0.5294	1.0971	0.1229
6.6316	0.4632	26.9404	2.5135	0.9524	0.0476	0.9524	0.0476	0.2381	0.0000	0.7619	0.9538	0.1093
7.0500	0.5050	26.5966	2.3005	1.0000	0.0000	0.9091	0.0909	0.2727	0.1364	0.5909	0.8148	0.0914
7.2143	0.5214	28.0494	2.9747	1.0000	0.0000	0.8750	0.0625	0.1875	0.1250	0.6875	0.7084	0.0888
6.2667	0.4267	25.2328	2.5919	1.0000	0.0000	0.8750	0.1250	0.3125	0.0000	0.6875	0.6411	0.0792
7.5000	0.5500	24.6962	2.2753	0.8462	0.1538	1.0000	0.0000	0.2308	0.0000	0.7692	1.2863	0.0940
7.5000	0.5500	26.3266	2.4902	1.0000	0.0000	1.0000	0.0000	0.3333	0.0000	0.6667	0.9032	0.1068
6.3125	0.4313	23.0097	2.3184	1.0000	0.0000	0.8824	0.1176	0.4706	0.0588	0.4706	0.9069	0.1043
7.7059	0.5706	24.9074	2.3915	0.9500	0.0500	1.0000	0.0000	0.2000	0.0500	0.7500	1.2674	0.1068
7.3636	0.5364	25.1227	2.4698	0.9167	0.0833	1.0000	0.0000	0.1667	0.0833	0.7500	0.6577	0.0839
7.8333	0.5833	24.2326	2.2000	0.8333	0.1667	1.0000	0.0000	0.0000	0.1667	0.8333	0.6425	0.0875
5.7143	0.3714	30.6022	2.6333	0.8750	0.1250	0.6250	0.0000	0.0000	0.0000	1.0000	3.1185	0.2218
8.1538	0.6154	22.1807	1.7612	0.9231	0.0769	0.9231	0.0769	0.2308	0.0769	0.6923	0.4285	0.0579
6.0000	0.4000	28.4179	2.5895	0.8750	0.1250	0.5625	0.0000	0.1875	0.0000	0.7500	2.7545	0.2144
6.3636	0.4364	27.5886	2.6937	1.0000	0.0000	0.8125	0.0625	0.4375	0.0000	0.5625	0.8827	0.0976
6.9545	0.4955	24.6662	2.0230	0.9545	0.0455	0.8636	0.1364	0.3182	0.0455	0.6364	1.3753	0.1184
7.0000	0.5000	27.7716	2.4225	1.0000	0.0000	0.9545	0.0455	0.2727	0.0455	0.6364	0.6522	0.0781
6.3333	0.4333	27.4771	2.9352	1.0000	0.0000	1.0000	0.0000	0.2143	0.0000	0.7857	0.6658	0.0821
7.3333	0.5333	24.7325	2.3092	0.9600	0.0400	1.0000	0.0000	0.2400	0.0000	0.7200	0.6310	0.0732
6.0000	0.4000	26.9633	2.3869	1.0000	0.0000	0.8750	0.1250	0.3750	0.0000	0.6250	0.8080	0.0934
7.0000	0.5000	23.9842	2.1276	0.9500	0.0500	0.9500	0.0000	0.2500	0.0500	0.6500	0.5750	0.0706
6.3000	0.4300	26.2333	2.6569	1.0000	0.0000	1.0000	0.0000	0.0909	0.0000	0.9091	0.7292	0.0865
8.1053	0.6105	24.8462	2.0241	0.8947	0.1053	0.8421	0.1053	0.2105	0.1053	0.6842	1.0946	0.0851
7.4444	0.5444	24.1429	2.3166	1.0000	0.0000	0.9500	0.0500	0.2500	0.1500	0.5500	0.5444	0.0693
7.0769	0.5077	27.0696	2.7590	1.0000	0.0000	1.0000	0.0000	0.1429	0.0714	0.7857	0.5720	0.0741
6.2353	0.4235	26.8008	2.5035	1.0000	0.0000	0.8889	0.0556	0.5000	0.0000	0.5000	1.0259	0.1158
7.4667	0.5467	22.1244	2.2387	0.9412	0.0588	1.0000	0.0000	0.2353	0.1176	0.5882	0.7117	0.0838
7.3077	0.5308	24.1133	2.3629	0.9286	0.0714	1.0000	0.0000	0.0714	0.1429	0.7857	0.6417	0.0833
7.5000	0.5500	22.8386	2.2732	0.8667	0.1333	0.9333	0.0667	0.3333	0.0000	0.6667	0.5282	0.0719
7.5294	0.5529	21.1557	2.2690	0.9412	0.0588	0.8824	0.0000	0.1765	0.0588	0.7647	0.6546	0.0778
8.0000	0.6000	26.1224	2.4038	0.8750	0.1250	0.9375	0.0000	0.1250	0.0625	0.6875	0.7831	0.0965
7.1429	0.5143	23.4422	2.4689	1.0000	0.0000	0.7857	0.0000	0.1429	0.1429	0.7143	1.4247	0.1259
7.2667	0.5267	27.5898	2.3390	1.0000	0.0000	0.7647	0.0588	0.2941	0.0588	0.5294	0.6830	0.0828
9.0000	0.7000	22.5448	1.8782	1.0000	0.0000	0.7500	0.0000	0.0833	0.0833	0.7500	0.6678	0.0831
7.2143	0.5214	24.4345	2.5019	0.9333	0.0667	0.8667	0.0667	0.3333	0.0000	0.6000	0.5527	0.0660
6.2353	0.4235	27.3341	2.4954	1.0000	0.0000	0.8889	0.0556	0.5000	0.0000	0.5000	0.9614	0.1086
7.7895	0.5789	23.6503	2.2404	0.9500	0.0500	0.8000	0.0000	0.1500	0.0000	0.8000	0.6614	0.0773
6.2273	0.4227	26.8242	2.5964	0.9583	0.0417	0.8750	0.0833	0.3333	0.0000	0.6667	0.6690	0.0811
7.3600	0.5360	25.2475	2.1134	0.9615	0.0385	0.8462	0.1154	0.3462	0.0385	0.6154	0.6056	0.0741
5.9091	0.3909	24.6202	2.6526	0.9231	0.0769	0.9231	0.0769	0.4615	0.0000	0.5385	0.8206	0.0941

6.8421	0.4842	24.0665	2.3595	0.9474	0.0526	0.7895	0.0526	0.4211	0.0000	0.5789	0.5877	0.0733
6.5556	0.4556	28.7685	2.4114	0.9000	0.1000	0.9000	0.1000	0.4000	0.0000	0.6000	0.7552	0.0907
5.6667	0.3667	32.6117	3.0708	1.0000	0.0000	0.8333	0.1667	0.6667	0.0000	0.3333	0.9450	0.1094
8.0000	0.6000	22.9493	2.3122	0.9000	0.1000	0.9000	0.0000	0.2000	0.0000	0.8000	0.3530	0.0492
6.6364	0.4636	26.9345	2.8469	1.0000	0.0000	0.9167	0.0833	0.4167	0.0000	0.5833	0.8109	0.0940
9.7778	0.7778	25.9938	1.5100	1.0000	0.0000	0.7778	0.0000	0.1111	0.2222	0.5556	0.5857	0.0780
8.8000	0.6800	20.2602	1.8155	0.8667	0.1333	0.9333	0.0667	0.2000	0.2000	0.5333	0.5679	0.0722
7.7647	0.5765	21.6506	2.1145	0.9474	0.0526	0.9474	0.0526	0.2632	0.0526	0.6842	0.4814	0.0636
6.0909	0.4091	27.2038	2.5075	1.0000	0.0000	0.9231	0.0769	0.1538	0.0000	0.8462	1.0487	0.1120
7.7500	0.5750	24.1509	2.4019	0.9286	0.0714	1.0000	0.0000	0.1429	0.0714	0.7857	0.6416	0.0820
8.8571	0.6857	18.6638	1.7200	0.8571	0.1429	0.9286	0.0000	0.1429	0.0714	0.7857	5.3250	0.1767
6.2000	0.4200	32.3800	2.9839	1.0000	0.0000	0.8750	0.1250	0.3750	0.0000	0.6250	0.9766	0.1083
9.3333	0.7333	21.5832	1.8014	0.9333	0.0667	1.0000	0.0000	0.0667	0.0667	0.8000	5.8262	0.1988
7.8000	0.5800	23.6169	2.4389	0.8667	0.1333	1.0000	0.0000	0.2000	0.0667	0.6667	0.6497	0.0820
7.4167	0.5417	25.9849	2.2812	0.9167	0.0833	1.0000	0.0000	0.0833	0.2500	0.6667	1.9764	0.1497
6.3333	0.4333	32.8028	3.4159	1.0000	0.0000	0.8750	0.1250	0.5000	0.0000	0.5000	1.0450	0.1168
8.3846	0.6385	19.7536	1.7205	0.9231	0.0769	0.9231	0.0769	0.1538	0.3077	0.5385	1.1858	0.0967
6.5294	0.4529	23.2865	2.2219	0.9412	0.0588	1.0000	0.0000	0.1765	0.0000	0.8235	0.9074	0.1036
7.1667	0.5167	24.7736	2.1559	0.8889	0.1111	0.9444	0.0556	0.2222	0.0556	0.6667	0.8394	0.0967
6.3333	0.4333	22.8334	2.4789	0.9167	0.0833	1.0000	0.0000	0.1667	0.0000	0.8333	0.6714	0.0790
7.0000	0.5000	23.5499	2.2022	0.9091	0.0909	1.0000	0.0000	0.1818	0.0000	0.8182	0.7160	0.0819
6.5000	0.4500	27.6925	2.7080	1.0000	0.0000	1.0000	0.0000	0.1111	0.0000	0.8889	0.8756	0.0998
7.0000	0.5000	26.9111	2.5240	1.0000	0.0000	0.9333	0.0667	0.3333	0.0667	0.6000	0.8006	0.0950
7.6875	0.5688	23.2676	2.3114	0.9412	0.0588	1.0000	0.0000	0.2353	0.0588	0.6471	0.7188	0.0851
8.1053	0.6105	25.1857	2.0521	0.9474	0.0526	1.0000	0.0000	0.0526	0.2105	0.7368	1.1251	0.1085
9.2000	0.7200	17.8101	2.1117	1.0000	0.0000	1.0000	0.0000	0.2000	0.0000	0.8000	1.9276	0.1452
9.1250	0.7125	18.9736	1.3141	0.8889	0.1111	0.8889	0.1111	0.1111	0.0000	0.8889	1.4939	0.1370
8.2857	0.6286	17.4272	1.5931	1.0000	0.0000	0.8750	0.0000	0.2500	0.1250	0.6250	1.3698	0.1159
9.0000	0.7000	17.7991	1.5221	1.0000	0.0000	0.8182	0.0909	0.1818	0.0909	0.7273	1.2976	0.1177
9.0000	0.7000	17.4125	1.5397	1.0000	0.0000	0.7143	0.1429	0.1429	0.1429	0.7143	1.6602	0.1389
9.1667	0.7167	17.9520	1.3457	1.0000	0.0000	0.8333	0.1667	0.3333	0.0000	0.6667	2.0215	0.1649
9.0000	0.7000	14.6842	1.4179	1.0000	0.0000	1.0000	0.0000	0.2500	0.0000	0.7500	2.0631	0.1392
8.2857	0.6286	15.0705	1.3717	1.0000	0.0000	1.0000	0.0000	0.2857	0.0000	0.7143	1.5393	0.1232
7.7143	0.5714	14.7087	1.2014	1.0000	0.0000	1.0000	0.0000	0.5714	0.0000	0.4286	1.2790	0.0962
8.4286	0.6429	18.0425	1.1673	0.8571	0.1429	0.8571	0.1429	0.4286	0.0000	0.5714	1.5436	0.1274
8.4444	0.6444	16.8796	1.3655	0.8889	0.1111	0.8889	0.1111	0.2222	0.0000	0.7778	1.4360	0.1254
9.0000	0.7000	17.9110	1.1967	0.8333	0.1667	0.8333	0.1667	0.3333	0.0000	0.6667	1.7642	0.1424
7.7143	0.5714	15.9796	1.3684	0.8571	0.1429	1.0000	0.0000	0.4286	0.0000	0.5714	1.3307	0.1029
8.4444	0.6444	16.7397	1.2987	0.9000	0.1000	0.9000	0.1000	0.4000	0.0000	0.6000	1.1602	0.1017
8.1818	0.6182	16.8397	1.2676	0.9091	0.0909	0.9091	0.0909	0.3636	0.0000	0.6364	1.0528	0.0922
8.8571	0.6857	17.4067	1.7688	0.8571	0.1429	1.0000	0.0000	0.2857	0.0000	0.7143	1.6656	0.1308
8.8750	0.6875	16.7880	1.8225	0.8750	0.1250	1.0000	0.0000	0.2500	0.0000	0.7500	1.5745	0.1316
8.1667	0.6167	16.5401	1.4203	0.8333	0.1667	1.0000	0.0000	0.3333	0.0000	0.6667	1.9808	0.1532
7.7143	0.5714	17.9729	1.2605	1.0000	0.0000	0.8750	0.0000	0.3750	0.2500	0.3750	1.3773	0.1175
8.5714	0.6571	20.1057	1.8611	0.8750	0.1250	1.0000	0.0000	0.2500	0.1250	0.6250	1.4583	0.1169
8.6667	0.6667	16.0250	1.2268	1.0000	0.0000	0.6667	0.0000	0.3333	0.3333	0.3333	2.7893	0.1894
7.2500	0.5250	16.3125	1.0803	0.7500	0.2500	0.7500	0.2500	0.5000	0.0000	0.5000	2.3664	0.1735
8.3333	0.6333	17.6667	0.8566	0.6667	0.3333	0.6667	0.3333	0.6667	0.0000	0.3333	0.8510	0.1104
8.0000	0.6000	14.2250	0.6816	0.8750	0.1250	0.8750	0.0000	0.5000	0.2500	0.2500	0.4678	0.0679
8.4000	0.6400	16.9025	1.5337	1.0000	0.0000	1.0000	0.0000	0.4000	0.0000	0.6000	0.4709	0.0652
7.2000	0.5200	15.3875	1.1447	1.0000	0.0000	1.0000	0.0000	0.6000	0.0000	0.4000	1.7473	0.1273
8.4000	0.6400	15.7425	1.3997	1.0000	0.0000	1.0000	0.0000	0.6000	0.0000	0.4000	0.4209	0.0582
8.2500	0.6250	14.5888	1.1640	0.7500	0.2500	1.0000	0.0000	0.5000	0.0000	0.5000	0.4827	0.0706
8.0000	0.6000	16.0730	1.4582	0.8333	0.1667	1.0000	0.0000	0.3333	0.0000	0.6667	0.6672	0.0912
7.7143	0.5714	16.4838	1.6214	0.8571	0.1429	1.0000	0.0000	0.4286	0.0000	0.5714	0.7304	0.0858
9.1667	0.7167	19.1120	1.4797	1.0000	0.0000	0.8333	0.1667	0.1667	0.0000	0.8333	1.8759	0.1548
9.1667	0.7167	18.7651	1.4747	1.0000	0.0000	0.8333	0.1667	0.1667	0.0000	0.8333	2.0995	0.1751
9.2000	0.7200	19.1120	1.4797	1.0000	0.0000	0.8000	0.2000	0.2000	0.0000	0.8000	2.1174	0.1668
8.4286	0.6429	16.3105	1.4673	1.0000	0.0000	0.8571	0.1429	0.2857	0.0000	0.7143	1.5860	0.1299
8.0000	0.6000	13.7131	1.3397	1.0000	0.0000	1.0000	0.0000	0.4000	0.0000	0.6000	1.9314	0.1397
9.0000	0.7000	16.6867	1.6329	1.0000	0.0000	0.7500	0.0000	0.2500	0.2500	0.5000	2.3920	0.1786
8.1667	0.6167	17.6700	1.2117	1.0000	0.0000	0.8333	0.1667	0.3333	0.0000	0.6667	1.7226	0.1381
8.2500	0.6250	19.2526	1.3422	1.0000	0.0000	0.7500	0.2500	0.5000	0.0000	0.5000	0.8143	0.1035
7.6667	0.5667	16.8525	1.4456	0.8333	0.1667	1.0000	0.0000	0.5000	0.0000	0.5000	0.5664	0.0793
9.0000	0.7000	16.8337	1.1889	1.0000	0.0000	0.8333	0.1667	0.5000	0.0000	0.5000	0.4223	0.0570
8.4286	0.6429	18.3005	1.2447	1.0000	0.0000	0.7143	0.1429	0.2857	0.0000	0.7143	0.5527	0.0758
8.5714	0.6571	17.7447	1.3569	1.0000	0.0000	0.8571	0.1429	0.4286	0.0000	0.5714	0.5605	0.0743
7.7143	0.5714	16.7146	1.1655	1.0000	0.0000	0.8571	0.1429	0.5714	0.0000	0.4286	0.4292	0.0599
7.8750	0.5875	16.7146	1.1655	1.0000	0.0000	0.8750	0.1250	0.5000	0.0000	0.5000	0.4590	0.0643
9.0000	0.7000	18.6610	1.1867	0.8333	0.1667	0.8333	0.1667	0.3333	0.0000	0.6667	0.6559	0.0893
7.6000	0.5600	15.9320	1.3352	1.0000	0.0000	0.8000	0.2000	0.4000	0.0000	0.6000	0.8294	0.1058
8.0000	0.6000	16.4086	1.2866	1.0000	0.0000	0.8571	0.1429	0.4286	0.0000	0.5714	0.6613	0.0876
7.7143	0.5714	16.4833	1.2135	1.0000	0.0000	0.7143	0.1429	0.2857	0.1429	0.5714	1.7446	0.1405
7.6667	0.5667	16.8221	1.2473	0.8571	0.1429	0.8571	0.1429	0.4286	0.0000	0.5714	0.6030	0.0828
7.5000	0.5500	16.5375	1.1414	1.0000	0.0000	0.8750	0.1250	0.5000	0.0000	0.5000	1.4411	0.1149
8.0000	0.6000	15.5513	1.3570	1.0000	0.0000	1.0000	0.0000	0.5000	0.0000	0.5000	1.4241	0.1140
8.5714	0.6571	17.8025	1.4337	1.0000	0.0000	0.8571	0.1429	0.2857	0.0000	0.7143	0.8191	0.1003

7.0000	0.5000	17.0026	1.5175	1.0000	0.0000	1.0000	0.0000	0.5000	0.0000	0.5000	0.5376	0.0744
8.0000	0.6000	16.7205	1.2617	1.0000	0.0000	0.8000	0.2000	0.4000	0.0000	0.6000	0.5168	0.0698
8.6667	0.6667	15.9405	1.0399	0.8333	0.1667	0.8333	0.1667	0.5000	0.0000	0.5000	0.5729	0.0771
8.1429	0.6143	15.5018	1.2500	0.8571	0.1429	0.8571	0.1429	0.5714	0.0000	0.4286	0.6071	0.0800
9.0000	0.7000	18.2013	1.1084	0.8000	0.2000	0.8000	0.2000	0.4000	0.0000	0.6000	0.6970	0.0933
8.6000	0.6600	16.5615	1.2139	0.6000	0.4000	0.8000	0.2000	0.4000	0.0000	0.6000	0.6699	0.0882
8.3333	0.6333	16.4830	1.5307	0.8333	0.1667	1.0000	0.0000	0.3333	0.0000	0.6667	0.6411	0.0890
7.8333	0.5833	16.6934	1.3627	1.0000	0.0000	0.8333	0.1667	0.3333	0.0000	0.6667	0.7535	0.0984
7.8333	0.5833	16.2434	1.3294	1.0000	0.0000	0.8571	0.1429	0.4286	0.0000	0.5714	0.8966	0.1096
7.7500	0.5750	17.8800	1.1617	1.0000	0.0000	0.8000	0.2000	0.6000	0.0000	0.4000	0.5675	0.0780
8.3333	0.6333	17.6667	0.8566	0.6667	0.3333	0.6667	0.3333	0.6667	0.0000	0.3333	0.8510	0.1104
8.0000	0.6000	17.0517	1.2699	0.8333	0.1667	0.8333	0.1667	0.5000	0.0000	0.5000	0.7473	0.0987
7.4000	0.5400	16.7125	1.2069	1.0000	0.0000	0.8000	0.2000	0.2000	0.0000	0.8000	0.9729	0.1163
8.0000	0.6000	15.8875	1.2131	1.0000	0.0000	1.0000	0.0000	0.5000	0.0000	0.5000	0.3067	0.0490
7.4000	0.5400	16.6583	1.1719	1.0000	0.0000	0.8333	0.1667	0.5000	0.0000	0.5000	0.5675	0.0780
9.0000	0.7000	16.7505	1.2167	1.0000	0.0000	0.8000	0.2000	0.4000	0.0000	0.6000	1.8307	0.1312
8.6000	0.6600	17.8596	1.5139	0.8000	0.2000	0.8000	0.2000	0.4000	0.0000	0.6000	1.0499	0.1152
6.5000	0.4500	15.3100	1.1055	0.8333	0.1667	1.0000	0.0000	0.3333	0.0000	0.6667	0.6365	0.0815
7.6667	0.5667	16.5114	1.3827	1.0000	0.0000	1.0000	0.0000	0.3750	0.0000	0.6250	0.5601	0.0741
7.6667	0.5667	19.0458	1.4251	1.0000	0.0000	0.8571	0.1429	0.1429	0.0000	0.8571	0.4730	0.0644
9.1250	0.7125	21.0507	1.9466	1.0000	0.0000	0.8889	0.1111	0.1111	0.0000	0.8889	1.5295	0.1286
9.0000	0.7000	20.1776	1.3832	0.8000	0.2000	0.8000	0.2000	0.2000	0.0000	0.8000	0.8432	0.1075
8.3000	0.6300	18.8745	1.3723	1.0000	0.0000	0.9091	0.0909	0.1818	0.0000	0.8182	1.2679	0.1098
8.0000	0.6000	21.5707	1.8495	1.0000	0.0000	1.0000	0.0000	0.1429	0.0000	0.8571	0.3816	0.0554
8.8571	0.6857	19.6450	1.9838	0.9000	0.1000	1.0000	0.0000	0.1000	0.0000	0.9000	0.4089	0.0573
9.1111	0.7111	21.6860	1.8039	1.0000	0.0000	0.8889	0.1111	0.1111	0.0000	0.8889	1.6704	0.1376
8.1429	0.6143	18.1683	1.6939	0.8750	0.1250	1.0000	0.0000	0.2500	0.0000	0.7500	1.2947	0.1066
9.0000	0.7000	16.2333	1.1000	1.0000	0.0000	0.6667	0.0000	0.0000	0.5000	0.3333	0.4537	0.0657
9.0000	0.7000	19.6967	1.4336	1.0000	0.0000	0.8571	0.1429	0.1429	0.0000	0.8571	1.5307	0.1258
7.5000	0.5500	16.4556	1.1408	0.9091	0.0909	0.8182	0.0909	0.4545	0.0909	0.4545	1.2342	0.1119
7.8333	0.5833	16.3200	0.9949	0.8571	0.1429	0.8571	0.1429	0.5714	0.0000	0.4286	0.7948	0.1009
7.6667	0.5667	15.6800	1.1562	1.0000	0.0000	0.8333	0.1667	0.3333	0.0000	0.6667	1.9771	0.1520
7.6667	0.5667	16.6583	1.1719	1.0000	0.0000	0.8571	0.1429	0.4286	0.0000	0.5714	0.7716	0.0968
8.0000	0.6000	18.5776	1.5125	1.0000	0.0000	0.6000	0.2000	0.2000	0.0000	0.6000	0.8249	0.1052
7.5000	0.5500	16.9643	1.4012	1.0000	0.0000	1.0000	0.0000	0.5714	0.0000	0.4286	2.0026	0.1408
7.6667	0.5667	15.8401	1.6000	1.0000	0.0000	0.8000	0.0000	0.4000	0.0000	0.4000	2.2126	0.1561
8.0000	0.6000	17.1375	1.1934	1.0000	0.0000	1.0000	0.0000	0.6000	0.0000	0.4000	1.9700	0.1461
7.3333	0.5333	15.1167	1.3183	1.0000	0.0000	1.0000	0.0000	0.5000	0.0000	0.5000	2.0796	0.1432
9.0000	0.7000	17.8000	1.3000	1.0000	0.0000	0.6667	0.0000	0.3333	0.0000	0.3333	0.4690	0.0696
NA	NA	NA	NA	1.0000	0.0000	0.0000	0.0000	0.0000	0.0000	0.0000	NA	NA
7.0000	0.5000	14.6500	1.3444	1.0000	0.0000	0.8000	0.0000	0.2000	0.0000	0.6000	2.6102	0.1676
7.5000	0.5500	17.7750	1.1113	1.0000	0.0000	0.5000	0.5000	0.0000	0.0000	1.0000	1.3692	0.1599
8.0000	0.6000	16.4500	0.9597	1.0000	0.0000	0.8000	0.2000	0.6000	0.0000	0.4000	0.5971	0.0811
NA	NA	NA	NA	1.0000	0.0000	0.0000	0.0000	0.0000	0.0000	0.0000	NA	NA
7.4000	0.5400	16.4508	1.3129	0.8333	0.1667	0.8333	0.1667	0.3333	0.0000	0.6667	1.9599	0.1490
7.6667	0.5667	16.0333	1.1802	1.0000	0.0000	0.8571	0.1429	0.4286	0.0000	0.5714	1.6926	0.1340
7.7500	0.5750	13.0500	1.0043	1.0000	0.0000	1.0000	0.0000	0.5000	0.0000	0.5000	0.8021	0.1006
9.0000	0.7000	16.8333	1.0779	1.0000	0.0000	1.0000	0.0000	0.5000	0.0000	0.5000	0.4465	0.0672
9.0000	0.7000	20.5000	1.5000	1.0000	0.0000	1.0000	0.0000	0.0000	0.0000	1.0000	0.4500	0.0689
7.4000	0.5400	16.7125	1.2069	1.0000	0.0000	0.8000	0.2000	0.2000	0.0000	0.8000	0.6919	0.0924
8.2000	0.6200	17.8017	1.3633	1.0000	0.0000	0.8333	0.1667	0.5000	0.0000	0.5000	0.6283	0.0832
8.4000	0.6400	16.7500	1.0175	1.0000	0.0000	0.8000	0.2000	0.2000	0.0000	0.8000	1.0073	0.1198
8.2500	0.6250	16.0375	1.2315	1.0000	0.0000	1.0000	0.0000	0.4000	0.0000	0.6000	0.4640	0.0698
9.5000	0.7500	18.7651	1.5801	1.0000	0.0000	1.0000	0.0000	0.2500	0.0000	0.7500	2.2223	0.1566
6.3333	0.4333	13.0500	1.2092	1.0000	0.0000	1.0000	0.0000	0.5000	0.0000	0.5000	0.7808	0.0935
7.6000	0.5600	18.2750	1.2582	1.0000	0.0000	0.8333	0.1667	0.3333	0.0000	0.6667	0.4981	0.0680
8.3333	0.6333	20.1833	1.2147	1.0000	0.0000	0.6667	0.3333	0.0000	0.0000	1.0000	0.7451	0.0906
7.8333	0.5833	17.0214	1.1118	1.0000	0.0000	0.8571	0.1429	0.5714	0.0000	0.4286	0.4515	0.0627
8.2000	0.6200	18.1000	1.0934	1.0000	0.0000	0.8333	0.1667	0.6667	0.0000	0.3333	0.4515	0.0627
7.6000	0.5600	15.7750	1.3248	1.0000	0.0000	1.0000	0.0000	0.2000	0.0000	0.8000	2.3234	0.1613
6.6667	0.4667	15.4500	1.2497	1.0000	0.0000	1.0000	0.0000	0.3333	0.0000	0.6667	0.2008	0.0335
8.5000	0.6500	19.7977	1.3611	1.0000	0.0000	1.0000	0.0000	0.2000	0.0000	0.8000	0.5654	0.0675
7.2500	0.5250	16.9300	1.3098	1.0000	0.0000	1.0000	0.0000	0.4000	0.0000	0.6000	0.2804	0.0450
6.5000	0.4500	15.6500	1.3025	1.0000	0.0000	1.0000	0.0000	0.5000	0.0000	0.5000	0.3653	0.0574
7.5000	0.5500	22.8663	1.8571	0.8462	0.1538	0.7692	0.0769	0.1538	0.0769	0.6923	0.5966	0.0699
6.8182	0.4818	22.3884	1.9374	0.8125	0.1875	0.8750	0.0000	0.1250	0.0000	0.8125	0.5710	0.0694
7.0000	0.5000	22.8815	2.0837	0.8235	0.1765	0.8824	0.0000	0.1765	0.0588	0.7059	0.5756	0.0700
8.1333	0.6133	20.4372	2.0704	0.7647	0.2353	0.9412	0.0000	0.1765	0.0588	0.7647	0.6583	0.0756
7.9000	0.5900	23.1781	2.1831	0.9167	0.0833	0.7917	0.0833	0.2500	0.0833	0.5833	0.5849	0.0687
7.6190	0.5619	21.8265	2.1549	0.8846	0.1154	0.8462	0.0385	0.2308	0.0769	0.6538	0.5876	0.0703
7.3333	0.5333	23.5586	2.2033	0.9706	0.0294	0.7941	0.1176	0.2941	0.0882	0.5588	1.0332	0.0881
7.6522	0.5652	22.3611	1.9174	0.8966	0.1034	0.8621	0.0690	0.2414	0.0345	0.6207	0.5041	0.0627
8.0000	0.6000	20.6796	1.7695	0.8571	0.1429	0.8571	0.0476	0.1429	0.0476	0.7143	0.5373	0.0662
8.1765	0.6176	20.8748	1.8701	0.8182	0.1818	0.9091	0.0000	0.0909	0.0455	0.7727	0.6091	0.0725

8.3571	0.6357	21.1212	1.9271	0.8824	0.1176	0.8824	0.0588	0.2353	0.0588	0.7059	0.5457	0.0654
7.8000	0.5800	21.8827	2.1590	0.8500	0.1500	0.8500	0.0000	0.2500	0.0500	0.6000	0.5608	0.0681
8.2308	0.6231	20.4014	1.9508	0.8824	0.1176	0.8824	0.0000	0.2353	0.0588	0.5882	0.5176	0.0619
7.8500	0.5850	22.7250	2.1957	0.8400	0.1600	0.8800	0.0400	0.2000	0.0400	0.6800	0.6070	0.0739
8.0000	0.6000	20.6754	1.7558	0.9048	0.0952	0.8095	0.0476	0.1905	0.0476	0.6667	0.6728	0.0795
8.3750	0.6375	19.7392	1.6345	0.9000	0.1000	0.8500	0.0500	0.2000	0.0500	0.6500	0.6924	0.0806
8.2941	0.6294	21.2068	1.9292	0.8500	0.1500	0.9500	0.0000	0.2000	0.0500	0.7500	0.5116	0.0646
7.8462	0.5846	23.0313	2.1258	0.8333	0.1667	0.7778	0.1111	0.2778	0.0556	0.5556	0.5630	0.0668
8.2308	0.6231	21.7730	1.7592	0.8235	0.1765	0.7647	0.0588	0.1176	0.0588	0.7647	0.6331	0.0760
7.3125	0.5313	20.8003	1.9309	0.8636	0.1364	0.8636	0.0000	0.1818	0.0455	0.6364	0.5254	0.0616
8.0000	0.6000	21.7641	1.4829	0.8824	0.1176	0.7059	0.0588	0.2941	0.0588	0.5882	0.7243	0.0846
7.3529	0.5353	21.0751	1.4788	0.8571	0.1429	0.7619	0.0476	0.2857	0.0952	0.5714	0.7862	0.0896
8.0000	0.6000	21.5941	2.0072	0.9167	0.0833	0.8333	0.0417	0.2083	0.0833	0.6250	0.6922	0.0802
7.4286	0.5429	20.4609	1.7233	0.8824	0.1176	0.7647	0.0588	0.3529	0.0000	0.5882	0.8958	0.1010
8.1667	0.6167	18.8002	1.3566	0.8182	0.1818	0.6364	0.0909	0.4545	0.0000	0.4545	0.5160	0.0628
7.5385	0.5538	19.8298	1.5431	0.9375	0.0625	0.6875	0.0625	0.3750	0.0625	0.5000	0.7544	0.0849
7.8000	0.5800	22.6678	1.6079	0.8500	0.1500	0.7500	0.1000	0.2000	0.1000	0.6000	0.7323	0.0851
7.8462	0.5846	19.8560	1.5727	0.8947	0.1053	0.7895	0.0526	0.2632	0.0526	0.5263	0.8092	0.0898
8.0000	0.6000	21.2099	1.4440	0.9167	0.0833	0.5833	0.0833	0.3333	0.0833	0.5000	0.5184	0.0631
7.9091	0.5909	20.1681	1.4299	0.9333	0.0667	0.6667	0.0667	0.2667	0.0667	0.6000	0.5994	0.0697
7.9286	0.5929	19.4028	1.4861	0.8947	0.1053	0.7895	0.0526	0.2632	0.1053	0.5789	0.7364	0.0846
8.1429	0.6143	20.0816	1.7995	0.8333	0.1667	0.8333	0.0556	0.1667	0.0556	0.6667	0.5760	0.0662
8.0556	0.6056	21.5100	2.0925	0.7727	0.2273	0.9545	0.0000	0.2727	0.0455	0.6818	2.8085	0.0986
7.7333	0.5733	20.9640	1.6500	0.9524	0.0476	0.8095	0.0476	0.2381	0.1429	0.5238	0.6444	0.0775
7.9286	0.5929	19.4916	1.7673	0.9500	0.0500	0.8500	0.0500	0.3000	0.0000	0.5500	0.9332	0.1048
7.3750	0.5375	20.7860	1.4815	0.9048	0.0952	0.7619	0.0476	0.3333	0.0476	0.5238	0.6998	0.0825
7.8462	0.5846	19.3680	1.4564	0.9444	0.0556	0.7778	0.0556	0.3889	0.0000	0.5000	0.9441	0.1035
8.0000	0.6000	23.0661	2.2114	0.9444	0.0556	0.7778	0.0556	0.2778	0.0556	0.6111	1.0171	0.1133
8.0769	0.6077	21.5724	1.5932	0.9444	0.0556	0.7778	0.1111	0.2778	0.0000	0.6111	0.8298	0.0945
7.4545	0.5455	20.7978	1.5669	0.8750	0.1250	0.8125	0.0625	0.1875	0.0000	0.6875	0.6250	0.0749
8.1111	0.6111	20.8780	1.6157	0.9286	0.0714	0.7857	0.0714	0.1429	0.0000	0.7143	0.6962	0.0822
8.0000	0.6000	20.2146	1.9126	0.8947	0.1053	0.8947	0.0000	0.1579	0.0526	0.6842	0.6912	0.0803
7.7857	0.5786	20.5744	1.5531	0.9048	0.0952	0.8095	0.0952	0.3333	0.0000	0.5714	0.8112	0.0950
8.0000	0.6000	19.2641	1.5749	0.9524	0.0476	0.8095	0.0476	0.2381	0.0952	0.5714	0.7264	0.0873
8.1538	0.6154	23.0044	2.0745	0.8824	0.1176	0.8235	0.0588	0.1765	0.0000	0.7059	3.8105	0.1207
8.1333	0.6133	21.1580	1.6499	0.9474	0.0526	0.8421	0.0526	0.3158	0.0000	0.6316	1.0116	0.1120
7.8235	0.5824	21.9397	2.0389	0.8571	0.1429	0.8571	0.0476	0.1905	0.0952	0.6667	4.9588	0.1513
8.2500	0.6250	19.5496	1.6162	0.9412	0.0588	0.8235	0.0588	0.2353	0.0000	0.6471	0.9044	0.1024
7.8462	0.5846	22.1058	1.8314	0.9474	0.0526	0.8421	0.0526	0.2632	0.0000	0.6316	0.8339	0.0961
7.9286	0.5929	22.6014	2.0433	0.9444	0.0556	0.7778	0.0556	0.2778	0.0000	0.6111	0.6031	0.0726
8.0000	0.6000	20.7222	1.8068	0.8889	0.1111	0.8889	0.0000	0.2778	0.1111	0.5000	0.4764	0.0582
7.7500	0.5750	22.1408	2.1130	0.8750	0.1250	0.7500	0.0625	0.2813	0.0938	0.5625	3.6025	0.1259
8.1333	0.6133	21.2987	2.1298	0.9474	0.0526	0.8947	0.0000	0.2632	0.0526	0.6316	0.6387	0.0777
8.1333	0.6133	20.6655	1.9748	0.9444	0.0556	0.8889	0.0000	0.1667	0.0556	0.7222	0.6582	0.0773
8.2105	0.6211	23.3368	2.0869	0.9583	0.0417	0.8750	0.0417	0.2500	0.0417	0.6250	0.5897	0.0744
7.3333	0.5333	22.0607	1.7371	0.9286	0.0714	0.6429	0.0714	0.3571	0.0714	0.4286	0.4401	0.0623
6.6667	0.4667	19.0158	1.7037	0.9091	0.0909	0.8182	0.0000	0.4545	0.0909	0.3636	0.4137	0.0571
7.9167	0.5917	20.7715	1.5554	0.9375	0.0625	0.8125	0.0625	0.2500	0.0625	0.6250	0.6533	0.0802
8.5000	0.6500	21.5801	1.6686	0.9000	0.1000	0.8000	0.1000	0.3000	0.0000	0.7000	0.3427	0.0478
8.2727	0.6273	18.8049	1.6340	0.8462	0.1538	0.8462	0.0000	0.1538	0.0769	0.7692	0.3955	0.0497
8.7500	0.6750	21.2796	1.7060	1.0000	0.0000	0.8889	0.0000	0.0000	0.0000	1.0000	0.5018	0.0618
8.4167	0.6417	22.3463	1.6030	0.9412	0.0588	0.7059	0.0588	0.2353	0.0588	0.5882	1.1225	0.1023
8.3846	0.6385	20.9024	1.5585	0.9375	0.0625	0.8125	0.1250	0.3125	0.0000	0.6875	0.6837	0.0829
8.3750	0.6375	19.7796	1.2523	0.9091	0.0909	0.8182	0.0909	0.3636	0.0000	0.6364	1.0046	0.1122
8.8750	0.6875	22.7734	1.6686	0.9000	0.1000	0.7000	0.1000	0.2000	0.1000	0.6000	0.6796	0.0786
8.6667	0.6667	22.0363	1.5364	0.9091	0.0909	0.6364	0.0909	0.0909	0.0909	0.7273	0.5358	0.0638
8.6667	0.6667	20.0100	1.4692	0.9444	0.0556	0.7222	0.0556	0.1667	0.1111	0.6667	0.5958	0.0683
8.6000	0.6600	19.0929	1.5606	0.9231	0.0769	0.8462	0.0000	0.1538	0.0769	0.7692	1.1403	0.1009
8.4286	0.6429	20.5501	1.7089	0.8235	0.1765	0.8824	0.0000	0.1176	0.0588	0.7647	0.4920	0.0616
8.3333	0.6333	21.6359	1.5508	0.9444	0.0556	0.8333	0.1111	0.2778	0.0000	0.7222	0.9610	0.0922
8.6429	0.6643	20.2494	1.5388	0.9333	0.0667	0.8667	0.0667	0.1333	0.0000	0.8667	1.1335	0.1009
8.5455	0.6545	19.4739	1.6219	0.8333	0.1667	0.8333	0.0833	0.0833	0.0000	0.9167	5.1045	0.1543
8.5556	0.6556	20.0146	1.5530	0.9091	0.0909	0.8182	0.0909	0.0909	0.0000	0.9091	1.2284	0.1069
8.7000	0.6700	20.3708	1.3103	0.9167	0.0833	0.6667	0.0833	0.0833	0.0833	0.7500	1.1729	0.0997
6.6667	0.4667	19.8775	1.2421	0.9000	0.1000	0.6000	0.1000	0.4000	0.0000	0.4000	0.2913	0.0392
8.0000	0.6000	17.4566	1.0411	0.7500	0.2500	0.7500	0.1250	0.5000	0.0000	0.5000	0.6836	0.0826
8.4000	0.6400	18.1390	1.0575	0.7778	0.2222	0.7778	0.1111	0.3333	0.0000	0.6667	0.5889	0.0697
8.2857	0.6286	18.0619	1.0811	0.8889	0.1111	0.7778	0.1111	0.3333	0.0000	0.6667	1.4871	0.1237
7.6667	0.5667	17.6877	1.1323	0.7273	0.2727	0.7273	0.0909	0.2727	0.0000	0.6364	0.4135	0.0572
7.5714	0.5571	17.4525	1.0329	0.7273	0.2727	0.8182	0.0909	0.3636	0.0000	0.6364	0.6426	0.0772
7.8750	0.5875	17.7902	1.0332	0.8333	0.1667	0.7500	0.0833	0.5000	0.0000	0.5000	0.7320	0.0833
7.6667	0.5667	17.5823	1.0488	0.7778	0.2222	0.7778	0.1111	0.3333	0.0000	0.6667	0.5928	0.0703
8.2857	0.6286	21.4943	1.2990	1.0000	0.0000	0.8000	0.0000	0.3000	0.1000	0.6000	0.6826	0.0727
8.0000	0.6000	17.6557	1.0248	0.9000	0.1000	0.8000	0.1000	0.4000	0.0000	0.6000	0.6372	0.0741
7.6667	0.5667	17.9806	1.0736	0.8000	0.2000	0.8000	0.1000	0.3000	0.0000	0.7000	0.6353	0.0749
7.3333	0.5333	15.9931	0.9620	0.6667	0.3333	0.7778	0.1111	0.3333	0.0000	0.6667	0.6638	0.0796
7.4444	0.5444	19.4248	1.0254	0.8462	0.1538	0.6154	0.0769	0.3846	0.0769	0.4615	0.8061	0.0932

8.5833	0.6583	20.2259	1.3827	0.8667	0.1333	0.8000	0.0667	0.2000	0.0000	0.7333	4.7155	0.1627
7.8571	0.5857	18.9817	1.0346	0.8889	0.1111	0.7778	0.1111	0.4444	0.1111	0.4444	0.8673	0.1031
8.1667	0.6167	17.6557	1.0248	0.8889	0.1111	0.7778	0.1111	0.4444	0.0000	0.5556	0.7015	0.0812
7.5714	0.5571	18.0661	1.0911	0.8182	0.1818	0.7273	0.0909	0.3636	0.0000	0.5455	0.6706	0.0792
8.1250	0.6125	20.7919	1.0141	0.9000	0.1000	0.7000	0.1000	0.4000	0.1000	0.5000	0.7158	0.0843
7.8750	0.5875	19.9817	1.0248	0.9091	0.0909	0.6364	0.0909	0.4545	0.0909	0.4545	0.8434	0.0960
8.1667	0.6167	18.2493	1.0141	0.8750	0.1250	0.7500	0.1250	0.3750	0.0000	0.6250	0.7855	0.0907
8.1429	0.6143	17.6557	1.0248	0.9000	0.1000	0.8000	0.1000	0.5000	0.0000	0.5000	0.6324	0.0733
8.1429	0.6143	18.2493	1.0141	0.8889	0.1111	0.7778	0.1111	0.4444	0.0000	0.5556	0.6993	0.0808
8.1818	0.6182	22.3690	1.1498	0.9286	0.0714	0.6429	0.1429	0.3571	0.0714	0.5000	0.6717	0.0824
8.2222	0.6222	20.7919	1.0141	0.9091	0.0909	0.7273	0.0909	0.3636	0.0909	0.5455	0.7243	0.0866
7.3636	0.5364	18.1251	1.0675	0.8571	0.1429	0.7143	0.0714	0.4286	0.0714	0.5000	0.6883	0.0794
8.2222	0.6222	19.1838	1.1110	0.9167	0.0833	0.7500	0.0833	0.4167	0.0833	0.5000	0.6003	0.0709
8.3571	0.6357	20.8178	1.2044	0.9375	0.0625	0.8125	0.0625	0.2500	0.0625	0.6875	1.1812	0.1114
8.2500	0.6250	22.2007	1.2213	0.9286	0.0714	0.7143	0.0714	0.2143	0.0714	0.6429	0.6350	0.0787
7.7500	0.5750	21.0873	1.1888	0.9286	0.0714	0.7857	0.0714	0.3571	0.0714	0.5714	0.5887	0.0715
7.4545	0.5455	17.7076	1.0106	0.8571	0.1429	0.7857	0.0000	0.5000	0.0714	0.4286	0.7857	0.0881
8.0769	0.6077	19.9613	1.1079	0.9375	0.0625	0.6875	0.0625	0.3125	0.0625	0.5625	0.7049	0.0828
8.5500	0.6550	22.1318	1.2597	0.9524	0.0476	0.6667	0.0952	0.2381	0.1429	0.5714	1.1427	0.1089
8.1667	0.6167	20.6940	1.2838	0.9333	0.0667	0.7333	0.0667	0.2667	0.0667	0.6000	0.5950	0.0750
8.0000	0.6000	20.3302	1.2499	0.9375	0.0625	0.6875	0.0625	0.3125	0.1250	0.5000	0.6430	0.0773
8.3077	0.6308	18.8973	1.0898	0.9375	0.0625	0.8125	0.0000	0.3125	0.0625	0.5625	0.6797	0.0798
8.4000	0.6400	21.6546	1.3215	1.0000	0.0000	0.7857	0.0000	0.2143	0.1429	0.6429	0.8752	0.1024
7.5556	0.5556	24.5629	1.7362	1.0000	0.0000	0.7500	0.1667	0.1667	0.0833	0.6667	1.7960	0.1137
7.7333	0.5733	23.1230	1.7742	0.8889	0.1111	0.8889	0.0556	0.1111	0.2222	0.5556	0.5525	0.0710
7.7143	0.5714	23.3559	1.7958	1.0000	0.0000	0.8000	0.1000	0.1000	0.0000	0.8000	0.6430	0.0786
7.5000	0.5500	23.7282	1.7251	0.9333	0.0667	0.8000	0.1333	0.1333	0.2000	0.6000	1.5249	0.1045
7.6000	0.5600	22.6465	1.7941	0.9444	0.0556	0.8333	0.1111	0.1111	0.2222	0.6111	1.4777	0.1079
7.6667	0.5667	23.7285	1.8957	0.9167	0.0833	0.8333	0.0833	0.0833	0.2500	0.6667	0.4816	0.0619
7.6250	0.5625	26.4471	1.6680	0.9167	0.0833	0.8333	0.0833	0.0833	0.2500	0.5833	0.5841	0.0724
7.5000	0.5500	23.3340	2.0141	0.9444	0.0556	0.8333	0.0556	0.1667	0.2778	0.5000	0.7345	0.0891
7.5833	0.5583	22.0526	1.8698	0.9375	0.0625	0.8750	0.0625	0.0625	0.1250	0.7500	0.6370	0.0794
7.5833	0.5583	24.5112	2.1262	1.0000	0.0000	0.8750	0.0000	0.1875	0.1250	0.6250	0.7848	0.0950
7.7778	0.5778	27.0461	2.1203	1.0000	0.0000	0.8000	0.1000	0.3000	0.2000	0.4000	0.6770	0.0857
7.3846	0.5385	24.6728	2.2250	0.9375	0.0625	0.8125	0.0625	0.1875	0.1875	0.6250	0.7090	0.0861
7.3636	0.5364	23.5598	2.3937	0.9286	0.0714	0.9286	0.0000	0.0000	0.2143	0.7143	0.6038	0.0739
7.3636	0.5364	26.5104	2.0623	0.9286	0.0714	0.8571	0.0714	0.1429	0.2143	0.5714	0.4627	0.0607
7.5000	0.5500	25.3281	2.1297	1.0000	0.0000	0.9167	0.0000	0.0833	0.1667	0.7500	0.5817	0.0709
7.2000	0.5200	25.1595	2.4206	1.0000	0.0000	0.9231	0.0000	0.0769	0.1538	0.6923	0.6290	0.0755
8.4706	0.6471	20.5558	1.2209	0.9444	0.0556	0.8333	0.0000	0.1667	0.3333	0.5000	0.8915	0.1058
8.8182	0.6818	19.2041	1.2677	1.0000	0.0000	1.0000	0.0000	0.0000	0.2727	0.7273	1.1975	0.1098
8.3077	0.6308	17.7885	1.2340	0.9286	0.0714	1.0000	0.0000	0.2143	0.2143	0.5714	0.7946	0.0936
8.4706	0.6471	20.6760	1.3674	0.8889	0.1111	0.8889	0.0556	0.1667	0.2222	0.6111	1.1177	0.1070
8.3125	0.6313	19.3138	1.3523	0.9412	0.0588	0.9412	0.0000	0.1765	0.1765	0.5882	0.8249	0.0984
8.2500	0.6250	19.8024	1.4040	0.8824	0.1176	0.9412	0.0000	0.1765	0.2353	0.5882	0.7771	0.0931
8.2308	0.6231	18.7812	1.2092	0.9286	0.0714	0.9286	0.0000	0.2143	0.2143	0.5714	1.3744	0.1266
8.3333	0.6333	19.5667	1.3016	0.8824	0.1176	0.8824	0.0000	0.1176	0.3529	0.5294	0.7592	0.0919
8.2727	0.6273	18.5202	1.3475	0.9167	0.0833	0.9167	0.0000	0.2500	0.1667	0.5833	0.8811	0.1005
8.4286	0.6429	18.5202	1.3475	0.9333	0.0667	0.8667	0.0000	0.2000	0.2000	0.6000	0.8942	0.1045
8.3333	0.6333	19.4813	1.4988	0.8750	0.1250	0.9375	0.0000	0.1250	0.1875	0.6250	1.2109	0.1122
8.5000	0.6500	20.8234	1.5654	0.9444	0.0556	0.9444	0.0556	0.1667	0.1111	0.7222	1.0830	0.1077
7.8000	0.5800	23.9563	1.9471	0.8824	0.1176	0.8824	0.0000	0.0000	0.2353	0.7647	0.6028	0.0731
7.5556	0.5556	24.9523	2.1930	0.9091	0.0909	0.9091	0.0000	0.0000	0.1818	0.7273	0.6285	0.0766
7.9000	0.5900	24.6704	2.2275	0.9286	0.0714	0.9286	0.0000	0.0000	0.1429	0.7857	0.7697	0.0920
7.6667	0.5667	24.6822	2.3531	0.9091	0.0909	1.0000	0.0000	0.0909	0.1818	0.7273	0.6034	0.0816
7.5000	0.5500	24.6299	2.2014	0.9231	0.0769	0.9231	0.0000	0.0769	0.1538	0.7692	0.9564	0.1061
7.3333	0.5333	23.7307	2.2543	1.0000	0.0000	0.9167	0.0000	0.0833	0.0833	0.7500	0.8829	0.1054
6.8889	0.4889	26.1795	2.5455	0.9091	0.0909	0.9091	0.0000	0.1818	0.0909	0.7273	0.6557	0.0790
6.8750	0.4875	27.4023	2.5055	0.9091	0.0909	0.9091	0.0000	0.1818	0.1818	0.6364	0.6590	0.0803
7.0000	0.5000	29.1779	2.7477	0.8889	0.1111	1.0000	0.0000	0.1111	0.2222	0.5556	0.5657	0.0704
7.1875	0.5188	24.1078	2.2285	0.9474	0.0526	1.0000	0.0000	0.1053	0.2105	0.6316	0.6477	0.0809
7.2727	0.5273	25.7384	2.3799	0.9286	0.0714	0.9286	0.0000	0.1429	0.1429	0.7143	0.6506	0.0810
7.1333	0.5133	24.1442	2.3029	0.8889	0.1111	0.8333	0.0556	0.1667	0.1667	0.6667	0.5243	0.0672
7.3333	0.5333	24.1516	1.8050	0.9375	0.0625	0.8750	0.0625	0.1875	0.1875	0.6250	0.6953	0.0843
7.1333	0.5133	23.9223	1.9480	0.9474	0.0526	0.7895	0.0526	0.2105	0.2105	0.5263	0.6062	0.0771
7.4615	0.5462	25.4876	1.8627	0.9333	0.0667	0.9333	0.0667	0.2000	0.2000	0.5333	0.8372	0.0993
6.7500	0.4750	25.0866	2.3447	1.0000	0.0000	0.8000	0.0667	0.3333	0.1333	0.5333	0.5780	0.0765
6.4000	0.4400	25.8729	2.8001	0.9444	0.0556	0.9444	0.0000	0.2778	0.1111	0.6111	0.8722	0.1016
6.8462	0.4846	23.9951	2.3316	0.9375	0.0625	0.8750	0.0000	0.1875	0.1875	0.6250	0.6515	0.0807
6.7500	0.4750	24.6733	2.3773	1.0000	0.0000	0.9333	0.0000	0.2667	0.2000	0.5333	0.7952	0.0917
6.6429	0.4643	25.5827	2.6245	0.9375	0.0625	0.8750	0.0625	0.3125	0.1250	0.5625	0.7469	0.0908
7.0909	0.5091	26.3386	2.2242	0.9231	0.0769	0.9231	0.0769	0.2308	0.2308	0.5385	0.6098	0.0761
7.1111	0.5111	22.7738	2.2497	0.9500	0.0500	0.9000	0.0500	0.2000	0.1500	0.6000	0.7537	0.0904
6.8571	0.4857	26.6911	2.2553	1.0000	0.0000	1.0000	0.0000	0.1250	0.2500	0.6250	0.3538	0.0520
6.5714	0.4571	25.7662	2.3963	1.0000	0.0000	1.0000	0.0000	0.2222	0.1111	0.6667	0.5629	0.0756
6.7778	0.4778	25.0102	2.2252	1.0000	0.0000	0.9091	0.0000	0.2727	0.1818	0.5455	0.6300	0.0765
6.6667	0.4667	24.4446	2.6960	1.0000	0.0000	1.0000	0.0000	0.1250	0.1250	0.7500	0.6982	0.0882

6.7778	0.4778	26.0307	2.3820	1.0000	0.0000	1.0000	0.0000	0.1667	0.1667	0.6667	0.8403	0.0954
NA	NA	24.0000	NA	0.0000	1.0000	1.0000	0.0000	0.0000	0.0000	1.0000	NA	NA
7.6667	0.5667	21.8732	1.5667	0.7143	0.2857	1.0000	0.0000	0.0000	0.5714	0.4286	0.6307	0.0857
7.9000	0.5900	22.1713	2.1087	0.8182	0.1818	1.0000	0.0000	0.0909	0.3636	0.5455	0.7523	0.0906
9.1429	0.7143	20.6752	1.4752	0.8750	0.1250	0.8750	0.1250	0.1250	0.2500	0.6250	0.6484	0.0861
8.0000	0.6000	21.3639	1.6766	0.6000	0.4000	1.0000	0.0000	0.0000	0.2000	0.8000	0.0473	0.0084
8.6923	0.6692	17.3589	1.5949	0.8571	0.1429	0.9286	0.0000	0.0714	0.2143	0.6429	0.5123	0.0656
8.8889	0.6889	22.1665	1.7485	0.7778	0.2222	1.0000	0.0000	0.0000	0.2222	0.7778	6.3586	0.1423
9.0000	0.7000	17.3531	1.7166	1.0000	0.0000	1.0000	0.0000	0.0000	0.0909	0.8182	0.9675	0.0760
8.3333	0.6333	25.4955	1.6590	1.0000	0.0000	0.9375	0.0625	0.0625	0.1250	0.7500	0.4941	0.0676
9.2500	0.7250	18.8069	1.4703	0.9167	0.0833	1.0000	0.0000	0.0833	0.1667	0.6667	0.7875	0.0853
10.0000	0.8000	22.6949	1.4609	1.0000	0.0000	1.0000	0.0000	0.0000	0.0000	1.0000	0.3285	0.0504
9.4000	0.7400	20.4405	1.7733	1.0000	0.0000	1.0000	0.0000	0.0000	0.3333	0.6667	0.4922	0.0679
8.2353	0.6235	19.6765	1.9994	0.9444	0.0556	0.8333	0.0556	0.1667	0.1111	0.7222	0.4430	0.0603
8.4118	0.6412	20.6241	2.1112	0.9444	0.0556	0.8333	0.0556	0.1111	0.1667	0.7222	1.3370	0.0850
7.9444	0.5944	21.2828	2.1763	0.9500	0.0500	0.9000	0.0500	0.1000	0.1000	0.8000	0.5391	0.0698
8.1429	0.6143	20.7020	2.1655	1.0000	0.0000	0.8750	0.0417	0.0833	0.1667	0.7083	0.5112	0.0672
8.2222	0.6222	20.3490	2.1137	0.9444	0.0556	0.9444	0.0556	0.1111	0.2222	0.6667	0.6760	0.0815
8.2941	0.6294	22.0752	2.2831	0.9412	0.0588	0.8824	0.0588	0.1176	0.1176	0.7647	1.5917	0.1069
8.2632	0.6263	22.4908	2.2867	1.0000	0.0000	0.9048	0.0476	0.0952	0.0952	0.8095	1.3584	0.1017
8.3333	0.6333	22.6190	2.1201	1.0000	0.0000	0.8500	0.0500	0.0500	0.1500	0.8000	1.4938	0.1091
7.8000	0.5800	24.4451	2.6970	0.9375	0.0625	0.8125	0.0625	0.2500	0.0625	0.6875	0.6864	0.0833
7.9091	0.5909	25.0728	2.4290	0.9286	0.0714	0.7143	0.0714	0.2143	0.0714	0.6429	0.4141	0.0558
8.3333	0.6333	23.0349	2.5529	0.8750	0.1250	0.8750	0.0000	0.1250	0.0000	0.8750	0.7524	0.0853
8.7500	0.6750	23.9872	2.4966	0.8182	0.1818	0.8182	0.0000	0.1818	0.0909	0.7273	0.6306	0.0730
7.7222	0.5722	26.5326	2.2869	0.9500	0.0500	0.8500	0.1000	0.1000	0.2000	0.7000	0.8042	0.0909
7.6957	0.5696	24.4606	2.1685	1.0000	0.0000	0.8889	0.0370	0.0741	0.1481	0.7407	0.5776	0.0749
7.6154	0.5615	24.4633	2.1286	0.9333	0.0667	0.8667	0.0667	0.1333	0.2000	0.6667	0.9125	0.0976
7.9048	0.5905	23.8268	2.1213	1.0000	0.0000	0.8750	0.0417	0.1667	0.1667	0.6667	0.6710	0.0871
8.0556	0.6056	23.0975	2.0605	1.0000	0.0000	1.0000	0.0000	0.0526	0.2105	0.7368	0.6094	0.0789
8.1818	0.6182	24.2254	1.9795	1.0000	0.0000	0.8846	0.0385	0.0385	0.2308	0.6923	0.5184	0.0699
8.3000	0.6300	27.9914	2.2439	1.0000	0.0000	1.0000	0.0000	0.0000	0.1667	0.7500	0.5785	0.0774
8.2632	0.6263	22.2159	2.1649	1.0000	0.0000	0.9000	0.0500	0.1000	0.2000	0.7000	0.4776	0.0661

15 snb	15 snb.s	16 seed buoy	17 autochor_	18 bythisoch	19 chamaech	20 hemeroch	21 meteoroc	22 nautocho	23 ombroch	24 other_1	25 zoochor_	26 inverb_1
1.7895	0.3947	62.5068	0.0800	0.0000	0.0000	0.3600	0.2800	0.8800	0.0000	0.1600	0.5200	0.0000
1.6429	0.3214	64.5054	0.2778	0.0000	0.0000	0.2778	0.1667	0.8333	0.0000	0.1667	0.6667	0.0000
2.1111	0.5556	70.6460	0.0769	0.0000	0.0000	0.3077	0.3077	0.8462	0.0000	0.1538	0.6154	0.0000
1.9286	0.4643	72.7105	0.1429	0.0000	0.0000	0.5000	0.5000	1.0000	0.0714	0.3571	0.5000	0.0000
1.7000	0.3500	66.0369	0.0952	0.0000	0.0000	0.3333	0.2381	0.9048	0.0476	0.1905	0.5714	0.0000
1.5714	0.2857	56.9225	0.3333	0.0000	0.0000	0.3333	0.1667	0.7500	0.0000	0.1667	0.6667	0.0000
1.7143	0.3571	71.3008	0.3529	0.0000	0.0000	0.3529	0.2941	0.8235	0.1176	0.1765	0.5882	0.0000
1.8889	0.4444	62.2687	0.2273	0.0000	0.0455	0.4091	0.3636	0.7273	0.0455	0.0909	0.7273	0.0455
1.5714	0.2857	60.2858	0.2941	0.0000	0.0000	0.4118	0.2353	0.8235	0.0000	0.1176	0.9412	0.0000
1.6923	0.3462	63.5725	0.0667	0.0000	0.0000	0.3333	0.3333	0.9333	0.0000	0.1333	0.6000	0.0000
1.9000	0.4500	69.8760	0.1429	0.0000	0.0000	0.3571	0.4286	0.8571	0.0714	0.1429	0.5000	0.0000
1.6471	0.3235	68.1388	0.1500	0.0000	0.0000	0.1500	0.2000	0.8000	0.0500	0.0000	0.4000	0.0000
1.7727	0.3864	59.1005	0.2069	0.0000	0.0000	0.2759	0.3103	0.8276	0.0345	0.1724	0.5862	0.0345
1.7391	0.3696	71.7057	0.0370	0.0000	0.0741	0.3704	0.4444	0.8889	0.0741	0.1852	0.5926	0.1852
1.8000	0.4000	74.8100	0.0526	0.0000	0.0526	0.2632	0.3684	0.7895	0.1053	0.0526	0.5789	0.1053
1.8824	0.4412	64.7311	0.1429	0.0000	0.0476	0.4286	0.2381	0.9524	0.0476	0.1905	0.6190	0.0476
1.5172	0.2586	69.7675	0.2188	0.0000	0.0313	0.4063	0.3750	0.8750	0.0938	0.0938	0.8125	0.2188
1.6250	0.3125	70.4472	0.1379	0.0000	0.0000	0.2759	0.2759	0.8276	0.0345	0.1724	0.6897	0.1379
1.8000	0.4000	74.5207	0.0500	0.0000	0.0000	0.3500	0.3500	0.7500	0.0500	0.2000	0.7000	0.1500
1.8095	0.4048	63.7973	0.1364	0.0000	0.0000	0.5000	0.2273	0.9545	0.0000	0.2727	0.5909	0.0455
1.8421	0.4211	58.5411	0.0952	0.0000	0.0000	0.4762	0.2381	0.9524	0.0000	0.1905	0.5714	0.0000
1.5789	0.2895	68.9229	0.1364	0.0000	0.0455	0.4091	0.3182	0.8636	0.0455	0.1818	0.7727	0.1818
1.6471	0.3235	75.8153	0.1429	0.0000	0.0000	0.3810	0.3333	0.7619	0.0000	0.1429	0.6667	0.1905
1.6552	0.3276	68.4915	0.1471	0.0000	0.0294	0.3529	0.3824	0.8529	0.0588	0.1471	0.6176	0.1176
1.6000	0.3000	75.9129	0.2581	0.0000	0.0323	0.3871	0.4194	0.8710	0.1290	0.1613	0.5806	0.1613
1.7500	0.3750	75.8327	0.2273	0.0000	0.0000	0.4091	0.4091	0.8182	0.0455	0.1818	0.5909	0.1364
1.7500	0.3750	65.6641	0.1481	0.0000	0.0000	0.4074	0.2963	0.9259	0.0000	0.1852	0.5185	0.0370
1.6071	0.3036	68.0381	0.1515	0.0000	0.0303	0.3939	0.2424	0.8788	0.0303	0.1212	0.6364	0.0606
1.7308	0.3654	67.7678	0.1613	0.0000	0.0323	0.4194	0.2903	0.9355	0.0323	0.1935	0.6452	0.0323
1.9524	0.4762	60.4079	0.1200	0.0000	0.0000	0.3600	0.2000	0.9600	0.0400	0.2000	0.5600	0.0000
2.0000	0.5000	61.9966	0.1667	0.0000	0.0556	0.3333	0.1667	0.9444	0.0556	0.2222	0.7222	0.0556
1.7500	0.3750	67.3196	0.1765	0.0000	0.0294	0.3824	0.3824	0.8235	0.0882	0.1176	0.6176	0.0882
1.9130	0.4565	58.5465	0.1111	0.0000	0.0370	0.3333	0.2593	0.9630	0.0741	0.1852	0.5926	0.0370
1.7368	0.3684	60.3784	0.2381	0.0000	0.0476	0.4762	0.2857	1.0000	0.0000	0.1429	0.5714	0.0952
1.7000	0.3500	70.0211	0.1481	0.0000	0.0370	0.3333	0.2593	0.8519	0.1111	0.1481	0.5556	0.0370
1.6667	0.3333	64.8120	0.0870	0.0000	0.0000	0.3043	0.1739	0.9565	0.0435	0.1304	0.6522	0.0000
2.0625	0.5313	60.9288	0.1000	0.0000	0.0000	0.4000	0.4000	0.9500	0.1000	0.1000	0.4500	0.0000
1.7143	0.3571	62.2460	0.1111	0.0000	0.0000	0.2963	0.3704	0.8519	0.0741	0.1111	0.5556	0.0000
2.0000	0.5000	63.8381	0.0500	0.0000	0.0000	0.3000	0.3000	0.9000	0.0500	0.1000	0.5500	0.0500
1.5000	0.2500	71.4982	0.1111	0.0000	0.0000	0.2593	0.4074	0.8889	0.0370	0.0370	0.7037	0.1852
1.7692	0.3846	75.5700	0.1429	0.0000	0.0000	0.2857	0.3571	0.8571	0.0714	0.2143	0.5000	0.0714
1.8000	0.4000	68.9218	0.0769	0.0000	0.0000	0.6154	0.0769	0.9231	0.0769	0.0769	0.7692	0.1538
1.7000	0.3500	67.1747	0.1250	0.0000	0.0000	0.2917	0.2500	0.7917	0.0417	0.0417	0.4583	0.0417
1.7500	0.3750	67.2064	0.2000	0.0000	0.0000	0.2000	0.2667	0.7333	0.0667	0.0000	0.7333	0.0000
1.5000	0.2500	70.3388	0.1905	0.0000	0.0000	0.2857	0.2381	0.7619	0.0476	0.0952	0.8571	0.0000
2.0000	0.5000	59.1983	0.1429	0.0000	0.0000	0.3333	0.1905	0.9048	0.0000	0.1905	0.6667	0.0000
2.0000	0.5000	71.1658	0.2000	0.0000	0.0000	0.4000	0.3500	0.9500	0.0500	0.1500	0.6500	0.1000
2.0000	0.5000	66.1452	0.0968	0.0000	0.0000	0.4194	0.3226	0.9032	0.0645	0.0968	0.5806	0.1290
1.6000	0.3000	70.0210	0.0769	0.0000	0.0385	0.3077	0.3077	0.8462	0.1154	0.0769	0.6154	0.0769
1.8000	0.4000	65.6613	0.1579	0.0000	0.0526	0.3158	0.1579	0.8947	0.0526	0.1579	0.7368	0.0526
1.7273	0.3636	65.8440	0.1053	0.0000	0.0526	0.3421	0.3158	0.8684	0.0789	0.1579	0.6053	0.0263
1.8462	0.4231	60.9338	0.1765	0.0000	0.0000	0.5294	0.2941	0.8824	0.0588	0.1176	0.6471	0.0588
1.6522	0.3261	67.3582	0.2083	0.0000	0.0000	0.3333	0.2083	0.9167	0.0417	0.1250	0.6667	0.0833
2.0000	0.5000	68.0913	0.1176	0.0000	0.0000	0.4118	0.2353	0.8824	0.0000	0.1765	0.5882	0.0588
1.6786	0.3393	72.1119	0.1379	0.0000	0.0000	0.4828	0.4483	0.9655	0.0690	0.1034	0.7586	0.1379
1.5484	0.2742	67.3662	0.1515	0.0000	0.0000	0.4848	0.3939	0.9394	0.0606	0.1212	0.7576	0.1818
1.6923	0.3462	60.7581	0.1379	0.0000	0.0345	0.5517	0.3103	0.9655	0.0345	0.2069	0.6897	0.0345
1.7600	0.3800	59.3250	0.1379	0.0000	0.0000	0.5517	0.2414	0.9310	0.0345	0.2069	0.6552	0.0000
1.8400	0.4200	63.6092	0.1111	0.0000	0.0000	0.7037	0.4815	0.9630	0.0000	0.2593	0.7407	0.0741
1.7778	0.3889	63.4776	0.0909	0.0000	0.0000	0.4091	0.3182	0.9091	0.0909	0.1818	0.6364	0.0000
2.3333	0.6667	74.7575	0.0000	0.0000	0.0000	0.2500	0.5000	1.0000	0.0000	0.2500	1.0000	0.0000
2.0000	0.5000	74.8200	0.0000	0.0000	0.0000	0.0000	0.0000	1.0000	0.0000	0.0000	1.0000	0.0000
2.0000	0.5000	70.5800	0.0000	0.0000	0.0000	0.1667	0.1667	1.0000	0.0000	0.1667	1.0000	0.0000
2.0000	0.5000	79.4233	0.0000	0.0000	0.0000	0.2500	0.2500	1.0000	0.0000	0.2500	1.0000	0.0000
2.0000	0.5000	74.8200	0.0000	0.0000	0.0000	0.0000	0.0000	1.0000	0.0000	0.0000	1.0000	0.0000
2.0000	0.5000	65.3150	0.0000	0.0000	0.0000	0.0000	0.0000	1.0000	0.0000	0.0000	1.0000	0.0000
2.0000	0.5000	65.3150	0.0000	0.0000	0.0000	0.0000	0.0000	1.0000	0.0000	0.0000	1.0000	0.0000
1.9286	0.4643	62.1792	0.0000	0.0000	0.0000	0.7143	0.5714	0.9286	0.2143	0.3571	0.9286	0.2143
1.8571	0.4286	67.1031	0.0714	0.0000	0.0000	0.7143	0.4286	0.9286	0.1429	0.2857	0.9286	0.2857
1.8889	0.4444	58.3833	0.0000	0.0000	0.0000	0.8889	0.6667	1.0000	0.2222	0.4444	1.0000	0.2222
2.0556	0.5278	62.9520	0.0000	0.0000	0.0556	0.8333	0.6111	0.8889	0.2222	0.3889	0.9444	0.1667
2.1000	0.5500	60.0800	0.0000	0.0000	0.0500	0.8500	0.4500	0.9500	0.3000	0.4000	1.0000	0.1500
2.0000	0.5000	67.2755	0.1667	0.0000	0.0000	0.7500	0.3333	0.9167	0.0833	0.3333	0.9167	0.1667
1.9333	0.4667	61.3093	0.0667	0.0000	0.0000	0.7333	0.5333	0.9333	0.2000	0.4000	0.9333	0.2000
2.3333	0.6667	73.3486	0.2222	0.0000	0.0000	0.2222	0.2222	0.7778	0.0000	0.0000	0.5556	0.0000
2.1250	0.5625	72.7880	0.0909	0.0000	0.0000	0.2727	0.3636	0.9091	0.0000	0.0909	0.6364	0.0000

2.2000	0.6000	75.5964	0.0833	0.0000	0.0833	0.3333	0.3333	0.9167	0.0000	0.0833	0.5833	0.0833
2.6667	0.8333	62.8975	0.0000	0.0000	0.0000	0.4000	0.4000	0.8000	0.0000	0.0000	0.4000	0.0000
2.2000	0.6000	69.3327	0.1176	0.0000	0.0588	0.5294	0.3529	0.8824	0.0588	0.2353	0.7647	0.1176
1.9375	0.4688	71.8400	0.0526	0.0000	0.0000	0.5263	0.3158	0.9474	0.1053	0.1579	0.7895	0.0526
1.9333	0.4667	64.6863	0.0556	0.0000	0.0000	0.3889	0.2778	0.9444	0.0000	0.1111	0.6111	0.0556
2.0000	0.5000	73.5500	0.1429	0.0000	0.0714	0.5714	0.3571	0.9286	0.0000	0.0714	0.7857	0.1429
2.4286	0.7143	71.9350	0.1000	0.0000	0.0000	0.5000	0.4000	0.9000	0.0000	0.2000	0.7000	0.0000
2.0000	0.5000	68.7623	0.0667	0.0000	0.0000	0.3333	0.2667	0.9333	0.0667	0.2000	0.8667	0.0667
1.9286	0.4643	67.7876	0.1111	0.0000	0.0556	0.3889	0.2222	0.9444	0.0556	0.1667	0.7778	0.1667
1.7333	0.3667	73.1505	0.1000	0.0000	0.0500	0.5000	0.2000	0.9500	0.0500	0.1000	0.8000	0.1500
2.0000	0.5000	70.4870	0.0909	0.0000	0.0000	0.6364	0.1818	0.9091	0.0000	0.0909	0.7273	0.0909
2.1667	0.5833	77.8286	0.1111	0.0000	0.0000	0.3333	0.2222	0.7778	0.0000	0.1111	0.5556	0.0000
2.1429	0.5714	76.3000	0.0000	0.0000	0.1250	0.3750	0.3750	0.8750	0.0000	0.1250	0.5000	0.1250
2.2222	0.6111	69.2980	0.0000	0.0000	0.0909	0.3636	0.3636	0.9091	0.0000	0.0909	0.5455	0.0909
2.1429	0.5714	85.4129	0.1250	0.0000	0.1250	0.3750	0.3750	0.8750	0.0000	0.1250	0.6250	0.1250
2.1111	0.5556	73.6822	0.0000	0.0000	0.1000	0.2000	0.3000	0.9000	0.0000	0.1000	0.6000	0.1000
1.8571	0.4286	69.7540	0.1250	0.0000	0.0625	0.5000	0.1875	0.9375	0.0625	0.1875	0.7500	0.1875
2.1818	0.5909	72.2600	0.1538	0.0000	0.0769	0.3846	0.3077	0.9231	0.0000	0.1538	0.5385	0.1538
2.0833	0.5417	73.4875	0.0769	0.0000	0.0769	0.3077	0.3846	0.9231	0.0000	0.2308	0.6154	0.1538
2.1111	0.5556	85.7556	0.0000	0.0000	0.1000	0.4000	0.4000	0.9000	0.0000	0.1000	0.6000	0.1000
2.0435	0.5217	62.2542	0.1200	0.0000	0.0400	0.7600	0.2800	0.9600	0.0800	0.2400	0.7600	0.1200
2.0000	0.5000	64.1095	0.0952	0.0000	0.0476	0.7143	0.2857	0.9524	0.0952	0.1429	0.7619	0.0952
1.9444	0.4722	69.1972	0.0526	0.0000	0.0526	0.7895	0.3684	0.9474	0.0526	0.2632	0.7895	0.1053
1.9200	0.4600	64.7235	0.1429	0.0000	0.0357	0.6071	0.2500	0.9643	0.0357	0.2143	0.7857	0.1429
2.0000	0.5000	71.7725	0.1538	0.0000	0.0000	0.3846	0.0769	0.9231	0.0769	0.1538	0.8462	0.0769
2.0000	0.5000	66.0075	0.1176	0.0000	0.0000	0.6471	0.2941	0.9412	0.0000	0.1765	0.7647	0.0588
1.8421	0.4211	65.1000	0.1429	0.0000	0.0476	0.7143	0.2381	0.9524	0.0476	0.1905	0.8095	0.1429
2.0500	0.5250	65.3955	0.1364	0.0000	0.0000	0.6818	0.3182	0.9545	0.0909	0.2273	0.9091	0.1364
1.8636	0.4318	56.3736	0.0833	0.0000	0.0000	0.6667	0.2500	0.9583	0.0833	0.1667	0.7917	0.0000
1.8421	0.4211	63.6973	0.1304	0.0000	0.0435	0.4783	0.2174	0.9565	0.0435	0.1739	0.7826	0.0870
1.8889	0.4444	69.8525	0.0000	0.0000	0.0000	0.4615	0.2308	0.9231	0.0000	0.0000	0.6923	0.0000
2.2500	0.6250	64.4867	0.1111	0.0000	0.0000	0.6667	0.3333	1.0000	0.0000	0.1111	0.7778	0.0000
2.0909	0.5455	66.1933	0.1667	0.0000	0.0000	0.5556	0.3333	0.8889	0.0000	0.1111	0.7778	0.0000
2.0000	0.5000	63.1233	0.1765	0.0000	0.0000	0.4118	0.2353	0.8824	0.0000	0.0588	0.6471	0.0000
2.2500	0.6250	70.0760	0.1000	0.0000	0.0000	0.6000	0.3000	1.0000	0.0000	0.1000	0.7000	0.0000
2.0000	0.5000	57.0200	0.2000	0.0000	0.0000	0.4000	0.1000	0.9000	0.0000	0.1000	0.6000	0.0000
2.1000	0.5500	65.8018	0.1818	0.0000	0.0000	0.4545	0.2727	1.0000	0.0000	0.0909	0.8182	0.0000
2.0000	0.5000	64.2442	0.2143	0.0000	0.0000	0.5714	0.2857	0.9286	0.0000	0.2143	0.7857	0.0000
2.0000	0.5000	67.5463	0.0588	0.0000	0.0000	0.8235	0.4706	1.0000	0.0588	0.2941	0.7647	0.0588
1.7778	0.3889	59.8742	0.0500	0.0000	0.0500	0.7500	0.4500	1.0000	0.1000	0.2500	0.9000	0.0500
1.9286	0.4643	64.0314	0.0625	0.0000	0.0000	0.7500	0.3750	0.9375	0.1250	0.1250	0.7500	0.0625
1.7619	0.3810	62.7191	0.1304	0.0000	0.0000	0.6522	0.2174	0.9565	0.1304	0.1304	0.7826	0.0870
1.9091	0.4545	65.0258	0.1538	0.0000	0.0769	0.6154	0.3846	0.9231	0.0000	0.1538	0.8462	0.1538
1.8182	0.4091	59.5459	0.0833	0.0000	0.0000	0.7917	0.3750	0.9583	0.1250	0.2500	0.7500	0.0833
1.8333	0.4167	69.1256	0.0500	0.0000	0.0000	0.7500	0.4500	0.9500	0.1000	0.2500	0.8000	0.1500
1.7857	0.3929	64.5193	0.0625	0.0000	0.0000	0.6875	0.3125	0.9375	0.0000	0.1875	0.7500	0.0000
2.0769	0.5385	66.9133	0.1250	0.0000	0.0000	0.5625	0.2500	0.9375	0.0000	0.0625	0.7500	0.0000
1.8462	0.4231	66.5657	0.0714	0.0000	0.0000	0.4286	0.3571	1.0000	0.0000	0.2143	0.7143	0.0714
2.0000	0.5000	65.6545	0.0000	0.0000	0.0000	0.5455	0.2727	1.0000	0.0909	0.0909	0.9091	0.0000
2.0000	0.5000	59.4075	0.1667	0.0000	0.0000	0.5000	0.2500	1.0000	0.0000	0.0833	0.9167	0.0000
1.7778	0.3889	64.8620	0.1000	0.0000	0.0000	0.6000	0.2000	1.0000	0.0000	0.1000	0.8000	0.0000
2.1250	0.5625	58.2444	0.0000	0.0000	0.0000	0.5000	0.3000	0.9000	0.0000	0.0000	0.7000	0.0000
2.0000	0.5000	60.8656	0.0625	0.0000	0.0000	0.5625	0.3125	1.0000	0.0625	0.1875	0.6875	0.0625
1.9231	0.4615	60.8853	0.0000	0.0000	0.0000	0.5333	0.2000	1.0000	0.0000	0.0667	0.8000	0.0000
1.8182	0.4091	70.2685	0.0714	0.0000	0.0714	0.3571	0.2143	0.9286	0.0000	0.0714	0.6429	0.0714
1.7222	0.3611	67.9505	0.1500	0.0000	0.0500	0.5500	0.2000	0.9500	0.1000	0.2000	0.7500	0.1500
2.0000	0.5000	73.1775	0.0588	0.0000	0.0000	0.6471	0.2941	0.9412	0.1176	0.1176	0.7059	0.0588
2.2000	0.6000	73.8838	0.1176	0.0000	0.0000	0.5294	0.4118	0.9412	0.0588	0.2353	0.8235	0.0588
2.2500	0.6250	63.0035	0.0769	0.0000	0.0000	0.2308	0.2308	0.8462	0.0000	0.1538	0.6154	0.0000
2.1250	0.5625	71.9609	0.0769	0.0000	0.0000	0.3077	0.3846	0.8462	0.0000	0.0769	0.6923	0.0000
2.1250	0.5625	77.4040	0.0000	0.0000	0.0909	0.4545	0.4545	0.9091	0.0000	0.1818	0.7273	0.0909
2.2857	0.6429	80.4563	0.1111	0.0000	0.0000	0.5556	0.4444	0.8889	0.0000	0.1111	0.6667	0.0000
2.0645	0.5323	65.3197	0.1250	0.0000	0.0938	0.8438	0.4688	0.9375	0.3125	0.3125	0.9375	0.1563
1.8750	0.4375	64.5293	0.1875	0.0000	0.0625	0.8750	0.4375	0.9375	0.1250	0.4375	0.8750	0.0000
1.9000	0.4500	62.2158	0.0000	0.0000	0.0769	0.4615	0.3846	0.9231	0.0000	0.1538	0.6923	0.0769
1.8235	0.4118	68.2760	0.1667	0.0000	0.0556	0.8889	0.4444	0.8889	0.1667	0.3889	0.8333	0.0556
1.6250	0.3125	64.1914	0.1667	0.0000	0.1111	0.7222	0.2778	0.8333	0.2222	0.2778	0.7778	0.1111
2.0000	0.5000	62.4530	0.0741	0.0000	0.1111	0.7778	0.4444	0.9259	0.2593	0.3704	0.8519	0.0741
1.8696	0.4348	67.9200	0.1250	0.0000	0.0833	0.8750	0.3750	0.9167	0.2500	0.4167	0.9583	0.0833
1.7222	0.3611	63.0463	0.1579	0.0000	0.0526	0.7895	0.3158	0.8947	0.1579	0.3158	0.7895	0.0526
2.0303	0.5152	64.1532	0.1176	0.0000	0.0882	0.8529	0.4412	0.9412	0.3235	0.3235	0.9412	0.1471
2.0000	0.5000	70.6050	0.1667	0.0000	0.1667	0.8750	0.4167	0.9583	0.3750	0.3333	0.9583	0.1667
1.7778	0.3889	67.5493	0.1667	0.0000	0.0556	0.8889	0.4444	0.8889	0.2222	0.2778	0.8889	0.1111
1.8462	0.4231	66.7864	0.1429	0.0000	0.0714	0.7143	0.4286	0.8571	0.0714	0.3571	0.7857	0.0000
1.7500	0.3750	68.5721	0.2353	0.0000	0.0588	0.8235	0.3529	0.8824	0.1765	0.3529	0.8235	0.1176
1.9286	0.4643	73.6660	0.0000	0.0000	0.0000	0.5882	0.4706	0.9412	0.0588	0.1765	0.7059	0.0588
1.8889	0.4444	68.7067	0.0500	0.0000	0.0000	0.6000	0.6000	0.9500	0.0000	0.1500	0.8000	0.1500

1.8095	0.4048	66.3558	0.0455	0.0000	0.0000	0.6818	0.5000	0.9091	0.0455	0.1364	0.7727	0.0909
1.8667	0.4333	70.5873	0.0588	0.0000	0.0000	0.5882	0.4706	0.9412	0.0000	0.2941	0.6471	0.1176
1.9000	0.4500	69.5750	0.0000	0.0000	0.0000	0.6154	0.3077	0.9231	0.0000	0.1538	0.6154	0.0000
1.8667	0.4333	64.9079	0.0000	0.0000	0.0000	0.5000	0.4375	0.9375	0.0000	0.3750	0.6875	0.0625
1.8500	0.4250	63.4539	0.0476	0.0000	0.0000	0.7143	0.3810	0.9048	0.0952	0.2381	0.6667	0.0476
1.9167	0.4583	65.6165	0.0800	0.0000	0.0000	0.8000	0.5200	0.9600	0.0800	0.3200	0.7600	0.0800
1.7222	0.3611	62.9817	0.1000	0.0000	0.0000	0.6000	0.3000	0.9500	0.0000	0.2000	0.7000	0.0500
1.7333	0.3667	66.3288	0.2222	0.0000	0.0000	0.6111	0.3333	0.9444	0.0000	0.2222	0.7778	0.0556
1.8125	0.4063	70.2856	0.1176	0.0000	0.0000	0.7059	0.4706	1.0000	0.0000	0.2353	0.8235	0.0588
2.0000	0.5000	68.2014	0.0000	0.0000	0.0000	0.3750	0.1250	0.8750	0.0000	0.0000	0.6250	0.0000
2.2500	0.6250	62.9260	0.0000	0.0000	0.0000	0.3333	0.1667	0.8333	0.0000	0.1667	0.6667	0.0000
1.8125	0.4063	65.3644	0.1111	0.0000	0.0000	0.6111	0.3333	0.9444	0.0556	0.2222	0.7222	0.0556
1.7333	0.3667	62.9164	0.1250	0.0000	0.0000	0.7500	0.2500	0.9375	0.0625	0.1875	0.6250	0.0625
1.9091	0.4545	67.6050	0.0000	0.0000	0.0000	0.4286	0.2857	0.9286	0.0000	0.1429	0.5000	0.0000
1.6842	0.3421	68.6074	0.1500	0.0000	0.0000	0.6000	0.3500	0.9500	0.0500	0.2000	0.8000	0.0500
1.7500	0.3750	64.2942	0.0870	0.0000	0.0000	0.6522	0.3478	0.9565	0.0435	0.3043	0.7826	0.0435
1.7500	0.3750	70.0989	0.2000	0.0000	0.0000	0.4000	0.1000	0.9000	0.0000	0.0000	0.7000	0.0000
1.6364	0.3182	63.3015	0.1143	0.0000	0.0286	0.6857	0.4857	0.9714	0.0857	0.2571	0.8571	0.1143
1.6842	0.3421	58.7500	0.0476	0.0000	0.0000	0.4762	0.2381	0.9048	0.0476	0.2381	0.7619	0.0952
1.7500	0.3750	64.0246	0.1111	0.0000	0.0000	0.5926	0.5185	0.8889	0.0370	0.1481	0.7037	0.1852
1.8889	0.4444	58.9863	0.2222	0.0000	0.0000	0.6667	0.2222	1.0000	0.1111	0.1111	0.5556	0.0000
1.7500	0.3750	63.5014	0.1250	0.0000	0.0000	0.5000	0.2500	1.0000	0.0000	0.1250	0.5000	0.0000
2.0385	0.5192	58.7263	0.1071	0.0000	0.0357	0.7143	0.4643	0.8571	0.2857	0.2857	0.8571	0.2143
1.6818	0.3409	64.6483	0.1690	0.0141	0.0704	0.7042	0.5070	0.8592	0.1268	0.1690	0.8732	0.1972
2.0345	0.5172	63.6256	0.0968	0.0000	0.0323	0.7742	0.4194	0.8710	0.2258	0.2903	0.8710	0.1935
1.6897	0.3448	63.8457	0.1452	0.0000	0.0484	0.7097	0.4677	0.8548	0.1452	0.2097	0.8871	0.1935
1.8261	0.4130	63.3489	0.1739	0.0000	0.0000	0.6522	0.2609	0.8696	0.1304	0.1739	0.8261	0.1739
2.0000	0.5000	60.3043	0.0435	0.0000	0.0000	0.6957	0.3478	0.9130	0.1304	0.1739	0.8261	0.0870
1.8889	0.4444	67.0125	0.1111	0.0000	0.0000	0.7778	0.3889	1.0000	0.2222	0.2778	1.0000	0.1667
2.0000	0.5000	71.7722	0.1500	0.0000	0.0000	0.7000	0.4000	0.9500	0.0500	0.3500	0.8000	0.1500
2.1000	0.5500	71.7778	0.1429	0.0000	0.0000	0.5238	0.3810	0.9048	0.0952	0.2381	0.8095	0.0952
2.0833	0.5417	61.2727	0.2308	0.0000	0.0000	0.4615	0.2308	0.8462	0.0769	0.1538	0.8462	0.0000
1.9412	0.4706	70.3294	0.1667	0.0000	0.1111	0.5000	0.2778	1.0000	0.0556	0.2222	0.9444	0.1111
2.1429	0.5714	57.1810	0.0800	0.0000	0.0800	0.7600	0.2400	0.8400	0.3600	0.2400	0.9200	0.2000
2.0000	0.5000	50.9021	0.0417	0.0000	0.0000	0.7917	0.3750	0.8333	0.2917	0.2083	0.8750	0.2500
1.8571	0.4286	63.0264	0.1613	0.0000	0.0323	0.6774	0.3548	0.9032	0.0645	0.1613	0.7419	0.1613
2.0000	0.5000	60.4780	0.1333	0.0000	0.0000	0.6333	0.4000	0.8667	0.0333	0.2000	0.8000	0.2000
1.8800	0.4400	58.4130	0.1538	0.0000	0.0385	0.6538	0.1923	0.9231	0.1154	0.1923	0.8077	0.1923
1.7500	0.3750	62.1094	0.1290	0.0000	0.0000	0.5806	0.2903	0.9032	0.0968	0.2258	0.8710	0.2581
2.1053	0.5526	63.4871	0.1905	0.0000	0.0000	0.7143	0.3333	0.9048	0.0476	0.2857	0.7619	0.0476
1.9048	0.4524	64.3532	0.1250	0.0000	0.0000	0.8333	0.4167	0.9167	0.0000	0.1667	0.7917	0.1250
2.0000	0.5000	60.1048	0.2083	0.0000	0.0000	0.8333	0.3333	0.9167	0.0417	0.2083	0.7917	0.0833
1.8095	0.4048	67.0639	0.1818	0.0000	0.0000	0.7273	0.4091	0.8636	0.0455	0.0000	0.9091	0.1818
1.8571	0.4286	65.1438	0.1429	0.0000	0.0000	0.7619	0.3333	0.8571	0.0952	0.1429	0.9048	0.1429
1.9231	0.4615	68.8413	0.1786	0.0000	0.0000	0.8214	0.3571	0.9286	0.0000	0.1429	0.6786	0.1071
1.9444	0.4722	70.2993	0.2500	0.0000	0.0000	0.7500	0.3500	0.8000	0.0000	0.0000	0.7000	0.1000
2.1250	0.5625	80.3738	0.2222	0.0000	0.0000	0.6667	0.3333	1.0000	0.0000	0.1111	0.7778	0.1111
2.0000	0.5000	71.9113	0.2222	0.0000	0.0000	0.5556	0.1111	1.0000	0.0000	0.3333	0.7778	0.0000
1.6842	0.3421	69.0222	0.2000	0.0000	0.0000	0.7500	0.2000	0.9500	0.0500	0.1500	0.8500	0.1000
1.7895	0.3947	70.7453	0.1000	0.0000	0.0000	0.5500	0.3000	0.9500	0.0500	0.2000	0.8500	0.0000
2.0952	0.5476	67.6415	0.1818	0.0000	0.0455	0.5455	0.3636	0.9545	0.0455	0.1818	0.7727	0.0455
2.0909	0.5455	68.8009	0.3333	0.0000	0.0000	0.5833	0.2500	0.9167	0.0000	0.0833	0.8333	0.0000
2.1875	0.5938	67.6880	0.1667	0.0000	0.0000	0.4444	0.2778	0.8889	0.0000	0.1667	0.8333	0.0000
2.0833	0.5417	68.4623	0.2143	0.0000	0.0000	0.4286	0.2143	0.9286	0.0000	0.1429	0.8571	0.0000
1.8125	0.4063	63.5687	0.1765	0.0000	0.0000	0.7647	0.1176	0.9412	0.1765	0.1176	0.8235	0.0000
1.7600	0.3800	67.8125	0.2222	0.0000	0.0000	0.5926	0.1481	0.8889	0.0741	0.0741	0.8519	0.1852
1.8182	0.4091	69.2916	0.2500	0.0000	0.0000	0.5417	0.2500	0.8333	0.0833	0.1250	0.7917	0.1667
1.9167	0.4583	69.0650	0.1481	0.0000	0.0000	0.6296	0.2963	0.8519	0.0370	0.1481	0.7778	0.0741
1.9600	0.4800	66.7348	0.2400	0.0000	0.0000	0.6800	0.2800	0.9200	0.0800	0.1600	0.7600	0.0400
1.9000	0.4500	68.1812	0.2000	0.0000	0.0000	0.6000	0.3000	1.0000	0.0000	0.2500	0.8500	0.1500
1.9500	0.4750	74.0342	0.2000	0.0000	0.0000	0.6000	0.3500	1.0000	0.0000	0.2000	0.9500	0.0500
2.0000	0.5000	67.1875	0.1333	0.0000	0.0000	0.9333	0.4000	0.9333	0.2667	0.6000	0.9333	0.0667
1.7917	0.3958	66.1720	0.0800	0.0000	0.0000	0.6400	0.2800	0.9600	0.0800	0.2800	0.8800	0.0400
1.6452	0.3226	62.7583	0.1250	0.0000	0.0000	0.7813	0.4688	0.9688	0.0938	0.4063	0.9375	0.1563
1.7500	0.3750	68.7150	0.0769	0.0000	0.0000	0.7308	0.3846	0.9231	0.0385	0.3462	0.7692	0.0385
1.7500	0.3750	65.2208	0.0455	0.0000	0.0000	0.5909	0.3182	0.9091	0.0455	0.3182	0.8182	0.0455
1.7692	0.3846	62.3079	0.1163	0.0000	0.0000	0.7907	0.3256	0.9535	0.2093	0.2791	0.8837	0.1163
1.7059	0.3529	62.1213	0.1111	0.0000	0.0000	0.7222	0.3333	0.9444	0.1667	0.2778	0.8333	0.0556
1.7241	0.3621	63.2285	0.1379	0.0000	0.0000	0.7586	0.3793	0.9655	0.1034	0.2414	0.8966	0.1379
2.0588	0.5294	64.2206	0.1053	0.0000	0.0000	0.8421	0.3684	0.8947	0.2632	0.5789	0.8421	0.1053
1.6154	0.3077	64.7300	0.1481	0.0000	0.0000	0.7778	0.3333	0.9630	0.1111	0.4074	0.8889	0.0370
1.6250	0.3125	63.8820	0.0909	0.0000	0.0000	0.7879	0.4242	0.9697	0.0606	0.3333	0.8485	0.1212
1.8000	0.4000	65.5183	0.1154	0.0000	0.0000	0.7692	0.3462	0.9615	0.0385	0.3462	0.8462	0.0769
1.7200	0.3600	65.3471	0.0769	0.0000	0.0000	0.7308	0.3077	0.9615	0.0385	0.3462	0.8077	0.0385
1.8571	0.4286	62.1429	0.1176	0.0000	0.0000	0.7059	0.2941	1.0000	0.1176	0.1765	0.9412	0.0000
1.6923	0.3462	78.1800	0.1333	0.0000	0.0000	0.4667	0.4667	0.8667	0.0667	0.0667	0.6667	0.0667
1.8947	0.4474	65.4633	0.1667	0.0000	0.0417	0.3750	0.2500	0.9583	0.0417	0.0833	0.7083	0.0833

2.3333	0.6667	64.7771	0.1818	0.0000	0.0000	0.1818	0.2727	0.7273	0.0000	0.1818	0.6364	0.0909
1.9412	0.4706	60.1350	0.1154	0.0000	0.0000	0.2692	0.2308	0.7692	0.0000	0.1154	0.7308	0.0385
1.6190	0.3095	60.9455	0.0435	0.0000	0.0435	0.6957	0.3913	0.9130	0.3043	0.3913	0.9130	0.1739
1.7059	0.3529	54.3813	0.0588	0.0000	0.0000	0.8235	0.4118	0.9412	0.4706	0.4706	0.9412	0.1765
1.8636	0.4318	58.0925	0.0909	0.0000	0.0000	0.8182	0.4545	0.9545	0.4091	0.5000	0.9545	0.2273
1.7895	0.3947	59.1150	0.1000	0.0000	0.0500	0.7500	0.4000	1.0000	0.3000	0.5500	0.9000	0.1500
2.0000	0.5000	79.8367	0.0000	0.0000	0.0000	0.3333	0.5000	1.0000	0.0000	0.0000	0.8333	0.1667
2.0000	0.5000	63.9775	0.3333	0.0000	0.0000	0.2222	0.1111	0.8889	0.0000	0.0000	0.6667	0.0000
1.6667	0.3333	61.2038	0.0833	0.0000	0.0000	0.6250	0.3750	0.9167	0.2500	0.3750	0.8750	0.0417
1.7778	0.3889	59.5732	0.0741	0.0000	0.0000	0.7778	0.4815	0.9630	0.2963	0.4444	0.9259	0.1111
1.9524	0.4762	60.5089	0.0476	0.0000	0.0000	0.8571	0.6190	0.9524	0.2381	0.3810	0.9524	0.1429
1.8800	0.4400	60.5524	0.1111	0.0000	0.0000	0.7778	0.4074	0.9630	0.2963	0.4074	0.8889	0.1111
1.7895	0.3947	68.3894	0.0500	0.0000	0.0000	0.6000	0.4000	0.9500	0.1000	0.3500	0.8500	0.0000
2.0588	0.5294	70.7724	0.1667	0.0000	0.0000	0.3333	0.2222	1.0000	0.0000	0.1667	0.7778	0.0000
2.1429	0.5714	55.5629	0.0909	0.0000	0.0909	0.8636	0.4091	0.9545	0.5455	0.3636	0.9545	0.0909
1.9375	0.4688	66.8220	0.1579	0.0000	0.0000	0.4737	0.4211	0.8947	0.0526	0.3158	0.7368	0.0000
1.9524	0.4762	59.0930	0.0800	0.0000	0.0000	0.5600	0.3600	0.8800	0.0400	0.2800	0.7600	0.0400
2.0000	0.5000	80.8033	0.0000	0.0000	0.0000	0.6667	0.3333	1.0000	0.3333	0.3333	1.0000	0.0000
2.1000	0.5500	70.1710	0.1667	0.0000	0.0000	0.3333	0.2500	0.8333	0.0000	0.1667	0.6667	0.1667
2.1000	0.5500	57.0650	0.0952	0.0000	0.0000	0.9048	0.3810	0.9048	0.3810	0.5714	0.9048	0.0476
2.1154	0.5577	63.0079	0.1071	0.0000	0.0357	0.9286	0.5000	0.8929	0.3214	0.4643	0.9286	0.1071
2.0000	0.5000	59.4905	0.0833	0.0000	0.0417	0.5417	0.4167	0.7917	0.1250	0.2083	0.8333	0.1250
2.2000	0.6000	63.3173	0.1538	0.0000	0.0000	0.4615	0.5385	0.8462	0.0769	0.0769	0.6923	0.0769
2.5000	0.7500	79.7300	0.2000	0.0000	0.0000	0.2000	0.2000	0.6000	0.0000	0.4000	0.6000	0.2000
1.9091	0.4545	55.1370	0.0000	0.0000	0.0000	0.9167	0.3333	0.8333	0.4167	0.5000	0.9167	0.2500
2.0000	0.5000	54.7213	0.0000	0.0000	0.0000	0.8824	0.3529	0.8824	0.5294	0.3529	0.9412	0.2353
2.0000	0.5000	54.5119	0.0556	0.0000	0.0000	0.8889	0.3333	0.8889	0.4444	0.3333	0.9444	0.1667
2.0714	0.5357	53.6215	0.0000	0.0000	0.0000	0.8571	0.5000	0.9286	0.5000	0.4286	0.9286	0.2143
1.7333	0.3667	67.7644	0.1765	0.0000	0.0000	0.5882	0.2353	1.0000	0.1176	0.1176	0.9412	0.0000
2.1176	0.5588	56.2083	0.0500	0.0000	0.1000	0.9000	0.3500	0.9000	0.4500	0.4000	0.9000	0.1000
2.0556	0.5278	55.7467	0.0500	0.0000	0.1000	0.8500	0.3500	0.9000	0.5500	0.3500	0.9000	0.1500
1.7895	0.3947	62.1056	0.1500	0.0000	0.0000	0.6000	0.3000	0.8000	0.0500	0.0500	0.8500	0.1500
2.0833	0.5417	67.8658	0.0769	0.0000	0.0000	0.8462	0.6154	0.9231	0.1538	0.3077	0.8462	0.0769
1.8000	0.4000	62.0360	0.0455	0.0000	0.0455	0.7727	0.3636	0.9091	0.3182	0.3636	0.9091	0.1364
2.3000	0.6500	60.4027	0.0769	0.0000	0.0000	0.4615	0.3846	0.9231	0.0769	0.3077	0.8462	0.1538
2.1111	0.5556	61.7250	0.0000	0.0000	0.0000	0.3182	0.2727	0.9091	0.0455	0.1364	0.7273	0.0909
2.2857	0.6429	68.0922	0.1000	0.0000	0.0000	0.3000	0.3000	1.0000	0.1000	0.1000	0.9000	0.0000
2.0000	0.5000	68.1025	0.0000	0.0000	0.0000	0.8000	0.4000	0.8000	0.0000	0.0000	1.0000	0.0000
2.2000	0.6000	76.3383	0.0000	0.0000	0.0000	0.1429	0.4286	1.0000	0.0000	0.0000	0.5714	0.0000
2.0588	0.5294	70.8768	0.1000	0.0000	0.0000	0.6500	0.3000	0.9500	0.1000	0.3000	0.8500	0.0500
2.5000	0.7500	79.1533	0.0000	0.0000	0.0000	0.2500	0.2500	1.0000	0.0000	0.0000	0.7500	0.0000
1.9412	0.4706	63.9850	0.1111	0.0000	0.0000	0.8333	0.3333	0.9444	0.3889	0.4444	0.9444	0.1111
2.0000	0.5000	66.3000	0.1579	0.0000	0.0000	0.3158	0.2632	0.8421	0.0000	0.1053	0.5789	0.0526
1.5833	0.2917	77.6982	0.1429	0.0000	0.0000	0.5000	0.4286	0.8571	0.0714	0.1429	0.7143	0.2143
2.0000	0.5000	55.6575	0.3333	0.0000	0.0000	0.0000	0.0000	1.0000	0.0000	0.0000	0.8333	0.0000
NA	NA	50.0000	0.5000	0.0000	0.0000	0.0000	0.0000	1.0000	0.0000	0.0000	0.7500	0.0000
1.9286	0.4643	59.0207	0.1333	0.0000	0.0000	0.9333	0.4000	1.0000	0.4000	0.4667	1.0000	0.0000
1.9375	0.4688	62.2344	0.1176	0.0000	0.0000	0.9412	0.4118	0.9412	0.3529	0.4706	0.9412	0.0588
2.1200	0.5600	68.9089	0.1290	0.0000	0.0323	0.5161	0.2903	0.9032	0.0645	0.1613	0.8710	0.0645
2.1176	0.5588	59.4785	0.1364	0.0000	0.0455	0.4545	0.2273	0.9091	0.0909	0.1818	0.8636	0.0455
3.0000	1.0000	79.0667	0.3333	0.0000	0.0000	0.1667	0.1667	0.6667	0.0000	0.0000	0.6667	0.0000
1.8333	0.4167	78.9786	0.1429	0.0000	0.0000	0.7143	0.2857	1.0000	0.2857	0.5714	1.0000	0.0000
2.2500	0.6250	73.6330	0.1000	0.0000	0.0000	0.5000	0.3000	1.0000	0.0000	0.3000	0.9000	0.0000
2.3333	0.6667	69.5619	0.0000	0.0000	0.0000	0.3750	0.1250	1.0000	0.0000	0.2500	0.8750	0.0000
1.8462	0.4231	73.7750	0.1333	0.0000	0.0000	0.4000	0.4667	0.9333	0.0667	0.2000	0.6667	0.1333
2.0000	0.5000	62.0384	0.0000	0.0000	0.0000	0.8421	0.6842	1.0000	0.3158	0.3684	1.0000	0.2105
2.4000	0.7000	80.4880	0.0000	0.0000	0.0000	0.6000	0.6000	1.0000	0.0000	0.4000	1.0000	0.0000
2.0000	0.5000	65.6465	0.0833	0.0000	0.0000	0.7500	0.4583	0.9583	0.2917	0.3750	1.0000	0.2500
1.8235	0.4118	63.3953	0.2222	0.0000	0.0000	0.6111	0.3333	0.8333	0.0556	0.1111	0.9444	0.1667
2.0000	0.5000	68.7700	0.2000	0.0000	0.0000	0.6000	0.4000	0.8000	0.0000	0.0000	0.6000	0.2000
2.0000	0.5000	62.3217	0.0000	0.0000	0.0000	0.3750	0.3750	0.7500	0.0000	0.0000	0.6250	0.0000
2.0000	0.5000	64.2325	0.0000	0.0000	0.0000	0.5000	0.7500	1.0000	0.0000	0.0000	0.7500	0.0000
1.8000	0.4000	67.1880	0.0909	0.0000	0.0000	0.2727	0.3636	0.9091	0.0000	0.0000	0.7273	0.1818
2.2500	0.6250	55.3067	0.1111	0.0000	0.0000	0.1111	0.2222	0.7778	0.0000	0.0000	0.7778	0.0000
2.2500	0.6250	61.5107	0.0588	0.0000	0.0000	0.2941	0.2353	0.8824	0.0000	0.1176	0.8235	0.0588
1.6500	0.3250	56.4944	0.2174	0.0000	0.0000	0.4348	0.1304	0.8696	0.0000	0.1304	0.6957	0.0870
1.6818	0.3409	58.3443	0.1250	0.0000	0.0417	0.6667	0.2083	0.9167	0.0833	0.2917	0.8333	0.1250
1.6667	0.3333	59.2175	0.1111	0.0000	0.0000	0.6667	0.2222	1.0000	0.0000	0.2222	1.0000	0.2222
1.6842	0.3421	61.2967	0.2250	0.0000	0.0250	0.7000	0.3250	0.8500	0.1000	0.2250	0.8750	0.2000
1.8333	0.4167	60.9872	0.1613	0.0000	0.0645	0.7419	0.3548	0.9677	0.3226	0.3871	0.8710	0.1935
1.7818	0.3909	56.3602	0.2143	0.0000	0.0536	0.8571	0.4107	0.9286	0.1786	0.2500	0.8571	0.2679
1.7500	0.3750	62.6552	0.1633	0.0000	0.0408	0.7143	0.2857	0.8980	0.2041	0.2449	0.8980	0.3265
1.7500	0.3750	53.8289	0.2581	0.0000	0.0323	0.8065	0.3871	0.9355	0.2258	0.3226	0.8387	0.1613
1.9200	0.4600	51.5244	0.1481	0.0000	0.1111	0.8889	0.3704	0.9630	0.3704	0.3333	0.9259	0.2593
1.7949	0.3974	53.3495	0.1463	0.0000	0.0732	0.8293	0.3659	0.9512	0.2683	0.2927	0.9024	0.2927
1.6591	0.3295	56.0662	0.2222	0.0000	0.0444	0.8000	0.4444	0.9556	0.2000	0.2222	0.8444	0.2444
1.8750	0.4375	55.5336	0.1667	0.0000	0.0714	0.8810	0.3571	0.9524	0.3333	0.2619	0.9048	0.2143

1.6316	0.3158	54.5212	0.2105	0.0000	0.0526	0.8421	0.4211	0.8947	0.1579	0.3158	0.7895	0.2105
2.0556	0.5278	52.6579	0.3500	0.0000	0.1000	0.8500	0.1500	0.7000	0.3000	0.2000	0.8000	0.3500
1.6538	0.3269	64.5717	0.1481	0.0000	0.0000	0.6667	0.3704	0.8889	0.1481	0.1852	0.8889	0.1481
1.7083	0.3542	65.5452	0.0000	0.0000	0.0000	0.7600	0.5600	0.9200	0.2400	0.3600	0.9200	0.2000
1.7308	0.3654	60.8770	0.0769	0.0000	0.0000	0.7692	0.5000	0.9231	0.1923	0.2692	0.8846	0.1923
1.5556	0.2778	65.6067	0.2162	0.0000	0.0270	0.7838	0.3514	0.8919	0.1081	0.2162	0.9189	0.2162
1.6842	0.3421	58.4489	0.1538	0.0000	0.0000	0.7436	0.4872	0.9487	0.1795	0.3077	0.8974	0.1282
1.8500	0.4250	72.1694	0.1500	0.0000	0.0000	0.7000	0.4000	0.8500	0.2000	0.2500	0.9000	0.0500
1.7143	0.3571	57.8063	0.0476	0.0000	0.0000	0.7143	0.5714	0.9524	0.1905	0.2857	0.9524	0.1905
1.8333	0.4167	70.2753	0.1111	0.0000	0.0000	0.7778	0.5000	1.0000	0.2778	0.2778	0.8333	0.0556
2.0000	0.5000	66.1989	0.1579	0.0000	0.0000	0.7368	0.3684	1.0000	0.2632	0.2632	0.8947	0.0000
1.8750	0.4375	69.5794	0.1250	0.0000	0.0000	0.8750	0.5000	1.0000	0.3125	0.2500	0.8750	0.0625
1.8421	0.4211	60.5188	0.1000	0.0000	0.0000	0.8000	0.4500	0.8000	0.2000	0.2500	0.8000	0.1000
1.6923	0.3462	72.9200	0.2667	0.0000	0.0000	0.4000	0.4000	0.8667	0.0667	0.0000	0.8000	0.2667
1.8000	0.4000	79.1430	0.4000	0.0000	0.0000	0.3000	0.3000	1.0000	0.0000	0.0000	1.0000	0.2000
1.6842	0.3421	72.2011	0.1905	0.0000	0.0000	0.3810	0.3810	0.9048	0.0476	0.0952	0.8095	0.2381
1.8571	0.4286	80.0257	0.4286	0.0000	0.0000	0.4286	0.4286	1.0000	0.0000	0.0000	1.0000	0.1429
1.7222	0.3611	63.6039	0.2500	0.0000	0.0000	0.4000	0.2500	0.9000	0.0500	0.0500	0.8000	0.0500
1.8571	0.4286	69.1954	0.2000	0.0000	0.0000	0.4000	0.4667	0.9333	0.0000	0.0667	0.8000	0.2000
1.5263	0.2632	60.7153	0.2000	0.0000	0.0000	0.5000	0.2000	0.9500	0.0000	0.0500	0.8500	0.1500
1.7692	0.3846	67.9183	0.2857	0.0000	0.0000	0.5000	0.4286	0.9286	0.0000	0.0714	0.7857	0.0714
1.7692	0.3846	64.3888	0.1111	0.0000	0.0370	0.6296	0.4815	0.9259	0.1111	0.2222	0.8519	0.2593
1.7500	0.3750	62.5577	0.0909	0.0000	0.0303	0.6061	0.4242	0.9394	0.0909	0.1515	0.8485	0.2424
1.7931	0.3966	67.0348	0.0968	0.0000	0.0323	0.5484	0.5161	0.9032	0.1290	0.1613	0.8065	0.1935
1.7500	0.3750	67.9493	0.1379	0.0000	0.0345	0.5862	0.4828	0.9655	0.1034	0.1379	0.8621	0.2414
1.7692	0.3846	67.6285	0.0741	0.0000	0.0370	0.6667	0.5556	0.9630	0.1111	0.1481	0.8519	0.2222
1.6818	0.3409	68.3695	0.0870	0.0000	0.0000	0.6522	0.5217	0.9565	0.0870	0.2174	0.9130	0.1739
2.0385	0.5192	68.9684	0.1667	0.0000	0.0000	0.7333	0.3333	0.9333	0.1667	0.4000	0.8000	0.0667
1.9722	0.4861	63.8100	0.2051	0.0000	0.0256	0.7179	0.3077	0.9487	0.3077	0.3590	0.8974	0.1795
2.0556	0.5278	69.2558	0.1842	0.0000	0.0000	0.6842	0.4211	0.9737	0.2105	0.2895	0.9211	0.1842
1.9722	0.4861	67.1974	0.2000	0.0000	0.0000	0.7500	0.3000	0.9500	0.2250	0.3250	0.8500	0.1750
1.9048	0.4524	72.7578	0.1250	0.0000	0.0000	0.5833	0.2917	0.9583	0.2083	0.2083	0.8750	0.0833
2.0714	0.5357	75.3100	0.1333	0.0000	0.0000	0.4667	0.2667	1.0000	0.2000	0.1333	0.9333	0.1333
2.0488	0.5244	70.3679	0.1905	0.0000	0.0000	0.6905	0.4048	0.9524	0.1905	0.3095	0.8571	0.2143
1.8936	0.4468	59.2859	0.1569	0.0000	0.0196	0.7059	0.3137	0.9412	0.2745	0.2745	0.8824	0.0980
1.8750	0.4375	70.0688	0.1316	0.0000	0.0000	0.5000	0.2368	0.9211	0.1053	0.2368	0.7368	0.0526
1.9706	0.4853	66.1249	0.1579	0.0000	0.0000	0.7105	0.3158	0.9474	0.2632	0.2632	0.8684	0.0789
2.0500	0.5250	70.6205	0.2000	0.0000	0.0000	0.6500	0.3000	1.0000	0.1500	0.3500	0.9000	0.0500
2.0000	0.5000	67.3200	0.1351	0.0000	0.0000	0.7297	0.4865	0.9459	0.1892	0.2973	0.8919	0.1351
1.8421	0.4211	57.4600	0.2000	0.0000	0.0000	0.8000	0.4000	0.9000	0.2500	0.3500	0.8500	0.1000
1.7692	0.3846	65.5117	0.3077	0.0000	0.0000	0.6923	0.3846	1.0000	0.1538	0.3077	1.0000	0.0769
1.9412	0.4706	66.2935	0.1622	0.0000	0.0270	0.7297	0.3784	0.9189	0.2703	0.2703	0.8649	0.1892
1.9677	0.4839	63.4732	0.2059	0.0000	0.0000	0.7941	0.3529	0.9412	0.2059	0.3529	0.9118	0.1471
2.1500	0.5750	60.9662	0.2083	0.0000	0.0417	0.7917	0.3333	0.8750	0.3750	0.3750	0.8750	0.1250
2.0667	0.5333	74.0291	0.1176	0.0000	0.0000	0.6176	0.4118	0.9706	0.2059	0.2941	0.8824	0.1176
1.9630	0.4815	62.7716	0.1786	0.0000	0.0000	0.6429	0.4286	0.9643	0.2143	0.3571	0.8929	0.0357
2.2000	0.6000	81.1713	0.1667	0.0000	0.0000	0.5000	0.3889	0.8333	0.1111	0.1667	0.7778	0.0556
2.1000	0.5500	71.9957	0.1818	0.0000	0.0303	0.5152	0.3333	0.9091	0.1818	0.2121	0.7879	0.1818
1.8387	0.4194	72.2544	0.2121	0.0000	0.0000	0.4848	0.2727	0.9697	0.0303	0.2424	0.9091	0.1515
1.8846	0.4423	67.9700	0.2500	0.0000	0.0000	0.5357	0.3214	0.9643	0.0000	0.2143	0.9286	0.1429
1.9231	0.4615	71.5450	0.1875	0.0000	0.0000	0.3125	0.1875	0.8750	0.0000	0.2500	0.8750	0.1250
1.8000	0.4000	64.7273	0.2000	0.0000	0.0000	0.2667	0.1333	0.8000	0.0000	0.2000	0.8000	0.0667
1.8750	0.4375	63.5095	0.2500	0.0000	0.0833	0.5417	0.4583	1.0000	0.1250	0.2083	0.8750	0.1667
1.8000	0.4000	67.2976	0.2188	0.0000	0.0000	0.6875	0.4063	0.9375	0.1563	0.3125	0.9063	0.1563
1.9355	0.4677	66.6229	0.2813	0.0000	0.0000	0.5625	0.4688	0.9063	0.0938	0.2813	0.8750	0.2500
2.1429	0.5714	65.0096	0.2667	0.0000	0.0000	0.6333	0.3333	0.9000	0.0000	0.2000	0.8000	0.1000
1.8462	0.4231	64.6518	0.1667	0.0000	0.0238	0.6667	0.3571	0.9524	0.1429	0.3333	0.8571	0.1190
1.7000	0.3500	61.8528	0.1905	0.0000	0.0000	0.6667	0.3571	0.9524	0.2143	0.3333	0.8333	0.2143
1.9643	0.4821	64.8696	0.1000	0.0000	0.0333	0.7000	0.4000	0.9333	0.1667	0.3000	0.8667	0.1333
1.8857	0.4429	63.1241	0.2162	0.0000	0.0000	0.5405	0.3514	0.9459	0.0541	0.2432	0.8378	0.1622
1.8947	0.4474	69.4905	0.1818	0.0000	0.0000	0.5455	0.3636	0.9091	0.0455	0.2727	0.8636	0.1364
1.8800	0.4400	68.9008	0.2143	0.0000	0.0000	0.5000	0.2857	0.9286	0.0357	0.2143	0.8571	0.1429
1.8000	0.4000	65.5952	0.2143	0.0000	0.0000	0.4286	0.2500	0.9286	0.0357	0.2857	0.8571	0.1786
1.7429	0.3714	62.7699	0.2222	0.0000	0.0000	0.6667	0.3333	0.9722	0.0833	0.4444	0.8611	0.1111
1.7949	0.3974	63.9868	0.2250	0.0000	0.0250	0.6750	0.3750	0.9750	0.1000	0.3750	0.8500	0.1250
1.9375	0.4688	69.8694	0.2353	0.0000	0.0000	0.5882	0.2941	0.9412	0.0000	0.1765	0.8235	0.0588
1.7037	0.3519	64.2573	0.1786	0.0000	0.0000	0.6429	0.3571	0.9643	0.0357	0.2857	0.8214	0.0357
1.7143	0.3571	53.9390	0.1818	0.0000	0.0000	0.6818	0.4545	0.9091	0.1364	0.3182	0.9091	0.2273
1.7895	0.3947	59.3544	0.1500	0.0000	0.0000	0.7500	0.4000	0.9000	0.1000	0.1000	0.8000	0.1500
1.7561	0.3780	62.9951	0.1818	0.0000	0.0227	0.6591	0.3409	0.9091	0.1136	0.3182	0.7727	0.1136
1.6522	0.3261	65.4545	0.2083	0.0000	0.0000	0.5833	0.3750	0.9583	0.0417	0.2500	0.8333	0.1250
1.7667	0.3833	63.1450	0.1875	0.0000	0.0000	0.5938	0.2813	0.9375	0.0625	0.3125	0.8438	0.0313
1.6667	0.3333	61.0731	0.2051	0.0000	0.0256	0.6667	0.3590	0.9231	0.1538	0.3333	0.8205	0.1538
1.8250	0.4125	59.4724	0.2381	0.0000	0.0000	0.6190	0.3333	0.9286	0.1190	0.2857	0.8095	0.1667
1.9286	0.4643	60.8314	0.0000	0.0000	0.0000	0.9333	0.5333	0.9333	0.3333	0.3333	0.9333	0.1333
1.8889	0.4444	64.1260	0.2727	0.0000	0.0000	0.7273	0.4545	0.9091	0.2727	0.1818	0.8182	0.0000
2.0952	0.5476	61.2535	0.0455	0.0000	0.0000	0.8636	0.5000	0.9091	0.2273	0.4545	0.9091	0.3182

1.9524	0.4762	53.5740	0.0952	0.0000	0.0000	0.9048	0.5238	1.0000	0.1905	0.3810	1.0000	0.1429
2.0833	0.5417	64.2223	0.1200	0.0000	0.0400	0.8400	0.4400	0.9200	0.2400	0.4000	0.9200	0.0400
2.0588	0.5294	67.2927	0.1765	0.0000	0.0588	0.8235	0.4706	0.9412	0.2941	0.4118	1.0000	0.0000
2.1000	0.5500	69.8322	0.0000	0.0000	0.0000	0.9000	0.6000	1.0000	0.2000	0.4000	1.0000	0.0000
2.0455	0.5227	58.8840	0.0909	0.0000	0.0000	0.7727	0.5000	0.9091	0.1818	0.4091	0.9091	0.1364
1.8333	0.4167	69.4944	0.1579	0.0000	0.0526	0.8947	0.4737	0.8947	0.2105	0.4211	0.9474	0.0526
1.9167	0.4583	72.0917	0.0769	0.0000	0.0000	0.6923	0.4615	1.0000	0.1538	0.3077	0.9231	0.0000
2.0769	0.5385	72.8700	0.2308	0.0000	0.0000	0.9231	0.5385	1.0000	0.1538	0.4615	1.0000	0.0000
2.1111	0.5556	75.6150	0.2222	0.0000	0.1111	1.0000	0.4444	0.8889	0.3333	0.4444	1.0000	0.0000
2.0556	0.5278	61.7012	0.1000	0.0000	0.0000	0.8000	0.4000	0.9000	0.3000	0.4000	0.8500	0.1000
2.0625	0.5313	57.4319	0.1176	0.0000	0.0000	0.8824	0.4118	0.9412	0.3529	0.4706	0.9412	0.1765
2.1429	0.5714	72.2029	0.0000	0.0000	0.0000	0.2500	0.1250	1.0000	0.0000	0.1250	0.5000	0.0000
2.1429	0.5714	64.0764	0.1333	0.0000	0.0000	0.9333	0.6000	0.9333	0.1333	0.5333	0.9333	0.2000
1.0000	0.0000	44.6667	0.2000	0.0000	0.0000	0.0000	0.0000	1.0000	0.2000	0.0000	0.8000	0.0000
1.0000	0.0000	46.0000	0.2000	0.0000	0.0000	0.0000	0.0000	1.0000	0.2000	0.0000	0.8000	0.0000
1.0000	0.0000	46.0000	0.2000	0.0000	0.0000	0.0000	0.0000	1.0000	0.2000	0.0000	0.8000	0.0000
1.7500	0.3750	75.7525	0.2000	0.0000	0.0000	0.5667	0.4333	0.8333	0.0333	0.1667	0.8000	0.1667
2.5714	0.7857	59.5520	0.2000	0.0000	0.0000	0.5000	0.5000	1.0000	0.0000	0.1000	0.8000	0.0000
2.2632	0.6316	73.0468	0.0455	0.0000	0.0000	0.5909	0.5000	0.9091	0.1364	0.3636	0.7727	0.1364
2.0000	0.5000	75.0389	0.0000	0.0000	0.0000	0.5789	0.5263	1.0000	0.1053	0.3684	0.8947	0.1053
1.9167	0.4583	73.7858	0.1667	0.0000	0.0000	0.5000	0.2500	1.0000	0.1667	0.4167	0.9167	0.0833
2.1667	0.5833	84.3271	0.0000	0.0000	0.0000	0.5714	0.4286	1.0000	0.0000	0.2857	0.8571	0.0000
2.1429	0.5714	77.6088	0.1250	0.0000	0.0000	0.6250	0.3125	1.0000	0.0625	0.3125	0.8750	0.0625
1.9167	0.4583	77.0815	0.1429	0.0000	0.0000	0.5000	0.2143	1.0000	0.0000	0.1429	0.8571	0.0714
1.7368	0.3684	64.8172	0.1500	0.0000	0.0000	0.7500	0.3500	1.0000	0.2000	0.4000	0.9500	0.0500
2.0833	0.5417	67.9809	0.1667	0.0000	0.0000	1.0000	0.5000	1.0000	0.1667	0.6667	0.9167	0.0000
1.8889	0.4444	73.5370	0.1000	0.0000	0.0000	0.9000	0.4000	1.0000	0.2000	0.4000	1.0000	0.0000
2.1667	0.5833	77.1038	0.0714	0.0000	0.0000	0.7857	0.4286	0.9286	0.1429	0.4286	0.7857	0.0000
1.6897	0.3448	72.8019	0.2333	0.0000	0.0000	0.6667	0.5667	0.9000	0.0000	0.2333	0.8667	0.2000
1.7333	0.3667	60.5608	0.2941	0.0000	0.0000	0.7647	0.3529	0.8235	0.0588	0.4118	0.6471	0.0588
2.0667	0.5333	76.0817	0.1176	0.0000	0.0588	0.7059	0.4118	0.9412	0.0588	0.3529	0.5882	0.0588
1.8500	0.4250	67.6469	0.2174	0.0000	0.0435	0.7826	0.3913	0.9565	0.0000	0.3043	0.6522	0.0870
1.8235	0.4118	64.2131	0.1667	0.0000	0.0000	0.8889	0.5556	0.9444	0.1111	0.6111	0.7222	0.1111
1.8571	0.4286	68.3665	0.2000	0.0000	0.0000	0.7600	0.3600	0.8400	0.1200	0.4000	0.7600	0.1200
1.9130	0.4565	64.2848	0.1538	0.0000	0.0000	0.6923	0.4615	0.9231	0.0769	0.4615	0.7692	0.0769
1.9412	0.4706	68.0453	0.1000	0.0000	0.0000	0.6000	0.4000	0.8500	0.1000	0.4000	0.7500	0.0500
2.0000	0.5000	68.3200	0.2000	0.0000	0.0000	0.6500	0.4000	0.8500	0.0500	0.3500	0.7000	0.0500
1.9333	0.4667	67.1764	0.1875	0.0000	0.0000	0.8125	0.4375	0.8750	0.0625	0.3750	0.8750	0.1250
1.9583	0.4792	67.4592	0.1481	0.0000	0.0000	0.7407	0.3704	0.9259	0.0741	0.2963	0.7778	0.0000
1.8095	0.4048	60.5505	0.1739	0.0000	0.0000	0.8696	0.3913	0.8696	0.1739	0.4348	0.7391	0.1739
1.7619	0.3810	63.7179	0.2174	0.0000	0.0000	0.8261	0.3913	0.8696	0.1739	0.4348	0.7826	0.1739
1.8636	0.4318	61.2555	0.1304	0.0000	0.0000	0.6957	0.3043	0.9130	0.1304	0.4783	0.8261	0.0870
1.7826	0.3913	61.2995	0.2000	0.0000	0.0000	0.8000	0.4000	0.8800	0.1600	0.5200	0.8000	0.0800
1.7143	0.3571	62.1785	0.1739	0.0000	0.0000	0.6522	0.3478	0.9130	0.1304	0.3478	0.9130	0.1304
1.8462	0.4231	64.4364	0.1724	0.0000	0.0000	0.6897	0.3793	0.9310	0.1034	0.4483	0.8276	0.0345
1.8333	0.4167	64.9336	0.1667	0.0000	0.0000	1.0000	0.5000	1.0000	0.2500	0.5833	0.9167	0.0000
1.8571	0.4286	58.6075	0.1429	0.0000	0.0000	0.9286	0.4286	0.9286	0.2857	0.5714	0.8571	0.0000
1.7778	0.3889	67.9119	0.1579	0.0000	0.0000	0.8421	0.4737	0.8947	0.1579	0.5263	0.8421	0.0526
1.8824	0.4412	66.0900	0.1667	0.0000	0.0000	0.8333	0.5000	0.8889	0.1111	0.4444	0.8333	0.0556
1.7333	0.3667	64.7867	0.1333	0.0000	0.0000	0.8667	0.5333	0.8667	0.1333	0.5333	0.8667	0.1333
1.7059	0.3529	59.9793	0.1667	0.0000	0.0000	0.8333	0.4444	0.8889	0.1667	0.3889	0.8333	0.1111
1.8235	0.4118	62.9557	0.1667	0.0000	0.0000	0.8889	0.5556	0.8889	0.1111	0.4444	0.8889	0.1111
1.8095	0.4048	64.8622	0.1905	0.0000	0.0000	0.9048	0.4762	0.9524	0.1429	0.4286	0.9048	0.0952
1.7778	0.3889	83.6467	0.4000	0.0000	0.0000	0.3000	0.4000	0.9000	0.1000	0.1000	0.9000	0.0000
1.6667	0.3333	84.0720	0.2941	0.0000	0.0000	0.5294	0.4706	0.8824	0.1176	0.0588	0.7647	0.1765
2.1667	0.5833	67.5657	0.2857	0.0000	0.0000	0.2857	0.7143	1.0000	0.1429	0.0000	0.5714	0.0000
2.5714	0.7857	74.2743	0.4286	0.0000	0.0000	0.2857	0.8571	1.0000	0.1429	0.0000	0.5714	0.0000
2.2500	0.6250	68.9638	0.3333	0.0000	0.0000	0.4444	0.7778	0.8889	0.1111	0.0000	0.5556	0.0000
2.1429	0.5714	64.4700	0.2857	0.0000	0.0000	0.2857	0.8571	1.0000	0.1429	0.0000	0.5714	0.0000
2.1667	0.5833	71.3267	0.2500	0.0000	0.0000	0.2500	0.7500	0.8750	0.1250	0.0000	0.3750	0.0000
2.2000	0.6000	78.7920	0.2000	0.0000	0.0000	0.4000	0.8000	1.0000	0.2000	0.0000	0.4000	0.0000
2.1667	0.5833	69.5483	0.1667	0.0000	0.0000	0.3333	0.8333	1.0000	0.1667	0.0000	0.5000	0.0000
2.2857	0.6429	71.3267	0.2857	0.0000	0.0000	0.2857	0.7143	0.8571	0.1429	0.0000	0.4286	0.0000
2.2857	0.6429	71.3267	0.2500	0.0000	0.0000	0.2500	0.7500	0.8750	0.1250	0.0000	0.3750	0.0000
2.1667	0.5833	71.3267	0.3333	0.0000	0.0000	0.3333	0.8333	1.0000	0.1667	0.0000	0.5000	0.0000
2.0000	0.5000	81.0650	0.3333	0.0000	0.0000	0.5000	0.6667	1.0000	0.1667	0.0000	0.5000	0.1667
2.3333	0.6667	78.7920	0.1667	0.0000	0.0000	0.3333	0.6667	0.8333	0.1667	0.0000	0.3333	0.0000
1.5455	0.2727	68.8005	0.2727	0.0000	0.0000	0.6364	0.4545	0.9091	0.0000	0.0000	1.0000	0.3636
1.7895	0.3947	65.3981	0.1579	0.0000	0.0526	0.9474	0.5263	0.9474	0.2105	0.4737	0.8947	0.1579
2.1818	0.5909	68.3325	0.0667	0.0000	0.0000	0.2000	0.2000	0.8000	0.0000	0.0000	0.7333	0.0000
1.9167	0.4583	64.4167	0.4167	0.0000	0.0000	0.6667	0.5833	1.0000	0.0833	0.0000	0.8333	0.0833
2.0556	0.5278	57.2219	0.1600	0.0000	0.0400	0.3200	0.2800	0.8800	0.0400	0.0800	0.7200	0.1200
2.4000	0.7000	70.7560	0.0000	0.0000	0.0000	0.2000	0.6000	1.0000	0.0000	0.2000	1.0000	0.2000
2.0000	0.5000	78.7060	0.4000	0.0000	0.0000	0.8000	0.8000	1.0000	0.0000	0.0000	0.8000	0.2000
2.2000	0.6000	74.7060	0.4000	0.0000	0.0000	0.8000	0.8000	1.0000	0.0000	0.0000	1.0000	0.2000
1.5000	0.2500	55.0150	0.2500	0.0000	0.0000	0.7500	0.5000	1.0000	0.0000	0.0000	0.7500	0.0000
2.0526	0.5263	59.4210	0.0455	0.0000	0.0000	0.5000	0.3182	1.0000	0.2727	0.2273	0.8636	0.0909

2.0714	0.5357	59.9153	0.1765	0.0000	0.0588	0.3529	0.2941	0.8824	0.0588	0.0000	0.7647	0.0588
1.6667	0.3333	68.1825	0.1111	0.0000	0.0000	1.0000	0.5556	1.0000	0.3333	0.6667	1.0000	0.0000
1.5556	0.2778	77.9106	0.2222	0.0000	0.0000	0.6667	0.4444	1.0000	0.0000	0.0000	0.8889	0.3333
1.5556	0.2778	75.6050	0.2222	0.0000	0.0000	0.6667	0.5556	1.0000	0.0000	0.0000	0.8889	0.3333
1.6667	0.3333	77.8556	0.2222	0.0000	0.0000	0.5556	0.5556	1.0000	0.0000	0.0000	0.8889	0.3333
1.6667	0.3333	50.0200	0.0000	0.0000	0.0000	0.6667	0.6667	1.0000	0.0000	0.0000	1.0000	0.0000
2.6667	0.8333	83.7000	0.3333	0.0000	0.0000	0.6667	1.0000	1.0000	0.0000	0.0000	1.0000	0.0000
1.9091	0.4545	57.1815	0.2857	0.0000	0.0000	0.2857	0.2857	0.9286	0.0000	0.0000	0.8571	0.0000
1.8000	0.4000	67.4111	0.2000	0.0000	0.0000	1.0000	0.6000	1.0000	0.2000	0.5000	1.0000	0.0000
2.4286	0.7143	73.5763	0.1000	0.0000	0.0000	0.2000	0.2000	0.8000	0.0000	0.0000	0.7000	0.0000
1.9231	0.4615	70.4077	0.3846	0.0000	0.0000	0.6923	0.4615	1.0000	0.0000	0.0769	1.0000	0.1538
2.1667	0.5833	59.5683	0.2500	0.0000	0.0000	0.2500	0.2500	0.7500	0.0000	0.0000	0.6250	0.0000
2.1250	0.5625	65.8700	0.0909	0.0000	0.0000	0.1818	0.1818	0.7273	0.0000	0.0000	0.7273	0.0909
1.6667	0.3333	57.9867	0.0000	0.0000	0.0000	0.0000	0.1667	0.8333	0.0000	0.0000	0.6667	0.0000
1.8125	0.4063	67.9592	0.1875	0.0000	0.0625	0.8125	0.4375	0.9375	0.1250	0.5000	1.0000	0.1875
2.2500	0.6250	69.5056	0.1111	0.0000	0.0000	0.2222	0.2222	1.0000	0.0000	0.0000	0.8889	0.0000
2.0000	0.5000	65.8422	0.4000	0.0000	0.0000	0.7000	0.5000	0.9000	0.0000	0.0000	0.8000	0.1000
2.2727	0.6364	63.0621	0.0000	0.0000	0.0000	0.1176	0.2941	0.8235	0.0000	0.1176	0.7647	0.0588
2.0556	0.5278	58.0558	0.1579	0.0000	0.0000	0.5263	0.2105	1.0000	0.1053	0.3158	0.8421	0.1053
1.8947	0.4474	66.0394	0.3684	0.0000	0.0000	0.7895	0.4737	1.0000	0.0000	0.2632	1.0000	0.1579
2.0000	0.5000	66.4271	0.2857	0.0000	0.0000	0.7143	0.2857	1.0000	0.0000	0.1429	0.8571	0.1429
1.5000	0.2500	57.9867	0.1429	0.0000	0.0000	0.0000	0.1429	0.8571	0.0000	0.0000	0.7143	0.0000
1.8824	0.4412	63.7050	0.1500	0.0000	0.0500	0.6500	0.3500	0.8500	0.1500	0.1500	0.7000	0.1500
1.7813	0.3906	69.2641	0.1111	0.0000	0.0000	0.5556	0.4722	0.8611	0.0278	0.1389	0.6944	0.1944
1.6818	0.3409	74.7258	0.1667	0.0000	0.0000	0.3750	0.4167	0.8750	0.0833	0.1667	0.7500	0.1667
1.5806	0.2903	65.3821	0.2121	0.0000	0.0000	0.4545	0.4545	0.9091	0.0909	0.1515	0.7273	0.1515
1.6923	0.3462	66.8883	0.1724	0.0000	0.0345	0.4483	0.4828	0.8966	0.1379	0.1034	0.7241	0.1034
1.6250	0.3125	63.5528	0.1714	0.0000	0.0286	0.5429	0.4000	0.9429	0.0857	0.1714	0.7429	0.1429
1.9130	0.4565	68.7357	0.1154	0.0000	0.0385	0.5000	0.5769	0.8846	0.1538	0.2308	0.6923	0.1538
1.6452	0.3226	63.5000	0.1471	0.0000	0.0588	0.5000	0.4706	0.9412	0.1176	0.2059	0.7353	0.1471
1.9545	0.4773	64.5833	0.1600	0.0000	0.0000	0.4000	0.2800	0.8400	0.0400	0.2000	0.7600	0.1200
1.6800	0.3400	60.5788	0.2143	0.0000	0.0000	0.5357	0.3571	0.9286	0.0357	0.2143	0.8214	0.1786
2.1111	0.5556	67.3955	0.0435	0.0000	0.0000	0.4783	0.2174	0.8696	0.0435	0.2174	0.7391	0.0870
1.9231	0.4615	59.0360	0.1250	0.0000	0.0000	0.5000	0.1250	0.9375	0.0625	0.1875	0.6875	0.0000
1.8667	0.4333	61.4807	0.1250	0.0000	0.0000	0.9375	0.5000	0.9375	0.1875	0.4375	0.9375	0.0000
1.7857	0.3929	58.1014	0.1333	0.0000	0.0000	0.8667	0.4667	0.9333	0.2000	0.3333	0.9333	0.0667
1.7778	0.3889	62.1244	0.0000	0.0000	0.0000	0.9000	0.5000	0.9000	0.3000	0.3000	0.9000	0.1000
2.0000	0.5000	63.6674	0.0909	0.0000	0.0000	0.5909	0.2727	0.8636	0.0909	0.2273	0.8182	0.0909
2.0000	0.5000	64.7992	0.0769	0.0000	0.0000	0.7692	0.3846	1.0000	0.3077	0.4615	1.0000	0.0769
1.7500	0.3750	79.4100	0.0000	0.0000	0.0000	1.0000	0.6000	1.0000	0.2000	0.6000	1.0000	0.0000
1.8750	0.4375	58.8871	0.1765	0.0000	0.0588	0.9412	0.4706	1.0000	0.2941	0.4706	0.9412	0.0588
1.8125	0.4063	60.6106	0.1765	0.0000	0.0000	0.8824	0.4118	0.9412	0.2941	0.4706	0.9412	0.0588
1.9091	0.4545	62.1227	0.0000	0.0000	0.0000	0.9167	0.5000	0.9167	0.4167	0.4167	0.9167	0.0000
1.9048	0.4524	59.9609	0.0909	0.0000	0.0455	0.4545	0.2727	1.0000	0.0455	0.1364	0.8636	0.2273
2.0000	0.5000	58.9379	0.1500	0.0000	0.0000	0.6000	0.3500	0.9500	0.0000	0.1500	0.7500	0.0500
2.2000	0.6000	68.0517	0.3333	0.0000	0.0000	0.5000	0.1667	1.0000	0.0000	0.1667	0.8333	0.0000
1.8182	0.4091	70.5325	0.0833	0.0000	0.0000	1.0000	0.5000	1.0000	0.1667	0.4167	1.0000	0.0833
1.8519	0.4259	67.2814	0.0690	0.0000	0.0345	0.5862	0.2759	1.0000	0.2414	0.3103	0.8966	0.0690
1.8421	0.4211	62.8655	0.0500	0.0000	0.0000	0.7000	0.3000	1.0000	0.3500	0.2500	0.9500	0.2000
1.6923	0.3462	67.0843	0.0667	0.0000	0.0000	0.5333	0.1333	1.0000	0.0667	0.0667	0.8000	0.0000
1.9444	0.4722	55.5217	0.1053	0.0000	0.0000	0.8947	0.5263	0.9474	0.3158	0.4211	0.9474	0.0000
1.8571	0.4286	60.9614	0.1333	0.0000	0.0000	0.9333	0.4667	0.9333	0.3333	0.4667	0.9333	0.0667
1.8684	0.4342	65.3305	0.1163	0.0000	0.0000	0.6279	0.3488	0.9302	0.0465	0.2791	0.9070	0.1628
1.9091	0.4545	65.2718	0.1818	0.0000	0.0000	0.8182	0.6364	1.0000	0.2727	0.4545	0.9091	0.0000
1.8750	0.4375	62.6933	0.1111	0.0000	0.0000	0.6667	0.4444	1.0000	0.1111	0.3333	0.8889	0.0000
2.0000	0.5000	65.1808	0.0833	0.0000	0.0000	0.5000	0.2500	1.0000	0.0833	0.1667	0.9167	0.0000
2.0000	0.5000	70.1846	0.0000	0.0000	0.0000	0.2857	0.2857	0.9286	0.0000	0.1429	0.9286	0.0714
2.0000	0.5000	66.3875	0.0000	0.0000	0.2000	0.4000	0.2000	0.8000	0.2000	0.2000	0.6000	0.0000
2.2000	0.6000	66.3609	0.1538	0.0000	0.0769	0.3846	0.1538	0.9231	0.0769	0.1538	0.8462	0.0769
1.8750	0.4375	72.0700	0.0000	0.0000	0.0000	0.2308	0.1538	1.0000	0.0769	0.0769	0.9231	0.0000
1.2500	0.1250	47.3333	0.1000	0.0000	0.0000	0.0000	0.0000	0.9000	0.1000	0.0000	0.9000	0.0000
1.8750	0.4375	66.3945	0.0000	0.0000	0.0000	0.2857	0.1429	0.9286	0.0000	0.0714	0.8571	0.1429
1.8000	0.4000	62.1414	0.2000	0.0000	0.0000	0.1000	0.1000	1.0000	0.0000	0.0000	0.9000	0.0000
1.8333	0.4167	78.7600	0.0000	0.0000	0.0000	0.3333	0.3333	1.0000	0.1667	0.3333	0.8333	0.1667
1.8333	0.4167	68.2067	0.1667	0.0000	0.0000	1.0000	0.5000	1.0000	0.1667	0.5000	1.0000	0.0000
1.8333	0.4167	59.9850	0.1600	0.0000	0.0000	0.2400	0.0800	0.9200	0.0400	0.1200	0.7600	0.0400
1.8750	0.4375	66.8200	0.0000	0.0000	0.0000	0.2000	0.1000	1.0000	0.1000	0.0000	0.9000	0.0000
1.3333	0.1667	46.0000	0.0000	0.0000	0.0000	0.0000	0.0000	0.8571	0.1429	0.0000	0.8571	0.0000
1.7000	0.3500	79.7560	0.2727	0.0000	0.0000	0.6364	0.3636	1.0000	0.0909	0.3636	1.0000	0.0000
1.7692	0.3846	74.8793	0.2000	0.0000	0.0000	0.5333	0.2667	1.0000	0.0667	0.3333	1.0000	0.0667
1.7000	0.3500	66.7567	0.1538	0.0000	0.0000	0.4615	0.2308	1.0000	0.0769	0.3846	1.0000	0.0769
2.0000	0.5000	55.9900	0.3750	0.0000	0.0000	0.0000	0.1250	0.8750	0.0000	0.0000	0.8750	0.0000
2.0000	0.5000	55.9900	0.3750	0.0000	0.0000	0.0000	0.1250	0.8750	0.0000	0.0000	0.8750	0.0000
NA	NA	50.0000	0.5000	0.0000	0.0000	0.0000	0.0000	1.0000	0.0000	0.0000	0.7500	0.0000
1.6667	0.3333	62.2422	0.3333	0.0000	0.0000	0.0833	0.0000	0.9167	0.0833	0.0833	0.9167	0.0833
3.0000	1.0000	64.5433	0.5000	0.0000	0.0000	0.1667	0.1667	0.8333	0.0000	0.0000	0.6667	0.0000
2.0000	0.5000	60.2186	0.4000	0.0000	0.0000	0.1000	0.1000	0.9000	0.0000	0.0000	0.8000	0.0000

1.6000	0.3000	68.1300	0.2353	0.0000	0.0000	0.5882	0.2353	1.0000	0.1176	0.2941	0.9412	0.1176
1.6923	0.3462	68.8007	0.0000	0.0000	0.0000	0.5714	0.2857	1.0000	0.1429	0.2857	0.9286	0.2143
1.8947	0.4474	59.1108	0.1111	0.0000	0.0000	0.3704	0.0741	0.9259	0.0370	0.1111	0.8889	0.0741
1.9333	0.4667	63.7044	0.0588	0.0000	0.0000	0.8824	0.3529	0.9412	0.2941	0.5294	0.8824	0.1176
2.0909	0.5455	60.3067	0.0000	0.0000	0.0000	1.0000	0.5833	1.0000	0.3333	0.5000	1.0000	0.1667
1.8000	0.4000	65.6141	0.1765	0.0000	0.0000	0.8235	0.2941	1.0000	0.1176	0.2941	0.9412	0.1176
2.0000	0.5000	74.0388	0.1765	0.0000	0.0000	0.8235	0.3529	1.0000	0.1176	0.2941	0.8824	0.1176
1.8571	0.4286	70.5429	0.0000	0.0000	0.0000	0.8750	0.3750	0.8750	0.2500	0.3750	0.8750	0.0000
1.5714	0.2857	64.2650	0.0000	0.0000	0.0000	0.8750	0.5000	1.0000	0.3750	0.3750	1.0000	0.0000
1.7000	0.3500	60.8600	0.1818	0.0000	0.0000	0.8182	0.2727	0.9091	0.3636	0.3636	0.9091	0.0000
2.0000	0.5000	69.9413	0.1111	0.0000	0.0000	0.3333	0.1111	1.0000	0.0000	0.0000	1.0000	0.1111
1.6923	0.3462	81.9518	0.0769	0.0000	0.0000	0.3077	0.4615	0.8462	0.0000	0.0000	0.7692	0.1538
1.6250	0.3125	87.4914	0.1250	0.0000	0.0000	0.3750	0.5000	0.8750	0.1250	0.0000	0.8750	0.1250
1.4000	0.2000	86.8975	0.2000	0.0000	0.0000	0.4000	0.2000	0.8000	0.0000	0.0000	1.0000	0.2000
1.7778	0.3889	81.8714	0.1111	0.0000	0.0000	0.2222	0.5556	0.8889	0.0000	0.0000	0.6667	0.1111
1.6250	0.3125	87.8629	0.1250	0.0000	0.0000	0.3750	0.5000	0.8750	0.0000	0.0000	0.7500	0.2500
1.7143	0.3571	85.1800	0.1429	0.0000	0.0000	0.2857	0.4286	0.8571	0.0000	0.0000	0.8571	0.1429
1.7143	0.3571	86.3700	0.1250	0.0000	0.0000	0.2500	0.5000	0.8750	0.0000	0.0000	0.8750	0.1250
1.3333	0.1667	87.6960	0.1667	0.0000	0.0000	0.5000	0.5000	0.8333	0.1667	0.0000	0.8333	0.1667
1.6250	0.3125	82.8914	0.1111	0.0000	0.0000	0.2222	0.5556	0.8889	0.0000	0.0000	0.7778	0.1111
1.6250	0.3125	84.1375	0.1111	0.0000	0.0000	0.2222	0.4444	0.8889	0.0000	0.0000	0.7778	0.1111
1.7143	0.3571	80.1117	0.0000	0.0000	0.0000	0.1429	0.5714	0.8571	0.0000	0.0000	0.7143	0.0000
1.5714	0.2857	86.0986	0.1250	0.0000	0.0000	0.3750	0.6250	0.8750	0.1250	0.0000	0.7500	0.1250
1.3750	0.1875	86.5863	0.1000	0.0000	0.0000	0.4000	0.5000	0.7000	0.1000	0.0000	0.7000	0.1000
1.5455	0.2727	86.1500	0.0769	0.0000	0.0000	0.3846	0.5385	0.7692	0.0769	0.0000	0.7692	0.1538
2.0000	0.5000	87.5529	0.0000	0.0000	0.1000	0.2000	0.4000	0.8000	0.1000	0.0000	0.6000	0.0000
2.0000	0.5000	76.8375	0.0000	0.0000	0.0000	0.2500	0.5000	0.7500	0.0000	0.0000	1.0000	0.0000
2.0000	0.5000	75.5300	0.0000	0.0000	0.0000	0.2500	0.5000	1.0000	0.0000	0.0000	1.0000	0.0000
2.1667	0.5833	88.8000	0.1667	0.0000	0.0000	0.5000	0.6667	1.0000	0.1667	0.0000	0.6667	0.1667
1.5000	0.2500	69.3867	0.1667	0.0000	0.0000	0.3333	0.3333	1.0000	0.0000	0.0000	1.0000	0.1667
1.8571	0.4286	88.8850	0.1250	0.0000	0.0000	0.2500	0.3750	1.0000	0.0000	0.0000	0.8750	0.1250
1.8333	0.4167	87.2971	0.1429	0.0000	0.0000	0.2857	0.4286	1.0000	0.0000	0.0000	0.8571	0.1429
1.6667	0.3333	88.5567	0.1429	0.0000	0.1429	0.4286	0.4286	0.8571	0.1429	0.0000	0.5714	0.1429
1.1667	0.0833	81.2560	0.1429	0.0000	0.1429	0.2857	0.4286	0.7143	0.0000	0.0000	0.7143	0.1429
1.6667	0.3333	86.3860	0.1250	0.0000	0.1250	0.3750	0.5000	0.7500	0.0000	0.0000	0.6250	0.2500
1.5556	0.2778	85.1833	0.0909	0.0000	0.0000	0.3636	0.5455	0.7273	0.0909	0.0000	0.7273	0.0909
1.6000	0.3000	89.4780	0.0833	0.0000	0.0000	0.4167	0.5000	0.7500	0.0833	0.0000	0.6667	0.1667
1.2500	0.1250	86.0650	0.1667	0.0000	0.1667	0.3333	0.3333	0.6667	0.0000	0.0000	0.6667	0.1667
1.4286	0.2143	80.0400	0.1429	0.0000	0.0000	0.2857	0.5714	0.8571	0.0000	0.0000	0.8571	0.1429
1.5714	0.2857	89.9014	0.1111	0.0000	0.1111	0.3333	0.3333	0.6667	0.0000	0.0000	0.7778	0.1111
1.2500	0.1250	90.8300	0.2000	0.0000	0.0000	0.4000	0.4000	0.6000	0.0000	0.0000	0.8000	0.2000
1.8750	0.4375	82.4400	0.1250	0.0000	0.0000	0.5000	0.5000	0.8750	0.0000	0.0000	0.8750	0.2500
2.2000	0.6000	88.5280	0.2000	0.0000	0.0000	0.4000	0.4000	1.0000	0.0000	0.0000	0.8000	0.2000
1.7500	0.3750	80.3875	0.2500	0.0000	0.0000	0.5000	0.5000	1.0000	0.0000	0.0000	1.0000	0.2500
1.2500	0.1250	73.5300	0.2500	0.0000	0.0000	0.5000	0.2500	0.7500	0.0000	0.0000	1.0000	0.2500
2.0000	0.5000	81.2350	0.1667	0.0000	0.0000	0.3333	0.3333	1.0000	0.0000	0.0000	0.8333	0.1667
1.8000	0.4000	85.6233	0.1667	0.0000	0.0000	0.5000	0.5000	0.8333	0.0000	0.0000	1.0000	0.1667
1.3333	0.1667	89.3229	0.1250	0.0000	0.1250	0.3750	0.3750	0.8750	0.0000	0.0000	0.8750	0.2500
1.3333	0.1667	85.4957	0.1250	0.0000	0.1250	0.3750	0.5000	0.7500	0.0000	0.0000	0.7500	0.1250
1.1667	0.0833	84.3800	0.1429	0.0000	0.0000	0.2857	0.4286	0.8571	0.0000	0.0000	0.8571	0.1429
1.3333	0.1667	82.4967	0.0833	0.0000	0.0833	0.1667	0.2500	0.6667	0.0000	0.0000	0.6667	0.0833
1.6000	0.3000	78.0900	0.2000	0.0000	0.0000	0.4000	0.2000	0.8000	0.0000	0.0000	0.8000	0.2000
1.5714	0.2857	86.4163	0.1111	0.0000	0.1111	0.3333	0.4444	0.7778	0.0000	0.0000	0.6667	0.1111
1.3333	0.1667	85.4957	0.1250	0.0000	0.1250	0.3750	0.5000	0.7500	0.0000	0.0000	0.7500	0.1250
1.5455	0.2727	78.6570	0.0769	0.0000	0.0769	0.2308	0.4615	0.7692	0.0000	0.0000	0.6923	0.0769
1.5714	0.2857	84.2600	0.1111	0.0000	0.1111	0.3333	0.3333	0.7778	0.0000	0.0000	0.7778	0.1111
1.8333	0.4167	85.1800	0.1429	0.0000	0.1429	0.2857	0.4286	0.8571	0.0000	0.0000	0.7143	0.1429
1.2500	0.1250	86.0650	0.2000	0.0000	0.2000	0.4000	0.4000	0.8000	0.0000	0.0000	0.8000	0.2000
2.0000	0.5000	87.4450	0.2000	0.0000	0.2000	0.4000	0.4000	0.8000	0.0000	0.0000	0.8000	0.2000
1.6250	0.3125	77.1650	0.1250	0.0000	0.0000	0.2500	0.5000	0.8750	0.0000	0.0000	0.8750	0.1250
2.0000	0.5000	85.1580	0.0000	0.0000	0.0000	0.1667	0.6667	1.0000	0.0000	0.0000	0.8333	0.0000
1.6000	0.3000	86.3700	0.1429	0.0000	0.1429	0.2857	0.4286	0.8571	0.0000	0.0000	0.8571	0.1429
1.1667	0.0833	84.4980	0.1250	0.0000	0.1250	0.2500	0.2500	0.5000	0.0000	0.0000	0.8750	0.1250
1.2500	0.1250	83.9071	0.0000	0.0000	0.0000	0.2000	0.3000	0.5000	0.0000	0.0000	0.9000	0.0000
1.2500	0.1250	87.9575	0.0000	0.0000	0.1667	0.1667	0.3333	0.6667	0.0000	0.0000	0.8333	0.0000
1.2500	0.1250	88.8520	0.1667	0.0000	0.1667	0.3333	0.3333	0.8333	0.0000	0.0000	0.8333	0.1667
1.6000	0.3000	89.5200	0.1429	0.0000	0.0000	0.2857	0.2857	0.8571	0.0000	0.0000	0.7143	0.1429
1.4000	0.2000	89.4083	0.1429	0.0000	0.1429	0.4286	0.4286	0.7143	0.0000	0.0000	0.8571	0.1429
1.2500	0.1250	88.8520	0.1667	0.0000	0.1667	0.3333	0.3333	0.8333	0.0000	0.0000	0.8333	0.1667
1.2857	0.1429	89.9686	0.1111	0.0000	0.1111	0.3333	0.3333	0.6667	0.0000	0.0000	0.8889	0.1111
1.5000	0.2500	86.5200	0.0000	0.0000	0.1250	0.1250	0.3750	0.7500	0.0000	0.0000	0.8750	0.0000
1.5000	0.2500	87.9100	0.5000	0.0000	0.0000	1.0000	0.5000	1.0000	0.0000	0.0000	1.0000	0.5000
1.8000	0.4000	84.5770	0.2000	0.0000	0.0000	0.6000	0.6000	1.0000	0.0000	0.0000	1.0000	0.2000
1.6667	0.3333	91.9400	0.3333	0.0000	0.0000	0.6667	0.3333	1.0000	0.0000	0.0000	1.0000	0.3333
2.0000	0.5000	87.4450	0.2500	0.0000	0.0000	0.5000	0.5000	1.0000	0.0000	0.0000	1.0000	0.2500
1.8000	0.4000	90.6040	0.1667	0.0000	0.1667	0.5000	0.5000	0.8333	0.0000	0.0000	0.6667	0.3333
1.5000	0.2500	87.9100	0.5000	0.0000	0.0000	1.0000	0.5000	1.0000	0.0000	0.0000	1.0000	0.5000

1.5000	0.2500	87.9100	0.5000	0.0000	0.0000	1.0000	0.5000	1.0000	0.0000	0.0000	1.0000	0.5000
1.5000	0.2500	64.0800	0.0000	0.0000	0.0000	0.5000	0.5000	1.0000	0.0000	0.0000	1.0000	0.0000
2.0000	0.5000	84.3625	0.2500	0.0000	0.0000	0.7500	0.7500	1.0000	0.0000	0.0000	1.0000	0.5000
1.6667	0.3333	87.9100	0.3333	0.0000	0.0000	0.6667	0.6667	0.6667	0.0000	0.0000	1.0000	0.3333
1.8000	0.4000	73.6375	0.2000	0.0000	0.0000	0.4000	0.6000	0.8000	0.0000	0.0000	1.0000	0.2000
1.2500	0.1250	83.8180	0.2000	0.0000	0.0000	0.4000	0.2000	1.0000	0.0000	0.0000	0.8000	0.2000
1.5000	0.2500	87.9100	0.5000	0.0000	0.0000	1.0000	0.5000	1.0000	0.0000	0.0000	1.0000	0.5000
1.7143	0.3571	78.4957	0.1250	0.0000	0.0000	0.6250	0.5000	0.8750	0.0000	0.0000	0.8750	0.5000
1.6667	0.3333	77.4900	0.1429	0.0000	0.1429	0.5714	0.4286	0.7143	0.0000	0.0000	0.8571	0.4286
1.8333	0.4167	77.8750	0.1667	0.0000	0.0000	0.5000	0.5000	1.0000	0.0000	0.1667	1.0000	0.1667
1.7500	0.3750	76.4625	0.0000	0.0000	0.0000	0.2500	0.5000	1.0000	0.0000	0.0000	0.7500	0.0000
1.4000	0.2000	84.2600	0.0833	0.0000	0.0833	0.2500	0.4167	0.7500	0.0000	0.0000	0.8333	0.1667
1.8889	0.4444	87.3311	0.0909	0.0000	0.0909	0.4545	0.4545	0.8182	0.0000	0.0000	0.8182	0.3636
1.3333	0.1667	87.9100	0.2500	0.0000	0.2500	0.5000	0.2500	0.5000	0.0000	0.0000	0.7500	0.2500
1.5000	0.2500	64.1650	0.0000	0.0000	0.0000	0.3333	0.3333	0.6667	0.0000	0.0000	0.6667	0.3333
1.5000	0.2500	64.1650	0.0000	0.0000	0.0000	0.3333	0.3333	0.6667	0.0000	0.0000	0.6667	0.3333
1.5000	0.2500	64.0800	0.0000	0.0000	0.0000	0.5000	0.5000	1.0000	0.0000	0.0000	1.0000	0.0000
1.7500	0.3750	62.8250	0.0000	0.0000	0.0000	0.0000	0.2500	1.0000	0.0000	0.0000	0.7500	0.2500
1.3333	0.1667	75.5533	0.0000	0.0000	0.0000	0.2500	0.2500	0.7500	0.0000	0.0000	0.5000	0.0000
2.5000	0.7500	73.9600	0.0000	0.0000	0.0000	0.0000	0.6667	0.3333	0.0000	0.0000	0.3333	0.0000
2.5000	0.7500	78.6750	0.0000	0.0000	0.0000	0.5000	1.0000	1.0000	0.0000	0.0000	1.0000	0.0000
1.0000	0.0000	44.9400	0.0000	0.0000	0.0000	0.0000	0.0000	0.5000	0.0000	0.0000	0.5000	0.5000
1.3333	0.1667	73.5300	0.3333	0.0000	0.0000	0.6667	0.3333	1.0000	0.0000	0.0000	1.0000	0.3333
2.0000	0.5000	83.3900	0.0000	0.0000	0.0000	0.5000	0.5000	0.5000	0.0000	0.0000	0.5000	0.0000
2.0000	0.5000	83.3900	0.0000	0.0000	0.0000	1.0000	1.0000	1.0000	0.0000	0.0000	1.0000	0.0000
1.4000	0.2000	80.1475	0.2000	0.0000	0.0000	0.4000	0.2000	0.8000	0.0000	0.0000	0.8000	0.2000
1.0000	0.0000	44.7700	0.0000	0.0000	0.0000	0.0000	0.0000	1.0000	0.0000	0.0000	1.0000	0.0000
1.5000	0.2500	64.0800	0.0000	0.0000	0.0000	0.3333	0.3333	0.6667	0.0000	0.0000	0.6667	0.0000
2.3333	0.6667	81.6733	0.0000	0.0000	0.0000	0.4000	0.8000	0.6000	0.0000	0.0000	0.6000	0.2000
1.5000	0.2500	100.0000	0.0000	0.0000	0.0000	0.0000	0.5000	0.2500	0.0000	0.0000	0.5000	0.0000
2.0000	0.5000	91.6950	0.0000	0.0000	0.0000	0.5000	0.5000	1.0000	0.0000	0.0000	1.0000	0.0000
1.5000	0.2500	87.9100	0.5000	0.0000	0.0000	1.0000	0.5000	1.0000	0.0000	0.0000	1.0000	0.5000
2.0000	0.5000	83.3900	0.0000	0.0000	0.0000	1.0000	1.0000	1.0000	0.0000	0.0000	1.0000	0.0000
1.3333	0.1667	47.3850	0.0000	0.0000	0.0000	0.3333	0.0000	0.6667	0.0000	0.0000	1.0000	0.3333
1.5000	0.2500	64.0800	0.0000	0.0000	0.0000	0.5000	0.5000	1.0000	0.0000	0.0000	1.0000	0.0000
1.0000	0.0000	50.0000	0.0000	0.0000	0.0000	0.0000	0.0000	1.0000	0.0000	0.0000	1.0000	0.0000
1.0000	0.0000	44.7700	0.0000	0.0000	0.0000	0.0000	0.0000	1.0000	0.0000	0.0000	1.0000	0.0000
2.0000	0.5000	67.3733	0.0000	0.0000	0.0000	0.3333	0.6667	1.0000	0.0000	0.0000	1.0000	0.0000
1.0000	0.0000	44.7700	0.0000	0.0000	0.0000	0.0000	0.0000	1.0000	0.0000	0.0000	1.0000	0.0000
3.0000	1.0000	73.9600	0.0000	0.0000	0.0000	0.0000	0.5000	0.5000	0.0000	0.0000	0.5000	0.0000
1.6667	0.3333	86.2300	0.0000	0.0000	0.0000	0.0000	0.6667	0.6667	0.0000	0.0000	0.6667	0.3333
3.0000	1.0000	73.9600	0.0000	0.0000	0.0000	0.0000	1.0000	1.0000	0.0000	0.0000	1.0000	0.0000
1.4000	0.2000	81.6650	0.0000	0.0000	0.0000	0.2000	0.4000	0.8000	0.0000	0.0000	0.8000	0.2000
1.8889	0.4444	76.4440	0.1111	0.0000	0.0000	0.5556	0.6667	0.5556	0.0000	0.0000	0.8889	0.4444
2.2500	0.6250	65.5875	0.0000	0.0000	0.0000	0.7500	1.0000	1.0000	0.0000	0.0000	1.0000	0.0000
2.0000	0.5000	56.6950	0.0000	0.0000	0.0000	1.0000	1.0000	1.0000	0.0000	0.0000	1.0000	0.0000
2.5000	0.7500	78.6750	0.0000	0.0000	0.0000	0.5000	1.0000	1.0000	0.0000	0.0000	1.0000	0.0000
2.0000	0.5000	78.6750	0.0000	0.0000	0.0000	0.3333	1.0000	0.6667	0.0000	0.0000	1.0000	0.3333
1.6667	0.3333	59.3650	0.0000	0.0000	0.0000	0.0000	0.6667	0.6667	0.0000	0.0000	1.0000	0.3333
1.0000	0.0000	68.6000	0.3333	0.0000	0.0000	0.3333	0.3333	0.6667	0.0000	0.0000	1.0000	0.6667
1.5000	0.2500	69.1120	0.0000	0.0000	0.0000	0.1429	0.4286	0.7143	0.0000	0.0000	0.7143	0.2857
1.8000	0.4000	49.6825	0.0000	0.0000	0.0000	0.3333	0.5000	0.8333	0.0000	0.0000	0.5000	0.0000
1.7500	0.3750	58.1000	0.0000	0.0000	0.0000	0.2000	0.4000	0.8000	0.0000	0.0000	0.6000	0.0000
2.1429	0.5714	62.8680	0.1250	0.0000	0.0000	0.3750	0.3750	0.7500	0.0000	0.0000	0.3750	0.0000
1.5714	0.2857	59.4500	0.1111	0.0000	0.0000	0.1111	0.3333	0.6667	0.0000	0.0000	0.4444	0.0000
2.0000	0.5000	66.5083	0.0000	0.0000	0.0000	0.2222	0.5556	0.6667	0.0000	0.0000	0.4444	0.0000
1.8000	0.4000	63.4060	0.3333	0.0000	0.0000	0.1667	0.3333	0.8333	0.0000	0.0000	0.5000	0.0000
1.6667	0.3333	56.2433	0.0000	0.0000	0.0000	0.2500	0.5000	0.7500	0.0000	0.0000	0.5000	0.0000
2.0000	0.5000	61.9800	0.0000	0.0000	0.0000	0.3333	0.6667	0.6667	0.0000	0.0000	0.3333	0.0000
2.0000	0.5000	68.7867	0.0000	0.0000	0.0000	0.0000	0.3333	1.0000	0.0000	0.0000	0.6667	0.0000
2.0000	0.5000	68.7867	0.0000	0.0000	0.0000	0.0000	0.3333	1.0000	0.0000	0.0000	0.6667	0.0000
2.3333	0.6667	85.1133	0.3333	0.0000	0.0000	0.0000	0.6667	1.0000	0.3333	0.0000	0.3333	0.0000
1.5000	0.2500	66.3150	0.0000	0.0000	0.0000	0.5000	0.0000	1.0000	0.0000	0.0000	1.0000	0.5000
1.6667	0.3333	55.3150	0.0000	0.0000	0.0000	0.0000	0.2500	0.5000	0.0000	0.0000	0.7500	0.0000
1.6667	0.3333	62.4980	0.0000	0.0000	0.0000	0.1667	0.1667	0.8333	0.0000	0.0000	0.6667	0.1667
1.6667	0.3333	55.3150	0.0000	0.0000	0.0000	0.0000	0.2500	0.5000	0.0000	0.0000	0.7500	0.0000
1.8333	0.4167	56.4020	0.1667	0.0000	0.0000	0.5000	0.3333	1.0000	0.0000	0.0000	0.6667	0.1667
1.8000	0.4000	62.2775	0.2000	0.0000	0.0000	0.5000	0.3000	0.9000	0.0000	0.1000	0.7000	0.1000
2.0000	0.5000	56.6700	0.2000	0.0000	0.0000	0.6000	0.4000	1.0000	0.0000	0.0000	0.4000	0.0000
1.8333	0.4167	68.5500	0.0000	0.0000	0.0000	0.3333	0.5000	0.8333	0.0000	0.0000	0.6667	0.0000
1.6250	0.3125	68.0271	0.0000	0.0000	0.0000	0.2500	0.2500	0.8750	0.0000	0.0000	0.8750	0.1250
2.0000	0.5000	55.3150	0.0000	0.0000	0.0000	0.0000	0.5000	1.0000	0.0000	0.0000	1.0000	0.0000
1.5000	0.2500	54.1000	0.0000	0.0000	0.0000	0.0000	0.2500	1.0000	0.0000	0.0000	0.7500	0.0000
1.6667	0.3333	67.4667	0.0000	0.0000	0.0000	0.0000	0.3333	1.0000	0.0000	0.0000	0.6667	0.0000
1.6667	0.3333	48.2100	0.0000	0.0000	0.0000	0.0000	0.3333	1.0000	0.0000	0.0000	0.6667	0.0000
1.6667	0.3333	51.8000	0.0000	0.0000	0.0000	0.0000	0.3333	1.0000	0.0000	0.0000	1.0000	0.0000

2.0000	0.5000	55.3150	0.0000	0.0000	0.0000	0.0000	0.5000	1.0000	0.0000	0.0000	1.0000	0.0000
3.0000	1.0000	73.9600	0.0000	0.0000	0.0000	0.0000	1.0000	1.0000	0.0000	0.0000	1.0000	0.0000
1.3333	0.1667	35.3350	0.0000	0.0000	0.0000	0.0000	0.0000	0.6667	0.0000	0.0000	1.0000	0.0000
1.6667	0.3333	55.3150	0.0000	0.0000	0.0000	0.0000	0.3333	0.6667	0.0000	0.0000	1.0000	0.0000
1.0000	0.0000	36.6700	0.0000	0.0000	0.0000	0.0000	0.0000	1.0000	0.0000	0.0000	1.0000	0.0000
1.6250	0.3125	49.3700	0.1111	0.0000	0.0000	0.4444	0.4444	0.8889	0.0000	0.0000	0.6667	0.1111
1.4000	0.2000	62.7300	0.0909	0.0000	0.0000	0.3636	0.2727	0.8182	0.0000	0.0000	0.5455	0.0909
1.6000	0.3000	59.4950	0.0909	0.0000	0.0000	0.3636	0.3636	0.9091	0.0000	0.0000	0.5455	0.0909
1.7500	0.3750	86.2300	0.0000	0.0000	0.0000	0.0000	0.2000	0.6000	0.0000	0.0000	0.2000	0.0000
2.0000	0.5000	51.9800	0.0000	0.0000	0.0000	0.2500	0.5000	0.7500	0.0000	0.0000	0.5000	0.0000
1.6667	0.3333	72.9475	0.0000	0.0000	0.0000	0.3333	0.1667	0.8333	0.0000	0.0000	0.5000	0.1667
1.5000	0.2500	61.5713	0.0000	0.0000	0.0000	0.3000	0.2000	0.9000	0.1000	0.0000	0.7000	0.1000
1.5000	0.2500	67.3617	0.0000	0.0000	0.0000	0.1429	0.2857	0.8571	0.0000	0.0000	0.8571	0.0000
1.5000	0.2500	58.2375	0.0000	0.0000	0.0000	0.2500	0.3750	1.0000	0.0000	0.0000	0.7500	0.1250
1.5000	0.2500	69.2513	0.0000	0.0000	0.0000	0.1000	0.1000	0.9000	0.0000	0.0000	0.7000	0.1000
1.4545	0.2273	66.1675	0.0000	0.0000	0.0000	0.1818	0.0909	0.8182	0.0000	0.0000	0.7273	0.0909
1.4286	0.2143	75.0567	0.0000	0.0000	0.0000	0.1429	0.1429	1.0000	0.0000	0.0000	0.7143	0.1429
1.4286	0.2143	71.4283	0.0000	0.0000	0.0000	0.2857	0.2857	1.0000	0.0000	0.0000	0.5714	0.1429
1.5714	0.2857	61.7000	0.0000	0.0000	0.0000	0.2857	0.4286	1.0000	0.0000	0.0000	0.8571	0.1429
1.5000	0.2500	60.2375	0.0000	0.0000	0.0000	0.3000	0.4000	0.9000	0.0000	0.0000	0.6000	0.1000
1.8000	0.4000	54.0788	0.0000	0.0000	0.0000	0.5000	0.5000	0.8000	0.0000	0.0000	0.7000	0.2000
1.9091	0.4545	63.4956	0.0909	0.0000	0.0909	0.2727	0.4545	0.9091	0.0909	0.0000	0.5455	0.0909
1.8462	0.4231	64.1400	0.0000	0.0000	0.0714	0.2857	0.3571	0.7857	0.0714	0.0000	0.5714	0.1429
1.7273	0.3636	52.9956	0.0833	0.0000	0.0000	0.4167	0.3333	0.8333	0.0000	0.0000	0.6667	0.1667
1.7000	0.3500	51.8850	0.1000	0.0000	0.1000	0.3000	0.4000	0.9000	0.1000	0.0000	0.6000	0.2000
1.8333	0.4167	59.1280	0.0833	0.0000	0.0000	0.3333	0.4167	0.9167	0.0000	0.0000	0.6667	0.0833
1.5000	0.2500	63.4463	0.1000	0.0000	0.0000	0.3000	0.3000	0.9000	0.0000	0.0000	0.6000	0.2000
1.6667	0.3333	56.0070	0.0769	0.0000	0.0000	0.3077	0.3077	0.8462	0.0000	0.0000	0.6923	0.1538
1.5000	0.2500	62.9570	0.0769	0.0000	0.0000	0.3846	0.3077	0.8462	0.0000	0.0000	0.5385	0.1538
1.4545	0.2273	62.8778	0.0909	0.0000	0.0000	0.1818	0.2727	0.8182	0.0000	0.0000	0.7273	0.0909
1.4444	0.2222	62.2571	0.1111	0.0000	0.0000	0.3333	0.3333	0.7778	0.0000	0.0000	0.6667	0.2222
1.7273	0.3636	58.2838	0.0909	0.0000	0.0000	0.2727	0.4545	0.8182	0.0000	0.0000	0.6364	0.0909
1.8571	0.4286	59.5467	0.0000	0.0000	0.0000	0.0000	0.4286	1.0000	0.0000	0.0000	0.5714	0.0000
1.8333	0.4167	63.7133	0.0000	0.0000	0.0000	0.3333	0.5000	1.0000	0.0000	0.0000	0.8333	0.1667
1.7143	0.3571	50.9550	0.0000	0.0000	0.0000	0.1429	0.2857	1.0000	0.0000	0.0000	0.7143	0.0000
1.0000	0.0000	61.7233	0.0000	0.0000	0.0000	0.3333	0.3333	1.0000	0.0000	0.0000	0.3333	0.0000
1.5000	0.2500	72.0400	0.2000	0.0000	0.0000	0.4000	0.6000	0.8000	0.0000	0.0000	0.6000	0.2000
1.3333	0.1667	77.6600	0.0000	0.0000	0.0000	0.0000	0.0000	1.0000	0.0000	0.0000	0.6667	0.0000
1.6000	0.3000	79.8740	0.2000	0.0000	0.0000	0.2000	0.2000	1.0000	0.0000	0.0000	0.8000	0.2000
1.0000	0.0000	NA	0.0000	0.0000	0.0000	0.0000	0.0000	0.0000	0.0000	0.0000	0.0000	0.0000
1.0000	0.0000	71.4250	0.2500	0.0000	0.0000	0.5000	0.2500	1.0000	0.0000	0.0000	0.5000	0.2500
1.1667	0.0833	68.3717	0.1429	0.0000	0.0000	0.4286	0.2857	0.8571	0.0000	0.0000	0.5714	0.2857
1.4000	0.2000	69.2660	0.1667	0.0000	0.0000	0.1667	0.3333	0.8333	0.0000	0.0000	0.6667	0.1667
1.0000	0.0000	44.7700	0.0000	0.0000	0.0000	0.0000	0.0000	0.5000	0.0000	0.0000	1.0000	0.0000
2.0000	0.5000	70.6067	0.0000	0.0000	0.0000	0.5000	0.7500	0.7500	0.0000	0.0000	0.5000	0.2500
1.0000	0.0000	44.7700	0.0000	0.0000	0.0000	0.0000	0.5000	0.5000	0.0000	0.0000	0.5000	0.0000
2.0000	0.5000	69.8900	0.0000	0.0000	0.0000	0.3333	0.5000	0.8333	0.0000	0.0000	0.5000	0.1667
3.0000	1.0000	73.9600	0.0000	0.0000	0.0000	0.0000	1.0000	0.5000	0.0000	0.0000	0.5000	0.0000
2.0000	0.5000	55.3150	0.0000	0.0000	0.0000	0.0000	0.6667	0.6667	0.0000	0.0000	0.6667	0.0000
NA	NA	NA	0.0000	0.0000	0.0000	0.0000	1.0000	0.0000	0.0000	0.0000	0.0000	0.0000
NA	NA	NA	0.0000	0.0000	0.0000	0.0000	1.0000	0.0000	0.0000	0.0000	0.0000	0.0000
2.0000	0.5000	59.3650	0.0000	0.0000	0.0000	0.0000	0.5000	1.0000	0.0000	0.0000	1.0000	0.0000
1.0000	0.0000	50.0000	0.0000	0.0000	0.0000	1.0000	1.0000	1.0000	0.0000	0.0000	0.0000	0.0000
NA	NA	NA	0.0000	0.0000	0.0000	0.0000	1.0000	0.0000	0.0000	0.0000	0.0000	0.0000
2.5000	0.7500	80.7950	0.0000	0.0000	0.0000	0.0000	0.6667	0.6667	0.0000	0.0000	0.3333	0.0000
1.8333	0.4167	63.3650	0.0000	0.0000	0.0000	0.4286	0.7143	0.8571	0.0000	0.0000	0.5714	0.0000
2.0000	0.5000	67.2240	0.0000	0.0000	0.0000	0.1667	0.3333	0.8333	0.0000	0.0000	0.6667	0.1667
1.5000	0.2500	64.2500	0.0000	0.0000	0.0000	0.3333	0.6667	0.6667	0.0000	0.0000	0.3333	0.0000
1.5000	0.2500	62.0025	0.2500	0.0000	0.0000	0.0000	0.0000	1.0000	0.0000	0.0000	0.5000	0.0000
1.7500	0.3750	70.1050	0.1111	0.0000	0.0000	0.4444	0.3333	0.8889	0.0000	0.0000	0.6667	0.2222
1.8571	0.4286	74.7267	0.0000	0.0000	0.0000	0.2500	0.5000	0.7500	0.0000	0.0000	0.7500	0.2500
1.7143	0.3571	67.6800	0.0000	0.0000	0.0000	0.4286	0.2857	1.0000	0.0000	0.0000	0.7143	0.1429
1.0000	0.0000	86.7500	0.0000	0.0000	0.0000	0.5000	0.0000	1.0000	0.0000	0.0000	0.5000	0.5000
1.6250	0.3125	64.1086	0.0000	0.0000	0.0000	0.3333	0.3333	0.7778	0.0000	0.0000	0.4444	0.0000
1.5000	0.2500	74.6713	0.1000	0.0000	0.0000	0.3000	0.3000	0.8000	0.0000	0.0000	0.7000	0.2000
1.6000	0.3000	70.8822	0.2000	0.0000	0.0000	0.3000	0.3000	0.9000	0.1000	0.0000	0.6000	0.2000
1.4000	0.2000	71.0578	0.1818	0.0000	0.0000	0.2727	0.3636	0.8182	0.0909	0.0000	0.7273	0.1818
2.0000	0.5000	75.8725	0.0000	0.0000	0.0000	0.0000	0.3333	0.6667	0.0000	0.0000	0.6667	0.0000
1.6000	0.3000	73.4844	0.0909	0.0000	0.0000	0.3636	0.2727	0.8182	0.0000	0.0909	0.6364	0.0909
1.4000	0.2000	54.9875	0.1000	0.0000	0.0000	0.3000	0.3000	0.8000	0.0000	0.0000	0.7000	0.0000
1.6667	0.3333	51.8000	0.0000	0.0000	0.0000	0.0000	0.3333	1.0000	0.0000	0.0000	1.0000	0.0000
1.5000	0.2500	51.8000	0.0000	0.0000	0.0000	0.0000	0.2500	0.7500	0.0000	0.0000	0.7500	0.0000
1.4000	0.2000	58.1000	0.0000	0.0000	0.0000	0.0000	0.0000	0.6000	0.0000	0.0000	0.6000	0.0000
1.0000	0.0000	36.6700	0.0000	0.0000	0.0000	0.0000	0.3333	0.3333	0.0000	0.0000	0.3333	0.0000
1.3750	0.1875	64.8257	0.0000	0.0000	0.0000	0.2222	0.4444	0.7778	0.0000	0.0000	0.5556	0.1111

1.6000	0.3000	74.5880	0.0000	0.0000	0.0000	0.2000	0.2000	1.0000	0.0000	0.0000	0.4000	0.0000
1.5000	0.2500	70.8900	0.0000	0.0000	0.0000	0.2500	0.2500	1.0000	0.0000	0.0000	0.5000	0.0000
1.0000	0.0000	44.7700	0.0000	0.0000	0.0000	0.0000	0.0000	1.0000	0.0000	0.0000	1.0000	0.0000
1.5000	0.2500	66.3150	0.0000	0.0000	0.0000	0.3333	0.3333	0.6667	0.0000	0.0000	0.6667	0.3333
1.6667	0.3333	73.4200	0.0000	0.0000	0.0000	0.2500	0.2500	0.7500	0.0000	0.0000	0.5000	0.2500
2.0000	0.5000	76.9067	0.0000	0.0000	0.0000	0.5000	0.5000	0.7500	0.0000	0.0000	0.2500	0.2500
1.6667	0.3333	63.6550	0.0000	0.0000	0.0000	1.0000	0.6667	1.0000	0.0000	0.0000	1.0000	0.3333

1.3333	0.1667	56.4333	0.0000	0.0000	0.0000	0.2500	0.2500	0.7500	0.0000	0.0000	0.7500	0.2500
2.0000	0.5000	59.3650	0.0000	0.0000	0.0000	0.0000	0.6667	0.6667	0.0000	0.0000	0.6667	0.0000
1.5000	0.2500	66.3150	0.0000	0.0000	0.0000	0.5000	0.0000	1.0000	0.0000	0.0000	1.0000	0.5000
1.0000	0.0000	71.6350	0.0000	0.0000	0.0000	0.0000	0.0000	1.0000	0.0000	0.0000	0.5000	0.0000
2.0000	0.5000	87.8600	0.0000	0.0000	0.0000	0.5000	0.5000	0.5000	0.0000	0.0000	0.5000	0.5000
2.0000	0.5000	87.8600	0.0000	0.0000	0.0000	0.5000	0.5000	0.5000	0.0000	0.0000	0.5000	0.5000
2.2500	0.6250	74.8625	0.0000	0.0000	0.0000	0.2000	0.4000	0.8000	0.0000	0.0000	0.6000	0.2000
1.7143	0.3571	81.7300	0.1250	0.0000	0.0000	0.2500	0.2500	0.7500	0.0000	0.0000	0.6250	0.2500

1.9583	0.4792	54.7393	0.1111	0.0000	0.0000	0.7037	0.4815	0.8519	0.2222	0.5185	0.8519	0.1481
1.8800	0.4400	55.4630	0.1111	0.0000	0.0370	0.7407	0.4815	0.8889	0.1852	0.4815	0.8519	0.1852
1.9167	0.4583	58.2468	0.0714	0.0000	0.0357	0.7857	0.5000	0.8571	0.1071	0.4643	0.7857	0.1071
1.8387	0.4194	59.9156	0.1471	0.0000	0.0294	0.7353	0.4412	0.8529	0.1765	0.3824	0.8235	0.2353
1.9091	0.4545	64.5850	0.2083	0.0000	0.0417	0.7917	0.4583	0.8750	0.1250	0.3750	0.8333	0.0833
1.8182	0.4091	58.3350	0.1250	0.0000	0.0000	0.7083	0.3750	0.8750	0.1667	0.4583	0.8333	0.1667
1.8636	0.4318	61.6438	0.0800	0.0000	0.0000	0.6400	0.4400	0.8400	0.0400	0.3200	0.7600	0.1200
1.8696	0.4348	61.5311	0.1200	0.0000	0.0000	0.7200	0.5200	0.8000	0.0400	0.4000	0.7600	0.2000
1.8947	0.4474	60.9994	0.0952	0.0000	0.0000	0.7143	0.4762	0.8571	0.0476	0.4286	0.7619	0.0476
1.8462	0.4231	61.0562	0.1429	0.0000	0.0000	0.7143	0.5357	0.8214	0.0357	0.3571	0.7857	0.2857
1.9231	0.4615	60.8214	0.1429	0.0000	0.0000	0.7143	0.5357	0.8214	0.0714	0.3214	0.7857	0.2500
1.8929	0.4464	60.3868	0.1000	0.0000	0.0000	0.7000	0.4667	0.9000	0.1000	0.3333	0.8333	0.2333
1.7778	0.3889	58.7148	0.1724	0.0000	0.0000	0.7586	0.4828	0.8621	0.0690	0.2759	0.7586	0.2759
1.7419	0.3710	59.5700	0.1515	0.0000	0.0000	0.7273	0.4848	0.8788	0.0606	0.3333	0.8485	0.2424
1.9286	0.4643	56.6348	0.0645	0.0000	0.0000	0.7097	0.5161	0.8710	0.0645	0.3548	0.8065	0.1290
1.9000	0.4500	60.3575	0.0952	0.0000	0.0476	0.7619	0.4286	0.9524	0.1429	0.4286	0.9048	0.2381
1.8462	0.4231	63.8763	0.1481	0.0000	0.0370	0.7407	0.4815	0.9259	0.0370	0.4074	0.8519	0.1481
1.9167	0.4583	64.2650	0.1200	0.0000	0.0400	0.8000	0.4400	0.9200	0.1200	0.4800	0.9200	0.2000
1.8800	0.4400	57.6948	0.0769	0.0000	0.0385	0.7308	0.3462	0.9615	0.1154	0.3846	0.8077	0.1538
1.8889	0.4444	57.1441	0.1053	0.0000	0.0000	0.6842	0.3158	0.9474	0.1053	0.4737	0.7368	0.0000
1.8636	0.4318	62.1626	0.1304	0.0000	0.0435	0.7391	0.4348	0.9130	0.0870	0.4348	0.7826	0.1739
1.9130	0.4565	64.3995	0.0870	0.0000	0.0000	0.8261	0.5652	0.9565	0.0870	0.4783	0.8696	0.1739
1.8750	0.4375	61.2555	0.0769	0.0000	0.0000	0.8077	0.5385	0.9231	0.0769	0.4231	0.8462	0.1923
1.7692	0.3846	59.5105	0.1111	0.0000	0.0370	0.7778	0.4444	0.8889	0.0741	0.4074	0.8889	0.2222
1.8182	0.4091	60.4244	0.1304	0.0000	0.0000	0.7826	0.4783	0.8261	0.0870	0.4348	0.7826	0.2174
1.8571	0.4286	61.3419	0.1304	0.0000	0.0000	0.8261	0.4783	0.8261	0.0870	0.4348	0.7826	0.1739
1.9091	0.4545	62.1006	0.0870	0.0000	0.0000	0.7391	0.4783	0.8261	0.0870	0.4348	0.7826	0.2174
1.8400	0.4200	63.2224	0.1111	0.0000	0.0000	0.7778	0.4815	0.8519	0.1111	0.4074	0.8519	0.2222
1.8077	0.4038	59.8405	0.1111	0.0000	0.0000	0.7407	0.4444	0.8519	0.1111	0.3704	0.8148	0.2963
1.7407	0.3704	60.6164	0.1071	0.0000	0.0357	0.7143	0.3571	0.8929	0.0714	0.3571	0.7857	0.2500
1.9500	0.4750	60.5300	0.0909	0.0000	0.0000	0.6818	0.4091	0.8636	0.0909	0.2727	0.7727	0.0909
1.9048	0.4524	65.6300	0.1364	0.0000	0.0000	0.7727	0.4545	0.8636	0.0909	0.3636	0.8636	0.0909
1.9000	0.4500	67.6922	0.1429	0.0000	0.0000	0.8571	0.5238	0.9048	0.1429	0.4286	0.9048	0.0952
1.8571	0.4286	67.3550	0.0435	0.0000	0.0000	0.7826	0.4783	0.8696	0.1304	0.4348	0.8261	0.0870
1.8500	0.4250	66.3882	0.0476	0.0000	0.0000	0.8571	0.5714	0.9048	0.0952	0.4762	0.8095	0.1429
1.6818	0.3409	64.8216	0.1667	0.0000	0.0000	0.7917	0.4167	0.8750	0.0417	0.3750	0.8750	0.1250
1.8214	0.4107	64.4792	0.1379	0.0000	0.0000	0.8276	0.4483	0.9310	0.1034	0.3448	0.8276	0.0690
1.8261	0.4130	66.6105	0.1250	0.0000	0.0000	0.8333	0.4583	0.9167	0.0833	0.3333	0.8333	0.0417
1.8667	0.4333	63.8867	0.1290	0.0000	0.0000	0.8387	0.4839	0.9355	0.0968	0.3871	0.8387	0.1290
1.9091	0.4545	64.6018	0.1538	0.0385	0.0000	0.7692	0.4231	0.9231	0.1154	0.3462	0.8462	0.1154
1.8000	0.4000	63.5661	0.1111	0.0000	0.0000	0.6667	0.4815	0.8889	0.0741	0.3333	0.8519	0.0741
1.7857	0.3929	60.9308	0.2333	0.0000	0.0000	0.7000	0.4000	0.9000	0.0667	0.3000	0.8333	0.1000
1.7931	0.3966	59.5862	0.1290	0.0000	0.0000	0.7742	0.4194	0.9032	0.0968	0.3226	0.8065	0.1290
1.7500	0.3750	59.4397	0.1176	0.0000	0.0000	0.7353	0.4118	0.9118	0.0882	0.2353	0.8529	0.1176
1.8148	0.4074	62.3560	0.1379	0.0000	0.0000	0.7931	0.4138	0.8966	0.1034	0.3103	0.8621	0.0690
1.9565	0.4783	70.1822	0.1429	0.0357	0.0000	0.6071	0.3571	0.8214	0.0714	0.3214	0.8214	0.0714
1.8421	0.4211	64.2519	0.1429	0.0000	0.0000	0.7143	0.4286	0.8571	0.0952	0.3333	0.8571	0.0476
1.7500	0.3750	60.4459	0.1538	0.0000	0.0000	0.8077	0.4231	0.8846	0.0769	0.4231	0.8462	0.1154
1.9545	0.4773	67.5920	0.1667	0.0000	0.0000	0.7500	0.5417	0.8750	0.0833	0.3333	0.8333	0.1250
1.7778	0.3889	60.6671	0.1379	0.0000	0.0000	0.6897	0.4138	0.8966	0.0690	0.3448	0.8276	0.1034
1.9000	0.4500	64.4993	0.2188	0.0000	0.0000	0.7188	0.4375	0.9063	0.0625	0.3438	0.8438	0.0938
1.8077	0.4038	60.9679	0.1071	0.0000	0.0000	0.6786	0.4286	0.8929	0.0714	0.3214	0.8571	0.1071
1.8621	0.4310	62.0100	0.1290	0.0000	0.0000	0.6774	0.4194	0.9032	0.0645	0.3226	0.8387	0.0645
1.8276	0.4138	58.6967	0.1290	0.0000	0.0323	0.6452	0.4194	0.8710	0.1290	0.3226	0.8710	0.1290
1.8000	0.4000	60.8894	0.1875	0.0000	0.0313	0.7500	0.3750	0.9063	0.1250	0.3750	0.8125	0.0938
1.7619	0.3810	57.1100	0.1304	0.0000	0.0435	0.6957	0.5217	0.8696	0.0870	0.2609	0.8696	0.1304
1.7895	0.3947	64.0044	0.1429	0.0000	0.0000	0.6667	0.4762	0.9048	0.0476	0.2857	0.7619	0.1429
1.7500	0.3750	57.9716	0.0909	0.0000	0.0000	0.7273	0.5000	0.9091	0.0455	0.2727	0.7727	0.1364
1.7600	0.3800	60.0133	0.1481	0.0000	0.0000	0.7037	0.4815	0.9259	0.0741	0.2593	0.8519	0.1852

1.8214	0.4107	61.2900	0.1333	0.0000	0.0000	0.7333	0.4000	0.9000	0.0667	0.2333	0.8333	0.1333
1.6250	0.3125	55.2200	0.0000	0.0000	0.0000	0.8889	0.3333	0.8889	0.5556	0.3333	0.8889	0.1111
1.6250	0.3125	62.3550	0.1111	0.0000	0.0000	0.8889	0.3333	0.8889	0.3333	0.5556	0.8889	0.1111
1.9000	0.4500	60.1495	0.1250	0.0000	0.0417	0.3750	0.2083	0.8750	0.1250	0.1667	0.7500	0.0417
1.8333	0.4167	56.6489	0.0952	0.0000	0.0000	0.3333	0.0952	0.8571	0.0000	0.1429	0.6667	0.0476
1.8182	0.4091	56.6891	0.0800	0.0000	0.0400	0.3600	0.2000	0.8800	0.0800	0.2000	0.7600	0.0800
1.6364	0.3182	52.2760	0.0769	0.0000	0.0000	0.3077	0.1538	0.7692	0.0769	0.1538	0.7692	0.0000
1.7647	0.3824	52.6550	0.1000	0.0000	0.0500	0.3000	0.1500	0.8500	0.1000	0.0500	0.7000	0.0500
1.7500	0.3750	60.1571	0.1111	0.0000	0.0000	0.2222	0.3333	0.7778	0.1111	0.3333	0.6667	0.1111
2.0000	0.5000	69.2862	0.1538	0.0000	0.0000	1.0000	0.6154	1.0000	0.2308	0.5385	1.0000	0.0769
1.6667	0.3333	57.4893	0.1176	0.0000	0.0000	0.4118	0.2353	0.8824	0.0588	0.1765	0.8235	0.1765
2.0000	0.5000	66.5908	0.1429	0.0000	0.0000	0.5714	0.2857	1.0000	0.1429	0.3571	0.8571	0.0714
1.8750	0.4375	81.6467	0.2222	0.0000	0.0000	0.8889	0.4444	1.0000	0.1111	0.4444	1.0000	0.0000
1.9375	0.4688	64.2842	0.1429	0.0000	0.0000	0.3810	0.1429	0.9524	0.0476	0.0952	0.7143	0.0476
1.7778	0.3889	69.1291	0.0833	0.0000	0.0000	0.6667	0.2500	1.0000	0.0000	0.1667	0.9167	0.0000
1.6667	0.3333	59.1055	0.2308	0.0000	0.0000	0.2308	0.0000	0.9231	0.0000	0.0000	0.7692	0.0000
1.7500	0.3750	66.7250	0.1000	0.0000	0.0000	0.5000	0.2000	1.0000	0.0000	0.1000	0.8000	0.0000
1.6667	0.3333	58.5070	0.3077	0.0000	0.0000	0.2308	0.0769	0.9231	0.0000	0.0000	0.6154	0.0000
2.0000	0.5000	54.8136	0.0000	0.0000	0.0000	0.3333	0.2500	1.0000	0.0833	0.1667	0.7500	0.0833
1.7500	0.3750	66.8025	0.2500	0.0000	0.0000	0.7500	0.3750	1.0000	0.0625	0.3750	0.9375	0.0625
1.5000	0.2500	53.8511	0.1111	0.0000	0.0000	0.1111	0.0000	1.0000	0.1111	0.0000	0.6667	0.0000
1.8333	0.4167	67.8314	0.0000	0.0000	0.0000	0.3333	0.2222	1.0000	0.0000	0.2222	1.0000	0.0000
1.8750	0.4375	64.0473	0.2000	0.0000	0.0000	0.1333	0.0667	0.8667	0.0000	0.0000	0.6000	0.0000
1.8889	0.4444	69.2388	0.1111	0.0000	0.0000	0.8889	0.5556	1.0000	0.2222	0.5556	1.0000	0.1111
2.3333	0.6667	64.8400	0.1111	0.0000	0.0000	0.8889	0.5556	0.8889	0.3333	0.6667	0.8889	0.0000
1.8095	0.4048	63.3119	0.2000	0.0000	0.0400	0.6400	0.2800	0.9200	0.0800	0.2400	0.8400	0.1200
1.7778	0.3889	67.0478	0.1579	0.0000	0.0526	0.7895	0.2632	1.0000	0.1053	0.4211	0.9474	0.1053
1.9286	0.4643	62.8927	0.1333	0.0000	0.0667	0.6667	0.2667	1.0000	0.1333	0.2667	1.0000	0.2000
2.2500	0.6250	68.0600	0.0000	0.0000	0.0000	0.8000	0.6000	0.8000	0.2000	0.2000	0.8000	0.0000
1.8000	0.4000	51.7580	0.0000	0.0000	0.0000	1.0000	0.7000	1.0000	0.4000	0.3000	1.0000	0.1000
1.8750	0.4375	67.7893	0.1875	0.0000	0.0000	0.8125	0.3750	0.9375	0.2500	0.5000	0.9375	0.0625
2.0476	0.5238	57.5905	0.0952	0.0000	0.0000	0.9524	0.5714	0.9524	0.2381	0.4762	0.9524	0.1905
1.7500	0.3750	50.8885	0.1765	0.0000	0.0000	0.7647	0.3529	0.8824	0.4118	0.4118	0.8824	0.1765
2.0000	0.5000	55.2667	0.0714	0.0000	0.0000	0.9286	0.5714	0.9286	0.2857	0.4286	0.9286	0.2143
1.9091	0.4545	54.4136	0.1667	0.0000	0.0000	0.9167	0.3333	0.9167	0.3333	0.4167	0.9167	0.0000
1.7619	0.3810	58.8290	0.1364	0.0000	0.0000	0.8636	0.4091	1.0000	0.3636	0.5455	1.0000	0.0909
1.8235	0.4118	47.1100	0.0000	0.0000	0.0000	0.8824	0.4706	0.9412	0.4706	0.4706	0.9412	0.1176
1.6875	0.3438	54.7325	0.0588	0.0000	0.0000	0.8235	0.4706	1.0000	0.2941	0.5294	1.0000	0.1176
1.7143	0.3571	57.2555	0.0476	0.0000	0.0000	0.7619	0.3810	0.9524	0.3333	0.3810	0.9048	0.1429
1.8571	0.4286	56.1295	0.1818	0.0000	0.0000	0.8636	0.3182	0.9545	0.3182	0.5000	0.8636	0.1364
1.8125	0.4063	57.7847	0.1250	0.0000	0.0000	0.7500	0.2500	1.0000	0.4375	0.5000	0.9375	0.1250
1.9333	0.4667	62.7138	0.1250	0.0000	0.0000	0.8125	0.5000	0.8750	0.2500	0.5000	0.8750	0.1250
2.0000	0.5000	70.0323	0.0769	0.0000	0.0000	0.7692	0.4615	1.0000	0.0769	0.3846	1.0000	0.2308
1.6000	0.3000	81.8000	0.0000	0.0000	0.0000	1.0000	0.6667	1.0000	0.1667	0.5000	1.0000	0.1667
1.7500	0.3750	57.0719	0.1176	0.0000	0.0000	0.9412	0.3529	0.9412	0.2941	0.3529	0.8824	0.1176
1.8500	0.4250	62.4195	0.0500	0.0000	0.0000	0.6500	0.4500	1.0000	0.2500	0.3500	0.9000	0.2000
1.8182	0.4091	71.1958	0.0000	0.0000	0.0000	0.8333	0.5000	1.0000	0.1667	0.5000	1.0000	0.1667
2.0000	0.5000	84.8167	0.0000	0.0000	0.0000	0.8333	0.5000	1.0000	0.0000	0.3333	1.0000	0.0000
1.7500	0.3750	62.1400	0.2500	0.0000	0.0000	0.6250	0.3750	0.8750	0.0000	0.0000	0.8750	0.1250
1.9091	0.4545	76.3792	0.2308	0.0000	0.0000	0.6923	0.3846	1.0000	0.0769	0.2308	0.8462	0.0769
1.6667	0.3333	60.1314	0.1875	0.0000	0.0000	0.4375	0.3750	0.8750	0.0000	0.0000	0.8125	0.1250
2.0000	0.5000	64.0844	0.0000	0.0000	0.0625	0.9375	0.4375	1.0000	0.3750	0.3750	1.0000	0.2500
1.6500	0.3250	59.2375	0.1818	0.0000	0.0000	0.6818	0.3182	0.9091	0.2273	0.2727	0.9091	0.0000
2.0500	0.5250	63.7835	0.1364	0.0000	0.0455	0.7273	0.4545	0.9545	0.2727	0.4545	0.9091	0.2273
2.0000	0.5000	64.1862	0.0714	0.0000	0.0000	0.8571	0.4286	0.9286	0.3571	0.5000	0.9286	0.1429
2.0909	0.5455	67.7342	0.0800	0.0400	0.0000	0.6800	0.5200	0.9600	0.2400	0.2800	0.8800	0.1600
1.8750	0.4375	50.2347	0.1875	0.0000	0.0000	0.8125	0.3750	0.9375	0.3750	0.4375	0.9375	0.0625
1.9333	0.4667	70.4559	0.1500	0.0500	0.0000	0.6500	0.5000	0.9000	0.2000	0.3000	0.9000	0.2000
1.9091	0.4545	64.1582	0.0909	0.0000	0.0000	0.9091	0.5455	1.0000	0.3636	0.5455	1.0000	0.0909
1.8333	0.4167	63.5106	0.2105	0.0000	0.0000	0.6316	0.4211	0.9474	0.1579	0.2632	0.7895	0.0000
2.0000	0.5000	73.5741	0.1000	0.0500	0.0000	0.7000	0.5000	0.9000	0.2000	0.4000	0.8500	0.1000
1.9167	0.4583	68.4479	0.0714	0.0000	0.0000	0.9286	0.5714	1.0000	0.2143	0.4286	0.9286	0.0714
2.0000	0.5000	55.2747	0.0000	0.0000	0.0000	0.8889	0.5556	0.9444	0.4444	0.4444	0.9444	0.0556
2.0000	0.5000	70.4100	0.1176	0.0000	0.0000	0.7059	0.4706	0.9412	0.1765	0.4706	0.8824	0.1765
1.9286	0.4643	63.4533	0.0714	0.0000	0.0000	0.8571	0.4286	1.0000	0.2143	0.5714	0.9286	0.0714
1.9286	0.4643	66.0886	0.1333	0.0000	0.0000	0.8000	0.4667	1.0000	0.2000	0.4667	0.9333	0.0667
1.9375	0.4688	67.2982	0.0588	0.0000	0.0000	0.6471	0.2941	1.0000	0.2353	0.3529	0.8824	0.0588
1.7857	0.3929	64.4113	0.0625	0.0000	0.0000	0.6875	0.3750	0.9375	0.1875	0.2500	0.7500	0.0625
1.9286	0.4643	59.0154	0.0000	0.0000	0.0000	0.5714	0.2143	1.0000	0.2857	0.3571	0.9286	0.0714
2.0000	0.5000	60.7987	0.0588	0.0000	0.0000	0.6471	0.1765	0.8824	0.4118	0.4118	0.7647	0.1176
1.9000	0.4500	74.0975	0.0000	0.0000	0.0000	0.2500	0.1667	0.9167	0.0000	0.3333	0.8333	0.0833
1.9167	0.4583	71.4621	0.2667	0.0000	0.0000	0.7333	0.4000	0.9333	0.2000	0.4000	0.8667	0.0667
1.9412	0.4706	58.5535	0.0000	0.0000	0.0000	0.8889	0.5556	0.9444	0.3889	0.4444	0.9444	0.0556
2.0000	0.5000	70.7621	0.1500	0.0000	0.0000	0.6000	0.3500	0.9500	0.2000	0.3500	0.8500	0.1000
2.1304	0.5652	58.0552	0.1667	0.0000	0.0833	0.8750	0.3750	0.9583	0.4167	0.4583	0.9583	0.1667
1.8261	0.4130	65.4208	0.1538	0.0000	0.0000	0.6923	0.5385	0.9615	0.2308	0.3077	0.9231	0.2308
1.8333	0.4167	56.6575	0.1538	0.0000	0.0000	0.9231	0.3846	0.9231	0.3077	0.3077	0.9231	0.0769

1.8750	0.4375	73.0783	0.1579	0.0526	0.0000	0.7368	0.3684	0.9474	0.1579	0.3158	0.9474	0.1579
2.0000	0.5000	68.9090	0.0000	0.0000	0.0000	1.0000	0.6000	1.0000	0.2000	0.5000	1.0000	0.0000
2.0000	0.5000	48.6120	0.0000	0.0000	0.0000	0.8333	0.3333	0.8333	0.5000	0.6667	0.8333	0.1667
2.1250	0.5625	83.0300	0.1000	0.1000	0.0000	0.6000	0.4000	1.0000	0.1000	0.3000	0.9000	0.1000
1.8182	0.4091	58.0517	0.0000	0.0000	0.0000	1.0000	0.5833	1.0000	0.4167	0.4167	1.0000	0.0833
2.0000	0.5000	73.0988	0.0000	0.0000	0.0000	0.3333	0.2222	1.0000	0.0000	0.1111	0.7778	0.0000
2.3333	0.6667	70.8229	0.1333	0.0000	0.0000	0.4000	0.4667	0.9333	0.0667	0.0667	0.6667	0.1333
1.8333	0.4167	66.5806	0.2632	0.0000	0.0000	0.5789	0.3684	1.0000	0.0526	0.2632	0.8947	0.1053
2.0000	0.5000	61.8027	0.0769	0.0000	0.0000	0.9231	0.6154	0.9231	0.2308	0.3846	0.8462	0.0000
2.0769	0.5385	73.5985	0.0000	0.0000	0.0000	0.7143	0.6429	1.0000	0.0714	0.4286	0.8571	0.0000
1.6923	0.3462	54.4500	0.0714	0.0000	0.0714	0.2857	0.0714	0.8571	0.0714	0.0714	0.7857	0.1429
2.1250	0.5625	61.7425	0.0000	0.0000	0.0000	1.0000	0.6250	1.0000	0.3750	0.7500	1.0000	0.1250
1.7692	0.3846	51.7377	0.1333	0.0000	0.0000	0.3333	0.2000	0.8667	0.0667	0.0667	0.7333	0.0667
1.8462	0.4231	73.7820	0.0667	0.0000	0.0000	0.8667	0.4000	1.0000	0.1333	0.2667	0.8667	0.0667
2.0000	0.5000	67.2091	0.0833	0.0000	0.0000	0.7500	0.2500	1.0000	0.1667	0.2500	0.9167	0.0000
2.0000	0.5000	54.3586	0.0000	0.0000	0.0000	0.8750	0.3750	0.8750	0.5000	0.5000	0.8750	0.1250
2.0909	0.5455	70.1625	0.1538	0.0000	0.0769	0.6154	0.3077	0.9231	0.1538	0.0769	0.7692	0.0000
1.9412	0.4706	63.1793	0.0588	0.0000	0.0000	0.8235	0.5882	0.9412	0.1765	0.4118	0.9412	0.0588
2.0000	0.5000	59.1176	0.1111	0.0000	0.0000	0.8333	0.5000	1.0000	0.1667	0.3889	0.8889	0.0556
2.0833	0.5417	63.1527	0.0833	0.0000	0.0000	0.9167	0.5000	0.9167	0.1667	0.5833	0.8333	0.0833
2.1000	0.5500	70.4900	0.0000	0.0000	0.0000	0.7273	0.6364	1.0000	0.2727	0.4545	0.9091	0.0909
2.1111	0.5556	65.8663	0.0000	0.0000	0.0000	0.8889	0.6667	0.8889	0.2222	0.5556	0.8889	0.1111
1.7857	0.3929	65.7350	0.2000	0.0000	0.0000	0.9333	0.5333	1.0000	0.2667	0.5333	1.0000	0.0667
1.9286	0.4643	69.4979	0.1765	0.0000	0.0000	0.7647	0.5882	0.9412	0.0588	0.4706	0.8824	0.0588
1.8235	0.4118	68.4044	0.1053	0.0000	0.0526	0.4211	0.1579	0.8421	0.1053	0.2105	0.7895	0.0526
1.4000	0.2000	57.3250	0.0000	0.0000	0.0000	0.4000	0.2000	0.8000	0.0000	0.0000	0.8000	0.2000
1.4444	0.2222	73.8025	0.0000	0.0000	0.0000	0.2222	0.2222	0.8889	0.0000	0.0000	0.4444	0.1111
1.2500	0.1250	63.1443	0.0000	0.0000	0.0000	0.2500	0.2500	0.8750	0.0000	0.0000	0.6250	0.0000
1.5000	0.2500	81.0867	0.0909	0.0000	0.0000	0.0909	0.1818	0.8182	0.0909	0.0000	0.5455	0.0000
1.5714	0.2857	83.5467	0.1429	0.0000	0.0000	0.0000	0.1429	0.8571	0.1429	0.0000	0.5714	0.0000
1.6000	0.3000	79.2900	0.0000	0.0000	0.0000	0.0000	0.0000	0.6667	0.0000	0.0000	0.5000	0.0000
1.2500	0.1250	75.4500	0.2500	0.0000	0.0000	0.0000	0.2500	0.7500	0.2500	0.0000	0.7500	0.0000
1.1429	0.0714	64.2240	0.1429	0.0000	0.0000	0.1429	0.2857	0.7143	0.1429	0.0000	0.5714	0.0000
1.1667	0.0833	74.2240	0.1429	0.0000	0.0000	0.0000	0.1429	0.7143	0.1429	0.0000	0.8571	0.0000
1.5000	0.2500	75.8580	0.0000	0.0000	0.0000	0.0000	0.1429	0.7143	0.0000	0.0000	0.7143	0.0000
1.4444	0.2222	66.8300	0.1111	0.0000	0.0000	0.1111	0.3333	0.7778	0.1111	0.0000	0.4444	0.0000
1.6000	0.3000	75.8580	0.0000	0.0000	0.0000	0.0000	0.1667	0.8333	0.0000	0.0000	0.6667	0.0000
1.2857	0.1429	55.8040	0.1429	0.0000	0.0000	0.1429	0.4286	0.7143	0.1429	0.0000	0.5714	0.0000
1.5556	0.2778	72.8522	0.1000	0.0000	0.0000	0.2000	0.3000	0.9000	0.1000	0.0000	0.6000	0.1000
1.5000	0.2500	73.3122	0.1818	0.0000	0.0000	0.0909	0.3636	0.8182	0.1818	0.0000	0.6364	0.0000
1.4286	0.2143	53.6150	0.1429	0.0000	0.0000	0.4286	0.4286	0.8571	0.0000	0.0000	0.5714	0.0000
1.3750	0.1875	63.2314	0.1250	0.0000	0.0000	0.3750	0.2500	0.8750	0.0000	0.0000	0.7500	0.1250
1.3333	0.1667	67.5240	0.1667	0.0000	0.0000	0.1667	0.1667	0.8333	0.0000	0.0000	0.6667	0.0000
1.1250	0.0625	66.0017	0.0000	0.0000	0.0000	0.0000	0.0000	0.7500	0.0000	0.0000	0.6250	0.0000
1.2857	0.1429	52.6600	0.0000	0.0000	0.0000	0.2500	0.1250	0.6250	0.0000	0.0000	0.6250	0.1250
1.0000	0.0000	65.5550	0.0000	0.0000	0.0000	0.0000	0.0000	0.6667	0.0000	0.0000	1.0000	0.0000
1.7500	0.3750	64.4225	0.0000	0.0000	0.0000	0.2500	0.2500	1.0000	0.0000	0.0000	0.7500	0.0000
2.0000	0.5000	88.1250	0.0000	0.0000	0.0000	0.3333	0.3333	0.6667	0.0000	0.0000	0.6667	0.0000
1.2857	0.1429	92.0240	0.0000	0.0000	0.0000	0.2500	0.1250	0.6250	0.0000	0.0000	0.7500	0.1250
1.4000	0.2000	77.1267	0.2000	0.0000	0.0000	0.2000	0.4000	0.6000	0.2000	0.0000	0.4000	0.0000
1.0000	0.0000	59.1233	0.0000	0.0000	0.0000	0.0000	0.0000	0.6000	0.0000	0.0000	1.0000	0.0000
1.5000	0.2500	93.7933	0.2000	0.0000	0.0000	0.0000	0.2000	0.6000	0.2000	0.0000	0.6000	0.0000
1.5000	0.2500	76.9150	0.0000	0.0000	0.0000	0.0000	0.5000	0.5000	0.0000	0.0000	0.5000	0.0000
1.4000	0.2000	79.1300	0.0000	0.0000	0.0000	0.0000	0.3333	0.5000	0.0000	0.0000	0.5000	0.0000
1.4286	0.2143	64.4560	0.1429	0.0000	0.0000	0.0000	0.2857	0.7143	0.1429	0.0000	0.7143	0.0000
1.5000	0.2500	72.6180	0.0000	0.0000	0.0000	0.1667	0.1667	0.8333	0.0000	0.0000	0.5000	0.0000
1.5000	0.2500	78.2725	0.0000	0.0000	0.0000	0.0000	0.0000	0.6667	0.0000	0.0000	0.5000	0.0000
1.6000	0.3000	66.7900	0.0000	0.0000	0.0000	0.2000	0.2000	0.8000	0.0000	0.0000	0.4000	0.0000
1.5714	0.2857	75.2683	0.1429	0.0000	0.0000	0.0000	0.1429	0.8571	0.1429	0.0000	0.5714	0.0000
1.2000	0.1000	58.3967	0.2000	0.0000	0.0000	0.0000	0.2000	0.6000	0.2000	0.0000	0.6000	0.0000
1.2500	0.1250	72.9133	0.0000	0.0000	0.0000	0.0000	0.0000	0.7500	0.0000	0.0000	0.7500	0.0000
1.3333	0.1667	64.0460	0.0000	0.0000	0.0000	0.1667	0.1667	0.8333	0.0000	0.0000	0.6667	0.0000
1.7500	0.3750	90.2450	0.0000	0.0000	0.0000	0.0000	0.0000	0.5000	0.0000	0.0000	0.5000	0.0000
1.3333	0.1667	59.0575	0.0000	0.0000	0.0000	0.1667	0.3333	0.6667	0.0000	0.0000	0.5000	0.0000
1.8000	0.4000	85.7600	0.1667	0.0000	0.0000	0.3333	0.5000	0.6667	0.1667	0.0000	0.5000	0.0000
1.4286	0.2143	86.4200	0.1429	0.0000	0.0000	0.1429	0.2857	0.7143	0.1429	0.0000	0.5714	0.0000
1.6667	0.3333	84.8480	0.1429	0.0000	0.0000	0.1429	0.2857	0.7143	0.1429	0.0000	0.4286	0.0000
1.5000	0.2500	76.2760	0.1429	0.0000	0.0000	0.1429	0.2857	0.7143	0.1429	0.0000	0.5714	0.0000
1.4286	0.2143	79.5517	0.1250	0.0000	0.0000	0.1250	0.2500	0.7500	0.1250	0.0000	0.6250	0.0000
1.6000	0.3000	78.5240	0.0000	0.0000	0.0000	0.1667	0.3333	0.8333	0.0000	0.0000	0.5000	0.0000
1.7500	0.3750	75.0867	0.0000	0.0000	0.0000	0.0000	0.2000	0.6000	0.0000	0.0000	0.4000	0.0000
1.6000	0.3000	75.0520	0.0000	0.0000	0.0000	0.1429	0.2857	0.7143	0.0000	0.0000	0.4286	0.0000
1.3333	0.1667	63.7480	0.0000	0.0000	0.0000	0.1429	0.2857	0.7143	0.0000	0.0000	0.5714	0.0000
1.6667	0.3333	65.8640	0.0000	0.0000	0.0000	0.2857	0.4286	0.7143	0.0000	0.0000	0.4286	0.1429
1.3333	0.1667	64.8600	0.0000	0.0000	0.0000	0.1250	0.2500	0.6250	0.0000	0.0000	0.6250	0.0000
1.3333	0.1667	67.8217	0.0000	0.0000	0.0000	0.2500	0.2500	0.7500	0.0000	0.0000	0.6250	0.1250
1.5714	0.2857	92.5425	0.1429	0.0000	0.0000	0.0000	0.1429	0.5714	0.1429	0.0000	0.4286	0.0000

1.2500	0.1250	60.8000	0.0000	0.0000	0.0000	0.2500	0.2500	0.7500	0.0000	0.0000	0.5000	0.0000
1.6000	0.3000	70.3450	0.2000	0.0000	0.0000	0.2000	0.4000	0.8000	0.2000	0.0000	0.4000	0.0000
1.8000	0.4000	93.1860	0.1667	0.0000	0.0000	0.1667	0.3333	0.8333	0.1667	0.0000	0.6667	0.0000
1.8333	0.4167	83.7333	0.1429	0.0000	0.0000	0.1429	0.2857	0.8571	0.1429	0.0000	0.5714	0.0000
1.7500	0.3750	85.6550	0.0000	0.0000	0.0000	0.0000	0.2000	0.8000	0.0000	0.0000	0.6000	0.0000
2.0000	0.5000	80.9575	0.2000	0.0000	0.0000	0.2000	0.6000	0.8000	0.2000	0.0000	0.4000	0.0000
1.3333	0.1667	66.4320	0.0000	0.0000	0.0000	0.1667	0.3333	0.8333	0.0000	0.0000	0.5000	0.0000
1.6000	0.3000	68.8150	0.0000	0.0000	0.0000	0.1667	0.3333	0.6667	0.0000	0.0000	0.3333	0.0000
1.6667	0.3333	78.2800	0.0000	0.0000	0.0000	0.1429	0.1429	0.5714	0.0000	0.0000	0.4286	0.1429
1.6000	0.3000	68.8725	0.0000	0.0000	0.0000	0.4000	0.2000	0.8000	0.0000	0.0000	0.6000	0.2000
2.0000	0.5000	88.1250	0.0000	0.0000	0.0000	0.3333	0.3333	0.6667	0.0000	0.0000	0.6667	0.0000
1.6667	0.3333	71.7300	0.0000	0.0000	0.0000	0.3333	0.3333	0.8333	0.0000	0.0000	0.5000	0.0000
1.5000	0.2500	62.5433	0.0000	0.0000	0.0000	0.2000	0.4000	0.6000	0.0000	0.0000	0.2000	0.0000
1.5000	0.2500	79.2867	0.0000	0.0000	0.0000	0.5000	0.5000	0.7500	0.0000	0.0000	0.5000	0.2500
1.6000	0.3000	68.8725	0.0000	0.0000	0.0000	0.3333	0.3333	0.6667	0.0000	0.0000	0.5000	0.1667
1.7500	0.3750	80.8200	0.2000	0.0000	0.0000	0.0000	0.2000	0.8000	0.2000	0.0000	0.6000	0.0000
2.0000	0.5000	76.0000	0.2000	0.0000	0.0000	0.2000	0.4000	0.8000	0.2000	0.0000	0.6000	0.0000
1.4000	0.2000	79.9700	0.0000	0.0000	0.0000	0.3333	0.1667	0.6667	0.0000	0.0000	0.5000	0.1667
1.2857	0.1429	62.2260	0.0000	0.0000	0.0000	0.3750	0.3750	0.6250	0.0000	0.0000	0.5000	0.1250
1.6667	0.3333	67.6400	0.0000	0.0000	0.0000	0.2857	0.4286	0.8571	0.0000	0.0000	0.4286	0.0000
1.6667	0.3333	64.1625	0.1111	0.0000	0.0000	0.3333	0.2222	0.8889	0.0000	0.0000	0.6667	0.1111
2.2000	0.6000	82.5680	0.0000	0.0000	0.0000	0.4000	0.4000	1.0000	0.0000	0.0000	0.6000	0.2000
1.6000	0.3000	67.6838	0.0000	0.0000	0.0000	0.1818	0.2727	0.7273	0.0000	0.0000	0.6364	0.0909
1.8333	0.4167	73.4340	0.0000	0.0000	0.0000	0.2857	0.4286	0.7143	0.0000	0.0000	0.5714	0.1429
1.8889	0.4444	75.3000	0.1000	0.0000	0.0000	0.4000	0.4000	0.8000	0.0000	0.0000	0.4000	0.2000
1.6667	0.3333	56.8611	0.1111	0.0000	0.0000	0.3333	0.4444	1.0000	0.0000	0.0000	0.6667	0.1111
1.6250	0.3125	63.5275	0.1250	0.0000	0.0000	0.6250	0.3750	1.0000	0.0000	0.0000	1.0000	0.2500
1.0000	0.0000	68.5925	0.0000	0.0000	0.0000	0.1667	0.1667	0.6667	0.0000	0.0000	0.3333	0.0000
1.4286	0.2143	67.3267	0.0000	0.0000	0.0000	0.2857	0.2857	0.8571	0.0000	0.0000	0.5714	0.0000
1.4000	0.2000	73.2400	0.0000	0.0000	0.0000	0.2727	0.2727	0.7273	0.0000	0.0000	0.7273	0.0909
1.5714	0.2857	80.9620	0.0000	0.0000	0.0000	0.2857	0.1429	0.7143	0.0000	0.0000	0.7143	0.1429
1.4000	0.2000	67.5575	0.0000	0.0000	0.0000	0.0000	0.1667	0.6667	0.0000	0.0000	0.6667	0.0000
1.5000	0.2500	68.8725	0.0000	0.0000	0.0000	0.2857	0.2857	0.5714	0.0000	0.0000	0.4286	0.1429
1.7500	0.3750	68.8150	0.0000	0.0000	0.0000	0.2000	0.2000	0.8000	0.0000	0.0000	0.2000	0.0000
1.0000	0.0000	55.2760	0.0000	0.0000	0.0000	0.1429	0.1429	0.7143	0.0000	0.0000	0.8571	0.1429
1.5000	0.2500	64.2325	0.0000	0.0000	0.0000	0.2000	0.0000	0.8000	0.0000	0.0000	0.6000	0.2000
1.2000	0.1000	62.2650	0.0000	0.0000	0.0000	0.2000	0.0000	0.4000	0.0000	0.0000	0.8000	0.2000
1.2500	0.1250	66.3075	0.0000	0.0000	0.0000	0.2500	0.0000	1.0000	0.0000	0.0000	1.0000	0.2500
1.5000	0.2500	91.8950	0.0000	0.0000	0.0000	0.3333	0.0000	0.6667	0.0000	0.0000	0.6667	0.3333
NA	NA	NA	0.0000	0.0000	0.0000	0.0000	0.0000	0.0000	0.0000	0.0000	0.0000	0.0000
1.0000	0.0000	43.8133	0.0000	0.0000	0.0000	0.2000	0.4000	0.6000	0.0000	0.0000	0.4000	0.0000
3.0000	1.0000	92.8600	0.0000	0.0000	0.0000	0.0000	0.5000	0.5000	0.0000	0.0000	0.0000	0.0000
1.5000	0.2500	83.3900	0.0000	0.0000	0.0000	0.0000	0.0000	0.8000	0.0000	0.0000	0.8000	0.0000
NA	NA	NA	0.0000	0.0000	0.0000	0.0000	0.0000	0.0000	0.0000	0.0000	0.0000	0.0000
1.8000	0.4000	63.1980	0.0000	0.0000	0.0000	0.1667	0.3333	0.8333	0.0000	0.0000	0.5000	0.1667
1.5000	0.2500	71.6180	0.0000	0.0000	0.0000	0.1429	0.1429	0.7143	0.0000	0.0000	0.7143	0.1429
1.0000	0.0000	70.3500	0.0000	0.0000	0.0000	0.0000	0.0000	0.5000	0.0000	0.0000	0.7500	0.0000
1.0000	0.0000	81.9767	0.0000	0.0000	0.0000	0.2500	0.2500	0.7500	0.0000	0.0000	0.7500	0.0000
1.0000	0.0000	50.0000	0.0000	0.0000	0.0000	1.0000	1.0000	1.0000	0.0000	0.0000	0.0000	0.0000
1.5000	0.2500	70.8900	0.0000	0.0000	0.0000	0.2000	0.4000	0.8000	0.0000	0.0000	0.4000	0.0000
1.8000	0.4000	77.1867	0.0000	0.0000	0.0000	0.3333	0.1667	1.0000	0.0000	0.0000	0.5000	0.1667
1.6667	0.3333	96.2633	0.0000	0.0000	0.0000	0.0000	0.2000	0.6000	0.0000	0.0000	0.4000	0.0000
1.2500	0.1250	77.9300	0.0000	0.0000	0.0000	0.4000	0.4000	0.6000	0.0000	0.0000	0.6000	0.2000
1.7500	0.3750	66.0867	0.0000	0.0000	0.0000	0.0000	0.2500	0.7500	0.0000	0.0000	0.7500	0.0000
1.3333	0.1667	66.3150	0.0000	0.0000	0.0000	0.2500	0.2500	0.5000	0.0000	0.0000	0.5000	0.2500
2.0000	0.5000	69.8900	0.0000	0.0000	0.0000	0.3333	0.5000	0.8333	0.0000	0.0000	0.5000	0.1667
3.0000	1.0000	83.4100	0.0000	0.0000	0.0000	0.0000	0.6667	0.6667	0.0000	0.0000	0.3333	0.0000
2.0000	0.5000	79.8900	0.0000	0.0000	0.0000	0.1429	0.2857	0.7143	0.0000	0.0000	0.7143	0.1429
2.0000	0.5000	79.8900	0.0000	0.0000	0.0000	0.1667	0.1667	0.8333	0.0000	0.0000	0.8333	0.1667
1.5000	0.2500	51.8000	0.0000	0.0000	0.0000	0.0000	0.4000	0.6000	0.0000	0.0000	0.6000	0.0000
2.0000	0.5000	59.3650	0.0000	0.0000	0.0000	0.0000	0.6667	0.6667	0.0000	0.0000	0.6667	0.0000
2.0000	0.5000	83.8433	0.0000	0.0000	0.0000	0.2000	0.4000	0.6000	0.0000	0.0000	0.6000	0.2000
1.7500	0.3750	64.1475	0.0000	0.0000	0.0000	0.4000	0.6000	0.8000	0.0000	0.0000	0.6000	0.2000
1.0000	0.0000	47.3850	0.0000	0.0000	0.0000	0.5000	0.5000	1.0000	0.0000	0.0000	0.5000	0.0000
2.0000	0.5000	73.1200	0.3846	0.0000	0.0000	0.6154	0.4615	0.9231	0.0000	0.0769	0.8462	0.0769
1.9333	0.4667	73.8690	0.1875	0.0000	0.0000	0.8125	0.7500	0.9375	0.0000	0.1250	0.9375	0.1250
2.0625	0.5313	72.0753	0.1765	0.0000	0.0000	0.6471	0.6471	0.8824	0.0000	0.1176	0.8824	0.0588
2.1250	0.5625	70.0681	0.2353	0.0000	0.0000	0.5882	0.5294	0.9412	0.0000	0.1176	0.8824	0.1176
2.0000	0.5000	69.8105	0.2083	0.0000	0.0000	0.5417	0.4583	0.9167	0.0833	0.1667	0.8750	0.1667
2.0000	0.5000	69.4061	0.1923	0.0000	0.0000	0.5769	0.4231	0.8846	0.0769	0.1538	0.8462	0.1538
1.9000	0.4500	63.9890	0.2647	0.0000	0.0000	0.6176	0.3529	0.8824	0.0882	0.2647	0.7941	0.1765
2.0000	0.5000	71.6088	0.2414	0.0000	0.0000	0.6207	0.4483	0.8966	0.0345	0.2414	0.7586	0.1379
2.1053	0.5526	68.5553	0.2857	0.0000	0.0000	0.6190	0.4762	0.9048	0.0000	0.0952	0.7619	0.1429
2.0556	0.5278	68.8068	0.2273	0.0000	0.0455	0.5909	0.4091	0.8636	0.0455	0.1364	0.8182	0.1364

2.2353	0.6176	73.1194	0.2353	0.0000	0.0000	0.5882	0.5882	1.0000	0.0000	0.0588	0.8235	0.1765
2.1765	0.5882	68.6119	0.2000	0.0000	0.0000	0.5000	0.5000	0.8000	0.0500	0.1500	0.7500	0.2000
2.2143	0.6071	69.8114	0.1765	0.0000	0.0000	0.4706	0.5294	0.8235	0.0000	0.0588	0.7647	0.1765
1.9545	0.4773	69.4105	0.2000	0.0000	0.0000	0.5600	0.3600	0.8400	0.0800	0.2000	0.8000	0.2000
2.0556	0.5278	75.0306	0.2381	0.0000	0.0000	0.6190	0.4762	0.8571	0.0000	0.0952	0.8095	0.1429
2.1111	0.5556	72.8983	0.2000	0.0000	0.0000	0.4500	0.4500	0.9000	0.0000	0.0500	0.8000	0.1500
2.1053	0.5526	68.5016	0.2000	0.0000	0.0500	0.5000	0.4500	0.9500	0.1000	0.1500	0.9000	0.2500
2.2143	0.6071	70.0343	0.2778	0.0000	0.0000	0.5000	0.5000	0.8333	0.0556	0.1111	0.6667	0.1111
2.1429	0.5714	68.8040	0.2941	0.0000	0.0000	0.5882	0.4118	0.8824	0.0000	0.0588	0.8235	0.1765
2.1579	0.5789	75.8547	0.2727	0.0000	0.0000	0.6818	0.5455	0.8636	0.0000	0.1364	0.8182	0.0909
2.1333	0.5667	75.0138	0.2353	0.0000	0.0000	0.4706	0.4706	0.8235	0.0000	0.0588	0.7647	0.2353
1.9474	0.4737	74.6662	0.2381	0.0000	0.0000	0.4762	0.4762	0.9048	0.0000	0.0476	0.8095	0.2381
2.0909	0.5455	72.4860	0.1667	0.0000	0.0000	0.5000	0.4167	0.8750	0.0417	0.1667	0.7917	0.2083
1.9375	0.4688	68.3563	0.1765	0.0000	0.0000	0.5882	0.5294	0.8824	0.0588	0.1176	0.8824	0.2353
2.2222	0.6111	81.6200	0.1818	0.0000	0.0000	0.4545	0.5455	0.6364	0.0000	0.0000	0.7273	0.0909
2.1429	0.5714	74.3215	0.1875	0.0000	0.0000	0.5625	0.6250	0.8750	0.0000	0.0625	0.8125	0.2500
1.9444	0.4722	71.3750	0.3000	0.0000	0.0000	0.5000	0.4000	0.9000	0.0000	0.0500	0.8000	0.2000
2.0625	0.5313	72.1843	0.3158	0.0000	0.0000	0.4737	0.4211	0.7895	0.0000	0.0526	0.7368	0.2105
2.0909	0.5455	83.5300	0.3333	0.0000	0.0000	0.5833	0.5000	0.7500	0.0000	0.0000	0.7500	0.2500
2.0714	0.5357	82.0900	0.3333	0.0000	0.0000	0.5333	0.5333	0.8000	0.0667	0.0667	0.7333	0.2000
2.0556	0.5278	75.9941	0.3158	0.0000	0.0000	0.5263	0.4737	0.9474	0.0526	0.0526	0.7895	0.2105
2.1250	0.5625	69.4875	0.2778	0.0000	0.0000	0.5000	0.3889	0.8889	0.0000	0.1111	0.7222	0.1111
2.1000	0.5500	73.9381	0.2273	0.0000	0.0000	0.6818	0.5000	0.9545	0.0455	0.1364	0.9091	0.1818
2.0000	0.5000	74.5959	0.2857	0.0000	0.0000	0.5238	0.4286	0.8571	0.0476	0.0476	0.7143	0.2381
2.0588	0.5294	76.6053	0.1500	0.0000	0.0000	0.5500	0.5500	0.8500	0.0000	0.1000	0.7500	0.2000
2.0000	0.5000	74.7028	0.2381	0.0000	0.0000	0.5238	0.5238	0.8571	0.0000	0.0476	0.7143	0.2857
1.9333	0.4667	73.5762	0.2222	0.0000	0.0000	0.5000	0.5000	0.8333	0.0000	0.0000	0.7222	0.2778
2.1765	0.5882	71.1224	0.1667	0.0000	0.0000	0.5556	0.6111	0.9444	0.0556	0.1111	0.8333	0.2222
1.9375	0.4688	69.2520	0.2778	0.0000	0.0000	0.5556	0.4444	0.8889	0.0000	0.0556	0.7778	0.2222
1.9231	0.4615	76.0571	0.1875	0.0000	0.0000	0.5000	0.5000	0.8750	0.0000	0.0625	0.6875	0.1875
2.0000	0.5000	76.3255	0.2143	0.0000	0.0000	0.5000	0.4286	0.8571	0.0000	0.0714	0.7143	0.2143
1.9412	0.4706	65.6676	0.2105	0.0000	0.0000	0.6316	0.4211	0.8947	0.0526	0.1579	0.8421	0.2105
1.9474	0.4737	68.0747	0.2381	0.0000	0.0000	0.6667	0.5238	0.9048	0.0000	0.0476	0.8095	0.1905
1.9474	0.4737	68.5937	0.1905	0.0000	0.0000	0.6190	0.3810	0.9048	0.0000	0.1429	0.8095	0.1905
2.0667	0.5333	66.2073	0.2353	0.0000	0.0000	0.7059	0.5294	0.8824	0.0588	0.1176	0.8824	0.1176
1.7778	0.3889	69.6235	0.2105	0.0000	0.0000	0.6316	0.5263	0.9474	0.0000	0.1053	0.8947	0.2632
1.9500	0.4750	64.6740	0.2857	0.0000	0.0000	0.8095	0.4286	0.9524	0.0476	0.1905	0.8571	0.1429
2.0000	0.5000	71.6187	0.2353	0.0000	0.0000	0.5294	0.5294	0.8824	0.0588	0.0588	0.7647	0.2353
2.0000	0.5000	68.9940	0.2105	0.0000	0.0000	0.5263	0.4737	0.8421	0.0000	0.0526	0.7368	0.2105
2.1250	0.5625	63.2853	0.2222	0.0000	0.0000	0.6111	0.5000	0.8333	0.0556	0.1667	0.7778	0.1667
2.0625	0.5313	73.6944	0.2222	0.0000	0.0000	0.5000	0.5000	0.8889	0.0000	0.0556	0.7778	0.2222
2.0000	0.5000	68.0152	0.2813	0.0000	0.0313	0.6563	0.4063	0.9063	0.0313	0.1563	0.8125	0.1875
2.0556	0.5278	66.7161	0.1579	0.0000	0.0526	0.6316	0.5263	0.9474	0.1053	0.2105	0.8421	0.1579
2.0000	0.5000	68.6518	0.1667	0.0000	0.0000	0.6667	0.5000	0.9444	0.0556	0.2222	0.8333	0.1111
2.0909	0.5455	66.0500	0.1667	0.0000	0.0417	0.6250	0.5417	0.9167	0.0833	0.1667	0.8750	0.1250
1.8333	0.4167	67.4258	0.3571	0.0000	0.0000	0.6429	0.5000	0.8571	0.0000	0.0000	0.7857	0.2143
1.9000	0.4500	76.7680	0.2727	0.0000	0.0000	0.7273	0.7273	0.8182	0.0000	0.0000	0.8182	0.3636
2.0667	0.5333	65.7480	0.2500	0.0000	0.0000	0.5000	0.5625	0.9375	0.0000	0.0625	0.8125	0.1875
2.1000	0.5500	65.0140	0.3000	0.0000	0.0000	0.5000	0.6000	1.0000	0.0000	0.1000	0.8000	0.1000
1.9231	0.4615	67.9667	0.4615	0.0000	0.0000	0.5385	0.3846	1.0000	0.0000	0.0000	0.8462	0.0769
1.7778	0.3889	62.2711	0.2222	0.0000	0.0000	0.4444	0.3333	1.0000	0.0000	0.0000	1.0000	0.1111
1.7333	0.3667	62.1607	0.2353	0.0000	0.0000	0.4118	0.4118	0.8824	0.0000	0.0000	0.8235	0.1176
1.8125	0.4063	65.2040	0.3750	0.0000	0.0000	0.5625	0.3750	1.0000	0.0000	0.0000	0.8750	0.2500
1.9091	0.4545	70.2791	0.2727	0.0000	0.0000	0.4545	0.4545	1.0000	0.0000	0.0000	0.9091	0.2727
2.0000	0.5000	66.2189	0.4000	0.0000	0.0000	0.4000	0.4000	0.9000	0.0000	0.0000	0.9000	0.1000
2.0909	0.5455	72.2980	0.3636	0.0000	0.0000	0.4545	0.4545	0.9091	0.0000	0.0909	0.9091	0.0000
2.0000	0.5000	71.6163	0.3889	0.0000	0.0000	0.3889	0.4444	0.8889	0.0556	0.1111	0.8333	0.1111
1.6667	0.3333	63.1533	0.2308	0.0000	0.0000	0.3846	0.3077	0.9231	0.0000	0.0769	0.9231	0.2308
1.9375	0.4688	66.7813	0.2941	0.0000	0.0000	0.5882	0.3529	0.9412	0.0000	0.0588	0.9412	0.1765
1.8889	0.4444	64.0633	0.2222	0.0000	0.0000	0.5556	0.5000	1.0000	0.0000	0.0556	0.8889	0.1667
2.0000	0.5000	65.0607	0.2000	0.0000	0.0000	0.4000	0.4000	1.0000	0.0000	0.0667	0.9333	0.0000
1.9167	0.4583	64.9392	0.1667	0.0000	0.0000	0.5000	0.4167	1.0000	0.0000	0.0833	0.9167	0.0000
1.8182	0.4091	66.0073	0.2727	0.0000	0.0000	0.4545	0.3636	1.0000	0.0000	0.0000	0.9091	0.0909
1.8182	0.4091	75.5264	0.3333	0.0000	0.0000	0.3333	0.3333	0.9167	0.1667	0.0000	0.8333	0.0833
2.2857	0.6429	88.2964	0.2000	0.0000	0.0000	0.5000	0.7000	0.7000	0.0000	0.0000	0.6000	0.1000
2.1429	0.5714	80.1917	0.1250	0.0000	0.0000	0.3750	0.5000	0.6250	0.0000	0.0000	0.7500	0.1250
2.1250	0.5625	87.5438	0.3333	0.0000	0.0000	0.5556	0.5556	0.7778	0.0000	0.0000	0.7778	0.1111
2.0000	0.5000	81.1289	0.3333	0.0000	0.0000	0.5556	0.5556	1.0000	0.0000	0.0000	0.8889	0.2222
2.0000	0.5000	82.9650	0.2727	0.0000	0.0000	0.5455	0.5455	0.7273	0.0000	0.0000	0.7273	0.0909
2.0000	0.5000	85.8785	0.2727	0.0000	0.0000	0.6364	0.6364	0.8182	0.0000	0.0000	0.8182	0.1818
2.0909	0.5455	83.5680	0.2500	0.0000	0.0000	0.5000	0.5833	0.7500	0.0000	0.0000	0.7500	0.2500
2.0000	0.5000	85.9394	0.3333	0.0000	0.0000	0.6667	0.6667	0.8889	0.0000	0.0000	0.8889	0.1111
2.0000	0.5000	76.7356	0.5000	0.0000	0.0000	0.6000	0.5000	0.9000	0.0000	0.0000	0.9000	0.2000
2.1000	0.5500	87.2978	0.4000	0.0000	0.0000	0.7000	0.6000	0.9000	0.0000	0.0000	0.8000	0.2000
2.0000	0.5000	86.1550	0.3000	0.0000	0.0000	0.6000	0.6000	0.8000	0.0000	0.0000	0.8000	0.2000
1.8750	0.4375	81.5081	0.2222	0.0000	0.0000	0.5556	0.5556	0.7778	0.0000	0.0000	0.7778	0.0000
1.9167	0.4583	86.5432	0.2308	0.0000	0.0000	0.5385	0.5385	0.7692	0.0000	0.0000	0.8462	0.3077

1.8571	0.4286	69.9800	0.2000	0.0000	0.0000	0.4000	0.4000	0.9333	0.0000	0.0667	0.8000	0.0667
1.8889	0.4444	83.3233	0.2222	0.0000	0.0000	0.5556	0.4444	0.8889	0.0000	0.0000	0.8889	0.3333
2.1111	0.5556	87.2978	0.3333	0.0000	0.0000	0.6667	0.6667	0.8889	0.0000	0.0000	0.8889	0.2222
2.0000	0.5000	81.5035	0.1818	0.0000	0.0000	0.5455	0.6364	0.8182	0.0000	0.0000	0.7273	0.1818
2.2000	0.6000	83.6680	0.3000	0.0000	0.0000	0.5000	0.6000	1.0000	0.0000	0.0000	0.8000	0.3000
2.0000	0.5000	87.8870	0.2727	0.0000	0.0000	0.5455	0.5455	0.8182	0.0000	0.0000	0.9091	0.3636
2.1250	0.5625	86.6863	0.3750	0.0000	0.0000	0.6250	0.6250	1.0000	0.0000	0.0000	0.8750	0.2500
2.2000	0.6000	83.5680	0.3000	0.0000	0.0000	0.6000	0.7000	0.9000	0.0000	0.0000	0.8000	0.2000
2.2222	0.6111	82.6100	0.3333	0.0000	0.0000	0.5556	0.6667	1.0000	0.0000	0.0000	0.7778	0.2222
2.0000	0.5000	77.0938	0.3571	0.0000	0.0000	0.5714	0.4286	0.9286	0.0000	0.0000	0.7857	0.2857
2.0909	0.5455	82.8800	0.2727	0.0000	0.0000	0.5455	0.5455	1.0000	0.0000	0.0000	0.8182	0.3636
1.9286	0.4643	85.1471	0.3571	0.0000	0.0000	0.5714	0.5714	0.8571	0.0714	0.0000	0.7143	0.2857
2.1667	0.5833	85.2183	0.3333	0.0000	0.0000	0.5000	0.6667	0.9167	0.0833	0.0000	0.7500	0.2500
1.8125	0.4063	72.7733	0.2500	0.0000	0.0000	0.3750	0.4375	1.0000	0.0625	0.0000	0.8125	0.2500
1.9286	0.4643	77.3569	0.2857	0.0000	0.0000	0.5000	0.3571	1.0000	0.0714	0.0000	0.7857	0.2857
2.0769	0.5385	77.6400	0.3571	0.0000	0.0000	0.5000	0.5000	1.0000	0.0000	0.0000	0.7143	0.2143
1.7857	0.3929	81.5754	0.2857	0.0000	0.0000	0.5000	0.6429	0.8571	0.0714	0.0000	0.7857	0.2857
2.0000	0.5000	81.1869	0.3125	0.0000	0.0000	0.4375	0.4375	0.8750	0.0625	0.0000	0.6875	0.3125
1.8000	0.4000	67.8983	0.2381	0.0000	0.0000	0.2857	0.3333	0.9524	0.0952	0.0476	0.7143	0.1429
1.7692	0.3846	68.1183	0.3333	0.0000	0.0000	0.5333	0.3333	0.8667	0.0000	0.0000	0.7333	0.2000
2.0000	0.5000	79.7721	0.3125	0.0000	0.0000	0.3750	0.5000	0.9375	0.0625	0.0000	0.6875	0.1875
1.8667	0.4333	78.5407	0.3125	0.0000	0.0000	0.3750	0.5625	0.9375	0.1250	0.0000	0.7500	0.2500
1.6429	0.3214	76.6300	0.2143	0.0000	0.0000	0.5000	0.4286	1.0000	0.0000	0.0714	0.9286	0.3571
1.3636	0.1818	69.1000	0.3333	0.0000	0.0000	0.6667	0.3333	0.9167	0.0000	0.0833	0.8333	0.2500
1.5294	0.2647	67.0800	0.3889	0.0000	0.0000	0.6667	0.2778	0.8889	0.0000	0.1111	0.8889	0.2222
1.6000	0.3000	67.7010	0.3000	0.0000	0.0000	0.8000	0.5000	1.0000	0.0000	0.2000	0.9000	0.1000
1.5333	0.2667	71.8000	0.4000	0.0000	0.0000	0.7333	0.3333	1.0000	0.0000	0.2000	0.9333	0.2000
1.5294	0.2647	71.5475	0.3333	0.0000	0.0000	0.7222	0.3333	0.9444	0.0000	0.2222	0.8333	0.2222
1.7500	0.3750	67.1782	0.3333	0.0000	0.0000	0.8333	0.5000	1.0000	0.0000	0.3333	0.9167	0.0000
1.7273	0.3636	73.2310	0.4167	0.0000	0.0000	0.7500	0.4167	0.9167	0.0000	0.2500	0.8333	0.0833
1.5294	0.2647	67.9913	0.3333	0.0000	0.0000	0.6667	0.3889	0.8889	0.0000	0.1667	0.8889	0.2222
1.6250	0.3125	68.6553	0.3125	0.0000	0.0000	0.8750	0.4375	1.0000	0.0000	0.2500	0.8750	0.1875
1.6667	0.3333	65.9592	0.1875	0.0000	0.0000	0.7500	0.5000	0.8750	0.0000	0.2500	0.8750	0.1250
1.7778	0.3889	63.1113	0.3000	0.0000	0.0000	0.9000	0.4000	0.9000	0.0000	0.2000	0.8000	0.1000
1.6667	0.3333	72.5354	0.2500	0.0000	0.0000	0.8750	0.5000	0.8750	0.0625	0.3750	0.9375	0.1250
1.9286	0.4643	70.7385	0.2857	0.0000	0.0000	0.9286	0.5714	1.0000	0.0714	0.4286	0.9286	0.0714
1.9231	0.4615	66.7558	0.3571	0.0000	0.0000	0.7857	0.5000	0.9286	0.0714	0.4286	0.8571	0.0000
1.6364	0.3182	69.3540	0.2500	0.0000	0.0000	0.8333	0.5833	0.9167	0.0833	0.4167	0.9167	0.0833
1.8333	0.4167	64.8745	0.2308	0.0000	0.0000	0.9231	0.6154	0.9231	0.0769	0.5385	0.8462	0.0000
1.5000	0.2500	71.2853	0.1667	0.0000	0.0000	0.2778	0.3889	0.8889	0.0000	0.0556	0.6111	0.1111
1.6000	0.3000	55.3050	0.0909	0.0000	0.0000	0.4545	0.0909	0.9091	0.0000	0.0000	0.7273	0.0909
1.3846	0.1923	67.1375	0.2143	0.0000	0.0000	0.5000	0.3571	0.9286	0.0000	0.0000	0.7143	0.2143
1.4706	0.2353	60.8738	0.2778	0.0000	0.0000	0.3889	0.2222	0.9444	0.0000	0.0000	0.7222	0.1111
1.4375	0.2188	64.4147	0.1765	0.0000	0.0000	0.5294	0.3529	0.9412	0.0000	0.0588	0.7059	0.1765
1.5625	0.2813	70.3047	0.2353	0.0000	0.0000	0.4118	0.4118	0.9412	0.0000	0.0588	0.7059	0.1176
1.3077	0.1538	69.1075	0.2143	0.0000	0.0000	0.4286	0.3571	0.9286	0.0000	0.0714	0.7143	0.1429
1.3125	0.1563	70.7160	0.1765	0.0000	0.0000	0.4118	0.3529	0.9412	0.0000	0.0000	0.7059	0.1176
1.3636	0.1818	67.1470	0.2500	0.0000	0.0000	0.4167	0.4167	0.9167	0.0000	0.0000	0.6667	0.1667
1.3571	0.1786	68.0654	0.2000	0.0000	0.0000	0.4667	0.3333	0.9333	0.0000	0.0000	0.7333	0.2000
1.4667	0.2333	62.6793	0.2500	0.0000	0.0000	0.4375	0.3125	0.9375	0.0000	0.0625	0.6875	0.1250
1.5882	0.2941	59.5618	0.1667	0.0000	0.0000	0.5556	0.2778	0.9444	0.0000	0.0556	0.7778	0.1111
1.8125	0.4063	78.7037	0.2941	0.0000	0.0000	0.7059	0.2941	0.9412	0.0588	0.3529	0.8824	0.1176
2.0000	0.5000	65.8210	0.2727	0.0000	0.0000	0.8182	0.3636	1.0000	0.0909	0.4545	0.8182	0.0000
1.6429	0.3214	71.6662	0.2857	0.0000	0.0000	0.7857	0.3571	1.0000	0.0714	0.2857	0.9286	0.1429
1.6364	0.3182	60.0220	0.1818	0.0000	0.0000	0.7273	0.3636	1.0000	0.0909	0.3636	1.0000	0.0909
1.7692	0.3846	71.9958	0.2308	0.0000	0.0000	0.8462	0.4615	1.0000	0.0769	0.4615	0.9231	0.0769
1.6667	0.3333	68.1464	0.1667	0.0000	0.0000	0.7500	0.5000	1.0000	0.0833	0.3333	0.8333	0.0000
2.0909	0.5455	68.8045	0.1818	0.0000	0.0000	0.9091	0.6364	1.0000	0.0909	0.5455	0.9091	0.0000
1.9091	0.4545	74.7980	0.2727	0.0000	0.0000	0.9091	0.7273	1.0000	0.0909	0.5455	1.0000	0.0000
2.1250	0.5625	70.9786	0.2222	0.0000	0.0000	0.7778	0.5556	0.8889	0.1111	0.5556	0.8889	0.0000
1.8235	0.4118	66.1844	0.2632	0.0000	0.0000	0.7895	0.4211	0.8947	0.0526	0.4211	0.8421	0.2105
1.7857	0.3929	69.1523	0.2143	0.0000	0.0000	0.9286	0.6429	1.0000	0.0714	0.5000	0.9286	0.0714
1.7059	0.3529	65.3607	0.2778	0.0000	0.0000	0.7222	0.3889	0.8889	0.0556	0.3333	0.8889	0.1667
1.7333	0.3667	69.7018	0.2500	0.0000	0.0000	0.6875	0.4375	0.9375	0.0000	0.3750	0.8125	0.0625
1.7778	0.3889	65.9638	0.2632	0.0000	0.0526	0.6316	0.3684	0.8947	0.0000	0.2632	0.8421	0.2632
1.6429	0.3214	65.2992	0.2000	0.0000	0.0000	0.7333	0.4667	0.9333	0.0000	0.3333	0.8667	0.0667
1.6429	0.3214	65.9055	0.2000	0.0000	0.0000	0.7333	0.4667	0.8000	0.0667	0.4000	0.8000	0.1333
1.7778	0.3889	67.3688	0.1667	0.0000	0.0000	0.8333	0.6111	0.9444	0.1111	0.5000	0.9444	0.1111
1.7333	0.3667	73.5669	0.1875	0.0000	0.0000	0.6875	0.5000	0.8750	0.0625	0.4375	0.8125	0.1250
1.6429	0.3214	70.8475	0.1333	0.0000	0.0000	0.8000	0.5333	0.8667	0.0667	0.4667	0.8667	0.0667
1.8667	0.4333	68.2915	0.2500	0.0000	0.0000	0.7500	0.5000	0.8750	0.0625	0.4375	0.9375	0.0625
1.8333	0.4167	65.2291	0.2308	0.0000	0.0000	0.8462	0.5385	0.9231	0.0769	0.5385	0.8462	0.0000
1.6842	0.3421	61.7435	0.2000	0.0000	0.0000	0.7000	0.4000	0.9000	0.0500	0.4000	0.8500	0.1000
2.0000	0.5000	60.4233	0.2500	0.0000	0.0000	0.8750	0.5000	0.8750	0.1250	0.6250	0.8750	0.0000
1.7778	0.3889	64.2088	0.2222	0.0000	0.0000	1.0000	0.6667	1.0000	0.1111	0.6667	1.0000	0.0000
1.9000	0.4500	63.1911	0.1818	0.0000	0.0909	0.9091	0.5455	0.9091	0.0909	0.6364	0.9091	0.0909
1.8750	0.4375	64.5143	0.2500	0.0000	0.0000	1.0000	0.6250	1.0000	0.1250	0.6250	1.0000	0.0000

1.7273	0.3636	66.8850	0.1667	0.0000	0.0000	0.9167	0.6667	0.9167	0.0833	0.5833	0.9167	0.0000
NA	NA	NA	0.0000	0.0000	0.0000	0.0000	0.0000	0.0000	0.0000	0.0000	0.0000	0.0000
1.6667	0.3333	59.9600	0.2857	0.0000	0.0000	0.1429	0.1429	0.8571	0.0000	0.0000	0.8571	0.1429
2.1111	0.5556	51.9900	0.2727	0.0000	0.0000	0.2727	0.3636	0.8182	0.0000	0.0909	0.8182	0.1818
1.6667	0.3333	59.7514	0.1250	0.0000	0.0000	0.2500	0.2500	0.8750	0.0000	0.0000	0.7500	0.0000
2.4000	0.7000	84.8280	0.6000	0.0000	0.0000	0.4000	0.6000	1.0000	0.2000	0.2000	0.8000	0.0000
1.8462	0.4231	73.2492	0.2857	0.0000	0.0000	0.4286	0.5000	0.9286	0.0714	0.0000	0.7143	0.1429
1.6250	0.3125	54.5133	0.2222	0.0000	0.0000	0.3333	0.1111	1.0000	0.0000	0.0000	0.7778	0.0000
2.0000	0.5000	65.4520	0.3636	0.0000	0.0000	0.2727	0.4545	0.9091	0.0909	0.0909	0.7273	0.1818
1.7857	0.3929	59.3664	0.1250	0.0000	0.0000	0.5625	0.4375	0.8750	0.0000	0.1875	0.6875	0.0625
1.9000	0.4500	52.7273	0.0833	0.0000	0.0000	0.1667	0.2500	0.9167	0.0000	0.0833	0.5833	0.0000
2.0000	0.5000	83.0450	0.0000	0.0000	0.0000	0.5000	1.0000	1.0000	0.0000	0.0000	1.0000	0.0000
1.8000	0.4000	69.2450	0.1667	0.0000	0.0000	0.3333	0.5000	1.0000	0.1667	0.0000	0.5000	0.0000
1.9412	0.4706	65.2941	0.1667	0.0000	0.0000	0.7222	0.4444	0.9444	0.1111	0.1667	0.7778	0.1111
2.0000	0.5000	63.2100	0.1667	0.0000	0.0000	0.6667	0.3889	0.9444	0.0556	0.2222	0.7778	0.0556
1.9000	0.4500	60.8960	0.2000	0.0000	0.0000	0.7500	0.4000	1.0000	0.0500	0.2000	0.8000	0.0500
1.8636	0.4318	59.0395	0.1667	0.0000	0.0000	0.6667	0.3333	0.9167	0.0417	0.2500	0.7083	0.0833
2.0000	0.5000	58.7100	0.2222	0.0000	0.0000	0.6667	0.3333	0.9444	0.1111	0.2222	0.7778	0.0556
2.0588	0.5294	61.3350	0.1765	0.0000	0.0000	0.6471	0.2941	1.0000	0.0588	0.2353	0.8235	0.0588
1.9524	0.4762	62.8890	0.0952	0.0000	0.0476	0.7143	0.4286	1.0000	0.0952	0.1905	0.8095	0.0952
1.8947	0.4474	62.3400	0.1000	0.0000	0.0000	0.7500	0.3500	0.9500	0.0500	0.2500	0.8500	0.1000
2.0625	0.5313	67.2829	0.1250	0.0000	0.0000	0.8750	0.6250	0.9375	0.0000	0.3125	0.9375	0.1875
2.1538	0.5769	68.1050	0.2143	0.0000	0.0000	0.7857	0.5714	0.9286	0.0000	0.2857	0.7857	0.0000
2.0000	0.5000	61.1838	0.2500	0.0000	0.0000	0.7500	0.6250	1.0000	0.0000	0.1250	0.8750	0.1250
1.8000	0.4000	60.5180	0.3636	0.0000	0.0000	0.5455	0.3636	0.9091	0.0000	0.0000	0.7273	0.0909
1.8947	0.4474	66.6994	0.3000	0.0000	0.0000	0.8500	0.4000	0.9500	0.0500	0.3000	0.8500	0.0500
1.7600	0.3800	66.9123	0.2222	0.0000	0.0000	0.7407	0.3333	0.8889	0.0370	0.3333	0.8148	0.0741
2.0714	0.5357	65.7071	0.2667	0.0000	0.0000	0.8667	0.4000	0.9333	0.0000	0.2000	0.8000	0.0667
1.6522	0.3261	62.5300	0.1250	0.0000	0.0000	0.7917	0.4167	0.9167	0.0000	0.2500	0.8750	0.1667
1.8333	0.4167	64.5988	0.0526	0.0000	0.0000	0.6842	0.2632	0.9474	0.0526	0.2632	0.7368	0.1579
1.7083	0.3542	62.0619	0.1538	0.0000	0.0000	0.7308	0.2692	0.9231	0.0000	0.2308	0.7692	0.1154
1.9000	0.4500	62.1000	0.0000	0.0000	0.0000	0.7500	0.2500	0.8333	0.0000	0.1667	0.6667	0.1667
1.7895	0.3947	63.1506	0.1500	0.0000	0.1000	0.6500	0.3000	0.9500	0.1000	0.3000	0.8000	0.1500

27 mammals	28 diversity	28 diversity.s	29 gr_CY	29 gr_FO	29 gr_PO	29 gr_PT	29 gr_SPH	29 gr_BM	30 pls_0	30 pls_1	31 pha_1	32 bry_1
0.0000	2.4783	0.2957	0.2800	0.5200	0.0400	0.0800	0.0000	0.1765	1.0000	0.0000	0.0588	0.2059
0.0000	2.5294	0.3059	0.3889	0.5000	0.0556	0.0556	0.0000	0.1667	1.0000	0.0000	0.0833	0.1667
0.0000	2.7273	0.3455	0.1538	0.6923	0.1538	0.0000	0.0000	0.1765	1.0000	0.0000	0.0000	0.2353
0.0714	3.0714	0.4143	0.2857	0.5000	0.1429	0.0000	0.0000	0.2500	1.0000	0.0000	0.0000	0.3000
0.0476	2.5000	0.3000	0.2381	0.4762	0.1429	0.0476	0.0323	0.2581	1.0000	0.0000	0.0000	0.3226
0.0000	2.6364	0.3273	0.2500	0.5833	0.0833	0.0833	0.0000	0.2500	1.0000	0.0000	0.0000	0.2500
0.0588	2.8750	0.3750	0.3529	0.4706	0.0588	0.0588	0.0400	0.2000	1.0000	0.0000	0.0800	0.2400
0.0909	2.9000	0.3800	0.2273	0.5455	0.0455	0.0909	0.0000	0.0800	1.0000	0.0000	0.0400	0.0800
0.1176	2.8235	0.3647	0.3529	0.5294	0.1176	0.0000	0.0000	0.2083	1.0000	0.0000	0.0833	0.2083
0.0000	2.5714	0.3143	0.2667	0.5333	0.0667	0.0667	0.0000	0.2381	1.0000	0.0000	0.0476	0.2381
0.0000	2.6923	0.3385	0.3571	0.3571	0.0714	0.0714	0.0000	0.1429	1.0000	0.0000	0.1429	0.1905
0.0500	2.0588	0.2118	0.4000	0.4500	0.0000	0.1000	0.0000	0.1200	0.9500	0.0500	0.0800	0.1200
0.0345	2.5000	0.3000	0.2069	0.5862	0.0690	0.0345	0.0244	0.1220	1.0000	0.0000	0.1220	0.1707
0.0741	3.0000	0.4000	0.2963	0.4815	0.1111	0.0000	0.0000	0.1842	0.9630	0.0000	0.0526	0.2368
0.0526	2.6875	0.3375	0.3158	0.4211	0.0526	0.0526	0.0000	0.2500	0.9474	0.0000	0.0938	0.3125
0.0000	2.8000	0.3600	0.2381	0.6667	0.0476	0.0476	0.0000	0.1200	1.0000	0.0000	0.0400	0.1200
0.0625	3.1000	0.4200	0.2500	0.6250	0.0938	0.0000	0.0426	0.1915	0.9688	0.0313	0.0851	0.2340
0.0345	2.6923	0.3385	0.3793	0.5172	0.0345	0.0345	0.0000	0.1212	0.9655	0.0000	0.0000	0.1212
0.0000	2.8824	0.3765	0.2500	0.5500	0.1000	0.0000	0.0000	0.2143	0.9500	0.0000	0.0714	0.2143
0.0000	2.8095	0.3619	0.1818	0.7273	0.0455	0.0455	0.0000	0.1786	1.0000	0.0000	0.0000	0.2143
0.0000	2.6500	0.3300	0.1429	0.7619	0.0476	0.0476	0.0000	0.1538	1.0000	0.0000	0.0000	0.1923
0.0909	2.9048	0.3810	0.3182	0.5455	0.0455	0.0000	0.0000	0.1724	1.0000	0.0000	0.0690	0.1724
0.0952	2.8333	0.3667	0.2857	0.4762	0.0952	0.0476	0.0000	0.1379	0.9524	0.0000	0.1034	0.1724
0.0588	2.9333	0.3867	0.2941	0.5000	0.0588	0.0588	0.0000	0.0930	0.9706	0.0294	0.1163	0.0930
0.0000	3.1429	0.4286	0.2258	0.5806	0.0645	0.0645	0.0000	0.1628	0.9677	0.0323	0.0930	0.1860
0.0000	2.9500	0.3900	0.1364	0.5909	0.0909	0.0455	0.0000	0.1111	0.9545	0.0000	0.0741	0.1111
0.0741	2.6800	0.3360	0.1852	0.6296	0.0741	0.0741	0.0000	0.1714	1.0000	0.0000	0.0286	0.2000
0.0606	2.6452	0.3290	0.2121	0.6364	0.0606	0.0606	0.0000	0.1591	1.0000	0.0000	0.0682	0.1818
0.0968	2.8000	0.3600	0.1935	0.6452	0.0968	0.0323	0.0000	0.1250	1.0000	0.0000	0.0750	0.1500
0.0800	2.5417	0.3083	0.2000	0.6000	0.1200	0.0400	0.0000	0.1613	1.0000	0.0000	0.0000	0.1935
0.0000	2.6667	0.3333	0.2778	0.6667	0.0556	0.0000	0.0385	0.1923	1.0000	0.0000	0.0385	0.2692
0.0588	2.8710	0.3742	0.1765	0.6176	0.0294	0.0588	0.0435	0.1957	1.0000	0.0000	0.0217	0.2391
0.0741	2.6538	0.3308	0.1481	0.6296	0.1481	0.0370	0.0000	0.1714	1.0000	0.0000	0.0286	0.2000
0.0952	2.7619	0.3524	0.0952	0.7619	0.0952	0.0000	0.0000	0.1852	1.0000	0.0000	0.0370	0.1852
0.0370	2.7500	0.3500	0.2222	0.5185	0.1111	0.0370	0.0000	0.2195	0.9259	0.0000	0.0976	0.2439
0.0435	2.4545	0.2909	0.2609	0.5217	0.1739	0.0435	0.0000	0.1613	1.0000	0.0000	0.0645	0.1935
0.1000	2.6316	0.3263	0.1500	0.5000	0.2000	0.0500	0.0417	0.0833	1.0000	0.0000	0.0417	0.1250
0.0370	2.4615	0.2923	0.2222	0.4815	0.1481	0.0370	0.0333	0.0000	1.0000	0.0000	0.0667	0.0333
0.1000	2.5000	0.3000	0.1500	0.6000	0.1000	0.1000	0.0000	0.1250	1.0000	0.0000	0.0000	0.1667
0.0000	2.5385	0.3077	0.4074	0.3333	0.0741	0.0370	0.0455	0.1818	1.0000	0.0000	0.1591	0.2273
0.0714	2.6154	0.3231	0.1429	0.5714	0.0714	0.0714	0.1111	0.1481	1.0000	0.0000	0.2222	0.2593
0.0769	2.8333	0.3667	0.2308	0.5385	0.1538	0.0769	0.0000	0.2500	1.0000	0.0000	0.1000	0.2500
0.0000	2.4000	0.2800	0.2500	0.5417	0.0417	0.1250	0.0667	0.0000	1.0000	0.0000	0.1333	0.0667
0.0000	2.3571	0.2714	0.3333	0.4667	0.0667	0.0667	0.0909	0.0455	1.0000	0.0000	0.1818	0.1364
0.0000	2.6000	0.3200	0.3810	0.4286	0.0952	0.0476	0.0714	0.0357	1.0000	0.0000	0.1429	0.1071
0.0000	2.5500	0.3100	0.1905	0.7143	0.0476	0.0476	0.0000	0.1600	1.0000	0.0000	0.0000	0.1600
0.1000	2.8947	0.3789	0.2500	0.6500	0.0500	0.0500	0.0400	0.0400	0.9500	0.0500	0.1200	0.0800
0.0323	2.7500	0.3500	0.0645	0.7097	0.0645	0.0968	0.0000	0.2195	1.0000	0.0000	0.0000	0.2439
0.0000	2.4800	0.2960	0.2308	0.5385	0.0769	0.0385	0.0256	0.1538	1.0000	0.0000	0.1282	0.2051
0.0000	2.6667	0.3333	0.2632	0.5789	0.1053	0.0526	0.0000	0.1600	1.0000	0.0000	0.0800	0.1600
0.0789	2.7429	0.3486	0.1842	0.5000	0.1316	0.0789	0.0435	0.1087	1.0000	0.0000	0.0217	0.1522
0.0588	3.0667	0.4133	0.1765	0.5294	0.1765	0.1176	0.0000	0.2273	1.0000	0.0000	0.0000	0.2273
0.0833	2.6087	0.3217	0.2917	0.5833	0.0417	0.0417	0.0294	0.0882	0.9583	0.0417	0.1471	0.1471
0.0000	2.7333	0.3467	0.2941	0.5882	0.0588	0.0588	0.0000	0.1667	1.0000	0.0000	0.0833	0.2083
0.1034	2.9655	0.3931	0.1724	0.5517	0.1724	0.0000	0.0769	0.0769	1.0000	0.0000	0.1026	0.1538
0.0909	2.9091	0.3818	0.2424	0.6061	0.0909	0.0000	0.0476	0.0714	1.0000	0.0000	0.0952	0.1190
0.1034	3.0357	0.4071	0.1724	0.6207	0.1724	0.0345	0.0000	0.1143	1.0000	0.0000	0.0286	0.1429
0.1034	2.8571	0.3714	0.1379	0.6897	0.1379	0.0345	0.0000	0.1622	1.0000	0.0000	0.0270	0.1892
0.1852	3.3846	0.4769	0.1852	0.5926	0.1852	0.0370	0.0303	0.1212	1.0000	0.0000	0.0303	0.1515
0.0909	2.9000	0.3800	0.2273	0.4091	0.1818	0.0909	0.0000	0.2258	1.0000	0.0000	0.0323	0.2581
0.0000	3.0000	0.4000	0.0000	0.2500	0.7500	0.0000	0.0000	0.0000	1.0000	0.0000	0.0000	0.0000
0.0000	2.0000	0.2000	0.2500	0.5000	0.2500	0.0000	0.0000	0.0000	1.0000	0.0000	0.0000	0.0000
0.0000	2.5000	0.3000	0.3333	0.3333	0.3333	0.0000	0.0000	0.0000	1.0000	0.0000	0.0000	0.0000
0.0000	2.7500	0.3500	0.2500	0.5000	0.2500	0.0000	0.0000	0.0000	1.0000	0.0000	0.0000	0.0000
0.0000	2.0000	0.2000	0.3333	0.3333	0.3333	0.0000	0.0000	0.0000	1.0000	0.0000	0.0000	0.0000
0.0000	2.0000	0.2000	0.0000	0.6667	0.3333	0.0000	0.0000	0.0000	1.0000	0.0000	0.0000	0.0000
0.0000	2.0000	0.2000	0.0000	0.6667	0.3333	0.0000	0.0000	0.0000	1.0000	0.0000	0.0000	0.0000
0.0000	4.0000	0.6000	0.0000	0.5714	0.4286	0.0000	0.0000	0.0000	1.0000	0.0000	0.0000	0.0000
0.0714	3.7692	0.5538	0.0714	0.5714	0.3571	0.0000	0.0000	0.0000	1.0000	0.0000	0.0000	0.0000
0.1111	4.2222	0.6444	0.0000	0.3333	0.6667	0.0000	0.0000	0.0000	1.0000	0.0000	0.0000	0.0000
0.1667	4.1765	0.6353	0.0000	0.6111	0.3889	0.0000	0.0000	0.0000	0.7778	0.2222	0.0000	0.0000
0.0500	4.0000	0.6000	0.0500	0.6000	0.3500	0.0000	0.0000	0.0000	0.7500	0.2500	0.0000	0.0000
0.1667	3.5000	0.5000	0.0000	0.7500	0.2500	0.0000	0.0000	0.0000	0.8333	0.1667	0.0000	0.0000
0.0667	4.0714	0.6143	0.0000	0.6000	0.4000	0.0000	0.0000	0.0000	1.0000	0.0000	0.0000	0.0000
0.1111	2.2500	0.2500	0.1111	0.6667	0.1111	0.1111	0.0000	0.1000	1.0000	0.0000	0.0000	0.1000
0.0909	2.6000	0.3200	0.2727	0.4545	0.1818	0.0909	0.0000	0.1429	1.0000	0.0000	0.0714	0.1429

0.0833	2.6364	0.3273	0.2500	0.5000	0.1667	0.0833	0.0000	0.0714	1.0000	0.0000	0.0714	0.0714
0.0000	2.5000	0.3000	0.2000	0.4000	0.2000	0.2000	0.0000	0.1250	1.0000	0.0000	0.2500	0.1250
0.1176	3.1875	0.4375	0.1176	0.5294	0.2353	0.0588	0.0000	0.0952	0.9412	0.0588	0.0952	0.0952
0.1053	3.0556	0.4111	0.2105	0.5263	0.2105	0.0526	0.0000	0.0455	0.9474	0.0526	0.0455	0.0909
0.1111	2.5294	0.3059	0.3333	0.4444	0.1667	0.0556	0.0000	0.1667	0.9444	0.0556	0.0417	0.2083
0.2143	3.1538	0.4308	0.2143	0.5714	0.1429	0.0714	0.0000	0.0556	0.9286	0.0714	0.1111	0.1111
0.1000	3.1111	0.4222	0.1000	0.6000	0.2000	0.1000	0.0000	0.0714	0.9000	0.1000	0.2143	0.0714
0.0667	2.9286	0.3857	0.1333	0.5333	0.2667	0.0667	0.0000	0.1000	0.8667	0.1333	0.1000	0.1500
0.1111	2.8824	0.3765	0.2222	0.5556	0.1667	0.0556	0.0000	0.0870	0.9444	0.0556	0.0870	0.1304
0.1000	2.8947	0.3789	0.2000	0.6000	0.1500	0.0500	0.0000	0.0417	0.9500	0.0500	0.0833	0.0833
0.1818	2.9000	0.3800	0.1818	0.5455	0.1818	0.0909	0.0000	0.0714	0.9091	0.0909	0.1429	0.0714
0.0000	2.3750	0.2750	0.1111	0.6667	0.1111	0.1111	0.0000	0.0909	0.8889	0.1111	0.0909	0.0909
0.0000	2.7143	0.3429	0.1250	0.5000	0.2500	0.1250	0.0000	0.1000	1.0000	0.0000	0.1000	0.1000
0.0000	2.6000	0.3200	0.1818	0.5455	0.1818	0.0909	0.0000	0.0769	0.9091	0.0909	0.0769	0.0769
0.1250	3.0000	0.4000	0.1250	0.5000	0.2500	0.1250	0.0000	0.1667	0.8750	0.1250	0.1667	0.1667
0.0000	2.4444	0.2889	0.1000	0.6000	0.2000	0.1000	0.0000	0.0833	0.8000	0.2000	0.0833	0.0833
0.1250	3.0000	0.4000	0.1875	0.6250	0.1250	0.0625	0.0000	0.1000	0.9375	0.0625	0.0500	0.1500
0.1538	2.7500	0.3500	0.1538	0.6923	0.0769	0.0769	0.0000	0.1176	1.0000	0.0000	0.1176	0.1176
0.0000	2.8333	0.3667	0.1538	0.6154	0.1538	0.0769	0.0000	0.0667	0.9231	0.0769	0.0667	0.0667
0.0000	2.7778	0.3556	0.2000	0.5000	0.2000	0.1000	0.0000	0.0833	0.9000	0.1000	0.0833	0.0833
0.1200	3.3750	0.4750	0.0400	0.7600	0.1600	0.0400	0.0000	0.1333	0.8800	0.1200	0.0333	0.1333
0.0952	3.2500	0.4500	0.0476	0.7143	0.1905	0.0476	0.0000	0.0769	0.9048	0.0952	0.0769	0.1154
0.1053	3.5000	0.5000	0.0526	0.6842	0.2105	0.0526	0.0000	0.1667	0.9474	0.0526	0.0417	0.1667
0.1786	3.1481	0.4296	0.1071	0.7143	0.1429	0.0357	0.0000	0.0667	0.9286	0.0714	0.0000	0.0667
0.1538	2.8333	0.3667	0.3077	0.3846	0.2308	0.0769	0.0000	0.0588	0.9231	0.0769	0.1176	0.1176
0.1176	3.1250	0.4250	0.1176	0.7647	0.0588	0.0588	0.0000	0.1000	0.8824	0.1176	0.0500	0.1000
0.0952	3.3000	0.4600	0.1429	0.7143	0.0952	0.0476	0.0000	0.0417	0.9524	0.0476	0.0417	0.0833
0.1818	3.4762	0.4952	0.0909	0.6364	0.2273	0.0455	0.0000	0.0800	0.9091	0.0909	0.0000	0.1200
0.1667	3.1304	0.4261	0.1667	0.5833	0.2083	0.0417	0.0000	0.0370	0.9167	0.0833	0.0741	0.0370
0.0870	2.9545	0.3909	0.2174	0.5652	0.1739	0.0435	0.0000	0.0741	0.9565	0.0435	0.0741	0.0741
0.0000	2.5000	0.3000	0.4615	0.3846	0.0769	0.0769	0.0000	0.1667	0.9231	0.0769	0.1111	0.1667
0.1111	3.0000	0.4000	0.2222	0.6667	0.1111	0.0000	0.0000	0.2308	0.8889	0.1111	0.0769	0.2308
0.0556	3.0000	0.4000	0.2222	0.6111	0.1111	0.0556	0.0000	0.1364	0.9444	0.0556	0.0455	0.1364
0.0000	2.5625	0.3125	0.3529	0.5294	0.0588	0.0588	0.0000	0.1429	1.0000	0.0000	0.0476	0.1429
0.1000	2.8000	0.3600	0.4000	0.6000	0.0000	0.0000	0.0000	0.1538	0.9000	0.1000	0.0769	0.1538
0.1000	2.5556	0.3111	0.3000	0.6000	0.0000	0.1000	0.0000	0.1538	1.0000	0.0000	0.0769	0.1538
0.2727	2.8182	0.3636	0.2727	0.5455	0.1818	0.0000	0.0000	0.1875	1.0000	0.0000	0.1250	0.1875
0.1429	3.2308	0.4462	0.2857	0.5714	0.0714	0.0714	0.0000	0.1579	1.0000	0.0000	0.1053	0.1579
0.1765	3.4706	0.4941	0.1765	0.5882	0.2353	0.0000	0.0000	0.1905	1.0000	0.0000	0.0000	0.1905
0.2500	3.5500	0.5100	0.1500	0.5000	0.3000	0.0000	0.0000	0.1200	1.0000	0.0000	0.0800	0.1200
0.2500	3.3333	0.4667	0.1875	0.5000	0.2500	0.0625	0.0000	0.1500	1.0000	0.0000	0.0500	0.1500
0.1739	3.1364	0.4273	0.3043	0.5217	0.1739	0.0000	0.0000	0.1071	1.0000	0.0000	0.0357	0.1429
0.1538	3.4167	0.4833	0.2308	0.6154	0.0769	0.0769	0.0000	0.1579	1.0000	0.0000	0.1579	0.1579
0.2083	3.4783	0.4957	0.1250	0.6250	0.2083	0.0417	0.0000	0.1111	0.9167	0.0833	0.0000	0.1111
0.1500	3.5263	0.5053	0.1500	0.5500	0.2500	0.0500	0.0000	0.1600	1.0000	0.0000	0.0400	0.1600
0.1250	3.1333	0.4267	0.3125	0.4375	0.1875	0.0625	0.0000	0.2083	1.0000	0.0000	0.0833	0.2500
0.1250	2.8667	0.3733	0.2500	0.5625	0.1250	0.0625	0.0000	0.1429	0.9375	0.0625	0.0952	0.1429
0.0714	2.7857	0.3571	0.5000	0.4286	0.0714	0.0000	0.0000	0.1579	1.0000	0.0000	0.1053	0.1579
0.1818	2.9091	0.3818	0.3636	0.3636	0.2727	0.0000	0.0000	0.2000	0.9091	0.0909	0.0667	0.2000
0.1667	2.9167	0.3833	0.3333	0.5000	0.1667	0.0000	0.0000	0.1765	0.9167	0.0833	0.1176	0.1765
0.1000	2.8000	0.3600	0.6000	0.4000	0.0000	0.0000	0.0000	0.1429	1.0000	0.0000	0.1429	0.1429
0.1000	2.4000	0.2800	0.5000	0.3000	0.1000	0.0000	0.0000	0.2000	1.0000	0.0000	0.1333	0.2000
0.1250	2.8750	0.3750	0.3750	0.4375	0.1875	0.0000	0.0000	0.1818	1.0000	0.0000	0.0909	0.1818
0.0667	2.6000	0.3200	0.4667	0.4000	0.1333	0.0000	0.0000	0.2000	0.8667	0.1333	0.0500	0.2000
0.0714	2.5385	0.3077	0.2857	0.5714	0.0714	0.0714	0.0000	0.0588	1.0000	0.0000	0.0588	0.1176
0.1000	3.1053	0.4211	0.2000	0.6500	0.1000	0.0500	0.0000	0.0800	0.9500	0.0500	0.0800	0.1200
0.0588	3.0625	0.4125	0.1765	0.5882	0.1765	0.0588	0.0000	0.1364	1.0000	0.0000	0.0455	0.1818
0.1176	3.3125	0.4625	0.1176	0.6471	0.1765	0.0588	0.0000	0.0909	0.8235	0.1765	0.0909	0.1364
0.0000	2.3333	0.2667	0.2308	0.6154	0.0769	0.0769	0.0000	0.0667	0.9231	0.0769	0.0667	0.0667
0.0769	2.5833	0.3167	0.2308	0.5385	0.1538	0.0769	0.0000	0.0667	1.0000	0.0000	0.0667	0.0667
0.0000	3.1000	0.4200	0.2727	0.4545	0.1818	0.0909	0.0000	0.0714	1.0000	0.0000	0.1429	0.0714
0.1111	3.1250	0.4250	0.0000	0.6667	0.2222	0.1111	0.0000	0.0833	0.8889	0.1111	0.1667	0.0833
0.3125	4.1613	0.6323	0.0000	0.7813	0.1875	0.0313	0.0000	0.0000	0.7188	0.2813	0.0000	0.0000
0.2500	3.9375	0.5875	0.0625	0.6250	0.3125	0.0000	0.0000	0.0000	0.9375	0.0625	0.0000	0.0000
0.0000	2.9167	0.3833	0.2308	0.6154	0.0769	0.0769	0.0000	0.0000	1.0000	0.0000	0.0000	0.0000
0.3333	4.0588	0.6118	0.0000	0.7222	0.2222	0.0556	0.0000	0.0000	0.8889	0.1111	0.0526	0.0000
0.2778	3.5882	0.5176	0.0000	0.7222	0.2222	0.0556	0.0000	0.0000	0.9444	0.0556	0.0526	0.0000
0.2222	3.9615	0.5923	0.0000	0.7037	0.2593	0.0370	0.0000	0.0000	0.7778	0.2222	0.0000	0.0000
0.2917	4.0000	0.6000	0.0000	0.7917	0.2083	0.0000	0.0000	0.0000	0.8333	0.1667	0.0000	0.0000
0.2632	3.6667	0.5333	0.0000	0.7368	0.2105	0.0526	0.0000	0.0000	0.8947	0.1053	0.0500	0.0000
0.3235	4.1515	0.6303	0.0000	0.7647	0.2059	0.0294	0.0000	0.0000	0.7059	0.2941	0.0000	0.0000
0.3750	4.2500	0.6500	0.0000	0.8333	0.1667	0.0000	0.0000	0.0000	0.7500	0.2500	0.0000	0.0000
0.2778	3.8333	0.5667	0.0000	0.7222	0.2778	0.0000	0.0000	0.0000	0.9444	0.0556	0.0000	0.0000
0.2143	3.6923	0.5385	0.0714	0.5714	0.2857	0.0714	0.0000	0.0000	0.9286	0.0714	0.0667	0.0000
0.2353	3.9375	0.5875	0.0000	0.7647	0.1765	0.0588	0.0000	0.0000	0.8824	0.1176	0.0000	0.0000
0.1176	3.1250	0.4250	0.1765	0.6471	0.1176	0.0588	0.0000	0.0000	0.9412	0.0588	0.0556	0.0000
0.1500	3.3158	0.4632	0.1000	0.7000	0.1500	0.0500	0.0000	0.0000	1.0000	0.0000	0.0000	0.0000

0.1818	3.4000	0.4800	0.1364	0.5909	0.2273	0.0455	0.0000	0.0000	1.0000	0.0000	0.0000	0.0000
0.0588	3.1875	0.4375	0.1765	0.6471	0.1176	0.0588	0.0000	0.0000	1.0000	0.0000	0.0556	0.0000
0.0000	2.8333	0.3667	0.3077	0.5385	0.0769	0.0769	0.0000	0.0000	1.0000	0.0000	0.0000	0.0000
0.1250	3.1333	0.4267	0.1875	0.6250	0.1250	0.0625	0.0000	0.0000	0.9375	0.0625	0.0588	0.0000
0.1429	3.3684	0.4737	0.1905	0.5714	0.1905	0.0476	0.0000	0.0000	1.0000	0.0000	0.0455	0.0000
0.2000	3.6667	0.5333	0.0800	0.6800	0.2000	0.0400	0.0000	0.0000	0.9600	0.0400	0.0385	0.0000
0.1500	3.0000	0.4000	0.2000	0.7000	0.0500	0.0500	0.0000	0.0000	1.0000	0.0000	0.0000	0.0000
0.1667	3.2941	0.4588	0.1111	0.7222	0.1111	0.0556	0.0000	0.0000	1.0000	0.0000	0.0000	0.0000
0.2353	3.3529	0.4706	0.1176	0.7647	0.1176	0.0000	0.0000	0.0000	1.0000	0.0000	0.0000	0.0000
0.0000	2.2857	0.2571	0.3750	0.5000	0.0000	0.1250	0.0000	0.2000	0.8750	0.1250	0.0000	0.2000
0.0000	2.6000	0.3200	0.1667	0.6667	0.0000	0.1667	0.0000	0.3000	1.0000	0.0000	0.1000	0.3000
0.0556	3.1765	0.4353	0.1667	0.6111	0.1667	0.0556	0.0000	0.1304	1.0000	0.0000	0.0870	0.1304
0.0625	3.1333	0.4267	0.1875	0.5625	0.1875	0.0625	0.0000	0.0556	0.9375	0.0625	0.0556	0.0556
0.0000	2.4615	0.2923	0.2143	0.6429	0.0714	0.0714	0.0000	0.0667	1.0000	0.0000	0.0000	0.0667
0.2500	3.2632	0.4526	0.2000	0.5500	0.2000	0.0500	0.0000	0.1667	1.0000	0.0000	0.0000	0.1667
0.1304	3.3182	0.4636	0.1304	0.6087	0.2174	0.0435	0.0000	0.2069	1.0000	0.0000	0.0000	0.2069
0.1000	2.5556	0.3111	0.2000	0.6000	0.1000	0.1000	0.0000	0.0000	1.0000	0.0000	0.0000	0.0000
0.1714	3.5882	0.5176	0.0571	0.6286	0.2571	0.0286	0.0256	0.0513	1.0000	0.0000	0.0256	0.0769
0.1429	2.8500	0.3700	0.2381	0.5238	0.1905	0.0476	0.0000	0.2333	1.0000	0.0000	0.0333	0.2667
0.1481	3.3750	0.4750	0.0741	0.7407	0.1111	0.0741	0.0000	0.2286	0.9630	0.0000	0.0000	0.2286
0.1111	2.8889	0.3778	0.2222	0.6667	0.1111	0.0000	0.0000	0.3333	1.0000	0.0000	0.0000	0.4000
0.0000	2.5000	0.3000	0.2500	0.7500	0.0000	0.0000	0.0000	0.3077	1.0000	0.0000	0.0000	0.3846
0.2500	3.8846	0.5769	0.0357	0.8214	0.1429	0.0000	0.0000	0.0000	0.8929	0.0714	0.0345	0.0000
0.1408	3.5942	0.5188	0.0704	0.6056	0.2817	0.0000	0.0000	0.0000	0.8873	0.0563	0.0000	0.0000
0.2258	3.8276	0.5655	0.0323	0.7742	0.1935	0.0000	0.0000	0.0000	0.8387	0.1290	0.0313	0.0000
0.1613	3.5833	0.5167	0.1129	0.5645	0.2903	0.0000	0.0000	0.0000	0.8871	0.0645	0.0000	0.0000
0.1739	3.0870	0.4174	0.1304	0.6522	0.2174	0.0000	0.0000	0.0000	1.0000	0.0000	0.0000	0.0000
0.1304	3.2727	0.4545	0.0435	0.5217	0.3913	0.0435	0.0000	0.0000	0.9565	0.0435	0.0000	0.0000
0.3333	3.7778	0.5556	0.0556	0.6667	0.2778	0.0000	0.0000	0.0000	0.8333	0.1667	0.0000	0.0000
0.1000	3.4000	0.4800	0.1000	0.7000	0.2000	0.0000	0.0000	0.0000	1.0000	0.0000	0.0000	0.0000
0.2381	3.2500	0.4500	0.0952	0.5238	0.2381	0.0476	0.0000	0.0000	1.0000	0.0000	0.0000	0.0000
0.1538	3.0833	0.4167	0.3077	0.5385	0.0769	0.0000	0.0000	0.0000	1.0000	0.0000	0.0000	0.0000
0.1111	3.2778	0.4556	0.1667	0.6111	0.2222	0.0000	0.0000	0.0000	1.0000	0.0000	0.0000	0.0000
0.3200	3.8261	0.5652	0.0000	0.8400	0.1600	0.0000	0.0000	0.0000	0.2400	0.6800	0.0000	0.0000
0.2500	3.9048	0.5810	0.0000	0.7917	0.2083	0.0000	0.0000	0.0000	0.4583	0.4167	0.0000	0.0000
0.0968	3.2000	0.4400	0.0968	0.7097	0.0968	0.0323	0.0000	0.0000	0.9355	0.0323	0.1143	0.0000
0.0667	3.1724	0.4345	0.0333	0.7000	0.1333	0.0333	0.0000	0.0000	0.9667	0.0333	0.0909	0.0000
0.0385	3.0769	0.4154	0.1154	0.6923	0.1538	0.0000	0.0000	0.0000	0.9615	0.0385	0.0714	0.0000
0.0968	3.0968	0.4194	0.1290	0.7419	0.0645	0.0000	0.0000	0.0000	1.0000	0.0000	0.0000	0.0000
0.0476	3.4000	0.4800	0.1429	0.5238	0.2857	0.0476	0.0000	0.0000	1.0000	0.0000	0.0000	0.0000
0.0417	3.3913	0.4783	0.0833	0.6667	0.2083	0.0417	0.0000	0.0000	0.9583	0.0000	0.0400	0.0000
0.0833	3.4783	0.4957	0.0833	0.7500	0.1250	0.0417	0.0000	0.0000	1.0000	0.0000	0.0000	0.0000
0.0455	3.1364	0.4273	0.0909	0.5909	0.2727	0.0000	0.0000	0.0000	1.0000	0.0000	0.0833	0.0000
0.1429	3.2381	0.4476	0.0476	0.6667	0.2857	0.0000	0.0000	0.0000	1.0000	0.0000	0.0870	0.0000
0.0357	3.1071	0.4214	0.1071	0.7143	0.1071	0.0000	0.0000	0.0000	1.0000	0.0000	0.0345	0.0000
0.0500	3.0000	0.4000	0.1000	0.5500	0.1500	0.0500	0.0000	0.0000	1.0000	0.0000	0.1304	0.0000
0.0000	3.1111	0.4222	0.2222	0.5556	0.1111	0.0000	0.0000	0.0000	1.0000	0.0000	0.2500	0.0000
0.0000	3.0000	0.4000	0.3333	0.6667	0.0000	0.0000	0.0000	0.0000	1.0000	0.0000	0.2500	0.0000
0.1000	3.1500	0.4300	0.2000	0.7000	0.1000	0.0000	0.0000	0.0000	0.9500	0.0500	0.0476	0.0000
0.1000	3.0000	0.4000	0.3500	0.4500	0.1500	0.0000	0.0000	0.0000	1.0000	0.0000	0.0909	0.0000
0.0909	3.0909	0.4182	0.2727	0.5000	0.1364	0.0000	0.0000	0.0000	1.0000	0.0000	0.0000	0.0000
0.1667	3.0000	0.4000	0.3333	0.4167	0.1667	0.0000	0.0000	0.0000	1.0000	0.0000	0.0000	0.0000
0.1111	2.7778	0.3556	0.3889	0.3889	0.1111	0.0000	0.0000	0.0000	1.0000	0.0000	0.0000	0.0000
0.1429	2.7857	0.3571	0.4286	0.4286	0.0714	0.0000	0.0000	0.0000	1.0000	0.0000	0.0000	0.0000
0.0588	3.1176	0.4235	0.1765	0.7059	0.1176	0.0000	0.0000	0.0000	0.9412	0.0588	0.1500	0.0000
0.1481	2.8519	0.3704	0.1852	0.7407	0.0741	0.0000	0.0000	0.0000	0.9630	0.0000	0.0000	0.0000
0.2083	3.0000	0.4000	0.2083	0.7083	0.0417	0.0000	0.0000	0.0000	0.9583	0.0000	0.0400	0.0000
0.1111	3.1200	0.4240	0.1852	0.5556	0.1481	0.0370	0.0000	0.0000	1.0000	0.0000	0.0690	0.0000
0.1200	3.2500	0.4500	0.1600	0.6400	0.1200	0.0000	0.0000	0.0000	0.9600	0.0400	0.0385	0.0000
0.0500	3.2000	0.4400	0.1500	0.6000	0.2000	0.0000	0.0000	0.0000	1.0000	0.0000	0.0000	0.0000
0.2000	3.3000	0.4600	0.2000	0.5500	0.2000	0.0000	0.0000	0.0000	1.0000	0.0000	0.0000	0.0000
0.4667	4.5000	0.7000	0.0000	0.4667	0.5333	0.0000	0.0000	0.0000	0.8667	0.1333	0.0000	0.0000
0.1200	3.3333	0.4667	0.2400	0.4000	0.3600	0.0000	0.0000	0.0000	0.9600	0.0000	0.0000	0.0000
0.3125	3.9032	0.5806	0.1250	0.5625	0.3125	0.0000	0.0000	0.0000	0.9375	0.0313	0.0303	0.0000
0.1538	3.5417	0.5083	0.1923	0.3846	0.3846	0.0385	0.0000	0.0000	0.9615	0.0000	0.0000	0.0000
0.0909	3.3500	0.4700	0.2727	0.3636	0.3182	0.0455	0.0000	0.0000	0.9545	0.0000	0.0000	0.0000
0.2791	3.7317	0.5463	0.0000	0.7442	0.2558	0.0000	0.0000	0.0000	0.8605	0.1163	0.0000	0.0000
0.2222	3.5882	0.5176	0.0556	0.6111	0.3333	0.0000	0.0000	0.0000	1.0000	0.0000	0.0000	0.0000
0.2759	3.6071	0.5214	0.1034	0.5862	0.3103	0.0000	0.0000	0.0000	1.0000	0.0000	0.0000	0.0000
0.3158	4.3529	0.6706	0.0000	0.5263	0.4211	0.0526	0.0000	0.0000	0.8421	0.1579	0.0000	0.0000
0.1481	3.7692	0.5538	0.1852	0.5185	0.2963	0.0000	0.0000	0.0000	0.9630	0.0000	0.0357	0.0000
0.2424	3.6250	0.5250	0.1212	0.5758	0.2727	0.0303	0.0000	0.0000	0.9697	0.0303	0.0000	0.0000
0.1538	3.5600	0.5120	0.1154	0.6154	0.2692	0.0000	0.0000	0.0000	0.9615	0.0000	0.0000	0.0000
0.0769	3.4000	0.4800	0.1923	0.5385	0.2692	0.0000	0.0000	0.0000	0.9615	0.0000	0.0000	0.0000
0.0588	3.3529	0.4706	0.1176	0.5882	0.2941	0.0000	0.0000	0.0000	0.9412	0.0588	0.0000	0.0000
0.0667	2.9286	0.3857	0.2667	0.4667	0.2000	0.0000	0.0000	0.2083	1.0000	0.0000	0.1667	0.2083
0.0000	2.7391	0.3478	0.1250	0.7500	0.1250	0.0000	0.0000	0.0000	0.9583	0.0000	0.1429	0.0000

0.0000	2.6667	0.3333	0.0909	0.5455	0.1818	0.0000	0.0000	0.0000	0.8182	0.0000	0.2143	0.0000
0.0000	2.5217	0.3043	0.3077	0.5385	0.1154	0.0000	0.0000	0.0000	0.9615	0.0000	0.1333	0.0000
0.2609	4.0476	0.6095	0.2174	0.4348	0.3043	0.0435	0.0000	0.0000	1.0000	0.0000	0.0000	0.0000
0.3529	4.3750	0.6750	0.1176	0.4706	0.4118	0.0000	0.0000	0.0000	1.0000	0.0000	0.0000	0.0000
0.4545	4.3810	0.6762	0.0455	0.5455	0.4091	0.0000	0.0000	0.0000	1.0000	0.0000	0.0000	0.0000
0.3500	4.0500	0.6100	0.1500	0.5000	0.3500	0.0000	0.0000	0.0000	1.0000	0.0000	0.0000	0.0000
0.0000	2.6667	0.3333	0.3333	0.5000	0.1667	0.0000	0.0000	0.0000	1.0000	0.0000	0.0000	0.0000
0.0000	2.2222	0.2444	0.2222	0.6667	0.1111	0.0000	0.0000	0.0000	1.0000	0.0000	0.0000	0.0000
0.2917	3.8182	0.5636	0.1250	0.5000	0.3750	0.0000	0.0000	0.0000	1.0000	0.0000	0.0000	0.0000
0.3333	4.1154	0.6231	0.1111	0.4815	0.4074	0.0000	0.0000	0.0000	1.0000	0.0000	0.0000	0.0000
0.4762	4.2500	0.6500	0.0000	0.4762	0.5238	0.0000	0.0000	0.0000	1.0000	0.0000	0.0000	0.0000
0.3333	4.0000	0.6000	0.0741	0.4815	0.4444	0.0000	0.0000	0.0000	0.9630	0.0370	0.0000	0.0000
0.3000	3.4737	0.4947	0.2500	0.4000	0.3500	0.0000	0.0000	0.0000	1.0000	0.0000	0.0000	0.0000
0.0556	2.6667	0.3333	0.2222	0.6111	0.1667	0.0000	0.0000	0.0000	1.0000	0.0000	0.0000	0.0000
0.2727	4.4762	0.6952	0.0455	0.5909	0.3636	0.0000	0.0000	0.0000	0.7727	0.2273	0.0000	0.0000
0.1579	3.4118	0.4824	0.0526	0.5789	0.2632	0.1053	0.0500	0.0000	1.0000	0.0000	0.0000	0.0500
0.1200	3.3636	0.4727	0.1200	0.5600	0.2000	0.1200	0.0000	0.0000	1.0000	0.0000	0.0000	0.0000
0.3333	3.6667	0.5333	0.0000	0.3333	0.6667	0.0000	0.0000	0.0000	1.0000	0.0000	0.0000	0.0000
0.0000	2.9000	0.3800	0.0833	0.6667	0.0833	0.1667	0.0000	0.0000	1.0000	0.0000	0.0000	0.0000
0.2381	4.3500	0.6700	0.0000	0.6190	0.3810	0.0000	0.0000	0.0000	0.8571	0.1429	0.0000	0.0000
0.2857	4.5000	0.7000	0.0000	0.6071	0.3929	0.0000	0.0000	0.0000	0.8214	0.1429	0.0000	0.0000
0.0833	3.6500	0.5300	0.0000	0.5417	0.3333	0.1250	0.0294	0.0000	0.8750	0.0833	0.2647	0.0294
0.0769	3.0833	0.4167	0.1538	0.5385	0.1538	0.0769	0.0588	0.0000	1.0000	0.0000	0.1765	0.0588
0.0000	2.7500	0.3500	0.2000	0.6000	0.0000	0.2000	0.0000	0.0000	1.0000	0.0000	0.0000	0.0000
0.3333	4.2727	0.6545	0.0000	0.4167	0.5833	0.0000	0.0000	0.0000	0.8333	0.1667	0.0000	0.0000
0.2941	4.1875	0.6375	0.0000	0.5294	0.4706	0.0000	0.0000	0.0000	0.7647	0.2353	0.0000	0.0000
0.2778	4.1176	0.6235	0.0000	0.4444	0.5556	0.0000	0.0000	0.0000	0.8333	0.1667	0.0000	0.0000
0.4286	4.4615	0.6923	0.0000	0.5714	0.4286	0.0000	0.0000	0.0000	0.9286	0.0714	0.0000	0.0000
0.1176	3.1765	0.4353	0.1765	0.4706	0.3529	0.0000	0.0000	0.0000	1.0000	0.0000	0.0000	0.0000
0.3500	4.5000	0.7000	0.0000	0.4000	0.6000	0.0000	0.0000	0.0000	0.7500	0.2000	0.0000	0.0000
0.3500	4.5000	0.7000	0.0500	0.4500	0.5000	0.0000	0.0000	0.0000	0.7500	0.2000	0.0000	0.0000
0.1500	3.1111	0.4222	0.0000	0.8000	0.1500	0.0500	0.0000	0.0000	0.7500	0.2000	0.2593	0.0000
0.2308	4.0833	0.6167	0.0000	0.6154	0.3846	0.0000	0.0000	0.0000	0.9231	0.0769	0.0000	0.0000
0.2727	4.1000	0.6200	0.1364	0.5000	0.3636	0.0000	0.0000	0.0000	0.9545	0.0000	0.0000	0.0000
0.0000	3.3333	0.4667	0.1538	0.6154	0.1538	0.0769	0.0000	0.0000	1.0000	0.0000	0.2778	0.0000
0.0000	2.6500	0.3300	0.3182	0.5455	0.0909	0.0455	0.0000	0.0000	1.0000	0.0000	0.0833	0.0000
0.1000	2.8000	0.3600	0.3000	0.6000	0.1000	0.0000	0.0000	0.0000	1.0000	0.0000	0.0000	0.0000
0.4000	3.0000	0.4000	0.0000	0.8000	0.2000	0.0000	0.0000	0.0000	0.6000	0.4000	0.0000	0.0000
0.0000	2.1429	0.2286	0.2857	0.7143	0.0000	0.0000	0.0000	0.0000	1.0000	0.0000	0.0000	0.0000
0.1500	3.2500	0.4500	0.1000	0.7000	0.2000	0.0000	0.0000	0.0000	0.9000	0.0500	0.0000	0.0000
0.0000	2.2500	0.2500	0.2500	0.7500	0.0000	0.0000	0.0000	0.0000	1.0000	0.0000	0.0000	0.0000
0.2778	4.2353	0.6471	0.0556	0.5000	0.4444	0.0000	0.0000	0.0000	1.0000	0.0000	0.0000	0.0000
0.0000	2.6875	0.3375	0.0526	0.7368	0.0526	0.1579	0.0000	0.0000	1.0000	0.0000	0.0000	0.0000
0.0714	3.0769	0.4154	0.2143	0.6429	0.1429	0.0000	0.0000	0.2692	1.0000	0.0000	0.1923	0.2692
0.0000	2.1667	0.2333	0.0000	1.0000	0.0000	0.0000	0.0000	0.0000	1.0000	0.0000	0.0000	0.0000
0.0000	2.2500	0.2500	0.0000	1.0000	0.0000	0.0000	0.0000	0.0000	1.0000	0.0000	0.0000	0.0000
0.3333	4.3333	0.6667	0.0000	0.4000	0.6000	0.0000	0.0000	0.0000	1.0000	0.0000	0.0000	0.0000
0.2941	4.4375	0.6875	0.0000	0.4118	0.5882	0.0000	0.0000	0.0000	1.0000	0.0000	0.0000	0.0000
0.0968	3.1724	0.4345	0.1290	0.6129	0.1935	0.0645	0.0000	0.0000	0.8710	0.1290	0.1622	0.0000
0.0909	3.0476	0.4095	0.2273	0.5455	0.1818	0.0455	0.0357	0.0000	0.9545	0.0455	0.1786	0.0357
0.0000	2.0000	0.2000	0.1667	0.8333	0.0000	0.0000	0.0000	0.0000	1.0000	0.0000	0.0000	0.0000
0.2857	4.0000	0.6000	0.0000	0.5714	0.4286	0.0000	0.0000	0.0000	0.8571	0.1429	0.0000	0.0000
0.1000	3.1000	0.4200	0.1000	0.6000	0.3000	0.0000	0.0000	0.0000	0.7000	0.3000	0.0000	0.0000
0.0000	2.6250	0.3250	0.3750	0.3750	0.2500	0.0000	0.0000	0.0000	0.7500	0.2500	0.0000	0.0000
0.0667	3.0714	0.4143	0.1333	0.6000	0.2000	0.0000	0.0000	0.2000	1.0000	0.0000	0.2000	0.2000
0.2105	4.2105	0.6421	0.0000	0.5789	0.4211	0.0000	0.0000	0.0000	0.8947	0.1053	0.0000	0.0000
0.2000	3.6000	0.5200	0.0000	0.8000	0.2000	0.0000	0.0000	0.0000	0.8000	0.2000	0.0000	0.0000
0.2500	3.9167	0.5833	0.0417	0.6250	0.3333	0.0000	0.0000	0.0000	0.8333	0.1667	0.0000	0.0000
0.1111	3.2941	0.4588	0.0000	0.6667	0.2778	0.0556	0.0000	0.0000	0.7222	0.2222	0.3333	0.0000
0.2000	3.2500	0.4500	0.2000	0.2000	0.2000	0.2000	0.0769	0.0000	0.8000	0.2000	0.5385	0.0769
0.1250	2.8333	0.3667	0.2500	0.1250	0.3750	0.2500	0.1667	0.0000	1.0000	0.0000	0.1667	0.1667
0.2500	3.0000	0.4000	0.2500	0.0000	0.7500	0.0000	0.2500	0.0000	1.0000	0.0000	0.2500	0.2500
0.1818	2.6000	0.3200	0.2727	0.1818	0.3636	0.0909	0.1667	0.0000	0.8182	0.1818	0.2222	0.1667
0.0000	2.2500	0.2500	0.3333	0.4444	0.1111	0.0000	0.0000	0.0000	0.8889	0.0000	0.1818	0.0000
0.0000	2.5625	0.3125	0.2353	0.5882	0.1176	0.0588	0.0000	0.0000	1.0000	0.0000	0.1905	0.0000
0.1739	2.7143	0.3429	0.2174	0.5652	0.1739	0.0435	0.0800	0.0000	0.9565	0.0435	0.0000	0.0800
0.2083	3.3043	0.4609	0.2083	0.5833	0.1667	0.0417	0.0714	0.0714	1.0000	0.0000	0.0000	0.1429
0.0000	3.2222	0.4444	0.3333	0.4444	0.2222	0.0000	0.0000	0.0000	1.0000	0.0000	0.0000	0.0000
0.2750	3.5000	0.5000	0.0750	0.7000	0.2000	0.0250	0.0000	0.0000	0.9000	0.0500	0.0244	0.0000
0.4516	4.0000	0.6000	0.0323	0.5161	0.4516	0.0000	0.0000	0.0313	0.7742	0.1935	0.0000	0.0313
0.2500	3.8182	0.5636	0.0179	0.7321	0.2321	0.0000	0.0000	0.0000	0.8036	0.1250	0.0000	0.0000
0.2449	3.5957	0.5191	0.0816	0.7143	0.1837	0.0000	0.0000	0.0926	0.9592	0.0000	0.0000	0.0926
0.3548	4.0690	0.6138	0.0645	0.5806	0.3226	0.0323	0.0000	0.0000	0.8387	0.1613	0.0000	0.0000
0.4444	4.2692	0.6538	0.0000	0.7037	0.2963	0.0000	0.0000	0.0000	0.6296	0.3333	0.0000	0.0000
0.2927	3.9250	0.5850	0.0488	0.6829	0.2683	0.0000	0.0000	0.0233	0.8780	0.1220	0.0233	0.0233
0.2000	3.7333	0.5467	0.0667	0.6000	0.3111	0.0000	0.0000	0.0217	0.8444	0.1333	0.0000	0.0217
0.3571	4.0244	0.6049	0.0476	0.6190	0.3333	0.0000	0.0000	0.0000	0.7381	0.2381	0.0000	0.0000

0.2632	3.8889	0.5778	0.1053	0.6316	0.2632	0.0000	0.0000	0.0000	0.7368	0.2105	0.0000	0.0000
0.3000	3.6316	0.5263	0.0000	0.9000	0.1000	0.0000	0.0000	0.0000	0.3000	0.6500	0.0000	0.0000
0.2963	3.4231	0.4846	0.1852	0.5185	0.2593	0.0370	0.0000	0.1000	1.0000	0.0000	0.0000	0.1000
0.2800	3.9167	0.5833	0.0400	0.6800	0.2800	0.0000	0.0000	0.0385	0.8800	0.1200	0.0000	0.0385
0.1923	3.6154	0.5231	0.0769	0.6154	0.3077	0.0000	0.0000	0.1034	0.9615	0.0000	0.0000	0.1034
0.2432	3.6111	0.5222	0.1622	0.5946	0.2162	0.0270	0.0000	0.0732	0.9730	0.0270	0.0244	0.0732
0.2564	3.8158	0.5632	0.1282	0.5641	0.2564	0.0256	0.0000	0.0714	0.9231	0.0513	0.0000	0.0714
0.2500	3.6316	0.5263	0.1000	0.5500	0.3500	0.0000	0.0000	0.0000	0.9500	0.0500	0.0476	0.0000
0.2381	3.7143	0.5429	0.0952	0.4762	0.4286	0.0000	0.0000	0.0435	0.9048	0.0952	0.0435	0.0435
0.2778	3.7778	0.5556	0.1111	0.6667	0.2222	0.0000	0.0000	0.1429	0.9444	0.0556	0.0000	0.1429
0.2105	3.6842	0.5368	0.1579	0.5789	0.2632	0.0000	0.0000	0.0952	1.0000	0.0000	0.0000	0.0952
0.3125	3.9375	0.5875	0.0625	0.5625	0.3750	0.0000	0.0000	0.1111	1.0000	0.0000	0.0000	0.1111
0.1500	3.7778	0.5556	0.0500	0.6000	0.3000	0.0500	0.0000	0.0476	0.8000	0.1500	0.0000	0.0476
0.2000	3.0000	0.4000	0.2000	0.5333	0.2000	0.0667	0.0556	0.0556	0.9333	0.0667	0.0556	0.1111
0.2000	3.0000	0.4000	0.3000	0.4000	0.3000	0.0000	0.0714	0.0714	1.0000	0.0000	0.1429	0.1429
0.1429	3.1053	0.4211	0.2857	0.4762	0.1905	0.0000	0.0400	0.1200	0.9524	0.0476	0.0000	0.1600
0.1429	3.2857	0.4571	0.2857	0.4286	0.2857	0.0000	0.1000	0.1000	1.0000	0.0000	0.1000	0.2000
0.1500	3.0000	0.4000	0.3500	0.5000	0.1000	0.0500	0.0435	0.0870	1.0000	0.0000	0.0000	0.1304
0.2667	3.0714	0.4143	0.1333	0.4000	0.3333	0.0667	0.0556	0.0556	0.8667	0.1333	0.0556	0.1111
0.1000	2.8947	0.3789	0.2500	0.5500	0.1500	0.0500	0.0435	0.0870	0.9500	0.0500	0.0000	0.1304
0.2143	3.2308	0.4462	0.2143	0.3571	0.2857	0.0714	0.0625	0.0625	1.0000	0.0000	0.0000	0.1250
0.2222	3.5000	0.5000	0.1111	0.6667	0.1481	0.0370	0.0345	0.0345	0.9259	0.0741	0.0000	0.0690
0.1515	3.2813	0.4563	0.1212	0.6364	0.1818	0.0303	0.0286	0.0286	0.9091	0.0909	0.0000	0.0571
0.1613	3.4138	0.4828	0.1290	0.5806	0.1935	0.0323	0.0244	0.1463	0.9355	0.0645	0.0488	0.1951
0.2069	3.4286	0.4857	0.1034	0.6552	0.1724	0.0345	0.0286	0.1143	0.9310	0.0690	0.0286	0.1429
0.1481	3.5385	0.5077	0.1111	0.6296	0.1852	0.0370	0.0313	0.0938	0.9630	0.0370	0.0313	0.1250
0.1739	3.5909	0.5182	0.1304	0.5652	0.2609	0.0435	0.0345	0.1379	0.9565	0.0435	0.0345	0.1724
0.2333	3.7857	0.5571	0.0667	0.7000	0.2000	0.0000	0.0000	0.2000	0.9000	0.0667	0.0500	0.2000
0.3333	3.9730	0.5946	0.0256	0.7692	0.2051	0.0000	0.0000	0.0465	0.7949	0.2051	0.0465	0.0465
0.3158	3.7838	0.5568	0.0000	0.7895	0.2105	0.0000	0.0000	0.1111	0.8158	0.1842	0.0444	0.1111
0.2750	3.7895	0.5579	0.0000	0.8750	0.1250	0.0000	0.0000	0.0652	0.7750	0.2000	0.0652	0.0652
0.2083	3.3913	0.4783	0.0417	0.7500	0.2083	0.0000	0.0000	0.1111	0.7500	0.2083	0.0000	0.1111
0.2000	3.1333	0.4267	0.0000	0.8000	0.2000	0.0000	0.0000	0.1176	0.7333	0.2667	0.0000	0.1176
0.2857	3.6829	0.5366	0.0238	0.8095	0.1429	0.0000	0.0000	0.1020	0.8571	0.1429	0.0408	0.1020
0.1569	3.7917	0.5583	0.0392	0.7059	0.2549	0.0000	0.0000	0.0727	0.7647	0.2157	0.0000	0.0727
0.0789	3.1143	0.4229	0.0000	0.7895	0.1579	0.0263	0.0000	0.0256	0.7632	0.2105	0.0000	0.0256
0.2105	3.7222	0.5444	0.0000	0.7632	0.2105	0.0263	0.0000	0.0500	0.7368	0.2368	0.0000	0.0500
0.2000	3.5500	0.5100	0.0000	0.8500	0.1500	0.0000	0.0000	0.0476	0.7500	0.2500	0.0000	0.0476
0.2162	3.7778	0.5556	0.0000	0.7027	0.2703	0.0000	0.0000	0.1522	0.8919	0.1081	0.0435	0.1522
0.2500	4.1667	0.6333	0.0500	0.7000	0.2500	0.0000	0.0000	0.1250	1.0000	0.0000	0.0417	0.1250
0.3077	3.8462	0.5692	0.0769	0.5385	0.3846	0.0000	0.0000	0.1875	1.0000	0.0000	0.0000	0.1875
0.2973	3.9412	0.5882	0.0270	0.8108	0.1622	0.0000	0.0000	0.1395	0.8108	0.1622	0.0000	0.1395
0.2353	4.0000	0.6000	0.0000	0.8529	0.1471	0.0000	0.0000	0.1250	0.8824	0.0882	0.0000	0.1500
0.3750	4.4286	0.6857	0.0417	0.7083	0.2500	0.0000	0.0000	0.2000	0.8333	0.1250	0.0000	0.2000
0.2353	3.6061	0.5212	0.0000	0.8235	0.1765	0.0000	0.0000	0.1000	0.8235	0.1471	0.0500	0.1000
0.2857	3.8148	0.5630	0.0000	0.7857	0.2143	0.0000	0.0000	0.2432	0.9643	0.0357	0.0000	0.2432
0.2222	3.5333	0.5067	0.0556	0.7778	0.1111	0.0000	0.0000	0.1818	0.7778	0.1667	0.0000	0.1818
0.1515	3.3548	0.4710	0.0303	0.7879	0.1212	0.0303	0.0000	0.0286	0.9394	0.0606	0.0286	0.0286
0.2727	3.2188	0.4438	0.1818	0.6061	0.2121	0.0000	0.0444	0.1556	1.0000	0.0000	0.0667	0.2000
0.2857	3.3333	0.4667	0.1429	0.7143	0.1429	0.0000	0.0286	0.1143	1.0000	0.0000	0.0286	0.1714
0.1875	3.0714	0.4143	0.1875	0.6250	0.1250	0.0000	0.0000	0.0588	0.9375	0.0000	0.0000	0.0588
0.2000	3.0000	0.4000	0.2667	0.5333	0.1333	0.0000	0.0000	0.0625	0.9333	0.0000	0.0000	0.0625
0.2083	3.5417	0.5083	0.0000	0.7917	0.2083	0.0000	0.0513	0.1795	0.9583	0.0417	0.1026	0.2821
0.2813	3.8667	0.5733	0.1250	0.6563	0.2188	0.0000	0.0000	0.0588	1.0000	0.0000	0.0000	0.0588
0.3125	3.8276	0.5655	0.0625	0.7500	0.1563	0.0313	0.0000	0.1837	1.0000	0.0000	0.0612	0.2857
0.2333	3.3571	0.4714	0.0333	0.7000	0.1667	0.0667	0.0833	0.1458	1.0000	0.0000	0.1042	0.2708
0.2619	3.6750	0.5350	0.1190	0.7143	0.1667	0.0000	0.0000	0.0435	0.9524	0.0476	0.0435	0.0435
0.2381	3.7250	0.5450	0.0952	0.7381	0.1667	0.0000	0.0000	0.0851	0.9524	0.0476	0.0213	0.0851
0.1667	3.7500	0.5500	0.0000	0.6333	0.3000	0.0667	0.0000	0.0476	0.9667	0.0333	0.2381	0.0476
0.1892	3.3714	0.4743	0.1081	0.7297	0.1351	0.0270	0.0714	0.1250	1.0000	0.0000	0.1250	0.2143
0.3636	3.5000	0.5000	0.1364	0.5909	0.2273	0.0000	0.0714	0.1071	1.0000	0.0000	0.0357	0.1786
0.2143	3.2692	0.4538	0.1786	0.5714	0.2143	0.0000	0.0882	0.0882	1.0000	0.0000	0.0000	0.1765
0.2500	3.2308	0.4462	0.1786	0.6071	0.1786	0.0000	0.1250	0.1250	1.0000	0.0000	0.0000	0.3000
0.2778	3.6857	0.5371	0.0556	0.8333	0.1111	0.0000	0.0000	0.0526	0.9444	0.0556	0.0000	0.0526
0.3000	3.6923	0.5385	0.0500	0.7750	0.1750	0.0000	0.0000	0.0476	0.9750	0.0250	0.0000	0.0476
0.2353	3.2500	0.4500	0.1176	0.7059	0.1765	0.0000	0.0000	0.0526	0.9412	0.0588	0.0526	0.0526
0.2500	3.4074	0.4815	0.2143	0.6071	0.1786	0.0000	0.0000	0.0667	0.9286	0.0714	0.0000	0.0667
0.3182	3.9500	0.5900	0.0909	0.7273	0.1818	0.0000	0.0000	0.1290	1.0000	0.0000	0.1613	0.1290
0.1500	3.5556	0.5111	0.1500	0.5500	0.3000	0.0000	0.0000	0.0833	1.0000	0.0000	0.0833	0.0833
0.2500	3.6500	0.5300	0.1818	0.6136	0.1818	0.0000	0.0000	0.0625	0.9545	0.0455	0.0208	0.0625
0.2500	3.3913	0.4783	0.2083	0.6250	0.1667	0.0000	0.0000	0.1333	1.0000	0.0000	0.0667	0.1333
0.2188	3.4333	0.4867	0.2188	0.5625	0.2188	0.0000	0.0000	0.1250	0.9688	0.0313	0.0750	0.1250
0.2308	3.7778	0.5556	0.1538	0.6410	0.2051	0.0000	0.0000	0.0714	1.0000	0.0000	0.0000	0.0714
0.2619	3.5897	0.5179	0.0238	0.8333	0.1190	0.0238	0.0000	0.0638	0.9762	0.0238	0.0213	0.0851
0.3333	4.0000	0.6000	0.0000	0.6000	0.4000	0.0000	0.0000	0.0000	0.8000	0.2000	0.0000	0.0000
0.1818	4.0000	0.6000	0.0000	0.6364	0.3636	0.0000	0.0000	0.0000	0.9091	0.0000	0.0000	0.0000
0.2273	4.0952	0.6190	0.0000	0.7273	0.2727	0.0000	0.0000	0.0000	0.8636	0.1364	0.0000	0.0000

0.2381	4.0952	0.6190	0.0476	0.5714	0.3810	0.0000	0.0000	0.0000	0.9048	0.0952	0.0000	0.0000
0.3200	4.0833	0.6167	0.0400	0.6400	0.3200	0.0000	0.0000	0.0000	0.8800	0.0800	0.0385	0.0000
0.2941	4.1765	0.6353	0.0588	0.4706	0.4706	0.0000	0.0000	0.0000	0.9412	0.0588	0.0000	0.0000
0.4000	4.1000	0.6200	0.0000	0.4000	0.6000	0.0000	0.0000	0.0000	1.0000	0.0000	0.0000	0.0000
0.3182	3.9524	0.5905	0.0455	0.5455	0.4091	0.0000	0.0000	0.0000	0.9545	0.0455	0.0000	0.0000
0.3684	4.2778	0.6556	0.0000	0.4737	0.4737	0.0000	0.0000	0.0000	0.9474	0.0526	0.0000	0.0000
0.3077	3.6154	0.5231	0.0769	0.3077	0.6154	0.0000	0.0000	0.0000	1.0000	0.0000	0.0000	0.0000
0.5385	4.3077	0.6615	0.0000	0.5385	0.4615	0.0000	0.0000	0.0000	1.0000	0.0000	0.0000	0.0000
0.3333	4.4444	0.6889	0.0000	0.5556	0.4444	0.0000	0.0000	0.0000	0.8889	0.1111	0.0000	0.0000
0.2500	4.1667	0.6333	0.0000	0.6500	0.3500	0.0000	0.0000	0.0000	0.7500	0.1500	0.0000	0.0000
0.3529	4.3750	0.6750	0.0000	0.7059	0.2941	0.0000	0.0000	0.0000	0.9412	0.0588	0.0000	0.0000
0.0000	2.0000	0.2000	0.0000	1.0000	0.0000	0.0000	0.0000	0.0000	0.7500	0.2500	0.0000	0.0000
0.3333	4.5000	0.7000	0.0000	0.6667	0.3333	0.0000	0.0000	0.0000	0.9333	0.0667	0.0000	0.0000
0.0000	2.2000	0.2400	0.0000	1.0000	0.0000	0.0000	0.0000	0.0000	1.0000	0.0000	0.0000	0.0000
0.0000	2.2000	0.2400	0.0000	1.0000	0.0000	0.0000	0.0000	0.0000	1.0000	0.0000	0.0000	0.0000
0.0000	2.2000	0.2400	0.0000	1.0000	0.0000	0.0000	0.0000	0.0000	1.0000	0.0000	0.0000	0.0000
0.0667	3.3704	0.4741	0.1333	0.6667	0.1667	0.0000	0.0000	0.0000	0.9000	0.0333	0.0000	0.0000
0.1000	3.1000	0.4200	0.3000	0.6000	0.1000	0.0000	0.0000	0.0000	1.0000	0.0000	0.0000	0.0000
0.0909	3.6500	0.5300	0.0000	0.8636	0.1364	0.0000	0.0000	0.0000	0.7727	0.1818	0.0000	0.0000
0.0526	3.4737	0.4947	0.1053	0.5263	0.3684	0.0000	0.0000	0.0000	0.9474	0.0526	0.0000	0.0000
0.1667	3.4167	0.4833	0.0833	0.5833	0.3333	0.0000	0.0000	0.0000	0.8333	0.1667	0.0000	0.0000
0.0000	3.1429	0.4286	0.0000	0.7143	0.2857	0.0000	0.0000	0.0000	0.8571	0.1429	0.1250	0.0000
0.1250	3.3125	0.4625	0.0625	0.5625	0.3750	0.0000	0.0000	0.0000	0.9375	0.0625	0.0588	0.0000
0.1429	2.8571	0.3714	0.0714	0.5714	0.3571	0.0000	0.0000	0.0000	0.9286	0.0714	0.0000	0.0000
0.2500	3.8000	0.5600	0.1000	0.5500	0.3500	0.0000	0.0000	0.0000	1.0000	0.0000	0.0000	0.0000
0.4167	4.4167	0.6833	0.0000	0.3333	0.6667	0.0000	0.0000	0.0000	1.0000	0.0000	0.0000	0.0000
0.2000	4.0000	0.6000	0.1000	0.3000	0.6000	0.0000	0.0000	0.0000	1.0000	0.0000	0.0000	0.0000
0.0714	3.8462	0.5692	0.0000	0.5714	0.4286	0.0000	0.0000	0.0000	0.7857	0.1429	0.0000	0.0000
0.1000	3.5862	0.5172	0.1000	0.6667	0.2000	0.0000	0.0000	0.0000	0.9333	0.0333	0.0000	0.0000
0.2941	4.0714	0.6143	0.1176	0.7647	0.0588	0.0588	0.0000	0.0526	0.9412	0.0588	0.0526	0.0526
0.1765	3.4375	0.4875	0.1176	0.6471	0.1176	0.0588	0.0000	0.1429	0.9412	0.0588	0.0476	0.1429
0.2174	3.5000	0.5000	0.0870	0.6522	0.1739	0.0435	0.0000	0.0769	0.9130	0.0870	0.0385	0.0769
0.3889	4.2353	0.6471	0.0556	0.7222	0.1111	0.0556	0.0000	0.0952	0.9444	0.0556	0.0476	0.0952
0.2800	3.9091	0.5818	0.0400	0.6800	0.1600	0.0800	0.0000	0.0357	0.9600	0.0400	0.0714	0.0357
0.1923	3.8333	0.5667	0.0769	0.6538	0.1923	0.0385	0.0000	0.0370	0.9615	0.0385	0.0000	0.0370
0.1500	3.5556	0.5111	0.0500	0.5500	0.2500	0.1000	0.0000	0.0455	0.9500	0.0500	0.0455	0.0455
0.2500	3.7647	0.5529	0.1000	0.6500	0.1000	0.1000	0.0000	0.0417	0.9500	0.0500	0.1250	0.0417
0.2500	3.8667	0.5733	0.0625	0.5625	0.3125	0.0625	0.0000	0.0588	1.0000	0.0000	0.0000	0.0588
0.1852	3.6000	0.5200	0.0370	0.5926	0.2593	0.0741	0.0000	0.0357	0.9259	0.0741	0.0000	0.0357
0.2609	4.0000	0.6000	0.0435	0.7391	0.1304	0.0435	0.0000	0.0400	0.9565	0.0435	0.0400	0.0400
0.2609	4.0476	0.6095	0.0435	0.6957	0.1739	0.0435	0.0000	0.0400	0.9565	0.0435	0.0400	0.0400
0.2609	3.6364	0.5273	0.0435	0.7391	0.1304	0.0435	0.0000	0.0417	0.9130	0.0870	0.0000	0.0417
0.3200	4.0870	0.6174	0.0800	0.6400	0.2000	0.0400	0.0000	0.0385	0.9600	0.0400	0.0000	0.0385
0.2609	3.6364	0.5273	0.0870	0.7391	0.1304	0.0435	0.0000	0.0385	0.9565	0.0435	0.0769	0.0385
0.2759	3.8148	0.5630	0.0690	0.6897	0.1379	0.0690	0.0000	0.0323	0.9655	0.0345	0.0323	0.0323
0.2500	4.4167	0.6833	0.0000	0.4167	0.5833	0.0000	0.0000	0.0000	0.9167	0.0833	0.0000	0.0000
0.2857	4.4615	0.6923	0.0000	0.5714	0.4286	0.0000	0.0000	0.0000	0.9286	0.0714	0.0000	0.0000
0.2632	4.3529	0.6706	0.0526	0.5789	0.3684	0.0000	0.0000	0.0000	0.9474	0.0526	0.0000	0.0000
0.2222	4.2500	0.6500	0.0556	0.6111	0.3333	0.0000	0.0000	0.0000	0.9444	0.0556	0.0000	0.0000
0.2667	4.2143	0.6429	0.0000	0.6667	0.3333	0.0000	0.0000	0.0000	0.9333	0.0667	0.0000	0.0000
0.2222	4.1875	0.6375	0.0000	0.6667	0.2778	0.0556	0.0000	0.0000	0.9444	0.0556	0.0000	0.0000
0.2778	4.1765	0.6353	0.0000	0.5556	0.3889	0.0556	0.0000	0.0000	0.9444	0.0556	0.0000	0.0000
0.2381	4.2000	0.6400	0.0000	0.5714	0.4286	0.0000	0.0000	0.0000	0.9524	0.0476	0.0000	0.0000
0.2000	3.4444	0.4889	0.4000	0.4000	0.1000	0.0000	0.0909	0.0000	1.0000	0.0000	0.0000	0.0909
0.0588	3.3125	0.4625	0.2353	0.5882	0.0588	0.0000	0.0909	0.0909	1.0000	0.0000	0.0000	0.2273
0.0000	3.0000	0.4000	0.2857	0.2857	0.2857	0.0000	0.2500	0.0000	1.0000	0.0000	0.1667	0.2500
0.0000	3.2857	0.4571	0.0000	0.4286	0.2857	0.0000	0.2727	0.0000	1.0000	0.0000	0.0000	0.3636
0.1111	3.5000	0.5000	0.1111	0.2222	0.3333	0.1111	0.2000	0.0000	1.0000	0.0000	0.0000	0.4000
0.0000	3.1429	0.4286	0.1429	0.4286	0.2857	0.0000	0.0000	0.2222	1.0000	0.0000	0.0000	0.2222
0.0000	3.0000	0.4000	0.1250	0.2500	0.2500	0.1250	0.0769	0.3077	1.0000	0.0000	0.0000	0.3846
0.0000	3.0000	0.4000	0.2000	0.2000	0.4000	0.0000	0.2222	0.2222	1.0000	0.0000	0.0000	0.4444
0.0000	3.0000	0.4000	0.1667	0.3333	0.3333	0.0000	0.1667	0.2500	1.0000	0.0000	0.0000	0.5000
0.0000	3.1667	0.4333	0.1429	0.2857	0.2857	0.1429	0.0000	0.0000	1.0000	0.0000	0.1250	0.0000
0.0000	3.0000	0.4000	0.1250	0.2500	0.2500	0.1250	0.1538	0.1538	1.0000	0.0000	0.0000	0.3846
0.0000	3.1667	0.4333	0.1667	0.3333	0.3333	0.0000	0.2500	0.0000	1.0000	0.0000	0.0000	0.5000
0.0000	3.1667	0.4333	0.1667	0.3333	0.3333	0.0000	0.2500	0.0833	1.0000	0.0000	0.0000	0.5000
0.0000	3.0000	0.4000	0.1667	0.1667	0.3333	0.1667	0.2000	0.2000	1.0000	0.0000	0.0667	0.5333
0.3636	3.2727	0.4545	0.1818	0.2727	0.4545	0.0000	0.0000	0.2500	1.0000	0.0000	0.0625	0.2500
0.3684	4.4444	0.6889	0.0000	0.6316	0.3684	0.0000	0.0000	0.1739	1.0000	0.0000	0.0000	0.1739
0.2000	2.5000	0.3000	0.1333	0.2667	0.2667	0.2000	0.1000	0.1667	0.9333	0.0667	0.2000	0.3000
0.2500	3.5833	0.5167	0.0833	0.5833	0.2500	0.0000	0.1111	0.2222	1.0000	0.0000	0.0000	0.3333
0.0000	2.7391	0.3478	0.0800	0.8000	0.0400	0.0800	0.0345	0.0690	1.0000	0.0000	0.0345	0.1034
0.0000	3.0000	0.4000	0.2000	0.4000	0.4000	0.0000	0.0000	0.0000	1.0000	0.0000	0.0000	0.0000
0.0000	3.8000	0.5600	0.2000	0.2000	0.4000	0.0000	0.3000	0.2000	1.0000	0.0000	0.0000	0.5000
0.2000	4.0000	0.6000	0.0000	0.2000	0.6000	0.0000	0.0000	0.2857	1.0000	0.0000	0.0000	0.2857
0.0000	3.2500	0.4500	0.5000	0.2500	0.2500	0.0000	0.1429	0.2857	1.0000	0.0000	0.0000	0.4286
0.0455	3.2273	0.4455	0.0000	0.8182	0.1818	0.0000	0.0000	0.0000	0.6364	0.3636	0.0000	0.0000

0.1176	2.9333	0.3867	0.0588	0.6471	0.1765	0.0588	0.0000	0.0556	0.9412	0.0000	0.0000	0.0556
0.5556	4.6667	0.7333	0.0000	0.4444	0.5556	0.0000	0.0000	0.1000	1.0000	0.0000	0.0000	0.1000
0.2222	3.2222	0.4444	0.3333	0.2222	0.3333	0.0000	0.0000	0.2500	0.8889	0.1111	0.0000	0.2500
0.3333	3.3333	0.4667	0.3333	0.2222	0.3333	0.0000	0.0833	0.0833	0.8889	0.1111	0.0833	0.1667
0.2222	3.2222	0.4444	0.1111	0.5556	0.3333	0.0000	0.0000	0.1818	0.8889	0.1111	0.0000	0.1818
0.3333	3.3333	0.4667	0.3333	0.0000	0.6667	0.0000	0.2000	0.2000	1.0000	0.0000	0.0000	0.4000
0.0000	4.0000	0.6000	0.0000	0.0000	0.6667	0.0000	0.2000	0.2000	1.0000	0.0000	0.0000	0.4000
0.1429	2.8462	0.3692	0.1429	0.5714	0.2143	0.0714	0.1000	0.1500	1.0000	0.0000	0.0000	0.3000
0.5000	4.5000	0.7000	0.0000	0.5000	0.5000	0.0000	0.0000	0.1667	1.0000	0.0000	0.0000	0.1667
0.1000	2.5000	0.3000	0.2000	0.2000	0.3000	0.2000	0.0769	0.3077	1.0000	0.0000	0.1923	0.4231
0.2308	3.6154	0.5231	0.0769	0.4615	0.4615	0.0000	0.0000	0.1176	1.0000	0.0000	0.1176	0.1176
0.0000	2.8333	0.3667	0.1250	0.3750	0.2500	0.2500	0.0625	0.1875	1.0000	0.0000	0.0625	0.4375
0.1818	2.3333	0.2667	0.0909	0.4545	0.1818	0.1818	0.0909	0.3030	0.8182	0.1818	0.1515	0.5152
0.0000	2.0000	0.2000	0.0000	0.6667	0.1667	0.0000	0.0000	0.0000	0.8333	0.0000	0.0000	0.0000
0.4375	4.0625	0.6125	0.0625	0.6875	0.2500	0.0000	0.0000	0.1579	0.9375	0.0625	0.0000	0.1579
0.2222	2.4444	0.2889	0.2222	0.3333	0.2222	0.0000	0.0800	0.3200	1.0000	0.0000	0.2400	0.4000
0.2000	3.6667	0.5333	0.1000	0.4000	0.3000	0.1000	0.0556	0.3333	1.0000	0.0000	0.0556	0.3889
0.0000	2.5714	0.3143	0.1176	0.5882	0.1176	0.1176	0.0000	0.1429	0.9412	0.0000	0.0000	0.1905
0.0526	3.1579	0.4316	0.1053	0.7368	0.1579	0.0000	0.0000	0.0000	1.0000	0.0000	0.0000	0.0000
0.3158	3.8947	0.5789	0.0526	0.6842	0.2632	0.0000	0.0000	0.1364	1.0000	0.0000	0.0000	0.1364
0.0000	3.2857	0.4571	0.1429	0.7143	0.1429	0.0000	0.0000	0.2222	1.0000	0.0000	0.0000	0.2222
0.0000	1.8571	0.1714	0.0000	0.8571	0.1429	0.0000	0.0000	0.0000	1.0000	0.0000	0.0000	0.0000
0.1500	3.3889	0.4778	0.0500	0.6000	0.2000	0.1000	0.0000	0.1429	1.0000	0.0000	0.1071	0.1786
0.1667	3.2188	0.4438	0.1389	0.5556	0.1389	0.0833	0.0204	0.1429	1.0000	0.0000	0.1020	0.1633
0.1250	2.9565	0.3913	0.2083	0.5000	0.1667	0.0417	0.0606	0.1818	1.0000	0.0000	0.0303	0.2424
0.1515	3.1935	0.4387	0.1818	0.5455	0.1515	0.0606	0.0000	0.2045	0.9697	0.0303	0.0455	0.2045
0.1724	3.2222	0.4444	0.1724	0.4828	0.1724	0.0690	0.0556	0.1111	1.0000	0.0000	0.0278	0.1667
0.2000	3.2727	0.4545	0.1714	0.5714	0.1714	0.0571	0.0233	0.1395	1.0000	0.0000	0.0233	0.1628
0.1538	3.4583	0.4917	0.0769	0.6154	0.1154	0.0769	0.0833	0.1667	1.0000	0.0000	0.0278	0.2500
0.2059	3.2727	0.4545	0.1765	0.5882	0.1176	0.0294	0.0244	0.1463	0.9412	0.0294	0.0000	0.1707
0.0800	3.0455	0.4091	0.1200	0.6000	0.1600	0.0800	0.0000	0.0000	1.0000	0.0000	0.0385	0.0000
0.1786	3.3462	0.4692	0.1429	0.6071	0.1786	0.0714	0.0313	0.0938	1.0000	0.0000	0.0000	0.1250
0.0870	3.0000	0.4000	0.1304	0.6087	0.1739	0.0870	0.0000	0.0000	0.9565	0.0435	0.0800	0.0000
0.1250	2.8000	0.3600	0.1875	0.6250	0.1250	0.0625	0.0000	0.0000	1.0000	0.0000	0.1111	0.0000
0.3125	4.3333	0.6667	0.0000	0.4375	0.5625	0.0000	0.0000	0.0000	0.9375	0.0625	0.0000	0.0000
0.2000	4.1429	0.6286	0.0000	0.4667	0.5333	0.0000	0.0000	0.0000	0.9333	0.0667	0.0000	0.0000
0.2000	4.2222	0.6444	0.0000	0.2000	0.8000	0.0000	0.0000	0.0000	1.0000	0.0000	0.0000	0.0000
0.1818	3.4211	0.4842	0.0909	0.5909	0.2727	0.0455	0.0000	0.0000	0.8636	0.1364	0.1538	0.0000
0.3077	4.0000	0.6000	0.0769	0.3846	0.5385	0.0000	0.0000	0.0000	0.8462	0.1538	0.0000	0.0000
0.0000	4.4000	0.6800	0.0000	0.2000	0.8000	0.0000	0.0000	0.0000	1.0000	0.0000	0.0000	0.0000
0.3529	4.3529	0.6706	0.0000	0.4706	0.5294	0.0000	0.0000	0.0000	1.0000	0.0000	0.0000	0.0000
0.2941	4.3750	0.6750	0.0000	0.5294	0.4706	0.0000	0.0000	0.0000	1.0000	0.0000	0.0000	0.0000
0.0833	4.4545	0.6909	0.0000	0.3333	0.6667	0.0000	0.0000	0.0000	0.9167	0.0833	0.0000	0.0000
0.0455	2.9091	0.3818	0.0455	0.7727	0.1818	0.0000	0.0000	0.0000	1.0000	0.0000	0.0833	0.0000
0.1000	2.9500	0.3900	0.0000	0.9000	0.1000	0.0000	0.0000	0.0000	1.0000	0.0000	0.0476	0.0000
0.1667	3.0000	0.4000	0.1667	0.6667	0.1667	0.0000	0.0000	0.0000	1.0000	0.0000	0.0000	0.0000
0.2500	4.1667	0.6333	0.0000	0.2500	0.7500	0.0000	0.0000	0.0000	1.0000	0.0000	0.0000	0.0000
0.2069	3.4138	0.4828	0.1379	0.5517	0.3103	0.0000	0.0000	0.0000	1.0000	0.0000	0.0000	0.0000
0.1500	3.6000	0.5200	0.2000	0.4500	0.3500	0.0000	0.0000	0.0000	0.9000	0.1000	0.0476	0.0000
0.0000	2.6667	0.3333	0.4000	0.4667	0.1333	0.0000	0.0000	0.0000	1.0000	0.0000	0.0000	0.0000
0.3158	4.3889	0.6778	0.0000	0.4211	0.5789	0.0000	0.0000	0.0000	1.0000	0.0000	0.0000	0.0000
0.4000	4.5000	0.7000	0.0000	0.4000	0.6000	0.0000	0.0000	0.0000	1.0000	0.0000	0.0000	0.0000
0.1163	3.5000	0.5000	0.0698	0.6047	0.2791	0.0465	0.0000	0.0000	0.9302	0.0698	0.0444	0.0000
0.0909	4.2727	0.6545	0.0000	0.5455	0.4545	0.0000	0.0000	0.0000	1.0000	0.0000	0.0000	0.0000
0.1111	3.5556	0.5111	0.1111	0.5556	0.3333	0.0000	0.0000	0.0000	1.0000	0.0000	0.0000	0.0000
0.0833	3.0000	0.4000	0.0833	0.7500	0.1667	0.0000	0.0000	0.0000	0.9167	0.0833	0.0000	0.0000
0.0000	2.5714	0.3143	0.1429	0.6429	0.2143	0.0000	0.0000	0.0000	0.8571	0.1429	0.0000	0.0000
0.0000	3.2500	0.4500	0.4000	0.6000	0.0000	0.0000	0.0000	0.0000	1.0000	0.0000	0.1667	0.0000
0.0769	3.0000	0.4000	0.1538	0.6154	0.1538	0.0769	0.0000	0.0000	0.9231	0.0769	0.0000	0.0000
0.0000	2.4615	0.2923	0.0769	0.7692	0.1538	0.0000	0.0000	0.0000	0.8462	0.1538	0.0000	0.0000
0.0000	2.2222	0.2444	0.0000	0.9000	0.0000	0.0000	0.0000	0.0000	0.9000	0.0000	0.0000	0.0000
0.0000	2.2857	0.2571	0.0714	0.9286	0.0000	0.0000	0.0000	0.0000	0.9286	0.0714	0.0000	0.0000
0.0000	2.3000	0.2600	0.1000	0.8000	0.1000	0.0000	0.0000	0.0000	1.0000	0.0000	0.0909	0.0000
0.0000	3.0000	0.4000	0.0000	0.8333	0.1667	0.0000	0.0000	0.0000	0.8333	0.1667	0.0000	0.0000
0.1667	4.3333	0.6667	0.1667	0.1667	0.6667	0.0000	0.0000	0.0000	1.0000	0.0000	0.0000	0.0000
0.0400	2.4167	0.2833	0.2000	0.6800	0.0800	0.0400	0.0000	0.0000	0.9200	0.0400	0.0385	0.0000
0.0000	2.3000	0.2600	0.0000	0.9000	0.1000	0.0000	0.0000	0.0000	0.8000	0.2000	0.0000	0.0000
0.0000	2.1667	0.2333	0.0000	0.8571	0.0000	0.0000	0.0000	0.0000	0.8571	0.0000	0.0000	0.0000
0.0909	3.7273	0.5455	0.0909	0.5455	0.3636	0.0000	0.0000	0.0000	0.8182	0.1818	0.0000	0.0000
0.1333	3.4000	0.4800	0.0667	0.6667	0.2667	0.0000	0.0000	0.0000	0.8667	0.1333	0.0000	0.0000
0.0769	3.3077	0.4615	0.0769	0.6154	0.3077	0.0000	0.0000	0.0000	0.8462	0.1538	0.0000	0.0000
0.0000	2.2500	0.2500	0.0000	0.8750	0.1250	0.0000	0.0000	0.0000	1.0000	0.0000	0.1111	0.0000
0.0000	2.2500	0.2500	0.0000	0.8750	0.1250	0.0000	0.0000	0.0000	1.0000	0.0000	0.1111	0.0000
0.0000	2.2500	0.2500	0.0000	1.0000	0.0000	0.0000	0.0000	0.0000	1.0000	0.0000	0.0000	0.0000
0.0000	2.4167	0.2833	0.1667	0.8333	0.0000	0.0000	0.0000	0.0000	0.8333	0.0833	0.0000	0.0000
0.0000	2.3333	0.2667	0.0000	1.0000	0.0000	0.0000	0.0000	0.0000	1.0000	0.0000	0.0000	0.0000
0.0000	2.3000	0.2600	0.2000	0.7000	0.1000	0.0000	0.0000	0.0000	0.9000	0.0000	0.0000	0.0000

0.1765	3.4118	0.4824	0.0588	0.7647	0.1765	0.0000	0.0000	0.0000	0.9412	0.0588	0.0000	0.0000
0.0000	3.2143	0.4429	0.0000	0.7143	0.2857	0.0000	0.0000	0.0000	0.7857	0.2143	0.0000	0.0000
0.0000	2.6154	0.3231	0.2222	0.5556	0.1852	0.0370	0.0000	0.0000	1.0000	0.0000	0.0690	0.0000
0.2353	4.1875	0.6375	0.0000	0.5294	0.4706	0.0000	0.0000	0.0000	0.8824	0.1176	0.0000	0.0000
0.3333	4.4167	0.6833	0.0000	0.2500	0.7500	0.0000	0.0000	0.0000	0.8333	0.1667	0.0000	0.0000
0.2941	3.6471	0.5294	0.0588	0.4706	0.4706	0.0000	0.0000	0.0000	0.8824	0.1176	0.0000	0.0000
0.2353	3.6471	0.5294	0.0000	0.6471	0.3529	0.0000	0.0000	0.0000	0.8824	0.1176	0.0000	0.0000
0.1250	4.1429	0.6286	0.0000	0.2500	0.7500	0.0000	0.0000	0.0000	1.0000	0.0000	0.0000	0.0000
0.1250	4.1250	0.6250	0.0000	0.1250	0.8750	0.0000	0.0000	0.0000	1.0000	0.0000	0.0000	0.0000
0.2727	4.2000	0.6400	0.0000	0.4545	0.5455	0.0000	0.0000	0.0000	0.9091	0.0909	0.0000	0.0000
0.0000	2.5556	0.3111	0.1111	0.6667	0.2222	0.0000	0.0000	0.0000	0.7778	0.1111	0.1818	0.0000
0.0769	2.4615	0.2923	0.3077	0.1538	0.2308	0.0000	0.0000	0.1765	1.0000	0.0000	0.0588	0.1765
0.0000	2.8750	0.3750	0.2500	0.2500	0.3750	0.0000	0.0000	0.1818	1.0000	0.0000	0.0909	0.1818
0.0000	2.6000	0.3200	0.2000	0.2000	0.4000	0.0000	0.0000	0.0000	1.0000	0.0000	0.1667	0.0000
0.0000	2.4444	0.2889	0.3333	0.1111	0.2222	0.0000	0.0000	0.2308	1.0000	0.0000	0.0769	0.2308
0.0000	2.6250	0.3250	0.2500	0.2500	0.2500	0.0000	0.0000	0.1667	1.0000	0.0000	0.1667	0.1667
0.0000	2.5714	0.3143	0.2857	0.1429	0.2857	0.0000	0.0000	0.2000	1.0000	0.0000	0.1000	0.2000
0.0000	2.6250	0.3250	0.2500	0.1250	0.2500	0.0000	0.0000	0.0000	1.0000	0.0000	0.0000	0.0000
0.0000	3.0000	0.4000	0.3333	0.3333	0.1667	0.0000	0.0000	0.3000	1.0000	0.0000	0.1000	0.3000
0.0000	2.5556	0.3111	0.3333	0.1111	0.2222	0.0000	0.0000	0.0909	1.0000	0.0000	0.0909	0.0909
0.0000	2.4444	0.2889	0.3333	0.1111	0.2222	0.0000	0.0000	0.1667	1.0000	0.0000	0.0833	0.1667
0.0000	2.2857	0.2571	0.4286	0.0000	0.2857	0.0000	0.0000	0.3846	1.0000	0.0000	0.0769	0.3846
0.0000	2.8750	0.3750	0.2500	0.2500	0.2500	0.0000	0.0000	0.2667	1.0000	0.0000	0.2000	0.2667
0.1000	2.7778	0.3556	0.3000	0.2000	0.1000	0.0000	0.0000	0.3529	0.9000	0.0000	0.0588	0.3529
0.0769	2.8333	0.3667	0.3077	0.2308	0.1538	0.0000	0.0000	0.2105	0.9231	0.0000	0.1053	0.2105
0.0000	2.4444	0.2889	0.2000	0.1000	0.3000	0.1000	0.0000	0.2500	0.9000	0.0000	0.0625	0.3125
0.0000	2.5000	0.3000	0.0000	0.0000	0.7500	0.0000	0.0000	0.3000	1.0000	0.0000	0.3000	0.3000
0.2500	2.7500	0.3500	0.0000	0.0000	0.7500	0.0000	0.0000	0.0000	1.0000	0.0000	0.3333	0.0000
0.0000	3.1667	0.4333	0.0000	0.3333	0.5000	0.0000	0.0000	0.2727	1.0000	0.0000	0.1818	0.2727
0.3333	2.8333	0.3667	0.1667	0.3333	0.3333	0.0000	0.0000	0.3333	1.0000	0.0000	0.0000	0.3333
0.0000	2.6250	0.3250	0.2500	0.1250	0.3750	0.0000	0.0000	0.0833	1.0000	0.0000	0.2500	0.0833
0.0000	2.7143	0.3429	0.2857	0.1429	0.2857	0.0000	0.0000	0.2222	1.0000	0.0000	0.0000	0.2222
0.0000	2.7143	0.3429	0.2857	0.2857	0.1429	0.1429	0.0000	0.3000	1.0000	0.0000	0.0000	0.3000
0.0000	2.4286	0.2857	0.4286	0.1429	0.1429	0.1429	0.0000	0.2727	1.0000	0.0000	0.0909	0.2727
0.0000	2.8571	0.3714	0.2500	0.2500	0.1250	0.1250	0.0000	0.3077	0.8750	0.0000	0.0769	0.3077
0.0909	2.8000	0.3600	0.2727	0.1818	0.1818	0.0000	0.0000	0.3182	0.9091	0.0000	0.0909	0.4091
0.0833	2.7273	0.3455	0.2500	0.2500	0.0833	0.0000	0.0000	0.2222	0.9167	0.0000	0.1111	0.2222
0.0000	2.8000	0.3600	0.3333	0.1667	0.1667	0.1667	0.0000	0.3333	0.8333	0.0000	0.0833	0.4167
0.0000	2.7143	0.3429	0.4286	0.1429	0.2857	0.0000	0.0000	0.2000	1.0000	0.0000	0.1000	0.2000
0.1111	2.3333	0.2667	0.2222	0.1111	0.1111	0.1111	0.0000	0.2857	1.0000	0.0000	0.0714	0.2857
0.0000	3.0000	0.4000	0.2000	0.2000	0.2000	0.0000	0.0000	0.4167	0.8000	0.0000	0.1667	0.4167
0.1250	2.8750	0.3750	0.1250	0.3750	0.2500	0.0000	0.0000	0.2727	1.0000	0.0000	0.0000	0.2727
0.0000	2.8000	0.3600	0.0000	0.2000	0.6000	0.0000	0.0000	0.3333	1.0000	0.0000	0.1111	0.3333
0.0000	3.2500	0.4500	0.2500	0.2500	0.5000	0.0000	0.0000	0.3333	1.0000	0.0000	0.0000	0.3333
0.2500	2.7500	0.3500	0.0000	0.2500	0.2500	0.0000	0.0000	0.2500	1.0000	0.0000	0.2500	0.2500
0.1667	2.6667	0.3333	0.0000	0.1667	0.5000	0.0000	0.0000	0.1429	1.0000	0.0000	0.0000	0.1429
0.1667	3.0000	0.4000	0.1667	0.1667	0.3333	0.0000	0.0000	0.2727	1.0000	0.0000	0.1818	0.2727
0.0000	2.7500	0.3500	0.3750	0.2500	0.1250	0.1250	0.0000	0.2727	1.0000	0.0000	0.0000	0.2727
0.1250	2.6250	0.3250	0.3750	0.1250	0.1250	0.1250	0.0000	0.2857	1.0000	0.0000	0.0000	0.4286
0.0000	2.5714	0.3143	0.4286	0.1429	0.1429	0.0000	0.0000	0.2727	1.0000	0.0000	0.0909	0.2727
0.0000	2.0909	0.2182	0.3333	0.0833	0.0833	0.0833	0.0000	0.2353	0.9167	0.0000	0.0588	0.2353
0.0000	2.4000	0.2800	0.2000	0.2000	0.2000	0.0000	0.0000	0.2222	1.0000	0.0000	0.2222	0.2222
0.1111	2.4444	0.2889	0.3333	0.1111	0.1111	0.1111	0.0000	0.2308	1.0000	0.0000	0.0769	0.2308
0.1250	2.6250	0.3250	0.3750	0.1250	0.1250	0.1250	0.0000	0.3077	1.0000	0.0000	0.0000	0.3846
0.1538	2.3077	0.2615	0.3077	0.0769	0.0769	0.0769	0.0000	0.2727	1.0000	0.0000	0.0909	0.3182
0.2222	2.4444	0.2889	0.2222	0.1111	0.1111	0.1111	0.0000	0.2667	1.0000	0.0000	0.1333	0.2667
0.0000	2.5714	0.3143	0.2857	0.1429	0.2857	0.1429	0.0000	0.1250	1.0000	0.0000	0.0000	0.1250
0.0000	2.8000	0.3600	0.4000	0.2000	0.2000	0.2000	0.0000	0.2500	1.0000	0.0000	0.1250	0.2500
0.0000	2.8000	0.3600	0.0000	0.2000	0.6000	0.2000	0.0000	0.2857	1.0000	0.0000	0.0000	0.2857
0.1250	2.6250	0.3250	0.2500	0.1250	0.2500	0.0000	0.0000	0.2000	1.0000	0.0000	0.0000	0.2000
0.0000	2.6667	0.3333	0.3333	0.0000	0.3333	0.0000	0.0000	0.3333	1.0000	0.0000	0.0833	0.4167
0.0000	2.7143	0.3429	0.2857	0.1429	0.2857	0.1429	0.0000	0.3333	1.0000	0.0000	0.0000	0.4167
0.0000	2.1250	0.2250	0.1250	0.1250	0.1250	0.1250	0.0000	0.3077	1.0000	0.0000	0.0769	0.3077
0.1000	2.1111	0.2222	0.3000	0.0000	0.1000	0.0000	0.0000	0.2667	0.9000	0.0000	0.0667	0.2667
0.0000	2.1667	0.2333	0.3333	0.0000	0.1667	0.1667	0.0000	0.4000	1.0000	0.0000	0.0000	0.4000
0.0000	2.6667	0.3333	0.3333	0.1667	0.1667	0.1667	0.0000	0.2222	1.0000	0.0000	0.1111	0.2222
0.0000	2.6667	0.3333	0.2857	0.1429	0.1429	0.0000	0.0000	0.2500	0.8571	0.0000	0.1667	0.2500
0.1429	2.7143	0.3429	0.2857	0.1429	0.1429	0.1429	0.0000	0.3077	1.0000	0.0000	0.1538	0.3077
0.0000	2.6667	0.3333	0.3333	0.1667	0.1667	0.1667	0.0000	0.3000	1.0000	0.0000	0.1000	0.3000
0.1111	2.4444	0.2889	0.3333	0.1111	0.1111	0.1111	0.0000	0.2308	1.0000	0.0000	0.0769	0.2308
0.0000	2.2500	0.2500	0.3750	0.0000	0.2500	0.1250	0.0000	0.3077	1.0000	0.0000	0.0769	0.3077
0.0000	4.0000	0.6000	0.0000	0.5000	0.5000	0.0000	0.0000	0.6000	1.0000	0.0000	0.0000	0.6000
0.2000	3.4000	0.4800	0.0000	0.2000	0.8000	0.0000	0.0000	0.0000	1.0000	0.0000	0.1667	0.0000
0.0000	3.3333	0.4667	0.0000	0.3333	0.6667	0.0000	0.0000	0.2500	1.0000	0.0000	0.0000	0.2500
0.0000	3.2500	0.4500	0.0000	0.2500	0.7500	0.0000	0.0000	0.2000	1.0000	0.0000	0.0000	0.2000
0.0000	2.8333	0.3667	0.1667	0.3333	0.1667	0.1667	0.0000	0.3333	1.0000	0.0000	0.0000	0.3333
0.0000	4.0000	0.6000	0.0000	0.5000	0.5000	0.0000	0.0000	0.0000	1.0000	0.0000	0.0000	0.0000

0.0000	4.0000	0.6000	0.0000	0.5000	0.5000	0.0000	0.0000	0.4286	1.0000	0.0000	0.1429	0.5714
0.5000	3.0000	0.4000	0.0000	0.0000	0.5000	0.0000	0.0000	0.3333	1.0000	0.0000	0.0000	0.3333
0.0000	3.7500	0.5500	0.0000	0.5000	0.5000	0.0000	0.0000	0.1667	1.0000	0.0000	0.1667	0.1667
0.0000	3.3333	0.4667	0.0000	0.3333	0.6667	0.0000	0.0000	0.0000	1.0000	0.0000	0.0000	0.0000
0.2000	3.0000	0.4000	0.0000	0.2000	0.6000	0.0000	0.0000	0.3000	1.0000	0.0000	0.1000	0.4000
0.2000	2.6000	0.3200	0.0000	0.4000	0.2000	0.0000	0.0000	0.4444	1.0000	0.0000	0.0000	0.4444
0.0000	4.0000	0.6000	0.0000	0.5000	0.5000	0.0000	0.0000	0.3333	1.0000	0.0000	0.0000	0.3333
0.1250	3.0000	0.4000	0.1250	0.5000	0.2500	0.0000	0.0000	0.1818	1.0000	0.0000	0.0909	0.1818
0.0000	2.8571	0.3714	0.0000	0.4286	0.2857	0.1429	0.0000	0.2727	1.0000	0.0000	0.0909	0.2727
0.3333	3.3333	0.4667	0.0000	0.5000	0.5000	0.0000	0.0000	0.0000	1.0000	0.0000	0.0000	0.0000
0.2500	2.5000	0.3000	0.0000	0.5000	0.5000	0.0000	0.0000	0.2000	1.0000	0.0000	0.0000	0.2000
0.1667	2.4167	0.2833	0.2500	0.2500	0.1667	0.0833	0.0000	0.0714	1.0000	0.0000	0.0714	0.0714
0.0909	2.7273	0.3455	0.1818	0.3636	0.1818	0.0909	0.0000	0.0769	1.0000	0.0000	0.0769	0.0769
0.0000	2.5000	0.3000	0.0000	0.2500	0.2500	0.2500	0.0000	0.2857	1.0000	0.0000	0.1429	0.2857
0.0000	3.0000	0.4000	0.0000	0.3333	0.3333	0.0000	0.0000	0.3000	0.3333	0.3333	0.4000	0.3000
0.0000	3.0000	0.4000	0.0000	0.3333	0.3333	0.0000	0.0000	0.4444	0.3333	0.3333	0.1111	0.5556
0.5000	3.0000	0.4000	0.0000	0.0000	0.5000	0.0000	0.0000	0.0000	1.0000	0.0000	0.5000	0.0000
0.2500	2.0000	0.2000	0.2500	0.2500	0.2500	0.0000	0.0000	0.3000	0.7500	0.2500	0.3000	0.3000
0.2500	2.3333	0.2667	0.0000	0.2500	0.2500	0.0000	0.0000	0.3333	0.7500	0.0000	0.0000	0.3333
0.0000	2.0000	0.2000	0.0000	0.0000	0.3333	0.0000	0.0000	0.2222	0.6667	0.0000	0.3333	0.3333
0.0000	3.5000	0.5000	0.0000	0.0000	1.0000	0.0000	0.0000	0.2500	1.0000	0.0000	0.0000	0.5000
0.0000	2.0000	0.2000	0.0000	0.5000	0.0000	0.0000	0.0000	0.3333	0.0000	0.5000	0.3333	0.3333
0.3333	3.3333	0.4667	0.0000	0.3333	0.3333	0.0000	0.0000	0.0000	1.0000	0.0000	0.2500	0.0000
0.0000	4.0000	0.6000	0.0000	0.0000	0.5000	0.0000	0.0000	0.4000	0.5000	0.0000	0.2000	0.4000
0.0000	4.0000	0.6000	0.0000	0.0000	1.0000	0.0000	0.0000	0.2500	1.0000	0.0000	0.2500	0.5000
0.2000	3.0000	0.4000	0.2000	0.2000	0.4000	0.0000	0.0000	0.0909	1.0000	0.0000	0.3636	0.1818
1.0000	2.0000	0.2000	0.0000	0.0000	0.0000	0.0000	0.0000	0.6000	1.0000	0.0000	0.2000	0.6000
0.3333	3.0000	0.4000	0.0000	0.0000	0.3333	0.0000	0.0000	0.1667	0.6667	0.0000	0.3333	0.1667
0.0000	3.0000	0.4000	0.0000	0.2000	0.4000	0.0000	0.0000	0.3846	0.8000	0.0000	0.2308	0.3846
0.0000	1.2500	0.0500	0.0000	0.0000	0.2500	0.2500	0.0000	0.1667	1.0000	0.0000	0.1667	0.1667
0.0000	3.0000	0.4000	0.0000	0.0000	1.0000	0.0000	0.0000	0.2857	1.0000	0.0000	0.2857	0.4286
0.0000	4.0000	0.6000	0.0000	0.5000	0.5000	0.0000	0.0000	0.0000	1.0000	0.0000	0.3333	0.0000
0.0000	4.0000	0.6000	0.0000	0.0000	1.0000	0.0000	0.0000	0.0000	1.0000	0.0000	0.0000	0.0000
1.0000	2.0000	0.2000	0.0000	0.6667	0.0000	0.0000	0.0000	0.2000	1.0000	0.0000	0.2000	0.2000
0.5000	3.0000	0.4000	0.0000	0.0000	0.5000	0.0000	0.0000	0.2000	1.0000	0.0000	0.4000	0.2000
1.0000	2.0000	0.2000	0.0000	1.0000	0.0000	0.0000	0.0000	0.2000	1.0000	0.0000	0.6000	0.2000
1.0000	2.0000	0.2000	0.0000	0.0000	0.0000	0.0000	0.0000	0.7500	1.0000	0.0000	0.0000	0.7500
0.3333	3.0000	0.4000	0.0000	0.0000	0.6667	0.0000	0.0000	0.3333	1.0000	0.0000	0.0000	0.5000
1.0000	2.0000	0.2000	0.0000	0.0000	0.0000	0.0000	0.0000	0.6667	1.0000	0.0000	0.0000	0.6667
0.0000	3.0000	0.4000	0.0000	0.0000	0.5000	0.0000	0.0000	0.5000	0.5000	0.0000	0.0000	0.5000
0.0000	2.0000	0.2000	0.0000	0.3333	0.6667	0.0000	0.0000	0.2000	1.0000	0.0000	0.2000	0.2000
0.0000	3.0000	0.4000	0.0000	0.0000	1.0000	0.0000	0.0000	0.6250	1.0000	0.0000	0.1250	0.7500
0.2000	2.2000	0.2400	0.0000	0.2000	0.6000	0.0000	0.0000	0.2222	1.0000	0.0000	0.1111	0.3333
0.2222	2.7778	0.3556	0.0000	0.3333	0.4444	0.0000	0.0000	0.3077	1.0000	0.0000	0.0000	0.3077
0.5000	3.7500	0.5500	0.0000	0.2500	0.7500	0.0000	0.0000	0.3636	1.0000	0.0000	0.1818	0.4545
0.5000	4.0000	0.6000	0.0000	0.0000	1.0000	0.0000	0.0000	0.3333	1.0000	0.0000	0.0000	0.3333
0.0000	3.5000	0.5000	0.0000	0.0000	1.0000	0.0000	0.0000	0.5000	1.0000	0.0000	0.1250	0.6250
0.0000	3.0000	0.4000	0.0000	0.0000	1.0000	0.0000	0.0000	0.6364	1.0000	0.0000	0.0000	0.7273
0.3333	2.3333	0.2667	0.0000	0.0000	0.6667	0.0000	0.0000	0.4444	1.0000	0.0000	0.2222	0.4444
0.3333	2.6667	0.3333	0.0000	0.3333	0.3333	0.0000	0.0000	0.5000	1.0000	0.0000	0.0000	0.5000
							0.0000	0.7500			0.0000	1.0000
0.1429	2.3333	0.2667	0.0000	0.2857	0.4286	0.1429	0.0000	0.0000	0.8571	0.1429	0.1250	0.0000
0.3333	2.6000	0.3200	0.1667	0.0000	0.5000	0.1667	0.0769	0.3077	1.0000	0.0000	0.1538	0.3846
0.2000	2.5000	0.3000	0.4000	0.0000	0.4000	0.2000	0.0000	0.4444	1.0000	0.0000	0.0000	0.4444
0.1250	2.2857	0.2571	0.1250	0.1250	0.5000	0.1250	0.0000	0.2857	1.0000	0.0000	0.1429	0.2857
0.1111	2.1429	0.2286	0.4444	0.1111	0.2222	0.2222	0.0952	0.2381	1.0000	0.0000	0.1429	0.4286
0.1111	2.1250	0.2250	0.3333	0.1111	0.2222	0.2222	0.0625	0.1875	1.0000	0.0000	0.1875	0.2500
0.1667	2.1667	0.2333	0.3333	0.3333	0.1667	0.1667	0.1429	0.2857	1.0000	0.0000	0.1429	0.4286
0.2500	2.6667	0.3333	0.2500	0.0000	0.2500	0.2500	0.1250	0.1250	1.0000	0.0000	0.2500	0.2500
0.0000	3.0000	0.4000	0.3333	0.0000	0.3333	0.3333	0.3333	0.1111	1.0000	0.0000	0.2222	0.4444
0.3333	2.0000	0.2000	0.3333	0.0000	0.3333	0.0000	0.2000	0.3000	1.0000	0.0000	0.2000	0.5000
0.3333	2.0000	0.2000	0.3333	0.0000	0.3333	0.0000	0.2500	0.1667	1.0000	0.0000	0.2500	0.5000
0.0000	2.6667	0.3333	0.3333	0.3333	0.3333	0.0000	0.3333	0.1111	1.0000	0.0000	0.2222	0.4444
1.0000	2.5000	0.3000	0.0000	0.0000	0.0000	0.0000	0.2222	0.3333	1.0000	0.0000	0.2222	0.5556
0.0000	2.0000	0.2000	0.2500	0.0000	0.2500	0.2500	0.2667	0.2667	1.0000	0.0000	0.2000	0.5333
0.0000	1.8333	0.1667	0.3333	0.1667	0.1667	0.0000	0.1818	0.1818	1.0000	0.0000	0.0909	0.3636
0.0000	2.0000	0.2000	0.2500	0.0000	0.2500	0.0000	0.2000	0.3333	0.7500	0.0000	0.1333	0.6000
0.3333	2.6667	0.3333	0.1667	0.3333	0.5000	0.0000	0.0000	0.5000	1.0000	0.0000	0.0714	0.5000
0.3000	2.7000	0.3400	0.3000	0.3000	0.3000	0.0000	0.0526	0.2632	1.0000	0.0000	0.1579	0.3158
0.4000	2.6000	0.3200	0.2000	0.4000	0.4000	0.0000	0.1053	0.4737	1.0000	0.0000	0.1053	0.6316
0.1667	2.3333	0.2667	0.3333	0.0000	0.3333	0.0000	0.1818	0.0909	1.0000	0.0000	0.1818	0.2727
0.1250	2.2500	0.2500	0.3750	0.1250	0.2500	0.0000	0.2941	0.1765	1.0000	0.0000	0.0588	0.4706
0.0000	2.5000	0.3000	0.5000	0.0000	0.5000	0.0000	0.2500	0.2500	1.0000	0.0000	0.2500	0.5000
0.0000	2.0000	0.2000	0.7500	0.0000	0.2500	0.0000	0.4667	0.2000	1.0000	0.0000	0.0667	0.6667
0.0000	2.0000	0.2000	0.6667	0.0000	0.3333	0.0000	0.4286	0.2857	1.0000	0.0000	0.0714	0.7143
0.0000	2.0000	0.2000	0.6667	0.0000	0.3333	0.0000	0.3529	0.2941	1.0000	0.0000	0.1176	0.7059
0.3333	2.3333	0.2667	0.3333	0.0000	0.3333	0.0000	0.3000	0.2000	1.0000	0.0000	0.2000	0.5000

0.0000	2.5000	0.3000	0.5000	0.0000	0.5000	0.0000	0.2222	0.3333	1.0000	0.0000	0.2222	0.5556
0.0000	3.0000	0.4000	0.0000	0.0000	1.0000	0.0000	0.5833	0.1667	1.0000	0.0000	0.1667	0.7500
0.0000	1.6667	0.1333	0.3333	0.3333	0.0000	0.0000	0.4545	0.0909	1.0000	0.0000	0.1818	0.5455
0.0000	2.0000	0.2000	0.3333	0.0000	0.3333	0.0000	0.4615	0.1538	1.0000	0.0000	0.1538	0.6154
0.0000	2.0000	0.2000	1.0000	0.0000	0.0000	0.0000	0.4444	0.2222	1.0000	0.0000	0.2222	0.6667
0.1111	2.8750	0.3750	0.2222	0.4444	0.2222	0.1111	0.0000	0.1818	1.0000	0.0000	0.0000	0.1818
0.0909	2.5556	0.3111	0.3636	0.3636	0.1818	0.0909	0.0526	0.2632	1.0000	0.0000	0.0526	0.3684
0.0909	2.5000	0.3000	0.3636	0.3636	0.1818	0.0909	0.0000	0.0833	1.0000	0.0000	0.0000	0.0833
0.0000	1.6667	0.1333	0.2000	0.2000	0.4000	0.2000	0.0833	0.3333	1.0000	0.0000	0.1667	0.4167
0.2500	2.6667	0.3333	0.2500	0.0000	0.7500	0.0000	0.0909	0.2727	1.0000	0.0000	0.1818	0.4545
0.0000	2.2000	0.2400	0.1667	0.5000	0.3333	0.0000	0.1333	0.2000	1.0000	0.0000	0.1333	0.4667
0.2000	2.2000	0.2400	0.3000	0.2000	0.4000	0.0000	0.2593	0.1852	1.0000	0.0000	0.0741	0.5556
0.1429	2.1429	0.2286	0.4286	0.0000	0.2857	0.0000	0.3333	0.1333	1.0000	0.0000	0.0667	0.4667
0.1250	2.3750	0.2750	0.3750	0.3750	0.2500	0.0000	0.1333	0.1333	1.0000	0.0000	0.2000	0.2667
0.1000	2.0000	0.2000	0.4000	0.3000	0.2000	0.0000	0.0625	0.1875	1.0000	0.0000	0.1250	0.2500
0.0909	2.0000	0.2000	0.2727	0.3636	0.1818	0.0000	0.2500	0.1667	1.0000	0.0000	0.1250	0.4167
0.0000	2.0000	0.2000	0.4286	0.2857	0.2857	0.0000	0.2353	0.1765	1.0000	0.0000	0.1176	0.4706
0.0000	2.1429	0.2286	0.4286	0.2857	0.2857	0.0000	0.2500	0.1250	1.0000	0.0000	0.1875	0.3750
0.1429	2.5714	0.3143	0.2857	0.4286	0.2857	0.0000	0.1538	0.4231	1.0000	0.0000	0.1154	0.6154
0.1000	2.4444	0.2889	0.4000	0.3000	0.3000	0.0000	0.1667	0.3333	1.0000	0.0000	0.0833	0.5000
0.1000	2.7778	0.3556	0.3000	0.4000	0.2000	0.0000	0.0000	0.3333	1.0000	0.0000	0.1429	0.3810
0.0909	2.7000	0.3400	0.0909	0.6364	0.2727	0.0000	0.0000	0.3333	1.0000	0.0000	0.0000	0.3889
0.0714	2.7273	0.3455	0.2143	0.5000	0.2143	0.0714	0.0000	0.2632	1.0000	0.0000	0.0000	0.2632
0.0833	2.8000	0.3600	0.1667	0.5000	0.2500	0.0000	0.0000	0.3636	0.9167	0.0000	0.0455	0.4091
0.1000	2.7778	0.3556	0.2000	0.5000	0.3000	0.0000	0.0500	0.4000	1.0000	0.0000	0.0000	0.5000
0.0833	2.6364	0.3273	0.2500	0.5000	0.2500	0.0000	0.0000	0.3182	1.0000	0.0000	0.0455	0.4091
0.1000	2.4444	0.2889	0.3000	0.4000	0.3000	0.0000	0.0588	0.2353	1.0000	0.0000	0.0588	0.3529
0.0769	2.6364	0.3273	0.2308	0.4615	0.2308	0.0769	0.0800	0.3200	1.0000	0.0000	0.0400	0.4400
0.0769	2.5455	0.3091	0.3077	0.3846	0.2308	0.0769	0.0909	0.2273	1.0000	0.0000	0.0455	0.3636
0.0909	2.3000	0.2600	0.2727	0.3636	0.2727	0.0000	0.0476	0.2857	1.0000	0.0000	0.0952	0.3810
0.1111	2.5000	0.3000	0.2222	0.4444	0.2222	0.0000	0.0952	0.2857	1.0000	0.0000	0.1429	0.4286
0.0909	2.5000	0.3000	0.1818	0.4545	0.2727	0.0000	0.0833	0.3333	1.0000	0.0000	0.0833	0.4583
0.0000	2.0000	0.2000	0.4286	0.2857	0.2857	0.0000	0.1053	0.3158	1.0000	0.0000	0.1579	0.4737
0.1667	2.6667	0.3333	0.3333	0.3333	0.3333	0.0000	0.0526	0.4211	1.0000	0.0000	0.1579	0.5263
0.0000	2.1429	0.2286	0.4286	0.2857	0.2857	0.0000	0.4118	0.0588	1.0000	0.0000	0.1176	0.4706
0.0000	2.0000	0.2000	0.6667	0.3333	0.0000	0.0000	0.0000	0.0000	1.0000	0.0000	0.2500	0.0000
0.0000	2.6000	0.3200	0.4000	0.2000	0.2000	0.2000	0.0909	0.2727	1.0000	0.0000	0.1818	0.3636
0.3333	1.6667	0.1333	0.0000	0.3333	0.3333	0.0000	0.3571	0.4286	1.0000	0.0000	0.0000	0.7857
0.2000	2.4000	0.2800	0.0000	0.4000	0.4000	0.0000	0.4000	0.0000	1.0000	0.0000	0.1000	0.4000
0.0000	NA	NA	1.0000	0.0000	0.0000	0.0000	0.4286	0.1429	1.0000	0.0000	0.2857	0.5714
0.2500	2.5000	0.3000	0.2500	0.5000	0.0000	0.0000	0.0000	0.0000	1.0000	0.0000	0.2000	0.0000
0.2857	2.2857	0.2571	0.2857	0.2857	0.0000	0.1429	0.0000	0.4118	1.0000	0.0000	0.1176	0.4706
0.1667	2.1667	0.2333	0.1667	0.3333	0.1667	0.1667	0.2143	0.2857	1.0000	0.0000	0.0714	0.5000
0.5000	1.5000	0.1000	0.0000	0.0000	0.0000	0.0000	0.3333	0.3333	1.0000	0.0000	0.0000	0.6667
							0.6667	0.3333			0.0000	1.0000
0.2500	2.5000	0.3000	0.2500	0.0000	0.2500	0.2500	0.0000	0.4000	1.0000	0.0000	0.1000	0.5000
0.5000	1.5000	0.1000	0.0000	0.0000	0.0000	0.5000	0.0000	0.2500	1.0000	0.0000	0.2500	0.2500
0.3333	2.1667	0.2333	0.1667	0.0000	0.1667	0.1667	0.0000	0.3000	1.0000	0.0000	0.0000	0.4000
0.0000	2.0000	0.2000	0.0000	0.0000	0.5000	0.5000	0.1667	0.5000	1.0000	0.0000	0.0000	0.6667
0.0000	2.0000	0.2000	0.3333	0.0000	0.3333	0.3333	0.2857	0.1429	1.0000	0.0000	0.1429	0.4286
0.0000	1.0000	0.0000	0.0000	0.0000	0.0000	1.0000	0.0000	0.5000	1.0000	0.0000	0.2500	0.5000
0.0000	1.0000	0.0000	0.0000	0.0000	0.0000	1.0000	0.0000	0.6000	1.0000	0.0000	0.2000	0.6000
0.5000	2.5000	0.3000	0.0000	0.0000	0.5000	0.0000	0.0000	0.3333	1.0000	0.0000	0.0000	0.3333
0.0000	3.0000	0.4000	1.0000	0.0000	0.0000	0.0000	0.0000	0.7500	1.0000	0.0000	0.0000	0.7500
0.0000	1.0000	0.0000	0.0000	0.0000	0.0000	1.0000	0.5000	0.3333	1.0000	0.0000	0.0000	0.8333
0.0000	1.6667	0.1333	0.3333	0.0000	0.3333	0.3333	0.0000	0.3750	1.0000	0.0000	0.1250	0.5000
0.1429	2.5714	0.3143	0.4286	0.1429	0.2857	0.1429	0.1111	0.3889	1.0000	0.0000	0.1111	0.5000
0.3333	2.0000	0.2000	0.1667	0.0000	0.1667	0.1667	0.2222	0.0000	1.0000	0.0000	0.1111	0.2222
0.3333	2.0000	0.2000	0.0000	0.3333	0.3333	0.3333	0.1818	0.3636	1.0000	0.0000	0.0909	0.6364
							0.0000	1.0000			0.0000	1.0000
0.0000	1.7500	0.1500	0.5000	0.2500	0.2500	0.0000	0.0000	0.3750	1.0000	0.0000	0.1250	0.3750
0.1111	2.7500	0.3500	0.0000	0.5556	0.3333	0.1111	0.0455	0.4545	1.0000	0.0000	0.0000	0.5909
0.1250	2.2500	0.2500	0.0000	0.3750	0.3750	0.1250	0.0833	0.2500	1.0000	0.0000	0.0000	0.3333
0.1429	2.4286	0.2857	0.2857	0.4286	0.2857	0.0000	0.1176	0.3529	1.0000	0.0000	0.0000	0.5882
0.0000	2.0000	0.2000	0.0000	1.0000	0.0000	0.0000	0.1667	0.5000	1.0000	0.0000	0.0000	0.6667
0.1111	2.4286	0.2857	0.4444	0.2222	0.2222	0.1111	0.0000	0.3333	1.0000	0.0000	0.0000	0.4000
0.1000	2.4444	0.2889	0.3000	0.4000	0.2000	0.0000	0.1250	0.1875	1.0000	0.0000	0.0625	0.3125
0.2000	2.6667	0.3333	0.2000	0.4000	0.2000	0.0000	0.2941	0.0588	1.0000	0.0000	0.0588	0.3529
0.1818	2.4545	0.2909	0.1818	0.3636	0.1818	0.0909	0.2273	0.1364	1.0000	0.0000	0.1364	0.3636
0.0000	1.6667	0.1333	0.1667	0.0000	0.1667	0.1667	0.3571	0.1429	1.0000	0.0000	0.0714	0.5000
0.0909	2.7778	0.3556	0.1818	0.3636	0.3636	0.0909	0.0000	0.0833	1.0000	0.0000	0.0000	0.0833
0.2000	2.2000	0.2400	0.2000	0.3000	0.2000	0.0000	0.0476	0.3810	1.0000	0.0000	0.0952	0.4286
0.3333	2.3333	0.2667	0.3333	0.0000	0.3333	0.0000	0.1429	0.2857	1.0000	0.0000	0.1429	0.4286
0.2500	2.3333	0.2667	0.5000	0.0000	0.2500	0.0000	0.5385	0.1538	1.0000	0.0000	0.0000	0.6923
0.2000	1.5000	0.1000	0.4000	0.0000	0.0000	0.0000	0.3846	0.1538	1.0000	0.0000	0.0769	0.5385
0.0000	1.5000	0.1000	0.6667	0.0000	0.0000	0.3333	0.0000	0.0000	1.0000	0.0000	0.2500	0.0000
0.2222	2.0000	0.2000	0.3333	0.1111	0.1111	0.1111	0.0000	0.3333	1.0000	0.0000	0.0000	0.4000

0.2000	1.8000	0.1600	0.2000	0.2000	0.2000	0.0000	0.0000	0.3636	1.0000	0.0000	0.1818	0.3636
0.2500	2.0000	0.2000	0.5000	0.0000	0.0000	0.0000	0.0000	0.5000	1.0000	0.0000	0.1000	0.5000
1.0000	2.0000	0.2000	0.0000	0.0000	0.0000	0.0000	0.0000	0.3333	1.0000	0.0000	0.3333	0.3333
0.6667	2.0000	0.2000	0.0000	0.0000	0.0000	0.3333	0.0000	0.4286	1.0000	0.0000	0.1429	0.4286
0.5000	1.7500	0.1500	0.2500	0.0000	0.0000	0.2500	0.0000	0.0000	1.0000	0.0000	0.0000	0.2000
0.2500	2.0000	0.2000	0.2500	0.0000	0.0000	0.2500	0.0000	0.2000	1.0000	0.0000	0.0000	0.2000
1.0000	3.6667	0.5333	0.0000	0.0000	0.6667	0.0000	0.0000	0.5000	1.0000	0.0000	0.1000	0.6000
							0.0000	0.0000			1.0000	0.0000
							0.0000	0.6667			0.3333	0.6667
0.5000	2.0000	0.2000	0.2500	0.0000	0.0000	0.2500	0.0000	0.4286	1.0000	0.0000	0.0000	0.4286
0.3333	2.0000	0.2000	0.0000	0.0000	0.3333	0.3333	0.0000	0.2500	1.0000	0.0000	0.0000	0.2500
1.0000	2.5000	0.3000	0.0000	0.0000	0.0000	0.0000	0.0000	0.5000	1.0000	0.0000	0.0000	0.5000
0.5000	1.5000	0.1000	0.0000	0.5000	0.0000	0.0000	0.0909	0.4545	1.0000	0.0000	0.1818	0.6364
0.5000	2.0000	0.2000	0.0000	0.0000	0.0000	0.5000	0.0000	0.5000	1.0000	0.0000	0.1250	0.6250
0.5000	2.0000	0.2000	0.0000	0.0000	0.0000	0.5000	0.1667	0.3333	1.0000	0.0000	0.1667	0.5000
0.4000	2.0000	0.2000	0.0000	0.0000	0.2000	0.2000	0.2222	0.2222	1.0000	0.0000	0.0000	0.4444
0.2500	2.0000	0.2000	0.0000	0.2500	0.1250	0.1250	0.1538	0.2308	1.0000	0.0000	0.0000	0.3846
							0.0000	0.6667			0.1667	0.8333
0.2963	4.3913	0.6783	0.0370	0.5926	0.2963	0.0741	0.0000	0.0000	0.9630	0.0370	0.0000	0.0000
0.2963	4.2500	0.6500	0.0370	0.5926	0.2963	0.0741	0.0000	0.0000	0.9259	0.0741	0.0000	0.0000
0.2500	4.2083	0.6417	0.0000	0.6071	0.3214	0.0714	0.0000	0.0000	0.9286	0.0714	0.0000	0.0000
0.3235	4.0667	0.6133	0.0294	0.6471	0.2647	0.0588	0.0000	0.0000	0.9412	0.0588	0.0000	0.0000
0.2917	4.2381	0.6476	0.0417	0.6250	0.2500	0.0833	0.0000	0.0000	0.9583	0.0417	0.0000	0.0000
0.2917	4.0476	0.6095	0.0417	0.7083	0.1667	0.0833	0.0000	0.0000	1.0000	0.0000	0.0000	0.0000
0.2400	3.5455	0.5091	0.0400	0.6000	0.2800	0.0800	0.0000	0.0000	0.8800	0.1200	0.0000	0.0000
0.2400	3.8182	0.5636	0.0400	0.6800	0.2000	0.0800	0.0000	0.0000	0.9600	0.0400	0.0000	0.0000
0.2381	3.7368	0.5474	0.0952	0.5714	0.2381	0.0952	0.0000	0.0000	0.9524	0.0476	0.0000	0.0000
0.2500	3.8000	0.5600	0.0357	0.7143	0.1786	0.0714	0.0000	0.0000	0.9286	0.0357	0.0000	0.0000
0.1786	3.8000	0.5600	0.0714	0.6786	0.1786	0.0714	0.0000	0.0000	0.9286	0.0357	0.0000	0.0000
0.1667	3.6786	0.5357	0.0667	0.5667	0.3000	0.0667	0.0000	0.0000	0.9000	0.0667	0.0000	0.0000
0.1724	3.6296	0.5259	0.0345	0.7241	0.1724	0.0690	0.0000	0.0000	0.9310	0.0345	0.0000	0.0000
0.2424	3.7097	0.5419	0.1212	0.6061	0.2121	0.0606	0.0000	0.0000	0.9394	0.0303	0.0000	0.0000
0.2258	3.7500	0.5500	0.0645	0.6129	0.2581	0.0645	0.0000	0.0000	0.8710	0.0968	0.0000	0.0000
0.2857	3.9500	0.5900	0.0476	0.6667	0.2857	0.0000	0.0000	0.0000	0.8571	0.1429	0.0000	0.0000
0.3333	3.9200	0.5840	0.0370	0.7037	0.2222	0.0370	0.0000	0.0000	0.9630	0.0370	0.0000	0.0000
0.2400	4.0000	0.6000	0.0400	0.6800	0.2800	0.0000	0.0000	0.0000	0.8800	0.1200	0.0000	0.0000
0.3077	3.6000	0.5200	0.0385	0.7308	0.2308	0.0000	0.0000	0.0000	0.8846	0.1154	0.0000	0.0000
0.2105	3.5556	0.5111	0.0526	0.6316	0.2632	0.0526	0.0000	0.0000	0.9474	0.0526	0.0000	0.0000
0.2609	3.9048	0.5810	0.0435	0.6522	0.2609	0.0435	0.0000	0.0000	0.9565	0.0435	0.0000	0.0000
0.3043	4.0455	0.6091	0.0435	0.6522	0.3043	0.0000	0.0000	0.0000	0.9130	0.0435	0.0000	0.0000
0.3077	4.0000	0.6000	0.0385	0.6538	0.2692	0.0385	0.0000	0.0000	0.8846	0.0769	0.0000	0.0000
0.2963	3.7692	0.5538	0.0370	0.7037	0.2593	0.0000	0.0000	0.0000	0.8889	0.1111	0.0000	0.0000
0.2609	3.8571	0.5714	0.0435	0.6957	0.2174	0.0435	0.0000	0.0000	0.9130	0.0435	0.0000	0.0000
0.2174	3.9048	0.5810	0.0435	0.6522	0.2609	0.0435	0.0000	0.0000	0.8696	0.0870	0.0000	0.0000
0.2609	3.7619	0.5524	0.0435	0.6957	0.2174	0.0435	0.0000	0.0000	0.9130	0.0435	0.0000	0.0000
0.2593	3.8800	0.5760	0.0741	0.5926	0.2963	0.0370	0.0000	0.0000	0.8889	0.0741	0.0000	0.0000
0.2593	3.7200	0.5440	0.0741	0.7037	0.1852	0.0370	0.0000	0.0000	0.9259	0.0370	0.0000	0.0000
0.2500	3.5769	0.5154	0.0714	0.7500	0.1429	0.0357	0.0000	0.0000	0.8929	0.0714	0.0000	0.0000
0.1364	3.6842	0.5368	0.0909	0.6818	0.1818	0.0455	0.0000	0.0000	0.9545	0.0455	0.0000	0.0000
0.2273	3.9000	0.5800	0.0455	0.6364	0.2727	0.0455	0.0000	0.0000	1.0000	0.0000	0.0000	0.0000
0.2381	4.1000	0.6200	0.0476	0.6190	0.2857	0.0476	0.0000	0.0000	1.0000	0.0000	0.0000	0.0000
0.2174	3.9048	0.5810	0.0870	0.5652	0.2609	0.0435	0.0000	0.0000	0.9565	0.0435	0.0000	0.0000
0.2381	3.9500	0.5900	0.0476	0.6190	0.2381	0.0476	0.0000	0.0000	0.9524	0.0476	0.0000	0.0000
0.2917	3.8636	0.5727	0.0833	0.6667	0.2083	0.0417	0.0000	0.0000	1.0000	0.0000	0.0000	0.0000
0.2414	3.7500	0.5500	0.0690	0.6207	0.2414	0.0345	0.0000	0.0000	0.9655	0.0345	0.0000	0.0000
0.2500	3.7391	0.5478	0.0833	0.5000	0.3333	0.0417	0.0000	0.0000	0.9583	0.0417	0.0000	0.0000
0.2258	3.8333	0.5667	0.0645	0.6452	0.2258	0.0323	0.0000	0.0000	1.0000	0.0000	0.0000	0.0000
0.1923	3.9167	0.5833	0.0385	0.7308	0.1538	0.0769	0.0000	0.0000	0.9615	0.0385	0.0370	0.0000
0.2593	3.6800	0.5360	0.1111	0.4815	0.3333	0.0741	0.0000	0.0000	1.0000	0.0000	0.0000	0.0000
0.2667	3.6786	0.5357	0.0667	0.6333	0.2333	0.0667	0.0000	0.0000	0.9667	0.0333	0.0000	0.0000
0.2258	3.6897	0.5379	0.0323	0.6452	0.2581	0.0645	0.0000	0.0000	0.9355	0.0645	0.0000	0.0000
0.2059	3.5625	0.5125	0.1176	0.5882	0.2353	0.0588	0.0000	0.0000	0.9706	0.0294	0.0000	0.0000
0.2069	3.7778	0.5556	0.1034	0.5862	0.2759	0.0345	0.0000	0.0000	0.9310	0.0345	0.0333	0.0000
0.2143	3.5600	0.5120	0.0714	0.5714	0.2857	0.0714	0.0000	0.0000	0.9643	0.0357	0.0000	0.0000
0.1905	3.7895	0.5579	0.0952	0.4762	0.3333	0.0952	0.0000	0.0000	1.0000	0.0000	0.0000	0.0000
0.2692	3.9167	0.5833	0.0385	0.6538	0.2308	0.0769	0.0000	0.0000	1.0000	0.0000	0.0000	0.0000
0.2500	3.9091	0.5818	0.0417	0.5833	0.2500	0.0833	0.0000	0.0000	1.0000	0.0000	0.0000	0.0000
0.1379	3.6296	0.5259	0.0690	0.6207	0.2069	0.0690	0.0000	0.0000	1.0000	0.0000	0.0000	0.0000
0.2188	3.7667	0.5533	0.0313	0.6563	0.2188	0.0625	0.0000	0.0000	1.0000	0.0000	0.0000	0.0000
0.1429	3.6154	0.5231	0.1429	0.5357	0.2500	0.0714	0.0000	0.0000	1.0000	0.0000	0.0000	0.0000
0.2258	3.5862	0.5172	0.1290	0.5806	0.1935	0.0645	0.0000	0.0000	1.0000	0.0000	0.0000	0.0000
0.2258	3.7857	0.5571	0.0968	0.6129	0.2258	0.0645	0.0000	0.0000	0.9677	0.0323	0.0313	0.0000
0.2500	3.8000	0.5600	0.0625	0.6563	0.2188	0.0625	0.0000	0.0000	0.9688	0.0313	0.0303	0.0000
0.2609	3.8095	0.5619	0.0870	0.4783	0.3478	0.0870	0.0000	0.0000	0.9130	0.0870	0.0000	0.0000
0.2857	3.6316	0.5263	0.0952	0.5238	0.2381	0.0952	0.0000	0.0000	0.9524	0.0476	0.0000	0.0000
0.2727	3.6500	0.5300	0.0909	0.4545	0.3182	0.0909	0.0000	0.0000	0.9545	0.0455	0.0435	0.0000
0.3333	3.7200	0.5440	0.0741	0.5556	0.2963	0.0741	0.0000	0.0000	0.9630	0.0370	0.0000	0.0000

0.2333	3.5357	0.5071	0.0667	0.5667	0.2667	0.0667	0.0000	0.0000	0.9667	0.0333	0.0000	0.0000
0.2222	4.3750	0.6750	0.0000	0.4444	0.5556	0.0000	0.0000	0.0000	1.0000	0.0000	0.0000	0.0000
0.2222	4.5000	0.7000	0.0000	0.5556	0.4444	0.0000	0.0000	0.0000	1.0000	0.0000	0.0000	0.0000
0.0417	3.0476	0.4095	0.2083	0.6250	0.0833	0.0833	0.0000	0.0000	1.0000	0.0000	0.1111	0.0000
0.0000	2.5556	0.3111	0.2857	0.5714	0.0476	0.0952	0.0000	0.0000	1.0000	0.0000	0.1923	0.0000
0.0400	2.9545	0.3909	0.1200	0.7200	0.0800	0.0800	0.0000	0.0000	1.0000	0.0000	0.1071	0.0000
0.0000	3.0000	0.4000	0.0769	0.6154	0.1538	0.1538	0.0000	0.0000	1.0000	0.0000	0.0000	0.0000
0.0000	2.7059	0.3412	0.2000	0.6000	0.1000	0.1000	0.0000	0.0000	1.0000	0.0000	0.0476	0.0000
0.0000	3.2857	0.4571	0.1111	0.5556	0.2222	0.1111	0.0000	0.0000	1.0000	0.0000	0.0000	0.0000
0.3077	4.5385	0.7077	0.0000	0.5385	0.4615	0.0000	0.0000	0.0000	0.9231	0.0769	0.0000	0.0000
0.0000	3.0667	0.4133	0.1765	0.6471	0.1176	0.0588	0.0000	0.0000	1.0000	0.0000	0.0000	0.0000
0.1429	3.3571	0.4714	0.2143	0.5714	0.2143	0.0000	0.0000	0.0000	0.9286	0.0714	0.0000	0.0000
0.2222	4.1111	0.6222	0.0000	0.3333	0.6667	0.0000	0.0000	0.0000	1.0000	0.0000	0.0000	0.0000
0.0476	2.6000	0.3200	0.1905	0.6667	0.0952	0.0476	0.0000	0.0000	0.9524	0.0000	0.0455	0.0000
0.0833	3.0833	0.4167	0.0833	0.4167	0.5000	0.0000	0.0000	0.0000	1.0000	0.0000	0.0000	0.0000
0.0000	2.1538	0.2308	0.1538	0.6923	0.1538	0.0000	0.0000	0.0000	1.0000	0.0000	0.0000	0.0000
0.0000	2.7000	0.3400	0.2000	0.4000	0.4000	0.0000	0.0000	0.0000	1.0000	0.0000	0.0000	0.0000
0.0000	2.1538	0.2308	0.1538	0.8462	0.0000	0.0000	0.0000	0.0000	0.9231	0.0000	0.0000	0.0000
0.0000	2.5833	0.3167	0.1667	0.5833	0.2500	0.0000	0.0000	0.0000	1.0000	0.0000	0.0000	0.0000
0.2500	3.7500	0.5500	0.1250	0.4375	0.4375	0.0000	0.0000	0.0000	1.0000	0.0000	0.0000	0.0000
0.0000	2.0000	0.2000	0.3333	0.6667	0.0000	0.0000	0.0000	0.0000	1.0000	0.0000	0.0000	0.0000
0.0000	2.7778	0.3556	0.1111	0.4444	0.4444	0.0000	0.0000	0.0000	1.0000	0.0000	0.0000	0.0000
0.0000	2.0000	0.2000	0.2667	0.6000	0.0667	0.0667	0.0000	0.0000	0.9333	0.0000	0.0000	0.0000
0.1111	4.3333	0.6667	0.0000	0.5556	0.4444	0.0000	0.0000	0.0000	0.8889	0.1111	0.0000	0.0000
0.3333	4.8750	0.7750	0.0000	0.5556	0.4444	0.0000	0.0000	0.0000	1.0000	0.0000	0.0000	0.0000
0.0800	3.3750	0.4750	0.0400	0.7200	0.2400	0.0000	0.0000	0.0000	1.0000	0.0000	0.0000	0.0000
0.1053	3.7368	0.5474	0.0000	0.6842	0.3158	0.0000	0.0000	0.0000	1.0000	0.0000	0.0000	0.0000
0.1333	3.5333	0.5067	0.0667	0.7333	0.2000	0.0000	0.0000	0.0000	0.9333	0.0667	0.0000	0.0000
0.6000	4.2500	0.6500	0.0000	0.6000	0.4000	0.0000	0.0000	0.0000	0.8000	0.0000	0.0000	0.0000
0.4000	4.4000	0.6800	0.0000	0.6000	0.4000	0.0000	0.0000	0.0000	1.0000	0.0000	0.0000	0.0000
0.3125	4.2667	0.6533	0.0000	0.6875	0.3125	0.0000	0.0000	0.0000	1.0000	0.0000	0.0000	0.0000
0.3333	4.4500	0.6900	0.0000	0.6190	0.3810	0.0000	0.0000	0.0000	0.9048	0.0952	0.0000	0.0000
0.2941	4.4000	0.6800	0.0588	0.5882	0.2941	0.0588	0.0000	0.0000	1.0000	0.0000	0.0000	0.0000
0.2857	4.4615	0.6923	0.0000	0.5714	0.4286	0.0000	0.0000	0.0000	0.9286	0.0714	0.0000	0.0000
0.2500	4.3636	0.6727	0.0000	0.5833	0.4167	0.0000	0.0000	0.0000	1.0000	0.0000	0.0000	0.0000
0.2727	4.3182	0.6636	0.0909	0.5000	0.4091	0.0000	0.0000	0.0000	1.0000	0.0000	0.0000	0.0000
0.3529	4.4375	0.6875	0.0588	0.5882	0.3529	0.0000	0.0000	0.0000	0.9412	0.0588	0.0000	0.0000
0.2941	4.1765	0.6353	0.1765	0.4706	0.3529	0.0000	0.0000	0.0000	1.0000	0.0000	0.0000	0.0000
0.2381	3.9500	0.5900	0.0952	0.5714	0.3333	0.0000	0.0000	0.0000	0.9524	0.0476	0.0000	0.0000
0.1818	4.1905	0.6381	0.0455	0.5909	0.3182	0.0455	0.0000	0.0000	1.0000	0.0000	0.0000	0.0000
0.2500	4.0000	0.6000	0.0625	0.6875	0.2500	0.0000	0.0000	0.0000	0.8125	0.1875	0.0000	0.0000
0.4375	4.5000	0.7000	0.0000	0.5000	0.4375	0.0625	0.0000	0.0000	1.0000	0.0000	0.0000	0.0000
0.1538	3.7692	0.5538	0.0769	0.5385	0.3846	0.0000	0.0000	0.0000	1.0000	0.0000	0.0000	0.0000
0.0000	4.3333	0.6667	0.0000	0.3333	0.6667	0.0000	0.0000	0.0000	1.0000	0.0000	0.0000	0.0000
0.2941	4.1250	0.6250	0.0000	0.5882	0.4118	0.0000	0.0000	0.0000	0.9412	0.0588	0.0000	0.0000
0.1500	3.6500	0.5300	0.0500	0.7500	0.2000	0.0000	0.0000	0.0000	0.9500	0.0500	0.0000	0.0000
0.0000	4.0000	0.6000	0.0833	0.4167	0.5000	0.0000	0.0000	0.0000	1.0000	0.0000	0.0000	0.0000
0.3333	3.6667	0.5333	0.1667	0.5000	0.3333	0.0000	0.0000	0.0000	1.0000	0.0000	0.0000	0.0000
0.1250	3.0000	0.4000	0.0000	0.7500	0.1250	0.1250	0.0000	0.0000	0.6250	0.3750	0.3846	0.0000
0.1538	3.4615	0.4923	0.0769	0.6923	0.2308	0.0000	0.0000	0.0000	1.0000	0.0000	0.0714	0.0000
0.1250	2.8667	0.3733	0.0000	0.7500	0.1875	0.0625	0.0000	0.0000	0.7500	0.2500	0.3333	0.0000
0.4375	4.1875	0.6375	0.0000	0.6250	0.3750	0.0000	0.0000	0.0000	0.7500	0.2500	0.0000	0.0000
0.2273	3.8500	0.5700	0.1364	0.4091	0.4091	0.0455	0.0000	0.0000	0.9545	0.0455	0.0000	0.0000
0.3182	4.1429	0.6286	0.0000	0.8182	0.1818	0.0000	0.0000	0.0000	0.8636	0.1364	0.0000	0.0000
0.3571	4.3846	0.6769	0.0714	0.5000	0.4286	0.0000	0.0000	0.0000	0.9286	0.0714	0.0000	0.0000
0.2000	3.8333	0.5667	0.0400	0.6000	0.3600	0.0000	0.0000	0.0000	1.0000	0.0000	0.0000	0.0000
0.3750	4.3333	0.6667	0.0625	0.5625	0.3750	0.0000	0.0000	0.0000	1.0000	0.0000	0.0000	0.0000
0.2500	4.0556	0.6111	0.0500	0.6500	0.3000	0.0000	0.0000	0.0000	0.9000	0.0500	0.0000	0.0000
0.3636	4.4545	0.6909	0.0909	0.4545	0.4545	0.0000	0.0000	0.0000	1.0000	0.0000	0.0000	0.0000
0.0526	3.6111	0.5222	0.1053	0.4737	0.3684	0.0526	0.0000	0.0000	1.0000	0.0000	0.0000	0.0000
0.2000	4.1111	0.6222	0.0500	0.6500	0.2500	0.0500	0.0000	0.0000	1.0000	0.0000	0.0000	0.0000
0.3571	4.1429	0.6286	0.0000	0.5000	0.5000	0.0000	0.0000	0.0000	1.0000	0.0000	0.0000	0.0000
0.2222	4.4706	0.6941	0.0556	0.3889	0.5556	0.0000	0.0000	0.0000	1.0000	0.0000	0.0000	0.0000
0.1765	4.0000	0.6000	0.0588	0.7059	0.2353	0.0000	0.0000	0.0000	1.0000	0.0000	0.0000	0.0000
0.2857	4.0714	0.6143	0.0000	0.5714	0.4286	0.0000	0.0000	0.0000	1.0000	0.0000	0.0000	0.0000
0.2667	4.0000	0.6000	0.1333	0.4667	0.4000	0.0000	0.0000	0.0000	1.0000	0.0000	0.0000	0.0000
0.1176	3.4706	0.4941	0.0000	0.5882	0.4118	0.0000	0.0000	0.0000	0.8235	0.1765	0.0000	0.0000
0.0625	3.4667	0.4933	0.0625	0.5000	0.4375	0.0000	0.0000	0.0000	0.9375	0.0000	0.0000	0.0000
0.0714	3.3571	0.4714	0.0714	0.6429	0.2857	0.0000	0.0000	0.0000	0.6429	0.3571	0.0000	0.0000
0.1176	3.8000	0.5600	0.1176	0.6471	0.2353	0.0000	0.0000	0.0000	0.6471	0.2353	0.0000	0.0000
0.0000	2.7273	0.3455	0.1667	0.5000	0.3333	0.0000	0.0000	0.0000	0.7500	0.1667	0.0000	0.0000
0.4000	4.0714	0.6143	0.0000	0.6667	0.3333	0.0000	0.0000	0.0000	1.0000	0.0000	0.0000	0.0000
0.2778	4.4118	0.6824	0.0556	0.3889	0.5556	0.0000	0.0000	0.0000	1.0000	0.0000	0.0000	0.0000
0.2000	3.6316	0.5263	0.0500	0.6500	0.3000	0.0000	0.0000	0.0000	0.7500	0.2000	0.0000	0.0000
0.3750	4.4783	0.6957	0.0000	0.7500	0.2500	0.0000	0.0000	0.0000	0.8333	0.1667	0.0000	0.0000
0.2692	3.9600	0.5920	0.0385	0.6538	0.2692	0.0385	0.0000	0.0000	1.0000	0.0000	0.0000	0.0000
0.3077	4.2500	0.6500	0.0000	0.4615	0.5385	0.0000	0.0000	0.0000	1.0000	0.0000	0.0000	0.0000

0.3158	3.8889	0.5778	0.0000	0.5789	0.4211	0.0000	0.0000	0.0000	0.8421	0.1579	0.0000	0.0000
0.2000	4.3000	0.6600	0.0000	0.3000	0.7000	0.0000	0.0000	0.0000	1.0000	0.0000	0.0000	0.0000
0.3333	4.8000	0.7600	0.0000	0.6667	0.3333	0.0000	0.0000	0.0000	0.8333	0.1667	0.0000	0.0000
0.2000	3.5000	0.5000	0.0000	0.4000	0.6000	0.0000	0.0000	0.0000	0.9000	0.1000	0.0000	0.0000
0.3333	4.4167	0.6833	0.0000	0.3333	0.6667	0.0000	0.0000	0.0000	1.0000	0.0000	0.0000	0.0000
0.0000	2.4444	0.2889	0.0000	0.7778	0.2222	0.0000	0.0000	0.0000	0.8889	0.1111	0.0000	0.0000
0.0667	2.9286	0.3857	0.2000	0.7333	0.0667	0.0000	0.0000	0.0000	1.0000	0.0000	0.1667	0.0000
0.2632	3.4211	0.4842	0.1053	0.7368	0.1579	0.0000	0.0000	0.0000	1.0000	0.0000	0.1739	0.0000
0.3077	4.3333	0.6667	0.0000	0.4615	0.5385	0.0000	0.0000	0.0000	1.0000	0.0000	0.0000	0.0000
0.1429	3.7143	0.5429	0.0714	0.4286	0.5000	0.0000	0.0000	0.0000	1.0000	0.0000	0.0000	0.0000
0.0000	2.6667	0.3333	0.2143	0.6429	0.0714	0.0714	0.0000	0.0000	0.9286	0.0714	0.0000	0.0000
0.5000	4.7500	0.7500	0.0000	0.6250	0.3750	0.0000	0.0000	0.0000	0.8750	0.1250	0.0000	0.0000
0.0000	2.5714	0.3143	0.2667	0.6000	0.0667	0.0667	0.0000	0.0000	1.0000	0.0000	0.0000	0.0000
0.0667	3.6000	0.5200	0.0000	0.4000	0.6000	0.0000	0.0000	0.0000	1.0000	0.0000	0.0000	0.0000
0.0833	3.4167	0.4833	0.1667	0.5833	0.2500	0.0000	0.0000	0.0000	0.8333	0.1667	0.0000	0.0000
0.2500	4.5714	0.7143	0.0000	0.6250	0.3750	0.0000	0.0000	0.0000	0.8750	0.1250	0.0000	0.0000
0.0000	3.3333	0.4667	0.1538	0.7692	0.0000	0.0769	0.0000	0.0000	0.9231	0.0769	0.0714	0.0000
0.2941	4.1875	0.6375	0.0588	0.2353	0.7059	0.0000	0.0000	0.0000	1.0000	0.0000	0.0000	0.0000
0.2222	3.8889	0.5778	0.1111	0.4444	0.4444	0.0000	0.0000	0.0000	1.0000	0.0000	0.0000	0.0000
0.3333	4.3636	0.6727	0.0000	0.4167	0.5833	0.0000	0.0000	0.0000	1.0000	0.0000	0.0000	0.0000
0.2727	4.0000	0.6000	0.0909	0.2727	0.6364	0.0000	0.0000	0.0000	1.0000	0.0000	0.0000	0.0000
0.2222	4.6250	0.7250	0.0000	0.4444	0.5556	0.0000	0.0000	0.0000	1.0000	0.0000	0.0000	0.0000
0.2667	4.4667	0.6933	0.0000	0.4667	0.5333	0.0000	0.0000	0.0000	1.0000	0.0000	0.0000	0.0000
0.2353	4.1250	0.6250	0.0000	0.5294	0.4706	0.0000	0.0000	0.0000	1.0000	0.0000	0.0556	0.0000
0.0526	3.1875	0.4375	0.1053	0.6842	0.1053	0.1053	0.0000	0.0000	0.8947	0.1053	0.0952	0.0000
0.2000	2.2000	0.2400	0.4000	0.2000	0.2000	0.0000	0.3000	0.2000	1.0000	0.0000	0.0000	0.5000
0.0000	1.7778	0.1556	0.5556	0.2222	0.0000	0.0000	0.2143	0.1429	1.0000	0.0000	0.0000	0.3571
0.2500	2.0000	0.2000	0.5000	0.1250	0.1250	0.0000	0.1875	0.1875	1.0000	0.0000	0.1250	0.3750
0.0909	2.0000	0.2000	0.4545	0.1818	0.0909	0.0000	0.2857	0.1429	1.0000	0.0000	0.0476	0.4286
0.0000	1.8571	0.1714	0.5714	0.1429	0.0000	0.0000	0.2857	0.0714	1.0000	0.0000	0.1429	0.3571
0.0000	1.4000	0.0800	0.5000	0.0000	0.0000	0.0000	0.2500	0.0000	1.0000	0.0000	0.2500	0.2500
0.0000	2.2500	0.2500	0.5000	0.2500	0.0000	0.0000	0.4615	0.2308	1.0000	0.0000	0.0000	0.6923
0.1429	2.3333	0.2667	0.5714	0.1429	0.0000	0.0000	0.4000	0.1500	1.0000	0.0000	0.1000	0.5500
0.1429	2.0000	0.2000	0.2857	0.1429	0.0000	0.0000	0.3889	0.1111	1.0000	0.0000	0.1111	0.5000
0.0000	1.5714	0.1143	0.4286	0.0000	0.0000	0.0000	0.3529	0.1765	1.0000	0.0000	0.0000	0.5882
0.1111	2.1250	0.2250	0.5556	0.1111	0.0000	0.0000	0.3684	0.1053	1.0000	0.0000	0.0526	0.4737
0.0000	1.6667	0.1333	0.5000	0.0000	0.0000	0.0000	0.4737	0.1579	1.0000	0.0000	0.0526	0.6316
0.1429	2.1429	0.2286	0.4286	0.1429	0.0000	0.0000	0.3125	0.1250	1.0000	0.0000	0.0625	0.5000
0.2000	2.2000	0.2400	0.4000	0.1000	0.0000	0.0000	0.1765	0.1765	1.0000	0.0000	0.0588	0.3529
0.0909	2.2727	0.2545	0.3636	0.1818	0.0000	0.0000	0.3200	0.1600	1.0000	0.0000	0.0800	0.4800
0.1429	2.4286	0.2857	0.4286	0.1429	0.2857	0.0000	0.0667	0.4667	1.0000	0.0000	0.0000	0.5333
0.1250	2.3750	0.2750	0.3750	0.2500	0.2500	0.0000	0.1667	0.0833	1.0000	0.0000	0.0833	0.2500
0.0000	2.0000	0.2000	0.5000	0.1667	0.1667	0.0000	0.0000	0.4286	1.0000	0.0000	0.0714	0.5000
0.1250	1.3750	0.0750	0.5000	0.1250	0.0000	0.0000	0.3529	0.1176	1.0000	0.0000	0.0588	0.4706
0.1250	1.8571	0.1714	0.3750	0.1250	0.1250	0.1250	0.2667	0.1333	1.0000	0.0000	0.0000	0.4667
0.0000	1.6667	0.1333	0.6667	0.0000	0.0000	0.0000	0.4615	0.1538	1.0000	0.0000	0.1538	0.6154
0.2500	2.2500	0.2500	0.2500	0.0000	0.2500	0.0000	0.0000	0.1667	1.0000	0.0000	0.1667	0.1667
0.0000	2.0000	0.2000	0.0000	0.0000	0.3333	0.0000	0.2000	0.0000	1.0000	0.0000	0.2000	0.2000
0.1250	2.0000	0.2000	0.2500	0.1250	0.1250	0.1250	0.0000	0.2500	1.0000	0.0000	0.0833	0.2500
0.0000	2.0000	0.2000	0.4000	0.2000	0.0000	0.0000	0.3889	0.2222	1.0000	0.0000	0.1111	0.6111
0.2000	1.6000	0.1200	0.4000	0.0000	0.0000	0.0000	0.2667	0.2000	1.0000	0.0000	0.2000	0.4667
0.0000	1.8000	0.1600	0.2000	0.2000	0.0000	0.0000	0.3333	0.2000	1.0000	0.0000	0.1333	0.5333
0.0000	1.5000	0.1000	0.2500	0.0000	0.0000	0.2500	0.2500	0.1250	1.0000	0.0000	0.1250	0.3750
0.0000	1.3333	0.0667	0.5000	0.0000	0.0000	0.1667	0.3125	0.2500	1.0000	0.0000	0.0625	0.5625
0.1429	2.0000	0.2000	0.2857	0.2857	0.0000	0.0000	0.3125	0.1250	1.0000	0.0000	0.1250	0.4375
0.0000	1.6667	0.1333	0.6667	0.0000	0.0000	0.0000	0.2500	0.0833	1.0000	0.0000	0.1667	0.3333
0.0000	1.4000	0.0800	0.6667	0.0000	0.0000	0.0000	0.1053	0.4211	1.0000	0.0000	0.1053	0.5789
0.0000	1.6000	0.1200	0.6000	0.0000	0.0000	0.0000	0.1429	0.0000	1.0000	0.0000	0.1429	0.1429
0.1429	1.8571	0.1714	0.4286	0.1429	0.0000	0.0000	0.2857	0.0714	1.0000	0.0000	0.1429	0.3571
0.2000	2.2500	0.2500	0.4000	0.2000	0.0000	0.0000	0.3077	0.1538	1.0000	0.0000	0.1538	0.4615
0.0000	1.5000	0.1000	0.7500	0.0000	0.0000	0.0000	0.2727	0.1818	1.0000	0.0000	0.1818	0.4545
0.1667	1.8333	0.1667	0.5000	0.0000	0.0000	0.0000	0.1818	0.1818	1.0000	0.0000	0.0909	0.3636
0.0000	1.0000	0.0000	0.2500	0.0000	0.0000	0.0000	0.3846	0.1538	1.0000	0.0000	0.1538	0.5385
0.1667	1.6667	0.1333	0.5000	0.0000	0.0000	0.0000	0.1111	0.2222	1.0000	0.0000	0.0000	0.3333
0.1667	2.3333	0.2667	0.1667	0.1667	0.0000	0.0000	0.3846	0.0769	1.0000	0.0000	0.0769	0.4615
0.0000	2.0000	0.2000	0.4286	0.1429	0.0000	0.0000	0.3636	0.0000	1.0000	0.0000	0.0000	0.3636
0.0000	1.8571	0.1714	0.2857	0.1429	0.0000	0.0000	0.4000	0.1333	1.0000	0.0000	0.0000	0.5333
0.1429	2.0000	0.2000	0.1429	0.1429	0.0000	0.0000	0.3889	0.1111	1.0000	0.0000	0.1111	0.5000
0.1250	2.0000	0.2000	0.2500	0.1250	0.0000	0.0000	0.3684	0.1053	1.0000	0.0000	0.1053	0.4737
0.0000	1.8333	0.1667	0.5000	0.0000	0.0000	0.0000	0.4000	0.1333	1.0000	0.0000	0.0667	0.5333
0.2000	1.2000	0.0400	0.2000	0.0000	0.0000	0.2000	0.1000	0.1000	1.0000	0.0000	0.3000	0.2000
0.1429	1.5714	0.1143	0.2857	0.0000	0.0000	0.1429	0.0000	0.1000	1.0000	0.0000	0.2000	0.1000
0.1429	1.7143	0.1429	0.4286	0.0000	0.0000	0.1429	0.0833	0.1667	1.0000	0.0000	0.0833	0.3333
0.2857	1.8571	0.1714	0.2857	0.0000	0.0000	0.1429	0.1429	0.1429	1.0000	0.0000	0.1429	0.3571
0.1250	1.6250	0.1250	0.2500	0.0000	0.0000	0.1250	0.2941	0.1765	1.0000	0.0000	0.0588	0.4706
0.2500	1.8750	0.1750	0.3750	0.0000	0.0000	0.1250	0.1333	0.1333	1.0000	0.0000	0.1333	0.3333
0.0000	1.6667	0.1333	0.4286	0.1429	0.0000	0.0000	0.3333	0.1333	1.0000	0.0000	0.0667	0.4667

0.2500	1.7500	0.1500	0.5000	0.0000	0.0000	0.0000	0.1818	0.3636	1.0000	0.0000	0.0909	0.5455
0.2000	2.2000	0.2400	0.2000	0.2000	0.0000	0.0000	0.3529	0.1765	1.0000	0.0000	0.1765	0.5294
0.0000	2.3333	0.2667	0.1667	0.1667	0.1667	0.0000	0.4000	0.1333	1.0000	0.0000	0.0667	0.5333
0.1429	2.1429	0.2286	0.1429	0.1429	0.1429	0.0000	0.1875	0.2500	1.0000	0.0000	0.1250	0.4375
0.0000	1.6000	0.1200	0.4000	0.0000	0.0000	0.0000	0.3846	0.1538	1.0000	0.0000	0.0769	0.5385
0.0000	2.4000	0.2800	0.2000	0.2000	0.2000	0.0000	0.5000	0.0625	1.0000	0.0000	0.1250	0.5625
0.1667	1.8333	0.1667	0.6667	0.0000	0.0000	0.0000	0.2143	0.3571	1.0000	0.0000	0.0000	0.5714
0.1667	1.5000	0.1000	0.3333	0.0000	0.0000	0.1667	0.2000	0.0000	1.0000	0.0000	0.2000	0.2000
0.2857	1.5000	0.1000	0.2857	0.0000	0.0000	0.1429	0.1818	0.0909	1.0000	0.0000	0.0909	0.2727
0.4000	2.0000	0.2000	0.2000	0.0000	0.0000	0.0000	0.0000	0.1429	1.0000	0.0000	0.1429	0.1429
0.0000	2.0000	0.2000	0.0000	0.0000	0.3333	0.0000	0.4000	0.3000	1.0000	0.0000	0.0000	0.7000
0.1667	2.0000	0.2000	0.3333	0.0000	0.1667	0.0000	0.3750	0.1250	1.0000	0.0000	0.1250	0.5000
0.2000	1.7500	0.1500	0.4000	0.0000	0.0000	0.2000	0.0000	0.0000	1.0000	0.0000	0.0000	0.0000
0.2500	2.2500	0.2500	0.2500	0.0000	0.0000	0.2500	0.0000	0.2000	1.0000	0.0000	0.0000	0.2000
0.3333	1.8333	0.1667	0.1667	0.0000	0.0000	0.1667	0.3077	0.1538	1.0000	0.0000	0.0769	0.4615
0.0000	2.0000	0.2000	0.2000	0.2000	0.0000	0.0000	0.3529	0.1765	1.0000	0.0000	0.1765	0.5294
0.0000	2.4000	0.2800	0.0000	0.4000	0.2000	0.0000	0.3571	0.1429	1.0000	0.0000	0.1429	0.5000
0.3333	2.0000	0.2000	0.1667	0.1667	0.1667	0.1667	0.0000	0.1429	1.0000	0.0000	0.0000	0.1429
0.3750	2.1429	0.2286	0.2500	0.1250	0.1250	0.1250	0.0000	0.1111	1.0000	0.0000	0.0000	0.1111
0.2857	2.0000	0.2000	0.1429	0.1429	0.2857	0.1429	0.0000	0.0000	1.0000	0.0000	0.0000	0.0000
0.1111	2.2222	0.2444	0.3333	0.2222	0.2222	0.0000	0.0000	0.3077	1.0000	0.0000	0.0000	0.3077
0.0000	2.4000	0.2800	0.2000	0.2000	0.4000	0.0000	0.2727	0.2727	1.0000	0.0000	0.0000	0.5455
0.1818	2.0000	0.2000	0.1818	0.1818	0.2727	0.0909	0.0000	0.1429	1.0000	0.0000	0.0714	0.1429
0.1429	2.3333	0.2667	0.0000	0.4286	0.4286	0.1429	0.0000	0.5000	1.0000	0.0000	0.0625	0.5000
0.1000	2.6250	0.3250	0.2000	0.5000	0.2000	0.1000	0.0625	0.3125	1.0000	0.0000	0.0000	0.3750
0.1111	2.5556	0.3111	0.3333	0.3333	0.2222	0.0000	0.0000	0.3077	1.0000	0.0000	0.0000	0.3077
0.2500	3.1250	0.4250	0.1250	0.3750	0.3750	0.0000	0.0000	0.2500	1.0000	0.0000	0.0000	0.3333
0.0000	2.0000	0.2000	0.6667	0.0000	0.0000	0.1667	0.0000	0.4000	0.8333	0.0000	0.1333	0.4667
0.1429	2.0000	0.2000	0.4286	0.1429	0.1429	0.0000	0.1250	0.0000	1.0000	0.0000	0.0000	0.1250
0.1818	2.0000	0.2000	0.3636	0.0000	0.0909	0.0909	0.2353	0.1176	1.0000	0.0000	0.0000	0.3529
0.2857	2.1667	0.2333	0.2857	0.0000	0.1429	0.0000	0.1818	0.0909	1.0000	0.0000	0.0909	0.2727
0.1667	1.5000	0.1000	0.3333	0.0000	0.0000	0.1667	0.1818	0.1818	1.0000	0.0000	0.0000	0.4545
0.2857	1.8333	0.1667	0.2857	0.0000	0.0000	0.1429	0.0714	0.2857	1.0000	0.0000	0.0714	0.4286
0.2000	1.7500	0.1500	0.4000	0.0000	0.0000	0.0000	0.0000	0.3333	0.8000	0.0000	0.1111	0.3333
0.1429	1.8571	0.1714	0.2857	0.1429	0.0000	0.0000	0.0000	0.3333	0.8571	0.1429	0.0000	0.4167
0.4000	2.0000	0.2000	0.4000	0.0000	0.0000	0.0000	0.1667	0.2500	0.8000	0.0000	0.1667	0.4167
							0.0000	0.8571			0.1429	0.8571
0.2000	1.7500	0.1500	0.4000	0.0000	0.0000	0.0000	0.1667	0.3333	1.0000	0.0000	0.0833	0.5000
0.5000	2.2500	0.2500	0.5000	0.0000	0.0000	0.0000	0.0000	0.3750	1.0000	0.0000	0.1250	0.3750
0.3333	2.5000	0.3000	0.3333	0.0000	0.0000	0.0000	0.0000	0.5000	0.6667	0.0000	0.1250	0.5000
0.0000	NA	NA	0.0000	0.0000	0.0000	0.0000	0.0000	0.5000	0.0000	0.0000	0.2500	0.6250
							0.0000	0.8750			0.0000	1.0000
0.2000	2.0000	0.2000	0.4000	0.0000	0.0000	0.2000	0.0000	0.4000	0.8000	0.0000	0.1000	0.4000
0.0000	1.0000	0.0000	0.0000	0.0000	0.0000	0.5000	0.0000	0.5000	1.0000	0.0000	0.1250	0.6250
0.2000	1.6000	0.1200	0.2000	0.0000	0.0000	0.0000	0.3750	0.0000	1.0000	0.0000	0.0000	0.3750
							0.0000	0.6667			0.1667	0.8333
0.0000	NA	NA	0.0000	0.0000	0.0000	0.0000	0.0000	0.5714	0.0000	0.0000	0.1429	0.7143
0.3333	1.8333	0.1667	0.3333	0.0000	0.0000	0.1667	0.0000	0.2222	1.0000	0.0000	0.1111	0.2222
0.2857	1.7143	0.1429	0.2857	0.0000	0.0000	0.1429	0.1667	0.1667	1.0000	0.0000	0.0833	0.3333
0.2500	1.6667	0.1333	0.5000	0.0000	0.0000	0.0000	0.2857	0.1429	1.0000	0.0000	0.0000	0.4286
0.0000	2.0000	0.2000	0.5000	0.0000	0.0000	0.0000	0.0667	0.3333	1.0000	0.0000	0.2667	0.4667
0.0000	3.0000	0.4000	1.0000	0.0000	0.0000	0.0000	0.0000	0.5000	1.0000	0.0000	0.2500	0.6250
0.2000	1.8000	0.1600	0.4000	0.0000	0.0000	0.2000	0.0000	0.1667	1.0000	0.0000	0.0000	0.1667
0.3333	2.0000	0.2000	0.3333	0.0000	0.0000	0.0000	0.0833	0.3333	1.0000	0.0000	0.0833	0.4167
0.0000	1.5000	0.1000	0.4000	0.0000	0.0000	0.2000	0.0000	0.3333	1.0000	0.0000	0.1111	0.3333
0.2000	2.0000	0.2000	0.4000	0.0000	0.0000	0.2000	0.2308	0.2308	1.0000	0.0000	0.1538	0.4615
0.0000	1.7500	0.1500	0.5000	0.0000	0.2500	0.0000	0.1429	0.1429	1.0000	0.0000	0.1429	0.2857
0.5000	2.0000	0.2000	0.2500	0.0000	0.0000	0.2500	0.0000	0.3750	1.0000	0.0000	0.0000	0.5000
0.3333	2.1667	0.2333	0.1667	0.0000	0.1667	0.1667	0.1538	0.3077	1.0000	0.0000	0.0769	0.4615
0.0000	1.6667	0.1333	0.0000	0.0000	0.3333	0.3333	0.0000	0.2500	1.0000	0.0000	0.0000	0.2500
0.2857	1.8571	0.1714	0.0000	0.0000	0.1429	0.1429	0.2143	0.1429	1.0000	0.0000	0.1429	0.3571
0.3333	2.0000	0.2000	0.0000	0.0000	0.1667	0.0000	0.1111	0.1111	1.0000	0.0000	0.1111	0.2222
0.2000	2.0000	0.2000	0.4000	0.0000	0.2000	0.2000	0.0000	0.0000	1.0000	0.0000	0.0000	0.0000
0.3333	2.0000	0.2000	0.0000	0.0000	0.3333	0.3333	0.0000	0.2000	1.0000	0.0000	0.2000	0.2000
0.2000	2.2500	0.2500	0.2000	0.0000	0.4000	0.2000	0.0000	0.0000	1.0000	0.0000	0.0000	0.0000
0.4000	2.4000	0.2800	0.2000	0.0000	0.2000	0.2000	0.0000	0.0000	1.0000	0.0000	0.0000	0.0000
0.5000	2.5000	0.3000	0.5000	0.0000	0.0000	0.0000	0.0000	0.5000	1.0000	0.0000	0.1250	0.6250
0.3077	3.5833	0.5167	0.0769	0.6923	0.2308	0.0000	0.0000	0.0588	1.0000	0.0000	0.1765	0.0588
0.4375	4.0000	0.6000	0.0000	0.5625	0.4375	0.0000	0.0000	0.0526	1.0000	0.0000	0.1053	0.0526
0.3529	3.8000	0.5600	0.0588	0.5882	0.3529	0.0000	0.0000	0.0500	1.0000	0.0000	0.1000	0.0500
0.2353	3.5000	0.5000	0.0588	0.5882	0.2941	0.0588	0.0417	0.1667	1.0000	0.0000	0.0417	0.2500
0.2500	3.5455	0.5091	0.0417	0.6250	0.2500	0.0417	0.0000	0.1071	1.0000	0.0000	0.0000	0.1429
0.2692	3.5652	0.5130	0.0385	0.6538	0.2692	0.0385	0.0000	0.0938	1.0000	0.0000	0.0313	0.1563
0.1471	3.7000	0.5400	0.0588	0.7941	0.0882	0.0294	0.0263	0.0263	0.9706	0.0294	0.0263	0.0789
0.2069	3.6154	0.5231	0.0345	0.6207	0.2759	0.0000	0.0294	0.0588	1.0000	0.0000	0.0294	0.1176
0.2381	3.4737	0.4947	0.0476	0.5714	0.2381	0.0000	0.0417	0.0000	1.0000	0.0000	0.0833	0.0417
0.1818	3.6316	0.5263	0.0000	0.6364	0.2727	0.0455	0.0000	0.0769	1.0000	0.0000	0.0385	0.1154

0.2353	3.2941	0.4588	0.0000	0.5294	0.3529	0.0000	0.0435	0.0870	1.0000	0.0000	0.0870	0.1739
0.1500	3.6875	0.5375	0.0000	0.6000	0.3000	0.0500	0.0000	0.0000	1.0000	0.0000	0.0909	0.0000
0.1765	3.4286	0.4857	0.0000	0.5882	0.2941	0.0588	0.0000	0.0000	1.0000	0.0000	0.1053	0.0000
0.2000	3.6190	0.5238	0.0400	0.6400	0.2400	0.0400	0.0000	0.0714	1.0000	0.0000	0.0357	0.0714
0.2381	3.6111	0.5222	0.0476	0.5714	0.2857	0.0000	0.0333	0.2000	1.0000	0.0000	0.0333	0.2667
0.1500	3.1667	0.4333	0.1000	0.5000	0.2500	0.0000	0.0370	0.1481	1.0000	0.0000	0.0741	0.1852
0.1500	3.4737	0.4947	0.0000	0.6500	0.3000	0.0500	0.0385	0.1538	1.0000	0.0000	0.0000	0.2308
0.1667	3.5333	0.5067	0.0000	0.5000	0.2778	0.0556	0.0370	0.1481	1.0000	0.0000	0.1111	0.2222
0.2353	3.4667	0.4933	0.0000	0.6471	0.2353	0.0588	0.0417	0.1667	0.9412	0.0588	0.0417	0.2500
0.2273	3.8421	0.5684	0.0000	0.5909	0.3182	0.0000	0.0000	0.0690	1.0000	0.0000	0.1379	0.1034
0.1765	3.2000	0.4400	0.0588	0.5294	0.2941	0.0000	0.0476	0.0476	0.9412	0.0588	0.0476	0.1429
0.1905	3.1000	0.4200	0.0476	0.5238	0.2857	0.0000	0.0400	0.0000	0.9524	0.0476	0.0800	0.0800
0.1250	3.3810	0.4762	0.0417	0.6250	0.2500	0.0000	0.0357	0.0357	1.0000	0.0000	0.0357	0.1071
0.1765	3.4375	0.4875	0.0588	0.3529	0.4706	0.0000	0.0000	0.1250	1.0000	0.0000	0.1250	0.1667
0.2727	3.1111	0.4222	0.0909	0.2727	0.3636	0.0000	0.0556	0.1667	1.0000	0.0000	0.1111	0.2778
0.2500	3.3333	0.4667	0.0000	0.5625	0.2500	0.0000	0.0417	0.1250	1.0000	0.0000	0.1250	0.2083
0.2000	3.2778	0.4556	0.0500	0.5000	0.3500	0.0000	0.0370	0.2222	0.9500	0.0500	0.0000	0.2593
0.1579	3.3125	0.4625	0.0526	0.5263	0.2632	0.0000	0.0000	0.0000	1.0000	0.0000	0.0500	0.0000
0.2500	3.1818	0.4364	0.0000	0.5000	0.2500	0.0000	0.0000	0.1875	0.9167	0.0833	0.0625	0.1875
0.2667	3.2857	0.4571	0.0667	0.4667	0.2667	0.0000	0.0909	0.1364	0.9333	0.0667	0.0455	0.2727
0.1579	3.3333	0.4667	0.1053	0.4737	0.3158	0.0000	0.0370	0.1481	0.9474	0.0526	0.0741	0.2222
0.2222	3.2500	0.4500	0.0556	0.5556	0.2778	0.0000	0.0370	0.1852	1.0000	0.0000	0.0741	0.2593
0.2273	3.6190	0.5238	0.0000	0.6364	0.3182	0.0455	0.0000	0.0000	1.0000	0.0000	0.0800	0.0400
0.1905	3.3889	0.4778	0.0476	0.4762	0.2857	0.0476	0.0333	0.1667	0.9524	0.0476	0.0667	0.2333
0.1500	3.4706	0.4941	0.0500	0.5500	0.2500	0.0000	0.0000	0.1786	1.0000	0.0000	0.1071	0.1786
0.1905	3.2105	0.4421	0.0476	0.4286	0.2857	0.0000	0.0606	0.2424	0.9524	0.0476	0.0303	0.3333
0.1111	3.1250	0.4250	0.0556	0.5000	0.2222	0.0000	0.0000	0.0000	1.0000	0.0000	0.1000	0.0000
0.1667	3.4706	0.4941	0.0556	0.6111	0.2778	0.0000	0.0000	0.3214	0.9444	0.0556	0.0000	0.3571
0.1667	3.3750	0.4750	0.0556	0.5000	0.3333	0.0000	0.0000	0.2069	1.0000	0.0000	0.1034	0.2759
0.2500	3.2143	0.4429	0.0625	0.3125	0.4375	0.0000	0.0435	0.2174	1.0000	0.0000	0.0000	0.3043
0.2143	3.2500	0.4500	0.0714	0.3571	0.4286	0.0000	0.0000	0.0000	1.0000	0.0000	0.0667	0.0000
0.2105	3.5882	0.5176	0.0526	0.5263	0.3684	0.0000	0.0000	0.1071	1.0000	0.0000	0.1071	0.2143
0.1905	3.5263	0.5053	0.0476	0.4762	0.3333	0.0000	0.0000	0.0000	1.0000	0.0000	0.0870	0.0000
0.1905	3.3684	0.4737	0.0476	0.5238	0.3333	0.0000	0.0000	0.1852	1.0000	0.0000	0.0000	0.2222
0.1765	3.8667	0.5733	0.0000	0.6471	0.2941	0.0000	0.0400	0.1600	1.0000	0.0000	0.0800	0.2400
0.1579	3.5000	0.5000	0.0526	0.6316	0.3158	0.0000	0.0000	0.0909	1.0000	0.0000	0.0000	0.1364
0.2381	3.7500	0.5500	0.0476	0.6667	0.2857	0.0000	0.0000	0.1852	1.0000	0.0000	0.0370	0.1852
0.1765	3.4667	0.4933	0.0588	0.5294	0.2941	0.0000	0.0400	0.1600	1.0000	0.0000	0.0800	0.2400
0.1579	3.3750	0.4750	0.0526	0.5263	0.3158	0.0000	0.0000	0.2222	1.0000	0.0000	0.0370	0.2593
0.1667	3.8000	0.5600	0.0000	0.5556	0.3333	0.0556	0.0000	0.1538	1.0000	0.0000	0.1154	0.1923
0.1667	3.3125	0.4625	0.0000	0.6667	0.2778	0.0000	0.0385	0.1923	1.0000	0.0000	0.0385	0.2692
0.2188	3.6207	0.5241	0.0313	0.7500	0.1875	0.0000	0.0000	0.0952	0.9688	0.0313	0.0952	0.1429
0.2105	3.6667	0.5333	0.0000	0.6316	0.3684	0.0000	0.0357	0.2143	1.0000	0.0000	0.0357	0.2857
0.2222	3.5882	0.5176	0.0556	0.6111	0.3333	0.0000	0.0000	0.1250	1.0000	0.0000	0.0417	0.2083
0.1667	3.7273	0.5455	0.0000	0.7083	0.2500	0.0000	0.0000	0.1000	1.0000	0.0000	0.0333	0.1667
0.2143	3.6667	0.5333	0.0714	0.5714	0.2857	0.0000	0.0526	0.1053	0.9286	0.0000	0.1053	0.1579
0.3636	3.7000	0.5400	0.0000	0.5455	0.2727	0.0000	0.1111	0.1111	1.0000	0.0000	0.1667	0.2222
0.2500	3.3333	0.4667	0.0625	0.5000	0.3125	0.0000	0.0526	0.0526	1.0000	0.0000	0.0526	0.1053
0.2000	3.3000	0.4600	0.0000	0.4000	0.4000	0.0000	0.1333	0.2000	1.0000	0.0000	0.0000	0.3333
0.3077	3.2308	0.4462	0.1538	0.5385	0.2308	0.0000	0.1176	0.1176	1.0000	0.0000	0.0000	0.2353
0.2222	3.0000	0.4000	0.2222	0.4444	0.3333	0.0000	0.1250	0.2500	1.0000	0.0000	0.0625	0.3750
0.1176	3.1333	0.4267	0.2353	0.4706	0.2353	0.0000	0.0870	0.0870	0.8824	0.0588	0.0870	0.1739
0.1875	3.1875	0.4375	0.0625	0.6250	0.2500	0.0000	0.0500	0.1500	1.0000	0.0000	0.0000	0.2000
0.0909	3.0909	0.4182	0.0909	0.5455	0.2727	0.0000	0.1429	0.0714	1.0000	0.0000	0.0000	0.2143
0.2000	3.0000	0.4000	0.0000	0.6000	0.3000	0.0000	0.0833	0.0000	0.9000	0.0000	0.0833	0.0833
0.2727	3.1818	0.4364	0.0909	0.5455	0.2727	0.0000	0.0667	0.2000	0.9091	0.0000	0.0000	0.2667
0.1667	3.2941	0.4588	0.1667	0.5556	0.1667	0.0000	0.0417	0.1250	0.9444	0.0000	0.0833	0.1667
0.1538	3.0833	0.4167	0.3077	0.3846	0.3077	0.0000	0.0556	0.1111	1.0000	0.0000	0.1111	0.1667
0.1765	3.3750	0.4750	0.1176	0.6471	0.2353	0.0000	0.0909	0.0909	0.9412	0.0000	0.0455	0.1818
0.1667	3.2222	0.4444	0.1111	0.4444	0.3333	0.0000	0.1111	0.1111	1.0000	0.0000	0.1111	0.2222
0.2000	3.0000	0.4000	0.2000	0.4667	0.2667	0.0000	0.1364	0.1818	1.0000	0.0000	0.0000	0.3182
0.1667	3.0833	0.4167	0.2500	0.4167	0.2500	0.0000	0.1579	0.1053	1.0000	0.0000	0.1053	0.2632
0.1818	3.0000	0.4000	0.2727	0.3636	0.2727	0.0000	0.1250	0.1250	1.0000	0.0000	0.0625	0.2500
0.0833	3.1818	0.4364	0.2500	0.5000	0.1667	0.0000	0.0625	0.1250	1.0000	0.0000	0.0625	0.1875
0.3000	3.3750	0.4750	0.1000	0.3000	0.2000	0.0000	0.0588	0.1765	0.9000	0.0000	0.1765	0.2353
0.1250	2.7143	0.3429	0.2500	0.1250	0.2500	0.0000	0.0909	0.0909	1.0000	0.0000	0.0909	0.1818
0.2222	3.3750	0.4750	0.2222	0.2222	0.2222	0.0000	0.1667	0.0833	1.0000	0.0000	0.0000	0.2500
0.1111	3.3333	0.4667	0.2222	0.3333	0.2222	0.0000	0.2308	0.0769	1.0000	0.0000	0.0000	0.3077
0.2727	3.4444	0.4889	0.1818	0.2727	0.2727	0.0000	0.1000	0.0500	1.0000	0.0000	0.3000	0.1500
0.2727	3.5000	0.5000	0.1818	0.2727	0.2727	0.0000	0.1765	0.0588	1.0000	0.0000	0.1176	0.2353
0.1667	3.0909	0.4182	0.1667	0.3333	0.1667	0.0000	0.1333	0.0667	1.0000	0.0000	0.0000	0.2000
0.3333	3.4444	0.4889	0.1111	0.2222	0.3333	0.0000	0.1538	0.0769	1.0000	0.0000	0.0769	0.2308
0.2000	3.4000	0.4800	0.1000	0.6000	0.1000	0.0000	0.0714	0.0000	0.9000	0.1000	0.1429	0.1429
0.2000	3.4000	0.4800	0.1000	0.4000	0.2000	0.0000	0.0769	0.0769	1.0000	0.0000	0.0769	0.1538
0.3000	3.4444	0.4889	0.2000	0.3000	0.2000	0.0000	0.0667	0.2000	1.0000	0.0000	0.0667	0.2667
0.3333	3.2500	0.4500	0.3333	0.1111	0.2222	0.0000	0.0909	0.0000	1.0000	0.0000	0.0909	0.0909
0.2308	3.1667	0.4333	0.0769	0.4615	0.2308	0.0000	0.0526	0.1053	0.9231	0.0769	0.1579	0.1579

0.0667	3.0000	0.4000	0.1333	0.6000	0.2000	0.0000	0.1724	0.1724	1.0000	0.0000	0.1034	0.3793
0.1111	3.0000	0.4000	0.2222	0.3333	0.1111	0.0000	0.0714	0.1429	0.8889	0.1111	0.1429	0.2143
0.2222	3.4444	0.4889	0.1111	0.3333	0.2222	0.0000	0.0000	0.0000	1.0000	0.0000	0.0000	0.0000
0.2727	3.2000	0.4400	0.0909	0.3636	0.2727	0.0000	0.1111	0.2222	1.0000	0.0000	0.0556	0.3333
0.1000	3.2000	0.4400	0.1000	0.4000	0.2000	0.0000	0.0769	0.0769	0.9000	0.1000	0.0769	0.1538
0.1818	3.0909	0.4182	0.0909	0.4545	0.1818	0.0000	0.0833	0.0000	0.9091	0.0909	0.0000	0.0833
0.1250	3.5000	0.5000	0.1250	0.3750	0.2500	0.0000	0.1667	0.0833	1.0000	0.0000	0.0833	0.2500
0.2000	3.3000	0.4600	0.1000	0.3000	0.2000	0.0000	0.1667	0.0000	1.0000	0.0000	0.0000	0.1667
0.1111	3.3333	0.4667	0.1111	0.3333	0.2222	0.0000	0.2308	0.0769	1.0000	0.0000	0.0000	0.3077
0.0714	3.3077	0.4615	0.0714	0.5714	0.1429	0.0000	0.1053	0.0000	0.9286	0.0714	0.1579	0.1053
0.0909	3.1818	0.4364	0.0909	0.4545	0.1818	0.0000	0.2353	0.0588	0.9091	0.0909	0.0588	0.2941
0.2143	3.1429	0.4286	0.0714	0.5000	0.1429	0.0000	0.1176	0.0000	0.9286	0.0714	0.0000	0.1765
0.1667	3.2500	0.4500	0.0833	0.4167	0.1667	0.0000	0.1250	0.0625	0.9167	0.0833	0.0625	0.1875
0.0625	2.9375	0.3875	0.1875	0.5625	0.1250	0.0000	0.1429	0.0000	0.9375	0.0625	0.0952	0.1429
0.0714	3.0000	0.4000	0.0714	0.6429	0.1429	0.0000	0.2273	0.0455	0.9286	0.0714	0.0909	0.2727
0.0714	3.0714	0.4143	0.1429	0.4286	0.1429	0.0000	0.2105	0.0526	0.9286	0.0714	0.0000	0.2632
0.2143	3.1429	0.4286	0.0714	0.5714	0.1429	0.0000	0.1500	0.0500	0.9286	0.0714	0.1000	0.2000
0.0625	3.0000	0.4000	0.0625	0.6250	0.1250	0.0000	0.1250	0.0417	0.9375	0.0625	0.1250	0.2083
0.0476	2.8000	0.3600	0.2381	0.5714	0.0952	0.0000	0.1071	0.0357	0.9524	0.0476	0.1071	0.1429
0.0667	3.2308	0.4462	0.2000	0.6000	0.1333	0.0000	0.1364	0.0455	1.0000	0.0000	0.1364	0.1818
0.0625	3.0667	0.4133	0.1875	0.4375	0.1250	0.0000	0.1364	0.0455	0.9375	0.0625	0.0455	0.2273
0.0625	3.2667	0.4533	0.1250	0.6250	0.1250	0.0000	0.1500	0.0500	0.9375	0.0625	0.0000	0.2000
0.0714	3.1429	0.4286	0.1429	0.6429	0.2143	0.0000	0.1667	0.0556	0.9286	0.0714	0.0000	0.2222
0.1667	3.4545	0.4909	0.2500	0.4167	0.2500	0.0833	0.0000	0.0000	1.0000	0.0000	0.0000	0.0000
0.1667	3.4118	0.4824	0.1667	0.6111	0.1667	0.0556	0.0000	0.0000	1.0000	0.0000	0.0000	0.0000
0.3000	3.7000	0.5400	0.2000	0.4000	0.4000	0.0000	0.0000	0.0000	1.0000	0.0000	0.0000	0.0000
0.2000	3.6000	0.5200	0.2000	0.5333	0.2667	0.0000	0.0000	0.0000	1.0000	0.0000	0.0000	0.0000
0.1111	3.5882	0.5176	0.1667	0.6111	0.1667	0.0556	0.0000	0.0000	1.0000	0.0000	0.0000	0.0000
0.2500	3.9167	0.5833	0.0833	0.5833	0.3333	0.0000	0.0000	0.0000	1.0000	0.0000	0.0000	0.0000
0.1667	3.9091	0.5818	0.0833	0.5833	0.2500	0.0833	0.0000	0.0000	1.0000	0.0000	0.0000	0.0000
0.2222	3.5294	0.5059	0.1111	0.6667	0.1667	0.0556	0.0000	0.0000	1.0000	0.0000	0.0000	0.0000
0.2500	3.7500	0.5500	0.1250	0.6250	0.2500	0.0000	0.0000	0.0000	1.0000	0.0000	0.0000	0.0000
0.2500	3.6667	0.5333	0.1250	0.5000	0.3125	0.0625	0.0000	0.0000	1.0000	0.0000	0.0000	0.0000
0.1000	3.8889	0.5778	0.1000	0.6000	0.2000	0.1000	0.0000	0.0000	1.0000	0.0000	0.0000	0.0000
0.2500	4.1333	0.6267	0.0000	0.6875	0.2500	0.0625	0.0000	0.0000	1.0000	0.0000	0.0000	0.0000
0.2857	4.2143	0.6429	0.0000	0.6429	0.3571	0.0000	0.0000	0.0000	1.0000	0.0000	0.0000	0.0000
0.2857	4.2308	0.6462	0.0000	0.5714	0.3571	0.0714	0.0000	0.0000	1.0000	0.0000	0.0000	0.0000
0.3333	4.3636	0.6727	0.0833	0.4167	0.4167	0.0833	0.0000	0.0000	1.0000	0.0000	0.0000	0.0000
0.3846	4.5000	0.7000	0.0000	0.5385	0.3846	0.0769	0.0000	0.0000	1.0000	0.0000	0.0000	0.0000
0.1111	2.6875	0.3375	0.3333	0.5000	0.1111	0.0556	0.0000	0.0000	0.9444	0.0556	0.0000	0.0000
0.0909	2.5000	0.3000	0.4545	0.4545	0.0000	0.0909	0.0000	0.0000	0.9091	0.0909	0.0000	0.0000
0.1429	2.9231	0.3846	0.2857	0.5000	0.1429	0.0714	0.0000	0.0000	1.0000	0.0000	0.0000	0.0000
0.1111	2.7059	0.3412	0.2778	0.5556	0.1111	0.0556	0.0000	0.0000	0.9444	0.0556	0.0000	0.0000
0.1765	2.9375	0.3875	0.2353	0.5882	0.1176	0.0588	0.0000	0.0000	1.0000	0.0000	0.0556	0.0000
0.1765	2.9375	0.3875	0.2353	0.5882	0.1176	0.0588	0.0000	0.0000	1.0000	0.0000	0.0556	0.0000
0.1429	2.9231	0.3846	0.3571	0.5000	0.0714	0.0714	0.0000	0.0000	1.0000	0.0000	0.1250	0.0000
0.0588	2.7500	0.3500	0.2941	0.4706	0.1765	0.0588	0.0000	0.0000	1.0000	0.0000	0.0000	0.0000
0.1667	2.9091	0.3818	0.3333	0.4167	0.1667	0.0833	0.0000	0.0000	1.0000	0.0000	0.0000	0.0000
0.1333	2.8571	0.3714	0.3333	0.4667	0.1333	0.0667	0.0000	0.0000	1.0000	0.0000	0.1176	0.0000
0.1875	2.8667	0.3733	0.3125	0.5000	0.1250	0.0625	0.0000	0.0000	1.0000	0.0000	0.1111	0.0000
0.1667	2.9412	0.3882	0.2778	0.5556	0.1111	0.0556	0.0000	0.0000	1.0000	0.0000	0.0000	0.0000
0.1765	3.7500	0.5500	0.1765	0.5294	0.2353	0.0588	0.0000	0.0000	0.9412	0.0588	0.0000	0.0000
0.1818	3.8182	0.5636	0.0909	0.6364	0.2727	0.0000	0.0000	0.0000	1.0000	0.0000	0.0000	0.0000
0.0714	3.7143	0.5429	0.1429	0.6429	0.2143	0.0000	0.0000	0.0000	1.0000	0.0000	0.0667	0.0000
0.1818	3.7273	0.5455	0.0909	0.6364	0.2727	0.0000	0.0000	0.0000	1.0000	0.0000	0.0000	0.0000
0.2308	4.0000	0.6000	0.0769	0.6154	0.3077	0.0000	0.0000	0.0000	0.9231	0.0769	0.0714	0.0000
0.1667	3.6667	0.5333	0.2500	0.4167	0.3333	0.0000	0.0000	0.0000	1.0000	0.0000	0.0000	0.0000
0.2727	4.2727	0.6545	0.0000	0.6364	0.3636	0.0000	0.0000	0.0000	1.0000	0.0000	0.0000	0.0000
0.2727	4.5455	0.7091	0.0000	0.5455	0.4545	0.0000	0.0000	0.0000	1.0000	0.0000	0.0000	0.0000
0.1111	4.5000	0.7000	0.0000	0.5556	0.4444	0.0000	0.0000	0.0000	1.0000	0.0000	0.0000	0.0000
0.2105	4.1176	0.6235	0.0000	0.6842	0.2632	0.0526	0.0000	0.0000	1.0000	0.0000	0.0000	0.0000
0.2143	4.2857	0.6571	0.0000	0.5714	0.4286	0.0000	0.0000	0.0000	1.0000	0.0000	0.0000	0.0000
0.2222	3.7647	0.5529	0.1111	0.5000	0.3333	0.0556	0.0000	0.0000	1.0000	0.0000	0.0526	0.0000
0.1250	3.7333	0.5467	0.1875	0.5000	0.2500	0.0625	0.0000	0.0000	1.0000	0.0000	0.0000	0.0000
0.2105	3.5000	0.5000	0.1053	0.5789	0.2632	0.0526	0.0000	0.0000	0.9474	0.0526	0.0000	0.0000
0.1333	3.7857	0.5571	0.1333	0.5333	0.2667	0.0667	0.0000	0.0000	1.0000	0.0000	0.0000	0.0000
0.2000	4.0000	0.6000	0.0667	0.5333	0.3333	0.0667	0.0000	0.0000	1.0000	0.0000	0.0000	0.0000
0.1667	4.1111	0.6222	0.0000	0.5000	0.5000	0.0000	0.0000	0.0000	1.0000	0.0000	0.0000	0.0000
0.1875	3.8000	0.5600	0.1250	0.5000	0.3125	0.0625	0.0000	0.0000	1.0000	0.0000	0.0000	0.0000
0.2000	4.0000	0.6000	0.0000	0.5333	0.4000	0.0667	0.0000	0.0000	1.0000	0.0000	0.0000	0.0000
0.1875	4.0667	0.6133	0.0000	0.5625	0.4375	0.0000	0.0000	0.0000	0.9375	0.0625	0.0000	0.0000
0.1538	4.3333	0.6667	0.0000	0.5385	0.3846	0.0769	0.0000	0.0000	1.0000	0.0000	0.0000	0.0000
0.2000	3.6842	0.5368	0.1500	0.5000	0.3000	0.0500	0.0000	0.0000	1.0000	0.0000	0.0000	0.0000
0.2500	4.7143	0.7429	0.0000	0.3750	0.5000	0.1250	0.0000	0.0000	1.0000	0.0000	0.0000	0.0000
0.3333	4.6667	0.7333	0.0000	0.4444	0.5556	0.0000	0.0000	0.0000	1.0000	0.0000	0.0000	0.0000
0.3636	4.7000	0.7400	0.0000	0.5455	0.3636	0.0909	0.0000	0.0000	0.9091	0.0909	0.0000	0.0000
0.2500	4.6250	0.7250	0.0000	0.5000	0.5000	0.0000	0.0000	0.0000	1.0000	0.0000	0.0000	0.0000

0.2500	4.6364	0.7273	0.0000	0.4167	0.5000	0.0833	0.0000	0.0000	1.0000	0.0000	0.0000	0.0000
0.0000	NA	NA	0.0000	0.0000	0.0000	1.0000	0.2857	0.4286	1.0000	0.0000	0.1429	0.7143
0.1429	2.2857	0.2571	0.5714	0.2857	0.1429	0.0000	0.0833	0.1667	1.0000	0.0000	0.1667	0.2500
0.2727	2.9000	0.3800	0.2727	0.3636	0.2727	0.0909	0.1250	0.0625	1.0000	0.0000	0.1250	0.1875
0.0000	2.5714	0.3143	0.5000	0.1250	0.2500	0.1250	0.0000	0.2500	1.0000	0.0000	0.0833	0.2500
0.2000	3.8000	0.5600	0.0000	0.6000	0.4000	0.0000	0.2500	0.1250	1.0000	0.0000	0.0000	0.3750
0.2143	3.1538	0.4308	0.1429	0.5000	0.2857	0.0714	0.1500	0.1000	0.9286	0.0714	0.0500	0.2500
0.0000	2.4444	0.2889	0.3333	0.4444	0.2222	0.0000	0.1429	0.1429	1.0000	0.0000	0.0714	0.2857
0.1818	3.2000	0.4400	0.1818	0.5455	0.1818	0.0909	0.1250	0.1250	1.0000	0.0000	0.0625	0.2500
0.0625	3.2857	0.4571	0.1875	0.3750	0.3125	0.1250	0.0952	0.0476	0.9375	0.0625	0.0952	0.1429
0.0000	2.2727	0.2545	0.4167	0.4167	0.0833	0.0833	0.0714	0.0714	1.0000	0.0000	0.0000	0.1429
0.0000	3.5000	0.5000	0.0000	0.0000	1.0000	0.0000	0.0000	0.2500	1.0000	0.0000	0.2500	0.2500
0.0000	2.6667	0.3333	0.5000	0.1667	0.3333	0.0000	0.1818	0.1818	1.0000	0.0000	0.0909	0.3636
0.3333	3.5294	0.5059	0.1667	0.6111	0.1667	0.0556	0.0000	0.1818	1.0000	0.0000	0.0000	0.1818
0.2778	3.4118	0.4824	0.1667	0.6111	0.1667	0.0556	0.0000	0.1364	1.0000	0.0000	0.0455	0.1364
0.2500	3.4000	0.4800	0.1500	0.7000	0.1500	0.0000	0.0000	0.1600	1.0000	0.0000	0.0400	0.1600
0.1667	3.3636	0.4727	0.1250	0.6667	0.1250	0.0833	0.0000	0.0741	1.0000	0.0000	0.0370	0.0741
0.2778	3.4706	0.4941	0.1111	0.6667	0.1667	0.0556	0.0000	0.1429	0.9444	0.0556	0.0000	0.1429
0.1765	3.2353	0.4471	0.1176	0.7647	0.1176	0.0000	0.0000	0.1000	1.0000	0.0000	0.0500	0.1000
0.2857	3.3810	0.4762	0.0952	0.7143	0.1905	0.0000	0.0000	0.1250	1.0000	0.0000	0.0000	0.1250
0.2000	3.4737	0.4947	0.1000	0.7000	0.1500	0.0500	0.0000	0.2000	1.0000	0.0000	0.0000	0.2000
0.2500	3.8125	0.5625	0.0000	0.8125	0.1875	0.0000	0.0000	0.0588	1.0000	0.0000	0.0000	0.0588
0.2143	3.8462	0.5692	0.0714	0.7143	0.1429	0.0714	0.0000	0.0000	1.0000	0.0000	0.0000	0.0000
0.5000	3.6250	0.5250	0.1250	0.6250	0.2500	0.0000	0.0833	0.2500	1.0000	0.0000	0.0000	0.3333
0.2727	3.2000	0.4400	0.1818	0.6364	0.0909	0.0909	0.1176	0.2353	1.0000	0.0000	0.0000	0.3529
0.3500	3.8947	0.5789	0.0500	0.7500	0.1500	0.0500	0.0000	0.0000	0.9500	0.0500	0.0000	0.0000
0.1852	3.6400	0.5280	0.0741	0.7037	0.1852	0.0370	0.0000	0.1563	1.0000	0.0000	0.0000	0.1563
0.2000	3.7143	0.5429	0.0667	0.8000	0.0667	0.0667	0.0000	0.0000	0.9333	0.0667	0.0000	0.0000
0.2500	3.5217	0.5043	0.0833	0.7083	0.1667	0.0417	0.0606	0.1515	1.0000	0.0000	0.0303	0.2424
0.1053	3.1667	0.4333	0.1053	0.7368	0.1053	0.0526	0.0000	0.1739	1.0000	0.0000	0.0000	0.1739
0.1538	3.3333	0.4667	0.0769	0.7308	0.1538	0.0385	0.0000	0.1613	1.0000	0.0000	0.0000	0.1613
0.0833	3.2000	0.4400	0.1667	0.6667	0.0833	0.0833	0.0000	0.0000	0.9167	0.0000	0.0000	0.0000
0.1500	3.5263	0.5053	0.1500	0.6500	0.1500	0.0500	0.0000	0.1200	1.0000	0.0000	0.0800	0.1200

33 st_c	34 st_s	35 st_r
0.4058	0.4493	0.1449
0.3854	0.5104	0.1042
0.4359	0.3974	0.1667
0.4762	0.4048	0.1190
0.4365	0.4365	0.1270
0.4500	0.4000	0.1500
0.3438	0.5313	0.1250
0.4750	0.4250	0.1000
0.4667	0.4667	0.0667
0.3889	0.4556	0.1556
0.4048	0.4762	0.1190
0.3917	0.5417	0.0667
0.4259	0.4630	0.1111
0.4487	0.4103	0.1410
0.3981	0.4537	0.1481
0.4417	0.4917	0.0667
0.3611	0.5111	0.1278
0.4487	0.4487	0.1026
0.5098	0.3922	0.0980
0.4773	0.4318	0.0909
0.4762	0.4286	0.0952
0.4127	0.4603	0.1270
0.4737	0.4211	0.1053
0.4167	0.4608	0.1225
0.4278	0.4444	0.1278
0.4474	0.4474	0.1053
0.4321	0.4321	0.1358
0.4219	0.4531	0.1250
0.4611	0.3944	0.1444
0.4533	0.4133	0.1333
0.4375	0.4375	0.1250
0.4495	0.4192	0.1313
0.4691	0.3951	0.1358
0.4683	0.3730	0.1587
0.4028	0.4861	0.1111
0.4783	0.4130	0.1087
0.4667	0.4167	0.1167
0.4038	0.4808	0.1154
0.5167	0.3417	0.1417
0.4259	0.5000	0.0741
0.3810	0.5238	0.0952
0.4861	0.4861	0.0278
0.3333	0.5833	0.0833
0.4231	0.5769	0.0000
0.4123	0.5175	0.0702
0.4561	0.4298	0.1140
0.4386	0.4649	0.0965
0.4785	0.4140	0.1075
0.3467	0.5467	0.1067
0.4537	0.4259	0.1204
0.4035	0.4298	0.1667
0.5294	0.3529	0.1176
0.3819	0.5278	0.0903
0.4479	0.5104	0.0417
0.3851	0.4885	0.1264
0.4343	0.4343	0.1313
0.4885	0.3851	0.1264
0.5119	0.3690	0.1190
0.5247	0.3395	0.1358
0.4242	0.4242	0.1515
0.6250	0.2500	0.1250
0.5000	0.2500	0.2500
0.5833	0.2500	0.1667
0.6250	0.1250	0.2500
0.5000	0.3333	0.1667
0.5000	0.1667	0.3333
0.5000	0.1667	0.3333
0.7179	0.1795	0.1026
0.6795	0.1795	0.1410
0.6667	0.1667	0.1667
0.6667	0.1373	0.1961
0.6500	0.1250	0.2250
0.5758	0.2576	0.1667
0.7024	0.1667	0.1310
0.4583	0.3958	0.1458
0.3939	0.4848	0.1212

0.4394	0.4394	0.1212
0.4583	0.4583	0.0833
0.5208	0.3646	0.1146
0.5093	0.3704	0.1204
0.4510	0.4216	0.1275
0.5385	0.3462	0.1154
0.4444	0.3889	0.1667
0.4524	0.3810	0.1667
0.5000	0.3529	0.1471
0.5175	0.3596	0.1228
0.5667	0.3167	0.1167
0.4583	0.3333	0.2083
0.4583	0.4583	0.0833
0.4500	0.4000	0.1500
0.4583	0.3958	0.1458
0.4500	0.3500	0.2000
0.5333	0.3000	0.1667
0.4861	0.4028	0.1111
0.4744	0.4359	0.0897
0.4667	0.4167	0.1167
0.5417	0.2708	0.1875
0.5083	0.3083	0.1833
0.5351	0.2982	0.1667
0.5309	0.3086	0.1605
0.5417	0.3333	0.1250
0.5000	0.3125	0.1875
0.4917	0.3667	0.1417
0.5635	0.2778	0.1587
0.5217	0.3043	0.1739
0.4697	0.3561	0.1742
0.4444	0.4861	0.0694
0.4630	0.4074	0.1296
0.4688	0.4063	0.1250
0.4444	0.4778	0.0778
0.4630	0.4074	0.1296
0.4444	0.4444	0.1111
0.4394	0.4394	0.1212
0.4405	0.4405	0.1190
0.4896	0.3646	0.1458
0.5439	0.2807	0.1754
0.5000	0.3667	0.1333
0.4924	0.3561	0.1515
0.4861	0.4028	0.1111
0.5435	0.2609	0.1957
0.5088	0.3509	0.1404
0.5111	0.3778	0.1111
0.4667	0.4000	0.1333
0.4231	0.5000	0.0769
0.4333	0.3833	0.1833
0.4545	0.4091	0.1364
0.5000	0.5000	0.0000
0.4444	0.4444	0.1111
0.4444	0.4444	0.1111
0.4286	0.4286	0.1429
0.4487	0.4103	0.1410
0.4737	0.3421	0.1842
0.5625	0.3125	0.1250
0.5104	0.2917	0.1979
0.4583	0.3333	0.2083
0.4242	0.4697	0.1061
0.4833	0.4833	0.0333
0.5185	0.3519	0.1296
0.5729	0.1354	0.2917
0.5313	0.2500	0.2188
0.4103	0.4872	0.1026
0.5556	0.2222	0.2222
0.5000	0.2500	0.2500
0.5926	0.1852	0.2222
0.5139	0.2222	0.2639
0.5263	0.2368	0.2368
0.5637	0.1373	0.2990
0.5208	0.1667	0.3125
0.5463	0.2407	0.2130
0.5119	0.2976	0.1905
0.4314	0.2549	0.3137
0.5882	0.3235	0.0882
0.6500	0.2500	0.1000

0.7121	0.2121	0.0758
0.5294	0.3529	0.1176
0.5000	0.4167	0.0833
0.5000	0.3438	0.1563
0.6190	0.2857	0.0952
0.5733	0.2733	0.1533
0.5333	0.3333	0.1333
0.5833	0.3611	0.0556
0.5882	0.2941	0.1176
0.3958	0.4583	0.1458
0.5000	0.4167	0.0833
0.4907	0.3519	0.1574
0.5729	0.2917	0.1354
0.4524	0.4167	0.1310
0.4917	0.3417	0.1667
0.5435	0.3261	0.1304
0.5333	0.2833	0.1833
0.5429	0.3143	0.1429
0.4286	0.3810	0.1905
0.5321	0.3013	0.1667
0.4815	0.3704	0.1481
0.4167	0.4167	0.1667
0.6533	0.1733	0.1733
0.6452	0.1935	0.1613
0.6264	0.1609	0.2126
0.6518	0.1964	0.1518
0.5870	0.2826	0.1304
0.7826	0.1304	0.0870
0.7059	0.1176	0.1765
0.5333	0.3333	0.1333
0.6167	0.2917	0.0917
0.6061	0.3333	0.0606
0.5098	0.3922	0.0980
0.4493	0.0580	0.4928
0.6032	0.0556	0.3413
0.6012	0.2619	0.1369
0.6552	0.2414	0.1034
0.5486	0.2986	0.1528
0.6379	0.2241	0.1379
0.6429	0.2619	0.0952
0.6736	0.2569	0.0694
0.7153	0.2153	0.0694
0.5583	0.3083	0.1333
0.5926	0.3148	0.0926
0.5595	0.3095	0.1310
0.5250	0.3250	0.1500
0.4630	0.4630	0.0741
0.4259	0.4259	0.1481
0.5667	0.2917	0.1417
0.5500	0.3500	0.1000
0.5635	0.3254	0.1111
0.5455	0.3636	0.0909
0.5490	0.3725	0.0784
0.5385	0.3846	0.0769
0.6373	0.2549	0.1078
0.5800	0.3000	0.1200
0.6061	0.2879	0.1061
0.6049	0.2901	0.1049
0.5833	0.2708	0.1458
0.5583	0.3583	0.0833
0.5833	0.3333	0.0833
0.6548	0.1190	0.2262
0.5208	0.3542	0.1250
0.5914	0.2527	0.1559
0.5200	0.3200	0.1600
0.4683	0.3730	0.1587
0.5278	0.2183	0.2540
0.5980	0.2451	0.1569
0.5833	0.2440	0.1726
0.5833	0.1667	0.2500
0.5962	0.2692	0.1346
0.5606	0.2879	0.1515
0.6267	0.2267	0.1467
0.5667	0.2867	0.1467
0.5686	0.3039	0.1275
0.4556	0.3889	0.1556
0.4783	0.3913	0.1304

0.5000	0.5000	0.0000
0.4667	0.5167	0.0167
0.5652	0.2609	0.1739
0.6875	0.1875	0.1250
0.6750	0.1500	0.1750
0.5083	0.3083	0.1833
0.4444	0.4444	0.1111
0.5000	0.4375	0.0625
0.5870	0.2826	0.1304
0.6218	0.2179	0.1603
0.7632	0.1053	0.1316
0.6067	0.2067	0.1867
0.5351	0.3246	0.1404
0.4537	0.4537	0.0926
0.5917	0.1167	0.2917
0.5789	0.3158	0.1053
0.5333	0.3333	0.1333
0.7778	0.1111	0.1111
0.5139	0.4306	0.0556
0.6083	0.1583	0.2333
0.6090	0.1474	0.2436
0.6159	0.2899	0.0942
0.4722	0.4722	0.0556
0.5000	0.5000	0.0000
0.5606	0.1515	0.2879
0.5778	0.1111	0.3111
0.5938	0.1250	0.2813
0.7361	0.1111	0.1528
0.5588	0.3235	0.1176
0.6389	0.1111	0.2500
0.6111	0.1389	0.2500
0.5750	0.2250	0.2000
0.7564	0.1026	0.1410
0.6333	0.2333	0.1333
0.5417	0.3750	0.0833
0.4683	0.4444	0.0873
0.5500	0.3500	0.1000
0.8000	0.0000	0.2000
0.4722	0.3889	0.1389
0.5351	0.2456	0.2193
0.5000	0.2500	0.2500
0.5490	0.2255	0.2255
0.4825	0.4035	0.1140
0.5119	0.3690	0.1190
0.5000	0.2500	0.2500
0.5000	0.0000	0.5000
0.6667	0.1667	0.1667
0.6771	0.1458	0.1771
0.5833	0.2833	0.1333
0.5083	0.3833	0.1083
0.5000	0.2500	0.2500
0.3571	0.2857	0.3571
0.3519	0.2963	0.3519
0.3810	0.3810	0.2381
0.4667	0.4000	0.1333
0.7593	0.0926	0.1481
0.9000	0.0000	0.1000
0.6736	0.1319	0.1944
0.5686	0.2157	0.2157
0.4667	0.4667	0.0667
0.4792	0.4792	0.0417
0.4583	0.4583	0.0833
0.4091	0.4545	0.1364
0.4444	0.3611	0.1944
0.4762	0.4405	0.0833
0.4242	0.4697	0.1061
0.4722	0.3889	0.1389
0.5741	0.3519	0.0741
0.6197	0.1966	0.1838
0.5278	0.2111	0.2611
0.5962	0.1731	0.2308
0.5593	0.2815	0.1593
0.6056	0.1722	0.2222
0.5385	0.1346	0.3269
0.5833	0.1833	0.2333
0.5778	0.2000	0.2222
0.5772	0.1504	0.2724

0.5278	0.1667	0.3056
0.3772	0.0614	0.5614
0.5741	0.2407	0.1852
0.5694	0.2153	0.2153
0.6400	0.2000	0.1600
0.5450	0.2748	0.1802
0.6581	0.1966	0.1453
0.5965	0.2018	0.2018
0.6429	0.2143	0.1429
0.5463	0.2685	0.1852
0.6140	0.2456	0.1404
0.7188	0.1563	0.1250
0.6759	0.1481	0.1759
0.3095	0.5238	0.1667
0.4500	0.4500	0.1000
0.3596	0.4649	0.1754
0.4524	0.4524	0.0952
0.3596	0.5175	0.1228
0.3889	0.4556	0.1556
0.3417	0.4917	0.1667
0.4167	0.4167	0.1667
0.4568	0.3827	0.1605
0.4545	0.3939	0.1515
0.4556	0.3889	0.1556
0.4310	0.3966	0.1724
0.4383	0.4012	0.1605
0.4565	0.3696	0.1739
0.4583	0.3155	0.2262
0.4279	0.2523	0.3198
0.5000	0.2432	0.2568
0.4274	0.2479	0.3248
0.4306	0.2222	0.3472
0.4667	0.2000	0.3333
0.5000	0.2561	0.2439
0.5000	0.2143	0.2857
0.4510	0.3039	0.2451
0.4775	0.2342	0.2883
0.4250	0.2750	0.3000
0.6019	0.2269	0.1713
0.6204	0.2037	0.1759
0.6154	0.2308	0.1538
0.4952	0.2095	0.2952
0.5152	0.2121	0.2727
0.4924	0.1742	0.3333
0.5147	0.2500	0.2353
0.5679	0.2901	0.1420
0.4271	0.2396	0.3333
0.5052	0.3333	0.1615
0.4479	0.3854	0.1667
0.5000	0.3148	0.1852
0.3929	0.4643	0.1429
0.3939	0.3939	0.2121
0.5278	0.3194	0.1528
0.5278	0.3111	0.1611
0.5722	0.3056	0.1222
0.5690	0.3103	0.1207
0.5625	0.2875	0.1500
0.4875	0.3250	0.1875
0.6000	0.2833	0.1167
0.5509	0.3426	0.1065
0.4833	0.3333	0.1833
0.4808	0.3654	0.1538
0.4615	0.3846	0.1538
0.5190	0.3048	0.1762
0.5385	0.3077	0.1538
0.4583	0.3958	0.1458
0.4630	0.3148	0.2222
0.6333	0.2083	0.1583
0.6019	0.2685	0.1296
0.4878	0.3049	0.2073
0.4710	0.3841	0.1449
0.4667	0.3667	0.1667
0.5139	0.3056	0.1806
0.5167	0.3292	0.1542
0.6889	0.1222	0.1889
0.6000	0.3000	0.1000
0.6667	0.1190	0.2143

0.7778	0.0635	0.1587
0.7014	0.1597	0.1389
0.7157	0.1569	0.1275
0.8333	0.1333	0.0333
0.7302	0.1587	0.1111
0.6574	0.1852	0.1574
0.7083	0.2083	0.0833
0.8077	0.0769	0.1154
0.6481	0.1481	0.2037
0.6574	0.1852	0.1574
0.6563	0.1563	0.1875
0.3810	0.3095	0.3095
0.7381	0.0952	0.1667
0.5000	0.1667	0.3333
0.5000	0.1667	0.3333
0.5000	0.1667	0.3333
0.4259	0.4074	0.1667
0.5185	0.3519	0.1296
0.5303	0.3258	0.1439
0.6228	0.2807	0.0965
0.5278	0.2778	0.1944
0.5952	0.1667	0.2381
0.5444	0.3111	0.1444
0.5641	0.2564	0.1795
0.5667	0.2917	0.1417
0.7361	0.1111	0.1528
0.6333	0.1833	0.1833
0.5595	0.2738	0.1667
0.5172	0.3276	0.1552
0.5556	0.2556	0.1889
0.5294	0.3235	0.1471
0.4855	0.2899	0.2246
0.5556	0.2500	0.1944
0.5000	0.2708	0.2292
0.5600	0.2600	0.1800
0.4500	0.3500	0.2000
0.5614	0.2719	0.1667
0.6354	0.2292	0.1354
0.4938	0.3086	0.1975
0.5379	0.2424	0.2197
0.5000	0.2500	0.2500
0.4783	0.3043	0.2174
0.5069	0.2778	0.2153
0.5145	0.3188	0.1667
0.5000	0.3103	0.1897
0.5278	0.2361	0.2361
0.5641	0.2179	0.2179
0.5980	0.2157	0.1863
0.6458	0.1771	0.1771
0.5476	0.2262	0.2262
0.5490	0.2255	0.2255
0.6204	0.2037	0.1759
0.5583	0.2333	0.2083
0.2778	0.6111	0.1111
0.2813	0.5938	0.1250
0.4286	0.5714	0.0000
0.4286	0.5714	0.0000
0.4259	0.5370	0.0370
0.4286	0.5714	0.0000
0.4167	0.5417	0.0417
0.4000	0.6000	0.0000
0.4167	0.5833	0.0000
0.5000	0.5000	0.0000
0.4792	0.4792	0.0417
0.4167	0.5833	0.0000
0.3889	0.5556	0.0556
0.5000	0.5000	0.0000
0.4091	0.4091	0.1818
0.5741	0.2130	0.2130
0.5952	0.3452	0.0595
0.4722	0.4722	0.0556
0.5870	0.3478	0.0652
0.8000	0.2000	0.0000
0.4667	0.4667	0.0667
0.4333	0.4333	0.1333
0.5000	0.5000	0.0000
0.4444	0.2302	0.3254

0.5417	0.4167	0.0417
0.5556	0.2222	0.2222
0.4074	0.4074	0.1852
0.3889	0.3889	0.2222
0.4630	0.3519	0.1852
0.4444	0.4444	0.1111
0.5000	0.5000	0.0000
0.4881	0.4881	0.0238
0.6667	0.1667	0.1667
0.6000	0.4000	0.0000
0.5641	0.3333	0.1026
0.5625	0.4375	0.0000
0.5500	0.3000	0.1500
0.4583	0.2083	0.3333
0.5833	0.2083	0.2083
0.6667	0.3333	0.0000
0.4667	0.4667	0.0667
0.5417	0.3854	0.0729
0.6404	0.3246	0.0351
0.6754	0.2544	0.0702
0.5000	0.5000	0.0000
0.4667	0.2667	0.2667
0.5083	0.3583	0.1333
0.5139	0.3472	0.1389
0.4861	0.3611	0.1528
0.4747	0.3990	0.1263
0.5057	0.3333	0.1609
0.5143	0.3714	0.1143
0.5256	0.3333	0.1410
0.4608	0.3578	0.1814
0.5667	0.3267	0.1067
0.5595	0.3095	0.1310
0.5794	0.3651	0.0556
0.5521	0.3333	0.1146
0.7556	0.0889	0.1556
0.6905	0.1190	0.1905
0.7037	0.1481	0.1481
0.6742	0.2424	0.0833
0.6410	0.1795	0.1795
0.5000	0.3000	0.2000
0.6275	0.1863	0.1863
0.6042	0.1979	0.1979
0.6061	0.1970	0.1970
0.6591	0.2955	0.0455
0.6333	0.2833	0.0833
0.5833	0.3333	0.0833
0.6806	0.1806	0.1389
0.5517	0.3103	0.1379
0.6417	0.2417	0.1167
0.5119	0.4405	0.0476
0.7222	0.1389	0.1389
0.6786	0.1429	0.1786
0.6190	0.2738	0.1071
0.6667	0.2121	0.1212
0.5556	0.2778	0.1667
0.5455	0.2727	0.1818
0.5139	0.3056	0.1806
0.5667	0.3667	0.0667
0.5694	0.3611	0.0694
0.5139	0.2222	0.2639
0.4792	0.1667	0.3542
0.4861	0.3194	0.1944
0.4792	0.2917	0.2292
0.5556	0.3056	0.1389
0.5833	0.2500	0.1667
0.5227	0.3409	0.1364
0.4833	0.2833	0.2333
0.5000	0.1667	0.3333
0.4242	0.3333	0.2424
0.5111	0.2778	0.2111
0.4861	0.2778	0.2361
0.4667	0.1667	0.3667
0.4667	0.1667	0.3667
0.5000	0.0000	0.5000
0.4815	0.3148	0.2037
0.5000	0.1667	0.3333
0.4762	0.3333	0.1905

0.3922	0.3922	0.2157
0.5238	0.2738	0.2024
0.5333	0.3733	0.0933
0.5521	0.2083	0.2396
0.7083	0.0833	0.2083
0.5980	0.1863	0.2157
0.6667	0.1667	0.1667
0.7143	0.1429	0.1429
0.6250	0.2500	0.1250
0.6000	0.2000	0.2000
0.6111	0.2778	0.1111
0.5769	0.3462	0.0769
0.4792	0.3542	0.1667
0.5333	0.3333	0.1333
0.4259	0.4259	0.1481
0.5833	0.3333	0.0833
0.4286	0.4286	0.1429
0.4167	0.4167	0.1667
0.3889	0.3889	0.2222
0.4259	0.4259	0.1481
0.4444	0.4444	0.1111
0.4524	0.4524	0.0952
0.4375	0.4375	0.1250
0.4259	0.4259	0.1481
0.4861	0.4028	0.1111
0.3889	0.5000	0.1111
0.5833	0.3333	0.0833
0.6250	0.3750	0.0000
0.5000	0.3333	0.1667
0.4167	0.4167	0.1667
0.5000	0.3750	0.1250
0.4286	0.4286	0.1429
0.3333	0.4762	0.1905
0.3571	0.5000	0.1429
0.4048	0.4048	0.1905
0.4333	0.4333	0.1333
0.5000	0.4091	0.0909
0.3000	0.5000	0.2000
0.4286	0.4286	0.1429
0.3889	0.5000	0.1111
0.4167	0.4167	0.1667
0.5000	0.3750	0.1250
0.5667	0.3667	0.0667
0.4167	0.4167	0.1667
0.4583	0.4583	0.0833
0.5556	0.3889	0.0556
0.4444	0.4444	0.1111
0.4375	0.4375	0.1250
0.3750	0.5000	0.1250
0.4286	0.4286	0.1429
0.3939	0.4848	0.1212
0.4000	0.4000	0.2000
0.3889	0.5000	0.1111
0.3750	0.5000	0.1250
0.3974	0.4744	0.1282
0.3889	0.5000	0.1111
0.3571	0.5000	0.1429
0.3000	0.5000	0.2000
0.4667	0.4667	0.0667
0.4167	0.4167	0.1667
0.4167	0.4167	0.1667
0.3571	0.5000	0.1429
0.3750	0.5000	0.1250
0.4444	0.4444	0.1111
0.3611	0.5278	0.1111
0.3333	0.5000	0.1667
0.4167	0.4167	0.1667
0.3571	0.5000	0.1429
0.3333	0.5000	0.1667
0.3889	0.5000	0.1111
0.3958	0.5208	0.0833
0.4167	0.4167	0.1667
0.5333	0.3333	0.1333
0.6111	0.2778	0.1111
0.5833	0.3333	0.0833
0.4444	0.4444	0.1111
0.4167	0.4167	0.1667

0.4167	0.4167	0.1667
0.5000	0.5000	0.0000
0.5833	0.3333	0.0833
0.6111	0.2778	0.1111
0.5667	0.3667	0.0667
0.3667	0.5667	0.0667
0.4167	0.4167	0.1667
0.5000	0.3750	0.1250
0.4524	0.4524	0.0952
0.6111	0.2778	0.1111
0.3333	0.5833	0.0833
0.4306	0.4306	0.1389
0.4848	0.3939	0.1212
0.3333	0.5833	0.0833
0.5000	0.2500	0.2500
0.5000	0.2500	0.2500
0.5000	0.5000	0.0000
0.5000	0.3750	0.1250
0.3333	0.6667	0.0000
0.2500	0.7500	0.0000
0.5000	0.5000	0.0000
0.5000	0.0000	0.5000
0.4444	0.4444	0.1111
0.5000	0.5000	0.0000
0.5000	0.5000	0.0000
0.4667	0.4667	0.0667
0.5000	0.5000	0.0000
0.5000	0.5000	0.0000
0.5000	0.5000	0.0000
0.5000	0.5000	0.0000
0.7500	0.2500	0.0000
0.4167	0.4167	0.1667
0.5000	0.5000	0.0000
0.3889	0.3889	0.2222
0.5000	0.5000	0.0000
0.3333	0.3333	0.3333
0.5000	0.5000	0.0000
0.5000	0.5000	0.0000
0.5000	0.5000	0.0000
0.5000	0.5000	0.0000
0.3333	0.6667	0.0000
0.5000	0.5000	0.0000
0.5000	0.5000	0.0000
0.6296	0.2963	0.0741
0.4167	0.4167	0.1667
0.4167	0.4167	0.1667
0.5000	0.5000	0.0000
0.5000	0.5000	0.0000
0.5000	0.5000	0.0000
0.4444	0.4444	0.1111
0.4048	0.4762	0.1190
0.4667	0.4667	0.0667
0.4000	0.4000	0.2000
0.6190	0.3333	0.0476
0.4074	0.5185	0.0741
0.4630	0.3519	0.1852
0.5278	0.3611	0.1111
0.5000	0.5000	0.0000
0.5000	0.5000	0.0000
0.5000	0.5000	0.0000
0.5000	0.5000	0.0000
0.3333	0.6667	0.0000
0.5000	0.5000	0.0000
0.5000	0.5000	0.0000
0.3889	0.5556	0.0556
0.5000	0.5000	0.0000
0.4333	0.4333	0.1333
0.4815	0.3148	0.2037
0.5417	0.2917	0.1667
0.4722	0.4722	0.0556
0.3958	0.5208	0.0833
0.5000	0.5000	0.0000
0.4167	0.4167	0.1667
0.3889	0.3889	0.2222
0.4444	0.4444	0.1111
0.5000	0.5000	0.0000

0.5000	0.5000	0.0000
0.5000	0.5000	0.0000
0.5000	0.5000	0.0000
0.5000	0.5000	0.0000
0.5000	0.5000	0.0000
0.4259	0.5370	0.0370
0.3333	0.6061	0.0606
0.3939	0.4848	0.1212
0.2500	0.7500	0.0000
0.2778	0.6111	0.1111
0.2000	0.8000	0.0000
0.3889	0.5000	0.1111
0.4048	0.4048	0.1905
0.3125	0.5625	0.1250
0.3148	0.6481	0.0370
0.3333	0.6333	0.0333
0.2778	0.6111	0.1111
0.3056	0.6389	0.0556
0.3095	0.5952	0.0952
0.2963	0.6296	0.0741
0.4667	0.4667	0.0667
0.4000	0.5000	0.1000
0.3846	0.4615	0.1538
0.4333	0.5333	0.0333
0.3889	0.5000	0.1111
0.4545	0.4545	0.0909
0.3519	0.5741	0.0741
0.4306	0.5139	0.0556
0.3889	0.5556	0.0556
0.3788	0.5606	0.0606
0.3704	0.5926	0.0370
0.4167	0.5167	0.0667
0.4167	0.4167	0.1667
0.3333	0.5000	0.1667
0.4444	0.4444	0.1111
0.3333	0.6667	0.0000
0.4333	0.4333	0.1333
0.3333	0.6667	0.0000
0.3667	0.5667	0.0667
0.0000	1.0000	0.0000
0.3333	0.5833	0.0833
0.4048	0.5476	0.0476
0.3889	0.5556	0.0556
0.5000	0.5000	0.0000

0.5000	0.5000	0.0000
0.5000	0.5000	0.0000
0.5000	0.5000	0.0000
0.5000	0.5000	0.0000
0.5000	0.5000	0.0000
0.5000	0.5000	0.0000
0.5000	0.5000	0.0000
0.5000	0.5000	0.0000
0.5000	0.5000	0.0000
0.5000	0.5000	0.0000
0.5000	0.5000	0.0000
0.4762	0.4762	0.0476
0.5000	0.5000	0.0000
0.2778	0.6111	0.1111

0.5000	0.5000	0.0000
0.4259	0.5370	0.0370
0.4375	0.5625	0.0000
0.3333	0.6190	0.0476
0.0000	1.0000	0.0000
0.3704	0.5926	0.0370
0.3000	0.6000	0.1000
0.2667	0.6667	0.0667
0.3182	0.5909	0.0909
0.5000	0.5000	0.0000
0.3182	0.5909	0.0909
0.4333	0.5333	0.0333
0.5000	0.5000	0.0000
0.3750	0.6250	0.0000
0.4000	0.6000	0.0000
0.3333	0.6667	0.0000
0.4444	0.5556	0.0000

0.4000	0.6000	0.0000
0.5000	0.5000	0.0000
0.5000	0.5000	0.0000
0.5000	0.5000	0.0000
0.5000	0.5000	0.0000
0.5000	0.5000	0.0000
0.3889	0.3889	0.2222

0.5000	0.5000	0.0000
0.5000	0.5000	0.0000
0.5000	0.5000	0.0000
0.2500	0.7500	0.0000
0.5000	0.5000	0.0000
0.5000	0.5000	0.0000
0.5000	0.5000	0.0000
0.4167	0.5417	0.0417

0.6400	0.1800	0.1800
0.6154	0.1731	0.2115
0.5833	0.1795	0.2372
0.6198	0.1823	0.1979
0.6087	0.1957	0.1957
0.5942	0.2246	0.1812
0.6042	0.2083	0.1875
0.6181	0.2222	0.1597
0.5476	0.2857	0.1667
0.6111	0.2222	0.1667
0.5988	0.2469	0.1543
0.5611	0.2611	0.1778
0.5862	0.2241	0.1897
0.5303	0.2727	0.1970
0.5556	0.2222	0.2222
0.6250	0.1500	0.2250
0.6667	0.1859	0.1474
0.5903	0.1944	0.2153
0.5667	0.1867	0.2467
0.5877	0.2193	0.1930
0.6212	0.1894	0.1894
0.6667	0.1894	0.1439
0.6667	0.1867	0.1467
0.5769	0.1923	0.2308
0.5985	0.2121	0.1894
0.6061	0.1970	0.1970
0.5833	0.2424	0.1742
0.6090	0.2244	0.1667
0.6026	0.2372	0.1603
0.5062	0.2654	0.2284
0.6500	0.2500	0.1000
0.6667	0.2143	0.1190
0.6825	0.1825	0.1349
0.5909	0.2727	0.1364
0.6429	0.2143	0.1429
0.6667	0.2101	0.1232
0.5805	0.2701	0.1494
0.5625	0.2708	0.1667
0.6290	0.2258	0.1452
0.5449	0.2756	0.1795
0.5494	0.3086	0.1420
0.6111	0.2611	0.1278
0.5538	0.2634	0.1828
0.5686	0.2745	0.1569
0.4938	0.3272	0.1790
0.4938	0.3457	0.1605
0.5556	0.3175	0.1270
0.5897	0.2628	0.1474
0.6250	0.2500	0.1250
0.5460	0.2874	0.1667
0.6042	0.2448	0.1510
0.5655	0.2798	0.1548
0.5484	0.2742	0.1774
0.5667	0.3000	0.1333
0.5365	0.2708	0.1927
0.6439	0.2348	0.1212
0.5714	0.2857	0.1429
0.6061	0.2424	0.1515
0.6667	0.2222	0.1111

0.5889	0.2556	0.1556
0.5833	0.2083	0.2083
0.4375	0.2500	0.3125
0.5347	0.3472	0.1181
0.4286	0.4762	0.0952
0.5000	0.4000	0.1000
0.5256	0.4487	0.0256
0.5333	0.4333	0.0333
0.5556	0.4444	0.0000
0.6282	0.1667	0.2051
0.6176	0.3824	0.0000
0.5595	0.3095	0.1310
0.5741	0.2407	0.1852
0.4603	0.4365	0.1032
0.5417	0.3333	0.1250
0.4861	0.4444	0.0694
0.5333	0.3833	0.0833
0.4722	0.3889	0.1389
0.5417	0.3333	0.1250
0.6042	0.2604	0.1354
0.4815	0.4259	0.0926
0.5185	0.2963	0.1852
0.5000	0.4286	0.0714
0.6481	0.1481	0.2037
0.6875	0.1250	0.1875
0.5556	0.3056	0.1389
0.5263	0.3158	0.1579
0.6111	0.2778	0.1111
1.0000	0.0000	0.0000
0.8000	0.1000	0.1000
0.5667	0.2000	0.2333
0.7917	0.0667	0.1417
0.6563	0.1563	0.1875
0.7051	0.1282	0.1667
0.5909	0.1818	0.2273
0.5833	0.2424	0.1742
0.7188	0.1250	0.1563
0.6471	0.2059	0.1471
0.7000	0.1750	0.1250
0.4924	0.2879	0.2197
0.5208	0.2083	0.2708
0.6778	0.1444	0.1778
0.7821	0.1667	0.0513
0.4722	0.3056	0.2222
0.6042	0.1667	0.2292
0.6417	0.2167	0.1417
0.5972	0.2639	0.1389
0.8333	0.1667	0.0000
0.3958	0.3958	0.2083
0.5385	0.3077	0.1538
0.4000	0.3667	0.2333
0.5938	0.1250	0.2813
0.6032	0.2222	0.1746
0.5455	0.2045	0.2500
0.6667	0.1282	0.2051
0.6667	0.1875	0.1458
0.6111	0.1778	0.2111
0.5877	0.1930	0.2193
0.7121	0.1212	0.1667
0.5965	0.2807	0.1228
0.5702	0.2281	0.2018
0.6071	0.2143	0.1786
0.7255	0.1373	0.1373
0.5833	0.2396	0.1771
0.6429	0.2143	0.1429
0.5889	0.2556	0.1556
0.5392	0.2157	0.2451
0.6111	0.2778	0.1111
0.4762	0.2619	0.2619
0.3889	0.2556	0.3556
0.5000	0.3182	0.1818
0.5119	0.2619	0.2262
0.7255	0.1373	0.1373
0.5088	0.2193	0.2719
0.5725	0.1594	0.2681
0.6795	0.1987	0.1218
0.6806	0.1389	0.1806

0.5000	0.1944	0.3056
0.6667	0.1667	0.1667
0.6333	0.1333	0.2333
0.5000	0.2500	0.2500
0.6111	0.1944	0.1944
0.5000	0.2222	0.2778
0.6190	0.2976	0.0833
0.5175	0.2807	0.2018
0.7361	0.1528	0.1111
0.6071	0.2500	0.1429
0.5119	0.4048	0.0833
0.6875	0.1250	0.1875
0.5238	0.4524	0.0238
0.6000	0.2667	0.1333
0.6111	0.2778	0.1111
0.5476	0.1905	0.2619
0.5256	0.3333	0.1410
0.7813	0.1250	0.0938
0.7500	0.1111	0.1389
0.7121	0.1212	0.1667
0.7727	0.1364	0.0909
0.7500	0.1250	0.1250
0.5778	0.2111	0.2111
0.6250	0.2188	0.1563
0.5439	0.3333	0.1228
0.3667	0.5667	0.0667
0.3889	0.6111	0.0000
0.3958	0.5208	0.0833
0.3333	0.6061	0.0606
0.4048	0.5476	0.0476
0.4167	0.5833	0.0000
0.3750	0.6250	0.0000
0.3571	0.6429	0.0000
0.4286	0.5714	0.0000
0.5000	0.5000	0.0000
0.3889	0.6111	0.0000
0.5000	0.5000	0.0000
0.4286	0.5714	0.0000
0.4500	0.5500	0.0000
0.4091	0.5909	0.0000
0.4762	0.4762	0.0476
0.4167	0.5417	0.0417
0.5000	0.5000	0.0000
0.3958	0.5208	0.0833
0.3958	0.5208	0.0833
0.4444	0.4444	0.1111
0.5000	0.5000	0.0000
0.5000	0.5000	0.0000
0.4167	0.5417	0.0417
0.4000	0.6000	0.0000
0.5000	0.5000	0.0000
0.4000	0.6000	0.0000
0.5000	0.5000	0.0000
0.5000	0.5000	0.0000
0.4286	0.5714	0.0000
0.5000	0.5000	0.0000
0.4167	0.5833	0.0000
0.5000	0.5000	0.0000
0.4286	0.5714	0.0000
0.3000	0.7000	0.0000
0.4583	0.4583	0.0833
0.5000	0.5000	0.0000
0.5000	0.5000	0.0000
0.5000	0.5000	0.0000
0.4167	0.5833	0.0000
0.3571	0.6429	0.0000
0.4286	0.5714	0.0000
0.4286	0.5714	0.0000
0.4375	0.5625	0.0000
0.5000	0.5000	0.0000
0.5000	0.5000	0.0000
0.5000	0.5000	0.0000
0.4762	0.4762	0.0476
0.5000	0.5000	0.0000
0.5000	0.5000	0.0000
0.5000	0.5000	0.0000
0.3571	0.6429	0.0000

0.5000	0.5000	0.0000
0.4000	0.6000	0.0000
0.4167	0.5833	0.0000
0.4286	0.5714	0.0000
0.5000	0.5000	0.0000
0.4000	0.6000	0.0000
0.5000	0.5000	0.0000
0.5000	0.5000	0.0000
0.4286	0.5714	0.0000
0.5000	0.5000	0.0000
0.5000	0.5000	0.0000
0.5000	0.5000	0.0000
0.4000	0.6000	0.0000
0.5000	0.5000	0.0000
0.5000	0.5000	0.0000
0.4000	0.6000	0.0000
0.4000	0.6000	0.0000
0.4167	0.5833	0.0000
0.3542	0.6042	0.0417
0.4048	0.5476	0.0476
0.4630	0.4630	0.0741
0.4000	0.6000	0.0000
0.3485	0.6212	0.0303
0.3056	0.6389	0.0556
0.3333	0.5333	0.1333
0.4259	0.5370	0.0370
0.3958	0.5208	0.0833
0.4333	0.4333	0.1333
0.4048	0.5476	0.0476
0.4848	0.4848	0.0303
0.4286	0.5714	0.0000
0.5000	0.5000	0.0000
0.4286	0.5714	0.0000
0.5000	0.5000	0.0000
0.5000	0.4286	0.0714
0.5000	0.5000	0.0000
0.4000	0.6000	0.0000
0.5000	0.5000	0.0000
0.5000	0.5000	0.0000
NA	NA	NA
0.5000	0.5000	0.0000
0.5000	0.5000	0.0000
0.5000	0.5000	0.0000
NA	NA	NA
0.5000	0.5000	0.0000
0.5000	0.5000	0.0000
0.3750	0.6250	0.0000
0.5000	0.5000	0.0000
0.5000	0.5000	0.0000
0.5000	0.5000	0.0000
0.5000	0.5000	0.0000
0.4000	0.6000	0.0000
0.5000	0.5000	0.0000
0.5000	0.5000	0.0000
0.3750	0.6250	0.0000
0.5000	0.5000	0.0000
0.5000	0.5000	0.0000
0.5000	0.5000	0.0000
0.5000	0.5000	0.0000
0.4000	0.6000	0.0000
0.5000	0.5000	0.0000
0.4000	0.6000	0.0000
0.5000	0.5000	0.0000
0.5000	0.5000	0.0000
0.6154	0.3077	0.0769
0.5625	0.3125	0.1250
0.5833	0.3333	0.0833
0.5490	0.3725	0.0784
0.5725	0.2899	0.1377
0.5267	0.3467	0.1267
0.5156	0.2969	0.1875
0.5432	0.3025	0.1543
0.5750	0.3250	0.1000
0.5476	0.3571	0.0952

0.5294	0.3529	0.1176
0.6204	0.2870	0.0926
0.6250	0.2813	0.0938
0.5145	0.3841	0.1014
0.4912	0.4386	0.0702
0.5088	0.4035	0.0877
0.5250	0.3750	0.1000
0.5588	0.3235	0.1176
0.5294	0.3529	0.1176
0.5750	0.3250	0.1000
0.5098	0.3922	0.0980
0.4841	0.3889	0.1270
0.5530	0.3258	0.1212
0.5196	0.3431	0.1373
0.5152	0.4242	0.0606
0.5104	0.3854	0.1042
0.5439	0.3333	0.1228
0.5686	0.3333	0.0980
0.4722	0.3889	0.1389
0.4667	0.4000	0.1333
0.5263	0.3684	0.1053
0.5588	0.3235	0.1176
0.6136	0.2955	0.0909
0.4750	0.4250	0.1000
0.5093	0.3981	0.0926
0.5000	0.3500	0.1500
0.5000	0.3824	0.1176
0.5833	0.3056	0.1111
0.5392	0.3627	0.0980
0.5333	0.3333	0.1333
0.5641	0.3333	0.1026
0.5463	0.3241	0.1296
0.5500	0.3500	0.1000
0.5333	0.3583	0.1083
0.5938	0.3438	0.0625
0.5088	0.4035	0.0877
0.6111	0.2778	0.1111
0.5313	0.4063	0.0625
0.5784	0.3431	0.0784
0.6275	0.2745	0.0980
0.5882	0.2941	0.1176
0.5944	0.2611	0.1444
0.5702	0.3070	0.1228
0.5463	0.3241	0.1296
0.5797	0.3188	0.1014
0.4231	0.4231	0.1538
0.4848	0.3939	0.1212
0.4583	0.4583	0.0833
0.4500	0.4500	0.1000
0.3846	0.4615	0.1538
0.3333	0.5556	0.1111
0.3958	0.4583	0.1458
0.3542	0.5417	0.1042
0.4242	0.5152	0.0606
0.3889	0.5000	0.1111
0.4000	0.5000	0.1000
0.3646	0.5521	0.0833
0.3750	0.5417	0.0833
0.4375	0.5000	0.0625
0.4167	0.4722	0.1111
0.4000	0.5333	0.0667
0.3889	0.5556	0.0556
0.3636	0.5455	0.0909
0.3636	0.5455	0.0909
0.5185	0.4074	0.0741
0.4167	0.5417	0.0417
0.4630	0.4630	0.0741
0.4630	0.4630	0.0741
0.4697	0.4697	0.0606
0.4545	0.4545	0.0909
0.4444	0.4444	0.1111
0.4444	0.4444	0.1111
0.4333	0.4333	0.1333
0.4500	0.4500	0.1000
0.4500	0.4500	0.1000
0.4074	0.5185	0.0741
0.4744	0.3974	0.1282

0.4778	0.4778	0.0444
0.3889	0.5000	0.1111
0.4630	0.4630	0.0741
0.4848	0.3939	0.1212
0.4333	0.4333	0.1333
0.4394	0.4394	0.1212
0.4583	0.4583	0.0833
0.4500	0.4500	0.1000
0.4444	0.4444	0.1111
0.4405	0.4405	0.1190
0.3939	0.4848	0.1212
0.3810	0.4524	0.1667
0.4028	0.4861	0.1111
0.3333	0.5833	0.0833
0.3571	0.5000	0.1429
0.3929	0.4643	0.1429
0.3571	0.5000	0.1429
0.3750	0.5000	0.1250
0.3667	0.5167	0.1167
0.3690	0.5119	0.1190
0.4063	0.4688	0.1250
0.3333	0.5833	0.0833
0.3690	0.5119	0.1190
0.4306	0.3472	0.2222
0.5093	0.2870	0.2037
0.4500	0.3500	0.2000
0.5000	0.3000	0.2000
0.4722	0.3056	0.2222
0.5139	0.2639	0.2222
0.5694	0.2361	0.1944
0.5185	0.2963	0.1852
0.5521	0.2396	0.2083
0.5729	0.2604	0.1667
0.6167	0.2167	0.1667
0.5625	0.2500	0.1875
0.5952	0.2024	0.2024
0.6071	0.2143	0.1786
0.5139	0.2639	0.2222
0.5641	0.2179	0.2179
0.4118	0.4706	0.1176
0.3939	0.4848	0.1212
0.3810	0.4524	0.1667
0.4074	0.4630	0.1296
0.4314	0.4314	0.1373
0.4412	0.4412	0.1176
0.4405	0.4405	0.1190
0.3824	0.4412	0.1765
0.3889	0.4722	0.1389
0.3889	0.4556	0.1556
0.5000	0.3750	0.1250
0.4074	0.4630	0.1296
0.4706	0.3235	0.2059
0.6061	0.2424	0.1515
0.4762	0.3333	0.1905
0.5606	0.2879	0.1515
0.5256	0.2949	0.1795
0.5417	0.2917	0.1667
0.6667	0.2121	0.1212
0.6515	0.1970	0.1515
0.6667	0.1667	0.1667
0.5926	0.2315	0.1759
0.5952	0.2381	0.1667
0.5278	0.2500	0.2222
0.5313	0.2813	0.1875
0.5175	0.2544	0.2281
0.5778	0.2444	0.1778
0.5357	0.2500	0.2143
0.6759	0.1481	0.1759
0.5521	0.2396	0.2083
0.5667	0.2000	0.2333
0.5889	0.1889	0.2222
0.5897	0.2051	0.2051
0.5250	0.2500	0.2250
0.6042	0.1667	0.2292
0.6481	0.1481	0.2037
0.5606	0.1515	0.2879
0.6042	0.1667	0.2292

0.6250	0.1667	0.2083
0.5000	0.5000	0.0000
0.4524	0.4524	0.0952
0.5000	0.4091	0.0909
0.3958	0.5208	0.0833
0.5667	0.3667	0.0667
0.4524	0.4524	0.0952
0.4259	0.5370	0.0370
0.4697	0.4697	0.0606
0.4375	0.4375	0.1250
0.4306	0.5139	0.0556
0.4167	0.4167	0.1667
0.3333	0.5333	0.1333
0.5648	0.3426	0.0926
0.5278	0.3611	0.1111
0.5333	0.3333	0.1333
0.4653	0.3611	0.1736
0.5648	0.3148	0.1204
0.5392	0.3627	0.0980
0.5556	0.3175	0.1270
0.5500	0.3500	0.1000
0.6042	0.2292	0.1667
0.5833	0.2976	0.1190
0.6042	0.3542	0.0417
0.5000	0.4091	0.0909
0.6167	0.2417	0.1417
0.5192	0.3077	0.1731
0.6667	0.2333	0.1000
0.5625	0.3125	0.1250
0.5351	0.3246	0.1404
0.4600	0.3800	0.1600
0.5606	0.2879	0.1515
0.4583	0.3583	0.1833
