# Supplementary material for: Are we restoring functional fens? – The outcomes of restoration projects in fens re-analysed with plant functional traits
Source: PLoS One. 2019 Apr 24;14(4):e0215645. doi: 10.1371/journal.pone.0215645 (PMC6481837; doi:10.1371/journal.pone.0215645)
Supplement: S4 File — (PDF) [file pone.0215645.s013.pdf]

RELEVE_NR	YEAR1	SITE3	TREATMENT	1 ch	1 ch.s	2 sla	2 sla.s	3 ldmc	3 ldmc.s	4 cs	5 fl	5 fl.s
501	2011	MIRE1	MIRE	0.2102	0.4387	0.2026	0.4406	0.7011	0.7011	0.6667	0.4286	0.4286
508	2011	MIRE3	MIRE	0.1379	0.3285	0.2064	0.4448	0.5911	0.5911	0.6667	0.4286	0.4286
512	2011	MIRE5	MIRE	0.3788	0.6259	0.5195	0.7453	0.7657	0.7657	0.6667	0.4286	0.4286
517	2011	MIRE8	MIRE	0.1234	0.2911	0.2308	0.4710	0.5519	0.5519	0.6667	0.4286	0.4286
518	2011	MIRE8	MIRE	0.1207	0.2727	0.2271	0.4672	0.5915	0.5915	0.6667	0.4286	0.4286
521	2011	MIRE9	MIRE	0.1379	0.3285	0.5061	0.6699	0.5531	0.5531	0.6667	0.4286	0.4286
522	2011	MIRE9	MIRE	0.4138	0.6569	0.2308	0.4710	0.5765	0.5765	0.6667	0.4286	0.4286
523	2011	MIRE10	MIRE	0.4138	0.6569	0.2421	0.4823	0.5341	0.5341	0.6667	0.4286	0.4286
524	2011	MIRE10	MIRE	0.1379	0.3285	0.1697	0.4005	0.5341	0.5341	0.6667	0.4286	0.4286
525	2011	MIRE11	MIRE	0.1310	0.3054	0.2271	0.4672	0.5519	0.5519	0.6667	0.4286	0.4286
530	2011	MIRE12	MIRE	0.4062	0.6316	0.2308	0.4710	0.6123	0.6123	0.6667	0.4286	0.4286
537	2011	MIRE15	MIRE	0.1324	0.3099	0.2064	0.4448	0.6156	0.6156	1.0000	0.4286	0.4286
551	2012	MIRE23	MIRE	0.1379	0.3285	0.2308	0.4710	0.6671	0.6671	0.6667	0.4286	0.4286
553	2012	MIRE24	MIRE	0.3699	0.5961	0.2555	0.5577	0.7722	0.7722	0.6667	0.4286	0.4286
554	2012	MIRE24	MIRE	0.1290	0.2986	0.2387	0.4660	0.7722	0.7722	0.6667	0.4286	0.4286
556	2012	MIRE25	MIRE	0.1806	0.3465	0.2421	0.4823	0.5620	0.5620	0.6667	0.4286	0.4286
559	2012	MIRE28	MIRE	0.1379	0.3285	0.2421	0.4823	0.7219	0.7219	1.0000	0.4286	0.4286
561	2012	MIRE29	MIRE	0.1145	0.2864	0.2371	0.4774	0.7746	0.7746	0.6667	0.4286	0.4286
564	2012	MIRE30	MIRE	0.3788	0.6259	0.2505	0.5528	0.7746	0.7746	0.6667	0.2857	0.2857
565	2012	MIRE31	MIRE	0.1084	0.2366	0.2371	0.4774	0.5620	0.5620	0.6667	0.4286	0.4286
566	2012	MIRE31	MIRE	0.1772	0.3419	0.2371	0.4774	0.6406	0.6406	0.6667	0.4286	0.4286
567	2012	MIRE32	MIRE	0.1224	0.3010	0.2371	0.4774	0.7722	0.7722	0.6667	0.4286	0.4286
568	2012	MIRE32	MIRE	0.1379	0.3285	0.2371	0.4774	0.7746	0.7746	0.6667	0.4286	0.4286
569	2012	MIRE33	MIRE	0.1290	0.2986	0.2421	0.4823	0.8250	0.8250	1.0000	0.4286	0.4286
571	2012	MIRE34	MIRE	0.3788	0.6259	0.2555	0.5577	0.7288	0.7288	1.0000	0.4286	0.4286
572	2012	MIRE34	MIRE	0.2102	0.4387	0.2371	0.4774	0.6123	0.6123	0.6667	0.4286	0.4286
573	2012	MIRE35	MIRE	0.1094	0.2395	0.2371	0.4774	0.5911	0.5911	0.6667	0.4286	0.4286
574	2012	MIRE35	MIRE	0.1379	0.3285	0.2421	0.4823	0.6766	0.6766	0.6667	0.4286	0.4286
575	2012	MIRE35	MIRE	0.2069	0.4342	0.2421	0.4823	0.5911	0.5911	0.6667	0.4286	0.4286
576	2012	MIRE36	MIRE	0.1789	0.3418	0.2237	0.4509	0.5412	0.5412	0.6667	0.4286	0.4286
578	2012	MIRE37	MIRE	0.2102	0.4387	0.2307	0.4308	0.4814	0.4814	0.6667	0.4286	0.4286
582	2012	MIRE39	MIRE	0.1379	0.3285	0.2421	0.4823	0.7722	0.7722	0.6667	0.4286	0.4286
584	2012	MIRE40	MIRE	0.1991	0.3729	0.2421	0.4823	0.6206	0.6206	0.6667	0.4286	0.4286
586	2012	MIRE41	MIRE	0.1172	0.2622	0.2271	0.4672	0.5269	0.5269	0.6667	0.4286	0.4286
590	2012	MIRE44	MIRE	0.2102	0.4387	0.2421	0.4823	0.6186	0.6186	0.6667	0.4286	0.4286
592	2012	MIRE45	MIRE	0.2069	0.4342	0.5061	0.6699	0.6418	0.6418	0.6667	0.4286	0.4286
601	2013	MIRE23	MIRE	0.2026	0.4134	0.2308	0.4710	0.7396	0.7396	0.6667	0.4286	0.4286
602	2013	MIRE23	MIRE	0.2102	0.4387	0.2308	0.4710	0.5472	0.5472	0.6667	0.4286	0.4286
606	2013	MIRE46	MIRE	0.2102	0.4387	0.5027	0.6536	0.5104	0.5104	0.6667	0.4286	0.4286
616	2013	MIRE7	MIRE	0.1303	0.3031	0.2308	0.4710	0.8250	0.8250	0.6667	0.4286	0.4286
617	2013	MIRE2	MIRE	0.2026	0.4134	0.2239	0.4382	0.6168	0.6168	0.6667	0.2857	0.2857
619	2013	MIRE2	MIRE	0.1943	0.3872	0.1843	0.3810	0.4826	0.4826	0.6667	0.4286	0.4286
621	2013	MIRE49	MIRE	0.2026	0.4134	0.2275	0.4547	0.6156	0.6156	0.6667	0.4286	0.4286
627	2013	MIRE51	MIRE	0.2069	0.4342	0.2353	0.4938	0.6957	0.6957	0.3333	0.4286	0.4286
628	2013	MIRE51	MIRE	0.1379	0.3285	0.2239	0.4382	0.6429	0.6429	0.6667	0.4286	0.4286
639	2013	MIRE52	MIRE	0.2102	0.4387	0.4953	0.6206	0.6465	0.6465	0.6667	0.4286	0.4286
641	2013	MIRE53	MIRE	0.2026	0.4134	0.2430	0.5387	0.6262	0.6262	1.0000	0.4286	0.4286
647	2013	MIRE39	MIRE	0.1964	0.3936	0.1993	0.4243	0.4826	0.4826	0.6667	0.4286	0.4286
650	2013	MIRE44	MIRE	0.2102	0.4387	0.2421	0.4823	0.6507	0.6507	0.6667	0.4286	0.4286
652	2013	MIRE44	MIRE	0.1379	0.3285	0.5061	0.6699	0.6507	0.6507	0.6667	0.4286	0.4286
654	2013	MIRE42	MIRE	0.1317	0.3076	0.2421	0.4823	0.6072	0.6072	0.6667	0.4286	0.4286
655	2013	MIRE41	MIRE	0.1083	0.2362	0.2026	0.4406	0.5519	0.5519	0.6667	0.4286	0.4286
657	2013	MIRE54	MIRE	0.1240	0.2921	0.2430	0.5387	0.6507	0.6507	1.0000	0.4286	0.4286
659	2013	MIRE25	MIRE	0.3444	0.5202	0.2198	0.5202	0.4826	0.4826	0.6667	0.2857	0.2857
660	2013	MIRE28	MIRE	0.1317	0.3076	0.2308	0.4710	0.7722	0.7722	0.6667	0.4286	0.4286
661	2013	MIRE28	MIRE	0.1303	0.3031	0.2371	0.4774	0.7722	0.7722	0.6667	0.4286	0.4286
662	2013	MIRE31	MIRE	0.1862	0.3680	0.2371	0.4774	0.6161	0.6161	0.6667	0.4286	0.4286
663	2013	MIRE31	MIRE	0.1774	0.3423	0.2874	0.5241	0.6672	0.6672	0.6667	0.4286	0.4286
664	2013	MIRE31	MIRE	0.1172	0.2622	0.2148	0.5082	0.6161	0.6161	0.6667	0.4286	0.4286
672	2014	MIRE56	MIRE	0.1303	0.3031	0.2275	0.4547	0.7646	0.7646	0.6667	0.4286	0.4286
BB_02a	2002	BB	BB_REWET	0.2276	0.4618	0.4652	0.5148	0.7657	0.7657	0.6667	0.4286	0.4286
BB_02b	2002	BB	BB_REWET	0.2276	0.4618	0.4469	0.4651	0.5104	0.5104	0.6667	0.2857	0.2857
BB_02c	2002	BB	BB_REWET	0.2276	0.4618	0.4523	0.4789	0.5282	0.5282	0.6667	0.2857	0.2857
BB_02d	2002	BB	BB_REWET	0.1857	0.4041	0.4469	0.4651	0.5104	0.5104	0.6667	0.2857	0.2857
BB_02e	2002	BB	BB_REWET	0.2276	0.4618	0.4469	0.4651	0.5104	0.5104	0.6667	0.2857	0.2857
BB_02f	2002	BB	BB_REWET	0.2276	0.4618	0.4365	0.4402	0.4259	0.4259	0.6667	0.2857	0.2857
BB_02g	2002	BB	BB_REWET	0.2276	0.4618	0.4365	0.4402	0.4259	0.4259	0.6667	0.2857	0.2857
BB_96a	1996	BB	BB_BEFORE	0.1650	0.3379	0.1299	0.2512	0.8199	0.8199	0.6667	0.4286	0.4286
BB_96b	1996	BB	BB_BEFORE	0.2069	0.3956	0.3057	0.5970	0.5933	0.5933	0.6667	0.4286	0.4286
BB_96c	1996	BB	BB_BEFORE	0.2069	0.3956	0.0892	0.1564	0.3600	0.3600	0.6667	0.2857	0.2857
BB_96d	1996	BB	BB_BEFORE	0.1650	0.3379	0.1307	0.2533	0.7518	0.7518	1.0000	0.4286	0.4286
BB_96e	1996	BB	BB_BEFORE	0.2069	0.3956	0.1203	0.2383	0.8012	0.8012	1.0000	0.4286	0.4286
BB_96f	1996	BB	BB_BEFORE	0.2069	0.3956	0.2051	0.4969	0.3648	0.3648	1.0000	0.2857	0.2857
BB_96g	1996	BB	BB_BEFORE	0.1650	0.3379	0.1299	0.2512	0.8199	0.8199	0.6667	0.5714	0.5714
CAL_14_115	2014	CAL	CAL_TSR	0.2102	0.4387	0.9655	0.8012	0.9066	0.9066	0.6667	0.4286	0.4286
CAL_14_119	2014	CAL	CAL_TSR	0.1815	0.3492	0.1589	0.3512	0.6292	0.6292	0.6667	0.2857	0.2857

CAL_14_123	2014 CAL	CAL_TSR	0.1895	0.3725	0.1918	0.3912	0.7396	0.7396	0.6667	0.2857	0.2857
CAL_14_127	2014 CAL	CAL_TSR	0.1688	0.3144	0.0929	0.2500	0.5040	0.5040	0.6667	0.2857	0.2857
CAL_14_148	2014 CAL	CAL_TSR	0.3931	0.5907	0.2148	0.5082	0.7746	0.7746	1.0000	0.4286	0.4286
CAL_14_152	2014 CAL	CAL_TSR	0.1299	0.2732	0.2026	0.4406	0.7746	0.7746	1.0000	0.5714	0.5714
CAL_14_156	2014 CAL	CAL_TSR	0.1815	0.3492	0.2026	0.4406	0.6262	0.6262	1.0000	0.4286	0.4286
CAL_14_160	2014 CAL	CAL_TSR	0.1895	0.3725	0.2148	0.5082	0.5911	0.5911	1.0000	0.4286	0.4286
CAL_14_177	2014 CAL	CAL_TSR	0.1740	0.3283	0.1918	0.3912	0.4464	0.4464	1.0000	0.2857	0.2857
CAL_14_181	2014 CAL	CAL_TSR	0.1092	0.2389	0.2263	0.4280	0.7746	0.7746	1.0000	0.2857	0.2857
CAL_14_185	2014 CAL	CAL_TSR	0.1172	0.2622	0.2148	0.5082	0.6262	0.6262	1.0000	0.2857	0.2857
CAL_14_189	2014 CAL	CAL_TSR	0.2102	0.4387	0.5183	0.7376	0.6262	0.6262	1.0000	0.5714	0.5714
CAL_14_19	2014 CAL	CAL_TSR	0.1688	0.3144	0.1903	0.4790	0.5481	0.5481	1.0000	0.4286	0.4286
CAL_14_193	2014 CAL	CAL_TSR	0.2102	0.4387	0.9655	0.8012	0.5679	0.5679	1.0000	0.4286	0.4286
CAL_14_197	2014 CAL	CAL_TSR	0.1895	0.3725	0.1589	0.3512	0.6131	0.6131	0.3333	0.2857	0.2857
CAL_14_201	2014 CAL	CAL_TSR	0.1895	0.3725	0.1918	0.3912	0.7396	0.7396	1.0000	0.2857	0.2857
CAL_14_205	2014 CAL	CAL_TSR	0.1895	0.3725	0.1673	0.3620	0.6131	0.6131	1.0000	0.4286	0.4286
CAL_14_228	2014 CAL	CAL_TSR	0.1172	0.2622	0.1918	0.3912	0.7396	0.7396	1.0000	0.2857	0.2857
CAL_14_23	2014 CAL	CAL_TSR	0.1172	0.2622	0.2148	0.5082	0.5481	0.5481	1.0000	0.2857	0.2857
CAL_14_232	2014 CAL	CAL_TSR	0.1895	0.3725	0.2148	0.5082	0.4814	0.4814	0.6667	0.2857	0.2857
CAL_14_236	2014 CAL	CAL_TSR	0.1895	0.3725	0.1956	0.3955	0.7746	0.7746	1.0000	0.2857	0.2857
CAL_14_240	2014 CAL	CAL_TSR	0.1895	0.3725	0.1918	0.3912	0.7396	0.7396	1.0000	0.4286	0.4286
CAL_14_257	2014 CAL	CAL_TSR	0.1895	0.3725	0.2493	0.5450	0.5481	0.5481	1.0000	0.2857	0.2857
CAL_14_261	2014 CAL	CAL_TSR	0.1895	0.3725	0.2493	0.5450	0.5606	0.5606	1.0000	0.2857	0.2857
CAL_14_265	2014 CAL	CAL_TSR	0.1895	0.3725	0.2827	0.4800	0.5130	0.5130	1.0000	0.2857	0.2857
CAL_14_269	2014 CAL	CAL_TSR	0.1895	0.3725	0.2493	0.5450	0.5481	0.5481	1.0000	0.2857	0.2857
CAL_14_27	2014 CAL	CAL_TSR	0.1017	0.2180	0.1818	0.4681	0.7746	0.7746	1.0000	0.2857	0.2857
CAL_14_292	2014 CAL	CAL_TSR	0.1895	0.3725	0.2493	0.5450	0.4814	0.4814	1.0000	0.2857	0.2857
CAL_14_296	2014 CAL	CAL_TSR	0.1895	0.3725	0.2493	0.5450	0.6262	0.6262	1.0000	0.2857	0.2857
CAL_14_300	2014 CAL	CAL_TSR	0.1379	0.2965	0.2148	0.5082	0.6067	0.6067	1.0000	0.2857	0.2857
CAL_14_304	2014 CAL	CAL_TSR	0.1172	0.2622	0.2263	0.4280	0.5911	0.5911	1.0000	0.2857	0.2857
CAL_14_31	2014 CAL	CAL_TSR	0.1862	0.3680	0.2493	0.5450	0.7396	0.7396	1.0000	0.2857	0.2857
CAL_14_339	2014 CAL	CAL_TSR	0.1815	0.3492	0.1781	0.4113	0.4808	0.4808	1.0000	0.5714	0.5714
CAL_14_343	2014 CAL	CAL_TSR	0.1688	0.3144	0.1584	0.3490	0.3355	0.3355	1.0000	0.2857	0.2857
CAL_14_347	2014 CAL	CAL_TSR	0.2102	0.4387	0.9885	0.9181	0.7582	0.7582	1.0000	0.5714	0.5714
CAL_14_351	2014 CAL	CAL_TSR	0.2102	0.4387	0.9763	0.8505	0.7582	0.7582	0.6667	0.4286	0.4286
CAL_14_370	2014 CAL	CAL_TSR	0.1815	0.3492	0.2148	0.5082	0.5911	0.5911	1.0000	0.4286	0.4286
CAL_14_374	2014 CAL	CAL_TSR	0.1017	0.2180	0.1956	0.3955	0.2937	0.2937	0.6667	0.1429	0.1429
CAL_14_378	2014 CAL	CAL_TSR	0.0886	0.2020	0.2148	0.5082	0.5911	0.5911	0.3333	0.2857	0.2857
CAL_14_382	2014 CAL	CAL_TSR	0.1172	0.2622	0.2148	0.5082	0.5911	0.5911	0.6667	0.4286	0.4286
CAL_14_403	2014 CAL	CAL_TSR	0.1895	0.3725	0.2026	0.4406	0.5620	0.5620	0.6667	0.4286	0.4286
CAL_14_407	2014 CAL	CAL_TSR	0.0983	0.2285	0.1894	0.3217	0.5380	0.5380	0.6667	0.2857	0.2857
CAL_14_411	2014 CAL	CAL_TSR	0.1895	0.3725	0.1918	0.3912	0.5911	0.5911	0.6667	0.2857	0.2857
CAL_14_415	2014 CAL	CAL_TSR	0.1895	0.3725	0.2493	0.5450	0.6262	0.6262	0.6667	0.4286	0.4286
CAL_14_434	2014 CAL	CAL_TSR	0.1172	0.2622	0.2148	0.5082	0.5911	0.5911	0.6667	0.2857	0.2857
CAL_14_438	2014 CAL	CAL_TSR	0.1172	0.2622	0.2148	0.5082	0.5519	0.5519	1.0000	0.4286	0.4286
CAL_14_442	2014 CAL	CAL_TSR	0.1895	0.3725	0.2371	0.4774	0.6262	0.6262	0.6667	0.4286	0.4286
CAL_14_446	2014 CAL	CAL_TSR	0.1895	0.3725	0.2263	0.4280	0.4588	0.4588	0.6667	0.1429	0.1429
CAL_14_449	2014 CAL	CAL_TSR	0.2102	0.4387	0.5183	0.7376	0.6173	0.6173	1.0000	0.5714	0.5714
CAL_14_453	2014 CAL	CAL_TSR	0.0983	0.2285	0.2064	0.4448	0.6262	0.6262	0.6667	0.4286	0.4286
CAL_14_457	2014 CAL	CAL_TSR	0.1172	0.2622	0.1974	0.4158	0.5911	0.5911	1.0000	0.4286	0.4286
CAL_14_461	2014 CAL	CAL_TSR	0.1029	0.2279	0.2148	0.5082	0.5911	0.5911	1.0000	0.2857	0.2857
CAL_14_484	2014 CAL	CAL_TSR	0.0903	0.2052	0.1818	0.4681	0.4384	0.4384	0.6667	0.2857	0.2857
CAL_14_488	2014 CAL	CAL_TSR	0.3851	0.5674	0.1673	0.3024	0.5911	0.5911	0.6667	0.4286	0.4286
CAL_14_492	2014 CAL	CAL_TSR	0.1815	0.3492	0.2148	0.5082	0.6262	0.6262	0.6667	0.4286	0.4286
CAL_14_496	2014 CAL	CAL_TSR	0.1092	0.2389	0.1781	0.4113	0.4549	0.4549	1.0000	0.5714	0.5714
CAL_14_50	2014 CAL	CAL_TSR	0.2102	0.4387	0.4953	0.6206	0.6173	0.6173	0.6667	0.4286	0.4286
CAL_14_54	2014 CAL	CAL_TSR	0.1895	0.3725	0.2493	0.5450	0.4814	0.4814	1.0000	0.2857	0.2857
CAL_14_58	2014 CAL	CAL_TSR	0.1895	0.3725	0.2493	0.5450	0.7396	0.7396	0.6667	0.2857	0.2857
CAL_14_62	2014 CAL	CAL_TSR	0.1862	0.3680	0.2148	0.5082	0.7396	0.7396	1.0000	0.2857	0.2857
CAL_14_82	2014 CAL	CAL_TSR	0.2102	0.4387	0.9807	0.8732	0.5679	0.5679	1.0000	0.4286	0.4286
CAL_14_86	2014 CAL	CAL_TSR	0.2102	0.4387	0.9655	0.8012	0.9066	0.9066	0.6667	0.4286	0.4286
CAL_14_90	2014 CAL	CAL_TSR	0.1895	0.3725	0.1589	0.3512	0.6292	0.6292	0.6667	0.2857	0.2857
CAL_14_94	2014 CAL	CAL_TSR	0.1688	0.3144	0.1903	0.4790	0.6292	0.6292	1.0000	0.4286	0.4286
CAL_D04_1	2004 CAL	CAL_BEFORI	0.1517	0.3396	0.2339	0.4356	0.5060	0.5060	1.0000	0.8571	0.8571
CAL_D04_10	2004 CAL	CAL_BEFORI	0.0931	0.2188	0.1649	0.2950	0.6161	0.6161	1.0000	0.5714	0.5714
CAL_D04_11	2004 CAL	CAL_BEFORI	0.1172	0.2622	0.2026	0.4406	0.5911	0.5911	0.6667	0.4286	0.4286
CAL_D04_12	2004 CAL	CAL_BEFORI	0.1138	0.2520	0.1918	0.3912	0.3833	0.3833	1.0000	0.5714	0.5714
CAL_D04_13	2004 CAL	CAL_BEFORI	0.0759	0.1996	0.1649	0.2950	0.5321	0.5321	1.0000	0.5714	0.5714
CAL_D04_2	2004 CAL	CAL_BEFORI	0.1379	0.2965	0.1971	0.3971	0.5130	0.5130	1.0000	0.5714	0.5714
CAL_D04_3	2004 CAL	CAL_BEFORI	0.1137	0.2745	0.1702	0.3010	0.5945	0.5945	1.0000	0.5714	0.5714
CAL_D04_4	2004 CAL	CAL_BEFORI	0.0966	0.2421	0.1649	0.2950	0.4448	0.4448	1.0000	0.4286	0.4286
CAL_D04_5	2004 CAL	CAL_BEFORI	0.1517	0.3396	0.2339	0.4356	0.5060	0.5060	1.0000	0.8571	0.8571
CAL_D04_6	2004 CAL	CAL_BEFORI	0.1447	0.3172	0.1702	0.3010	0.4801	0.4801	1.0000	0.5714	0.5714
CAL_D04_7	2004 CAL	CAL_BEFORI	0.0621	0.1690	0.1649	0.2950	0.5380	0.5380	1.0000	0.5714	0.5714
CAL_D04_8	2004 CAL	CAL_BEFORI	0.0879	0.2072	0.1649	0.2950	0.6102	0.6102	1.0000	0.4286	0.4286
CAL_D04_9	2004 CAL	CAL_BEFORI	0.0931	0.2188	0.1649	0.2950	0.3836	0.3836	1.0000	0.5714	0.5714
CAL_FM04_1	2004 CAL	CAL_REF	0.1862	0.3680	0.2371	0.4774	0.5911	0.5911	1.0000	0.4286	0.4286
CAL_FM04_10	2004 CAL	CAL_REF	0.1368	0.2841	0.2219	0.4424	0.6161	0.6161	0.6667	0.2857	0.2857

CAL_FM04_11	2004	CAL	CAL_REF	0.1368	0.2841	0.2319	0.4525	0.6161	0.6161	0.6667	0.5714	0.5714
CAL_FM04_2	2004	CAL	CAL_REF	0.1172	0.2622	0.2026	0.4406	0.6507	0.6507	0.6667	0.4286	0.4286
CAL_FM04_3	2004	CAL	CAL_REF	0.1862	0.3680	0.2026	0.4406	0.5911	0.5911	0.6667	0.4286	0.4286
CAL_FM04_4	2004	CAL	CAL_REF	0.1172	0.2622	0.2271	0.4672	0.6773	0.6773	1.0000	0.4286	0.4286
CAL_FM04_5	2004	CAL	CAL_REF	0.1172	0.2622	0.2371	0.4774	0.6161	0.6161	0.6667	0.4286	0.4286
CAL_FM04_6	2004	CAL	CAL_REF	0.1448	0.3074	0.2263	0.4280	0.6161	0.6161	1.0000	0.4286	0.4286
CAL_FM04_7	2004	CAL	CAL_REF	0.1782	0.3447	0.2263	0.4280	0.5911	0.5911	0.6667	0.4286	0.4286
CAL_FM04_8	2004	CAL	CAL_REF	0.1862	0.3680	0.2186	0.5124	0.5396	0.5396	0.6667	0.2857	0.2857
CAL_FM04_9	2004	CAL	CAL_REF	0.1172	0.2622	0.2393	0.5348	0.5911	0.5911	0.6667	0.2857	0.2857
CAL_FM14_1	2014	CAL	CAL_REF	0.1092	0.2389	0.1918	0.3912	0.5911	0.5911	1.0000	0.4286	0.4286
CAL_FM14_2	2014	CAL	CAL_REF	0.1379	0.3285	0.4953	0.6206	0.3970	0.3970	0.6667	0.2857	0.2857
CAL_FM14_3	2014	CAL	CAL_REF	0.1379	0.3285	0.5061	0.6699	0.6173	0.6173	0.6667	0.4286	0.4286
CAL_FM14_4	2014	CAL	CAL_REF	0.1092	0.2389	0.2371	0.4774	0.5911	0.5911	1.0000	0.4286	0.4286
CAL_FM14_5	2014	CAL	CAL_REF	0.1379	0.3285	0.5061	0.6699	0.6173	0.6173	0.6667	0.4286	0.4286
CAL_FM14_6	2014	CAL	CAL_REF	0.1172	0.2622	0.2393	0.5348	0.5911	0.5911	0.6667	0.4286	0.4286
CAL_FM14_7	2014	CAL	CAL_REF	0.1092	0.2389	0.2263	0.4280	0.5911	0.5911	0.6667	0.4286	0.4286
CAL_FM14_8	2014	CAL	CAL_REF	0.1379	0.3285	0.5183	0.7376	0.5392	0.5392	0.6667	0.4286	0.4286
CIE1	1998	CIE	CIE_BEFORE	0.1317	0.3076	0.2313	0.4330	0.7219	0.7219	0.6667	0.4286	0.4286
CIE2	1998	CIE	CIE_BEFORE	0.1159	0.2581	0.2163	0.4178	0.7219	0.7219	0.6667	0.4286	0.4286
CIE3	2013	CIE	CIE_REWET	0.1241	0.2833	0.2163	0.4178	0.6418	0.6418	0.6667	0.2857	0.2857
CIE4	2013	CIE	CIE_REWET	0.0531	0.1304	0.2026	0.4406	0.5269	0.5269	0.6667	0.4286	0.4286
CIE5	2013	CIE	CIE_REWET	0.0531	0.1304	0.2271	0.4672	0.5269	0.5269	0.3333	0.4286	0.4286
DE_D3_E_85	1985	DE	DE_BEFORE	0.2538	0.4503	0.1521	0.2745	0.7200	0.7200	1.0000	0.5714	0.5714
DE_D3_E_95	1995	DE	DE_TSR	0.1438	0.3149	0.3296	0.4826	0.6730	0.6730	1.0000	0.7143	0.7143
DE_D3_W_85	1985	DE	DE_BEFORE	0.2552	0.4544	0.1544	0.2816	0.7200	0.7200	1.0000	0.5714	0.5714
DE_D3_W_95	1995	DE	DE_TSR	0.1438	0.3149	0.3296	0.4826	0.6730	0.6730	1.0000	0.7143	0.7143
DO_D10_03	2003	DO	DO_TSR	0.0872	0.2052	0.2493	0.5450	0.6881	0.6881	0.6667	0.4286	0.4286
DO_D11_03	2003	DO	DO_TSR	0.0941	0.2181	0.2272	0.4478	0.7209	0.7209	1.0000	0.5714	0.5714
DO_D12_03	2003	DO	DO_TSR	0.1024	0.2361	0.1425	0.2855	0.7144	0.7144	1.0000	0.5714	0.5714
DO_D25_03	2003	DO	DO_TSR	0.1895	0.3725	0.1816	0.3778	0.6519	0.6519	0.6667	0.4286	0.4286
DO_D26_03	2003	DO	DO_TSR	0.3931	0.5907	0.1818	0.4681	0.7746	0.7746	0.6667	0.5714	0.5714
DO_D29_03	2003	DO	DO_TSR	0.3862	0.5705	0.1818	0.4681	0.7022	0.7022	0.6667	0.4286	0.4286
DO_D30_03	2003	DO	DO_TSR	0.1895	0.3725	0.2542	0.5500	0.7746	0.7746	0.6667	0.4286	0.4286
DO_D8_91	1991	DO	DO_BEFORE	0.1648	0.3217	0.2921	0.3810	0.4368	0.4368	1.0000	0.8571	0.8571
DO_D8_92	1992	DO	DO_BEFORE	0.1712	0.3397	0.2842	0.3610	0.4368	0.4368	1.0000	0.8571	0.8571
DO_donorHH_1	1993	DO	DO_REF	0.3841	0.6011	0.1447	0.3303	0.7671	0.7671	0.6667	0.5714	0.5714
DO_donorHH_2	1993	DO	DO_REF	0.6428	0.7881	0.2075	0.3691	0.8199	0.8199	0.6667	0.5714	0.5714
DO_donorLI_1	1993	DO	DO_REF	0.1719	0.3589	0.1677	0.2968	0.7671	0.7671	0.6667	0.4286	0.4286
DO_donorLI_2	1993	DO	DO_REF	0.1732	0.3632	0.1397	0.2550	0.7671	0.7671	0.6667	0.2857	0.2857
DO_donorZE_1	1993	DO	DO_REF	0.1581	0.3177	0.1767	0.3975	0.8199	0.8199	0.6667	0.4286	0.4286
DO_donorZE_2	1993	DO	DO_REF	0.1561	0.3122	0.1767	0.3975	0.8199	0.8199	0.6667	0.4286	0.4286
DO_donorZE_3	1993	DO	DO_REF	0.2069	0.3956	0.2561	0.6222	0.8012	0.8012	0.6667	0.4286	0.4286
DO_donorZS_1	1993	DO	DO_REF	0.3808	0.5905	0.1100	0.2781	0.5909	0.5909	0.6667	0.5714	0.5714
DO_donorZS_2	1993	DO	DO_REF	0.1158	0.2661	0.1184	0.2369	0.5909	0.5909	0.6667	0.2857	0.2857
DO_donorZS_3	1993	DO	DO_REF	0.3808	0.5905	0.1447	0.3303	0.5907	0.5907	0.6667	0.5714	0.5714
DO_donorZS_4	1993	DO	DO_REF	0.3980	0.6056	0.1452	0.4798	0.5471	0.5471	0.6667	0.5714	0.5714
DO_donorZS_5	1993	DO	DO_REF	0.2463	0.4532	0.1736	0.4207	0.4907	0.4907	0.6667	0.4286	0.4286
DO_donorZS_6	1993	DO	DO_REF	0.2190	0.3757	0.2100	0.4647	0.5919	0.5919	0.3333	0.2857	0.2857
DO_M1_03	2003	DO	DO_TSR	0.1158	0.2661	0.2351	0.5304	0.6471	0.6471	1.0000	0.4286	0.4286
DO_M10_03	2003	DO	DO_TSR	0.3862	0.5705	0.1736	0.4207	0.6881	0.6881	0.6667	0.4286	0.4286
DO_M11_03	2003	DO	DO_TSR	0.3931	0.5907	0.2148	0.5082	0.7396	0.7396	0.6667	0.5714	0.5714
DO_M12_03	2003	DO	DO_TSR	0.3776	0.5465	0.1821	0.4685	0.7022	0.7022	0.6667	0.4286	0.4286
DO_M13_03	2003	DO	DO_TSR	0.3862	0.5705	0.1818	0.4681	0.7238	0.7238	0.6667	0.5714	0.5714
DO_M14_03	2003	DO	DO_TSR	0.3862	0.5705	0.1818	0.4681	0.5869	0.5869	0.6667	0.4286	0.4286
DO_M2_03	2003	DO	DO_TSR	0.1021	0.2255	0.1818	0.4681	0.5653	0.5653	1.0000	0.4286	0.4286
DO_M3_03	2003	DO	DO_TSR	0.1109	0.2512	0.2607	0.6269	0.6353	0.6353	0.6667	0.4286	0.4286
DO_M4_03	2003	DO	DO_TSR	0.3931	0.5907	0.2068	0.5670	0.6353	0.6353	0.6667	0.4286	0.4286
DO_M5_03	2003	DO	DO_TSR	0.3980	0.6056	0.2607	0.6269	0.6881	0.6881	0.6667	0.5714	0.5714
DO_M6_03	2003	DO	DO_TSR	0.3980	0.6056	0.2263	0.5901	0.7396	0.7396	1.0000	0.5714	0.5714
DO_M8_03	2003	DO	DO_TSR	0.3690	0.5555	0.2319	0.4525	0.8199	0.8199	0.6667	0.5714	0.5714
DO_M9_03	2003	DO	DO_TSR	0.3690	0.5555	0.1933	0.5500	0.5998	0.5998	0.6667	0.5714	0.5714
DU_1	1995	DU	DU_BEFORE	0.0931	0.2188	0.2070	0.3394	0.8012	0.8012	1.0000	0.7143	0.7143
DU_132	2010	DU	DU_REWET	0.2069	0.3956	0.1818	0.4681	0.4914	0.4914	0.6667	0.2857	0.2857
DU_138	2010	DU	DU_REWET	0.1650	0.3379	0.1818	0.4681	0.6672	0.6672	0.6667	0.5714	0.5714
DU_167	2010	DU	DU_REWET	0.1650	0.3379	0.1591	0.3515	0.5907	0.5907	0.6667	0.2857	0.2857
DU_172	2010	DU	DU_REWET	0.1650	0.3379	0.2163	0.4178	0.5338	0.5338	0.6667	0.2857	0.2857
DU_173	1987	DU	DU_BEFORE	0.2137	0.4163	0.3573	0.6377	0.7225	0.7225	1.0000	0.8571	0.8571
DU_174	1987	DU	DU_BEFORE	0.2069	0.3956	0.1749	0.4590	0.7459	0.7459	0.6667	0.2857	0.2857
DU_175	1987	DU	DU_BEFORE	0.2137	0.4163	0.2493	0.5450	0.7118	0.7118	0.6667	0.5714	0.5714
DU_2	1997	DU	DU_BEFORE	0.1650	0.3379	0.2413	0.4687	0.8012	0.8012	1.0000	0.8571	0.8571
DU_72	2010	DU	DU_REWET	0.1650	0.3379	0.1986	0.3297	0.7459	0.7459	0.6667	0.4286	0.4286
DU_78	2010	DU	DU_REWET	0.1317	0.3076	0.2493	0.5450	0.7118	0.7118	0.6667	0.4286	0.4286
DU_83	2010	DU	DU_REWET	0.1650	0.3379	0.2493	0.5450	0.6672	0.6672	0.6667	0.4286	0.4286
DU_84	2010	DU	DU_REWET	0.1650	0.3379	0.1986	0.3297	0.6672	0.6672	0.6667	0.4286	0.4286
HAV_1_2002	2002	HAV	HAV_BEFOR	0.0787	0.1733	0.2351	0.5304	0.7459	0.7459	1.0000	0.1429	0.1429
HAV_10_2002	2002	HAV	HAV_BEFOR	0.2483	0.4594	0.1505	0.3891	0.6888	0.6888	0.6667	0.2857	0.2857
HAV_100_2010	2010	HAV	HAV_REWE	0.2102	0.4387	0.3190	0.5074	0.7746	0.7746	0.6667	0.2857	0.2857

HAV_101_2010	2010	HAV	HAV_REWE	0.2621	0.5046	0.9803	0.8707	0.9066	0.9066	0.6667	0.1429	0.1429
HAV_102_2010	2010	HAV	HAV_REWE	0.2621	0.5046	0.9876	0.9123	0.9074	0.9074	0.6667	0.4286	0.4286
HAV_103_2010	2010	HAV	HAV_REWE	0.1172	0.2622	0.1597	0.3756	0.6900	0.6900	0.6667	0.4286	0.4286
HAV_104_2010	2010	HAV	HAV_REWE	0.0589	0.1496	0.1253	0.2463	0.6020	0.6020	0.6667	0.4286	0.4286
HAV_105_2010	2010	HAV	HAV_REWE	0.0904	0.2213	0.1253	0.2463	0.5239	0.5239	0.6667	0.5714	0.5714
HAV_106_2010	2010	HAV	HAV_REWE	0.0589	0.1496	0.1438	0.3087	0.6020	0.6020	0.6667	0.4286	0.4286
HAV_107_2010	2010	HAV	HAV_REWE	0.1092	0.2389	0.1617	0.2854	0.7396	0.7396	0.6667	0.4286	0.4286
HAV_108_2010	2010	HAV	HAV_REWE	0.1316	0.3174	0.9390	0.7063	0.9066	0.9066	0.6667	0.4286	0.4286
HAV_11_2002	2002	HAV	HAV_BEFOR	0.0817	0.1968	0.1438	0.3087	0.7241	0.7241	0.6667	0.2857	0.2857
HAV_12_2002	2002	HAV	HAV_BEFOR	0.0895	0.2197	0.1438	0.3087	0.7241	0.7241	0.6667	0.5714	0.5714
HAV_13_2002	2002	HAV	HAV_BEFOR	0.0930	0.2264	0.1253	0.2463	0.7241	0.7241	0.6667	0.5714	0.5714
HAV_14_2002	2002	HAV	HAV_BEFOR	0.0964	0.2330	0.1438	0.3087	0.6977	0.6977	1.0000	0.5714	0.5714
HAV_15_2002	2002	HAV	HAV_BEFOR	0.1172	0.2622	0.1438	0.3087	0.6095	0.6095	0.6667	0.2857	0.2857
HAV_16_2002	2002	HAV	HAV_BEFOR	0.1793	0.3478	0.2176	0.5065	0.7396	0.7396	0.6667	0.4286	0.4286
HAV_17_2002	2002	HAV	HAV_BEFOR	0.1447	0.3172	0.1916	0.2959	0.8012	0.8012	1.0000	0.8571	0.8571
HAV_18_2002	2002	HAV	HAV_BEFOR	0.1103	0.2421	0.2044	0.4495	0.5966	0.5966	0.6667	0.4286	0.4286
HAV_19_2002	2002	HAV	HAV_BEFOR	0.1094	0.2395	0.1993	0.4243	0.7219	0.7219	0.6667	0.2857	0.2857
HAV_2_2002	2002	HAV	HAV_BEFOR	0.0641	0.1394	0.0954	0.1709	0.4120	0.4120	0.3333	0.1429	0.1429
HAV_20_2002	2002	HAV	HAV_BEFOR	0.1066	0.2316	0.2030	0.4285	0.6067	0.6067	0.6667	0.4286	0.4286
HAV_21_2002	2002	HAV	HAV_BEFOR	0.0945	0.2214	0.2063	0.3372	0.8012	0.8012	1.0000	0.8571	0.8571
HAV_22_2002	2002	HAV	HAV_BEFOR	0.1717	0.3585	0.2070	0.3394	0.8012	0.8012	1.0000	0.8571	0.8571
HAV_23_2002	2002	HAV	HAV_BEFOR	0.6759	0.8039	0.3543	0.4555	0.6519	0.6519	1.0000	0.4286	0.4286
HAV_24_2002	2002	HAV	HAV_BEFOR	0.2545	0.4792	0.2348	0.4912	0.7396	0.7396	0.6667	0.2857	0.2857
HAV_25_2002	2002	HAV	HAV_BEFOR	0.2621	0.5046	0.1596	0.4020	0.6784	0.6784	0.3333	0.1429	0.1429
HAV_26_2002	2002	HAV	HAV_BEFOR	0.0643	0.1573	0.1389	0.1816	0.5060	0.5060	1.0000	0.7143	0.7143
HAV_27_2002	2002	HAV	HAV_BEFOR	0.0836	0.2006	0.2063	0.3372	0.8012	0.8012	1.0000	0.8571	0.8571
HAV_28_2002	2002	HAV	HAV_BEFOR	0.0870	0.2110	0.2063	0.3372	0.8012	0.8012	1.0000	0.8571	0.8571
HAV_29_2002	2002	HAV	HAV_BEFOR	0.1862	0.3680	0.1684	0.2402	0.5060	0.5060	1.0000	0.7143	0.7143
HAV_3_2002	2002	HAV	HAV_BEFOR	0.0931	0.2046	0.1989	0.4896	0.6341	0.6341	0.6667	0.2857	0.2857
HAV_30_2002	2002	HAV	HAV_BEFOR	0.1618	0.3285	0.2063	0.3372	0.8012	0.8012	1.0000	0.8571	0.8571
HAV_31_2002	2002	HAV	HAV_BEFOR	0.0681	0.1666	0.2063	0.3372	0.6844	0.6844	1.0000	0.8571	0.8571
HAV_32_2002	2002	HAV	HAV_BEFOR	0.5200	0.6646	0.3487	0.4518	0.4733	0.4733	1.0000	0.2857	0.2857
HAV_4_2002	2002	HAV	HAV_BEFOR	0.0931	0.2188	0.2148	0.5082	0.7459	0.7459	1.0000	0.2857	0.2857
HAV_5_2002	2002	HAV	HAV_BEFOR	0.1069	0.2441	0.1753	0.4595	0.7459	0.7459	0.6667	0.2857	0.2857
HAV_6_2002	2002	HAV	HAV_BEFOR	0.1145	0.2312	0.1617	0.2854	0.7746	0.7746	0.6667	0.4286	0.4286
HAV_7_2002	2002	HAV	HAV_BEFOR	0.2069	0.4342	0.5105	0.6927	0.8332	0.8332	0.6667	0.4286	0.4286
HAV_73_2010	2010	HAV	HAV_REWE	0.2102	0.4387	0.5183	0.7376	0.7657	0.7657	0.6667	0.4286	0.4286
HAV_74_2010	2010	HAV	HAV_REWE	0.0735	0.1636	0.1595	0.2476	0.5944	0.5944	1.0000	0.2857	0.2857
HAV_75_2010	2010	HAV	HAV_REWE	0.2102	0.4387	0.4948	0.6184	0.5282	0.5282	0.6667	0.4286	0.4286
HAV_76_2010	2010	HAV	HAV_REWE	0.2102	0.4387	0.5183	0.7376	0.5481	0.5481	1.0000	0.4286	0.4286
HAV_77_2010	2010	HAV	HAV_REWE	0.2102	0.4387	0.4948	0.6184	0.5104	0.5104	0.6667	0.2857	0.2857
HAV_78_2010	2010	HAV	HAV_REWE	0.1051	0.2492	0.1434	0.3082	0.5162	0.5162	0.6667	0.5714	0.5714
HAV_79_2010	2010	HAV	HAV_REWE	0.2102	0.4387	0.5079	0.6789	0.5978	0.5978	0.6667	0.4286	0.4286
HAV_8_2002	2002	HAV	HAV_BEFOR	0.2483	0.4594	0.1505	0.3891	0.7219	0.7219	0.6667	0.2857	0.2857
HAV_80_2010	2010	HAV	HAV_REWE	0.1517	0.3516	0.3998	0.3658	0.1976	0.1976	0.3333	0.2857	0.2857
HAV_81_2010	2010	HAV	HAV_REWE	0.1414	0.3344	0.1758	0.1111	0.1224	0.1224	0.0000	0.2857	0.2857
HAV_82_2010	2010	HAV	HAV_REWE	0.0869	0.2070	0.1753	0.4595	0.7241	0.7241	0.6667	0.4286	0.4286
HAV_83_2010	2010	HAV	HAV_REWE	0.1650	0.3379	0.1753	0.4595	0.7241	0.7241	0.6667	0.4286	0.4286
HAV_84_2010	2010	HAV	HAV_REWE	0.6897	0.8491	0.5183	0.7376	0.6519	0.6519	1.0000	0.4286	0.4286
HAV_85_2010	2010	HAV	HAV_REWE	0.2621	0.5046	0.2542	0.5500	0.7746	0.7746	1.0000	0.2857	0.2857
HAV_86_2010	2010	HAV	HAV_REWE	0.2621	0.5046	0.9803	0.8707	0.8104	0.8104	0.6667	0.4286	0.4286
HAV_87_2010	2010	HAV	HAV_REWE	0.0589	0.1496	0.1434	0.3082	0.2889	0.2889	1.0000	0.2857	0.2857
HAV_88_2010	2010	HAV	HAV_REWE	0.1034	0.2379	0.2088	0.3850	0.8738	0.8738	1.0000	0.2857	0.2857
HAV_89_2010	2010	HAV	HAV_REWE	0.1655	0.3099	0.2393	0.5299	0.8738	0.8738	1.0000	0.2857	0.2857
HAV_9_2002	2002	HAV	HAV_BEFOR	0.2483	0.4594	0.1505	0.3891	0.7746	0.7746	0.6667	0.2857	0.2857
HAV_90_2010	2010	HAV	HAV_REWE	0.1650	0.3379	0.1600	0.2489	0.6812	0.6812	1.0000	0.2857	0.2857
HAV_91_2010	2010	HAV	HAV_REWE	0.1194	0.2183	0.1262	0.2109	0.2495	0.2495	1.0000	0.1429	0.1429
HAV_92_2010	2010	HAV	HAV_REWE	0.1650	0.3379	0.2493	0.5450	0.7560	0.7560	1.0000	0.2857	0.2857
HAV_93_2010	2010	HAV	HAV_REWE	0.5379	0.7175	0.3543	0.4555	0.4955	0.4955	1.0000	0.4286	0.4286
HAV_94_2010	2010	HAV	HAV_REWE	0.0328	0.0824	0.2056	0.4857	0.6025	0.6025	1.0000	0.5714	0.5714
HAV_95_2010	2010	HAV	HAV_REWE	0.0759	0.1672	0.1794	0.4535	0.5585	0.5585	0.3333	0.5714	0.5714
HAV_96_2010	2010	HAV	HAV_REWE	0.0759	0.1672	0.1266	0.3755	0.2260	0.2260	0.0000	0.5714	0.5714
HAV_97_2010	2010	HAV	HAV_REWE	0.0879	0.2002	0.3112	0.5890	0.6990	0.6990	1.0000	0.5714	0.5714
HAV_98_2010	2010	HAV	HAV_REWE	0.1241	0.3042	0.9354	0.6954	0.9066	0.9066	0.6667	0.4286	0.4286
HAV_99_2010	2010	HAV	HAV_REWE	0.1379	0.3285	0.5009	0.6451	0.7746	0.7746	0.6667	0.4286	0.4286
HO_1_04	2004	HO	HO_REWET	0.2214	0.4410	0.2393	0.5348	0.5880	0.5880	1.0000	0.4286	0.4286
HO_1_87	1987	HO	HO_BEFORE	0.1317	0.3076	0.2078	0.5001	0.6161	0.6161	0.6667	0.4286	0.4286
HO_3_04	2004	HO	HO_REWET	0.1989	0.3723	0.1244	0.2328	0.6714	0.6714	0.6667	0.4286	0.4286
KB_1994_10	1994	KB	KB_BEFORE	0.1193	0.2685	0.2493	0.5450	0.6672	0.6672	1.0000	0.5714	0.5714
KB_1994_13	1994	KB	KB_BEFORE	0.0931	0.2267	0.2254	0.4018	0.8012	0.8012	1.0000	0.8571	0.8571
KB_1994_17	1994	KB	KB_BEFORE	0.1876	0.3721	0.2402	0.5357	0.8012	0.8012	1.0000	0.5714	0.5714
KB_1994_18	1994	KB	KB_BEFORE	0.1221	0.2771	0.2078	0.5001	0.7219	0.7219	0.6667	0.5714	0.5714
KB_1994_19	1994	KB	KB_BEFORE	0.0883	0.2112	0.2402	0.5357	0.7459	0.7459	1.0000	0.5714	0.5714
KB_1994_20	1994	KB	KB_BEFORE	0.2566	0.4585	0.2066	0.2848	0.5060	0.5060	1.0000	0.8571	0.8571
KB_1994_23	1994	KB	KB_BEFORE	0.1186	0.2664	0.2569	0.5526	0.8012	0.8012	1.0000	0.8571	0.8571
KB_1994_7	1994	KB	KB_BEFORE	0.1186	0.2664	0.2493	0.5450	0.6291	0.6291	1.0000	0.5714	0.5714
KB_1994_8	1994	KB	KB_BEFORE	0.1074	0.2467	0.3062	0.5973	0.8012	0.8012	1.0000	0.8571	0.8571

KB_1997_7a	1997	KB	KB_BEFORE	0.1038	0.2402	0.1962	0.2799	0.7118	0.7118	1.0000	0.7143	0.7143
KB_1997_8a	1997	KB	KB_BEFORE	0.2566	0.4585	0.2384	0.3689	0.3485	0.3485	1.0000	0.8571	0.8571
KB_2008_10	2008	KB	KB_REWET	0.1136	0.2658	0.1993	0.4243	0.7118	0.7118	0.6667	0.5714	0.5714
KB_2008_13	2008	KB	KB_REWET	0.1017	0.2348	0.1163	0.2085	0.6672	0.6672	1.0000	0.4286	0.4286
KB_2008_17	2008	KB	KB_REWET	0.1172	0.2622	0.2469	0.5866	0.7459	0.7459	0.6667	0.2857	0.2857
KB_2008_18	2008	KB	KB_REWET	0.1248	0.2855	0.2078	0.5001	0.7118	0.7118	1.0000	0.5714	0.5714
KB_2008_19	2008	KB	KB_REWET	0.1079	0.2537	0.2118	0.5731	0.7459	0.7459	0.6667	0.4286	0.4286
KB_2008_20	2008	KB	KB_REWET	0.0817	0.1968	0.2016	0.3775	0.7753	0.7753	1.0000	0.4286	0.4286
KB_2008_23	2008	KB	KB_REWET	0.0921	0.2143	0.2469	0.5866	0.7459	0.7459	0.6667	0.4286	0.4286
KB_2008_7	2008	KB	KB_REWET	0.1017	0.2348	0.2148	0.5082	0.6672	0.6672	0.6667	0.4286	0.4286
KB_2008_7a	2008	KB	KB_REWET	0.1109	0.2512	0.2225	0.5600	0.6161	0.6161	0.6667	0.4286	0.4286
KB_2008_8	2008	KB	KB_REWET	0.0972	0.2169	0.2148	0.5082	0.7209	0.7209	0.6667	0.4286	0.4286
KB_2008_8a	2008	KB	KB_REWET	0.1172	0.2622	0.1936	0.4831	0.7459	0.7459	1.0000	0.4286	0.4286
KO_A1_12	2001	KO	KO_TSR	0.1066	0.2657	0.3467	0.5712	0.7364	0.7364	0.6667	0.4286	0.4286
KO_A2_13	1991	KO	KO_BEFORE	0.1014	0.2425	0.1569	0.4743	0.5139	0.5139	0.6667	0.4286	0.4286
KO_A3_23	2001	KO	KO_TSR	0.1562	0.3521	0.1470	0.3694	0.7746	0.7746	0.6667	0.4286	0.4286
KO_A4_24	1991	KO	KO_BEFORE	0.0994	0.2363	0.1302	0.4308	0.4998	0.4998	0.6667	0.2857	0.2857
KO_B1_35	2001	KO	KO_TSR	0.1278	0.3046	0.1698	0.4014	0.7364	0.7364	0.6667	0.4286	0.4286
KO_B2_36	1991	KO	KO_BEFORE	0.1041	0.2577	0.1436	0.3532	0.7364	0.7364	1.0000	0.4286	0.4286
KO_B3_47	2001	KO	KO_TSR	0.1254	0.2966	0.1922	0.4162	0.6836	0.6836	1.0000	0.4286	0.4286
KO_B4_48	1991	KO	KO_BEFORE	0.1254	0.2966	0.1668	0.4889	0.6137	0.6137	0.6667	0.4286	0.4286
KO_C_1	1991	KO	KO_REF	0.1317	0.3076	0.1922	0.4162	0.7118	0.7118	1.0000	0.4286	0.4286
KO_C_2	1991	KO	KO_REF	0.1386	0.3193	0.1922	0.4162	0.7646	0.7646	1.0000	0.4286	0.4286
KO_C_3	1991	KO	KO_REF	0.1538	0.3441	0.2275	0.4547	0.7646	0.7646	0.6667	0.4286	0.4286
KO_C_4	2001	KO	KO_REF	0.1317	0.3076	0.1922	0.4162	0.7646	0.7646	0.6667	0.4286	0.4286
KO_C_5	2001	KO	KO_REF	0.1317	0.3076	0.1922	0.4162	0.7646	0.7646	0.6667	0.4286	0.4286
KO_C_6	2001	KO	KO_REF	0.1317	0.3076	0.1554	0.3697	0.7646	0.7646	0.6667	0.4286	0.4286
KR_900001	1998	OUD	OUD_TSR	0.1992	0.4023	0.2479	0.5876	0.7384	0.7384	1.0000	0.8571	0.8571
KR_900006	1996	PBG	PBG_TSR	0.1531	0.3442	0.2569	0.5526	0.8199	0.8199	1.0000	0.8571	0.8571
KR_900013	1997	PBG	PBG_TSR	0.1531	0.3442	0.2469	0.5866	0.7940	0.7940	1.0000	0.8571	0.8571
KR_900019	1996	PBG	PBG_TSR	0.2047	0.4202	0.2403	0.5358	0.7384	0.7384	1.0000	0.8571	0.8571
KR_900026	1997	PBG	PBG_TSR	0.2276	0.4618	0.5259	0.7894	0.6585	0.6585	1.0000	0.8571	0.8571
KR_900032	1997	PBG	PBG_TSR	0.2276	0.4618	0.5259	0.7894	0.4553	0.4553	1.0000	0.8571	0.8571
KR_900038	1997	PBG	PBG_TSR	0.6497	0.8103	0.2469	0.5866	0.6291	0.6291	1.0000	0.8571	0.8571
KR_900048	1994	PBN	PBN_TSR	0.2166	0.4254	0.2542	0.5500	0.8552	0.8552	1.0000	0.8571	0.8571
KR_900057	1994	PBN	PBN_TSR	0.2276	0.4618	0.5183	0.7376	0.7384	0.7384	1.0000	0.4286	0.4286
KR_900070	1994	PBN	PBN_TSR	0.2241	0.4501	0.3057	0.5970	0.8552	0.8552	1.0000	0.8571	0.8571
KR_900079	1994	PBN	PBN_TSR	0.2047	0.4202	0.2403	0.5358	0.7384	0.7384	1.0000	0.4286	0.4286
KR_900104	1998	PMB	PMB_TSR	0.9793	0.9338	0.3890	0.7067	0.7560	0.7560	1.0000	0.7143	0.7143
KR_900106	1999	PMB	PMB_TSR	0.0945	0.2214	0.2079	0.5431	0.7459	0.7459	0.6667	0.5714	0.5714
KR_900109	1999	PMB	PMB_TSR	0.0945	0.2214	0.1829	0.5113	0.5504	0.5504	0.6667	0.2857	0.2857
KR_900112	1997	COM	COM_TSR	0.1992	0.4023	0.2201	0.5141	0.4553	0.4553	1.0000	0.8571	0.8571
KR_900118	1997	COM	COM_TSR	0.1992	0.4023	0.2393	0.5348	0.4317	0.4317	1.0000	0.5714	0.5714
KR_900124	1997	COM	COM_TSR	0.1095	0.2612	0.2201	0.5141	0.4259	0.4259	1.0000	0.8571	0.8571
KR_900130	1997	COM	COM_TSR	0.2047	0.4202	0.3334	0.6194	0.4812	0.4812	1.0000	0.8571	0.8571
KR_900136	1998	PVB	PVB_TSR	0.2047	0.4202	0.3814	0.6548	0.6392	0.6392	1.0000	0.5714	0.5714
KR_900140	1998	PVB	PVB_TSR	0.2047	0.4202	0.2201	0.5141	0.7940	0.7940	1.0000	0.5714	0.5714
KR_900144	1998	PVB	PVB_TSR	0.6497	0.8103	0.2298	0.5683	0.7746	0.7746	1.0000	0.5714	0.5714
KR_900190	2007	PBG	PBG_TSR	0.1324	0.3099	0.2059	0.5406	0.7746	0.7746	0.6667	0.4286	0.4286
KR_900192	2007	PBG	PBG_TSR	0.1324	0.3099	0.2469	0.5866	0.7364	0.7364	0.6667	0.4286	0.4286
KR_900193	2007	PBG	PBG_TSR	0.1090	0.2678	0.1982	0.4887	0.7746	0.7746	0.3333	0.2857	0.2857
KR_900194	2007	PBG	PBG_TSR	0.1048	0.2600	0.2078	0.3840	0.7364	0.7364	0.6667	0.4286	0.4286
KR_900204	2007	PVB	PVB_TSR	0.1269	0.2920	0.2619	0.6018	0.7746	0.7746	1.0000	0.4286	0.4286
KR_900218	2008	COM	COM_TSR	0.1317	0.3076	0.1895	0.5200	0.7646	0.7646	0.6667	0.5714	0.5714
KR_900219	2008	COM	COM_TSR	0.2064	0.4259	0.1895	0.5200	0.7746	0.7746	0.6667	0.5714	0.5714
KR_900220	2008	COM	COM_TSR	0.9945	0.9814	0.2127	0.5487	0.7746	0.7746	0.6667	0.5714	0.5714
KR_900225	2010	OUD	OUD_TSR	0.2166	0.4254	0.2619	0.6018	0.6672	0.6672	1.0000	0.5714	0.5714
KR_900226	2011	PBN	PBN_TSR	0.2040	0.4179	0.2003	0.4912	0.6392	0.6392	1.0000	0.5714	0.5714
KR_900227	2011	PMB	PMB_TSR	0.2069	0.3956	0.2542	0.5500	0.7560	0.7560	1.0000	0.4286	0.4286
KR_900228	2011	COM	COM_TSR	0.2064	0.4259	0.2446	0.5403	0.7746	0.7746	0.6667	0.5714	0.5714
KR_900230	2012	PBG	PBG_TSR	0.1545	0.3464	0.2059	0.5406	0.7364	0.7364	0.6667	0.4286	0.4286
KR_900231	2012	PBG	PBG_TSR	0.1545	0.3464	0.2059	0.5406	0.7746	0.7746	0.6667	0.4286	0.4286
KR_900232	2012	PBG	PBG_TSR	0.1545	0.3464	0.2059	0.5406	0.7746	0.7746	0.6667	0.4286	0.4286
KR_900233	2012	PBN	PBN_TSR	0.1324	0.3099	0.2262	0.5643	0.6773	0.6773	1.0000	0.5714	0.5714
KR_900234	2012	PBN	PBN_TSR	0.1324	0.3099	0.2619	0.6018	0.6773	0.6773	1.0000	0.4286	0.4286
KR_900235	2012	PBN	PBN_TSR	0.1152	0.2793	0.1895	0.5200	0.6009	0.6009	1.0000	0.2857	0.2857
KR_900236	2012	PBN	PBN_TSR	0.1317	0.3076	0.1895	0.5200	0.7118	0.7118	1.0000	0.4286	0.4286
KR_900237	2012	PMB	PMB_TSR	0.1207	0.2681	0.2079	0.5431	0.7118	0.7118	0.6667	0.5714	0.5714
KR_900238	2012	PMB	PMB_TSR	0.2697	0.4998	0.2065	0.4986	0.7118	0.7118	0.6667	0.4286	0.4286
KR_900239	2013	PBN	PBN_TSR	0.1317	0.3076	0.2619	0.6018	0.7118	0.7118	1.0000	0.5714	0.5714
KR_900240	2013	PBN	PBN_TSR	0.1317	0.3076	0.1895	0.5200	0.6836	0.6836	0.6667	0.4286	0.4286
KR_900241	2013	PBN	PBN_TSR	0.1186	0.2856	0.1895	0.5200	0.7118	0.7118	1.0000	0.4286	0.4286
KR_900272	2011	PBN	PBN_TSR	0.1862	0.3680	0.2003	0.4912	0.7118	0.7118	0.6667	0.5714	0.5714
KR_900273	2011	PBN	PBN_TSR	0.2047	0.4202	0.2469	0.5866	0.7746	0.7746	1.0000	0.5714	0.5714
LA_1_2007	2007	LA	LA_BEFORE	0.1000	0.2317	0.1912	0.2946	0.8012	0.8012	1.0000	0.8571	0.8571
LA_10_2007	2007	LA	LA_BEFORE	0.1740	0.3283	0.5619	0.7597	0.6526	0.6526	0.3333	0.2857	0.2857
LA_11_2007	2007	LA	LA_BEFORE	0.1293	0.2825	0.1818	0.2711	0.5060	0.5060	1.0000	0.8571	0.8571

LA_12_2007	2007	LA	LA_BEFORE	0.1152	0.2423	0.1993	0.3318	0.4733	0.4733	1.0000	0.5714	0.5714
LA_13_2007	2007	LA	LA_BEFORE	0.1794	0.3832	0.2743	0.4462	0.5933	0.5933	1.0000	0.7143	0.7143
LA_14_2007	2007	LA	LA_BEFORE	0.1794	0.3832	0.2743	0.4462	0.5098	0.5098	1.0000	0.4286	0.4286
LA_15_2007	2007	LA	LA_BEFORE	0.1794	0.3832	0.2558	0.3838	0.5380	0.5380	0.6667	0.1429	0.1429
LA_16_2007	2007	LA	LA_BEFORE	0.2069	0.3956	0.2000	0.4908	0.7200	0.7200	1.0000	0.5714	0.5714
LA_19_2007	2007	LA	LA_BEFORE	0.1794	0.3832	0.3057	0.5970	0.5098	0.5098	1.0000	0.4286	0.4286
LA_2_2007	2007	LA	LA_BEFORE	0.2276	0.4618	0.2434	0.5390	0.5504	0.5504	0.6667	0.2857	0.2857
LA_20_2007	2007	LA	LA_BEFORE	0.1650	0.3379	0.3057	0.5970	0.5380	0.5380	0.6667	0.4286	0.4286
LA_21_2007	2007	LA	LA_BEFORE	0.1379	0.2965	0.1203	0.2383	0.4332	0.4332	1.0000	0.4286	0.4286
LA_22_2007	2007	LA	LA_BEFORE	0.1379	0.2965	0.2063	0.3372	0.8012	0.8012	1.0000	0.8571	0.8571
LA_3_2007	2007	LA	LA_BEFORE	0.0945	0.2214	0.2569	0.5526	0.5060	0.5060	1.0000	0.8571	0.8571
LA_59_2013	2013	LA	LA_REWET	0.2102	0.4387	0.4948	0.6184	0.3698	0.3698	1.0000	0.4286	0.4286
LA_6_2007	2007	LA	LA_BEFORE	0.1650	0.3379	0.2569	0.5526	0.4812	0.4812	1.0000	0.8571	0.8571
LA_60_2013	2013	LA	LA_REWET	0.1379	0.3285	0.4526	0.4796	0.3224	0.3224	0.6667	0.2857	0.2857
LA_61_2013	2013	LA	LA_REWET	0.2793	0.5245	0.4526	0.4796	0.3224	0.3224	0.6667	0.2857	0.2857
LA_62_2013	2013	LA	LA_REWET	0.2793	0.5245	0.4526	0.4796	0.3224	0.3224	0.6667	0.2857	0.2857
LA_63_2013	2013	LA	LA_REWET	0.1331	0.3122	0.2238	0.4708	0.8443	0.8443	1.0000	0.4286	0.4286
LA_64_2013	2013	LA	LA_REWET	0.2102	0.4387	0.5183	0.7376	0.7657	0.7657	0.6667	0.4286	0.4286
LA_65_2013	2013	LA	LA_REWET	0.1895	0.3725	0.2403	0.5358	0.8738	0.8738	1.0000	0.2857	0.2857
LA_66_2013	2013	LA	LA_REWET	0.2069	0.3956	0.1913	0.3891	0.6392	0.6392	1.0000	0.4286	0.4286
LA_67_2013	2013	LA	LA_REWET	0.2069	0.3956	0.2403	0.5358	0.7384	0.7384	1.0000	0.2857	0.2857
LA_68_2013	2013	LA	LA_REWET	0.2102	0.4387	0.4948	0.6184	0.5392	0.5392	1.0000	0.4286	0.4286
LA_69_2013	2013	LA	LA_REWET	0.2069	0.3956	0.1749	0.4590	0.6978	0.6978	1.0000	0.2857	0.2857
LA_70_2013	2013	LA	LA_REWET	0.2276	0.4618	0.5183	0.7376	0.6392	0.6392	1.0000	0.4286	0.4286
LA_71_2013	2013	LA	LA_REWET	0.1794	0.3832	0.1753	0.4595	0.6161	0.6161	0.6667	0.4286	0.4286
LA_72_2013	2013	LA	LA_REWET	0.1650	0.3379	0.1753	0.4595	0.6694	0.6694	0.6667	0.4286	0.4286
LA_73_2013	2013	LA	LA_REWET	0.1069	0.2441	0.2551	0.3816	0.4332	0.4332	0.6667	0.4286	0.4286
LA_74_2013	2013	LA	LA_REWET	0.1895	0.3725	0.2403	0.5358	0.8552	0.8552	1.0000	0.2857	0.2857
LA_9_2007	2007	LA	LA_BEFORE	0.1331	0.3122	0.2238	0.4708	0.8443	0.8443	1.0000	0.2857	0.2857
LM_101	2005	LM	LM_TSR	0.1083	0.2362	0.1591	0.4371	0.5907	0.5907	1.0000	0.5714	0.5714
LM_102	2005	LM	LM_TSR	0.1895	0.3725	0.2201	0.5141	0.4741	0.4741	1.0000	0.4286	0.4286
LM_104	2005	LM	LM_TSR	0.1172	0.2622	0.1631	0.3918	0.6978	0.6978	1.0000	0.5714	0.5714
LM_105	2005	LM	LM_TSR	0.1172	0.2622	0.1631	0.3918	0.6049	0.6049	1.0000	0.5714	0.5714
LM_112	2008	LM	LM_TSR	0.1269	0.2920	0.1771	0.4619	0.4756	0.4756	1.0000	0.5714	0.5714
LM_113	2008	LM	LM_TSR	0.1172	0.2622	0.2163	0.4178	0.6049	0.6049	1.0000	0.5714	0.5714
LM_114	2008	LM	LM_TSR	0.1172	0.2622	0.2237	0.4509	0.3335	0.3335	1.0000	0.4286	0.4286
LM_115	2008	LM	LM_TSR	0.1103	0.2421	0.1753	0.4595	0.4054	0.4054	1.0000	0.5714	0.5714
LM_116	2008	LM	LM_TSR	0.1148	0.2551	0.1818	0.4681	0.5907	0.5907	0.6667	0.5714	0.5714
LM_117	2008	LM	LM_TSR	0.1269	0.2920	0.2185	0.5124	0.6585	0.6585	1.0000	0.5714	0.5714
LM_118	2008	LM	LM_TSR	0.1240	0.2829	0.1771	0.4619	0.6672	0.6672	1.0000	0.5714	0.5714
LM_119	2008	LM	LM_TSR	0.1240	0.2829	0.1771	0.4619	0.4259	0.4259	1.0000	0.5714	0.5714
LM_120	2008	LM	LM_TSR	0.1269	0.2920	0.2163	0.4178	0.6967	0.6967	1.0000	0.5714	0.5714
LM_121	2008	LM	LM_TSR	0.1172	0.2622	0.1771	0.4619	0.6672	0.6672	1.0000	0.5714	0.5714
LM_122	2008	LM	LM_TSR	0.1148	0.2551	0.2393	0.5348	0.6672	0.6672	1.0000	0.5714	0.5714
LM_123	2008	LM	LM_TSR	0.1269	0.2920	0.2444	0.4717	0.6731	0.6731	1.0000	0.5714	0.5714
LM_124	1995	LM	LM_BEFORE	0.0621	0.1565	0.0837	0.1403	0.5283	0.5283	1.0000	0.5714	0.5714
LM_125	1995	LM	LM_BEFORE	0.0621	0.1565	0.0837	0.1403	0.6291	0.6291	1.0000	0.5714	0.5714
LM_126	1995	LM	LM_BEFORE	0.1650	0.3379	0.1753	0.4595	0.5665	0.5665	1.0000	0.5714	0.5714
LM_127	1995	LM	LM_BEFORE	0.1172	0.2622	0.1753	0.4595	0.7459	0.7459	1.0000	0.5714	0.5714
LM_128	1995	LM	LM_BEFORE	0.1085	0.2555	0.0900	0.1526	0.5665	0.5665	1.0000	0.5714	0.5714
LM_129	1995	LM	LM_BEFORE	0.0937	0.2277	0.2393	0.5348	0.6291	0.6291	1.0000	0.5714	0.5714
LM_130	1995	LM	LM_BEFORE	0.1172	0.2622	0.1771	0.4619	0.6672	0.6672	1.0000	0.5714	0.5714
LM_131	1995	LM	LM_BEFORE	0.0937	0.2277	0.2393	0.5348	0.6150	0.6150	1.0000	0.5714	0.5714
MOS_1	1999	MOS	MOS_TSR	0.0876	0.2280	0.1342	0.3281	0.8233	0.8233	0.3333	0.2857	0.2857
MOS_4	2012	MOS	MOS_TSR	0.0876	0.2280	0.2151	0.4291	0.9313	0.9313	0.3333	0.4286	0.4286
NP_1	2007	NP	NP_TSR	0.2062	0.4182	0.2326	0.4800	0.6957	0.6957	0.6667	0.4286	0.4286
NP_11	2007	NP	NP_TSR	0.2062	0.4182	0.2353	0.4938	0.7826	0.7826	0.3333	0.4286	0.4286
NP_14	2007	NP	NP_TSR	0.2062	0.4182	0.2326	0.4800	0.7826	0.7826	0.3333	0.4286	0.4286
NP_16	2007	NP	NP_TSR	0.2062	0.4182	0.2326	0.4800	0.6957	0.6957	0.6667	0.4286	0.4286
NP_19	2007	NP	NP_TSR	0.2062	0.4182	0.2326	0.4800	0.8250	0.8250	0.3333	0.4286	0.4286
NP_21	2007	NP	NP_TSR	0.2062	0.4182	0.2308	0.4710	0.6957	0.6957	0.3333	0.4286	0.4286
NP_24	2007	NP	NP_TSR	0.2062	0.4182	0.2308	0.4710	0.6957	0.6957	0.6667	0.4286	0.4286
NP_26	2007	NP	NP_TSR	0.2062	0.4182	0.2326	0.4800	0.6957	0.6957	0.3333	0.4286	0.4286
NP_29	2007	NP	NP_TSR	0.2062	0.4182	0.2326	0.4800	0.8250	0.8250	0.3333	0.4286	0.4286
NP_4	2007	NP	NP_TSR	0.2062	0.4182	0.2326	0.4800	0.6957	0.6957	0.3333	0.4286	0.4286
NP_6	2007	NP	NP_TSR	0.2062	0.4182	0.2308	0.4710	0.6957	0.6957	0.6667	0.4286	0.4286
NP_9	2007	NP	NP_TSR	0.2062	0.4182	0.2308	0.4710	0.6957	0.6957	0.3333	0.4286	0.4286
OOS_1	2000	OOS	OOS_REWE	0.0448	0.1232	0.1019	0.2563	0.6665	0.6665	0.3333	0.5714	0.5714
OOS_10	2000	OOS	OOS_REWE	0.1172	0.2622	0.1821	0.4685	0.6291	0.6291	0.6667	0.5714	0.5714
OOS_11	2000	OOS	OOS_REWE	0.6697	0.7849	0.2012	0.4923	0.6239	0.6239	1.0000	0.4286	0.4286
OOS_12	2000	OOS	OOS_REWE	0.1254	0.2966	0.2430	0.5387	0.8515	0.8515	0.6667	0.4286	0.4286
OOS_13	2000	OOS	OOS_REWE	0.2759	0.5206	0.9807	0.8732	0.9066	0.9066	0.6667	0.4286	0.4286
OOS_14	2000	OOS	OOS_REWE	0.1319	0.2480	0.1512	0.2790	0.6151	0.6151	0.3333	0.2857	0.2857
OOS_15	2000	OOS	OOS_REWE	0.0946	0.2272	0.1104	0.3114	0.3891	0.3891	0.6667	0.4286	0.4286
OOS_2	2000	OOS	OOS_REWE	0.0946	0.2272	0.1019	0.2563	0.4197	0.4197	0.6667	0.2857	0.2857
OOS_3	2000	OOS	OOS_REWE	0.1019	0.2252	0.0792	0.2511	0.1693	0.1693	0.3333	0.4286	0.4286
OOS_32	2004	OOS	OOS_REWE	0.1857	0.4041	0.4652	0.5148	0.8738	0.8738	1.0000	0.4286	0.4286

OOS_4	2000	OOS	OOS_REWE	0.2040	0.4179	0.2542	0.5500	0.7364	0.7364	0.6667	0.2857	0.2857
OOS_5	2000	OOS	OOS_REWE	0.0589	0.1496	0.0727	0.1176	0.2736	0.2736	0.3333	0.2857	0.2857
OOS_50	2014	OOS	OOS_REWE	0.0421	0.1148	0.1104	0.3114	0.7289	0.7289	0.6667	0.4286	0.4286
OOS_51	2014	OOS	OOS_REWE	0.0421	0.1148	0.1168	0.3224	0.7232	0.7232	0.6667	0.4286	0.4286
OOS_52	2014	OOS	OOS_REWE	0.0994	0.2363	0.1366	0.4419	0.7080	0.7080	0.3333	0.2857	0.2857
OOS_53	2014	OOS	OOS_REWE	0.0724	0.1607	0.0971	0.2379	0.2100	0.2100	0.0000	0.1429	0.1429
OOS_54	2014	OOS	OOS_REWE	0.0552	0.1191	0.0643	0.1849	0.2097	0.2097	0.3333	0.0000	0.0000
OOS_55	2014	OOS	OOS_REWE	0.2007	0.4134	0.2186	0.5124	0.7364	0.7364	0.6667	0.2857	0.2857
OOS_56	2014	OOS	OOS_REWE	0.0869	0.2070	0.1753	0.4595	0.4507	0.4507	0.3333	0.2857	0.2857
OOS_57	2014	OOS	OOS_REWE	0.1586	0.2842	0.2012	0.4923	0.6151	0.6151	0.6667	0.4286	0.4286
OOS_58	2014	OOS	OOS_REWE	0.1221	0.2771	0.1829	0.5113	0.5380	0.5380	0.6667	0.4286	0.4286
OOS_59	2014	OOS	OOS_REWE	0.2086	0.3324	0.1891	0.4062	0.6653	0.6653	0.6667	0.2857	0.2857
OOS_6	2000	OOS	OOS_REWE	0.1931	0.3774	0.5268	0.7420	0.8218	0.8218	1.0000	0.4286	0.4286
OOS_60	2014	OOS	OOS_REWE	0.1103	0.2785	0.4652	0.5148	0.9074	0.9074	0.6667	0.4286	0.4286
OOS_61	2014	OOS	OOS_REWE	0.1650	0.3379	0.3057	0.5970	0.4532	0.4532	1.0000	0.5714	0.5714
OOS_62	2014	OOS	OOS_REWE	0.6697	0.7849	0.2012	0.4923	0.6239	0.6239	0.6667	0.4286	0.4286
OOS_63	2014	OOS	OOS_REWE	0.1254	0.2966	0.1450	0.4160	0.8233	0.8233	0.6667	0.4286	0.4286
OOS_64	2014	OOS	OOS_REWE	0.2069	0.4342	0.5009	0.6451	0.7894	0.7894	0.6667	0.4286	0.4286
OOS_65	2014	OOS	OOS_REWE	0.2483	0.4342	0.2996	0.5917	0.7746	0.7746	0.6667	0.4286	0.4286
OOS_7	2000	OOS	OOS_REWE	0.1221	0.2771	0.1895	0.5200	0.7646	0.7646	0.6667	0.4286	0.4286
OOS_8	2000	OOS	OOS_REWE	0.2600	0.4690	0.1578	0.2867	0.6132	0.6132	0.3333	0.4286	0.4286
OOS_9	2000	OOS	OOS_REWE	0.1103	0.2785	0.9354	0.6954	0.9074	0.9074	0.6667	0.4286	0.4286
PLI_B1_14	2014	PLI	PLI_REWET	0.1221	0.2771	0.2313	0.4330	0.5385	0.5385	0.6667	0.4286	0.4286
PLI_B1_95	1995	PLI	PLI_BEFORE	0.1256	0.2830	0.2144	0.4410	0.7873	0.7873	0.6667	0.4286	0.4286
PLI_B2_14	2014	PLI	PLI_REWET	0.1303	0.3031	0.2301	0.4445	0.7219	0.7219	0.6667	0.4286	0.4286
PLI_B2_95	1995	PLI	PLI_BEFORE	0.1303	0.3031	0.2200	0.4217	0.6818	0.6818	1.0000	0.4286	0.4286
PLI_B3_14	2014	PLI	PLI_REWET	0.1303	0.3031	0.2313	0.4330	0.6647	0.6647	0.6667	0.4286	0.4286
PLI_B3_95	1995	PLI	PLI_BEFORE	0.1221	0.2771	0.2542	0.5500	0.7118	0.7118	0.6667	0.4286	0.4286
PLI_B4_14	2014	PLI	PLI_REWET	0.1303	0.3031	0.2313	0.4330	0.6647	0.6647	0.6667	0.4286	0.4286
PLI_B4_95	1995	PLI	PLI_BEFORE	0.1303	0.3031	0.2313	0.4330	0.6818	0.6818	1.0000	0.4286	0.4286
PLI_B5_14	2014	PLI	PLI_REWET	0.2069	0.3956	0.2370	0.4386	0.6067	0.6067	0.6667	0.4286	0.4286
PLI_B5_95	1995	PLI	PLI_BEFORE	0.1317	0.3076	0.2237	0.4509	0.7118	0.7118	0.6667	0.4286	0.4286
PLI_B6_14	2014	PLI	PLI_REWET	0.2276	0.4618	0.5183	0.7376	0.6978	0.6978	1.0000	0.4286	0.4286
PLI_B6_95	1995	PLI	PLI_BEFORE	0.1655	0.3736	0.4953	0.6206	0.5779	0.5779	0.6667	0.4286	0.4286
RR_1_1998	1998	RR	RR_BEFORE	0.1650	0.3379	0.1753	0.4595	0.5380	0.5380	1.0000	0.5714	0.5714
RR_10_1998	1998	RR	RR_BEFORE	0.0869	0.2070	0.2185	0.5124	0.4801	0.4801	1.0000	0.4286	0.4286
RR_100_1998	1998	RR	RR_BEFORE	0.1570	0.3146	0.0815	0.1359	0.6436	0.6436	0.6667	0.2857	0.2857
RR_117_1998	1998	RR	RR_BEFORE	0.5114	0.6412	0.2502	0.3775	0.8199	0.8199	1.0000	0.8571	0.8571
RR_124_1998	1998	RR	RR_BEFORE	0.1650	0.3379	0.2569	0.5526	0.5824	0.5824	1.0000	0.7143	0.7143
RR_133_1998	1998	RR	RR_BEFORE	0.1650	0.3379	0.0815	0.1359	0.4336	0.4336	0.3333	0.1429	0.1429
RR_142_1998	1998	RR	RR_BEFORE	0.1650	0.3379	0.1749	0.4590	0.6073	0.6073	0.6667	0.4286	0.4286
RR_151_1998	1998	RR	RR_BEFORE	0.1650	0.3379	0.2003	0.4912	0.6291	0.6291	0.6667	0.4286	0.4286
RR_160_1998	1998	RR	RR_BEFORE	0.1650	0.3379	0.0864	0.1466	0.6073	0.6073	1.0000	0.4286	0.4286
RR_169_1998	1998	RR	RR_BEFORE	0.2483	0.4342	0.2501	0.3799	0.6409	0.6409	0.6667	0.4286	0.4286
RR_177_1998	1998	RR	RR_BEFORE	0.2483	0.4342	0.1915	0.3175	0.8199	0.8199	0.6667	0.2857	0.2857
RR_185_1998	1998	RR	RR_BEFORE	0.2276	0.4618	0.5183	0.7376	0.4737	0.4737	0.6667	0.2857	0.2857
RR_19_1998	1998	RR	RR_BEFORE	0.1650	0.3379	0.1753	0.4595	0.4336	0.4336	0.6667	0.1429	0.1429
RR_194_1998	1998	RR	RR_BEFORE	0.1650	0.3379	0.2542	0.5500	0.6392	0.6392	0.6667	0.4286	0.4286
RR_203_1998	1998	RR	RR_BEFORE	0.1570	0.3146	0.2569	0.5526	0.6844	0.6844	1.0000	0.8571	0.8571
RR_212_1998	1998	RR	RR_BEFORE	0.1561	0.3122	0.1570	0.2867	0.5797	0.5797	0.6667	0.2857	0.2857
RR_221_1998	1998	RR	RR_BEFORE	0.1650	0.3379	0.1753	0.4595	0.4507	0.4507	0.6667	0.4286	0.4286
RR_230_1998	1998	RR	RR_BEFORE	0.1650	0.3379	0.1753	0.4595	0.3484	0.3484	0.6667	0.4286	0.4286
RR_239_1998	1998	RR	RR_BEFORE	0.1698	0.3525	0.3251	0.6854	0.8199	0.8199	1.0000	0.4286	0.4286
RR_246_2012	2012	RR	RR_REWET	0.1339	0.2532	0.2148	0.5082	0.6350	0.6350	0.6667	0.2857	0.2857
RR_247_2012	2012	RR	RR_REWET	0.1857	0.4041	0.5183	0.7376	0.6303	0.6303	0.6667	0.2857	0.2857
RR_249_2012	2012	RR	RR_REWET	0.2102	0.4387	0.5183	0.7376	0.4962	0.4962	1.0000	0.4286	0.4286
RR_250_2012	2012	RR	RR_REWET	0.2276	0.4618	0.4652	0.5148	0.7940	0.7940	1.0000	0.4286	0.4286
RR_251_2012	2012	RR	RR_REWET	0.0828	0.1770	0.1828	0.2775	0.2852	0.2852	0.6667	0.2857	0.2857
RR_252_2012	2012	RR	RR_REWET	0.1138	0.2294	0.2542	0.5500	0.7940	0.7940	1.0000	0.2857	0.2857
RR_253_2012	2012	RR	RR_REWET	0.2276	0.4618	0.4948	0.6184	0.5008	0.5008	1.0000	0.4286	0.4286
RR_254_2012	2012	RR	RR_REWET	0.2793	0.5245	0.5073	0.5072	0.3461	0.3461	0.6667	0.2857	0.2857
RR_255_2012	2012	RR	RR_REWET	0.2102	0.4387	0.4948	0.6184	0.4962	0.4962	1.0000	0.4286	0.4286
RR_256_2012	2012	RR	RR_REWET	0.1414	0.3344	0.4683	0.5242	0.7894	0.7894	0.6667	0.4286	0.4286
RR_257_2012	2012	RR	RR_REWET	0.1360	0.2584	0.1607	0.2760	0.3954	0.3954	1.0000	0.2857	0.2857
RR_258_2012	2012	RR	RR_REWET	0.1167	0.2119	0.1710	0.4537	0.3502	0.3502	0.3333	0.1429	0.1429
RR_259_2012	2012	RR	RR_REWET	0.1586	0.3627	0.9711	0.8257	0.8101	0.8101	1.0000	0.4286	0.4286
RR_260_2012	2012	RR	RR_REWET	0.2276	0.4618	0.4948	0.6184	0.4542	0.4542	1.0000	0.4286	0.4286
RR_261_2012	2012	RR	RR_REWET	0.2793	0.5245	0.4526	0.4796	0.3224	0.3224	0.6667	0.2857	0.2857
RR_262_2012	2012	RR	RR_REWET	0.1650	0.3379	0.1586	0.3279	0.4921	0.4921	1.0000	0.4286	0.4286
RR_263_2012	2012	RR	RR_REWET	0.1857	0.4041	0.5183	0.7376	0.6392	0.6392	1.0000	0.4286	0.4286
RR_264_2012	2012	RR	RR_REWET	0.1857	0.4041	0.4868	0.5868	0.6392	0.6392	1.0000	0.4286	0.4286
RR_265_2012	2012	RR	RR_REWET	0.1414	0.3344	0.9354	0.6954	0.9066	0.9066	0.6667	0.4286	0.4286
RR_266_2012	2012	RR	RR_REWET	0.1414	0.3344	0.9354	0.6954	0.9066	0.9066	0.6667	0.4286	0.4286
RR_267_2012	2012	RR	RR_REWET	0.1414	0.3344	0.1758	0.1111	0.1224	0.1224	0.0000	0.2857	0.2857
RR_268_2012	2012	RR	RR_REWET	0.1414	0.3344	0.9228	0.6602	0.6691	0.6691	1.0000	0.4286	0.4286
RR_269_2012	2012	RR	RR_REWET	0.2102	0.4387	0.9650	0.7990	0.4026	0.4026	0.6667	0.2857	0.2857
RR_270_2012	2012	RR	RR_REWET	0.1414	0.3344	0.9354	0.6954	0.9066	0.9066	0.6667	0.4286	0.4286

RR_271_2012	2012	RR	RR_REWET	0.1000	0.2317	0.2393	0.5348	0.6392	0.6392	1.0000	0.4286	0.4286
RR_272_2012	2012	RR	RR_REWET	0.1650	0.3379	0.2338	0.3338	0.6844	0.6844	1.0000	0.2857	0.2857
RR_273_2012	2012	RR	RR_REWET	0.2069	0.4342	0.9711	0.8257	0.9066	0.9066	0.6667	0.4286	0.4286
RR_28_1998	1998	RR	RR_BEFORE	0.1650	0.3379	0.2063	0.3372	0.6844	0.6844	1.0000	0.7143	0.7143
RR_37_1998	1998	RR	RR_BEFORE	0.1650	0.3379	0.2063	0.3372	0.5933	0.5933	1.0000	0.7143	0.7143
RR_46_1998	1998	RR	RR_BEFORE	0.0928	0.2039	0.2493	0.5450	0.6585	0.6585	1.0000	0.5714	0.5714
RR_55_1998	1998	RR	RR_BEFORE	0.1379	0.2965	0.2185	0.5124	0.6585	0.6585	1.0000	0.5714	0.5714
RR_64_1998	1998	RR	RR_BEFORE	0.1650	0.3379	0.1243	0.2436	0.4357	0.4357	0.6667	0.2857	0.2857
RR_73_1998	1998	RR	RR_BEFORE	0.1443	0.2798	0.1091	0.2010	0.3502	0.3502	0.3333	0.1429	0.1429
RR_82_1998	1998	RR	RR_BEFORE	0.1603	0.3240	0.1243	0.2207	0.5268	0.5268	1.0000	0.4286	0.4286
RR_91_1998	1998	RR	RR_BEFORE	0.2276	0.4618	0.5119	0.5463	0.8199	0.8199	1.0000	0.2857	0.2857
SE_A1_2010	2010	SE	SE_REWET	0.4000	0.6118	0.1101	0.3099	0.3109	0.3109	0.6667	0.4286	0.4286
SE_A1a_2010	2010	SE	SE_REWET	0.0966	0.2334	0.0992	0.2604	0.6888	0.6888	0.6667	0.2857	0.2857
SE_A1b_2010	2010	SE	SE_REWET	0.0497	0.1328	0.0955	0.2452	0.2494	0.2494	0.3333	0.2857	0.2857
SE_A2_2010	2010	SE	SE_REWET	0.2000	0.3984	0.0992	0.2604	0.5484	0.5484	0.6667	0.4286	0.4286
SE_A2a_2010	2010	SE	SE_REWET	0.2000	0.3984	0.0992	0.2604	0.1967	0.1967	0.3333	0.4286	0.4286
SE_A2b_2010	2010	SE	SE_REWET	0.1980	0.3922	0.0992	0.2604	0.3021	0.3021	0.6667	0.4286	0.4286
SE_A3_2010	2010	SE	SE_REWET	0.0946	0.2272	0.1119	0.3188	0.5484	0.5484	0.6667	0.2857	0.2857
SE_A3a_2010	2010	SE	SE_REWET	0.0517	0.1390	0.0992	0.2604	0.6360	0.6360	0.3333	0.2857	0.2857
SE_A3b_2010	2010	SE	SE_REWET	0.0966	0.2334	0.1119	0.3188	0.5484	0.5484	0.6667	0.2857	0.2857
SE_A4_2010	2010	SE	SE_REWET	0.2000	0.3984	0.1119	0.3188	0.3021	0.3021	0.6667	0.4286	0.4286
SE_A4a_2010	2010	SE	SE_REWET	0.2000	0.3984	0.0885	0.2411	0.2647	0.2647	0.6667	0.4286	0.4286
SE_A4b_2010	2010	SE	SE_REWET	0.0966	0.2334	0.1119	0.3188	0.6888	0.6888	0.6667	0.2857	0.2857
SE_A5_2010	2010	SE	SE_REWET	0.0517	0.1390	0.1119	0.3188	0.6480	0.6480	0.3333	0.4286	0.4286
SE_A5a_2010	2010	SE	SE_REWET	0.1241	0.2833	0.1119	0.3188	0.6888	0.6888	0.6667	0.4286	0.4286
SE_A5b_2010	2010	SE	SE_REWET	0.2034	0.4093	0.1246	0.3404	0.6888	0.6888	0.6667	0.4286	0.4286
SE_B1_2010	2010	SE	SE_REWET	0.0828	0.1921	0.0800	0.2548	0.1227	0.1227	0.3333	0.2857	0.2857
SE_B1a_2010	2010	SE	SE_REWET	0.0903	0.2144	0.0789	0.2496	0.1315	0.1315	0.3333	0.2857	0.2857
SE_B1b_2010	2010	SE	SE_REWET	0.0966	0.2334	0.0694	0.1562	0.6888	0.6888	0.6667	0.2857	0.2857
SE_B2_2010	2010	SE	SE_REWET	0.0946	0.2272	0.1131	0.3152	0.3109	0.3109	0.6667	0.2857	0.2857
SE_B2a_2010	2010	SE	SE_REWET	0.1980	0.3922	0.1119	0.3188	0.3021	0.3021	0.6667	0.4286	0.4286
SE_B2b_2010	2010	SE	SE_REWET	0.1980	0.3922	0.1119	0.3188	0.3021	0.3021	0.6667	0.4286	0.4286
SE_B3_2010	2010	SE	SE_REWET	0.2034	0.4093	0.1119	0.2820	0.6360	0.6360	0.3333	0.4286	0.4286
SE_B3a_2010	2010	SE	SE_REWET	0.0552	0.1499	0.1119	0.2820	0.2494	0.2494	0.3333	0.2857	0.2857
SE_B3b_2010	2010	SE	SE_REWET	0.1276	0.2942	0.1119	0.2820	0.4957	0.4957	0.3333	0.2857	0.2857
SE_B4_2010	2010	SE	SE_REWET	0.0966	0.2334	0.1119	0.3188	0.6888	0.6888	0.6667	0.4286	0.4286
SE_B4a_2010	2010	SE	SE_REWET	0.2000	0.3984	0.1119	0.3188	0.6480	0.6480	0.3333	0.5714	0.5714
SE_B4b_2010	2010	SE	SE_REWET	0.0448	0.1252	0.1119	0.2820	0.2494	0.2494	0.3333	0.2857	0.2857
SE_B5_2010	2010	SE	SE_REWET	0.0966	0.2334	0.0992	0.2604	0.3021	0.3021	0.6667	0.2857	0.2857
SE_B5a_2010	2010	SE	SE_REWET	0.2034	0.4093	0.1246	0.3404	0.3013	0.3013	0.3333	0.5714	0.5714
SE_B5b_2010	2010	SE	SE_REWET	0.0497	0.1328	0.0992	0.2604	0.1794	0.1794	0.3333	0.2857	0.2857
SE_C1_2010	2010	SE	SE_REWET	0.1980	0.3922	0.0955	0.2452	0.4598	0.4598	0.6667	0.4286	0.4286
SE_C1a_2010	2010	SE	SE_REWET	0.1980	0.3922	0.0939	0.2392	0.3021	0.3021	0.6667	0.4286	0.4286
SE_C1b_2010	2010	SE	SE_REWET	0.0946	0.2272	0.0694	0.1562	0.3021	0.3021	0.6667	0.2857	0.2857
SE_C2_2010	2010	SE	SE_REWET	0.0497	0.1328	0.1101	0.3099	0.3109	0.3109	0.6667	0.2857	0.2857
SE_C2a_2010	2010	SE	SE_REWET	0.1980	0.3922	0.1101	0.3099	0.3109	0.3109	0.6667	0.4286	0.4286
SE_C2b_2010	2010	SE	SE_REWET	0.0946	0.2272	0.1119	0.3188	0.3421	0.3421	0.6667	0.4286	0.4286
SE_C3_2010	2010	SE	SE_REWET	0.1276	0.2942	0.1246	0.3404	0.2613	0.2613	0.3333	0.2857	0.2857
SE_C3a_2010	2010	SE	SE_REWET	0.0517	0.1418	0.1246	0.3404	0.3013	0.3013	0.3333	0.4286	0.4286
SE_C3b_2010	2010	SE	SE_REWET	0.0517	0.1390	0.1119	0.3188	0.2613	0.2613	0.3333	0.2857	0.2857
SE_C4_2010	2010	SE	SE_REWET	0.2034	0.4093	0.1246	0.3404	0.2613	0.2613	0.3333	0.4286	0.4286
SE_C4a_2010	2010	SE	SE_REWET	0.1980	0.3922	0.1111	0.3151	0.2558	0.2558	0.3333	0.4286	0.4286
SE_C4b_2010	2010	SE	SE_REWET	0.2034	0.4093	0.1246	0.3404	0.3013	0.3013	0.3333	0.5714	0.5714
SE_C5_2010	2010	SE	SE_REWET	0.0517	0.1418	0.1246	0.3404	0.3013	0.3013	0.3333	0.4286	0.4286
SE_C5a_2010	2010	SE	SE_REWET	0.0552	0.1499	0.1246	0.3404	0.7110	0.7110	0.6667	0.4286	0.4286
SE_C5b_2010	2010	SE	SE_REWET	0.2034	0.4093	0.1246	0.3404	0.3509	0.3509	0.6667	0.5714	0.5714
SE_D1_2010	2010	SE	SE_REWET	0.2034	0.4093	0.1119	0.2820	0.3021	0.3021	0.6667	0.4286	0.4286
SE_D1a_2010	2010	SE	SE_REWET	0.0448	0.1252	0.1119	0.2820	0.2494	0.2494	0.3333	0.2857	0.2857
SE_D1b_2010	2010	SE	SE_REWET	0.1000	0.2443	0.0822	0.1778	0.3021	0.3021	0.6667	0.2857	0.2857
SE_D2_2010	2010	SE	SE_REWET	0.0946	0.2272	0.1101	0.3099	0.5572	0.5572	0.6667	0.2857	0.2857
SE_D2a_2010	2010	SE	SE_REWET	0.0828	0.1921	0.1011	0.2994	0.5484	0.5484	0.6667	0.2857	0.2857
SE_D2b_2010	2010	SE	SE_REWET	0.1000	0.2443	0.1246	0.3404	0.3021	0.3021	0.6667	0.2857	0.2857
SE_D3_2010	2010	SE	SE_REWET	0.0552	0.1499	0.1246	0.3404	0.2613	0.2613	0.6667	0.2857	0.2857
SE_D3a_2010	2010	SE	SE_REWET	0.0379	0.0978	0.1011	0.2994	0.3013	0.3013	0.6667	0.4286	0.4286
SE_D3b_2010	2010	SE	SE_REWET	0.0552	0.1499	0.1246	0.3404	0.2239	0.2239	0.3333	0.2857	0.2857
SE_D4_2010	2010	SE	SE_REWET	0.0448	0.1252	0.1246	0.3404	0.2613	0.2613	0.3333	0.2857	0.2857
SE_D4a_2010	2010	SE	SE_REWET	0.1980	0.3922	0.1119	0.3188	0.2613	0.2613	0.3333	0.4286	0.4286
SE_D4b_2010	2010	SE	SE_REWET	0.0517	0.1418	0.1246	0.3404	0.3013	0.3013	0.3333	0.4286	0.4286
SE_D5_2010	2010	SE	SE_REWET	0.0448	0.1252	0.1246	0.3404	0.2613	0.2613	0.3333	0.2857	0.2857
SE_D5a_2010	2010	SE	SE_REWET	0.0552	0.1499	0.1246	0.3404	0.3013	0.3013	0.3333	0.4286	0.4286
SE_D5b_2010	2010	SE	SE_REWET	0.1000	0.2443	0.1246	0.3404	0.2647	0.2647	0.6667	0.2857	0.2857
SE_E1_2002	2002	SE	SE_BEFORE	0.0394	0.1081	0.0312	0.0603	0.1794	0.1794	0.3333	0.2857	0.2857
SE_E2_2002	2002	SE	SE_BEFORE	0.0946	0.2272	0.0814	0.1940	0.3133	0.3133	0.6667	0.2857	0.2857
SE_E3_2002	2002	SE	SE_BEFORE	0.0394	0.1081	0.0470	0.0962	0.1967	0.1967	0.3333	0.2857	0.2857
SE_E4_2002	2002	SE	SE_BEFORE	0.0946	0.2272	0.0694	0.1562	0.3021	0.3021	0.6667	0.2857	0.2857
SE_E5_2002	2002	SE	SE_BEFORE	0.2034	0.4093	0.1119	0.2820	0.1794	0.1794	0.3333	0.4286	0.4286
SE_F1_2002	2002	SE	SE_BEFORE	0.0394	0.1081	0.0312	0.0603	0.1794	0.1794	0.3333	0.2857	0.2857

SE_F2_2002	2002 SE	SE_BEFORE	0.0394	0.1081	0.0312	0.0603	0.1794	0.1794	0.3333	0.2857	0.2857
SE_F3_2002	2002 SE	SE_BEFORE	0.0352	0.0953	0.0789	0.2496	0.1315	0.1315	0.3333	0.2857	0.2857
SE_F4_2002	2002 SE	SE_BEFORE	0.1221	0.2771	0.0694	0.1562	0.3021	0.3021	0.6667	0.2857	0.2857
SE_F5_2002	2002 SE	SE_BEFORE	0.0704	0.1787	0.0312	0.0603	0.1794	0.1794	0.3333	0.2857	0.2857
SE_G1_2002	2002 SE	SE_BEFORE	0.0946	0.2272	0.1101	0.3099	0.3109	0.3109	0.6667	0.2857	0.2857
SE_G2_2002	2002 SE	SE_BEFORE	0.0414	0.1143	0.2727	0.5173	0.6660	0.6660	0.6667	0.2857	0.2857
SE_G3_2002	2002 SE	SE_BEFORE	0.0394	0.1081	0.0312	0.0603	0.1794	0.1794	0.3333	0.2857	0.2857
SE_G4_2002	2002 SE	SE_BEFORE	0.1241	0.2833	0.1378	0.3611	0.4409	0.4409	0.6667	0.4286	0.4286
SE_G5_2002	2002 SE	SE_BEFORE	0.1276	0.2942	0.1214	0.2875	0.4409	0.4409	0.6667	0.4286	0.4286
SE_H1_2002	2002 SE	SE_BEFORE	0.1066	0.2497	0.0725	0.1615	0.3021	0.3021	0.6667	0.2857	0.2857
SE_H2_2002	2002 SE	SE_BEFORE	0.0966	0.2334	0.2302	0.3547	0.6572	0.6572	0.3333	0.2857	0.2857
SE_H3_2002	2002 SE	SE_BEFORE	0.0572	0.1598	0.1246	0.3404	0.5678	0.5678	0.6667	0.4286	0.4286
SE_H4_2002	2002 SE	SE_BEFORE	0.2034	0.4093	0.1246	0.3404	0.4833	0.4833	0.6667	0.4286	0.4286
SE_H5_2002	2002 SE	SE_BEFORE	0.0448	0.1252	0.0847	0.1853	0.2285	0.2285	0.6667	0.2857	0.2857
SE_I1_2010	2010 SE	SE_REWET	0.0083	0.0208	0.0078	0.0163	0.4124	0.4124	0.6667	0.2857	0.2857
SE_I2_2010	2010 SE	SE_REWET	0.0083	0.0208	0.0078	0.0163	0.4124	0.4124	0.6667	0.2857	0.2857
SE_I3_2010	2010 SE	SE_REWET	0.0352	0.0953	0.0789	0.2496	0.1315	0.1315	0.3333	0.2857	0.2857
SE_I4_2010	2010 SE	SE_REWET	0.0903	0.2144	0.0867	0.2659	0.5439	0.5439	1.0000	0.2857	0.2857
SE_I5_2010	2010 SE	SE_REWET	0.0414	0.1143	0.2709	0.5083	0.6660	0.6660	0.3333	0.2857	0.2857
SE_J1_2010	2010 SE	SE_REWET	0.0938	0.2248	0.0299	0.0775	0.2251	0.2251	0.0000	0.1429	0.1429
SE_J2_2010	2010 SE	SE_REWET	0.0552	0.1191	0.0383	0.0959	0.1227	0.1227	0.3333	0.0000	0.0000
SE_J3_2010	2010 SE	SE_REWET	0.0000	0.0000	0.0000	0.0000	0.0000	0.0000	0.0000	0.0000	0.0000
SE_J4_2010	2010 SE	SE_REWET	0.0394	0.1081	0.1101	0.3099	0.3109	0.3109	0.6667	0.2857	0.2857
SE_J5_2010	2010 SE	SE_REWET	0.0000	0.0000	0.0000	0.0000	0.0000	0.0000	0.0000	0.0000	0.0000
SE_K2_2010	2010 SE	SE_REWET	0.0000	0.0000	0.0000	0.0000	0.0000	0.0000	0.0000	0.0000	0.0000
SE_K3_2010	2010 SE	SE_REWET	0.0414	0.1143	0.1101	0.3099	0.3109	0.3109	0.6667	0.2857	0.2857
SE_K4_2010	2010 SE	SE_REWET	0.0000	0.0000	0.0000	0.0000	0.0000	0.0000	0.0000	0.0000	0.0000
SE_K5_2010	2010 SE	SE_REWET	0.0352	0.0953	0.0789	0.2496	0.1315	0.1315	0.3333	0.2857	0.2857
SE_L1_2010	2010 SE	SE_REWET	0.1241	0.2833	0.0383	0.0959	0.4347	0.4347	0.6667	0.1429	0.1429
SE_L2_2010	2010 SE	SE_REWET	0.0517	0.1390	0.1017	0.2559	0.3292	0.3292	0.3333	0.1429	0.1429
SE_L3_2010	2010 SE	SE_REWET	0.0055	0.0137	0.0158	0.0359	0.0173	0.0173	0.0000	0.1429	0.1429
SE_L4_2010	2010 SE	SE_REWET	0.0394	0.1081	0.0312	0.0603	0.1794	0.1794	0.3333	0.2857	0.2857
SE_L5_2010	2010 SE	SE_REWET	0.0000	0.0000	0.0000	0.0000	0.0000	0.0000	0.0000	0.0000	0.0000
SE_M1_2002	2002 SE	SE_BEFORE	0.0069	0.0206	0.1131	0.3152	0.2779	0.2779	0.0000	0.0000	0.0000
SE_M2_2002	2002 SE	SE_BEFORE	0.0352	0.0953	0.0789	0.2496	0.1315	0.1315	0.3333	0.2857	0.2857
SE_M3_2002	2002 SE	SE_BEFORE	0.0000	0.0000	0.0000	0.0000	0.0000	0.0000	0.0000	0.0000	0.0000
SE_M4_2002	2002 SE	SE_BEFORE	0.0000	0.0000	0.0000	0.0000	0.0000	0.0000	0.0000	0.0000	0.0000
SE_M5_2002	2002 SE	SE_BEFORE	0.0903	0.2144	0.0789	0.2496	0.1315	0.1315	0.3333	0.2857	0.2857
SE_N1_2002	2002 SE	SE_BEFORE	0.0000	0.0000	0.0000	0.0000	0.0000	0.0000	0.0000	0.0000	0.0000
SE_N2_2002	2002 SE	SE_BEFORE	0.0000	0.0000	0.0000	0.0000	0.0000	0.0000	0.0000	0.0000	0.0000
SE_N3_2002	2002 SE	SE_BEFORE	0.0966	0.2334	0.2302	0.3547	0.6572	0.6572	0.3333	0.2857	0.2857
SE_N4_2002	2002 SE	SE_BEFORE	0.0000	0.0000	0.0000	0.0000	0.0000	0.0000	0.0000	0.0000	0.0000
SE_N5_2002	2002 SE	SE_BEFORE	0.0566	0.1501	0.2709	0.5083	0.6660	0.6660	0.3333	0.2857	0.2857
SE_O1_2002	2002 SE	SE_BEFORE	0.3980	0.6056	0.1996	0.4359	0.3109	0.3109	0.6667	0.4286	0.4286
SE_O2_2002	2002 SE	SE_BEFORE	0.0690	0.1542	0.0759	0.1673	0.4598	0.4598	0.3333	0.2857	0.2857
SE_O3_2002	2002 SE	SE_BEFORE	0.0138	0.0351	0.0376	0.0714	0.2100	0.2100	0.0000	0.1429	0.1429
SE_O4_2002	2002 SE	SE_BEFORE	0.0552	0.1191	0.0383	0.0959	0.1227	0.1227	0.3333	0.0000	0.0000
SE_O5_2002	2002 SE	SE_BEFORE	0.0552	0.1191	0.1589	0.2823	0.2174	0.2174	0.3333	0.0000	0.0000
SE_P1_2002	2002 SE	SE_BEFORE	0.0903	0.2144	0.1996	0.4359	0.2261	0.2261	0.3333	0.2857	0.2857
SE_P2_2002	2002 SE	SE_BEFORE	0.0546	0.1439	0.1996	0.4359	0.3109	0.3109	0.6667	0.0000	0.0000
SE_P4_2002	2002 SE	SE_BEFORE									
SE_P5_2002	2002 SE	SE_BEFORE	0.0966	0.2334	0.2709	0.5083	0.6660	0.6660	1.0000	0.2857	0.2857
ST_A1_2010	2010 ST	ST_REWET	0.1345	0.2919	0.4239	0.6231	0.6031	0.6031	0.3333	0.4286	0.4286
ST_A1a_2010	2010 ST	ST_REWET	0.0966	0.2041	0.1200	0.2959	0.4909	0.4909	0.3333	0.2857	0.2857
ST_A1b_2010	2010 ST	ST_REWET	0.9586	0.8757	0.1956	0.3955	0.5464	0.5464	0.6667	0.5714	0.5714
ST_A2_2010	2010 ST	ST_REWET	0.1241	0.2833	0.1956	0.3955	0.5464	0.5464	0.3333	0.2857	0.2857
ST_A2a_2010	2010 ST	ST_REWET	0.9793	0.9338	0.1918	0.3912	0.7396	0.7396	0.3333	0.5714	0.5714
ST_A2b_2010	2010 ST	ST_REWET	0.0945	0.2214	0.2262	0.5643	0.4937	0.4937	0.3333	0.2857	0.2857
ST_A3_2010	2010 ST	ST_REWET	0.1179	0.2643	0.0409	0.1551	0.3775	0.3775	0.0000	0.4286	0.4286
ST_A3a_2010	2010 ST	ST_REWET	0.1083	0.2362	0.0409	0.1551	0.3687	0.3687	0.0000	0.4286	0.4286
ST_A3b_2010	2010 ST	ST_REWET	0.0903	0.2144	0.0521	0.1858	0.4418	0.4418	0.3333	0.2857	0.2857
ST_A4_2010	2010 ST	ST_REWET	0.0903	0.2144	0.0521	0.1858	0.4418	0.4418	0.3333	0.2857	0.2857
ST_A4a_2010	2010 ST	ST_REWET	0.1028	0.2532	0.1899	0.3159	0.6957	0.6957	0.3333	0.2857	0.2857
ST_A4b_2010	2010 ST	ST_REWET	0.0145	0.0416	0.0736	0.2380	0.0140	0.0140	0.0000	0.0000	0.0000
ST_A5_2010	2010 ST	ST_REWET	0.0724	0.1443	0.0301	0.1058	0.3687	0.3687	0.3333	0.2857	0.2857
ST_A5a_2010	2010 ST	ST_REWET	0.1979	0.3920	0.1807	0.3664	0.5972	0.5972	0.3333	0.4286	0.4286
ST_A5b_2010	2010 ST	ST_REWET	0.0483	0.1009	0.0260	0.0890	0.1579	0.1579	0.3333	0.1429	0.1429
ST_B1_2010	2010 ST	ST_REWET	0.1386	0.3045	0.2078	0.5001	0.5972	0.5972	0.3333	0.4286	0.4286
ST_B1a_2010	2010 ST	ST_REWET	0.9841	0.9484	0.2078	0.5001	0.6597	0.6597	0.3333	0.5714	0.5714
ST_B1b_2010	2010 ST	ST_REWET	0.1688	0.3144	0.1430	0.4128	0.2228	0.2228	0.3333	0.1429	0.1429
ST_B2_2010	2010 ST	ST_REWET	0.1966	0.3878	0.1168	0.3224	0.3327	0.3327	0.3333	0.4286	0.4286
ST_B2a_2010	2010 ST	ST_REWET	0.1979	0.3920	0.1807	0.3664	0.5972	0.5972	0.3333	0.4286	0.4286
ST_B2b_2010	2010 ST	ST_REWET	0.0034	0.0065	0.0213	0.0706	0.1239	0.1239	0.3333	0.1429	0.1429
ST_B3_2010	2010 ST	ST_REWET	0.0752	0.1681	0.1112	0.2607	0.4909	0.4909	0.3333	0.2857	0.2857
ST_B3a_2010	2010 ST	ST_REWET	0.0552	0.1191	0.0899	0.1901	0.4909	0.4909	0.3333	0.2857	0.2857
ST_B3b_2010	2010 ST	ST_REWET	0.0517	0.1090	0.1112	0.2607	0.4909	0.4909	0.3333	0.2857	0.2857
ST_B4_2010	2010 ST	ST_REWET	0.0938	0.2209	0.0406	0.1537	0.1327	0.1327	0.3333	0.2857	0.2857

ST_B4a_2010	2010	ST	ST_REWET	0.0034	0.0065	0.0213	0.0706	0.1239	0.1239	0.3333	0.1429	0.1429
ST_B4b_2010	2010	ST	ST_REWET	0.0000	0.0000	0.0000	0.0000	0.0000	0.0000	0.0000	0.0000	0.0000
ST_B5_2010	2010	ST	ST_REWET	0.0825	0.1882	0.0914	0.2380	0.4442	0.4442	0.0000	0.1429	0.1429
ST_B5a_2010	2010	ST	ST_REWET	0.0483	0.1009	0.0260	0.0890	0.1579	0.1579	0.3333	0.1429	0.1429
ST_B5b_2010	2010	ST	ST_REWET	0.0000	0.0000	0.0000	0.0000	0.0000	0.0000	0.0000	0.0000	0.0000
ST_C1_2010	2010	ST	ST_REWET	0.1221	0.2769	0.2064	0.4448	0.5972	0.5972	0.6667	0.4286	0.4286
ST_C1a_2010	2010	ST	ST_REWET	0.1241	0.2833	0.2712	0.5098	0.6572	0.6572	0.3333	0.4286	0.4286
ST_C1b_2010	2010	ST	ST_REWET	0.1221	0.2769	0.2064	0.4448	0.7396	0.7396	0.3333	0.4286	0.4286
ST_C2_2010	2010	ST	ST_REWET	0.1407	0.3109	0.2604	0.4605	0.6572	0.6572	0.0000	0.2857	0.2857
ST_C2a_2010	2010	ST	ST_REWET	0.1407	0.3109	0.0759	0.1673	0.3327	0.3327	0.3333	0.2857	0.2857
ST_C2b_2010	2010	ST	ST_REWET	0.1407	0.3109	0.2302	0.3547	0.6572	0.6572	0.3333	0.4286	0.4286
ST_C3_2010	2010	ST	ST_REWET	0.1407	0.3109	0.2563	0.4437	0.6572	0.6572	0.3333	0.4286	0.4286
ST_C3a_2010	2010	ST	ST_REWET	0.0828	0.1921	0.1323	0.3527	0.4909	0.4909	0.3333	0.2857	0.2857
ST_C3b_2010	2010	ST	ST_REWET	0.1241	0.2833	0.2515	0.4253	0.6572	0.6572	0.6667	0.4286	0.4286
ST_C4_2010	2010	ST	ST_REWET	0.1407	0.3109	0.2709	0.5083	0.6660	0.6660	0.3333	0.4286	0.4286
ST_C4a_2010	2010	ST	ST_REWET	0.1407	0.3109	0.2709	0.5083	0.6660	0.6660	0.3333	0.4286	0.4286
ST_C4b_2010	2010	ST	ST_REWET	0.1407	0.3109	0.2515	0.4253	0.6572	0.6572	0.3333	0.4286	0.4286
ST_C5_2010	2010	ST	ST_REWET	0.1407	0.3109	0.2712	0.5098	0.6572	0.6572	0.3333	0.4286	0.4286
ST_C5a_2010	2010	ST	ST_REWET	0.1241	0.2833	0.2515	0.4253	0.6572	0.6572	0.6667	0.4286	0.4286
ST_C5b_2010	2010	ST	ST_REWET	0.1407	0.3109	0.2712	0.5098	0.6572	0.6572	0.6667	0.4286	0.4286
ST_D1_2010	2010	ST	ST_REWET	0.4000	0.6118	0.1759	0.3479	0.5972	0.5972	0.6667	0.4286	0.4286
ST_D1a_2010	2010	ST	ST_REWET	0.1407	0.3109	0.2302	0.3547	0.7396	0.7396	0.6667	0.2857	0.2857
ST_D1b_2010	2010	ST	ST_REWET	0.1407	0.3109	0.2313	0.4330	0.7396	0.7396	0.6667	0.4286	0.4286
ST_D2_2010	2010	ST	ST_REWET	0.1407	0.3109	0.1867	0.3603	0.5972	0.5972	0.6667	0.4286	0.4286
ST_D2a_2010	2010	ST	ST_REWET	0.1407	0.3109	0.2011	0.3272	0.5972	0.5972	0.6667	0.4286	0.4286
ST_D2b_2010	2010	ST	ST_REWET	0.1407	0.3109	0.1867	0.3603	0.7396	0.7396	0.6667	0.2857	0.2857
ST_D3_2010	2010	ST	ST_REWET	0.1407	0.3109	0.2515	0.4253	0.6572	0.6572	0.6667	0.4286	0.4286
ST_D3a_2010	2010	ST	ST_REWET	0.1407	0.3109	0.1867	0.3603	0.5972	0.5972	0.6667	0.4286	0.4286
ST_D3b_2010	2010	ST	ST_REWET	0.1407	0.3109	0.2712	0.5098	0.6572	0.6572	0.6667	0.4286	0.4286
ST_D4_2010	2010	ST	ST_REWET	0.1241	0.2833	0.2563	0.4437	0.6572	0.6572	0.6667	0.4286	0.4286
ST_D4a_2010	2010	ST	ST_REWET	0.1241	0.2833	0.2563	0.4437	0.6572	0.6572	0.6667	0.4286	0.4286
ST_D4b_2010	2010	ST	ST_REWET	0.1407	0.3109	0.2787	0.4632	0.7396	0.7396	0.6667	0.4286	0.4286
ST_D5_2010	2010	ST	ST_REWET	0.1028	0.2052	0.1830	0.3560	0.7396	0.7396	0.6667	0.2857	0.2857
ST_D5a_2010	2010	ST	ST_REWET	0.0979	0.2335	0.1830	0.3560	0.7396	0.7396	0.3333	0.4286	0.4286
ST_D5b_2010	2010	ST	ST_REWET	0.0924	0.1800	0.1112	0.2607	0.4909	0.4909	0.6667	0.2857	0.2857
ST_E1_2002	2002	ST	ST_BEFORE	0.1000	0.2399	0.2712	0.5098	0.5333	0.5333	0.3333	0.2857	0.2857
ST_E2_2002	2002	ST	ST_BEFORE	0.0946	0.2272	0.1166	0.3220	0.3021	0.3021	0.6667	0.4286	0.4286
ST_E3_2002	2002	ST	ST_BEFORE	0.1034	0.2464	0.2709	0.5083	0.6660	0.6660	0.3333	0.1429	0.1429
ST_E4_2002	2002	ST	ST_BEFORE	0.1034	0.2464	0.2709	0.5083	0.6660	0.6660	0.6667	0.2857	0.2857
ST_E5_2002	2002	ST	ST_BEFORE	0.0000	0.0000	0.0000	0.0000	0.0000	0.0000	0.0000	0.0000	0.0000
ST_F1_2002	2002	ST	ST_BEFORE	0.0159	0.0471	0.2712	0.5098	0.6660	0.6660	0.6667	0.1429	0.1429
ST_F2_2002	2002	ST	ST_BEFORE	0.1000	0.2399	0.2712	0.5098	0.6799	0.6799	0.6667	0.4286	0.4286
ST_F3_2002	2002	ST	ST_BEFORE	0.1000	0.2399	0.2709	0.5083	0.6660	0.6660	0.6667	0.2857	0.2857
ST_F4_2002	2002	ST	ST_BEFORE	0.0455	0.1200	0.0146	0.0646	0.1667	0.1667	0.3333	0.1429	0.1429
ST_F5_2002	2002	ST	ST_BEFORE									
ST_G1_2002	2002	ST	ST_BEFORE	0.0897	0.2123	0.1166	0.3220	0.2354	0.2354	0.0000	0.4286	0.4286
ST_G2_2002	2002	ST	ST_BEFORE	0.0007	0.0021	0.1163	0.3206	0.1688	0.1688	0.0000	0.2857	0.2857
ST_G3_2002	2002	ST	ST_BEFORE	0.1938	0.3794	0.1166	0.3220	0.2354	0.2354	0.0000	0.4286	0.4286
ST_G4_2002	2002	ST	ST_BEFORE	0.0897	0.2123	0.0756	0.1669	0.1600	0.1600	0.0000	0.0000	0.0000
ST_G5_2002	2002	ST	ST_BEFORE	0.0931	0.2188	0.0969	0.2375	0.1600	0.1600	0.3333	0.1429	0.1429
ST_H1_2002	2002	ST	ST_BEFORE	0.0000	0.0000	0.0000	0.0000	0.0000	0.0000	0.0000	0.0000	0.0000
ST_H2_2002	2002	ST	ST_BEFORE	0.0000	0.0000	0.0000	0.0000	0.0000	0.0000	0.0000	0.0000	0.0000
ST_H3_2002	2002	ST	ST_BEFORE	0.0903	0.2144	0.0406	0.1537	0.0088	0.0088	0.0000	0.2857	0.2857
ST_H4_2002	2002	ST	ST_BEFORE	0.0000	0.0000	0.0000	0.0000	0.0000	0.0000	0.0000	0.0000	0.0000
ST_H5_2002	2002	ST	ST_BEFORE	0.0000	0.0000	0.0000	0.0000	0.0000	0.0000	0.0000	0.0000	0.0000
ST_I1_2010	2010	ST	ST_REWET	0.0897	0.2123	0.0756	0.1669	0.4330	0.4330	0.3333	0.2857	0.2857
ST_I2_2010	2010	ST	ST_REWET	0.1930	0.3829	0.1168	0.3224	0.5040	0.5040	0.3333	0.4286	0.4286
ST_I3_2010	2010	ST	ST_REWET	0.1938	0.3794	0.1163	0.3206	0.2283	0.2283	0.3333	0.4286	0.4286
ST_I4_2010	2010	ST	ST_REWET	0.0276	0.0792	0.1546	0.1878	0.4972	0.4972	0.3333	0.2857	0.2857
ST_I5_2010	2010	ST	ST_REWET									
ST_J1_2010	2010	ST	ST_REWET	0.0483	0.0994	0.2012	0.4200	0.4225	0.4225	0.0000	0.1429	0.1429
ST_J2_2010	2010	ST	ST_REWET	0.1241	0.2833	0.2660	0.4850	0.6572	0.6572	0.6667	0.4286	0.4286
ST_J3_2010	2010	ST	ST_REWET	0.1241	0.2833	0.2660	0.4850	0.6799	0.6799	0.6667	0.4286	0.4286
ST_J4_2010	2010	ST	ST_REWET	0.1000	0.2399	0.2515	0.4253	0.6572	0.6572	0.3333	0.4286	0.4286
ST_J5_2010	2010	ST	ST_REWET	0.0021	0.0064	0.0756	0.0773	0.0600	0.0600	0.3333	0.1429	0.1429
ST_K1_2010	2010	ST	ST_REWET	0.1241	0.2833	0.2712	0.5098	0.6572	0.6572	0.3333	0.4286	0.4286
ST_K2_2010	2010	ST	ST_REWET	0.1000	0.2399	0.2563	0.4437	0.7396	0.7396	0.6667	0.4286	0.4286
ST_K3_2010	2010	ST	ST_REWET	0.2062	0.4182	0.2709	0.5083	0.7045	0.7045	0.6667	0.4286	0.4286
ST_K4_2010	2010	ST	ST_REWET	0.1062	0.2597	0.2709	0.5083	0.7045	0.7045	0.6667	0.4286	0.4286
ST_K5_2010	2010	ST	ST_REWET	0.1931	0.3774	0.1181	0.3295	0.2056	0.2056	0.3333	0.4286	0.4286
ST_L1_2010	2010	ST	ST_REWET	0.1241	0.2833	0.2660	0.4850	0.6572	0.6572	0.3333	0.4286	0.4286
ST_L2_2010	2010	ST	ST_REWET	0.1000	0.2399	0.2712	0.5098	0.6660	0.6660	0.3333	0.4286	0.4286
ST_L3_2010	2010	ST	ST_REWET	0.0938	0.2209	0.0406	0.1537	0.1327	0.1327	0.3333	0.2857	0.2857
ST_L4_2010	2010	ST	ST_REWET	0.1000	0.2399	0.0953	0.2829	0.2070	0.2070	0.3333	0.2857	0.2857
ST_L5_2010	2010	ST	ST_REWET	0.2000	0.3984	0.0953	0.2829	0.2144	0.2144	0.3333	0.2857	0.2857
ST_M1_2002	2002	ST	ST_BEFORE	0.1000	0.2399	0.0969	0.2375	0.0743	0.0743	0.3333	0.2857	0.2857
ST_M2_2002	2002	ST	ST_BEFORE	0.1000	0.2399	0.2712	0.5098	0.6799	0.6799	0.3333	0.4286	0.4286

ST_M3_2002	2002	ST	ST_BEFORE	0.2000	0.3984	0.2712	0.5098	0.6660	0.6660	0.0000	0.2857	0.2857
ST_M4_2002	2002	ST	ST_BEFORE	0.1966	0.3878	0.0272	0.1114	0.2214	0.2214	0.3333	0.1429	0.1429
ST_M5_2002	2002	ST	ST_BEFORE	0.0000	0.0000	0.0000	0.0000	0.0000	0.0000	0.0000	0.0000	0.0000
ST_N1_2002	2002	ST	ST_BEFORE	0.0145	0.0416	0.1163	0.3206	0.1828	0.1828	0.0000	0.2857	0.2857
ST_N2_2002	2002	ST	ST_BEFORE	0.0628	0.1586	0.1163	0.3206	0.4558	0.4558	0.3333	0.2857	0.2857
ST_N3_2002	2002	ST	ST_BEFORE	0.1931	0.3774	0.1166	0.3220	0.2354	0.2354	0.0000	0.4286	0.4286
ST_N4_2002	2002	ST	ST_BEFORE	0.0204	0.0571	0.0879	0.2051	0.3555	0.3555	0.6667	0.1429	0.1429
ST_N5_2002	2002	ST	ST_BEFORE									
ST_O1_2002	2002	ST	ST_BEFORE									
ST_O2_2002	2002	ST	ST_BEFORE	0.0938	0.2209	0.1163	0.3206	0.1828	0.1828	0.3333	0.2857	0.2857
ST_O3_2002	2002	ST	ST_BEFORE	0.0903	0.2144	0.1163	0.3206	0.1688	0.1688	0.0000	0.2857	0.2857
ST_O4_2002	2002	ST	ST_BEFORE	0.0145	0.0416	0.0736	0.2380	0.0140	0.0140	0.0000	0.0000	0.0000
ST_O5_2002	2002	ST	ST_BEFORE	0.0062	0.0190	0.2709	0.5083	0.6660	0.6660	0.0000	0.0000	0.0000
ST_P1_2002	2002	ST	ST_BEFORE	0.0138	0.0395	0.0427	0.0826	0.1828	0.1828	0.0000	0.2857	0.2857
ST_P2_2002	2002	ST	ST_BEFORE	0.0138	0.0395	0.0427	0.0826	0.1828	0.1828	0.0000	0.2857	0.2857
ST_P3_2002	2002	ST	ST_BEFORE	0.1938	0.3794	0.1163	0.3206	0.2283	0.2283	0.0000	0.4286	0.4286
ST_P4_2002	2002	ST	ST_BEFORE	0.2000	0.3984	0.2709	0.5083	0.6799	0.6799	0.6667	0.4286	0.4286
ST_P5_2002	2002	ST	ST_BEFORE									
TA_279	1972	TA	TA_BEFORE	0.2069	0.3956	0.1957	0.4202	0.6731	0.6731	1.0000	0.4286	0.4286
TA_280	1972	TA	TA_BEFORE	0.2083	0.3997	0.2237	0.4509	0.6672	0.6672	1.0000	0.5714	0.5714
TA_281	1972	TA	TA_BEFORE	0.2083	0.3997	0.2237	0.4509	0.6731	0.6731	1.0000	0.5714	0.5714
TA_282	1972	TA	TA_BEFORE	0.2083	0.3997	0.2413	0.4687	0.7225	0.7225	1.0000	0.8571	0.8571
TA_283	1972	TA	TA_BEFORE	0.2083	0.3997	0.2175	0.4444	0.6731	0.6731	1.0000	0.4286	0.4286
TA_284	1972	TA	TA_BEFORE	0.2069	0.3956	0.2237	0.4509	0.6731	0.6731	0.6667	0.4286	0.4286
TA_285	1972	TA	TA_BEFORE	0.2069	0.3956	0.1957	0.4202	0.6672	0.6672	1.0000	0.5714	0.5714
TA_286	1972	TA	TA_BEFORE	0.2145	0.4188	0.1957	0.4202	0.6672	0.6672	1.0000	0.5714	0.5714
TA_287	1972	TA	TA_BEFORE	0.1172	0.2622	0.1616	0.3780	0.7646	0.7646	1.0000	0.5714	0.5714
TA_288	1972	TA	TA_BEFORE	0.1248	0.2855	0.2669	0.4927	0.7646	0.7646	1.0000	0.5714	0.5714
TA_289	1972	TA	TA_BEFORE	0.1248	0.2855	0.2669	0.4927	0.7646	0.7646	1.0000	0.5714	0.5714
TA_290	1972	TA	TA_BEFORE	0.2069	0.3956	0.2669	0.4927	0.7646	0.7646	1.0000	0.5714	0.5714
TA_291	1972	TA	TA_BEFORE	0.1248	0.2855	0.2669	0.4927	0.7646	0.7646	1.0000	0.5714	0.5714
TA_292	1972	TA	TA_BEFORE	0.1248	0.2855	0.2669	0.4927	0.7646	0.7646	1.0000	0.5714	0.5714
TA_293	1972	TA	TA_BEFORE	0.1862	0.3680	0.2669	0.4927	0.7646	0.7646	1.0000	0.5714	0.5714
TA_294	1972	TA	TA_BEFORE	0.2083	0.3997	0.1855	0.2801	0.7225	0.7225	1.0000	0.8571	0.8571
TA_295	1972	TA	TA_BEFORE	0.2083	0.3997	0.2393	0.5348	0.6672	0.6672	1.0000	0.5714	0.5714
TA_296	1972	TA	TA_BEFORE	0.2083	0.3997	0.1855	0.2801	0.7225	0.7225	1.0000	0.8571	0.8571
TA_297	1972	TA	TA_BEFORE	0.2083	0.3997	0.1855	0.2801	0.7225	0.7225	1.0000	0.7143	0.7143
TA_298	1972	TA	TA_BEFORE	0.2069	0.3956	0.2163	0.4178	0.6672	0.6672	1.0000	0.4286	0.4286
TA_299	1972	TA	TA_BEFORE	0.2083	0.3997	0.1882	0.3871	0.6672	0.6672	1.0000	0.5714	0.5714
TA_300	1972	TA	TA_BEFORE	0.2069	0.3956	0.2325	0.3634	0.6672	0.6672	1.0000	0.5714	0.5714
TA_301	1972	TA	TA_BEFORE	0.2069	0.3956	0.2594	0.4596	0.6672	0.6672	1.0000	0.5714	0.5714
TA_302	1972	TA	TA_BEFORE	0.2145	0.4188	0.1894	0.3217	0.6672	0.6672	1.0000	0.5714	0.5714
TA_303	1972	TA	TA_BEFORE	0.1248	0.2855	0.2594	0.4596	0.6672	0.6672	1.0000	0.5714	0.5714
TA_304	1972	TA	TA_BEFORE	0.1248	0.2855	0.2594	0.4596	0.6672	0.6672	1.0000	0.5714	0.5714
TA_305	1972	TA	TA_BEFORE	0.2145	0.4188	0.2594	0.4596	0.6672	0.6672	1.0000	0.5714	0.5714
TA_306	1972	TA	TA_BEFORE	0.1726	0.3611	0.2594	0.4596	0.6672	0.6672	1.0000	0.5714	0.5714
TA_307	1972	TA	TA_BEFORE	0.1248	0.2855	0.2594	0.4596	0.6672	0.6672	1.0000	0.5714	0.5714
TA_308	1972	TA	TA_BEFORE	0.1186	0.2664	0.2594	0.4596	0.6672	0.6672	1.0000	0.5714	0.5714
TA_937	2009	TA	TA_REWET	0.1172	0.2622	0.2112	0.5041	0.7646	0.7646	1.0000	0.4286	0.4286
TA_938	2009	TA	TA_REWET	0.1172	0.2622	0.1818	0.4681	0.7646	0.7646	0.6667	0.5714	0.5714
TA_939	2009	TA	TA_REWET	0.1379	0.2965	0.1771	0.4619	0.6672	0.6672	0.6667	0.5714	0.5714
TA_940	2009	TA	TA_REWET	0.1172	0.2622	0.1589	0.3512	0.6672	0.6672	1.0000	0.4286	0.4286
TA_941	2009	TA	TA_REWET	0.1172	0.2622	0.1541	0.3449	0.6672	0.6672	1.0000	0.4286	0.4286
TA_942	2009	TA	TA_REWET	0.1172	0.2622	0.1818	0.4681	0.6672	0.6672	0.6667	0.5714	0.5714
TA_943	2009	TA	TA_REWET	0.1172	0.2622	0.2393	0.5348	0.6672	0.6672	1.0000	0.4286	0.4286
TA_944	2009	TA	TA_REWET	0.1172	0.2622	0.1818	0.4681	0.6672	0.6672	1.0000	0.4286	0.4286
TA_945	2009	TA	TA_REWET	0.1379	0.2965	0.2112	0.5041	0.6672	0.6672	0.6667	0.5714	0.5714
TA_946	2009	TA	TA_REWET	0.1310	0.2763	0.2237	0.4509	0.6672	0.6672	1.0000	0.4286	0.4286
TA_947	2009	TA	TA_REWET	0.1148	0.2551	0.2237	0.4509	0.6881	0.6881	0.6667	0.5714	0.5714
TA_948	2009	TA	TA_REWET	0.1148	0.2551	0.2469	0.5866	0.7646	0.7646	1.0000	0.5714	0.5714
TA_949	2009	TA	TA_REWET	0.1148	0.2551	0.2393	0.5348	0.6881	0.6881	1.0000	0.4286	0.4286
TA_950	2009	TA	TA_REWET	0.1355	0.2894	0.2393	0.5348	0.7646	0.7646	1.0000	0.4286	0.4286
TA_951	2009	TA	TA_REWET	0.1379	0.2965	0.2163	0.4178	0.5719	0.5719	1.0000	0.5714	0.5714
TA_952	2009	TA	TA_REWET	0.2069	0.3956	0.2303	0.5253	0.7610	0.7610	1.0000	0.5714	0.5714
TA_953	2009	TA	TA_REWET	0.1148	0.2551	0.1818	0.4681	0.7646	0.7646	0.6667	0.4286	0.4286
TA_954	2009	TA	TA_REWET	0.1172	0.2622	0.1899	0.4784	0.7646	0.7646	0.6667	0.5714	0.5714
TA_955	2009	TA	TA_REWET	0.1172	0.2622	0.2112	0.5041	0.7646	0.7646	0.6667	0.4286	0.4286
TA_956	2009	TA	TA_REWET	0.1172	0.2622	0.2237	0.4509	0.7646	0.7646	0.6667	0.5714	0.5714
TA_957	2009	TA	TA_REWET	0.1172	0.2622	0.2469	0.5866	0.7646	0.7646	0.6667	0.5714	0.5714
TA_958	2009	TA	TA_REWET	0.1172	0.2622	0.2188	0.5559	0.7646	0.7646	0.6667	0.4286	0.4286
TA_959	2009	TA	TA_REWET	0.1172	0.2622	0.2469	0.5866	0.7646	0.7646	0.6667	0.5714	0.5714
TA_960	2009	TA	TA_REWET	0.1172	0.2622	0.2393	0.5348	0.7646	0.7646	1.0000	0.4286	0.4286
TA_961	2009	TA	TA_REWET	0.1172	0.2622	0.2393	0.5348	0.7646	0.7646	1.0000	0.5714	0.5714
TA_962	2009	TA	TA_REWET	0.1241	0.2833	0.1957	0.4202	0.7646	0.7646	1.0000	0.4286	0.4286
TA_963	2009	TA	TA_REWET	0.1159	0.2581	0.1957	0.4202	0.7022	0.7022	1.0000	0.4286	0.4286
TA_964	2009	TA	TA_REWET	0.1159	0.2581	0.1957	0.4202	0.6049	0.6049	1.0000	0.4286	0.4286
TA_965	2009	TA	TA_REWET	0.1159	0.2581	0.2188	0.5559	0.6672	0.6672	1.0000	0.5714	0.5714

TA_966	2009	TA	TA_REWET	0.1159	0.2581	0.2188	0.5559	0.6672	0.6672	1.0000	0.4286	0.4286
TR_1_1998	1998	TR	TR_BEFORE	0.0690	0.1711	0.0708	0.1142	0.3484	0.3484	0.6667	0.2857	0.2857
TR_10_1998	1998	TR	TR_BEFORE	0.0690	0.1711	0.0708	0.1142	0.3235	0.3235	0.6667	0.4286	0.4286
TR_102_2008	2008	TR	TR_REWET	0.1586	0.3627	0.5009	0.6451	0.6773	0.6773	0.6667	0.4286	0.4286
TR_103_1995	1995	TR	TR_BEFORE	0.1317	0.3076	0.2012	0.4200	0.7219	0.7219	0.6667	0.4286	0.4286
TR_109_2008	2008	TR	TR_REWET	0.1379	0.3285	0.5009	0.6451	0.6773	0.6773	0.6667	0.4286	0.4286
TR_110_1995	1995	TR	TR_BEFORE	0.1581	0.3177	0.2219	0.4424	0.5013	0.5013	0.6667	0.4286	0.4286
TR_116_2008	2008	TR	TR_REWET	0.1066	0.2316	0.2368	0.4575	0.5641	0.5641	0.6667	0.4286	0.4286
TR_117_1995	1995	TR	TR_BEFORE	0.1167	0.2119	0.2012	0.4200	0.6067	0.6067	0.6667	0.4286	0.4286
TR_12_2008	2008	TR	TR_REWET	0.1650	0.3379	0.3057	0.5970	0.6291	0.6291	1.0000	0.5714	0.5714
TR_123_2008	2008	TR	TR_REWET	0.1379	0.2421	0.2272	0.4478	0.6959	0.6959	0.6667	0.4286	0.4286
TR_124_1995	1995	TR	TR_BEFORE	0.1650	0.3379	0.2148	0.5082	0.5372	0.5372	1.0000	0.4286	0.4286
TR_13_1998	1998	TR	TR_BEFORE	0.1650	0.3379	0.1749	0.4590	0.4336	0.4336	0.6667	0.1429	0.1429
TR_130_2008	2008	TR	TR_REWET	0.2102	0.4387	0.5619	0.7597	0.6396	0.6396	0.6667	0.4286	0.4286
TR_131_1995	1995	TR	TR_BEFORE	0.2276	0.4618	0.5183	0.7376	0.6303	0.6303	0.6667	0.4286	0.4286
TR_137_2008	2008	TR	TR_REWET	0.2276	0.4618	0.9511	0.7457	0.6691	0.6691	0.6667	0.4286	0.4286
TR_138_1995	1995	TR	TR_BEFORE	0.2276	0.4618	0.4605	0.5012	0.6303	0.6303	0.6667	0.4286	0.4286
TR_144_2008	2008	TR	TR_REWET	0.1517	0.3516	0.9307	0.6818	0.6691	0.6691	0.6667	0.4286	0.4286
TR_145_1995	1995	TR	TR_BEFORE	0.2276	0.4618	0.4608	0.5022	0.5824	0.5824	0.6667	0.4286	0.4286
TR_15_2008	2008	TR	TR_REWET	0.1570	0.3145	0.2393	0.5348	0.5338	0.5338	0.6667	0.5714	0.5714
TR_151_2008	2008	TR	TR_REWET	0.1517	0.3516	0.4683	0.5242	0.5519	0.5519	0.6667	0.4286	0.4286
TR_152_1995	1995	TR	TR_BEFORE	0.2276	0.4618	0.4523	0.4789	0.5519	0.5519	0.6667	0.2857	0.2857
TR_158_2008	2008	TR	TR_REWET	0.2276	0.4618	0.9655	0.8012	0.6691	0.6691	0.6667	0.4286	0.4286
TR_159_1995	1995	TR	TR_BEFORE	0.1650	0.3379	0.1251	0.1893	0.7753	0.7753	1.0000	0.2857	0.2857
TR_16_1998	1998	TR	TR_BEFORE	0.0828	0.1990	0.1002	0.1780	0.4507	0.4507	0.6667	0.5714	0.5714
TR_165_2008	2008	TR	TR_REWET	0.1981	0.3699	0.2542	0.5500	0.8012	0.8012	0.6667	0.4286	0.4286
TR_166_1995	1995	TR	TR_BEFORE	0.2069	0.3956	0.2393	0.5348	0.5162	0.5162	0.6667	0.2857	0.2857
TR_172_2008	2008	TR	TR_REWET	0.2276	0.4618	0.5183	0.7376	0.5824	0.5824	1.0000	0.4286	0.4286
TR_173_1995	1995	TR	TR_BEFORE	0.0666	0.1456	0.0754	0.1428	0.3342	0.3342	0.6667	0.2857	0.2857
TR_179_2008	2008	TR	TR_REWET	0.0931	0.2188	0.0850	0.1252	0.4507	0.4507	0.6667	0.4286	0.4286
TR_18_2008	2008	TR	TR_REWET	0.1379	0.2965	0.2078	0.3840	0.4427	0.4427	0.6667	0.5714	0.5714
TR_180_1995	1995	TR	TR_BEFORE	0.1650	0.3379	0.2569	0.5526	0.8012	0.8012	1.0000	0.8571	0.8571
TR_186_2008	2008	TR	TR_REWET	0.1172	0.2622	0.1506	0.2918	0.7459	0.7459	0.6667	0.4286	0.4286
TR_187_1995	1995	TR	TR_BEFORE	0.1172	0.2622	0.1544	0.2110	0.8012	0.8012	1.0000	0.7143	0.7143
TR_19_1998	1998	TR	TR_BEFORE	0.1862	0.3680	0.0864	0.1466	0.7241	0.7241	0.6667	0.4286	0.4286
TR_193_2008	2008	TR	TR_REWET	0.1069	0.2441	0.1253	0.2463	0.7459	0.7459	0.6667	0.4286	0.4286
TR_194_1995	1995	TR	TR_BEFORE	0.1172	0.2622	0.1437	0.2699	0.4766	0.4766	1.0000	0.4286	0.4286
TR_200_2008	2008	TR	TR_REWET	0.1172	0.2622	0.1437	0.2699	0.7459	0.7459	0.3333	0.4286	0.4286
TR_201_1995	1995	TR	TR_BEFORE	0.1172	0.2622	0.1511	0.2803	0.7459	0.7459	1.0000	0.4286	0.4286
TR_207_2008	2008	TR	TR_REWET	0.2069	0.3956	0.1895	0.4130	0.7459	0.7459	0.6667	0.5714	0.5714
TR_208_1995	1995	TR	TR_BEFORE	0.1650	0.3379	0.1912	0.2946	0.5824	0.5824	1.0000	0.8571	0.8571
TR_21_2008	2008	TR	TR_REWET	0.1172	0.2622	0.1323	0.2682	0.4608	0.4608	0.6667	0.5714	0.5714
TR_214_2008	2008	TR	TR_REWET	0.1319	0.2480	0.2446	0.5403	0.6409	0.6409	0.6667	0.4286	0.4286
TR_215_1995	1995	TR	TR_BEFORE	0.1650	0.3379	0.0815	0.1359	0.4401	0.4401	0.3333	0.1429	0.1429
TR_22_1998	1998	TR	TR_BEFORE	0.1172	0.2622	0.0936	0.1625	0.3742	0.3742	1.0000	0.5714	0.5714
TR_221_2008	2008	TR	TR_REWET	0.1650	0.3379	0.2393	0.5348	0.5319	0.5319	1.0000	0.2857	0.2857
TR_222_1995	1995	TR	TR_BEFORE	0.1650	0.3379	0.1497	0.2759	0.7459	0.7459	0.6667	0.4286	0.4286
TR_228_2008	2008	TR	TR_REWET	0.1290	0.2409	0.1312	0.2528	0.4895	0.4895	0.6667	0.2857	0.2857
TR_229_1995	1995	TR	TR_BEFORE	0.1131	0.2586	0.2723	0.4176	0.3695	0.3695	1.0000	0.5714	0.5714
TR_23_2008	2008	TR	TR_REWET	0.1794	0.3832	0.1749	0.4590	0.3977	0.3977	0.6667	0.2857	0.2857
TR_235_2008	2008	TR	TR_REWET	0.1459	0.3130	0.2735	0.4187	0.4207	0.4207	1.0000	0.5714	0.5714
TR_236_1995	1995	TR	TR_BEFORE	0.1186	0.2664	0.1818	0.2711	0.5060	0.5060	1.0000	0.8571	0.8571
TR_24_1998	1998	TR	TR_BEFORE	0.1650	0.3379	0.1597	0.3756	0.6436	0.6436	1.0000	0.5714	0.5714
TR_242_2008	2008	TR	TR_REWET	0.1172	0.2622	0.1886	0.3634	0.4359	0.4359	1.0000	0.5714	0.5714
TR_243_1995	1995	TR	TR_BEFORE	0.1650	0.3379	0.1784	0.2630	0.5319	0.5319	1.0000	0.7143	0.7143
TR_249_2008	2008	TR	TR_REWET	0.2069	0.3956	0.1988	0.3745	0.6291	0.6291	0.6667	0.5714	0.5714
TR_250_1995	1995	TR	TR_BEFORE	0.0836	0.2006	0.1438	0.3087	0.4766	0.4766	0.6667	0.4286	0.4286
TR_256_2008	2008	TR	TR_REWET	0.1447	0.3172	0.2303	0.5253	0.6291	0.6291	1.0000	0.5714	0.5714
TR_257_1995	1995	TR	TR_BEFORE	0.1650	0.3379	0.0968	0.1699	0.4766	0.4766	0.3333	0.4286	0.4286
TR_26_2008	2008	TR	TR_REWET	0.1815	0.3492	0.2508	0.6167	0.7396	0.7396	0.6667	0.4286	0.4286
TR_263_2008	2008	TR	TR_REWET	0.1650	0.3379	0.2148	0.4414	0.5092	0.5092	0.6667	0.5714	0.5714
TR_264_1995	1995	TR	TR_BEFORE	0.1017	0.2348	0.0882	0.1502	0.6672	0.6672	0.3333	0.4286	0.4286
TR_27_1998	1998	TR	TR_BEFORE	0.1650	0.3379	0.0968	0.1699	0.4825	0.4825	0.6667	0.4286	0.4286
TR_270_2008	2008	TR	TR_REWET	0.1650	0.3379	0.2148	0.5082	0.5193	0.5193	0.6667	0.5714	0.5714
TR_271_1995	1995	TR	TR_BEFORE	0.1017	0.2348	0.1319	0.2550	0.6672	0.6672	0.6667	0.4286	0.4286
TR_277_2008	2008	TR	TR_REWET	0.1570	0.3146	0.2148	0.5082	0.6422	0.6422	0.6667	0.2857	0.2857
TR_278_1995	1995	TR	TR_BEFORE	0.1650	0.3379	0.1870	0.3616	0.7753	0.7753	1.0000	0.2857	0.2857
TR_284_2008	2008	TR	TR_REWET	0.1650	0.3379	0.2996	0.5917	0.6131	0.6131	0.6667	0.4286	0.4286
TR_285_1995	1995	TR	TR_BEFORE	0.1650	0.3379	0.1691	0.2717	0.5565	0.5565	1.0000	0.4286	0.4286
TR_29_2008	2008	TR	TR_REWET	0.1618	0.3285	0.1742	0.2772	0.6363	0.6363	1.0000	0.4286	0.4286
TR_291_2008	2008	TR	TR_REWET	0.1862	0.3375	0.1573	0.2876	0.5372	0.5372	1.0000	0.4286	0.4286
TR_3_2008	2008	TR	TR_REWET	0.0869	0.2070	0.2263	0.5901	0.6041	0.6041	0.3333	0.5714	0.5714
TR_30_1998	1998	TR	TR_BEFORE	0.1650	0.3379	0.0968	0.1699	0.4825	0.4825	0.6667	0.2857	0.2857
TR_32_2008	2008	TR	TR_REWET	0.1650	0.3379	0.2403	0.5358	0.7384	0.7384	1.0000	0.2857	0.2857
TR_33_1998	1998	TR	TR_BEFORE	0.0869	0.2070	0.2569	0.5526	0.4812	0.4812	1.0000	0.8571	0.8571
TR_35_2008	2008	TR	TR_REWET	0.1603	0.3240	0.3057	0.5970	0.6392	0.6392	0.6667	0.5714	0.5714
TR_36_1998	1998	TR	TR_BEFORE	0.0836	0.2006	0.1243	0.2436	0.5380	0.5380	0.6667	0.4286	0.4286

TR_38_2008	2008	TR	TR_REWET	0.0904	0.2213	0.1988	0.3745	0.6585	0.6585	1.0000	0.5714	0.5714
TR_39_1998	1998	TR	TR_BEFORE	0.0817	0.1968	0.1246	0.2441	0.7459	0.7459	0.6667	0.1429	0.1429
TR_4_1998	1998	TR	TR_BEFORE	0.0781	0.1852	0.1818	0.2711	0.4368	0.4368	1.0000	0.8571	0.8571
TR_41_2008	2008	TR	TR_REWET	0.0466	0.1123	0.1988	0.3745	0.6256	0.6256	1.0000	0.2857	0.2857
TR_42_1998	1998	TR	TR_BEFORE	0.1650	0.3379	0.0815	0.1359	0.4507	0.4507	0.6667	0.2857	0.2857
TR_44_2008	2008	TR	TR_REWET	0.2102	0.4387	0.4948	0.6184	0.3908	0.3908	1.0000	0.4286	0.4286
TR_45_2008	2008	TR	TR_REWET	0.2102	0.4387	0.5183	0.7376	0.7657	0.7657	0.6667	0.4286	0.4286
TR_46_2008	2008	TR	TR_REWET	0.1014	0.2524	0.2393	0.5348	0.5242	0.5242	0.6667	0.5714	0.5714
TR_47_1995	1995	TR	TR_BEFORE	0.1650	0.3379	0.1253	0.2463	0.7459	0.7459	0.6667	0.2857	0.2857
TR_53_2008	2008	TR	TR_REWET	0.1650	0.3379	0.1649	0.2950	0.5380	0.5380	0.6667	0.4286	0.4286
TR_54_1995	1995	TR	TR_BEFORE	0.1066	0.2316	0.2368	0.4575	0.6519	0.6519	1.0000	0.4286	0.4286
TR_6_2008	2008	TR	TR_REWET	0.0931	0.2188	0.1818	0.2711	0.4368	0.4368	1.0000	0.8571	0.8571
TR_60_2008	2008	TR	TR_REWET	0.2102	0.4387	0.9711	0.8257	0.7827	0.7827	0.6667	0.4286	0.4286
TR_67_2008	2008	TR	TR_REWET	0.2069	0.3956	0.1818	0.4681	0.5682	0.5682	0.6667	0.1429	0.1429
TR_68_1995	1995	TR	TR_BEFORE	0.5114	0.6412	0.1798	0.3014	0.4445	0.4445	1.0000	0.2857	0.2857
TR_7_1998	1998	TR	TR_BEFORE	0.0690	0.1711	0.1416	0.1865	0.4368	0.4368	1.0000	0.8571	0.8571
TR_74_2008	2008	TR	TR_REWET	0.5222	0.6708	0.2313	0.4330	0.4842	0.4842	1.0000	0.4286	0.4286
TR_75_1995	1995	TR	TR_BEFORE	0.2069	0.3956	0.1437	0.2699	0.5431	0.5431	0.6667	0.4286	0.4286
TR_81_2008	2008	TR	TR_REWET	0.1561	0.3122	0.1649	0.2950	0.5130	0.5130	0.6667	0.5714	0.5714
TR_82_1995	1995	TR	TR_BEFORE	0.1650	0.3379	0.1430	0.2677	0.4615	0.4615	0.6667	0.4286	0.4286
TR_88_2008	2008	TR	TR_REWET	0.1385	0.2646	0.1649	0.2950	0.5130	0.5130	0.6667	0.2857	0.2857
TR_89_1995	1995	TR	TR_BEFORE	0.1650	0.3379	0.1002	0.1600	0.4507	0.4507	0.6667	0.2857	0.2857
TR_9_2008	2008	TR	TR_REWET	0.1650	0.3379	0.1438	0.3087	0.7459	0.7459	0.3333	0.5714	0.5714
TR_95_2008	2008	TR	TR_REWET	0.1512	0.2983	0.1833	0.3574	0.6291	0.6291	0.3333	0.5714	0.5714
TR_96_1995	1995	TR	TR_BEFORE	0.5232	0.6737	0.2963	0.5111	0.6773	0.6773	1.0000	0.4286	0.4286
UL_A1_2010	2010	UL	UL_REWET	0.0979	0.2335	0.1807	0.3664	0.4733	0.4733	0.0000	0.2857	0.2857
UL_A1a_2010	2010	UL	UL_REWET	0.2000	0.3984	0.2810	0.5628	0.5504	0.5504	0.3333	0.4286	0.4286
UL_A1b_2010	2010	UL	UL_REWET	0.1000	0.2399	0.2712	0.5098	0.6660	0.6660	0.3333	0.2857	0.2857
UL_A2_2010	2010	UL	UL_REWET	0.2062	0.4182	0.2727	0.5173	0.6549	0.6549	0.3333	0.2857	0.2857
UL_A2a_2010	2010	UL	UL_REWET	0.2062	0.4182	0.2160	0.4049	0.5718	0.5718	0.0000	0.2857	0.2857
UL_A2b_2010	2010	UL	UL_REWET	0.2000	0.3984	0.0971	0.2918	0.3922	0.3922	0.3333	0.2857	0.2857
UL_A3_2010	2010	UL	UL_REWET	0.1062	0.2597	0.2160	0.4049	0.5718	0.5718	0.0000	0.2857	0.2857
UL_A3a_2010	2010	UL	UL_REWET	0.1062	0.2597	0.2308	0.4710	0.7045	0.7045	0.3333	0.2857	0.2857
UL_A3b_2010	2010	UL	UL_REWET	0.1062	0.2597	0.2323	0.4785	0.7045	0.7045	0.3333	0.2857	0.2857
UL_A4_2010	2010	UL	UL_REWET	0.1966	0.3878	0.0482	0.2006	0.1648	0.1648	0.3333	0.4286	0.4286
UL_A4a_2010	2010	UL	UL_REWET	0.2062	0.4182	0.2406	0.5241	0.7045	0.7045	0.3333	0.4286	0.4286
UL_A4b_2010	2010	UL	UL_REWET	0.1966	0.3878	0.0369	0.1644	0.1648	0.1648	0.0000	0.4286	0.4286
UL_A5_2010	2010	UL	UL_REWET	0.1062	0.2597	0.2406	0.5241	0.7045	0.7045	0.3333	0.4286	0.4286
UL_A5a_2010	2010	UL	UL_REWET	0.2062	0.4182	0.2406	0.5241	0.7184	0.7184	0.3333	0.4286	0.4286
UL_A5b_2010	2010	UL	UL_REWET	0.2100	0.4307	0.2406	0.5241	0.7045	0.7045	0.3333	0.5714	0.5714
UL_B1_2010	2010	UL	UL_REWET	0.0841	0.1928	0.2064	0.4448	0.4237	0.4237	0.3333	0.4286	0.4286
UL_B1a_2010	2010	UL	UL_REWET	0.0979	0.2335	0.1915	0.3787	0.4745	0.4745	0.0000	0.4286	0.4286
UL_B1b_2010	2010	UL	UL_REWET	0.0966	0.2293	0.1867	0.3603	0.4727	0.4727	0.3333	0.4286	0.4286
UL_B2_2010	2010	UL	UL_REWET	0.1000	0.2399	0.2709	0.5083	0.6660	0.6660	0.3333	0.1429	0.1429
UL_B2a_2010	2010	UL	UL_REWET	0.0979	0.2335	0.1807	0.3664	0.5236	0.5236	0.6667	0.4286	0.4286
UL_B2b_2010	2010	UL	UL_REWET	0.0586	0.1256	0.0524	0.1583	0.0340	0.0340	0.0000	0.1429	0.1429
UL_B3_2010	2010	UL	UL_REWET	0.1938	0.3794	0.0789	0.2496	0.2144	0.2144	0.3333	0.4286	0.4286
UL_B3a_2010	2010	UL	UL_REWET	0.1586	0.2842	0.0643	0.1849	0.0829	0.0829	0.0000	0.4286	0.4286
UL_B3b_2010	2010	UL	UL_REWET	0.1241	0.2833	0.2604	0.4605	0.6799	0.6799	0.3333	0.4286	0.4286
UL_B4_2010	2010	UL	UL_REWET	0.0752	0.1974	0.2308	0.4710	0.6220	0.6220	0.3333	0.2857	0.2857
UL_B4a_2010	2010	UL	UL_REWET	0.0966	0.2293	0.0381	0.1461	0.1744	0.1744	0.3333	0.2857	0.2857
UL_B4b_2010	2010	UL	UL_REWET	0.0752	0.1974	0.2323	0.4785	0.6549	0.6549	0.3333	0.1429	0.1429
UL_B5_2010	2010	UL	UL_REWET	0.0448	0.1179	0.1264	0.3751	0.1192	0.1192	0.3333	0.5714	0.5714
UL_B5a_2010	2010	UL	UL_REWET	0.0655	0.1669	0.1264	0.3751	0.3594	0.3594	0.3333	0.5714	0.5714
UL_B5b_2010	2010	UL	UL_REWET	0.0579	0.1588	0.2406	0.5241	0.7045	0.7045	0.3333	0.4286	0.4286
UL_C1_2010	2010	UL	UL_REWET	0.1966	0.3878	0.0524	0.1872	0.3091	0.3091	0.3333	0.2857	0.2857
UL_C1a_2010	2010	UL	UL_REWET	0.2000	0.3984	0.0807	0.2183	0.3091	0.3091	0.3333	0.2857	0.2857
UL_C1b_2010	2010	UL	UL_REWET	0.1841	0.3513	0.0524	0.1872	0.3091	0.3091	0.3333	0.2857	0.2857
UL_C2_2010	2010	UL	UL_REWET	0.2062	0.4182	0.2305	0.4696	0.7045	0.7045	0.3333	0.2857	0.2857
UL_C2a_2010	2010	UL	UL_REWET	0.1062	0.2597	0.2305	0.4696	0.7045	0.7045	0.3333	0.1429	0.1429
UL_C2b_2010	2010	UL	UL_REWET	0.0586	0.1256	0.0524	0.1583	0.3091	0.3091	0.0000	0.1429	0.1429
UL_C3_2010	2010	UL	UL_REWET	0.1966	0.3878	0.0272	0.1114	0.2214	0.2214	0.3333	0.2857	0.2857
UL_C3a_2010	2010	UL	UL_REWET	0.1586	0.2842	0.0375	0.1211	0.3594	0.3594	0.3333	0.2857	0.2857
UL_C3b_2010	2010	UL	UL_REWET	0.0628	0.1586	0.0622	0.2403	0.4418	0.4418	0.3333	0.4286	0.4286
UL_C4_2010	2010	UL	UL_REWET	0.2062	0.4182	0.2323	0.4785	0.6549	0.6549	0.3333	0.5714	0.5714
UL_C4a_2010	2010	UL	UL_REWET	0.2062	0.4182	0.2308	0.4710	0.6220	0.6220	0.3333	0.2857	0.2857
UL_C4b_2010	2010	UL	UL_REWET	0.2062	0.4182	0.2323	0.4785	0.6549	0.6549	0.3333	0.2857	0.2857
UL_C5_2010	2010	UL	UL_REWET	0.2062	0.4182	0.2323	0.4785	0.7045	0.7045	0.3333	0.2857	0.2857
UL_C5a_2010	2010	UL	UL_REWET	0.2062	0.4182	0.2323	0.4785	0.7045	0.7045	0.3333	0.2857	0.2857
UL_C5b_2010	2010	UL	UL_REWET	0.1966	0.3878	0.0369	0.1644	0.1718	0.1718	0.3333	0.4286	0.4286
UL_D1_2010	2010	UL	UL_REWET	0.1938	0.3794	0.1163	0.3206	0.4418	0.4418	0.3333	0.4286	0.4286
UL_D1a_2010	2010	UL	UL_REWET	0.1938	0.3794	0.1181	0.3295	0.4418	0.4418	0.3333	0.4286	0.4286
UL_D1b_2010	2010	UL	UL_REWET	0.1938	0.3794	0.1166	0.3220	0.2214	0.2214	0.3333	0.4286	0.4286
UL_D2_2010	2010	UL	UL_REWET	0.1938	0.3794	0.1264	0.3751	0.2354	0.2354	0.3333	0.5714	0.5714
UL_D2a_2010	2010	UL	UL_REWET	0.1938	0.3794	0.1181	0.3295	0.2214	0.2214	0.3333	0.4286	0.4286
UL_D2b_2010	2010	UL	UL_REWET	0.0938	0.2209	0.1181	0.3295	0.4558	0.4558	0.3333	0.4286	0.4286
UL_D3_2010	2010	UL	UL_REWET	0.2062	0.4182	0.2160	0.4049	0.6220	0.6220	0.3333	0.2857	0.2857

UL_D3a_2010	2010	UL	UL_REWET	0.0628	0.1586	0.0524	0.1872	0.4418	0.4418	0.3333	0.1429	0.1429
UL_D3b_2010	2010	UL	UL_REWET	0.2062	0.4182	0.2308	0.4710	0.7045	0.7045	0.3333	0.2857	0.2857
UL_D4_2010	2010	UL	UL_REWET	0.2062	0.4182	0.2323	0.4785	0.6549	0.6549	0.0000	0.4286	0.4286
UL_D4b_2010	2010	UL	UL_REWET	0.2062	0.4182	0.2323	0.4785	0.7045	0.7045	0.3333	0.4286	0.4286
UL_D5_2010	2010	UL	UL_REWET	0.1966	0.3878	0.0369	0.1644	0.1648	0.1648	0.0000	0.4286	0.4286
UL_D5a_2010	2010	UL	UL_REWET	0.2062	0.4182	0.2406	0.5241	0.5890	0.5890	0.0000	0.5714	0.5714
UL_D5b_2010	2010	UL	UL_REWET	0.0655	0.1669	0.0622	0.2403	0.4418	0.4418	0.3333	0.4286	0.4286
UL_E1_2002	2002	UL	UL_BEFORE	0.1938	0.3794	0.1166	0.3220	0.4418	0.4418	0.3333	0.4286	0.4286
UL_E2_2002	2002	UL	UL_BEFORE	0.2000	0.3984	0.1163	0.3206	0.4558	0.4558	0.3333	0.4286	0.4286
UL_E3_2002	2002	UL	UL_BEFORE	0.1938	0.3794	0.0739	0.2395	0.2354	0.2354	0.3333	0.2857	0.2857
UL_E4_2002	2002	UL	UL_BEFORE	0.1586	0.2842	0.0643	0.1849	0.0829	0.0829	0.0000	0.4286	0.4286
UL_E5_2002	2002	UL	UL_BEFORE	0.1938	0.3794	0.0792	0.2511	0.4418	0.4418	0.3333	0.4286	0.4286
UL_F1_2002	2002	UL	UL_BEFORE	0.2000	0.3984	0.1166	0.3220	0.2214	0.2214	0.0000	0.4286	0.4286
UL_F2_2002	2002	UL	UL_BEFORE	0.0138	0.0395	0.1181	0.3295	0.2354	0.2354	0.3333	0.4286	0.4286
UL_F3_2002	2002	UL	UL_BEFORE	0.1938	0.3794	0.1166	0.3220	0.2354	0.2354	0.3333	0.4286	0.4286
UL_F4_2002	2002	UL	UL_BEFORE	0.2062	0.4182	0.2323	0.4785	0.6549	0.6549	0.0000	0.2857	0.2857
UL_F5_2002	2002	UL	UL_BEFORE	0.2062	0.4182	0.2160	0.4049	0.5730	0.5730	0.0000	0.4286	0.4286
UL_G1_2002	2002	UL	UL_BEFORE	0.0552	0.1470	0.2709	0.5083	0.6799	0.6799	0.6667	0.2857	0.2857
UL_G2_2002	2002	UL	UL_BEFORE	0.0517	0.1390	0.2712	0.5098	0.6799	0.6799	0.3333	0.4286	0.4286
UL_G3_2002	2002	UL	UL_BEFORE	0.2000	0.3984	0.2712	0.5098	0.6660	0.6660	0.3333	0.4286	0.4286
UL_G4_2002	2002	UL	UL_BEFORE	0.1980	0.3922	0.1168	0.3224	0.4442	0.4442	0.6667	0.2857	0.2857
UL_G5_2002	2002	UL	UL_BEFORE	0.1979	0.3920	0.1792	0.3603	0.5972	0.5972	0.3333	0.4286	0.4286
UL_H1_2002	2002	UL	UL_BEFORE	0.2000	0.3984	0.2709	0.5083	0.6660	0.6660	0.3333	0.4286	0.4286
UL_H2_2002	2002	UL	UL_BEFORE	0.1034	0.2464	0.2660	0.4850	0.6622	0.6622	0.6667	0.4286	0.4286
UL_H3_2002	2002	UL	UL_BEFORE	0.0973	0.2358	0.2712	0.5098	0.7396	0.7396	0.6667	0.4286	0.4286
UL_H4_2002	2002	UL	UL_BEFORE	0.1979	0.3920	0.2064	0.4448	0.5972	0.5972	0.6667	0.4286	0.4286
UL_H5_2002	2002	UL	UL_BEFORE	0.0980	0.2337	0.1953	0.4310	0.6060	0.6060	0.6667	0.4286	0.4286
UL_I1_2010	2010	UL	UL_REWET	0.1207	0.2727	0.1308	0.3452	0.3335	0.3335	0.3333	0.1429	0.1429
UL_I2_2010	2010	UL	UL_REWET	0.2000	0.3984	0.2712	0.5098	0.5333	0.5333	0.3333	0.2857	0.2857
UL_I3_2010	2010	UL	UL_REWET	0.1966	0.3878	0.1166	0.3220	0.2354	0.2354	0.3333	0.4286	0.4286
UL_I4_2010	2010	UL	UL_REWET	0.2000	0.3984	0.0953	0.2829	0.2283	0.2283	0.3333	0.4286	0.4286
UL_I5_2010	2010	UL	UL_REWET	0.1966	0.3878	0.1163	0.3206	0.2144	0.2144	0.3333	0.4286	0.4286
UL_J1_2010	2010	UL	UL_REWET	0.2000	0.3984	0.1166	0.3220	0.2354	0.2354	0.3333	0.4286	0.4286
UL_J2_2010	2010	UL	UL_REWET	0.1938	0.3794	0.0524	0.1872	0.4418	0.4418	0.3333	0.1429	0.1429
UL_J3_2010	2010	UL	UL_REWET	0.0938	0.2209	0.0885	0.2748	0.5439	0.5439	1.0000	0.2857	0.2857
UL_J4_2010	2010	UL	UL_REWET	0.0938	0.2209	0.0736	0.2380	0.4558	0.4558	0.3333	0.1429	0.1429
UL_J5_2010	2010	UL	UL_REWET									
UL_K1_2010	2010	UL	UL_REWET	0.1000	0.2399	0.0807	0.2183	0.2210	0.2210	0.3333	0.1429	0.1429
UL_K2_2010	2010	UL	UL_REWET	0.0966	0.2293	0.0736	0.2380	0.1883	0.1883	0.3333	0.2857	0.2857
UL_K3_2010	2010	UL	UL_REWET	0.0172	0.0499	0.0467	0.1281	0.1883	0.1883	0.3333	0.1429	0.1429
UL_K4_2010	2010	UL	UL_REWET	#NAME?	#NAME?	#NAME?	#NAME?	#NAME?	#NAME?	#NAME?	#NAME?	#NAME?
UL_K5_2010	2010	UL	UL_REWET									
UL_L1_2010	2010	UL	UL_REWET	0.0938	0.2209	0.1166	0.3220	0.2214	0.2214	0.3333	0.4286	0.4286
UL_L2_2010	2010	UL	UL_REWET	0.1931	0.3774	0.1001	0.2499	0.0456	0.0456	0.0000	0.4286	0.4286
UL_L3_2010	2010	UL	UL_REWET	0.1966	0.3878	0.0287	0.1188	0.2144	0.2144	0.3333	0.2857	0.2857
UL_L4_2010	2010	UL	UL_REWET									
UL_L5_2010	2010	UL	UL_REWET	#NAME?	#NAME?	#NAME?	#NAME?	#NAME?	#NAME?	#NAME?	#NAME?	#NAME?
UL_M1_2002	2002	UL	UL_BEFORE	0.1938	0.3794	0.1264	0.3751	0.2283	0.2283	0.3333	0.5714	0.5714
UL_M2_2002	2002	UL	UL_BEFORE	0.1966	0.3878	0.1163	0.3206	0.2283	0.2283	0.3333	0.4286	0.4286
UL_M3_2002	2002	UL	UL_BEFORE	0.0517	0.1390	0.0953	0.2829	0.2070	0.2070	0.3333	0.2857	0.2857
UL_M4_2002	2002	UL	UL_BEFORE	0.0483	0.1284	0.0287	0.1188	0.1718	0.1718	0.3333	0.2857	0.2857
UL_M5_2002	2002	UL	UL_BEFORE	0.0000	0.0000	0.0000	0.0000	0.0000	0.0000	0.0000	0.0000	0.0000
UL_N1_2002	2002	UL	UL_BEFORE	0.1966	0.3878	0.1166	0.3220	0.2214	0.2214	0.3333	0.4286	0.4286
UL_N2_2002	2002	UL	UL_BEFORE	0.1938	0.3794	0.0754	0.2469	0.4558	0.4558	0.3333	0.1429	0.1429
UL_N3_2002	2002	UL	UL_BEFORE	0.2000	0.3984	0.1181	0.3295	0.1648	0.1648	0.3333	0.4286	0.4286
UL_N4_2002	2002	UL	UL_BEFORE	0.0483	0.1284	0.1166	0.3220	0.2354	0.2354	0.3333	0.4286	0.4286
UL_N5_2002	2002	UL	UL_BEFORE	0.0483	0.1009	0.0375	0.1211	0.4330	0.4330	0.3333	0.2857	0.2857
UL_O1_2002	2002	UL	UL_BEFORE	0.0207	0.0606	0.1163	0.3206	0.2210	0.2210	0.0000	0.2857	0.2857
UL_O2_2002	2002	UL	UL_BEFORE	0.1938	0.3794	0.1166	0.3220	0.2354	0.2354	0.0000	0.4286	0.4286
UL_O3_2002	2002	UL	UL_BEFORE	0.1931	0.3774	0.1001	0.2499	0.2056	0.2056	0.0000	0.4286	0.4286
UL_O4_2002	2002	UL	UL_BEFORE	0.1938	0.3794	0.1181	0.3295	0.2283	0.2283	0.3333	0.4286	0.4286
UL_O5_2002	2002	UL	UL_BEFORE	0.1938	0.3794	0.0754	0.2469	0.2283	0.2283	0.3333	0.4286	0.4286
UL_P1_2002	2002	UL	UL_BEFORE	0.1000	0.2399	0.1163	0.3206	0.2070	0.2070	0.3333	0.2857	0.2857
UL_P2_2002	2002	UL	UL_BEFORE	0.0903	0.2144	0.1163	0.3206	0.1688	0.1688	0.0000	0.2857	0.2857
UL_P3_2002	2002	UL	UL_BEFORE	0.1034	0.2464	0.1114	0.2972	0.2210	0.2210	0.3333	0.2857	0.2857
UL_P4_2002	2002	UL	UL_BEFORE	0.0903	0.2144	0.1166	0.3220	0.2354	0.2354	0.0000	0.4286	0.4286
UL_P5_2002	2002	UL	UL_BEFORE	0.0097	0.0281	0.0003	0.0015	0.2214	0.2214	0.0000	0.1429	0.1429
UZ_101	1982	UZ	UZ_BEFORE	0.1290	0.2986	0.1829	0.5113	0.7210	0.7210	0.6667	0.4286	0.4286
UZ_102	1982	UZ	UZ_BEFORE	0.1290	0.2986	0.1753	0.4595	0.7646	0.7646	0.6667	0.4286	0.4286
UZ_103	1982	UZ	UZ_BEFORE	0.1290	0.2986	0.1829	0.5113	0.7646	0.7646	0.6667	0.4286	0.4286
UZ_104	1982	UZ	UZ_BEFORE	0.1172	0.2622	0.2225	0.5600	0.7396	0.7396	0.6667	0.2857	0.2857
UZ_105	1982	UZ	UZ_BEFORE	0.1393	0.2987	0.2397	0.5791	0.7746	0.7746	0.6667	0.5714	0.5714
UZ_106	1982	UZ	UZ_BEFORE	0.1317	0.3076	0.2225	0.5600	0.7746	0.7746	0.6667	0.5714	0.5714
UZ_107	1982	UZ	UZ_BEFORE	0.1538	0.3441	0.2225	0.5600	0.7646	0.7646	1.0000	0.5714	0.5714
UZ_108	1982	UZ	UZ_BEFORE	0.1442	0.3136	0.2225	0.5600	0.7746	0.7746	0.6667	0.5714	0.5714
UZ_109	1982	UZ	UZ_BEFORE	0.2076	0.4227	0.1829	0.5113	0.8515	0.8515	0.6667	0.4286	0.4286
UZ_110	1982	UZ	UZ_BEFORE	0.1538	0.3441	0.2619	0.6018	0.7364	0.7364	0.6667	0.4286	0.4286

UZ_111	1982	UZ	UZ_BEFORE	0.1980	0.3922	0.2225	0.5600	0.7396	0.7396	0.6667	0.4286	0.4286
UZ_112	1982	UZ	UZ_BEFORE	0.1442	0.3136	0.2225	0.5600	0.7704	0.7704	0.6667	0.4286	0.4286
UZ_113	1982	UZ	UZ_BEFORE	0.1186	0.2406	0.2225	0.5600	0.7396	0.7396	0.6667	0.5714	0.5714
UZ_114	1982	UZ	UZ_BEFORE	0.1442	0.3136	0.2225	0.5600	0.7746	0.7746	0.6667	0.4286	0.4286
UZ_115	1982	UZ	UZ_BEFORE	0.2076	0.4227	0.2078	0.5001	0.8233	0.8233	0.6667	0.4286	0.4286
UZ_116	1982	UZ	UZ_BEFORE	0.2076	0.4227	0.2225	0.5600	0.8265	0.8265	0.6667	0.4286	0.4286
UZ_117	1982	UZ	UZ_BEFORE	0.1317	0.3076	0.2619	0.6018	0.7746	0.7746	0.6667	0.2857	0.2857
UZ_118	1982	UZ	UZ_BEFORE	0.1980	0.3922	0.2225	0.5600	0.7608	0.7608	0.6667	0.4286	0.4286
UZ_119	1982	UZ	UZ_BEFORE	0.2076	0.4227	0.1895	0.5200	0.7364	0.7364	1.0000	0.4286	0.4286
UZ_120	1982	UZ	UZ_BEFORE	0.1566	0.3438	0.2062	0.5410	0.8080	0.8080	0.6667	0.4286	0.4286
UZ_121	1982	UZ	UZ_BEFORE	0.2076	0.4227	0.1727	0.4437	0.8233	0.8233	0.6667	0.4286	0.4286
UZ_122	1982	UZ	UZ_BEFORE	0.2076	0.4227	0.1829	0.5113	0.8477	0.8477	0.6667	0.4286	0.4286
UZ_123	1982	UZ	UZ_BEFORE	0.1980	0.3922	0.2225	0.5600	0.7746	0.7746	0.6667	0.4286	0.4286
UZ_124	1982	UZ	UZ_BEFORE	0.2000	0.3984	0.1715	0.3554	0.6442	0.6442	0.6667	0.4286	0.4286
UZ_125	1982	UZ	UZ_BEFORE	0.2000	0.3984	0.1603	0.4267	0.6442	0.6442	0.6667	0.5714	0.5714
UZ_126	1982	UZ	UZ_BEFORE	0.2076	0.4227	0.1482	0.3257	0.8477	0.8477	0.6667	0.4286	0.4286
UZ_127	1982	UZ	UZ_BEFORE	0.1980	0.3922	0.1829	0.5113	0.6363	0.6363	0.6667	0.4286	0.4286
UZ_128	1982	UZ	UZ_BEFORE	0.2000	0.3984	0.1829	0.5113	0.6442	0.6442	0.6667	0.4286	0.4286
UZ_129	1982	UZ	UZ_BEFORE	0.2000	0.3984	0.1358	0.3087	0.7232	0.7232	0.6667	0.5714	0.5714
UZ_130	1982	UZ	UZ_BEFORE	0.2062	0.4182	0.2160	0.4049	0.7826	0.7826	0.6667	0.5714	0.5714
UZ_131	1982	UZ	UZ_BEFORE	0.2062	0.4182	0.2507	0.5905	0.7883	0.7883	0.6667	0.4286	0.4286
UZ_132	1982	UZ	UZ_BEFORE	0.1980	0.3922	0.2225	0.5600	0.7396	0.7396	0.6667	0.4286	0.4286
UZ_133	1982	UZ	UZ_BEFORE	0.1256	0.2830	0.2225	0.5600	0.7396	0.7396	0.6667	0.2857	0.2857
UZ_134	1982	UZ	UZ_BEFORE	0.2062	0.4182	0.2275	0.4547	0.8136	0.8136	0.6667	0.4286	0.4286
UZ_135	1982	UZ	UZ_BEFORE	0.2048	0.4138	0.1877	0.3745	0.8265	0.8265	0.6667	0.4286	0.4286
UZ_136	1982	UZ	UZ_BEFORE	0.2000	0.3984	0.1482	0.3257	0.8477	0.8477	0.6667	0.4286	0.4286
UZ_137	1982	UZ	UZ_BEFORE	0.2000	0.3984	0.1548	0.3344	0.8477	0.8477	0.6667	0.4286	0.4286
UZ_138	1982	UZ	UZ_BEFORE	0.1980	0.3922	0.1862	0.3684	0.7645	0.7645	0.6667	0.4286	0.4286
UZ_139	1982	UZ	UZ_BEFORE	0.1980	0.3922	0.1579	0.3670	0.5998	0.5998	0.6667	0.4286	0.4286
UZ_140	1982	UZ	UZ_BEFORE	0.1980	0.3922	0.1579	0.3670	0.7608	0.7608	0.6667	0.4286	0.4286
UZ_141	1982	UZ	UZ_BEFORE	0.1980	0.3922	0.1579	0.3670	0.4998	0.4998	0.6667	0.4286	0.4286
UZ_142	1982	UZ	UZ_BEFORE	0.1442	0.3136	0.1895	0.5200	0.6881	0.6881	0.6667	0.4286	0.4286
UZ_143	1982	UZ	UZ_BEFORE	0.1980	0.3922	0.1548	0.3344	0.8572	0.8572	0.6667	0.4286	0.4286
UZ_144	1982	UZ	UZ_BEFORE	0.1980	0.3922	0.1645	0.3757	0.7807	0.7807	0.6667	0.5714	0.5714
UZ_145	1982	UZ	UZ_BEFORE	0.1442	0.3136	0.1645	0.3757	0.7645	0.7645	0.6667	0.4286	0.4286
UZ_146	1982	UZ	UZ_BEFORE	0.1221	0.2771	0.1904	0.4077	0.7608	0.7608	0.6667	0.4286	0.4286
UZ_147	1982	UZ	UZ_BEFORE	0.2601	0.4692	0.2142	0.5505	0.6881	0.6881	0.6667	0.4286	0.4286
UZ_148	1982	UZ	UZ_BEFORE	0.2062	0.4182	0.2144	0.3989	0.6957	0.6957	0.6667	0.4286	0.4286
UZ_149	1982	UZ	UZ_BEFORE	0.1980	0.3922	0.1579	0.3670	0.7646	0.7646	0.6667	0.4286	0.4286
UZ_150	1982	UZ	UZ_BEFORE	0.1822	0.3705	0.1579	0.3670	0.6881	0.6881	0.6667	0.4286	0.4286
UZ_151	1982	UZ	UZ_BEFORE	0.1442	0.3136	0.2225	0.5600	0.7396	0.7396	0.6667	0.4286	0.4286
UZ_152	1982	UZ	UZ_BEFORE	0.2648	0.4841	0.2619	0.6018	0.7746	0.7746	1.0000	0.4286	0.4286
UZ_153	1982	UZ	UZ_BEFORE	0.1256	0.2830	0.2368	0.4575	0.7396	0.7396	0.6667	0.4286	0.4286
UZ_154	1982	UZ	UZ_BEFORE	0.1221	0.2771	0.1974	0.4158	0.7396	0.7396	0.6667	0.4286	0.4286
UZ_155	1982	UZ	UZ_BEFORE	0.1538	0.3441	0.2368	0.4575	0.7646	0.7646	0.6667	0.4286	0.4286
UZ_401	2013	UZ	UZ_REWET	0.1254	0.2966	0.1504	0.2814	0.6137	0.6137	0.6667	0.4286	0.4286
UZ_402	2013	UZ	UZ_REWET	0.0946	0.2272	0.1195	0.2570	0.6118	0.6118	0.3333	0.5714	0.5714
UZ_403	2013	UZ	UZ_REWET	0.2076	0.4227	0.1467	0.3197	0.7364	0.7364	0.6667	0.4286	0.4286
UZ_404	2013	UZ	UZ_REWET	0.2076	0.4227	0.1227	0.2857	0.7364	0.7364	0.6667	0.4286	0.4286
UZ_405	2013	UZ	UZ_REWET	0.1261	0.2989	0.1450	0.4160	0.7364	0.7364	0.6667	0.2857	0.2857
UZ_406	2013	UZ	UZ_REWET	0.1254	0.2966	0.1277	0.3236	0.7364	0.7364	0.3333	0.2857	0.2857
UZ_407	2013	UZ	UZ_REWET	0.1317	0.3076	0.1273	0.2849	0.7364	0.7364	0.6667	0.4286	0.4286
UZ_408	2013	UZ	UZ_REWET	0.2076	0.4227	0.1792	0.3603	0.7364	0.7364	0.6667	0.4286	0.4286
UZ_409	2013	UZ	UZ_REWET	0.2076	0.4227	0.1164	0.2762	0.7364	0.7364	0.6667	0.4286	0.4286
UZ_410	2013	UZ	UZ_REWET	0.2083	0.4250	0.7474	0.7098	0.8738	0.8738	0.6667	0.4286	0.4286
UZ_411	2013	UZ	UZ_REWET	0.2083	0.4250	0.7474	0.7098	0.8738	0.8738	0.3333	0.4286	0.4286
UZ_412	2013	UZ	UZ_REWET	0.2083	0.4250	0.7489	0.7159	0.9608	0.9608	0.6667	0.4286	0.4286
UZ_413	2013	UZ	UZ_REWET	0.1254	0.2966	0.1132	0.2638	0.7746	0.7746	0.6667	0.2857	0.2857
UZ_414	2013	UZ	UZ_REWET	0.1317	0.3076	0.2154	0.5519	0.7364	0.7364	0.6667	0.4286	0.4286
UZ_415	2013	UZ	UZ_REWET	0.2076	0.4227	0.1579	0.3670	0.7364	0.7364	0.6667	0.4286	0.4286
UZ_416	2013	UZ	UZ_REWET	0.2076	0.4227	0.1645	0.3757	0.7364	0.7364	0.6667	0.4286	0.4286
UZ_417	2013	UZ	UZ_REWET	0.2076	0.4227	0.1164	0.2762	0.7364	0.7364	0.3333	0.4286	0.4286
UZ_418	2013	UZ	UZ_REWET	0.2076	0.4227	0.1164	0.2762	0.7364	0.7364	0.6667	0.4286	0.4286
UZ_419	2013	UZ	UZ_REWET	0.2100	0.4307	0.1842	0.3662	0.7364	0.7364	0.6667	0.4286	0.4286
UZ_420	2013	UZ	UZ_REWET	0.1928	0.3764	0.1239	0.2913	0.8477	0.8477	0.6667	0.5714	0.5714
UZ_421	2013	UZ	UZ_REWET	0.2000	0.3984	0.1603	0.4267	0.6442	0.6442	0.6667	0.5714	0.5714
UZ_422	2013	UZ	UZ_REWET	0.1980	0.3922	0.1200	0.3632	0.5867	0.5867	0.6667	0.5714	0.5714
UZ_423	2013	UZ	UZ_REWET	0.1980	0.3922	0.0955	0.2452	0.6868	0.6868	0.6667	0.4286	0.4286
UZ_424	2013	UZ	UZ_REWET	0.1980	0.3922	0.1200	0.3632	0.5867	0.5867	0.6667	0.5714	0.5714
UZ_425	2013	UZ	UZ_REWET	0.1980	0.3922	0.1200	0.3632	0.6868	0.6868	0.6667	0.5714	0.5714
UZ_426	2013	UZ	UZ_REWET	0.2000	0.3984	0.1603	0.4267	0.8215	0.8215	0.6667	0.5714	0.5714
UZ_427	2013	UZ	UZ_REWET	0.1980	0.3922	0.0955	0.2452	0.5867	0.5867	0.6667	0.5714	0.5714
UZ_428	2013	UZ	UZ_REWET	0.1221	0.2771	0.0955	0.2452	0.7232	0.7232	0.6667	0.4286	0.4286
UZ_429	2013	UZ	UZ_REWET	0.1980	0.3922	0.0955	0.2452	0.6868	0.6868	0.6667	0.5714	0.5714
UZ_430	2013	UZ	UZ_REWET	0.1980	0.3922	0.1200	0.3632	0.6868	0.6868	0.6667	0.5714	0.5714
UZ_431	2013	UZ	UZ_REWET	0.1928	0.3764	0.1092	0.3439	0.5867	0.5867	0.3333	0.5714	0.5714
UZ_432	2013	UZ	UZ_REWET	0.2000	0.3984	0.1358	0.3087	0.7232	0.7232	0.6667	0.5714	0.5714

UZ_433	2013	UZ	UZ_REWET	0.2076	0.4227	0.1277	0.3236	0.7608	0.7608	0.6667	0.4286	0.4286
UZ_434	2013	UZ	UZ_REWET	0.1980	0.3922	0.0955	0.2452	0.7232	0.7232	0.3333	0.5714	0.5714
UZ_435	2013	UZ	UZ_REWET	0.1980	0.3922	0.0955	0.2452	0.6868	0.6868	0.6667	0.5714	0.5714
UZ_436	2013	UZ	UZ_REWET	0.1980	0.3922	0.0939	0.2392	0.7345	0.7345	0.6667	0.5714	0.5714
UZ_437	2013	UZ	UZ_REWET	0.1980	0.3922	0.0955	0.2452	0.8215	0.8215	0.6667	0.4286	0.4286
UZ_438	2013	UZ	UZ_REWET	0.2000	0.3984	0.1358	0.3087	0.7232	0.7232	0.6667	0.5714	0.5714
UZ_439	2013	UZ	UZ_REWET	0.1980	0.3922	0.0955	0.2452	0.6868	0.6868	0.6667	0.4286	0.4286
UZ_440	2013	UZ	UZ_REWET	0.1980	0.3922	0.0955	0.2452	0.8215	0.8215	0.6667	0.5714	0.5714
UZ_441	2013	UZ	UZ_REWET	0.1980	0.3922	0.0955	0.2452	0.8215	0.8215	0.6667	0.4286	0.4286
UZ_442	2013	UZ	UZ_REWET	0.1980	0.3922	0.1807	0.3664	0.8215	0.8215	0.6667	0.4286	0.4286
UZ_443	2013	UZ	UZ_REWET	0.1980	0.3922	0.1807	0.3664	0.8215	0.8215	0.6667	0.4286	0.4286
UZ_444	2013	UZ	UZ_REWET	0.2062	0.4182	0.2160	0.4049	0.8215	0.8215	0.3333	0.5714	0.5714
UZ_445	2013	UZ	UZ_REWET	0.2062	0.4182	0.2160	0.4049	0.8215	0.8215	0.6667	0.5714	0.5714
UZ_446	2013	UZ	UZ_REWET	0.2100	0.4307	0.1792	0.3603	0.7608	0.7608	0.6667	0.4286	0.4286
UZ_447	2013	UZ	UZ_REWET	0.2076	0.4227	0.1842	0.3662	0.7364	0.7364	0.6667	0.4286	0.4286
UZ_448	2013	UZ	UZ_REWET	0.1980	0.3922	0.1239	0.2913	0.8477	0.8477	0.6667	0.4286	0.4286
UZ_449	2013	UZ	UZ_REWET	0.0852	0.2200	0.2160	0.4049	0.8477	0.8477	0.3333	0.4286	0.4286
UZ_450	2013	UZ	UZ_REWET	0.2076	0.4227	0.2160	0.4049	0.8233	0.8233	0.6667	0.4286	0.4286
UZ_451	2013	UZ	UZ_REWET	0.2076	0.4227	0.2188	0.4165	0.7746	0.7746	0.6667	0.4286	0.4286
UZ_452	2013	UZ	UZ_REWET	0.1254	0.2966	0.1596	0.2924	0.7364	0.7364	0.6667	0.4286	0.4286
UZ_453	2013	UZ	UZ_REWET	0.2062	0.4182	0.2188	0.4165	0.8477	0.8477	0.6667	0.4286	0.4286
UZ_454	2013	UZ	UZ_REWET	0.1066	0.2657	0.2160	0.4049	0.8477	0.8477	0.6667	0.4286	0.4286
UZ_455	2013	UZ	UZ_REWET	0.1254	0.2966	0.1836	0.3780	0.7608	0.7608	0.6667	0.4286	0.4286
VK_A10_1	2008	VK	VK_TSR	0.1109	0.2574	0.1436	0.3532	0.4852	0.4852	0.6667	0.2857	0.2857
VK_A10_2	2008	VK	VK_TSR	0.0994	0.2363	0.2030	0.5370	0.7459	0.7459	0.6667	0.2857	0.2857
VK_A10_3	2008	VK	VK_TSR	0.0877	0.2138	0.1271	0.2518	0.4852	0.4852	0.3333	0.2857	0.2857
VK_A10_4	2008	VK	VK_TSR	0.1109	0.2574	0.1829	0.5113	0.4953	0.4953	0.6667	0.2857	0.2857
VK_A10_5	2008	VK	VK_TSR	0.1221	0.2771	0.1668	0.4889	0.6353	0.6353	0.6667	0.4286	0.4286
VK_A5_1	2008	VK	VK_TSR	0.0857	0.1958	0.1829	0.5113	0.7118	0.7118	0.3333	0.2857	0.2857
VK_A5_2	2008	VK	VK_TSR	0.0994	0.2363	0.1829	0.5113	0.7118	0.7118	0.6667	0.2857	0.2857
VK_A5_3	2008	VK	VK_TSR	0.1221	0.2771	0.1847	0.5138	0.7118	0.7118	0.6667	0.4286	0.4286
VK_A5_4	2008	VK	VK_TSR	0.1221	0.2771	0.1753	0.4595	0.7118	0.7118	0.3333	0.4286	0.4286
VK_A5_5	2008	VK	VK_TSR	0.1083	0.2528	0.1663	0.3842	0.7459	0.7459	0.6667	0.2857	0.2857
VK_A85_1	2008	VK	VK_TSR	0.0938	0.1969	0.2291	0.4564	0.5706	0.5706	0.6667	0.4286	0.4286
VK_AM_1	2008	VK	VK_REWET	0.1172	0.2622	0.1818	0.4681	0.4259	0.4259	0.6667	0.4286	0.4286
VK_AM_2	2008	VK	VK_REWET	0.1172	0.2622	0.1829	0.5113	0.4318	0.4318	0.6667	0.5714	0.5714
VK_AM_3	2008	VK	VK_REWET	0.0804	0.1812	0.1829	0.5113	0.7646	0.7646	0.6667	0.5714	0.5714
VK_AM_4	2008	VK	VK_REWET	0.0756	0.1895	0.1597	0.3756	0.7118	0.7118	0.6667	0.2857	0.2857
VK_AM_5	2008	VK	VK_REWET	0.0828	0.1990	0.1597	0.3756	0.4318	0.4318	0.3333	0.4286	0.4286
VK_BM_1	2008	VK	VK_REF	0.1162	0.2802	0.2064	0.4448	0.7608	0.7608	1.0000	0.4286	0.4286
VK_BM_10	2008	VK	VK_REF	0.0989	0.2208	0.1697	0.4005	0.5307	0.5307	1.0000	0.2857	0.2857
VK_BM_11	2008	VK	VK_REF	0.1118	0.2590	0.2064	0.4448	0.7080	0.7080	0.6667	0.4286	0.4286
VK_BM_12	2008	VK	VK_REF	0.1090	0.2668	0.2262	0.5643	0.7080	0.7080	1.0000	0.4286	0.4286
VK_BM_2	2008	VK	VK_REF	0.1162	0.2802	0.2064	0.4448	0.7080	0.7080	0.6667	0.4286	0.4286
VK_BM_3	2008	VK	VK_REF	0.1162	0.2802	0.2262	0.5643	0.7080	0.7080	0.6667	0.4286	0.4286
VK_BM_4	2008	VK	VK_REF	0.1066	0.2497	0.2064	0.4448	0.7080	0.7080	0.6667	0.4286	0.4286
VK_BM_5	2008	VK	VK_REF	0.1090	0.2668	0.2262	0.5643	0.7080	0.7080	0.6667	0.4286	0.4286
VK_BM_6	2008	VK	VK_REF	0.0532	0.1408	0.2064	0.4448	0.7080	0.7080	0.6667	0.4286	0.4286
VK_BM_7	2008	VK	VK_REF	0.0532	0.1408	0.2064	0.4448	0.7080	0.7080	0.6667	0.4286	0.4286
VK_BM_8	2008	VK	VK_REF	0.1066	0.2497	0.2262	0.5643	0.7080	0.7080	0.6667	0.4286	0.4286
VK_BM_9	2008	VK	VK_REF	0.1214	0.2895	0.2064	0.4448	0.6836	0.6836	0.6667	0.4286	0.4286
VK_E5_1	2008	VK	VK_TSR	0.0994	0.2363	0.1895	0.5200	0.5341	0.5341	1.0000	0.2857	0.2857
VK_E5_2	2008	VK	VK_TSR	0.0766	0.1707	0.1895	0.5200	0.7118	0.7118	0.0000	0.2857	0.2857
VK_E5_3	2008	VK	VK_TSR	0.0994	0.2363	0.1895	0.5200	0.7118	0.7118	0.6667	0.2857	0.2857
VK_E5_4	2008	VK	VK_TSR	0.0865	0.1981	0.2469	0.5866	0.6161	0.6161	0.3333	0.2857	0.2857
VK_E5_5	2008	VK	VK_TSR	0.0945	0.2214	0.1895	0.5200	0.5341	0.5341	1.0000	0.4286	0.4286
VK_E85_1	2008	VK	VK_TSR	0.0828	0.1990	0.1271	0.2518	0.7459	0.7459	0.6667	0.2857	0.2857
VK_EM_1	2008	VK	VK_REWET	0.0945	0.2214	0.1895	0.5200	0.7459	0.7459	0.3333	0.1429	0.1429
VK_EM_2	2008	VK	VK_REWET	0.0945	0.2214	0.1829	0.5113	0.7459	0.7459	0.3333	0.2857	0.2857
VK_EM_3	2008	VK	VK_REWET	0.0945	0.2214	0.1829	0.5113	0.7459	0.7459	0.3333	0.2857	0.2857
VK_EM_4	2008	VK	VK_REWET	0.1221	0.2771	0.1895	0.5200	0.7459	0.7459	0.6667	0.5714	0.5714
VK_EM_5	2008	VK	VK_REWET	0.0945	0.2214	0.1829	0.5113	0.7459	0.7459	0.3333	0.2857	0.2857
VK_G10_1	2008	VK	VK_TSR	0.0921	0.2143	0.2079	0.5431	0.7459	0.7459	0.6667	0.2857	0.2857
VK_G10_2	2008	VK	VK_TSR	0.0945	0.2214	0.1829	0.5113	0.7459	0.7459	0.6667	0.2857	0.2857
VK_G10_3	2008	VK	VK_TSR	0.0959	0.2256	0.2079	0.5431	0.7646	0.7646	1.0000	0.4286	0.4286
VK_G10_4	2008	VK	VK_TSR	0.0945	0.2214	0.1829	0.5113	0.7459	0.7459	0.6667	0.2857	0.2857
VK_G10_5	2008	VK	VK_TSR	0.0792	0.1896	0.1616	0.3780	0.7459	0.7459	0.6667	0.2857	0.2857
VK_G5_1	2008	VK	VK_TSR	0.1650	0.3379	0.2079	0.5431	0.7459	0.7459	0.3333	0.5714	0.5714
VK_G5_2	2008	VK	VK_TSR	0.0945	0.2214	0.1847	0.5138	0.7459	0.7459	0.6667	0.2857	0.2857
VK_G5_3	2008	VK	VK_TSR	0.1024	0.2361	0.1616	0.3780	0.7459	0.7459	0.6667	0.5714	0.5714
VK_G5_4	2008	VK	VK_TSR	0.0945	0.2214	0.1847	0.5138	0.7646	0.7646	1.0000	0.5714	0.5714
VK_G5_5	2008	VK	VK_TSR	0.0945	0.2214	0.1829	0.5113	0.7459	0.7459	0.6667	0.2857	0.2857
VK_G85_1	2008	VK	VK_TSR	0.0945	0.2214	0.1847	0.5138	0.7459	0.7459	0.6667	0.4286	0.4286
VK_GM_1	2008	VK	VK_BEFORE	0.0610	0.1387	0.1597	0.3756	0.7459	0.7459	0.3333	0.4286	0.4286
VK_GM_2	2008	VK	VK_BEFORE	0.0676	0.1565	0.0727	0.1176	0.7459	0.7459	0.0000	0.4286	0.4286
VK_GM_3	2008	VK	VK_BEFORE	0.0830	0.2009	0.1597	0.3756	0.7459	0.7459	1.0000	0.4286	0.4286
VK_GM_4	2008	VK	VK_BEFORE	0.0817	0.1968	0.0625	0.0982	0.7459	0.7459	0.3333	0.4286	0.4286

VK_GM_5	2008	VK	VK_BEFORE	0.1024	0.2361	0.1597	0.3756	0.7459	0.7459	0.3333	0.4286	0.4286
VP_ACM_99	1999	VPA	VPA_BEFOR	0.0000	0.0000	0.0000	0.0000	0.0000	0.0000	0.0000	0.0000	0.0000
VP_AP_02	2002	VPA	VPA_TSR	0.0945	0.2214	0.1668	0.4889	0.3655	0.3655	0.3333	0.1429	0.1429
VP_BCM_99	1999	VPB	VPB_BEFOR	0.1172	0.2622	0.1221	0.2367	0.5139	0.5139	0.6667	0.5714	0.5714
VP_BP_02	2002	VPB	VPB_TSR	0.1092	0.2389	0.1362	0.3201	0.5380	0.5380	0.6667	0.4286	0.4286
VP_CCM_99	1999	VPC	VPC_BEFOR	0.1076	0.2623	0.2507	0.5905	0.6957	0.6957	0.3333	0.2857	0.2857
VP_CP_02	2002	VPC	VPC_TSR	0.1303	0.3031	0.2430	0.5387	0.6957	0.6957	1.0000	0.4286	0.4286
VP_DCM_99	1999	VPD	VPD_BEFOR	0.1029	0.2279	0.2012	0.4200	0.6132	0.6132	0.6667	0.2857	0.2857
VP_DP_02	2002	VPD	VPD_TSR	0.1303	0.3031	0.2326	0.4800	0.7746	0.7746	0.6667	0.2857	0.2857
VP_FCM_99	1999	VPF	VPF_BEFORI	0.1041	0.2577	0.1899	0.4325	0.7364	0.7364	1.0000	0.4286	0.4286
VP_FP_02	2002	VPF	VPF_TSR	0.1317	0.3076	0.1346	0.3573	0.7364	0.7364	0.6667	0.4286	0.4286
VP_GCM_99	1999	VPG	VPG_BEFOR	0.0756	0.1720	0.1060	0.2143	0.3916	0.3916	0.3333	0.4286	0.4286
VP_GP_02	2002	VPG	VPG_TSR	0.1028	0.2532	0.1899	0.3159	0.6957	0.6957	0.6667	0.4286	0.4286
ZB_B3_1992	1992	ZB	ZB_BEFORE	0.1092	0.2389	0.2148	0.5082	0.6422	0.6422	0.6667	0.2857	0.2857
ZB_B3_1998	1998	ZB	ZB_REWET	0.1092	0.2389	0.2148	0.5082	0.6422	0.6422	0.6667	0.2857	0.2857
ZB_B4_1992	1992	ZB	ZB_BEFORE	0.1172	0.2622	0.2393	0.5348	0.6161	0.6161	0.6667	0.2857	0.2857
ZB_B4_1998	1998	ZB	ZB_REWET	0.1172	0.2622	0.2163	0.4178	0.6161	0.6161	0.6667	0.5714	0.5714
ZB_B5_1992	1992	ZB	ZB_BEFORE	0.1092	0.2389	0.2201	0.5141	0.6422	0.6422	1.0000	0.4286	0.4286
ZB_B5_1998	1998	ZB	ZB_REWET	0.1092	0.2389	0.2148	0.5082	0.6422	0.6422	0.6667	0.2857	0.2857
ZB_B6_1992	1992	ZB	ZB_BEFORE	0.1092	0.2389	0.2061	0.3423	0.6672	0.6672	0.6667	0.4286	0.4286
ZB_B6_1998	1998	ZB	ZB_REWET	0.1092	0.2389	0.1759	0.3726	0.6672	0.6672	0.6667	0.4286	0.4286
ZB_B7_1992	1992	ZB	ZB_BEFORE	0.1207	0.2681	0.2148	0.5082	0.6672	0.6672	0.6667	0.4286	0.4286
ZB_B7_1998	1998	ZB	ZB_REWET	0.1570	0.3145	0.2148	0.5082	0.6672	0.6672	0.6667	0.2857	0.2857
ZB_B8_1992	1992	ZB	ZB_BEFORE	0.1221	0.2769	0.2078	0.5001	0.4318	0.4318	0.3333	0.4286	0.4286
ZB_B8_1998	1998	ZB	ZB_REWET	0.1221	0.2769	0.2078	0.5001	0.4725	0.4725	0.6667	0.4286	0.4286
ZB_C1_1988	1988	ZB	ZB_BEFORE	0.1172	0.2622	0.1837	0.4705	0.6672	0.6672	1.0000	0.4286	0.4286
ZB_C1_1999	1999	ZB	ZB_REWET	0.1172	0.2622	0.2163	0.4178	0.6672	0.6672	0.6667	0.5714	0.5714
ZB_C2_1988	1988	ZB	ZB_BEFORE	0.1207	0.2681	0.1837	0.4705	0.4725	0.4725	1.0000	0.2857	0.2857
ZB_C2_2006	2006	ZB	ZB_REWET	0.1255	0.2828	0.2163	0.4178	0.6672	0.6672	0.6667	0.4286	0.4286
ZB_C6_1992	1992	ZB	ZB_BEFORE	0.1172	0.2622	0.2163	0.4178	0.6262	0.6262	0.6667	0.4286	0.4286
ZB_C6_1999	1999	ZB	ZB_REWET	0.1221	0.2769	0.2163	0.4178	0.6262	0.6262	0.6667	0.4286	0.4286
ZB_C9_1988	1988	ZB	ZB_BEFORE	0.0966	0.2041	0.1663	0.3842	0.6672	0.6672	0.6667	0.4286	0.4286
ZB_C9_2006	2006	ZB	ZB_REWET	0.1172	0.2622	0.2313	0.4330	0.6422	0.6422	0.6667	0.4286	0.4286

6 w	6 w.s	7 nit	8 pho	14 sm	14 sm.s	33 st_c	34 st_s	35 st_r
0.5000	0.5000	0.5362	0.4184	0.0325	0.3879	1.0000	1.0000	0.3333
0.5000	0.5000	0.4792	0.4184	0.0123	0.2415	0.5000	0.6667	0.3333
0.4000	0.4000	0.2897	0.3734	0.0144	0.2553	0.1667	0.5000	0.5000
0.4000	0.4000	0.3755	0.2480	0.0061	0.1575	1.0000	1.0000	0.3333
0.4000	0.4000	0.5295	0.4470	0.0123	0.2415	1.0000	1.0000	0.3333
0.5000	0.5000	0.4792	0.3773	0.0123	0.2415	0.1667	0.5000	0.5000
0.5000	0.5000	0.5295	0.4381	0.0123	0.2415	1.0000	1.0000	0.3333
0.5000	0.5000	0.3997	0.3773	0.0325	0.3879	1.0000	1.0000	0.3333
0.5000	0.5000	0.2256	0.1989	0.0324	0.3843	1.0000	1.0000	0.3333
0.3000	0.3000	0.5362	0.4184	0.0123	0.2415	1.0000	1.0000	0.3333
0.3000	0.3000	0.3158	0.3040	0.0123	0.2415	1.0000	1.0000	0.3333
0.2000	0.2000	0.5295	0.4381	0.0325	0.3879	0.5000	0.6667	0.3333
0.6000	0.6000	0.4792	0.4184	0.0127	0.2468	1.0000	1.0000	0.3333
0.5000	0.5000	0.3313	0.4734	0.0106	0.2216	1.0000	1.0000	0.3333
0.3000	0.3000	0.2267	0.2245	0.0106	0.2224	1.0000	1.0000	0.3333
0.3000	0.3000	0.4792	0.4184	0.0146	0.2660	0.5000	0.6667	0.3333
0.7000	0.7000	0.2087	0.3599	0.0127	0.2438	1.0000	1.0000	0.5000
0.5000	0.5000	0.1650	0.3455	0.0123	0.2415	1.0000	1.0000	0.3333
0.5000	0.5000	0.2676	0.4572	0.0073	0.1768	1.0000	1.0000	0.3333
0.3000	0.3000	0.5362	0.4381	0.0762	0.5325	1.0000	1.0000	0.3333
0.3000	0.3000	0.5362	0.4381	0.0762	0.5325	1.0000	1.0000	0.3333
0.7000	0.7000	0.2256	0.3084	0.0122	0.2407	1.0000	1.0000	0.3333
0.5000	0.5000	0.2256	0.2165	0.0106	0.2224	1.0000	1.0000	0.3333
0.3000	0.3000	0.4792	0.3773	0.0127	0.2468	1.0000	1.0000	0.5000
0.5000	0.5000	0.4792	0.5343	0.0325	0.3879	1.0000	1.0000	0.5000
0.5000	0.5000	0.2897	0.1173	0.0073	0.1776	1.0000	1.0000	0.3333
0.4000	0.4000	0.5295	0.4381	0.0123	0.2415	1.0000	1.0000	0.3333
0.6000	0.6000	0.5295	0.4381	0.0123	0.2415	1.0000	1.0000	0.3333
0.6000	0.6000	0.5295	0.4381	0.0146	0.2660	1.0000	1.0000	0.3333
0.5000	0.5000	0.4387	0.3652	0.0146	0.2660	1.0000	1.0000	0.3333
0.5000	0.5000	0.2897	0.3332	0.0120	0.2308	0.1667	0.1667	0.3333
0.6000	0.6000	0.5028	0.4184	0.0127	0.2468	1.0000	1.0000	0.3333
0.5000	0.5000	0.5994	0.4381	0.0146	0.2660	0.6667	0.5000	0.3333
0.4000	0.4000	0.3688	0.2677	0.0122	0.2407	0.6667	0.5000	0.3333
0.6000	0.6000	0.3158	0.3164	0.0123	0.2415	1.0000	1.0000	0.3333
0.6000	0.6000	0.4792	0.4184	0.0325	0.3879	0.6667	0.5000	0.5000
0.5000	0.5000	0.2029	0.3414	0.0123	0.2415	1.0000	1.0000	0.3333
0.6000	0.6000	0.4792	0.4470	0.0127	0.2468	1.0000	1.0000	0.3333
0.6000	0.6000	0.4083	0.4184	0.0123	0.2415	0.6667	0.5000	0.5000
0.3000	0.3000	0.2003	0.3175	0.0325	0.3879	1.0000	1.0000	0.3333
0.4000	0.4000	0.0936	0.3414	0.0070	0.1721	1.0000	1.0000	0.3333
0.5000	0.5000	0.1483	0.2084	0.0055	0.1461	1.0000	1.0000	0.3333
0.4000	0.4000	0.4792	0.4184	0.0363	0.4063	0.5000	0.6667	0.3333
0.4000	0.4000	0.2003	0.3164	0.0325	0.3879	0.5000	0.5000	0.0000
0.5000	0.5000	0.3158	0.3164	0.0325	0.3879	0.5000	0.6667	0.3333
0.5000	0.5000	0.5455	0.4184	0.0325	0.3879	0.1667	0.5000	0.5000
0.3000	0.3000	0.3313	0.3576	0.0127	0.2468	1.0000	1.0000	0.5000
0.4000	0.4000	0.5259	0.4184	0.0220	0.3262	1.0000	1.0000	0.3333
0.6000	0.6000	0.3158	0.3164	0.0325	0.3879	0.5000	0.6667	0.3333
0.5000	0.5000	0.4792	0.4184	0.0325	0.3879	0.1667	0.5000	0.5000
0.4000	0.4000	0.5362	0.4470	0.0127	0.2468	1.0000	1.0000	0.3333
0.5000	0.5000	0.5362	0.4184	0.0123	0.2415	0.6667	0.5000	0.3333
0.3000	0.3000	0.3158	0.3206	0.0325	0.3879	1.0000	1.0000	0.5000
0.3000	0.3000	0.2897	0.4082	0.0146	0.2660	0.5000	0.6667	0.3333
0.3000	0.3000	0.1789	0.3599	0.0127	0.2459	1.0000	1.0000	0.3333
0.3000	0.3000	0.3661	0.3611	0.0324	0.3870	1.0000	1.0000	0.3333
0.5000	0.5000	0.3883	0.3773	0.0146	0.2660	1.0000	1.0000	0.3333
0.5000	0.5000	0.3883	0.3773	0.0146	0.2660	1.0000	1.0000	0.3333
0.4000	0.4000	0.5362	0.4381	0.0127	0.2468	1.0000	1.0000	0.3333
0.5000	0.5000	0.5362	0.4381	0.0123	0.2415	1.0000	1.0000	0.3333
0.3000	0.3000	0.1525	0.1069	0.0026	0.0772	0.5000	0.5000	0.5000
0.3000	0.3000	0.2604	0.1947	0.0044	0.1132	0.0000	0.5000	0.5000
0.4000	0.4000	0.2604	0.2069	0.0051	0.1269	0.5000	0.5000	0.5000
0.4000	0.4000	0.2604	0.2069	0.0018	0.0383	0.5000	0.5000	0.5000
0.2000	0.2000	0.2604	0.1947	0.0044	0.1132	0.0000	0.5000	0.5000
0.2000	0.2000	0.1525	0.0000	0.0000	0.0000	0.0000	0.5000	0.5000
0.2000	0.2000	0.1525	0.0000	0.0000	0.0000	0.0000	0.5000	0.5000
0.6000	0.6000	0.3290	0.2862	0.0505	0.4468	0.6667	0.5000	0.3333
0.5000	0.5000	0.2128	0.2681	0.0508	0.4591	0.6667	0.5000	0.5000
0.5000	0.5000	0.1813	0.2681	0.0117	0.2192	0.6667	0.5000	0.5000
0.4000	0.4000	0.3006	0.2681	0.0121	0.2325	0.6667	0.5000	0.5000
0.6000	0.6000	0.3783	0.3862	0.0506	0.4527	1.0000	0.5000	1.0000
0.5000	0.5000	0.3712	0.2579	0.0187	0.2985	0.6667	0.5000	0.5000
0.6000	0.6000	0.4070	0.2862	0.0505	0.4468	0.6667	0.5000	0.5000
0.3000	0.3000	0.5039	0.2854	0.0055	0.1461	0.1667	0.5000	0.5000
0.3000	0.3000	0.4792	0.2854	0.0055	0.1461	0.5000	0.6667	0.3333

0.3000	0.3000	0.4792	0.4184	0.0055	0.1461	0.1667	0.1667	0.3333
0.1000	0.1000	0.3636	0.1984	0.0033	0.1016	0.1667	0.1667	0.3333
0.3000	0.3000	0.4792	0.4470	0.0107	0.2241	1.0000	1.0000	0.5000
0.4000	0.4000	0.4962	0.4470	0.0130	0.2493	1.0000	1.0000	0.5000
0.3000	0.3000	0.3914	0.4196	0.0107	0.2241	1.0000	1.0000	0.5000
0.3000	0.3000	0.4962	0.4196	0.0130	0.2493	1.0000	1.0000	0.5000
0.3000	0.3000	0.4792	0.4184	0.0107	0.2241	0.1667	0.5000	0.5000
0.3000	0.3000	0.4792	0.4470	0.0107	0.2241	1.0000	1.0000	0.5000
0.3000	0.3000	0.4792	0.4470	0.0107	0.2241	1.0000	1.0000	0.5000
0.5000	0.5000	0.4962	0.3689	0.0130	0.2493	1.0000	1.0000	0.5000
0.3000	0.3000	0.4962	0.4196	0.0130	0.2493	0.6667	0.5000	0.5000
0.5000	0.5000	0.5039	0.2854	0.0055	0.1461	0.1667	0.5000	0.5000
0.3000	0.3000	0.4792	0.2854	0.0055	0.1461	0.1667	0.1667	0.3333
0.3000	0.3000	0.4792	0.4184	0.0107	0.2241	0.1667	0.5000	0.5000
0.3000	0.3000	0.4962	0.2854	0.0130	0.2493	0.1667	0.5000	0.5000
0.3000	0.3000	0.4792	0.4184	0.0107	0.2241	0.1667	0.5000	0.5000
0.3000	0.3000	0.4255	0.4470	0.0107	0.2241	0.6667	0.5000	0.5000
0.3000	0.3000	0.4792	0.4184	0.0055	0.1461	0.6667	0.5000	0.3333
0.3000	0.3000	0.4792	0.4184	0.0107	0.2241	0.1667	0.5000	0.5000
0.3000	0.3000	0.4962	0.4184	0.0130	0.2493	0.1667	0.5000	0.5000
0.5000	0.5000	0.5954	0.4470	0.0187	0.3021	0.6667	0.5000	0.5000
0.5000	0.5000	0.5954	0.4470	0.0187	0.3021	0.6667	0.5000	0.5000
0.5000	0.5000	0.5954	0.4470	0.0187	0.3021	0.6667	0.5000	0.5000
0.5000	0.5000	0.5954	0.4196	0.0187	0.3021	0.6667	0.5000	0.5000
0.3000	0.3000	0.4255	0.4470	0.0055	0.1461	0.6667	0.5000	0.5000
0.5000	0.5000	0.5954	0.4196	0.0187	0.3021	0.6667	0.5000	0.5000
0.5000	0.5000	0.5954	0.4196	0.0187	0.3021	1.0000	1.0000	0.5000
0.5000	0.5000	0.6672	0.4470	0.0187	0.3021	0.6667	0.5000	0.5000
0.5000	0.5000	0.5954	0.4470	0.0187	0.3021	1.0000	1.0000	0.5000
0.3000	0.3000	0.4792	0.4470	0.0146	0.2660	1.0000	1.0000	0.5000
0.2000	0.2000	0.4962	0.2854	0.0130	0.2493	0.5000	1.0000	0.5000
0.2000	0.2000	0.1483	0.3052	0.0052	0.1361	0.1667	0.5000	0.5000
0.5000	0.5000	0.5039	0.4184	0.0130	0.2493	1.0000	1.0000	0.5000
0.4000	0.4000	0.5039	0.4184	0.0055	0.1461	0.5000	1.0000	0.5000
0.3000	0.3000	0.3067	0.3040	0.0129	0.2458	1.0000	1.0000	0.5000
0.2000	0.2000	0.3120	0.2854	0.0055	0.1461	0.1667	0.1667	0.3333
0.3000	0.3000	0.1732	0.3052	0.0054	0.1426	1.0000	1.0000	0.3333
0.3000	0.3000	0.3535	0.4196	0.0104	0.2202	1.0000	1.0000	0.3333
0.4000	0.4000	0.3133	0.3700	0.0102	0.2102	0.6667	0.5000	0.3333
0.4000	0.4000	0.3106	0.3426	0.0104	0.2193	0.6667	0.5000	0.3333
0.5000	0.5000	0.5028	0.4196	0.0104	0.2202	1.0000	1.0000	0.3333
0.5000	0.5000	0.3133	0.3700	0.0103	0.2166	1.0000	1.0000	0.3333
0.3000	0.3000	0.5028	0.4196	0.0104	0.2202	1.0000	1.0000	0.3333
0.5000	0.5000	0.5362	0.4470	0.0107	0.2241	0.6667	0.5000	0.5000
0.4000	0.4000	0.4255	0.4470	0.0104	0.2202	1.0000	1.0000	0.3333
0.4000	0.4000	0.5028	0.4196	0.0104	0.2202	1.0000	1.0000	0.3333
0.4000	0.4000	0.5028	0.4196	0.0130	0.2493	1.0000	1.0000	0.5000
0.3000	0.3000	0.1426	0.2936	0.0102	0.2102	0.5000	0.6667	0.3333
0.4000	0.4000	0.2928	0.3052	0.0127	0.2386	1.0000	1.0000	0.5000
0.2000	0.2000	0.1734	0.3052	0.0107	0.2205	1.0000	1.0000	0.5000
0.3000	0.3000	0.0877	0.1150	0.0054	0.1426	1.0000	1.0000	0.0000
0.3000	0.3000	0.1548	0.3052	0.0052	0.1354	1.0000	1.0000	0.3333
0.3000	0.3000	0.2360	0.3326	0.0054	0.1426	1.0000	1.0000	0.3333
0.5000	0.5000	0.3920	0.3052	0.0185	0.2920	0.5000	1.0000	0.5000
0.4000	0.4000	0.4792	0.4184	0.0055	0.1461	1.0000	1.0000	0.5000
0.4000	0.4000	0.5028	0.4470	0.0107	0.2241	0.6667	0.5000	0.5000
0.3000	0.3000	0.4761	0.4470	0.0104	0.2202	0.6667	0.5000	0.3333
0.5000	0.5000	0.5954	0.4470	0.0187	0.3021	0.6667	0.5000	0.5000
0.5000	0.5000	0.5039	0.2854	0.0107	0.2241	0.1667	0.5000	0.5000
0.5000	0.5000	0.5039	0.2854	0.0055	0.1461	0.5000	1.0000	0.5000
0.3000	0.3000	0.4792	0.2854	0.0055	0.1461	0.1667	0.1667	0.3333
0.3000	0.3000	0.4962	0.2019	0.0130	0.2493	0.6667	0.5000	0.5000
0.6000	0.6000	0.6348	0.6387	0.0187	0.3021	1.0000	0.5000	1.0000
0.5000	0.5000	0.4741	0.3976	0.0101	0.2062	1.0000	1.0000	0.5000
0.3000	0.3000	0.3535	0.4184	0.0123	0.2415	0.5000	0.6667	0.3333
0.6000	0.6000	0.6615	0.4746	0.0187	0.3021	0.6667	0.5000	0.5000
0.6000	0.6000	0.3344	0.3700	0.0220	0.3262	0.6667	0.5000	0.5000
0.6000	0.6000	0.6348	0.4746	0.0220	0.3262	1.0000	0.5000	0.5000
0.5000	0.5000	0.5008	0.3976	0.0186	0.2969	0.6667	0.5000	0.5000
0.5000	0.5000	0.5008	0.3414	0.0293	0.3713	0.6667	0.5000	0.5000
0.6000	0.6000	0.6615	0.6387	0.0293	0.3713	1.0000	0.5000	1.0000
0.5000	0.5000	0.4844	0.4463	0.0185	0.2913	1.0000	0.3333	1.0000
0.5000	0.5000	0.3592	0.3414	0.0219	0.3208	0.6667	0.5000	0.5000
0.5000	0.5000	0.2919	0.3414	0.0078	0.1841	1.0000	1.0000	0.3333
0.5000	0.5000	0.4741	0.3976	0.0187	0.3021	0.6667	0.3333	0.5000
0.4000	0.4000	0.6348	0.4746	0.0146	0.2660	1.0000	1.0000	0.5000
0.6000	0.6000	0.5362	0.4260	0.0107	0.2236	1.0000	1.0000	0.3333

0.6000	0.6000	0.5362	0.4184	0.0094	0.2067	1.0000	1.0000	0.3333
0.4000	0.4000	0.5164	0.4184	0.0325	0.3879	0.6667	0.5000	0.3333
0.3000	0.3000	0.5164	0.4184	0.0146	0.2660	1.0000	1.0000	0.3333
0.4000	0.4000	0.4792	0.4184	0.0325	0.3879	0.6667	0.5000	0.5000
0.5000	0.5000	0.5362	0.4184	0.0104	0.2202	1.0000	1.0000	0.3333
0.6000	0.6000	0.5362	0.4184	0.0104	0.2202	1.0000	1.0000	0.5000
0.6000	0.6000	0.5295	0.4381	0.0146	0.2660	1.0000	1.0000	0.3333
0.4000	0.4000	0.5164	0.3725	0.0146	0.2660	1.0000	1.0000	0.3333
0.4000	0.4000	0.3056	0.3414	0.0103	0.2166	1.0000	1.0000	0.3333
0.2000	0.2000	0.4962	0.4184	0.0130	0.2493	0.5000	1.0000	0.5000
0.3000	0.3000	0.4083	0.3773	0.0123	0.2415	0.0000	0.5000	0.5000
0.5000	0.5000	0.5028	0.4470	0.0104	0.2202	1.0000	1.0000	0.5000
0.5000	0.5000	0.5954	0.4470	0.0187	0.3021	1.0000	1.0000	0.5000
0.4000	0.4000	0.5295	0.4381	0.0104	0.2202	1.0000	1.0000	0.5000
0.5000	0.5000	0.5028	0.4196	0.0104	0.2202	1.0000	1.0000	0.3333
0.4000	0.4000	0.5164	0.4470	0.0078	0.1841	1.0000	1.0000	0.3333
0.4000	0.4000	0.5028	0.4184	0.0055	0.1461	0.6667	0.5000	0.5000
0.7000	0.7000	0.6615	0.4138	0.0663	0.5082	1.0000	1.0000	0.3333
0.6000	0.6000	0.5295	0.4470	0.0123	0.2415	1.0000	1.0000	0.3333
0.6000	0.6000	0.5994	0.4184	0.0663	0.5082	1.0000	1.0000	0.3333
0.6000	0.6000	0.3186	0.2677	0.0119	0.2275	0.6667	0.5000	0.3333
0.2000	0.2000	0.3186	0.2677	0.0119	0.2275	0.1667	0.1667	0.3333
0.6000	0.6000	0.5008	0.3849	0.0123	0.2408	0.6667	0.5000	0.5000
0.7000	0.7000	0.5266	0.9311	0.0930	0.5667	1.0000	0.5000	0.5000
0.6000	0.6000	0.5008	0.3849	0.0187	0.3003	1.0000	0.5000	1.0000
0.7000	0.7000	0.5602	0.9311	0.0930	0.5667	1.0000	0.5000	0.5000
0.5000	0.5000	0.3893	0.4033	0.0105	0.2189	0.6667	0.5000	0.3333
0.4000	0.4000	0.4321	0.3781	0.0549	0.4756	0.6667	0.5000	0.5000
0.5000	0.5000	0.4715	0.3781	0.0506	0.4513	0.6667	0.5000	0.5000
0.4000	0.4000	0.3521	0.4033	0.0103	0.2166	0.6667	0.5000	0.3333
0.6000	0.6000	0.2365	0.3036	0.0106	0.2224	1.0000	1.0000	0.5000
0.5000	0.5000	0.2735	0.2805	0.0101	0.2138	0.6667	0.5000	0.3333
0.3000	0.3000	0.3521	0.1664	0.0056	0.1467	0.6667	0.5000	0.3333
0.6000	0.6000	0.5240	0.5437	0.1300	0.6168	1.0000	0.5000	1.0000
0.4000	0.4000	0.4609	0.4598	0.1298	0.6076	1.0000	0.5000	1.0000
0.6000	0.6000	0.3675	0.2453	0.0549	0.4756	0.6667	0.5000	0.5000
0.7000	0.7000	0.3541	0.4058	0.0549	0.4756	0.6667	0.5000	0.5000
0.4000	0.4000	0.3803	0.3862	0.2197	0.7210	0.6667	0.5000	0.3333
0.6000	0.6000	0.3534	0.3618	0.0548	0.4748	0.6667	0.5000	0.3333
0.5000	0.5000	0.2881	0.3651	0.0781	0.5369	0.6667	0.5000	0.3333
0.6000	0.6000	0.4324	0.2312	0.2197	0.7218	0.6667	0.5000	0.3333
0.6000	0.6000	0.3803	0.4138	0.2197	0.7218	0.6667	0.5000	0.3333
0.5000	0.5000	0.3164	0.3672	0.0260	0.3387	0.6667	0.5000	0.3333
0.6000	0.6000	0.3423	0.3672	0.0261	0.3446	0.6667	0.5000	0.3333
0.5000	0.5000	0.3557	0.4289	0.0170	0.2872	0.6667	0.5000	0.3333
0.5000	0.5000	0.2979	0.2399	0.0170	0.2880	0.6667	0.5000	0.3333
0.4000	0.4000	0.1637	0.1826	0.0100	0.2149	0.1667	0.1667	0.3333
0.3000	0.3000	0.1771	0.1826	0.0098	0.2057	0.1667	0.1667	0.3333
0.4000	0.4000	0.5077	0.4138	0.0369	0.4055	0.6667	0.5000	0.5000
0.4000	0.4000	0.3893	0.2232	0.2196	0.7183	0.6667	0.5000	0.3333
0.4000	0.4000	0.3521	0.3089	0.0101	0.2138	0.6667	0.5000	0.3333
0.4000	0.4000	0.3313	0.2385	0.0075	0.1778	0.6667	0.5000	0.3333
0.3000	0.3000	0.2619	0.2519	0.0105	0.2189	0.6667	0.5000	0.3333
0.3000	0.3000	0.2731	0.1826	0.0105	0.2189	0.6667	0.5000	0.3333
0.5000	0.5000	0.3893	0.3753	0.0101	0.2138	0.6667	0.5000	0.5000
0.4000	0.4000	0.3893	0.4114	0.0781	0.5333	0.6667	0.5000	0.3333
0.4000	0.4000	0.3893	0.2533	0.0105	0.2189	0.6667	0.5000	0.3333
0.5000	0.5000	0.3893	0.4138	0.0106	0.2224	0.6667	0.5000	0.5000
0.5000	0.5000	0.3893	0.3495	0.0105	0.2189	0.6667	0.5000	0.5000
0.3000	0.3000	0.3893	0.2439	0.0508	0.4591	0.6667	0.5000	0.3333
0.4000	0.4000	0.3521	0.2439	0.0105	0.2189	0.6667	0.5000	0.3333
0.5000	0.5000	0.4844	0.4573	0.0101	0.2095	0.6667	0.3333	0.5000
0.5000	0.5000	0.4361	0.3862	0.0103	0.2166	1.0000	1.0000	0.3333
0.6000	0.6000	0.5135	0.3862	0.0103	0.2166	1.0000	1.0000	0.5000
0.4000	0.4000	0.5295	0.4470	0.0104	0.2202	1.0000	1.0000	0.3333
0.4000	0.4000	0.5295	0.4470	0.0104	0.2202	1.0000	1.0000	0.3333
0.6000	0.6000	0.6720	0.5617	0.0103	0.2166	1.0000	0.5000	1.0000
0.6000	0.6000	0.4372	0.2403	0.0103	0.2166	0.6667	0.5000	0.3333
0.5000	0.5000	0.4844	0.3618	0.0105	0.2189	0.6667	0.5000	0.5000
0.5000	0.5000	0.5008	0.5658	0.0104	0.2202	0.6667	0.3333	0.5000
0.6000	0.6000	0.5135	0.3862	0.2194	0.7111	1.0000	1.0000	0.5000
0.5000	0.5000	0.6615	0.4470	0.0106	0.2224	1.0000	1.0000	0.3333
0.3000	0.3000	0.3755	0.3700	0.0103	0.2166	0.6667	0.5000	0.3333
0.3000	0.3000	0.3883	0.3862	0.0101	0.2095	1.0000	1.0000	0.3333
0.6000	0.6000	0.3597	0.3509	0.0369	0.4055	0.6667	0.5000	0.5000
0.4000	0.4000	0.1895	0.1745	0.0127	0.2459	0.6667	0.5000	0.3333
0.5000	0.5000	0.4931	0.3495	0.2196	0.7183	0.6667	0.5000	0.5000

0.4000	0.4000	0.3143	0.1288	0.0125	0.2360	0.0000	0.0000	0.0000
0.5000	0.5000	0.3297	0.2232	0.2196	0.7183	0.5000	0.6667	0.3333
0.4000	0.4000	0.5135	0.3862	0.0106	0.2224	1.0000	1.0000	0.3333
0.3000	0.3000	0.4365	0.3279	0.0099	0.1993	1.0000	1.0000	0.3333
0.2000	0.2000	0.4844	0.3279	0.0101	0.2095	0.6667	0.3333	0.5000
0.5000	0.5000	0.5008	0.3279	0.0103	0.2166	1.0000	1.0000	0.3333
0.2000	0.2000	0.2157	0.3576	0.0070	0.1636	1.0000	1.0000	0.3333
0.4000	0.4000	0.3559	0.2245	0.0056	0.1373	0.0000	0.5000	0.5000
0.6000	0.6000	0.4365	0.3618	0.0105	0.2189	1.0000	1.0000	0.3333
0.4000	0.4000	0.4844	0.3618	0.0123	0.2390	1.0000	1.0000	0.5000
0.2000	0.2000	0.4844	0.2950	0.0121	0.2311	0.6667	0.3333	0.5000
0.5000	0.5000	0.5008	0.3618	0.0123	0.2390	1.0000	1.0000	0.5000
0.5000	0.5000	0.5135	0.3862	0.0105	0.2189	1.0000	1.0000	0.3333
0.3000	0.3000	0.3313	0.3576	0.0095	0.2064	0.1667	0.1667	0.3333
0.4000	0.4000	0.4284	0.5644	0.0121	0.2318	1.0000	0.3333	1.0000
0.4000	0.4000	0.5362	0.4470	0.0078	0.1841	0.6667	0.5000	0.3333
0.4000	0.4000	0.5362	0.4470	0.0325	0.3879	0.6667	0.5000	0.3333
0.1000	0.1000	0.4715	0.3781	0.0006	0.0223	0.6667	0.3333	0.3333
0.2000	0.2000	0.3766	0.4184	0.0123	0.2415	0.6667	0.5000	0.3333
0.7000	0.7000	0.5008	0.4598	0.0121	0.2339	0.6667	0.5000	0.5000
0.5000	0.5000	0.4844	0.5437	0.0121	0.2325	1.0000	0.5000	1.0000
0.4000	0.4000	0.4607	0.4058	0.0245	0.3428	0.6667	0.5000	0.5000
0.2000	0.2000	0.2003	0.3218	0.0127	0.2468	1.0000	1.0000	0.3333
0.4000	0.4000	0.0947	0.0666	0.0101	0.2157	0.0000	0.0000	0.0000
0.3000	0.3000	0.4284	0.4598	0.0121	0.2318	1.0000	0.3333	1.0000
0.3000	0.3000	0.4651	0.4598	0.0121	0.2318	1.0000	0.3333	1.0000
0.4000	0.4000	0.4284	0.5644	0.0121	0.2318	1.0000	0.3333	1.0000
0.2000	0.2000	0.4284	0.4598	0.0290	0.3606	0.6667	0.3333	0.5000
0.4000	0.4000	0.3549	0.3862	0.0101	0.2138	0.6667	0.5000	0.3333
0.4000	0.4000	0.4284	0.5437	0.0121	0.2318	1.0000	0.3333	1.0000
0.5000	0.5000	0.5717	0.5437	0.0121	0.2318	1.0000	0.5000	1.0000
0.4000	0.4000	0.3945	0.4092	0.1104	0.5764	1.0000	0.5000	0.5000
0.3000	0.3000	0.4240	0.2957	0.0123	0.2390	0.6667	0.3333	0.5000
0.4000	0.4000	0.5135	0.3862	0.0103	0.2166	1.0000	1.0000	0.3333
0.3000	0.3000	0.4563	0.3210	0.0127	0.2468	0.6667	0.5000	0.3333
0.4000	0.4000	0.4515	0.3983	0.0127	0.2468	1.0000	1.0000	0.5000
0.5000	0.5000	0.4361	0.1400	0.0049	0.1330	0.5000	0.5000	0.5000
0.1000	0.1000	0.4715	0.4024	0.0367	0.3976	0.5000	0.0000	0.5000
0.2000	0.2000	0.5516	0.3576	0.0054	0.1396	0.1667	0.5000	0.5000
0.6000	0.6000	0.4719	0.3486	0.0103	0.2166	0.6667	0.5000	0.5000
0.3000	0.3000	0.2604	0.1375	0.0047	0.1259	0.0000	0.5000	0.5000
0.4000	0.4000	0.4844	0.3209	0.0103	0.2166	0.6667	0.5000	0.5000
0.3000	0.3000	0.4083	0.4184	0.0073	0.1776	0.6667	0.5000	0.5000
0.4000	0.4000	0.1681	0.1745	0.0126	0.2432	0.6667	0.5000	0.3333
0.4000	0.4000	0.2743	0.0000	0.0029	0.0344	0.0000	0.5000	0.5000
0.1000	0.1000	0.2234	#NAME?	0.0000	0.0000	0.0000	0.0000	0.0000
0.6000	0.6000	0.4844	0.2681	0.0123	0.2390	0.6667	0.5000	0.5000
0.3000	0.3000	0.4844	0.2681	0.0123	0.2390	0.6667	0.3333	0.5000
0.6000	0.6000	0.5193	0.4138	0.0252	0.3473	1.0000	0.5000	0.5000
0.6000	0.6000	0.2329	0.3324	0.0127	0.2468	0.6667	0.5000	0.5000
0.2000	0.2000	0.3143	0.0375	0.0098	0.2050	0.0000	0.5000	0.5000
0.4000	0.4000	0.4520	0.2615	0.0103	0.2166	0.1667	0.3333	0.1667
0.3000	0.3000	0.2734	0.2117	0.0107	0.2205	0.5000	0.5000	0.5000
0.2000	0.2000	0.2734	0.0692	0.0070	0.1603	0.5000	0.5000	0.5000
0.4000	0.4000	0.1895	0.1745	0.0127	0.2459	0.6667	0.5000	0.3333
0.4000	0.4000	0.4212	0.3849	0.0367	0.3983	0.6667	0.3333	0.5000
0.3000	0.3000	0.4578	0.2663	0.0062	0.1465	0.5000	0.0000	0.5000
0.5000	0.5000	0.5717	0.3573	0.0123	0.2390	0.6667	0.5000	0.5000
0.3000	0.3000	0.3514	0.3573	0.0360	0.3753	1.0000	0.5000	0.5000
0.2000	0.2000	0.2941	0.1502	0.0045	0.1061	0.1667	0.1667	0.3333
0.2000	0.2000	0.2025	0.3507	0.0042	0.1199	0.1667	0.1667	0.3333
0.2000	0.2000	0.2025	0.3507	0.0033	0.0961	0.1667	0.1667	0.3333
0.5000	0.5000	0.4176	0.3507	0.0249	0.3366	0.5000	0.1667	0.5000
0.3000	0.3000	0.3885	0.3697	0.0054	0.1363	0.1667	0.5000	0.5000
0.4000	0.4000	0.3816	0.4381	0.0127	0.2468	0.1667	0.5000	0.5000
0.4000	0.4000	0.3872	0.4196	0.0781	0.5369	1.0000	1.0000	0.3333
0.5000	0.5000	0.6615	0.4196	0.2197	0.7218	1.0000	1.0000	0.3333
0.3000	0.3000	0.1752	0.0974	0.0503	0.4393	1.0000	1.0000	0.3333
0.4000	0.4000	0.6093	0.4228	0.0777	0.5359	1.0000	1.0000	0.5000
0.5000	0.5000	0.4844	0.5241	0.0103	0.2166	1.0000	0.5000	1.0000
0.6000	0.6000	0.6093	0.4252	0.0776	0.5324	1.0000	0.5000	1.0000
0.5000	0.5000	0.5008	0.3952	0.0548	0.4720	1.0000	1.0000	0.5000
0.5000	0.5000	0.5379	0.4186	0.0549	0.4756	1.0000	0.5000	1.0000
0.4000	0.4000	0.4844	0.5437	0.0367	0.4001	1.0000	0.5000	1.0000
0.5000	0.5000	0.5379	0.5893	0.0548	0.4720	1.0000	0.5000	1.0000
0.6000	0.6000	0.6093	0.9506	0.0777	0.5351	1.0000	1.0000	1.0000
0.5000	0.5000	0.5008	0.5437	0.0662	0.5046	1.0000	0.5000	1.0000

0.4000	0.4000	0.4709	0.3618	0.0545	0.4630	1.0000	0.3333	1.0000
0.6000	0.6000	0.4349	0.5437	0.1237	0.6029	1.0000	0.5000	1.0000
0.5000	0.5000	0.4723	0.3993	0.0663	0.5082	1.0000	1.0000	0.3333
0.5000	0.5000	0.4284	0.9506	0.0184	0.2871	1.0000	0.5000	1.0000
0.4000	0.4000	0.3663	0.4228	0.0662	0.5048	0.6667	0.5000	0.3333
0.5000	0.5000	0.6429	0.3993	0.0777	0.5359	1.0000	1.0000	0.5000
0.5000	0.5000	0.4372	0.9271	0.0549	0.4756	1.0000	1.0000	0.3333
0.4000	0.4000	0.3061	0.3894	0.0105	0.2189	0.6667	0.5000	0.5000
0.5000	0.5000	0.3573	0.9506	0.0114	0.2294	1.0000	1.0000	0.3333
0.5000	0.5000	0.3253	0.3700	0.0103	0.2166	1.0000	1.0000	0.3333
0.5000	0.5000	0.3253	0.3700	0.0103	0.2168	1.0000	1.0000	0.3333
0.5000	0.5000	0.4240	0.3976	0.0662	0.5046	0.6667	0.5000	0.3333
0.4000	0.4000	0.6760	0.9271	0.0777	0.5359	0.6667	0.3333	0.5000
0.3000	0.3000	0.3989	0.3507	0.0106	0.2207	0.5000	0.6667	0.3333
0.5000	0.5000	0.1895	0.1468	0.0106	0.2205	1.0000	1.0000	0.3333
0.3000	0.3000	0.3989	0.3507	0.0106	0.2207	1.0000	1.0000	0.3333
0.3000	0.3000	0.1895	0.1468	0.0106	0.2205	1.0000	1.0000	0.3333
0.2000	0.2000	0.3133	0.3467	0.0325	0.3879	0.5000	0.6667	0.3333
0.5000	0.5000	0.3989	0.3507	0.0057	0.1504	1.0000	1.0000	0.3333
0.2000	0.2000	0.3287	0.3507	0.0325	0.3879	0.5000	0.6667	0.3333
0.3000	0.3000	0.2173	0.3507	0.0048	0.1324	1.0000	1.0000	0.3333
0.5000	0.5000	0.3112	0.9271	0.0123	0.2415	1.0000	1.0000	0.3333
0.6000	0.6000	0.4405	0.9311	0.0325	0.3879	1.0000	1.0000	0.3333
0.6000	0.6000	0.4097	0.9311	0.0325	0.3879	1.0000	1.0000	0.3333
0.6000	0.6000	0.3989	0.9311	0.0123	0.2415	1.0000	1.0000	0.3333
0.6000	0.6000	0.3621	0.9311	0.0123	0.2415	1.0000	1.0000	0.3333
0.6000	0.6000	0.3621	0.9311	0.0115	0.2328	1.0000	1.0000	0.3333
0.5000	0.5000	0.5008	0.2656	0.0104	0.2174	1.0000	0.5000	1.0000
0.6000	0.6000	0.5008	0.5437	0.0104	0.2184	1.0000	1.0000	1.0000
0.5000	0.5000	0.5717	0.3064	0.0762	0.5308	1.0000	1.0000	1.0000
0.6000	0.6000	0.5717	0.3647	0.0104	0.2184	1.0000	1.0000	1.0000
0.6000	0.6000	0.5008	0.2392	0.0104	0.2174	1.0000	0.5000	1.0000
0.6000	0.6000	0.4421	0.2392	0.0092	0.2028	1.0000	0.5000	1.0000
0.5000	0.5000	0.5717	0.2539	0.0762	0.5308	1.0000	0.5000	1.0000
0.7000	0.7000	0.4844	0.3723	0.0762	0.5297	1.0000	1.0000	1.0000
0.8000	0.8000	0.5100	0.3210	0.2196	0.7183	1.0000	1.0000	1.0000
0.7000	0.7000	0.4844	0.2933	0.0243	0.3390	1.0000	1.0000	1.0000
0.6000	0.6000	0.3552	0.1911	0.0762	0.5308	1.0000	0.5000	1.0000
0.6000	0.6000	0.6598	0.3210	0.2196	0.7184	0.6667	0.5000	0.5000
0.6000	0.6000	0.5008	0.3211	0.0103	0.2168	0.6667	0.5000	0.5000
0.6000	0.6000	0.3597	0.3618	0.0105	0.2190	0.6667	0.5000	0.3333
0.6000	0.6000	0.4844	0.3100	0.0762	0.5297	1.0000	0.5000	1.0000
0.6000	0.6000	0.4844	0.3100	0.0762	0.5297	1.0000	0.5000	1.0000
0.4000	0.4000	0.4844	0.3100	0.0104	0.2174	1.0000	0.5000	1.0000
0.5000	0.5000	0.4719	0.3210	0.0762	0.5308	1.0000	0.5000	1.0000
0.6000	0.6000	0.5726	0.3210	0.0762	0.5308	0.6667	0.5000	0.5000
0.6000	0.6000	0.2897	0.2117	0.0104	0.2184	1.0000	0.5000	1.0000
0.5000	0.5000	0.4777	0.3839	0.0762	0.5325	1.0000	0.5000	1.0000
0.5000	0.5000	0.5008	0.3490	0.0762	0.5308	1.0000	1.0000	0.3333
0.5000	0.5000	0.4741	0.4041	0.0106	0.2207	1.0000	1.0000	0.3333
0.3000	0.3000	0.2897	0.1313	0.0106	0.2207	1.0000	1.0000	0.3333
0.4000	0.4000	0.1741	0.1149	0.0106	0.2207	1.0000	1.0000	0.3333
0.6000	0.6000	0.3597	0.3798	0.0127	0.2440	0.6667	0.5000	0.3333
0.5000	0.5000	0.4844	0.3618	0.0105	0.2197	1.0000	1.0000	0.5000
0.6000	0.6000	0.4719	0.3344	0.0127	0.2450	1.0000	1.0000	0.3333
0.5000	0.5000	0.3621	0.3507	0.0762	0.5325	1.0000	1.0000	0.5000
0.5000	0.5000	0.5717	0.2688	0.2196	0.7191	0.6667	0.5000	0.5000
0.7000	0.7000	0.4844	0.3287	0.0762	0.5297	1.0000	1.0000	1.0000
0.6000	0.6000	0.4233	0.3210	0.2196	0.7183	0.6667	0.5000	0.5000
0.5000	0.5000	0.3621	0.4058	0.2196	0.7183	1.0000	1.0000	0.5000
0.5000	0.5000	0.5008	0.3490	0.0106	0.2207	1.0000	1.0000	0.3333
0.5000	0.5000	0.3755	0.3764	0.2196	0.7201	1.0000	1.0000	0.3333
0.5000	0.5000	0.5008	0.3490	0.0762	0.5308	1.0000	1.0000	0.3333
0.7000	0.7000	0.5008	0.3700	0.2196	0.7201	1.0000	1.0000	1.0000
0.5000	0.5000	0.5008	0.3700	0.2196	0.7201	1.0000	1.0000	0.3333
0.4000	0.4000	0.2897	0.3013	0.2196	0.7201	1.0000	1.0000	0.3333
0.6000	0.6000	0.5008	0.3344	0.0127	0.2440	1.0000	1.0000	1.0000
0.6000	0.6000	0.4844	0.3501	0.0126	0.2434	0.6667	0.5000	0.5000
0.5000	0.5000	0.3120	0.3618	0.1240	0.6153	1.0000	1.0000	0.3333
0.6000	0.6000	0.5008	0.3618	0.0105	0.2197	1.0000	1.0000	1.0000
0.5000	0.5000	0.4519	0.3344	0.2196	0.7184	1.0000	1.0000	0.3333
0.5000	0.5000	0.5008	0.3618	0.2196	0.7201	1.0000	1.0000	0.3333
0.6000	0.6000	0.5008	0.3618	0.2196	0.7195	1.0000	1.0000	0.5000
0.6000	0.6000	0.5008	0.3624	0.2197	0.7218	1.0000	1.0000	0.5000
0.6000	0.6000	0.4384	0.4598	0.0187	0.2935	0.6667	0.5000	0.5000
0.5000	0.5000	0.8105	0.7460	0.0123	0.2390	0.6667	0.5000	0.3333
0.4000	0.4000	0.4578	0.5489	0.0367	0.3983	0.6667	0.5000	0.5000

0.3000	0.3000	0.4578	0.3849	0.0364	0.3870	0.6667	0.3333	0.5000
0.5000	0.5000	0.4741	0.5204	0.0123	0.2398	1.0000	1.0000	0.5000
0.5000	0.5000	0.3022	0.3287	0.0123	0.2390	1.0000	1.0000	0.5000
0.4000	0.4000	0.4092	0.3563	0.0045	0.1213	1.0000	1.0000	0.3333
0.7000	0.7000	0.4741	0.3849	0.0119	0.2347	0.6667	0.5000	0.5000
0.5000	0.5000	0.4844	0.3563	0.0123	0.2390	1.0000	1.0000	0.5000
0.6000	0.6000	0.5516	0.3117	0.0103	0.2166	0.6667	0.5000	0.3333
0.4000	0.4000	0.4578	0.2932	0.0103	0.2166	0.6667	0.3333	0.5000
0.4000	0.4000	0.4578	0.2727	0.0101	0.2095	0.6667	0.3333	0.5000
0.6000	0.6000	0.4741	0.4598	0.0660	0.4982	1.0000	1.0000	0.5000
0.6000	0.6000	0.4578	0.4598	0.0123	0.2390	0.6667	0.5000	0.5000
0.2000	0.2000	0.5100	0.1497	0.0045	0.1221	0.5000	0.5000	0.5000
0.3000	0.3000	0.4578	0.4598	0.0123	0.2390	0.6667	0.3333	0.5000
0.1000	0.1000	0.2958	#NAME?	0.0095	0.1502	0.0000	0.5000	0.5000
0.1000	0.1000	0.2912	0.0000	0.0226	0.2556	0.0000	0.5000	0.5000
0.1000	0.1000	0.2912	0.0000	0.0226	0.2556	0.0000	0.5000	0.5000
0.5000	0.5000	0.3287	0.4289	0.0106	0.2216	1.0000	1.0000	0.5000
0.4000	0.4000	0.2188	0.2925	0.0037	0.1087	0.6667	0.5000	0.5000
0.5000	0.5000	0.4453	0.3486	0.0119	0.2347	1.0000	1.0000	0.5000
0.4000	0.4000	0.4869	0.4138	0.0121	0.2325	0.6667	0.5000	0.5000
0.4000	0.4000	0.2734	0.3279	0.0103	0.2166	1.0000	0.5000	0.5000
0.4000	0.4000	0.2188	0.1742	0.0101	0.2095	0.6667	0.5000	0.5000
0.3000	0.3000	0.4099	0.3210	0.0103	0.2166	0.6667	0.5000	0.5000
0.4000	0.4000	0.5100	0.1417	0.0054	0.1426	0.6667	0.5000	0.5000
0.6000	0.6000	0.4361	0.3862	0.0103	0.2166	1.0000	1.0000	0.5000
0.3000	0.3000	0.3185	0.2656	0.0103	0.2166	0.6667	0.3333	0.5000
0.4000	0.4000	0.3313	0.3862	0.0121	0.2318	0.6667	0.5000	0.5000
0.5000	0.5000	0.3945	0.2544	0.0103	0.2166	1.0000	0.5000	0.5000
0.4000	0.4000	0.3766	0.4289	0.0548	0.4748	1.0000	1.0000	0.5000
0.5000	0.5000	0.5028	0.3901	0.0106	0.2219	0.6667	0.5000	0.5000
0.4000	0.4000	0.5994	0.4381	0.0104	0.2202	0.6667	0.5000	0.5000
0.5000	0.5000	0.5994	0.4381	0.0106	0.2219	1.0000	0.5000	1.0000
0.5000	0.5000	0.6615	0.3725	0.0106	0.2219	0.6667	0.5000	0.5000
0.5000	0.5000	0.6615	0.4722	0.0106	0.2219	0.6667	0.5000	0.5000
0.5000	0.5000	0.5164	0.4470	0.0106	0.2219	0.6667	0.5000	0.5000
0.5000	0.5000	0.4255	0.4722	0.0106	0.2219	0.6667	0.5000	0.3333
0.5000	0.5000	0.5362	0.4381	0.0106	0.2219	0.6667	0.5000	0.5000
0.5000	0.5000	0.5164	0.4722	0.0094	0.2067	0.6667	0.5000	0.5000
0.5000	0.5000	0.5362	0.4470	0.0106	0.2219	1.0000	1.0000	0.5000
0.5000	0.5000	0.6615	0.4722	0.0106	0.2219	0.6667	0.5000	0.5000
0.5000	0.5000	0.6615	0.4722	0.0106	0.2219	0.6667	0.5000	0.5000
0.5000	0.5000	0.5362	0.4722	0.0106	0.2219	1.0000	1.0000	0.5000
0.5000	0.5000	0.6615	0.4722	0.0106	0.2219	1.0000	1.0000	0.5000
0.5000	0.5000	0.6615	0.4722	0.0106	0.2219	1.0000	1.0000	0.5000
0.5000	0.5000	0.6615	0.4470	0.0106	0.2219	1.0000	1.0000	0.5000
0.4000	0.4000	0.3022	0.3209	0.0103	0.2112	0.6667	0.5000	0.5000
0.4000	0.4000	0.4844	0.3209	0.0103	0.2112	0.6667	0.5000	0.5000
0.4000	0.4000	0.3022	0.3209	0.0105	0.2183	0.6667	0.5000	0.5000
0.4000	0.4000	0.3592	0.3209	0.0105	0.2183	0.6667	0.5000	0.5000
0.4000	0.4000	0.2485	0.3461	0.0100	0.1998	0.6667	0.5000	0.5000
0.4000	0.4000	0.3592	0.3741	0.0106	0.2219	0.6667	0.5000	0.5000
0.5000	0.5000	0.5362	0.4722	0.0106	0.2219	0.6667	0.5000	0.5000
0.4000	0.4000	0.3022	0.3976	0.0105	0.2183	0.6667	0.5000	0.5000
0.2000	0.2000	0.1563	0.1368	0.0106	0.2205	0.5000	0.6667	0.3333
0.2000	0.2000	0.2091	0.6937	0.0106	0.2205	0.5000	0.6667	0.3333
0.3000	0.3000	0.2003	0.1380	0.0062	0.1576	0.5000	0.5000	0.0000
0.3000	0.3000	0.2137	0.1582	0.0062	0.1576	0.5000	0.5000	0.0000
0.3000	0.3000	0.2137	0.3710	0.0063	0.1599	0.5000	0.6667	0.3333
0.3000	0.3000	0.2003	0.1380	0.0127	0.2445	0.5000	0.5000	0.0000
0.3000	0.3000	0.2003	0.1380	0.0063	0.1599	0.5000	0.6667	0.3333
0.3000	0.3000	0.2003	0.1380	0.0062	0.1569	0.5000	0.5000	0.0000
0.3000	0.3000	0.2003	0.1380	0.0127	0.2438	0.5000	0.5000	0.0000
0.3000	0.3000	0.2003	0.1380	0.0062	0.1576	1.0000	1.0000	0.0000
0.3000	0.3000	0.2003	0.1380	0.0062	0.1590	1.0000	1.0000	0.3333
0.3000	0.3000	0.2003	0.1380	0.0062	0.1576	0.5000	0.5000	0.0000
0.3000	0.3000	0.2003	0.1468	0.0062	0.1569	0.5000	0.6667	0.3333
0.3000	0.3000	0.2003	0.1380	0.0062	0.1569	1.0000	1.0000	0.0000
0.5000	0.5000	0.2960	0.3710	0.0106	0.2202	0.1667	0.1667	0.3333
0.4000	0.4000	0.4844	0.2766	0.0103	0.2166	0.6667	0.5000	0.5000
0.6000	0.6000	0.5223	0.3507	0.0244	0.3392	0.6667	0.5000	0.5000
0.3000	0.3000	0.3863	0.3710	0.0035	0.1046	1.0000	1.0000	0.3333
0.6000	0.6000	0.4576	0.4260	0.2197	0.7218	0.6667	0.5000	0.5000
0.3000	0.3000	0.4269	0.3032	0.0091	0.1889	0.5000	0.5000	0.0000
0.3000	0.3000	0.2137	0.1670	0.0032	0.0978	0.1667	0.1667	0.3333
0.3000	0.3000	0.2137	0.3710	0.0032	0.0978	0.1667	0.1667	0.3333
0.2000	0.2000	0.1637	0.1745	0.0313	0.3445	0.0000	0.0000	0.0000
0.6000	0.6000	0.5031	0.3654	0.0761	0.5289	1.0000	0.5000	1.0000

0.3000	0.3000	0.4777	0.3013	0.2196	0.7195	1.0000	1.0000	0.3333
0.4000	0.4000	0.4284	0.2766	0.0101	0.2088	0.6667	0.3333	0.3333
0.5000	0.5000	0.4231	0.2347	0.0106	0.2202	0.1667	0.1667	0.3333
0.5000	0.5000	0.4231	0.3710	0.0106	0.2202	0.1667	0.1667	0.3333
0.5000	0.5000	0.3989	0.3798	0.0107	0.2213	0.6667	0.5000	0.3333
0.2000	0.2000	0.1307	0.3507	0.0322	0.3771	0.1667	0.1667	0.3333
0.3000	0.3000	0.2137	0.1122	0.0032	0.0978	0.0000	0.0000	0.0000
0.3000	0.3000	0.1670	0.3013	0.2196	0.7195	1.0000	1.0000	0.3333
0.4000	0.4000	0.3120	0.2766	0.0103	0.2166	0.6667	0.3333	0.3333
0.3000	0.3000	0.4931	0.3321	0.0067	0.1655	0.5000	0.5000	0.0000
0.4000	0.4000	0.3287	0.3507	0.0073	0.1742	1.0000	1.0000	0.3333
0.4000	0.4000	0.0388	0.0825	0.1241	0.6188	0.5000	0.5000	0.0000
0.5000	0.5000	0.4931	0.3241	0.0158	0.2746	1.0000	0.5000	1.0000
0.2000	0.2000	0.3190	0.1122	0.0002	0.0081	0.1667	0.5000	0.5000
0.5000	0.5000	0.3631	0.2656	0.0103	0.2166	1.0000	0.5000	0.5000
0.6000	0.6000	0.5223	0.3241	0.0244	0.3392	0.5000	0.5000	0.0000
0.3000	0.3000	0.2137	0.3710	0.0032	0.0978	1.0000	1.0000	0.3333
0.4000	0.4000	0.4563	0.3697	0.0146	0.2660	0.6667	0.5000	0.5000
0.4000	0.4000	0.4576	0.3600	0.2196	0.7183	0.6667	0.5000	0.3333
0.4000	0.4000	0.4253	0.3507	0.0662	0.5048	0.6667	0.5000	0.3333
0.4000	0.4000	0.1541	0.1312	0.2194	0.7118	1.0000	1.0000	0.0000
0.2000	0.2000	0.3190	0.1122	0.0003	0.0114	0.1667	0.5000	0.5000
0.4000	0.4000	0.5114	0.4470	0.0220	0.3262	1.0000	1.0000	0.3333
0.6000	0.6000	0.5507	0.4381	0.0266	0.3560	1.0000	1.0000	0.3333
0.6000	0.6000	0.2049	0.3529	0.0123	0.2415	1.0000	1.0000	0.3333
0.7000	0.7000	0.6615	0.4138	0.0220	0.3262	1.0000	1.0000	0.5000
0.7000	0.7000	0.5114	0.4138	0.0220	0.3262	1.0000	1.0000	0.3333
0.7000	0.7000	0.6615	0.4138	0.0220	0.3262	1.0000	1.0000	0.3333
0.6000	0.6000	0.6615	0.3773	0.0123	0.2415	1.0000	1.0000	0.3333
0.6000	0.6000	0.6615	0.3773	0.0123	0.2415	1.0000	1.0000	0.5000
0.4000	0.4000	0.6672	0.4470	0.0549	0.4756	0.6667	0.5000	0.3333
0.7000	0.7000	0.5362	0.4184	0.0549	0.4756	1.0000	1.0000	0.3333
0.5000	0.5000	0.6672	0.4470	0.2197	0.7218	0.6667	0.5000	0.5000
0.6000	0.6000	0.5994	0.4083	0.0071	0.1740	0.6667	0.5000	0.5000
0.3000	0.3000	0.4844	0.2605	0.0123	0.2390	0.6667	0.3333	0.5000
0.4000	0.4000	0.3592	0.2992	0.0123	0.2390	0.6667	0.3333	0.5000
0.3000	0.3000	0.2461	0.2681	0.0121	0.2318	0.6667	0.3333	0.3333
0.5000	0.5000	0.5193	0.5779	0.2197	0.7218	0.6667	0.5000	0.5000
0.6000	0.6000	0.3895	0.4598	0.0123	0.2390	0.6667	0.5000	0.5000
0.2000	0.2000	0.1039	0.2036	0.0095	0.1865	0.6667	0.5000	0.3333
0.4000	0.4000	0.3840	0.2681	0.0123	0.2390	0.6667	0.5000	0.5000
0.4000	0.4000	0.4844	0.2681	0.0123	0.2390	0.6667	0.5000	0.5000
0.4000	0.4000	0.3483	0.2681	0.0121	0.2318	0.6667	0.5000	0.5000
0.4000	0.4000	0.4383	0.4138	0.1237	0.6008	0.6667	0.5000	0.3333
0.4000	0.4000	0.4576	0.3976	0.2194	0.7118	0.6667	0.5000	0.3333
0.4000	0.4000	0.1841	0.0927	0.0054	0.1426	0.5000	0.5000	0.5000
0.4000	0.4000	0.2550	0.2681	0.0123	0.2390	0.6667	0.5000	0.3333
0.6000	0.6000	0.4372	0.3700	0.0103	0.2166	0.6667	0.5000	0.3333
0.6000	0.6000	0.4658	0.5779	0.0508	0.4591	0.6667	0.5000	0.5000
0.3000	0.3000	0.3744	0.2312	0.2192	0.7043	0.6667	0.5000	0.3333
0.6000	0.6000	0.4844	0.3209	0.0123	0.2390	0.6667	0.5000	0.5000
0.3000	0.3000	0.4844	0.2681	0.0123	0.2390	0.6667	0.3333	0.5000
0.5000	0.5000	0.5193	0.4138	0.0509	0.4627	1.0000	1.0000	0.5000
0.4000	0.4000	0.3731	0.2992	0.0111	0.2255	0.6667	0.5000	0.3333
0.4000	0.4000	0.3597	0.0751	0.0054	0.1426	0.6667	0.5000	0.5000
0.4000	0.4000	0.4099	0.2191	0.0068	0.1663	0.6667	0.5000	0.5000
0.3000	0.3000	0.3900	0.2434	0.2194	0.7118	0.6667	0.5000	0.5000
0.1000	0.1000	0.1640	0.1318	0.0065	0.1576	0.6667	0.5000	0.3333
0.3000	0.3000	0.6672	0.3930	0.2197	0.7218	0.6667	0.5000	0.5000
0.4000	0.4000	0.4099	0.2191	0.0144	0.2553	0.6667	0.5000	0.5000
0.2000	0.2000	0.3336	0.6219	0.0754	0.5035	0.1667	0.5000	0.5000
0.3000	0.3000	0.4099	0.2677	0.2194	0.7111	0.1667	0.5000	0.5000
0.3000	0.3000	0.4176	0.2434	0.2191	0.7007	0.1667	0.5000	0.5000
0.3000	0.3000	0.3744	0.2923	0.2194	0.7111	0.6667	0.5000	0.5000
0.2000	0.2000	0.2550	0.2491	0.0054	0.1426	0.6667	0.5000	0.3333
0.5000	0.5000	0.8521	0.8763	0.0127	0.2468	0.6667	0.5000	0.5000
0.4000	0.4000	0.4099	0.2069	0.0066	0.1591	0.1667	0.5000	0.5000
0.2000	0.2000	0.2904	0.0000	0.0732	0.4379	0.0000	0.5000	0.5000
0.5000	0.5000	0.3745	0.2021	0.0103	0.2166	1.0000	1.0000	0.5000
0.4000	0.4000	0.4361	0.3117	0.0107	0.2205	0.6667	0.5000	0.5000
0.4000	0.4000	0.3745	0.1531	0.0107	0.2205	0.6667	0.5000	0.5000
0.2000	0.2000	0.2635	0.1122	0.0120	0.2237	0.1667	0.5000	0.5000
0.2000	0.2000	0.2635	0.1122	0.0120	0.2237	0.1667	0.5000	0.5000
0.1000	0.1000	0.2234	#NAME?	0.0000	0.0000	0.0000	0.0000	0.0000
0.3000	0.3000	0.8521	0.8763	0.0118	0.2158	0.1667	0.5000	0.5000
0.2000	0.2000	0.4376	0.0375	0.0123	0.2341	0.0000	0.5000	0.5000
0.3000	0.3000	0.7441	0.8763	0.0120	0.2237	0.1667	0.5000	0.5000

0.5000	0.5000	0.3744	0.3038	0.2196	0.7183	1.0000	1.0000	1.0000
0.5000	0.5000	0.3745	0.2465	0.0506	0.4520	0.6667	0.5000	0.5000
0.5000	0.5000	0.5193	0.4260	0.2197	0.7218	0.6667	0.5000	0.5000
0.4000	0.4000	0.5008	0.4598	0.0121	0.2318	0.6667	0.5000	0.5000
0.3000	0.3000	0.3985	0.4598	0.0114	0.2088	1.0000	0.3333	1.0000
0.4000	0.4000	0.2776	0.3862	0.0083	0.1896	1.0000	0.5000	1.0000
0.5000	0.5000	0.4578	0.3486	0.0103	0.2166	0.6667	0.5000	0.5000
0.3000	0.3000	0.2040	0.2681	0.0114	0.2088	0.6667	0.3333	0.3333
0.6000	0.6000	0.3509	0.2681	0.0121	0.2318	0.6667	0.5000	0.3333
0.6000	0.6000	0.4844	0.2681	0.0118	0.2205	0.6667	0.5000	0.5000
0.5000	0.5000	0.7904	0.7641	0.2194	0.7111	0.5000	0.5000	0.5000
0.6000	0.6000	0.2188	0.1652	0.0103	0.2110	0.6667	0.5000	0.3333
0.3000	0.3000	0.2003	0.1468	0.0104	0.2158	0.6667	0.5000	0.3333
0.2000	0.2000	0.1630	0.1468	0.0103	0.2110	0.6667	0.5000	0.3333
0.3000	0.3000	0.1895	0.1468	0.0106	0.2216	0.1667	0.1667	0.3333
0.3000	0.3000	0.2003	0.1652	0.0071	0.1662	0.6667	0.5000	0.3333
0.3000	0.3000	0.1895	0.1468	0.0101	0.2014	0.1667	0.1667	0.3333
0.3000	0.3000	0.1895	0.1468	0.0106	0.2216	0.1667	0.1667	0.3333
0.2000	0.2000	0.1522	0.1468	0.0104	0.2158	0.1667	0.1667	0.3333
0.3000	0.3000	0.1895	0.1468	0.0106	0.2216	0.1667	0.1667	0.3333
0.3000	0.3000	0.1895	0.1468	0.0101	0.2014	0.1667	0.1667	0.3333
0.3000	0.3000	0.1895	0.0920	0.0101	0.2014	0.1667	0.1667	0.3333
0.3000	0.3000	0.1895	0.1468	0.0031	0.0934	0.1667	0.1667	0.3333
0.3000	0.3000	0.1522	0.1468	0.0105	0.2171	0.1667	0.1667	0.3333
0.3000	0.3000	0.1895	0.1652	0.0105	0.2171	0.6667	0.5000	0.3333
0.3000	0.3000	0.1895	0.1258	0.0104	0.2158	0.5000	0.6667	0.3333
0.4000	0.4000	0.2008	0.0920	0.0041	0.1142	0.6667	0.5000	0.3333
0.6000	0.6000	0.2188	0.0920	0.0030	0.0886	0.5000	0.5000	0.0000
0.3000	0.3000	0.2003	0.1468	0.0033	0.0992	0.6667	0.5000	0.3333
0.6000	0.6000	0.2188	0.4666	0.0465	0.4285	0.1667	0.1667	0.3333
0.3000	0.3000	0.2003	0.1468	0.0103	0.2110	0.6667	0.5000	0.3333
0.3000	0.3000	0.1895	0.1468	0.0101	0.2014	0.1667	0.1667	0.3333
0.2000	0.2000	0.1895	0.1468	0.0104	0.2158	0.5000	0.6667	0.3333
0.3000	0.3000	0.1522	0.1468	0.0096	0.1865	0.5000	0.6667	0.3333
0.2000	0.2000	0.1522	0.1652	0.0106	0.2216	1.0000	1.0000	0.3333
0.3000	0.3000	0.1895	0.1468	0.0105	0.2171	0.1667	0.1667	0.3333
0.3000	0.3000	0.1895	0.1652	0.0072	0.1722	0.6667	0.5000	0.3333
0.1000	0.1000	0.1522	0.1468	0.0096	0.1865	0.5000	0.6667	0.3333
0.3000	0.3000	0.1895	0.1468	0.0101	0.2014	0.1667	0.1667	0.3333
0.2000	0.2000	0.1895	0.1468	0.0105	0.2171	0.5000	0.6667	0.3333
0.2000	0.2000	0.1522	0.1468	0.0023	0.0641	0.1667	0.1667	0.3333
0.3000	0.3000	0.2666	0.1652	0.0101	0.2014	0.6667	0.5000	0.3333
0.3000	0.3000	0.2003	0.1468	0.0060	0.1485	0.6667	0.5000	0.3333
0.3000	0.3000	0.1895	0.1468	0.0101	0.2014	0.1667	0.1667	0.3333
0.5000	0.5000	0.1815	0.1468	0.0020	0.0543	0.1667	0.1667	0.3333
0.6000	0.6000	0.2188	0.1468	0.0060	0.1485	0.6667	0.5000	0.3333
0.3000	0.3000	0.1895	0.1468	0.0105	0.2171	0.1667	0.1667	0.3333
0.2000	0.2000	0.1522	0.1652	0.0097	0.1887	1.0000	1.0000	0.3333
0.3000	0.3000	0.1522	0.1468	0.0105	0.2171	0.5000	0.6667	0.3333
0.3000	0.3000	0.1522	0.1468	0.0097	0.1887	0.1667	0.1667	0.3333
0.4000	0.4000	0.2008	0.1468	0.0097	0.1887	0.5000	0.6667	0.3333
0.3000	0.3000	0.2008	0.1468	0.0090	0.1655	0.1667	0.1667	0.3333
0.3000	0.3000	0.1895	0.1468	0.0105	0.2171	0.5000	0.6667	0.3333
0.3000	0.3000	0.1522	0.1468	0.0105	0.2171	0.5000	0.6667	0.3333
0.6000	0.6000	0.1815	0.1468	0.0106	0.2216	0.5000	0.6667	0.3333
0.5000	0.5000	0.2188	0.1468	0.0105	0.2171	0.5000	0.6667	0.3333
0.3000	0.3000	0.1895	0.1468	0.0101	0.2014	0.5000	0.6667	0.3333
0.1000	0.1000	0.1522	0.1468	0.0096	0.1865	0.5000	0.6667	0.3333
0.3000	0.3000	0.2003	0.1468	0.0030	0.0886	1.0000	1.0000	0.3333
0.6000	0.6000	0.2188	0.1468	0.0106	0.2216	0.1667	0.1667	0.3333
0.3000	0.3000	0.1895	0.0920	0.0106	0.2216	0.1667	0.1667	0.3333
0.3000	0.3000	0.1895	0.1468	0.0101	0.2014	0.5000	0.6667	0.3333
0.4000	0.4000	0.1635	0.1468	0.0035	0.0919	0.5000	0.6667	0.3333
0.4000	0.4000	0.1037	0.0528	0.0105	0.2171	0.1667	0.1667	0.3333
0.2000	0.2000	0.0560	0.0289	0.0097	0.1887	0.5000	0.6667	0.3333
0.2000	0.2000	0.1522	0.1468	0.0097	0.1887	0.5000	0.6667	0.3333
0.2000	0.2000	0.1895	0.1468	0.0097	0.1887	0.1667	0.1667	0.3333
0.2000	0.2000	0.1522	0.1468	0.0105	0.2171	0.5000	0.6667	0.3333
0.2000	0.2000	0.1522	0.1468	0.0097	0.1887	0.5000	0.6667	0.3333
0.2000	0.2000	0.1522	0.1468	0.0105	0.2171	0.5000	0.6667	0.3333
0.3000	0.3000	0.1895	0.0920	0.0101	0.2014	0.5000	0.6667	0.3333
0.0000	0.0000	0.1522	0.1468	0.0016	0.0431	0.1667	0.1667	0.3333
0.6000	0.6000	0.2003	0.1468	0.0030	0.0886	0.6667	0.5000	0.3333
0.0000	0.0000	0.1630	0.1468	0.0030	0.0886	0.6667	0.5000	0.3333
0.3000	0.3000	0.2003	0.1468	0.0030	0.0886	0.6667	0.5000	0.3333
0.2000	0.2000	0.1895	0.1652	0.0064	0.1417	1.0000	1.0000	0.3333
0.0000	0.0000	0.1522	0.1468	0.0016	0.0431	0.1667	0.1667	0.3333

0.0000	0.0000	0.1522	0.1468	0.0016	0.0431	0.1667	0.1667	0.3333
0.3000	0.3000	0.0293	0.0534	0.0020	0.0543	0.0000	0.0000	0.0000
0.3000	0.3000	0.1895	0.1652	0.0068	0.1565	0.6667	0.5000	0.3333
0.2000	0.2000	0.1522	0.3875	0.0016	0.0431	0.6667	0.5000	0.3333
0.6000	0.6000	0.2188	0.3875	0.0027	0.0789	0.6667	0.5000	0.3333
0.5000	0.5000	0.1815	0.2157	0.0024	0.0663	0.5000	0.6667	0.3333
0.0000	0.0000	0.1522	0.1468	0.0016	0.0431	0.1667	0.1667	0.3333
0.6000	0.6000	0.4405	0.4301	0.0068	0.1565	0.6667	0.5000	0.3333
0.6000	0.6000	0.4405	0.1652	0.0068	0.1565	1.0000	1.0000	0.3333
0.4000	0.4000	0.2364	0.4666	0.0468	0.4382	0.6667	0.5000	0.3333
0.4000	0.4000	0.1895	0.5355	0.0465	0.4285	0.5000	0.6667	0.3333
0.6000	0.6000	0.1815	0.1468	0.0105	0.2171	1.0000	1.0000	0.3333
0.5000	0.5000	0.2111	0.4788	0.0101	0.2014	1.0000	1.0000	0.3333
0.2000	0.2000	0.1522	0.1468	0.0023	0.0632	0.5000	0.6667	0.3333
0.0000	0.0000	0.1433	0.2719	0.0210	0.2412	0.0000	0.5000	0.5000
0.0000	0.0000	0.1433	0.2719	0.0210	0.2412	0.0000	0.5000	0.5000
0.3000	0.3000	0.0293	0.0534	0.0020	0.0543	0.0000	0.0000	0.0000
0.6000	0.6000	0.2188	0.2185	0.0237	0.3202	0.0000	0.5000	0.5000
0.3000	0.3000	0.1333	0.1223	0.0020	0.0543	0.5000	0.5000	0.0000
0.5000	0.5000	0.0960	0.0000	0.0005	0.0210	0.5000	0.5000	0.0000
0.3000	0.3000	0.1895	0.0920	0.0027	0.0789	0.0000	0.0000	0.0000
#NAME?	#NAME?	0.0000	0.0000	0.0000	0.0000	0.0000	0.0000	0.0000
0.3000	0.3000	0.1815	0.1468	0.0020	0.0543	0.1667	0.1667	0.3333
0.0000	0.0000	0.0000	0.0000	0.0000	0.0000	0.0000	0.0000	0.0000
0.0000	0.0000	0.0000	0.0000	0.0000	0.0000	0.0000	0.0000	0.0000
0.5000	0.5000	0.1815	0.1468	0.0079	0.1789	1.0000	1.0000	0.3333
0.0000	0.0000	0.0000	0.0000	0.0000	0.0000	0.0000	0.0000	0.0000
0.3000	0.3000	0.0293	0.0534	0.0020	0.0543	0.0000	0.0000	0.0000
0.4000	0.4000	0.1895	0.1652	0.0073	0.1768	1.0000	1.0000	0.0000
0.3000	0.3000	0.0732	0.0616	0.0021	0.0703	1.0000	1.0000	0.0000
0.0000	0.0000	0.0108	0.0277	0.0030	0.0886	0.5000	0.5000	0.0000
0.0000	0.0000	0.1522	0.1468	0.0016	0.0431	0.1667	0.1667	0.3333
0.0000	0.0000	0.0000	0.0000	0.0000	0.0000	0.0000	0.0000	0.0000
0.2000	0.2000	0.1656	0.4132	0.0458	0.4038	0.1667	0.1667	0.3333
0.3000	0.3000	0.0293	0.0534	0.0020	0.0543	0.0000	0.0000	0.0000
0.0000	0.0000	0.0000	0.0000	0.0000	0.0000	0.0000	0.0000	0.0000
0.0000	0.0000	0.0000	0.0000	0.0000	0.0000	0.0000	0.0000	0.0000
0.6000	0.6000	0.2188	0.0920	0.0027	0.0789	0.0000	0.0000	0.0000
0.0000	0.0000	0.0000	0.0000	0.0000	0.0000	0.0000	0.0000	0.0000
0.0000	0.0000	0.0000	0.0000	0.0000	0.0000	0.0000	0.0000	0.0000
0.6000	0.6000	0.1349	0.1947	0.0099	0.1996	0.5000	0.5000	0.0000
0.0000	0.0000	0.0000	0.0000	0.0000	0.0000	0.0000	0.0000	0.0000
0.3000	0.3000	0.2682	0.1947	0.0102	0.2093	1.0000	1.0000	0.0000
0.6000	0.6000	0.2682	0.4301	0.0099	0.1996	0.6667	0.5000	0.3333
0.3000	0.3000	0.2666	0.3507	0.0049	0.1296	0.1667	0.1667	0.3333
0.2000	0.2000	0.1307	0.3507	0.0030	0.0893	0.1667	0.1667	0.3333
0.3000	0.3000	0.1895	0.0920	0.0027	0.0789	0.0000	0.0000	0.0000
0.6000	0.6000	0.2389	0.1258	0.0099	0.1996	0.0000	0.0000	0.0000
0.6000	0.6000	0.2682	0.0724	0.0099	0.1996	0.0000	0.0000	0.0000
0.0000	0.0000	0.2682	0.0934	0.0092	0.1750	0.1667	0.1667	0.3333
0.6000	0.6000	0.2682	0.3408	0.0242	0.3412	0.5000	1.0000	0.5000
0.6000	0.6000	0.2188	0.2973	0.0018	0.0581	0.1667	0.1667	0.3333
0.2000	0.2000	0.3914	0.4196	0.0037	0.1089	0.1667	0.1667	0.3333
0.4000	0.4000	0.3946	0.4196	0.0055	0.1461	0.6667	0.5000	0.3333
0.4000	0.4000	0.3636	0.4196	0.0325	0.3879	0.5000	0.6667	0.3333
0.4000	0.4000	0.3946	0.4196	0.0106	0.2224	0.6667	0.5000	0.3333
0.4000	0.4000	0.1638	0.3344	0.0105	0.2190	0.6667	0.5000	0.3333
0.6000	0.6000	0.3636	0.1704	0.0021	0.0688	0.0000	0.0000	0.0000
0.1000	0.1000	0.3636	0.1704	0.0021	0.0688	0.0000	0.0000	0.0000
0.6000	0.6000	0.2188	0.1631	0.0049	0.1250	0.0000	0.0000	0.0000
0.6000	0.6000	0.2188	0.1631	0.0049	0.1250	0.0000	0.0000	0.0000
0.1000	0.1000	0.2003	0.1245	0.0054	0.1432	0.5000	0.5000	0.0000
0.0000	0.0000	0.1079	0.0237	0.0000	0.0015	0.0000	0.0000	0.0000
0.1000	0.1000	0.3636	0.1765	0.0325	0.3879	0.0000	0.0000	0.0000
0.1000	0.1000	0.1772	0.0969	0.0319	0.3668	0.5000	0.6667	0.3333
0.1000	0.1000	0.1495	0.0787	0.0319	0.3668	0.0000	0.0000	0.0000
0.3000	0.3000	0.1671	0.2588	0.0036	0.1064	1.0000	1.0000	0.3333
0.5000	0.5000	0.2176	0.3344	0.0105	0.2189	1.0000	1.0000	0.5000
0.3000	0.3000	0.2018	0.2249	0.0015	0.0520	0.6667	0.5000	0.3333
0.1000	0.1000	0.1495	0.3218	0.0060	0.1492	0.1667	0.1667	0.3333
0.2000	0.2000	0.1495	0.3344	0.0322	0.3771	0.5000	0.6667	0.3333
0.1000	0.1000	0.1271	0.0156	0.0319	0.3668	0.0000	0.0000	0.0000
0.2000	0.2000	0.1612	0.1095	0.0319	0.3668	0.1667	0.1667	0.3333
0.2000	0.2000	0.0277	0.0339	0.0031	0.0878	0.1667	0.1667	0.3333
0.1000	0.1000	0.1549	0.0339	0.0319	0.3668	0.1667	0.1667	0.3333
0.6000	0.6000	0.2188	0.0542	0.0319	0.3668	0.0000	0.0000	0.0000

0.1000	0.1000	0.1271	0.0156	0.0319	0.3668	0.0000	0.0000	0.0000
0.0000	0.0000	0.0000	0.0000	0.0000	0.0000	0.0000	0.0000	0.0000
0.0000	0.0000	0.1386	0.2795	0.0303	0.3167	0.0000	0.0000	0.0000
0.1000	0.1000	0.1495	0.0787	0.0319	0.3668	0.0000	0.0000	0.0000
0.0000	0.0000	0.0000	0.0000	0.0000	0.0000	0.0000	0.0000	0.0000
0.1000	0.1000	0.3636	0.4196	0.0325	0.3879	0.5000	0.6667	0.3333
0.2000	0.2000	0.3636	0.4196	0.0325	0.3879	0.5000	0.6667	0.3333
0.2000	0.2000	0.3914	0.4196	0.0325	0.3879	0.5000	0.6667	0.3333
0.1000	0.1000	0.3636	0.1609	0.0082	0.1903	0.5000	0.5000	0.0000
0.1000	0.1000	0.0589	0.2588	0.0079	0.1796	0.5000	0.6667	0.3333
0.1000	0.1000	0.0855	0.1726	0.0077	0.1727	0.5000	0.5000	0.0000
0.5000	0.5000	0.1772	0.4196	0.0322	0.3771	1.0000	1.0000	0.3333
0.2000	0.2000	0.1864	0.3344	0.0103	0.2117	0.1667	0.1667	0.3333
0.2000	0.2000	0.1549	0.4196	0.0322	0.3771	0.5000	0.6667	0.3333
0.6000	0.6000	0.2188	0.3773	0.0320	0.3703	0.5000	0.6667	0.3333
0.6000	0.6000	0.2188	0.3773	0.0320	0.3703	0.5000	0.6667	0.3333
0.2000	0.2000	0.1335	0.1765	0.0320	0.3703	0.5000	0.6667	0.3333
0.2000	0.2000	0.1271	0.1765	0.0320	0.3703	0.5000	0.6667	0.3333
0.2000	0.2000	0.1271	0.4196	0.0322	0.3771	0.5000	0.6667	0.3333
0.2000	0.2000	0.1271	0.4196	0.0322	0.3771	0.5000	0.6667	0.3333
0.3000	0.3000	0.1549	0.2588	0.0322	0.3771	1.0000	1.0000	0.3333
0.2000	0.2000	0.1528	0.4196	0.0125	0.2360	1.0000	1.0000	0.3333
0.3000	0.3000	0.3914	0.4196	0.0127	0.2468	1.0000	1.0000	0.3333
0.2000	0.2000	0.1271	0.2588	0.0322	0.3771	1.0000	1.0000	0.3333
0.2000	0.2000	0.1357	0.2588	0.0125	0.2360	1.0000	1.0000	0.3333
0.2000	0.2000	0.1528	0.2588	0.0322	0.3771	1.0000	1.0000	0.3333
0.2000	0.2000	0.1335	0.4196	0.0322	0.3771	1.0000	1.0000	0.3333
0.2000	0.2000	0.2365	0.3101	0.0322	0.3771	1.0000	1.0000	0.3333
0.2000	0.2000	0.3636	0.4196	0.0325	0.3879	1.0000	1.0000	0.3333
0.2000	0.2000	0.2003	0.4196	0.0322	0.3771	1.0000	1.0000	0.3333
0.2000	0.2000	0.1495	0.4196	0.0322	0.3771	1.0000	1.0000	0.3333
0.5000	0.5000	0.1528	0.3218	0.0322	0.3771	1.0000	1.0000	0.3333
0.2000	0.2000	0.1805	0.2576	0.0321	0.3738	0.1667	0.1667	0.3333
0.2000	0.2000	0.1528	0.3344	0.0322	0.3771	0.5000	0.6667	0.3333
0.1000	0.1000	0.2643	0.0852	0.0320	0.3703	0.1667	0.1667	0.3333
0.0000	0.0000	0.0578	0.1765	0.0304	0.3190	0.5000	0.5000	0.0000
0.4000	0.4000	0.2227	0.1305	0.0101	0.2014	0.1667	0.1667	0.3333
0.5000	0.5000	0.2318	0.1704	0.0024	0.0731	0.5000	0.5000	0.0000
0.6000	0.6000	0.2318	0.2157	0.0024	0.0731	0.5000	0.6667	0.3333
0.0000	0.0000	#NAME?	#NAME?	0.0000	0.0000	0.0000	0.0000	0.0000
0.5000	0.5000	0.1815	0.2157	0.0015	0.0413	0.5000	0.6667	0.3333
0.5000	0.5000	0.1854	0.2157	0.0312	0.3436	0.5000	0.6667	0.3333
0.6000	0.6000	0.2227	0.2157	0.0319	0.3668	0.5000	0.6667	0.3333
0.5000	0.5000	0.0693	0.0245	0.0009	0.0253	0.0000	0.0000	0.0000
0.4000	0.4000	0.2227	0.0338	0.0015	0.0477	0.0000	0.0000	0.0000
0.2000	0.2000	0.0039	0.0143	0.0000	0.0000	0.0000	0.0000	0.0000
0.6000	0.6000	0.2227	0.0609	0.0057	0.1388	0.0000	0.0000	0.0000
0.4000	0.4000	0.2227	0.0242	0.0000	0.0000	0.0000	0.0000	0.0000
0.4000	0.4000	0.2227	0.0399	0.0319	0.3668	0.0000	0.0000	0.0000
0.0000	0.0000	0.0000	0.0000	#NAME?	#NAME?	0.0000	0.0000	0.0000
0.0000	0.0000	0.0000	0.0000	#NAME?	#NAME?	0.0000	0.0000	0.0000
0.6000	0.6000	0.2188	0.0385	0.0007	0.0247	0.0000	0.0000	0.0000
0.0000	0.0000	0.0000	0.0000	0.0000	0.0000	0.0000	0.0000	0.0000
0.0000	0.0000	0.0000	0.0000	#NAME?	#NAME?	0.0000	0.0000	0.0000
0.4000	0.4000	0.2227	0.1488	0.0049	0.1250	0.0000	0.0000	0.0000
0.4000	0.4000	0.2227	0.2830	0.0322	0.3771	0.1667	0.1667	0.3333
0.6000	0.6000	0.2227	0.0669	0.0319	0.3668	0.0000	0.0000	0.0000
0.3000	0.3000	0.1638	0.4196	0.0025	0.0764	0.5000	0.6667	0.3333
0.1000	0.1000	0.1401	0.1150	0.0321	0.3739	0.0000	0.0000	0.0000
0.2000	0.2000	0.3766	0.4196	0.0073	0.1776	1.0000	1.0000	0.3333
0.5000	0.5000	0.2356	0.4564	0.0124	0.2328	1.0000	1.0000	0.0000
0.1000	0.1000	0.1271	0.4196	0.0322	0.3771	0.5000	0.6667	0.3333
0.0000	0.0000	0.0000	0.0000	0.0010	0.0229	0.0000	0.0000	0.0000
0.1000	0.1000	0.3636	0.4196	0.0325	0.3879	0.5000	0.6667	0.3333
0.1000	0.1000	0.1528	0.4196	0.0322	0.3771	0.5000	0.6667	0.3333
0.6000	0.6000	0.2188	0.4196	0.0324	0.3849	0.5000	0.6667	0.3333
0.6000	0.6000	0.2227	0.4196	0.0324	0.3849	0.5000	0.6667	0.3333
0.4000	0.4000	0.2227	0.0876	0.0319	0.3668	0.0000	0.0000	0.0000
0.3000	0.3000	0.4792	0.4196	0.0082	0.1903	0.5000	0.6667	0.3333
0.6000	0.6000	0.2188	0.4196	0.0322	0.3771	0.5000	0.6667	0.3333
0.6000	0.6000	0.2188	0.0542	0.0319	0.3668	0.0000	0.0000	0.0000
0.6000	0.6000	0.2188	0.0542	0.0319	0.3668	0.5000	0.5000	0.0000
0.5000	0.5000	0.2188	0.0787	0.0312	0.3421	0.5000	0.5000	0.0000
0.3000	0.3000	0.0955	0.0399	0.0243	0.1975	0.5000	0.5000	0.0000
0.6000	0.6000	0.2227	0.1765	0.0319	0.3668	0.5000	0.5000	0.0000

0.5000	0.5000	0.2188	0.1725	0.0050	0.1141	0.5000	0.5000	0.0000
0.5000	0.5000	0.2188	0.0609	0.0050	0.1141	0.0000	0.0000	0.0000
0.0000	0.0000	0.0000	0.0000	0.0000	0.0000	0.0000	0.0000	0.0000
0.2000	0.2000	0.1117	0.0237	0.0000	0.0015	0.0000	0.0000	0.0000
0.6000	0.6000	0.1149	0.1631	0.0043	0.1019	0.0000	0.0000	0.0000
0.3000	0.3000	0.2227	0.0609	0.0050	0.1156	0.0000	0.0000	0.0000
0.5000	0.5000	0.1043	0.3064	0.0015	0.0509	0.1667	0.1667	0.3333
0.5000	0.5000	0.1117	0.0542	0.0312	0.3436	0.0000	0.0000	0.0000
0.6000	0.6000	0.2227	0.0385	0.0007	0.0247	0.0000	0.0000	0.0000
0.0000	0.0000	0.1079	0.0237	0.0000	0.0015	0.0000	0.0000	0.0000
0.0000	0.0000	0.1333	0.1223	0.0015	0.0413	0.5000	0.5000	0.0000
0.0000	0.0000	0.1117	0.0094	0.0000	0.0000	0.0000	0.0000	0.0000
0.0000	0.0000	0.1117	0.0094	0.0000	0.0000	0.0000	0.0000	0.0000
0.6000	0.6000	0.2227	0.0513	0.0057	0.1388	0.0000	0.0000	0.0000
0.6000	0.6000	0.2227	0.2157	0.0057	0.1388	0.5000	0.6667	0.3333
0.6000	0.6000	0.5823	0.4470	0.0104	0.2202	1.0000	0.5000	1.0000
0.6000	0.6000	0.5823	0.4470	0.0104	0.2202	1.0000	0.5000	1.0000
0.6000	0.6000	0.5823	0.4470	0.0104	0.2202	1.0000	0.5000	1.0000
0.5000	0.5000	0.6348	0.6387	0.0104	0.2202	1.0000	0.5000	1.0000
0.5000	0.5000	0.6348	0.4990	0.0190	0.3042	1.0000	0.5000	1.0000
0.5000	0.5000	0.6348	0.4990	0.0190	0.3042	0.6667	0.5000	0.5000
0.6000	0.6000	0.5362	0.4990	0.0199	0.3108	0.6667	0.5000	0.5000
0.6000	0.6000	0.5823	0.4722	0.0106	0.2219	0.6667	0.5000	0.5000
0.5000	0.5000	0.5362	0.4722	0.0106	0.2219	1.0000	1.0000	0.5000
0.6000	0.6000	0.5994	0.4722	0.0663	0.5082	0.6667	0.5000	0.5000
0.6000	0.6000	0.5994	0.4722	0.0663	0.5082	1.0000	1.0000	0.5000
0.5000	0.5000	0.5994	0.4746	0.0663	0.5082	1.0000	1.0000	0.5000
0.6000	0.6000	0.5994	0.4722	0.0663	0.5082	0.6667	0.5000	0.5000
0.6000	0.6000	0.5823	0.4746	0.0121	0.2397	1.0000	1.0000	0.5000
0.6000	0.6000	0.5362	0.4990	0.0293	0.3713	1.0000	0.5000	1.0000
0.6000	0.6000	0.4658	0.5779	0.0658	0.4873	1.0000	0.5000	1.0000
0.5000	0.5000	0.8327	0.4990	0.0663	0.5082	1.0000	0.5000	1.0000
0.6000	0.6000	0.6848	0.5779	0.0102	0.2102	1.0000	0.5000	1.0000
0.6000	0.6000	0.4658	0.5779	0.0187	0.2909	1.0000	0.5000	1.0000
0.5000	0.5000	0.5362	0.4470	0.0199	0.3108	0.6667	0.5000	0.5000
0.5000	0.5000	0.5994	0.4470	0.0663	0.5082	1.0000	0.5000	1.0000
0.5000	0.5000	0.4515	0.4381	0.0660	0.4982	0.6667	0.5000	0.5000
0.6000	0.6000	0.5994	0.4990	0.0663	0.5082	0.6667	0.5000	0.5000
0.6000	0.6000	0.3922	0.4381	0.0288	0.3504	1.0000	0.5000	1.0000
0.6000	0.6000	0.5362	0.4722	0.0106	0.2219	0.6667	0.5000	0.5000
0.6000	0.6000	0.5362	0.4722	0.0106	0.2219	0.6667	0.5000	0.5000
0.6000	0.6000	0.5362	0.4722	0.0106	0.2219	0.6667	0.5000	0.5000
0.6000	0.6000	0.5994	0.4722	0.0663	0.5082	1.0000	1.0000	0.5000
0.6000	0.6000	0.5994	0.4722	0.0663	0.5082	1.0000	1.0000	0.5000
0.5000	0.5000	0.5362	0.4722	0.0110	0.2272	1.0000	1.0000	1.0000
0.5000	0.5000	0.5362	0.4470	0.0106	0.2219	1.0000	1.0000	0.3333
0.5000	0.5000	0.5362	0.4722	0.0104	0.2202	0.6667	0.5000	0.5000
0.5000	0.5000	0.5362	0.4722	0.0104	0.2202	0.6667	0.5000	0.5000
0.5000	0.5000	0.6615	0.4722	0.0106	0.2219	1.0000	1.0000	0.3333
0.5000	0.5000	0.5823	0.4722	0.0106	0.2219	0.6667	0.5000	0.3333
0.5000	0.5000	0.5823	0.4722	0.0104	0.2202	1.0000	1.0000	0.5000
0.5000	0.5000	0.6615	0.4722	0.0106	0.2219	1.0000	1.0000	0.3333
0.5000	0.5000	0.5362	0.4722	0.0106	0.2219	1.0000	1.0000	0.3333
0.5000	0.5000	0.5823	0.4722	0.0104	0.2202	1.0000	1.0000	0.5000
0.4000	0.4000	0.5362	0.4915	0.0078	0.1841	1.0000	0.5000	1.0000
0.5000	0.5000	0.6615	0.4722	0.0094	0.2067	1.0000	1.0000	0.5000
0.5000	0.5000	0.6615	0.4722	0.0663	0.5082	1.0000	1.0000	0.5000
0.6000	0.6000	0.6615	0.4722	0.0663	0.5082	1.0000	0.5000	1.0000
0.6000	0.6000	0.6615	0.4722	0.0663	0.5082	1.0000	1.0000	1.0000
0.7000	0.7000	0.6615	0.4722	0.0106	0.2219	1.0000	1.0000	0.5000
0.6000	0.6000	0.5295	0.4722	0.0104	0.2202	1.0000	1.0000	0.5000
0.4000	0.4000	0.5362	0.4722	0.0094	0.2067	1.0000	1.0000	0.3333
0.5000	0.5000	0.5994	0.4722	0.0663	0.5082	1.0000	1.0000	0.5000
0.4000	0.4000	0.5994	0.4722	0.0663	0.5082	0.6667	0.5000	0.3333
0.5000	0.5000	0.5994	0.4722	0.0663	0.5082	1.0000	1.0000	0.5000
0.5000	0.5000	0.5994	0.4722	0.0663	0.5082	0.6667	0.5000	0.5000
0.5000	0.5000	0.5994	0.4722	0.0663	0.5082	1.0000	1.0000	0.3333
0.5000	0.5000	0.6615	0.4722	0.0106	0.2224	1.0000	1.0000	0.5000
0.5000	0.5000	0.6615	0.4722	0.0106	0.2219	1.0000	1.0000	0.3333
0.5000	0.5000	0.6615	0.4722	0.0106	0.2219	0.6667	0.5000	0.5000
0.5000	0.5000	0.5994	0.4470	0.0663	0.5082	1.0000	1.0000	0.3333
0.5000	0.5000	0.5994	0.2921	0.0663	0.5082	1.0000	1.0000	0.3333
0.5000	0.5000	0.5994	0.3529	0.0663	0.5082	1.0000	1.0000	0.3333
0.5000	0.5000	0.5994	0.4470	0.0663	0.5082	1.0000	1.0000	0.3333

0.5000	0.5000	0.5994	0.4722	0.0663	0.5082	1.0000	1.0000	0.3333
0.3000	0.3000	0.4284	0.2681	0.0114	0.2088	0.6667	0.3333	0.3333
0.3000	0.3000	0.4844	0.2681	0.0121	0.2318	0.6667	0.3333	0.5000
0.4000	0.4000	0.6672	0.4746	0.0325	0.3879	0.6667	0.5000	0.5000
0.3000	0.3000	0.5295	0.4381	0.0127	0.2468	0.5000	0.6667	0.3333
0.4000	0.4000	0.5295	0.4746	0.0127	0.2468	1.0000	1.0000	0.5000
0.3000	0.3000	0.5840	0.3773	0.2197	0.7218	0.6667	0.5000	0.3333
0.4000	0.4000	0.5840	0.3773	0.0325	0.3879	0.6667	0.5000	0.3333
0.3000	0.3000	0.5840	0.2677	0.0127	0.2468	0.5000	0.5000	0.0000
0.4000	0.4000	0.4844	0.3100	0.0120	0.2359	0.6667	0.5000	0.5000
0.4000	0.4000	0.5840	0.4746	0.2197	0.7218	0.5000	0.5000	0.0000
0.6000	0.6000	0.3929	0.2923	0.0103	0.2166	0.6667	0.5000	0.5000
0.2000	0.2000	0.2550	0.2615	0.0103	0.2166	0.6667	0.5000	0.3333
0.4000	0.4000	1.0000	0.9249	0.2197	0.7218	1.0000	1.0000	0.5000
0.4000	0.4000	0.2881	0.0974	0.2196	0.7183	0.6667	0.5000	0.5000
0.4000	0.4000	0.4083	0.2434	0.2191	0.6984	0.1667	0.5000	0.5000
0.3000	0.3000	0.2003	0.0749	0.2191	0.6984	0.6667	0.5000	0.5000
0.4000	0.4000	0.7904	0.8763	0.2193	0.7078	0.1667	0.5000	0.5000
0.4000	0.4000	0.3929	0.2258	0.0506	0.4527	0.6667	0.5000	0.5000
0.5000	0.5000	0.3258	0.2992	0.0111	0.2255	1.0000	1.0000	0.5000
0.2000	0.2000	0.3898	0.2434	0.2189	0.6928	0.1667	0.5000	0.5000
0.5000	0.5000	0.4083	0.2191	0.0054	0.1396	0.6667	0.5000	0.5000
0.3000	0.3000	1.0000	0.9249	0.0146	0.2660	0.0000	0.5000	0.5000
0.3000	0.3000	0.2146	0.2656	0.0101	0.2095	0.6667	0.3333	0.5000
0.2000	0.2000	0.3181	0.3100	0.0101	0.2095	0.6667	0.3333	0.5000
0.4000	0.4000	0.4054	0.3700	0.2196	0.7183	0.6667	0.5000	0.3333
0.4000	0.4000	0.3351	0.2544	0.2196	0.7183	0.6667	0.5000	0.3333
0.6000	0.6000	0.4253	0.3486	0.2196	0.7183	0.6667	0.5000	0.5000
0.1000	0.1000	0.4018	0.2906	0.0184	0.2817	0.0000	0.0000	0.0000
0.2000	0.2000	0.4284	0.3201	0.0184	0.2817	0.6667	0.3333	0.3333
0.4000	0.4000	0.4844	0.3700	0.0103	0.2166	0.6667	0.3333	0.5000
0.3000	0.3000	0.4578	0.4598	0.0123	0.2390	0.6667	0.3333	0.5000
0.6000	0.6000	0.4844	0.3279	0.0120	0.2383	0.6667	0.5000	0.5000
0.4000	0.4000	0.3807	0.4598	0.0121	0.2311	0.6667	0.3333	0.5000
0.3000	0.3000	0.4844	0.3242	0.0287	0.3493	0.6667	0.3333	0.5000
0.6000	0.6000	0.5135	0.3862	0.0109	0.2183	0.6667	0.5000	0.5000
0.2000	0.2000	0.4651	0.3279	0.0114	0.2088	0.6667	0.3333	0.5000
0.3000	0.3000	0.4844	0.3279	0.0114	0.2170	1.0000	1.0000	0.5000
0.6000	0.6000	0.4651	0.3600	0.0119	0.2245	0.6667	0.5000	0.5000
0.5000	0.5000	0.5135	0.3862	0.0220	0.3262	0.6667	0.5000	0.5000
0.6000	0.6000	0.4844	0.4368	0.0101	0.2095	0.6667	0.5000	0.5000
0.4000	0.4000	0.4844	0.3486	0.0104	0.2202	0.6667	0.5000	0.5000
0.3000	0.3000	0.4106	0.3849	0.0508	0.4591	0.6667	0.5000	0.3333
0.3000	0.3000	0.1576	0.1706	0.0101	0.2095	0.6667	0.5000	0.3333
0.3000	0.3000	0.5008	0.3700	0.0118	0.2205	0.6667	0.3333	0.5000
0.5000	0.5000	0.5451	0.3600	0.0508	0.4591	0.6667	0.5000	0.5000
0.3000	0.3000	0.3922	0.3862	0.0101	0.2095	0.6667	0.5000	0.3333
0.3000	0.3000	0.4869	0.4138	0.0044	0.1140	0.5000	0.5000	0.0000
0.2000	0.2000	0.2924	0.3529	0.0364	0.3865	1.0000	1.0000	0.5000
0.3000	0.3000	0.3421	0.2084	0.0073	0.1741	1.0000	1.0000	0.3333
0.3000	0.3000	0.3253	0.3529	0.0364	0.3865	1.0000	1.0000	0.5000
0.3000	0.3000	0.4651	0.5214	0.0121	0.2318	1.0000	0.3333	1.0000
0.6000	0.6000	0.4844	0.3741	0.0549	0.4756	1.0000	1.0000	0.5000
0.4000	0.4000	0.4844	0.3849	0.0119	0.2347	1.0000	0.5000	1.0000
0.3000	0.3000	0.4578	0.5196	0.0101	0.2095	0.6667	0.3333	0.5000
0.5000	0.5000	0.4895	0.3849	0.0119	0.2347	0.6667	0.5000	0.5000
0.5000	0.5000	0.4844	0.3279	0.0123	0.2390	0.6667	0.3333	0.5000
0.4000	0.4000	0.3592	0.3573	0.0103	0.2166	1.0000	0.5000	1.0000
0.3000	0.3000	0.4578	0.3555	0.0101	0.2095	0.6667	0.3333	0.5000
0.5000	0.5000	0.3945	0.3741	0.0549	0.4756	1.0000	1.0000	0.3333
0.4000	0.4000	0.4895	0.4138	0.0104	0.2202	0.6667	0.5000	0.5000
0.4000	0.4000	0.4578	0.2932	0.0101	0.2095	0.6667	0.5000	0.5000
0.3000	0.3000	0.4284	0.3723	0.0121	0.2318	0.6667	0.3333	0.3333
0.4000	0.4000	0.4054	0.3862	0.0119	0.2347	0.6667	0.5000	0.5000
0.4000	0.4000	0.3106	0.3324	0.0101	0.2095	0.6667	0.5000	0.3333
0.3000	0.3000	0.2919	0.2766	0.0069	0.1677	1.0000	1.0000	0.3333
0.5000	0.5000	0.3185	0.3324	0.0107	0.2205	0.6667	0.5000	0.5000
0.5000	0.5000	0.3597	0.2491	0.0103	0.2166	0.6667	0.5000	0.3333
0.4000	0.4000	0.2828	0.3324	0.0367	0.3983	1.0000	1.0000	0.5000
0.5000	0.5000	0.4506	0.3279	0.0101	0.2138	1.0000	0.5000	1.0000
0.3000	0.3000	0.2698	0.1417	0.0105	0.2134	0.6667	0.5000	0.5000
0.4000	0.4000	0.4844	0.2656	0.0103	0.2166	0.6667	0.5000	0.5000
0.3000	0.3000	0.4284	0.3555	0.0121	0.2318	0.6667	0.3333	0.3333
0.3000	0.3000	0.2734	0.3279	0.0107	0.2205	1.0000	0.5000	1.0000
0.6000	0.6000	0.4844	0.5437	0.0103	0.2166	1.0000	0.5000	1.0000
0.6000	0.6000	0.6615	0.4470	0.0120	0.2383	0.6667	0.5000	0.5000
0.3000	0.3000	0.4844	0.3209	0.0118	0.2217	0.6667	0.3333	0.5000

0.4000	0.4000	0.4844	0.3209	0.0103	0.2166	1.0000	0.5000	1.0000
0.3000	0.3000	0.4065	0.3209	0.0101	0.2095	0.6667	0.3333	0.3333
0.2000	0.2000	0.3144	0.4598	0.0114	0.2088	0.6667	0.3333	0.5000
0.3000	0.3000	0.2698	0.2656	0.0054	0.1426	0.6667	0.5000	0.5000
0.4000	0.4000	0.4284	0.3126	0.0121	0.2318	0.6667	0.3333	0.3333
0.3000	0.3000	0.4099	0.2191	0.0062	0.1531	0.6667	0.5000	0.5000
0.4000	0.4000	0.3574	0.3576	0.0073	0.1741	0.6667	0.5000	0.5000
0.5000	0.5000	0.3592	0.3700	0.0103	0.2166	1.0000	1.0000	0.5000
0.4000	0.4000	0.4092	0.3043	0.0214	0.3037	0.6667	0.5000	0.3333
0.3000	0.3000	0.3253	0.2766	0.0101	0.2095	0.6667	0.5000	0.3333
0.3000	0.3000	0.4002	0.3773	0.2197	0.7218	0.6667	0.5000	0.5000
0.2000	0.2000	0.3158	0.4598	0.0118	0.2200	0.6667	0.3333	0.5000
0.5000	0.5000	0.5840	0.3773	0.2197	0.7218	0.6667	0.5000	0.3333
0.4000	0.4000	0.2550	0.2491	0.0103	0.2166	0.6667	0.5000	0.3333
0.4000	0.4000	0.4869	0.4138	0.0367	0.3990	0.6667	0.5000	0.5000
0.3000	0.3000	0.3080	0.4598	0.0114	0.2088	0.6667	0.3333	0.5000
0.5000	0.5000	0.5028	0.4184	0.0369	0.4090	0.6667	0.5000	0.5000
0.5000	0.5000	0.3737	0.3279	0.0117	0.2276	0.6667	0.5000	0.5000
0.4000	0.4000	0.4902	0.3700	0.0117	0.2276	0.6667	0.3333	0.5000
0.3000	0.3000	0.3900	0.2656	0.0117	0.2276	0.6667	0.3333	0.5000
0.4000	0.4000	0.3399	0.3279	0.0117	0.2276	0.6667	0.5000	0.3333
0.3000	0.3000	0.3177	0.2656	0.0117	0.2276	0.6667	0.3333	0.3333
0.4000	0.4000	0.4844	0.3126	0.0123	0.2390	0.6667	0.5000	0.5000
0.4000	0.4000	0.4054	0.2992	0.0119	0.2347	0.6667	0.5000	0.5000
0.5000	0.5000	0.6672	0.4746	0.0252	0.3473	1.0000	0.5000	0.5000
0.1000	0.1000	0.0906	0.3218	0.0322	0.3771	0.5000	0.6667	0.3333
0.1000	0.1000	0.1495	0.2854	0.0304	0.3190	0.5000	0.5000	0.0000
0.6000	0.6000	0.1599	0.4196	0.0322	0.3771	0.5000	0.6667	0.3333
0.2000	0.2000	0.2003	0.4196	0.0324	0.3849	0.5000	0.6667	0.3333
0.2000	0.2000	0.2003	0.1876	0.0324	0.3849	0.5000	0.6667	0.3333
0.1000	0.1000	0.1587	0.1966	0.0316	0.3541	0.5000	0.5000	0.0000
0.0000	0.0000	0.0732	0.1091	0.0324	0.3849	0.5000	0.5000	0.0000
0.5000	0.5000	0.1495	0.1091	0.0324	0.3849	0.5000	0.5000	0.0000
0.5000	0.5000	0.1217	0.1181	0.0324	0.3849	0.5000	0.5000	0.0000
0.4000	0.4000	0.1587	0.1059	0.0316	0.3541	0.0000	0.0000	0.0000
0.5000	0.5000	0.2188	0.1091	0.0324	0.3849	0.5000	0.5000	0.0000
0.0000	0.0000	0.1587	0.1059	0.0316	0.3541	0.0000	0.0000	0.0000
0.5000	0.5000	0.1495	0.1091	0.0324	0.3849	0.5000	0.5000	0.0000
0.5000	0.5000	0.2188	0.1181	0.0324	0.3849	0.5000	0.5000	0.0000
0.5000	0.5000	0.2188	0.1181	0.0324	0.3849	0.5000	0.5000	0.0000
0.3000	0.3000	0.1307	0.3507	0.0322	0.3771	0.1667	0.1667	0.3333
0.3000	0.3000	0.1307	0.3507	0.0322	0.3771	0.5000	0.6667	0.3333
0.5000	0.5000	0.0924	0.2165	0.0315	0.3511	0.0000	0.0000	0.0000
0.6000	0.6000	0.2465	0.2854	0.0315	0.3511	0.5000	0.6667	0.3333
0.5000	0.5000	0.1772	0.3218	0.0322	0.3771	0.5000	0.6667	0.3333
0.1000	0.1000	0.0223	0.0787	0.0303	0.3167	0.1667	0.1667	0.3333
0.5000	0.5000	0.2188	0.1076	0.0312	0.3421	0.0000	0.0000	0.0000
0.2000	0.2000	0.1895	0.0406	0.0041	0.0888	0.0000	0.0000	0.0000
0.5000	0.5000	0.2781	0.1460	0.0037	0.1089	0.5000	0.6667	0.3333
0.5000	0.5000	0.1309	0.1876	0.0054	0.1432	0.5000	0.5000	0.0000
0.5000	0.5000	0.1217	0.0787	0.0315	0.3511	0.0000	0.0000	0.0000
0.5000	0.5000	0.1032	0.1966	0.0054	0.1432	0.5000	0.5000	0.0000
0.3000	0.3000	0.1118	0.1059	0.0027	0.0731	0.0000	0.0000	0.0000
0.5000	0.5000	0.1256	0.1876	0.0045	0.1093	0.0000	0.0000	0.0000
0.5000	0.5000	0.2079	0.2795	0.0122	0.2386	0.5000	0.5000	0.0000
0.1000	0.1000	0.1495	0.1876	0.0304	0.3190	0.0000	0.0000	0.0000
0.1000	0.1000	0.1495	0.1876	0.0303	0.3167	0.5000	0.5000	0.0000
0.1000	0.1000	0.1495	0.1876	0.0304	0.3190	0.0000	0.0000	0.0000
0.6000	0.6000	0.2188	0.1876	0.0324	0.3849	0.5000	0.5000	0.0000
0.5000	0.5000	0.0917	0.1091	0.0324	0.3849	0.5000	0.5000	0.0000
0.2000	0.2000	0.0418	0.1876	0.0303	0.3167	0.1667	0.1667	0.3333
0.5000	0.5000	0.2188	0.0787	0.0312	0.3421	0.0000	0.0000	0.0000
0.5000	0.5000	0.1495	0.1876	0.0053	0.1231	0.0000	0.0000	0.0000
0.6000	0.6000	0.1495	0.1876	0.0045	0.1093	0.0000	0.0000	0.0000
0.0000	0.0000	0.2003	0.1181	0.0062	0.1569	0.5000	0.5000	0.0000
0.4000	0.4000	0.2003	0.1091	0.0062	0.1569	0.5000	0.5000	0.0000
0.5000	0.5000	0.2003	0.1966	0.0062	0.1569	0.5000	0.5000	0.0000
0.5000	0.5000	0.2188	0.1181	0.0062	0.1569	0.5000	0.5000	0.0000
0.5000	0.5000	0.2188	0.1181	0.0062	0.1569	0.5000	0.5000	0.0000
0.0000	0.0000	0.1587	0.1059	0.0053	0.1262	0.0000	0.0000	0.0000
0.6000	0.6000	0.2227	0.1876	0.0050	0.1141	0.0000	0.0000	0.0000
0.6000	0.6000	0.2227	0.1966	0.0053	0.1262	0.0000	0.0000	0.0000
0.5000	0.5000	0.2227	0.0787	0.0312	0.3421	0.1667	0.1667	0.3333
0.5000	0.5000	0.2227	0.0969	0.0050	0.1156	0.0000	0.0000	0.0000
0.5000	0.5000	0.2227	0.0876	0.0316	0.3541	0.0000	0.0000	0.0000
0.6000	0.6000	0.1533	0.1966	0.0316	0.3541	0.0000	0.0000	0.0000
0.5000	0.5000	0.2003	0.1876	0.0081	0.1874	0.5000	0.5000	0.0000

0.6000	0.6000	0.1495	0.1734	0.0045	0.1093	0.0000	0.0000	0.0000
0.5000	0.5000	0.2188	0.1091	0.0062	0.1569	0.5000	0.5000	0.0000
0.2000	0.2000	0.2003	0.1380	0.0062	0.1569	0.5000	0.5000	0.0000
0.6000	0.6000	0.2188	0.2165	0.0062	0.1569	0.5000	0.5000	0.0000
0.0000	0.0000	0.1587	0.1059	0.0053	0.1262	0.0000	0.0000	0.0000
0.2000	0.2000	0.2003	0.1380	0.0062	0.1569	0.5000	0.5000	0.0000
0.6000	0.6000	0.1495	0.1876	0.0042	0.1004	0.0000	0.0000	0.0000
0.6000	0.6000	0.2227	0.1876	0.0050	0.1141	0.0000	0.0000	0.0000
0.6000	0.6000	0.2227	0.1876	0.0070	0.1461	0.5000	0.5000	0.0000
0.5000	0.5000	0.2188	0.0726	0.0050	0.1156	0.0000	0.0000	0.0000
0.2000	0.2000	0.1895	0.0406	0.0041	0.0888	0.0000	0.0000	0.0000
0.6000	0.6000	0.2188	0.2165	0.0050	0.1141	0.0000	0.0000	0.0000
0.5000	0.5000	0.2227	0.0609	0.0069	0.1446	0.5000	0.5000	0.0000
0.3000	0.3000	0.1533	0.0815	0.0011	0.0351	0.0000	0.0000	0.0000
0.5000	0.5000	0.2227	0.0726	0.0050	0.1156	0.0000	0.0000	0.0000
0.0000	0.0000	0.2003	0.1181	0.0324	0.3849	0.5000	0.5000	0.0000
0.2000	0.2000	0.2003	0.3084	0.0122	0.2386	0.5000	0.5000	0.0000
0.5000	0.5000	0.1371	0.2069	0.0071	0.1505	1.0000	1.0000	0.0000
0.5000	0.5000	0.1638	0.4196	0.0079	0.1796	0.5000	0.6667	0.3333
0.5000	0.5000	0.2356	0.4196	0.0060	0.1492	0.5000	0.6667	0.3333
0.1000	0.1000	0.1624	0.3218	0.0322	0.3771	0.1667	0.1667	0.3333
0.3000	0.3000	0.1895	0.2165	0.0057	0.1388	0.5000	0.5000	0.0000
0.6000	0.6000	0.2356	0.4196	0.0322	0.3771	0.5000	0.6667	0.3333
0.5000	0.5000	0.2597	0.4196	0.0034	0.0992	0.5000	0.6667	0.3333
0.4000	0.4000	0.2157	0.4196	0.0055	0.1462	0.5000	0.6667	0.3333
0.1000	0.1000	0.1271	0.3101	0.0322	0.3771	0.5000	0.6667	0.3333
0.6000	0.6000	0.2188	0.3507	0.0322	0.3771	0.5000	0.6667	0.3333
0.2000	0.2000	0.3914	0.1947	0.0037	0.1089	0.1667	0.1667	0.3333
0.0000	0.0000	0.1495	0.4196	0.0322	0.3771	0.5000	0.6667	0.3333
0.5000	0.5000	0.2227	0.1076	0.0315	0.3511	0.1667	0.1667	0.3333
0.5000	0.5000	0.2188	0.0772	0.0070	0.1461	0.5000	0.5000	0.0000
0.5000	0.5000	0.2227	0.0787	0.0312	0.3421	0.0000	0.0000	0.0000
0.5000	0.5000	0.2227	0.0726	0.0070	0.1461	0.5000	0.5000	0.0000
0.6000	0.6000	0.2188	0.1759	0.0050	0.1141	0.0000	0.0000	0.0000
0.5000	0.5000	0.1726	0.2519	0.0316	0.3541	0.0000	0.5000	0.5000
0.6000	0.6000	0.1111	0.1631	0.0312	0.3436	0.0000	0.0000	0.0000
0.4000	0.4000	0.0524	0.0787	0.0315	0.3511	0.5000	0.5000	0.0000
0.5000	0.5000	0.1079	0.0542	0.0312	0.3436	0.0000	0.0000	0.0000
0.0000	0.0000	0.0000	0.0000	0.0018	0.0506	0.0000	0.0000	0.0000
#NAME?	#NAME?	#NAME?	#NAME?	#NAME?	#NAME?	#NAME?	#NAME?	#NAME?
0.5000	0.5000	0.1533	0.0542	0.0312	0.3421	0.0000	0.0000	0.0000
0.3000	0.3000	0.2227	0.0271	0.0000	0.0000	0.0000	0.0000	0.0000
0.5000	0.5000	0.2188	0.0335	0.0053	0.1262	0.0000	0.0000	0.0000
#NAME?	#NAME?	#NAME?	#NAME?	#NAME?	#NAME?	#NAME?	#NAME?	#NAME?
0.5000	0.5000	0.2227	0.0852	0.0312	0.3436	0.0000	0.0000	0.0000
0.5000	0.5000	0.2227	0.0787	0.0312	0.3436	0.0000	0.0000	0.0000
0.5000	0.5000	0.0693	0.0245	0.0069	0.1446	0.5000	0.5000	0.0000
0.0000	0.0000	0.0894	0.0815	0.0021	0.0611	0.0000	0.0000	0.0000
0.0000	0.0000	0.0000	0.0000	0.0000	0.0000	0.0000	0.0000	0.0000
0.5000	0.5000	0.2227	0.0609	0.0050	0.1141	0.0000	0.0000	0.0000
0.6000	0.6000	0.2188	0.1966	0.0053	0.1262	0.0000	0.0000	0.0000
0.3000	0.3000	0.2227	0.0478	0.0073	0.1566	0.5000	0.5000	0.0000
0.3000	0.3000	0.1533	0.0726	0.0018	0.0506	0.0000	0.0000	0.0000
0.1000	0.1000	0.1495	0.1876	0.0319	0.3668	0.0000	0.0000	0.0000
0.5000	0.5000	0.1117	0.0237	0.0070	0.1461	0.5000	0.5000	0.0000
0.6000	0.6000	0.2227	0.0609	0.0057	0.1388	0.0000	0.0000	0.0000
0.4000	0.4000	0.2227	0.0513	0.0057	0.1388	0.0000	0.0000	0.0000
0.6000	0.6000	0.2227	0.0720	0.0057	0.1388	0.0000	0.0000	0.0000
0.6000	0.6000	0.2188	0.0720	0.0057	0.1388	0.0000	0.0000	0.0000
0.6000	0.6000	0.2227	0.0542	0.0319	0.3668	0.5000	0.5000	0.0000
0.6000	0.6000	0.2227	0.0385	0.0007	0.0247	0.0000	0.0000	0.0000
0.4000	0.4000	0.2356	0.0338	0.0078	0.1764	0.5000	0.5000	0.0000
0.6000	0.6000	0.2227	0.0481	0.0015	0.0477	0.0000	0.0000	0.0000
0.5000	0.5000	0.1495	0.0481	0.0008	0.0231	0.0000	0.0000	0.0000
0.5000	0.5000	0.2785	0.0947	0.0127	0.2448	1.0000	1.0000	0.3333
0.7000	0.7000	0.3621	0.3507	0.0127	0.2448	1.0000	1.0000	0.3333
0.7000	0.7000	0.3621	0.3507	0.0127	0.2448	1.0000	1.0000	0.3333
0.4000	0.4000	0.2025	0.3507	0.0127	0.2448	1.0000	1.0000	0.3333
0.5000	0.5000	0.4506	0.3313	0.0127	0.2468	0.6667	0.5000	0.5000
0.5000	0.5000	0.4873	0.3522	0.0127	0.2468	1.0000	1.0000	0.5000
0.6000	0.6000	0.5008	0.3362	0.0663	0.5082	1.0000	1.0000	1.0000
0.5000	0.5000	0.5008	0.3495	0.0127	0.2450	0.6667	0.5000	0.5000
0.4000	0.4000	0.3863	0.3710	0.0127	0.2448	1.0000	1.0000	0.3333
0.4000	0.4000	0.2159	0.3507	0.0127	0.2448	1.0000	1.0000	0.3333

0.4000	0.4000	0.2025	0.3507	0.0127	0.2450	0.6667	0.5000	0.3333
0.5000	0.5000	0.2514	0.3781	0.0127	0.2448	0.6667	0.5000	0.3333
0.4000	0.4000	0.2025	0.3507	0.0127	0.2468	0.6667	0.5000	0.5000
0.5000	0.5000	0.4873	0.3781	0.0127	0.2448	1.0000	1.0000	0.3333
0.5000	0.5000	0.2137	0.3710	0.0127	0.2448	1.0000	1.0000	0.3333
0.4000	0.4000	0.2267	0.3710	0.0127	0.2448	1.0000	1.0000	0.3333
0.4000	0.4000	0.2514	0.3781	0.0127	0.2448	1.0000	1.0000	0.3333
0.5000	0.5000	0.3287	0.3781	0.0127	0.2466	0.6667	0.5000	0.3333
0.4000	0.4000	0.1895	0.3507	0.0127	0.2448	1.0000	1.0000	1.0000
0.5000	0.5000	0.2469	0.3710	0.0127	0.2448	1.0000	1.0000	0.3333
0.4000	0.4000	0.4231	0.3710	0.0127	0.2448	1.0000	1.0000	0.3333
0.6000	0.6000	0.4231	0.3710	0.0127	0.2466	1.0000	1.0000	0.3333
0.5000	0.5000	0.2514	0.3781	0.0127	0.2448	0.6667	0.5000	0.3333
0.6000	0.6000	0.2756	0.3983	0.0127	0.2445	0.6667	0.5000	0.3333
0.4000	0.4000	0.2137	0.3710	0.0096	0.2077	0.6667	0.5000	0.3333
0.5000	0.5000	0.2338	0.3710	0.0127	0.2466	1.0000	1.0000	0.3333
0.6000	0.6000	0.3989	0.3507	0.0127	0.2448	0.6667	0.5000	0.3333
0.4000	0.4000	0.2137	0.3710	0.0127	0.2448	0.6667	0.5000	0.3333
0.4000	0.4000	0.4231	0.3710	0.0096	0.2077	0.6667	0.5000	0.3333
0.4000	0.4000	0.4231	0.3710	0.0106	0.2205	1.0000	1.0000	0.3333
0.4000	0.4000	0.4231	0.3710	0.0127	0.2448	1.0000	1.0000	0.3333
0.4000	0.4000	0.2159	0.3507	0.0127	0.2448	0.6667	0.5000	0.3333
0.4000	0.4000	0.2619	0.3781	0.2196	0.7199	0.6667	0.5000	0.3333
0.5000	0.5000	0.4231	0.3710	0.0127	0.2468	1.0000	1.0000	0.3333
0.5000	0.5000	0.2137	0.3710	0.0127	0.2448	1.0000	1.0000	0.3333
0.6000	0.6000	0.4231	0.3710	0.0106	0.2223	0.6667	0.5000	0.3333
0.5000	0.5000	0.2338	0.3710	0.0127	0.2466	0.6667	0.5000	0.3333
0.4000	0.4000	0.3989	0.4226	0.0127	0.2448	0.6667	0.5000	0.3333
0.4000	0.4000	0.3287	0.3507	0.0127	0.2448	0.6667	0.5000	0.3333
0.6000	0.6000	0.2096	0.3507	0.0106	0.2223	0.6667	0.5000	0.3333
0.4000	0.4000	0.2025	0.3507	0.0106	0.2205	0.6667	0.5000	0.3333
0.4000	0.4000	0.2648	0.3781	0.0127	0.2448	0.6667	0.5000	0.3333
0.6000	0.6000	0.3863	0.3710	0.0127	0.2448	0.6667	0.5000	0.3333
0.5000	0.5000	0.2159	0.3507	0.0127	0.2448	0.6667	0.5000	0.5000
0.4000	0.4000	0.3287	0.4226	0.2196	0.7199	0.6667	0.5000	0.3333
0.4000	0.4000	0.3287	0.3798	0.0127	0.2448	1.0000	1.0000	0.3333
0.4000	0.4000	0.4387	0.3781	0.2196	0.7199	0.6667	0.5000	0.3333
0.4000	0.4000	0.2003	0.3507	0.0127	0.2448	1.0000	1.0000	0.3333
0.5000	0.5000	0.3621	0.3507	0.0127	0.2448	0.6667	0.5000	0.3333
0.4000	0.4000	0.3421	0.3781	0.0127	0.2448	0.6667	0.5000	0.3333
0.4000	0.4000	0.2025	0.3507	0.0127	0.2448	0.6667	0.5000	0.3333
0.5000	0.5000	0.4387	0.3571	0.2196	0.7199	1.0000	0.5000	1.0000
0.4000	0.4000	0.2754	0.3781	0.0127	0.2448	0.6667	0.5000	0.3333
0.4000	0.4000	0.2648	0.3781	0.0127	0.2448	0.6667	0.5000	0.3333
0.4000	0.4000	0.3621	0.3781	0.0127	0.2448	1.0000	1.0000	0.3333
0.4000	0.4000	0.3287	0.3507	0.0062	0.1585	1.0000	1.0000	0.3333
0.5000	0.5000	0.1710	0.3507	0.0062	0.1585	0.6667	0.5000	0.3333
0.5000	0.5000	0.1895	0.3507	0.0127	0.2450	1.0000	1.0000	0.3333
0.3000	0.3000	0.1895	0.3507	0.0062	0.1581	1.0000	1.0000	0.3333
0.3000	0.3000	0.1895	0.3507	0.0106	0.2207	1.0000	1.0000	0.3333
0.2000	0.2000	0.1516	0.3344	0.0106	0.2205	0.5000	0.6667	0.3333
0.3000	0.3000	0.3989	0.3507	0.0324	0.3861	1.0000	1.0000	0.3333
0.3000	0.3000	0.3287	0.3507	0.0127	0.2448	0.5000	0.6667	0.3333
0.3000	0.3000	0.1895	0.1745	0.0127	0.2448	0.5000	0.6667	0.3333
0.3000	0.3000	0.4237	0.3507	0.0127	0.2450	0.5000	0.6667	0.3333
0.3000	0.3000	0.4237	0.3507	0.0106	0.2207	0.5000	0.6667	0.3333
0.3000	0.3000	0.4479	0.3710	0.0127	0.2450	0.5000	0.6667	0.3333
0.3000	0.3000	0.1895	0.3507	0.0324	0.3859	0.5000	0.6667	0.3333
0.3000	0.3000	0.2025	0.3507	0.0127	0.2448	1.0000	1.0000	0.3333
0.4000	0.4000	0.3287	0.3507	0.0324	0.3861	1.0000	1.0000	0.3333
0.3000	0.3000	0.2025	0.3507	0.0324	0.3859	0.5000	0.6667	0.3333
0.3000	0.3000	0.1895	0.3507	0.2196	0.7199	0.5000	0.6667	0.3333
0.3000	0.3000	0.1895	0.3507	0.0324	0.3859	0.5000	0.6667	0.3333
0.3000	0.3000	0.1895	0.1745	0.0324	0.3859	0.5000	0.6667	0.3333
0.6000	0.6000	0.2338	0.2313	0.0063	0.1597	0.6667	0.5000	0.3333
0.4000	0.4000	0.2137	0.1217	0.0095	0.2075	0.5000	0.6667	0.3333
0.3000	0.3000	0.2137	0.1670	0.0106	0.2205	0.1667	0.1667	0.3333
0.3000	0.3000	0.2137	0.1670	0.0324	0.3859	0.1667	0.1667	0.3333
0.6000	0.6000	0.2137	0.1670	0.0062	0.1579	1.0000	1.0000	0.3333
0.6000	0.6000	0.2137	0.1670	0.0106	0.2205	0.1667	0.1667	0.3333
0.4000	0.4000	0.2137	0.1670	0.0106	0.2207	0.1667	0.1667	0.3333
0.6000	0.6000	0.2137	0.1670	0.0106	0.2205	0.1667	0.1667	0.3333
0.3000	0.3000	0.4231	0.1947	0.0127	0.2448	0.1667	0.1667	0.3333
0.3000	0.3000	0.2137	0.1670	0.0106	0.2205	0.1667	0.1667	0.3333
0.6000	0.6000	0.2137	0.1670	0.0106	0.2205	0.1667	0.1667	0.3333
0.5000	0.5000	0.2137	0.1217	0.0106	0.2205	0.5000	0.6667	0.3333
0.6000	0.6000	0.4231	0.1670	0.0106	0.2205	0.6667	0.5000	0.3333

0.3000	0.3000	0.2025	0.2523	0.2196	0.7199	1.0000	1.0000	0.3333
0.2000	0.2000	0.4231	0.1670	0.0106	0.2202	0.5000	0.6667	0.3333
0.3000	0.3000	0.2137	0.1670	0.0106	0.2205	0.1667	0.1667	0.3333
0.6000	0.6000	0.1895	0.1468	0.0106	0.2207	0.6667	0.5000	0.3333
0.3000	0.3000	0.4231	0.1670	0.0106	0.2207	0.1667	0.1667	0.3333
0.4000	0.4000	0.4231	0.1670	0.0106	0.2205	0.1667	0.1667	0.3333
0.3000	0.3000	0.2137	0.1670	0.0106	0.2205	0.1667	0.1667	0.3333
0.3000	0.3000	0.2137	0.1670	0.0106	0.2207	0.1667	0.1667	0.3333
0.3000	0.3000	0.2137	0.1670	0.0106	0.2207	0.1667	0.1667	0.3333
0.3000	0.3000	0.4231	0.1947	0.0106	0.2207	1.0000	1.0000	0.3333
0.3000	0.3000	0.4231	0.1670	0.0106	0.2207	0.5000	0.6667	0.3333
0.5000	0.5000	0.4231	0.1670	0.0106	0.2207	0.5000	0.6667	0.3333
0.3000	0.3000	0.4231	0.1670	0.0106	0.2207	0.5000	0.6667	0.3333
0.3000	0.3000	0.3989	0.2036	0.0324	0.3861	0.5000	0.6667	0.3333
0.3000	0.3000	0.3989	0.1745	0.0106	0.2207	0.5000	0.6667	0.3333
0.5000	0.5000	0.4231	0.1947	0.0106	0.2223	0.5000	0.6667	0.3333
0.5000	0.5000	0.4231	0.1759	0.0107	0.2229	0.5000	0.6667	0.3333
0.4000	0.4000	0.4231	0.1670	0.0106	0.2207	1.0000	1.0000	0.3333
0.3000	0.3000	0.4097	0.2036	0.0324	0.3861	0.5000	0.6667	0.3333
0.3000	0.3000	0.3287	0.1745	0.0106	0.2207	1.0000	1.0000	0.3333
0.5000	0.5000	0.4231	0.1947	0.0106	0.2223	1.0000	1.0000	0.3333
0.3000	0.3000	0.4231	0.1759	0.0107	0.2229	1.0000	1.0000	0.3333
0.3000	0.3000	0.3635	0.2036	0.0107	0.2227	0.5000	0.6667	0.3333
0.3000	0.3000	0.3744	0.3467	0.0663	0.5082	1.0000	1.0000	0.3333
0.3000	0.3000	0.3120	0.3467	0.0106	0.2224	1.0000	1.0000	0.3333
0.3000	0.3000	0.2778	0.3344	0.0106	0.2205	1.0000	1.0000	0.3333
0.3000	0.3000	0.3744	0.3426	0.0662	0.5062	1.0000	1.0000	0.3333
0.3000	0.3000	0.4387	0.3467	0.0663	0.5082	1.0000	1.0000	0.3333
0.3000	0.3000	0.3755	0.3426	0.0106	0.2205	0.6667	0.5000	0.3333
0.3000	0.3000	0.3061	0.3354	0.0106	0.2224	0.6667	0.5000	0.3333
0.4000	0.4000	0.3112	0.3993	0.0106	0.2224	1.0000	1.0000	0.3333
0.3000	0.3000	0.3755	0.3426	0.0106	0.2205	0.6667	0.5000	0.3333
0.3000	0.3000	0.3061	0.3993	0.0106	0.2224	0.6667	0.5000	0.3333
0.3000	0.3000	0.2471	0.4017	0.0073	0.1776	0.6667	0.5000	0.3333
0.3000	0.3000	0.3120	0.3993	0.0104	0.2202	0.6667	0.5000	0.3333
0.4000	0.4000	0.3592	0.3700	0.0104	0.2182	0.6667	0.5000	0.5000
0.5000	0.5000	0.3592	0.3741	0.0078	0.1841	0.6667	0.5000	0.5000
0.3000	0.3000	0.3061	0.3741	0.0106	0.2224	0.6667	0.5000	0.3333
0.4000	0.4000	0.3592	0.3741	0.0104	0.2202	0.6667	0.5000	0.5000
0.3000	0.3000	0.2256	0.2279	0.0108	0.2247	1.0000	1.0000	0.3333
0.1000	0.1000	0.2393	0.1247	0.0325	0.3879	0.5000	0.6667	0.3333
0.2000	0.2000	0.2267	0.3798	0.0108	0.2247	0.5000	0.6667	0.3333
0.2000	0.2000	0.3287	0.3798	0.0325	0.3879	1.0000	1.0000	0.3333
0.2000	0.2000	0.2256	0.3798	0.0108	0.2247	1.0000	1.0000	0.3333
0.2000	0.2000	0.2256	0.3798	0.0108	0.2247	1.0000	1.0000	0.3333
0.2000	0.2000	0.2256	0.2279	0.0325	0.3879	1.0000	1.0000	0.3333
0.2000	0.2000	0.2005	0.3798	0.0108	0.2247	1.0000	1.0000	0.3333
0.2000	0.2000	0.2005	0.3798	0.0108	0.2247	0.5000	0.6667	0.3333
0.2000	0.2000	0.2005	0.3798	0.0108	0.2247	0.5000	0.6667	0.3333
0.2000	0.2000	0.2256	0.3798	0.0325	0.3879	1.0000	1.0000	0.3333
0.2000	0.2000	0.3816	0.3773	0.0325	0.3879	1.0000	1.0000	0.3333
0.4000	0.4000	0.2550	0.3741	0.0106	0.2224	1.0000	0.5000	0.5000
0.3000	0.3000	0.3755	0.3618	0.0106	0.2205	0.6667	0.5000	0.3333
0.2000	0.2000	0.3112	0.3618	0.0106	0.2205	1.0000	1.0000	0.3333
0.3000	0.3000	0.3112	0.2766	0.0077	0.1807	1.0000	1.0000	0.3333
0.3000	0.3000	0.2491	0.3618	0.0106	0.2205	0.6667	0.5000	0.3333
0.3000	0.3000	0.3112	0.3618	0.0106	0.2205	1.0000	1.0000	0.3333
0.3000	0.3000	0.3755	0.2656	0.0104	0.2182	0.6667	0.5000	0.3333
0.3000	0.3000	0.2436	0.2766	0.0104	0.2182	0.6667	0.5000	0.3333
0.3000	0.3000	0.1906	0.2656	0.0103	0.2168	0.6667	0.3333	0.3333
0.4000	0.4000	0.3755	0.3741	0.0104	0.2202	0.6667	0.5000	0.5000
0.3000	0.3000	0.3755	0.2766	0.0104	0.2182	0.6667	0.5000	0.3333
0.3000	0.3000	0.3794	0.3993	0.0106	0.2224	0.6667	0.5000	0.3333
0.3000	0.3000	0.3755	0.3354	0.0106	0.2224	1.0000	1.0000	0.3333
0.5000	0.5000	0.3794	0.3993	0.0106	0.2224	1.0000	0.5000	1.0000
0.3000	0.3000	0.3112	0.3354	0.0106	0.2224	1.0000	1.0000	0.3333
0.4000	0.4000	0.3755	0.3993	0.0078	0.1841	0.6667	0.5000	0.3333
0.4000	0.4000	0.3631	0.3018	0.0124	0.2410	0.6667	0.3333	0.5000
0.3000	0.3000	0.3755	0.3993	0.0104	0.2202	0.6667	0.5000	0.3333
0.4000	0.4000	0.3592	0.3993	0.0125	0.2444	0.6667	0.3333	0.5000
0.6000	0.6000	0.3592	0.3114	0.0103	0.2168	0.6667	0.5000	0.5000
0.3000	0.3000	0.3755	0.3741	0.0104	0.2202	0.6667	0.3333	0.3333
0.4000	0.4000	0.3755	0.3993	0.0106	0.2224	1.0000	1.0000	0.5000
0.4000	0.4000	0.3592	0.3741	0.0036	0.1068	0.6667	0.3333	0.5000
0.4000	0.4000	0.3592	0.2766	0.0075	0.1734	0.6667	0.3333	0.5000
0.4000	0.4000	0.3592	0.3741	0.0104	0.2202	1.0000	0.3333	1.0000
0.4000	0.4000	0.3592	0.2766	0.0098	0.1981	0.6667	0.3333	0.5000

0.4000	0.4000	0.3592	0.3741	0.0125	0.2444	0.6667	0.3333	0.5000
#NAME?	#NAME?	0.0000	#NAME?	#NAME?	#NAME?	0.0000	0.0000	0.0000
0.4000	0.4000	0.1664	0.0243	0.0058	0.1494	1.0000	1.0000	0.3333
0.5000	0.5000	0.2435	0.3894	0.0125	0.2360	0.6667	0.5000	0.3333
0.2000	0.2000	0.5028	0.2854	0.0057	0.1503	0.5000	0.6667	0.3333
0.3000	0.3000	0.3158	0.0460	0.0005	0.0182	1.0000	1.0000	0.3333
0.3000	0.3000	0.3872	0.4196	0.0073	0.1776	1.0000	1.0000	0.3333
0.2000	0.2000	0.1818	0.1312	0.2193	0.7079	0.5000	0.6667	0.3333
0.2000	0.2000	0.3636	0.4196	0.0325	0.3879	1.0000	1.0000	0.3333
0.4000	0.4000	0.2842	0.3624	0.0057	0.1504	1.0000	1.0000	0.3333
0.2000	0.2000	0.3914	0.3773	0.0147	0.2664	0.5000	0.6667	0.3333
0.0000	0.0000	0.0710	0.0095	0.0019	0.0584	0.1667	0.1667	0.3333
0.1000	0.1000	0.2280	0.1245	0.0054	0.1432	0.5000	0.6667	0.3333
0.5000	0.5000	0.5028	0.4196	0.0073	0.1776	1.0000	1.0000	0.3333
0.4000	0.4000	0.5028	0.4470	0.0762	0.5325	1.0000	1.0000	0.3333
0.5000	0.5000	0.3755	0.3426	0.0104	0.2182	1.0000	1.0000	0.3333
0.5000	0.5000	0.5362	0.4470	0.0104	0.2202	1.0000	1.0000	0.5000
0.5000	0.5000	0.5028	0.4196	0.0123	0.2415	1.0000	1.0000	0.5000
0.4000	0.4000	0.2919	0.2766	0.0761	0.5289	1.0000	1.0000	0.3333
0.5000	0.5000	0.3253	0.3611	0.0762	0.5305	1.0000	1.0000	0.3333
0.4000	0.4000	0.5362	0.4470	0.0762	0.5325	1.0000	1.0000	0.3333
0.3000	0.3000	0.3253	0.2907	0.0103	0.2166	0.6667	0.5000	0.3333
0.2000	0.2000	0.3253	0.2556	0.0069	0.1687	1.0000	1.0000	0.3333
0.4000	0.4000	0.3120	0.2382	0.0127	0.2448	1.0000	1.0000	0.3333
0.3000	0.3000	0.3120	0.2249	0.0127	0.2448	1.0000	1.0000	0.3333
0.5000	0.5000	0.6615	0.4381	0.0187	0.3021	0.6667	0.5000	0.5000
0.5000	0.5000	0.8327	0.4722	0.0104	0.2202	1.0000	1.0000	0.5000
0.5000	0.5000	0.5954	0.4260	0.0187	0.3021	0.6667	0.5000	0.5000
0.4000	0.4000	0.8327	0.4722	0.0078	0.1841	1.0000	1.0000	0.3333
0.5000	0.5000	0.8327	0.4381	0.0104	0.2202	1.0000	1.0000	0.3333
0.4000	0.4000	0.8327	0.4381	0.0104	0.2202	1.0000	1.0000	0.3333
0.2000	0.2000	0.6720	0.3652	0.0073	0.1776	0.6667	0.5000	0.3333
0.4000	0.4000	0.5295	0.4470	0.0073	0.1776	1.0000	1.0000	0.3333
